# The Random Nature of Genome Architecture: Predicting Open Reading Frame Distributions

**DOI:** 10.1371/journal.pone.0006456

**Published:** 2009-07-30

**Authors:** Michael W. McCoy, Andrew P. Allen, James F. Gillooly

**Affiliations:** 1 Department of Biology, Boston University, Boston, Massachusetts, United States of America; 2 Department of Biological Sciences, Macquarie University, Sydney, New South Wales, Australia; 3 Department of Biology, University of Florida, Gainesville, Florida, United States of America; Pasteur Institute, France

## Abstract

**Background:**

A better understanding of the size and abundance of open reading frames (ORFS) in whole genomes may shed light on the factors that control genome complexity. Here we examine the statistical distributions of open reading frames (i.e. distribution of start and stop codons) in the fully sequenced genomes of 297 prokaryotes, and 14 eukaryotes.

**Methodology/Principal Findings:**

By fitting mixture models to data from whole genome sequences we show that the size-frequency distributions for ORFS are strikingly similar across prokaryotic and eukaryotic genomes. Moreover, we show that i) a large fraction (60–80%) of ORF size-frequency distributions can be predicted a priori with a stochastic assembly model based on GC content, and that (ii) size-frequency distributions of the remaining “non-random” ORFs are well-fitted by log-normal or gamma distributions, and similar to the size distributions of annotated proteins.

**Conclusions/Significance:**

Our findings suggest stochastic processes have played a primary role in the evolution of genome complexity, and that common processes govern the conservation and loss of functional genomics units in both prokaryotes and eukaryotes.

## Introduction

Understanding the origins of genome complexity remains a central challenge in evolutionary biology. The sequencing of genomes across the tree of life has revealed considerable heterogeneity in both coding and non-coding portions of genomes that does not appear to be related to organismal complexity [e.g. *1*], [2]–[*5*]]. Stark differences between prokaryotic and eukaryotic genomes have sparked debate regarding the relative importance of neutral versus adaptive processes in the evolution of genome architecture [*2*], [3], [6]–[*8*], as well as the relative importance of epigenetic phenomena [*9*].

Still, some clear patterns in genome architecture have emerged in recent years. In general, multicellular organisms have larger genomes than their unicellular prokaryotic and eukaryotic ancestors. Although larger genomes generally have larger genes and more introns, most of the increase in genome size has been attributed to an increase in what appears to be non-coding DNA [*4*], [6], [7], [10]–[*13*]. This observation has led some to hypothesize as to the possible adaptive significance of non-coding DNA [e.g., the skeletal–DNA hypothesis, *14*] [i.e. the buffering–DNA hypothesis, *11*], and others to suggest a primary role for neutral processes owing to the generally smaller effective population sizes of more derived organisms [*2*].

In this study, we assess the contribution of stochastic processes to observed variation in genome architecture. We do so by evaluating the extent to which a random assembly model can predict the size distribution of open reading frames (ORFs) in genomes, and the extent to which the remaining “non-random” ORF size distribution corresponds to the size distribution of annotated proteins. We test the model using data from 311 fully sequenced and referenced genomes from simple bacteria to multicellular eukaryotes (Table S1). Our results show that the vast majority of the heterogeneity in the size distributions of ORFs can be predicted based on random assembly, and that much of the remaining, non-random variation shows a size distribution similar to that of proteins. However, we observe a much larger absolute number of non-random ORFs than predicted based on known annotated proteins. We conclude by speculating as to how the further development of purely stochastic models, such as the one presented here, may provide insights into the origin and maintenance of genome complexity.

### Models

We assessed the contributions of random and non-random processes to variation in the size distributions of ORFs among prokaryotic and eukaryotic genomes by evaluating two hypotheses. Hypothesis 1 is that ORFs that do not code for proteins follow expectations for a random sequence of nucleotides. Hypothesis 2 is that ORFs that do code for proteins adhere to a size distribution that is distinct from that observed for random ORFs, but similar to that of annotated proteins, as previously suggested [*15*], [*16*]. Together, Hypotheses 1 and 2 imply that the size-frequency distribution of ORFs should adhere to a “mixture” model [*17*], [*18*] comprised of two statistical distributions, one corresponding to non-coding sequences, and another corresponding to coding sequences.

We evaluated Hypotheses 1 and 2 by first fitting two different mixture models to the ORF size data, a mixture of an exponential distribution and a lognormal distribution
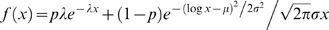
(1)and a mixture of an exponential distribution and a gamma distribution
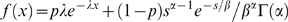
(2)


In these expressions, 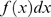 is the overall probability of obtaining an ORF in the size range (*x* – *dx*/2, *x*+*dx*/2), 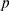 is the probability that a given ORF adheres to the first distribution in the mixture, 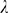 characterizes the exponential distributions, 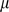 and 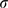 characterize the lognormal distribution, and 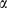 and 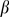 characterize the shape and scale of the gamma distribution. We chose to consider lognormal and gamma distributions for the non-random portion of the models because sizes of genes and proteins are often fitted to these distributions [*16*]. Both distributions arise naturally assuming a birth-death process whereby genomes increase in size due to random processes (e.g. self-replicating elements), that lead to genome degradation (e.g. mutations) [*18*]–[*20*]. These distributions correspond to somewhat different stochastic processes [e.g. *21*], [*22*], so distinguishing between them may be important.

Hypothesis 1 predicts a one-to-one correspondence between 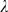, which is estimated based on the observed size distribution of ORFs, and 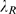, which is calculated based on the nucleotide composition of the sequence. Thus, evaluating hypothesis 1 entails comparing the parameter estimate 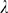 in the first term of the mixture model, 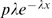, to its expected value for a random sequence of nucleotides, 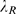. The lengths of random sequences between successive occurrences of a specific codon should follow a geometric distribution (GD) [*5*] but can be well approximated by an exponential distribution (the continuous counterpart to the GD) since the lengths of the sequences extend over several orders of magnitude. In the exponential distribution 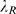 is the probability that a given nucleotide triplet is a stop codon [*23*], [*24*]. This quantity is calculated based on the overall nucleotide composition of the sequence by summing the probabilities of obtaining each of the three stop codons. Note that the three nucleotides that constitute a start or stop codon differ in the sense (start = ATG; stop = TAA, TAG, TGA) and antisense directions (start = CAT; stop = TTA, CTA, TCA) such that 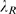 is the same in either direction.

Hypothesis 2 predicts a correspondence between parameter estimates of the lognormal (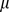, 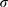), and gamma (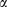, 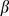) parameters obtained from the ORF mixture models (Eqs. 1–2) and from size distributions of proteins. Thus, evaluating hypothesis 2 entails comparing the estimated parameters of the second terms of the ORF-size models to parameter estimates obtained by fitting the lognormal, and gamma distributions to size distributions of annotated protein sequences.

## Methods

### Genome Sequence Data and ORF Counting

We used the contributed packages GeneR [*25*]and seqinR [*26*] in the R statistical programming environment [*27*] to acquire and analyze 311 complete genome sequences representing 297 species of prokaryotes and 14 species of eukaryotes, including both unicellular and multicellular forms (Table S1). Genomes ranged in size from less than 48 thousand base pairs for the bacterium *Geobacillus kaustophilus* to more than 120 million base pairs for the eukaryote *Drosophila melanogaster*, and these ranged in GC content from approximately 16 to 75 percent. When species were represented by more than one genome sequence, we randomly selected one sequence for inclusion in this comparative analysis. All sequences used in this study were acquired from the RefSeq library at NCBI (Table S1). For each sequence, we collected data on nucleotide composition, genome size, number of annotated genes, and protein size from the NCBI database. We then quantified the numbers and sizes of ORFs by summing across all six reading frames (+1, +2, +3, −1, −2, or −3) based on the first stop codon found upstream of each start codon in the sequence. Introns were not removed from ORFs prior to analysis.

#### Statistics

For each genome, the two mixture models (Eqs 1–2) were fitted to ORF-size data using the package “bbmle” in the R statistical programming environment [*28*]. The fits for the models for each genome were compared using Akaike's Information Criterion (AIC) [*29*].

To evaluate whether frequency distributions of ORFs adhered to random expectations (Hypothesis 1), we regressed 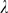 against 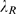 using ordinary least squares regression (OLS). For this analysis we only used the estimates of 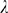 obtained from fits of the exponential-lognormal mixture because the estimates of lambda from model fits were highly correlated (r = 0.99). A linear relationship between the 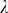 and 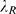 with a slope of 1 and an intercept of 0 would provide statistical support for Hypothesis 1 by demonstrating a one-to-one relationship between the two variables. Similarly, to evaluate the extent to which non-random ORF-size distributions correspond to protein-size distributions (Hypothesis 2), we used OLS to compare ORF-derived estimates of the lognormal (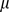, 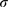), gamma (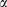, 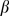) distributional parameters in Eqs. 1–2 to estimates obtained from size distributions of annotated proteins. As an additional test of Hypothesis 2, we used OLS to assess whether there was a one-to-one correspondence between the numbers of non-random ORFs (as estimated from the mixture models) and the number of annotated proteins. Differences between prokaryote and eukaryotes in the observed relationships were assessed using ANCOVA.

Finally, we wanted to determine if genome size was correlated with randomly generated ORFs. Because genome size and GC content are often correlated [e.g. *30*], [*31*] we used non-parametric smoothing functions in generalized additive models [*32*] to test for relationships between genome size, GC content for both the total numbers of ORFs in a genome, and for the fraction of ORFs explained by random processes (represented by *p* in Eqs. 1–2).

## Results

**Figure 1:**
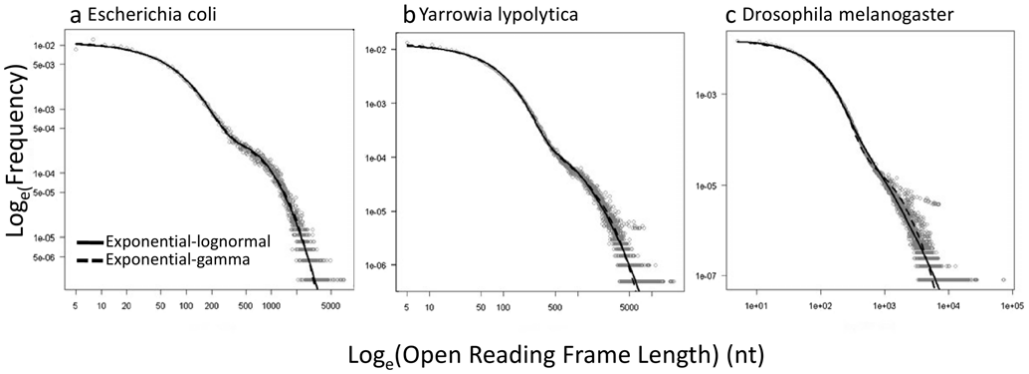
Fits of the 2 mixture models (Eqs. 1–2) to the genomes of three representative taxa. (a) *Escherichia coli*, a prokaryote, (b) *Yarrowia lypolytica*, a unicellular eukaryote, and (c) *Drosophila melanogaster*, a multicellular eukaryote.

**Figure 2:**
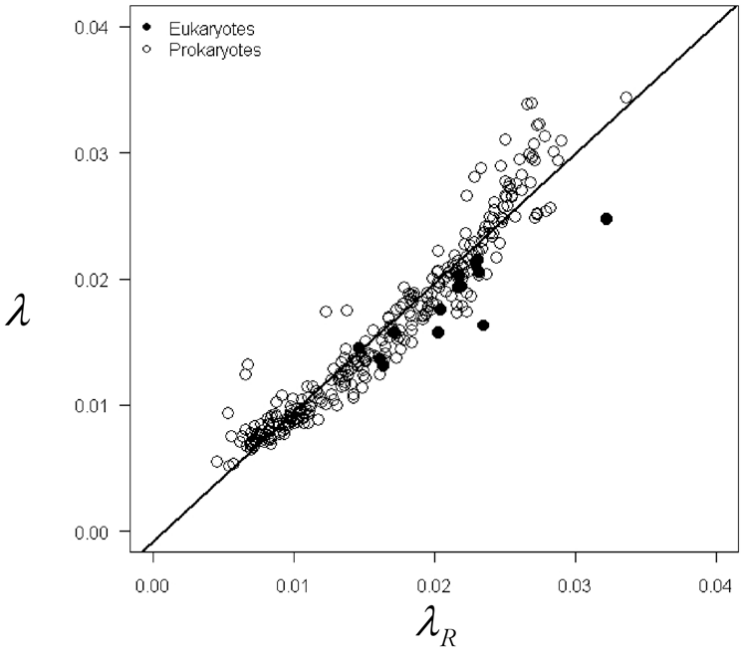
The size distributions of small ORFs in 311 whole genomes of prokaryotes and eukaryotes are consistent with random expectations (each point represents a genome). Observed values obtained by fitting the exponential components of the mixture models (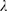 in Eqs. 1–2) were linearly related to the expected value for a random sequence of a given GC content, 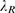, with a slope statistically indistinguishable from 1 and an intercept near 0 (P>0.05, r^2^ = 0.92).

The representative examples of model fits in Figure 1 illustrate that the ORF size distributions for entire genomes of both prokaryotes and eukaryotes are well described by both mixture models. Most deviations occur for large ORFs in the upper tails of the distributions (e.g., Fig. 1c and Fig. 2). In general, size distributions of small ORFs are well-characterized by the first (i.e. exponential) component of the mixture models in Eqs. 1–2. Size distributions of large ORFS are well-characterized by the second component of the mixture models (Eqs. 1–2). Specifically, AIC comparisons of overall model fits indicate that size distributions of large ORFs in 60% of the genomes are best characterized by the lognormal distribution (Eq. 1), while the remaining 40% of genomes are well characterized by the gamma distribution (Eq. 2). Comparisons of model fits, however, should be interpreted with some caution because even modest differences in goodness of fit will be statistically significant owing to the large number of points used to fit the models. However, this caution is only necessary for interpreting relative model fits and does not affect subsequent results and interpretation, as both mixture models have similar shapes, and may therefore indicate similar processes, as we will show.

**Figure 3:**
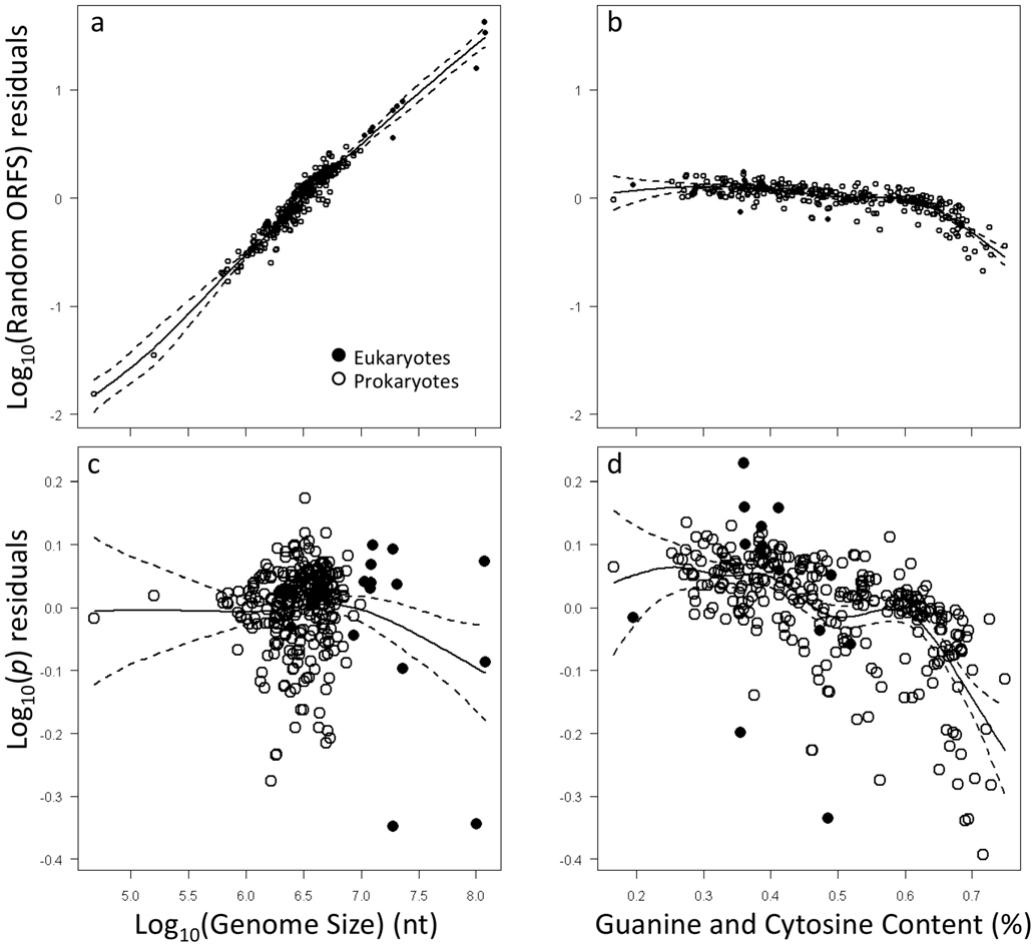
The relationship between ORFs and genome characteristics. Panels a and b: show the relationships between the total number of random ORFs versus genome size and GC content. Panels c and d: Show the relationships between the fraction of all ORFs that are randomly generated (*p* in Eqs. 1–2) versus genome size and GC content. Data were fitted using generalized additive models with non-parametric smoothing functions. Dashed lines represent 95% point wise confidence intervals.

Consistent with Hypothesis 1, the parameter estimate for the first component of the mixture model, 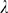
(taken from the best fit mixture model), is linearly related to the value expected for a random sequence of nucleotides, 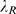, with a slope of 1 (95% CI = 0.99 to 1.06) and an intercept near 0 (95% CI = −0.002 to −0.0003) (F_1,309_ = 3588.1, P<0.0001, R^2^ = 0.92; Fig. 2). Note that similar relationships were observed for both eukaryotes and prokaryotes. Moreover, as expected, genome size and GC content affected both the total number and the fractions of ORFs described by the random distributions (Table 1). The total number of random ORFs, as estimated from 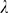, significantly increases with genome size (F = 682.5, p<0.0001; Fig. 3a), but significantly decreases with increasing GC content (F = 79.2, p<0.0001;Fig. 3b), given the relationship between GC content and the probability of getting a stop codon (Figure S1). Indeed, GC content and genome size explained 94.9 percent of the deviance in number of random ORFs (Table 1A). Interestingly, genome size did not explain a significant amount of the deviance in the total fractions of ORFs (*p* in Eqs. 1–2) described by the random components of the mixture models (F = 2.36, p = 0.06; Fig. 3c). However, GC content did explain a significant amount of the deviance (F = 23.12, p<0.0001; Table 1B) in the fraction of ORFs described by the random distribution such that the fraction of random ORFs decreases with increasing GC content (Fig. 3d).

**Table 1:** Results of a generalized additive model (GAM) using non-parametric smoothers.

	Factor	edf*	F-value	p-value	R^2^	Dev. expl.#
**A**	Genome Size GC Content	6.722 6.286	682.5 79.2	<0.0001 <0.0001	0.95	94.9%
**B**	Genome Size GC Content	3.199 6.434	2.36 23.12	0.0584 <0.0001	0.37	39.1%

**A**. GAM testing for a relationship between number of random ORFs, genome size, and GC content. **B**. GAM testing for a relationship between proportions of random ORFs, genome size, and GC content.

* edf is the estimated degrees of freedom accounting for the smoothing function.

# Deviance explained by the model with both factors.

**Figure 4:**
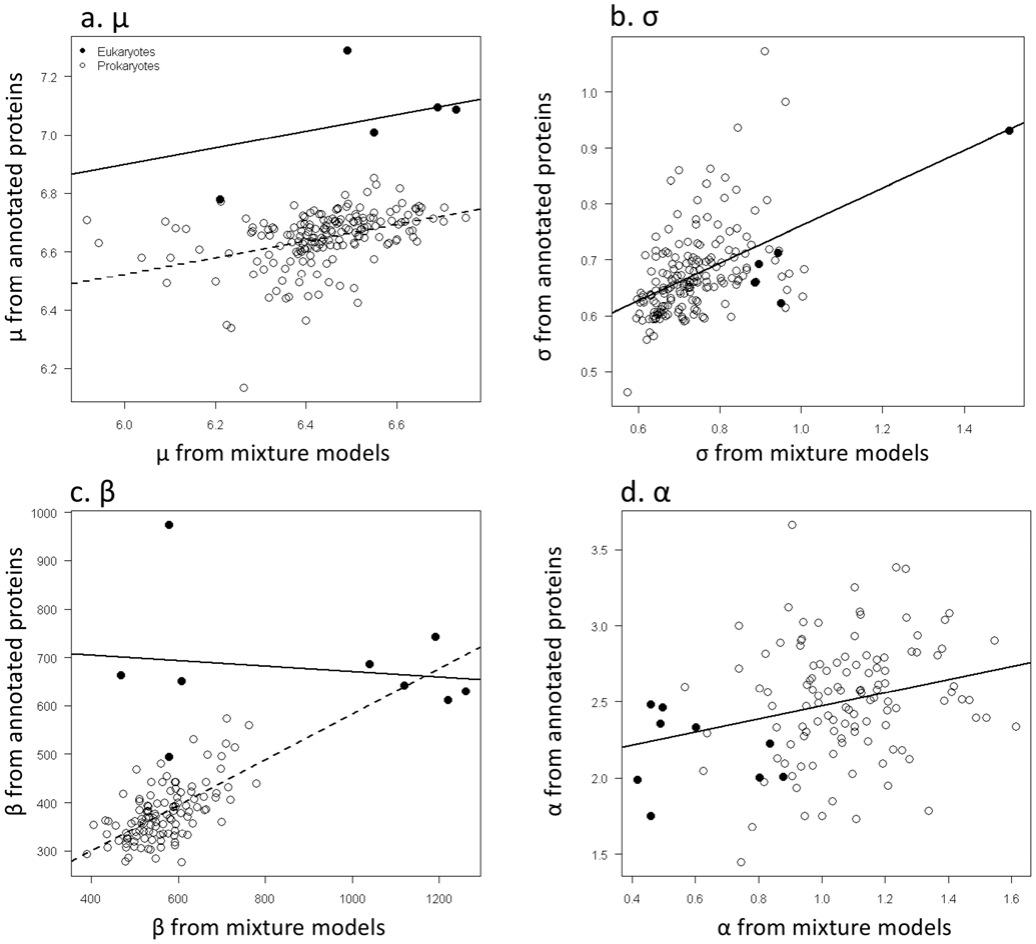
The relationship between parameters estimated from the mixture models and annotated. Panels a and b: Show the relationships between the parameters μ and σ of the lognormal distribution estimated from the mixture model fits with the μ and σ parameters estimated from fits to annotated proteins. Panels c and d: Show the relationships between parameters α and β of the gamma distribution estimated from the mixture model fits with the α and β parameters estimated from fits to annotated proteins. Data were fitted using analysis of covariance.

Consistent with Hypothesis 2, the size distribution of the remaining fraction of ORFs, described by the non-random distribution, is qualitatively quite similar to annotated protein distributions for genomes (Table S1, Figures S2, and S3). Indeed, supplements three and four illustrate the similarities between the size distributions of ORFS described by the non-random distribution and annotated proteins by illustrating the shapes of the distributions drawn using the parameters from the mixture model fits to the ORFs and proteins listed in Table S1. However, the non-random distribution of ORFs varied for both exponential-lognormal and exponential-gamma models such that the number of small non-random ORFs was greater (i.e. smaller scale and mean parameters in the exponential-gamma and exponential-log normal distributions respectively) than the number of small annotated proteins. Consequently, the peaks of the distributions in supplements three and four are shifted to the left of those for annotated proteins. Moreover, the number of small non-random ORFs was greater in multicellular eukaryotes than in prokaryotes. Specifically, for those species for which equation 1 provided the best fit, the parameter 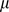 of the lognormal distribution for non-random ORFS was linearly correlated with those estimated for the annotated protein distributions for both prokaryotes and eukaryotes (slope - F_1,182_ = 47.65, p<0.0001, intercepts = F_1,182_ = 80.39, p<0.0001, interaction = F_1,182_ = 1.292, p = 0.297) (Fig. 4a). However, the estimated slopes of the relationships were substantially less than the predicted value of one (95% CIs = 0.19 to 0.38), and the intercepts were different from zero (95% CIs – Prokaryotes = 4.09 to 5.53; Eukaryotes = 4.55 to 5.82). Similarly, the parameter 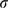 of the lognormal distribution (Eq. 1) or non-random ORFS was linearly correlated with those estimated for the annotated protein distributions for both prokaryotes and eukaryotes (slope - F_1,182_ = 57.01, p<0.0001, intercepts - F_1,182_ = 3.6411, p = 0.058, interaction- F_1,182_ = 0.201, p = 0.654) (Fig. 4b), but again the estimated slopes of these relationships were less than the predicted value of one ( 95% CIs = 0.249 to 0.426), and the intercept was different from zero (95% CIs – 0.358 to 0.491).

For those species with ORF distributions best described by the exponential-gamma mixture (Eq. 2), the relationship of the parameter 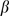 of the gamma distribution with those estimated from the annotated protein distributions was different for prokaryotes and eukaryotes (F_1,121_ = 33.595, p<0.0001) (Fig. 4c). For prokaryotes, there was a significant positive relationship between the estimates of 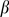 from the mixture models and estimates from the annotated protein distributions (t_121_ = 0.939, p<0.0001), though the slope and intercept of this relationship was different than predicted (95% CIs – slope = 0.172 to 0.772; intercept = −139.970 to 361.702). In contrast, for eukaryotes the estimates of 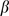 from the mixture model were independent of those estimated from annotated protein distributions (t_121_ = 5.796, p = 0.350) (Fig. 4c). Similarly, the estimates of 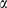 from the mixture model fits were linearly correlated with those estimated from annotated protein distributions (F_1,121_ = 8.9709, p = 0.003), but were not different for prokaryotes and eukaryotes (F_1,121_ = 0.6678, p = 0.415) (95% CIs –Intercept = 1.739 to 2.351; slope = 0.146 0.716) (Fig. 4d). These relationships, for both the log-normal and gamma parameters, suggest that the parameters estimated from the mixture models are consistently smaller than those estimated from the annotated proteins and thus are classifying more small ORFs as non random than are known to be protein coding. Nevertheless the linear relationships between these parameters suggest that the mixture models could be used for predicting protein size distributions.

**Figure 5:**
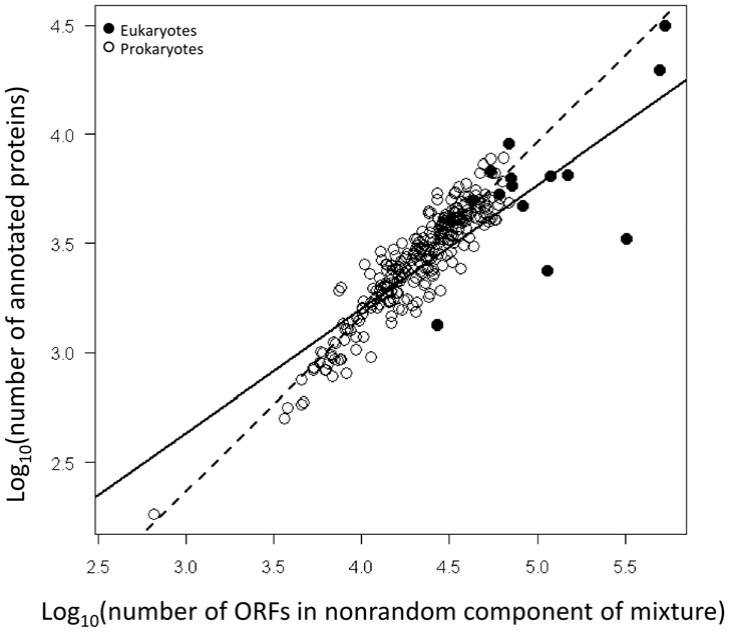
Relationship between the numbers of non-random ORFs based on the mixture model fits with the number of annotated proteins for 311 prokaryotic and eukaryotic genomes. Best fit lines determined from Analysis of Covariance where the dashed line represents the fit for prokaryotes, and the solid line represents the fit for eukaryotes.

Furthermore, with respect to Hypothesis 2, a plot of total non-random ORFs versus total annotated proteins (Fig. 5) reveals that for both prokaryotes and eukaryotes the number of nonrandom ORFs is strongly correlated with the number of annotated proteins. But, differences in the intercepts (F_1,544_ = 35.710, p<0.0001) and slopes (F_1,544_ = 9.144, p = 0.003) of these relationships indicate systematic deviations such that there are substantially larger numbers of non-random ORFS than annotated proteins (Fig. 5).

This finding is consistent with systematic differences in the estimated non-random ORF size distributions (e.g. differences in the β and μ parameters from the gamma and lognormal distributions respectively) as discussed above and illustrated in Figure 4.

## Discussion

Our findings suggest stochastic processes have played a primary role in the evolution of genome complexity. Surprisingly, the mixture models developed here fit all species across a broad range of genome sizes, both prokaryotes and eukaryotes, equally well (Figures 1 and 2, Table S1). The size distributions of ORFs in both random and non-random components were similar for all organisms.

Moreover, in both prokaryotes and eukaryotes, a large fraction of the total number of ORFs (60–80%) was predicted by a random assembly process. This finding was surprising given that it is widely held that larger, eukaryotic genomes (i.e. higher eukaryotes) contain mostly noncoding “Junk” DNA [e.g.s *6*], [7], [11], [33], [*34*], whereas the vast majority of DNA in unicellular genomes is thought to be protein-coding. Thus, these results suggest that eukaryotic and prokaryotic genomes may be more similar than previously thought, and that the processes governing these common features of genome architecture are shared.

The extent to which the ORF distributions deviate from the random expectation could serve as a metric for predicting the coding content of genomes [*5*]. Larger deviations from random expectation could suggest greater potential coding content of the genome [*5*]. In our analysis this deviation is described by the weighting parameters p and 1-p such that as p gets smaller, and 1-p larger, the greater the contribution of nonrandom ORFS is the genome [e.g., *5*]. Furthermore, in both prokaryotes and eukaryotes, the number and distribution of “non-random” ORFs are reasonably well explained by log-normal or gamma distributions. This, too, suggests that larger values of 1-p might indicate higher coding content of the genome. It may also suggest a common “birth-death” evolutionary process governing the conservation and loss of functional genomic units and that the processes governing the conversion of non-coding DNA into functional non-random units (i.e. genes) might be similar across taxa. Yet, we observed that the number of small non-random ORFs far exceeds that of annotated proteins. This points to the fact that these small ORFs, while not coding per se, are nonetheless being conserved. Perhaps, this large number of small non-random ORFs reflect the presence of transposable elements (TEs) and/or non-protein coding genes. Indeed, our observation that higher eukaryotes have a larger number of these small, non-random ORFs is consistent with the observation that these genomes are known to have more TEs than are lower eukaryotes and prokaryotes [*35]*–[*37*]. Some of these small sequences could also be small proteins or other functional units not previously identified [*38*]–[*40*]. In addition, because the start codon ATG also codes for the amino acid methionine some conserved small ORFs may be explained by the occurrence of ATG as a normal codon coding for methionine in the protein coding region.

Finally, we wish to point out that in some respects our results are consistent with the proposition that the evolution of genome complexity occurs mainly via genetic drift [*2*]. Our observation that the number of small “random” ORFs increases as genome size (and complexity) increases appears to be consistent with the hypothesis that large genomes, have evolved via neutral accumulation of junk DNA fragments [*6*]–[*8*]. Yet, contrary to this explanation, the total fraction of ORFs generated via random processes was observed to decrease with increasing genome size. Further research using newly available genomic data, combined with modeling efforts that account for stochasticity, promise to reveal much about genome evolution in the years ahead.

## Supporting Information

Table S1 Species Names, Accession Numbers, and Statistical Results. Table containing the names and accession numbers for the 311 genome and protein coding sequences analyzed in this study. This table also contains the parameter estimates and statistical results from all model fits for each species.
(0.10 MB PDF)

Figure S1 Supporting information for model development. Figure illustrating how the expected probability of a randomly generated stop codon declines with increasing GC content.
(0.18 MB PDF)

Figure S2 Relationships and illustrations of the shapes of the size distributions of ORFS and annotated proteins using log normal models. This supplement includes figures and analyses that depict the statistical relationships between the parameters of the exponential-log normal model and annotated proteins presented in Supplement 1. This supplement also includes figures that illustrate the shapes of each of these distributions.
(0.74 MB PDF)

Figure S3 Relationships and illustrations of the shapes of the size distributions of ORFS and annotated proteins using gamma distributions. This supplement includes figures and analyses that depict the statistical relationships between the parameters of the exponential-gamma model and annotated proteins presented in Supplement 1. This supplement also includes figures that illustrate the shapes of each of these distributions.
(0.58 MB PDF)
