# Supplementary material for: The Random Nature of Genome Architecture: Predicting Open Reading Frame Distributions
Source: PLoS One. 2009 Jul 30;4(7):e6456. doi: 10.1371/journal.pone.0006456 (PMC2714469; doi:10.1371/journal.pone.0006456)
Supplement: Table S1 — Species Names, Accession Numbers, and Statistical Results. Table containing the names and accession numbers for the 311 genome and protein coding sequences analyzed in this study. This table also contains the parameter estimates and statistical results from all model fits for each species. (0.10 MB PDF) [file pone.0006456.s001.pdf]

Genomes analysed		Exponential Log-normal fits to ORFs					Exponential Gamma fits to ORFs					Model Comparisons		Log-normal fits to proteins			Gamma fits to Proteins			Genome Summary Statistics							
species	accnum	*p*	λ	μ	σ	neg. loglikelihood	*p*	λ	α	β	neg. logliklihood	Δ AIC	best fit model	μ	σ	neg. logliklihood	α	β	neg. loglikelihood	no. ORFs	% T	% C	% A	% G	% Unknown	genome size (bp)	no. proteins
Arabidopsis_thaliana	NC_003070 - NC_003076	6.976E-01	2.160E-02	4.050E+00	1.380E+00	-2.310E+07	8.808E-01	2.000E-02	4.590E-01	5.790E+02	-2.310E+07	7.02E+03	exponential-gamma	6.899E+00	6.732E-01	-2.512E+05	2.484E+00	4.945E+02	-2.515E+05	4.454E+06	0.319	0.180	0.320	0.180	0.005	1.192E+08	3.171E+04
Caenorhabditis_elegan	NC_003279 - NC_003284	4.621E-01	1.630E-02	3.690E+00	1.200E+00	-1.650E+07	8.975E-01	1.740E-02	4.160E-01	4.670E+02	-1.650E+07	2.98E+04	exponential-gamma	6.912E+00	7.239E-01	-1.829E+05	1.988E+00	6.634E+02	-1.844E+05	3.162E+06	0.323	0.177	0.323	0.177	0.000	1.003E+08	3.328E+03
Candida_glabrata_CBS138	NC_005967 - NC_006036	8.765E-01	2.030E-02	6.690E+00	8.960E-01	-2.460E+06	8.348E-01	2.120E-02	7.320E-01	1.230E+03	-2.460E+06	-4.54E+03	exponential-log normal	7.095E+00	6.925E-01	-4.036E+04	2.346E+00	6.454E+02	-4.042E+04	4.374E+05	0.307	0.193	0.306	0.193	0.000	1.228E+07	6.796E+03
Cryptococcus_neoformans_var_JEC21	NC_006670 - NC_006670	3.968E-01	1.310E-02	4.100E+00	1.320E+00	-3.900E+06	8.278E-01	1.340E-02	4.950E-01	6.080E+02	-3.910E+06	4.69E+03	exponential-gamma	7.165E+00	6.877E-01	-5.316E+04	2.461E+00	6.523E+02	-5.310E+04	6.934E+05	0.257	0.242	0.257	0.243	0.000	1.905E+07	6.475E+03
Debaryomyces_hansenii_CBS767	NC_006043 - NC_006049	8.808E-01	2.130E-02	6.690E+00	8.530E-01	-2.600E+06	8.481E-01	2.230E-02	8.770E-01	1.040E+03	-2.600E+06	1.09E+03	exponential-gamma	6.960E+00	7.828E-01	-5.138E+04	2.010E+00	6.861E+02	-5.124E+04	4.668E+05	0.318	0.181	0.318	0.182	0.001	1.222E+07	6.317E+03
Drosophila_melanogaster_2R	NT_033777-NT_033777, NT_037436,NC_004353,NC _004354	7.112E-01	1.570E-02	4.100E+00	1.450E+00	-2.260E+07	8.808E-01	2.000E-02	4.590E-01	5.790E+02	-2.310E+07	2.45E+04	exponential-gamma	7.132E+00	7.825E-01	-1.642E+05	1.753E+00	9.753E+02	-1.653E+05	4.175E+06	0.294	0.206	0.293	0.207	0.000	1.204E+08	1.977E+04
Encephalitozoon_cuniculi	NC_003229 - NC_003242	7.203E-01	1.570E-02	6.210E+00	9.500E-01	-6.040E+05	5.886E-01	1.490E-02	5.320E-01	1.000E+03	-6.040E+05	-5.10E+02	exponential-log normal	6.779E+00	6.225E-01	-1.542E+04	2.615E+00	4.118E+02	-1.553E+04	9.648E+04	0.263	0.235	0.264	0.238	0.000	2.498E+06	1.337E+03
Eremothecium_gossypii	NC_005782 - NC5788	8.006E-01	1.450E-02	6.630E+00	8.890E-01	-1.700E+06	6.978E-01	1.520E-02	6.020E-01	1.260E+03	-1.700E+06	2.80E+01	exponential-gamma	7.065E+00	6.902E-01	-3.824E+04	2.335E+00	6.303E+02	-3.833E+04	2.742E+05	0.240	0.259	0.240	0.261	0.000	8.742E+06	4.714E+03
Kluyveromyces_lactis_NRRL_Y-1140	NC_006037 - NC_006042	8.849E-01	1.940E-02	6.740E+00	8.400E-01	-2.270E+06	8.494E-01	2.030E-02	8.350E-01	1.120E+03	-2.270E+06	2.44E+02	exponential-gamma	7.026E+00	7.087E-01	-4.250E+04	2.226E+00	6.432E+02	-4.260E+04	4.024E+05	0.306	0.194	0.306	0.194	0.000	1.069E+07	5.327E+03
Pichia_stipitis	NC_009042 - NC_009068, NT_166255, NT_166256	8.919E-01	1.760E-02	6.731E+00	8.859E-01	-3.622E+06	8.481E-01	1.833E-02	6.826E-01	1.300E+03	-3.622E+06	1.31E+03	exponential-log normal	7.086E+00	6.592E-01	-4.704E+04	2.534E+00	5.819E+02	-4.714E+04	6.383E+05	0.294	0.205	0.295	0.206	0.000	1.895E+07	9.052E+03
Plasmodium_falciparumCHR1	NC_004314 - NC_004318, NC_004325 - NC_004331, NC_00910, NC_00521	8.787E-01	2.480E-02	6.490E+00	1.510E+00	-5.090E+06	7.103E-01	3.430E-02	1.180E-01	5.530E+03	-5.090E+06	5.78E+03	exponential-log normal	7.290E+00	9.310E-01	-4.549E+04	1.275E+00	1.787E+03	-4.590E+04	9.379E+05	0.402	0.098	0.402	0.098	0.000	2.286E+07	2.375E+03
Saccharomyces_cerevisiae	NC_001133 - NC_001148	8.765E-01	1.950E-02	6.750E+00	8.610E-01	-2.520E+06	8.389E-01	2.040E-02	8.020E-01	1.190E+03	-2.520E+06	4.76E+02	exponential-gamma	7.035E+00	7.709E-01	-4.802E+04	2.005E+00	7.425E+02	-4.798E+04	4.447E+05	0.307	0.192	0.308	0.192	0.000	1.207E+07	5.860E+03
Schizosaccharomyces_pombe	NC_003421- NC_003424	9.072E-01	2.060E-02	6.550E+00	9.430E-01	-2.490E+06	8.688E-01	2.110E-02	6.030E-01	1.290E+03	-2.490E+06	-9.60E+01	exponential-log normal	7.010E+00	7.123E-01	-4.039E+04	2.178E+00	6.508E+02	-4.052E+04	4.580E+05	0.320	0.180	0.319	0.181	0.000	1.257E+07	4.993E+03
Yarrowia_lipolytica	NC_006067 -NC_006072	8.710E-01	1.360E-02	6.480E+00	9.100E-01	-4.040E+06	7.807E-01	1.360E-02	4.900E-01	1.220E+03	-4.040E+06	3.87E+02	exponential-gamma	7.047E+00	6.887E-01	-5.277E+04	2.358E+00	6.115E+02	-5.286E+04	6.835E+05	0.255	0.245	0.255	0.245	0.000	2.050E+07	6.520E+03
Acidovorax avenae	NC_008752	6.925E-01	6.483E-03	6.680E+00	6.805E-01	-1.042E+06	4.404E-01	7.533E-03	8.753E-01	7.293E+02	-1.042E+06	-5.66E+02	exponential-log normal	6.714E+00	6.267E-01	-1.490E+04	2.645E+00	3.802E+02	-1.498E+04	1.498E+05	0.158	0.342	0.157	0.343	0.000	5.353E+06	4.709E+03
Acidovorax JS42	NC_008782	7.475E-01	7.060E-03	6.618E+00	6.115E-01	-8.815E+05	4.803E-01	9.078E-03	9.566E-01	5.641E+02	-8.815E+05	-2.78E+01	exponential-log normal	6.705E+00	6.259E-01	-2.019E+04	2.800E+00	3.535E+02	-2.021E+04	1.308E+05	0.169	0.330	0.169	0.331	0.000	4.449E+06	4.007E+03
Acinetobacter baumannii	NC_009085	8.496E-01	2.150E-02	6.394E+00	7.077E-01	-8.448E+05	8.143E-01	2.285E-02	1.253E+00	5.107E+02	-8.448E+05	-3.95E+01	exponential-log normal	6.567E+00	5.982E-01	-1.723E+04	2.891E+00	2.954E+02	-1.733E+04	1.498E+05	0.305	0.194	0.306	0.196	0.000	3.977E+06	3.352E+03
Acinetobacter sp	NC_005966	8.594E-01	1.899E-02	6.480E+00	6.835E-01	-8.346E+05	8.259E-01	2.016E-02	1.329E+00	5.168E+02	-8.347E+05	-6.64E+01	exponential-log normal	6.623E+00	6.618E-01	-9.108E+03	2.602E+00	3.541E+02	-9.105E+03	1.463E+05	0.298	0.201	0.298	0.203	0.000	3.599E+06	3.325E+03
Actinobacillus pleuropneumoniae	NC_009053	7.870E-01	2.066E-02	6.396E+00	7.362E-01	-3.761E+05	7.260E-01	2.275E-02	1.071E+00	5.885E+02	-3.761E+05	-1.17E+02	exponential-log normal	6.696E+00	6.014E-01	-5.828E+03	3.013E+00	3.199E+02	-5.836E+03	6.349E+04	0.294	0.207	0.293	0.206	0.000	2.274E+06	2.012E+03
Aeromonas hydrophila	NC_008570	7.632E-01	1.049E-02	6.535E+00	6.629E-01	-9.540E+05	6.165E-01	1.224E-02	9.765E-01	6.032E+02	-9.543E+05	-5.33E+02	exponential-log normal	6.687E+00	6.455E-01	-1.846E+04	2.768E+00	3.510E+02	-1.844E+04	1.478E+05	0.192	0.307	0.193	0.308	0.000	4.744E+06	4.122E+03
Aeromonas salmonicida	NC_009348	7.734E-01	1.148E-02	6.456E+00	6.602E-01	-1.010E+06	6.460E-01	1.327E-02	1.050E+00	5.318E+02	-1.010E+06	-1.72E+02	exponential-log normal	6.669E+00	6.443E-01	-1.441E+04	2.703E+00	3.540E+02	-1.442E+04	1.593E+05	0.208	0.292	0.207	0.293	0.000	4.702E+06	4.086E+03
Aeropyrum pernix	NC_000854	6.895E-01	1.745E-02	5.996E+00	8.424E-01	-2.422E+05	4.760E-01	1.747E-02	5.667E-01	6.214E+02	-2.421E+05	6.48E+01	exponential-gamma	6.554E+00	6.585E-01	-5.898E+03	2.600E+00	3.332E+02	-5.900E+03	3.920E+04	0.221	0.284	0.216	0.280	0.000	1.670E+06	1.700E+03
Alcanivorax borkumensis	NC_008260	8.012E-01	1.225E-02	6.475E+00	7.062E-01	-6.409E+05	6.910E-01	1.352E-02	9.104E-01	6.395E+02	-6.410E+05	-2.75E+02	exponential-log normal	6.711E+00	6.148E-01	-1.092E+04	2.860E+00	3.459E+02	-1.095E+04	1.029E+05	0.226	0.273	0.226	0.274	0.000	3.120E+06	2.755E+03
Alkalilimnicola ehrlichei	NC_008340	7.553E-01	7.672E-03	6.649E+00	6.300E-01	-5.393E+05	5.646E-01	9.301E-03	1.038E+00	5.968E+02	-5.394E+05	-1.89E+02	exponential-log normal	6.758E+00	6.349E-01	-1.677E+04	2.697E+00	3.890E+02	-1.683E+04	8.055E+04	0.162	0.338	0.163	0.338	0.000	3.276E+06	2.865E+03
Alkaliphilus metalliredigens	NC_009633	8.137E-01	2.298E-02	6.264E+00	7.820E-01	-1.152E+06	7.531E-01	2.489E-02	9.487E-01	5.802E+02	-1.152E+06	5.16E+02	exponential-gamma	6.510E+00	7.024E-01	-1.760E+04	2.304E+00	3.680E+02	-1.762E+04	2.017E+05	0.316	0.183	0.316	0.185	0.000	4.930E+06	4.625E+03
Anabaena variabilis	NC_007413	8.531E-01	2.224E-02	6.404E+00	8.619E-01	-1.029E+06	7.962E-01	2.331E-02	7.181E-01	9.047E+02	-1.029E+06	-3.94E+02	exponential-log normal	6.670E+00	7.113E-01	-1.361E+04	2.089E+00	4.894E+02	-1.370E+04	1.832E+05	0.292	0.207	0.294	0.208	0.000	6.366E+06	5.043E+03
Anaeromyxobacter dehalogenans	NC_007760	6.474E-01	5.474E-03	6.704E+00	6.998E-01	-4.871E+05	3.975E-01	5.693E-03	8.868E-01	7.758E+02	-4.872E+05	-1.92E+02	exponential-log normal	6.752E+00	6.117E-01	-1.423E+04	2.879E+00	3.579E+02	-1.427E+04	6.825E+04	0.126	0.375	0.125	0.374	0.000	5.013E+06	4.346E+03
Anaplasma marginale	NC_004842	7.245E-01	1.591E-02	6.089E+00	1.008E+00	-2.551E+05	6.120E-01	1.427E-02	4.791E-01	1.072E+03	-2.553E+05	-2.91E+02	exponential-log normal	6.704E+00	6.839E-01	-6.216E+03	2.213E+00	4.695E+02	-6.263E+03	4.123E+04	0.251	0.251	0.252	0.247	0.000	1.198E+06	9.490E+02
Anaplasma phagocytophilum	NC_007797	8.471E-01	2.065E-02	6.263E+00	9.099E-01	-3.193E+05	7.674E-01	2.122E-02	5.672E-01	9.433E+02	-3.193E+05	-1.30E+02	exponential-log normal	6.134E+00	1.074E+00	-5.249E+03	1.031E+00	7.832E+02	-5.293E+03	5.645E+04	0.295	0.204	0.289	0.212	0.000	1.471E+06	1.264E+03
Archaeoglobus fulgidus	NC_000917	7.507E-01	1.573E-02	6.247E+00	7.046E-01	-3.866E+05	6.207E-01	1.842E-02	9.710E-01	4.805E+02	-3.866E+05	1.46E+02	exponential-gamma	6.495E+00	6.652E-01	-9.622E+03	2.580E+00	3.153E+02	-9.620E+03	6.288E+04	0.256	0.242	0.258	0.244	0.000	2.178E+06	2.420E+03
Arthrobacter aurescens	NC_008711	7.610E-01	8.363E-03	6.434E+00	6.642E-01	-8.014E+05	6.404E-01	9.075E-03	1.124E+00	4.999E+02	-8.014E+05	-5.89E+01	exponential-log normal	6.729E+00	6.173E-01	-1.357E+04	2.992E+00	3.330E+02	-1.356E+04	1.226E+05	0.187	0.312	0.189	0.312	0.000	4.598E+06	4.041E+03
Aster yellows	NC_007716	8.603E-01	2.562E-02	6.316E+00	7.877E-01	-1.031E+05	8.258E-01	2.691E-02	1.033E+00	5.927E+02	-1.030E+05	4.06E+01	exponential-gamma	6.413E+00	7.855E-01	-2.316E+03	1.850E+00	4.434E+02	-2.323E+03	1.890E+04	0.368	0.133	0.363	0.135	0.000	7.066E+05	
Azoarcus BH72	NC_008702	7.179E-01	6.854E-03	6.700E+00	6.075E-01	-6.959E+05	4.797E-01	9.231E-03	1.079E+00	5.842E+02	-6.959E+05	5.10E+01	exponential-gamma	6.692E+00	6.421E-01	-1.442E+04	2.697E+00	3.647E+02	-1.444E+04	1.014E+05	0.160	0.340	0.161	0.339	0.000	4.376E+06	3.989E+03
Azoarcus sp	NC_006513	7.358E-01	6.889E-03	6.694E+00	6.066E-01	-7.300E+05	5.034E-01	9.049E-03	1.064E+00	5.815E+02	-7.300E+05	3.73E+01	exponential-gamma	6.614E+00	7.227E-01	-1.869E+04	2.231E+00	4.250E+02	-1.869E+04	1.071E+05	0.174	0.325	0.175	0.326	0.000	4.296E+06	4.133E+03
Bacillus anthracis	NC_003997	8.166E-01	2.363E-02	6.220E+00	7.575E-01	-1.156E+06	7.534E-01	2.576E-02	9.699E-01	5.291E+02	-1.156E+06	2.85E+02	exponential-gamma	6.419E+00	7.609E-01	-2.685E+04	2.081E+00	3.827E+02	-2.680E+04	2.040E+05	0.324	0.178	0.322	0.176	0.000	5.227E+06	5.311E+03
Bacillus cereus	NC_004722	8.152E-01	2.420E-02	6.200E+00	8.037E-01	-1.180E+06	7.257E-01	2.634E-02	7.166E-01	6.684E+02	-1.181E+06	-7.88E+02	exponential-log normal	6.498E+00	6.951E-01	-1.925E+04	2.330E+00	3.580E+02	-1.929E+04	2.087E+05	0.323	0.176	0.324	0.177	0.000	5.412E+06	5.234E+03
Bacillus clausii	NC_006582	8.324E-01	1.495E-02	6.412E+00	6.433E-01	-9.739E+05	7.713E-01	1.640E-02	1.298E+00	4.506E+02	-9.740E+05	-8.26E+01	exponential-log normal	6.637E+00	5.953E-01	-1.556E+04	3.171E+00	2.842E+02	-1.555E+04	1.634E+05	0.276	0.224	0.276	0.223	0.000	4.304E+06	4.096E+03
Bacillus halodurans	NC_002570	8.384E-01	1.708E-02	6.433E+00	6.523E-01	-9.615E+05	7.996E-01	1.835E-02	1.486E+00	4.328E+02	-9.614E+05	2.36E+02	exponential-gamma	6.548E+00	7.135E-01	-1.534E+04	2.395E+00	3.638E+02	-1.528E+04	1.643E+05	0.281	0.217	0.282	0.220	0.000	4.202E+06	4.066E+03
Bacillus licheniformis	NC_006270	8.103E-01	1.440E-02	6.353E+00	7.260E-01	-9.410E+05	6.972E-01	1.570E-02	8.132E-01	6.261E+02	-9.414E+05	-7.83E+02	exponential-log normal	6.542E+00	6.814E-01	-1.750E+04	2.506E+00	3.430E+02	-1.748E+04	1.555E+05	0.270	0.231	0.268	0.231	0.000	4.222E+06	4.152E+03
Bacillus subtilis	NC_000964	7.747E-01	1.792E-02	6.101E+00	9.651E-01	-9.730E+05	6.579E-01	1.705E-02	4.582E-01	1.064E+03	-9.746E+05	-3.24E+03	exponential-log normal	6.580E+00	6.465E-01	-1.783E+04	2.706E+00	3.234E+02	-1.783E+04	1.630E+05	0.283	0.218	0.282	0.217	0.000	4.215E+06	4.105E+03
Bacillus thuringiensis	NC_008600	8.169E-01	2.371E-02	6.246E+00	7.606E-01	-1.158E+06	7.519E-01	2.591E-02	9.402E-01	5.593E+02	-1.158E+06	1.17E+01	exponential-gamma	6.657E+00	6.026E-01	-1.823E+04	3.024E+00	3.065E+02	-1.825E+04	2.042E+05	0.323	0.177	0.323	0.177	0.000	5.257E+06	4.736E+03
Bacteroides fragilis	NC_003228	7.861E-01	1.711E-02	6.464E+00	7.756E-01	-1.147E+06	7.099E-01	1.881E-02	9.413E-01	6.969E+02	-1.147E+06	8.28E+02	exponential-gamma	6.743E+00	7.180E-01	-1.574E+04	2.277E+00	4.708E+02	-1.573E+04	1.889E+05	0.284	0.216	0.284	0.216	0.000	5.205E+06	4.184E+03
Bacteroides thetaiotaomicron	NC_004663	7.861E-01	1.752E-02	6.505E+00	7.869E-01	-1.406E+06	7.400E-01	1.875E-02	1.073E+00	6.991E+02	-1.406E+06	1.04E+03	exponential-gamma	6.835E+00	6.931E-01	-2.204E+04	2.359E+00	4.964E+02	-2.206E+04	2.316E+05	0.287	0.213	0.285	0.215	0.000	6.260E+06	4.778E+03
Bacteroides vulgatus	NC_009614	7.917E-01	1.759E-02	6.453E+00	7.955E-01	-1.246E+06	7.176E-01	1.915E-02	9.013E-01	7.294E+02	-1.246E+06	9.65E+02	exponential-gamma	6.801E+00	7.175E-01	-1.273E+04	2.220E+00	5.141E+02	-1.274E+04	2.066E+05	0.289	0.212	0.289	0.210	0.000	5.163E+06	4.065E+03
Bartonella bacilliformis	NC_008783	8.545E-01	1.892E-02	6.459E+00	7.459E-01	-3.193E+05	8.055E-01	2.017E-02	9.917E-01	6.612E+02	-3.193E+05	-7.42E+01	exponential-log normal	6.576E+00	8.066E-01	-5.158E+03	1.909E+00	4.992E+02	-5.142E+03	5.571E+04	0.312	0.185	0.306	0.197	0.000	1.445E+06	1.283E+03
Bartonella henselae	NC_005956	8.647E-01	1.793E-02	6.409E+00	8.224E-01	-4.245E+05	8.044E-01	1.867E-02	7.337E-01	8.381E+02	-4.246E+05	-2.74E+02	exponential-log normal	6.587E+00	7.182E-01	-5.121E+03	1.981E+00	4.793E+02	-5.172E+03	7.424E+04	0.305	0.193	0.313	0.189	0.000	1.931E+06	1.488E+03
Bartonella quintana	NC_005955	8.695E-01	1.741E-02	6.488E+00	7.319E-01	-3.477E+05	8.235E-01	1.846E-02	1.013E+00	6.550E+02	-3.477E+05	-5.57E+01	exponential-log normal	6.755E+00	6.275E-01	-3.331E+03	2.658E+00	3.946E+02	-3.347E+03	6.064E+04	0.304	0.196	0.308	0.192	0.000	1.581E+06	1.142E+03
Baumannia cicadellinicola	NC_007984	8.363E-01	2.898E-02	6.413E+00	7.433E-01	-1.376E+05	8.144E-01	3.043E-02	1.365E+00	5.207E+02	-1.376E+05	4.46E+01	exponential-gamma	6.726E+00	6.267E-01	-2.241E+03	2.806E+00	3.578E+02	-2.244E+03	2.504E+04	0.334	0.166	0.334	0.166	0.000	6.862E+05	5.950E+02
Bdellovibrio bacteriovorus	NC_005363	7.483E-01	1.385E-02	6.408E+00	7.254E-01	-7.247E+05	6.254E-01	1.571E-02	9.175E-01	6.168E+02	-7.247E+05	-1.11E+02	exponential-log normal	6.684E+00	6.517E-01	-1.549E+04	2.648E+00	3.693E+02	-1.549E+04	1.150E+05	0.247	0.253	0.246	0.254	0.000	3.783E+06	3.587E+03
Bifidobacterium adolescentis	NC_008618	7.446E-01	8.571E-03	6.550E+00	6.728E-01	-5.077E+05	5.510E-01	1.007E-02	9.183E-01	6.201E+02	-5.077E+05	-1.22E+02	exponential-log normal	6.854E+00	6.020E-01	-6.818E+03	3.014E+00	3.739E+02	-6.826E+03	7.663E+04	0.203	0.297	0.205	0.295	0.000	2.090E+06	1.631E+03
Bifidobacterium longum	NC_004307	7.750E-01	9.021E-03	6.554E+00	7.016E-01	-5.048E+05	6.145E-01	9.908E-03	8.432E-01	6.954E+02	-5.049E+05	-2.23E+02	exponential-log normal	6.831E+00	6.435E-01	-8.158E+03	2.629E+00	4.318E+02	-8.175E+03	7.741E+04	0.199	0.301	0.200	0.301	0.000	2.257E+06	1.727E+03
Bordetella bronchiseptica	NC_002927	7.167E-01	6.685E-03	6.653E+00	6.216E-01	-1.063E+06	5.679E-01	7.918E-03	1.325E+00	5.291E+02	-1.063E+06	-9.46E+02	exponential-log normal	6.751E+00	5.571E-01	-1.885E+04	3.511E+00	2.814E+02	-1.886E+04	1.549E+05	0.160	0.339	0.159	0.342	0.000	5.339E+06	4.994E+03
Bordetella parapertussis	NC_002928	7.172E-01	6.631E-03	6.650E+00	6.285E-01	-9.487E+05	4.493E-01	8.424E-03	9.595E-01	6.092E+02	-9.490E+05	-6.03E+02	exponential-log normal	6.738E+00	5.695E-01	-1.874E+04	3.280E+00	3.025E+02	-1.878E+04	1.383E+05	0.160	0.338	0.159	0.343	0.000	4.774E+06	4.185E+03
Bordetella pertussis	NC_002929	7.181E-01	7.280E-03	6.610E+00	6.382E-01	-8.048E+05	4.831E-01	9.035E-03	9.800E-01	5.953E+02	-8.051E+05	-5.32E+02	exponential-log normal	6.701E+00	5.631E-01	-4.814E+03	3.243E+00	2.959E+02	-4.840E+03	1.184E+05	0.161	0.337	0.161	0.340	0.000	4.086E+06	3.436E+03
Borrelia afzelii	NC_008277	8.359E-01	2.510E-02	6.409E+00	7.683E-01	-1.497E+05	8.008E-01	2.671E-02	1.122E+00	6.057E+02	-1.497E+05	3.66E+01	exponential-gamma	6.683E+00	7.013E-01	-3.051E+03	2.582E+00	3.784E+02	-3.029E+03	2.676E+04	0.360	0.143	0.357	0.140	0.000	9.054E+05	8.550E+02
Borrelia burgdorferi	NC_001318	8.355E-01	2.486E-02	6.408E+00	7.629E-01	-1.514E+05	8.013E-01	2.641E-02	1.146E+00	5.930E+02	-1.514E+05	3.14E+01	exponential-gamma	6.664E+00	7.428E-01	-5.143E+03	2.240E+00	4.428E+02	-5.122E+03	2.701E+04	0.359	0.144	0.355	0.142	0.000	9.107E+05	8.510E+02
Borrelia garinii	NC_006156	8.372E-01	2.526E-02	6.405E+00	7.650E-01	-1.495E+05	8.031E-01	2.682E-02	1.142E+00	5.929E+02	-1.495E+05	3.77E+01	exponential-gamma	6.716E+00	6.657E-01	-2.952E+03	2.805E+00	3.553E+02	-2.933E+03	2.676E+04	0.360	0.143	0.357	0.140	0.000	9.042E+05	8.320E+02
Bradyrhizobium ORS278	NC_009445	7.467E-01	7.936E-03	6.599E+00	6.313E-01	-1.449E+06	5.282E-01	9.725E-03	9.544E-01	5.941E+02	-1.449E+06	-8.31E+02	exponential-log normal	6.645E+00	6.799E-01	-3.722E+04	2.504E+00	3.784E+02	-3.719E+04	2.170E+05	0.172	0.327	0.172	0.328	0.000	7.457E+06	6.717E+03
Buchnera aphidicola	NC_004545	8.563E-01	3.097E-02	6.397E+00	7.777E-01	-1.189E+05	8.356E-01	3.237E-02	1.266E+00	5.578E+02	-1.188E+05	1.06E+02	exponential-gamma	6.752E+00	6.193E-01	-1.101E+03	3.057E+00	3.332E+02	-1.097E+03	2.223E+04	0.375	0.127	0.371	0.127	0.000	6.160E+05	5.040E+02
Buchnera sp	NC_002528	8.469E-01	2.939E-02	6.438E+00	7.535E-01	-1.190E+05	8.274E-01	3.075E-02	1.386E+00	5.284E+02	-1.190E+05	1.09E+02	exponential-gamma	6.681E+00	6.706E-01	-2.771E+03	2.505E+00	3.938E+02	-2.775E+03	2.188E+04	0.366	0.131	0.371	0.132	0.000	6.407E+05	5.640E+02
Caldicellulosiruptor saccharolyticus	NC_009437	8.201E-01	2.288E-02	6.247E+00	7.982E-01	-5.676E+05	7.525E-01	2.476E-02	8.674E-01	6.095E+02	-5.675E+05	3.69E+02	exponential-gamma	6.699E+00	6.240E-01	-9.464E+03	2.891E+00	3.364E+02	-9.461E+03	9.987E+04	0.316	0.166	0.331	0.187	0.000	2.970E+06	2.679E+03
Campylobacter fetus	NC_008599	7.847E-01	2.778E-02	6.259E+00	8.098E-01	-3.097E+05	7.330E-01	3.009E-02	9.945E-01	5.917E+02	-3.096E+05	2.05E+02	exponential-gamma	6.577E+00	6.402E-01	-5.059E+03	2.750E+00	3.176E+02	-5.060E+03	5.422E+04	0.329	0.165	0.338	0.169	0.000	1.774E+06	1.719E+03
Campylobacter jejuni	NC_008787	8.190E-01	2.702E-02	6.334E+00	7.539E-01	-2.997E+05	7.862E-01	2.880E-02	1.235E+00	5.161E+02	-2.996E+05	1.58E+02	exponential-gamma	6.584E+00	6.949E-01	-7.197E+03	2.460E+00	3.662E+02	-7.180E+03	5.352E+04	0.346	0.154	0.348	0.152	0.000	1.617E+06	1.653E+03
Candidatus Blochmannia	NC_005061	8.573E-01	3.012E-02	6.459E+00	7.351E-01	-1.527E+05	8.411E-01	3.137E-02	1.467E+00	5.110E+02	-1.527E+05	9.08E+01	exponential-gamma	6.707E+00	6.704E-01	-2.094E+03	2.511E+00	4.030E+02	-2.095E+03	2.842E+04	0.362	0.132	0.364	0.142	0.000	7.056E+05	5.830E+02
Candidatus Carsonella	NC_008512	8.472E-01	3.439E-02	6.290E+00	8.830E-01	-1.893E+04	8.158E-01	3.594E-02	9.061E-01	7.198E+02	-1.892E+04	1.66E+01	exponential-gamma	6.440E+00	7.497E-01	-1.219E+03	2.013E+00	4.067E+02	-1.222E+03	3.568E+03	0.416	0.085	0.418	0.081	0.000	1.597E+05	1.820E+02
Candidatus Pelagibacter	NC_007205	8.229E-01	2.764E-02	6.326E+00	7.588E-01	-2.456E+05	7.642E-01	3.004E-02	8.461E-01	7.004E+02	-2.459E+05	-7.76E+02	exponential-log normal	6.683E+00	5.975E-01	-2.891E+03	3.031E+00	3.132E+02	-2.896E+03	4.410E+04	0.350	0.148	0.353	0.149	0.000	1.309E+06	1.354E+03
Candidatus Ruthia	NC_008610	8.449E-01	2.175E-02	6.399E+00	7.421E-01	-2.458E+05	8.042E-01	2.312E-02	1.103E+00	5.836E+02	-2.458E+05	-1.57E+01	exponential-log normal	6.656E+00	6.278E-01	-4.907E+03	2.801E+00	3.346E+02	-4.913E+03	4.345E+04	0.330	0.169	0.330	0.171	0.000	1.161E+06	9.760E+02
Candidatus Vesicomyosocius	NC_009465	8.257E-01	2.494E-02	6.376E+00	7.524E-01	-2.043E+05	7.907E-01	2.658E-02	1.198E+00	5.485E+02	-2.043E+05	8.30E+01	exponential-gamma	6.666E+00	6.362E-01	-3.877E+03	2.705E+00	3.521E+02	-3.884E+03	3.625E+04	0.340	0.158	0.344	0.158	0.000	1.022E+06	9.370E+02
Carboxydothermus hydrogenoformans	NC_007503	7.830E-01	2.006E-02	6.305E+00	7.174E-01	-3.593E+05	7.245E-01	2.209E-02	1.209E+00	4.737E+02	-3.592E+05	2.48E+02	exponential-gamma	6.423E+00	8.083E-01	-1.487E+04	1.952E+00	4.178E+02	-1.479E+04	6.066E+04	0.289	0.210	0.290	0.210	0.000	2.402E+06	2.620E+03
Caulobacter crescentus	NC_002696	7.446E-01	7.821E-03	6.628E+00	6.089E-01	-6.022E+05	5.623E-01	9.869E-03	1.163E+00	5.303E+02	-6.022E+05	4.80E+01	exponential-gamma	6.660E+00	6.764E-01	-1.907E+04	2.524E+00	3.823E+02	-1.905E+04	8.991E+04	0.163	0.337	0.165	0.335	0.000	4.017E+06	3.737E+03
Chlamydia muridarum	NC_002620	8.296E-01	2.025E-02	6.443E+00	8.769E-01	-1.948E+05	7.522E-01	2.126E-02	6.229E-01	1.062E+03	-1.949E+05	-2.87E+02	exponential-log normal	6.692E+00	7.271E-01	-5.635E+03	2.212E+00	4.642E+02	-5.631E+03	3.362E+04	0.298	0.201	0.299	0.202	0.000	1.073E+06	9.040E+02
Chlamydia trachomatis	NC_000117	8.378E-01	1.935E-02	6.497E+00	7.287E-01	-1.862E+05	7.997E-01	2.062E-02	1.207E+00	5.929E+02	-1.862E+05	5.63E+01	exponential-gamma	6.737E+00	6.741E-01	-5.488E+03	2.444E+00	4.290E+02	-5.498E+03	3.214E+04	0.293	0.206	0.294	0.207	0.000	1.043E+06	8.950E+02
Chlamydophila abortus	NC_004552	8.474E-01	2.061E-02	6.478E+00	7.357E-01	-2.152E+05	8.129E-01	2.185E-02	1.200E+00	5.891E+02	-2.152E+05	7.83E+01	exponential-gamma	6.733E+00	6.369E-01	-3.636E+03	2.625E+00	3.923E+02	-3.651E+03	3.775E+04	0.300	0.200	0.301	0.199	0.000	1.144E+06	9.320E+02
Chlamydophila caviae	NC_003361	8.417E-01	2.192E-02	6.473E+00	7.886E-01	-2.165E+05	7.945E-01	2.335E-02	9.250E-01	7.550E+02	-2.166E+05	-8.18E+01	exponential-log normal	6.640E+00	7.289E-01	-4.371E+03	2.137E+00	4.617E+02	-4.376E+03	3.808E+04	0.303	0.196	0.304	0.196	0.000	1.173E+06	9.980E+02
Chlamydophila felis	NC_007899	8.377E-01	2.119E-02	6.466E+00	7.790E-01	-2.076E+05	7.868E-01	2.263E-02	9.201E-01	7.436E+02	-2.077E+05	-1.15E+02	exponential-log normal	6.746E+00	6.950E-01	-5.291E+03	2.336E+00	4.578E+02	-5.296E+03	3.626E+04	0.304	0.196	0.303	0.197	0.000	1.166E+06	1.005E+03
Chlamydophila pneumoniae	NC_002179	8.507E-01	1.999E-02	6.494E+00	7.239E-01	-2.181E+05	8.178E-01	2.118E-02	1.252E+00	5.729E+02	-2.180E+05	7.16E+01	exponential-gamma	6.655E+00	7.306E-01	-3.625E+03	2.182E+00	4.547E+02	-3.623E+03	3.818E+04	0.299	0.203	0.296	0.203	0.000	1.230E+06	1.112E+03
Chlorobium phaeobacteroides	NC_008639	7.868E-01	1.348E-02	6.279E+00	8.657E-01	-6.921E+05	6.960E-01	1.329E-02	6.707E-01	8.445E+02	-6.926E+05	-1.02E+03	exponential-log normal	6.667E+00	6.819E-01	-1.429E+04	2.233E+00	4.469E+02	-1.439E+04	1.124E+05	0.260	0.245	0.256	0.238	0.000	3.134E+06	2.650E+03
Chlorobium tepidum	NC_002932	7.743E-01	1.003E-02	6.499E+00	6.617E-01	-4.415E+05	6.268E-01	1.160E-02	1.000E+00	5.601E+02	-4.415E+05	1.17E+01	exponential-gamma	6.425E+00	8.537E-01	-1.259E+04	1.753E+00	4.809E+02	-1.255E+04	6.864E+04	0.216	0.284	0.219	0.281	0.000	2.155E+06	2.252E+03
Chromobacterium violaceum	NC_005085	6.727E-01	9.172E-03	6.494E+00	7.119E-01	-8.723E+05	4.568E-01	1.065E-02	8.557E-01	6.658E+02	-8.727E+05	-6.88E+02	exponential-log normal	6.675E+00	6.394E-01	-1.065E+04	2.582E+00	3.769E+02	-1.070E+04	1.292E+05	0.176	0.324	0.175	0.324	0.000	4.751E+06	4.407E+03
Chromohalobacter salexigens	NC_007963	7.587E-01	8.497E-03	6.599E+00	5.951E-01	-8.023E+05	5.942E-01	1.059E-02	1.203E+00	5.006E+02	-8.024E+05	-1.23E+02	exponential-log normal	6.748E+00	5.956E-01	-1.421E+04	3.038E+00	3.336E+02	-1.423E+04	1.215E+05	0.181	0.318	0.180	0.321	0.000	3.697E+06	3.298E+03
Clavibacter michiganensis	NC_009480	7.410E-01	5.129E-03	6.751E+00	6.375E-01	-4.120E+05	4.522E-01	5.945E-03	9.059E-01	6.839E+02	-4.120E+05	-1.01E+02	exponential-log normal	6.716E+00	6.000E-01	-1.263E+04	3.054E+00	3.211E+02	-1.264E+04	5.897E+04	0.138	0.363	0.136	0.364	0.000	3.298E+06	2.984E+03
Clostridium acetobutylicum	NC_003030	8.110E-01	2.952E-02	6.229E+00	8.512E-01	-7.989E+05	7.516E-01	3.178E-02	8.029E-01	6.990E+02	-7.989E+05	4.02E+01	exponential-gamma	6.633E+00	6.489E-01	-1.093E+04	2.592E+00	3.599E+02	-1.095E+04	1.438E+05	0.345	0.154	0.346	0.155	0.000	3.941E+06	3.672E+03
Clostridium beijerinckii	NC_009617	8.370E-01	2.956E-02	6.296E+00	8.074E-01	-1.247E+06	7.961E-01	3.153E-02	9.615E-01	6.322E+02	-1.247E+06	5.58E+01	exponential-gamma	6.651E+00	6.467E-01	-2.524E+04	2.642E+00	3.568E+02	-2.528E+04	2.288E+05	0.348	0.148	0.354	0.151	0.000	6.001E+06	5.020E+03
Clostridium novyi	NC_008593	8.109E-01	3.219E-02	6.248E+00	8.305E-01	-5.201E+05	7.668E-01	3.462E-02	9.525E-01	6.217E+02	-5.199E+05	3.27E+02	exponential-gamma	6.673E+00	6.677E-01	-1.308E+04	2.613E+00	3.710E+02	-1.306E+04	9.450E+04	0.353	0.144	0.358	0.145	0.000	2.548E+06	2.315E+03
Clostridium perfringens	NC_003366	8.171E-01	3.387E-02	6.259E+00	8.359E-01	-5.800E+05	7.800E-01	3.606E-02	9.893E-01	6.201E+02	-5.798E+05	3.96E+02	exponential-gamma	6.640E+00	6.570E-01	-6.754E+03	2.543E+00	3.713E+02	-6.770E+03	1.065E+05	0.365	0.147	0.350	0.138	0.000	3.031E+06	2.660E+03
Clostridium tetani	NC_004557	7.966E-01	3.390E-02	6.173E+00	8.524E-01	-5.506E+05	7.469E-01	3.662E-02	9.058E-01	6.081E+02	-5.503E+05	5.55E+02	exponential-gamma	6.780E+00	5.225E-01	-4.855E+03	3.667E+00	2.764E+02	-4.882E+03	9.987E+04	0.359	0.146	0.353	0.141	0.000	2.799E+06	2.373E+03
Clostridium thermocellum	NC_009012	7.709E-01	2.055E-02	6.134E+00	9.515E-01	-7.994E+05	6.459E-01	2.056E-02	4.923E-01	9.782E+02	-7.999E+05	-1.16E+03	exponential-log normal	6.679E+00	6.704E-01	-6.483E+03	2.248E+00	4.487E+02	-6.538E+03	1.352E+05	0.306	0.195	0.305	0.194	0.000	3.843E+06	3.191E+03
Colwellia psychrerythraea	NC_003910	8.434E-01	2.275E-02	6.456E+00	7.587E-01	-1.088E+06	8.073E-01	2.413E-02	1.108E+00	6.339E+02	-1.088E+06	4.32E+01	exponential-gamma	6.507E+00	8.577E-01	-1.985E+04	1.735E+00	5.314E+02	-1.978E+04	1.927E+05	0.310	0.190	0.310	0.190	0.000	5.373E+06	4.910E+03
Corynebacterium diphtheriae	NC_002935	7.968E-01	1.271E-02	6.402E+00	7.663E-01	-4.983E+05	6.759E-01	1.351E-02	7.532E-01	7.231E+02	-4.984E+05	-2.48E+02	exponential-log normal	6.614E+00	6.965E-01	-1.226E+04	2.404E+00	3.897E+02	-1.225E+04	8.040E+04	0.233	0.267	0.232	0.267	0.000	2.489E+06	2.272E+03
Corynebacterium efficiens	NC_004369	7.941E-01	7.979E-03	6.529E+00	6.704E-01	-6.533E+05	6.233E-01	8.706E-03	8.843E-01	6.141E+02	-6.535E+05	-2.91E+02	exponential-log normal	6.684E+00	6.737E-01	-6.135E+03	2.496E+00	3.964E+02	-6.138E+03	1.001E+05	0.185	0.315	0.184	0.316	0.000	3.147E+06	2.950E+03
Corynebacterium glutamicum	NC_003450	8.108E-01	1.154E-02	6.419E+00	7.443E-01	-6.672E+05	6.994E-01	1.220E-02	8.127E-01	6.791E+02	-6.674E+05	-3.09E+02	exponential-log normal	6.633E+00	6.626E-01	-1.014E+04	2.517E+00	3.724E+02	-1.017E+04	1.073E+05	0.231	0.270	0.231	0.268	0.000	3.309E+06	2.993E+03
Corynebacterium jeikeium	NC_007164	7.507E-01	9.154E-03	6.519E+00	7.008E-01	-3.995E+05	5.659E-01	1.019E-02	8.243E-01	6.774E+02	-3.996E+05	-2.33E+02	exponential-log normal	6.751E+00	6.428E-01	-1.141E+04	2.734E+00	3.793E+02	-1.141E+04	6.086E+04	0.194	0.308	0.192	0.306	0.000	2.462E+06	2.104E+03
Coxiella burnetii	NC_002971	8.354E-01	1.793E-02	6.466E+00	6.833E-01	-3.649E+05	7.956E-01	1.923E-02	1.338E+00	5.038E+02	-3.648E+05	4.65E+01	exponential-gamma	6.420E+00	8.401E-01	-8.614E+03	1.784E+00	4.689E+02	-8.585E+03	6.245E+04	0.286	0.213	0.287	0.213	0.000	1.995E+06	2.016E+03
Cytophaga hutchinsonii	NC_008255	8.336E-01	2.018E-02	6.330E+00	9.211E-01	-9.641E+05	7.463E-01	2.084E-02	5.468E-01	1.054E+03	-9.643E+05	-5.38E+02	exponential-log normal	6.679E+00	7.190E-01	-1.115E+04	1.973E+00	5.291E+02	-1.126E+04	1.678E+05	0.305	0.194	0.307	0.194	0.000	4.433E+06	3.785E+03
Dechloromonas aromatica	NC_007298	7.800E-01	9.244E-03	6.553E+00	6.717E-01	-9.269E+05	6.322E-01	1.048E-02	9.601E-01	6.158E+02	-9.270E+05	-2.07E+02	exponential-log normal	6.689E+00	6.474E-01	-1.984E+04	2.530E+00	3.911E+02	-1.993E+04	1.429E+05	0.204	0.296	0.203	0.296	0.000	4.501E+06	4.171E+03
Dehalococcoides BAV1	NC_009455	7.536E-01	1.571E-02	6.250E+00	7.466E-01	-2.492E+05	6.344E-01	1.749E-02	8.937E-01	5.476E+02	-2.492E+05	3.33E+01	exponential-gamma	6.624E+00	6.079E-01	-1.033E+03	3.122E+00	2.850E+02	-1.031E+03	4.052E+04	0.265	0.238	0.264	0.234	0.000	1.342E+06	1.371E+03
Dehalococcoides CBDB1	NC_007356	7.592E-01	1.563E-02	6.286E+00	7.406E-01	-2.595E+05	6.495E-01	1.744E-02	9.373E-01	5.475E+02	-2.595E+05	8.59E+01	exponential-gamma	6.512E+00	7.094E-01	-2.965E+03	2.336E+00	3.607E+02	-2.961E+03	4.221E+04	0.265	0.235	0.265	0.236	0.000	1.396E+06	1.458E+03
Dehalococcoides ethenogenes	NC_002936	7.443E-01	1.509E-02	6.283E+00	7.482E-01	-2.631E+05	6.164E-01	1.694E-02	8.807E-01	5.688E+02	-2.631E+05	6.31E+01	exponential-gamma	6.473E+00	7.539E-01	-6.805E+03	2.095E+00	4.007E+02	-6.797E+03	4.235E+04	0.257	0.242	0.255	0.247	0.000	1.470E+06	1.580E+03
Deinococcus geothermalis	NC_008025	7.329E-01	8.493E-03	6.543E+00	6.111E-01	-3.063E+05	5.353E-01	1.089E-02	1.123E+00	4.986E+02	-3.063E+05	2.42E+00	exponential-gamma	6.684E+00	5.939E-01	-7.517E+03	3.074E+00	3.087E+02	-7.527E+03	4.609E+04	0.167	0.334	0.166	0.333	0.000	2.467E+06	2.335E+03
Desulfitobacterium hafniense	NC_007907	8.124E-01	1.463E-02	6.434E+00	6.878E-01	-1.220E+06	7.357E-01	1.617E-02	1.105E+00	5.386E+02	-1.220E+06	1.09E+02	exponential-gamma	6.668E+00	6.073E-01	-2.586E+04	2.934E+00	3.217E+02	-2.592E+04	2.014E+05	0.265	0.246	0.261	0.227	0.000	5.728E+06	5.060E+03
Desulfotalea psychrophila	NC_006138	8.109E-01	1.371E-02	6.435E+00	7.309E-01	-7.273E+05	7.131E-01	1.489E-02	8.694E-01	6.629E+02	-7.275E+05	-4.33E+02	exponential-log normal	6.635E+00	6.983E-01	-1.192E+04	2.308E+00	4.152E+02	-1.194E+04	1.190E+05	0.263	0.236	0.269	0.232	0.000	3.523E+06	3.116E+03
Desulfotomaculum reducens	NC_009253	8.102E-01	1.812E-02	6.356E+00	7.114E-01	-7.177E+05	7.457E-01	1.986E-02	1.109E+00	5.206E+02	-7.176E+05	1.34E+02	exponential-gamma	6.646E+00	6.382E-01	-1.164E+04	2.745E+00	3.401E+02	-1.164E+04	1.216E+05	0.288	0.210	0.290	0.213	0.000	3.608E+06	3.276E+03
Desulfovibrio desulfuricans	NC_007519	7.641E-01	8.860E-03	6.505E+00	6.787E-01	-9.042E+05	5.796E-01	9.980E-03	8.734E-01	6.170E+02	-9.044E+05	-3.70E+02	exponential-log normal	6.474E+00	8.417E-01	-2.196E+04	1.764E+00	4.999E+02	-2.191E+04	1.383E+05	0.210	0.288	0.211	0.290	0.000	3.730E+06	3.775E+03
Dichelobacter nodosus	NC_009446	8.243E-01	1.590E-02	6.513E+00	7.005E-01	-2.837E+05	7.686E-01	1.723E-02	1.147E+00	5.948E+02	-2.837E+05	-4.00E+01	exponential-log normal	6.655E+00	6.903E-01	-5.375E+03	2.434E+00	3.979E+02	-5.373E+03	4.739E+04	0.280	0.225	0.277	0.219	0.000	1.389E+06	1.280E+03
Ehrlichia canis	NC_007354	8.621E-01	3.073E-02	6.211E+00	1.004E+00	-2.818E+05	7.853E-01	3.170E-02	4.385E-01	1.228E+03	-2.819E+05	-2.84E+02	exponential-log normal	6.772E+00	6.344E-01	-2.714E+03	2.556E+00	4.199E+02	-2.732E+03	5.319E+04	0.355	0.146	0.356	0.144	0.000	1.315E+06	9.250E+02
Ehrlichia chaffeensis	NC_007799	8.518E-01	2.990E-02	6.234E+00	9.620E-01	-2.585E+05	7.780E-01	3.123E-02	5.083E-01	1.079E+03	-2.586E+05	-1.84E+02	exponential-log normal	6.340E+00	9.829E-01	-3.811E+03	1.327E+00	6.519E+02	-3.808E+03	4.814E+04	0.348	0.148	0.351	0.153	0.000	1.176E+06	1.105E+03
Ehrlichia ruminantium	NC_006831	8.857E-01	3.131E-02	6.302E+00	9.351E-01	-3.114E+05	8.337E-01	3.259E-02	5.517E-01	1.076E+03	-3.115E+05	-2.06E+02	exponential-log normal	6.656E+00	6.996E-01	-4.022E+03	2.200E+00	4.529E+02	-4.043E+03	6.011E+04	0.361	0.139	0.364	0.136	0.000	1.500E+06	9.500E+02
Enterobacter 638	NC_009436	7.669E-01	1.285E-02	6.444E+00	6.789E-01	-8.887E+05	6.484E-01	1.483E-02	1.023E+00	5.556E+02	-8.888E+05	-1.88E+02	exponential-log normal	6.676E+00	6.316E-01	-1.294E+04	2.761E+00	3.471E+02	-1.296E+04	1.412E+05	0.235	0.265	0.235	0.264	0.000	4.519E+06	4.115E+03
Enterococcus faecalis	NC_004668	8.063E-01	2.183E-02	6.384E+00	7.702E-01	-6.319E+05	7.492E-01	2.357E-02	9.888E-01	6.426E+02	-6.320E+05	-1.10E+02	exponential-log normal	6.496E+00	7.766E-01	-7.242E+03	1.927E+00	4.542E+02	-7.255E+03	1.086E+05	0.314	0.188	0.311	0.187	0.000	3.218E+06	3.113E+03

Erwinia carotovora	NC_004547	7.861E-01	1.506E-02	6.364E+00	8.027E-01	-1.024E+06	6.555E-01	1.582E-02	6.239E-01	8.666E+02	-1.026E+06	-2.98E+03	exponential-log normal	6.658E+00	6.677E-01	-1.894E+04	2.411E+00	4.029E+02	-1.901E+04	1.673E+05	0.245	0.255	0.246	0.255	0.000	5.064E+06	4.472E+03
Erythrobacter litoralis	NC_007722	7.516E-01	7.700E-03	6.580E+00	6.177E-01	-5.952E+05	6.039E-01	9.219E-03	1.232E+00	5.044E+02	-5.952E+05	-4.03E+01	exponential-log normal	6.625E+00	7.084E-01	-7.775E+03	2.360E+00	3.990E+02	-7.765E+03	8.927E+04	0.184	0.315	0.185	0.316	0.000	3.052E+06	3.011E+03
Escherichia coli	NC_008253	7.855E-01	1.405E-02	6.439E+00	7.223E-01	-1.018E+06	6.742E-01	1.565E-02	8.798E-01	6.532E+02	-1.018E+06	-6.04E+02	exponential-log normal	6.644E+00	7.076E-01	-1.364E+04	2.224E+00	4.379E+02	-1.367E+04	1.646E+05	0.247	0.253	0.248	0.252	0.000	4.939E+06	4.629E+03
Flavobacterium johnsoniae	NC_009441	8.277E-01	2.336E-02	6.417E+00	8.456E-01	-1.073E+06	7.674E-01	2.489E-02	7.651E-01	8.649E+02	-1.074E+06	-4.54E+02	exponential-log normal	6.743E+00	6.648E-01	-2.593E+04	2.346E+00	4.530E+02	-2.609E+04	1.885E+05	0.329	0.173	0.330	0.168	0.000	6.097E+06	5.017E+03
Flavobacterium psychrophilum	NC_009613	8.149E-01	2.490E-02	6.317E+00	8.383E-01	-4.951E+05	7.510E-01	2.672E-02	7.929E-01	7.525E+02	-4.951E+05	-6.92E+01	exponential-log normal	6.676E+00	7.147E-01	-9.964E+03	2.160E+00	4.706E+02	-9.996E+03	8.722E+04	0.342	0.165	0.332	0.161	0.000	2.862E+06	2.412E+03
Francisella tularensis	NC_008245	8.398E-01	2.665E-02	6.329E+00	7.292E-01	-3.616E+05	8.112E-01	2.814E-02	1.303E+00	4.811E+02	-3.616E+05	7.63E+01	exponential-gamma	6.682E+00	6.213E-01	-7.700E+03	2.936E+00	3.255E+02	-7.696E+03	6.557E+04	0.337	0.159	0.340	0.163	0.000	1.893E+06	1.605E+03
Frankia alni	NC_008278	4.758E-01	9.370E-03	6.229E+00	8.870E-01	-7.655E+05	4.648E-01	4.625E-03	6.353E-01	1.012E+03	-7.665E+05	-1.89E+03	exponential-log normal	6.595E+00	7.890E-01	-3.202E+04	1.868E+00	5.224E+02	-3.208E+04	1.090E+05	0.136	0.364	0.136	0.365	0.000	7.498E+06	6.711E+03
Frankia CcI3	NC_007777	6.624E-01	7.483E-03	6.438E+00	7.718E-01	-6.659E+05	4.937E-01	6.525E-03	7.405E-01	7.959E+02	-6.663E+05	-9.30E+02	exponential-log normal	6.710E+00	6.507E-01	-1.696E+04	2.574E+00	3.908E+02	-1.701E+04	9.731E+04	0.149	0.350	0.150	0.350	0.000	5.434E+06	4.499E+03
Fusobacterium nucleatum	NC_003454	8.062E-01	3.231E-02	6.320E+00	8.578E-01	-3.952E+05	7.511E-01	3.484E-02	7.832E-01	8.153E+02	-3.954E+05	-4.90E+02	exponential-log normal	6.655E+00	6.719E-01	-9.008E+03	2.496E+00	3.839E+02	-9.015E+03	7.122E+04	0.370	0.140	0.358	0.132	0.000	2.175E+06	2.067E+03
Geobacillus kaustophilus	NC_006509	8.154E-01	1.629E-02	6.306E+00	6.558E-01	-9.989E+03	7.587E-01	1.785E-02	1.402E+00	3.890E+02	-9.986E+03	6.11E+00	exponential-gamma	6.636E+00	6.139E-01	-3.177E+02	3.083E+00	2.929E+02	-3.169E+02	1.684E+03	0.284	0.225	0.275	0.217	0.000	4.789E+04	
Geobacillus thermodenitrificans	NC_009328	8.299E-01	1.242E-02	6.417E+00	6.453E-01	-8.288E+05	7.382E-01	1.384E-02	1.126E+00	4.789E+02	-8.288E+05	3.37E+01	exponential-gamma	6.556E+00	6.623E-01	-1.291E+04	2.562E+00	3.380E+02	-1.291E+04	1.360E+05	0.254	0.246	0.255	0.244	0.000	3.550E+06	3.392E+03
Geobacter metallireducens	NC_007517	7.557E-01	9.620E-03	6.509E+00	6.962E-01	-8.073E+05	5.850E-01	1.085E-02	8.638E-01	6.511E+02	-8.075E+05	-3.45E+02	exponential-log normal	6.730E+00	6.551E-01	-1.364E+04	2.556E+00	4.023E+02	-1.367E+04	1.239E+05	0.201	0.299	0.204	0.296	0.000	3.997E+06	3.519E+03
Geobacter sulfurreducens	NC_002939	7.610E-01	9.363E-03	6.528E+00	7.001E-01	-7.647E+05	5.643E-01	1.121E-02	8.336E-01	6.688E+02	-7.650E+05	-6.95E+02	exponential-log normal	6.671E+00	7.281E-01	-9.958E+03	2.204E+00	4.576E+02	-9.955E+03	1.171E+05	0.196	0.304	0.195	0.305	0.000	3.814E+06	3.446E+03
Geobacter uraniumreducens	NC_009483	7.041E-01	1.334E-02	6.111E+00	9.451E-01	-1.092E+06	6.083E-01	1.158E-02	5.564E-01	9.652E+02	-1.093E+06	-3.21E+03	exponential-log normal	6.682E+00	7.171E-01	-1.499E+04	1.966E+00	5.342E+02	-1.514E+04	1.723E+05	0.229	0.273	0.228	0.270	0.000	5.136E+06	4.357E+03
Gloeobacter violaceus	NC_005125	7.225E-01	9.874E-03	6.386E+00	7.482E-01	-5.879E+05	5.297E-01	1.035E-02	7.488E-01	6.773E+02	-5.880E+05	-2.35E+02	exponential-log normal	6.607E+00	6.961E-01	-2.523E+04	2.282E+00	4.092E+02	-2.531E+04	8.984E+04	0.189	0.310	0.191	0.310	0.000	4.659E+06	4.430E+03
Gluconobacter oxydans	NC_006677	7.695E-01	8.919E-03	6.556E+00	6.243E-01	-6.231E+05	6.139E-01	1.066E-02	1.120E+00	5.223E+02	-6.231E+05	1.31E+01	exponential-gamma	6.743E+00	5.929E-01	-1.335E+04	3.093E+00	3.257E+02	-1.336E+04	9.538E+04	0.196	0.301	0.193	0.310	0.000	2.702E+06	2.432E+03
Gramella forsetii	NC_008571	8.217E-01	2.189E-02	6.393E+00	7.946E-01	-7.192E+05	7.652E-01	2.347E-02	9.053E-01	7.047E+02	-7.192E+05	-4.72E+01	exponential-log normal	6.629E+00	7.639E-01	-1.506E+04	1.966E+00	5.069E+02	-1.509E+04	1.250E+05	0.320	0.184	0.314	0.182	0.000	3.798E+06	3.584E+03
Granulobacter bethesdensis	NC_008343	7.790E-01	9.983E-03	6.473E+00	6.868E-01	-6.686E+05	6.320E-01	1.105E-02	9.088E-01	6.091E+02	-6.689E+05	-5.66E+02	exponential-log normal	6.701E+00	6.833E-01	-1.328E+04	2.410E+00	4.210E+02	-1.330E+04	1.042E+05	0.204	0.296	0.205	0.295	0.000	2.708E+06	2.437E+03
Haemophilus ducreyi	NC_002940	8.232E-01	2.362E-02	6.334E+00	7.680E-01	-3.313E+05	7.671E-01	2.550E-02	9.310E-01	6.374E+02	-3.314E+05	-2.56E+02	exponential-log normal	6.464E+00	8.368E-01	-5.659E+03	1.812E+00	4.840E+02	-5.643E+03	5.837E+04	0.312	0.185	0.305	0.197	0.000	1.699E+06	1.717E+03
Haemophilus influenzae	NC_000907	8.261E-01	2.116E-02	6.418E+00	6.994E-01	-3.479E+05	7.921E-01	2.260E-02	1.380E+00	4.829E+02	-3.478E+05	1.17E+02	exponential-gamma	6.645E+00	6.274E-01	-5.525E+03	2.850E+00	3.261E+02	-5.527E+03	6.047E+04	0.308	0.192	0.310	0.190	0.000	1.830E+06	1.657E+03
Haemophilus somnus	NC_008309	8.223E-01	2.270E-02	6.372E+00	8.290E-01	-3.890E+05	7.357E-01	2.432E-02	6.285E-01	9.404E+02	-3.895E+05	-1.02E+03	exponential-log normal	6.683E+00	6.670E-01	-1.037E+04	2.198E+00	4.642E+02	-1.048E+04	6.797E+04	0.314	0.186	0.314	0.186	0.000	2.008E+06	1.792E+03
Hahella chejuensis	NC_007645	7.627E-01	1.371E-02	6.306E+00	8.400E-01	-1.406E+06	6.456E-01	1.387E-02	6.747E-01	8.128E+02	-1.407E+06	-1.92E+03	exponential-log normal	6.541E+00	8.256E-01	-2.569E+04	1.751E+00	5.397E+02	-2.571E+04	2.256E+05	0.231	0.270	0.231	0.269	0.000	7.215E+06	6.778E+03
Halorhodospira halophila	NC_008789	7.464E-01	7.816E-03	6.611E+00	6.424E-01	-3.920E+05	5.441E-01	9.434E-03	9.821E-01	5.998E+02	-3.920E+05	-2.14E+01	exponential-log normal	6.766E+00	6.221E-01	-1.099E+04	2.831E+00	3.687E+02	-1.101E+04	5.858E+04	0.155	0.355	0.165	0.325	0.000	2.678E+06	2.407E+03
Helicobacter hepaticus	NC_004917	8.172E-01	2.036E-02	6.361E+00	7.643E-01	-3.729E+05	7.509E-01	2.218E-02	9.322E-01	6.308E+02	-3.729E+05	5.31E+01	exponential-gamma	6.550E+00	7.602E-01	-6.964E+03	2.078E+00	4.344E+02	-6.957E+03	6.423E+04	0.319	0.182	0.322	0.177	0.000	1.799E+06	1.875E+03
Helicobacter pylori	NC_000915	8.206E-01	2.106E-02	6.342E+00	8.005E-01	-3.253E+05	7.548E-01	2.259E-02	8.464E-01	6.996E+02	-3.254E+05	-1.18E+02	exponential-log normal	6.622E+00	7.364E-01	-6.266E+03	2.208E+00	4.337E+02	-6.252E+03	5.641E+04	0.308	0.196	0.303	0.193	0.000	1.668E+06	1.576E+03
Herminiimonas arsenicoxydans	NC_009138	7.789E-01	1.141E-02	6.382E+00	7.175E-01	-8.297E+05	6.435E-01	1.244E-02	8.684E-01	5.986E+02	-8.298E+05	-3.25E+02	exponential-log normal	6.593E+00	6.944E-01	-1.966E+04	2.357E+00	3.889E+02	-1.967E+04	1.316E+05	0.228	0.272	0.229	0.271	0.000	3.424E+06	3.325E+03
Hyperthermus butylicus	NC_008818	7.934E-01	1.752E-02	6.037E+00	8.277E-01	-2.888E+05	6.696E-01	1.792E-02	6.494E-01	5.957E+02	-2.888E+05	-6.35E+00	exponential-log normal	6.581E+00	5.984E-01	-5.352E+03	3.013E+00	2.849E+02	-5.364E+03	4.910E+04	0.230	0.268	0.233	0.269	0.000	1.667E+06	1.602E+03
Hyphomonas neptunium	NC_008358	7.730E-01	8.941E-03	6.529E+00	6.298E-01	-7.907E+05	6.028E-01	1.067E-02	1.045E+00	5.278E+02	-7.907E+05	-5.55E+01	exponential-log normal	6.648E+00	6.385E-01	-1.454E+04	2.751E+00	3.409E+02	-1.455E+04	1.215E+05	0.191	0.310	0.190	0.309	0.000	3.705E+06	3.505E+03
Idiomarina loihiensis	NC_006512	8.054E-01	1.587E-02	6.533E+00	6.745E-01	-5.275E+05	7.609E-01	1.719E-02	1.417E+00	5.106E+02	-5.274E+05	1.74E+02	exponential-gamma	6.705E+00	6.532E-01	-1.118E+04	2.605E+00	3.849E+02	-1.120E+04	8.703E+04	0.264	0.242	0.265	0.228	0.000	2.839E+06	2.628E+03
Jannaschia CCS1	NC_007802	7.501E-01	9.024E-03	6.494E+00	6.094E-01	-9.317E+05	5.562E-01	1.136E-02	1.093E+00	4.802E+02	-9.318E+05	-1.25E+02	exponential-log normal	6.677E+00	5.917E-01	-1.801E+04	3.091E+00	3.039E+02	-1.804E+04	1.423E+05	0.189	0.308	0.188	0.315	0.000	4.318E+06	4.212E+03
Lactobacillus acidophilus	NC_006814	8.228E-01	2.365E-02	6.369E+00	7.500E-01	-4.196E+05	7.759E-01	2.549E-02	1.062E+00	5.908E+02	-4.197E+05	-1.38E+02	exponential-log normal	6.632E+00	7.268E-01	-6.899E+03	2.093E+00	4.707E+02	-6.925E+03	7.383E+04	0.326	0.171	0.327	0.176	0.000	1.994E+06	1.862E+03
Lactobacillus brevis	NC_008497	8.240E-01	1.761E-02	6.443E+00	6.643E-01	-4.551E+05	7.836E-01	1.898E-02	1.442E+00	4.560E+02	-4.550E+05	5.78E+01	exponential-gamma	6.578E+00	6.823E-01	-8.187E+03	2.517E+00	3.530E+02	-8.171E+03	7.724E+04	0.270	0.238	0.268	0.224	0.000	2.291E+06	2.185E+03
Lactobacillus casei	NC_008526	8.371E-01	1.517E-02	6.450E+00	6.748E-01	-6.510E+05	7.774E-01	1.650E-02	1.180E+00	5.212E+02	-6.510E+05	-3.73E+01	exponential-log normal	6.496E+00	7.549E-01	-1.414E+04	2.110E+00	4.067E+02	-1.412E+04	1.095E+05	0.268	0.233	0.266	0.233	0.000	2.895E+06	2.751E+03
Lactobacillus delbrueckii	NC_008054	7.872E-01	1.391E-02	6.368E+00	7.027E-01	-3.269E+05	6.817E-01	1.555E-02	9.869E-01	5.432E+02	-3.269E+05	6.00E+01	exponential-gamma	6.541E+00	7.132E-01	-4.746E+03	2.371E+00	3.678E+02	-4.728E+03	5.312E+04	0.252	0.249	0.251	0.248	0.000	1.865E+06	1.562E+03
Lactobacillus gasseri	NC_008530	8.152E-01	2.422E-02	6.378E+00	7.657E-01	-3.706E+05	7.643E-01	2.616E-02	9.885E-01	6.445E+02	-3.708E+05	-2.83E+02	exponential-log normal	6.601E+00	6.717E-01	-1.031E+04	2.473E+00	3.691E+02	-1.033E+04	6.497E+04	0.321	0.175	0.326	0.178	0.000	1.894E+06	1.755E+03
Lactobacillus johnsonii	NC_005362	8.145E-01	2.491E-02	6.389E+00	8.047E-01	-3.972E+05	7.556E-01	2.688E-02	8.392E-01	7.680E+02	-3.974E+05	-5.13E+02	exponential-log normal	6.670E+00	6.625E-01	-6.183E+03	2.414E+00	4.052E+02	-6.215E+03	6.973E+04	0.325	0.171	0.329	0.175	0.000	1.993E+06	1.821E+03
Lactobacillus plantarum	NC_004567	8.204E-01	1.863E-02	6.430E+00	7.279E-01	-6.666E+05	7.472E-01	2.035E-02	8.982E-01	6.806E+02	-6.672E+05	-1.17E+03	exponential-log normal	6.626E+00	6.600E-01	-1.449E+04	2.542E+00	3.654E+02	-1.452E+04	1.135E+05	0.278	0.223	0.277	0.222	0.000	3.308E+06	3.007E+03
Lactobacillus reuteri	NC_009513	8.280E-01	2.026E-02	6.428E+00	6.727E-01	-4.231E+05	7.976E-01	2.158E-02	1.546E+00	4.365E+02	-4.230E+05	1.35E+02	exponential-gamma	6.614E+00	6.178E-01	-7.239E+03	2.906E+00	3.071E+02	-7.237E+03	7.323E+04	0.305	0.192	0.306	0.196	0.000	2.000E+06	1.900E+03
Lactobacillus sakei	NC_007576	8.075E-01	1.983E-02	6.421E+00	6.724E-01	-3.631E+05	7.729E-01	2.126E-02	1.520E+00	4.389E+02	-3.631E+05	6.95E+01	exponential-gamma	6.541E+00	7.162E-01	-7.614E+03	2.394E+00	3.627E+02	-7.580E+03	6.185E+04	0.293	0.207	0.294	0.206	0.000	1.885E+06	1.879E+03
Lactobacillus salivarius	NC_007929	8.252E-01	2.661E-02	6.337E+00	7.532E-01	-3.788E+05	7.909E-01	2.838E-02	1.201E+00	5.280E+02	-3.787E+05	1.50E+02	exponential-gamma	6.574E+00	7.030E-01	-6.486E+03	2.350E+00	3.812E+02	-6.480E+03	6.783E+04	0.334	0.164	0.337	0.165	0.000	1.827E+06	1.717E+03
Lactococcus lactis	NC_002662	8.247E-01	2.039E-02	6.394E+00	7.163E-01	-4.592E+05	7.800E-01	2.192E-02	1.193E+00	5.313E+02	-4.592E+05	9.69E+00	exponential-gamma	6.455E+00	7.201E-01	-6.416E+03	2.096E+00	3.931E+02	-6.447E+03	7.944E+04	0.323	0.176	0.324	0.178	0.000	2.366E+06	2.321E+03
Lawsonia intracellularis	NC_008011	8.559E-01	2.452E-02	6.401E+00	7.355E-01	-2.988E+05	8.285E-01	2.582E-02	1.264E+00	5.305E+02	-2.988E+05	1.04E+02	exponential-gamma	6.778E+00	5.542E-01	-3.743E+03	3.376E+00	3.045E+02	-3.759E+03	5.415E+04	0.333	0.165	0.335	0.167	0.000	1.458E+06	1.180E+03
Legionella pneumophila	NC_009494	8.463E-01	2.065E-02	6.450E+00	7.777E-01	-7.918E+05	7.870E-01	2.211E-02	8.292E-01	7.816E+02	-7.922E+05	-9.04E+02	exponential-log normal	6.642E+00	7.042E-01	-9.820E+03	2.306E+00	4.191E+02	-9.828E+03	1.389E+05	0.306	0.197	0.310	0.188	0.000	3.576E+06	3.206E+03
Leifsonia xyli	NC_006087	7.549E-01	7.054E-03	6.549E+00	6.295E-01	-3.437E+05	5.534E-01	8.358E-03	1.085E+00	5.147E+02	-3.437E+05	5.19E+00	exponential-gamma	6.562E+00	7.056E-01	-1.142E+04	2.447E+00	3.598E+02	-1.137E+04	5.136E+04	0.161	0.339	0.162	0.338	0.000	2.584E+06	2.030E+03
Leuconostoc mesenteroides	NC_008531	8.347E-01	2.059E-02	6.414E+00	7.115E-01	-4.524E+05	7.921E-01	2.211E-02	1.191E+00	5.406E+02	-4.525E+05	-8.30E+01	exponential-log normal	6.649E+00	6.534E-01	-1.101E+04	2.729E+00	3.434E+02	-1.098E+04	7.886E+04	0.312	0.187	0.311	0.190	0.000	2.038E+06	1.970E+03
Listeria monocytogenes	NC_003210	7.947E-01	2.097E-02	6.357E+00	7.455E-01	-5.991E+05	7.336E-01	2.296E-02	1.047E+00	5.768E+02	-5.990E+05	2.40E+01	exponential-gamma	6.635E+00	6.254E-01	-1.643E+04	2.759E+00	3.358E+02	-1.648E+04	1.020E+05	0.310	0.191	0.310	0.189	0.000	2.945E+06	2.846E+03
Listeria welshimeri	NC_008555	8.015E-01	2.218E-02	6.328E+00	7.516E-01	-5.824E+05	7.433E-01	2.416E-02	1.034E+00	5.710E+02	-5.824E+05	-1.65E+01	exponential-log normal	6.562E+00	6.815E-01	-1.723E+04	2.450E+00	3.592E+02	-1.724E+04	1.004E+05	0.317	0.182	0.320	0.181	0.000	2.814E+06	2.774E+03
Magnetospirillum magneticum	NC_007626	6.765E-01	9.011E-03	6.433E+00	7.905E-01	-1.077E+06	5.165E-01	8.514E-03	7.253E-01	8.364E+02	-1.078E+06	-3.01E+03	exponential-log normal	6.648E+00	6.870E-01	-1.718E+04	2.383E+00	4.046E+02	-1.721E+04	1.599E+05	0.174	0.326	0.175	0.325	0.000	4.967E+06	4.559E+03
Mannheimia succiniciproducens	NC_006300	7.997E-01	1.898E-02	6.427E+00	7.001E-01	-4.118E+05	7.419E-01	2.089E-02	1.172E+00	5.439E+02	-4.119E+05	-1.82E+02	exponential-log normal	6.451E+00	8.604E-01	-9.024E+03	1.775E+00	4.871E+02	-8.969E+03	6.940E+04	0.287	0.215	0.287	0.211	0.000	2.314E+06	2.380E+03
Maricaulis maris	NC_008347	7.863E-01	8.144E-03	6.564E+00	6.387E-01	-7.308E+05	6.069E-01	9.433E-03	9.683E-01	5.735E+02	-7.309E+05	-2.59E+02	exponential-log normal	6.704E+00	6.054E-01	-1.533E+04	2.807E+00	3.512E+02	-1.542E+04	1.117E+05	0.186	0.314	0.187	0.314	0.000	3.369E+06	3.063E+03
Marinobacter aquaeolei	NC_008740	7.802E-01	1.053E-02	6.527E+00	6.636E-01	-8.479E+05	6.527E-01	1.208E-02	1.034E+00	5.756E+02	-8.479E+05	-3.27E+01	exponential-log normal	6.706E+00	6.389E-01	-1.285E+04	2.707E+00	3.676E+02	-1.287E+04	1.325E+05	0.211	0.291	0.216	0.282	0.000	4.327E+06	3.858E+03
Mesorhizobium BNC1	NC_008254	7.746E-01	8.957E-03	6.506E+00	6.551E-01	-9.175E+05	5.995E-01	1.028E-02	9.393E-01	5.729E+02	-9.178E+05	-4.61E+02	exponential-log normal	6.700E+00	6.118E-01	-1.669E+04	2.851E+00	3.432E+02	-1.674E+04	1.412E+05	0.195	0.306	0.194	0.304	0.000	4.412E+06	4.064E+03
Methylibium petroleiphilum	NC_008825	7.142E-01	6.719E-03	6.703E+00	6.029E-01	-6.066E+05	5.286E-01	8.703E-03	1.283E+00	5.361E+02	-6.066E+05	-1.10E+01	exponential-log normal	6.703E+00	6.032E-01	-9.529E+03	3.001E+00	3.231E+02	-9.540E+03	8.813E+04	0.154	0.347	0.154	0.345	0.000	4.044E+06	3.819E+03
Methylobacillus flagellatus	NC_007947	8.030E-01	1.086E-02	6.421E+00	7.271E-01	-7.550E+05	6.895E-01	1.154E-02	8.742E-01	6.332E+02	-7.551E+05	-2.66E+02	exponential-log normal	6.679E+00	6.503E-01	-1.123E+04	2.597E+00	3.754E+02	-1.126E+04	1.202E+05	0.222	0.279	0.221	0.278	0.000	2.972E+06	2.753E+03
Methylococcus capsulatus	NC_002977	7.527E-01	7.897E-03	6.627E+00	6.603E-01	-6.277E+05	5.628E-01	9.284E-03	9.464E-01	6.449E+02	-6.277E+05	-9.70E+01	exponential-log normal	6.671E+00	6.761E-01	-1.153E+04	2.467E+00	3.966E+02	-1.154E+04	9.394E+04	0.182	0.319	0.182	0.317	0.000	3.305E+06	2.956E+03
Moorella thermoacetica	NC_007644	7.794E-01	1.195E-02	6.388E+00	6.644E-01	-4.301E+05	6.538E-01	1.374E-02	1.060E+00	4.915E+02	-4.300E+05	7.63E+01	exponential-gamma	6.622E+00	6.587E-01	-1.002E+04	2.576E+00	3.577E+02	-1.003E+04	6.858E+04	0.220	0.279	0.222	0.279	0.000	2.629E+06	2.465E+03
Mycobacterium avium	NC_008595	4.220E-01	1.315E-02	5.942E+00	9.700E-01	-7.313E+05	5.567E-01	4.297E-03	4.346E-01	1.316E+03	-7.333E+05	-3.97E+03	exponential-log normal	6.631E+00	6.750E-01	-2.186E+04	2.499E+00	3.760E+02	-2.186E+04	1.067E+05	0.156	0.344	0.155	0.345	0.000	5.475E+06	5.120E+03
Mycobacterium gilvum	NC_009338	6.350E-01	8.318E-03	6.409E+00	7.167E-01	-7.590E+05	4.021E-01	9.113E-03	8.780E-01	6.034E+02	-7.592E+05	-3.69E+02	exponential-log normal	6.735E+00	5.915E-01	-2.028E+04	3.062E+00	3.270E+02	-2.033E+04	1.112E+05	0.161	0.340	0.160	0.340	0.000	5.620E+06	5.241E+03
Mycobacterium JLS	NC_009077	6.943E-01	7.113E-03	6.542E+00	6.458E-01	-8.014E+05	3.806E-01	8.784E-03	8.622E-01	5.908E+02	-8.017E+05	-4.53E+02	exponential-log normal	6.716E+00	6.021E-01	-2.357E+04	2.970E+00	3.322E+02	-2.362E+04	1.174E+05	0.158	0.342	0.158	0.341	0.000	6.048E+06	5.739E+03
Mycobacterium KMS	NC_008705	6.917E-01	7.053E-03	6.547E+00	6.528E-01	-7.539E+05	3.832E-01	8.602E-03	8.598E-01	6.048E+02	-7.541E+05	-4.90E+02	exponential-log normal	6.716E+00	6.046E-01	-3.036E+04	2.952E+00	3.351E+02	-3.042E+04	1.102E+05	0.158	0.342	0.158	0.342	0.000	5.737E+06	5.460E+03
Mycobacterium leprae	NC_002677	8.722E-01	1.095E-02	6.388E+00	8.414E-01	-5.324E+05	8.103E-01	1.083E-02	6.794E-01	8.767E+02	-5.325E+05	-2.09E+02	exponential-log normal	6.695E+00	6.809E-01	-8.345E+03	2.327E+00	4.386E+02	-8.379E+03	8.811E+04	0.212	0.287	0.210	0.291	0.000	3.268E+06	1.605E+03
Mycobacterium MCS	NC_008146	6.914E-01	7.042E-03	6.549E+00	6.516E-01	-7.495E+05	3.584E-01	8.744E-03	8.236E-01	6.121E+02	-7.497E+05	-4.91E+02	exponential-log normal	6.733E+00	5.947E-01	-2.730E+04	3.054E+00	3.264E+02	-2.735E+04	1.095E+05	0.158	0.342	0.158	0.342	0.000	5.705E+06	5.391E+03
Mycobacterium smegmatis	NC_008596	6.848E-01	7.460E-03	6.445E+00	6.876E-01	-1.010E+06	3.549E-01	8.000E-03	7.239E-01	6.497E+02	-1.011E+06	-1.86E+03	exponential-log normal	6.660E+00	6.547E-01	-1.781E+04	2.667E+00	3.566E+02	-1.779E+04	1.489E+05	0.163	0.337	0.163	0.337	0.000	6.988E+06	6.716E+03
Mycobacterium tuberculosis	NC_002755	6.911E-01	8.945E-03	6.318E+00	8.238E-01	-6.716E+05	5.721E-01	7.839E-03	7.090E-01	8.075E+02	-6.720E+05	-9.13E+02	exponential-log normal	6.591E+00	7.518E-01	-1.850E+04	2.061E+00	4.599E+02	-1.851E+04	1.011E+05	0.172	0.329	0.172	0.327	0.000	4.404E+06	4.189E+03
Mycobacterium ulcerans	NC_008611	7.258E-01	8.482E-03	6.393E+00	7.091E-01	-8.771E+05	5.313E-01	8.869E-03	8.280E-01	6.114E+02	-8.776E+05	-1.06E+03	exponential-log normal	6.725E+00	5.939E-01	-1.077E+04	3.149E+00	3.125E+02	-1.076E+04	1.328E+05	0.172	0.329	0.173	0.326	0.000	5.632E+06	4.160E+03
Mycobacterium vanbaalenii	NC_008726	6.987E-01	7.296E-03	6.524E+00	6.670E-01	-8.874E+05	4.158E-01	8.414E-03	8.385E-01	6.186E+02	-8.877E+05	-7.12E+02	exponential-log normal	6.716E+00	6.000E-01	-2.464E+04	3.015E+00	3.262E+02	-2.469E+04	1.305E+05	0.161	0.339	0.161	0.339	0.000	6.492E+06	5.979E+03
Neisseria gonorrhoeae	NC_002946	7.723E-01	1.055E-02	6.423E+00	6.718E-01	-3.943E+05	6.265E-01	1.206E-02	9.918E-01	5.304E+02	-3.943E+05	-9.07E+00	exponential-log normal	6.523E+00	7.125E-01	-7.275E+03	2.298E+00	3.746E+02	-7.272E+03	6.181E+04	0.238	0.263	0.235	0.264	0.000	2.154E+06	2.002E+03
Neisseria meningitidis	NC_008767	7.703E-01	1.132E-02	6.390E+00	6.954E-01	-4.051E+05	6.322E-01	1.272E-02	9.484E-01	5.500E+02	-4.051E+05	-1.84E+01	exponential-log normal	6.601E+00	6.719E-01	-6.382E+03	2.476E+00	3.681E+02	-6.388E+03	6.395E+04	0.242	0.257	0.242	0.260	0.000	2.195E+06	1.917E+03
Neorickettsia sennetsu	NC_007798	8.161E-01	1.973E-02	6.270E+00	8.279E-01	-1.643E+05	7.328E-01	2.098E-02	7.429E-01	7.112E+02	-1.643E+05	4.88E+01	exponential-gamma	6.337E+00	9.386E-01	-3.455E+03	1.446E+00	5.744E+02	-3.450E+03	2.829E+04	0.296	0.206	0.293	0.205	0.000	8.590E+05	9.320E+02
Nitrobacter hamburgensis	NC_007964	7.795E-01	8.991E-03	6.506E+00	6.824E-01	-8.346E+05	6.200E-01	9.994E-03	9.083E-01	6.109E+02	-8.347E+05	-1.93E+02	exponential-log normal	6.631E+00	6.924E-01	-1.464E+04	2.279E+00	4.217E+02	-1.469E+04	1.287E+05	0.191	0.308	0.192	0.309	0.000	4.407E+06	3.804E+03
Nitrobacter winogradskyi	NC_007406	7.832E-01	8.935E-03	6.508E+00	6.597E-01	-6.575E+05	6.215E-01	1.018E-02	9.686E-01	5.640E+02	-6.575E+05	9.14E+00	exponential-gamma	6.654E+00	6.425E-01	-4.937E+03	2.656E+00	3.563E+02	-4.947E+03	1.015E+05	0.189	0.311	0.190	0.309	0.000	3.402E+06	3.122E+03
Nitrosococcus oceani	NC_007484	8.386E-01	1.401E-02	6.512E+00	7.113E-01	-6.568E+05	7.779E-01	1.503E-02	1.076E+00	6.167E+02	-6.567E+05	9.80E+01	exponential-gamma	6.661E+00	6.610E-01	-1.067E+04	2.462E+00	3.947E+02	-1.071E+04	1.091E+05	0.247	0.252	0.250	0.251	0.000	3.482E+06	2.974E+03
Nitrosomonas europaea	NC_004757	8.284E-01	1.211E-02	6.455E+00	7.243E-01	-6.347E+05	7.362E-01	1.300E-02	9.034E-01	6.416E+02	-6.347E+05	-2.98E+01	exponential-log normal	6.674E+00	6.557E-01	-5.738E+03	2.521E+00	3.887E+02	-5.758E+03	1.034E+05	0.244	0.258	0.248	0.249	0.000	2.812E+06	2.461E+03
Nitrosomonas eutropha	NC_008344	8.300E-01	1.356E-02	6.424E+00	7.360E-01	-5.855E+05	7.447E-01	1.456E-02	8.984E-01	6.398E+02	-5.856E+05	-8.90E+00	exponential-log normal	6.612E+00	6.796E-01	-6.943E+03	2.378E+00	3.916E+02	-6.961E+03	9.683E+04	0.257	0.242	0.258	0.243	0.000	2.661E+06	2.444E+03
Nitrosospira multiformis	NC_007614	7.913E-01	1.179E-02	6.359E+00	7.866E-01	-7.006E+05	6.762E-01	1.206E-02	7.362E-01	7.377E+02	-7.009E+05	-6.62E+02	exponential-log normal	6.658E+00	6.517E-01	-1.244E+04	2.501E+00	3.850E+02	-1.250E+04	1.122E+05	0.230	0.270	0.231	0.270	0.000	3.184E+06	2.757E+03
Nocardioides JS614	NC_008699	7.127E-01	5.334E-03	6.732E+00	5.717E-01	-4.959E+05	3.679E-01	8.756E-03	1.104E+00	5.356E+02	-4.959E+05	6.25E+00	exponential-gamma	6.728E+00	5.877E-01	-2.334E+04	3.250E+00	3.020E+02	-2.332E+04	7.083E+04	0.142	0.358	0.141	0.359	0.000	4.986E+06	4.645E+03
Nostoc sp	NC_003272	8.536E-01	2.226E-02	6.398E+00	8.424E-01	-1.032E+06	8.006E-01	2.340E-02	7.691E-01	8.376E+02	-1.032E+06	-2.83E+02	exponential-log normal	6.611E+00	7.572E-01	-2.121E+04	1.954E+00	5.049E+02	-2.130E+04	1.840E+05	0.294	0.206	0.293	0.207	0.000	6.414E+06	5.366E+03
Novosphingobium aromaticivorans	NC_007794	7.544E-01	7.224E-03	6.642E+00	6.050E-01	-6.744E+05	5.384E-01	9.154E-03	1.070E+00	5.496E+02	-6.744E+05	-6.31E+01	exponential-log normal	6.704E+00	6.237E-01	-1.025E+04	2.828E+00	3.489E+02	-1.026E+04	1.003E+05	0.174	0.326	0.175	0.326	0.000	3.562E+06	3.324E+03
Oceanobacillus iheyensis	NC_004193	8.324E-01	2.307E-02	6.327E+00	7.080E-01	-8.137E+05	7.958E-01	2.469E-02	1.300E+00	4.631E+02	-8.136E+05	2.07E+02	exponential-gamma	6.627E+00	6.391E-01	-1.264E+04	2.828E+00	3.216E+02	-1.262E+04	1.442E+05	0.322	0.179	0.321	0.178	0.000	3.631E+06	3.500E+03
Oenococcus oeni	NC_008528	8.191E-01	1.745E-02	6.424E+00	6.447E-01	-3.098E+05	7.836E-01	1.870E-02	1.612E+00	4.046E+02	-3.098E+05	7.88E+01	exponential-gamma	6.491E+00	7.126E-01	-7.503E+03	2.338E+00	3.542E+02	-7.487E+03	5.244E+04	0.310	0.189	0.311	0.190	0.000	1.781E+06	1.691E+03
Onion yellows	NC_005303	8.539E-01	2.537E-02	6.213E+00	7.894E-01	-1.309E+05	8.097E-01	2.675E-02	9.490E-01	5.639E+02	-1.309E+05	2.49E+01	exponential-gamma	6.505E+00	6.577E-01	-4.854E+03	2.471E+00	3.357E+02	-4.877E+03	2.395E+04	0.357	0.139	0.365	0.138	0.000	8.606E+05	7.540E+02
Orientia tsutsugamushi	NC_009488	8.669E-01	2.826E-02	6.092E+00	8.670E-01	-4.090E+05	8.145E-01	2.942E-02	7.147E-01	6.579E+02	-4.091E+05	-4.25E+01	exponential-log normal	6.492E+00	7.419E-01	-7.474E+03	1.969E+00	4.405E+02	-7.516E+03	7.717E+04	0.347	0.153	0.347	0.152	0.000	2.127E+06	1.182E+03
Parabacteroides distasonis	NC_009615	7.689E-01	1.709E-02	6.471E+00	7.686E-01	-9.985E+05	6.914E-01	1.892E-02	9.720E-01	6.874E+02	-9.982E+05	6.08E+02	exponential-gamma	6.853E+00	6.350E-01	-1.449E+04	2.738E+00	4.207E+02	-1.451E+04	1.627E+05	0.273	0.224	0.276	0.226	0.000	4.811E+06	3.850E+03
Parachlamydia sp	NC_005861	8.645E-01	2.364E-02	6.477E+00	8.063E-01	-4.432E+05	8.286E-01	2.487E-02	9.451E-01	7.629E+02	-4.432E+05	1.41E+01	exponential-gamma	6.576E+00	7.998E-01	-1.160E+04	1.750E+00	5.609E+02	-1.165E+04	8.018E+04	0.327	0.173	0.326	0.174	0.000	2.414E+06	2.031E+03
Paracoccus denitrificans	NC_008686	6.912E-01	7.606E-03	6.637E+00	6.587E-01	-6.291E+05	4.646E-01	9.543E-03	9.703E-01	6.453E+02	-6.292E+05	-2.34E+02	exponential-log normal	6.652E+00	6.164E-01	-8.325E+03	2.864E+00	3.245E+02	-8.345E+03	9.172E+04	0.165	0.334	0.167	0.333	0.000	2.852E+06	2.799E+03
Pasteurella multocida	NC_002663	8.258E-01	2.091E-02	6.396E+00	7.443E-01	-4.644E+05	7.689E-01	2.267E-02	9.904E-01	6.250E+02	-4.645E+05	-2.09E+02	exponential-log normal	6.719E+00	6.087E-01	-1.327E+04	2.849E+00	3.501E+02	-1.333E+04	8.059E+04	0.297	0.199	0.299	0.205	0.000	2.257E+06	2.015E+03
Pediococcus pentosaceus	NC_008525	8.135E-01	2.159E-02	6.392E+00	7.230E-01	-3.822E+05	7.700E-01	2.327E-02	1.208E+00	5.330E+02	-3.822E+05	2.89E+00	exponential-gamma	6.625E+00	6.778E-01	-6.922E+03	2.505E+00	3.715E+02	-6.922E+03	6.604E+04	0.313	0.187	0.313	0.187	0.000	1.832E+06	1.755E+03
Pelobacter carbinolicus	NC_007498	7.884E-01	9.945E-03	6.527E+00	6.472E-01	-7.815E+05	6.638E-01	1.141E-02	1.104E+00	5.350E+02	-7.815E+05	7.52E+01	exponential-gamma	6.628E+00	7.102E-01	-1.139E+04	2.303E+00	4.148E+02	-1.138E+04	1.220E+05	0.225	0.275	0.224	0.276	0.000	3.666E+06	3.352E+03
Pelobacter propionicus	NC_008609	7.666E-01	9.546E-03	6.520E+00	6.813E-01	-8.649E+05	6.083E-01	1.088E-02	9.242E-01	6.174E+02	-8.650E+05	-1.07E+02	exponential-log normal	6.677E+00	6.643E-01	-1.961E+04	2.508E+00	3.920E+02	-1.964E+04	1.332E+05	0.204	0.295	0.206	0.295	0.000	4.008E+06	3.576E+03
Pelodictyon luteolum	NC_007512	7.480E-01	1.023E-02	6.281E+00	8.132E-01	-5.878E+05	6.321E-01	9.517E-03	6.769E-01	7.924E+02	-5.885E+05	-1.48E+03	exponential-log normal	6.684E+00	6.211E-01	-3.775E+03	2.843E+00	3.393E+02	-3.782E+03	9.156E+04	0.211	0.288	0.216	0.286	0.000	2.365E+06	2.083E+03
Pelotomaculum thermopropionicum	NC_009454	7.404E-01	1.342E-02	6.287E+00	7.233E-01	-4.862E+05	5.803E-01	1.536E-02	8.561E-01	5.482E+02	-4.862E+05	1.10E+02	exponential-gamma	6.540E+00	7.056E-01	-1.108E+04	2.335E+00	3.728E+02	-1.107E+04	7.735E+04	0.234	0.264	0.237	0.266	0.000	3.025E+06	2.920E+03
Picrophilus torridus	NC_005877	8.086E-01	2.217E-02	6.161E+00	8.130E-01	-4.360E+05	7.317E-01	2.376E-02	8.205E-01	5.902E+02	-4.359E+05	1.89E+02	exponential-gamma	6.649E+00	6.322E-01	-7.358E+03	2.818E+00	3.310E+02	-7.359E+03	7.616E+04	0.320	0.181	0.320	0.178	0.000	1.546E+06	1.535E+03
Pirellula sp	NC_005027	7.688E-01	1.041E-02	6.514E+00	8.447E-01	-1.373E+06	6.503E-01	1.023E-02	6.264E-01	1.080E+03	-1.375E+06	-3.21E+03	exponential-log normal	6.424E+00	9.361E-01	-4.029E+04	1.335E+00	7.024E+02	-4.052E+04	2.126E+05	0.222	0.279	0.224	0.275	0.000	7.146E+06	7.325E+03
Polaromonas JS666	NC_007948	7.636E-01	8.469E-03	6.487E+00	6.403E-01	-1.071E+06	5.550E-01	1.001E-02	9.449E-01	5.355E+02	-1.071E+06	-4.06E+02	exponential-log normal	6.682E+00	5.942E-01	-1.935E+04	3.060E+00	3.084E+02	-1.940E+04	1.635E+05	0.187	0.312	0.188	0.313	0.000	5.200E+06	4.817E+03
Polaromonas naphthalenivorans	NC_008781	7.471E-01	8.674E-03	6.504E+00	6.557E-01	-9.345E+05	5.472E-01	1.029E-02	9.533E-01	5.612E+02	-9.346E+05	-2.08E+02	exponential-log normal	6.704E+00	6.067E-01	-2.230E+04	2.951E+00	3.302E+02	-2.234E+04	1.418E+05	0.187	0.313	0.188	0.312	0.000	4.410E+06	4.084E+03
Polynucleobacter QLW-P1DMWA-1	NC_009379	8.192E-01	1.847E-02	6.387E+00	7.611E-01	-4.736E+05	7.223E-01	2.001E-02	7.017E-01	8.017E+02	-4.743E+05	-1.42E+03	exponential-log normal	6.653E+00	5.955E-01	-7.492E+03	3.093E+00	2.966E+02	-7.499E+03	8.064E+04	0.275	0.225	0.277	0.223	0.000	2.159E+06	2.077E+03
Porphyromonas gingivalis	NC_002950	7.770E-01	1.408E-02	6.422E+00	7.666E-01	-5.161E+05	6.631E-01	1.539E-02	8.153E-01	7.099E+02	-5.161E+05	7.39E+00	exponential-gamma	6.662E+00	7.776E-01	-3.969E+03	1.974E+00	5.226E+02	-3.968E+03	8.312E+04	0.259	0.241	0.258	0.242	0.000	2.343E+06	1.909E+03
Prochlorococcus marinus	NC_008816	8.594E-01	2.755E-02	6.224E+00	8.120E-01	-2.832E+05	8.123E-01	2.909E-02	8.395E-01	6.440E+02	-2.832E+05	-1.17E+02	exponential-log normal	6.348E+00	8.115E-01	-1.034E+04	1.778E+00	4.374E+02	-1.036E+04	5.260E+04	0.344	0.156	0.343	0.157	0.000	1.670E+06	1.921E+03
Propionibacterium acnes	NC_006085	8.091E-01	8.948E-03	6.572E+00	6.314E-01	-5.088E+05	6.746E-01	1.028E-02	1.073E+00	5.465E+02	-5.088E+05	1.28E+01	exponential-gamma	6.773E+00	6.378E-01	-4.336E+03	2.797E+00	3.769E+02	-4.332E+03	7.920E+04	0.200	0.300	0.199	0.300	0.000	2.560E+06	2.297E+03
Prosthecochloris vibrioformis	NC_009337	7.663E-01	1.165E-02	6.266E+00	8.439E-01	-4.873E+05	6.594E-01	1.102E-02	6.327E-01	8.596E+02	-4.880E+05	-1.36E+03	exponential-log normal	6.713E+00	6.620E-01	-7.797E+03	2.219E+00	4.709E+02	-7.882E+03	7.740E+04	0.232	0.266	0.238	0.264	0.000	1.967E+06	1.753E+03
Pseudoalteromonas atlantica	NC_008228	8.308E-01	1.821E-02	6.477E+00	7.184E-01	-1.071E+06	7.865E-01	1.955E-02	1.197E+00	5.725E+02	-1.071E+06	3.00E+02	exponential-gamma	6.767E+00	6.246E-01	-1.580E+04	2.795E+00	3.762E+02	-1.583E+04	1.829E+05	0.277	0.223	0.277	0.223	0.000	5.187E+06	4.281E+03
Pseudomonas aeruginosa	NC_002516	6.834E-01	7.965E-03	6.589E+00	6.717E-01	-9.674E+05	4.361E-01	9.720E-03	8.740E-01	6.715E+02	-9.680E+05	-1.20E+03	exponential-log normal	6.707E+00	6.174E-01	-2.333E+04	2.800E+00	3.525E+02	-2.341E+04	1.416E+05	0.166	0.336	0.169	0.330	0.000	6.264E+06	5.568E+03
Pseudomonas entomophila	NC_008027	6.407E-01	1.023E-02	6.282E+00	8.658E-01	-1.043E+06	5.115E-01	8.593E-03	6.437E-01	9.066E+02	-1.045E+06	-3.08E+03	exponential-log normal	6.672E+00	6.859E-01	-1.625E+04	2.280E+00	4.385E+02	-1.633E+04	1.559E+05	0.179	0.320	0.179	0.321	0.000	5.889E+06	5.134E+03
Pseudomonas fluorescens	NC_004129	6.585E-01	1.073E-02	6.307E+00	8.408E-01	-1.226E+06	5.126E-01	9.751E-03	6.721E-01	8.512E+02	-1.228E+06	-3.82E+03	exponential-log normal	6.699E+00	6.557E-01	-2.126E+04	2.419E+00	4.176E+02	-2.140E+04	1.846E+05	0.182	0.318	0.185	0.315	0.000	7.075E+06	6.138E+03
Pseudomonas mendocina	NC_009439	7.447E-01	8.562E-03	6.603E+00	6.380E-01	-8.957E+05	5.588E-01	1.038E-02	9.978E-01	6.013E+02	-8.960E+05	-5.85E+02	exponential-log normal	6.693E+00	6.262E-01	-1.768E+04	2.849E+00	3.412E+02	-1.770E+04	1.348E+05	0.177	0.324	0.176	0.323	0.000	5.073E+06	4.594E+03
Pseudomonas putida	NC_009512	7.411E-01	9.483E-03	6.468E+00	7.183E-01	-1.126E+06	5.595E-01	1.023E-02	7.909E-01	6.908E+02	-1.126E+06	-1.50E+03	exponential-log normal	6.697E+00	6.564E-01	-2.909E+04	2.466E+00	4.094E+02	-2.921E+04	1.718E+05	0.190	0.310	0.191	0.309	0.000	5.960E+06	5.252E+03
Pseudomonas stutzeri	NC_009434	7.602E-01	8.323E-03	6.624E+00	6.112E-01	-8.474E+05	6.017E-01	1.016E-02	1.171E+00	5.347E+02	-8.474E+05	-1.81E+01	exponential-log normal	6.726E+00	6.373E-01	-9.985E+03	2.753E+00	3.673E+02	-9.986E+03	1.280E+05	0.181	0.319	0.181	0.320	0.000	4.567E+06	4.128E+03
Pseudomonas syringae	NC_005773	7.693E-01	1.106E-02	6.458E+00	7.122E-01	-1.154E+06	6.238E-01	1.218E-02	8.374E-01	6.648E+02	-1.155E+06	-1.25E+03	exponential-log normal	6.714E+00	6.566E-01	-2.313E+04	2.664E+00	3.771E+02	-2.310E+04	1.808E+05	0.211	0.289	0.209	0.291	0.000	5.929E+06	4.984E+03
Psychrobacter arcticum	NC_007204	8.369E-01	1.863E-02	6.470E+00	7.450E-01	-5.896E+05	7.736E-01	2.009E-02	8.934E-01	7.250E+02	-5.900E+05	-7.46E+02	exponential-log normal	6.748E+00	6.247E-01	-1.029E+04	2.808E+00	3.657E+02	-1.030E+04	1.013E+05	0.286	0.214	0.286	0.214	0.000	2.651E+06	2.120E+03
Psychrobacter cryohalolentis	NC_007969	8.388E-01	1.882E-02	6.492E+00	6.975E-01	-6.891E+05	8.019E-01	2.008E-02	1.292E+00	5.442E+02	-6.891E+05	-5.60E+01	exponential-log normal	6.764E+00	6.059E-01	-1.304E+04	3.035E+00	3.407E+02	-1.304E+04	1.187E+05	0.289	0.211	0.288	0.212	0.000	3.060E+06	2.467E+03
Psychrobacter PRwf-1	NC_009524	8.326E-01	1.877E-02	6.470E+00	7.390E-01	-6.302E+05	7.709E-01	2.029E-02	9.321E-01	6.994E+02	-6.305E+05	-6.84E+02	exponential-log normal	6.748E+00	6.112E-01	-1.046E+04	2.980E+00	3.395E+02	-1.046E+04	1.081E+05	0.277	0.225	0.274	0.224	0.000	2.979E+06	2.370E+03
Psychromonas ingrahamii	NC_008709	8.418E-01	2.048E-02	6.429E+00	7.672E-01	-8.843E+05	7.857E-01	2.190E-02	8.924E-01	7.178E+02	-8.847E+05	-7.64E+02	exponential-log normal	6.730E+00	6.606E-01	-1.477E+04	2.456E+00	4.237E+02	-1.483E+04	1.545E+05	0.299	0.200	0.300	0.201	0.000	4.560E+06	3.545E+03
Ralstonia solanacearum	NC_003295	6.844E-01	7.681E-03	6.517E+00	7.275E-01	-7.035E+05	4.745E-01	7.941E-03	8.078E-01	7.246E+02	-7.039E+05	-7.57E+02	exponential-log normal	6.653E+00	6.602E-01	-1.748E+04	2.481E+00	3.854E+02	-1.753E+04	1.032E+05	0.166	0.333	0.164	0.337	0.000	3.716E+06	3.440E+03
Rhizobium etli	NC_007761	7.721E-01	8.571E-03	6.521E+00	6.257E-01	-9.299E+05	5.890E-01	1.018E-02	1.023E+00	5.258E+02	-9.301E+05	-3.41E+02	exponential-log normal	6.679E+00	5.941E-01	-2.132E+04	3.024E+00	3.126E+02	-2.138E+04	1.423E+05	0.193	0.310	0.194	0.303	0.000	4.382E+06	4.035E+03
Rhizobium leguminosarum	NC_008378	7.679E-01	8.618E-03	6.534E+00	5.728E-01	-1.915E+05	5.947E-01	1.086E-02	1.245E+00	4.412E+02	-1.916E+05	-9.37E+01	exponential-log normal	6.806E+00	4.631E-01	-2.274E+03	5.315E+00	1.874E+02	-2.264E+03	2.930E+04	0.196	0.303	0.194	0.307	0.000	8.700E+05	7.800E+02
Rhodoferax ferrireducens	NC_007908	7.689E-01	9.391E-03	6.510E+00	6.589E-01	-9.740E+05	6.030E-01	1.091E-02	9.686E-01	5.698E+02	-9.741E+05	-1.81E+02	exponential-log normal	6.721E+00	6.018E-01	-1.885E+04	2.989E+00	3.314E+02	-1.889E+04	1.501E+05	0.201	0.299	0.200	0.300	0.000	4.712E+06	4.170E+03

Rhodopseudomonas palustris	NC_008435	6.714E-01	9.284E-03	6.396E+00	7.727E-01	-9.491E+05	4.826E-01	9.045E-03	7.320E-01	7.555E+02	-9.500E+05	-1.94E+03	exponential-log normal	6.635E+00	7.000E-01	-2.488E+04	2.164E+00	4.511E+02	-2.504E+04	1.415E+05	0.178	0.322	0.178	0.322	0.000	5.505E+06	4.878E+03
Rhodospirillum rubrum	NC_007643	7.384E-01	7.866E-03	6.640E+00	6.108E-01	-7.859E+05	5.547E-01	9.925E-03	1.146E+00	5.465E+02	-7.859E+05	-3.03E+01	exponential-log normal	6.750E+00	6.326E-01	-1.091E+04	2.773E+00	3.733E+02	-1.092E+04	1.170E+05	0.173	0.327	0.173	0.327	0.000	4.353E+06	3.791E+03
Rickettsia bellii	NC_007940	8.415E-01	2.757E-02	6.321E+00	7.974E-01	-2.646E+05	8.054E-01	2.915E-02	1.035E+00	6.054E+02	-2.645E+05	1.26E+02	exponential-gamma	6.564E+00	7.077E-01	-6.020E+03	2.159E+00	4.216E+02	-6.048E+03	4.822E+04	0.345	0.161	0.339	0.156	0.000	1.522E+06	1.429E+03
Rickettsia conorii	NC_003103	8.352E-01	2.733E-02	6.221E+00	8.693E-01	-2.137E+05	7.811E-01	2.885E-02	7.796E-01	7.144E+02	-2.136E+05	8.70E+01	exponential-gamma	6.260E+00	7.851E-01	-3.987E+03	1.678E+00	4.323E+02	-4.029E+03	3.885E+04	0.339	0.161	0.337	0.163	0.000	1.269E+06	1.374E+03
Rickettsia felis	NC_007109	8.324E-01	2.715E-02	6.281E+00	8.320E-01	-2.455E+05	7.821E-01	2.891E-02	8.587E-01	6.862E+02	-2.455E+05	2.36E+01	exponential-gamma	6.521E+00	7.033E-01	-5.124E+03	2.131E+00	4.108E+02	-5.157E+03	4.439E+04	0.335	0.161	0.340	0.164	0.000	1.485E+06	1.400E+03
Rickettsia prowazekii	NC_000963	8.579E-01	2.941E-02	6.393E+00	7.811E-01	-1.980E+05	8.325E-01	3.089E-02	1.153E+00	5.974E+02	-1.979E+05	9.21E+01	exponential-gamma	6.729E+00	6.612E-01	-2.892E+03	2.511E+00	4.116E+02	-2.900E+03	3.681E+04	0.356	0.144	0.354	0.146	0.000	1.112E+06	8.350E+02
Rickettsia typhi	NC_006142	8.604E-01	2.978E-02	6.394E+00	8.084E-01	-2.001E+05	8.318E-01	3.132E-02	1.038E+00	6.630E+02	-2.000E+05	7.74E+01	exponential-gamma	6.701E+00	7.047E-01	-4.500E+03	2.309E+00	4.436E+02	-4.500E+03	3.734E+04	0.355	0.147	0.355	0.143	0.000	1.111E+06	8.380E+02
Roseiflexus RS-1	NC_009523	7.803E-01	9.714E-03	6.485E+00	7.228E-01	-1.162E+06	6.408E-01	1.040E-02	8.385E-01	6.835E+02	-1.163E+06	-7.15E+02	exponential-log normal	6.794E+00	6.504E-01	-2.612E+04	2.539E+00	4.327E+02	-2.621E+04	1.806E+05	0.198	0.302	0.198	0.302	0.000	5.802E+06	4.517E+03
Roseobacter denitrificans	NC_008209	7.670E-01	9.884E-03	6.467E+00	6.428E-01	-9.650E+05	5.911E-01	1.171E-02	9.704E-01	5.327E+02	-9.652E+05	-3.61E+02	exponential-log normal	6.621E+00	7.059E-01	-1.568E+04	2.457E+00	3.806E+02	-1.561E+04	1.497E+05	0.205	0.295	0.205	0.295	0.000	4.133E+06	3.946E+03
Rubrobacter xylanophilus	NC_008148	7.181E-01	7.094E-03	6.624E+00	5.781E-01	-3.552E+05	4.865E-01	9.992E-03	1.234E+00	4.776E+02	-3.551E+05	3.34E+01	exponential-gamma	6.690E+00	5.692E-01	-1.557E+04	3.385E+00	2.782E+02	-1.557E+04	5.222E+04	0.147	0.353	0.148	0.351	0.000	3.226E+06	3.140E+03
Saccharophagus degradans	NC_007912	7.945E-01	1.938E-02	6.373E+00	8.881E-01	-8.610E+05	6.663E-01	2.023E-02	4.874E-01	1.173E+03	-8.624E+05	-2.77E+03	exponential-log normal	6.759E+00	6.611E-01	-2.735E+04	2.357E+00	4.596E+02	-2.754E+04	1.449E+05	0.271	0.229	0.271	0.229	0.000	5.058E+06	4.008E+03
Salinibacter ruber	NC_007677	7.592E-01	7.256E-03	6.686E+00	6.330E-01	-4.766E+05	5.658E-01	8.806E-03	1.015E+00	6.249E+02	-4.766E+05	1.37E+01	exponential-gamma	6.742E+00	6.535E-01	-6.575E+03	2.708E+00	3.816E+02	-6.568E+03	7.080E+04	0.168	0.331	0.169	0.331	0.000	3.552E+06	2.801E+03
Salinispora tropica	NC_009380	4.248E-01	1.239E-02	5.916E+00	9.623E-01	-5.638E+05	6.815E-01	3.977E-03	3.865E-01	1.656E+03	-5.648E+05	-2.11E+03	exponential-log normal	6.708E+00	6.134E-01	-2.008E+04	2.909E+00	3.372E+02	-2.012E+04	8.249E+04	0.153	0.347	0.153	0.348	0.000	5.183E+06	4.536E+03
Salmonella enterica	NC_006905	7.805E-01	1.342E-02	6.411E+00	6.913E-01	-9.513E+05	6.642E-01	1.523E-02	9.648E-01	5.677E+02	-9.515E+05	-3.28E+02	exponential-log normal	6.564E+00	7.041E-01	-1.966E+04	2.364E+00	3.762E+02	-1.964E+04	1.531E+05	0.239	0.261	0.239	0.261	0.000	4.756E+06	4.427E+03
Salmonella typhimurium	NC_003197	7.809E-01	1.335E-02	6.437E+00	6.911E-01	-9.740E+05	6.695E-01	1.516E-02	9.827E-01	5.777E+02	-9.742E+05	-3.00E+02	exponential-log normal	6.615E+00	6.669E-01	-1.729E+04	2.489E+00	3.714E+02	-1.732E+04	1.565E+05	0.239	0.261	0.239	0.261	0.000	4.857E+06	4.425E+03
Shewanella amazonensis	NC_008700	7.881E-01	1.229E-02	6.495E+00	7.270E-01	-9.068E+05	6.799E-01	1.350E-02	9.041E-01	6.754E+02	-9.068E+05	-1.17E+02	exponential-log normal	6.716E+00	6.422E-01	-1.086E+04	2.602E+00	3.889E+02	-1.090E+04	1.445E+05	0.232	0.267	0.232	0.269	0.000	4.306E+06	3.645E+03
Shewanella baltica	NC_009052	8.269E-01	1.778E-02	6.470E+00	7.237E-01	-1.039E+06	7.761E-01	1.915E-02	1.121E+00	5.979E+02	-1.039E+06	6.68E+01	exponential-gamma	6.727E+00	6.507E-01	-1.604E+04	2.574E+00	3.999E+02	-1.608E+04	1.764E+05	0.268	0.232	0.269	0.231	0.000	5.127E+06	4.307E+03
Shewanella denitrificans	NC_007954	8.372E-01	1.890E-02	6.448E+00	8.104E-01	-9.879E+05	7.693E-01	2.001E-02	7.513E-01	8.648E+02	-9.886E+05	-1.30E+03	exponential-log normal	6.708E+00	6.575E-01	-1.419E+04	2.441E+00	4.170E+02	-1.427E+04	1.702E+05	0.274	0.226	0.275	0.225	0.000	4.546E+06	3.754E+03
Shewanella frigidimarina	NC_008345	8.393E-01	2.061E-02	6.461E+00	7.352E-01	-1.076E+06	8.012E-01	2.195E-02	1.174E+00	5.891E+02	-1.076E+06	1.82E+02	exponential-gamma	6.712E+00	6.561E-01	-9.478E+03	2.582E+00	3.905E+02	-9.493E+03	1.878E+05	0.292	0.208	0.292	0.208	0.000	4.845E+06	4.029E+03
Shewanella loihica	NC_009092	7.967E-01	1.441E-02	6.463E+00	7.364E-01	-9.167E+05	7.075E-01	1.579E-02	9.352E-01	6.602E+02	-9.167E+05	-5.83E+01	exponential-log normal	6.705E+00	6.609E-01	-1.516E+04	2.458E+00	4.128E+02	-1.522E+04	1.493E+05	0.232	0.269	0.232	0.268	0.000	4.603E+06	3.859E+03
Shewanella MR-4	NC_008321	8.158E-01	1.696E-02	6.505E+00	7.289E-01	-9.332E+05	7.605E-01	1.835E-02	1.101E+00	6.297E+02	-9.332E+05	1.18E+02	exponential-gamma	6.711E+00	6.656E-01	-1.799E+04	2.420E+00	4.226E+02	-1.806E+04	1.562E+05	0.261	0.239	0.261	0.240	0.000	4.706E+06	3.924E+03
Shewanella MR-7	NC_008322	8.150E-01	1.703E-02	6.489E+00	7.447E-01	-9.519E+05	7.537E-01	1.844E-02	1.020E+00	6.635E+02	-9.518E+05	5.37E+01	exponential-gamma	6.696E+00	6.521E-01	-1.483E+04	2.571E+00	3.864E+02	-1.486E+04	1.593E+05	0.260	0.240	0.262	0.239	0.000	4.793E+06	4.006E+03
Shewanella oneidensis	NC_004347	8.244E-01	1.804E-02	6.454E+00	7.440E-01	-1.013E+06	7.652E-01	1.945E-02	9.970E-01	6.526E+02	-1.013E+06	-1.76E+02	exponential-log normal	6.624E+00	7.010E-01	-1.699E+04	2.251E+00	4.246E+02	-1.703E+04	1.721E+05	0.270	0.230	0.270	0.230	0.000	4.970E+06	4.318E+03
Shewanella putrefaciens	NC_009438	8.250E-01	1.894E-02	6.478E+00	7.456E-01	-9.685E+05	7.752E-01	2.035E-02	1.065E+00	6.457E+02	-9.685E+05	-4.60E+00	exponential-log normal	6.669E+00	6.698E-01	-2.052E+04	2.451E+00	4.004E+02	-2.058E+04	1.654E+05	0.278	0.223	0.277	0.222	0.000	4.659E+06	3.972E+03
Shewanella W3-18-1	NC_008750	8.252E-01	1.875E-02	6.474E+00	7.428E-01	-9.799E+05	7.758E-01	2.015E-02	1.076E+00	6.369E+02	-9.799E+05	8.99E+01	exponential-gamma	6.695E+00	6.647E-01	-1.931E+04	2.465E+00	4.073E+02	-1.938E+04	1.672E+05	0.277	0.223	0.277	0.224	0.000	4.708E+06	4.044E+03
Shigella boydii	NC_007613	7.926E-01	1.370E-02	6.381E+00	6.912E-01	-9.188E+05	6.931E-01	1.532E-02	1.037E+00	5.261E+02	-9.188E+05	1.28E+02	exponential-gamma	6.564E+00	6.652E-01	-1.415E+04	2.381E+00	3.675E+02	-1.417E+04	1.494E+05	0.244	0.255	0.244	0.257	0.000	4.520E+06	4.136E+03
Shigella dysenteriae	NC_007606	8.171E-01	1.275E-02	6.401E+00	6.741E-01	-8.903E+05	7.224E-01	1.413E-02	1.062E+00	5.106E+02	-8.903E+05	1.09E+02	exponential-gamma	6.412E+00	6.881E-01	-1.463E+04	2.262E+00	3.421E+02	-1.470E+04	1.455E+05	0.244	0.258	0.243	0.255	0.000	4.369E+06	4.274E+03
Shigella flexneri	NC_004337	7.991E-01	1.354E-02	6.410E+00	6.640E-01	-9.384E+05	7.130E-01	1.517E-02	1.179E+00	4.833E+02	-9.383E+05	2.02E+02	exponential-gamma	6.560E+00	6.521E-01	-2.037E+04	2.613E+00	3.316E+02	-2.040E+04	1.528E+05	0.245	0.255	0.246	0.254	0.000	4.607E+06	4.182E+03
Shigella sonnei	NC_007384	7.948E-01	1.349E-02	6.420E+00	6.724E-01	-9.855E+05	7.065E-01	1.514E-02	1.149E+00	5.020E+02	-9.854E+05	1.93E+02	exponential-gamma	6.621E+00	6.457E-01	-1.767E+04	2.693E+00	3.393E+02	-1.767E+04	1.599E+05	0.245	0.255	0.245	0.255	0.000	4.825E+06	4.223E+03
Silicibacter pomeroyi	NC_003911	6.907E-01	9.096E-03	6.516E+00	6.478E-01	-8.578E+05	4.522E-01	1.156E-02	9.126E-01	5.938E+02	-8.585E+05	-1.43E+03	exponential-log normal	6.707E+00	6.038E-01	-1.316E+04	2.949E+00	3.318E+02	-1.320E+04	1.278E+05	0.179	0.320	0.179	0.322	0.000	4.109E+06	
Silicibacter TM1040	NC_008044	7.511E-01	9.500E-03	6.509E+00	6.352E-01	-6.799E+05	5.687E-01	1.158E-02	1.016E+00	5.338E+02	-6.799E+05	-6.53E+01	exponential-log normal	6.666E+00	5.941E-01	-1.180E+04	3.052E+00	3.061E+02	-1.184E+04	1.041E+05	0.198	0.302	0.198	0.302	0.000	3.201E+06	3.030E+03
Sinorhizobium medicae	NC_009636	7.542E-01	8.727E-03	6.508E+00	6.426E-01	-7.649E+05	5.574E-01	1.036E-02	9.640E-01	5.523E+02	-7.651E+05	-4.14E+02	exponential-log normal	6.626E+00	6.453E-01	-1.426E+04	2.679E+00	3.436E+02	-1.427E+04	1.165E+05	0.193	0.308	0.192	0.307	0.000	3.782E+06	3.529E+03
Sinorhizobium meliloti	NC_003047	7.472E-01	8.360E-03	6.561E+00	6.312E-01	-7.322E+05	5.435E-01	1.021E-02	9.929E-01	5.614E+02	-7.324E+05	-3.06E+02	exponential-log normal	6.651E+00	6.370E-01	-1.840E+04	2.719E+00	3.462E+02	-1.842E+04	1.104E+05	0.186	0.315	0.186	0.312	0.000	3.654E+06	3.341E+03
Sodalis glossinidius	NC_007712	8.097E-01	1.248E-02	6.164E+00	7.133E-01	-8.123E+05	6.946E-01	1.315E-02	9.067E-01	4.638E+02	-8.124E+05	-1.11E+02	exponential-log normal	6.609E+00	5.898E-01	-8.550E+03	3.009E+00	2.941E+02	-8.595E+03	1.337E+05	0.226	0.271	0.227	0.276	0.000	4.171E+06	2.432E+03
Solibacter usitatus	NC_008536	7.409E-01	9.224E-03	6.606E+00	7.058E-01	-1.651E+06	5.651E-01	1.056E-02	8.500E-01	7.356E+02	-1.651E+06	-2.60E+02	exponential-log normal	6.818E+00	6.726E-01	-4.269E+04	2.396E+00	4.770E+02	-4.286E+04	2.491E+05	0.191	0.310	0.190	0.309	0.000	9.966E+06	7.826E+03
Sphingomonas wittichii	NC_009511	7.113E-01	6.794E-03	6.727E+00	5.721E-01	-1.005E+06	5.273E-01	9.185E-03	1.389E+00	5.029E+02	-1.005E+06	7.96E+01	exponential-gamma	6.735E+00	6.058E-01	-2.113E+04	3.041E+00	3.299E+02	-2.113E+04	1.458E+05	0.158	0.342	0.158	0.342	0.000	5.382E+06	4.850E+03
Sphingopyxis alaskensis	NC_008048	7.424E-01	7.285E-03	6.634E+00	5.977E-01	-6.593E+05	5.004E-01	9.687E-03	1.052E+00	5.396E+02	-6.593E+05	-3.90E+00	exponential-log normal	6.673E+00	6.288E-01	-2.266E+04	2.757E+00	3.470E+02	-2.272E+04	9.782E+04	0.172	0.328	0.173	0.327	0.000	3.345E+06	3.165E+03
Staphylococcus aureus	NC_002952	8.476E-01	2.574E-02	6.292E+00	8.354E-01	-6.926E+05	7.654E-01	2.738E-02	5.715E-01	9.408E+02	-6.937E+05	-2.25E+03	exponential-log normal	6.568E+00	7.045E-01	-1.157E+04	2.360E+00	3.793E+02	-1.155E+04	1.258E+05	0.338	0.164	0.334	0.164	0.000	2.903E+06	2.656E+03
Staphylococcus epidermidis	NC_004461	8.483E-01	2.650E-02	6.318E+00	8.131E-01	-5.689E+05	7.776E-01	2.836E-02	6.511E-01	8.634E+02	-5.697E+05	-1.54E+03	exponential-log normal	6.441E+00	8.470E-01	-5.329E+03	1.612E+00	5.458E+02	-5.350E+03	1.037E+05	0.344	0.162	0.335	0.159	0.000	2.499E+06	2.419E+03
Staphylococcus haemolyticus	NC_007168	8.433E-01	2.587E-02	6.347E+00	7.416E-01	-6.187E+05	8.085E-01	2.756E-02	1.157E+00	5.416E+02	-6.187E+05	-9.70E+01	exponential-log normal	6.500E+00	7.727E-01	-1.735E+04	2.071E+00	4.163E+02	-1.728E+04	1.120E+05	0.334	0.163	0.338	0.165	0.000	2.685E+06	2.676E+03
Staphylococcus saprophyticus	NC_007350	8.501E-01	2.479E-02	6.324E+00	7.041E-01	-5.973E+05	8.233E-01	2.616E-02	1.409E+00	4.359E+02	-5.972E+05	2.64E+02	exponential-gamma	6.549E+00	6.737E-01	-1.189E+04	2.564E+00	3.351E+02	-1.186E+04	1.083E+05	0.333	0.165	0.335	0.167	0.000	2.517E+06	2.446E+03
Streptococcus agalactiae	NC_004116	8.228E-01	2.241E-02	6.394E+00	7.152E-01	-4.273E+05	7.853E-01	2.402E-02	1.276E+00	5.104E+02	-4.273E+05	2.36E+01	exponential-gamma	6.508E+00	7.488E-01	-1.318E+04	2.124E+00	4.081E+02	-1.317E+04	7.465E+04	0.321	0.179	0.323	0.178	0.000	2.160E+06	2.124E+03
Streptococcus mutans	NC_004350	8.346E-01	1.871E-02	6.444E+00	7.221E-01	-4.062E+05	7.829E-01	2.011E-02	1.074E+00	6.025E+02	-4.062E+05	-1.46E+02	exponential-log normal	6.561E+00	7.329E-01	-1.221E+04	2.185E+00	4.126E+02	-1.220E+04	6.983E+04	0.317	0.185	0.315	0.183	0.000	2.031E+06	1.960E+03
Streptococcus pneumoniae	NC_008533	8.181E-01	1.867E-02	6.364E+00	7.454E-01	-3.930E+05	7.493E-01	2.031E-02	9.605E-01	6.047E+02	-3.930E+05	-1.58E+01	exponential-log normal	6.569E+00	7.301E-01	-6.078E+03	2.237E+00	4.052E+02	-6.066E+03	6.706E+04	0.301	0.198	0.302	0.199	0.000	2.046E+06	1.914E+03
Streptococcus pyogenes	NC_002737	8.251E-01	2.010E-02	6.406E+00	7.181E-01	-3.702E+05	7.764E-01	2.165E-02	1.173E+00	5.422E+02	-3.702E+05	9.61E+01	exponential-gamma	6.628E+00	6.436E-01	-5.836E+03	2.728E+00	3.374E+02	-5.835E+03	6.392E+04	0.306	0.191	0.309	0.194	0.000	1.852E+06	1.697E+03
Streptococcus sanguinis	NC_009009	8.087E-01	1.678E-02	6.382E+00	7.384E-01	-4.617E+05	7.237E-01	1.837E-02	8.976E-01	6.332E+02	-4.618E+05	-2.29E+02	exponential-log normal	6.636E+00	6.689E-01	-1.493E+04	2.452E+00	3.869E+02	-1.498E+04	7.732E+04	0.283	0.217	0.283	0.217	0.000	2.388E+06	2.270E+03
Streptococcus suis	NC_009442	8.219E-01	1.798E-02	6.355E+00	7.411E-01	-4.145E+05	7.505E-01	1.952E-02	9.496E-01	5.955E+02	-4.145E+05	-2.57E+00	exponential-log normal	6.440E+00	7.630E-01	-8.771E+03	2.020E+00	4.066E+02	-8.769E+03	7.064E+04	0.294	0.210	0.295	0.202	0.000	2.096E+06	2.186E+03
Streptococcus thermophilus	NC_006449	8.402E-01	1.801E-02	6.391E+00	6.980E-01	-3.545E+05	7.938E-01	1.930E-02	1.225E+00	4.997E+02	-3.544E+05	9.84E+01	exponential-gamma	6.432E+00	7.252E-01	-1.162E+04	2.190E+00	3.623E+02	-1.163E+04	6.109E+04	0.303	0.195	0.306	0.196	0.000	1.796E+06	1.915E+03
Streptomyces coelicolor	NC_003888	5.620E-01	7.528E-03	6.407E+00	7.681E-01	-8.747E+05	3.865E-01	5.031E-03	7.099E-01	8.148E+02	-8.756E+05	-1.75E+03	exponential-log normal	6.694E+00	6.494E-01	-3.258E+04	2.679E+00	3.664E+02	-3.260E+04	1.246E+05	0.140	0.360	0.139	0.361	0.000	8.668E+06	7.769E+03
Sulfolobus acidocaldarius	NC_007181	8.198E-01	2.812E-02	6.098E+00	8.091E-01	-4.045E+05	7.644E-01	3.016E-02	9.298E-01	5.136E+02	-4.043E+05	3.74E+02	exponential-gamma	6.590E+00	6.238E-01	-1.216E+04	2.868E+00	3.052E+02	-1.217E+04	7.346E+04	0.317	0.183	0.315	0.184	0.000	2.226E+06	2.223E+03
Sulfolobus solfataricus	NC_002754	8.180E-01	2.876E-02	6.095E+00	7.957E-01	-5.155E+05	7.669E-01	3.088E-02	9.896E-01	4.854E+02	-5.152E+05	4.73E+02	exponential-gamma	6.585E+00	6.059E-01	-7.333E+03	3.018E+00	2.863E+02	-7.336E+03	9.378E+04	0.323	0.179	0.319	0.179	0.000	2.992E+06	2.977E+03
Sulfolobus tokodaii	NC_003106	8.364E-01	3.110E-02	6.101E+00	8.057E-01	-4.745E+05	7.944E-01	3.301E-02	9.897E-01	4.988E+02	-4.743E+05	3.72E+02	exponential-gamma	6.462E+00	6.604E-01	-7.433E+03	2.491E+00	3.195E+02	-7.457E+03	8.836E+04	0.338	0.163	0.334	0.165	0.000	2.695E+06	2.825E+03
Symbiobacterium thermophilum	NC_006177	7.401E-01	6.884E-03	6.644E+00	6.071E-01	-5.240E+05	4.654E-01	9.078E-03	9.712E-01	5.697E+02	-5.240E+05	-1.86E+01	exponential-log normal	6.637E+00	6.408E-01	-1.655E+04	2.721E+00	3.396E+02	-1.656E+04	7.726E+04	0.156	0.344	0.157	0.343	0.000	3.566E+06	3.338E+03
Synechococcus CC9311	NC_008319	8.402E-01	1.360E-02	6.362E+00	6.964E-01	-4.809E+05	7.556E-01	1.471E-02	9.780E-01	5.357E+02	-4.809E+05	-1.58E+01	exponential-log normal	6.445E+00	7.824E-01	-7.114E+03	1.960E+00	4.225E+02	-7.107E+03	8.033E+04	0.238	0.263	0.237	0.262	0.000	2.607E+06	2.892E+03
Synechococcus CC9605	NC_007516	8.084E-01	1.059E-02	6.404E+00	6.592E-01	-4.278E+05	6.722E-01	1.190E-02	9.788E-01	5.085E+02	-4.278E+05	-1.19E+01	exponential-log normal	6.446E+00	7.116E-01	-1.252E+04	2.201E+00	3.643E+02	-1.255E+04	6.831E+04	0.204	0.296	0.204	0.296	0.000	2.511E+06	2.645E+03
Synechococcus CC9902	NC_007513	8.353E-01	1.223E-02	6.418E+00	6.784E-01	-4.114E+05	7.400E-01	1.333E-02	9.814E-01	5.480E+02	-4.115E+05	-7.77E+01	exponential-log normal	6.649E+00	6.498E-01	-3.979E+03	2.814E+00	3.316E+02	-3.965E+03	6.753E+04	0.230	0.271	0.228	0.270	0.000	2.235E+06	2.307E+03
Synechococcus elongatus	NC_006576	8.372E-01	1.286E-02	6.502E+00	6.617E-01	-3.616E+05	7.685E-01	1.404E-02	1.174E+00	5.330E+02	-3.616E+05	2.56E+01	exponential-gamma	6.662E+00	6.368E-01	-6.409E+03	2.776E+00	3.414E+02	-6.410E+03	5.952E+04	0.223	0.277	0.222	0.277	0.000	2.696E+06	2.527E+03
Synechococcus RCC307	NC_009482	8.235E-01	9.733E-03	6.475E+00	6.422E-01	-3.709E+05	6.916E-01	1.092E-02	1.005E+00	5.219E+02	-3.709E+05	-2.62E+01	exponential-log normal	6.482E+00	7.424E-01	-9.562E+03	2.073E+00	4.091E+02	-9.574E+03	5.892E+04	0.195	0.305	0.196	0.304	0.000	2.225E+06	2.535E+03
Synechococcus sp	NC_005070	7.789E-01	1.143E-02	6.283E+00	7.341E-01	-4.195E+05	6.261E-01	1.183E-02	7.370E-01	6.244E+02	-4.199E+05	-7.93E+02	exponential-log normal	6.524E+00	7.043E-01	-1.304E+04	2.122E+00	4.128E+02	-1.313E+04	6.696E+04	0.204	0.297	0.202	0.297	0.000	2.434E+06	2.519E+03
Synechococcus WH	NC_009481	7.829E-01	1.037E-02	6.389E+00	6.666E-01	-4.101E+05	6.246E-01	1.178E-02	9.497E-01	5.146E+02	-4.101E+05	-2.24E+01	exponential-log normal	6.526E+00	7.148E-01	-1.219E+04	2.235E+00	3.870E+02	-1.220E+04	6.463E+04	0.200	0.301	0.198	0.301	0.000	2.367E+06	2.533E+03
Synechocystis PCC6803	NC_000911	8.424E-01	1.539E-02	6.438E+00	7.447E-01	-6.344E+05	7.736E-01	1.642E-02	9.092E-01	6.674E+02	-6.345E+05	-1.71E+02	exponential-log normal	6.678E+00	6.599E-01	-1.582E+04	2.502E+00	3.939E+02	-1.587E+04	1.071E+05	0.262	0.238	0.261	0.239	0.000	3.573E+06	3.172E+03
Syntrophobacter fumaroxidans	NC_008554	7.739E-01	8.602E-03	6.616E+00	6.420E-01	-9.868E+05	6.188E-01	1.017E-02	1.051E+00	5.882E+02	-9.868E+05	2.87E+01	exponential-gamma	6.701E+00	6.503E-01	-2.129E+04	2.597E+00	3.836E+02	-2.133E+04	1.502E+05	0.199	0.299	0.201	0.300	0.000	4.990E+06	4.064E+03
Syntrophomonas wolfei	NC_008346	7.923E-01	1.741E-02	6.338E+00	7.796E-01	-5.785E+05	7.015E-01	1.896E-02	8.425E-01	6.603E+02	-5.785E+05	3.27E+01	exponential-gamma	6.645E+00	6.652E-01	-9.805E+03	2.475E+00	3.843E+02	-9.832E+03	9.649E+04	0.275	0.226	0.276	0.223	0.000	2.936E+06	2.504E+03
Syntrophus aciditrophicus	NC_007759	8.012E-01	1.229E-02	6.466E+00	6.736E-01	-7.046E+05	7.061E-01	1.375E-02	1.097E+00	5.381E+02	-7.045E+05	1.28E+02	exponential-gamma	6.535E+00	7.608E-01	-1.670E+04	2.027E+00	4.430E+02	-1.670E+04	1.133E+05	0.242	0.256	0.243	0.259	0.000	3.179E+06	3.168E+03
Thermoanaerobacter tengcongensis	NC_003869	7.414E-01	2.661E-02	6.027E+00	8.881E-01	-5.007E+05	6.173E-01	2.908E-02	6.371E-01	6.585E+02	-5.004E+05	4.66E+02	exponential-gamma	6.620E+00	7.207E-01	-6.025E+03	2.297E+00	4.129E+02	-6.011E+03	8.598E+04	0.313	0.188	0.312	0.188	0.000	2.689E+06	2.588E+03
Thermosipho melanesiensis	NC_009616	8.096E-01	2.656E-02	6.187E+00	8.096E-01	-3.572E+05	7.520E-01	2.872E-02	9.349E-01	5.604E+02	-3.571E+05	3.67E+02	exponential-gamma	6.658E+00	6.343E-01	-5.284E+03	2.916E+00	3.210E+02	-5.268E+03	6.365E+04	0.346	0.159	0.340	0.155	0.000	1.915E+06	1.879E+03
Thermosynechococcus elongatus	NC_004113	8.458E-01	1.277E-02	6.515E+00	6.639E-01	-4.434E+05	7.776E-01	1.389E-02	1.141E+00	5.503E+02	-4.434E+05	7.61E+00	exponential-gamma	6.628E+00	6.960E-01	-1.377E+04	2.373E+00	3.981E+02	-1.377E+04	7.325E+04	0.230	0.269	0.231	0.270	0.000	2.594E+06	2.476E+03
Thermotoga maritima	NC_000853	7.216E-01	1.511E-02	6.266E+00	7.364E-01	-3.204E+05	5.244E-01	1.783E-02	7.372E-01	5.917E+02	-3.203E+05	1.58E+02	exponential-gamma	6.643E+00	6.559E-01	-6.333E+03	2.719E+00	3.429E+02	-6.319E+03	5.110E+04	0.268	0.228	0.270	0.235	0.000	1.861E+06	1.858E+03
Thermotoga petrophila	NC_009486	7.204E-01	1.533E-02	6.262E+00	7.389E-01	-3.156E+05	5.248E-01	1.813E-02	7.367E-01	5.924E+02	-3.155E+05	1.67E+02	exponential-gamma	6.670E+00	6.103E-01	-8.545E+03	3.002E+00	3.112E+02	-8.544E+03	5.037E+04	0.269	0.234	0.270	0.227	0.000	1.824E+06	1.785E+03
Thermus thermophilus	NC_005835	7.154E-01	8.087E-03	6.550E+00	6.136E-01	-1.794E+05	4.642E-01	1.078E-02	1.004E+00	5.309E+02	-1.794E+05	-5.90E+01	exponential-log normal	6.576E+00	6.232E-01	-5.040E+03	2.863E+00	3.021E+02	-5.044E+03	2.671E+04	0.152	0.347	0.153	0.348	0.000	1.895E+06	1.982E+03
Thiobacillus denitrificans	NC_007404	6.976E-01	7.617E-03	6.598E+00	6.607E-01	-4.526E+05	4.288E-01	9.687E-03	8.840E-01	6.412E+02	-4.527E+05	-1.49E+02	exponential-log normal	6.638E+00	6.497E-01	-1.571E+04	2.533E+00	3.726E+02	-1.577E+04	6.632E+04	0.170	0.330	0.170	0.331	0.000	2.910E+06	2.827E+03
Thiomicrospira crunogena	NC_007520	8.206E-01	1.732E-02	6.484E+00	6.997E-01	-5.388E+05	7.765E-01	1.861E-02	1.284E+00	5.363E+02	-5.387E+05	1.47E+02	exponential-gamma	6.721E+00	6.218E-01	-6.043E+03	2.833E+00	3.544E+02	-6.053E+03	9.083E+04	0.284	0.213	0.285	0.218	0.000	2.428E+06	2.196E+03
Thiomicrospira denitrificans	NC_007575	8.120E-01	2.609E-02	6.305E+00	7.926E-01	-4.724E+05	7.645E-01	2.812E-02	1.018E+00	5.936E+02	-4.722E+05	3.20E+02	exponential-gamma	6.630E+00	6.712E-01	-5.560E+03	2.409E+00	3.929E+02	-5.575E+03	8.363E+04	0.325	0.171	0.330	0.173	0.000	2.202E+06	2.097E+03
Treponema denticola	NC_002967	7.917E-01	2.039E-02	6.382E+00	7.804E-01	-5.184E+05	7.211E-01	2.235E-02	9.201E-01	6.676E+02	-5.183E+05	7.17E+01	exponential-gamma	6.594E+00	8.164E-01	-1.180E+04	1.936E+00	4.988E+02	-1.174E+04	8.766E+04	0.311	0.191	0.310	0.188	0.000	2.843E+06	2.767E+03
Treponema pallidum	NC_000919	8.104E-01	1.082E-02	6.546E+00	6.608E-01	-2.018E+05	7.109E-01	1.216E-02	1.104E+00	5.625E+02	-2.018E+05	5.66E+01	exponential-gamma	6.710E+00	7.307E-01	-5.795E+03	2.337E+00	4.427E+02	-5.763E+03	3.207E+04	0.237	0.262	0.235	0.266	0.000	1.138E+06	1.036E+03
Trichodesmium erythraeum	NC_008312	8.879E-01	2.545E-02	6.396E+00	9.153E-01	-1.149E+06	8.433E-01	2.628E-02	6.251E-01	1.070E+03	-1.150E+06	-7.98E+02	exponential-log normal	6.611E+00	8.073E-01	-2.971E+04	1.670E+00	6.204E+02	-2.992E+04	2.149E+05	0.329	0.170	0.329	0.171	0.000	7.750E+06	4.451E+03
Tropheryma whipplei	NC_004551	8.485E-01	1.586E-02	6.360E+00	7.548E-01	-1.717E+05	7.686E-01	1.696E-02	8.286E-01	6.428E+02	-1.717E+05	3.97E+01	exponential-gamma	6.703E+00	6.582E-01	-5.044E+03	2.566E+00	3.904E+02	-5.053E+03	2.934E+04	0.272	0.231	0.265	0.232	0.000	9.259E+05	7.830E+02
uncultured methanogenic	NC_009464	7.533E-01	1.200E-02	6.280E+00	6.826E-01	-6.561E+05	5.848E-01	1.389E-02	9.323E-01	4.804E+02	-6.561E+05	9.52E+01	exponential-gamma	6.614E+00	6.174E-01	-7.259E+03	2.904E+00	3.076E+02	-7.269E+03	1.041E+05	0.228	0.273	0.226	0.273	0.000	3.180E+06	3.085E+03
Verminephrobacter eiseniae	NC_008786	7.263E-01	7.488E-03	6.585E+00	6.458E-01	-1.234E+06	4.803E-01	9.033E-03	9.069E-01	6.130E+02	-1.234E+06	-9.51E+02	exponential-log normal	6.736E+00	6.056E-01	-2.496E+04	2.923E+00	3.438E+02	-2.504E+04	1.827E+05	0.173	0.327	0.174	0.326	0.000	5.567E+06	4.908E+03
Wolbachia endosymbiont	NC_006833	8.501E-01	2.493E-02	6.145E+00	8.993E-01	-2.026E+05	7.801E-01	2.591E-02	6.236E-01	7.784E+02	-2.026E+05	1.48E+01	exponential-gamma	6.538E+00	7.368E-01	-5.240E+03	2.046E+00	4.396E+02	-5.260E+03	3.699E+04	0.326	0.177	0.332	0.165	0.000	1.080E+06	8.050E+02
Xanthomonas campestris	NC_003902	7.609E-01	7.459E-03	6.642E+00	6.432E-01	-9.586E+05	5.622E-01	8.832E-03	9.630E-01	6.259E+02	-9.588E+05	-2.64E+02	exponential-log normal	6.747E+00	6.612E-01	-1.810E+04	2.538E+00	4.115E+02	-1.814E+04	1.432E+05	0.174	0.325	0.175	0.325	0.000	5.076E+06	4.181E+03
Xanthomonas oryzae	NC_006834	7.711E-01	7.686E-03	6.582E+00	6.424E-01	-9.560E+05	5.766E-01	8.997E-03	9.714E-01	5.813E+02	-9.560E+05	-1.40E+02	exponential-log normal	6.631E+00	7.160E-01	-1.881E+04	2.247E+00	4.291E+02	-1.882E+04	1.443E+05	0.181	0.319	0.182	0.318	0.000	4.941E+06	4.145E+03
Xylella fastidiosa	NC_002488	8.392E-01	1.306E-02	6.400E+00	7.763E-01	-5.841E+05	7.514E-01	1.361E-02	7.706E-01	7.272E+02	-5.842E+05	-1.75E+02	exponential-log normal	6.363E+00	8.631E-01	-8.696E+03	1.559E+00	5.281E+02	-8.726E+03	9.678E+04	0.248	0.249	0.225	0.277	0.000	2.679E+06	2.766E+03
Yersinia enterocolitica	NC_008800	8.231E-01	1.596E-02	6.465E+00	7.025E-01	-9.817E+05	7.558E-01	1.744E-02	1.054E+00	5.953E+02	-9.819E+05	-3.28E+02	exponential-log normal	6.704E+00	6.392E-01	-1.154E+04	2.747E+00	3.605E+02	-1.155E+04	1.644E+05	0.264	0.237	0.264	0.236	0.000	4.616E+06	3.979E+03
Yersinia pestis	NC_008150	8.191E-01	1.611E-02	6.420E+00	7.403E-01	-9.823E+05	7.336E-01	1.751E-02	8.604E-01	6.787E+02	-9.827E+05	-7.79E+02	exponential-log normal	6.615E+00	6.932E-01	-1.812E+04	2.316E+00	4.062E+02	-1.817E+04	1.645E+05	0.261	0.239	0.262	0.238	0.000	4.702E+06	4.167E+03
Yersinia pseudotuberculosis	NC_006155	8.192E-01	1.630E-02	6.440E+00	7.647E-01	-9.869E+05	7.321E-01	1.758E-02	7.940E-01	7.583E+02	-9.874E+05	-1.09E+03	exponential-log normal	6.681E+00	6.714E-01	-1.940E+04	2.342E+00	4.275E+02	-1.951E+04	1.652E+05	0.262	0.237	0.262	0.239	0.000	4.745E+06	3.901E+03
