# Supplementary material for: The Random Nature of Genome Architecture: Predicting Open Reading Frame Distributions
Source: PLoS One. 2009 Jul 30;4(7):e6456. doi: 10.1371/journal.pone.0006456 (PMC2714469; doi:10.1371/journal.pone.0006456)

Appendix 2. Plot showing that we would expect the probability of a stop codon to decline with increasing GC content.

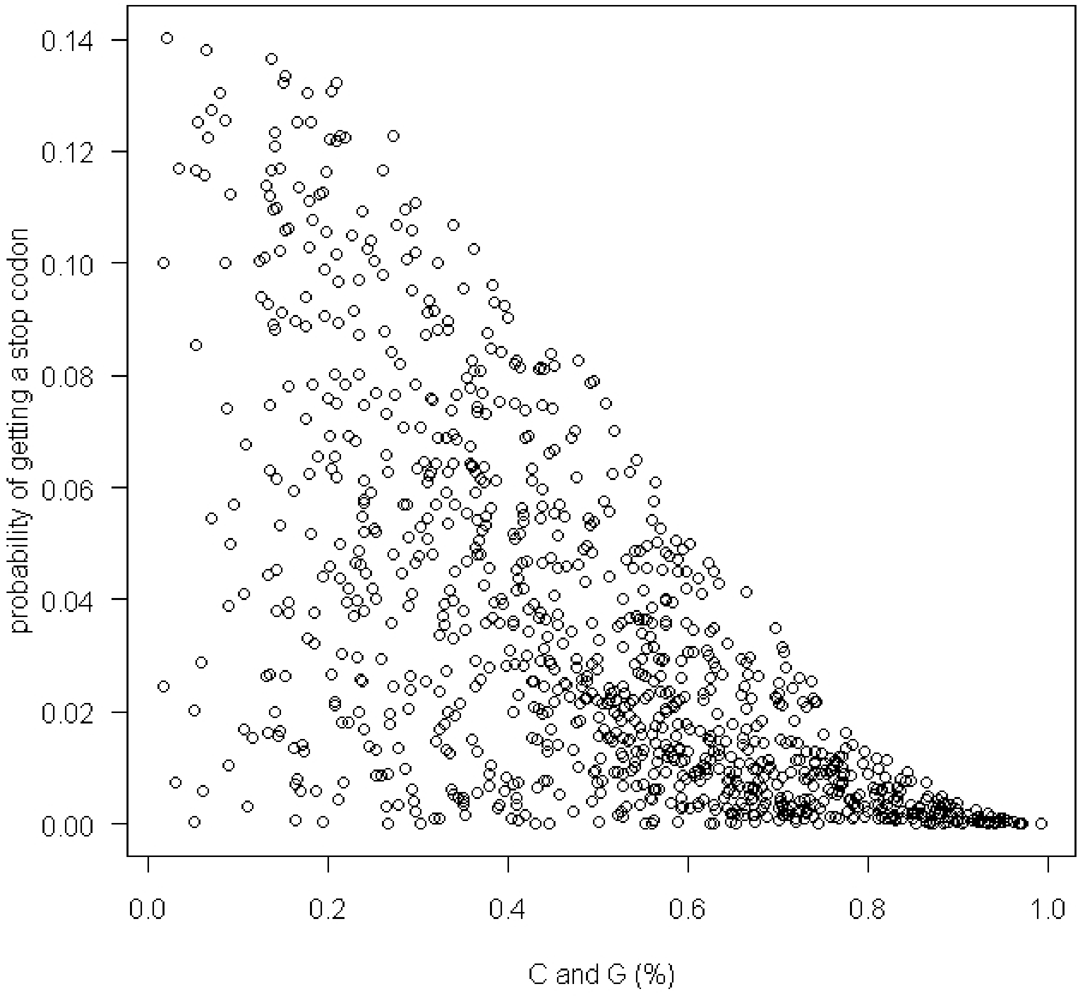

Supplement: Figure S1 — Supporting information for model development. Figure illustrating how the expected probability of a randomly generated stop codon declines with increasing GC content. (0.18 MB PDF) [file pone.0006456.s002.pdf]
