# Supplementary material for: The Random Nature of Genome Architecture: Predicting Open Reading Frame Distributions
Source: PLoS One. 2009 Jul 30;4(7):e6456. doi: 10.1371/journal.pone.0006456 (PMC2714469; doi:10.1371/journal.pone.0006456)

Supplement 3

Significant relationships and illustrations of the shapes of the size distributions of ORFS described by the non-random distribution of the exponential-log normal mixture model and annotated proteins. All parameter values are listed in Supplement 1. Within this supplement, figure one depicts the statistical relationships between the parameters estimated with the exponential-log normal model and parameters estimated from annotated proteins (also see figure 4 in main document), and figures two through 312 illustrate the shapes of each of these distributions (arranged alphabetically by species to correspond with Supplement 1). In these figures red lines represent the shapes of the distributions estimated from the mixture model and blue lines show the shape of the distribution from the annotated proteins. It is important to note from these figures that the shapes of the size distributions of non-random ORFs deviate from the annotated proteins such that the number of small non-random ORFs is consistently greater than the number of small annotated proteins. Consequently, the means of the lognormal distributions estimated from the mixture models are smaller and the peaks of the distributions shifted to the left of those for annotated proteins. Moreover, the magnitude of the shift is greater in multicellular eukaryotes than prokaryotes. Nevertheless, the mean and standard deviation estimated from the mixture model fits are significantly correlated with the parameter estimates from fits to annotated proteins (S4 – Fig. 1).

Supplement 3 – Figure 1

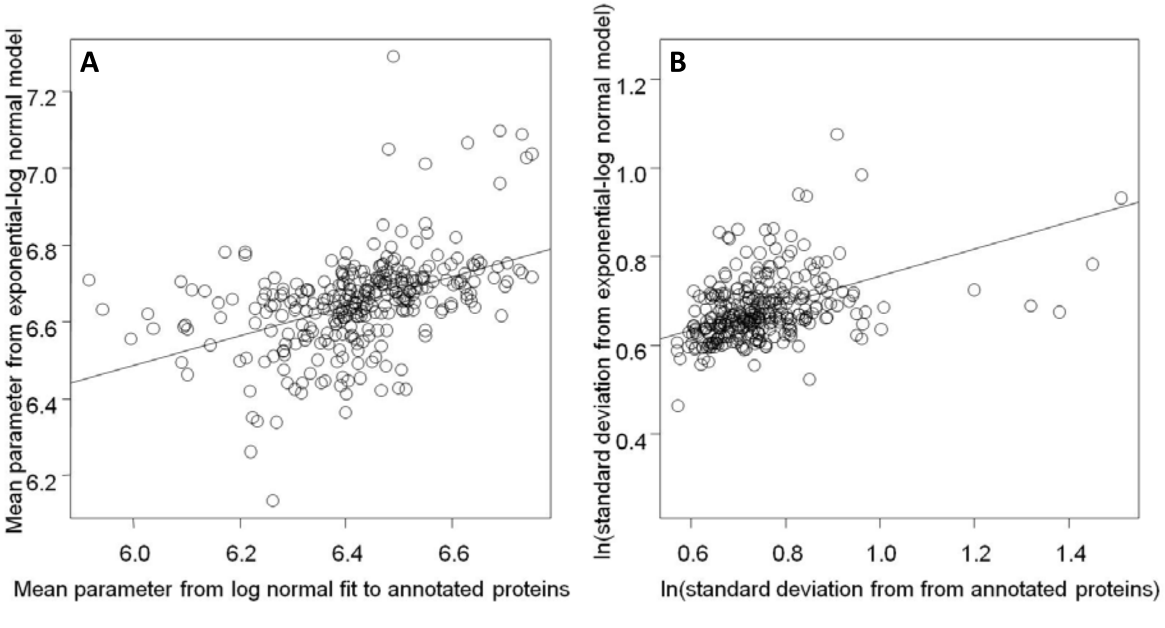

S3 - Figure 1. Plots of parameter estimates determined from the exponential-log normal mixture model fit to the ORF size distributions against parameter estimates determined from gamma model fits to annotated proteins. Panel **A** illustrates the significant relationship between the scale parameter estimates ($F_{1,309}$ = 76.21, $p < 0.0001$). Panel **B** illustrates the significant relationship between the shape parameters estimates ($F_{1,309}$ = 62.452, $p < 0.0001$).

Supplement 3 − Figure 2

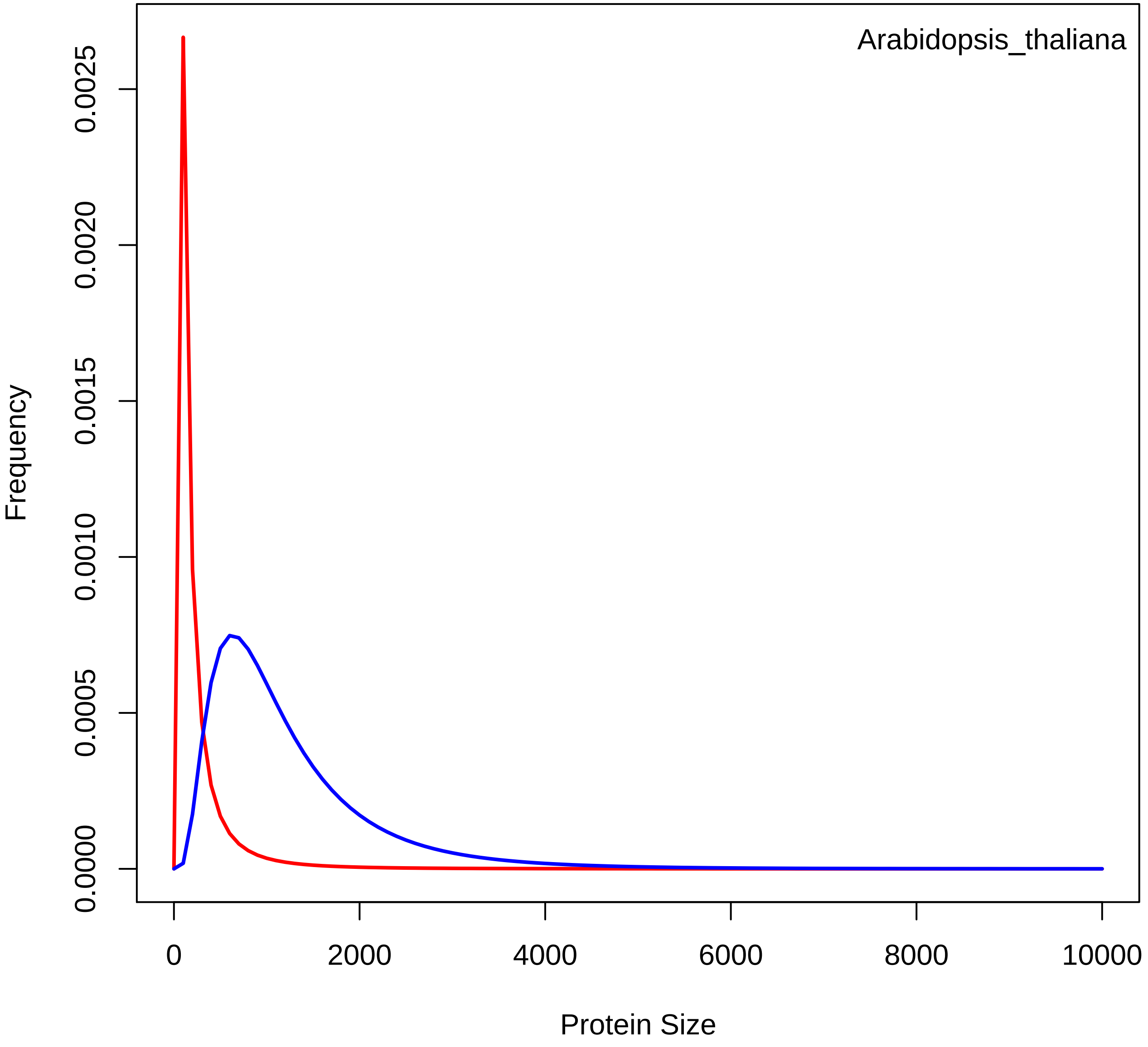

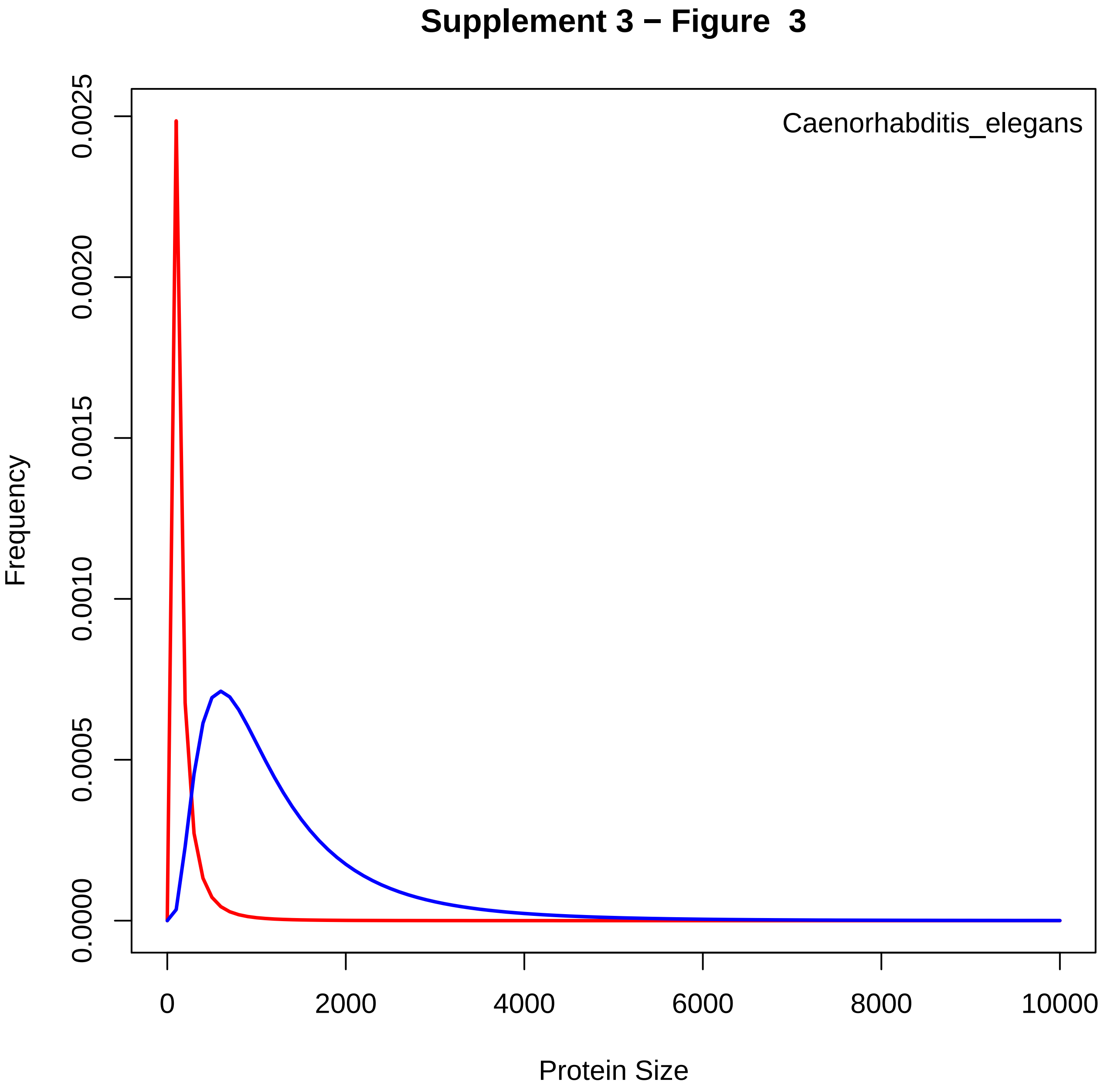
Supplement 3 − Figure 3
Caenorhabditis_elegans
Frequency
0.0000
0.0005
0.0010
0.0015
0.0020
0.0025
0
2000
4000
6000
8000
10000
Protein Size

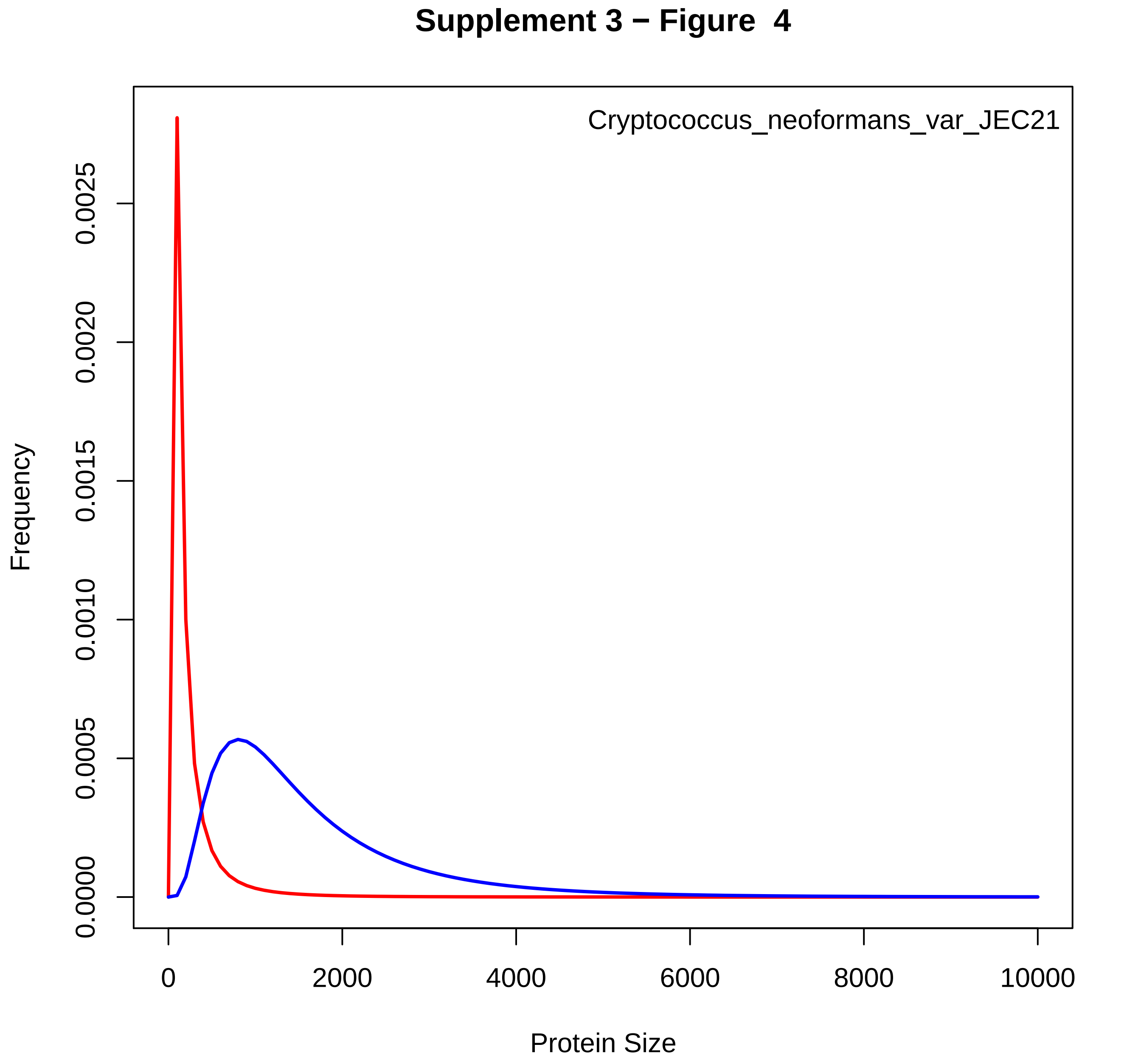

Supplement 3 − Figure 4
Cryptococcus_neoformans_var_JEC21
Frequency
0.0000
0.0005
0.0010
0.0015
0.0020
0.0025
0
2000
4000
6000
8000
10000
Protein Size

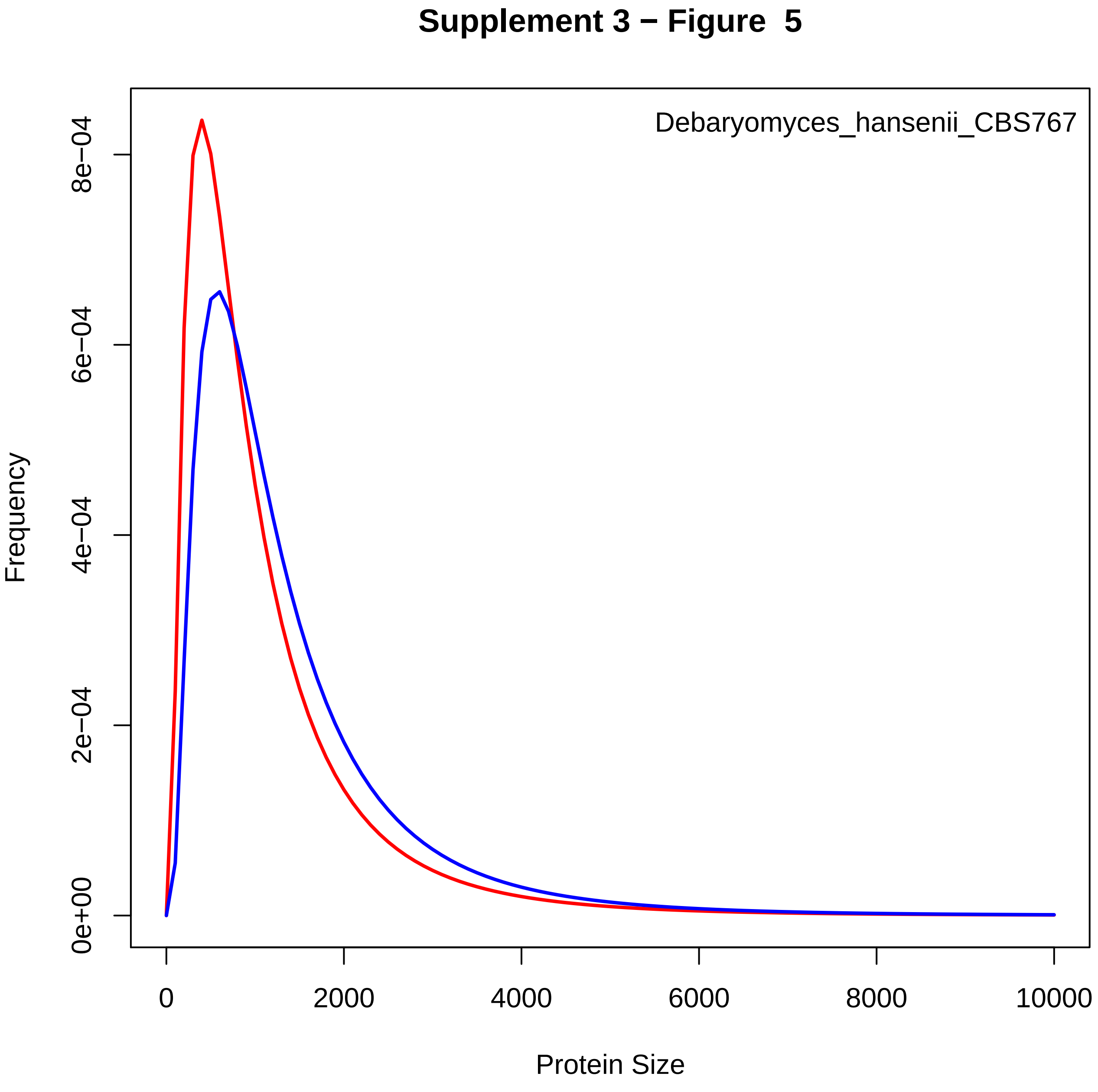
Supplement 3 − Figure 5
Debaryomyces_hansenii_CBS767
Frequency
0e+00
2e−04
4e−04
6e−04
8e−04
0
2000
4000
6000
8000
10000
Protein Size

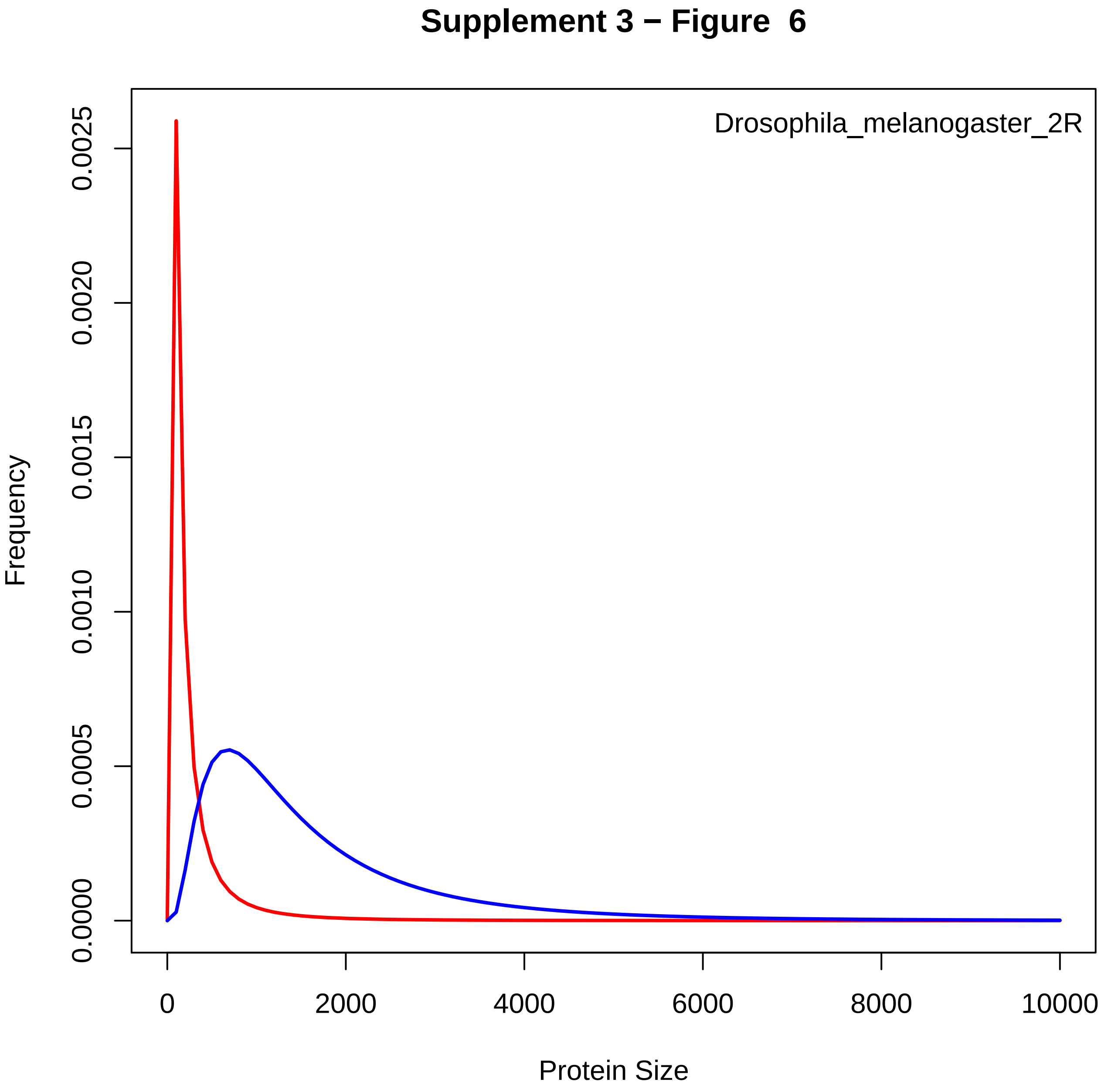

Supplement 3 − Figure 6
Drosophila_melanogaster_2R
Frequency
0.0000
0.0005
0.0010
0.0015
0.0020
0.0025
0
2000
4000
6000
8000
10000
Protein Size

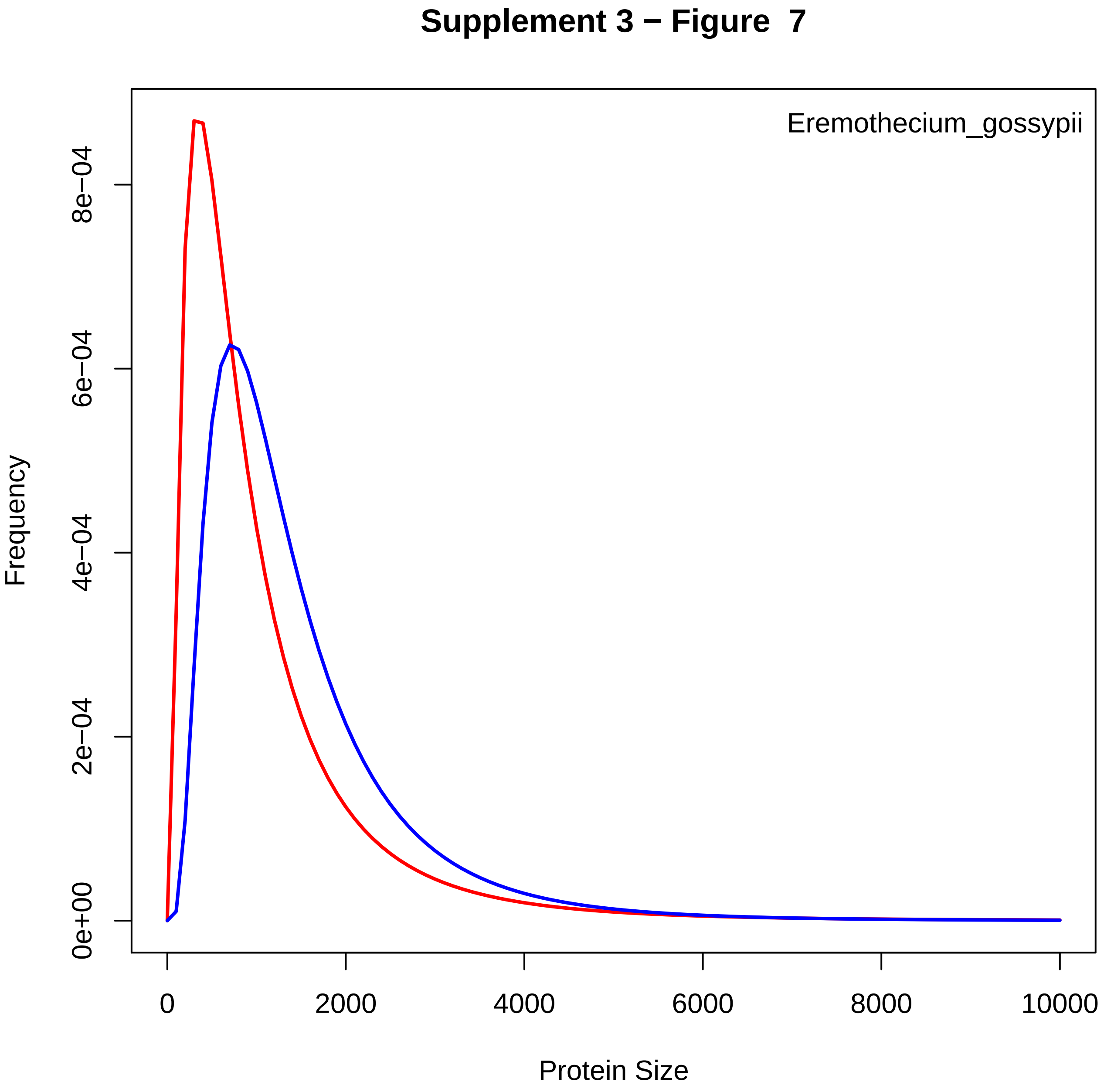
Supplement 3 − Figure 7
Eremothecium_gossypii
Frequency
0e+00
2e−04
4e−04
6e−04
8e−04
0
2000
4000
6000
8000
10000
Protein Size

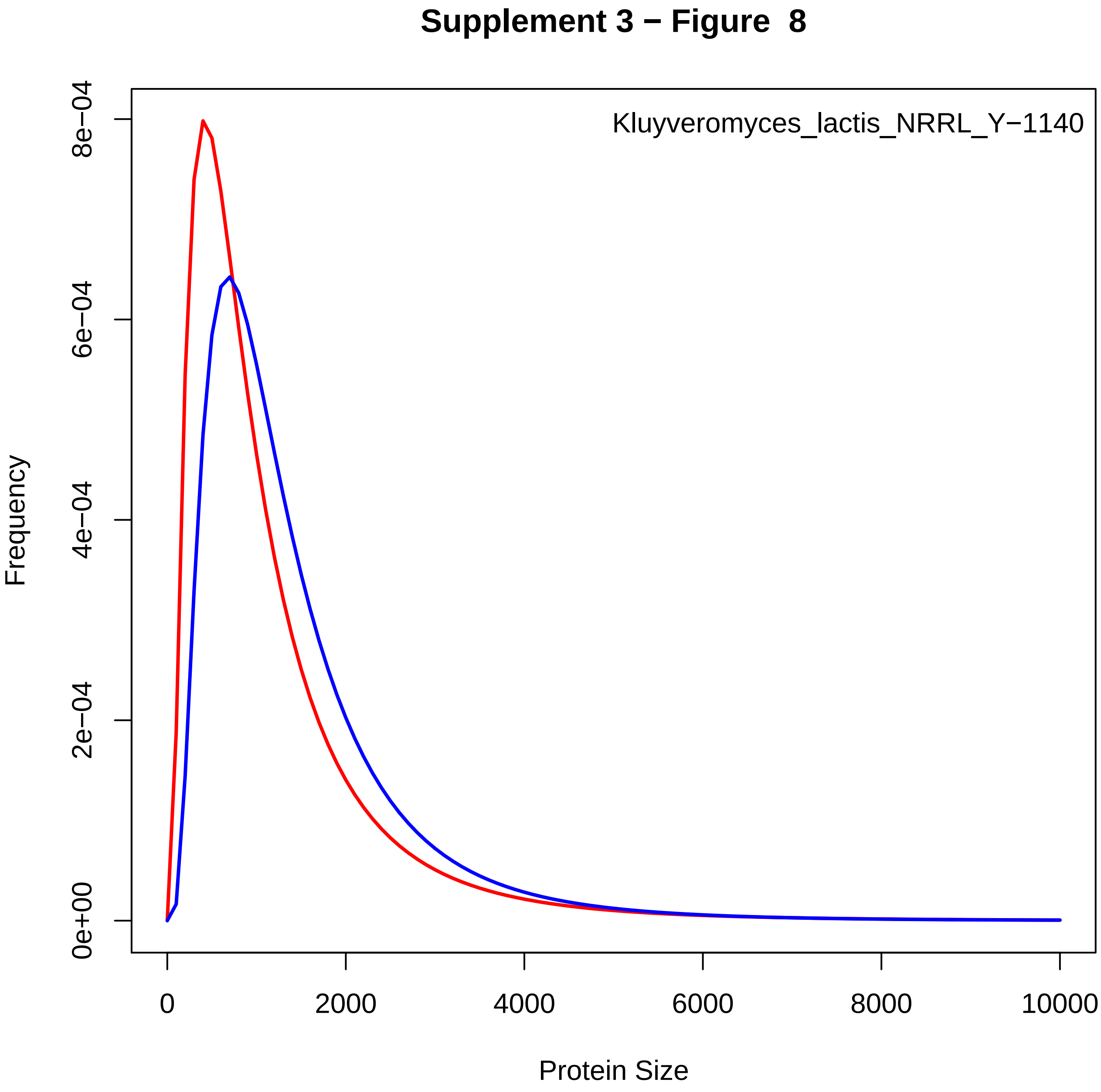
Supplement 3 − Figure 8
Kluyveromyces_lactis_NRRL_Y−1140
Frequency
0e+00
2e−04
4e−04
6e−04
8e−04
0
2000
4000
6000
8000
10000
Protein Size

Supplement 3 − Figure 9

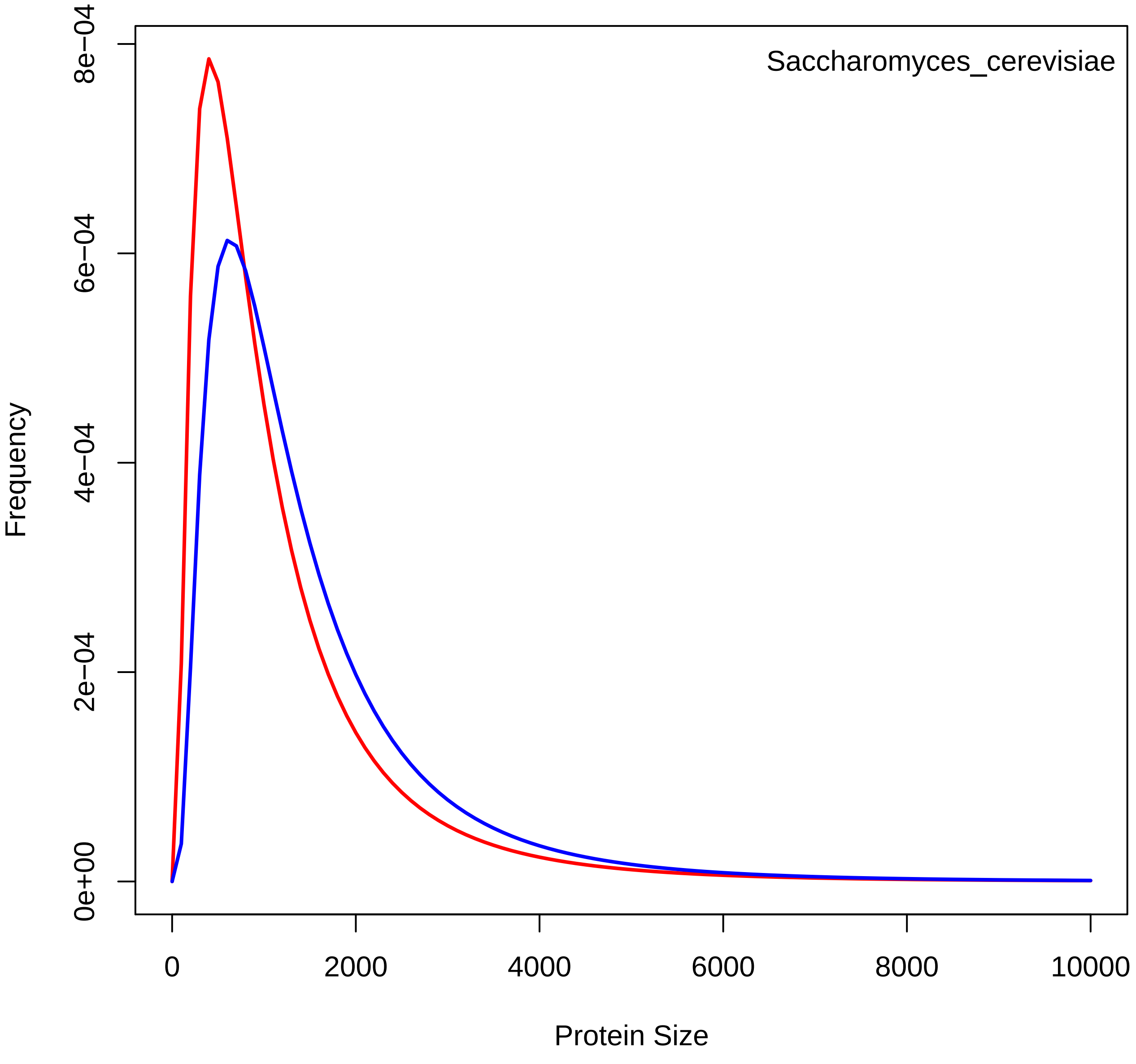

Supplement 3 − Figure 10

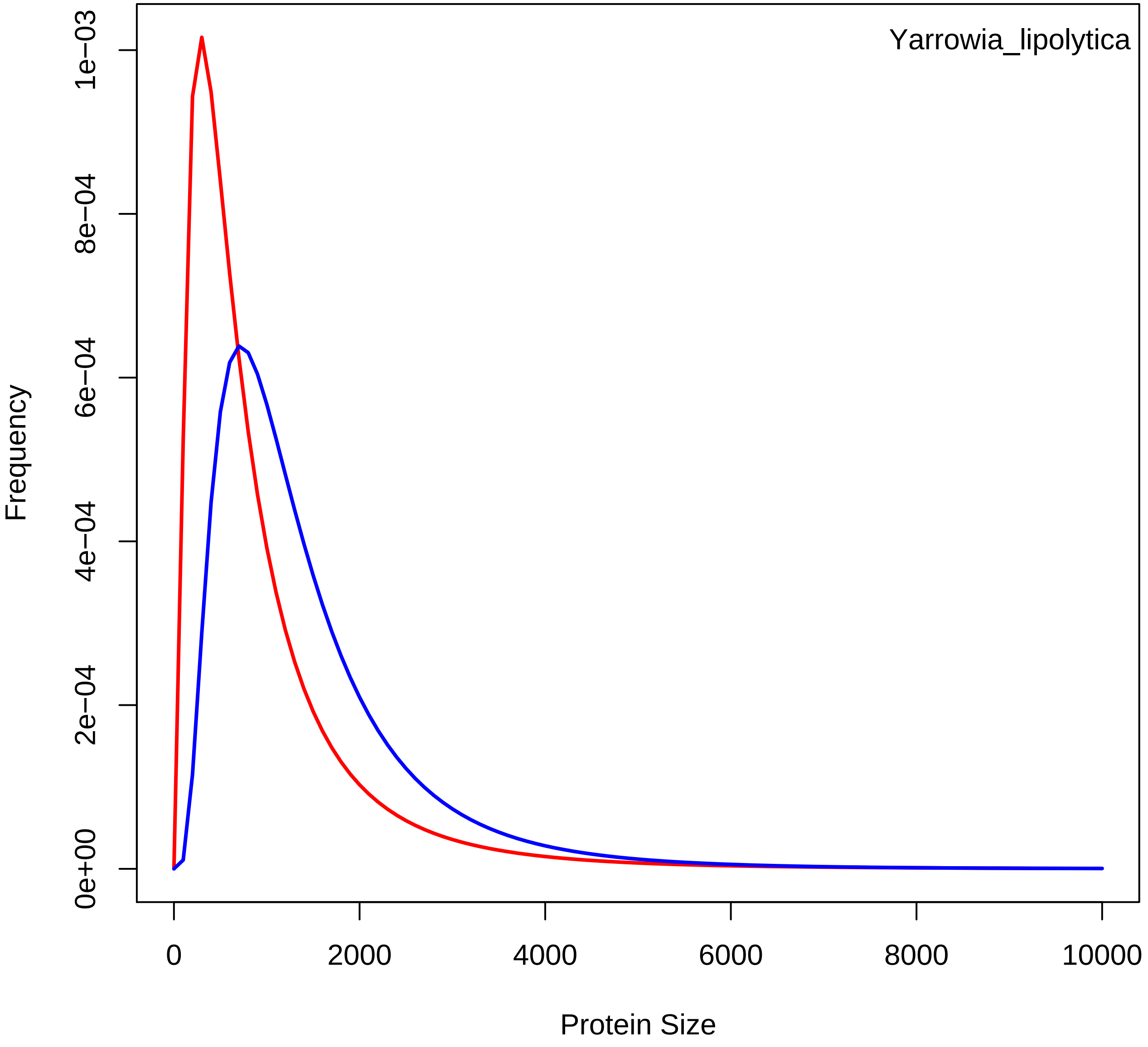

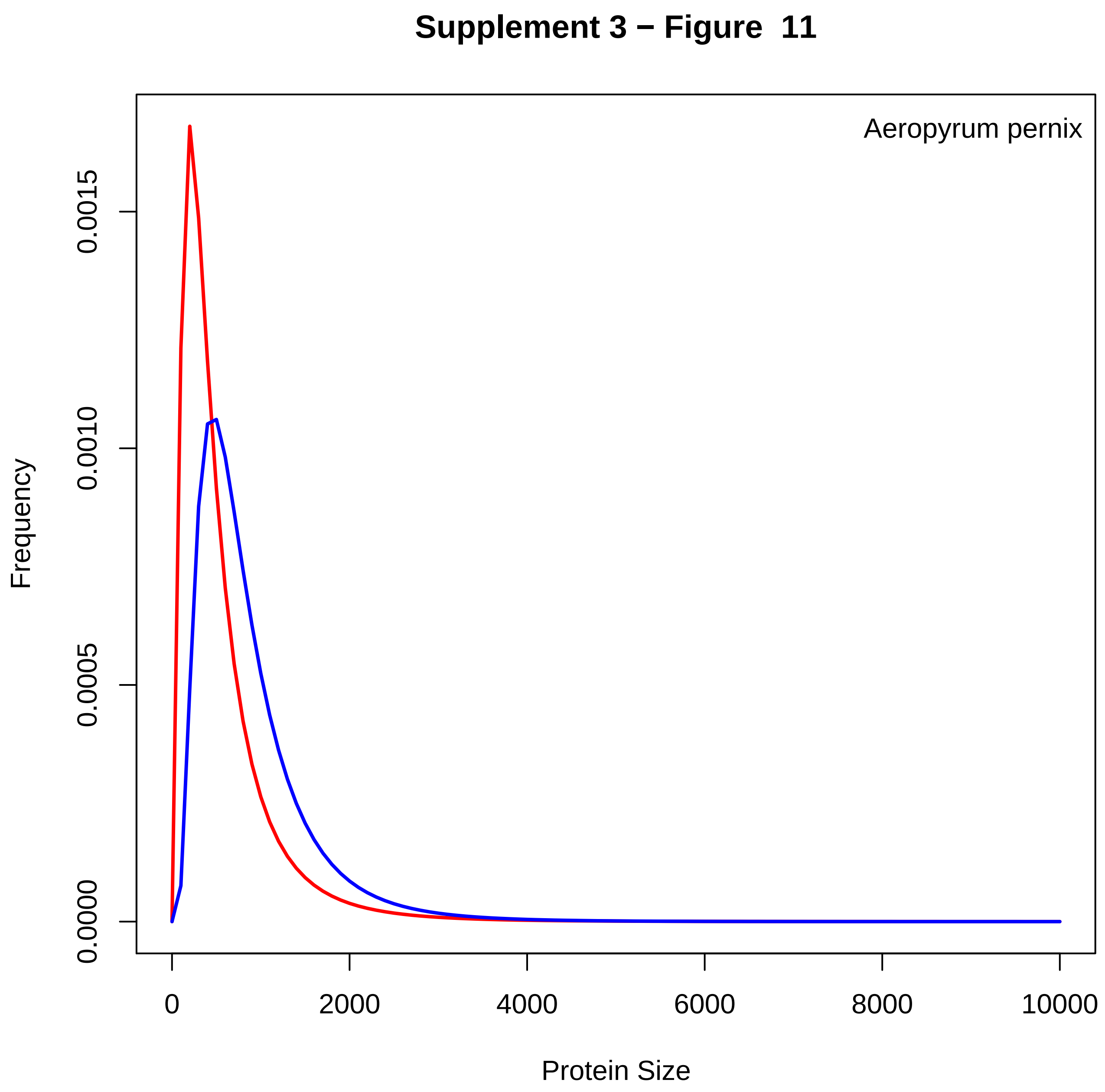
Supplement 3 − Figure 11
Aeropyrum pernix
Frequency
0.0000
0.0005
0.0010
0.0015
0
2000
4000
6000
8000
10000
Protein Size

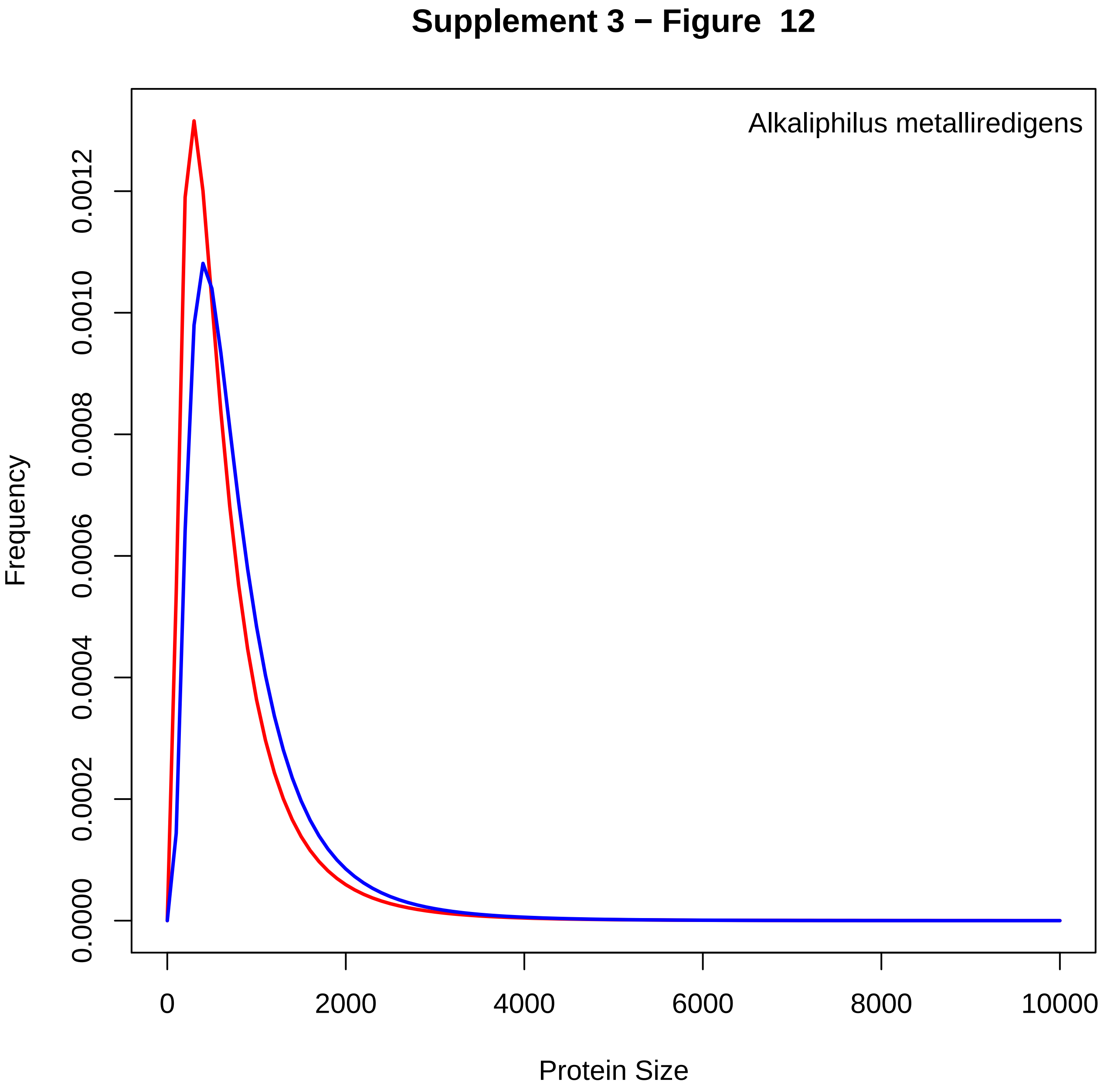
Supplement 3 − Figure 12
Alkaliphilus metalliredigens
Frequency
0.0000
0.0002
0.0004
0.0006
0.0008
0.0010
0.0012
0
2000
4000
6000
8000
10000
Protein Size

Supplement 3 − Figure 13

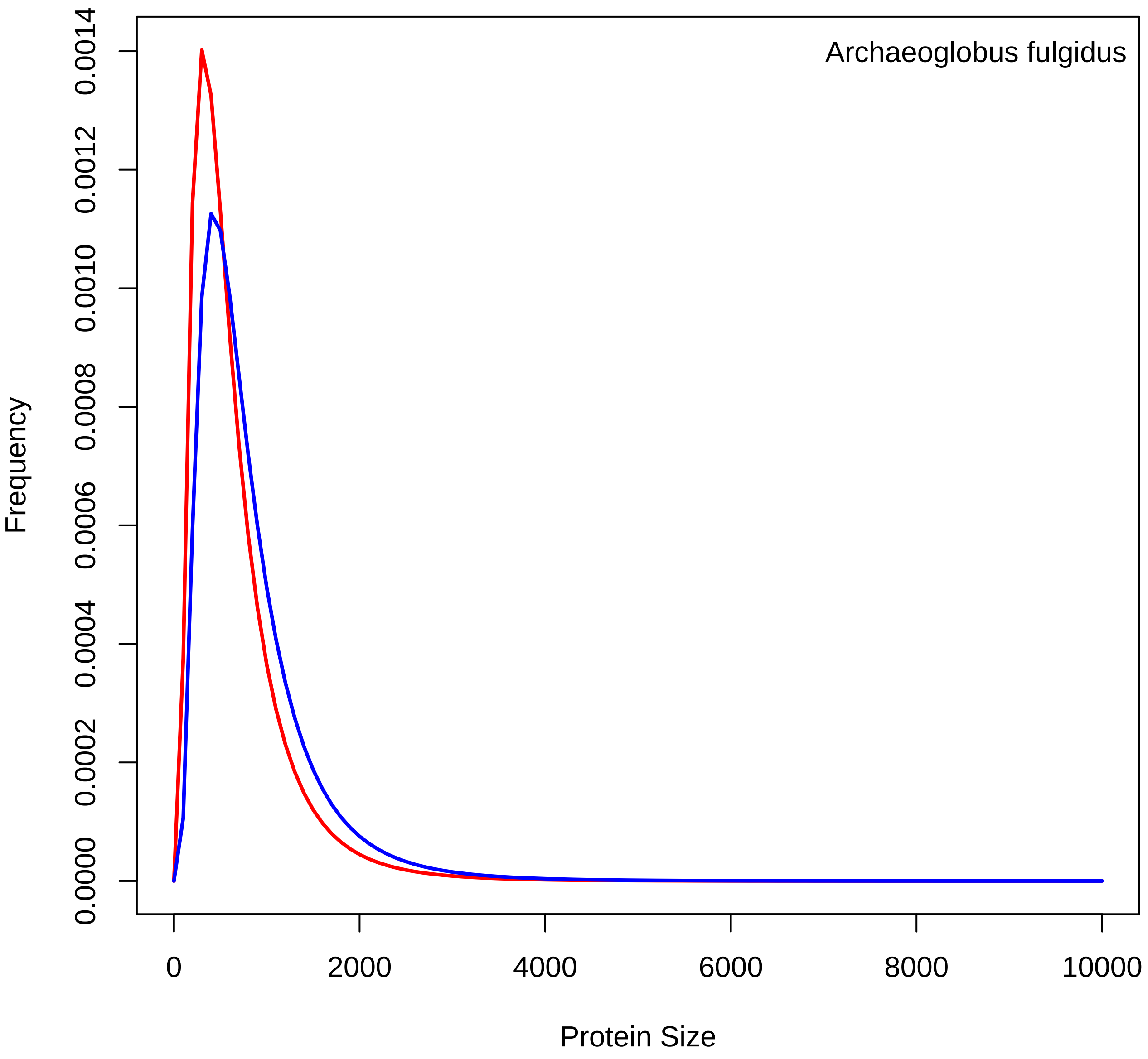

Supplement 3 − Figure 14

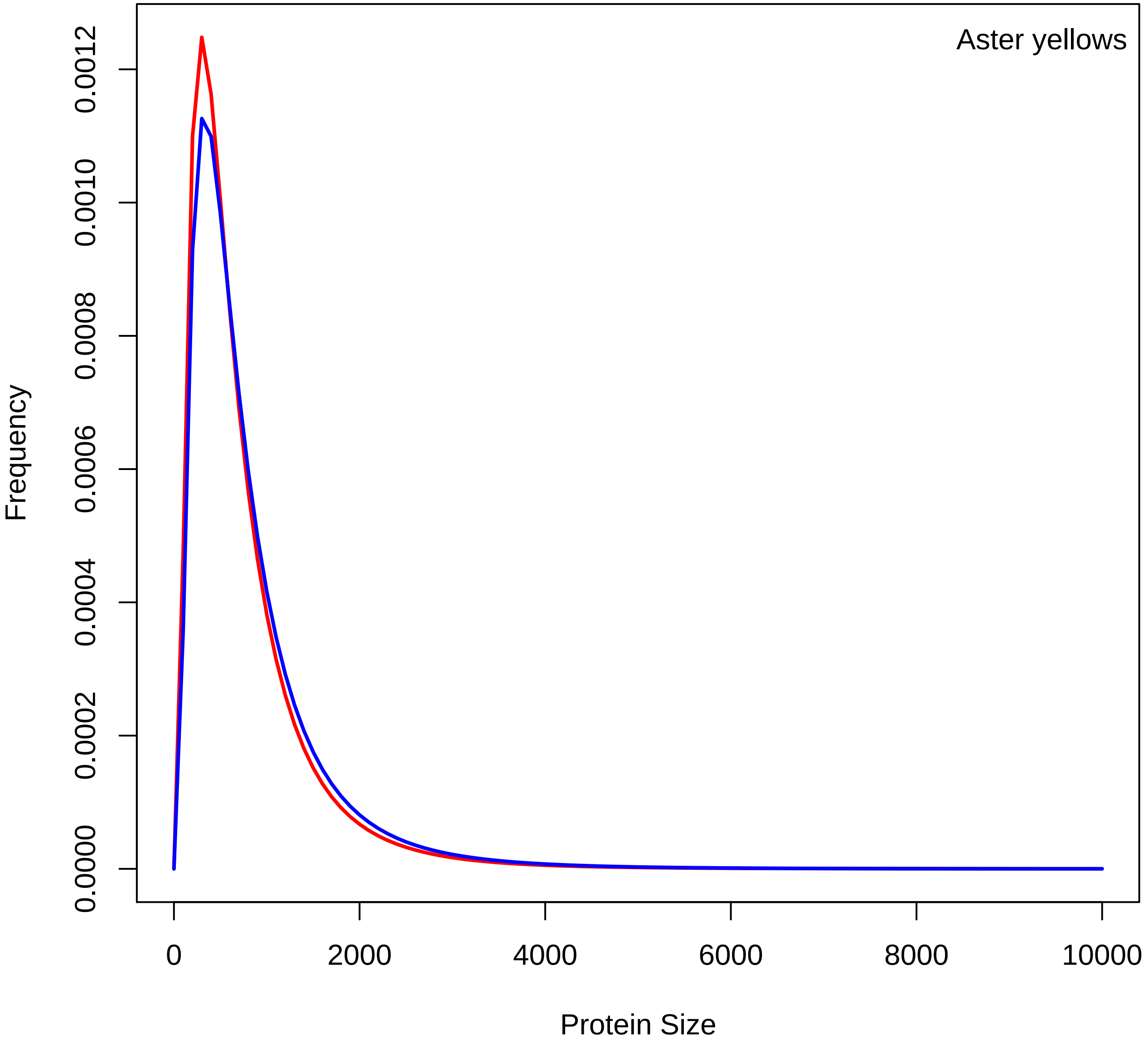

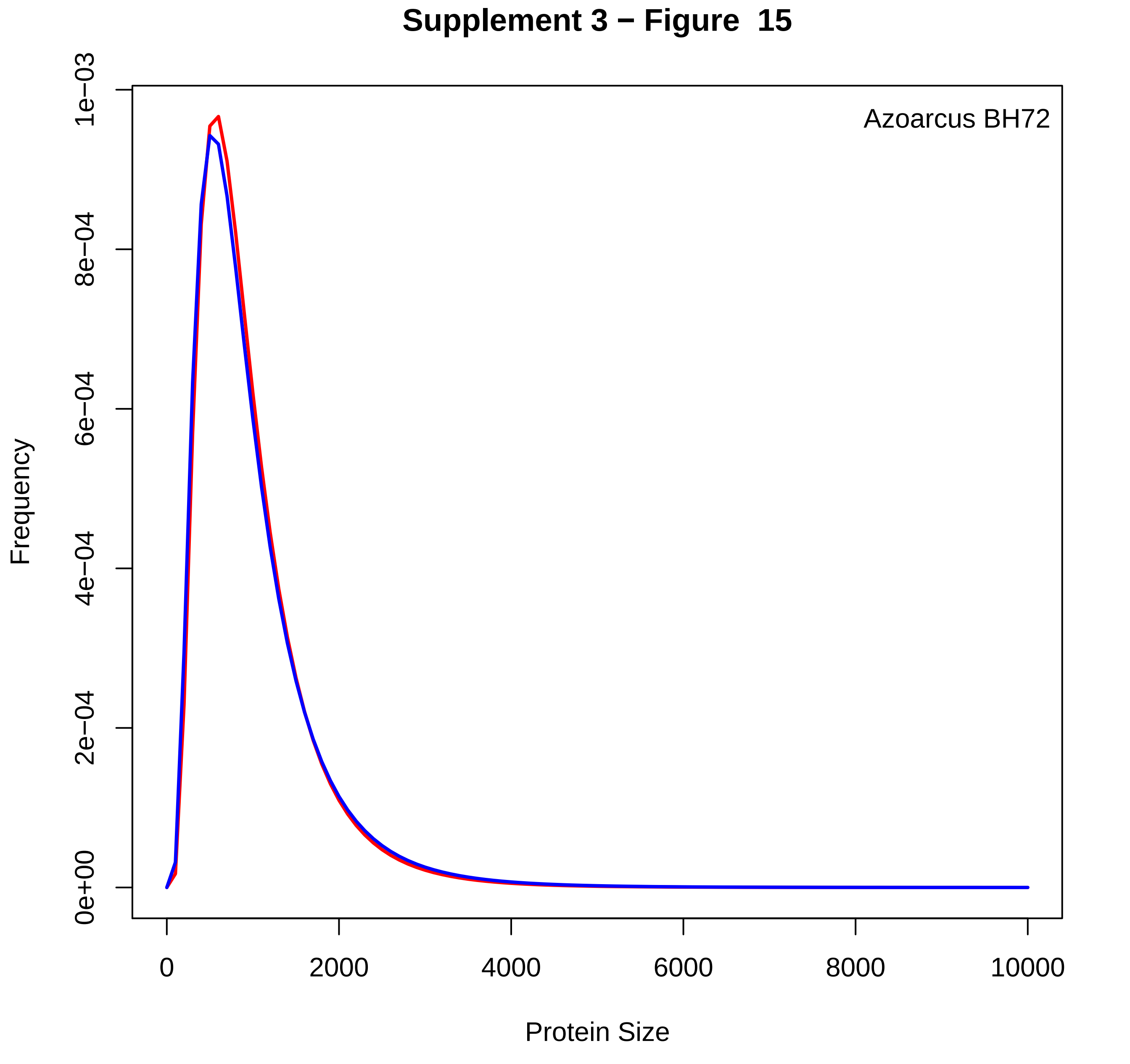
Supplement 3 − Figure 15
Azoarcus BH72
Frequency
0e+00
2e−04
4e−04
6e−04
8e−04
1e−03
0
2000
4000
6000
8000
10000
Protein Size

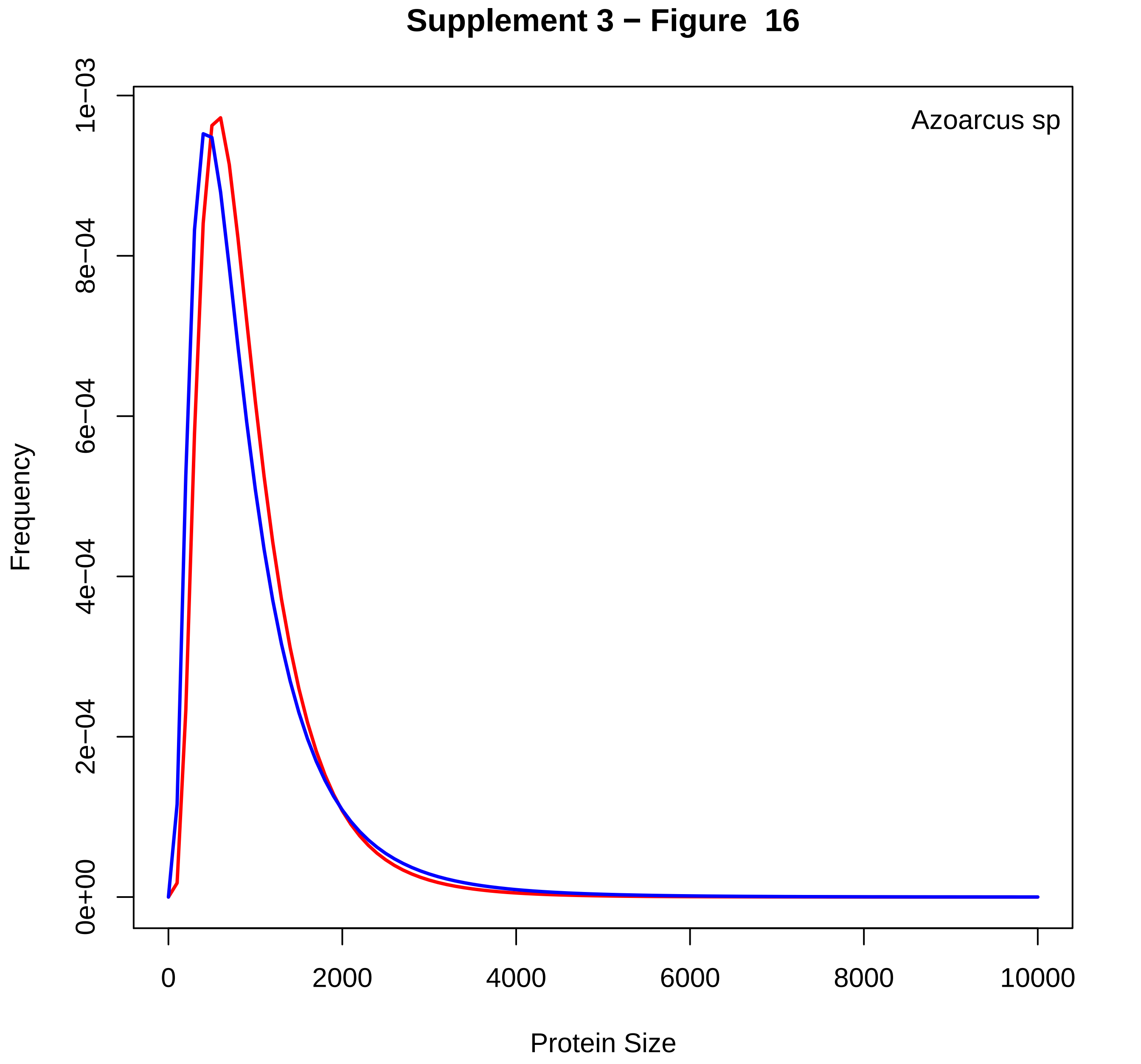
Supplement 3 − Figure 16
Azoarcus sp
Frequency
0e+00
2e−04
4e−04
6e−04
8e−04
1e−03
0
2000
4000
6000
8000
10000
Protein Size

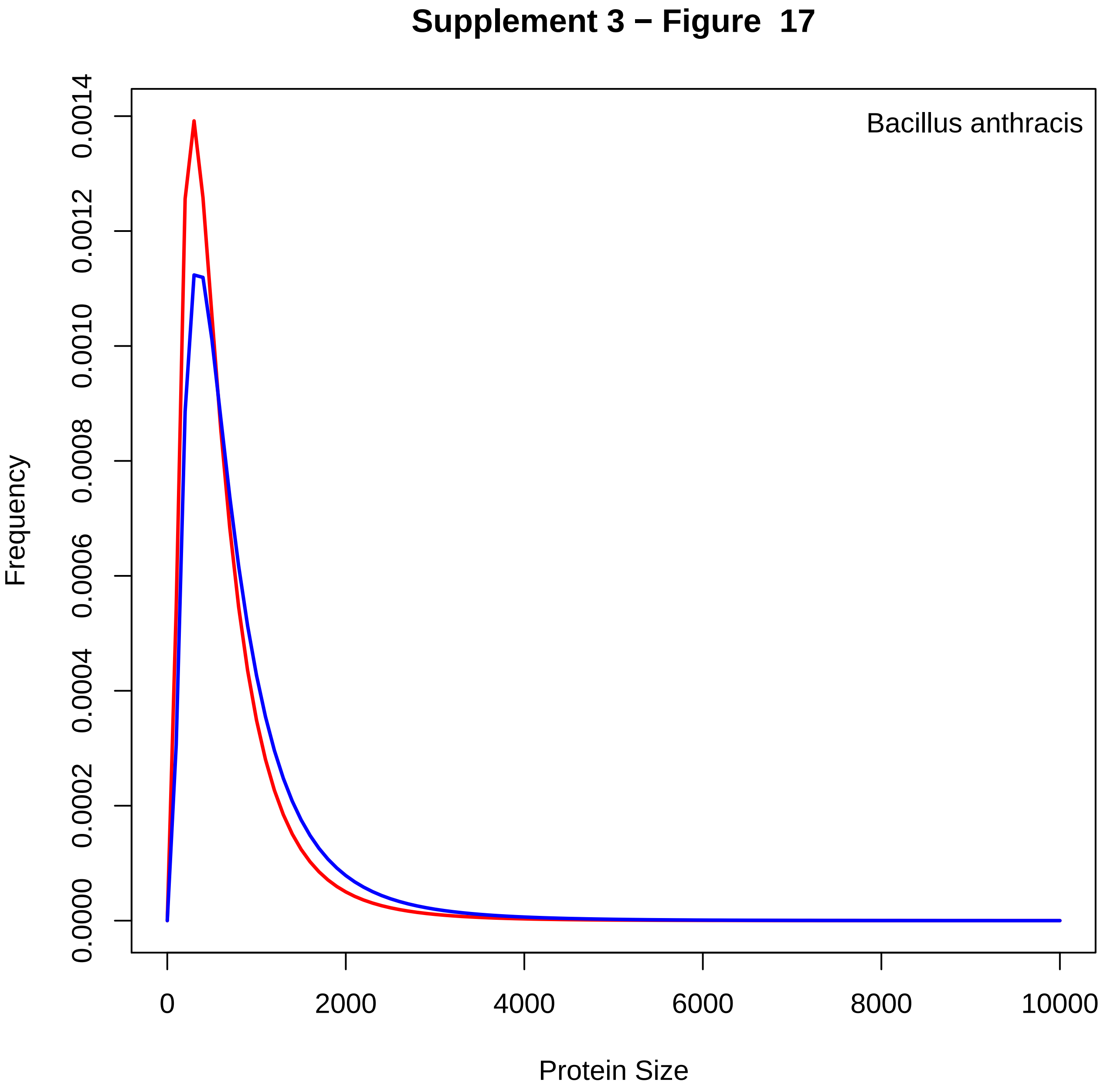
Supplement 3 − Figure 17
Bacillus anthracis
Frequency
0.0000
0.0002
0.0004
0.0006
0.0008
0.0010
0.0012
0.0014
0
2000
4000
6000
8000
10000
Protein Size

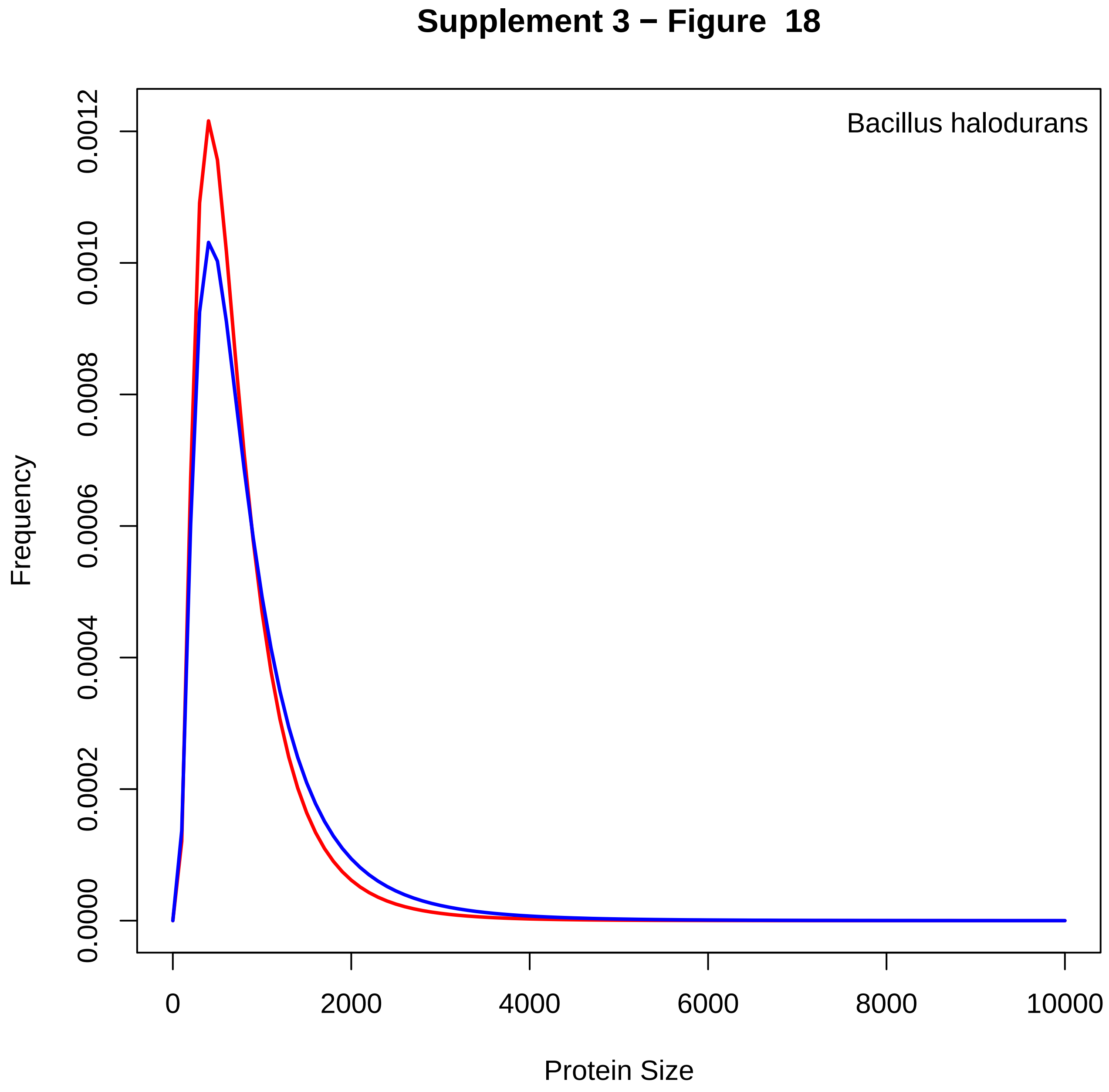
Supplement 3 − Figure 18
Bacillus halodurans
Frequency
0.0000
0.0002
0.0004
0.0006
0.0008
0.0010
0.0012
0
2000
4000
6000
8000
10000
Protein Size

Supplement 3 − Figure 19

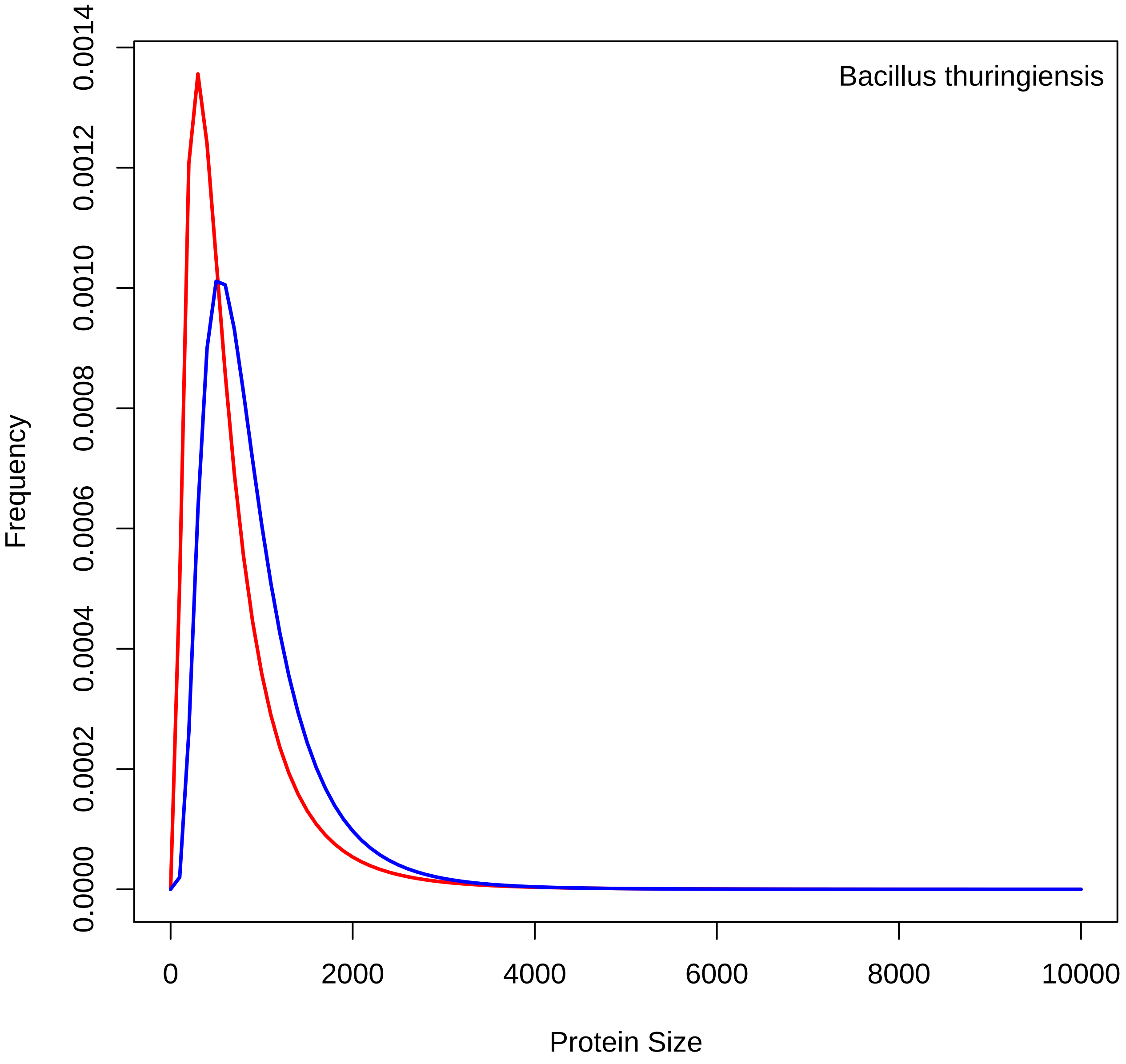

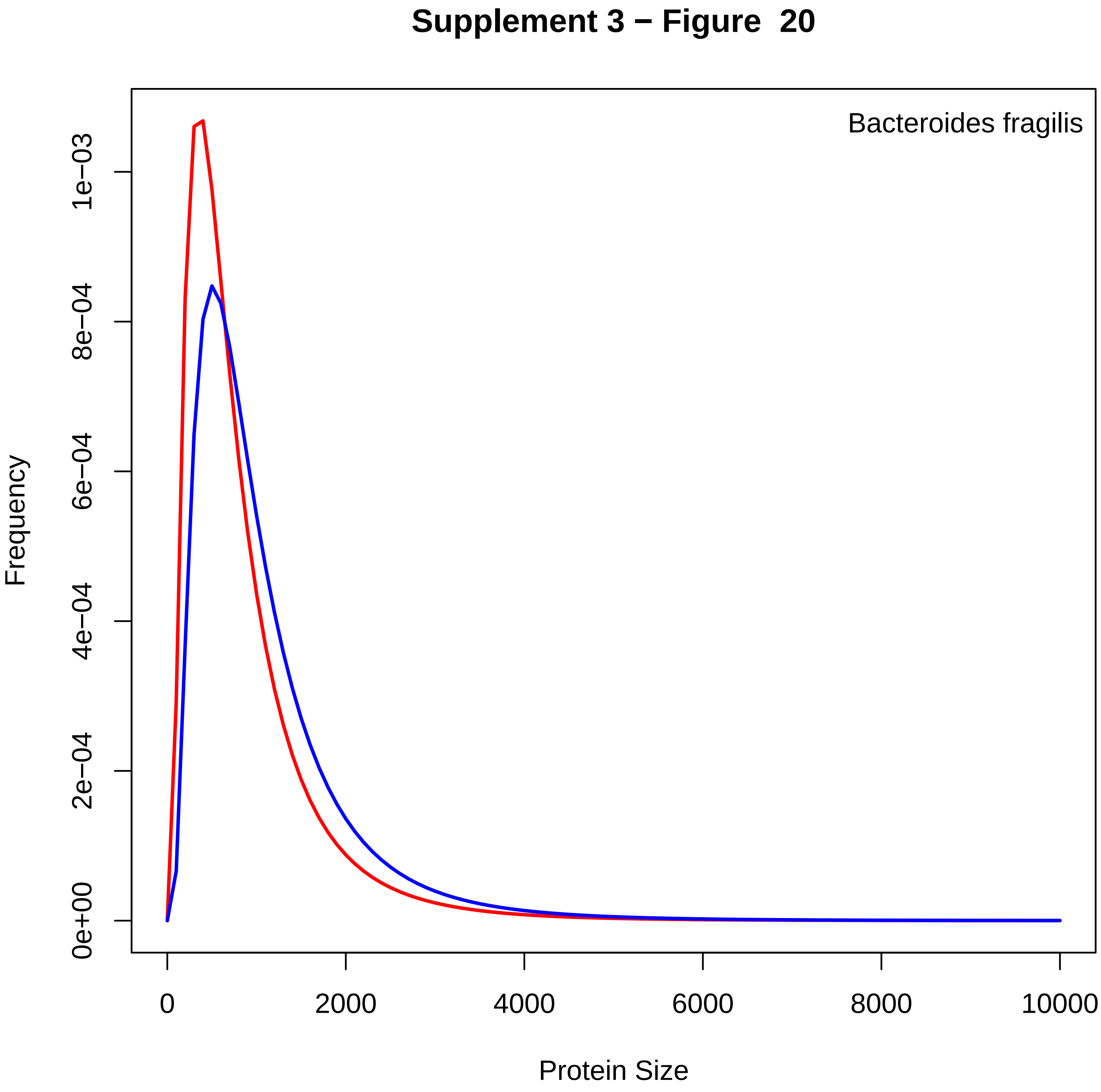
Supplement 3 − Figure 20
Bacteroides fragilis
Frequency
0e+00
2e−04
4e−04
6e−04
8e−04
1e−03
0
2000
4000
6000
8000
10000
Protein Size

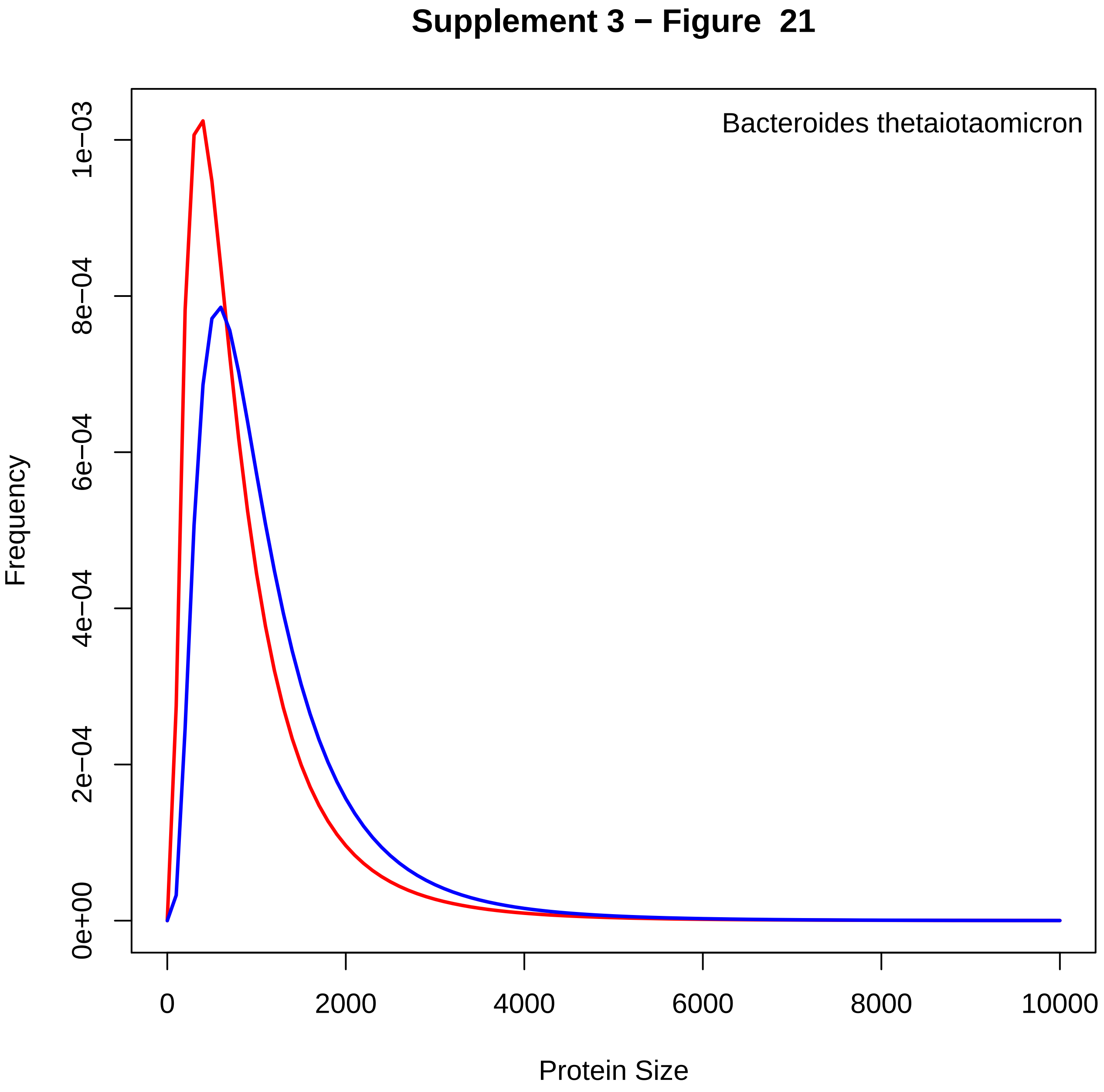
Supplement 3 − Figure 21
Bacteroides thetaiotaomicron
Frequency
0e+00
2e−04
4e−04
6e−04
8e−04
1e−03
0
2000
4000
6000
8000
10000
Protein Size

Supplement 3 − Figure 22

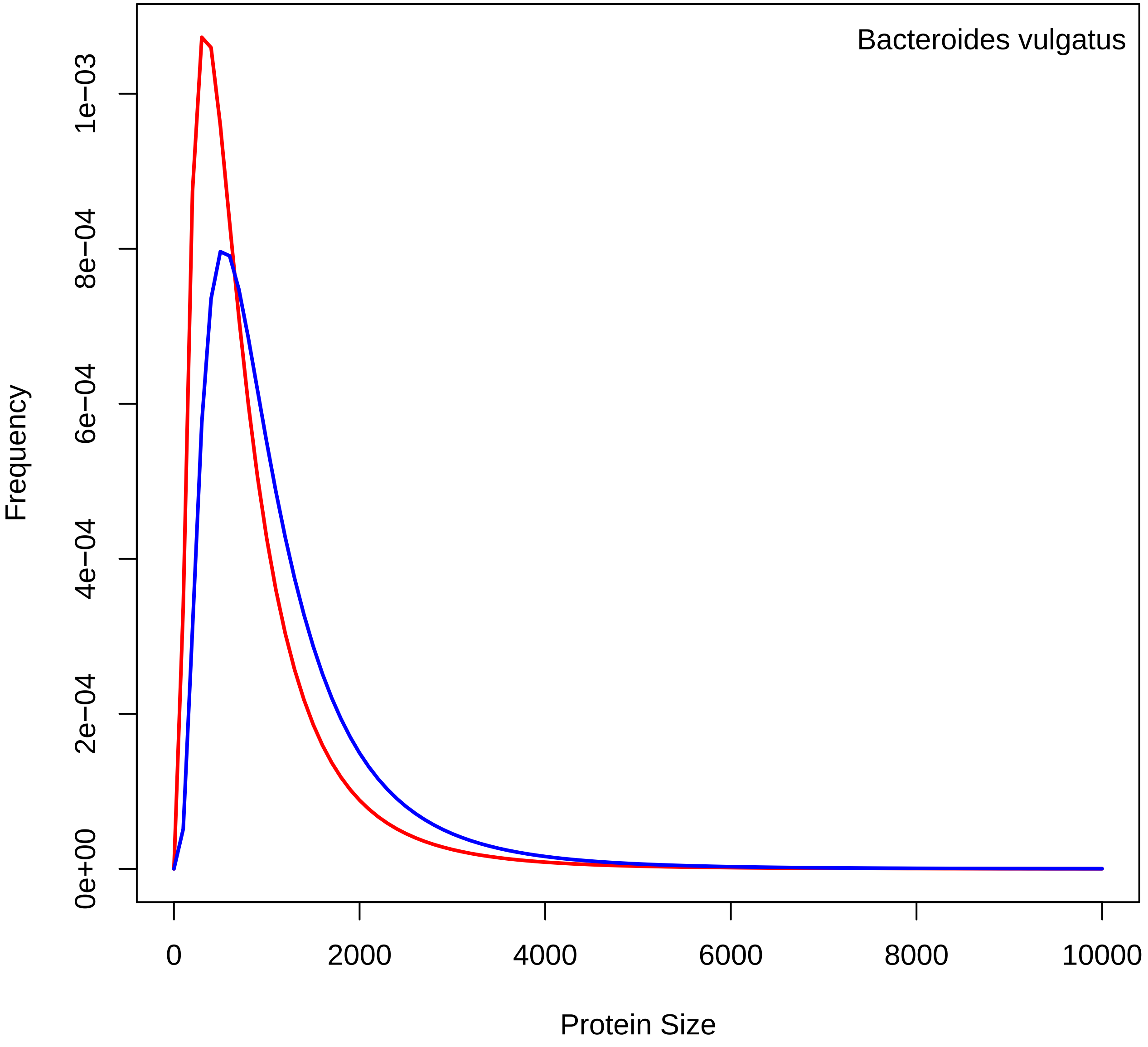

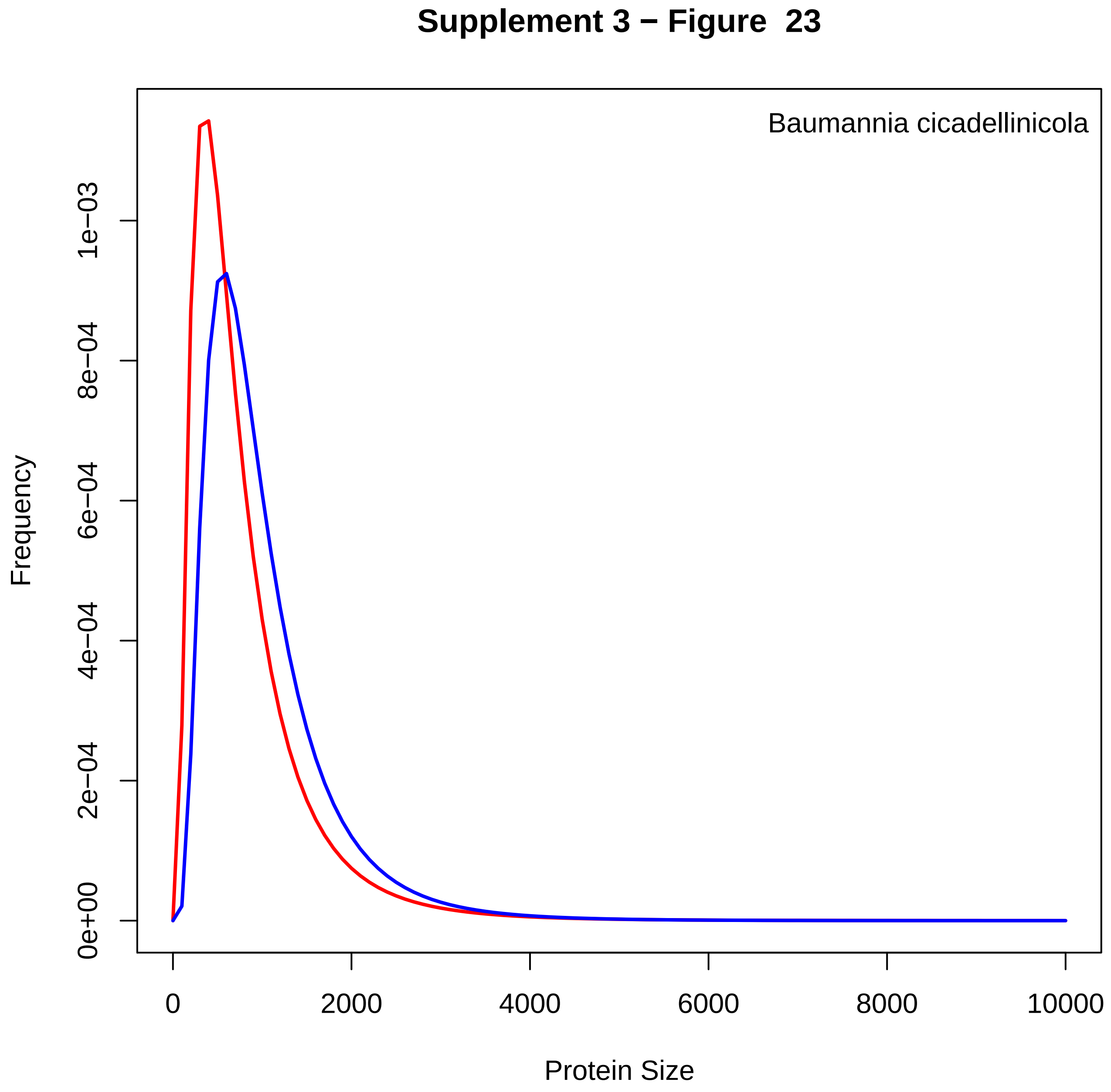
Supplement 3 − Figure 23
Baumannia cicadellinicola
Frequency
0e+00
2e−04
4e−04
6e−04
8e−04
1e−03
0
2000
4000
6000
8000
10000
Protein Size

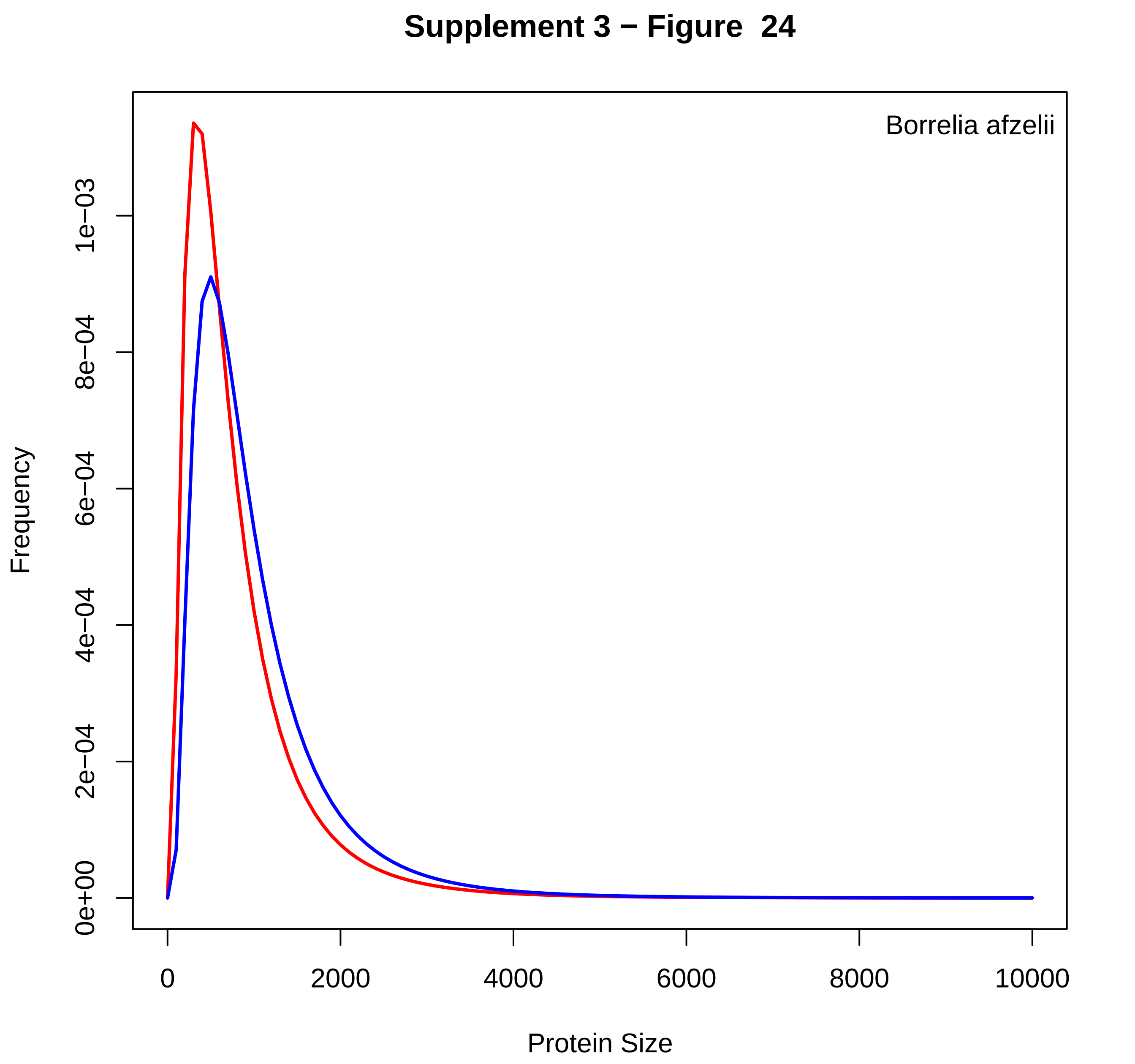
Supplement 3 − Figure 24
Borrelia afzelii
Frequency
0e+00
2e−04
4e−04
6e−04
8e−04
1e−03
0
2000
4000
6000
8000
10000
Protein Size

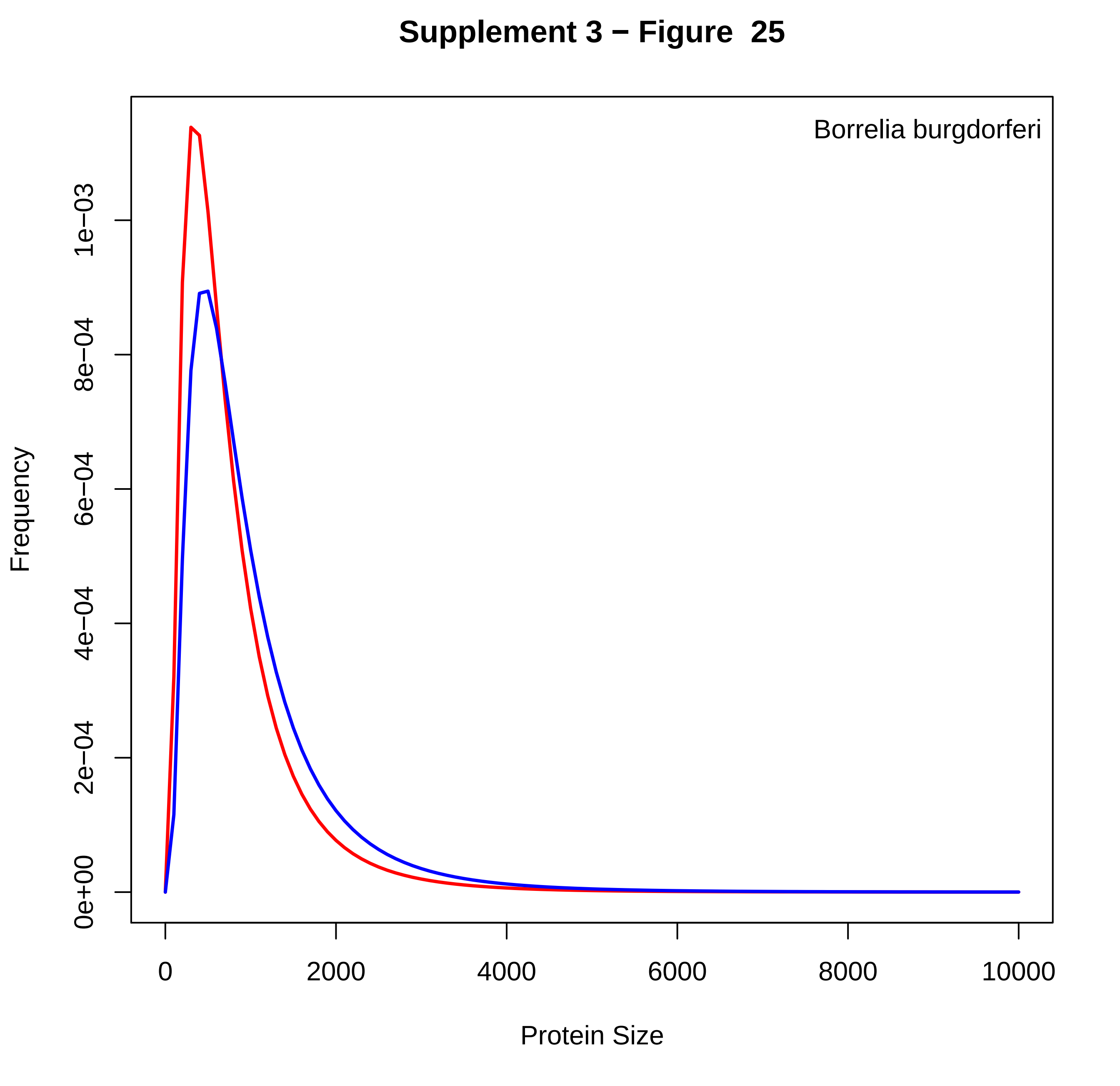
Supplement 3 − Figure 25
Borrelia burgdorferi
Frequency
Protein Size
0e+00
2e−04
4e−04
6e−04
8e−04
1e−03
0
2000
4000
6000
8000
10000

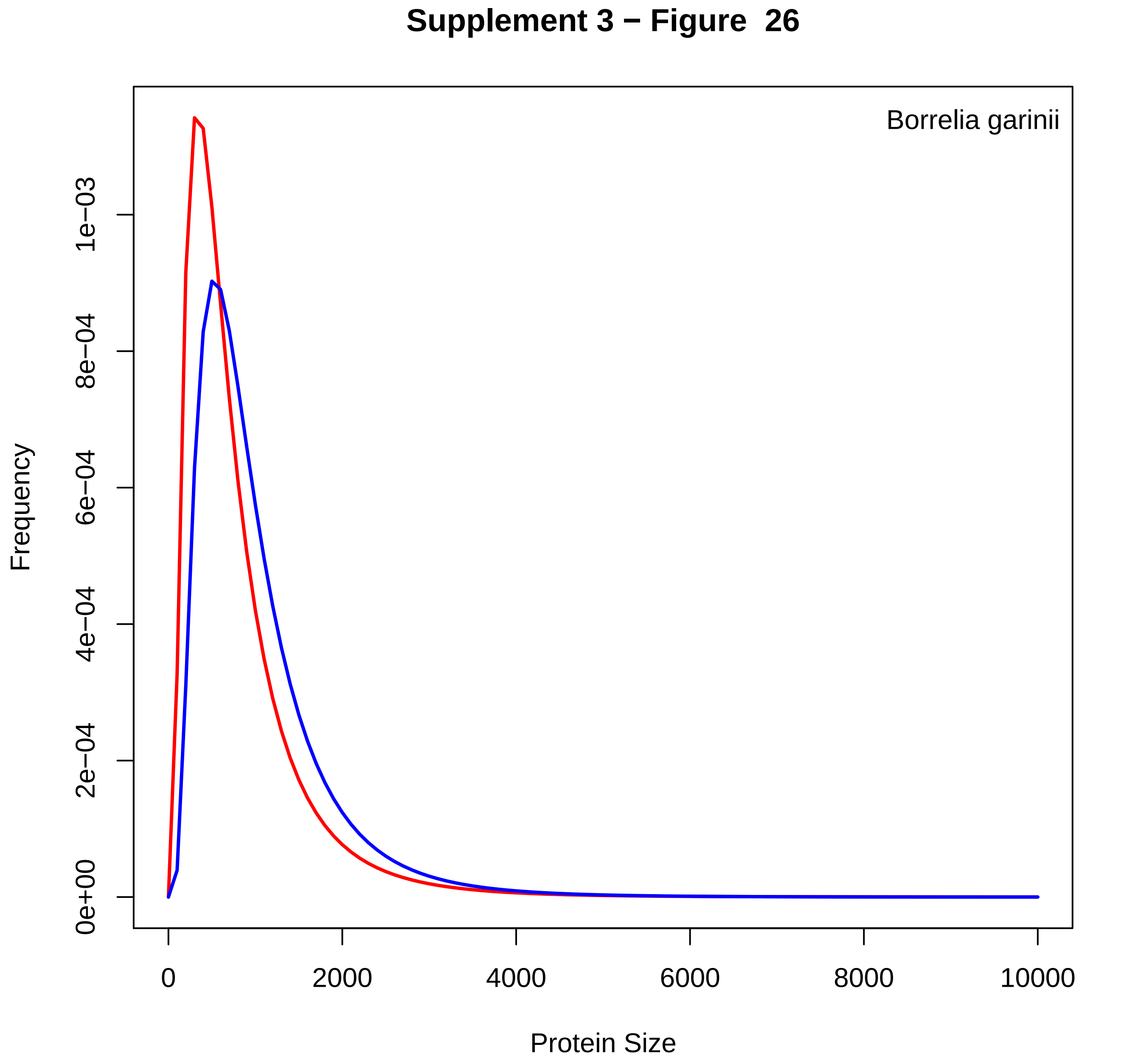

Supplement 3 − Figure 26
Borrelia garinii
Frequency
0e+00
2e−04
4e−04
6e−04
8e−04
1e−03
0
2000
4000
6000
8000
10000
Protein Size

Supplement 3 − Figure 27

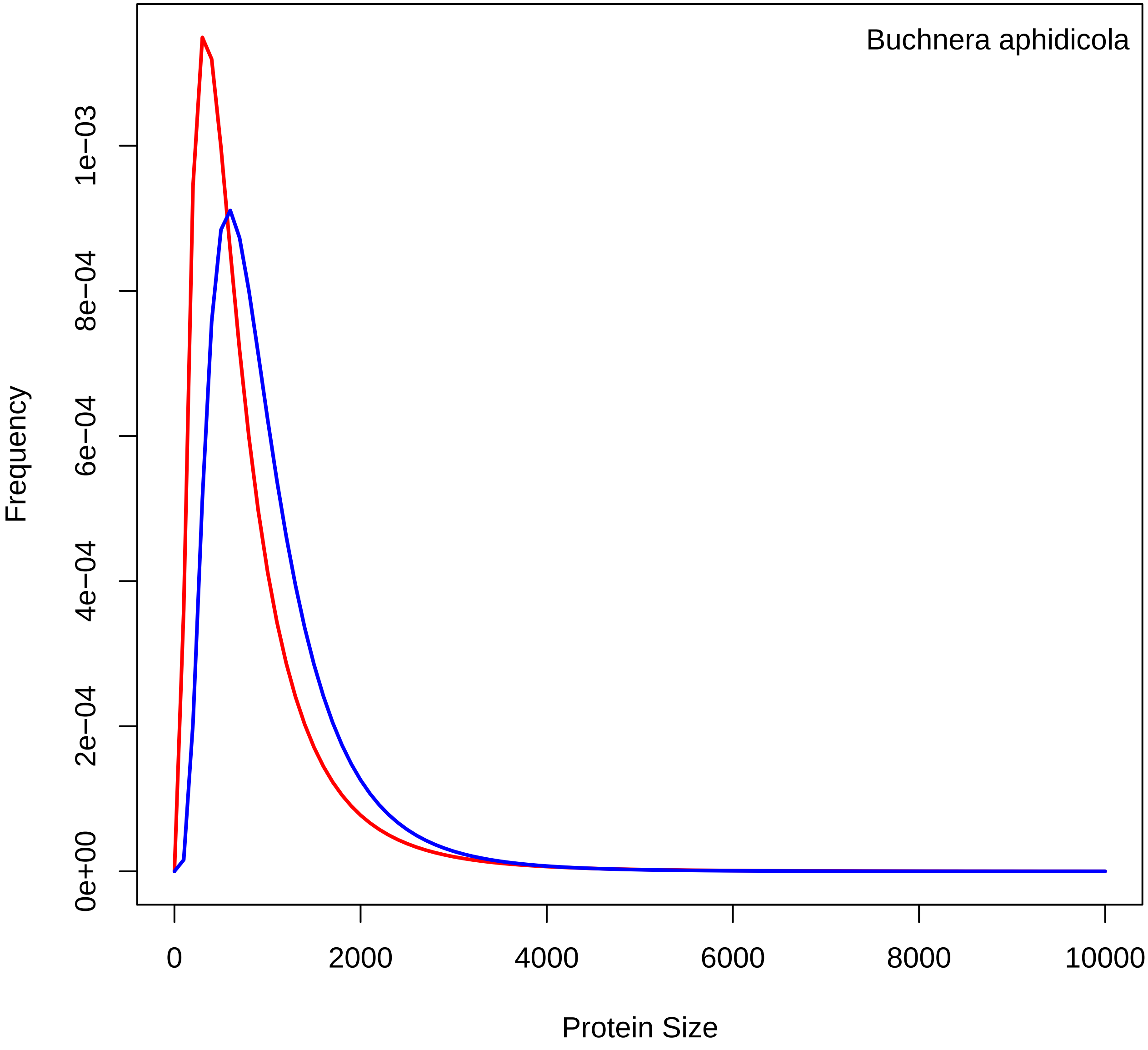

Supplement 3 − Figure 28

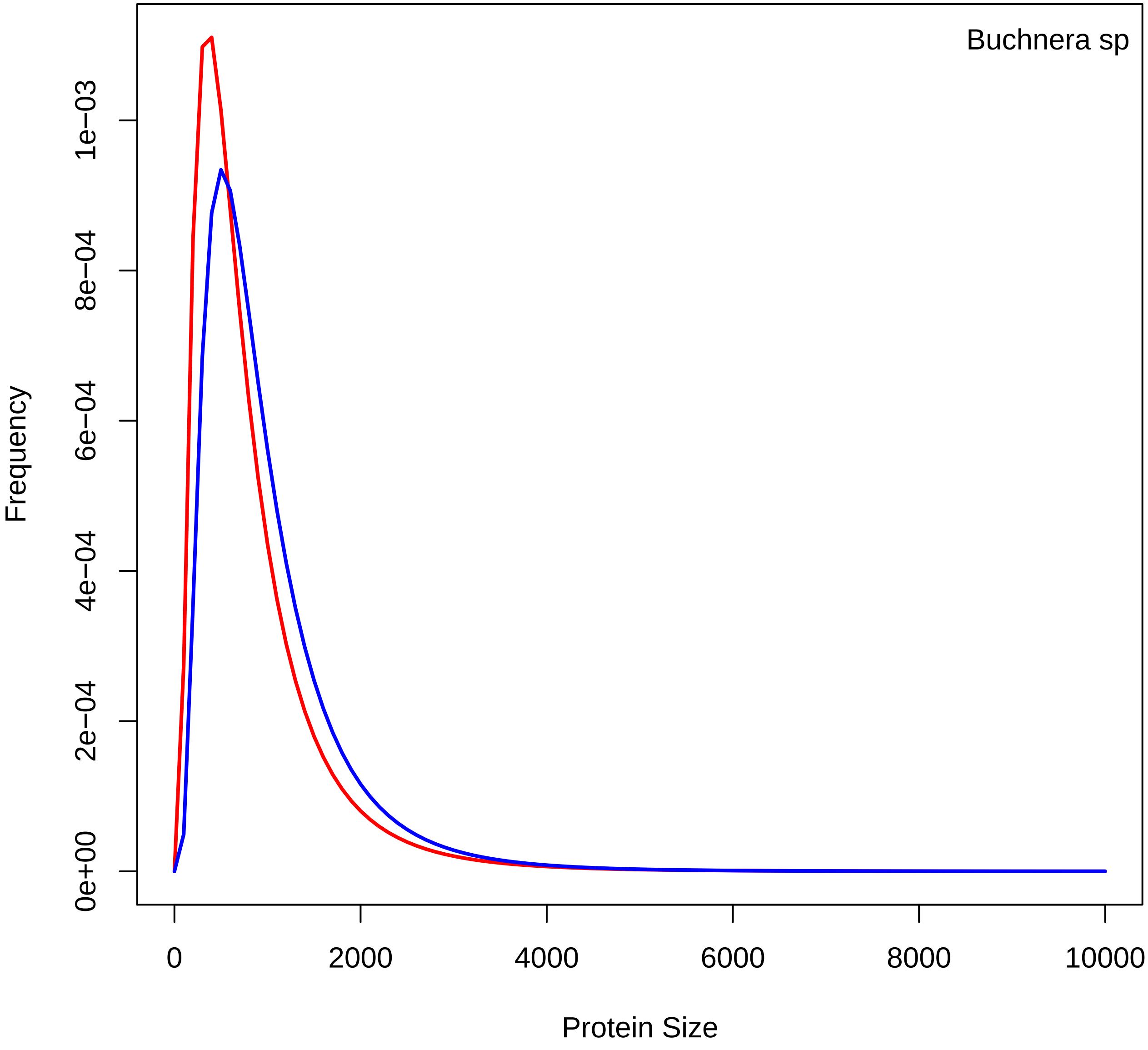

Supplement 3 − Figure 29

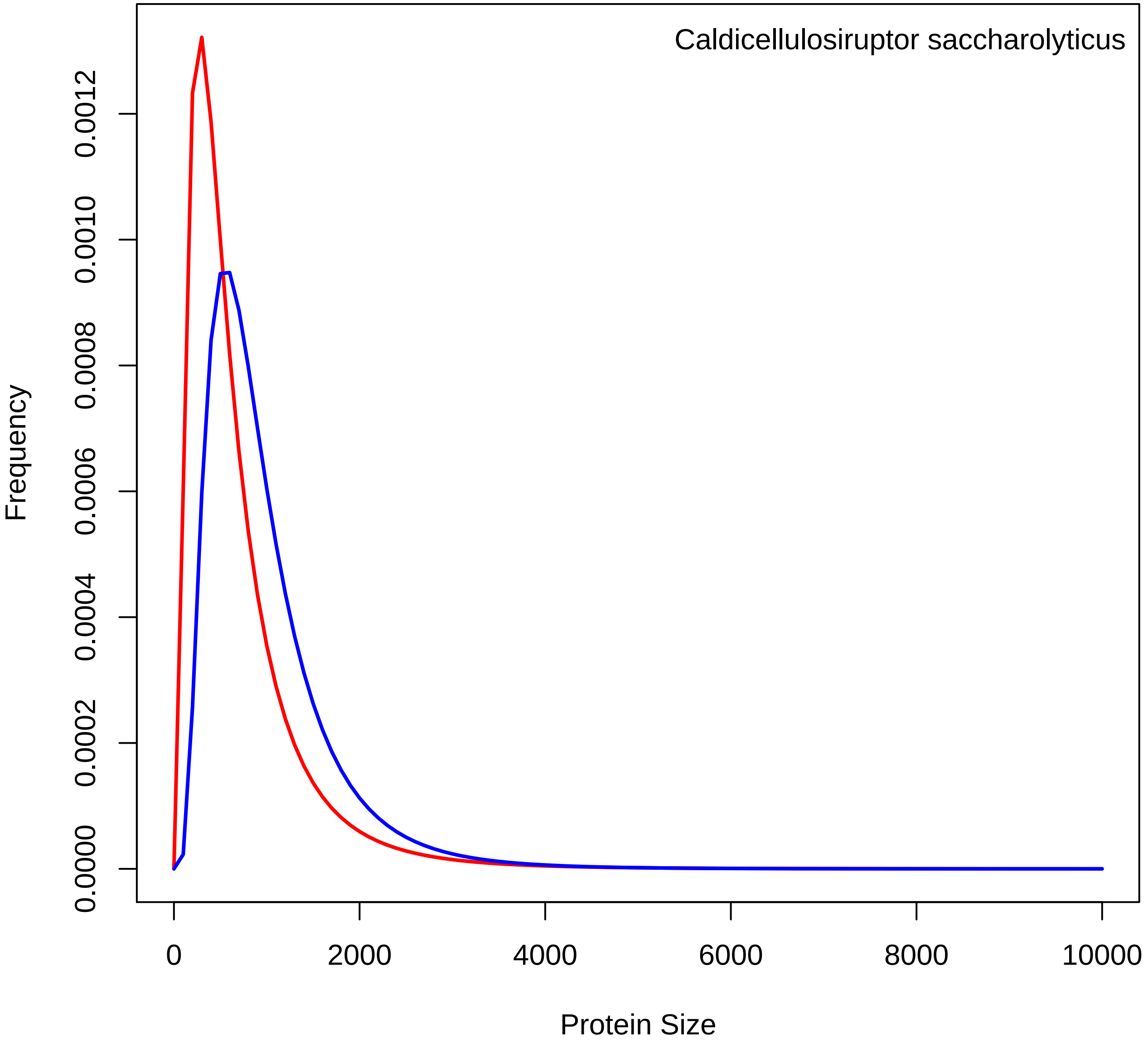

Supplement 3 − Figure 30

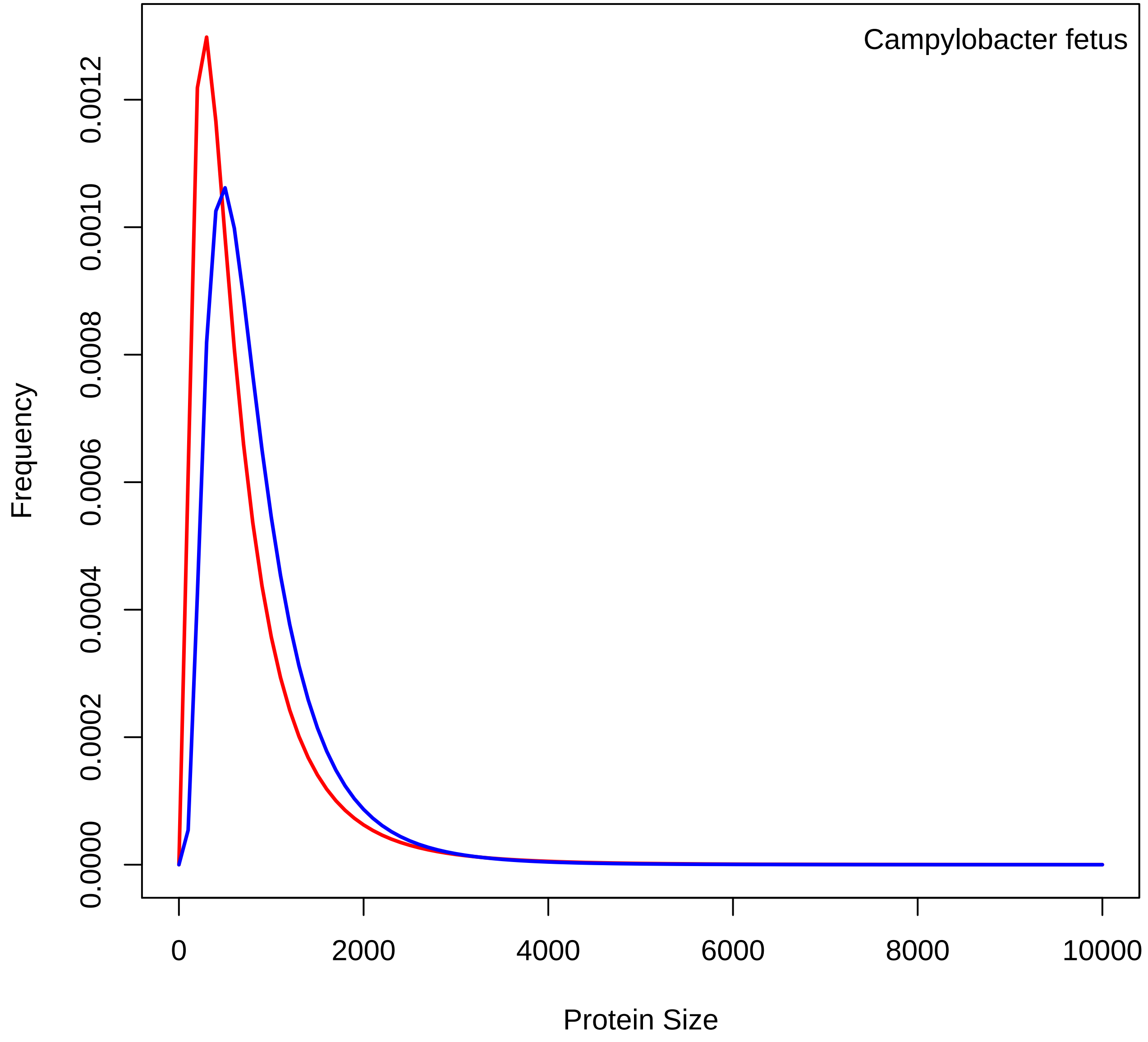

Supplement 3 − Figure 31

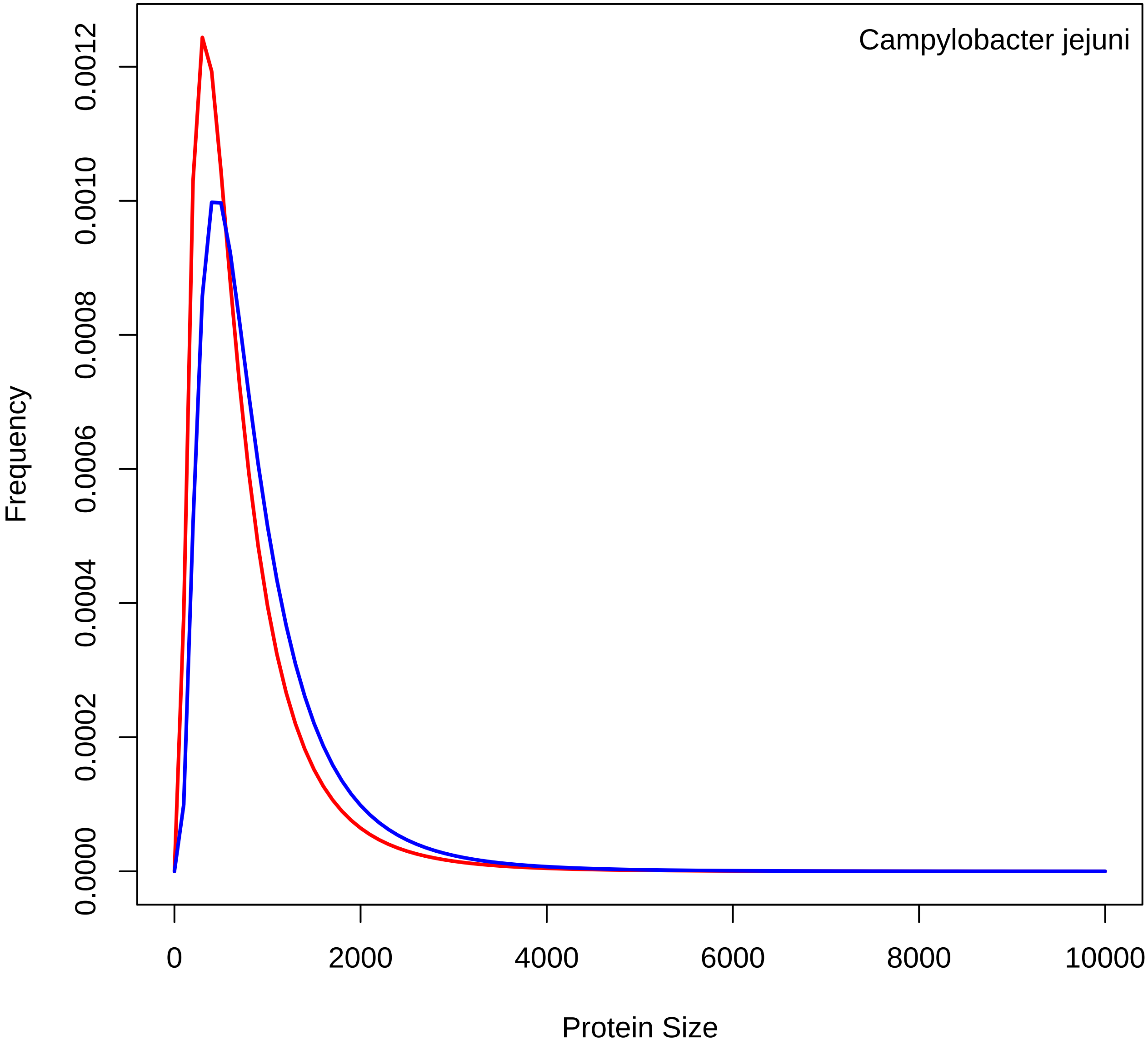

Supplement 3 − Figure 32

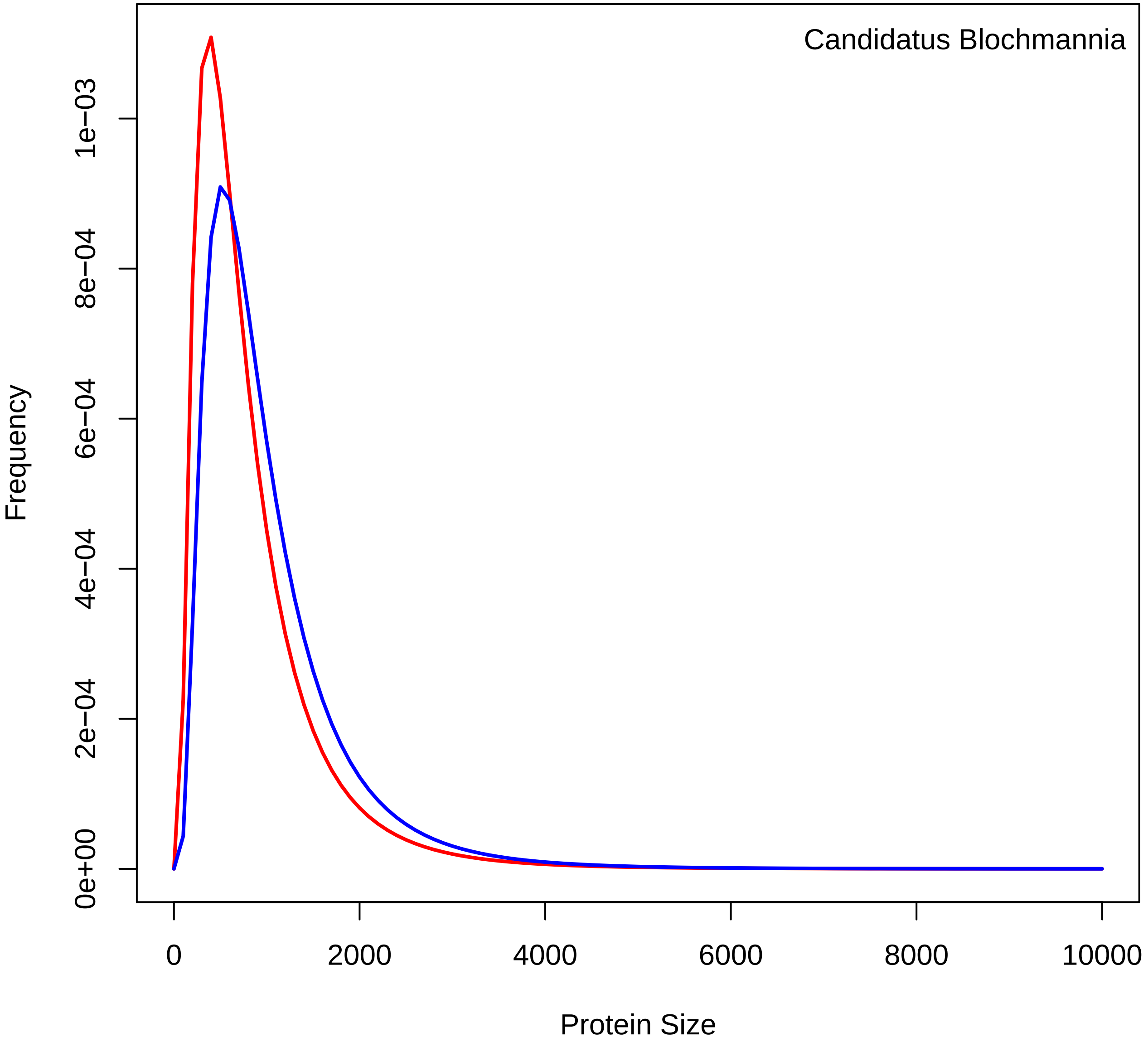

Supplement 3 − Figure 33

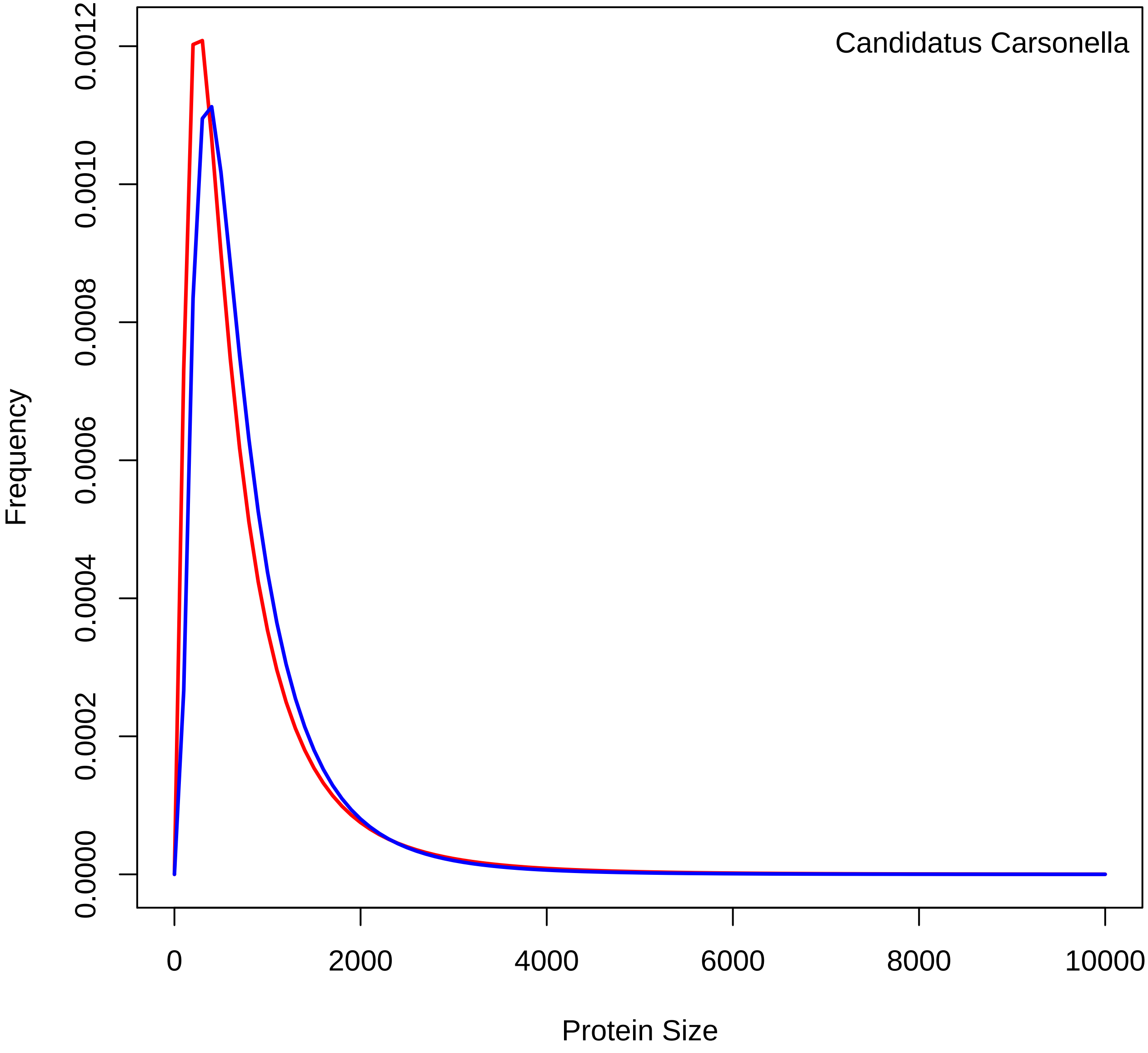

Supplement 3 − Figure 34

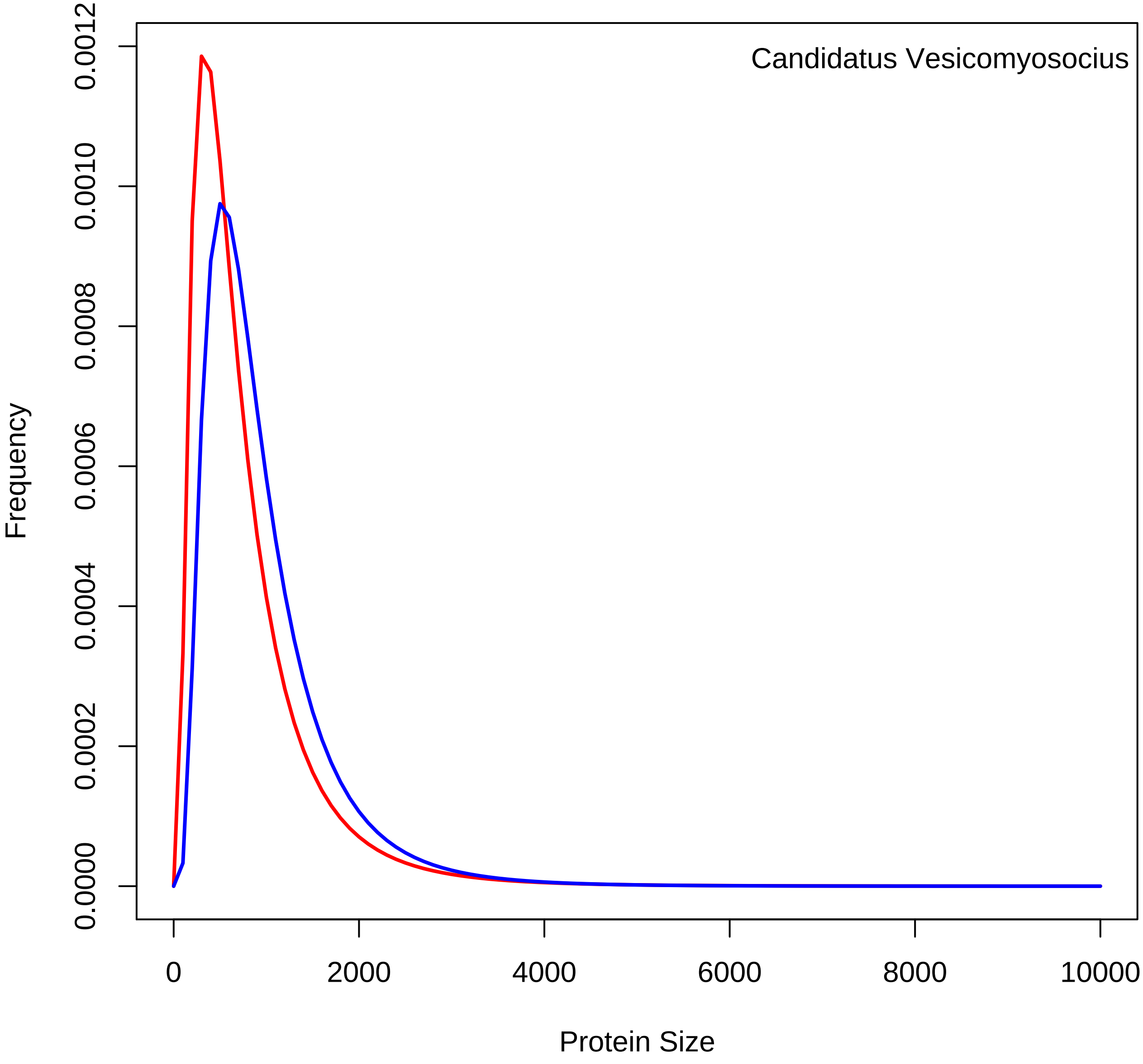

Supplement 3 − Figure 35

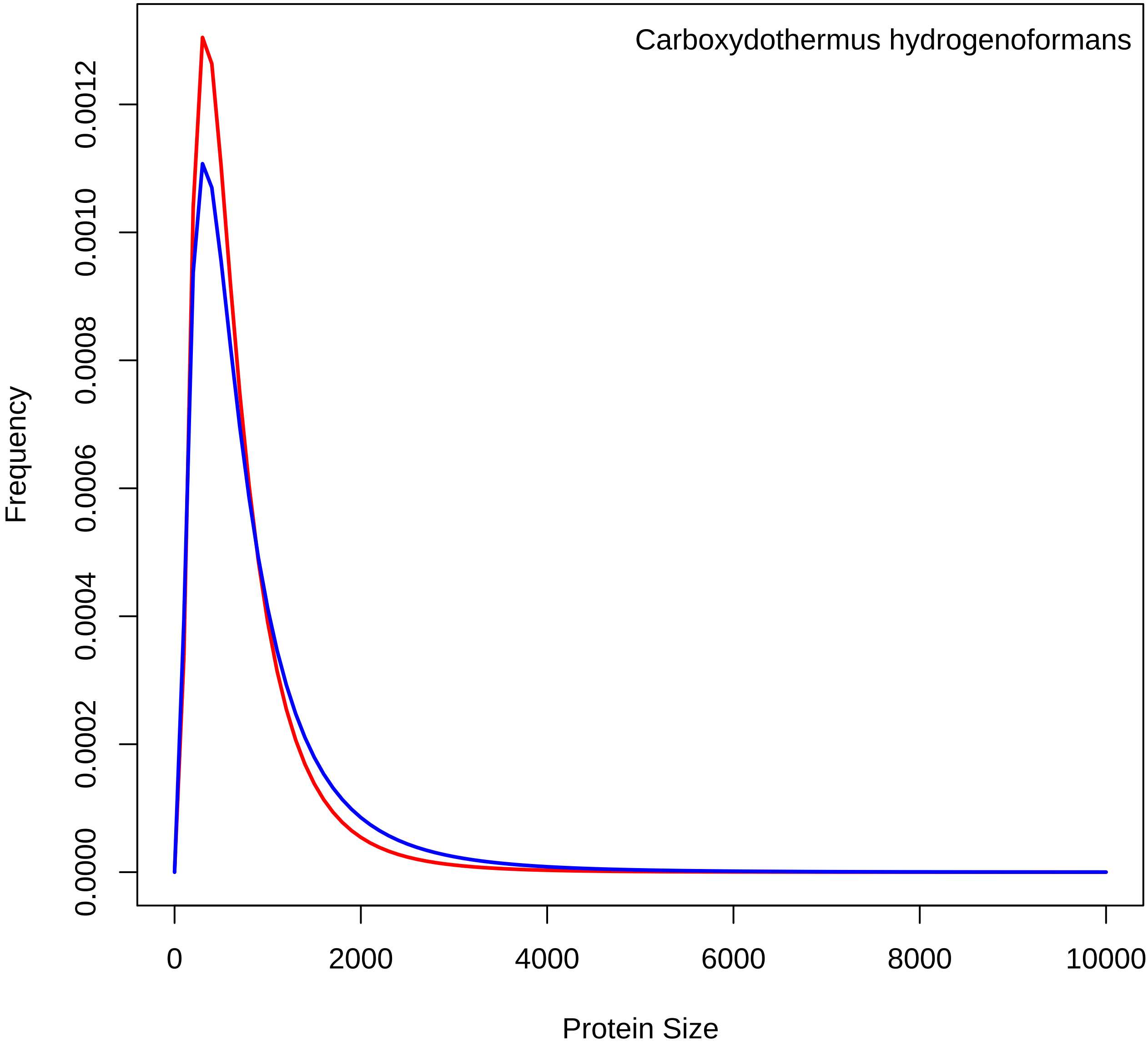

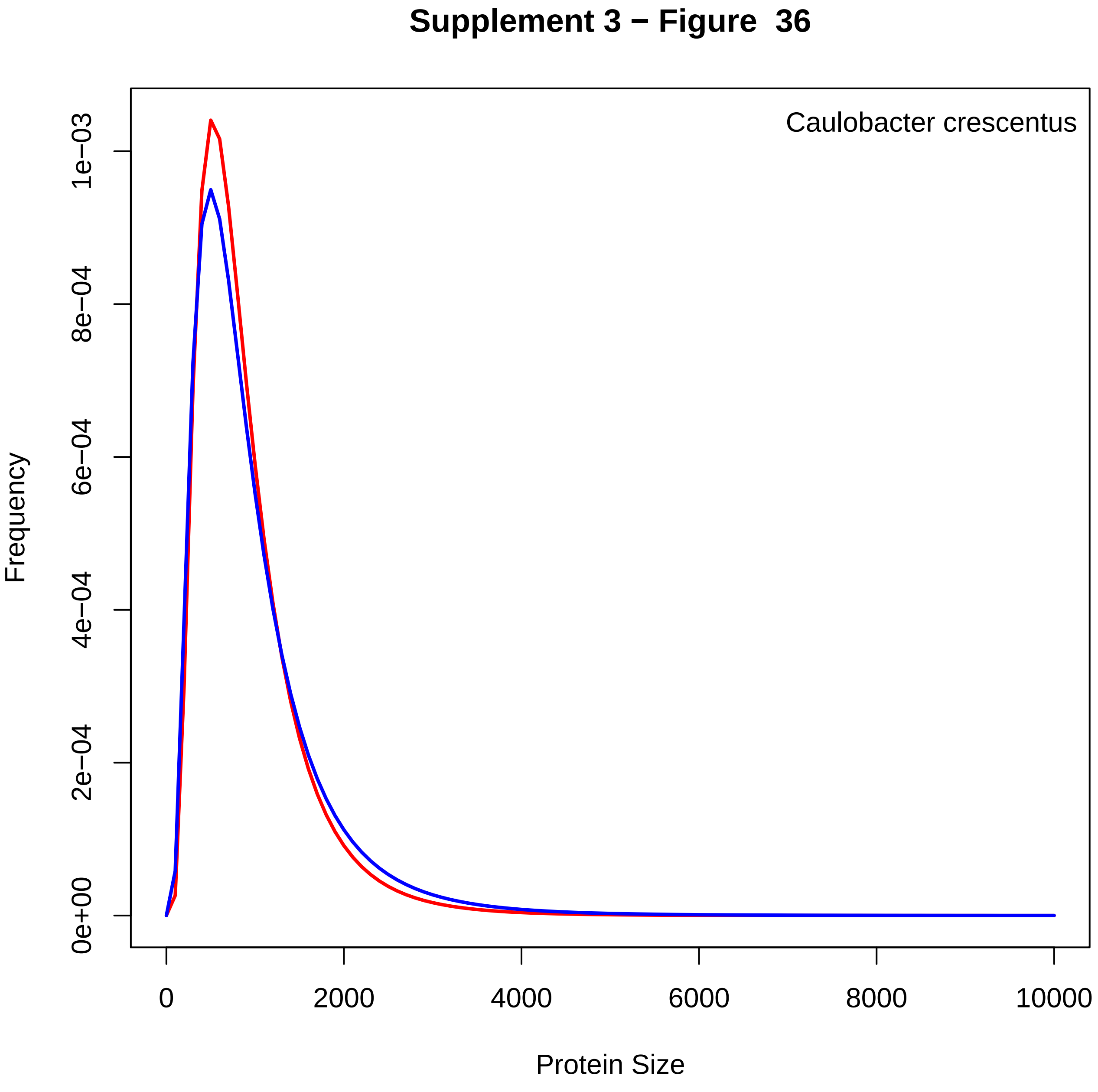
Supplement 3 − Figure 36
Caulobacter crescentus
Frequency
0e+00
2e−04
4e−04
6e−04
8e−04
1e−03
0
2000
4000
6000
8000
10000
Protein Size

Supplement 3 − Figure 37

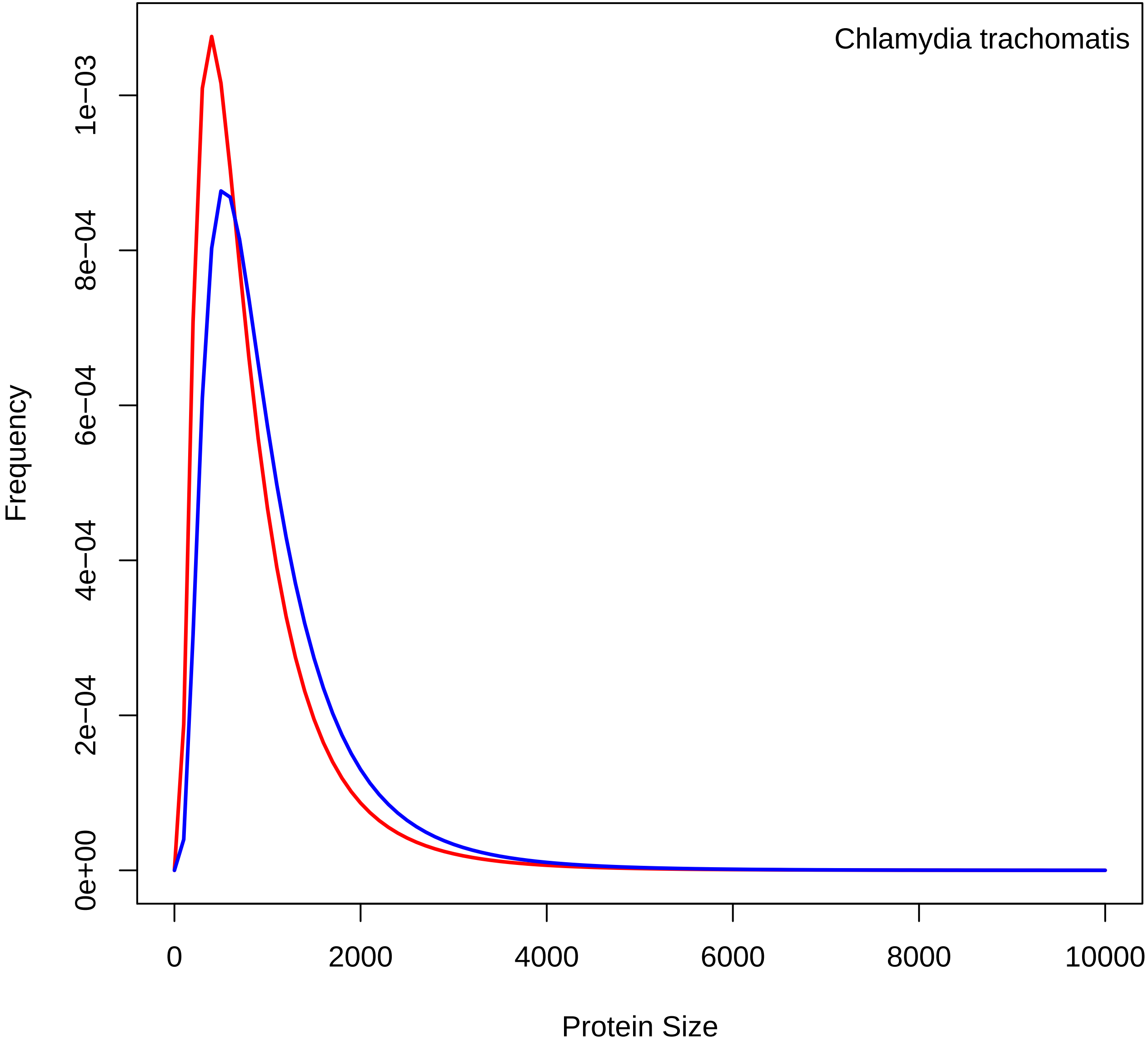

Supplement 3 − Figure 38

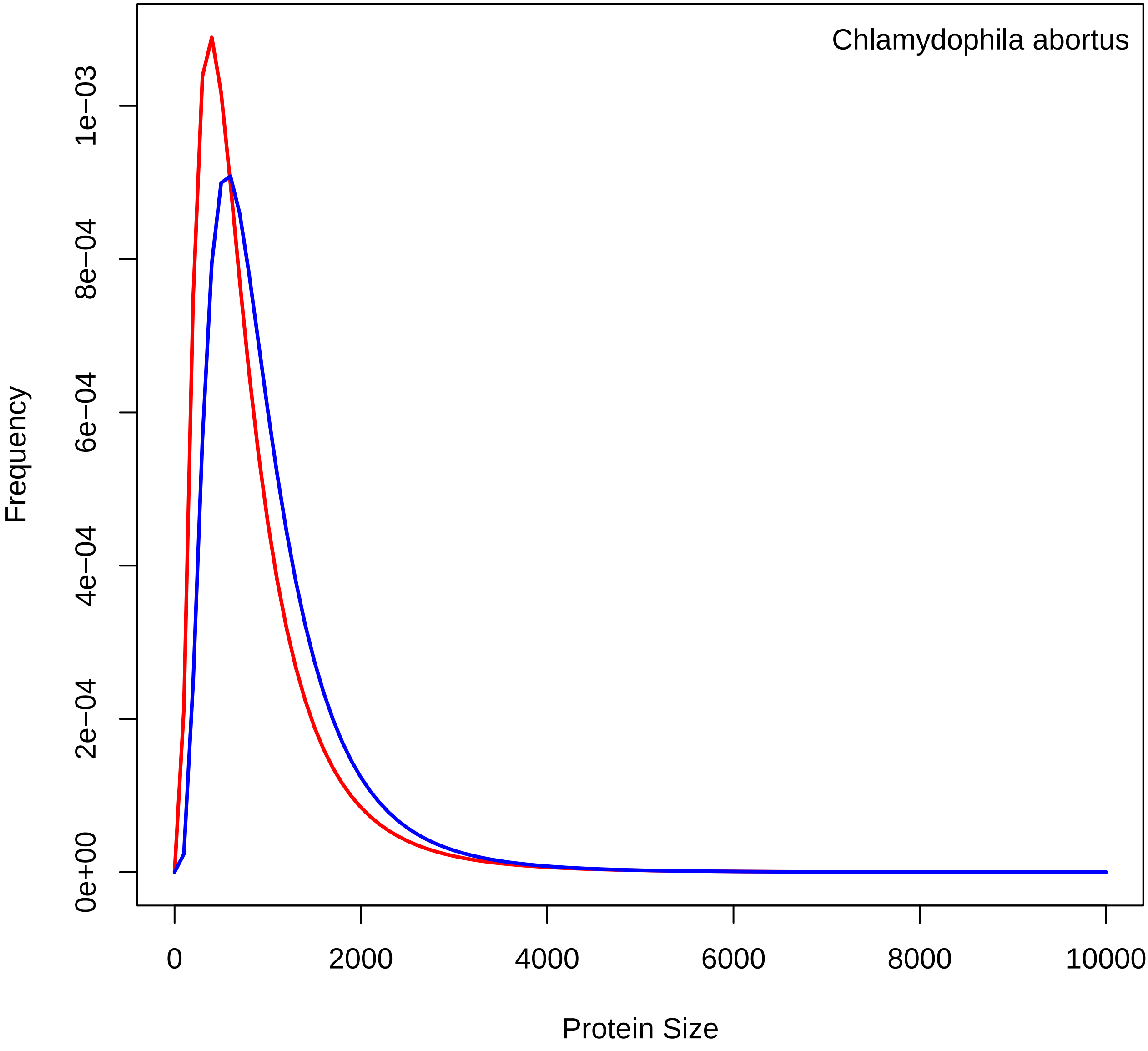

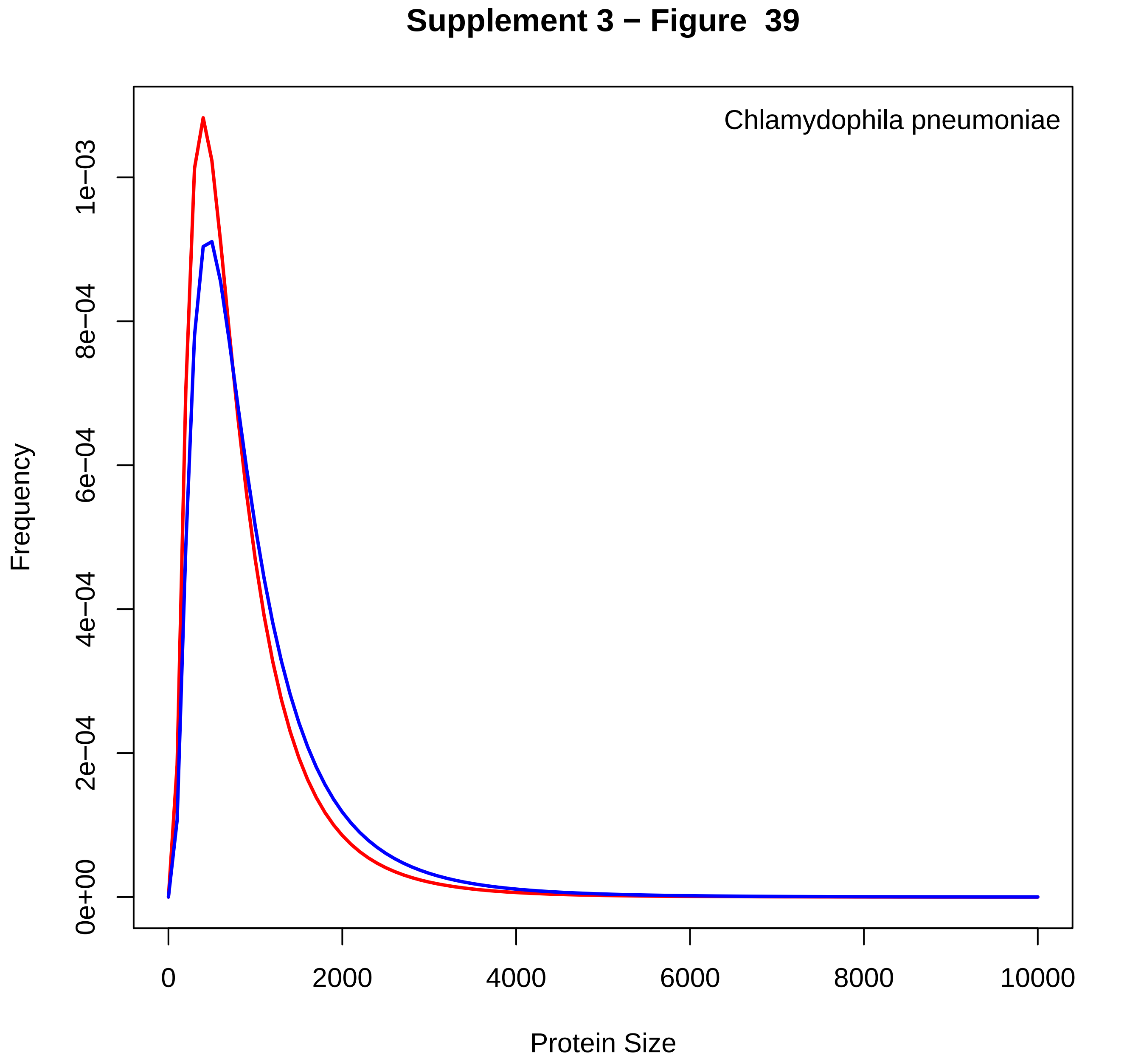

Supplement 3 − Figure 39
Chlamydophila pneumoniae
Frequency
0e+00
2e−04
4e−04
6e−04
8e−04
1e−03
0
2000
4000
6000
8000
10000
Protein Size

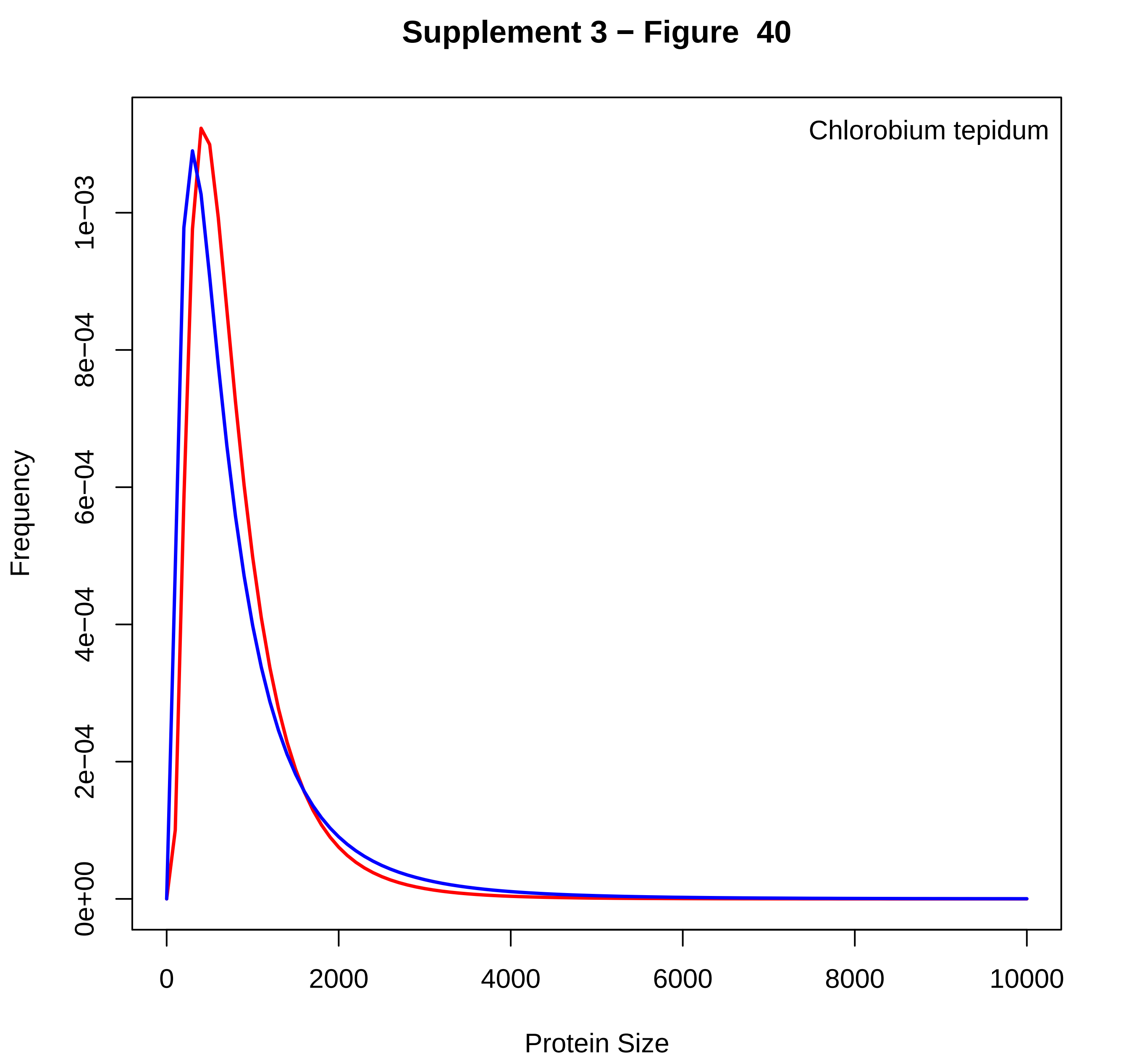

Supplement 3 − Figure 40
Chlorobium tepidum
Frequency
0e+00
2e−04
4e−04
6e−04
8e−04
1e−03
0
2000
4000
6000
8000
10000
Protein Size

Supplement 3 − Figure 41

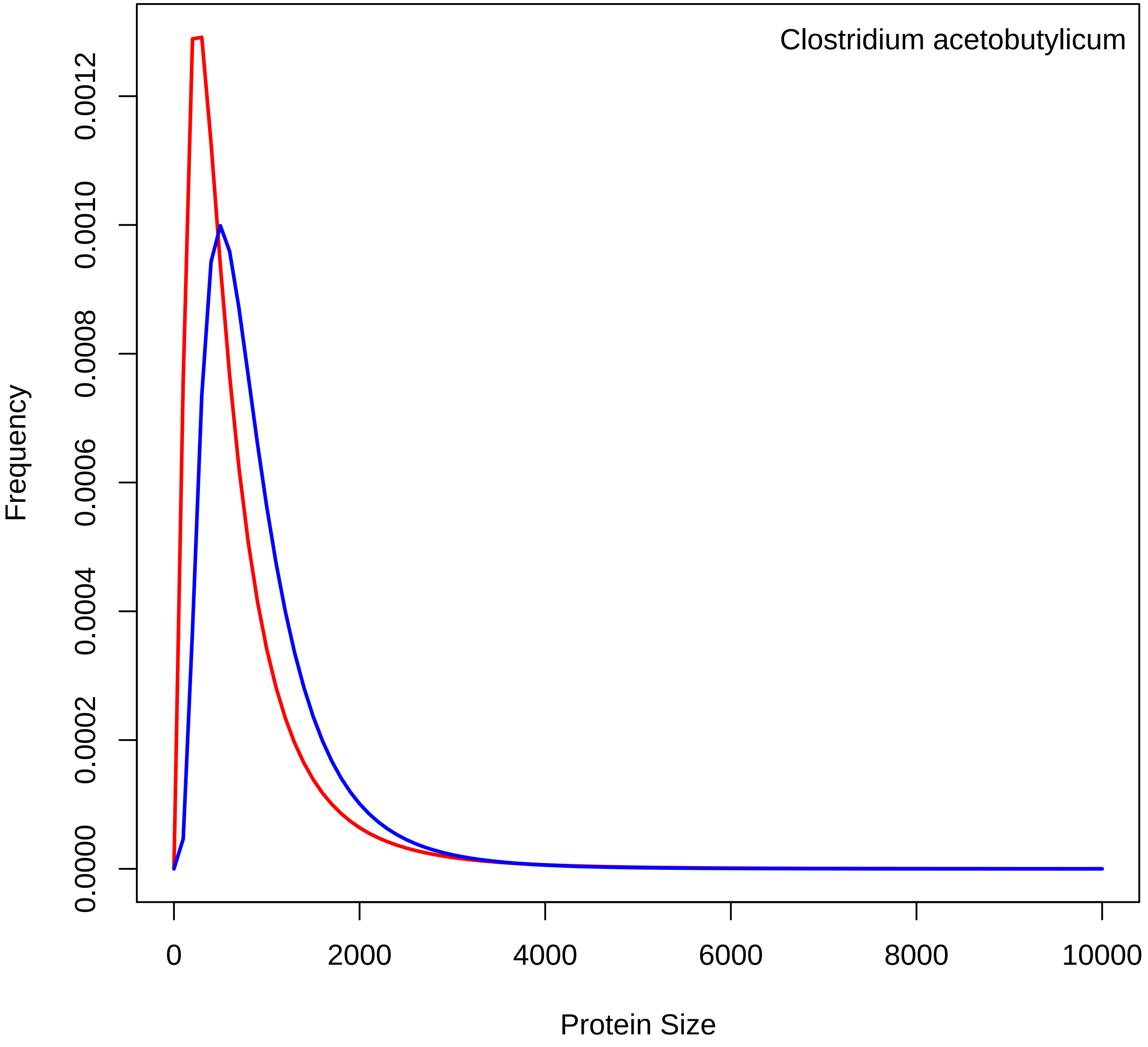

Supplement 3 − Figure 42

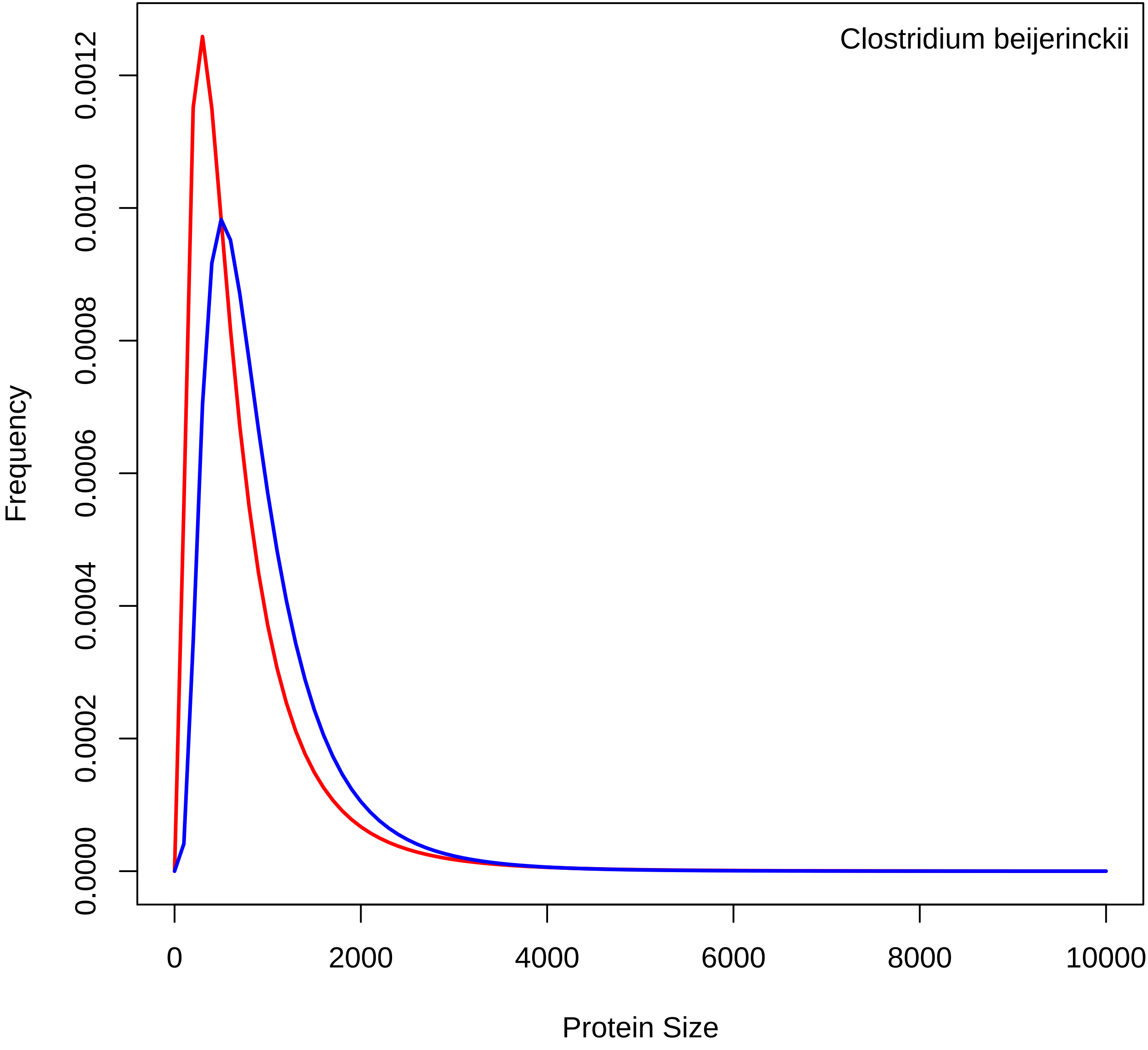

Supplement 3 − Figure 43

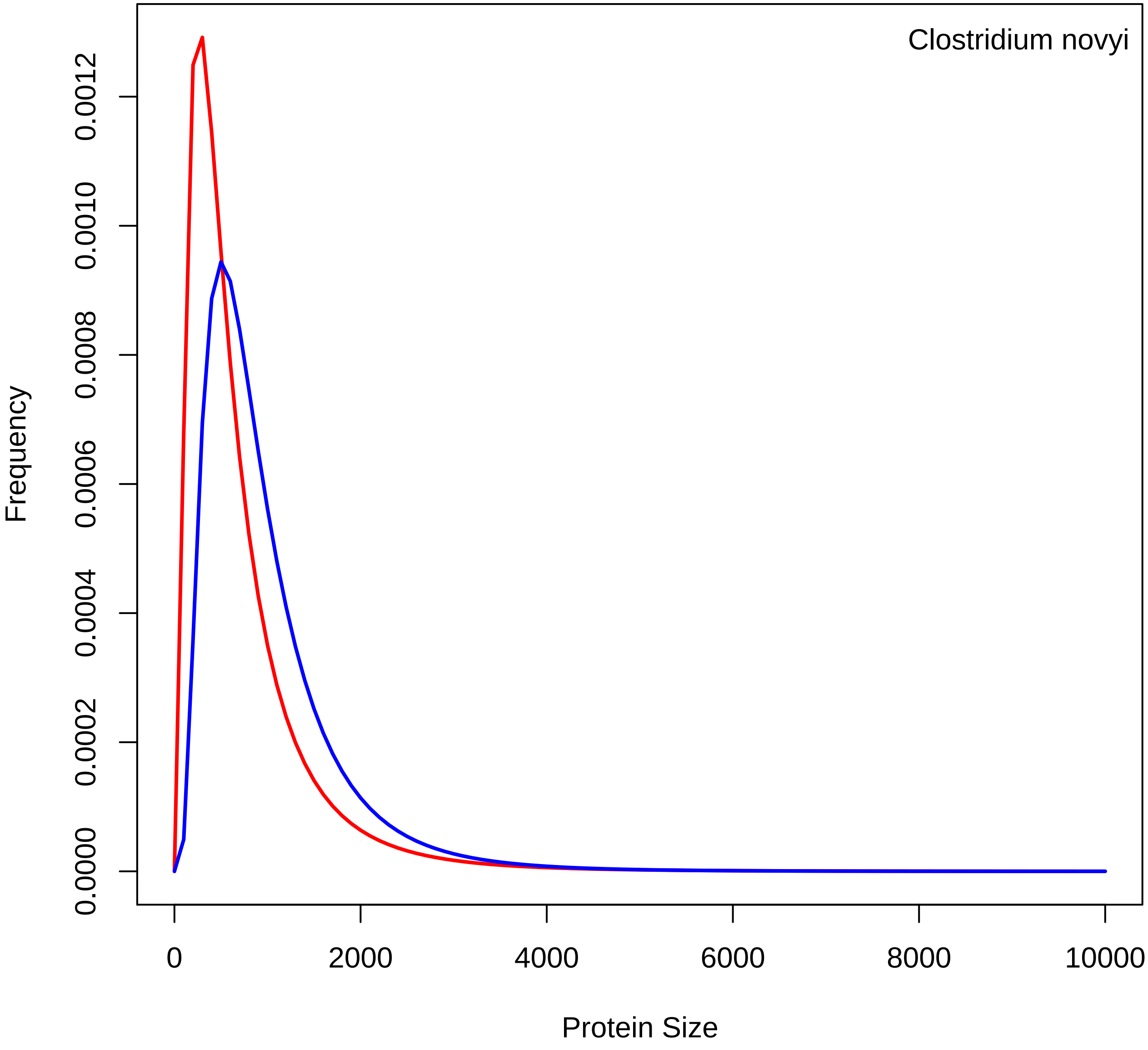

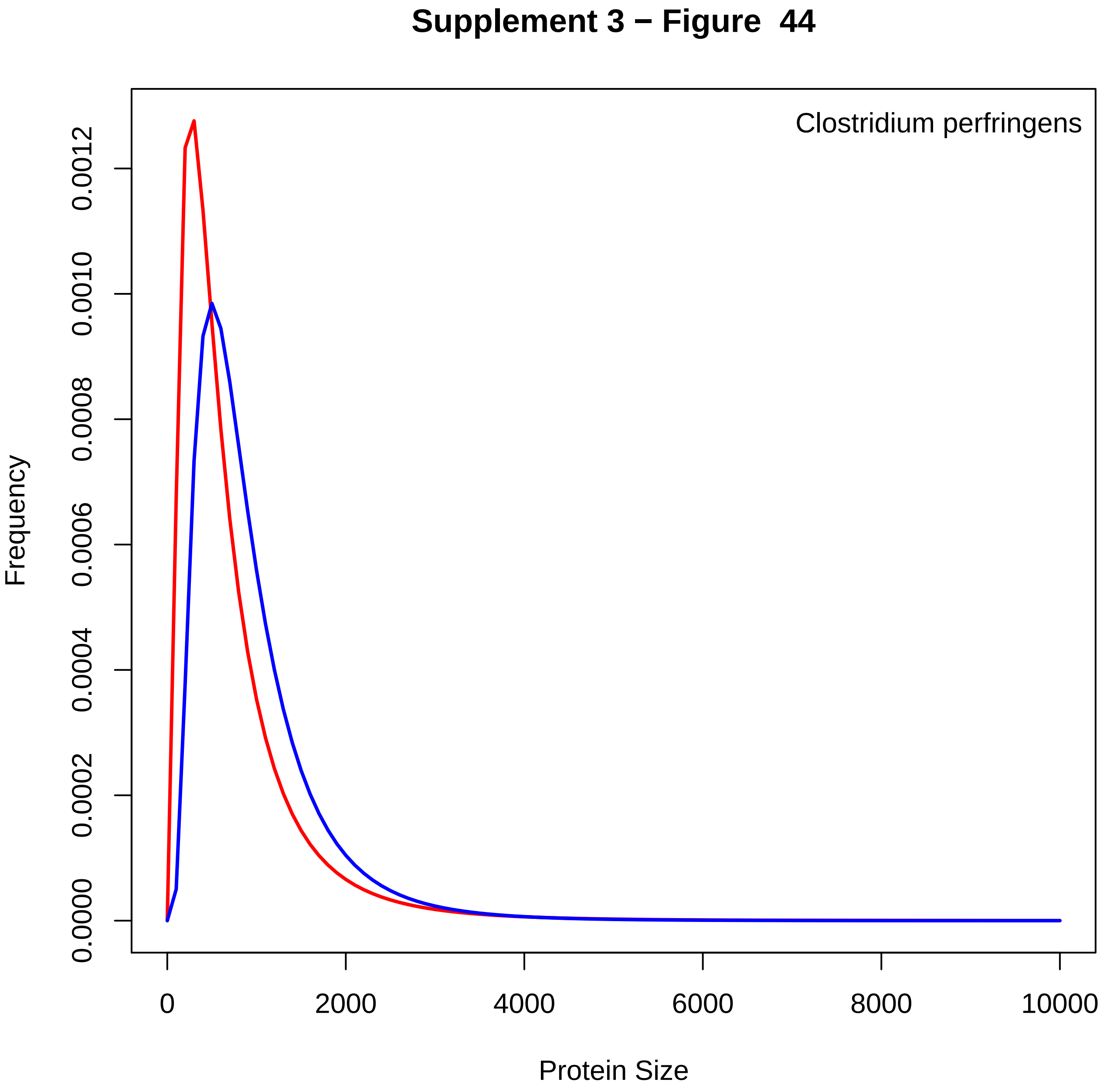
Supplement 3 − Figure 44
Clostridium perfringens
Frequency
Protein Size
0.0000
0.0002
0.0004
0.0006
0.0008
0.0010
0.0012
0
2000
4000
6000
8000
10000

Supplement 3 − Figure 45

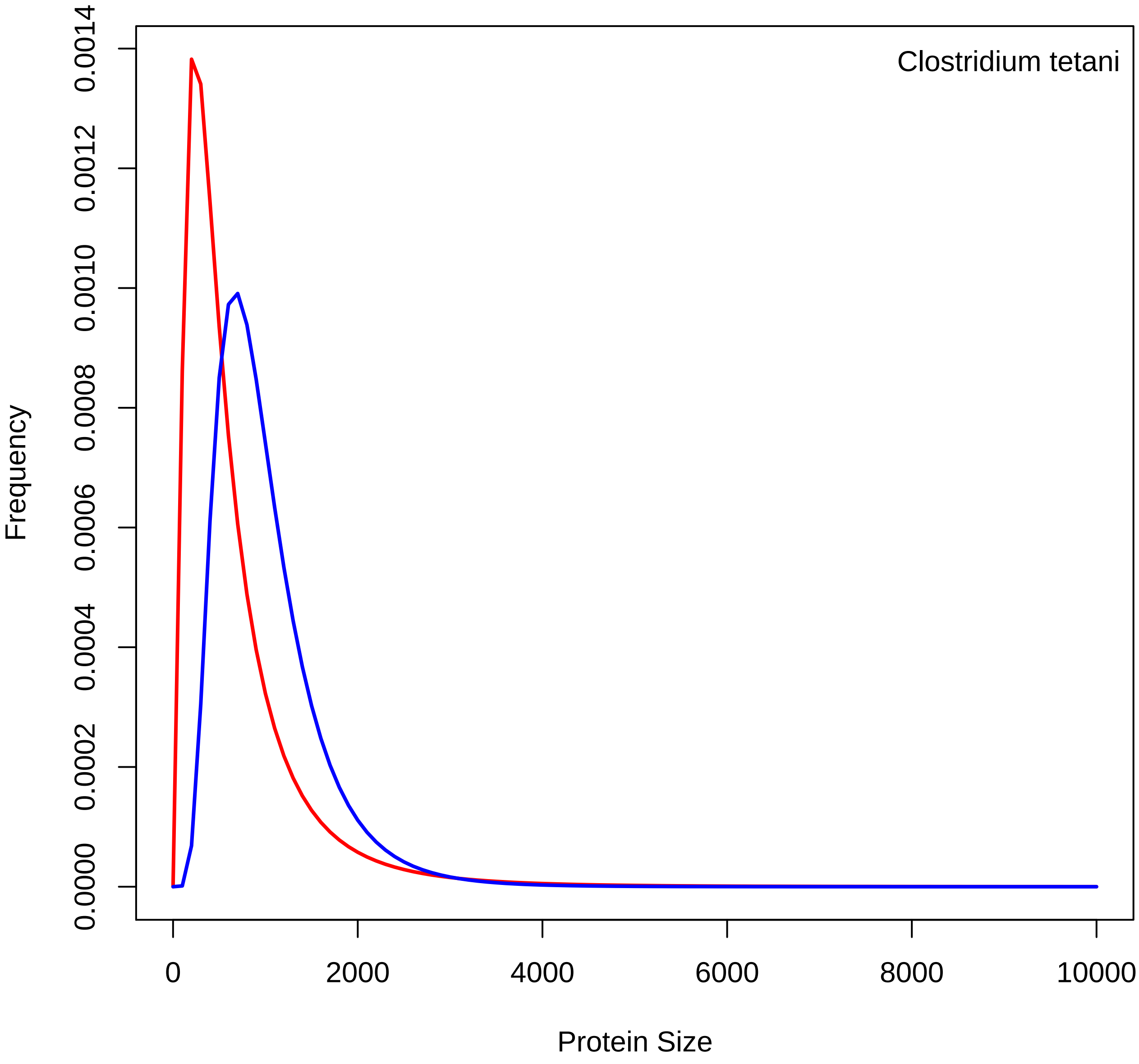

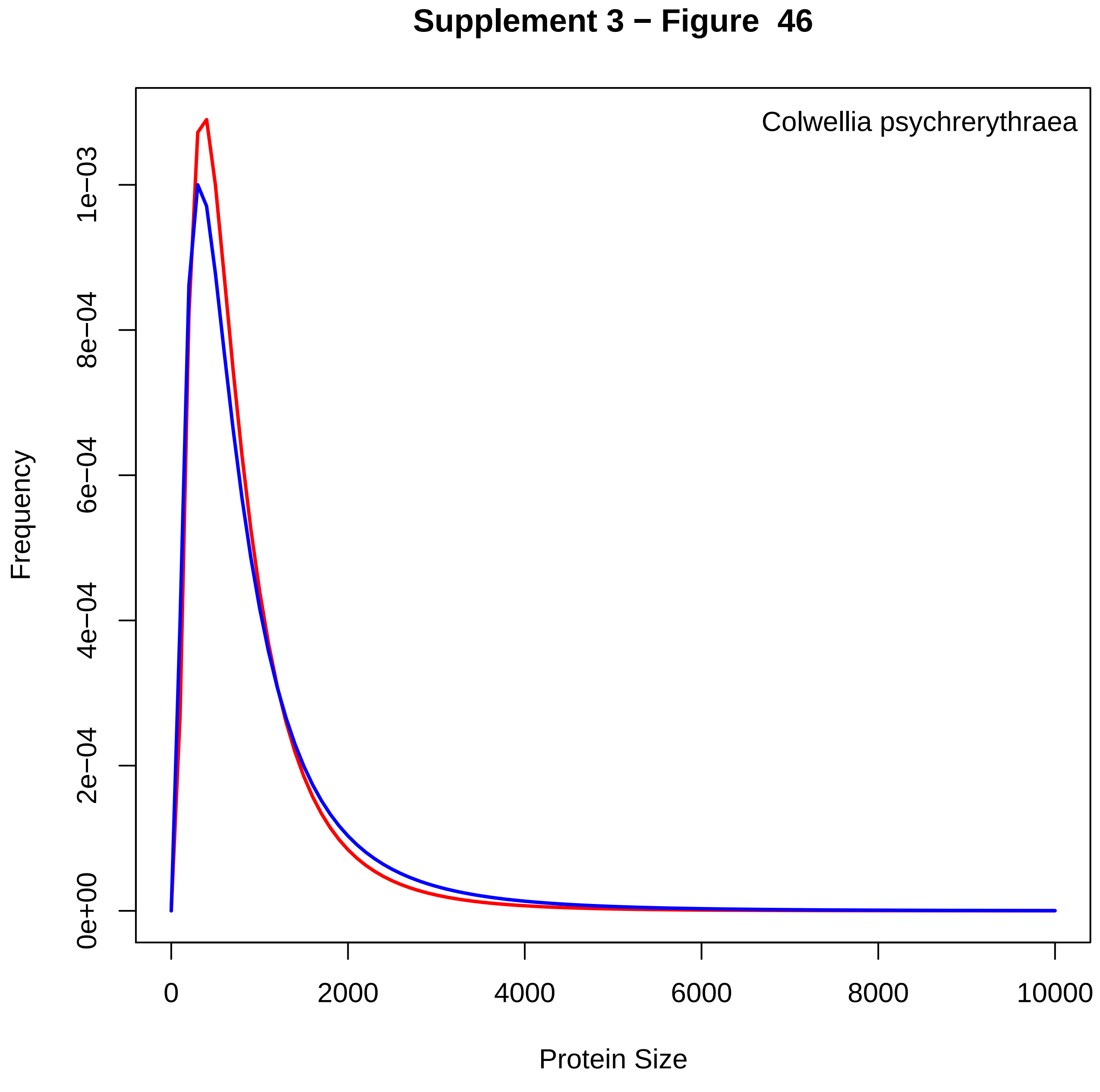
Supplement 3 − Figure 46
Colwellia psychrerythraea
Frequency
0e+00
2e−04
4e−04
6e−04
8e−04
1e−03
0
2000
4000
6000
8000
10000
Protein Size

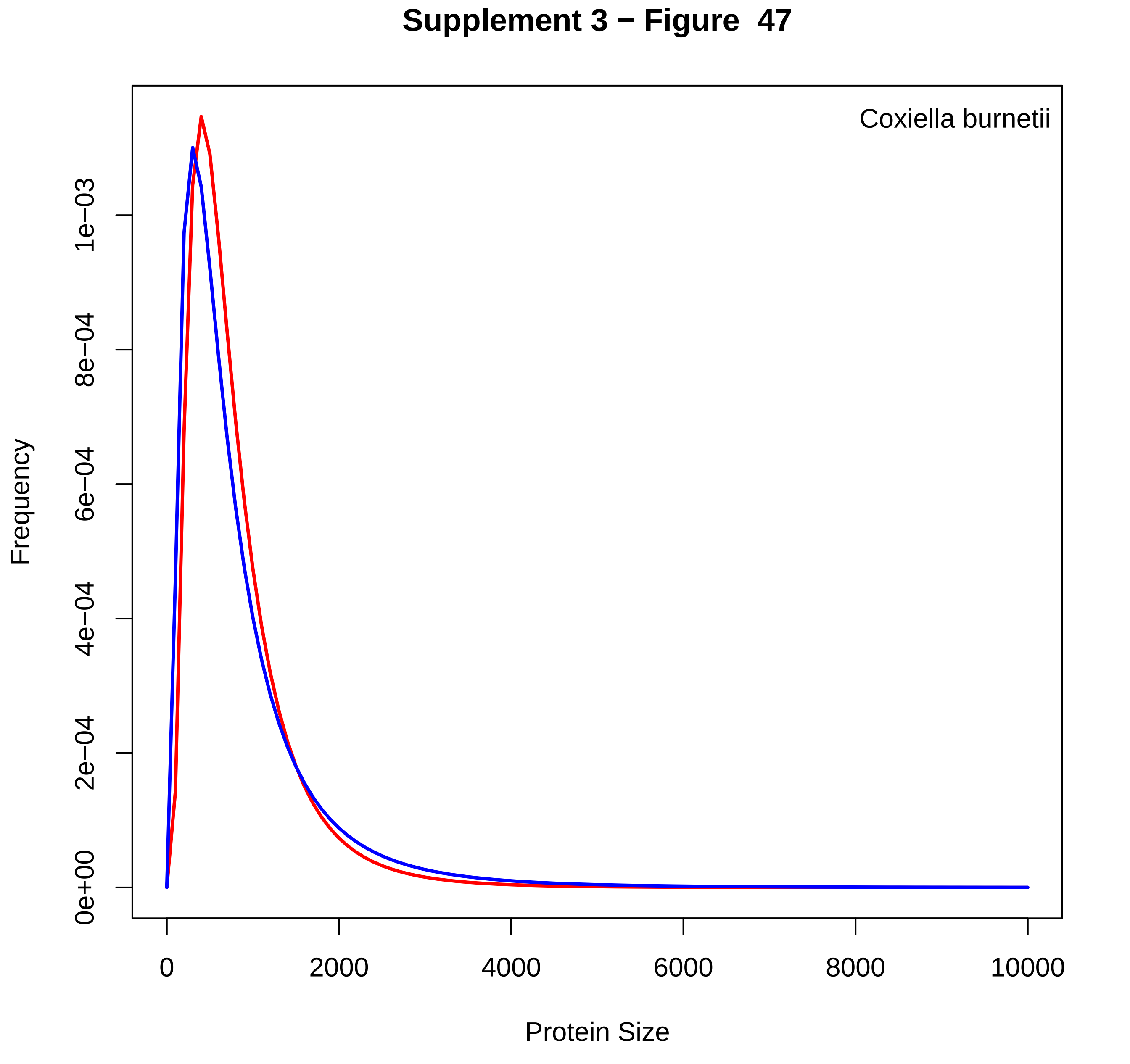
Supplement 3 − Figure 47
Coxiella burnetii
Frequency
0e+00
2e−04
4e−04
6e−04
8e−04
1e−03
0
2000
4000
6000
8000
10000
Protein Size

Supplement 3 − Figure 48

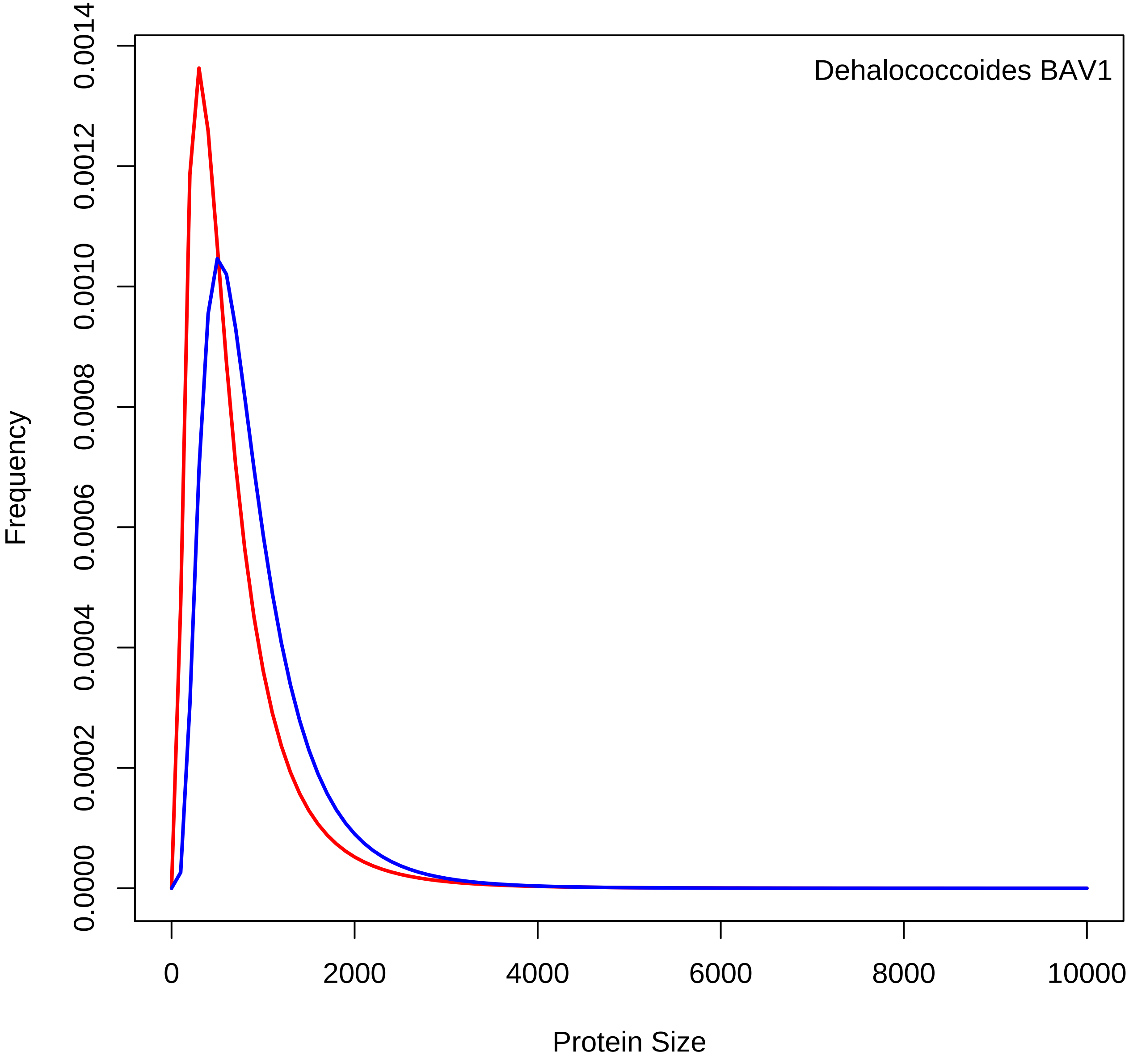

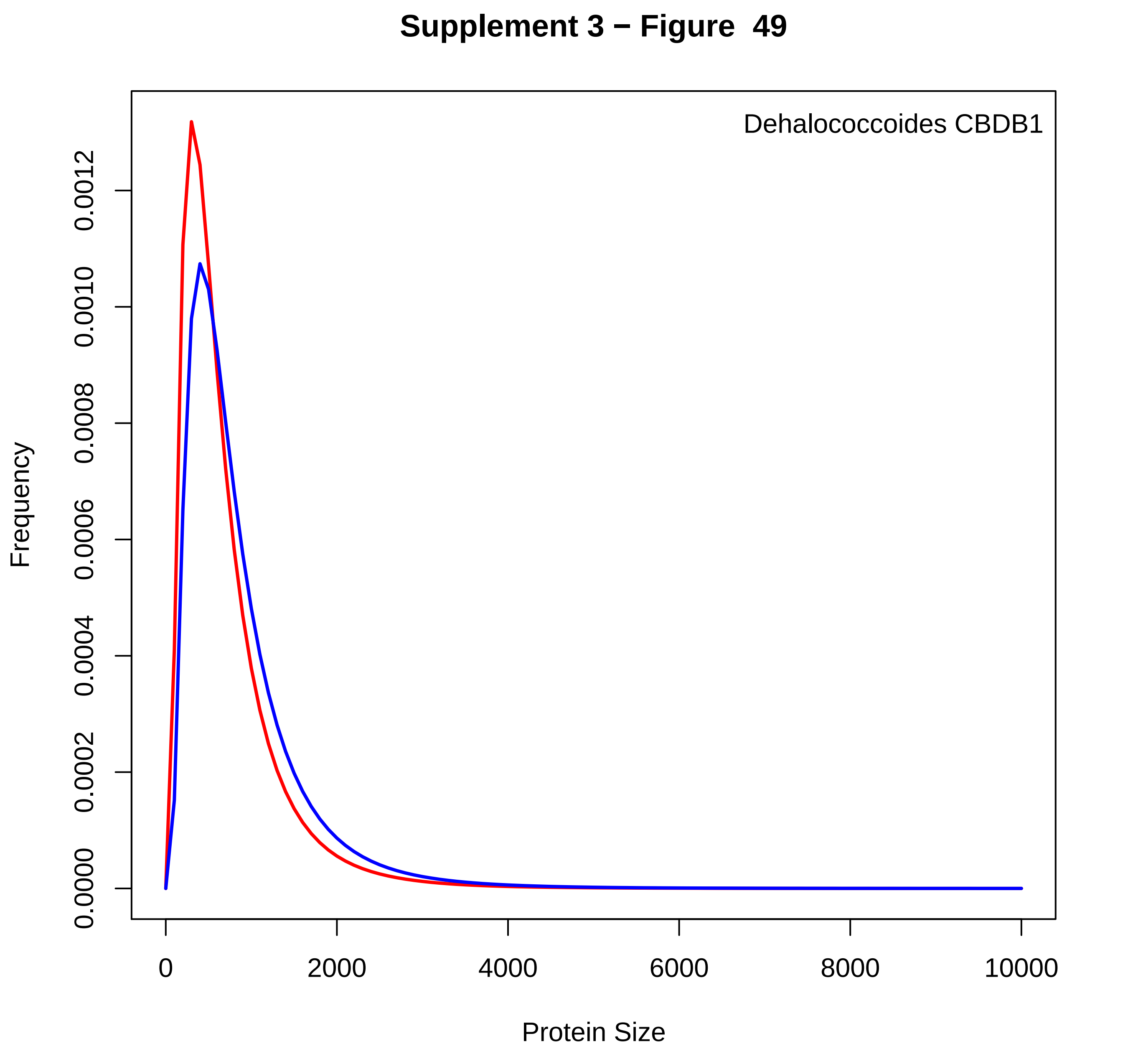
Supplement 3 − Figure 49
Dehalococcoides CBDB1
Frequency
0.0000
0.0002
0.0004
0.0006
0.0008
0.0010
0.0012
0
2000
4000
6000
8000
10000
Protein Size

Supplement 3 − Figure 50

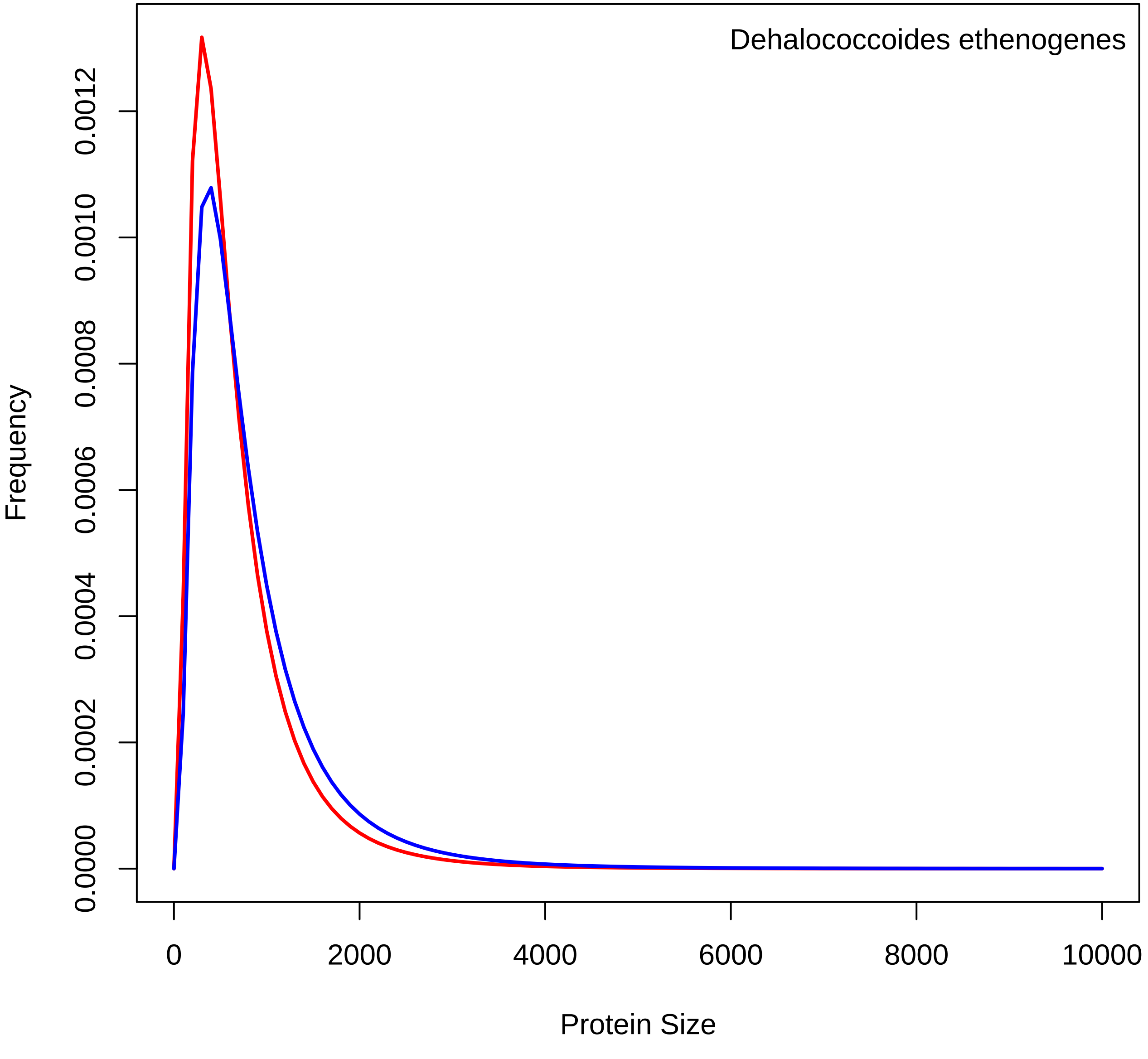

Supplement 3 − Figure 51

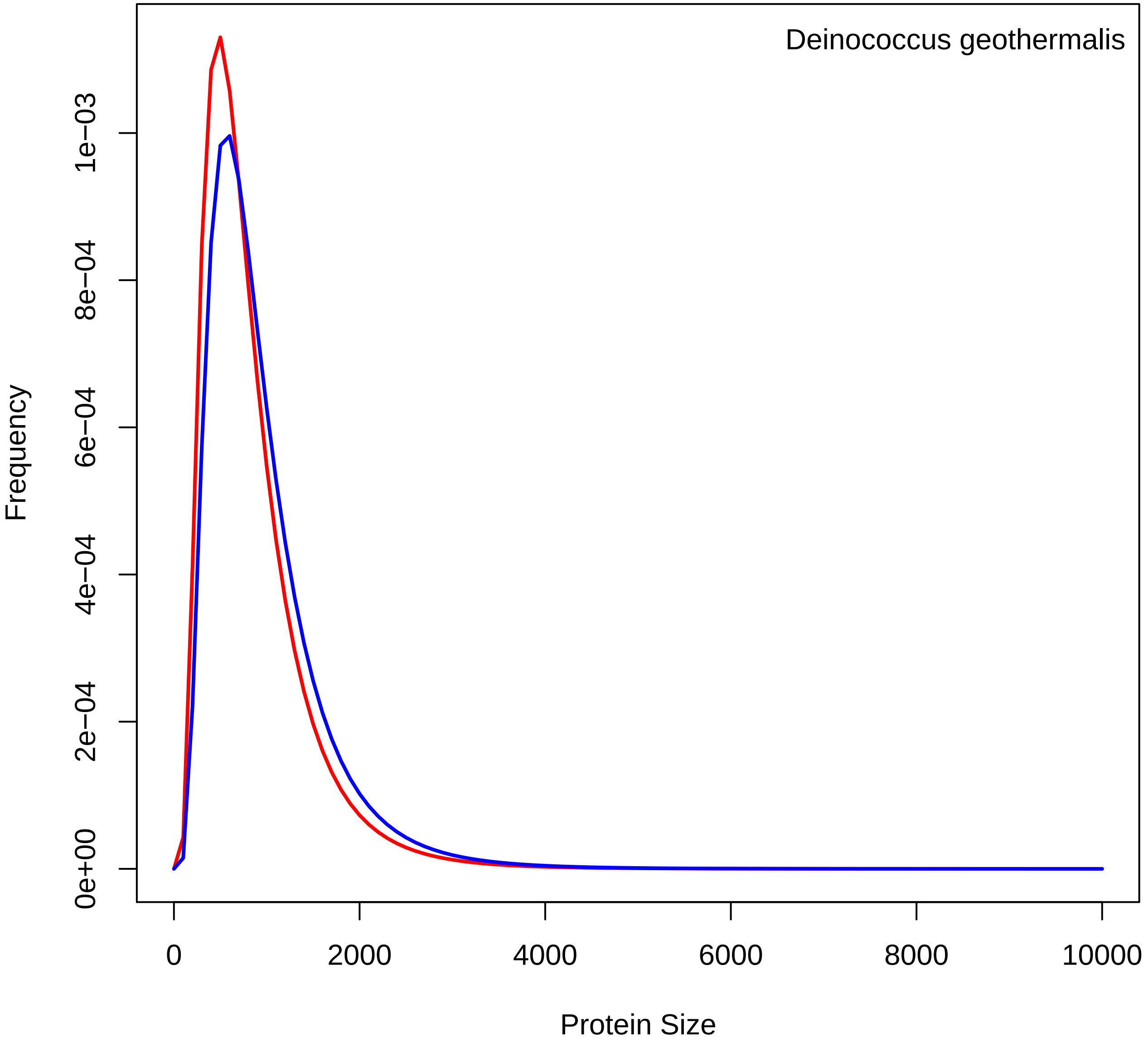

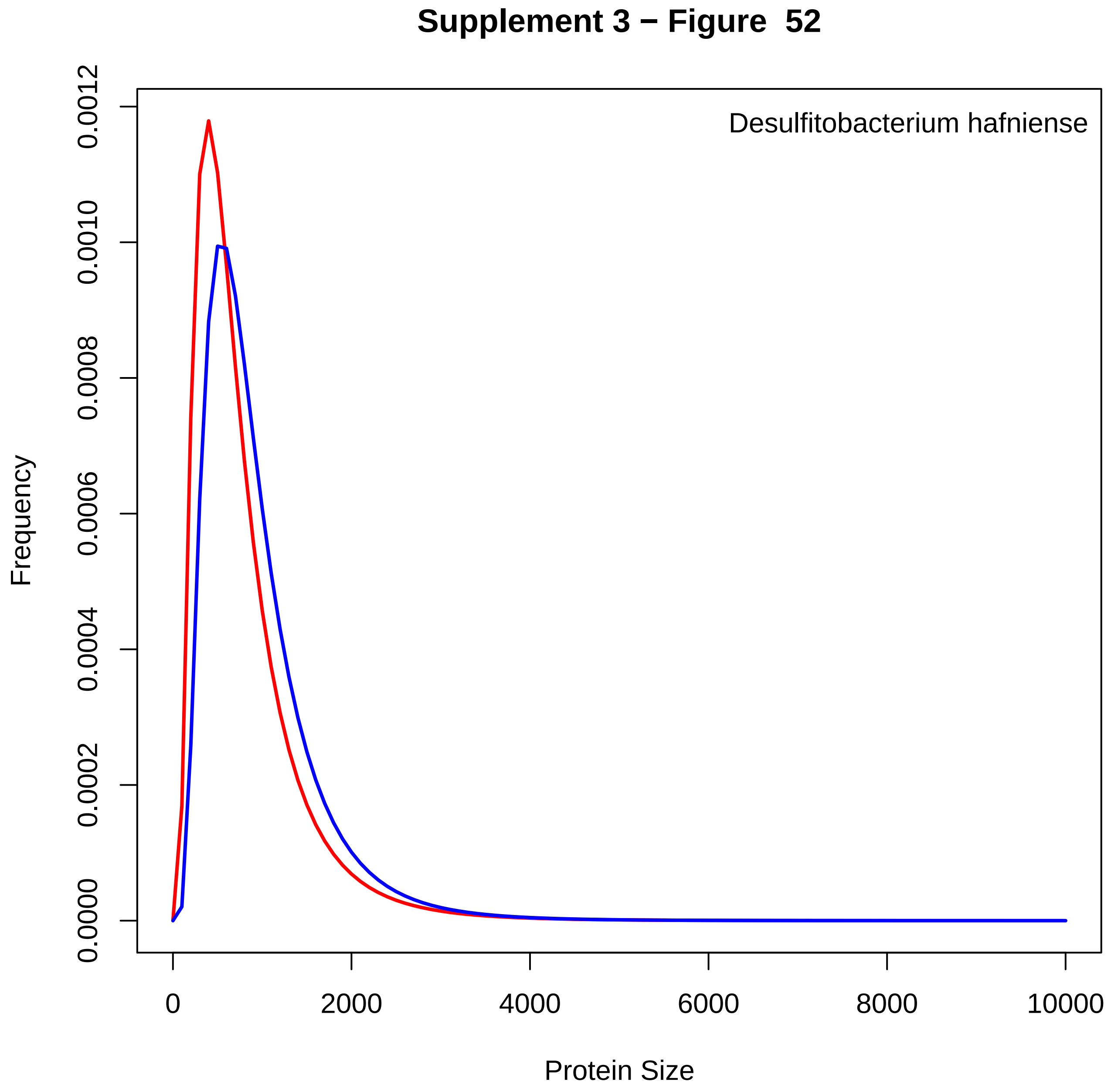
Supplement 3 − Figure 52
Desulfitobacterium hafniense
Frequency
0.0000
0.0002
0.0004
0.0006
0.0008
0.0010
0.0012
0
2000
4000
6000
8000
10000
Protein Size

Supplement 3 − Figure 53

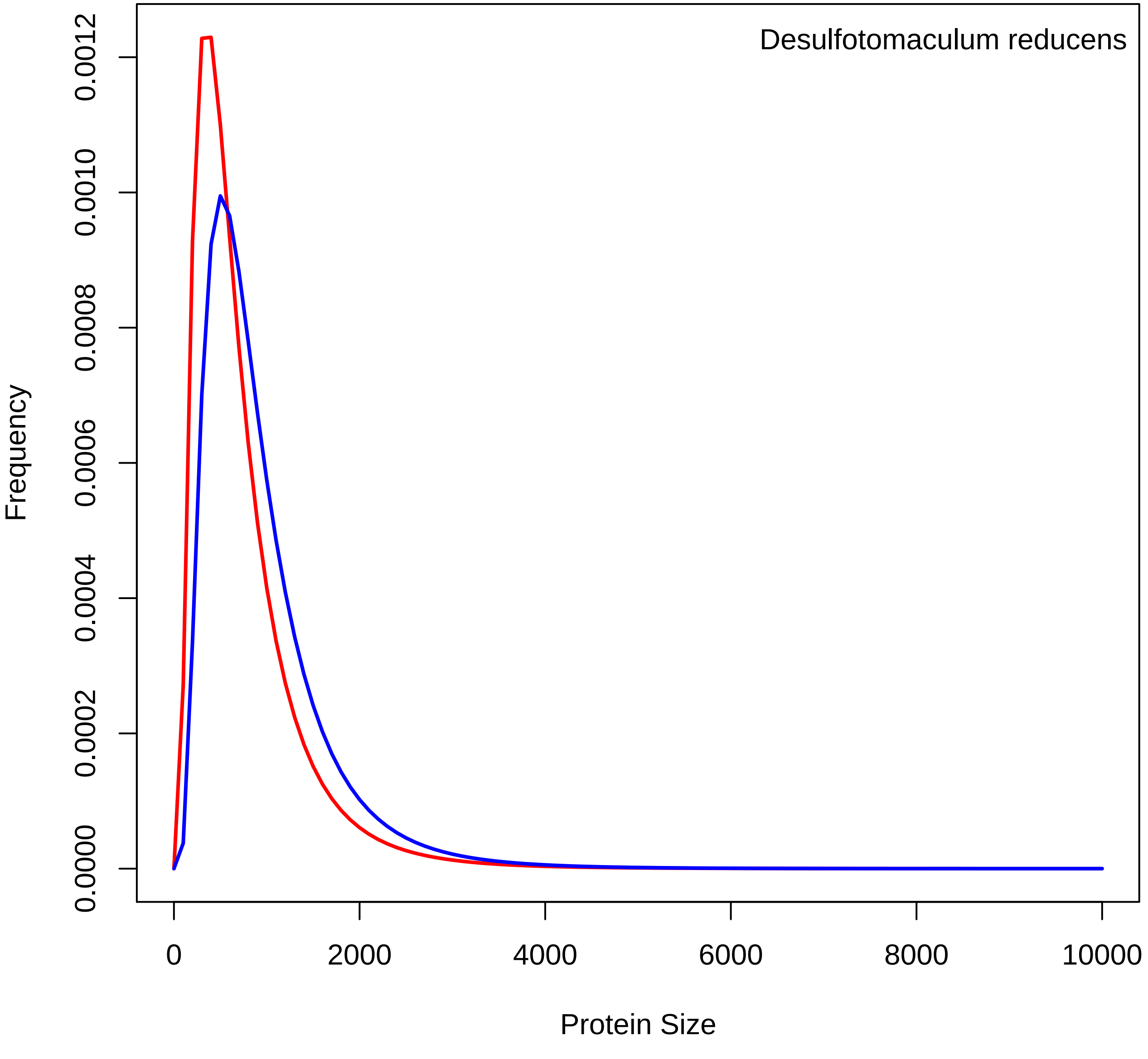

Supplement 3 − Figure 54

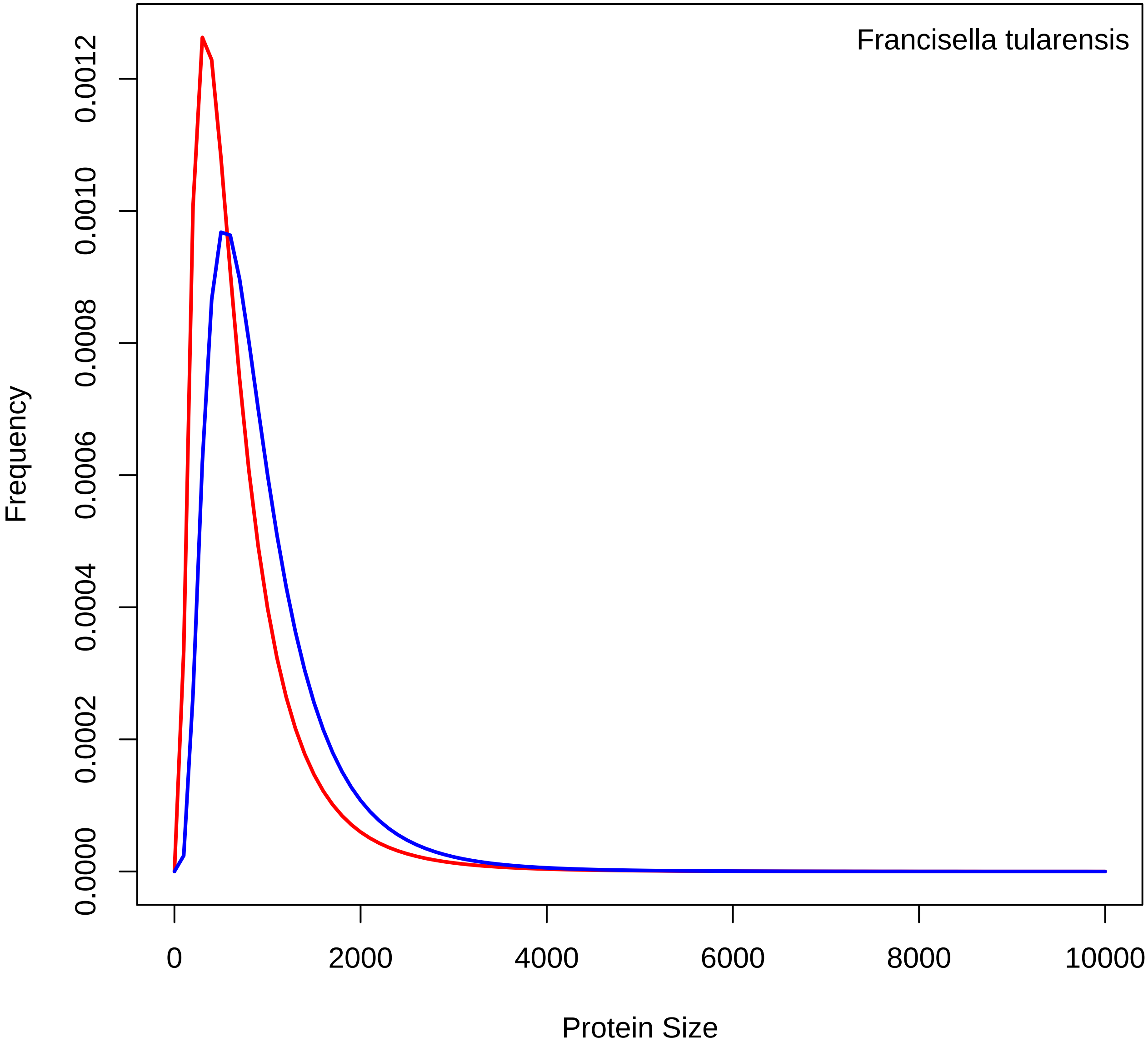

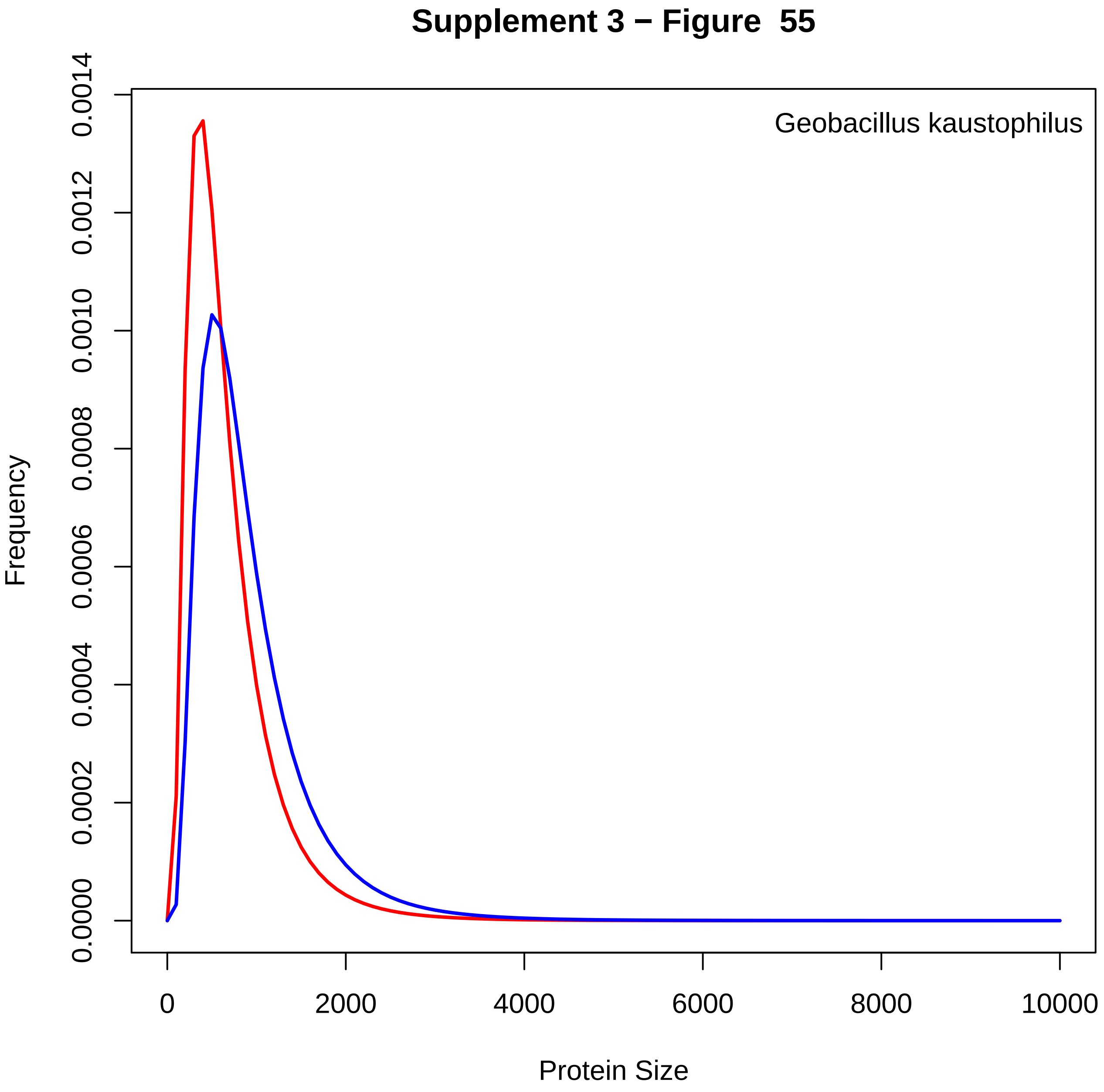
Supplement 3 − Figure 55
Geobacillus kaustophilus
Frequency
0.0000
0.0002
0.0004
0.0006
0.0008
0.0010
0.0012
0.0014
0
2000
4000
6000
8000
10000
Protein Size

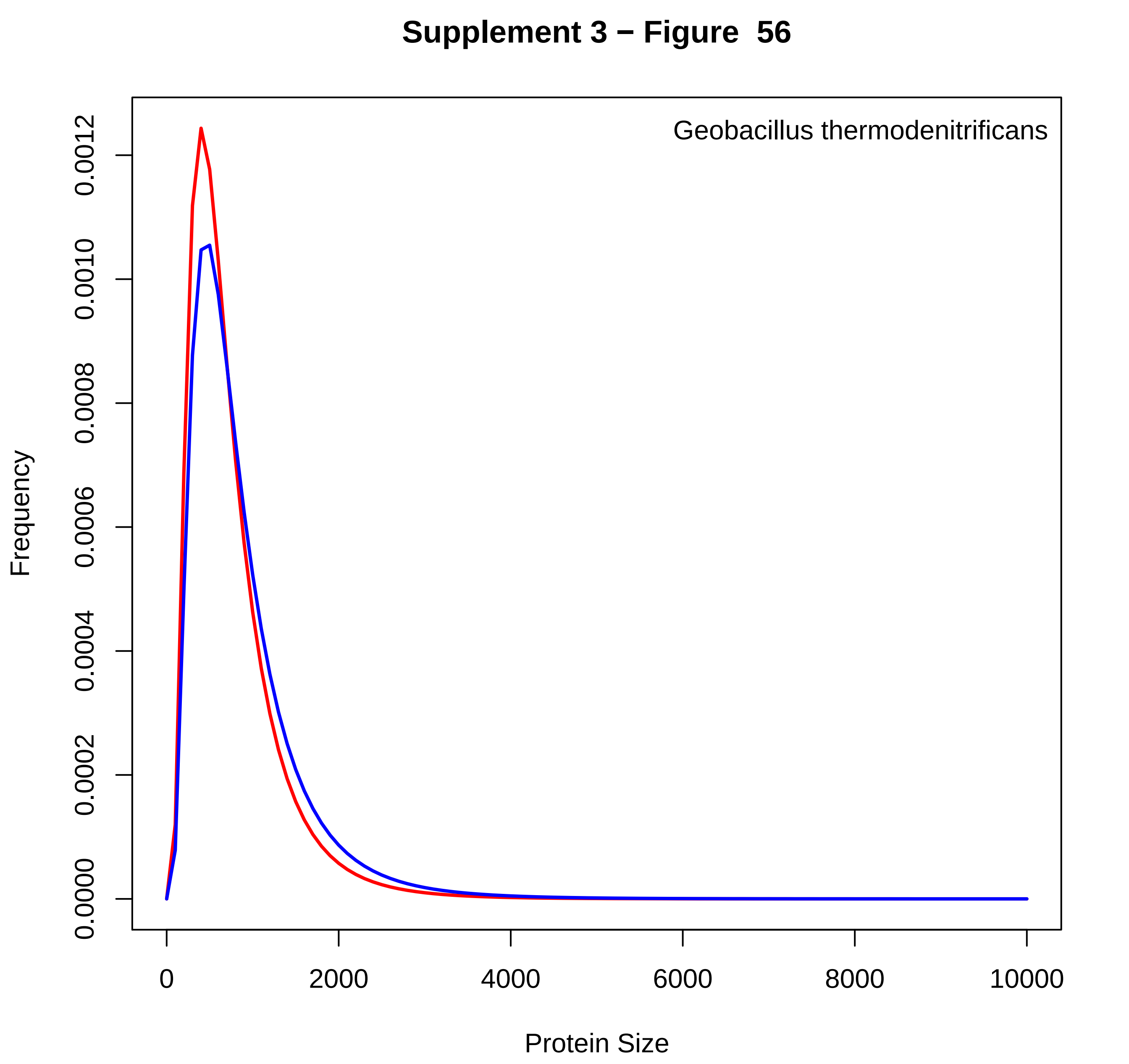
Supplement 3 − Figure 56
Geobacillus thermodenitrificans
Frequency
0.0000
0.0002
0.0004
0.0006
0.0008
0.0010
0.0012
0
2000
4000
6000
8000
10000
Protein Size

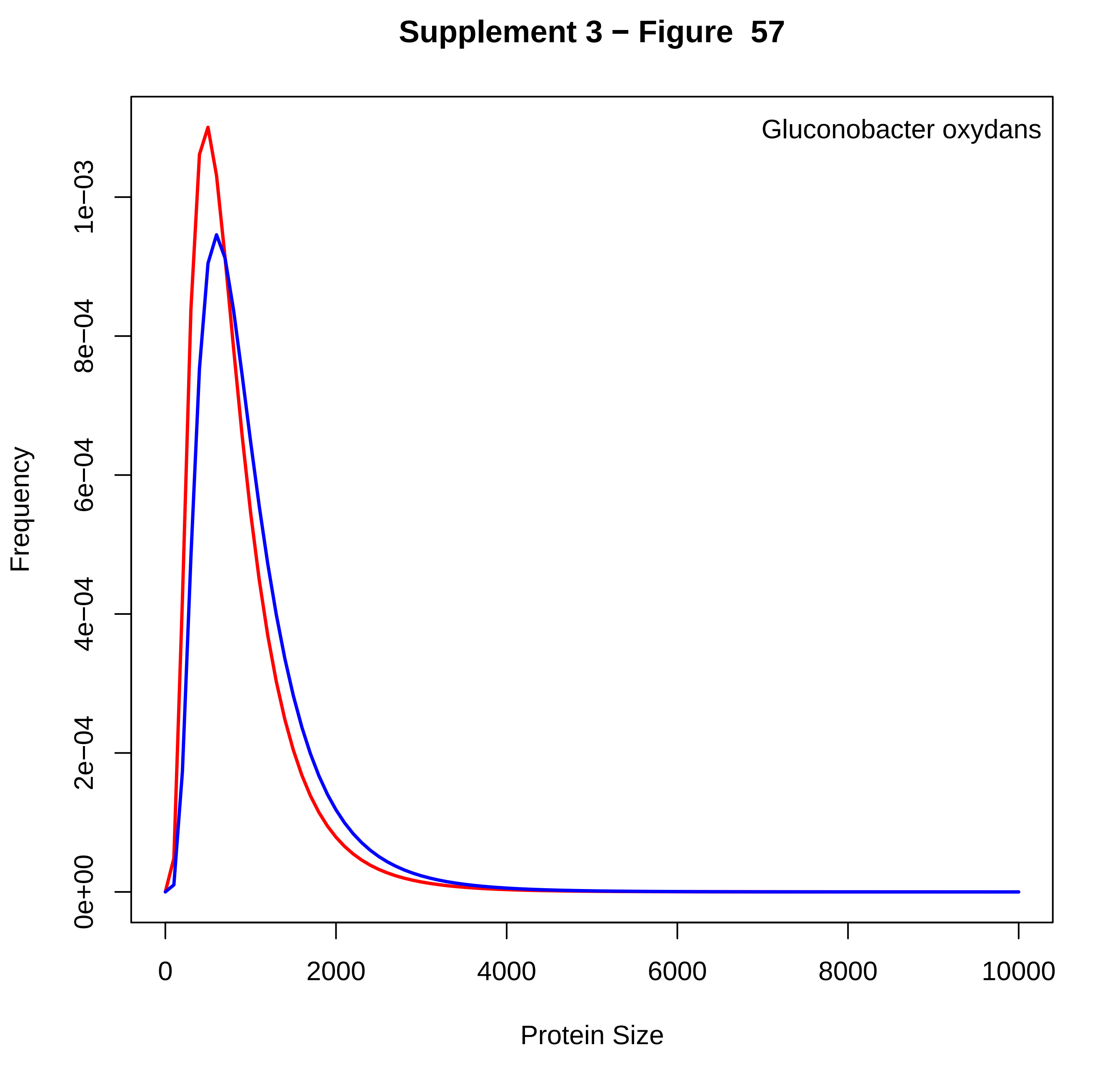
Supplement 3 − Figure 57
Gluconobacter oxydans
Frequency
0e+00
2e−04
4e−04
6e−04
8e−04
1e−03
0
2000
4000
6000
8000
10000
Protein Size

Supplement 3 − Figure 58

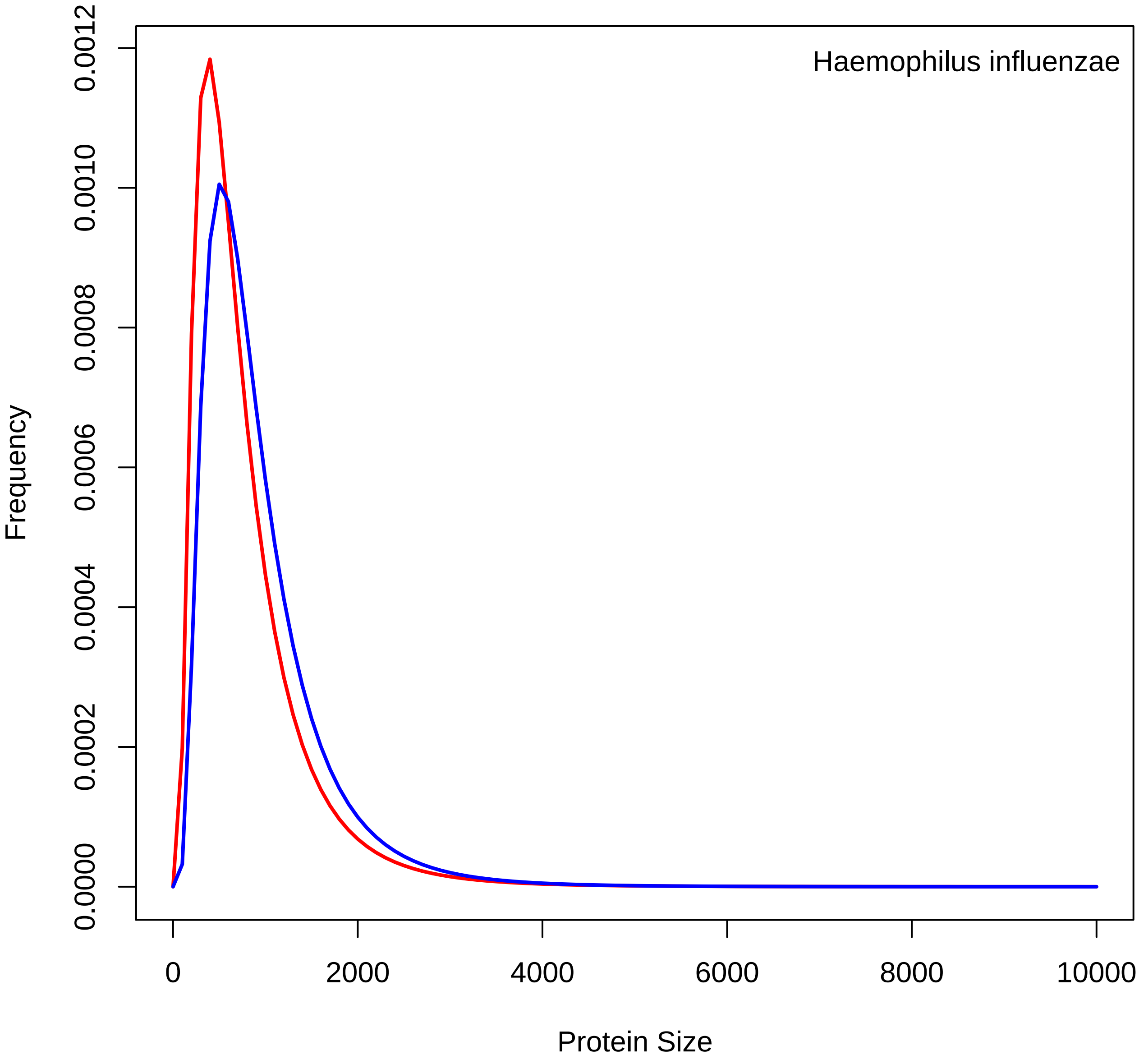

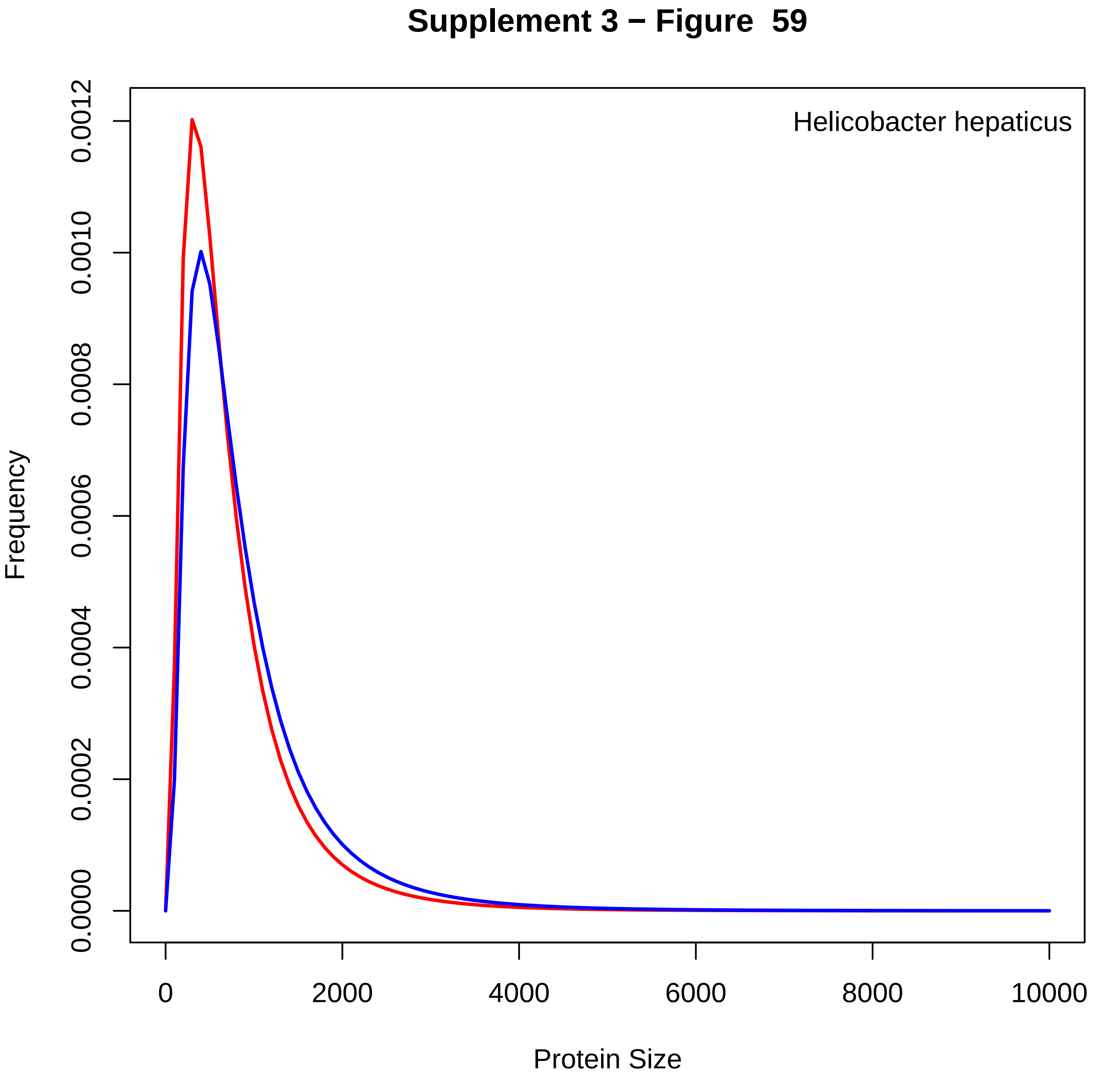

Supplement 3 − Figure 59
Helicobacter hepaticus
Frequency
0.0000
0.0002
0.0004
0.0006
0.0008
0.0010
0.0012
0
2000
4000
6000
8000
10000
Protein Size

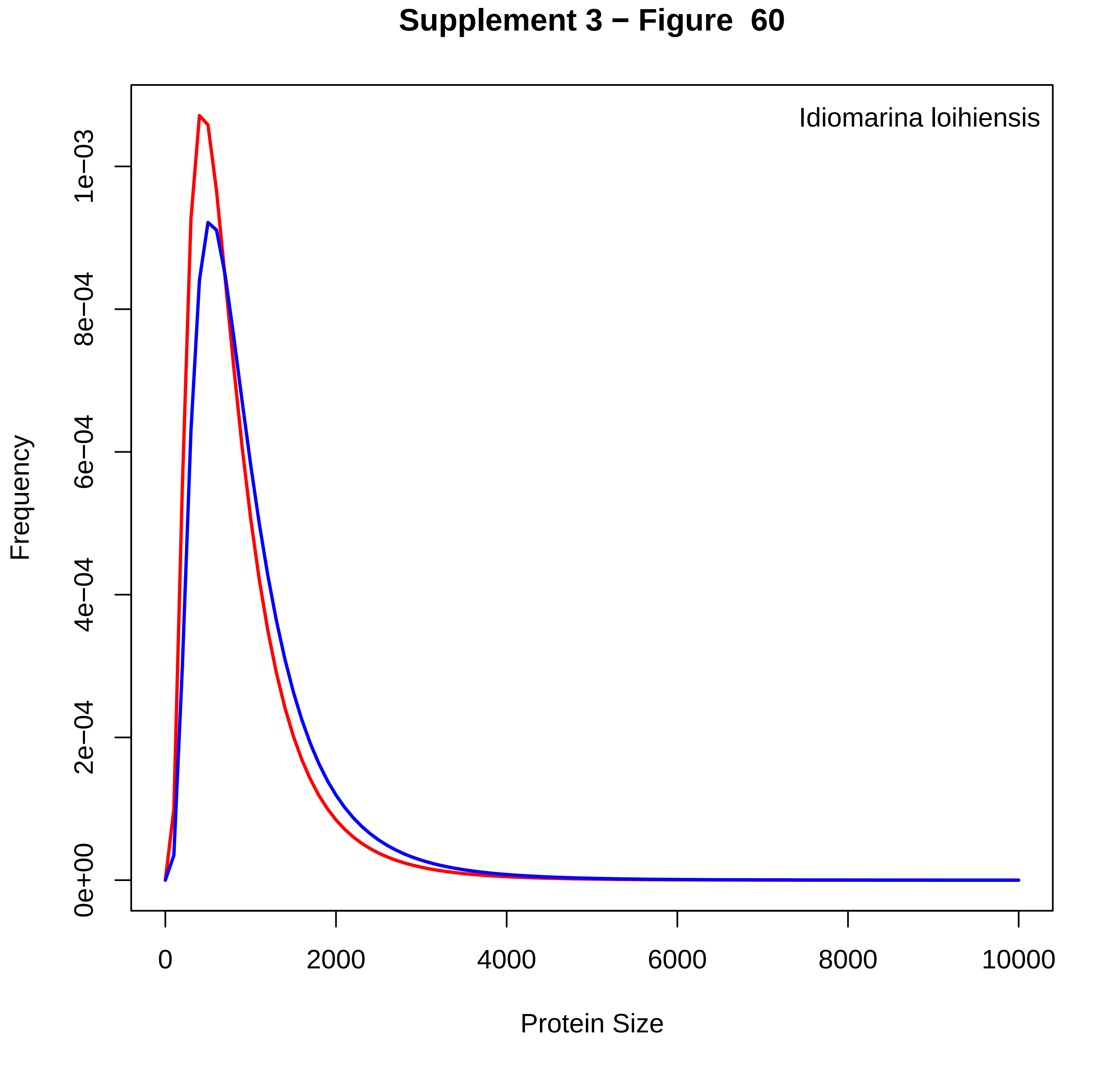

Supplement 3 − Figure 60
Idiomarina loihiensis
Frequency
0e+00
2e−04
4e−04
6e−04
8e−04
1e−03
0
2000
4000
6000
8000
10000
Protein Size

Supplement 3 − Figure 61

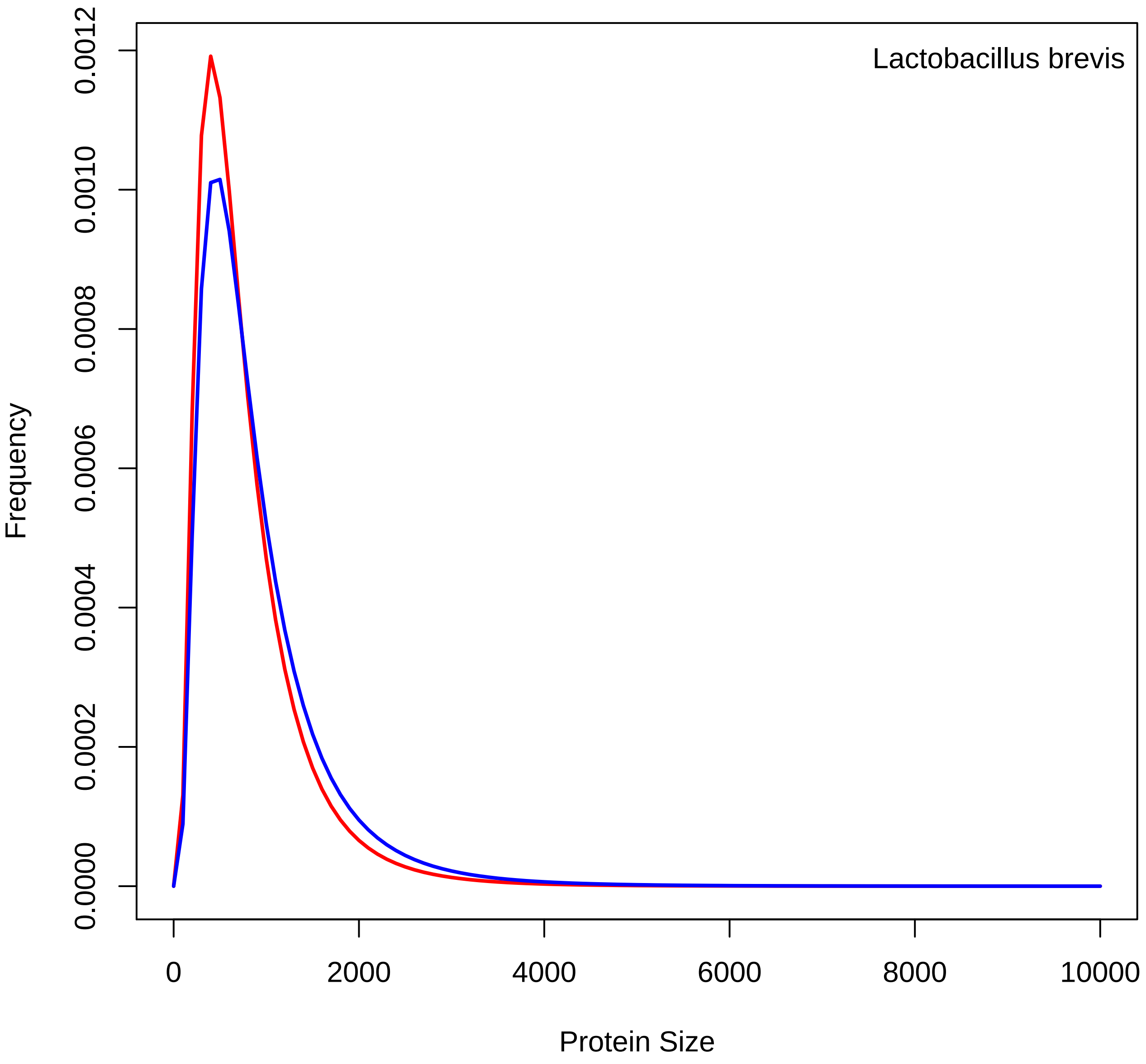

Supplement 3 − Figure 62

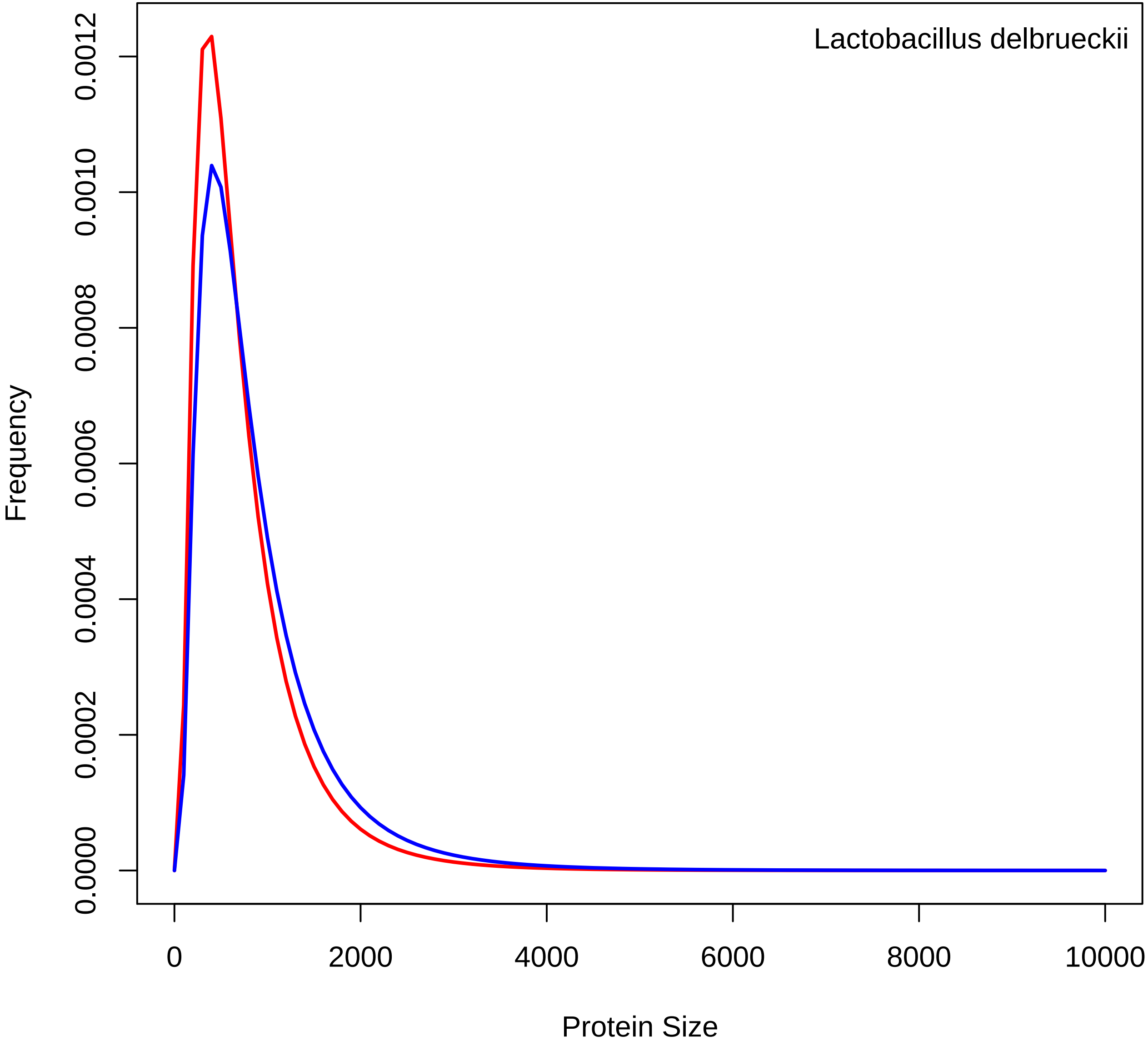

Supplement 3 − Figure 63

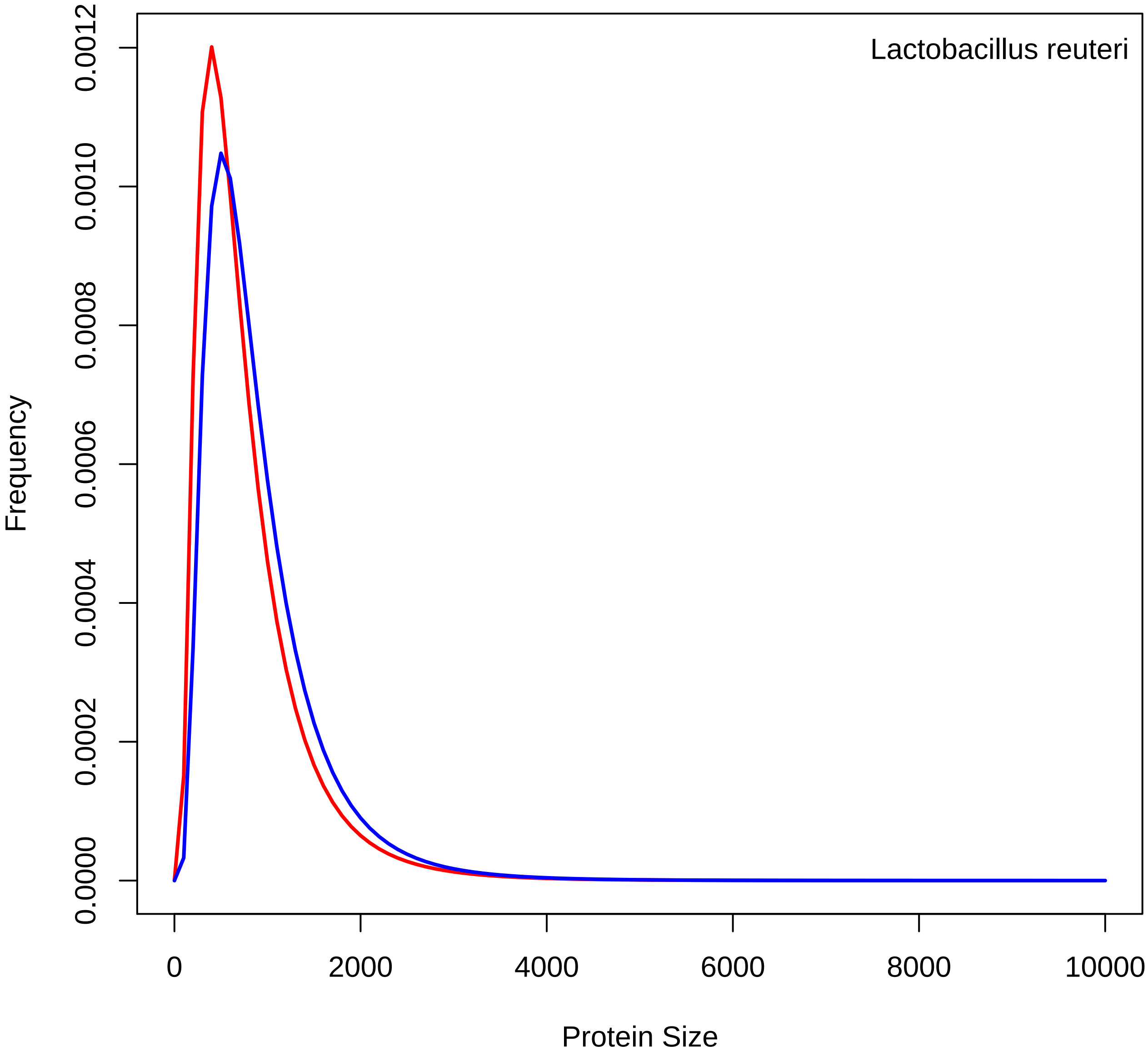

Supplement 3 − Figure 64

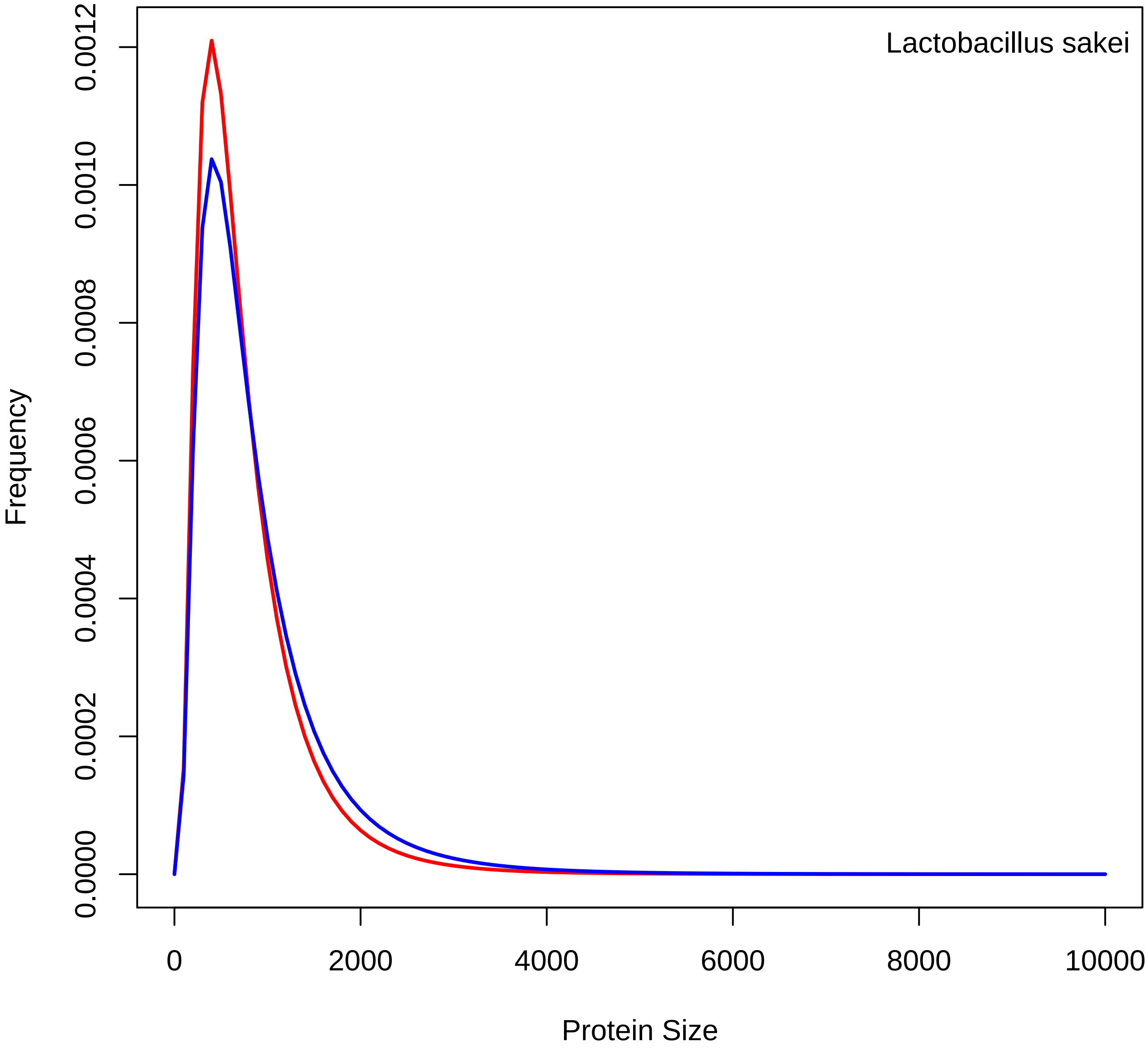

Supplement 3 − Figure 65

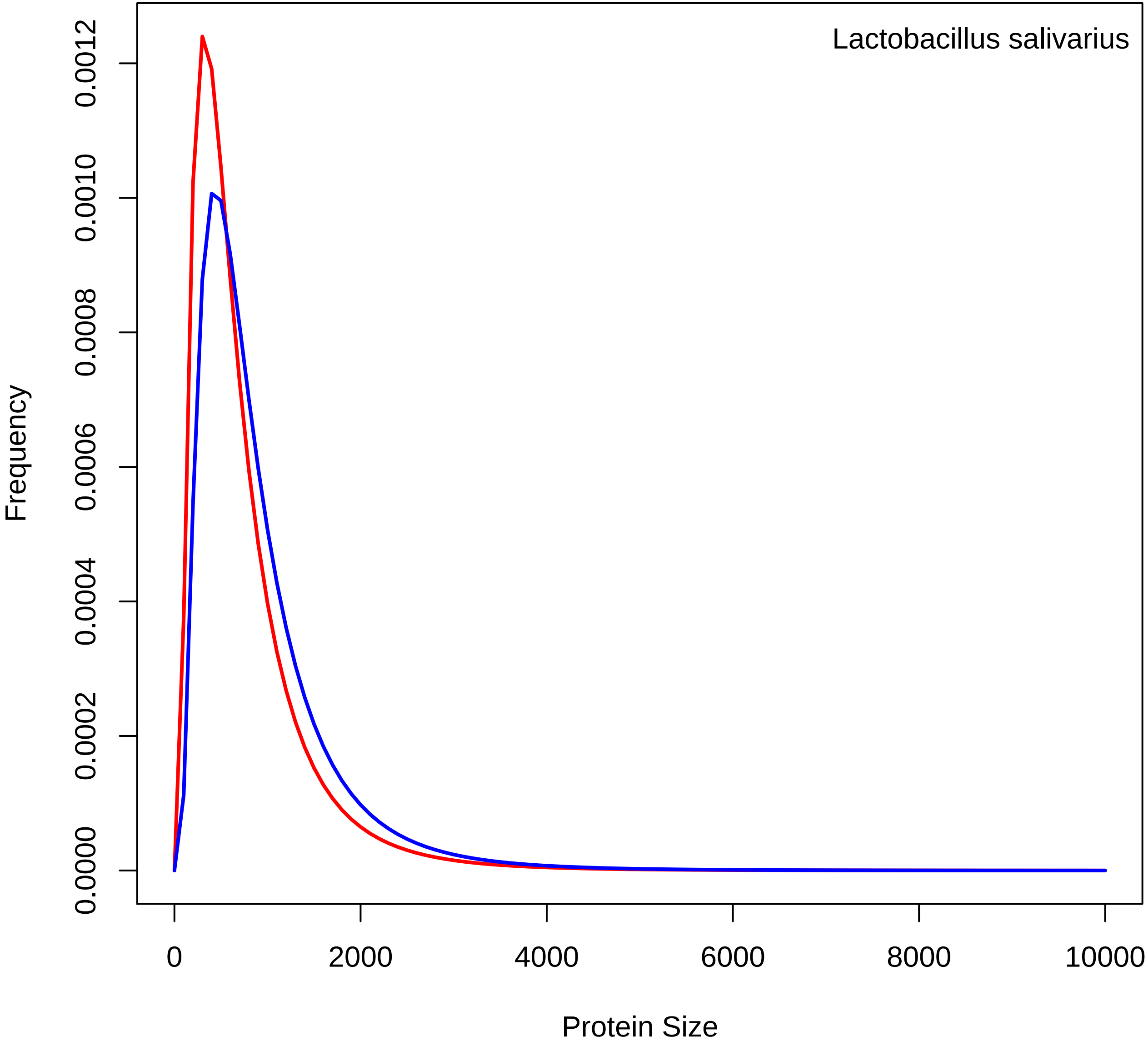

Supplement 3 − Figure 66

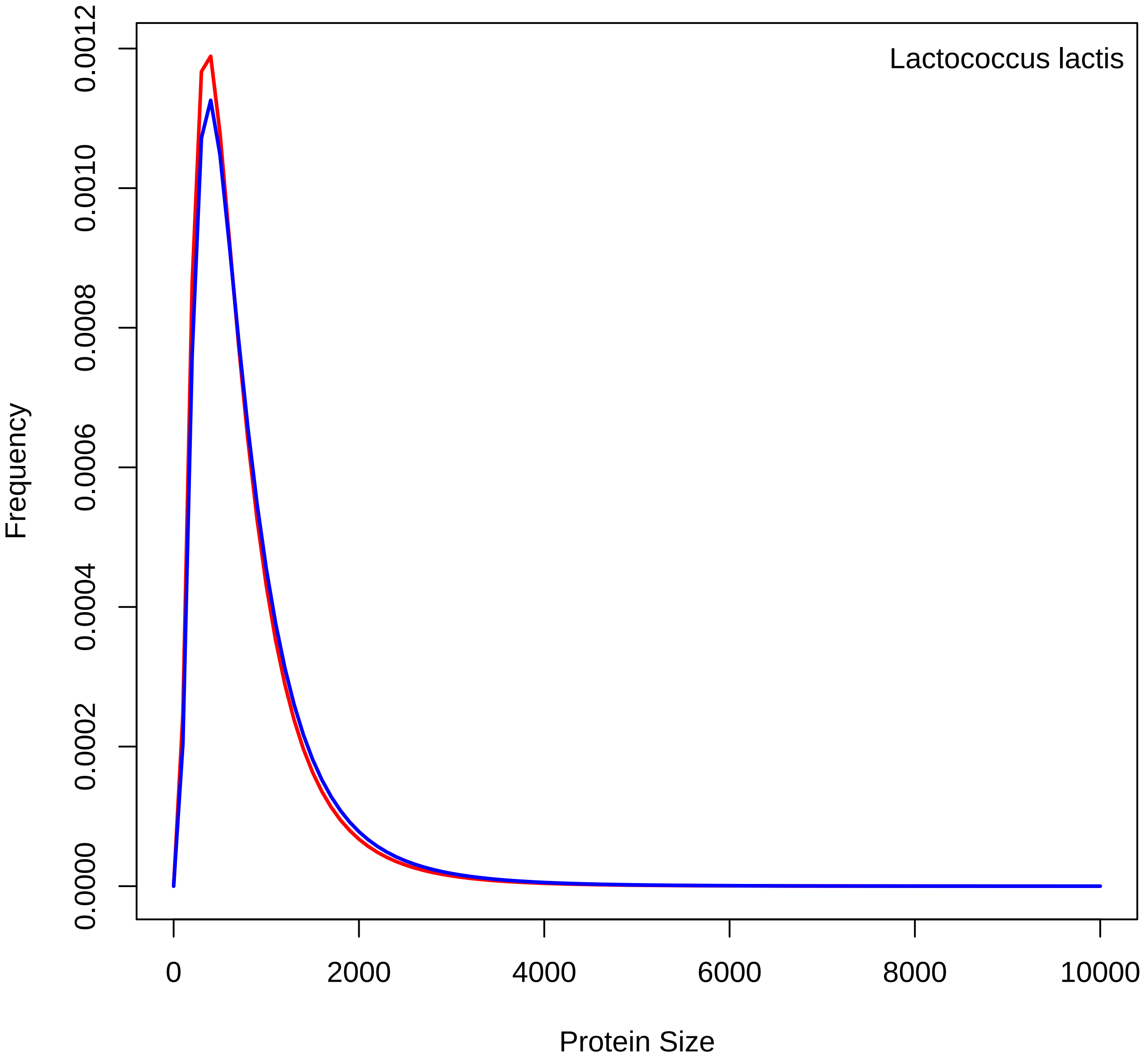

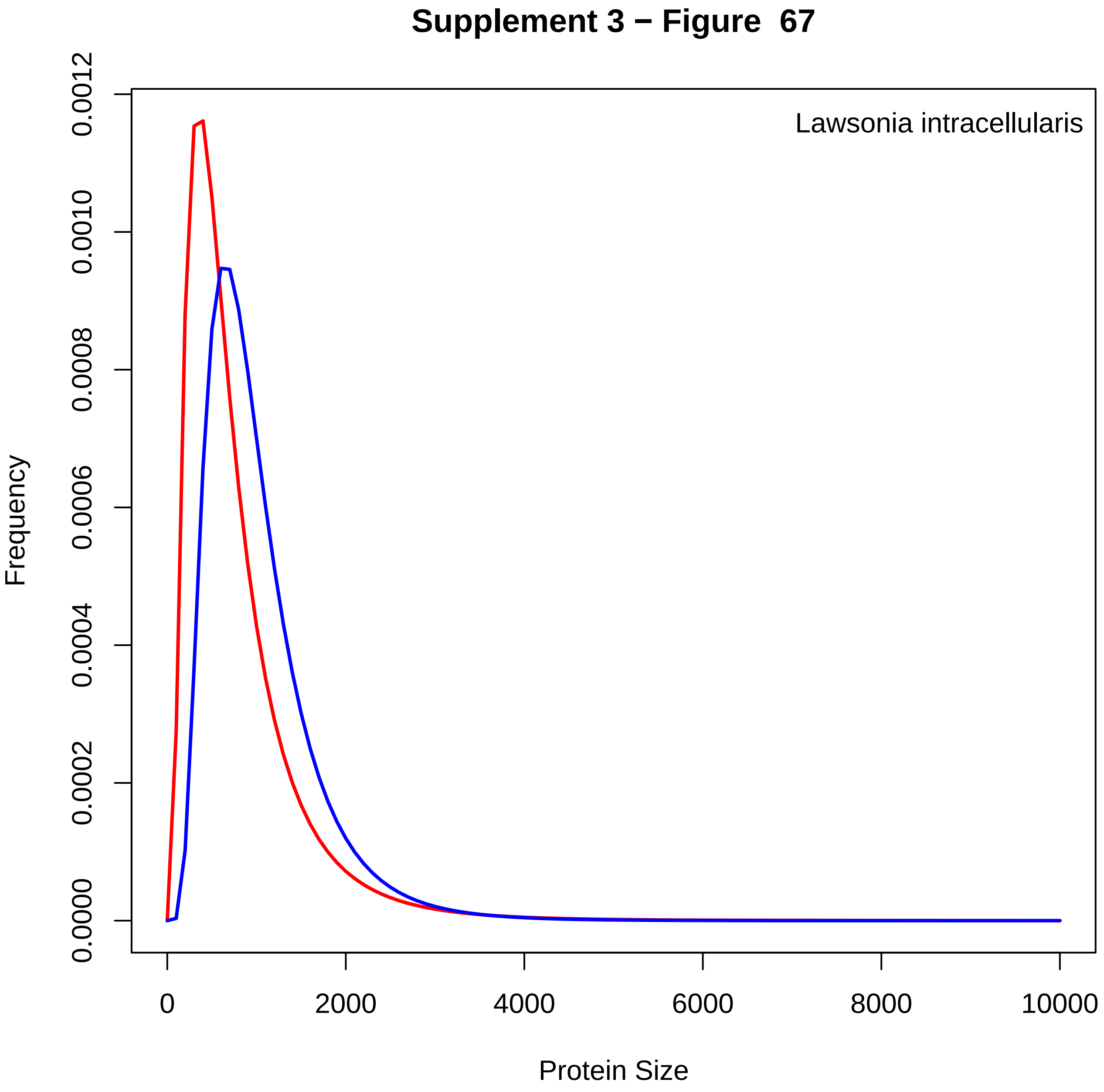

Supplement 3 − Figure 67
Lawsonia intracellularis
Frequency
0.0000
0.0002
0.0004
0.0006
0.0008
0.0010
0.0012
Protein Size
0
2000
4000
6000
8000
10000

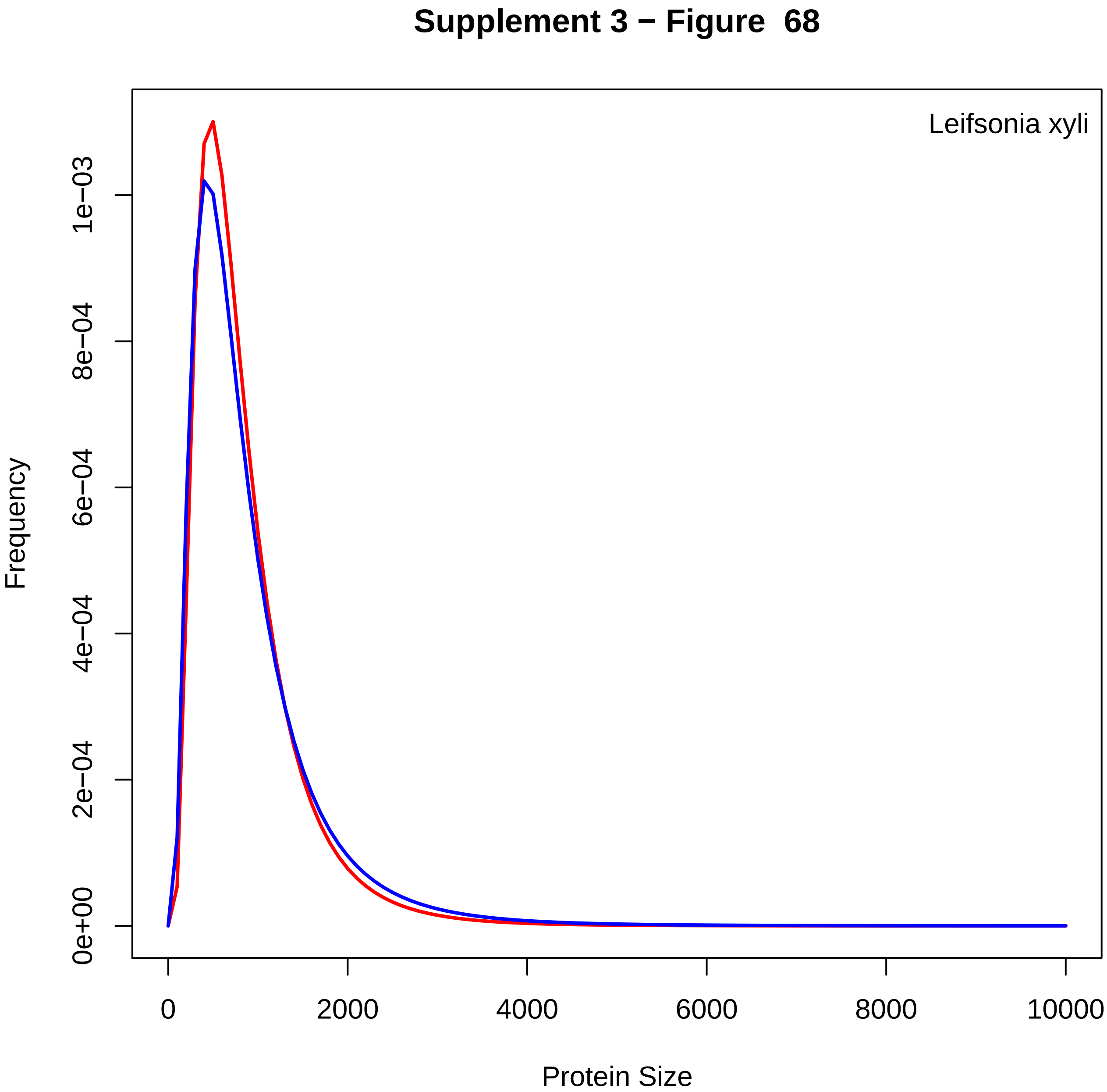
Supplement 3 − Figure 68
Leifsonia xyli
Frequency
0e+00
2e−04
4e−04
6e−04
8e−04
1e−03
0
2000
4000
6000
8000
10000
Protein Size

Supplement 3 − Figure 69

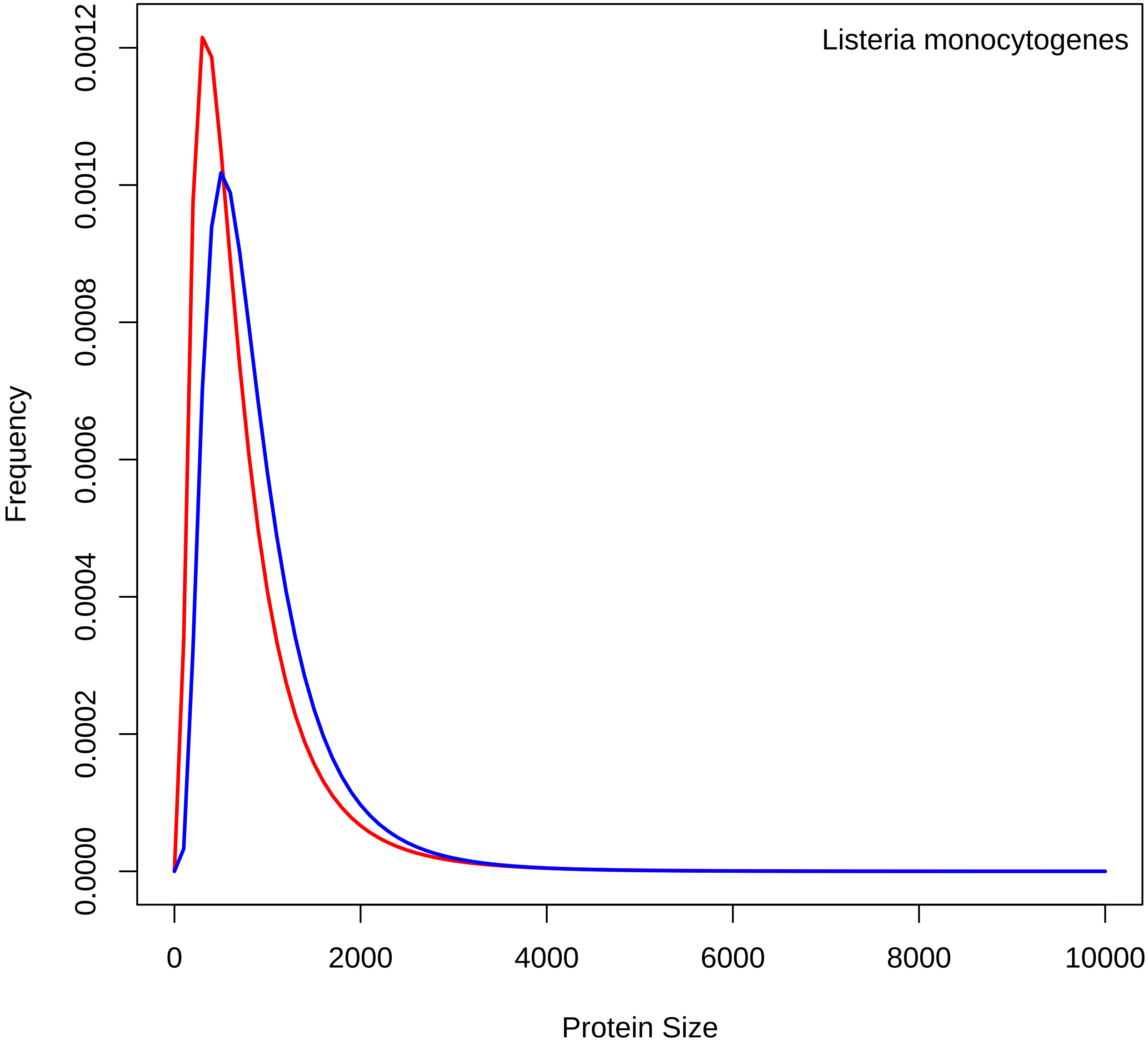

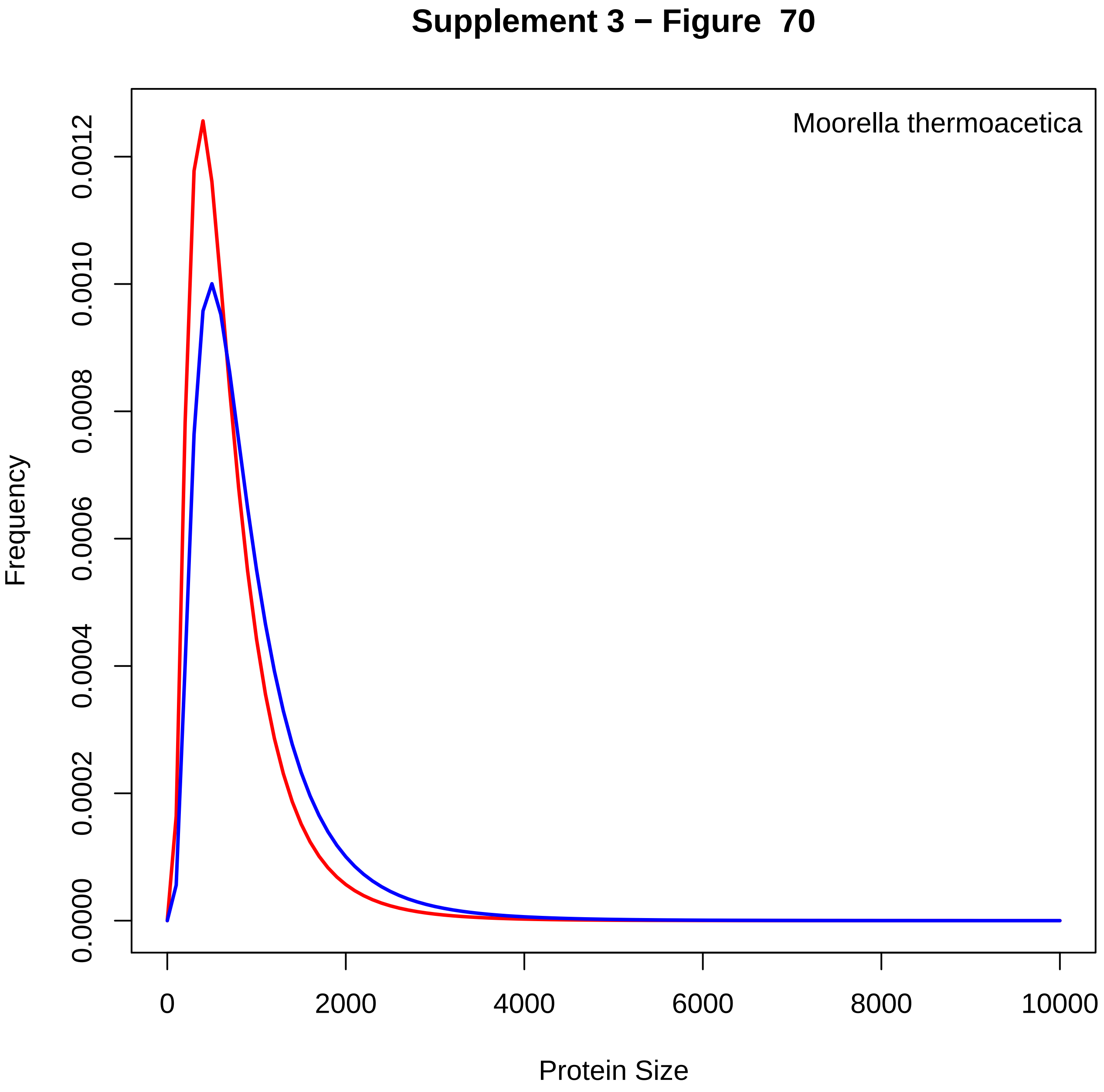

Supplement 3 − Figure 70
Moorella thermoacetica
Frequency
0.0000
0.0002
0.0004
0.0006
0.0008
0.0010
0.0012
0
2000
4000
6000
8000
10000
Protein Size

Supplement 3 − Figure 71

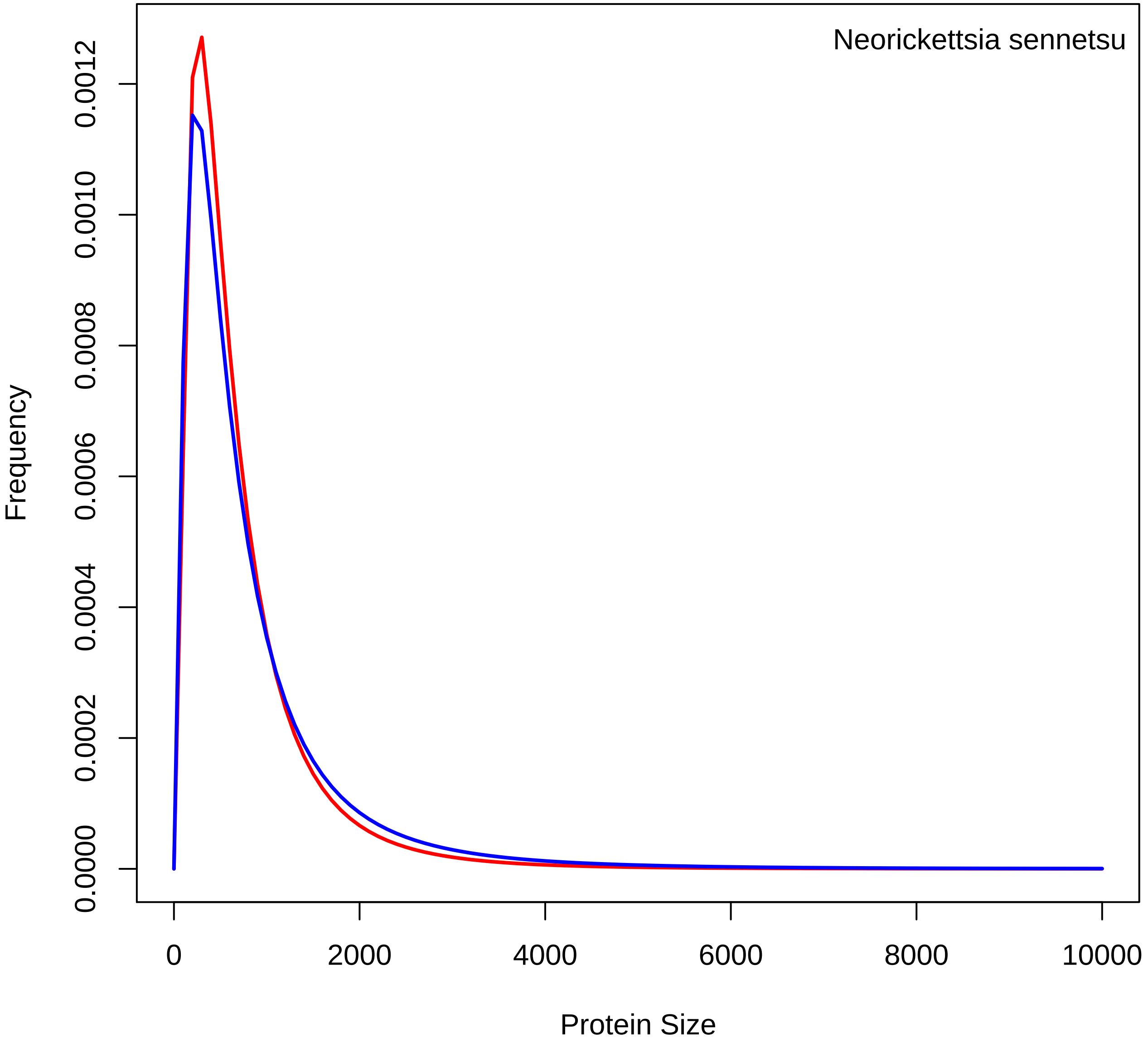

Supplement 3 − Figure 72

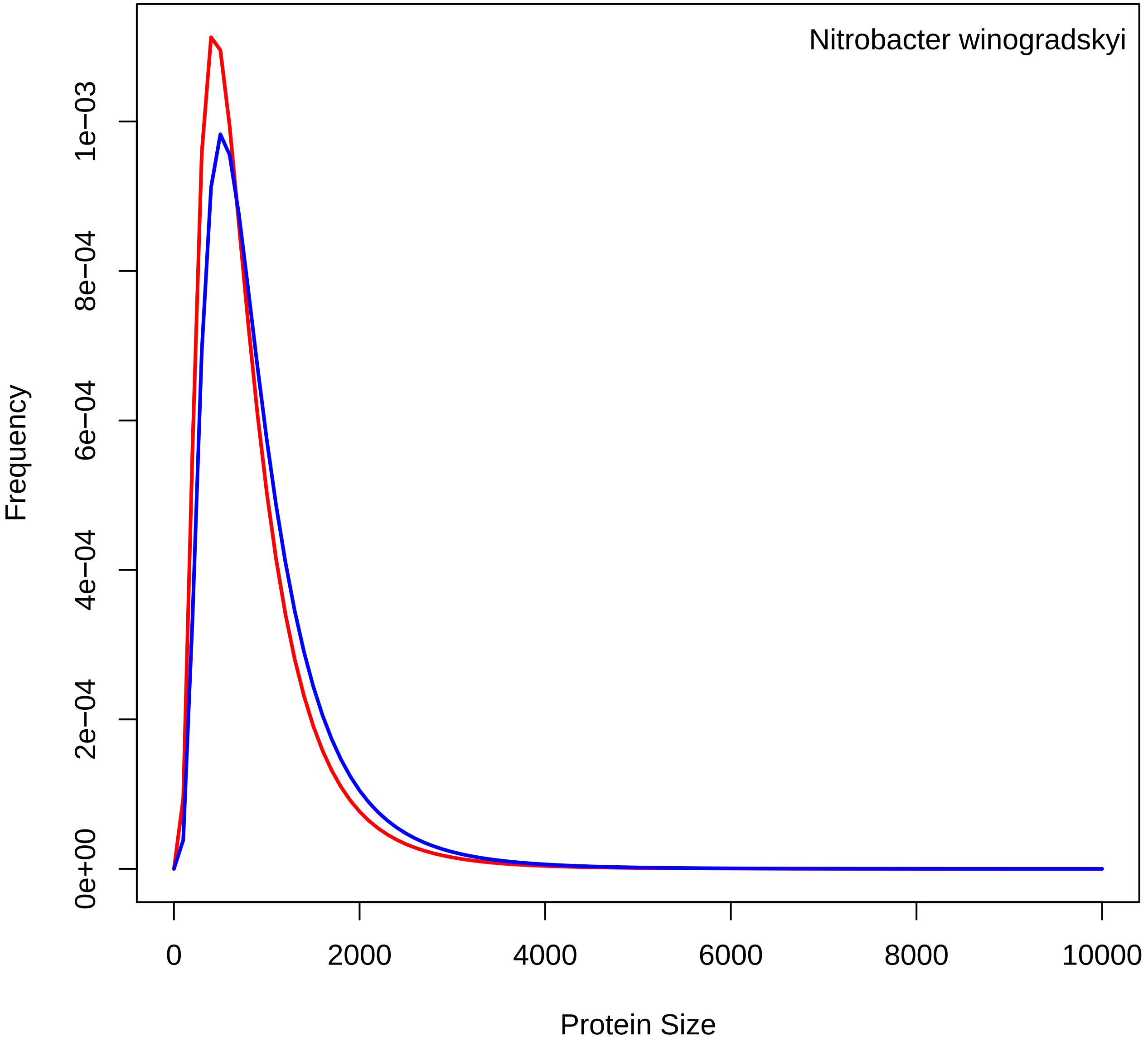

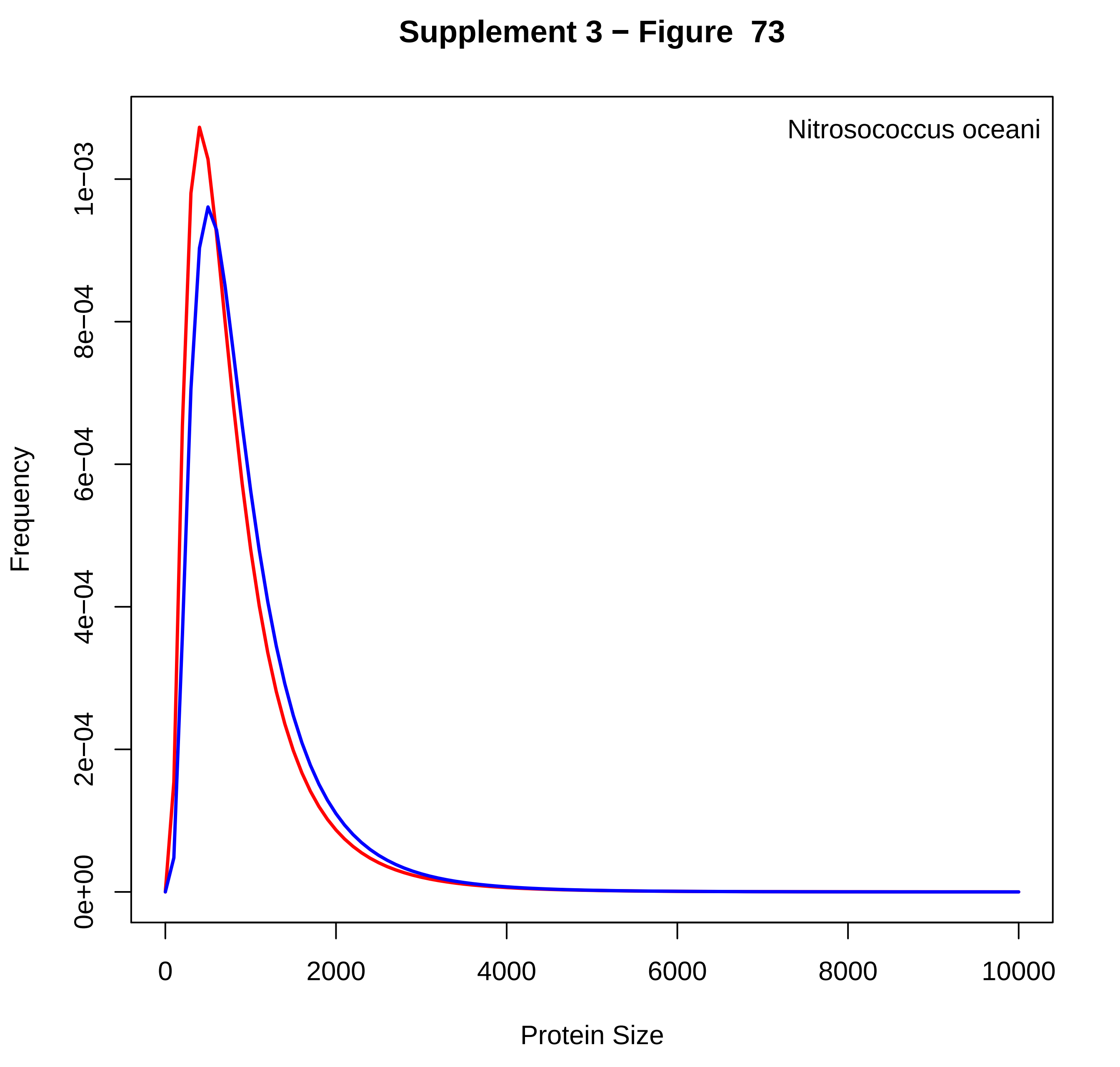

Supplement 3 − Figure 73
Nitrosococcus oceani
Frequency
0e+00
2e−04
4e−04
6e−04
8e−04
1e−03
0
2000
4000
6000
8000
10000
Protein Size

Supplement 3 − Figure 74

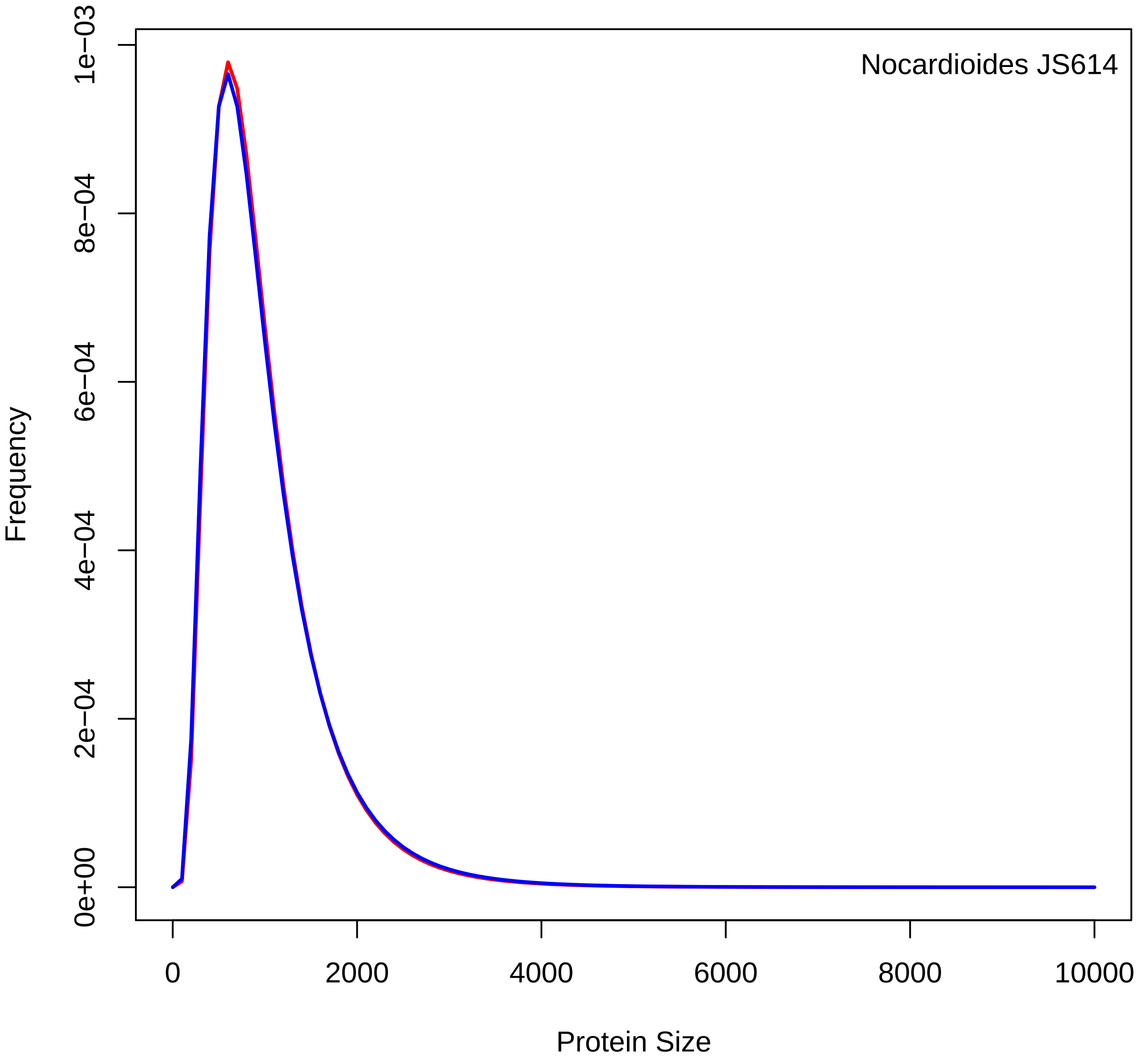

Supplement 3 − Figure 75

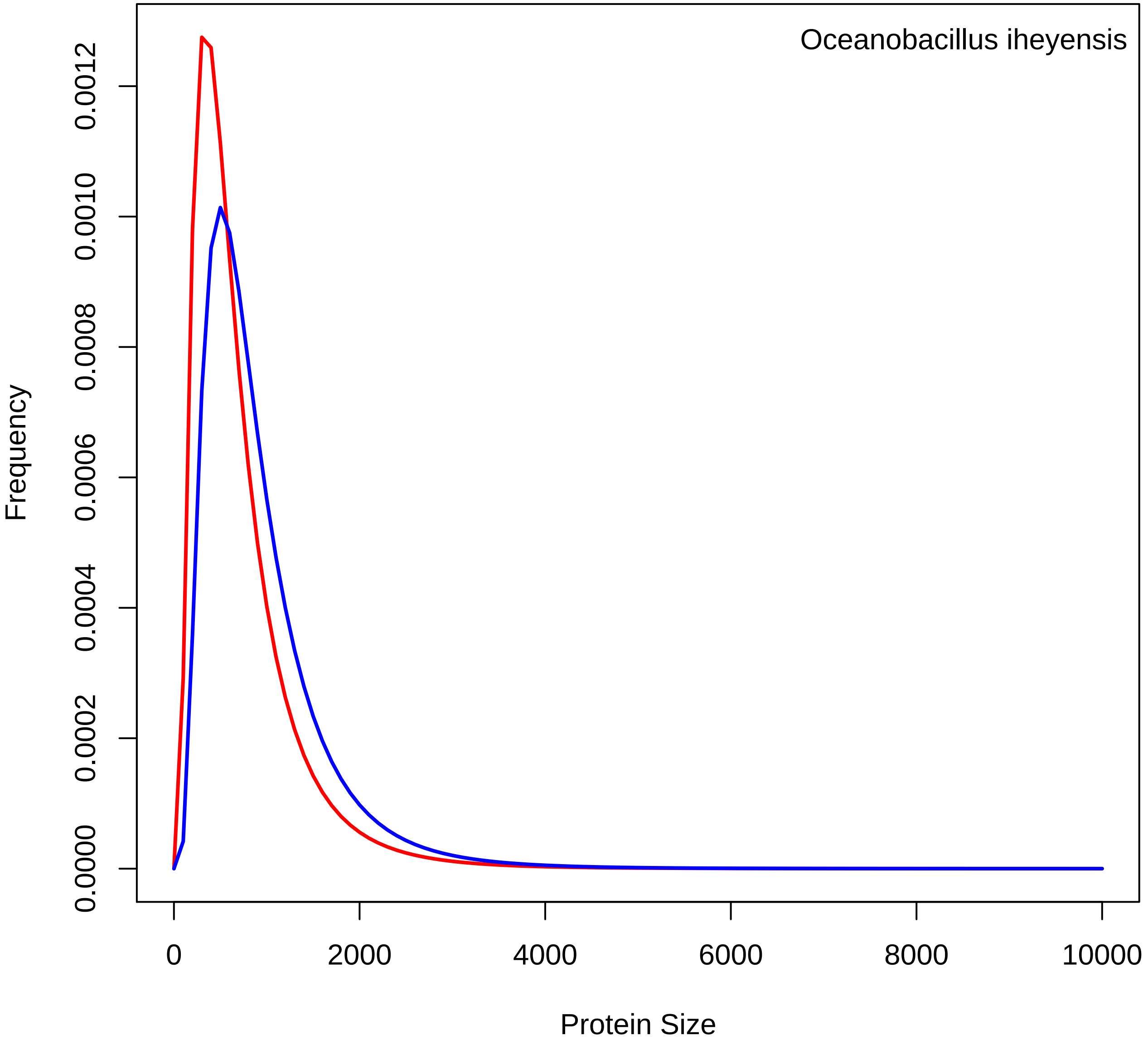

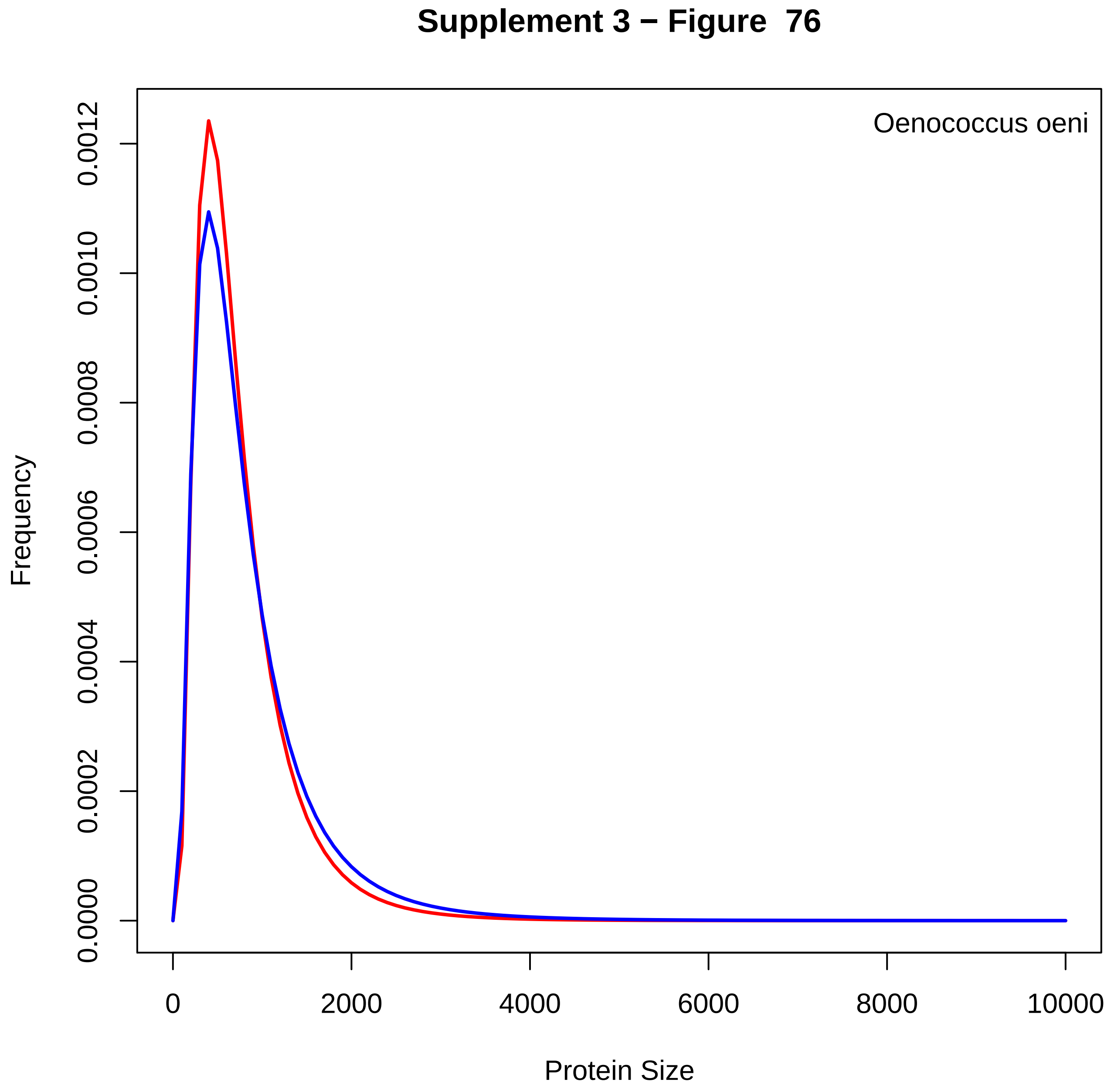
Supplement 3 − Figure 76
Oenococcus oeni
Frequency
0.0000
0.0002
0.0004
0.0006
0.0008
0.0010
0.0012
0
2000
4000
6000
8000
10000
Protein Size

Supplement 3 − Figure 77

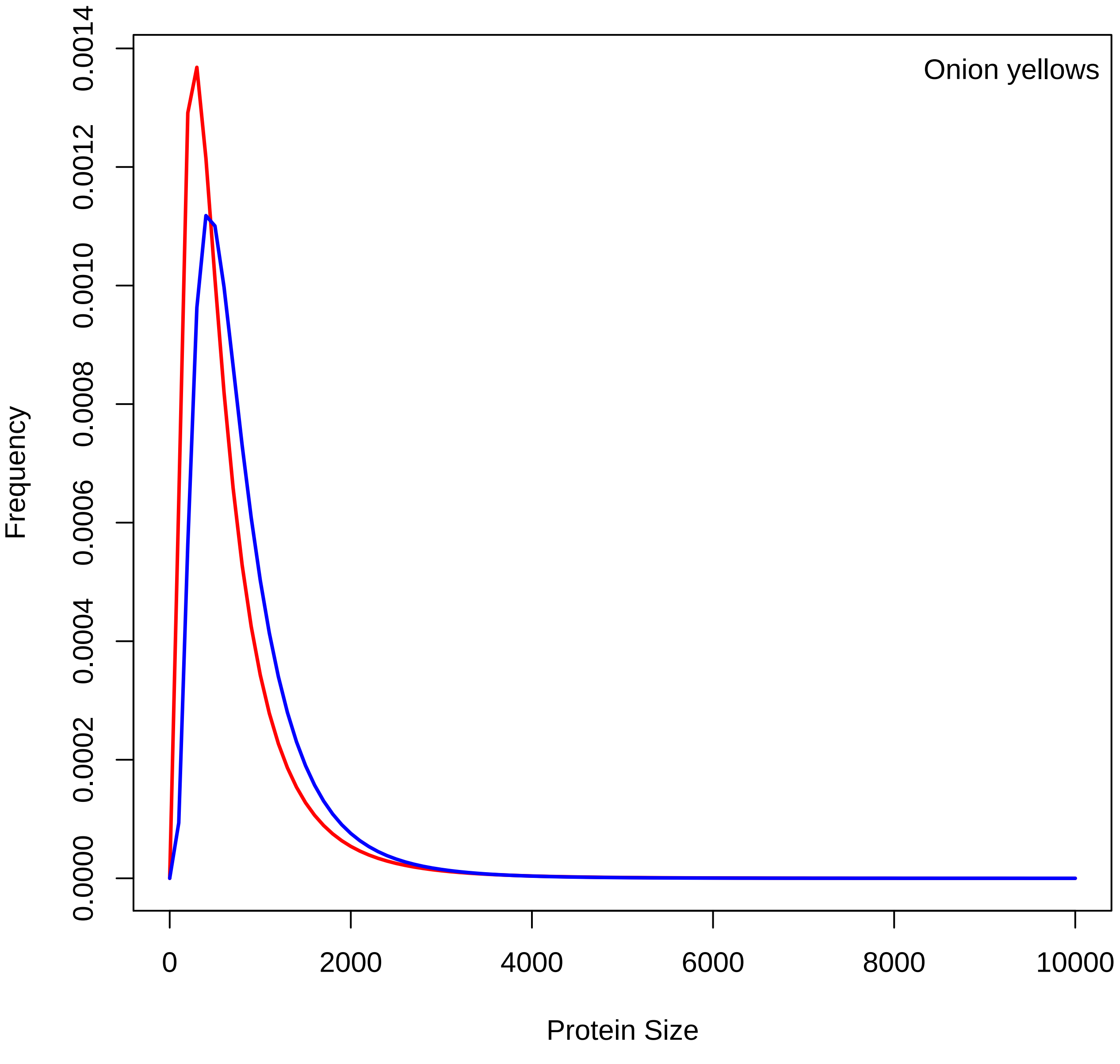

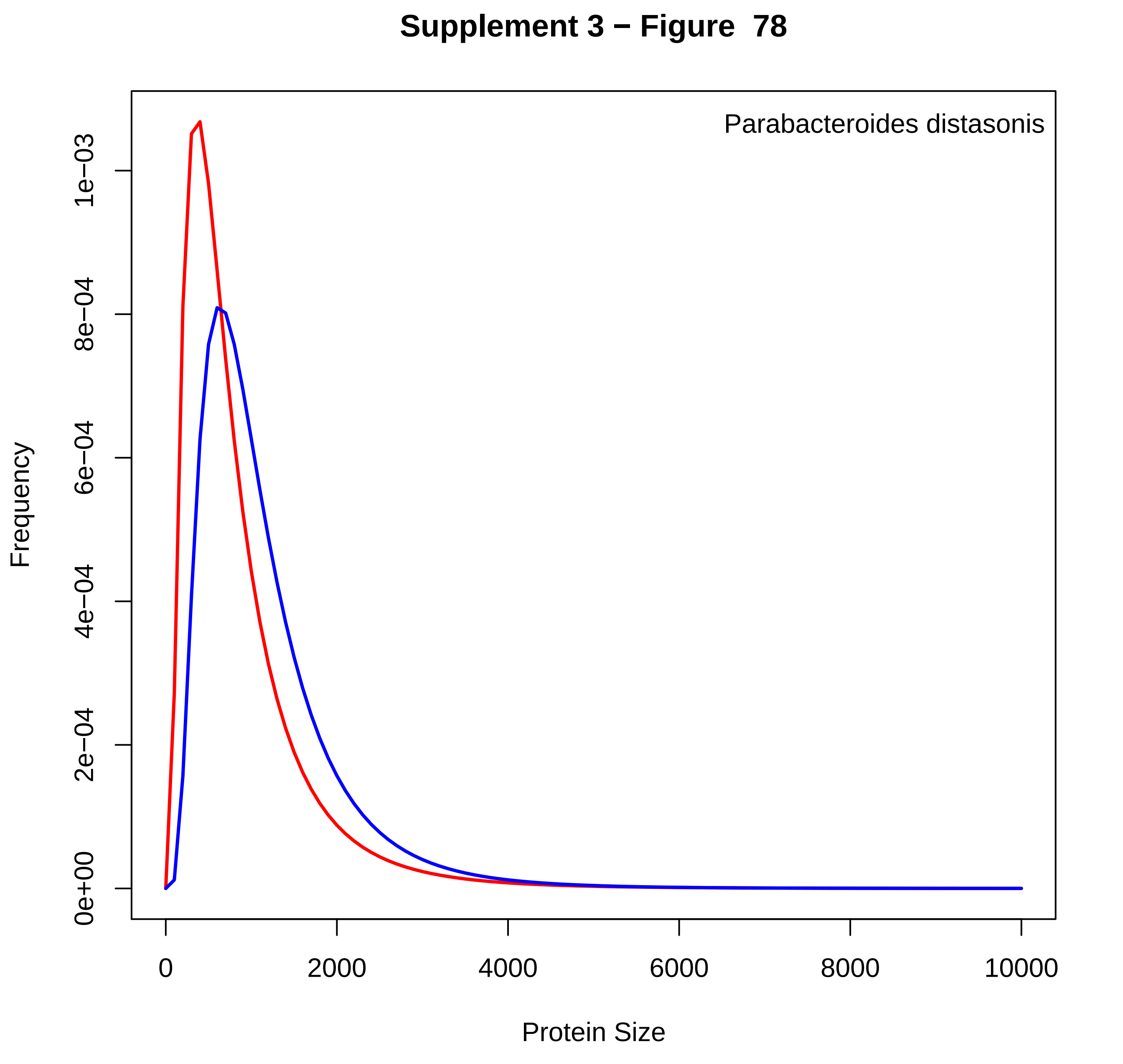
Supplement 3 − Figure 78
Parabacteroides distasonis
Frequency
0e+00
2e−04
4e−04
6e−04
8e−04
1e−03
0
2000
4000
6000
8000
10000
Protein Size

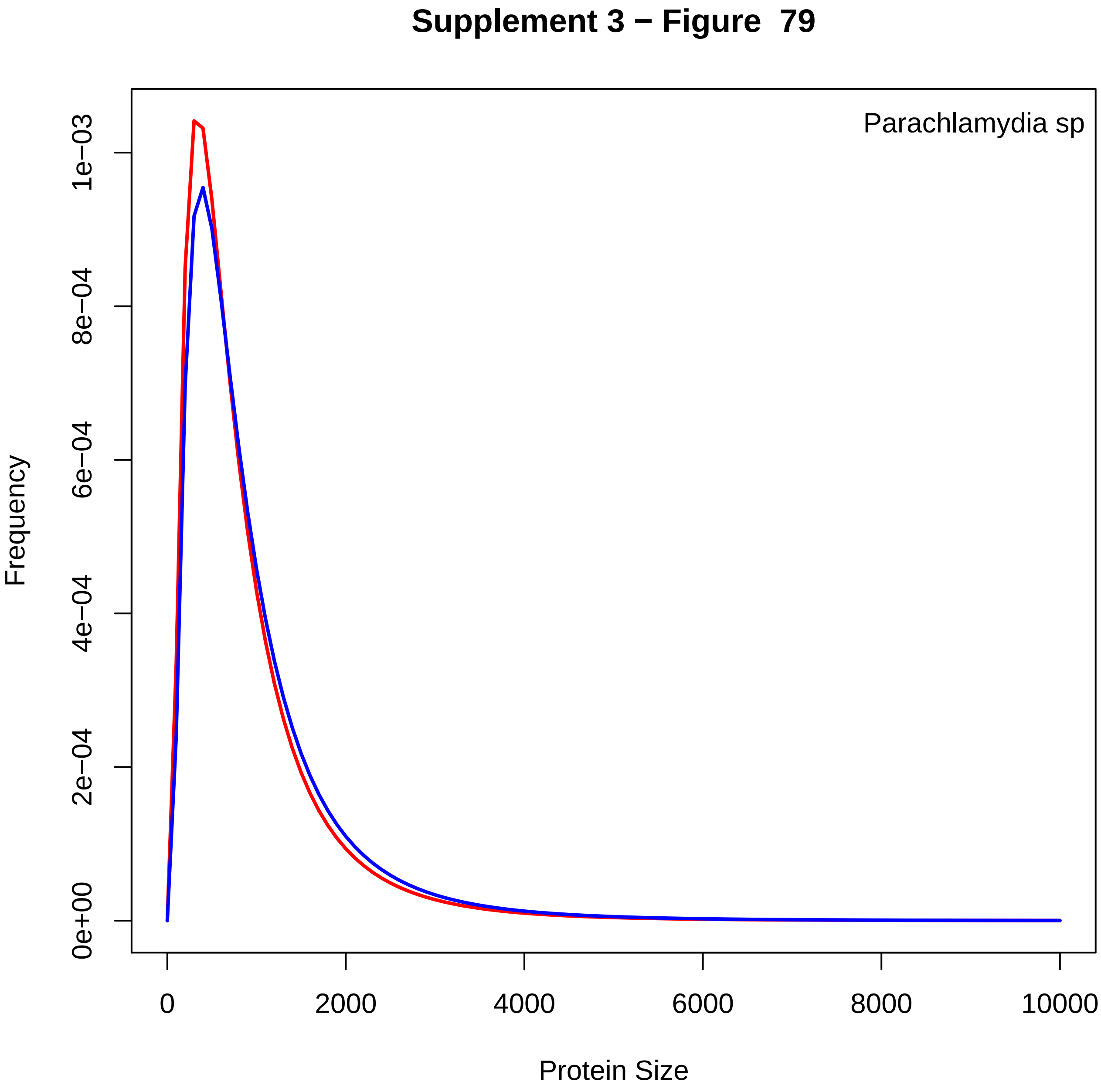
Supplement 3 − Figure 79
Parachlamydia sp
Frequency
0e+00
2e−04
4e−04
6e−04
8e−04
1e−03
0
2000
4000
6000
8000
10000
Protein Size

Supplement 3 − Figure 80

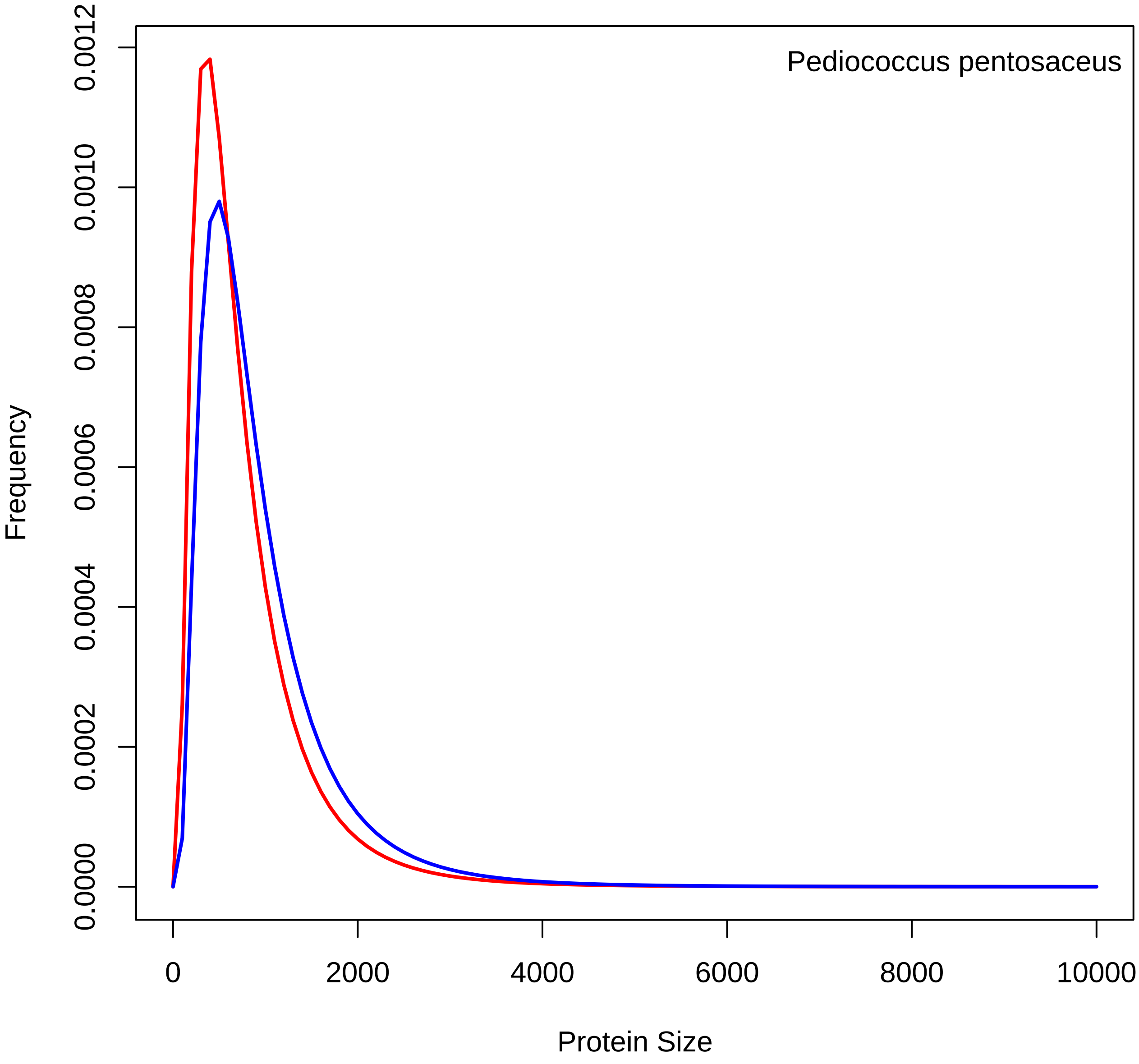

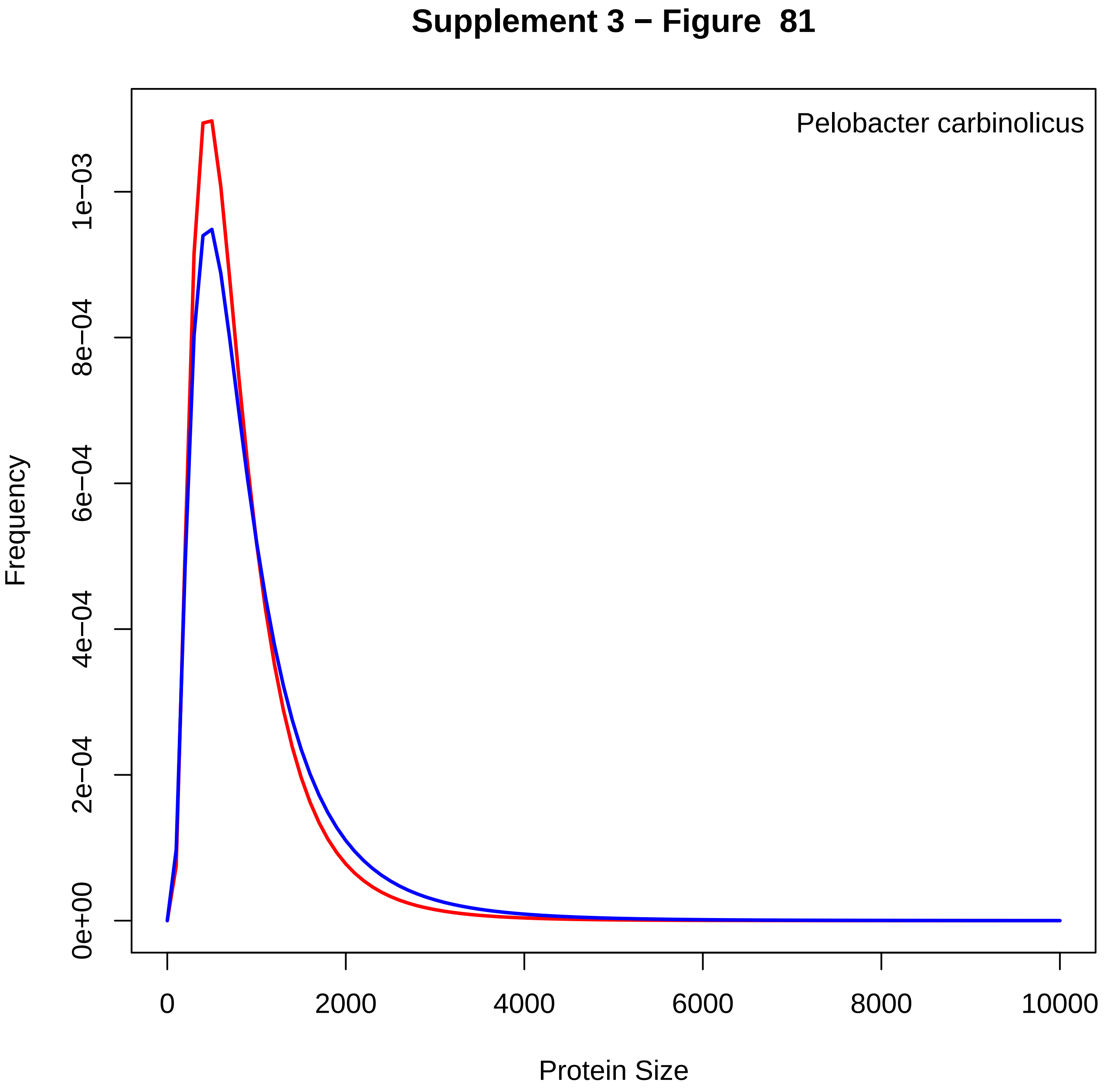
Supplement 3 − Figure 81
Pelobacter carbinolicus
Frequency
0e+00
2e−04
4e−04
6e−04
8e−04
1e−03
0
2000
4000
6000
8000
10000
Protein Size

Supplement 3 − Figure 82

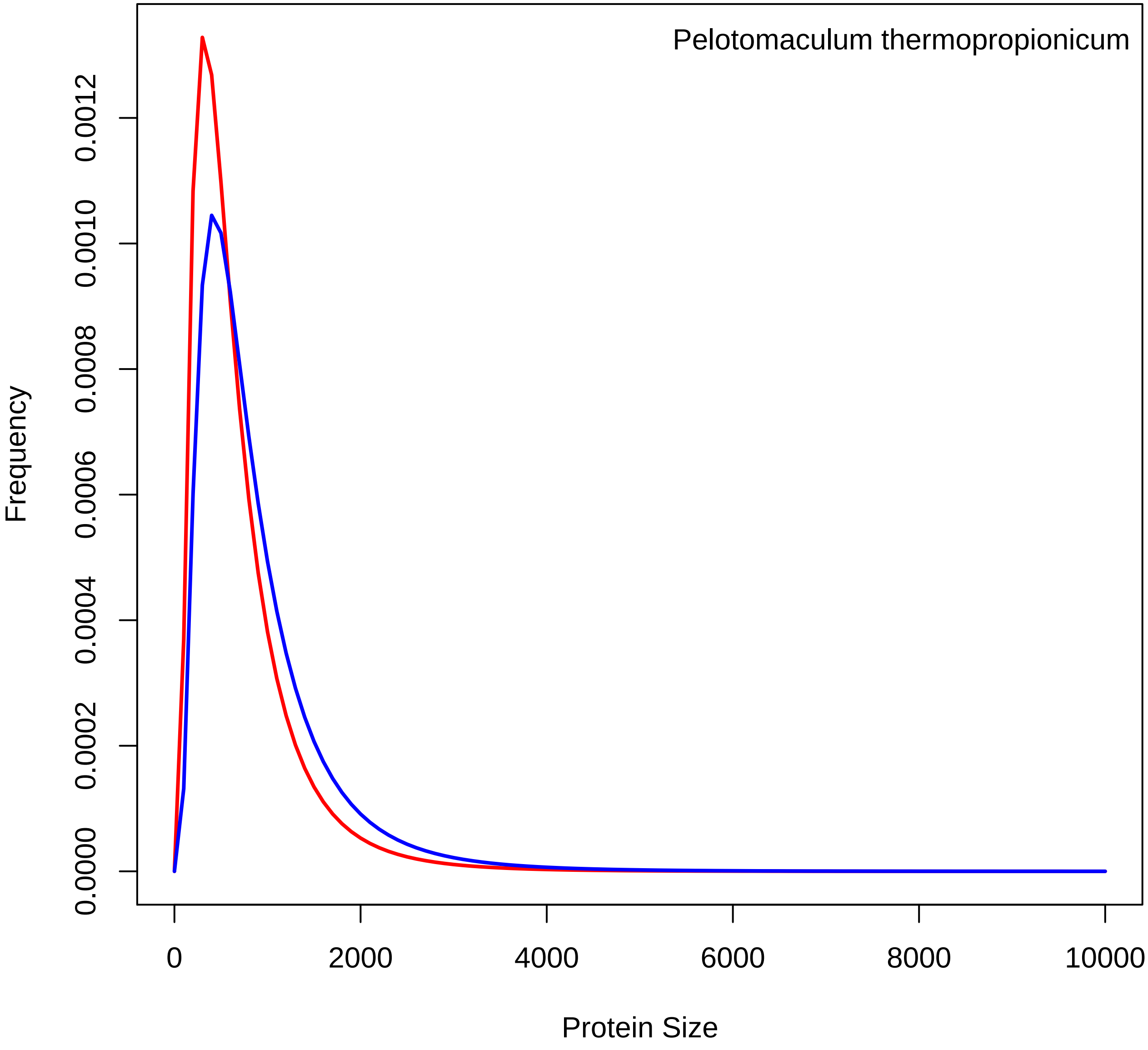

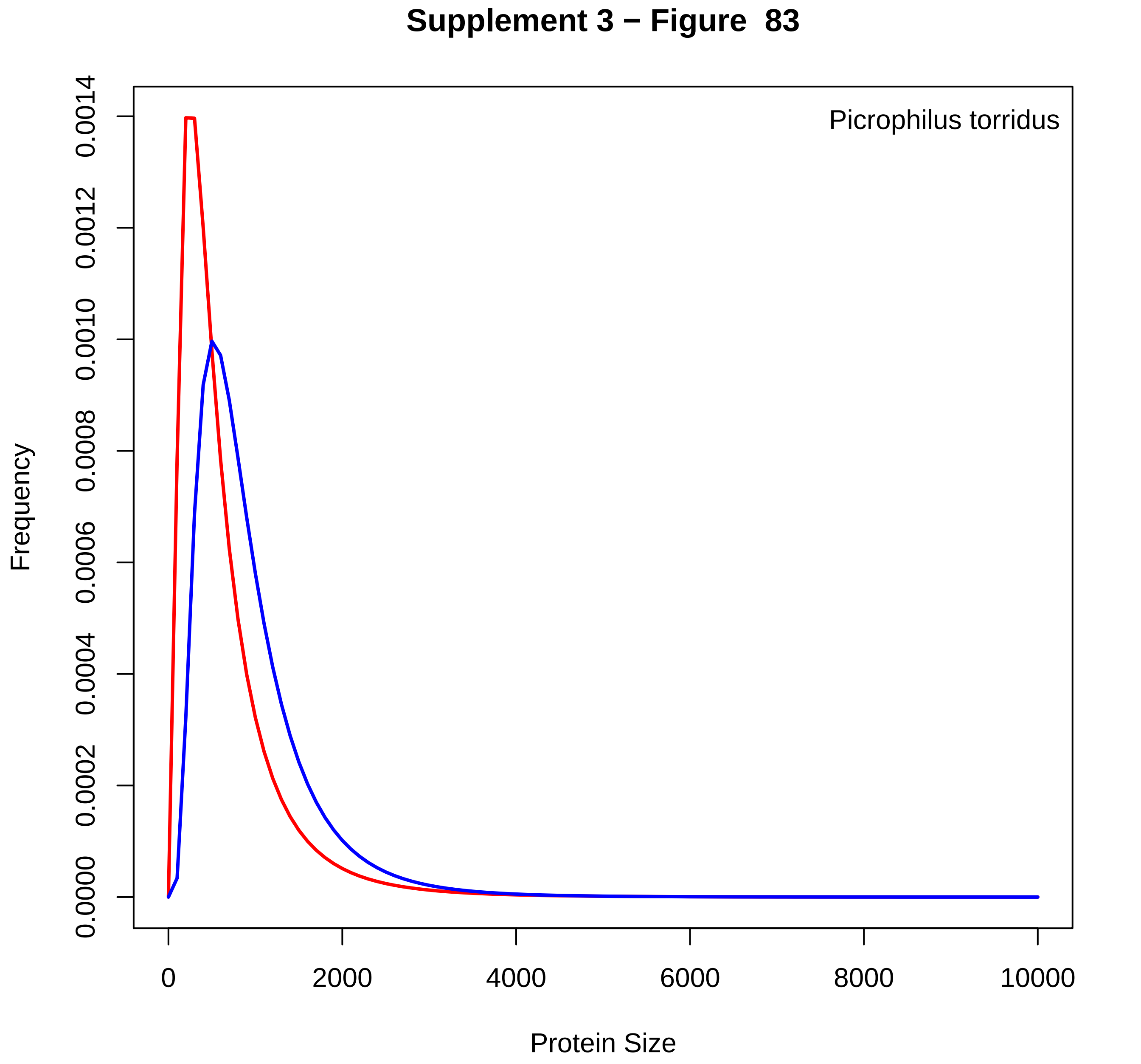

Supplement 3 − Figure 83
Picrophilus torridus
Frequency
0.0000
0.0002
0.0004
0.0006
0.0008
0.0010
0.0012
0.0014
0
2000
4000
6000
8000
10000
Protein Size

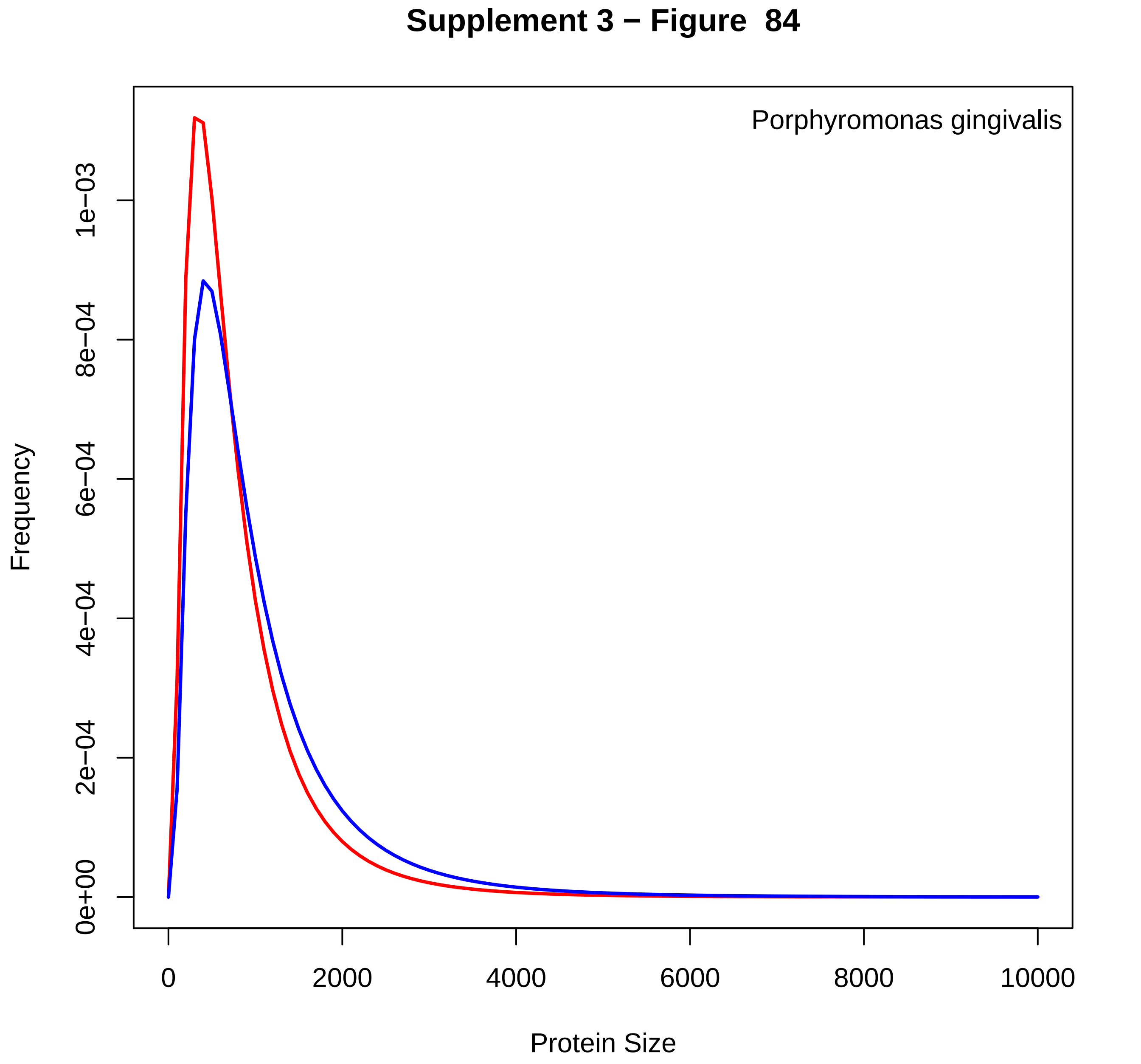
Supplement 3 − Figure 84
Porphyromonas gingivalis
Frequency
0e+00
2e−04
4e−04
6e−04
8e−04
1e−03
0
2000
4000
6000
8000
10000
Protein Size

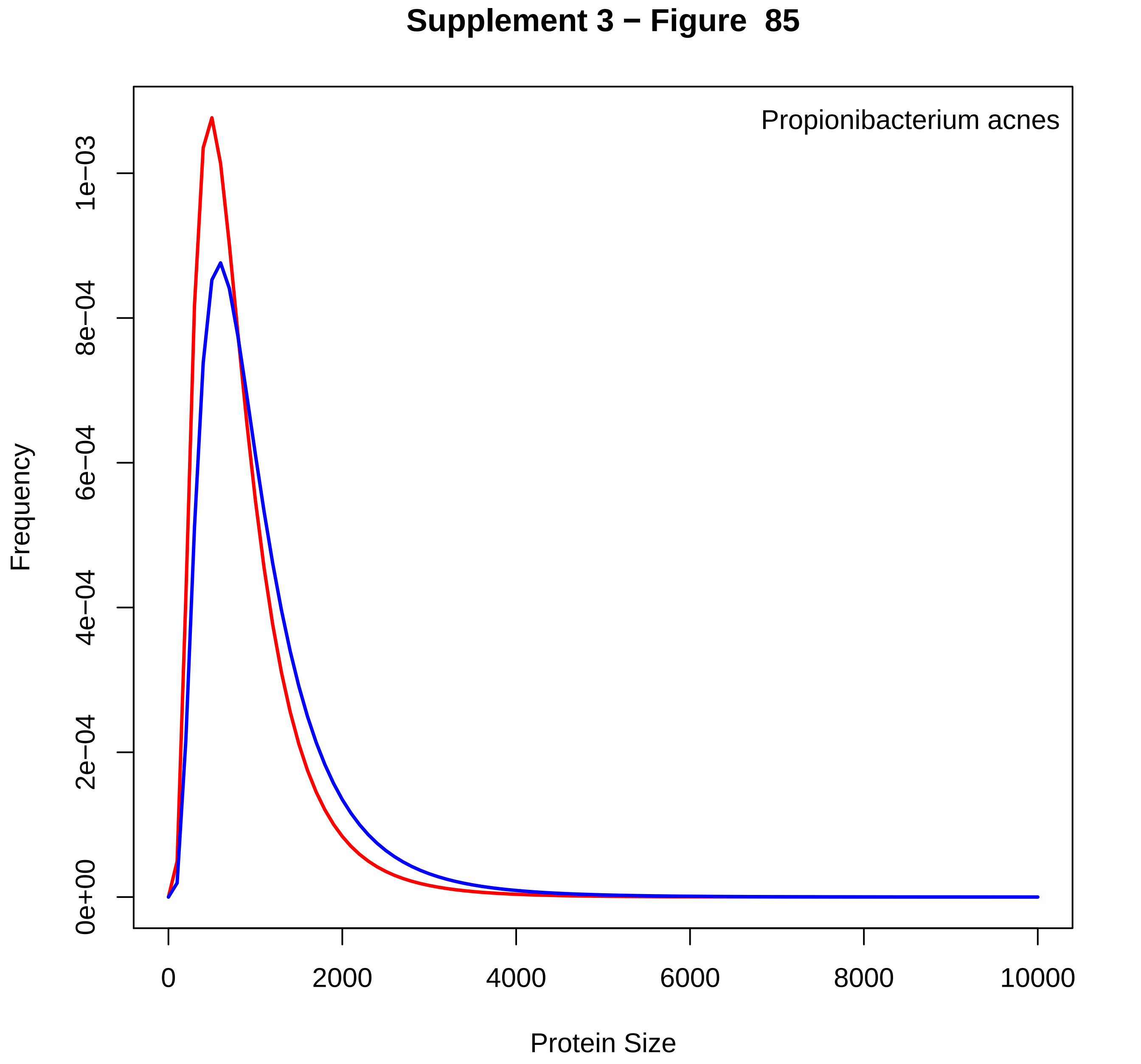

Supplement 3 − Figure 85
Propionibacterium acnes
Frequency
0e+00
2e−04
4e−04
6e−04
8e−04
1e−03
0
2000
4000
6000
8000
10000
Protein Size

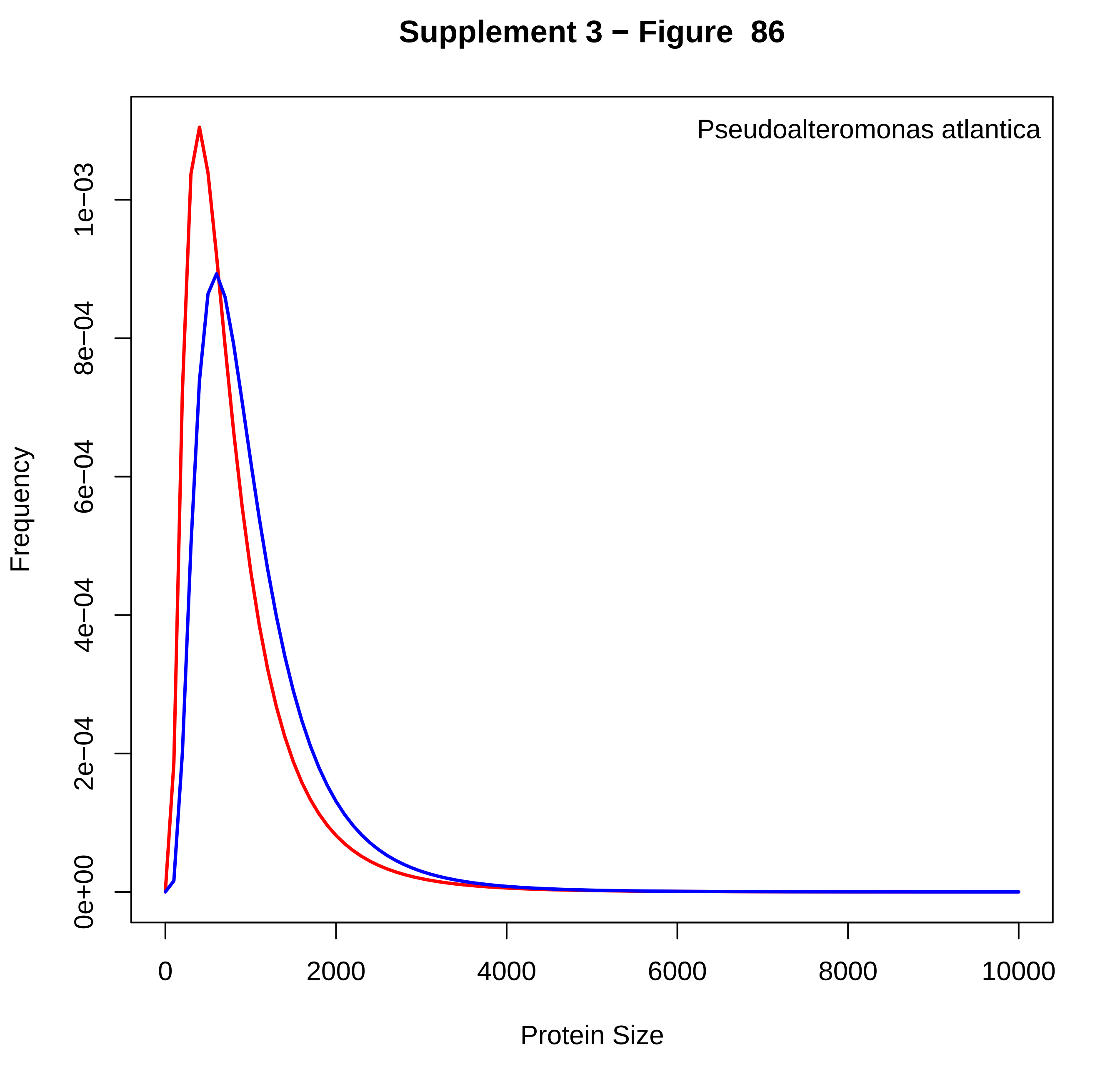

Supplement 3 − Figure 86
Pseudoalteromonas atlantica
Frequency
0e+00
2e−04
4e−04
6e−04
8e−04
1e−03
0
2000
4000
6000
8000
10000
Protein Size

Supplement 3 − Figure 87

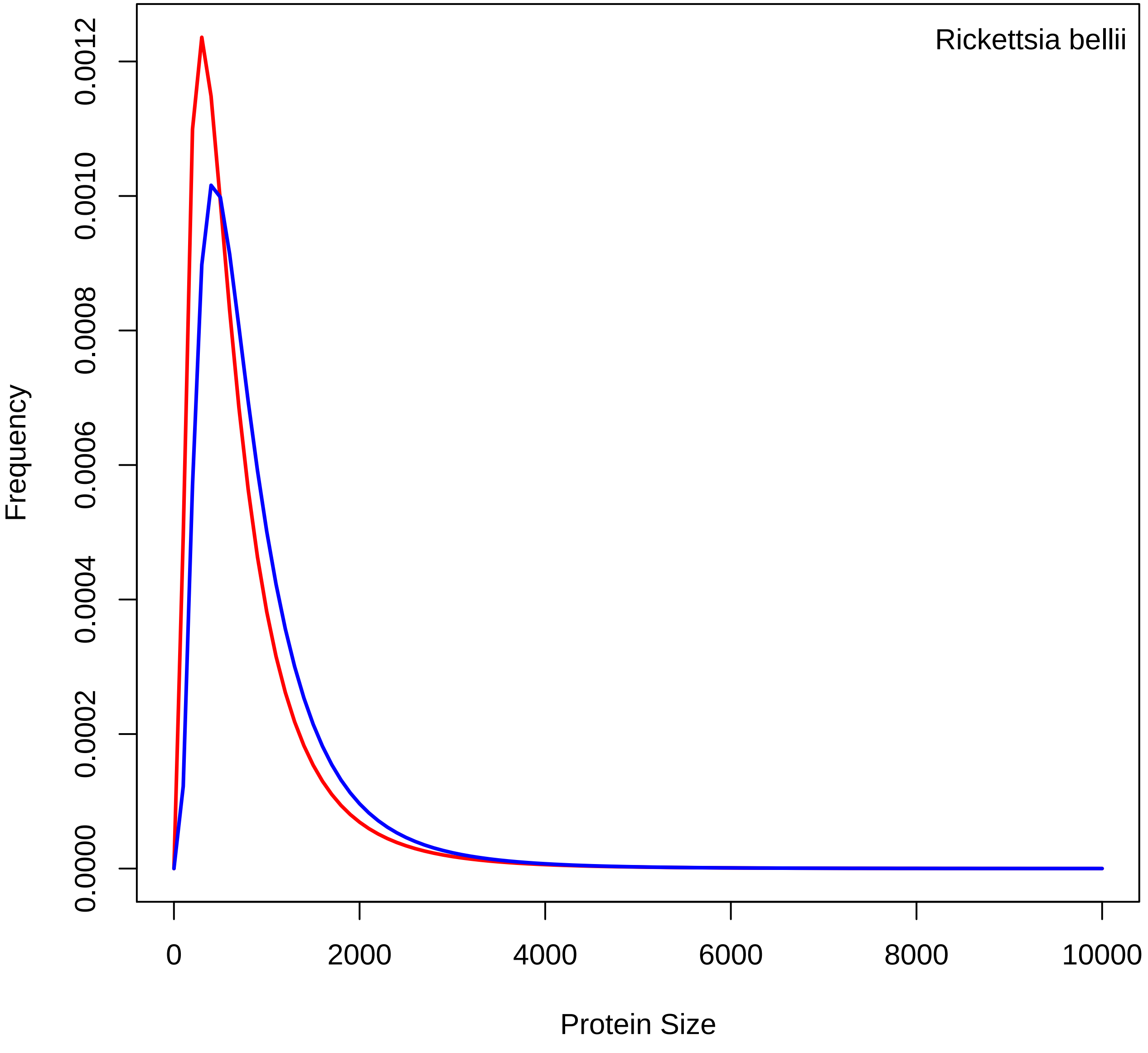

Supplement 3 − Figure 88

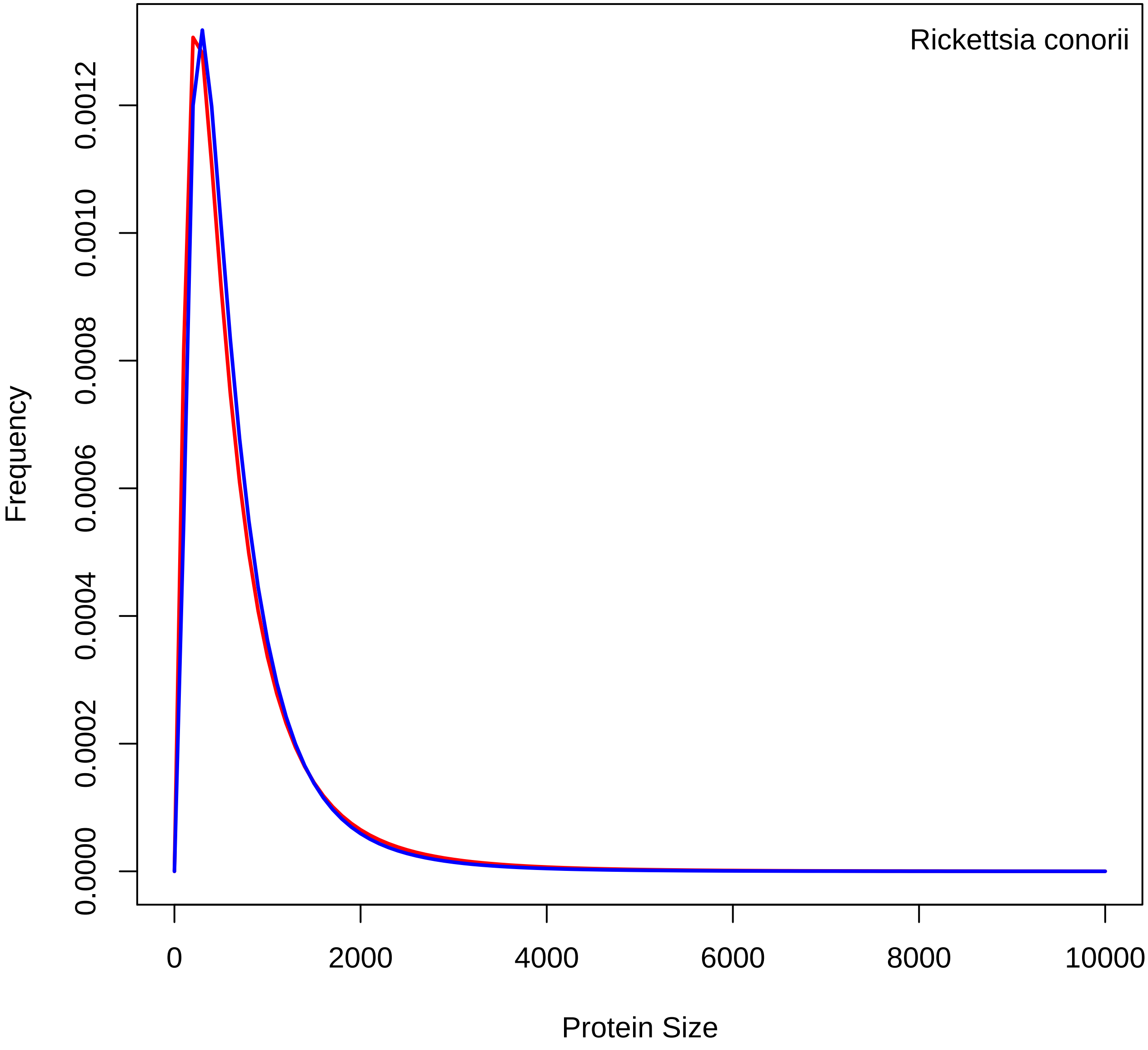

Supplement 3 − Figure 89

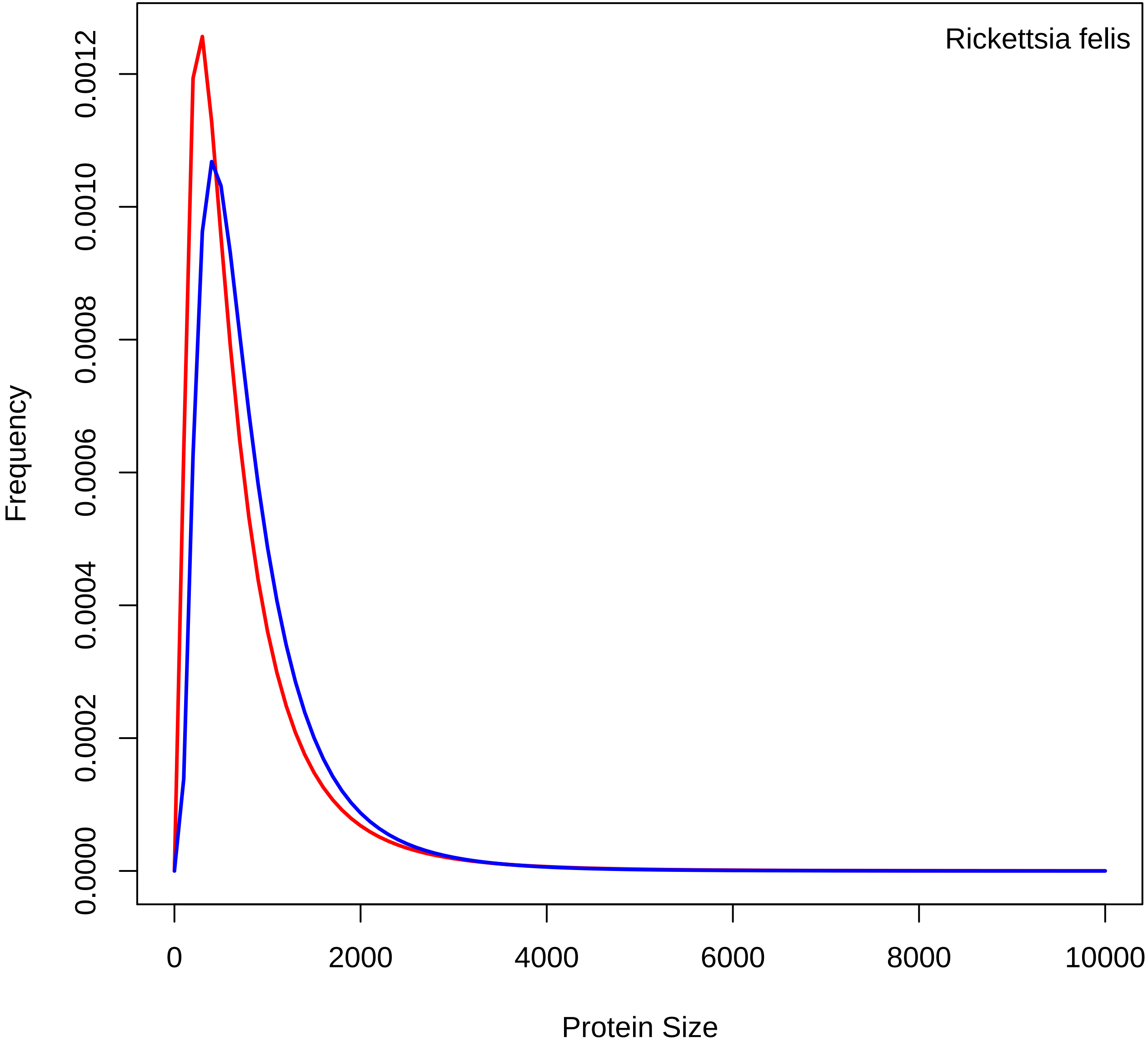

Supplement 3 − Figure 90

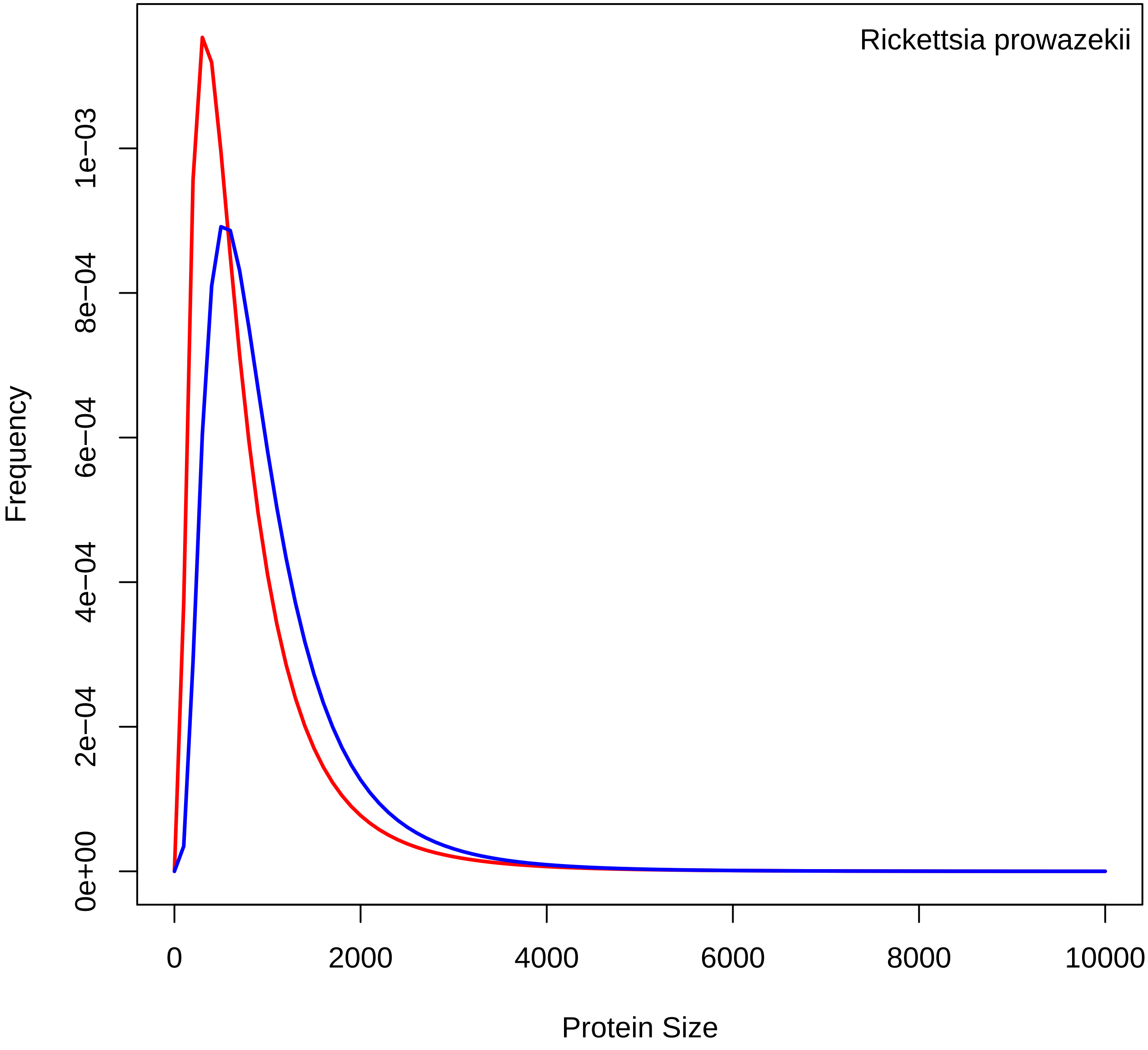

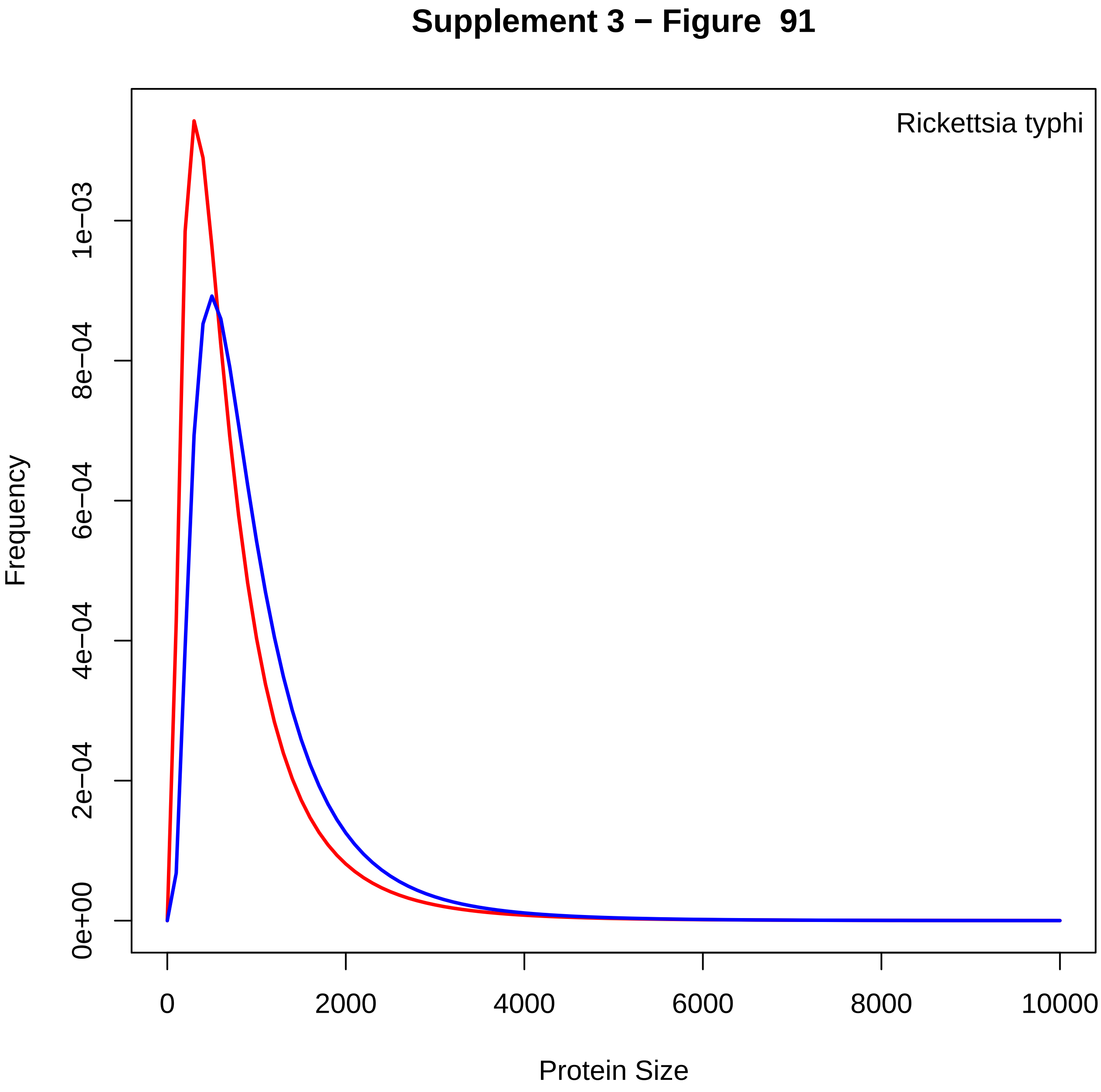
Supplement 3 − Figure 91
Rickettsia typhi
Frequency
0e+00
2e−04
4e−04
6e−04
8e−04
1e−03
0
2000
4000
6000
8000
10000
Protein Size

Supplement 3 − Figure 92

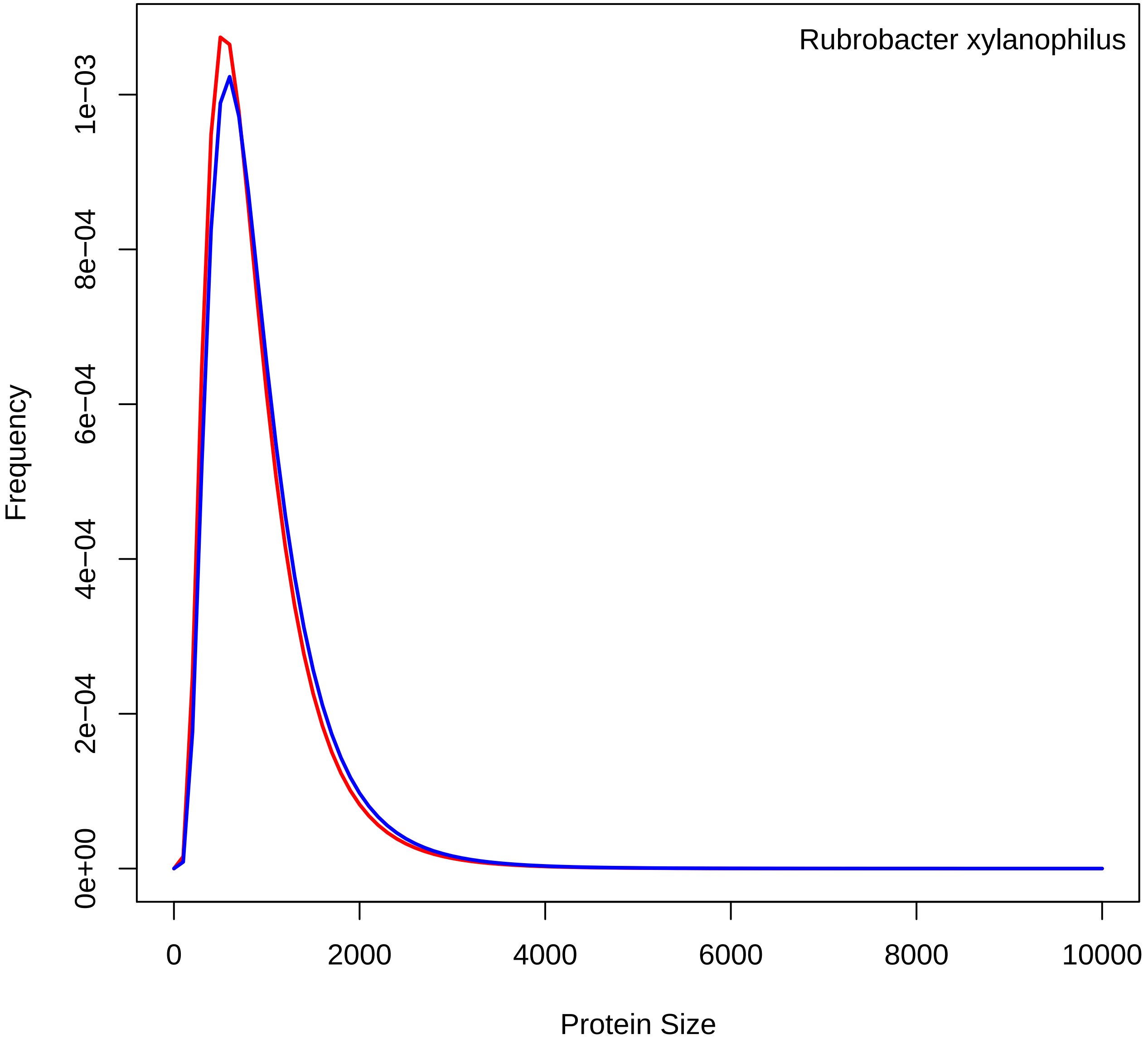

Supplement 3 − Figure 93

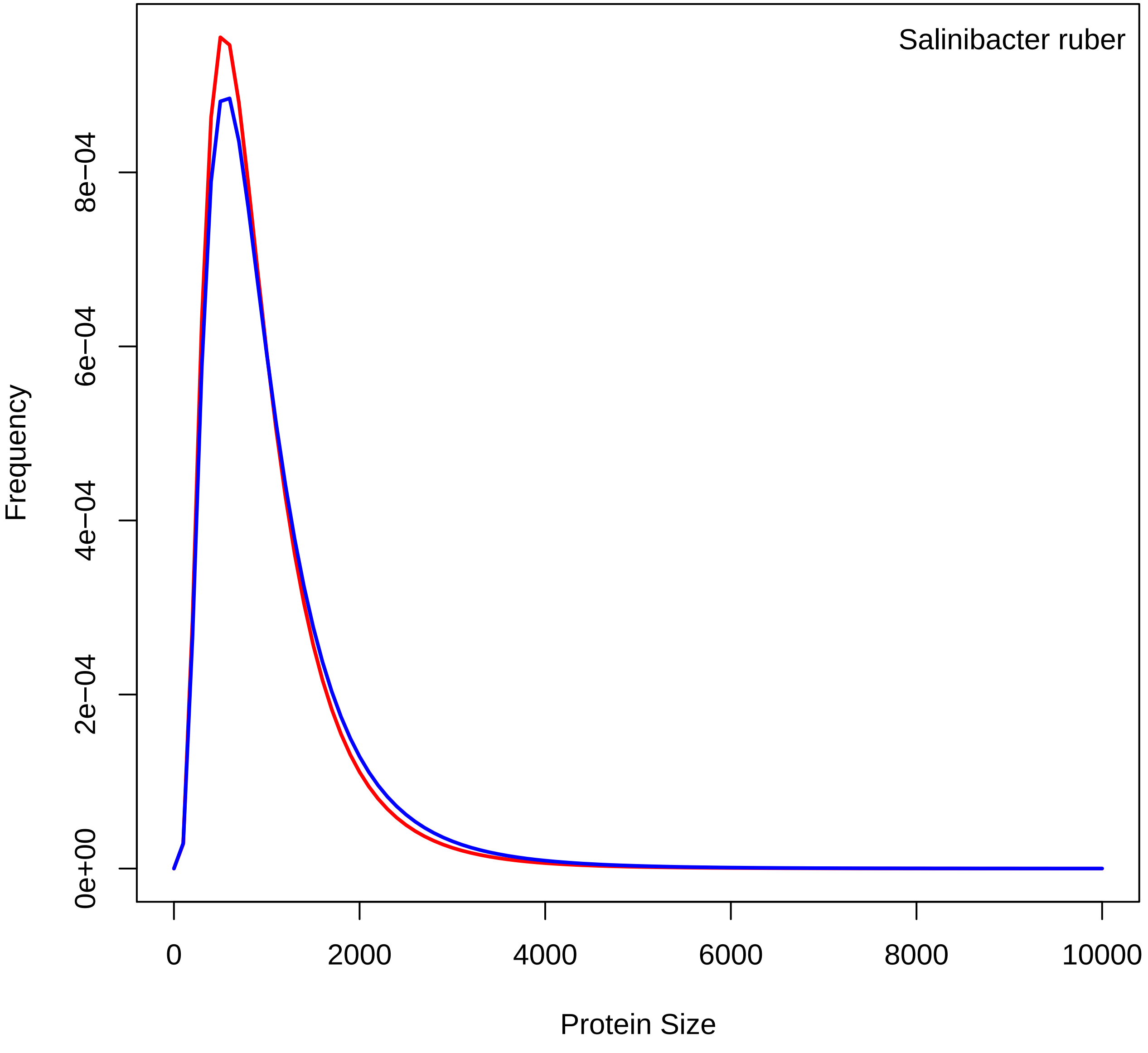

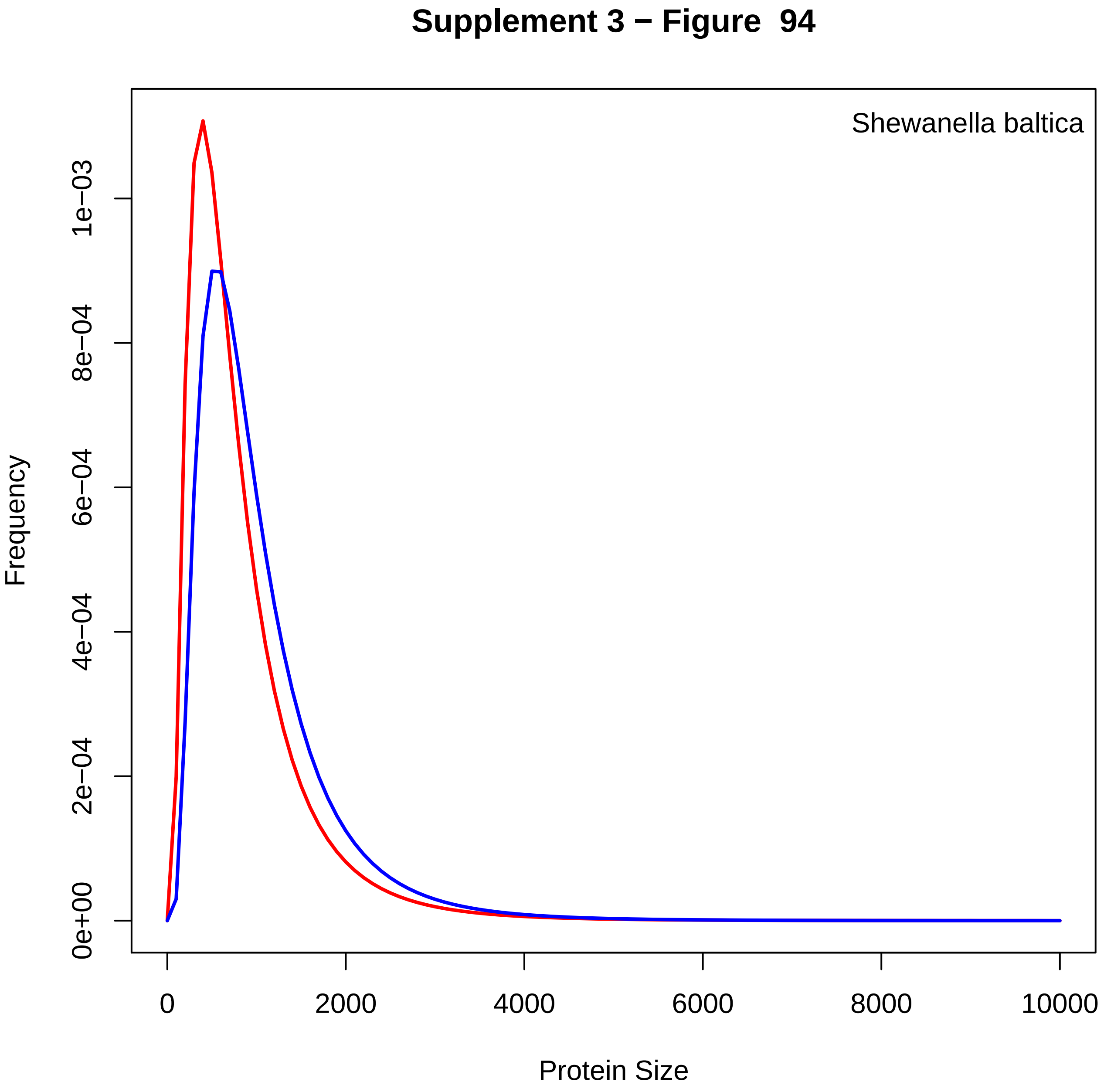
Supplement 3 − Figure 94
Shewanella baltica
Frequency
0e+00
2e−04
4e−04
6e−04
8e−04
1e−03
0
2000
4000
6000
8000
10000
Protein Size

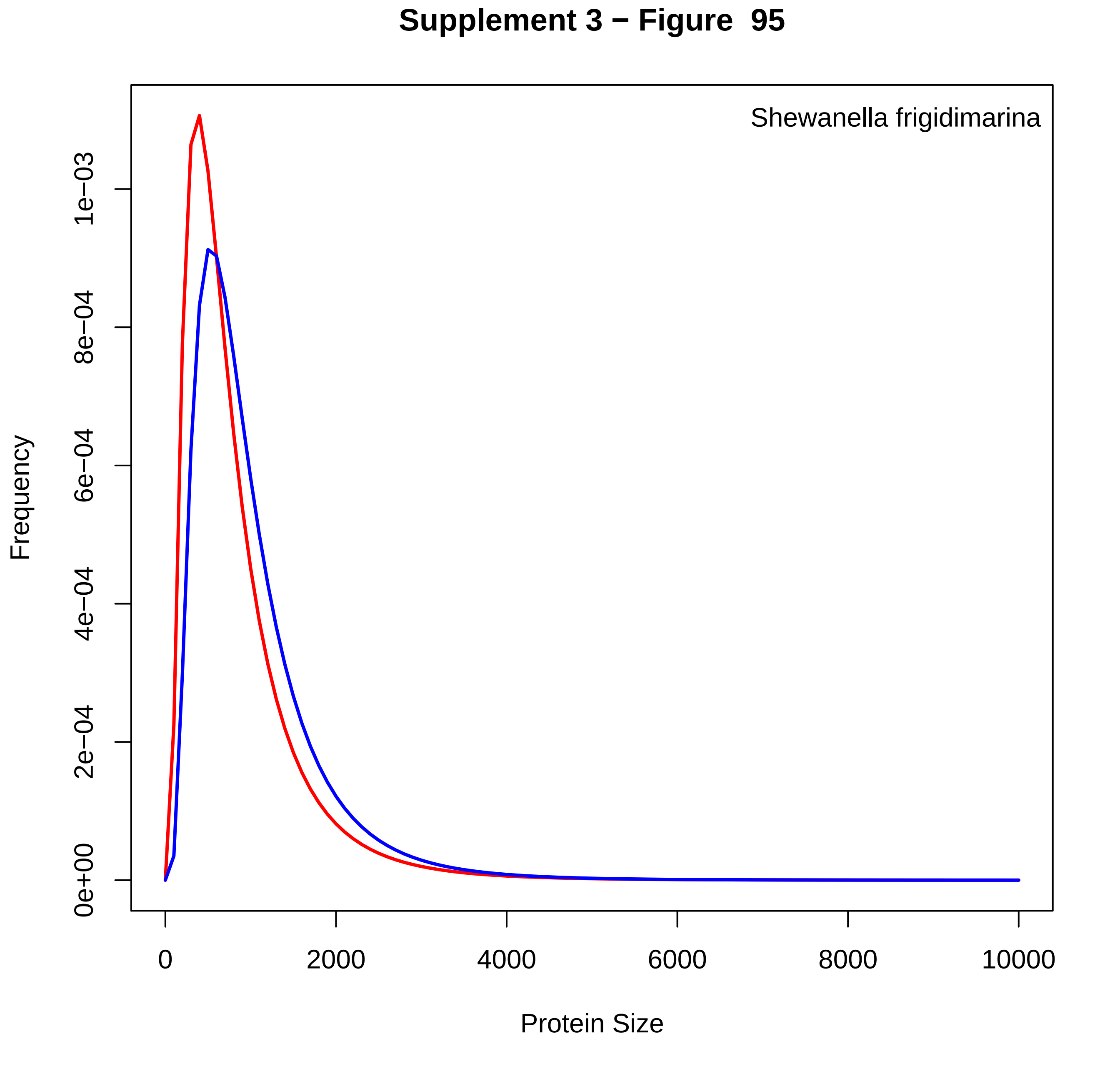
Supplement 3 − Figure 95
Shewanella frigidimarina
Frequency
0e+00
2e−04
4e−04
6e−04
8e−04
1e−03
0
2000
4000
6000
8000
10000
Protein Size

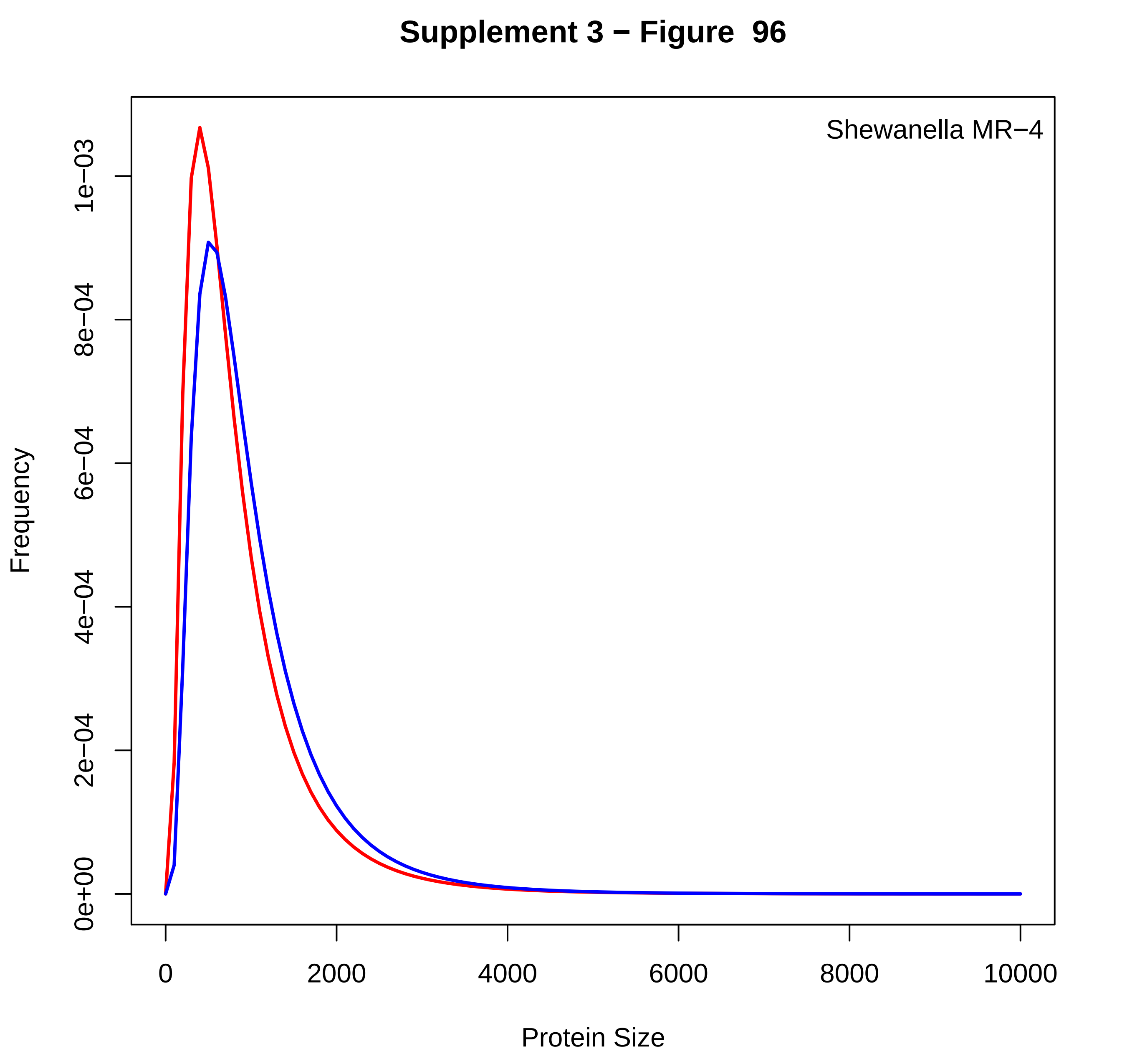
Supplement 3 − Figure 96
Shewanella MR−4
Frequency
Protein Size
0e+00
2e−04
4e−04
6e−04
8e−04
1e−03
0
2000
4000
6000
8000
10000

Supplement 3 − Figure 97

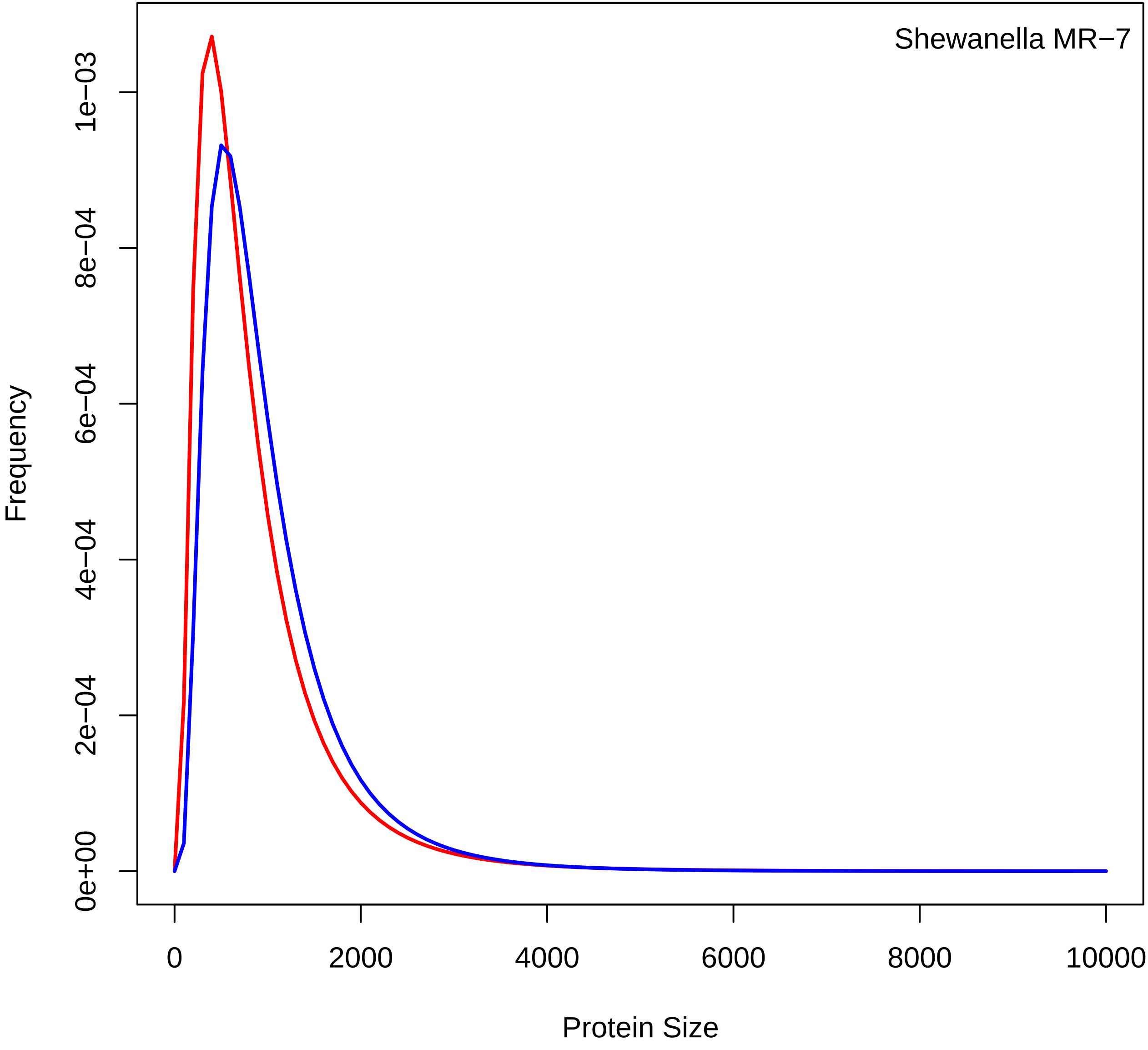

Supplement 3 − Figure 98

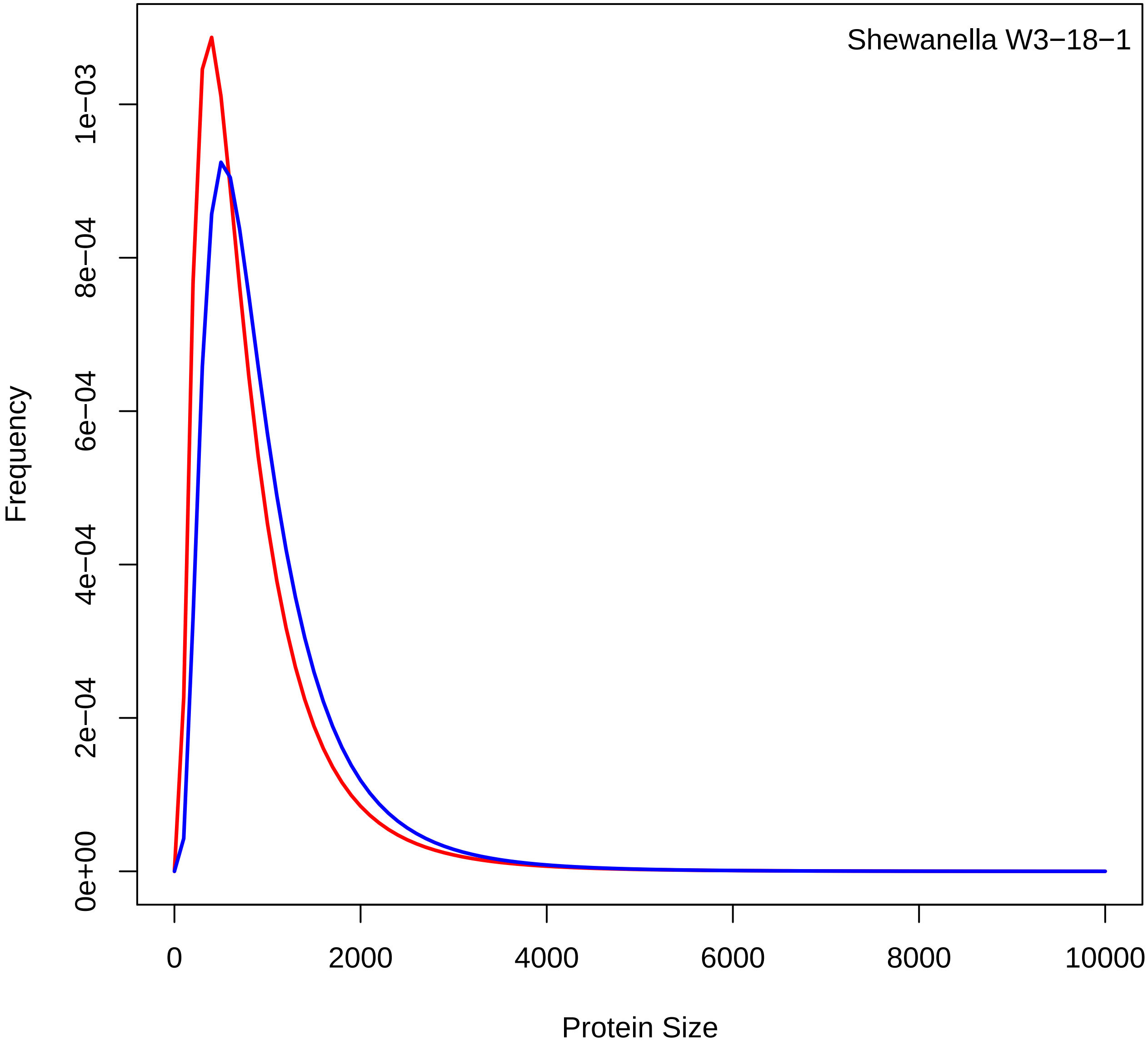

Supplement 3 − Figure 99

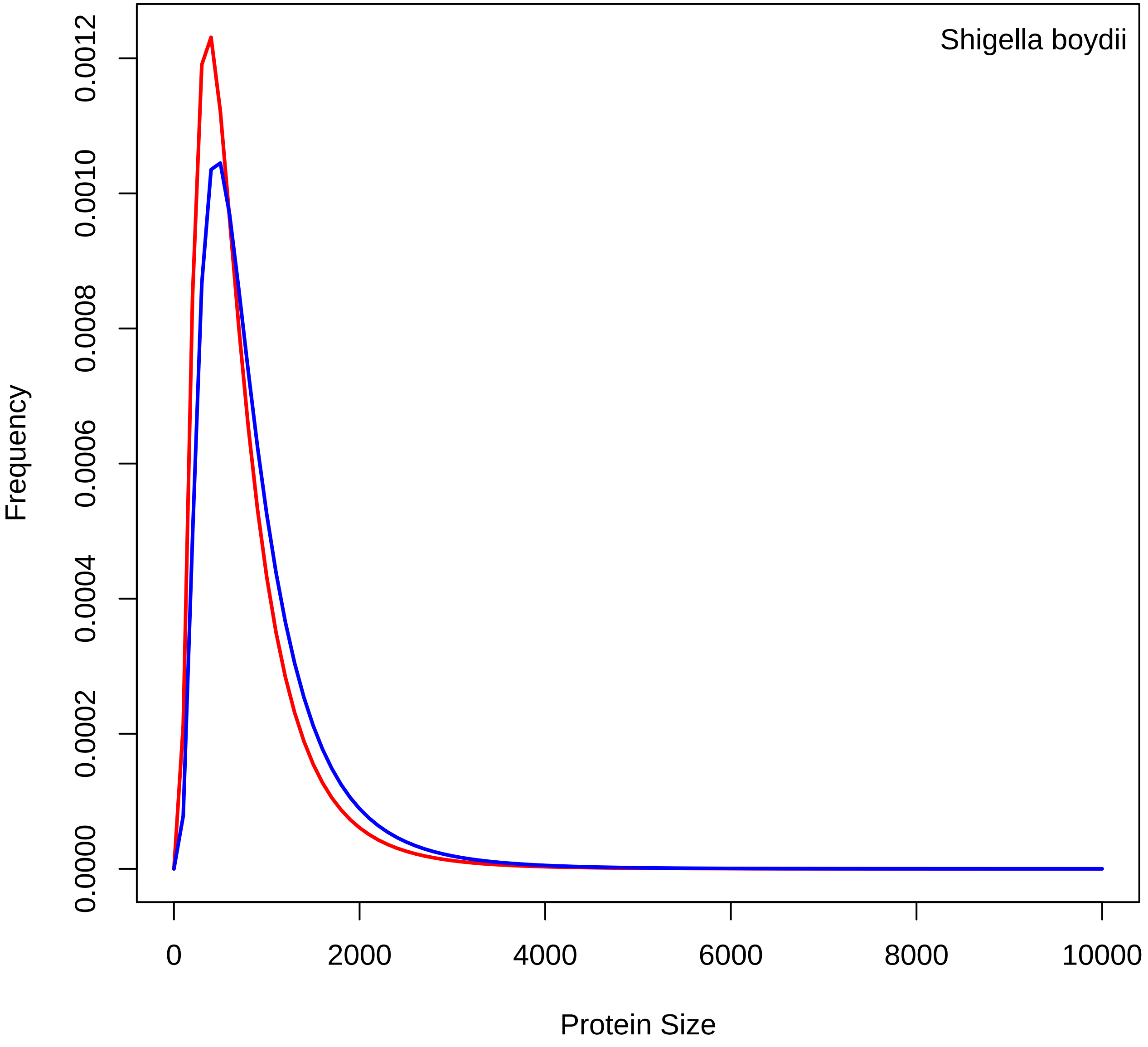

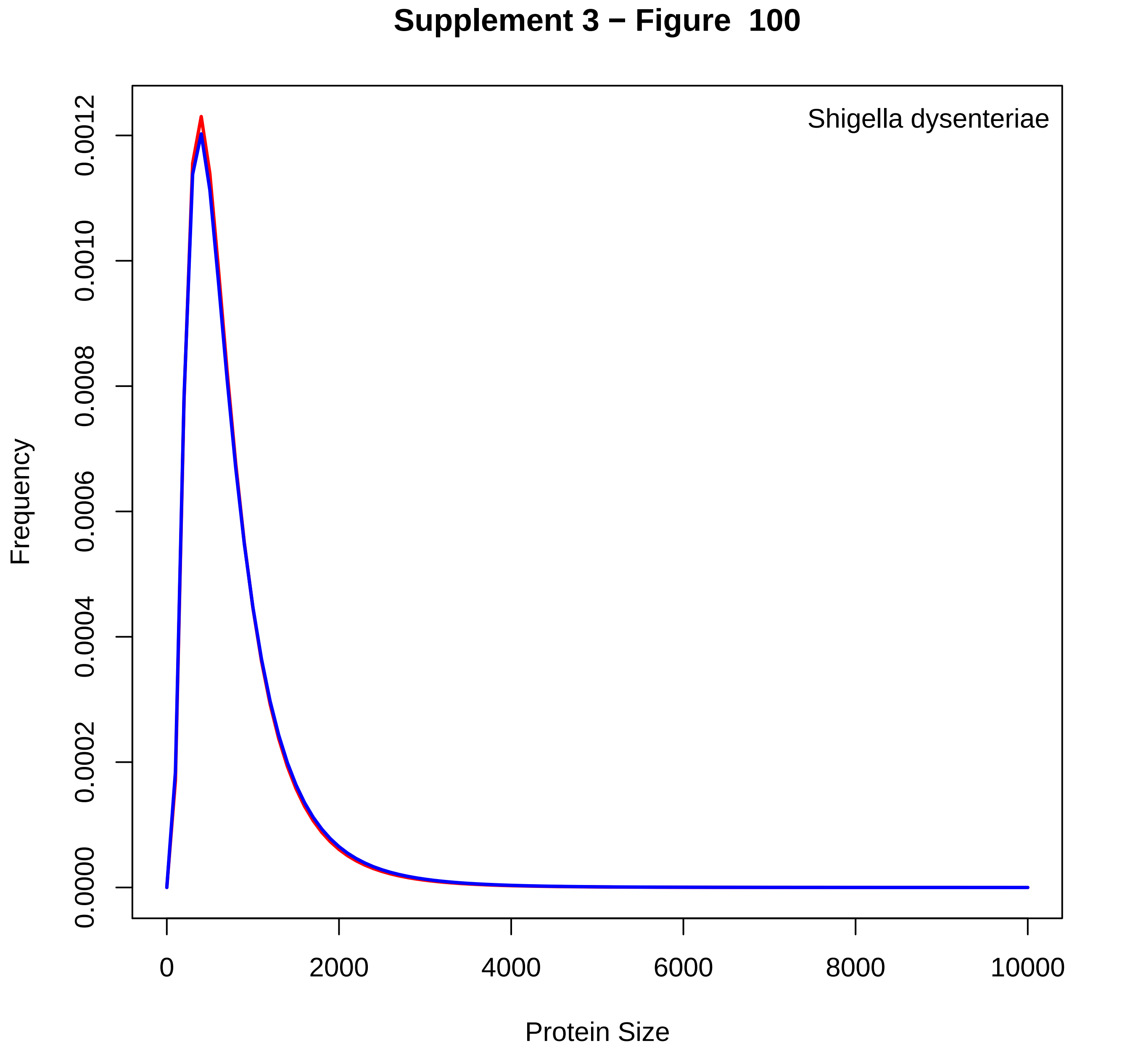
Supplement 3 − Figure 100
Shigella dysenteriae
Frequency
0.0000
0.0002
0.0004
0.0006
0.0008
0.0010
0.0012
0
2000
4000
6000
8000
10000
Protein Size

Supplement 3 − Figure 101

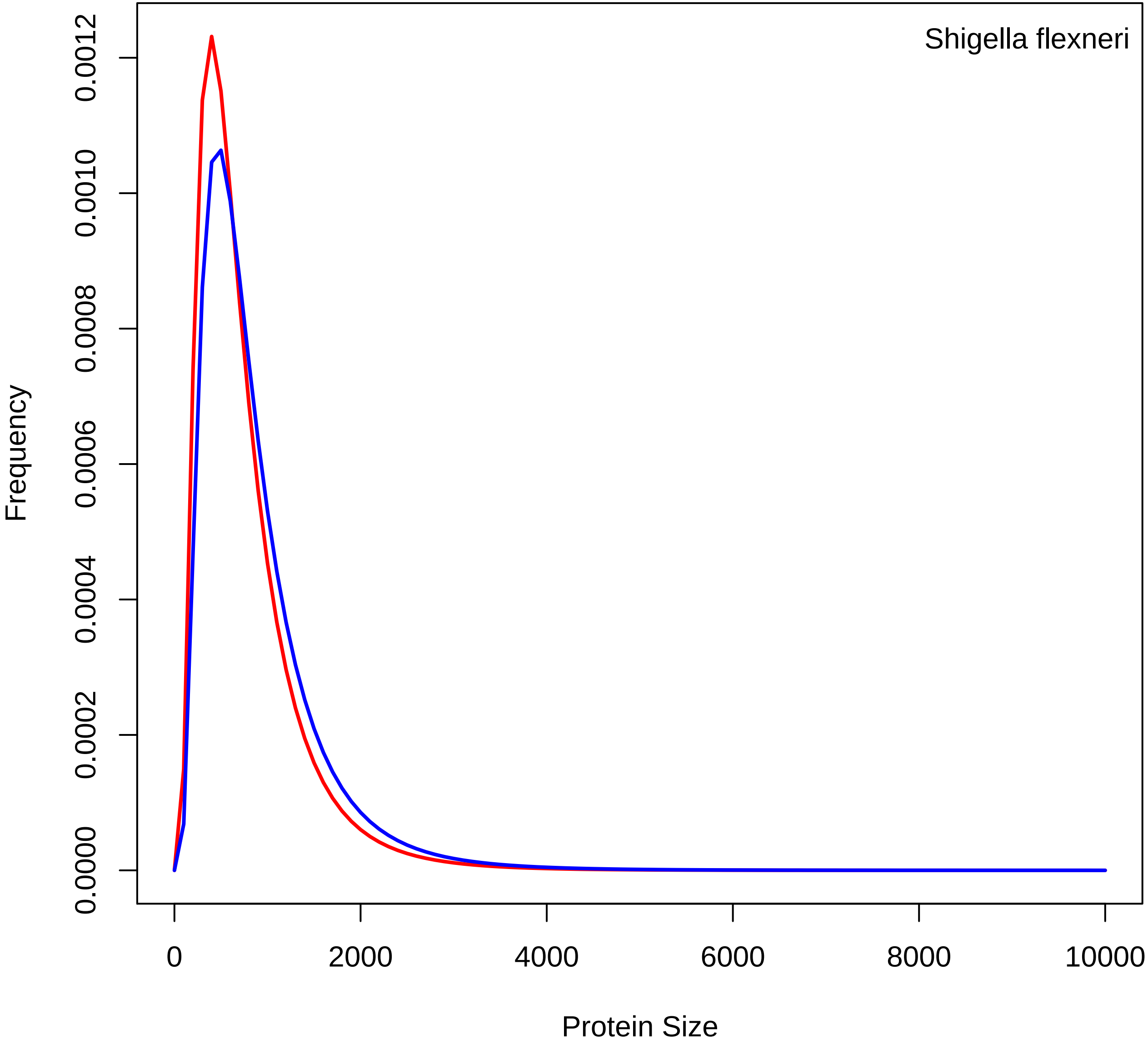

Supplement 3 − Figure 102

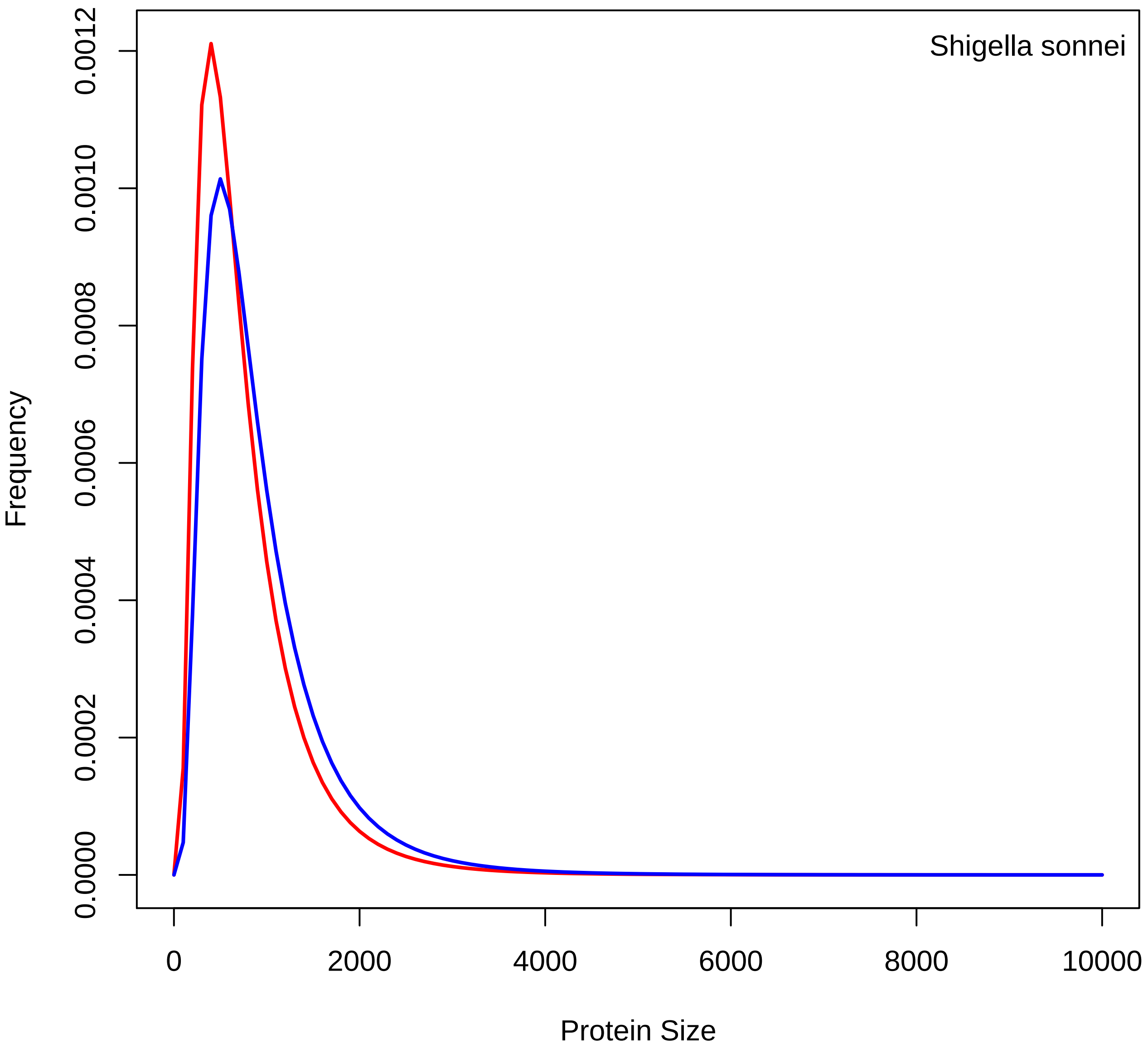

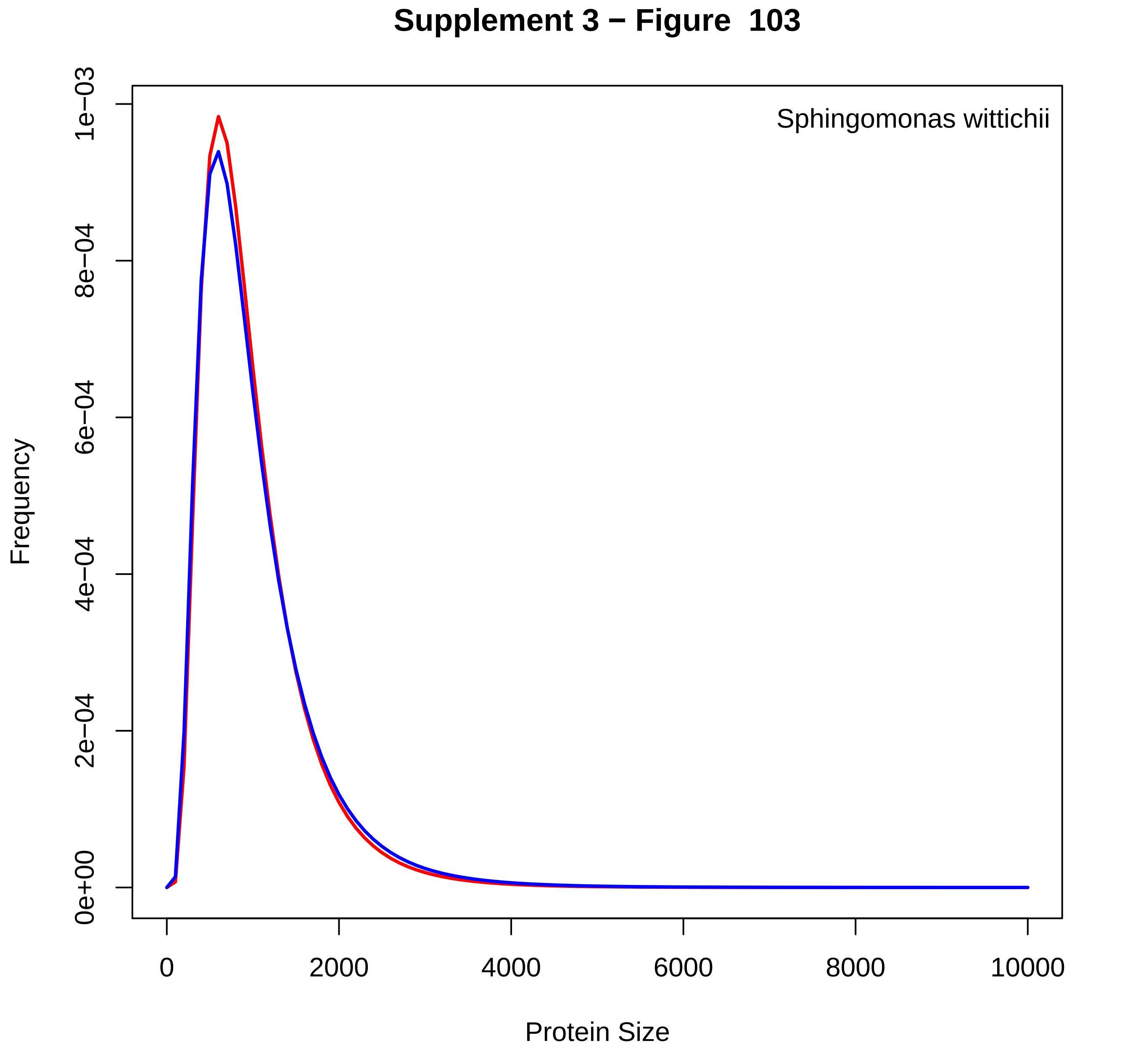

Supplement 3 − Figure 103
Sphingomonas wittichii
Frequency
1e−03
8e−04
6e−04
4e−04
2e−04
0e+00
0
2000
4000
6000
8000
10000
Protein Size

Supplement 3 − Figure 104

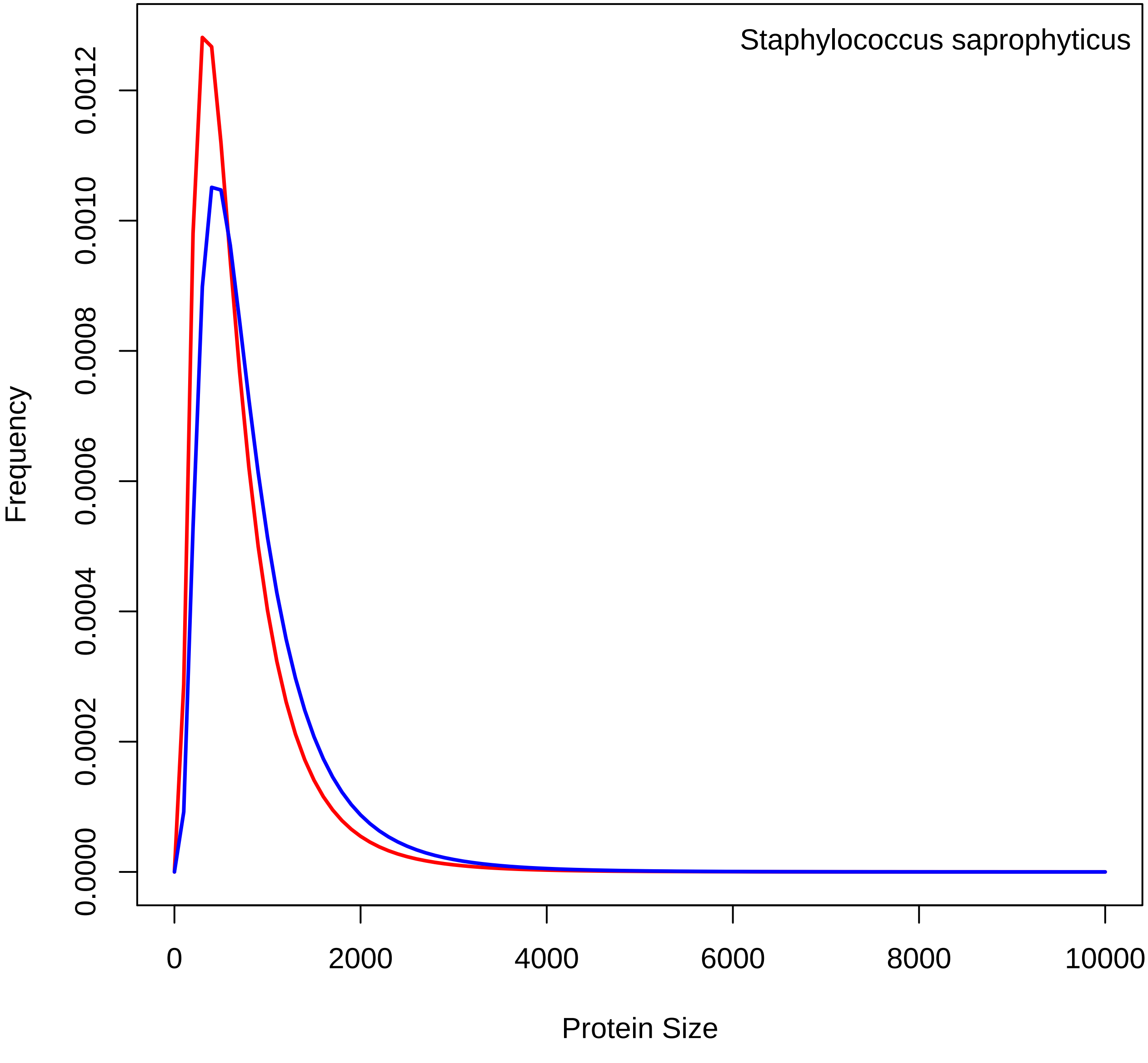

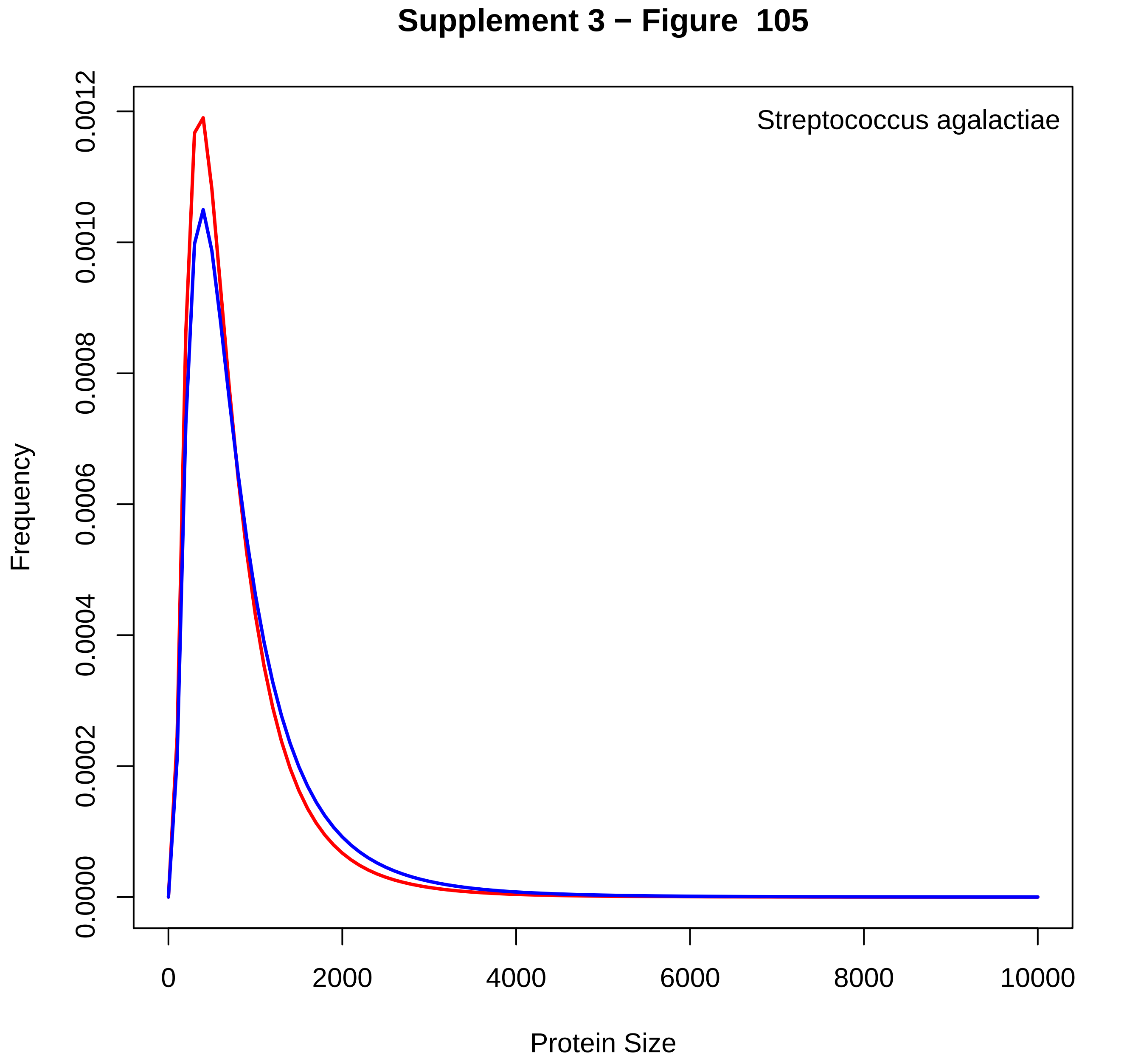
Supplement 3 − Figure 105
Streptococcus agalactiae
Frequency
0.0000
0.0002
0.0004
0.0006
0.0008
0.0010
0.0012
0
2000
4000
6000
8000
10000
Protein Size

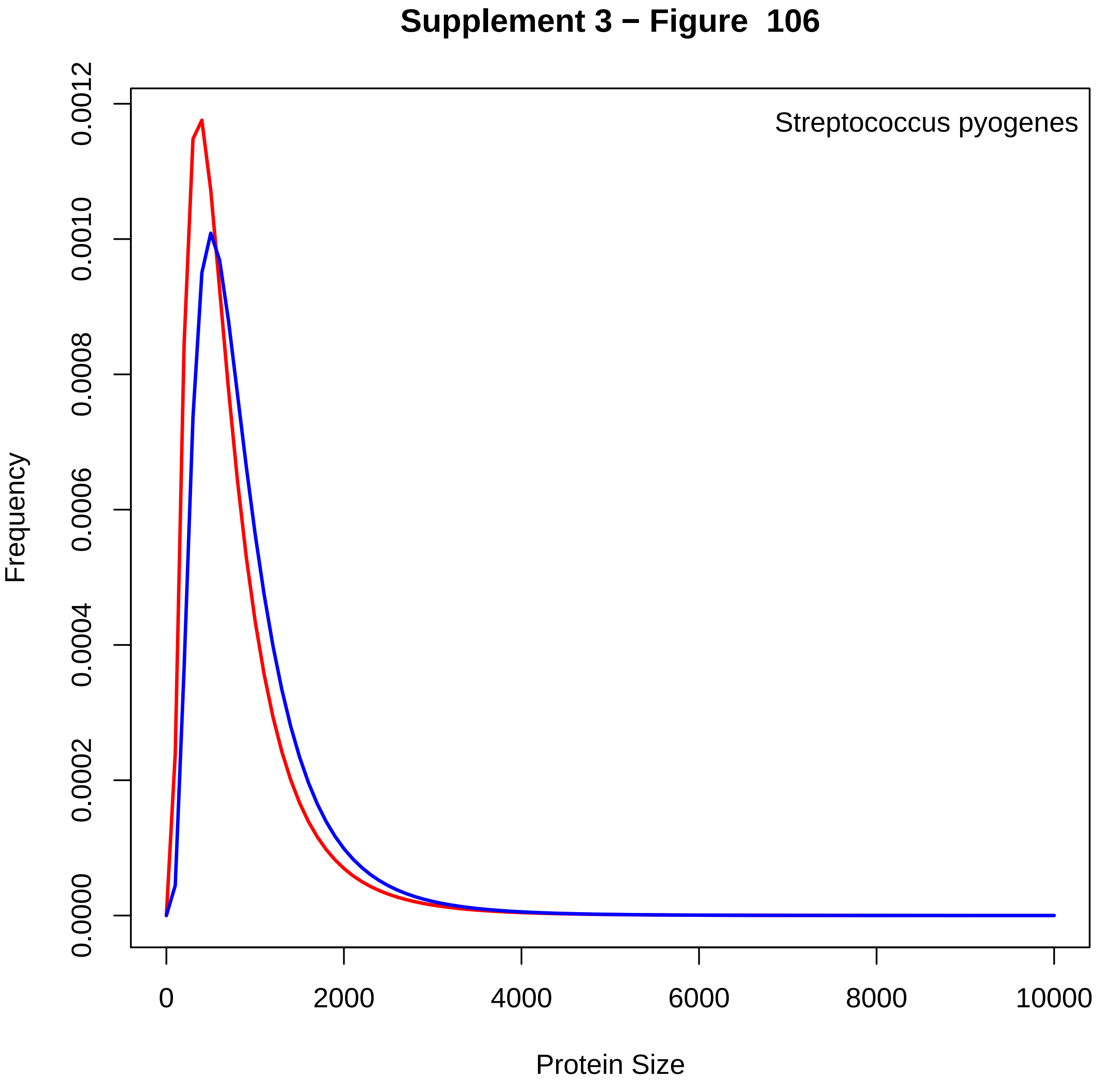
Supplement 3 − Figure 106
Streptococcus pyogenes
Frequency
0.0000
0.0002
0.0004
0.0006
0.0008
0.0010
0.0012
0
2000
4000
6000
8000
10000
Protein Size

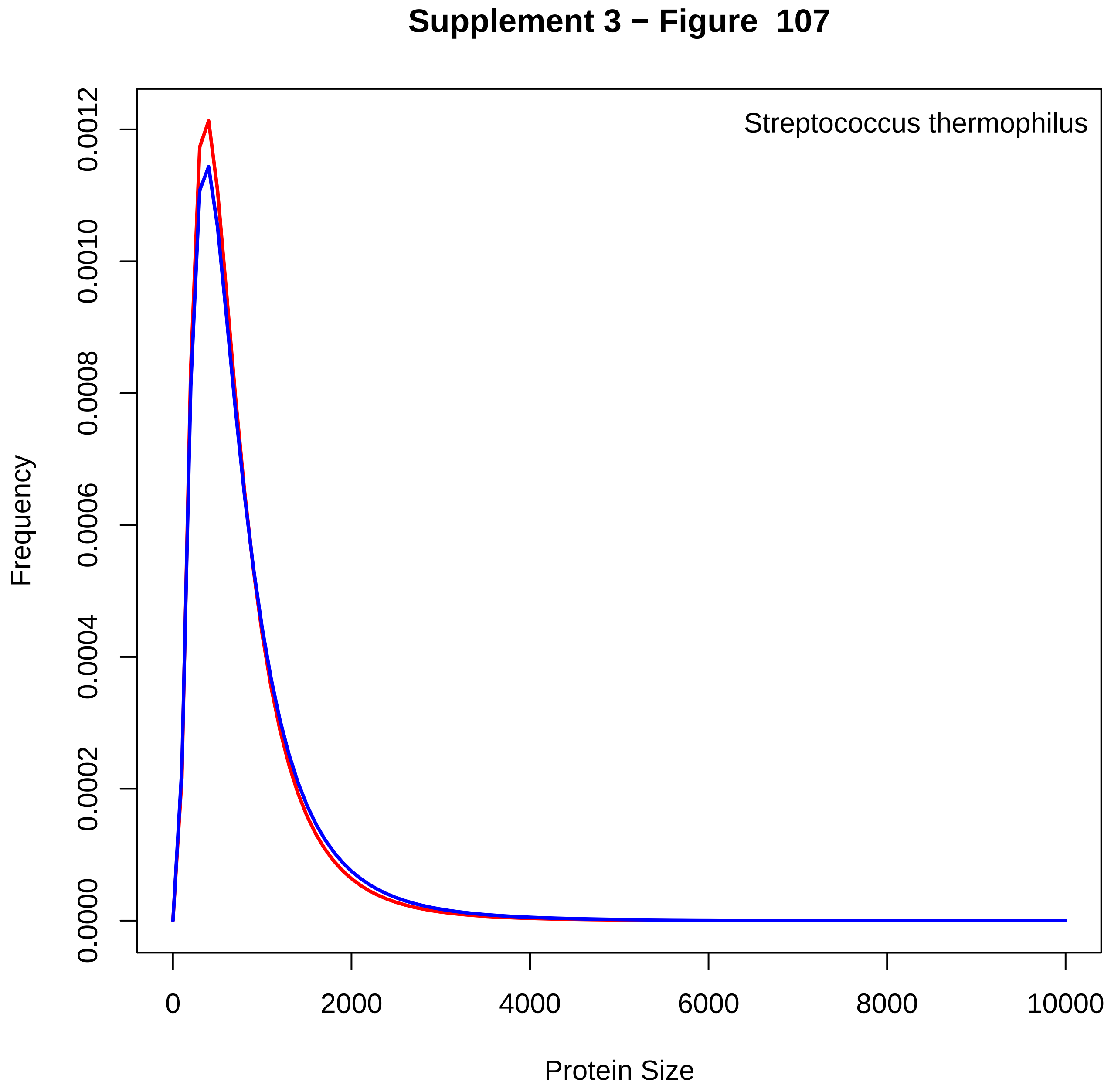
Supplement 3 − Figure 107
Streptococcus thermophilus
Frequency
0.0000
0.0002
0.0004
0.0006
0.0008
0.0010
0.0012
0
2000
4000
6000
8000
10000
Protein Size

Supplement 3 − Figure 108

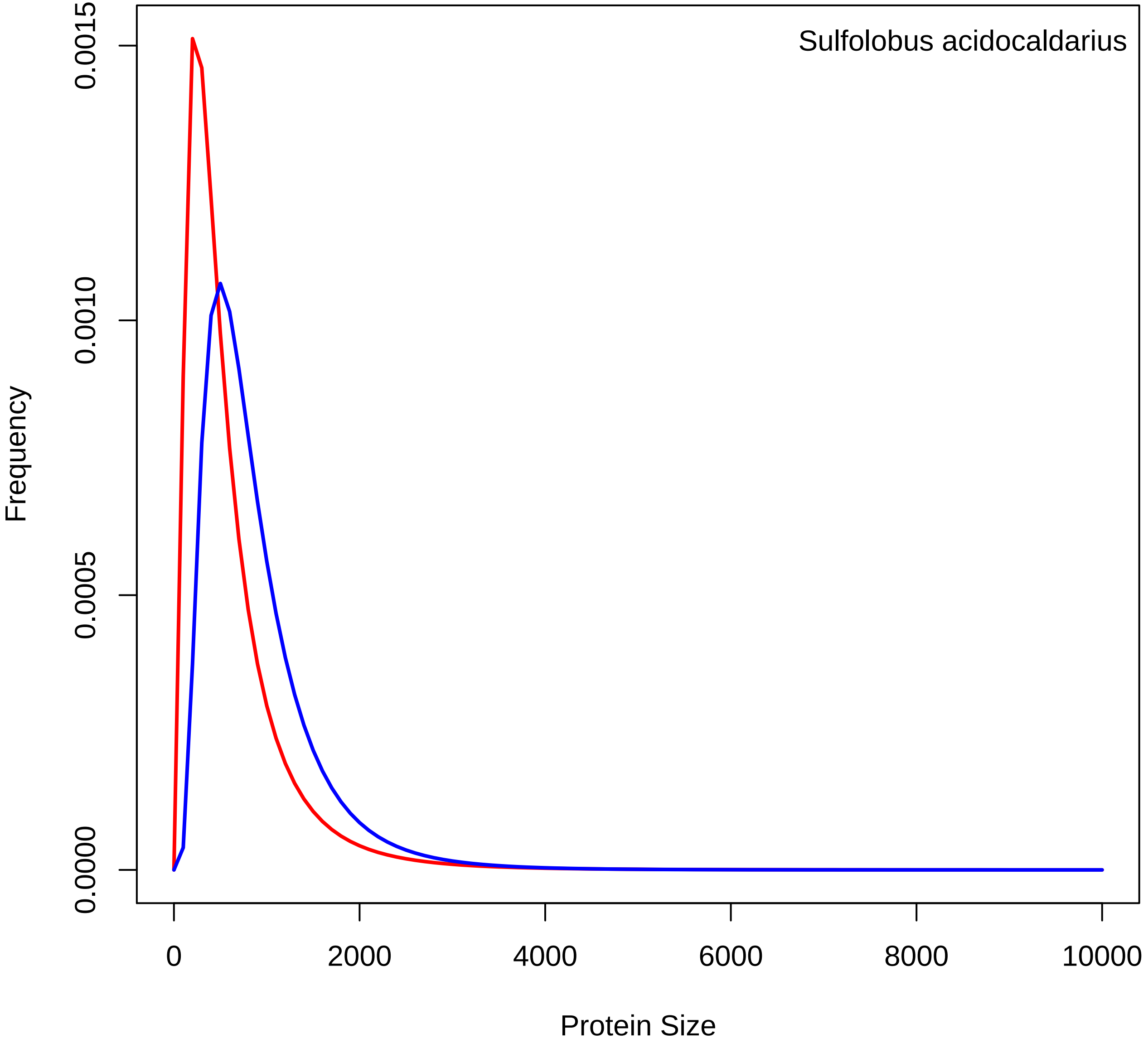

Supplement 3 − Figure 109

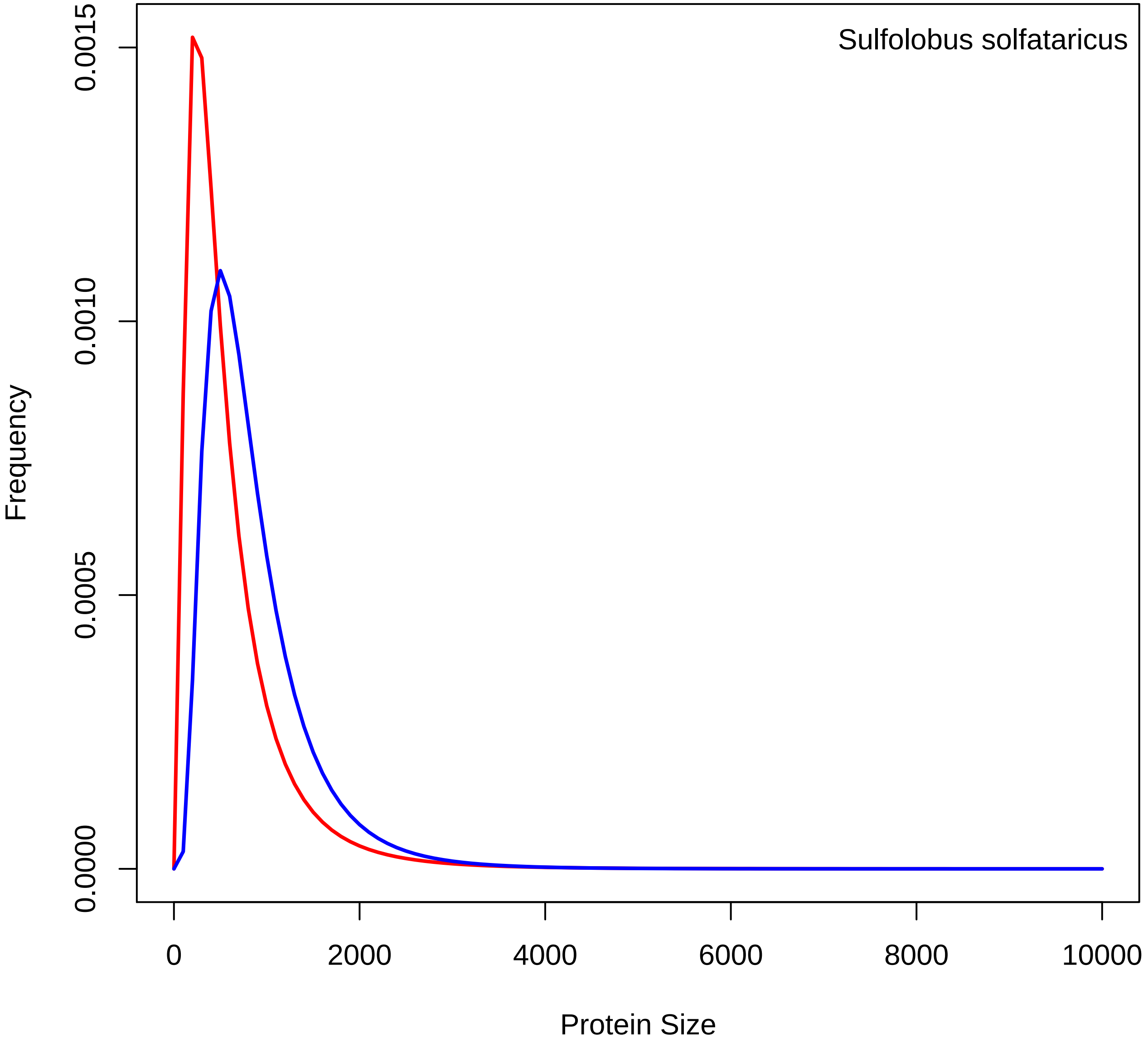

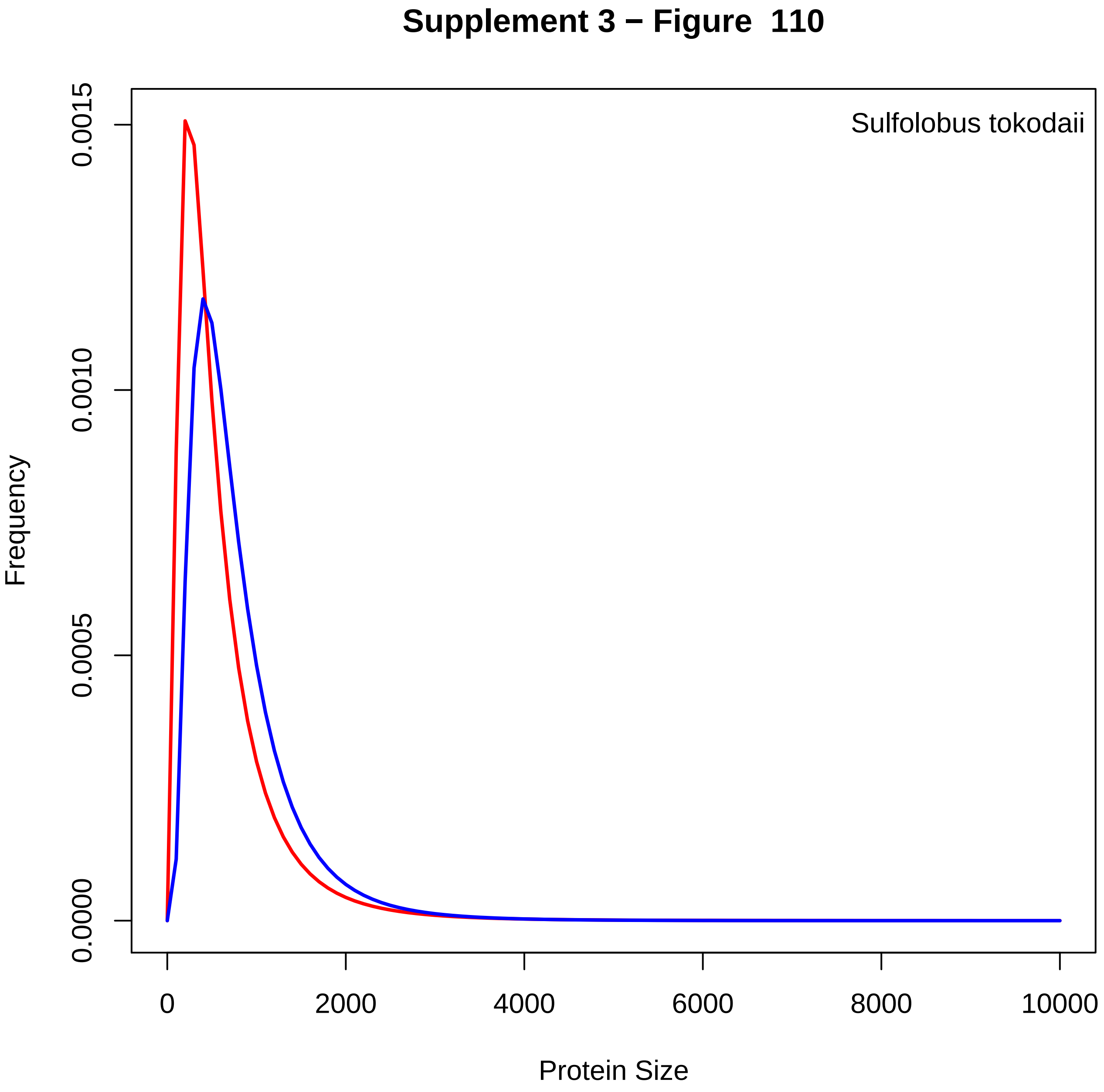
Supplement 3 − Figure 110
Sulfolobus tokodaii
Frequency
0.0000
0.0005
0.0010
0.0015
0
2000
4000
6000
8000
10000
Protein Size

Supplement 3 − Figure 111

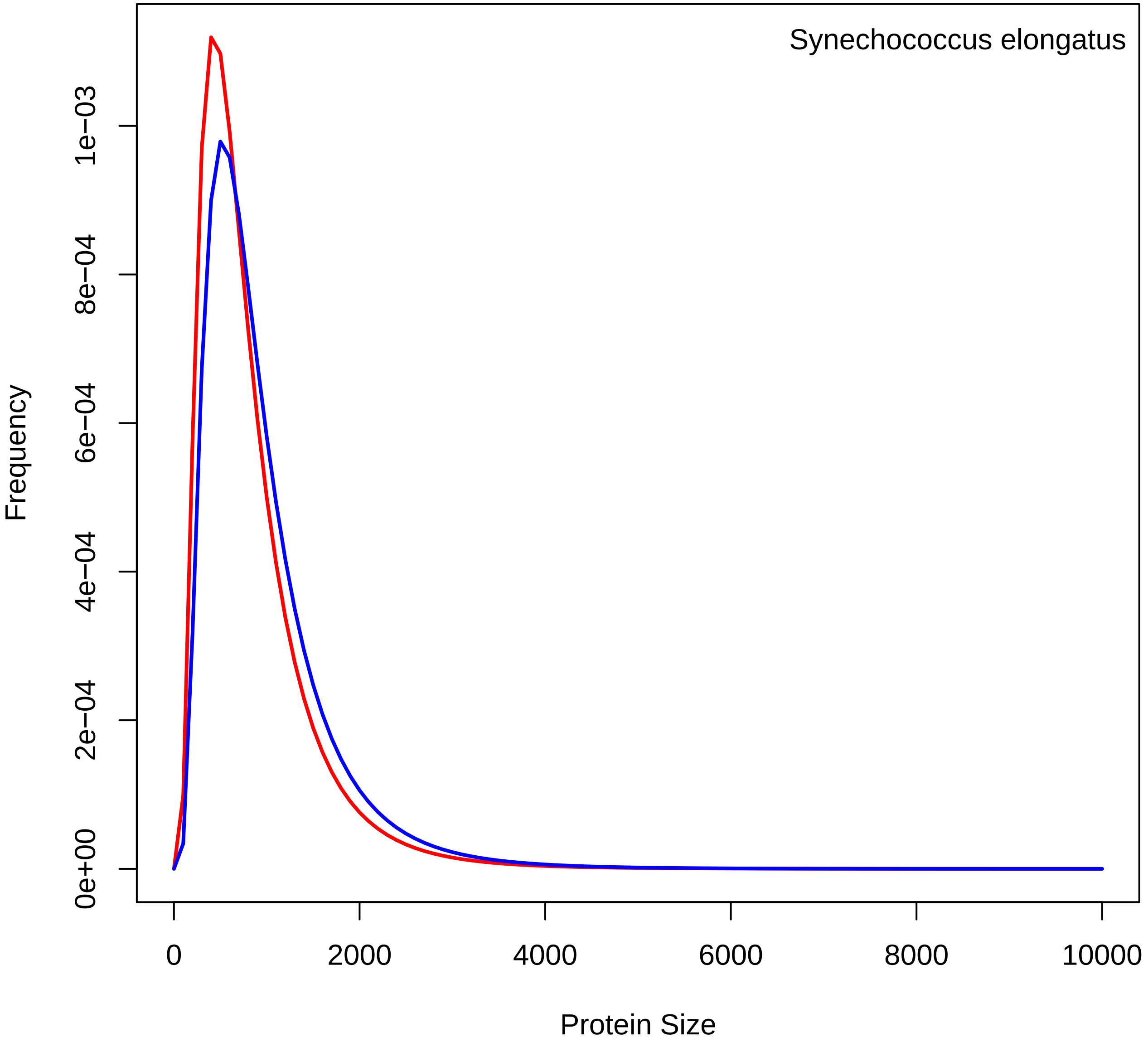

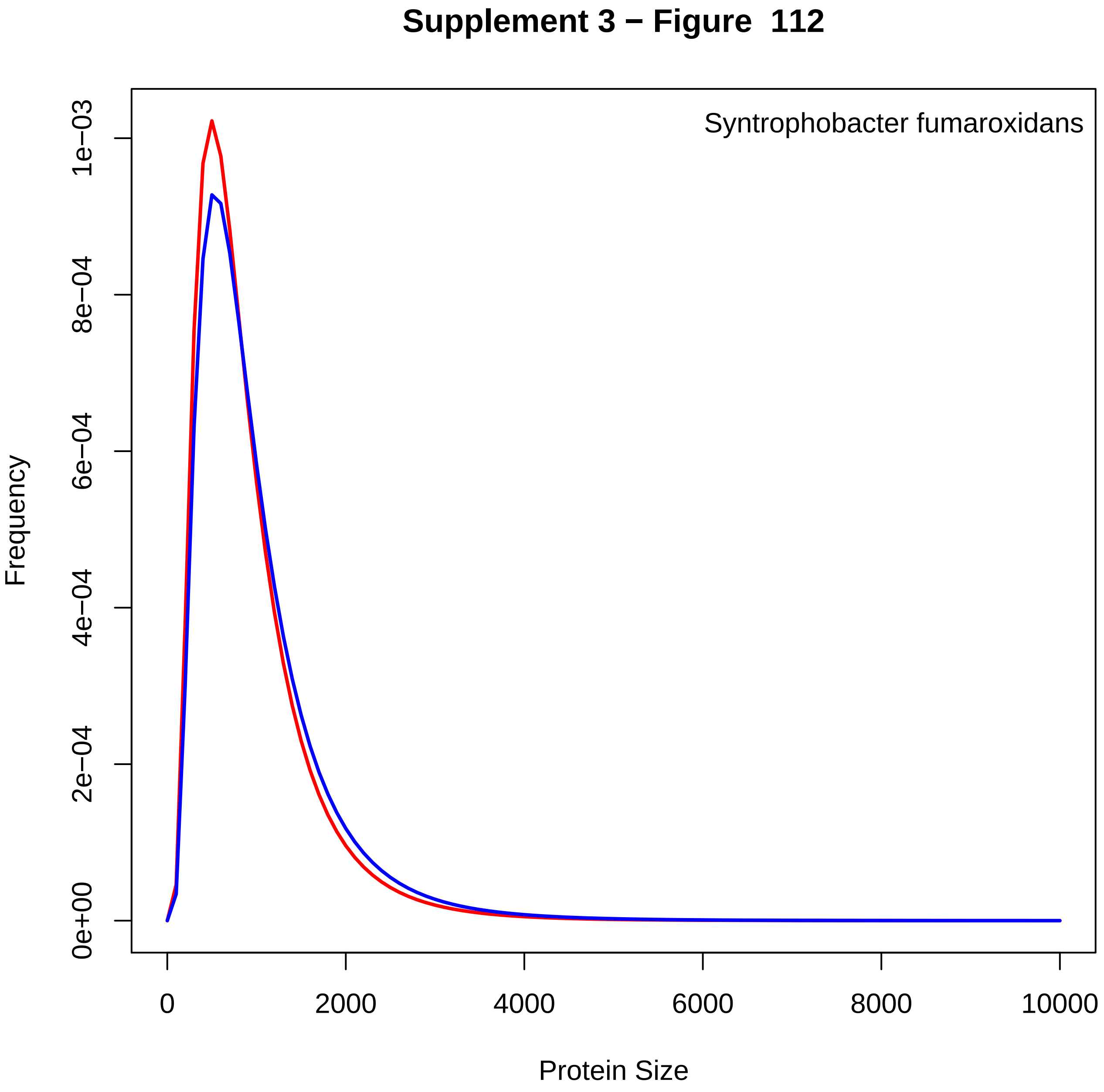
Supplement 3 − Figure 112
Syntrophobacter fumaroxidans
Frequency
0e+00
2e−04
4e−04
6e−04
8e−04
1e−03
0
2000
4000
6000
8000
10000
Protein Size

Supplement 3 − Figure 113

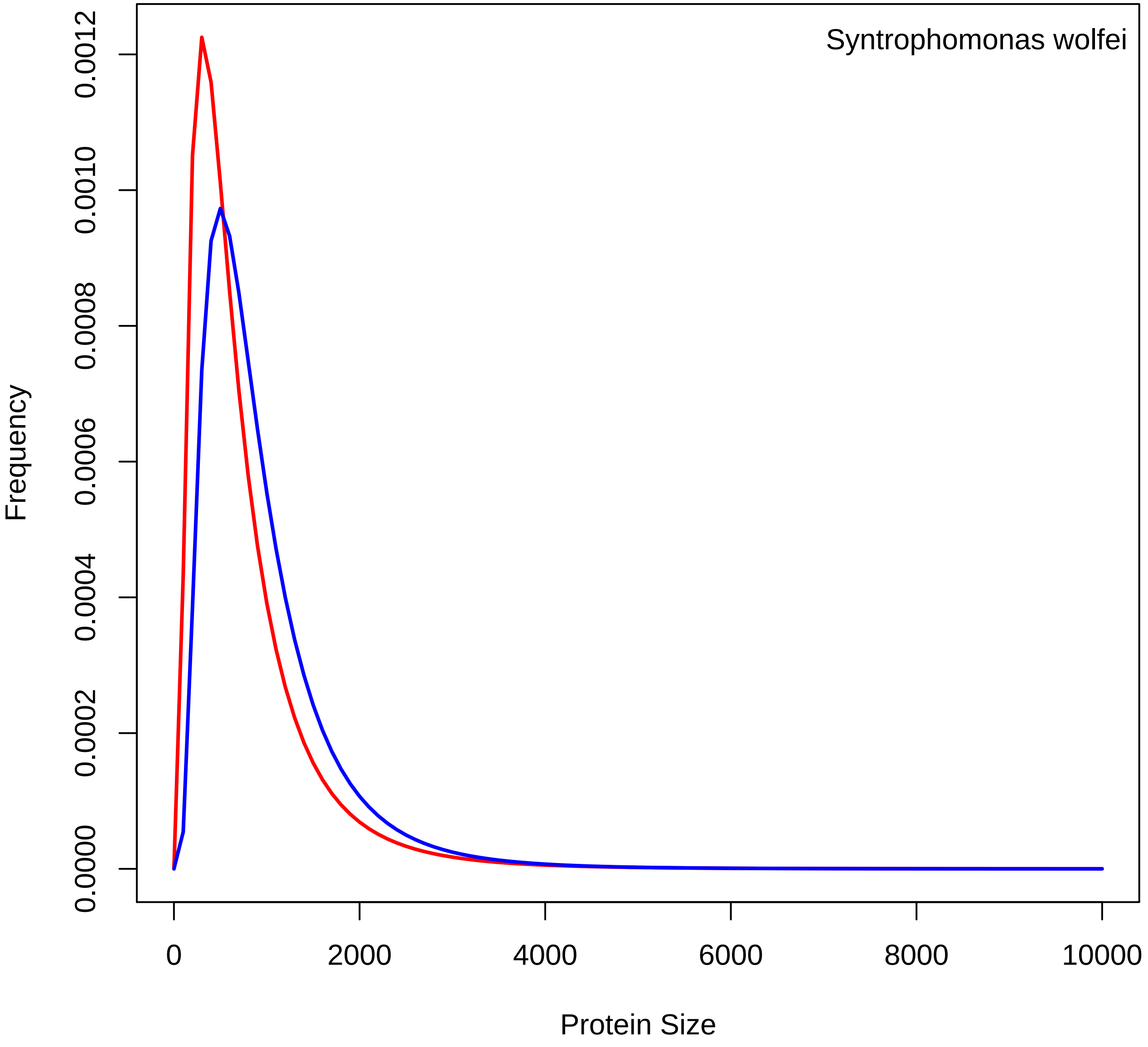

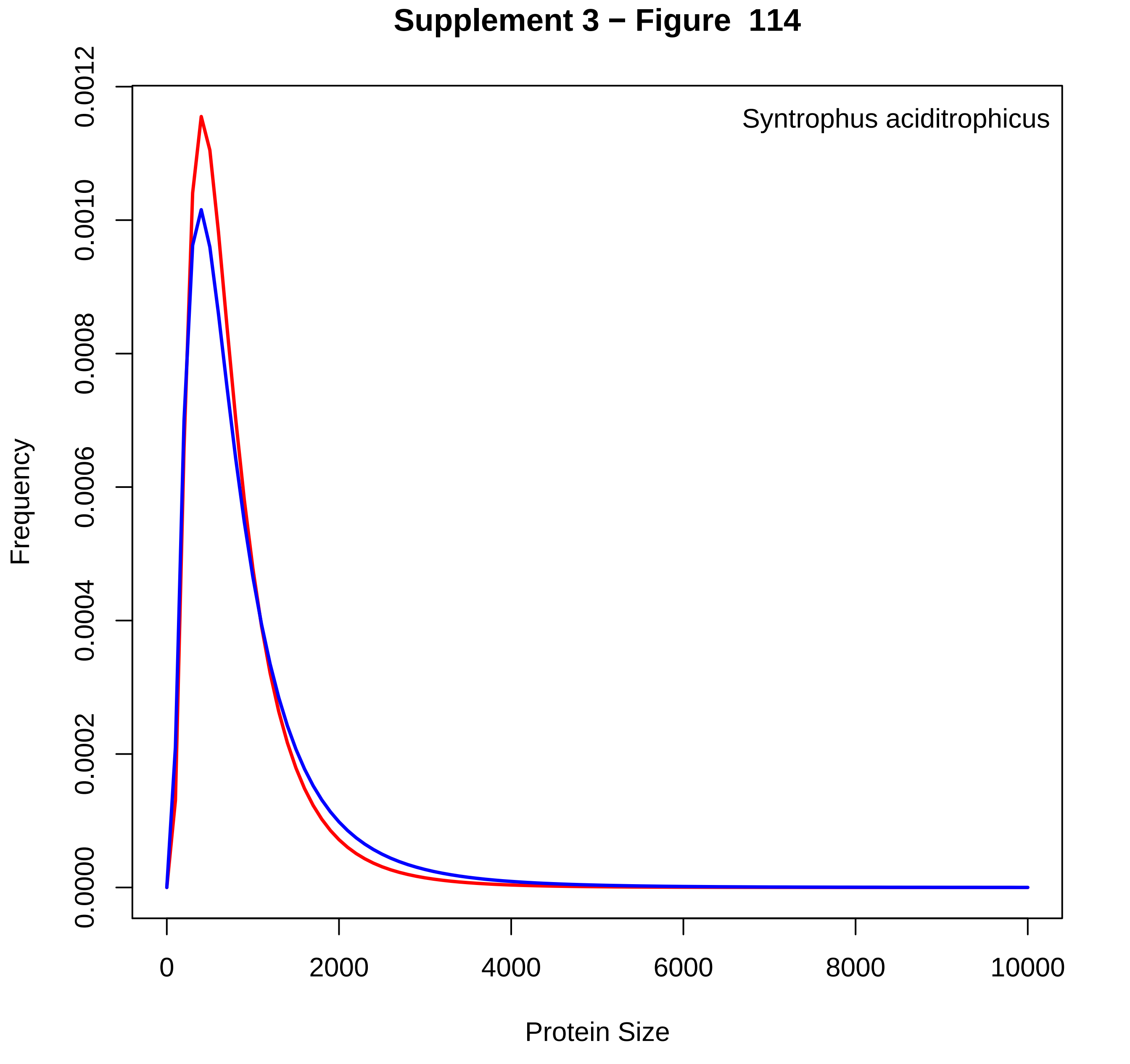
Supplement 3 − Figure 114
Syntrophus aciditrophicus
Frequency
Protein Size
0.0000
0.0002
0.0004
0.0006
0.0008
0.0010
0.0012
0
2000
4000
6000
8000
10000

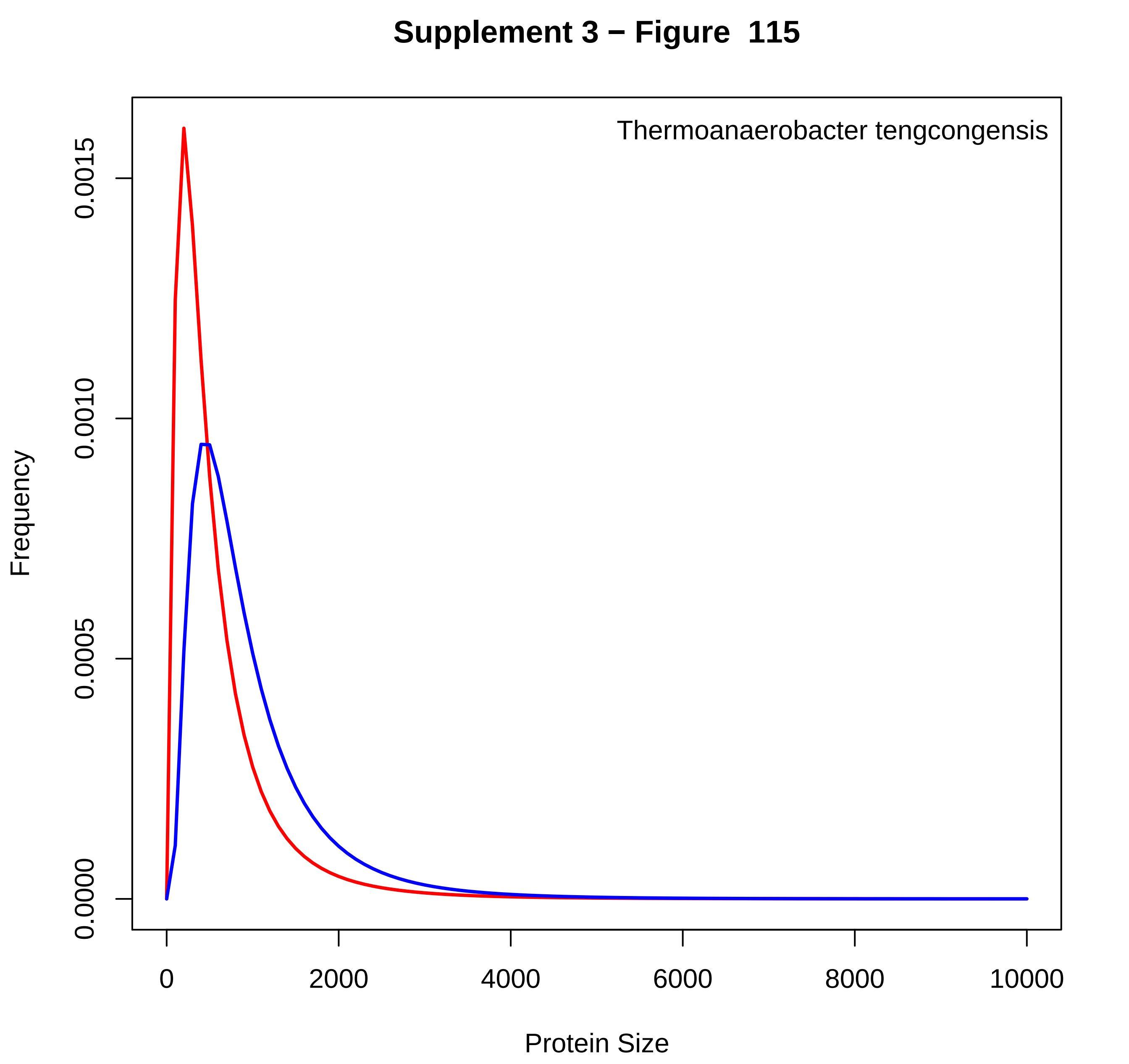
Supplement 3 − Figure 115
Thermoanaerobacter tengcongensis
Frequency
0.0000
0.0005
0.0010
0.0015
0
2000
4000
6000
8000
10000
Protein Size

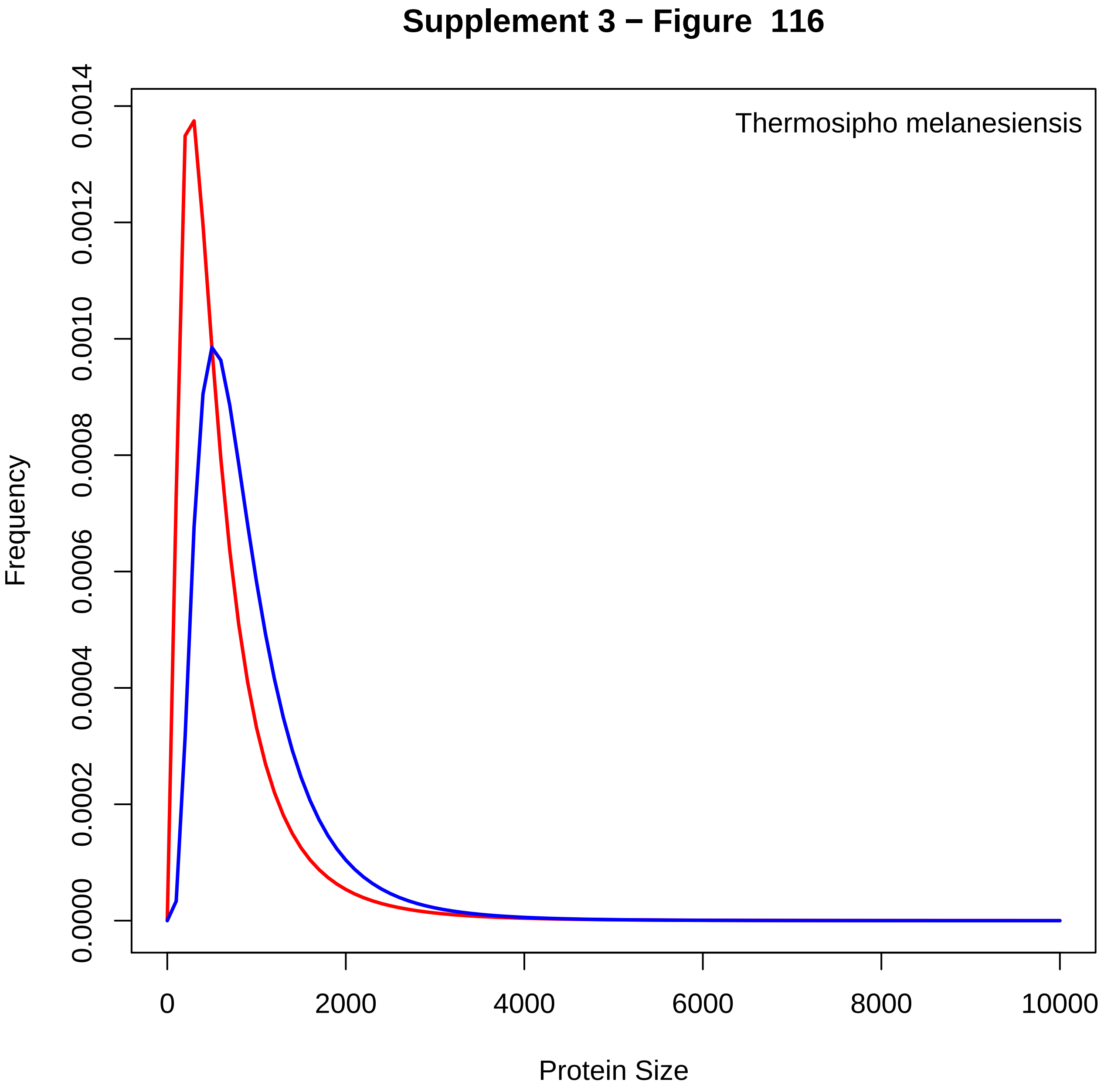
Supplement 3 − Figure 116
Thermosipho melanesiensis
Frequency
0.0000
0.0002
0.0004
0.0006
0.0008
0.0010
0.0012
0.0014
0
2000
4000
6000
8000
10000
Protein Size

Supplement 3 − Figure 117

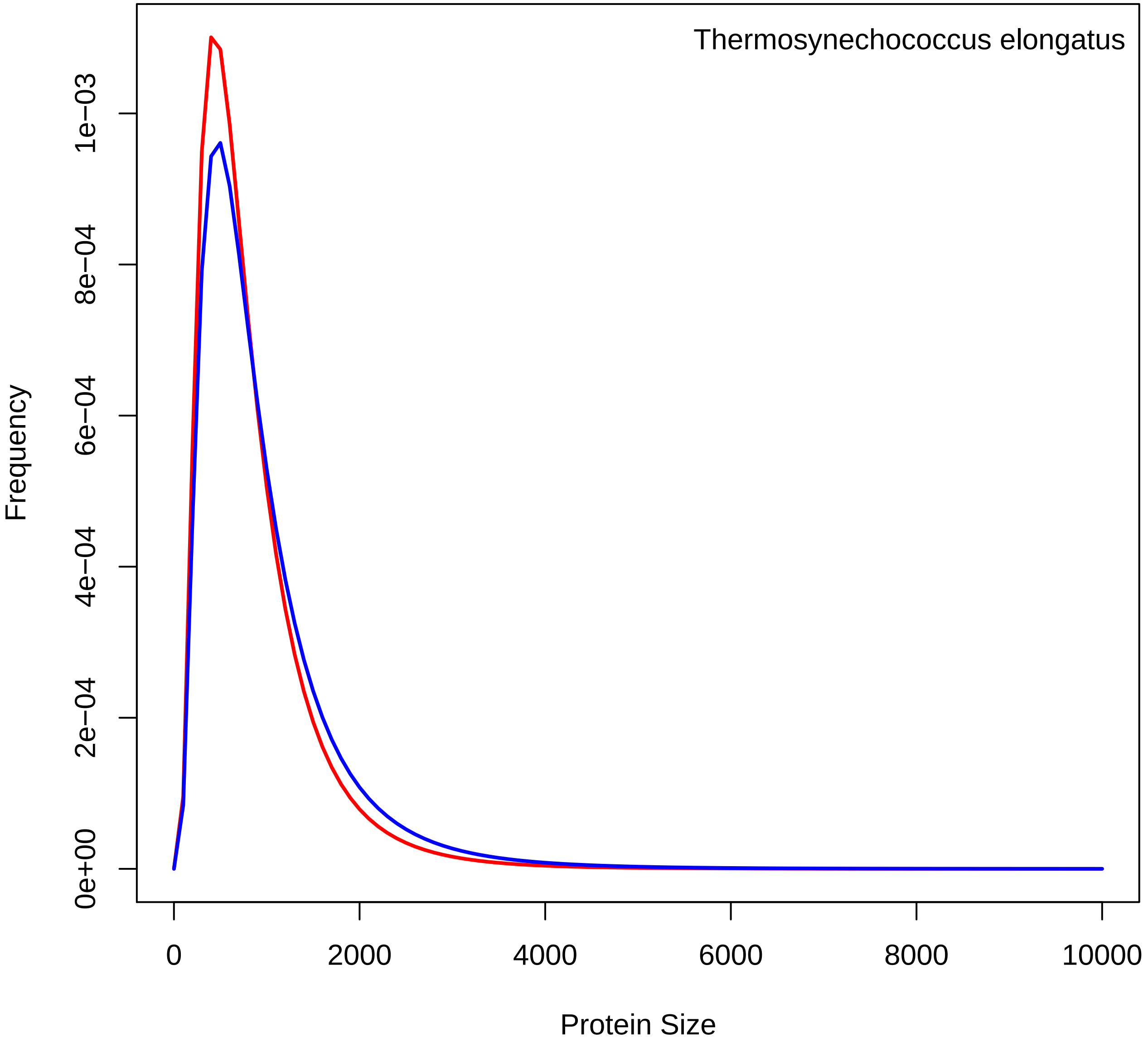

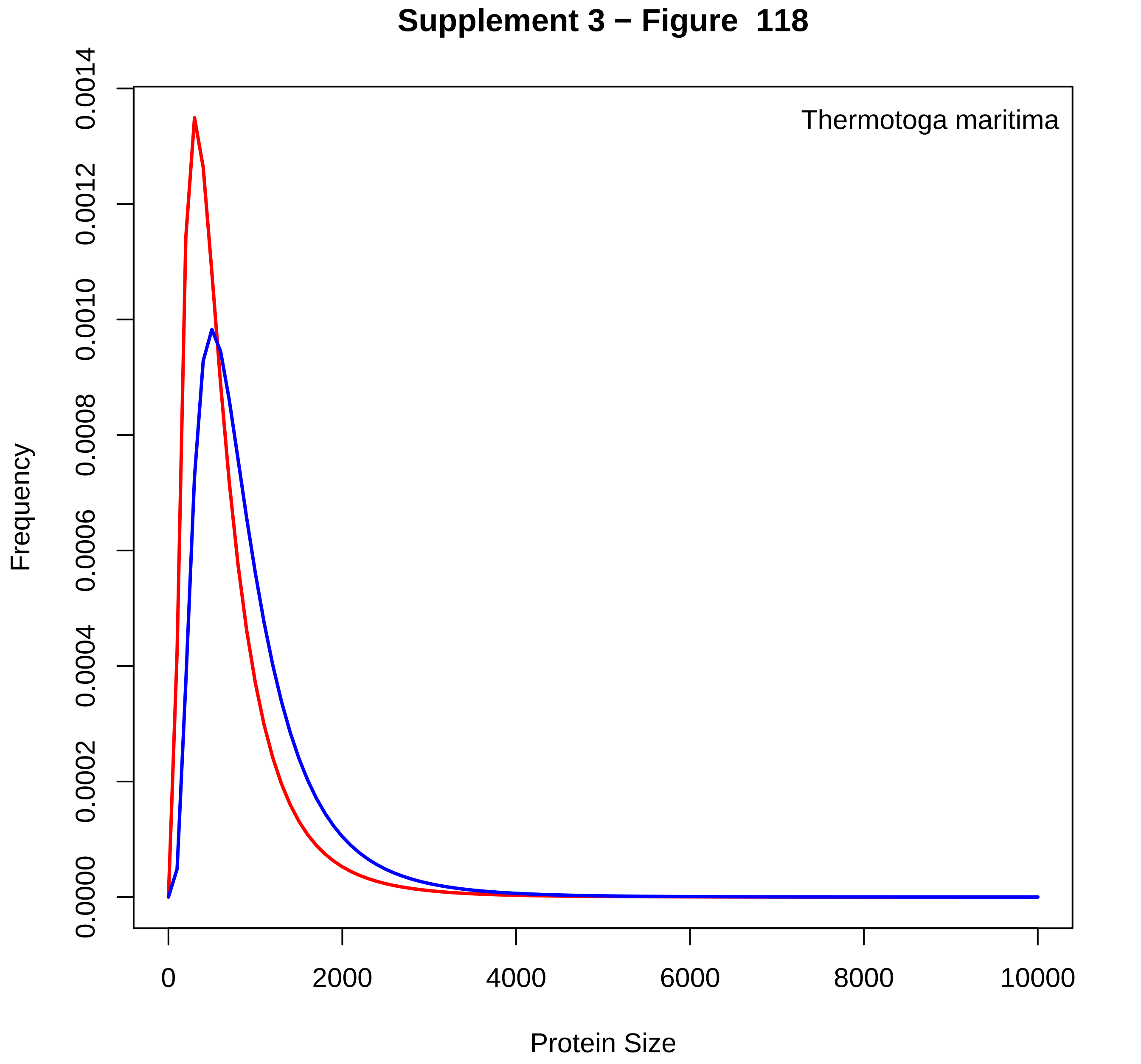
Supplement 3 − Figure 118
Thermotoga maritima
Frequency
0.0000
0.0002
0.0004
0.0006
0.0008
0.0010
0.0012
0.0014
0
2000
4000
6000
8000
10000
Protein Size

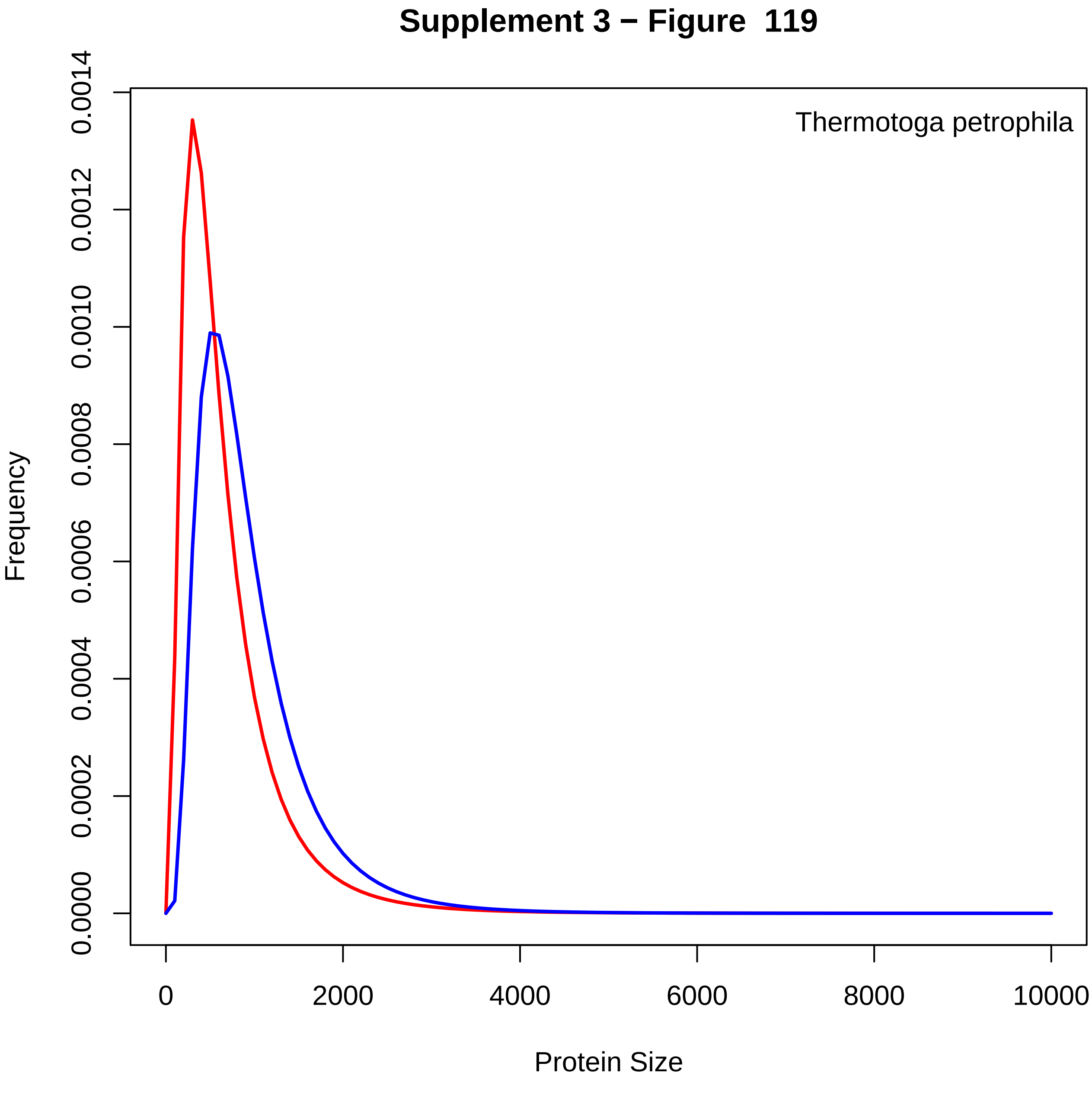

Supplement 3 − Figure 119
Thermotoga petrophila
Frequency
0.0000
0.0002
0.0004
0.0006
0.0008
0.0010
0.0012
0.0014
0
2000
4000
6000
8000
10000
Protein Size

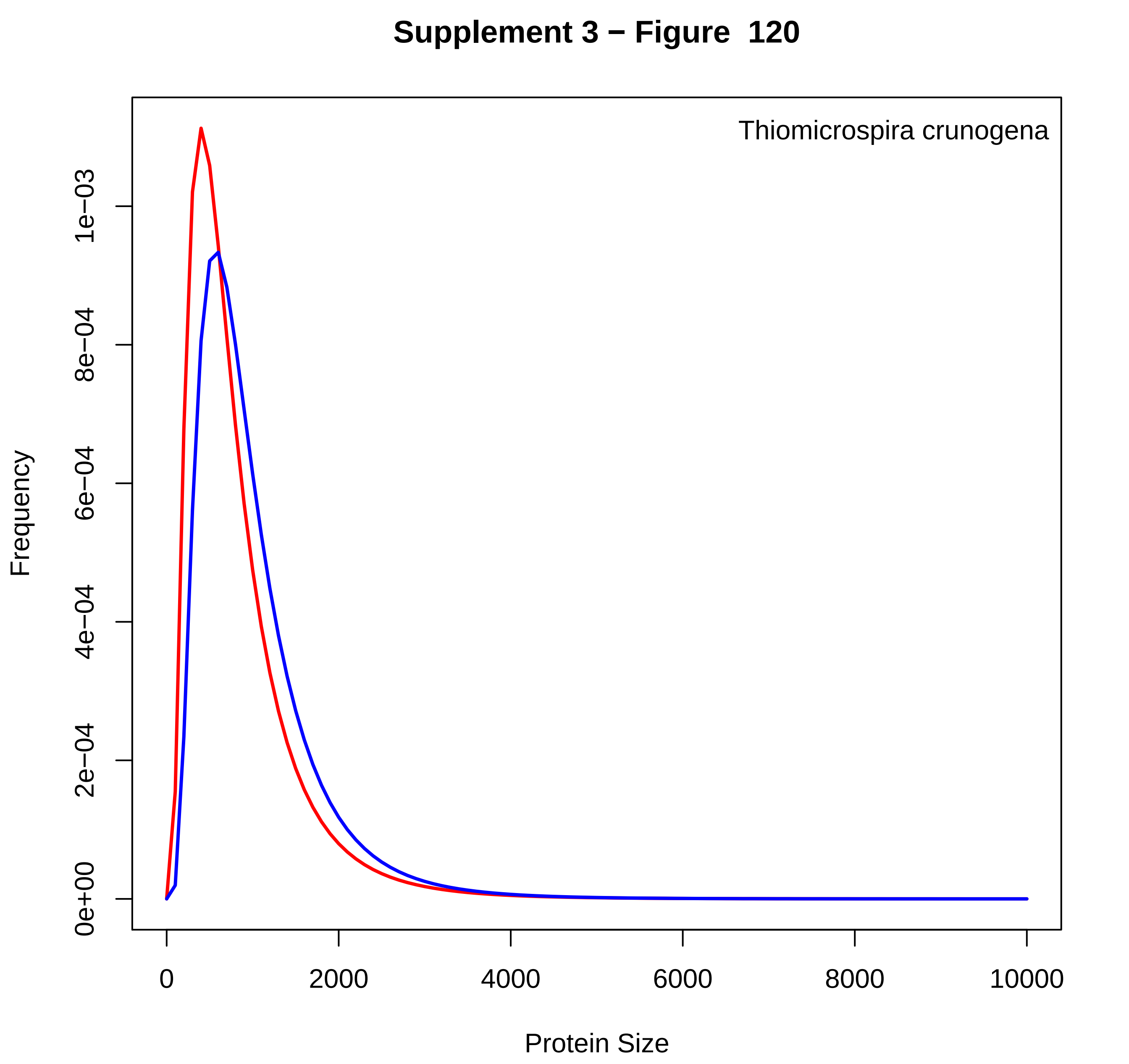
Supplement 3 − Figure 120
Thiomicrospira crunogena
Frequency
Protein Size
0e+00
2e−04
4e−04
6e−04
8e−04
1e−03
0
2000
4000
6000
8000
10000

Supplement 3 − Figure 121

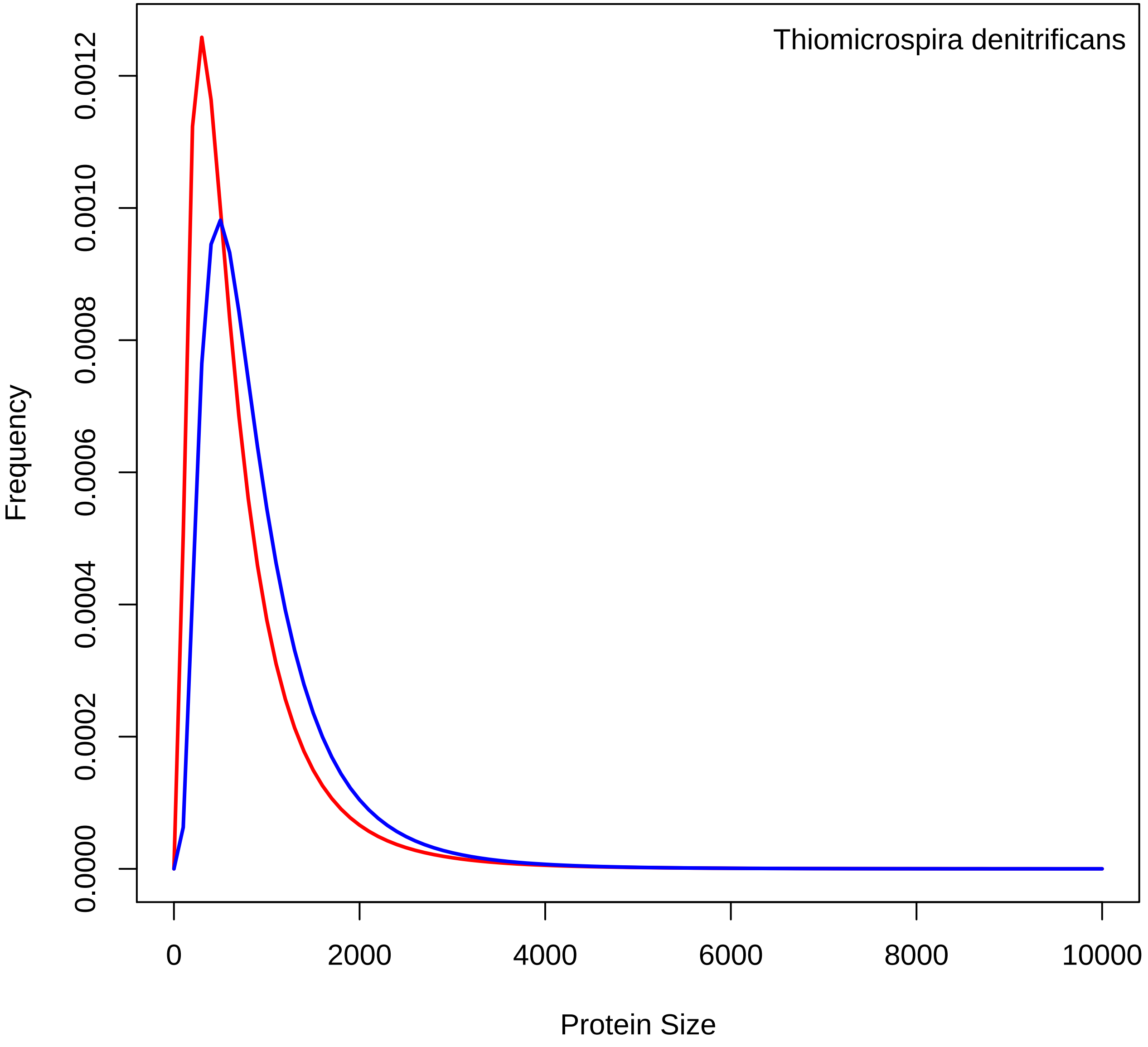

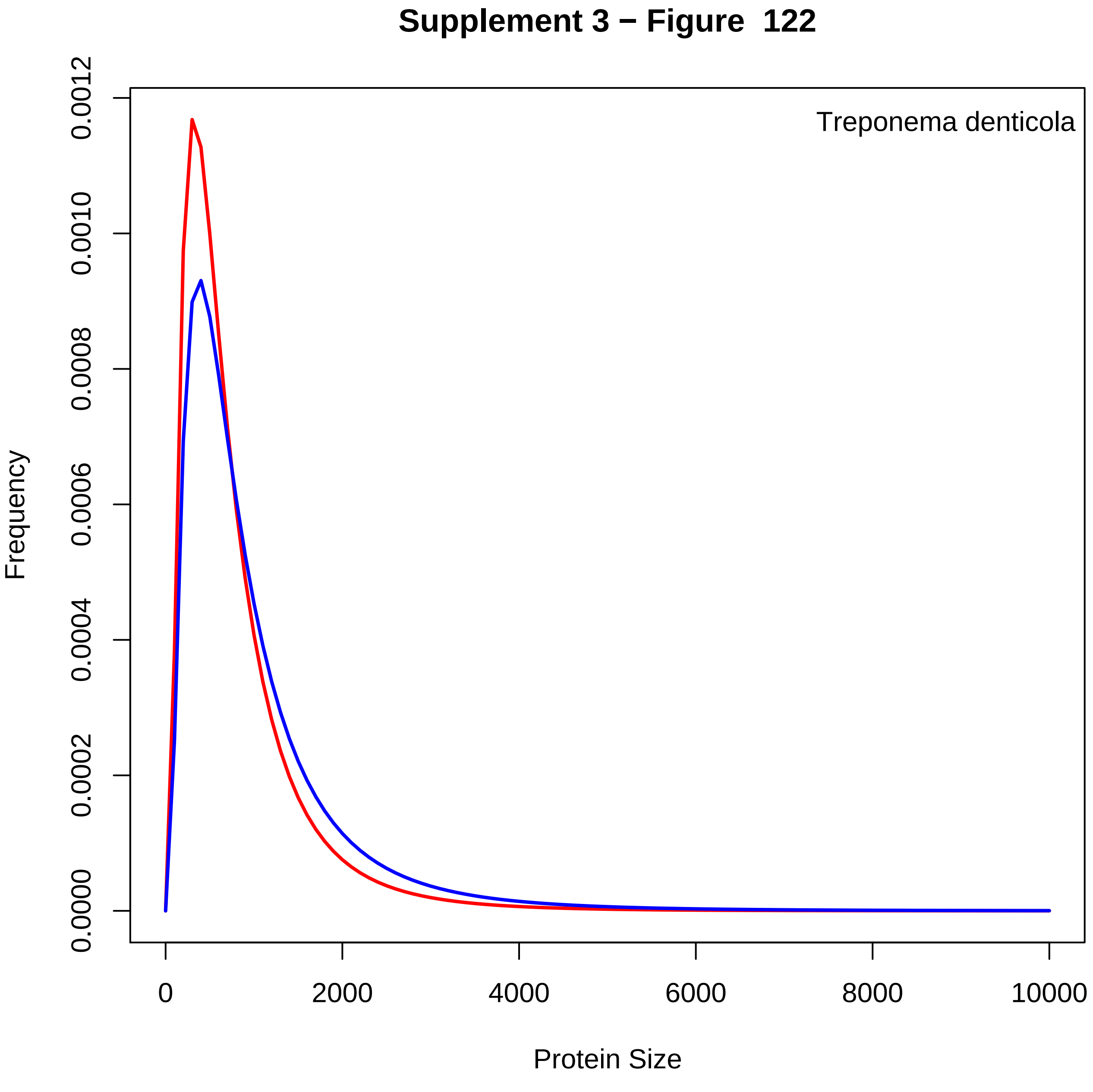
Supplement 3 − Figure 122
Treponema denticola
Frequency
0.0000
0.0002
0.0004
0.0006
0.0008
0.0010
0.0012
0
2000
4000
6000
8000
10000
Protein Size

Supplement 3 − Figure 123

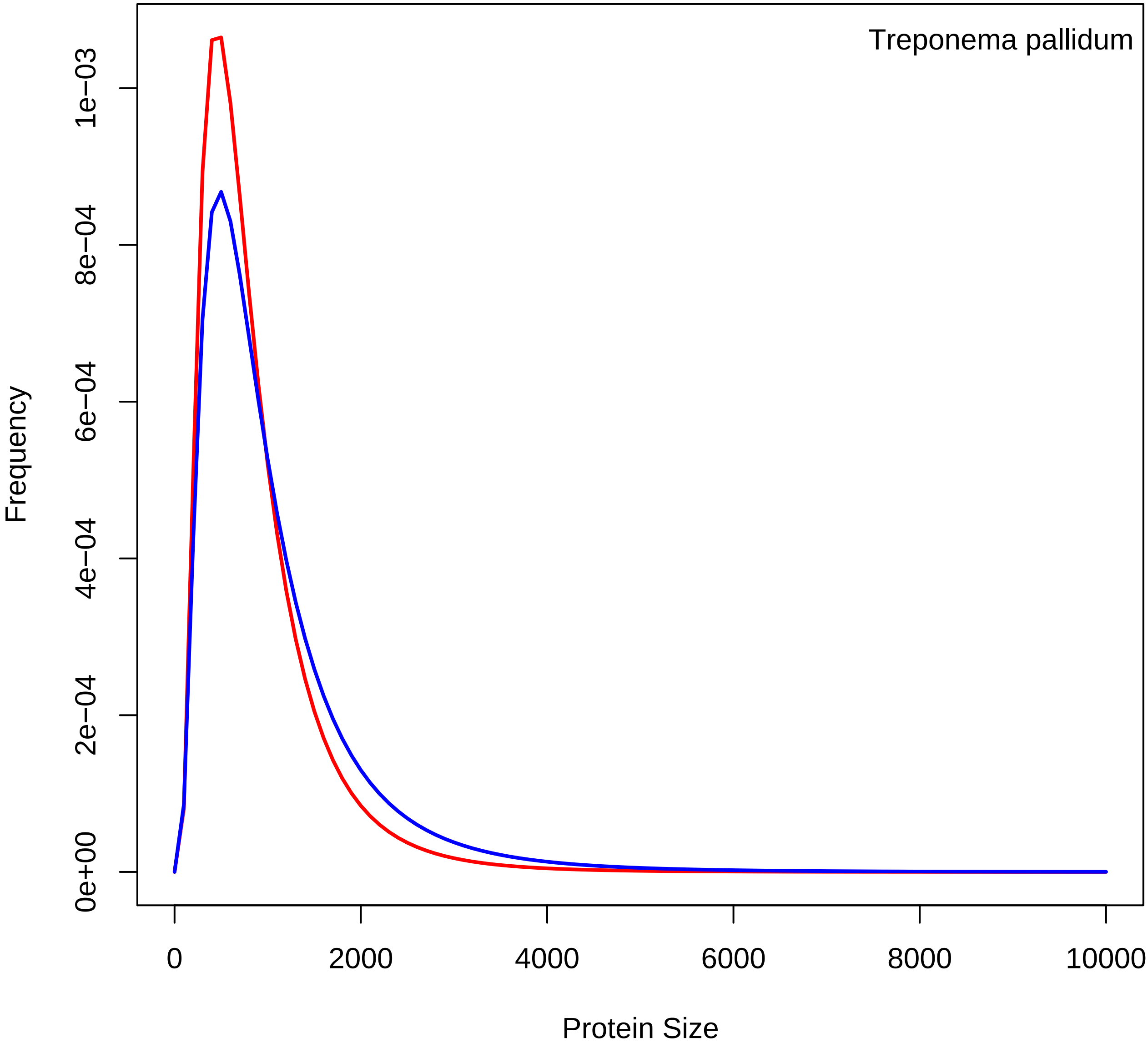

Supplement 3 − Figure 124

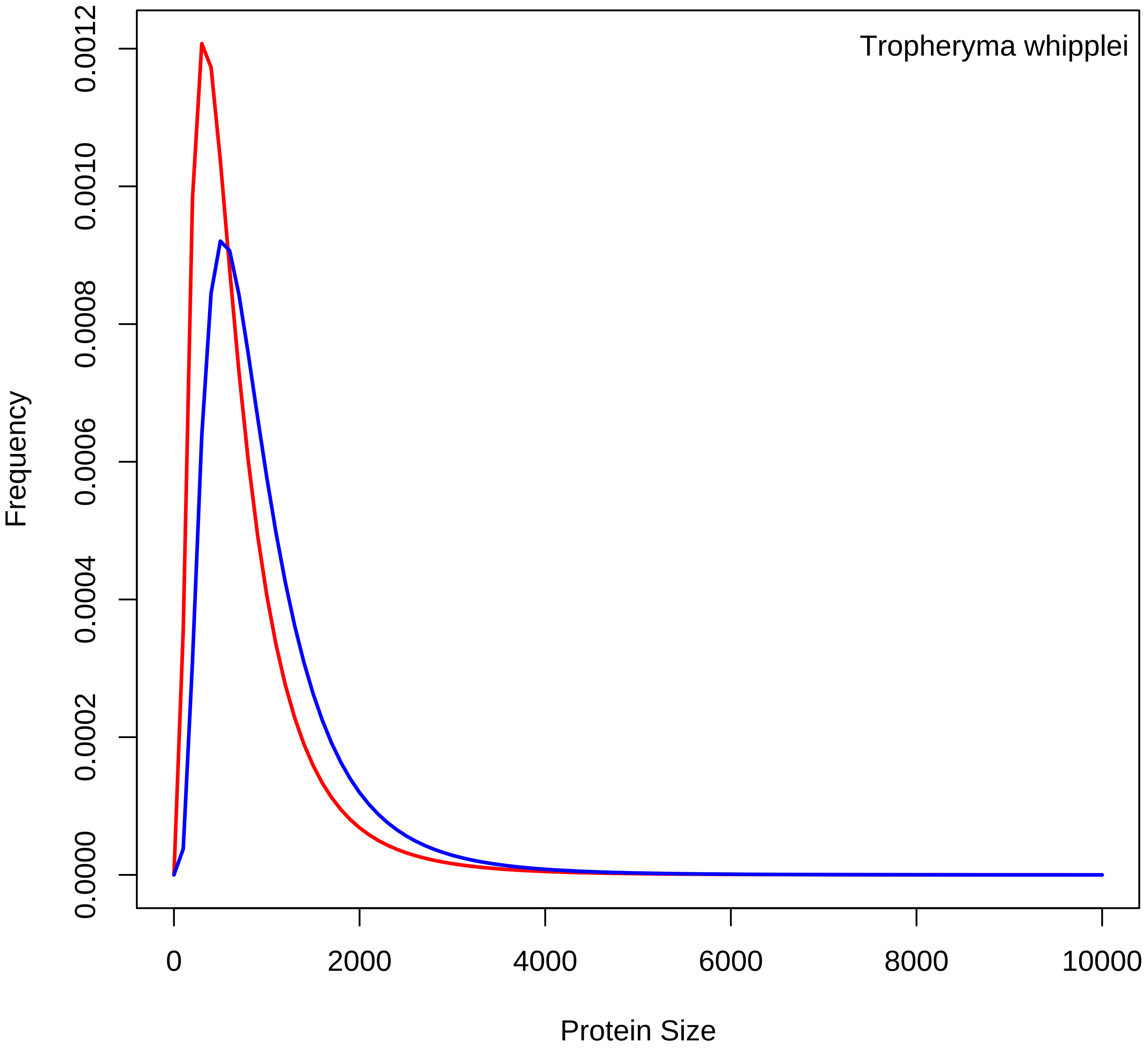

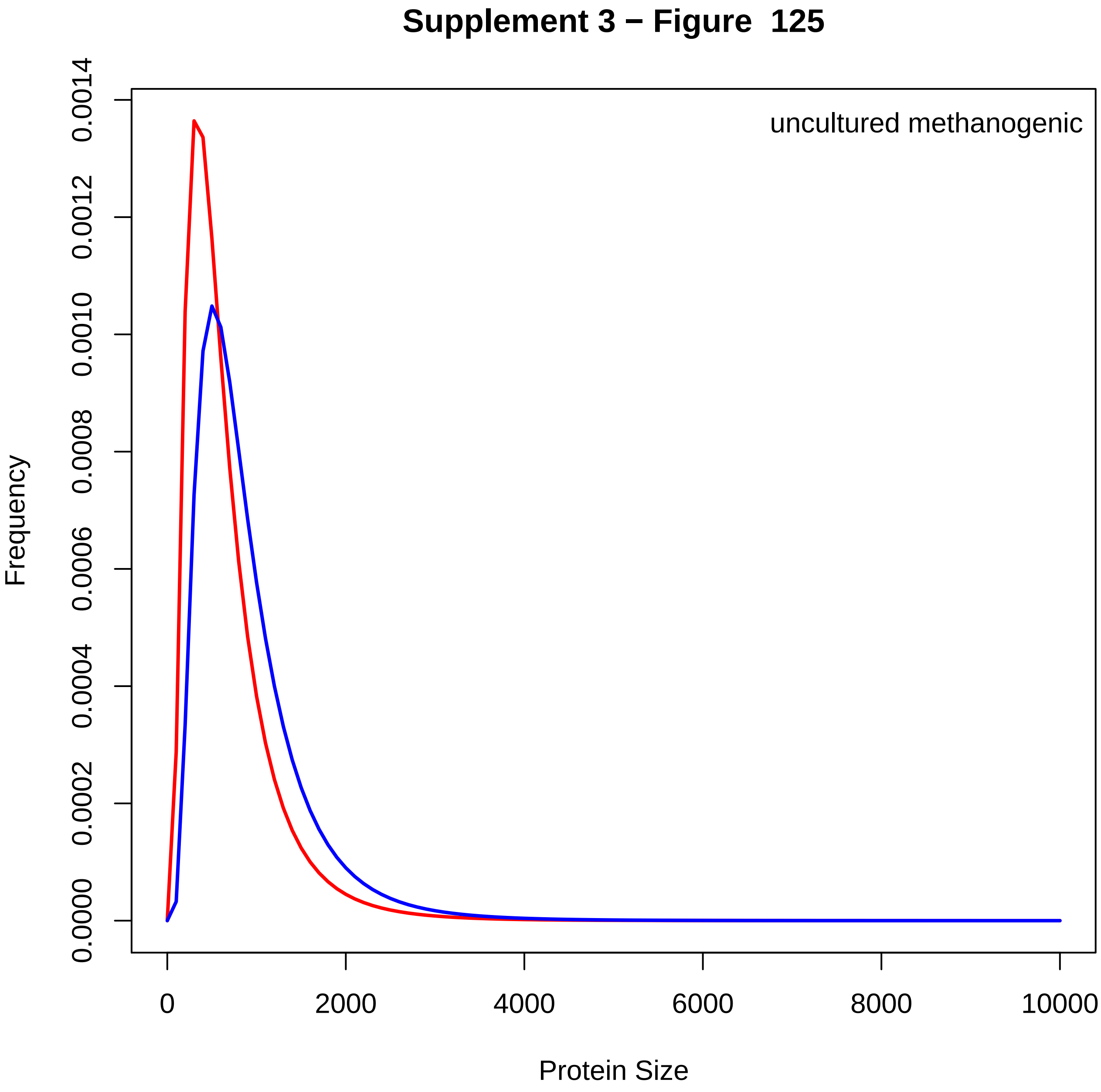
Supplement 3 − Figure 125
uncultured methanogenic
Frequency
0.0000
0.0002
0.0004
0.0006
0.0008
0.0010
0.0012
0.0014
0
2000
4000
6000
8000
10000
Protein Size

Supplement 3 − Figure 126

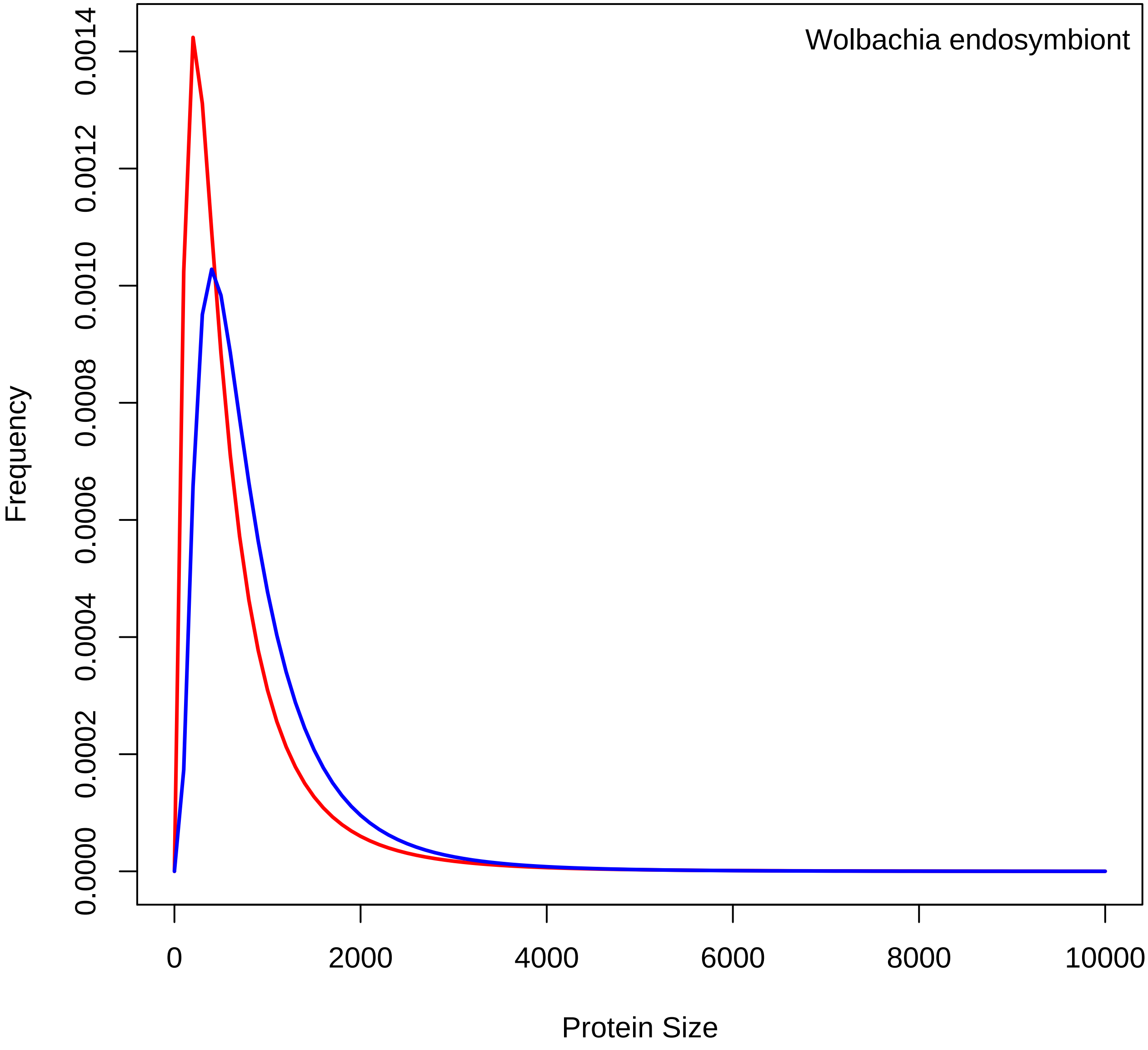

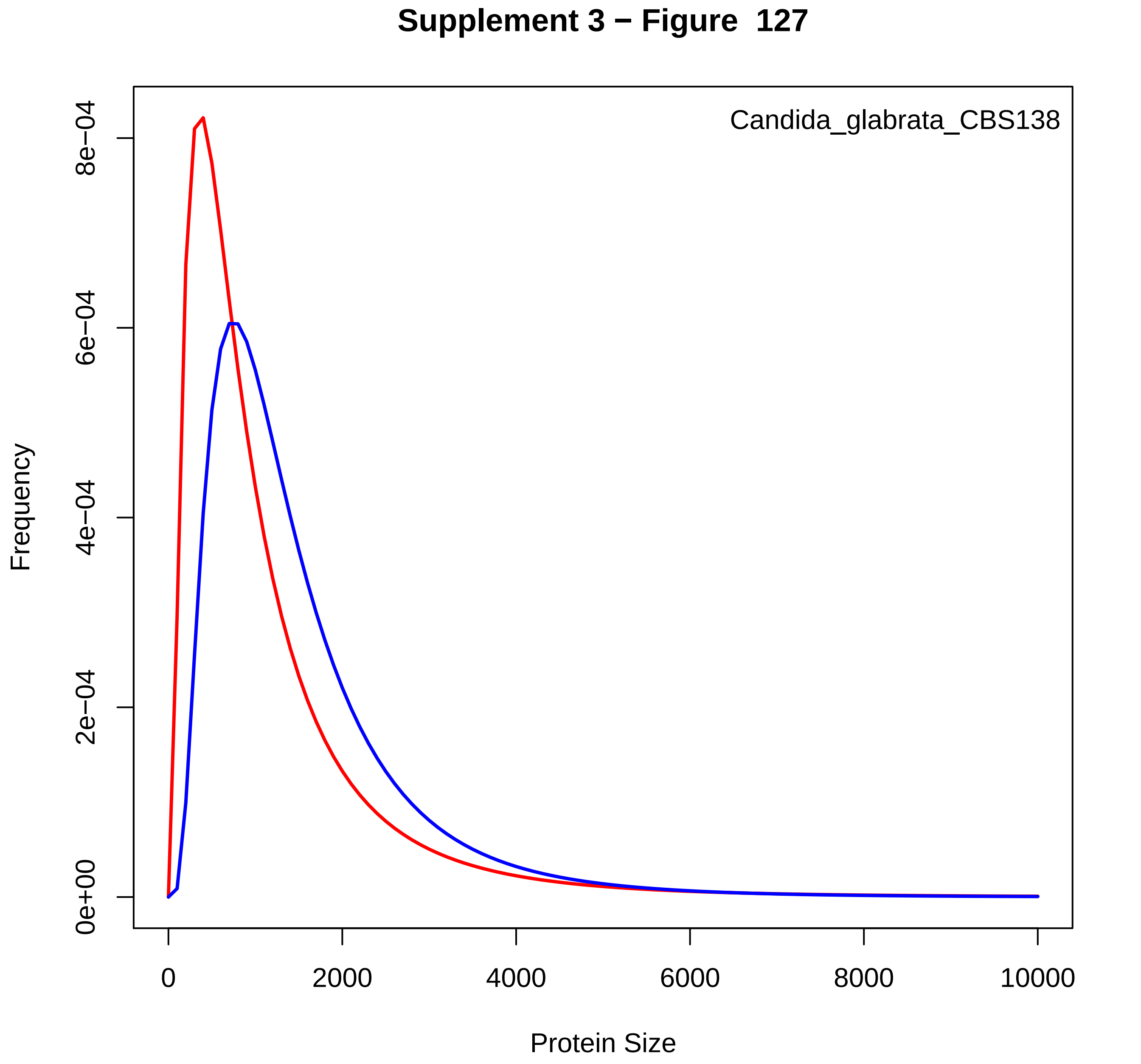

Supplement 3 − Figure 127
Candida_glabrata_CBS138
Frequency
0e+00
2e−04
4e−04
6e−04
8e−04
0
2000
4000
6000
8000
10000
Protein Size

Supplement 3 − Figure 128

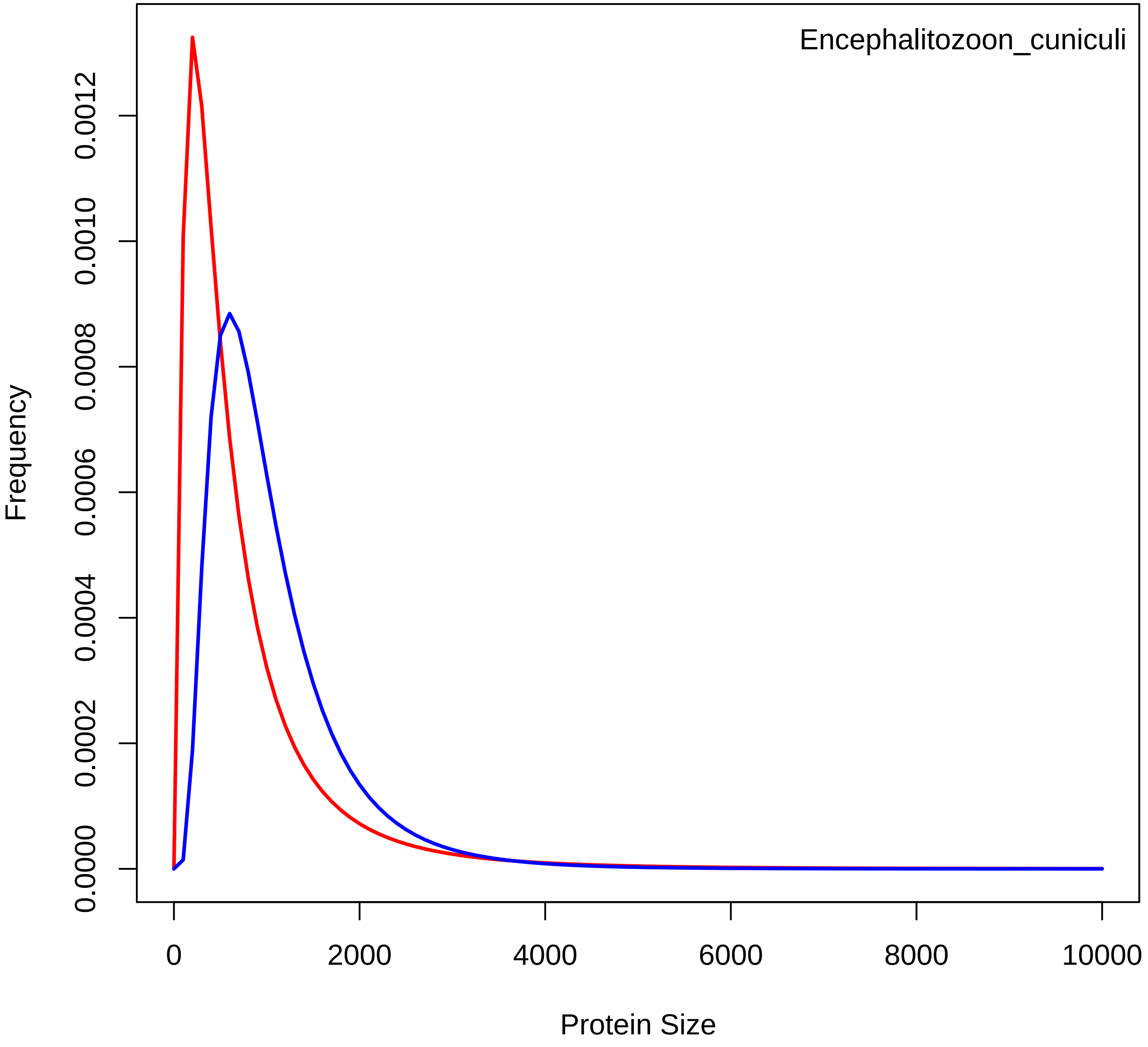

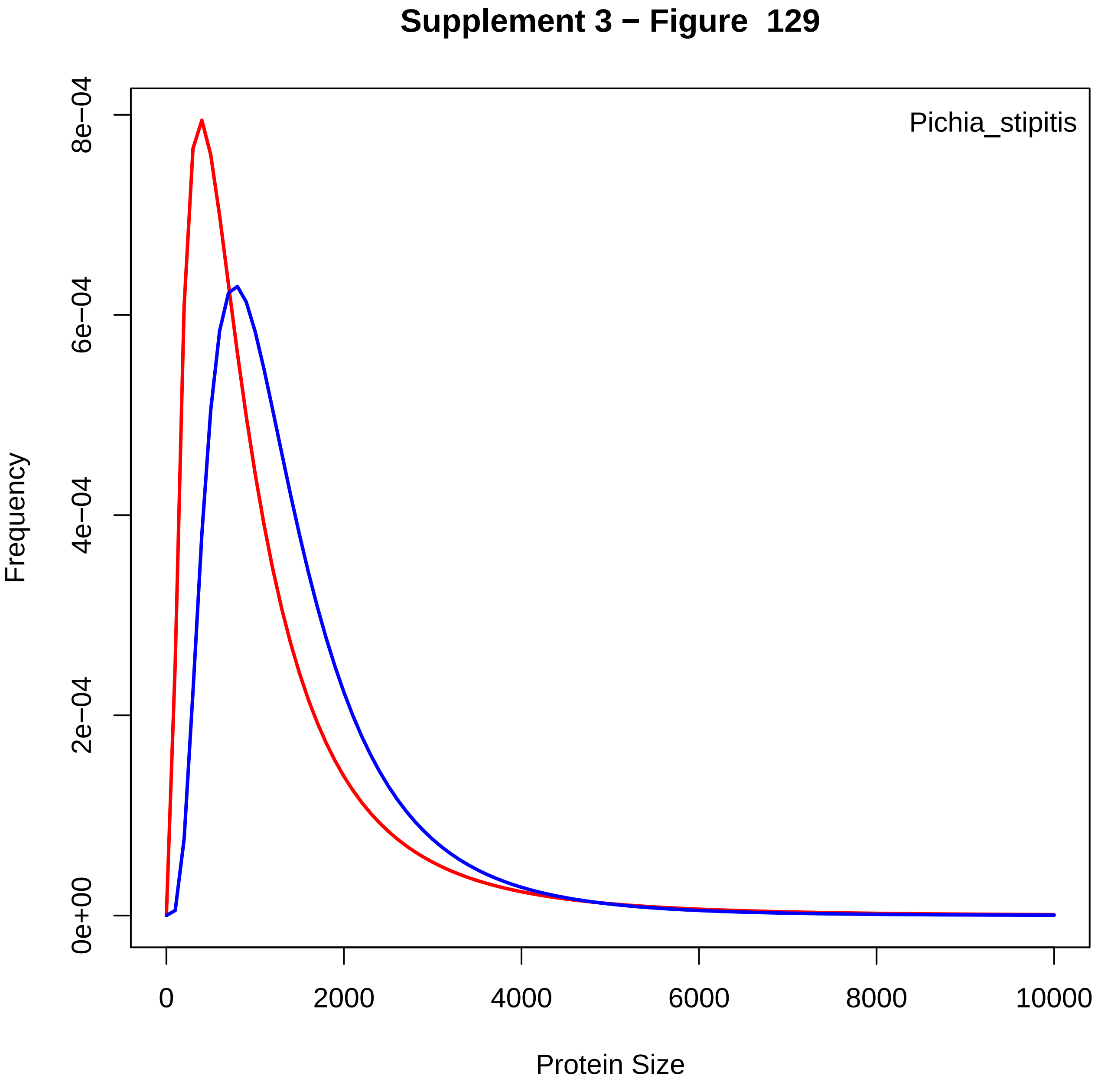
Supplement 3 − Figure 129
Pichia_stipitis
Frequency
0e+00
2e−04
4e−04
6e−04
8e−04
0
2000
4000
6000
8000
10000
Protein Size

Supplement 3 − Figure 130

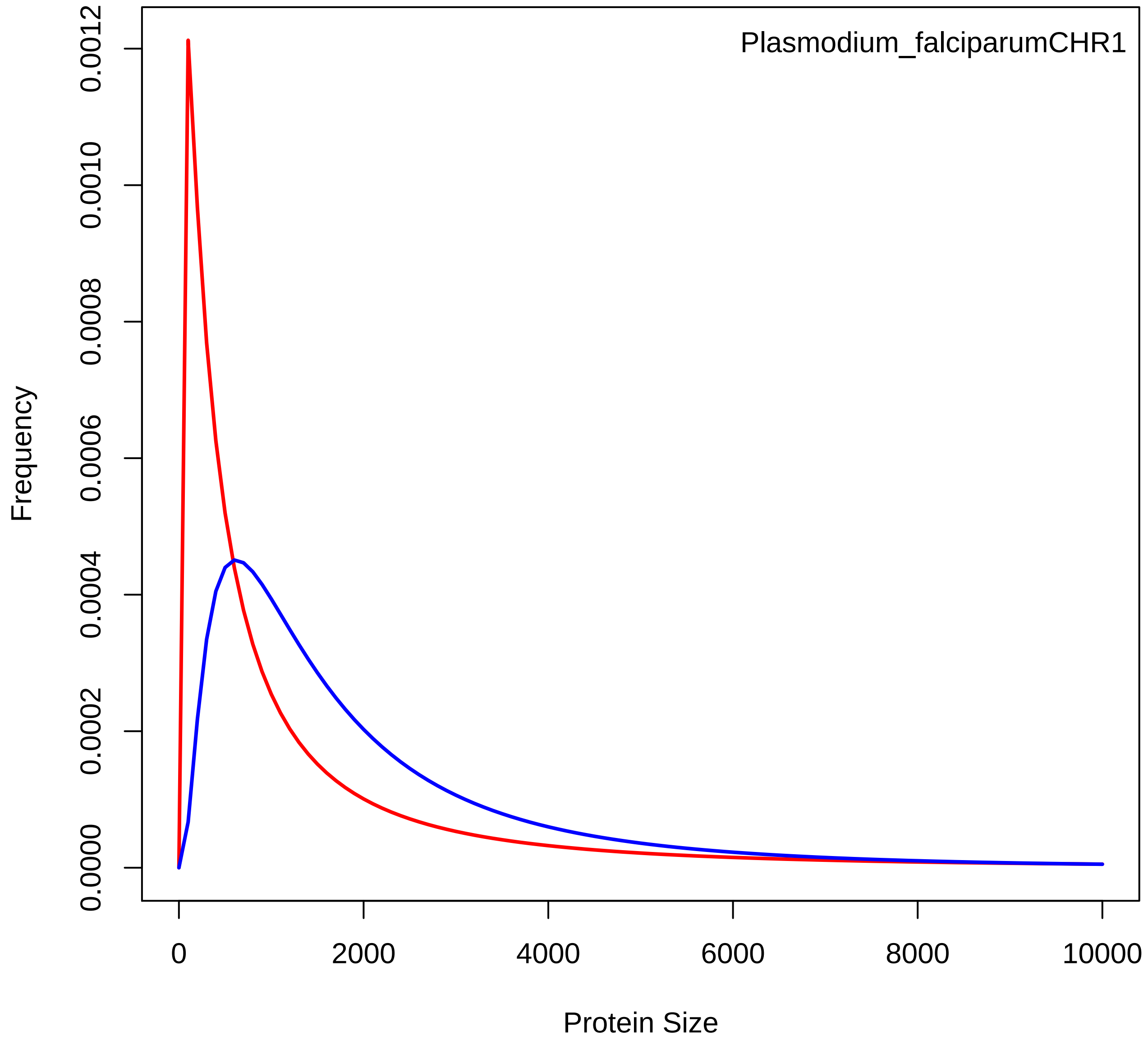

Supplement 3 − Figure 131

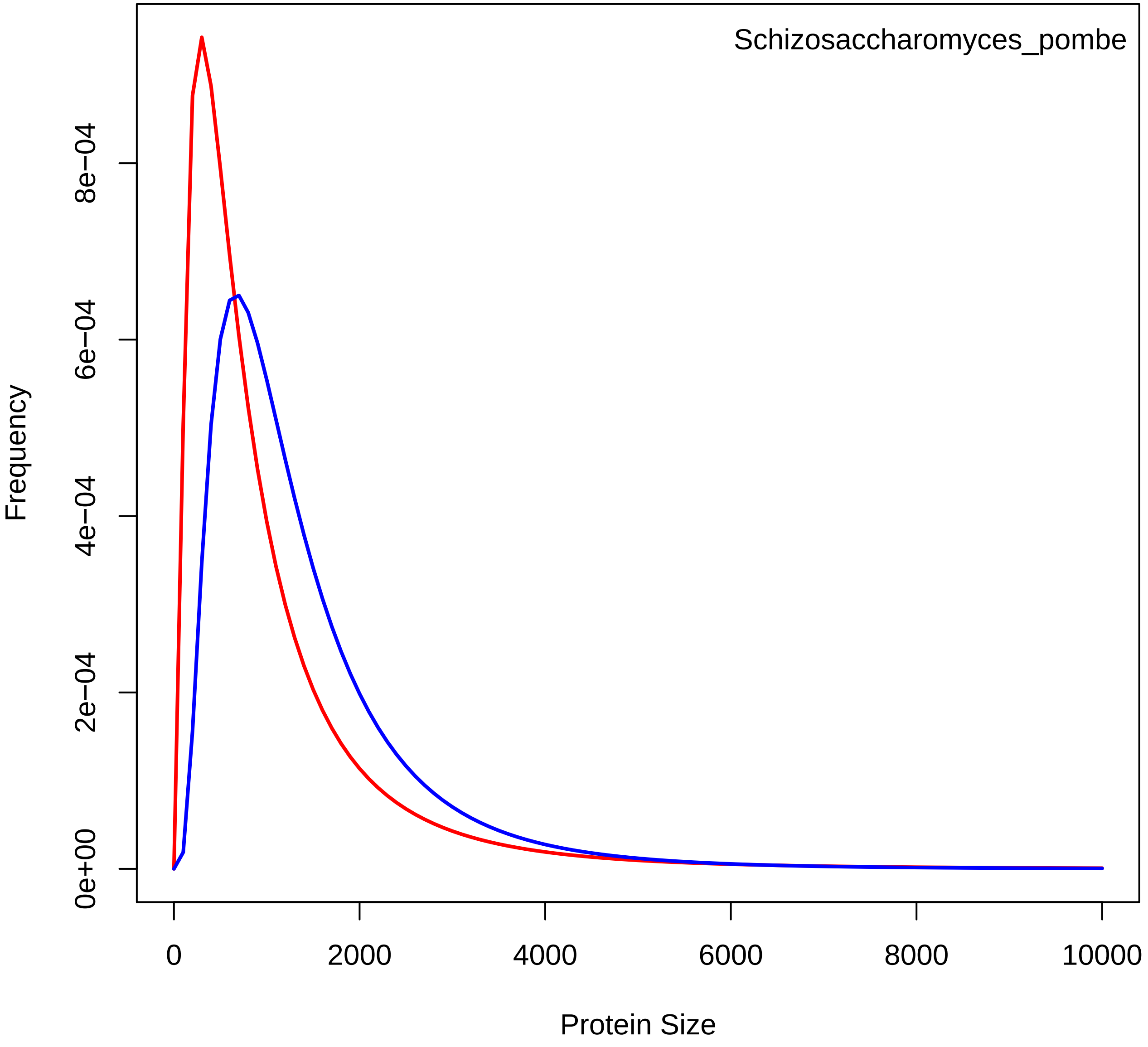

Supplement 3 − Figure 132

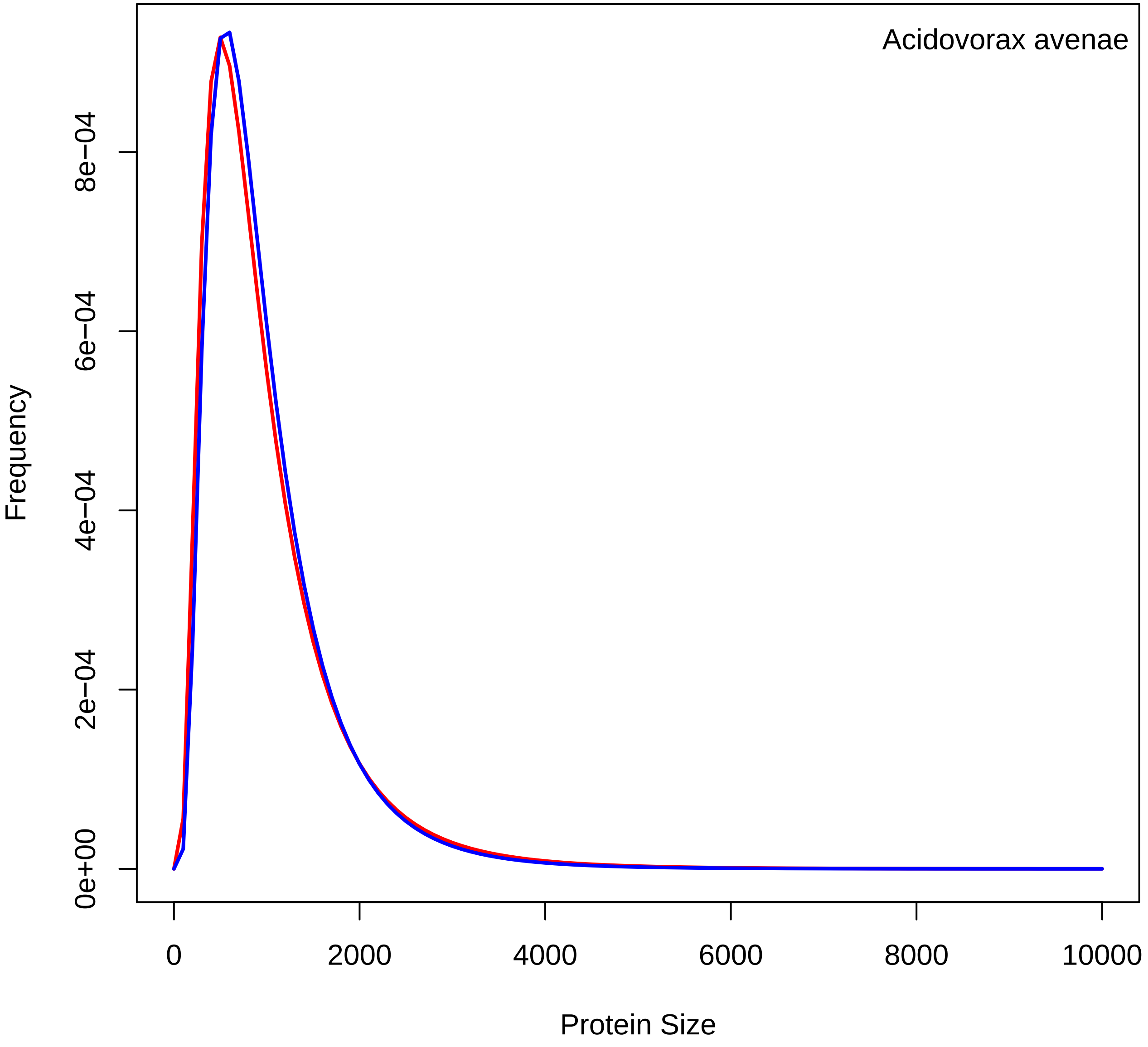

Supplement 3 − Figure 133

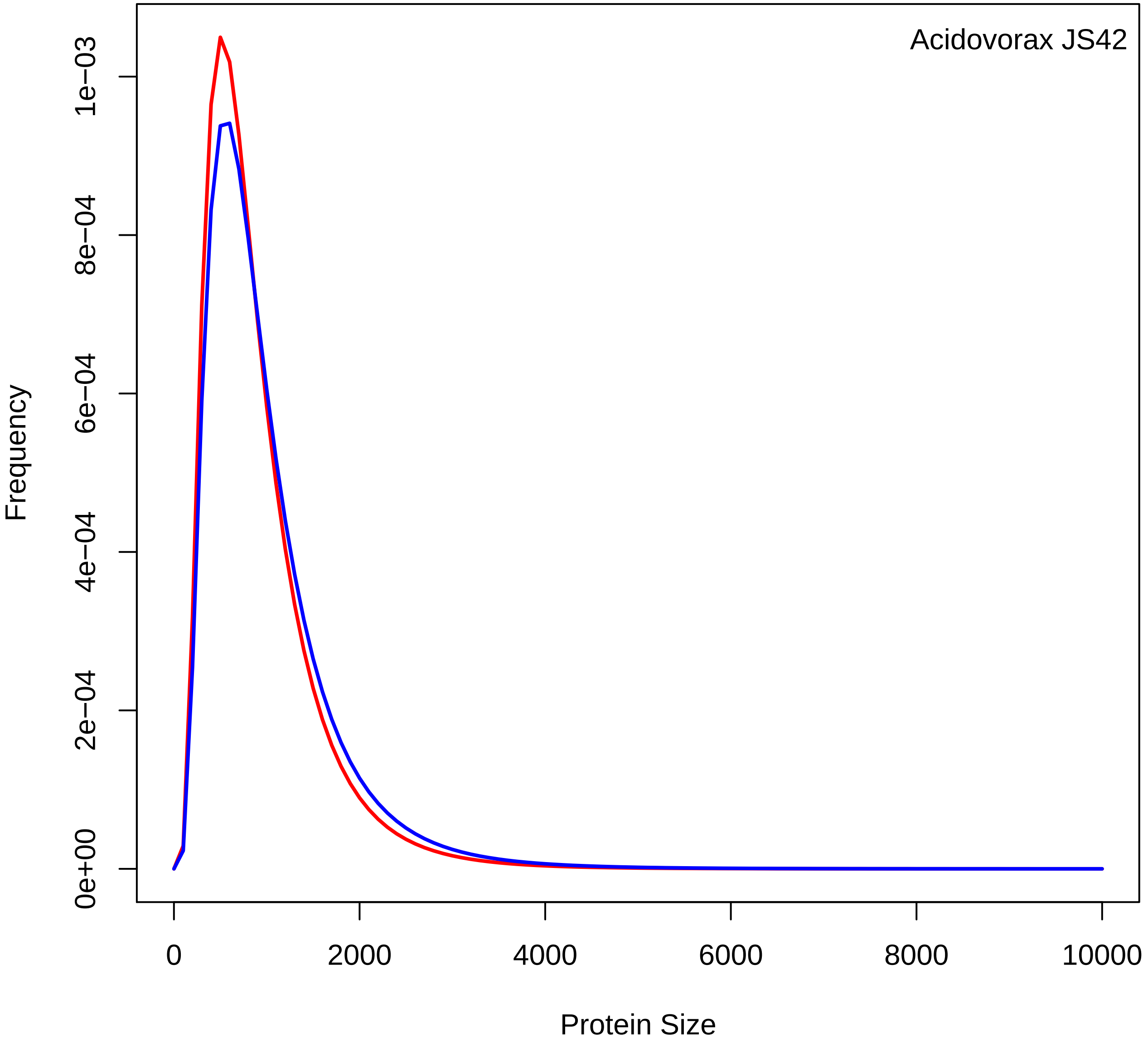

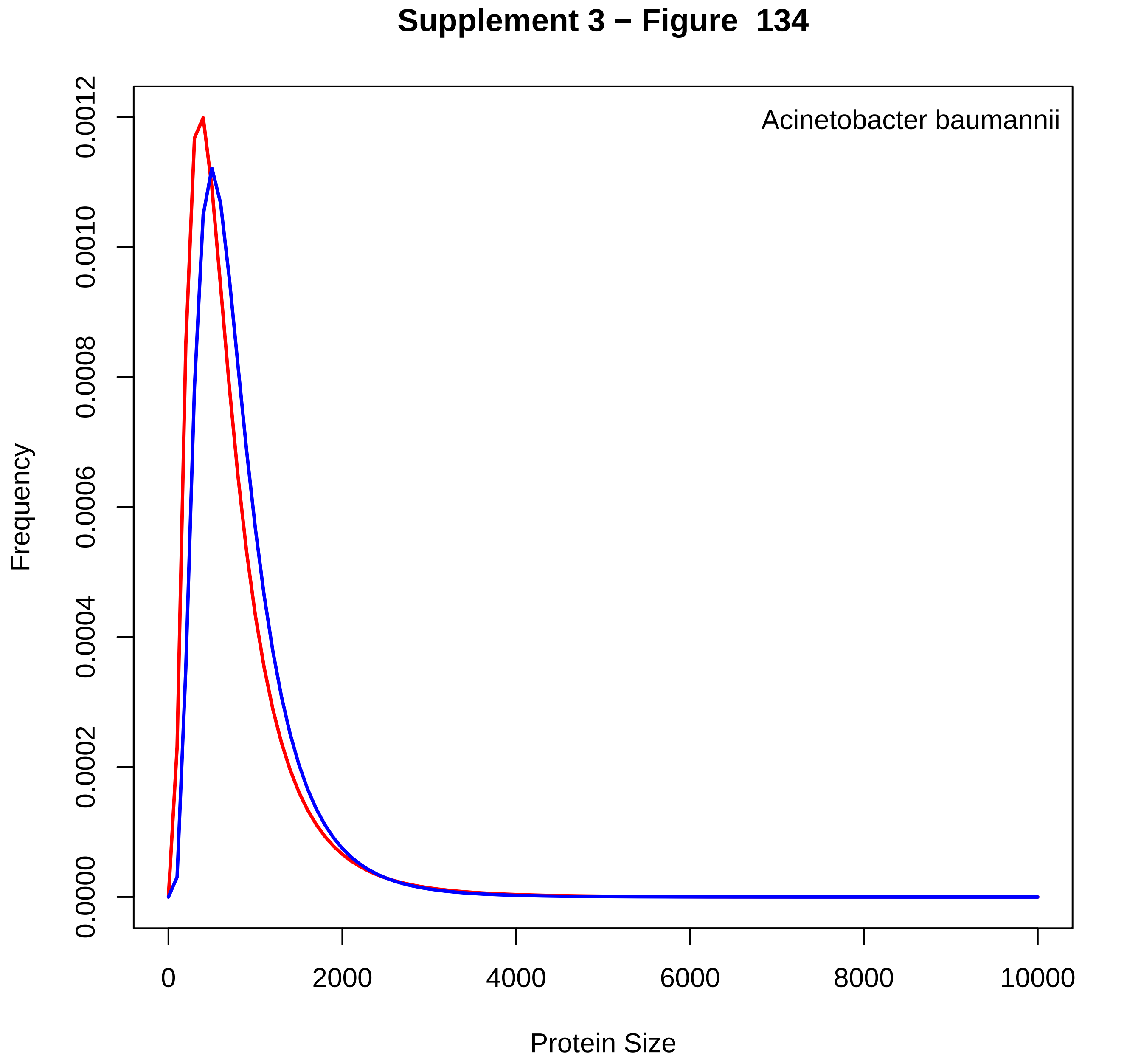

Supplement 3 − Figure 134
Acinetobacter baumannii
Frequency
0.0000
0.0002
0.0004
0.0006
0.0008
0.0010
0.0012
0
2000
4000
6000
8000
10000
Protein Size

Supplement 3 − Figure 135

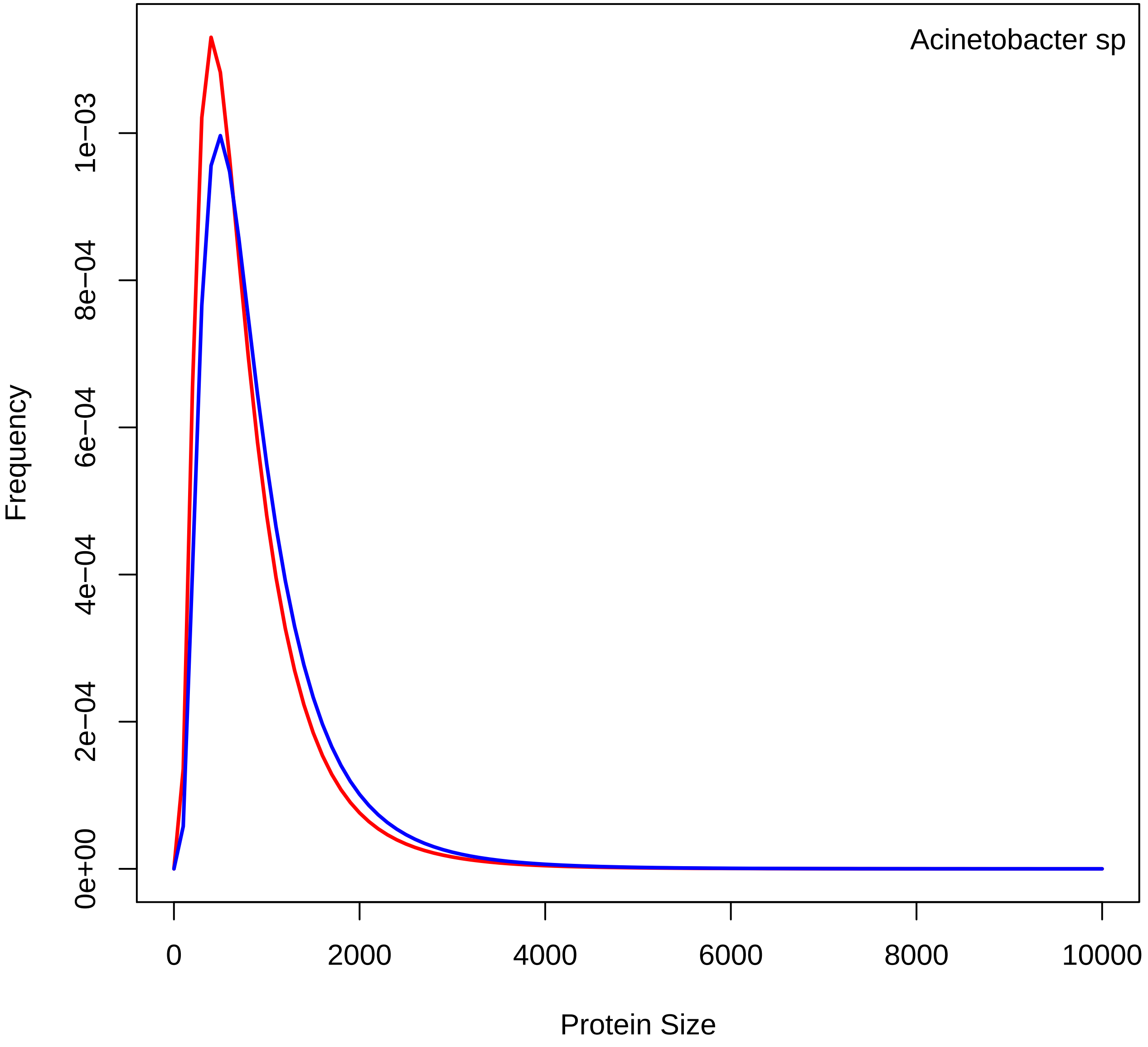

Supplement 3 − Figure 136

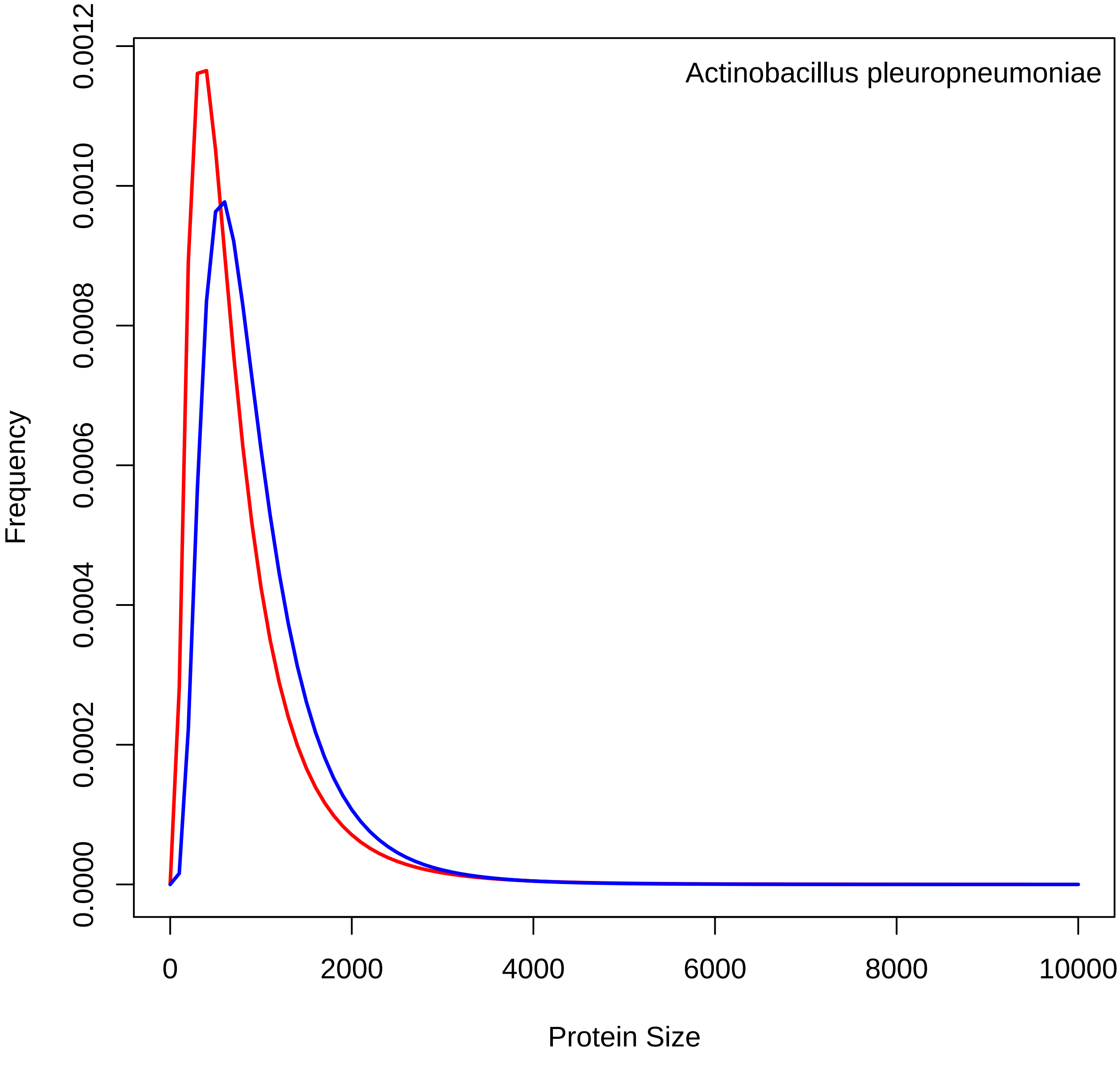

Supplement 3 − Figure 137

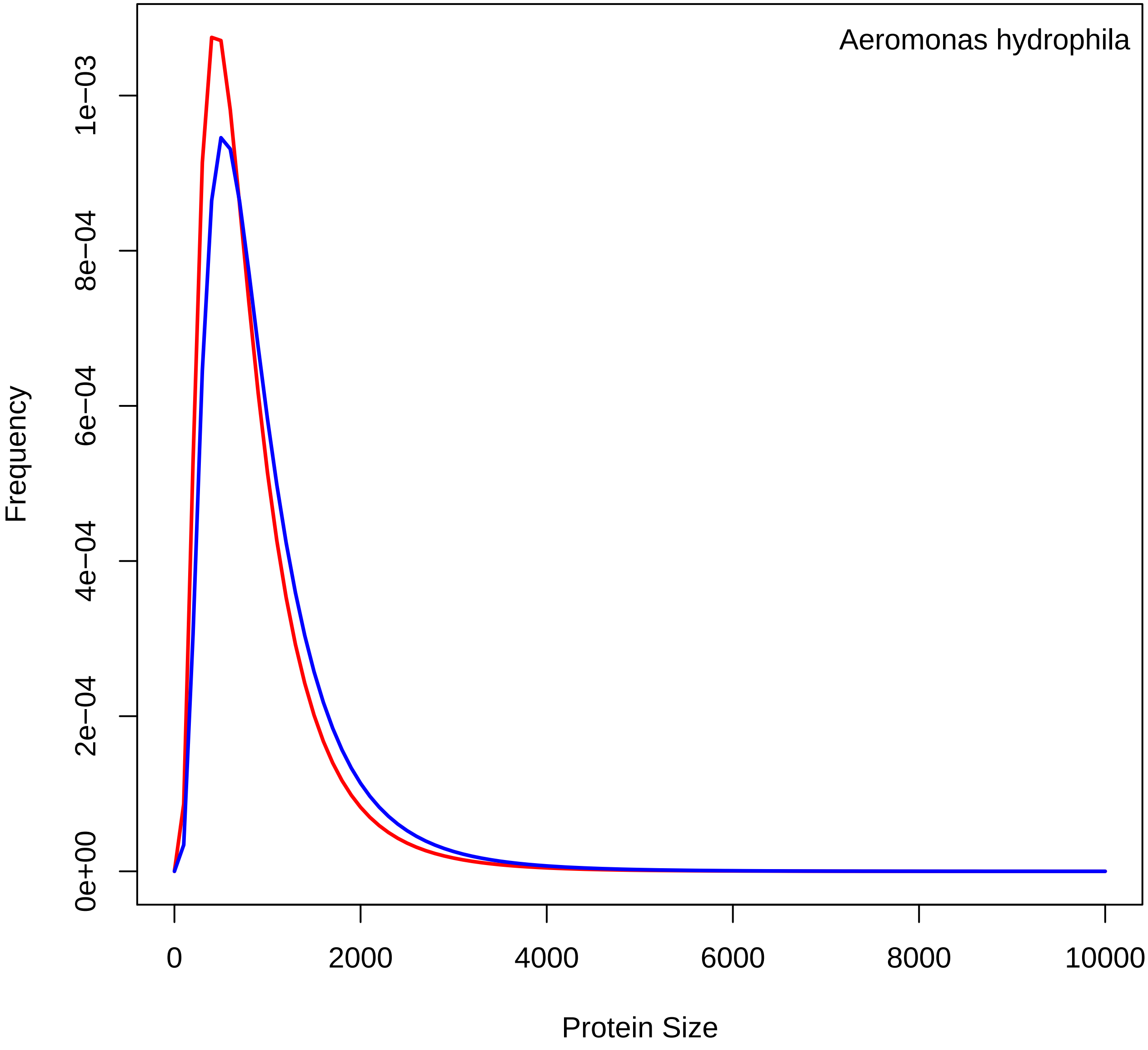

Supplement 3 − Figure 138

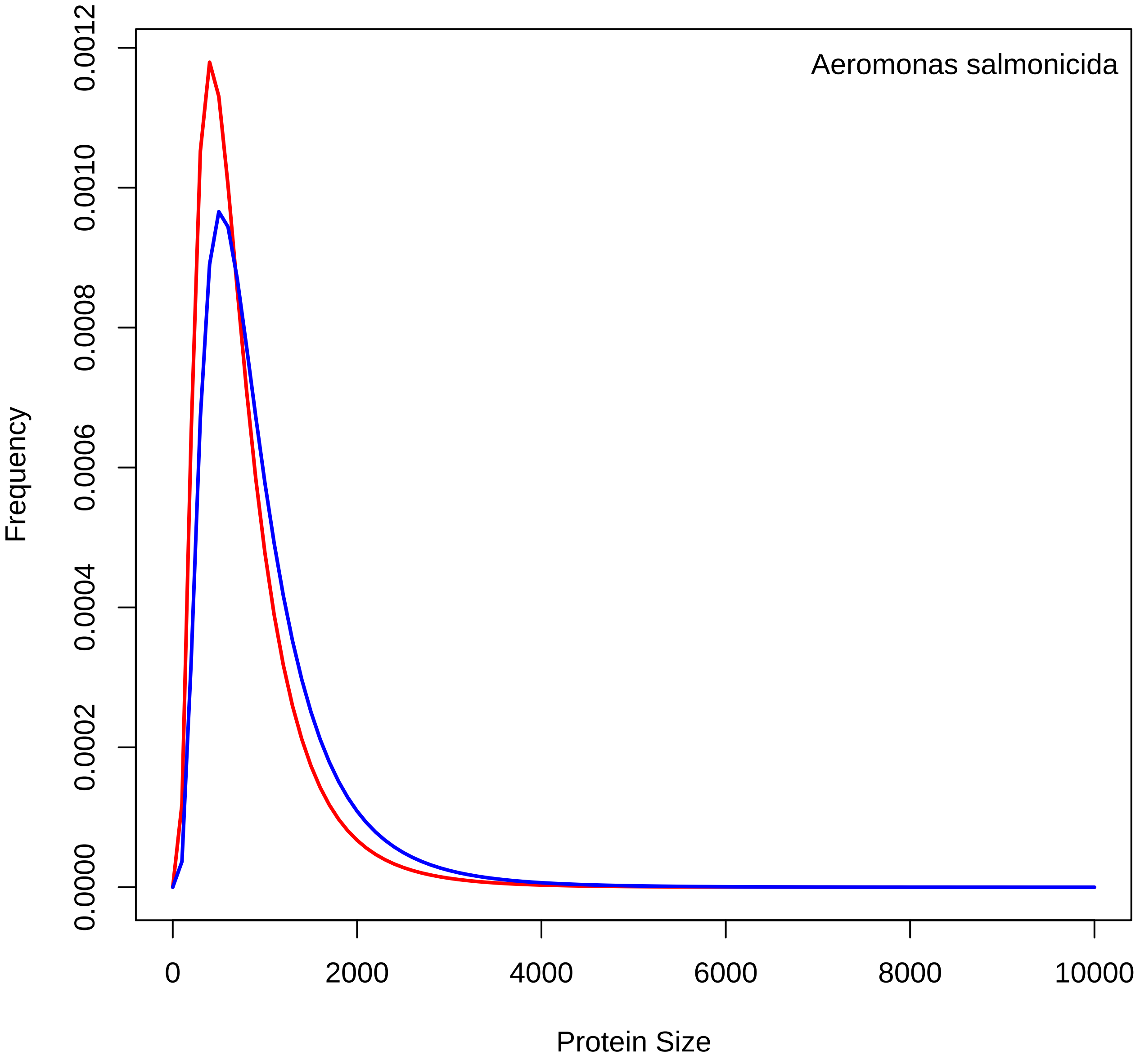

Supplement 3 − Figure 139

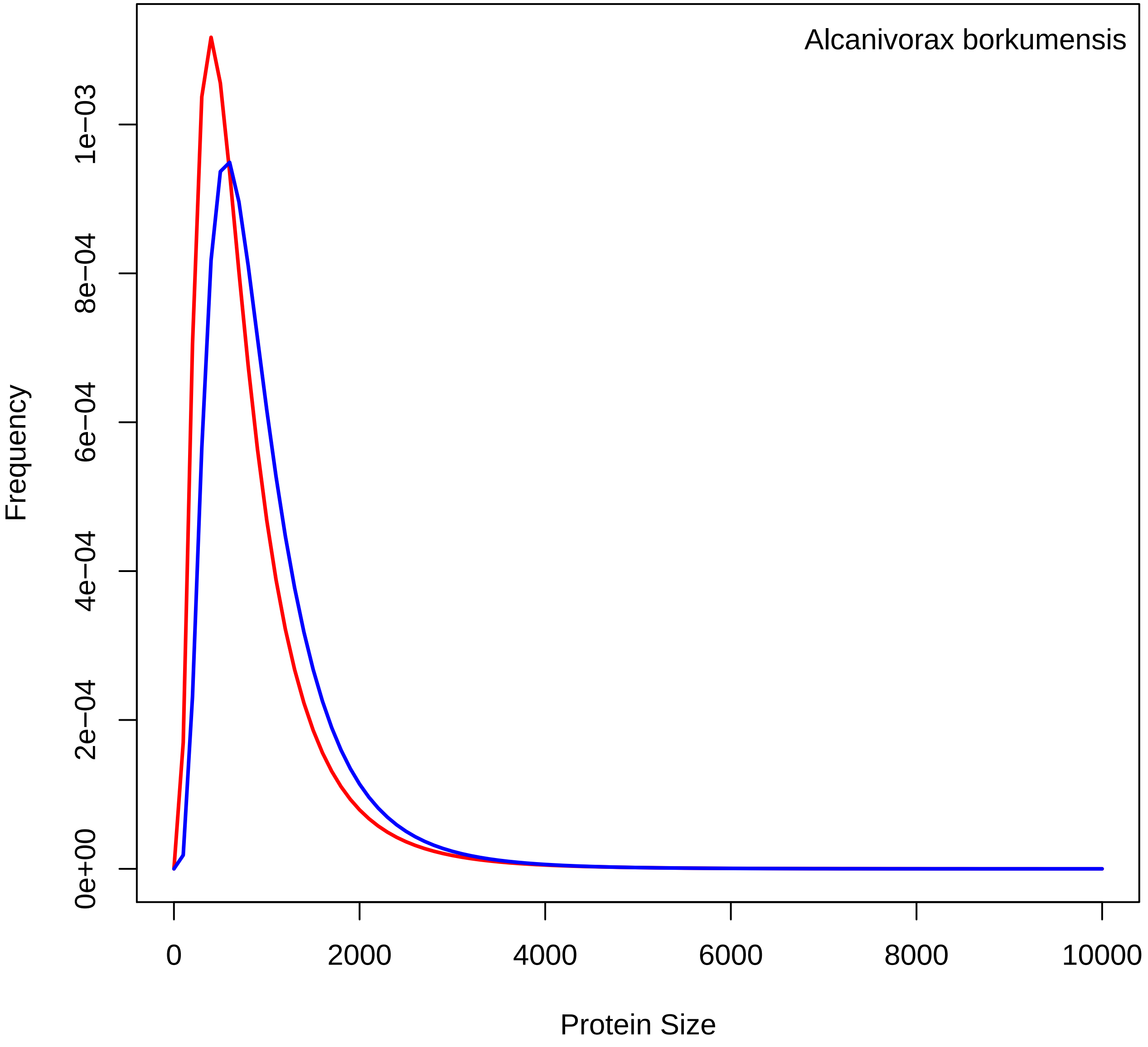

Supplement 3 − Figure 140

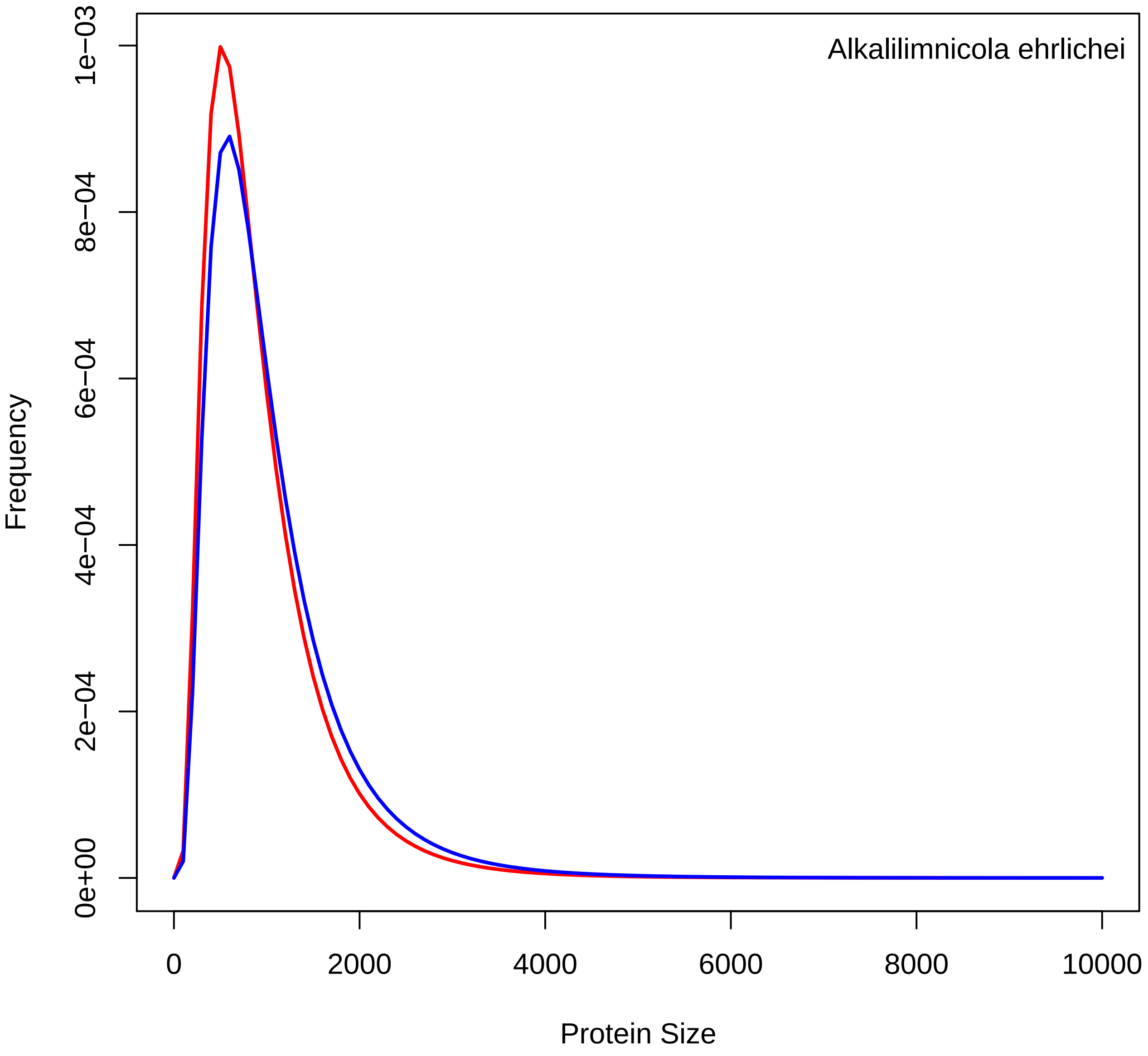

Supplement 3 − Figure 141

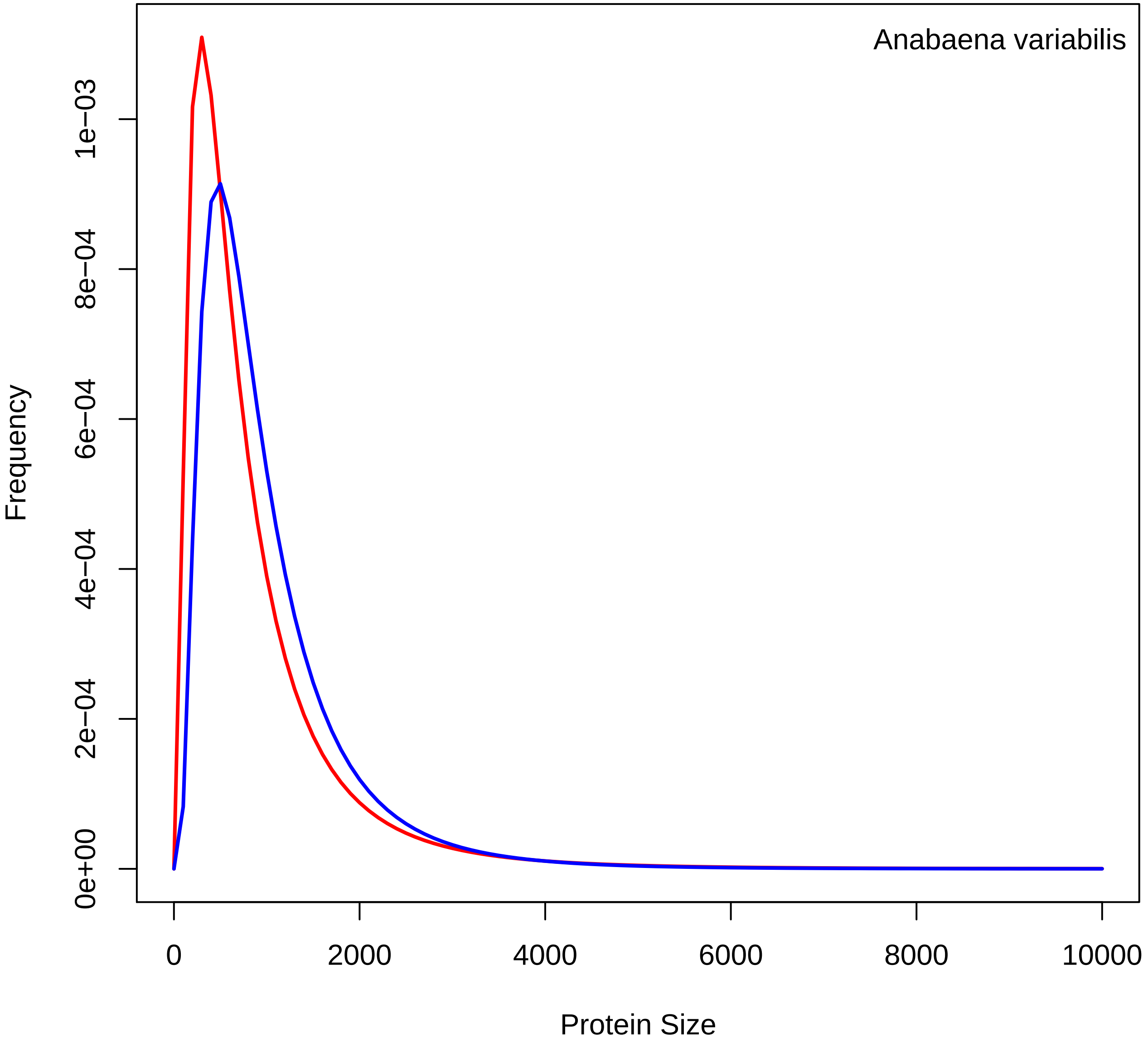

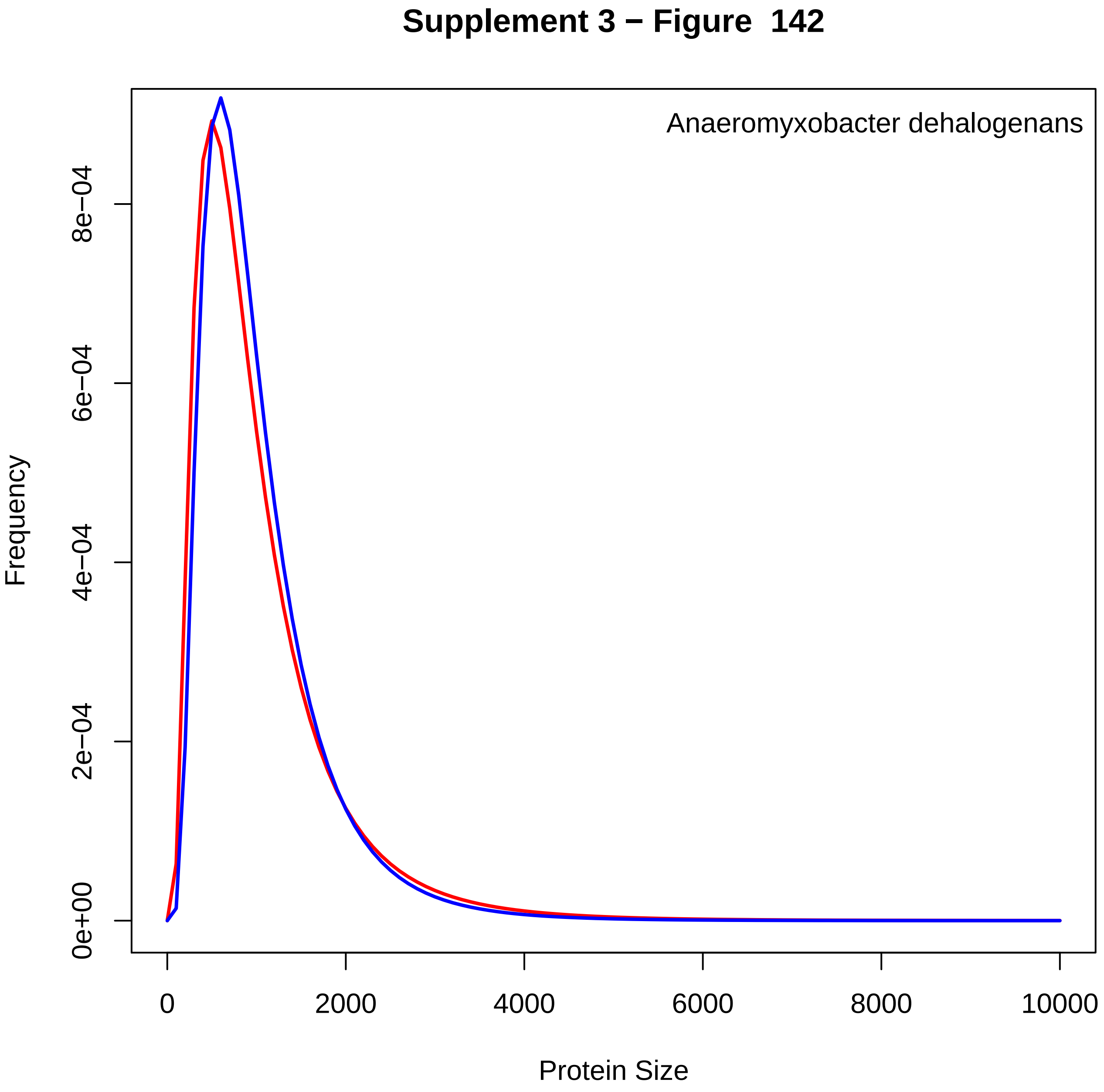

Supplement 3 − Figure 142
Anaeromyxobacter dehalogenans
Frequency
0e+00
2e−04
4e−04
6e−04
8e−04
0
2000
4000
6000
8000
10000
Protein Size

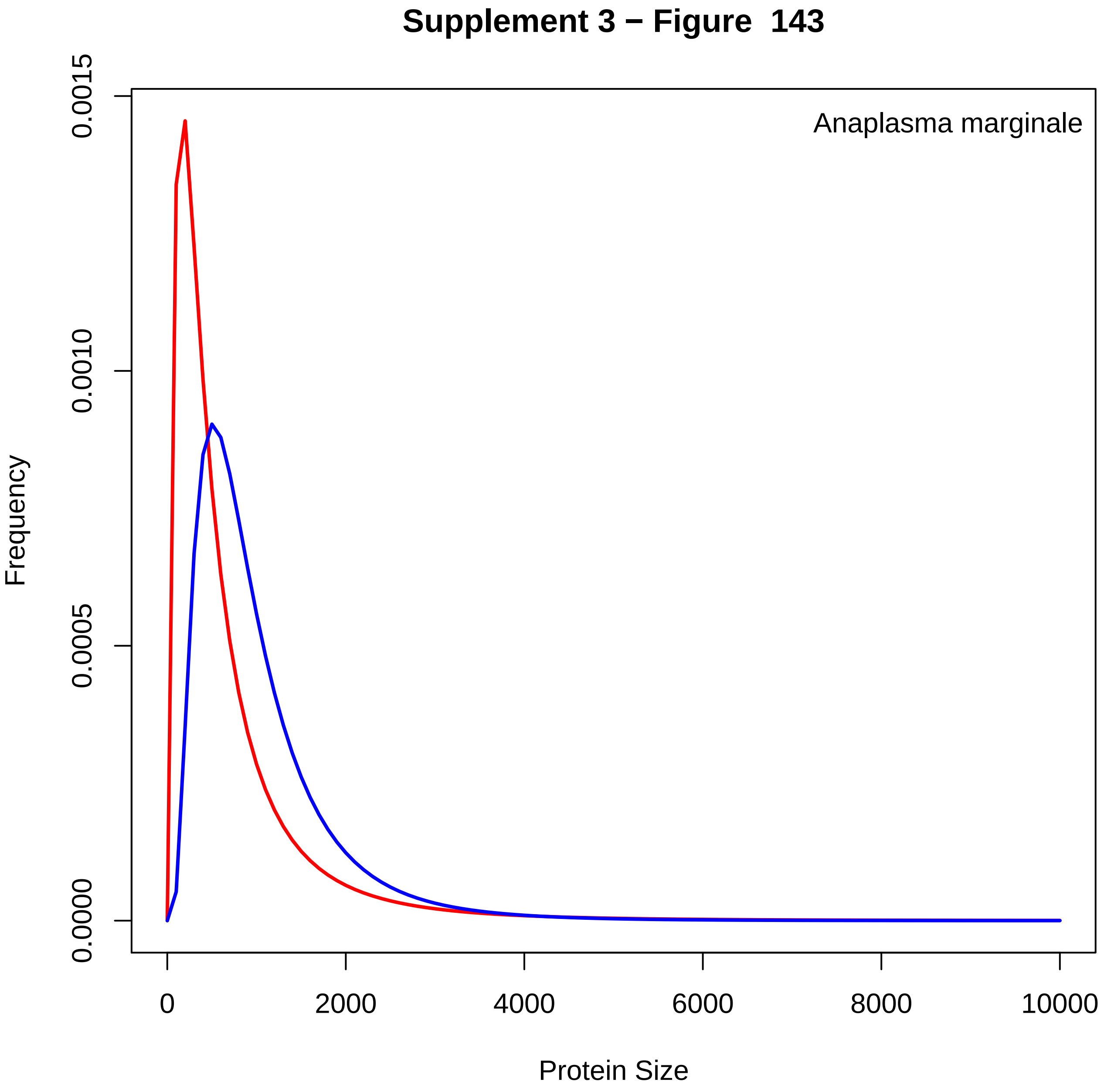
Supplement 3 − Figure 143
Anaplasma marginale
Frequency
0.0000
0.0005
0.0010
0.0015
0
2000
4000
6000
8000
10000
Protein Size

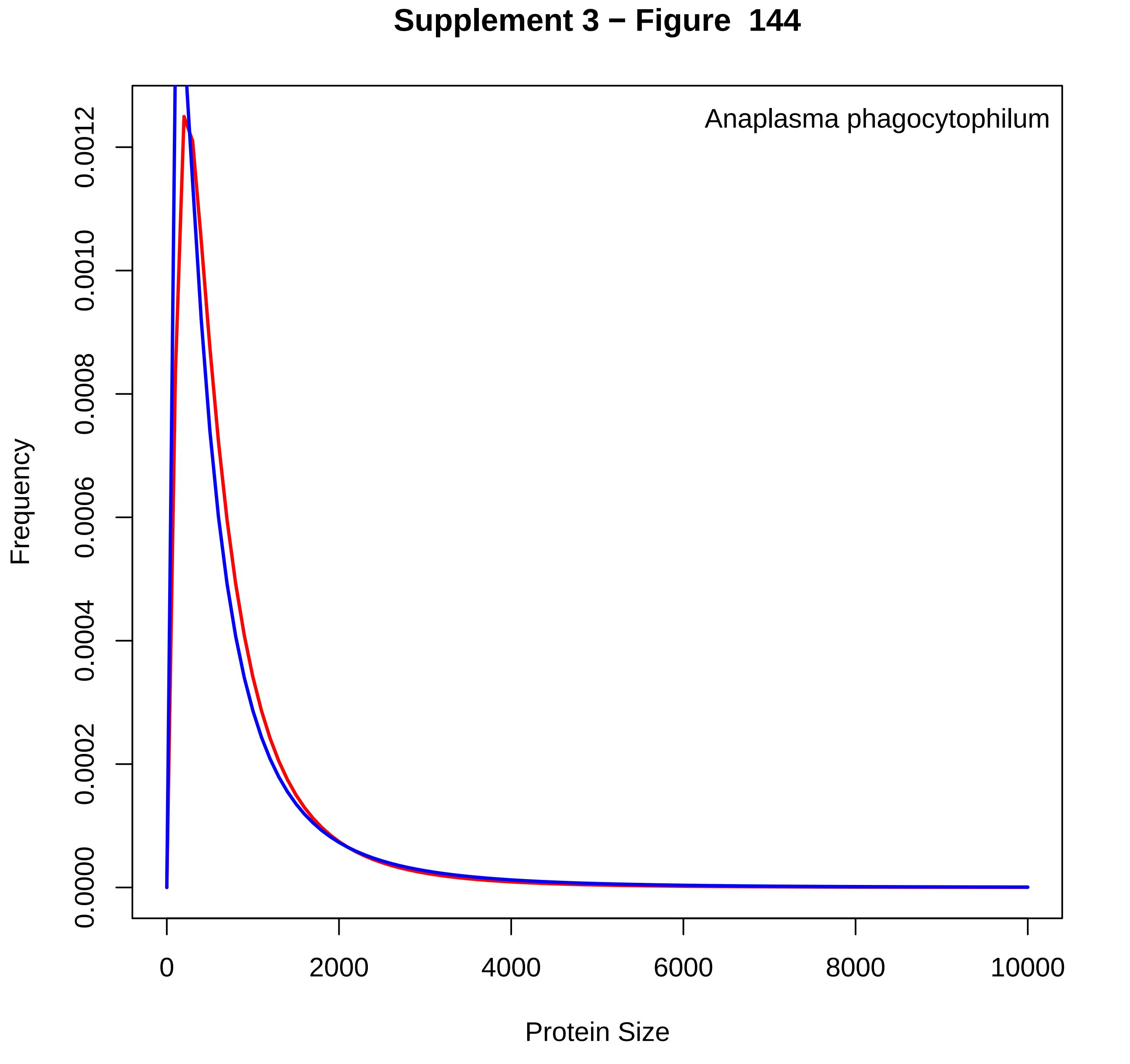
Supplement 3 − Figure 144
Anaplasma phagocytophilum
Frequency
0.0000
0.0002
0.0004
0.0006
0.0008
0.0010
0.0012
0
2000
4000
6000
8000
10000
Protein Size

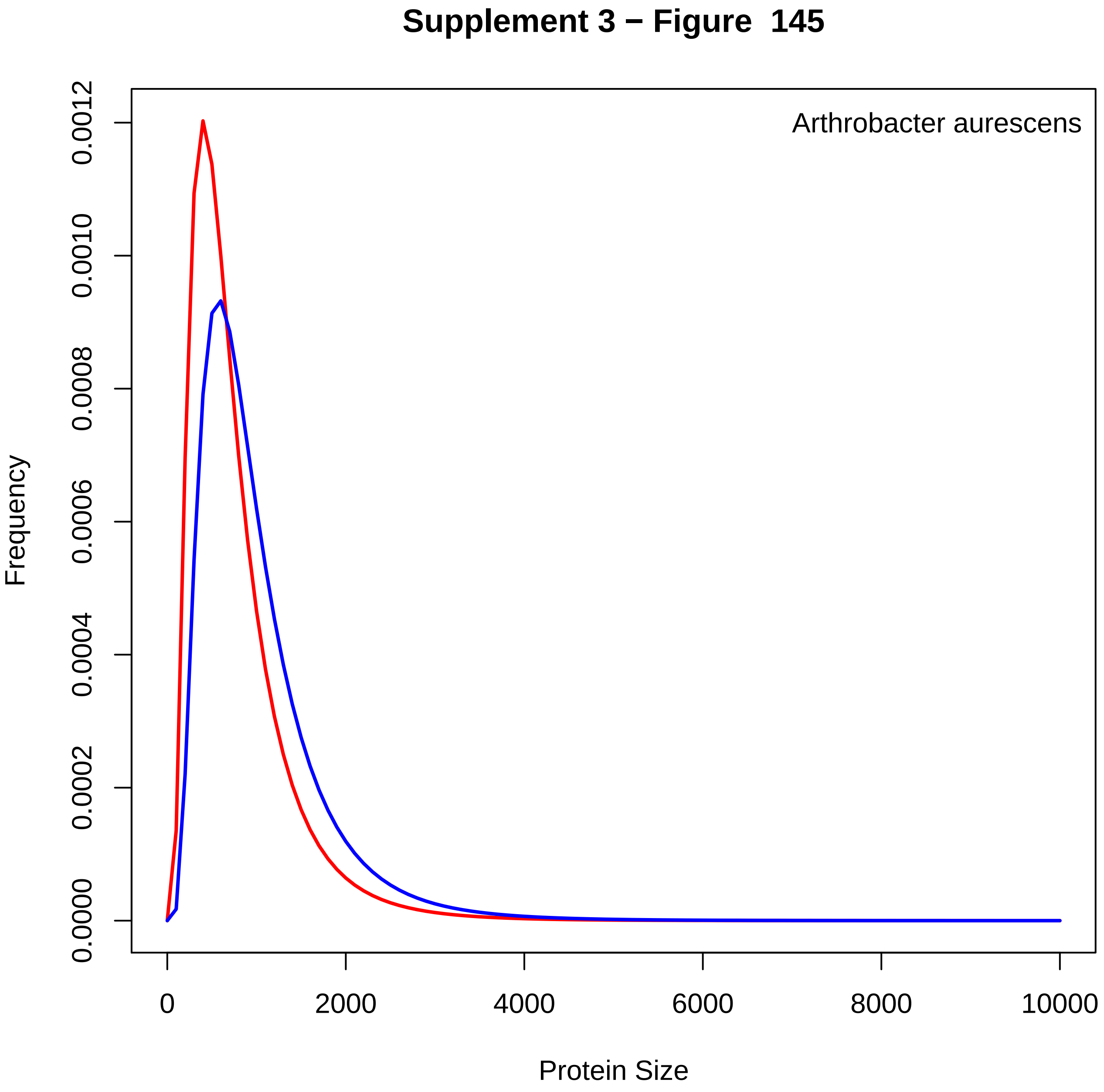
Supplement 3 − Figure 145
Arthrobacter aurescens
Frequency
0.0000
0.0002
0.0004
0.0006
0.0008
0.0010
0.0012
0
2000
4000
6000
8000
10000
Protein Size

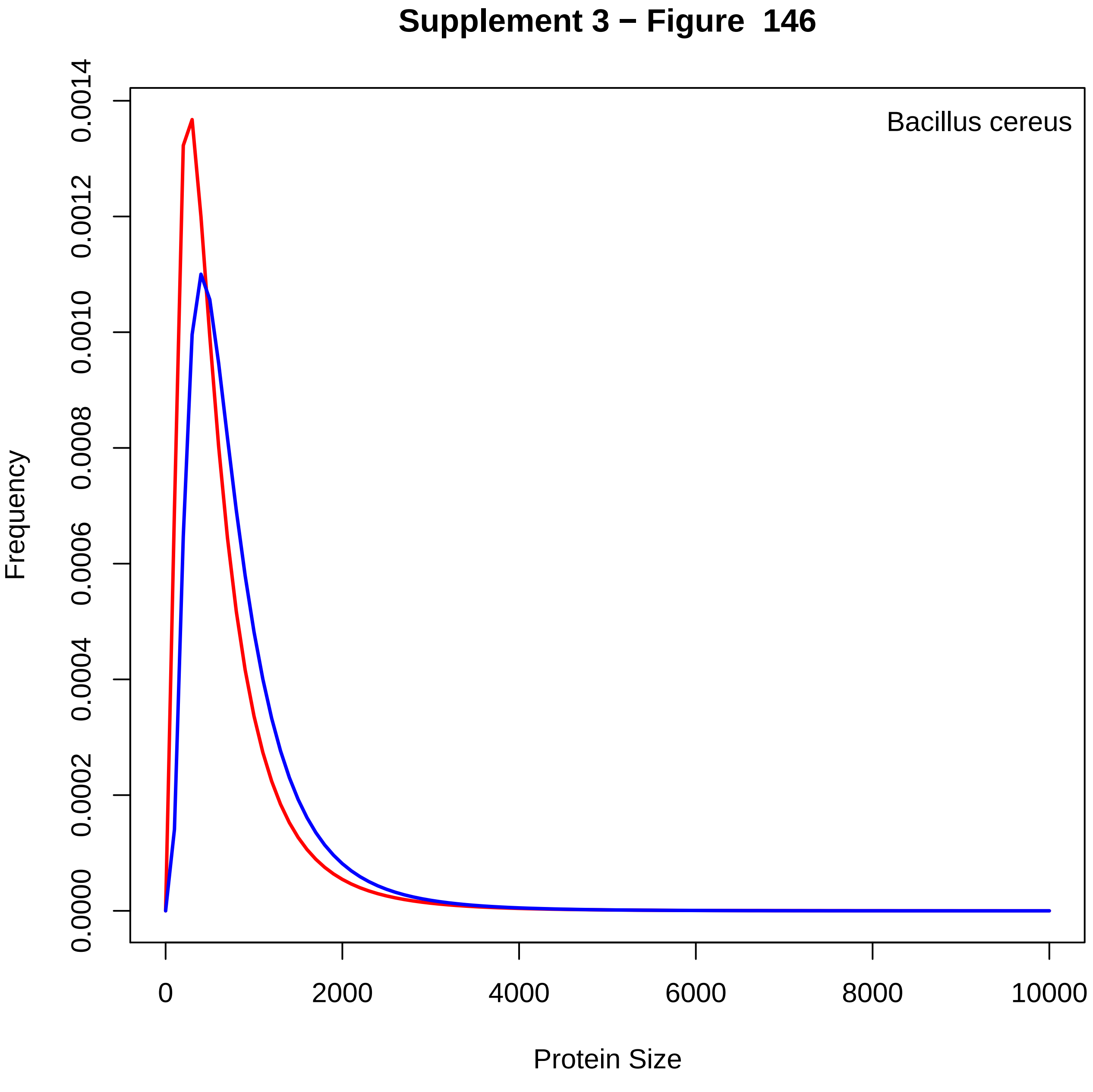

Supplement 3 − Figure 146
Bacillus cereus
Frequency
0.0000
0.0002
0.0004
0.0006
0.0008
0.0010
0.0012
0.0014
0
2000
4000
6000
8000
10000
Protein Size

Supplement 3 − Figure 147

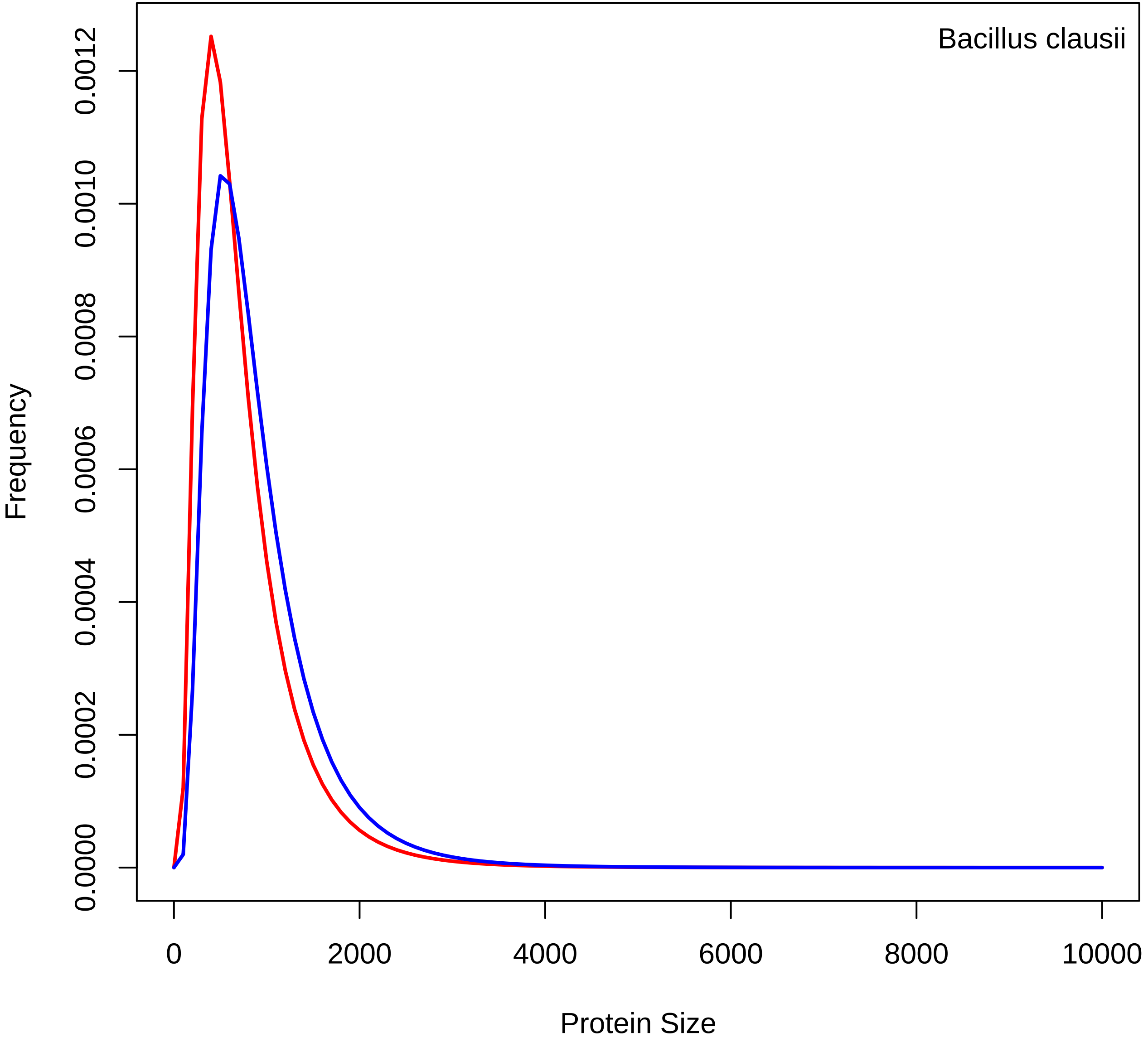

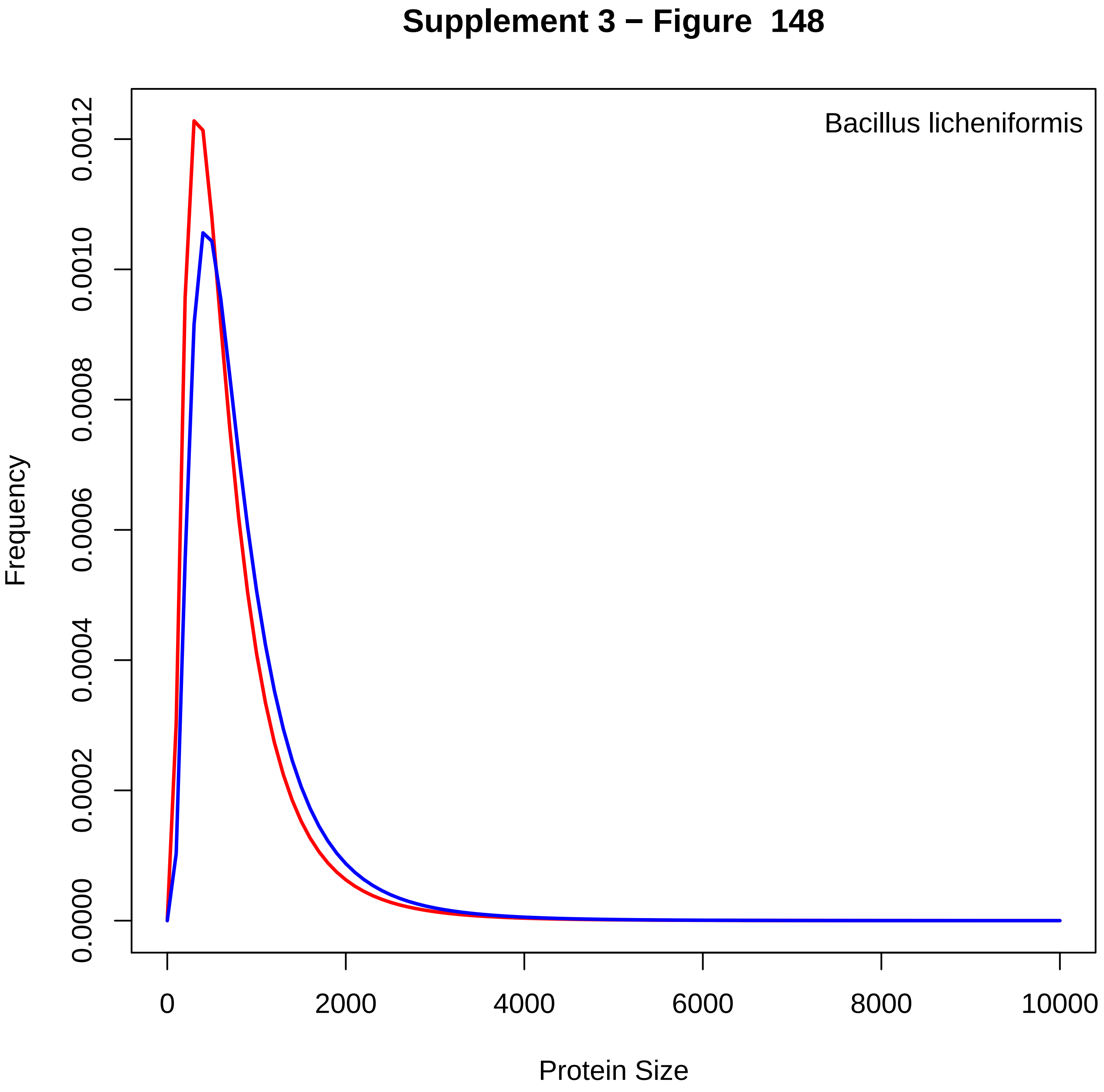
Supplement 3 − Figure 148
Bacillus licheniformis
Frequency
0.0000
0.0002
0.0004
0.0006
0.0008
0.0010
0.0012
0
2000
4000
6000
8000
10000
Protein Size

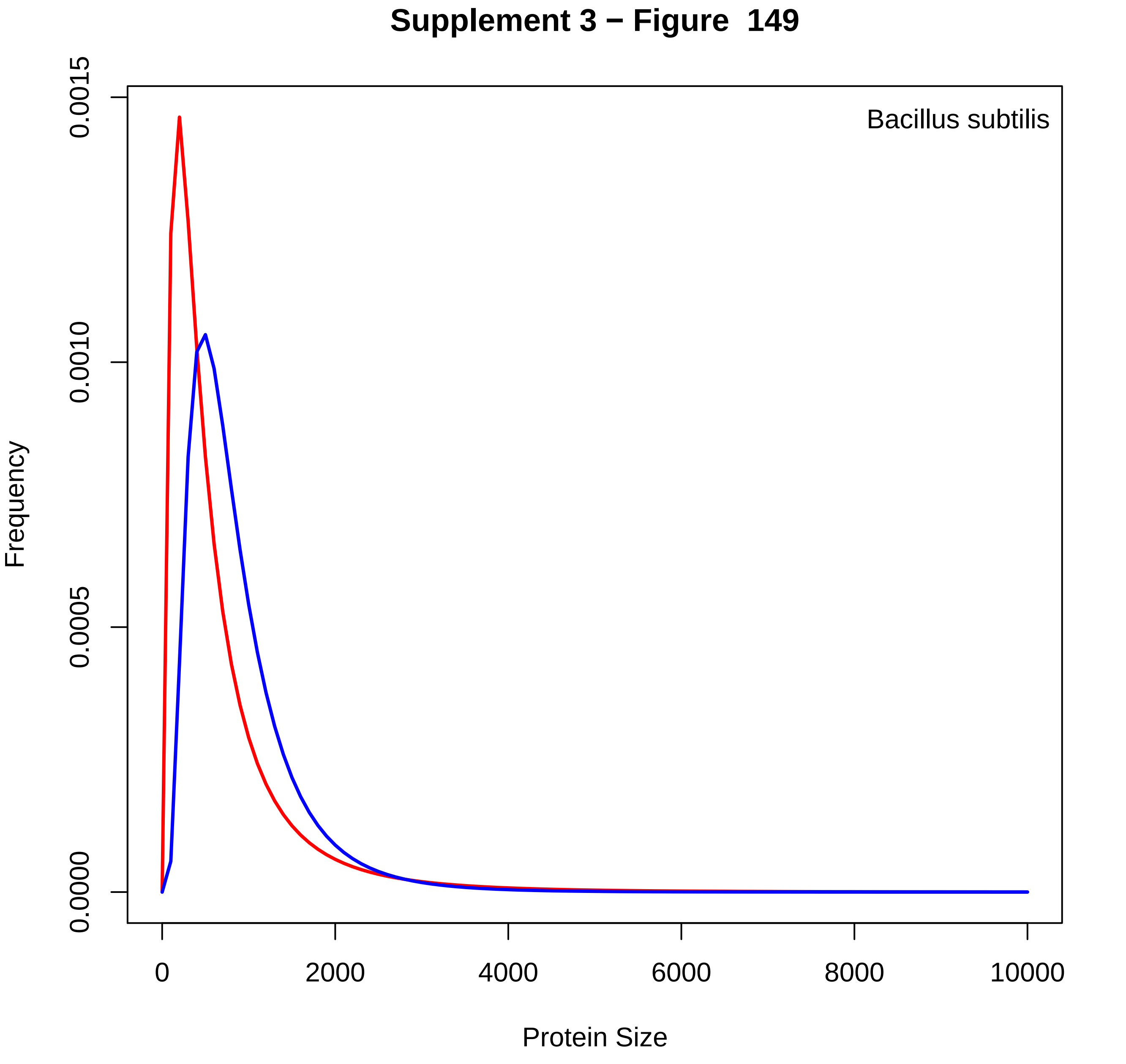

Supplement 3 − Figure 149
Bacillus subtilis
Frequency
0.0000
0.0005
0.0010
0.0015
0
2000
4000
6000
8000
10000
Protein Size

Supplement 3 − Figure 150

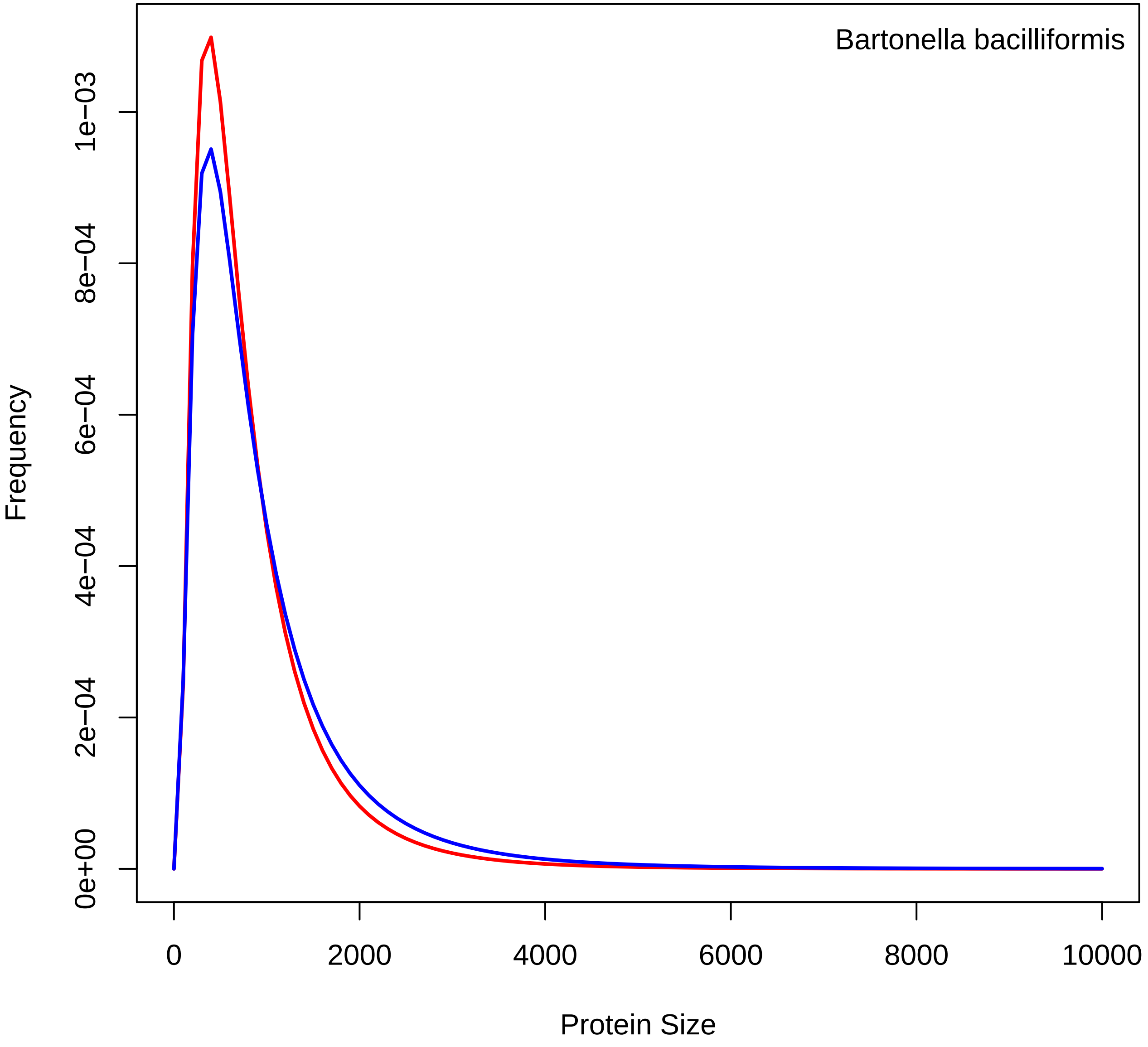

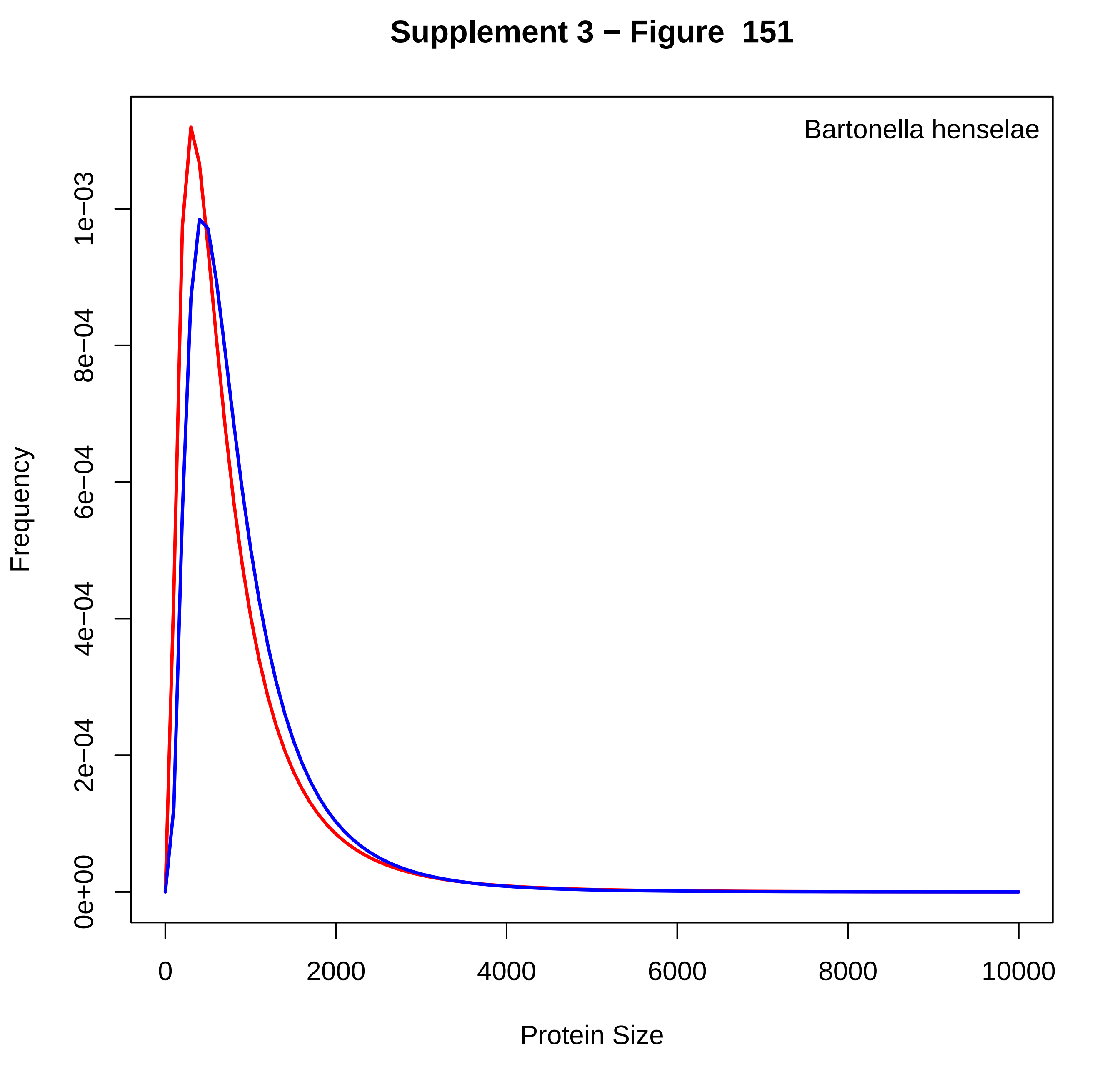

Supplement 3 − Figure 151
Bartonella henselae
Frequency
0e+00
2e−04
4e−04
6e−04
8e−04
1e−03
0
2000
4000
6000
8000
10000
Protein Size

Supplement 3 − Figure 152

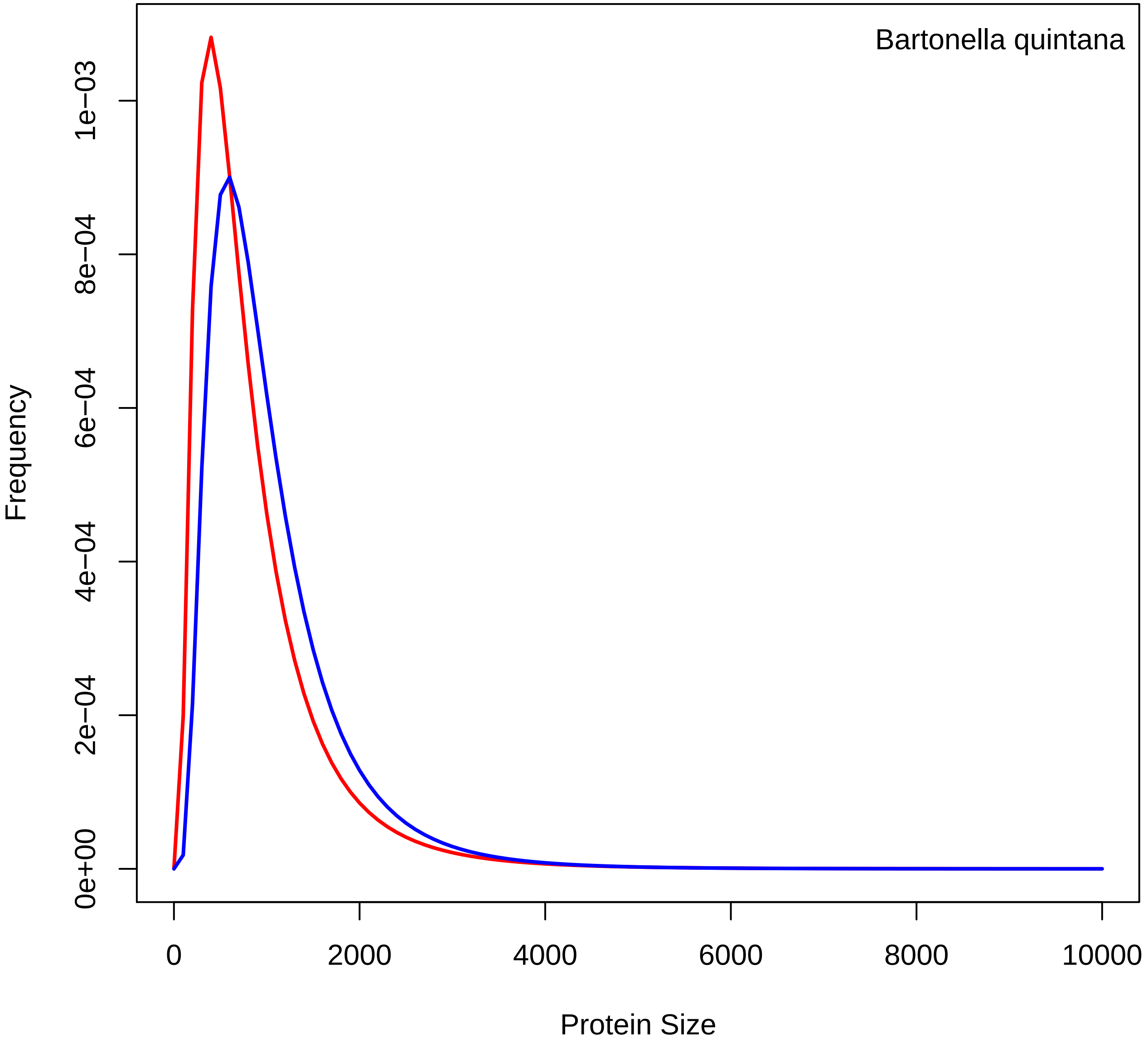

Supplement 3 − Figure 153

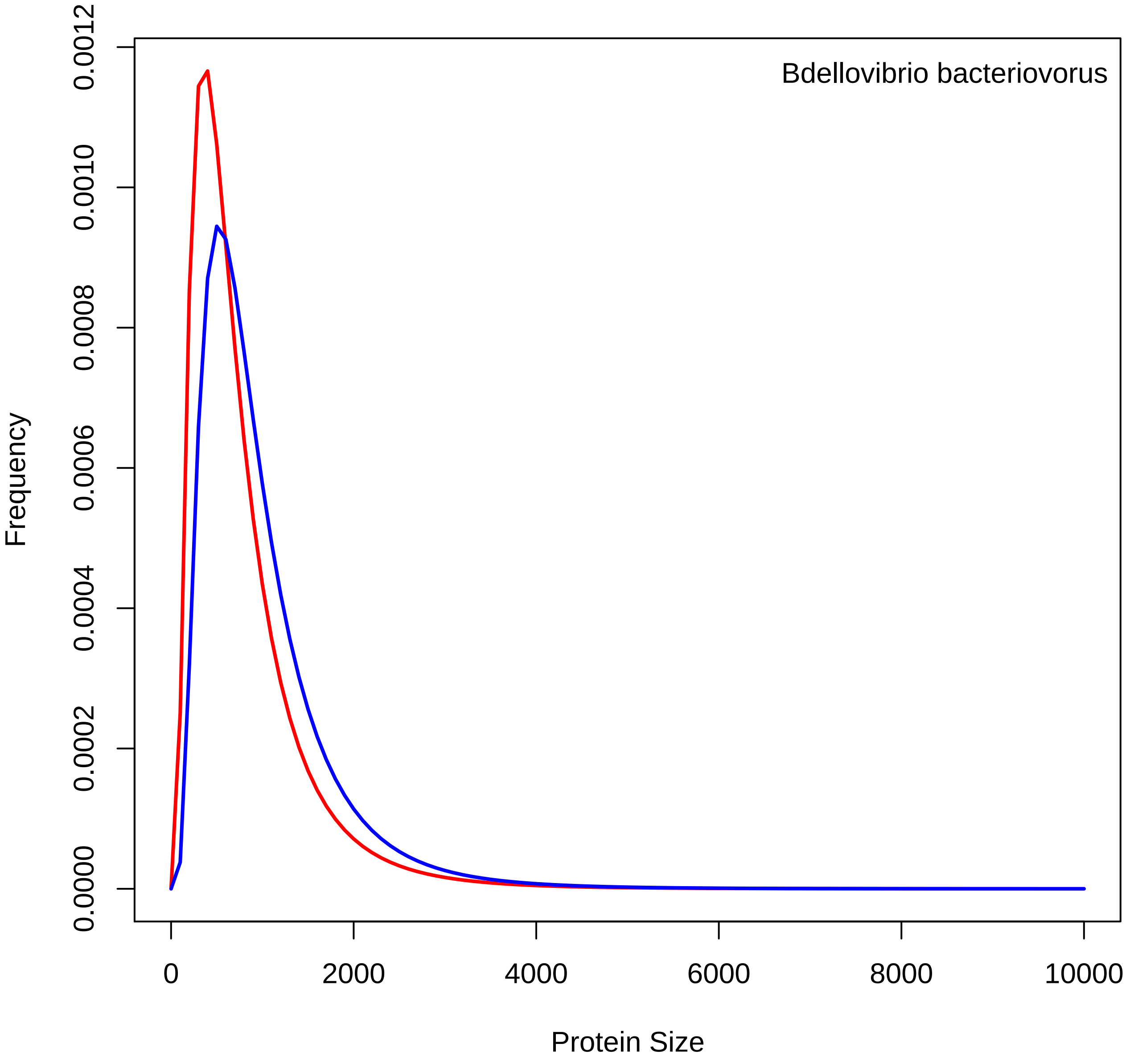

Supplement 3 − Figure 154

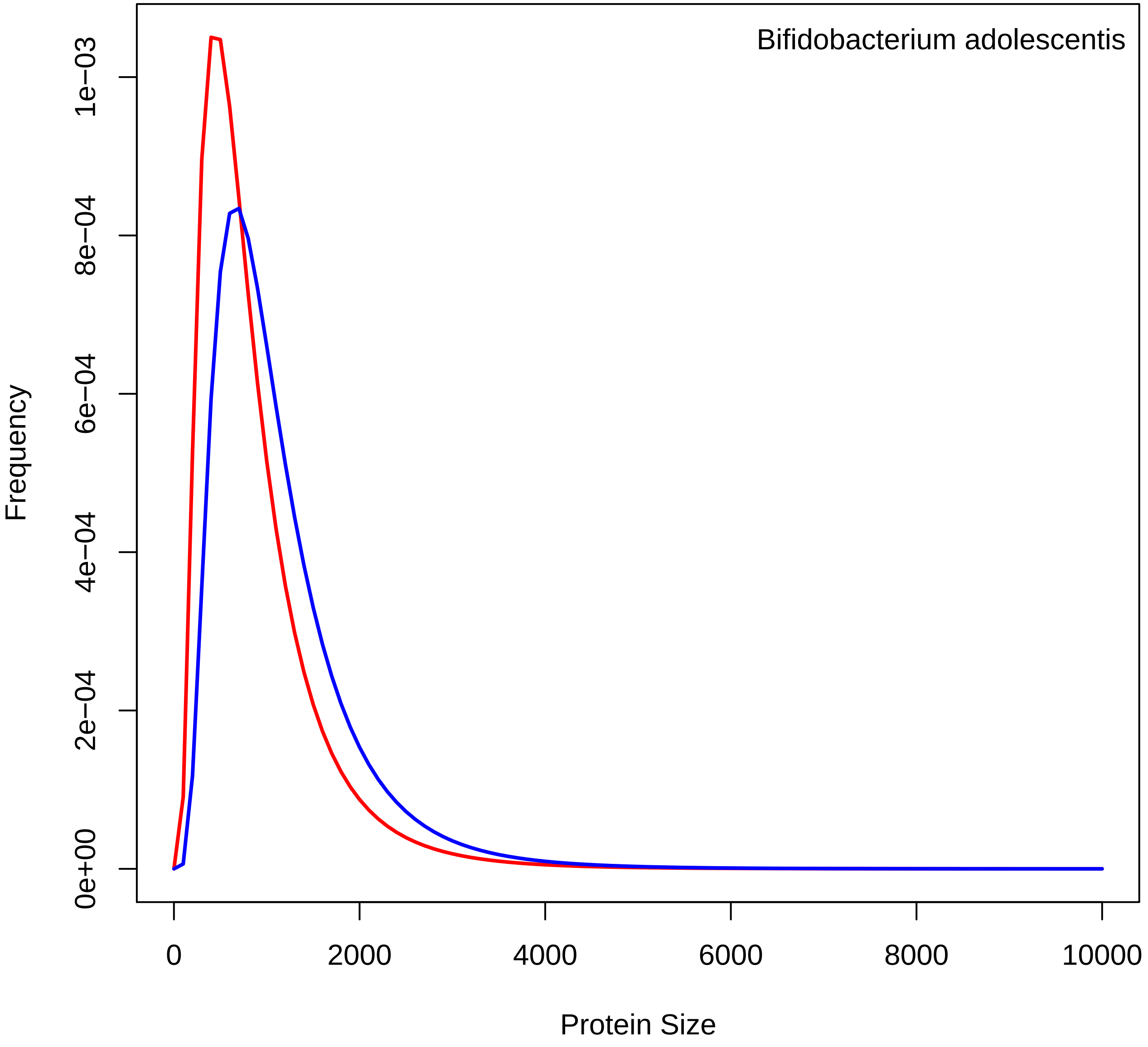

Supplement 3 − Figure 155

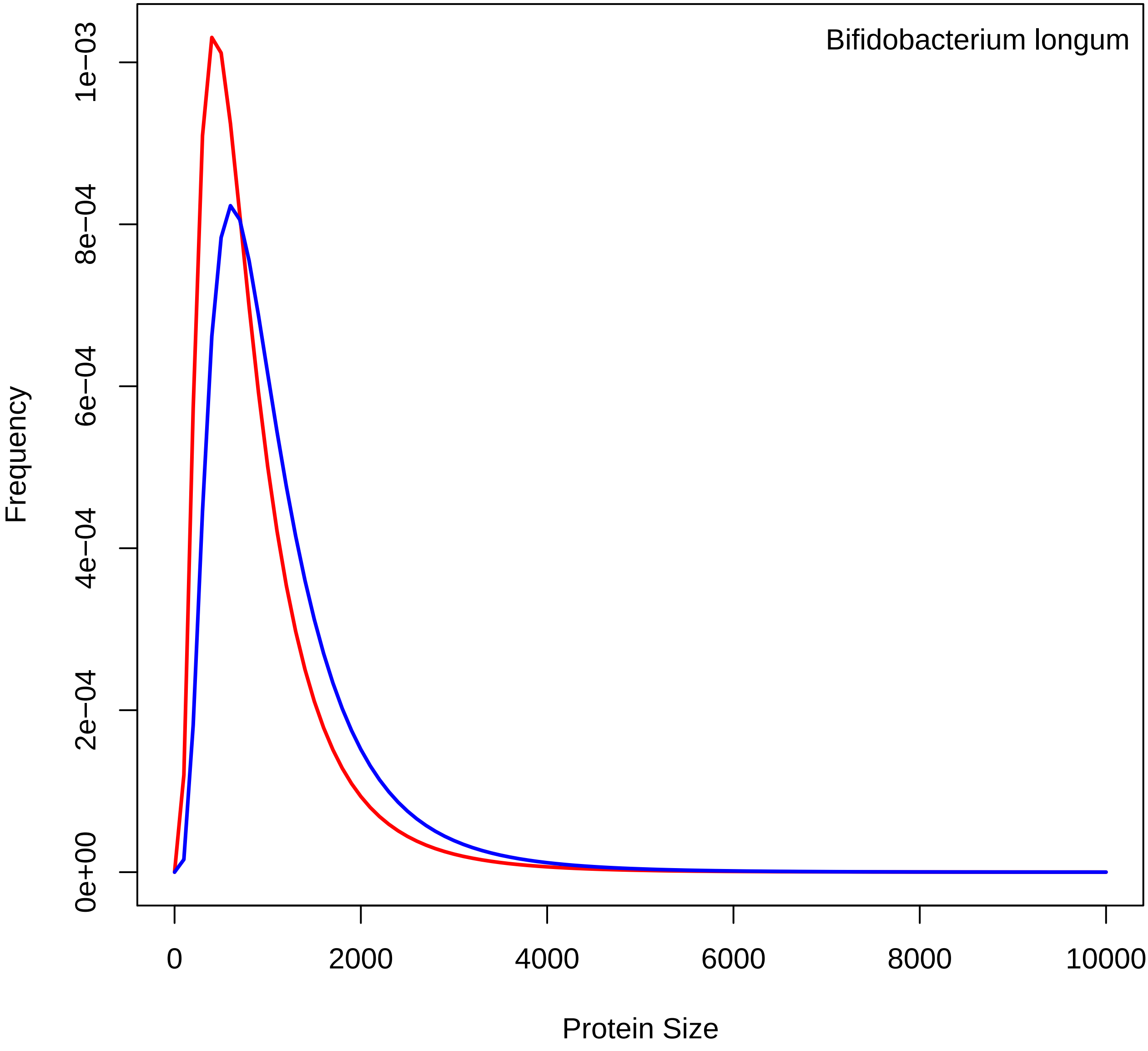

Supplement 3 − Figure 156

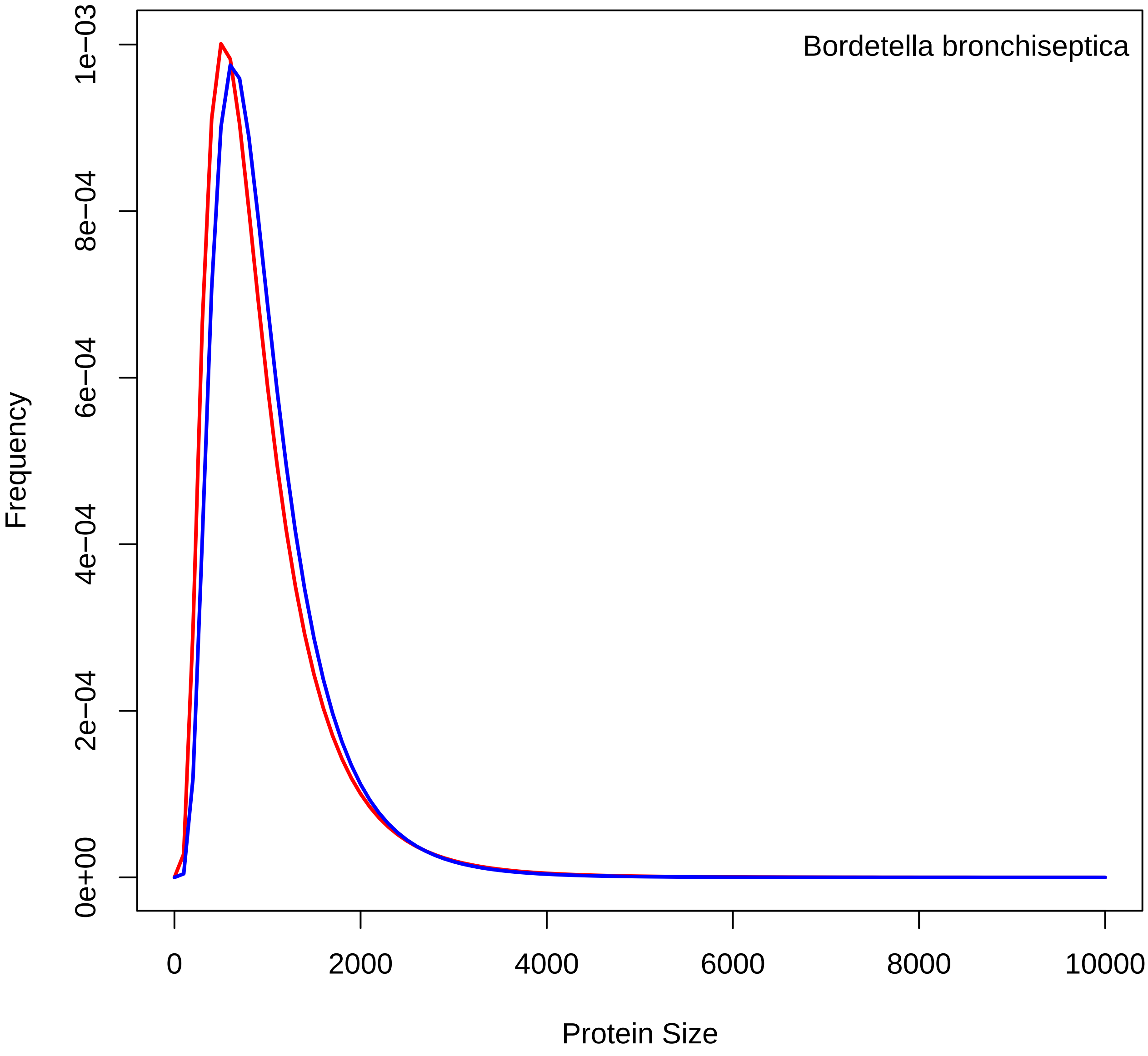

Supplement 3 − Figure 157

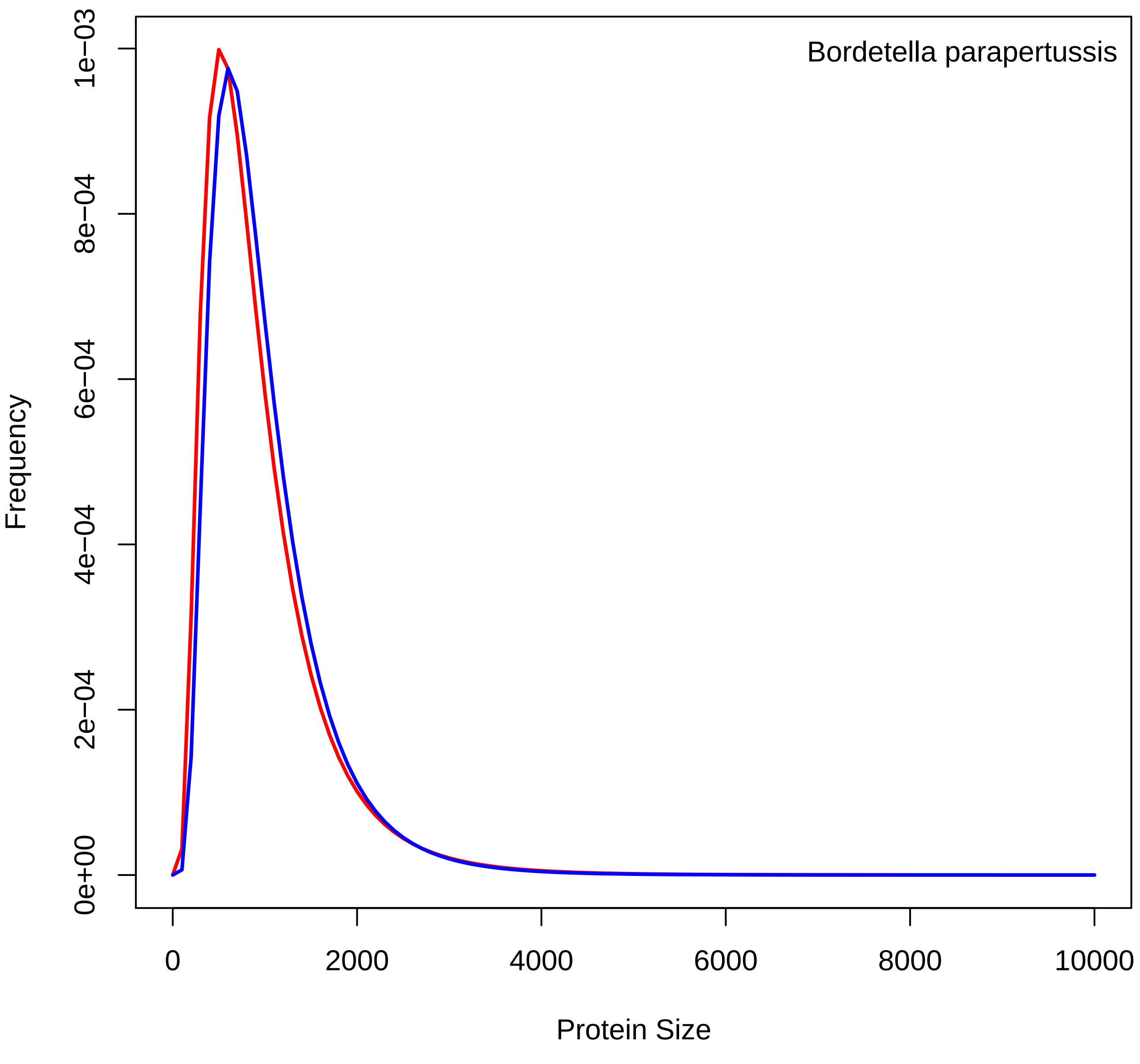

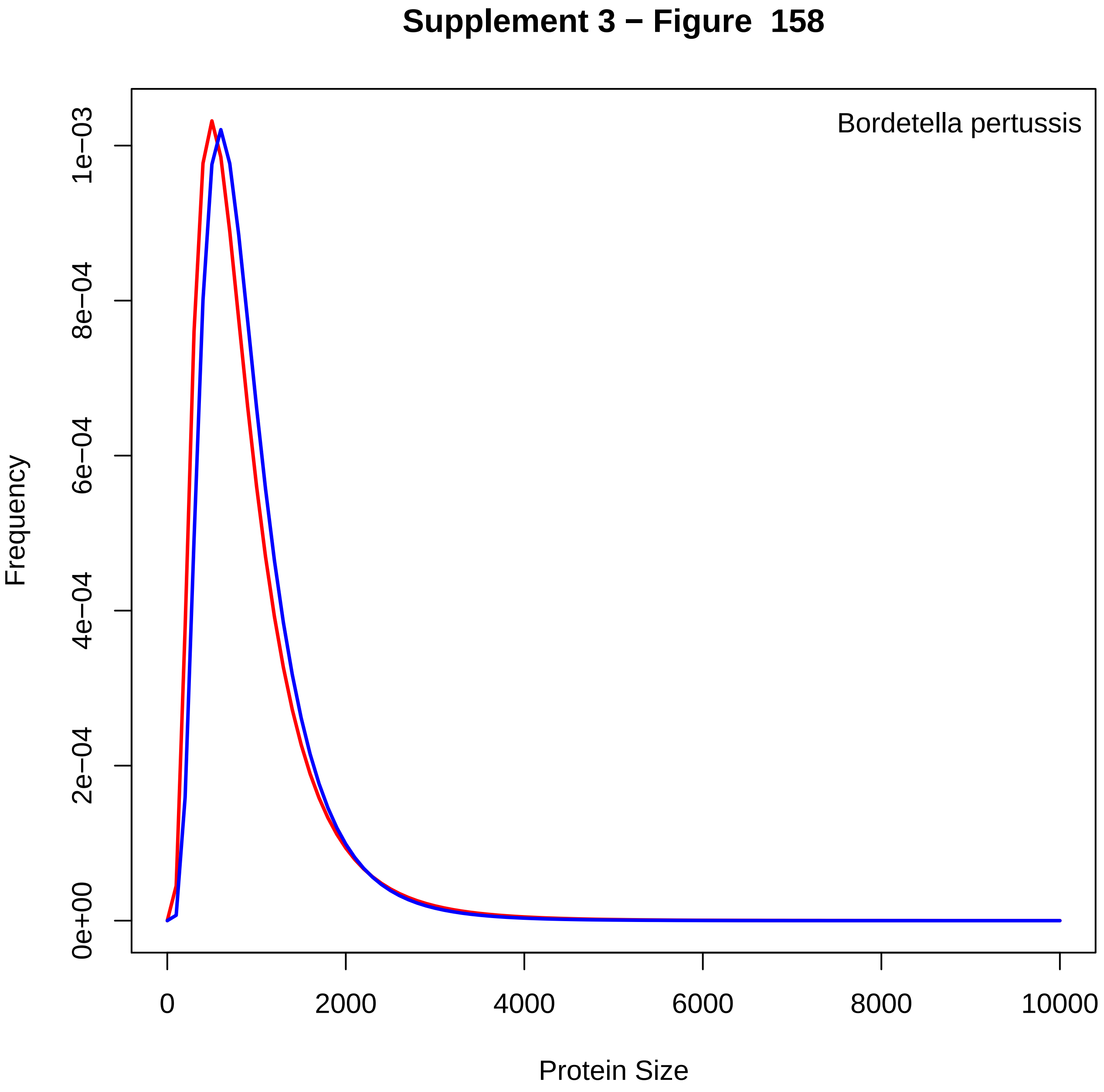
Supplement 3 − Figure 158
Bordetella pertussis
Frequency
0e+00
2e−04
4e−04
6e−04
8e−04
1e−03
0
2000
4000
6000
8000
10000
Protein Size

Supplement 3 − Figure 159

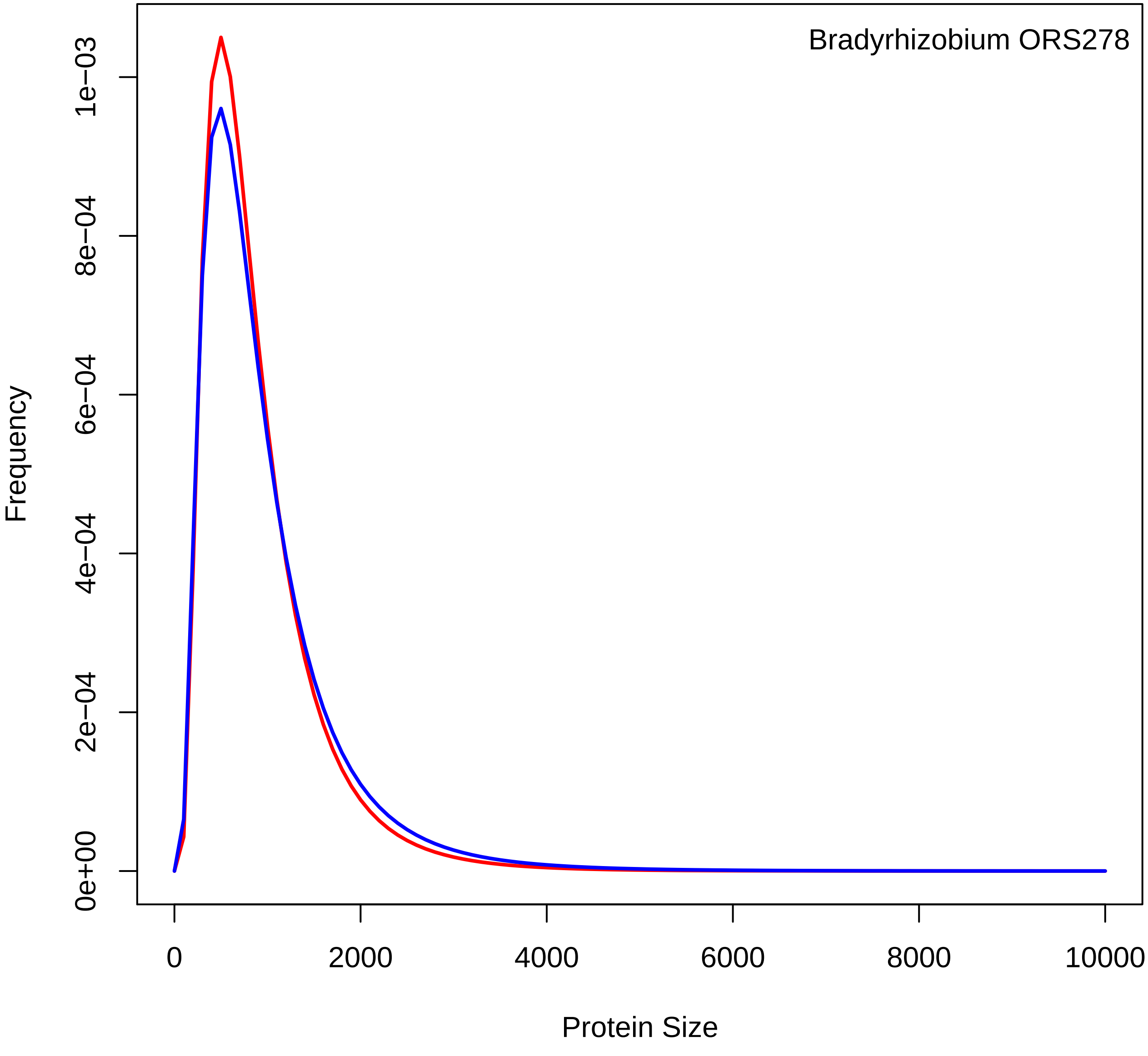

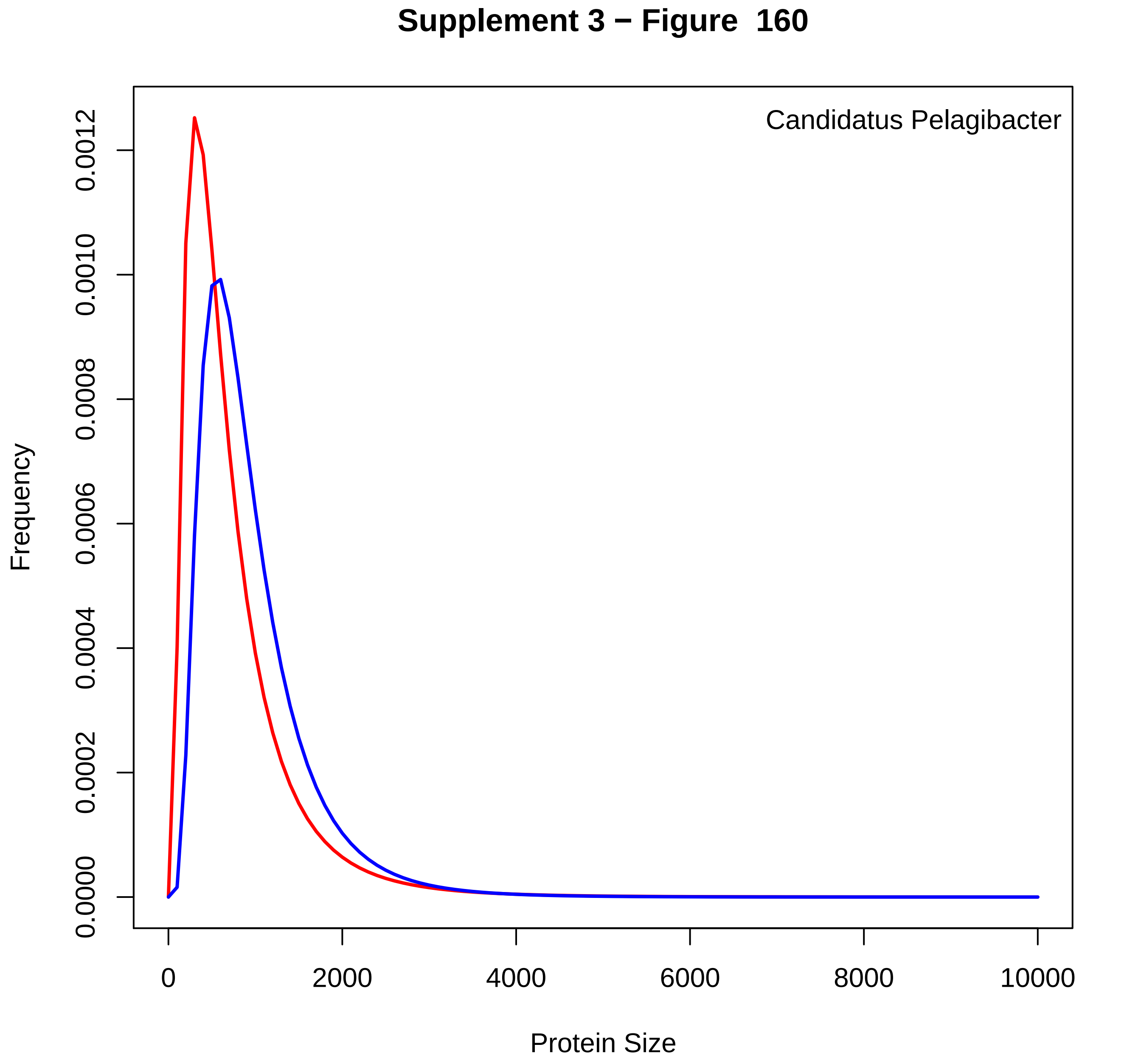
Supplement 3 − Figure 160
Candidatus Pelagibacter
Frequency
0.0000
0.0002
0.0004
0.0006
0.0008
0.0010
0.0012
Protein Size
0
2000
4000
6000
8000
10000

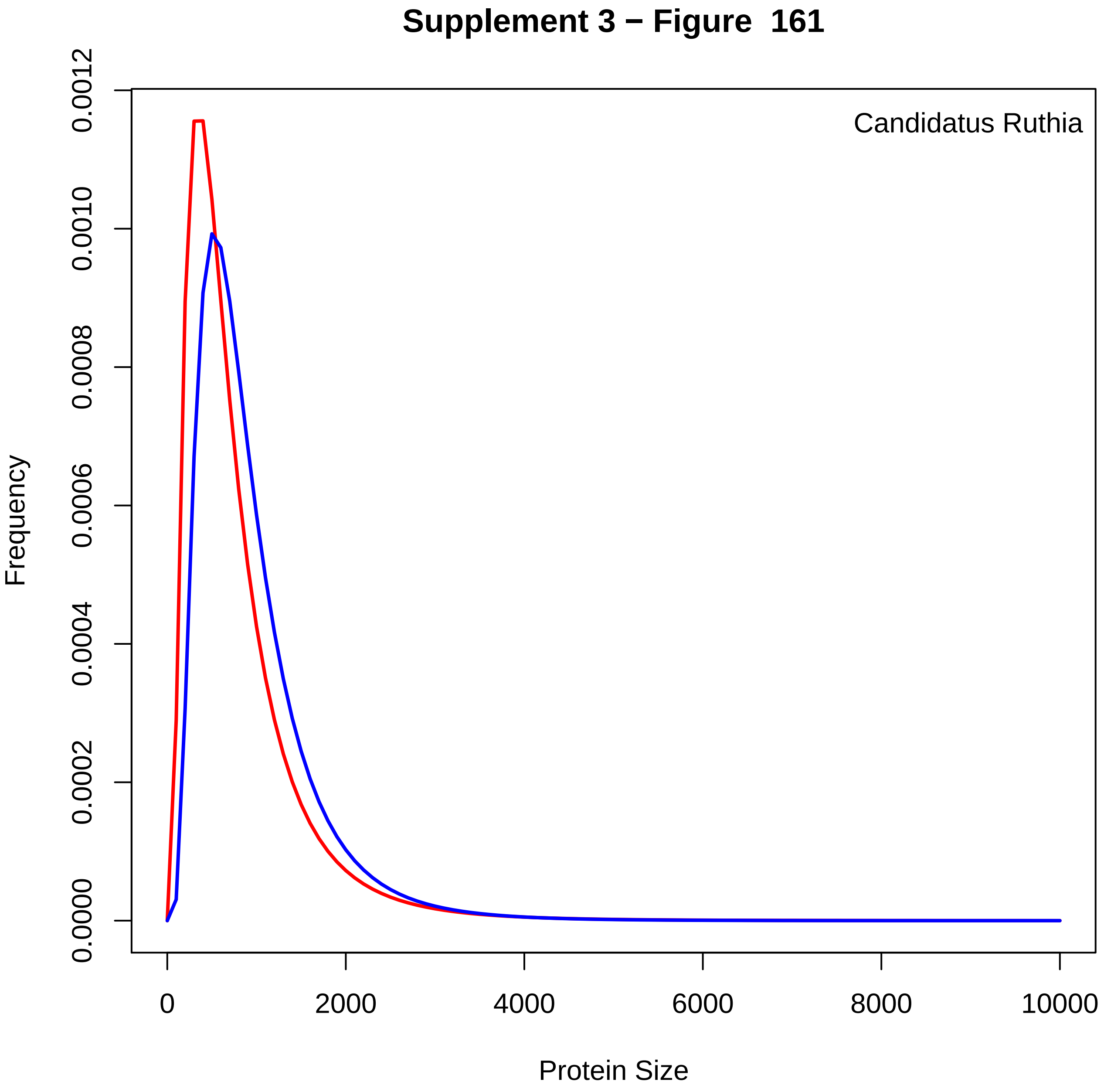

Supplement 3 − Figure 161
Candidatus Ruthia
Frequency
0.0000
0.0002
0.0004
0.0006
0.0008
0.0010
0.0012
0
2000
4000
6000
8000
10000
Protein Size

Supplement 3 − Figure 162

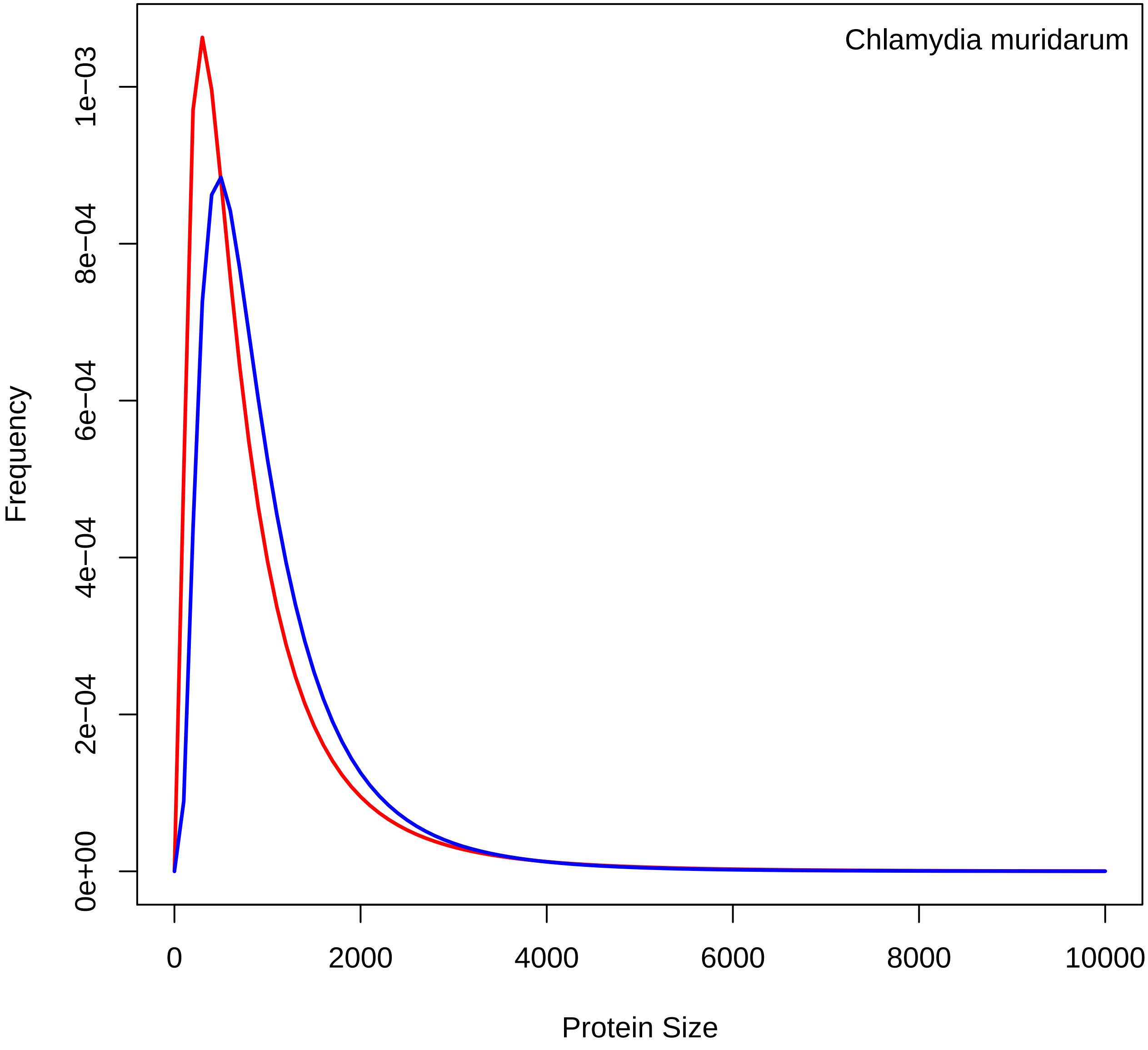

Supplement 3 − Figure 163

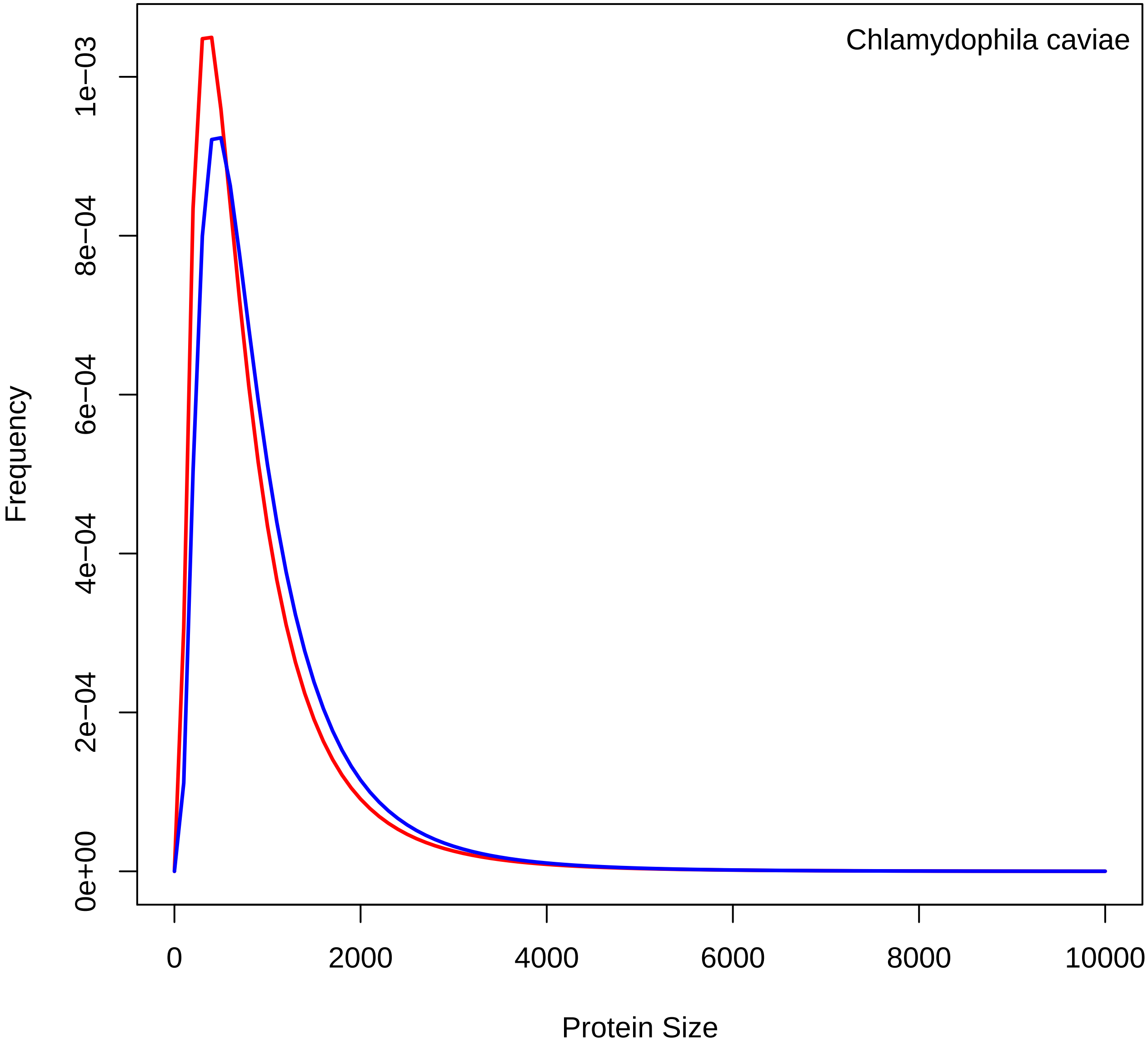

Supplement 3 − Figure 164

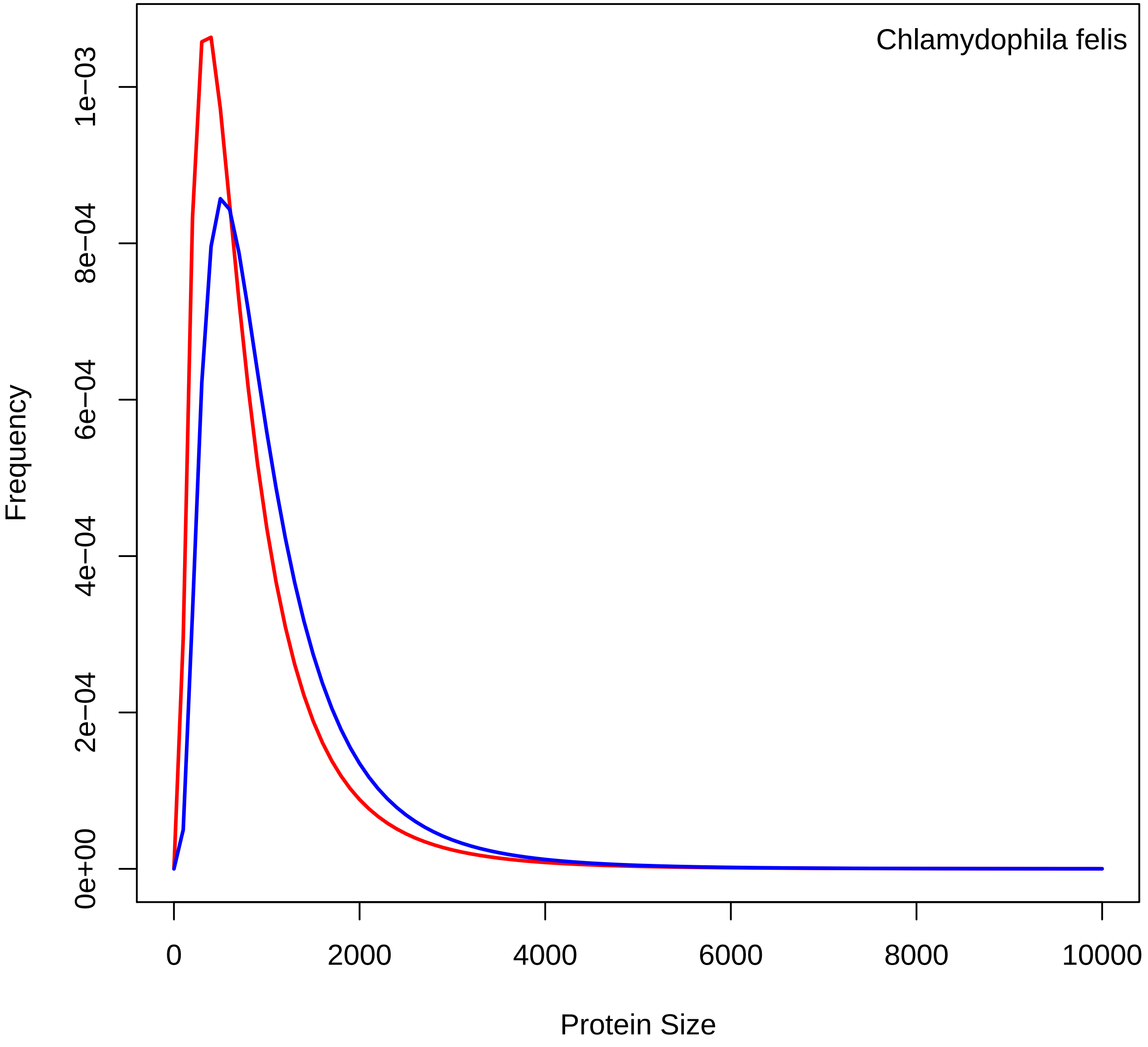

Supplement 3 − Figure 165

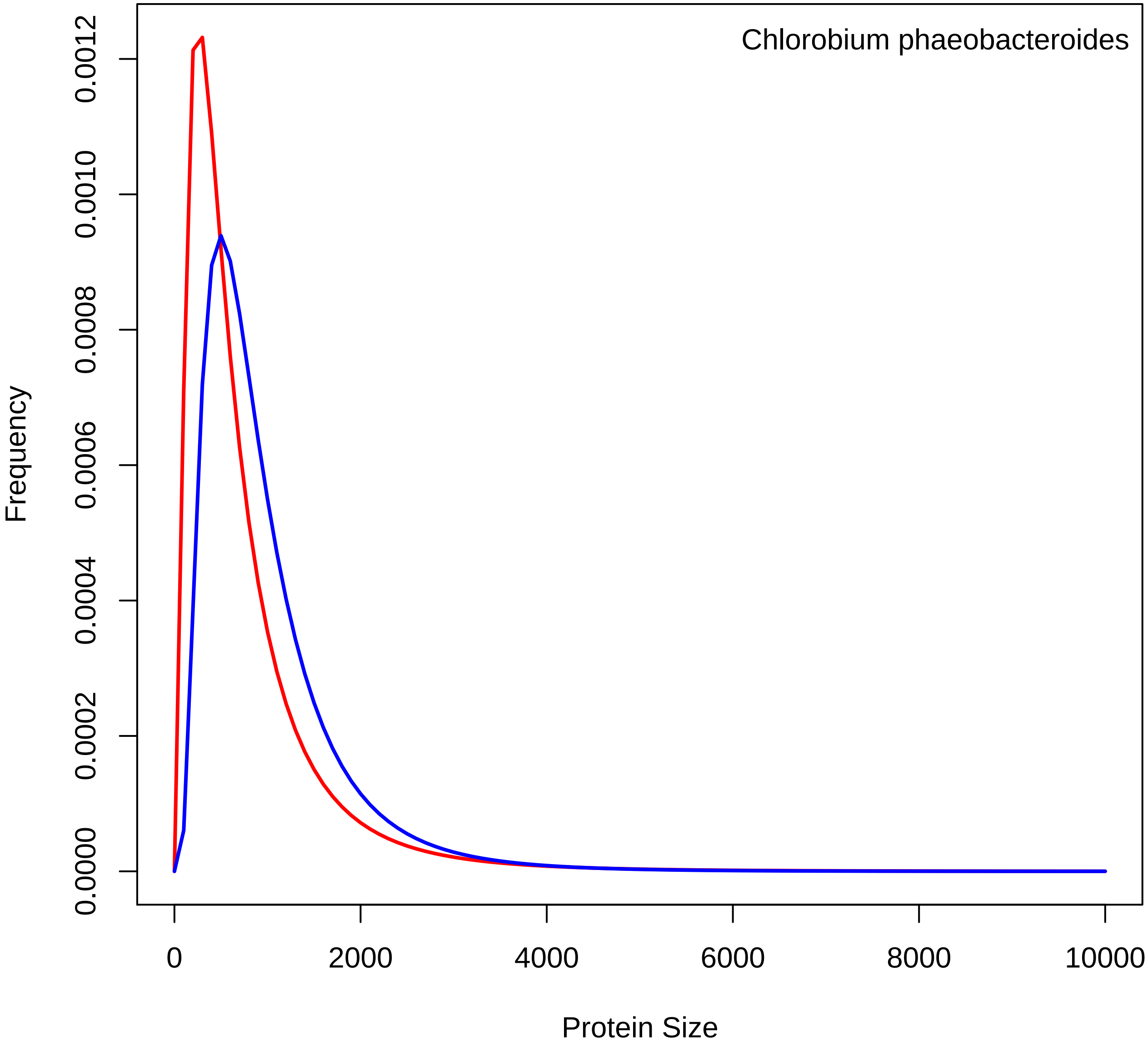

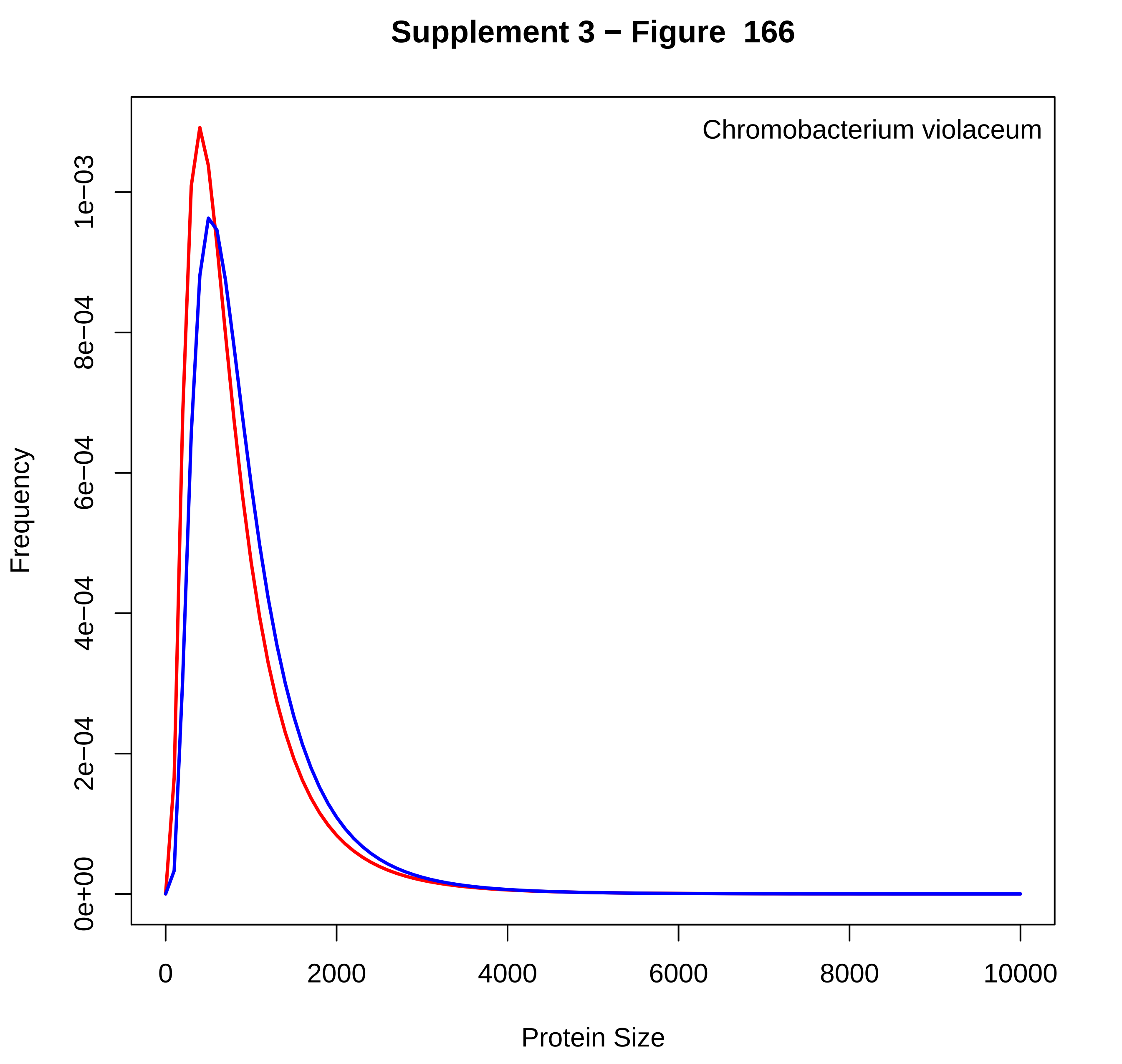

Supplement 3 − Figure 166
Chromobacterium violaceum
Frequency
0e+00
2e−04
4e−04
6e−04
8e−04
1e−03
0
2000
4000
6000
8000
10000
Protein Size

Supplement 3 − Figure 167

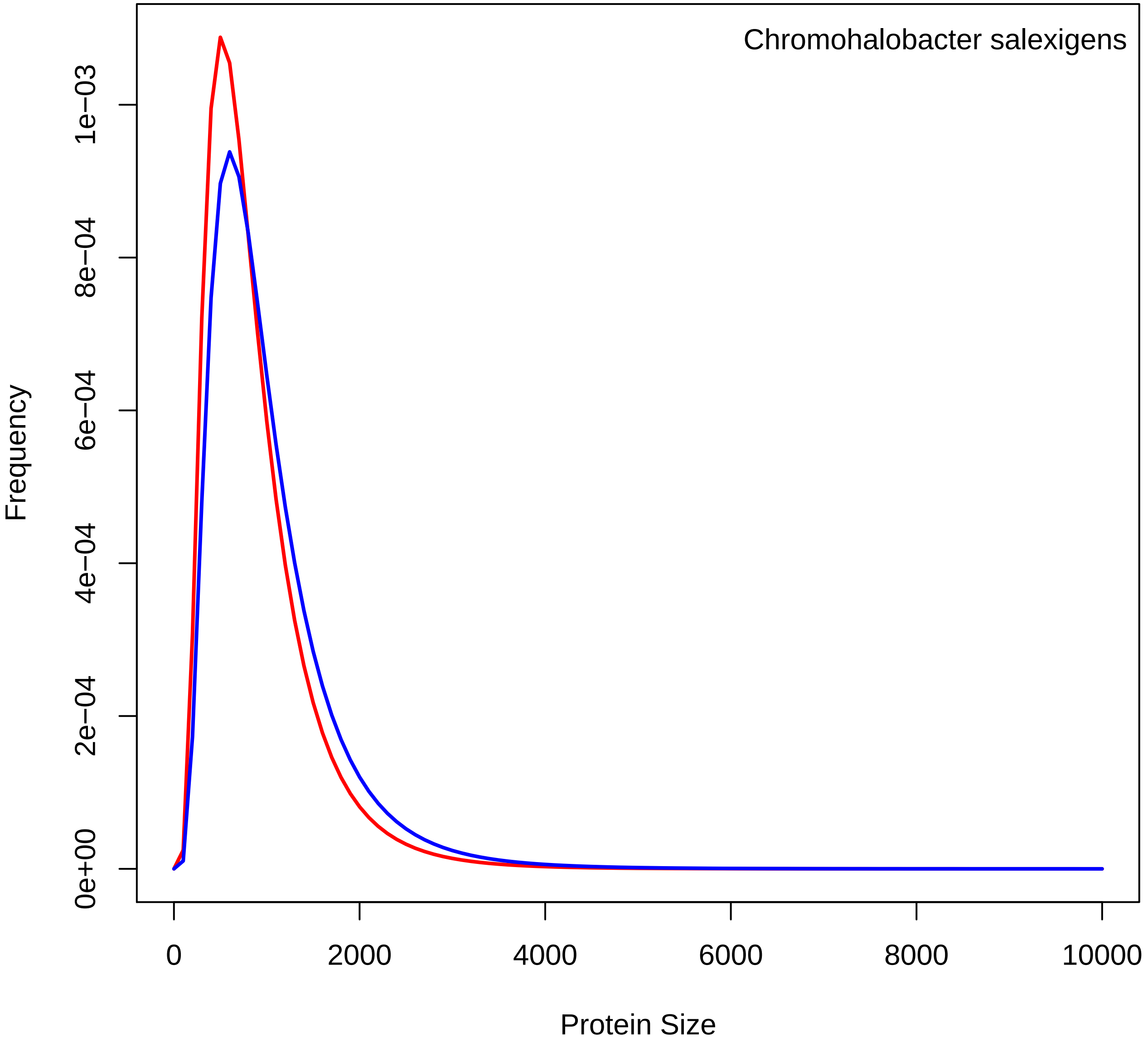

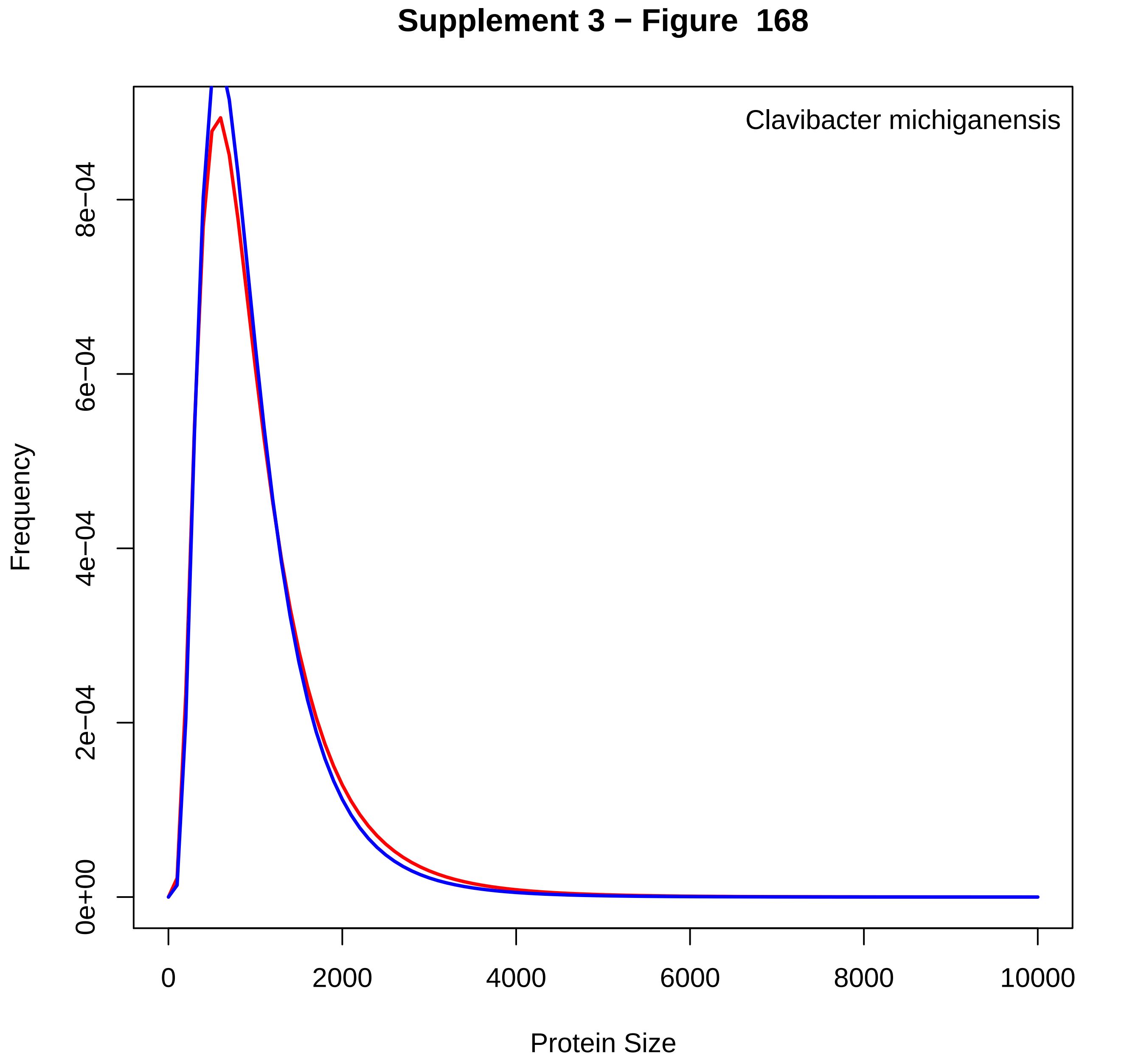
Supplement 3 − Figure 168
Clavibacter michiganensis
Frequency
0e+00
2e−04
4e−04
6e−04
8e−04
0
2000
4000
6000
8000
10000
Protein Size

Supplement 3 − Figure 169

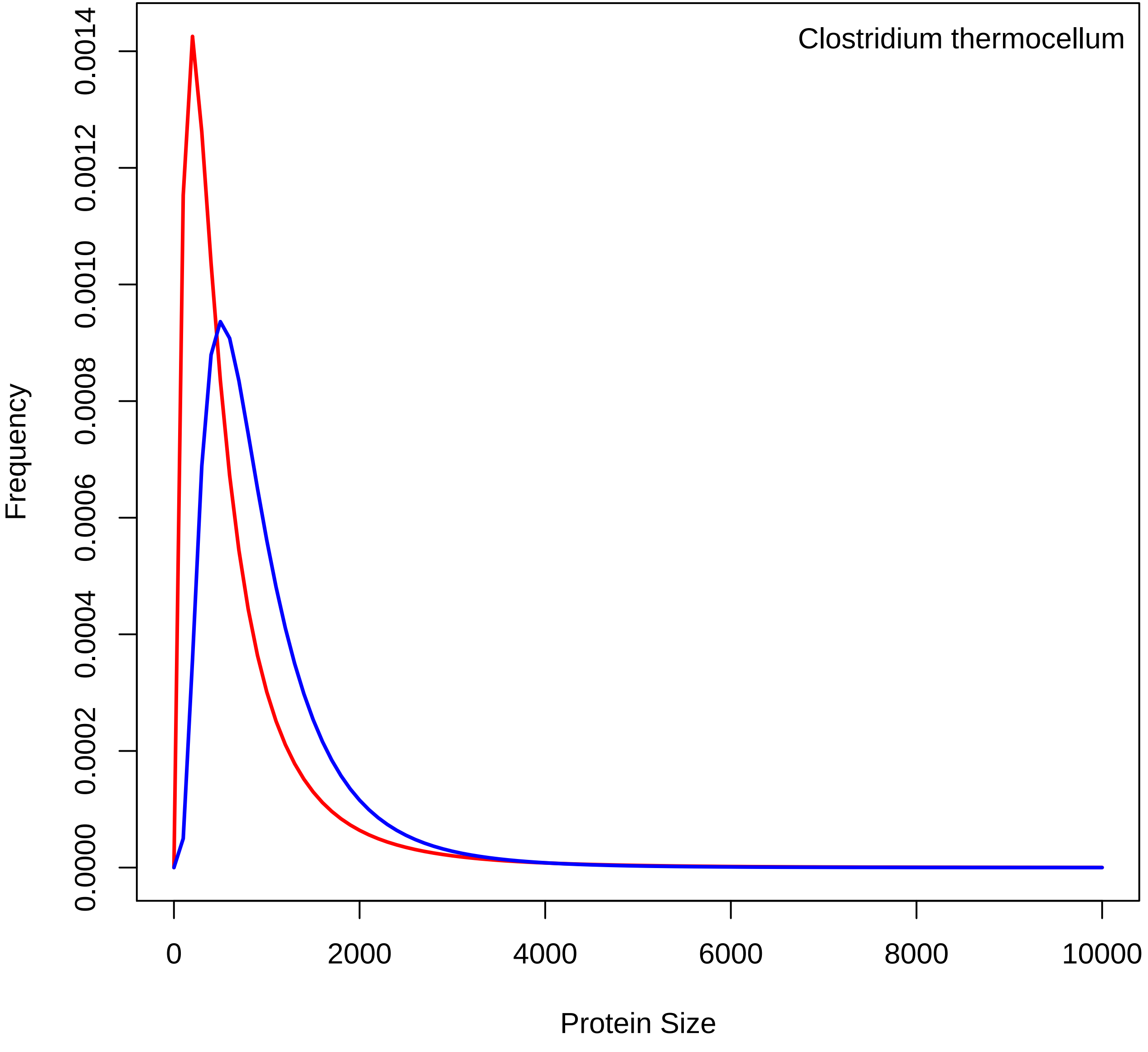

Supplement 3 − Figure 170

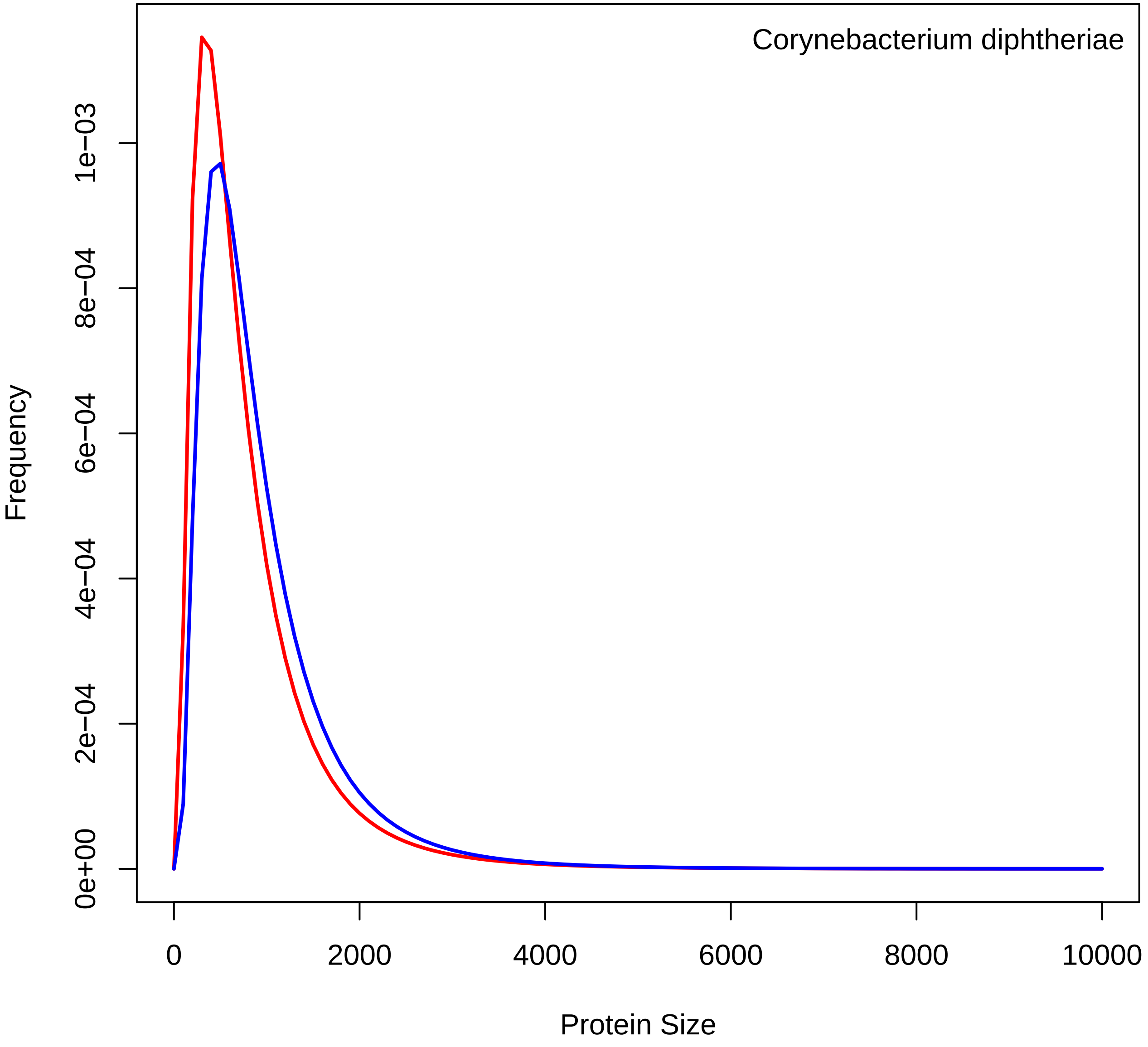

Supplement 3 − Figure 171

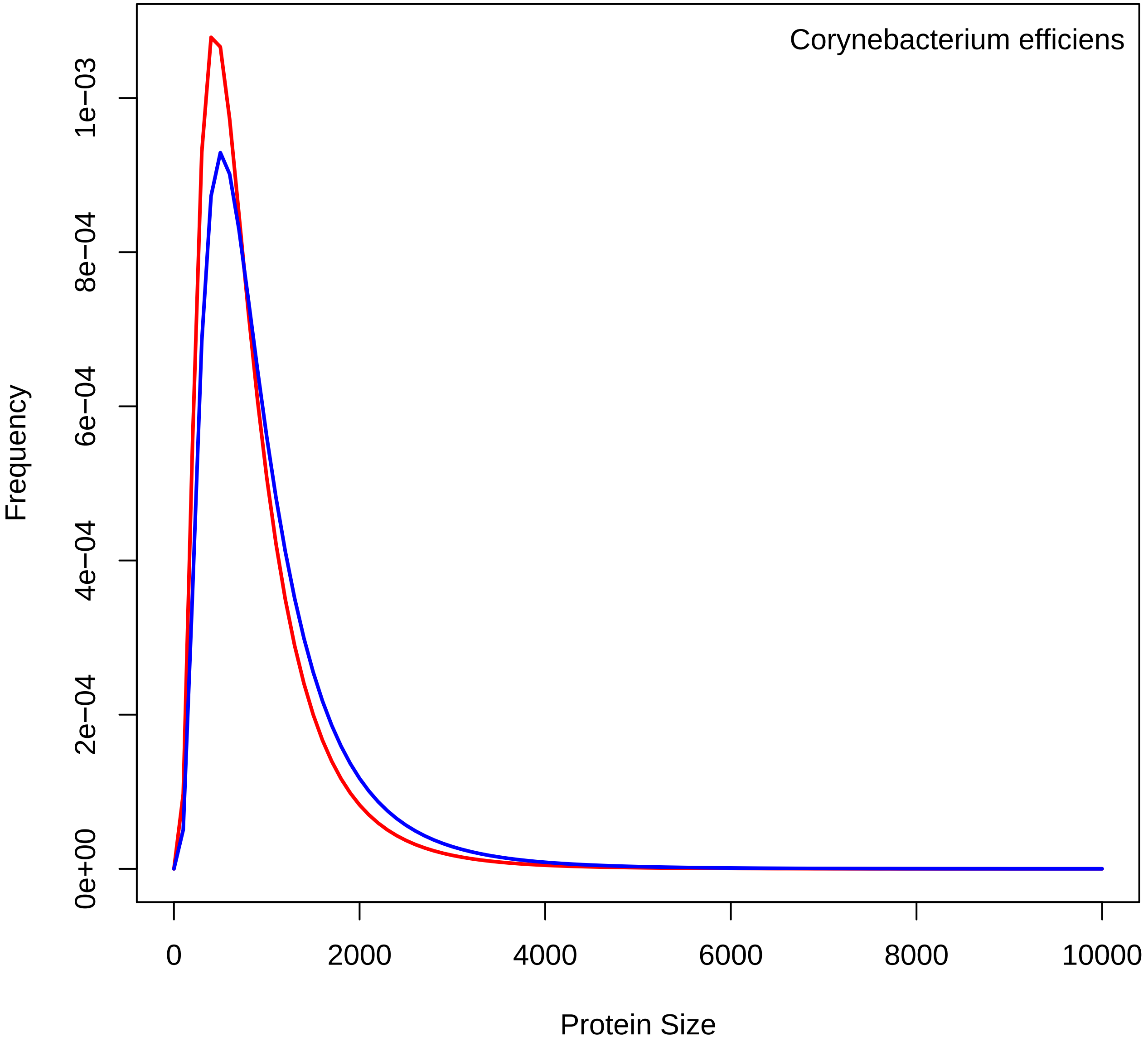

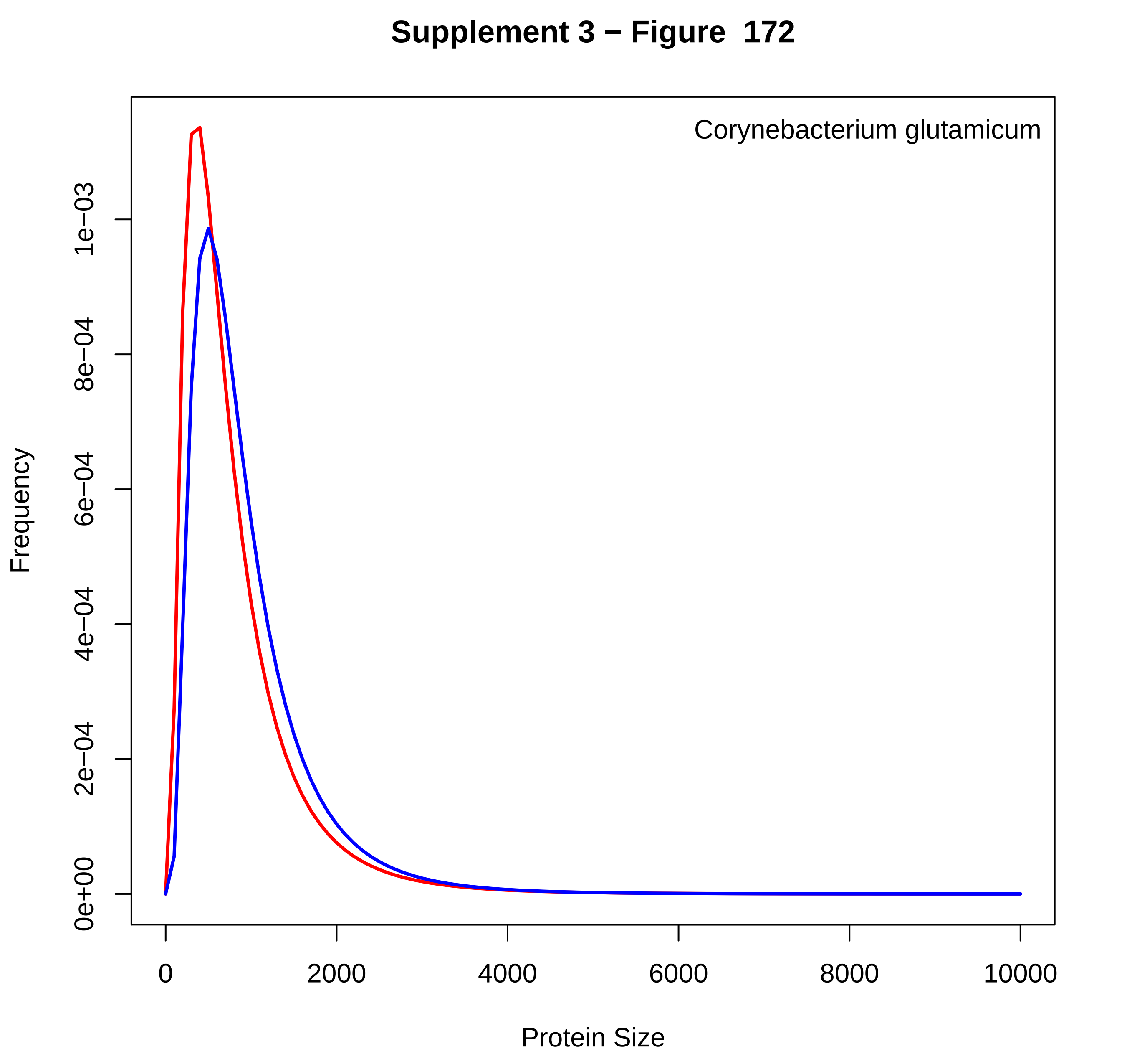
Supplement 3 − Figure 172
Corynebacterium glutamicum
Frequency
0e+00
2e−04
4e−04
6e−04
8e−04
1e−03
0
2000
4000
6000
8000
10000
Protein Size

Supplement 3 − Figure 173

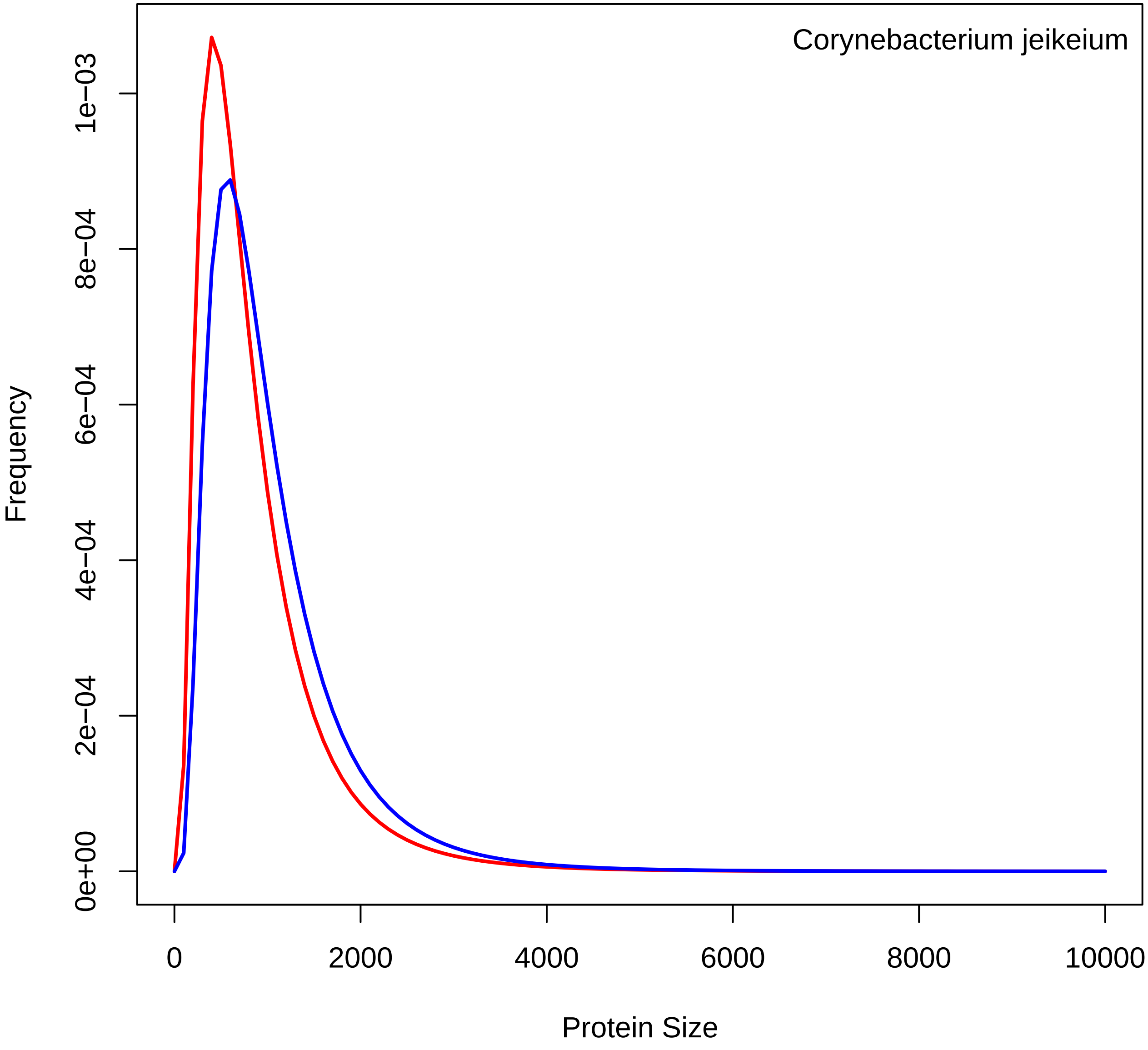

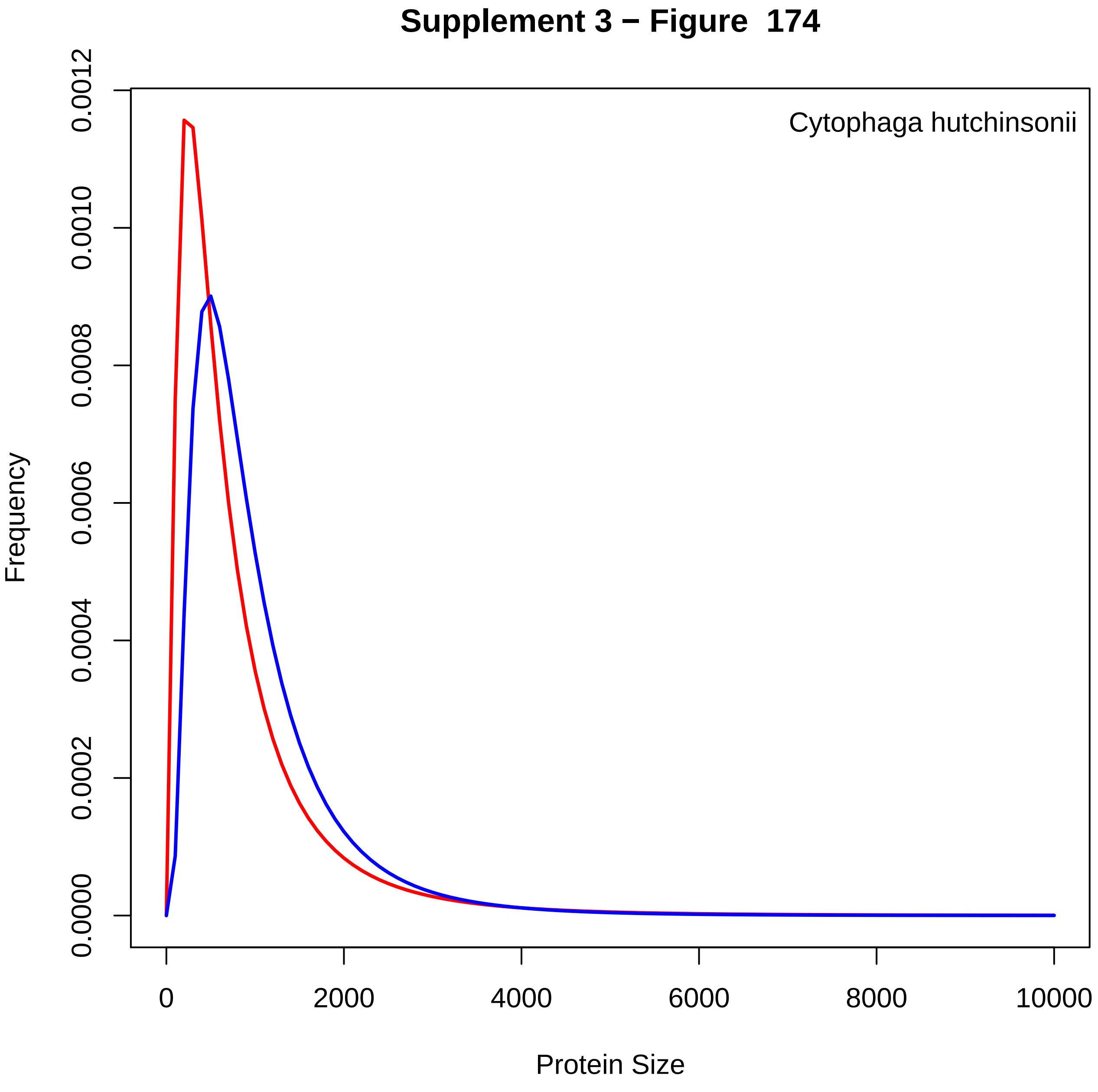

Supplement 3 − Figure 174
Cytophaga hutchinsonii
Frequency
0.0000
0.0002
0.0004
0.0006
0.0008
0.0010
0.0012
0
2000
4000
6000
8000
10000
Protein Size

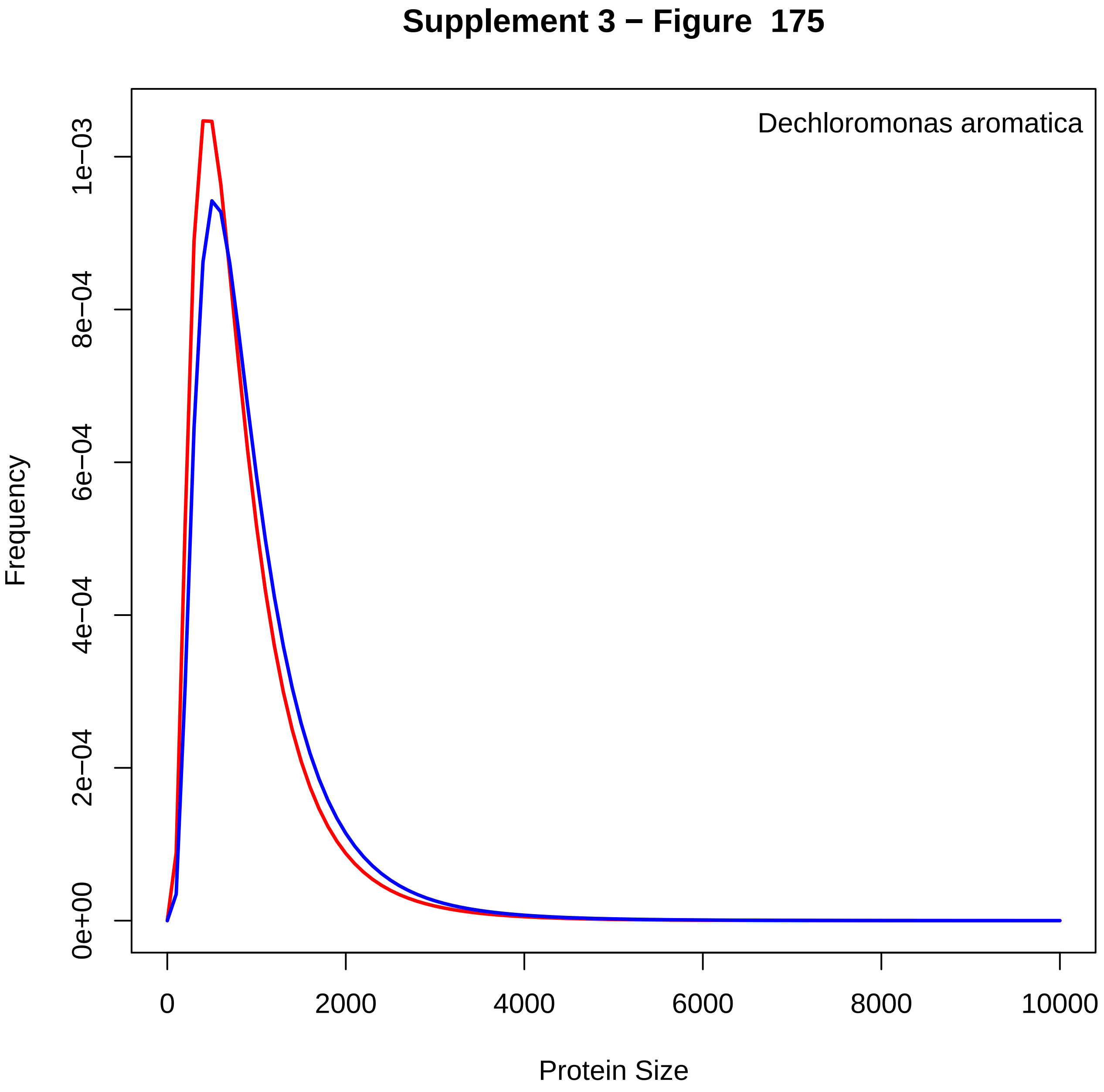

Supplement 3 − Figure 175
Dechloromonas aromatica
Frequency
0e+00
2e−04
4e−04
6e−04
8e−04
1e−03
0
2000
4000
6000
8000
10000
Protein Size

Supplement 3 − Figure 176

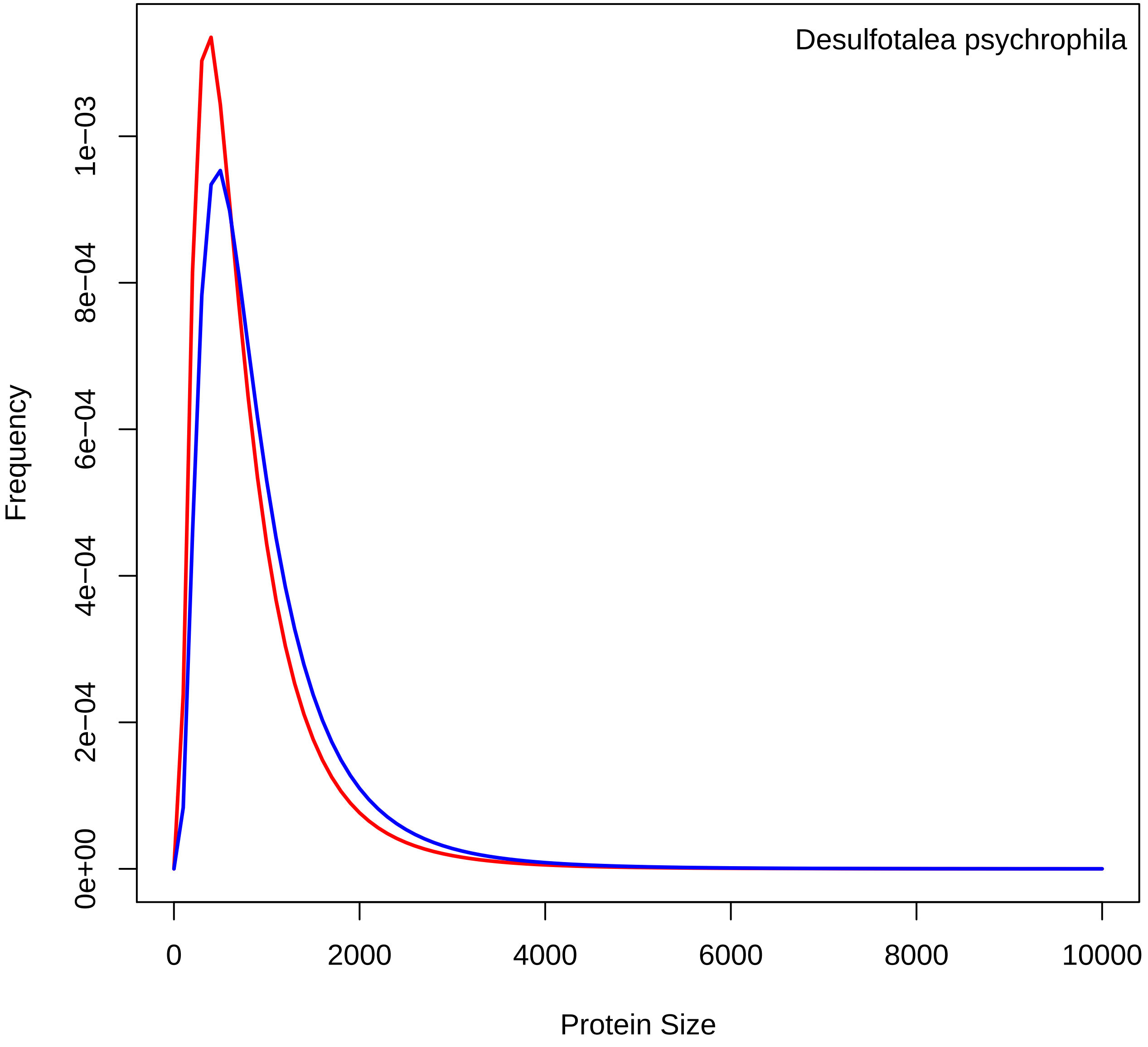

Supplement 3 − Figure 177

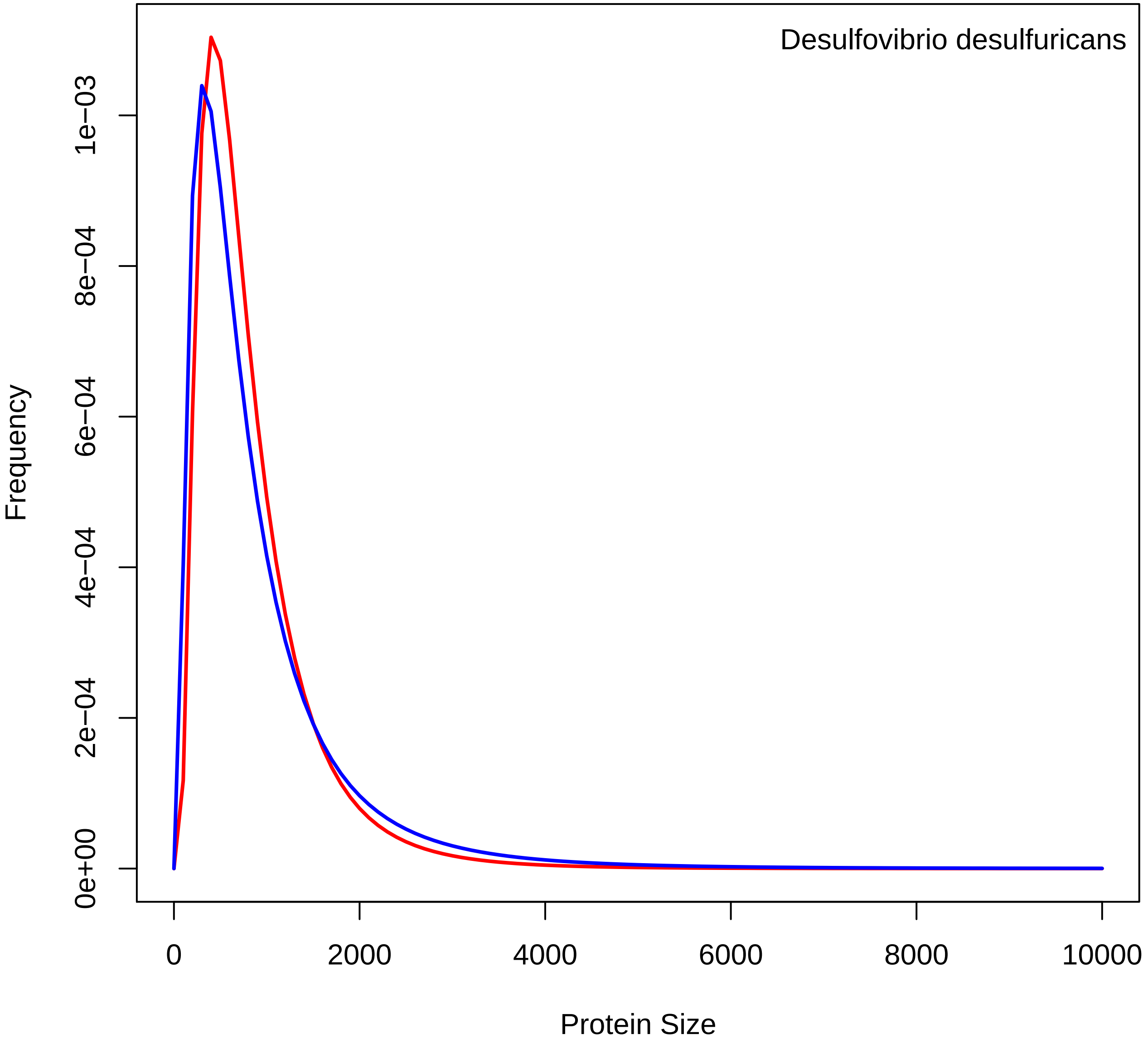

Supplement 3 − Figure 178

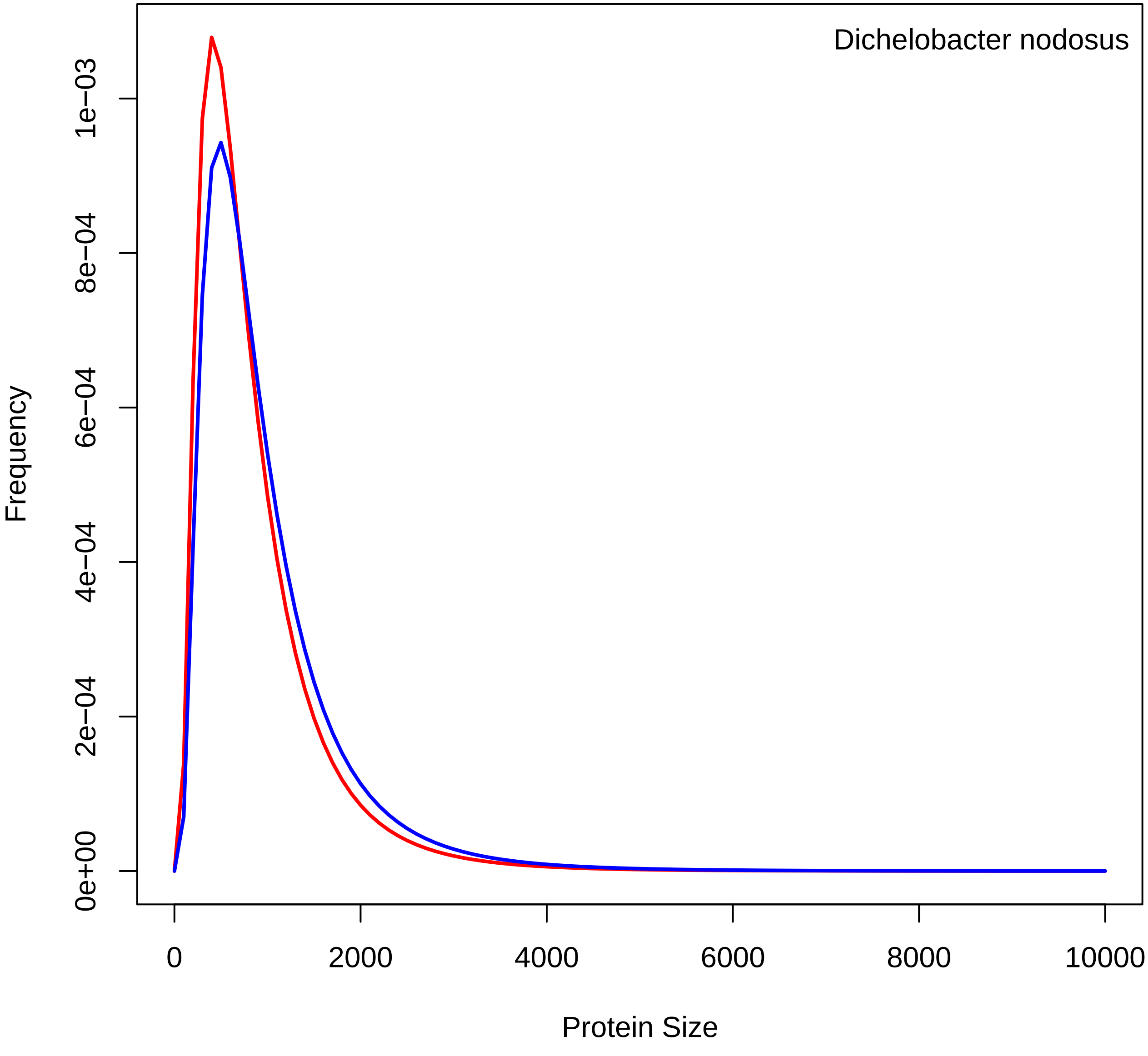

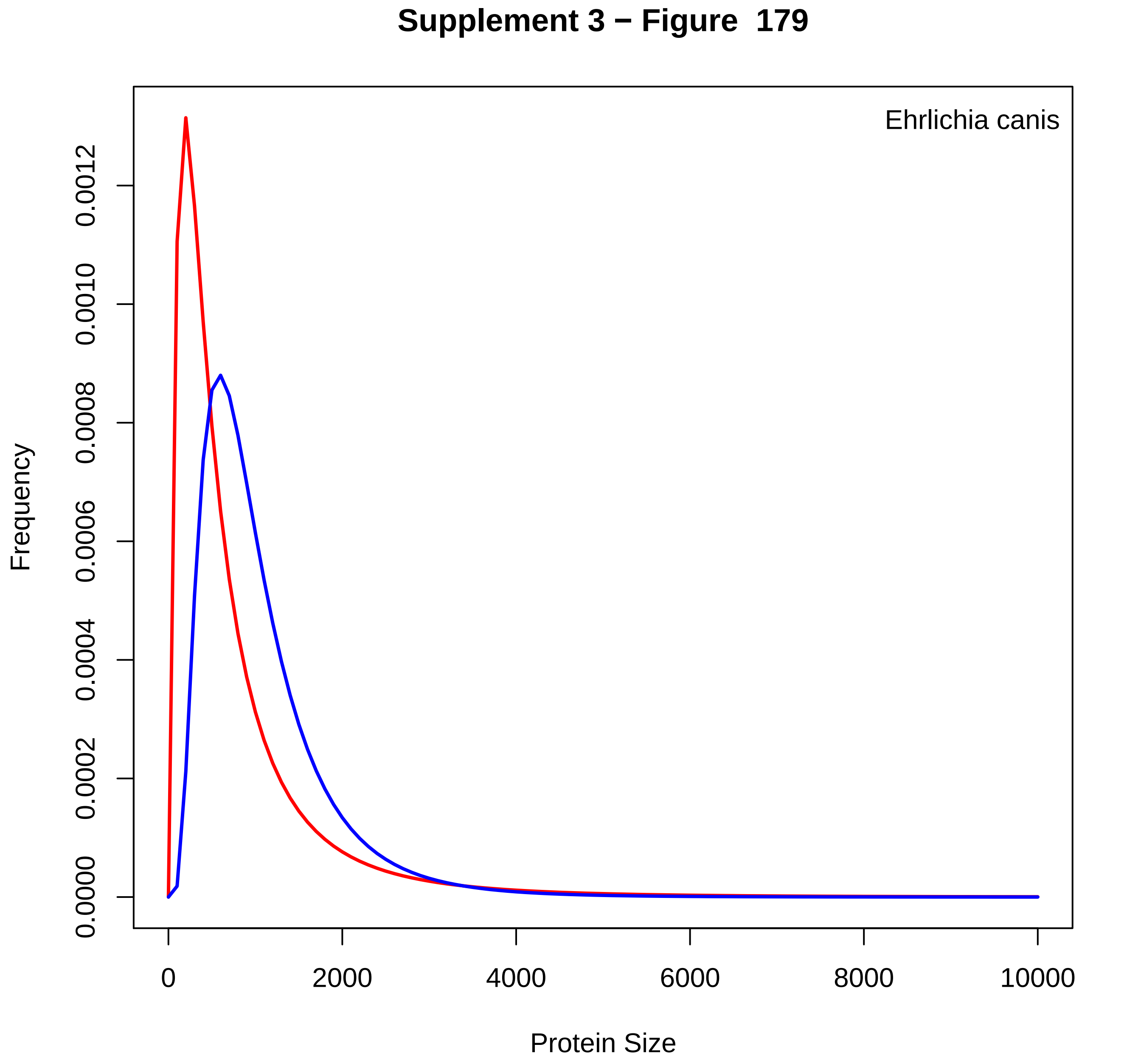
Supplement 3 − Figure 179
Ehrlichia canis
Frequency
0.0000
0.0002
0.0004
0.0006
0.0008
0.0010
0.0012
0
2000
4000
6000
8000
10000
Protein Size

Supplement 3 − Figure 180

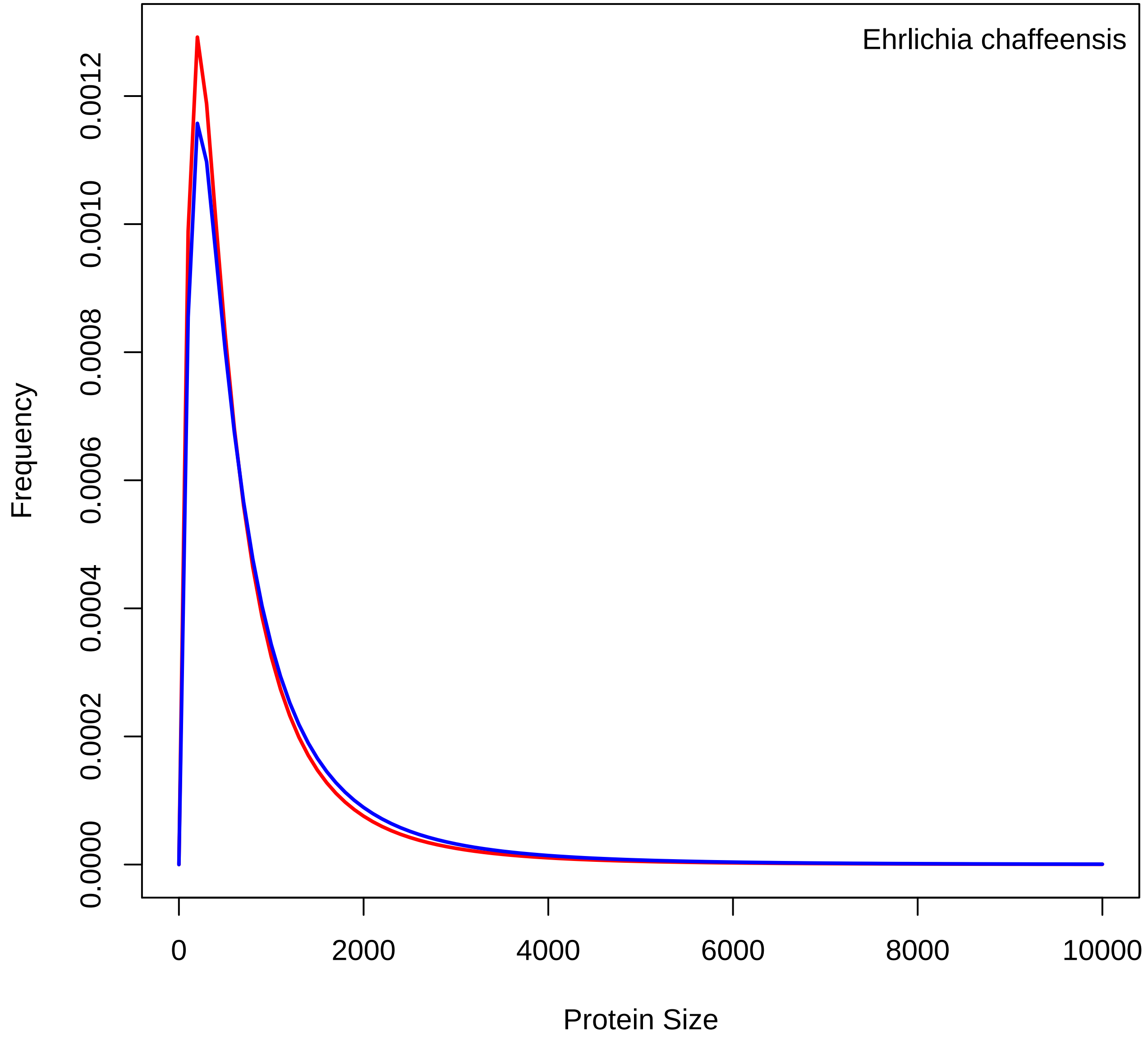

Supplement 3 − Figure 181

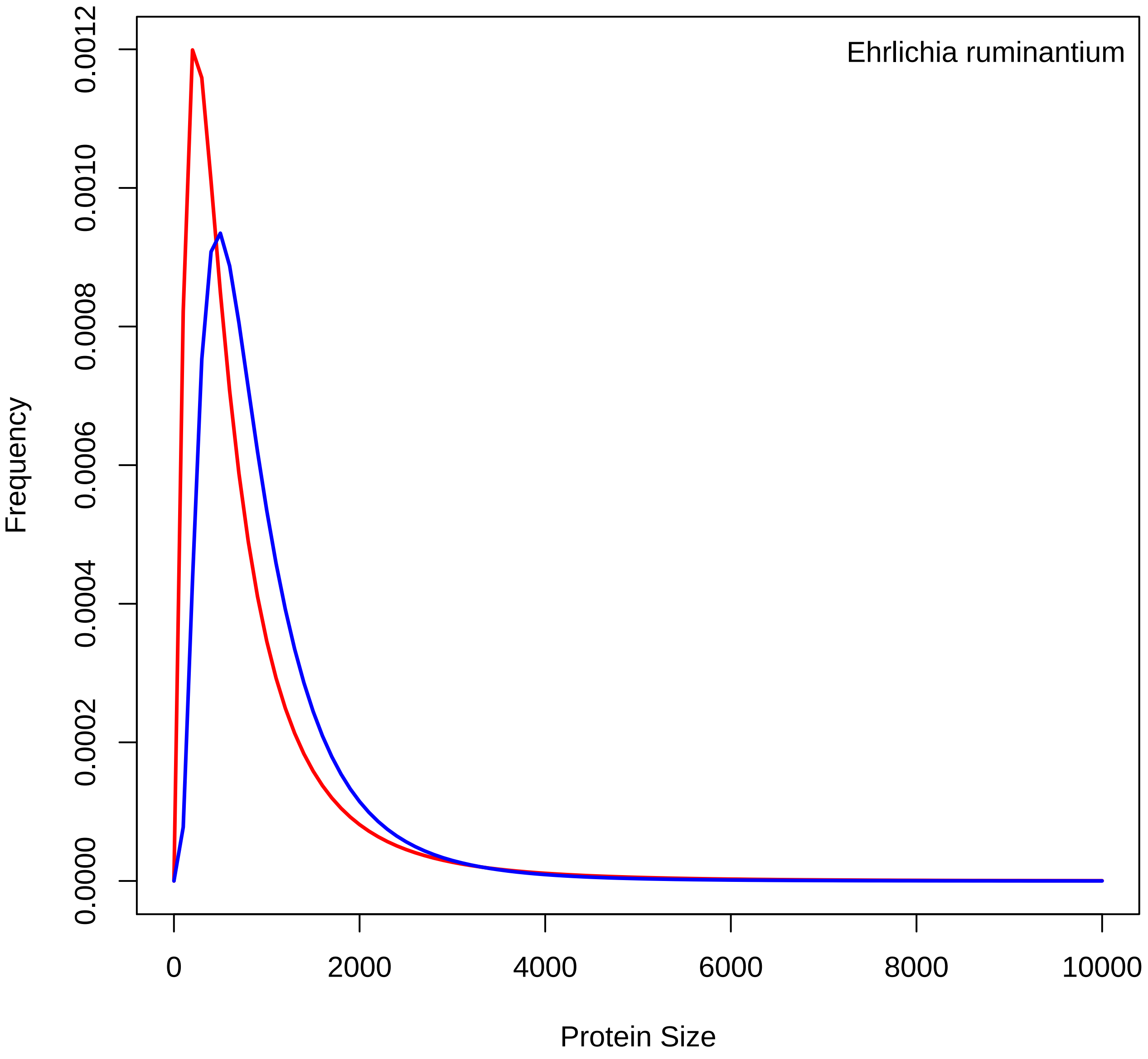

Supplement 3 − Figure 182

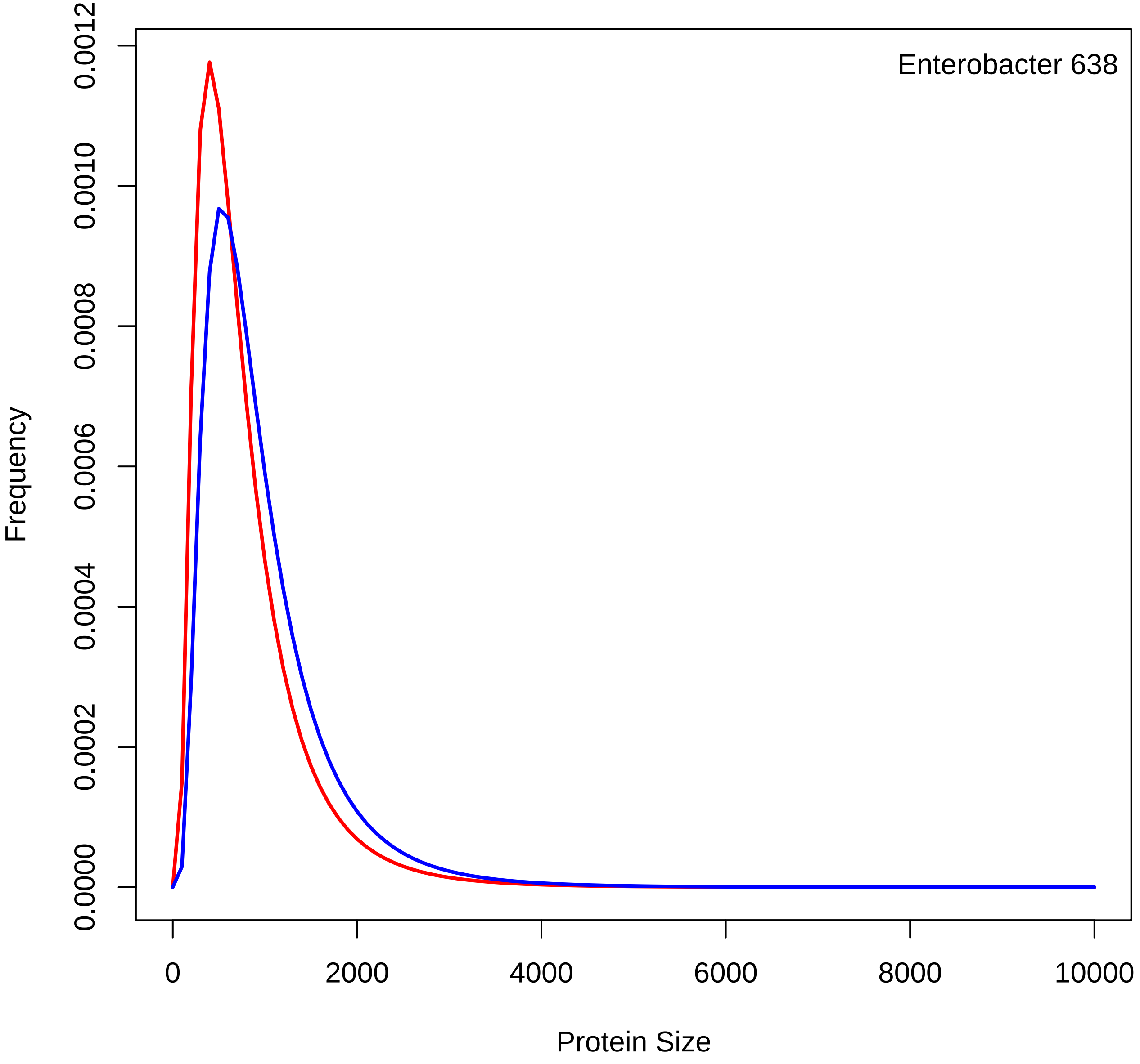

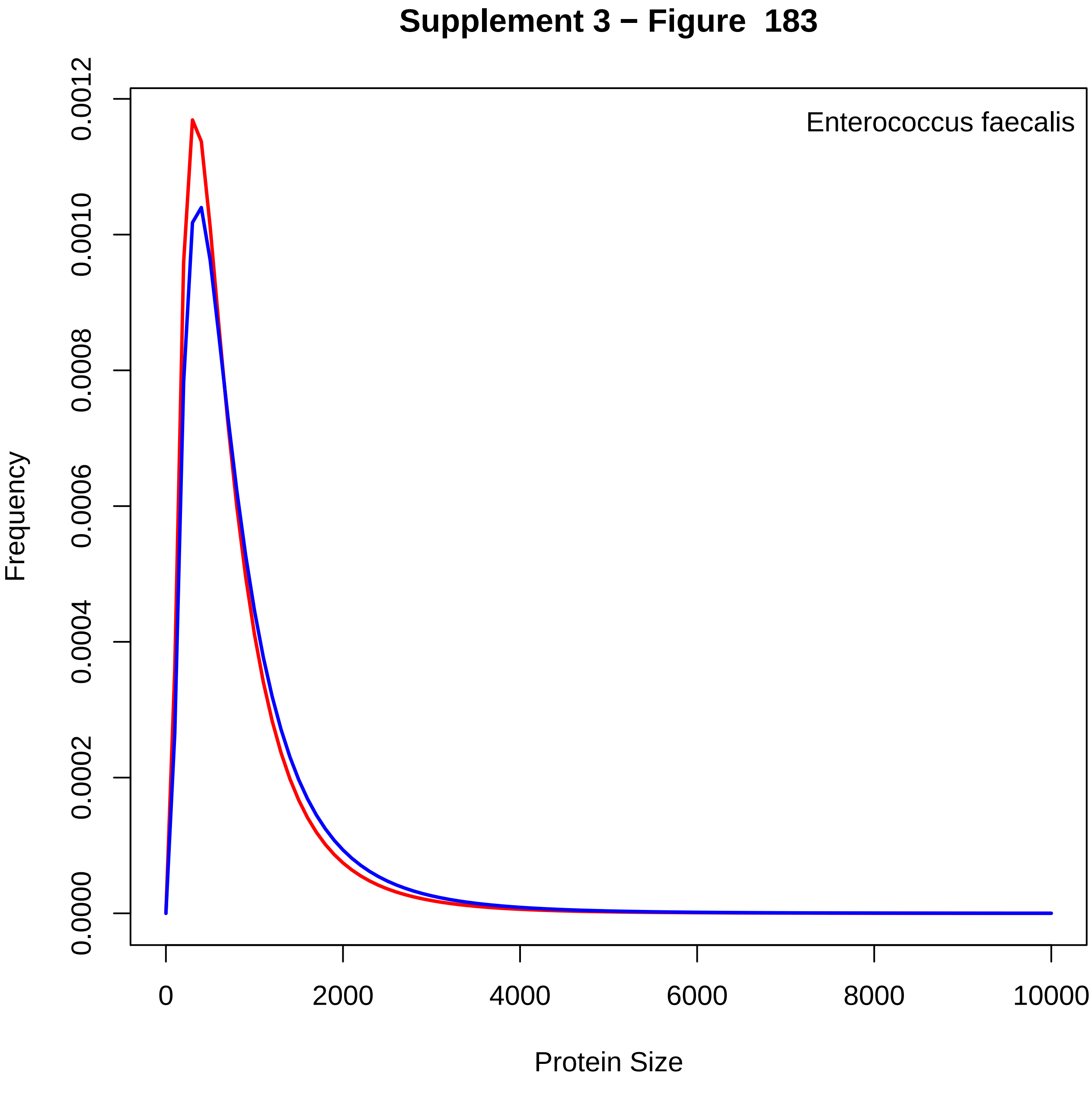

Supplement 3 − Figure 183
Enterococcus faecalis
Frequency
0.0000
0.0002
0.0004
0.0006
0.0008
0.0010
0.0012
0
2000
4000
6000
8000
10000
Protein Size

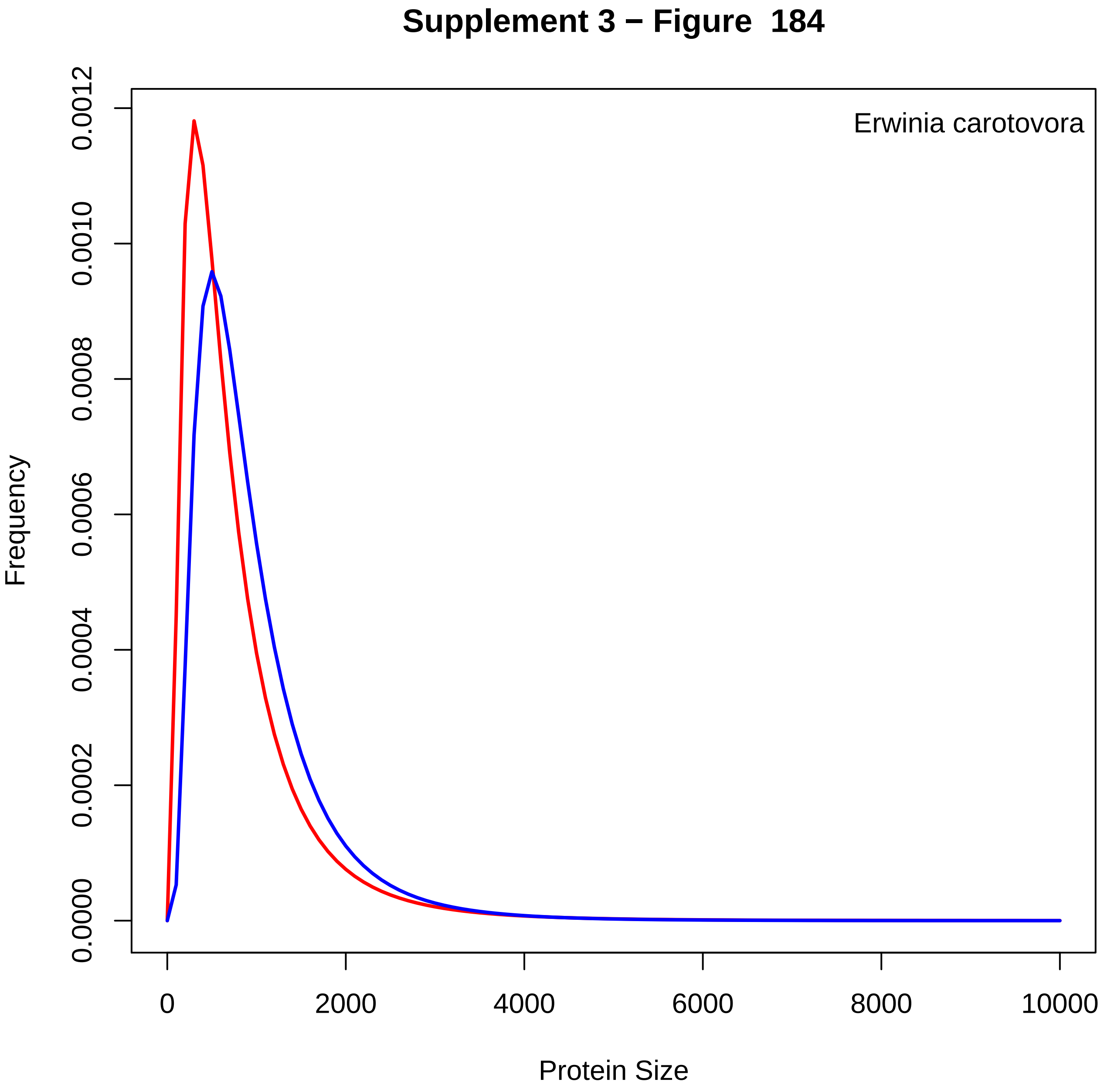

Supplement 3 − Figure 184
Erwinia carotovora
Frequency
0.0000
0.0002
0.0004
0.0006
0.0008
0.0010
0.0012
0
2000
4000
6000
8000
10000
Protein Size

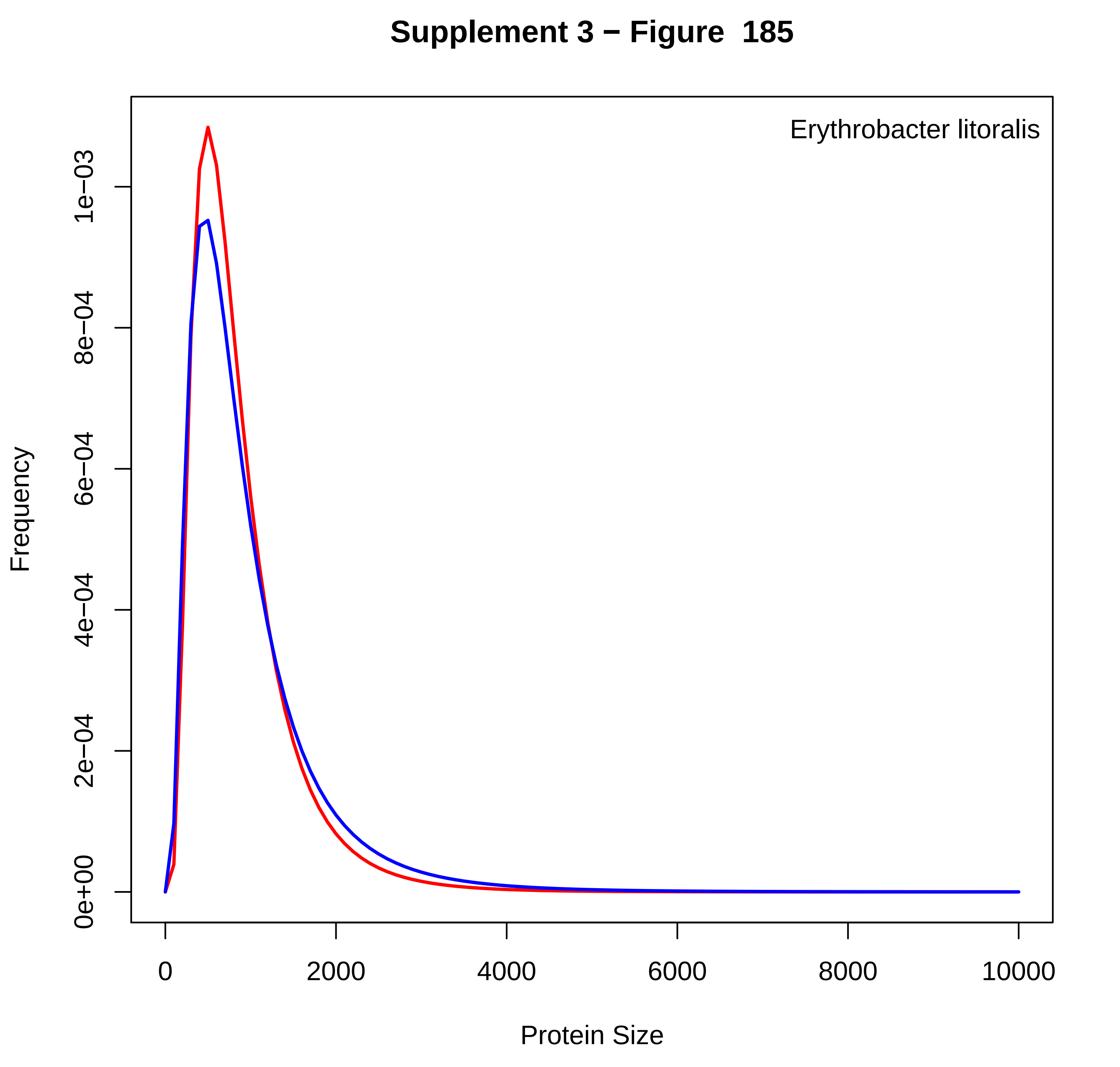

Supplement 3 − Figure 185
Erythrobacter litoralis
Frequency
0e+00
2e−04
4e−04
6e−04
8e−04
1e−03
0
2000
4000
6000
8000
10000
Protein Size

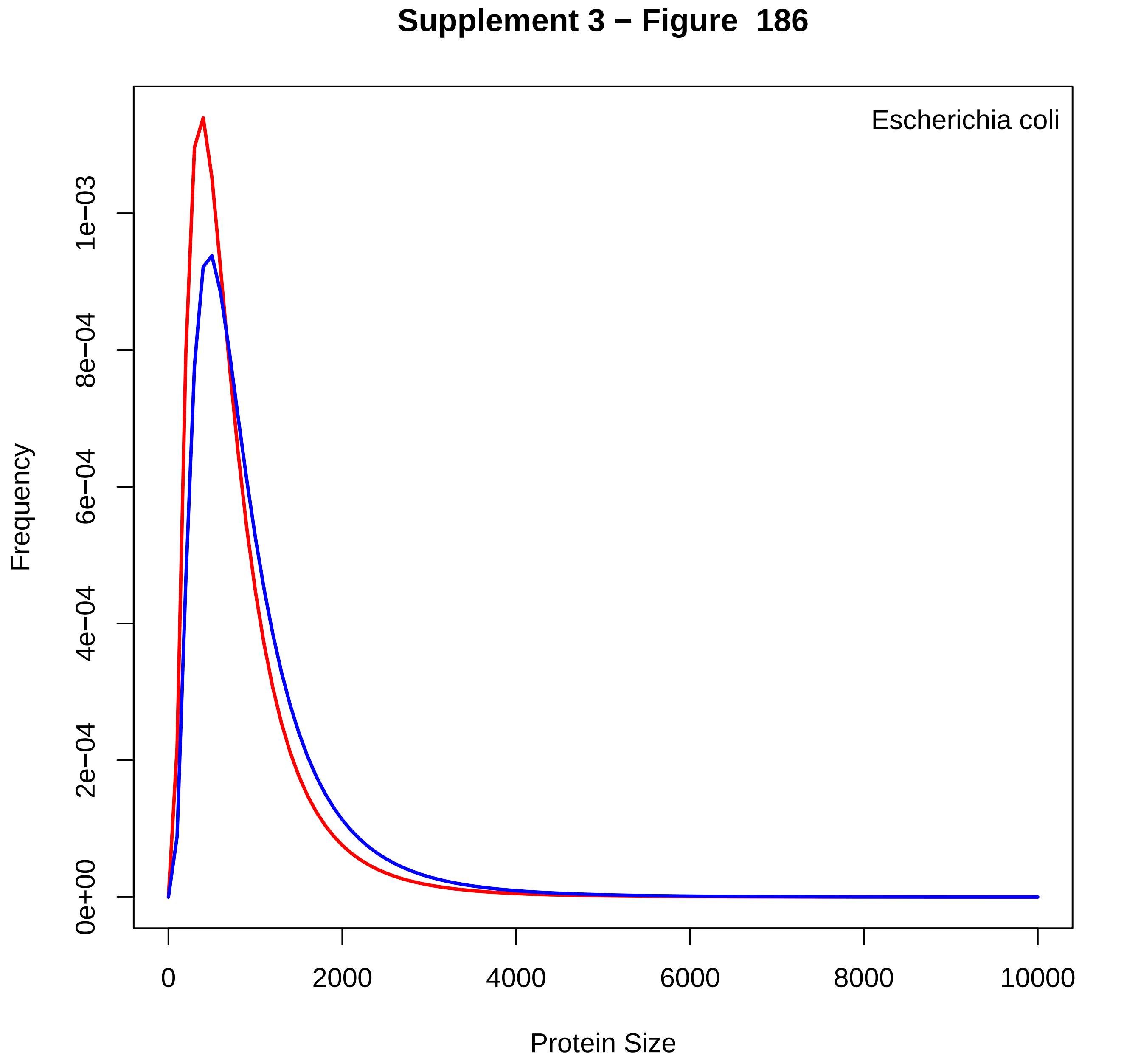
Supplement 3 − Figure 186
Escherichia coli
Frequency
0e+00
2e−04
4e−04
6e−04
8e−04
1e−03
0
2000
4000
6000
8000
10000
Protein Size

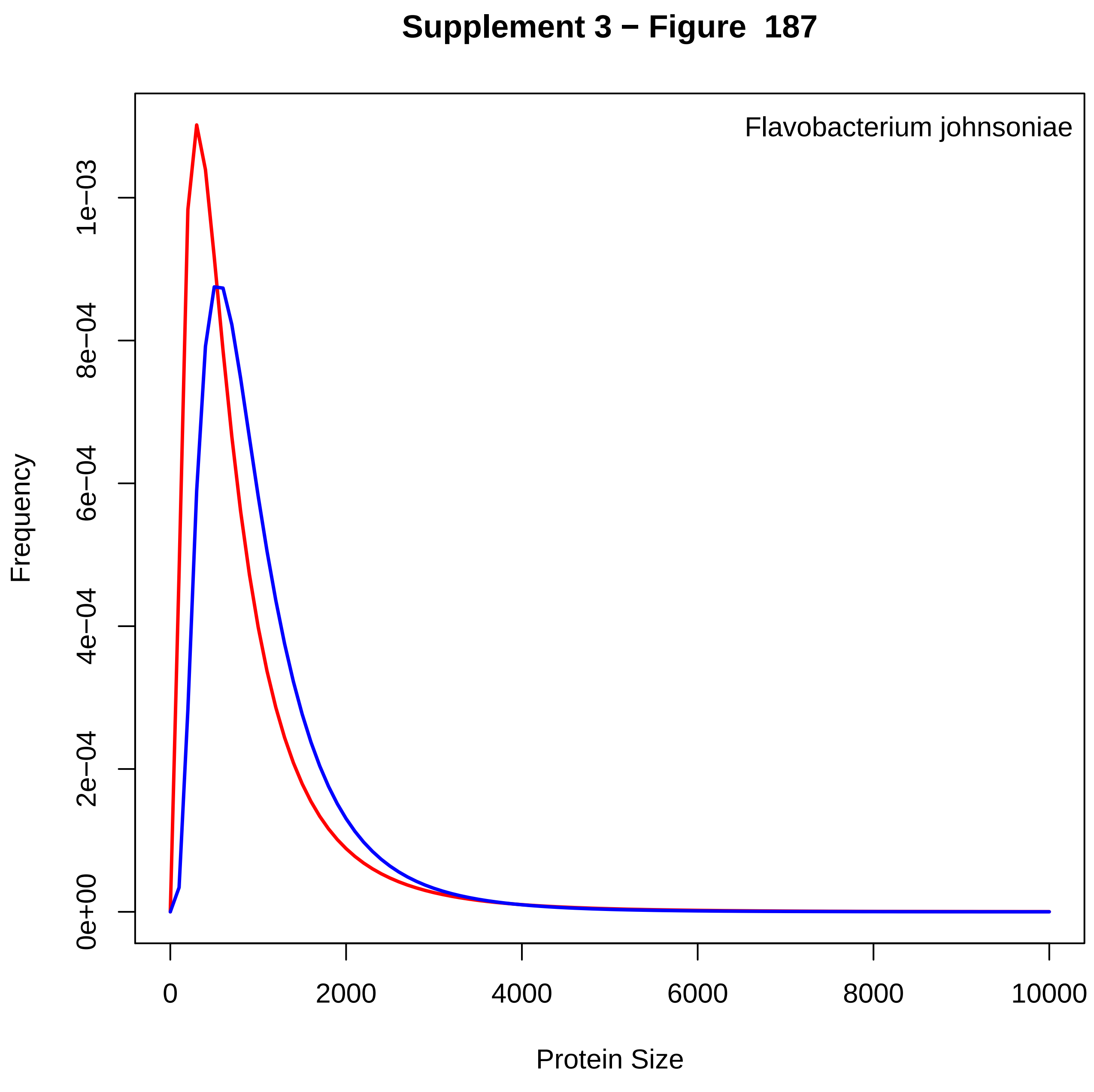
Supplement 3 − Figure 187
Flavobacterium johnsoniae
Frequency
0e+00
2e−04
4e−04
6e−04
8e−04
1e−03
0
2000
4000
6000
8000
10000
Protein Size

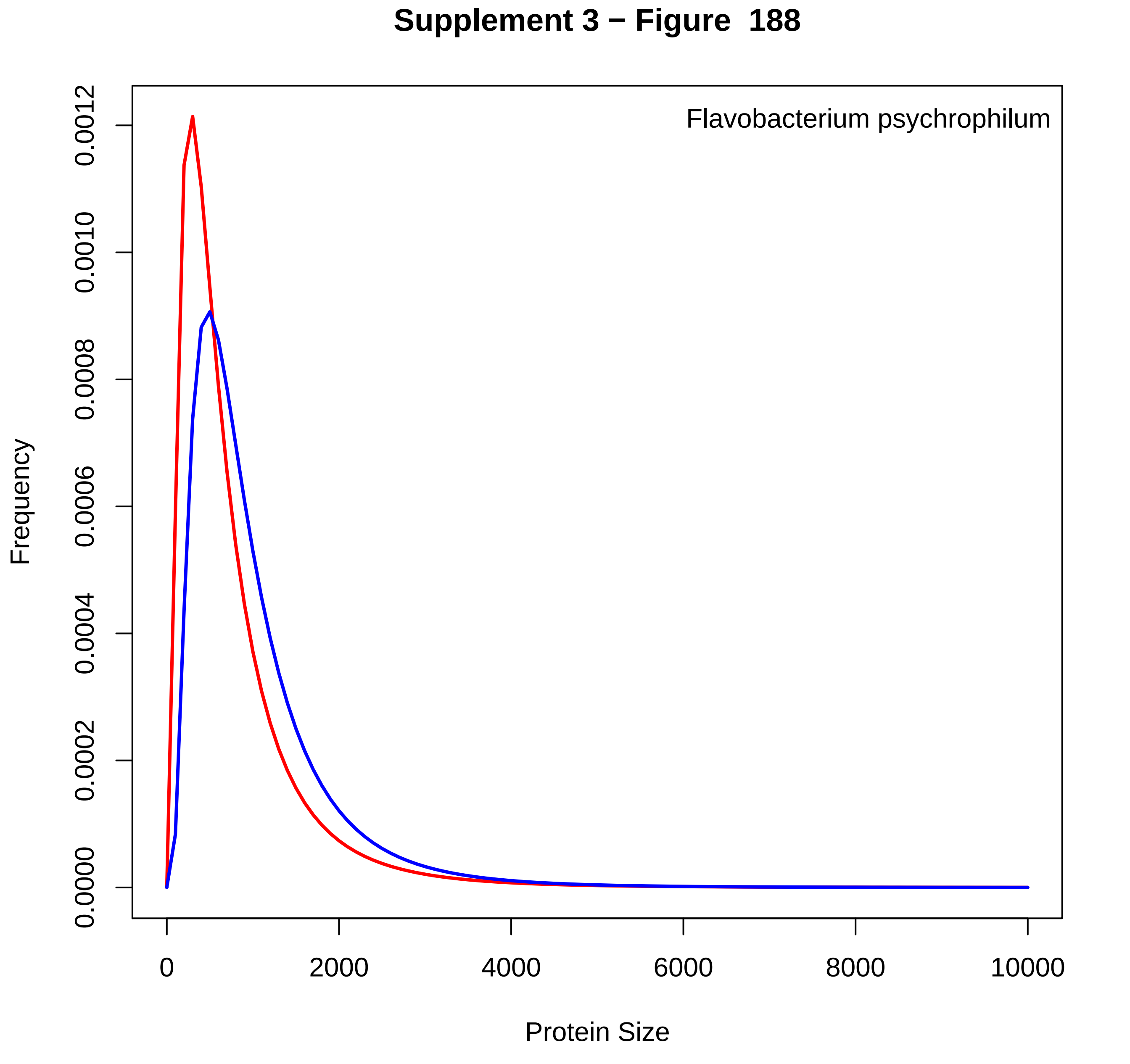
Supplement 3 − Figure 188
Flavobacterium psychrophilum
Frequency
0.0000
0.0002
0.0004
0.0006
0.0008
0.0010
0.0012
0
2000
4000
6000
8000
10000
Protein Size

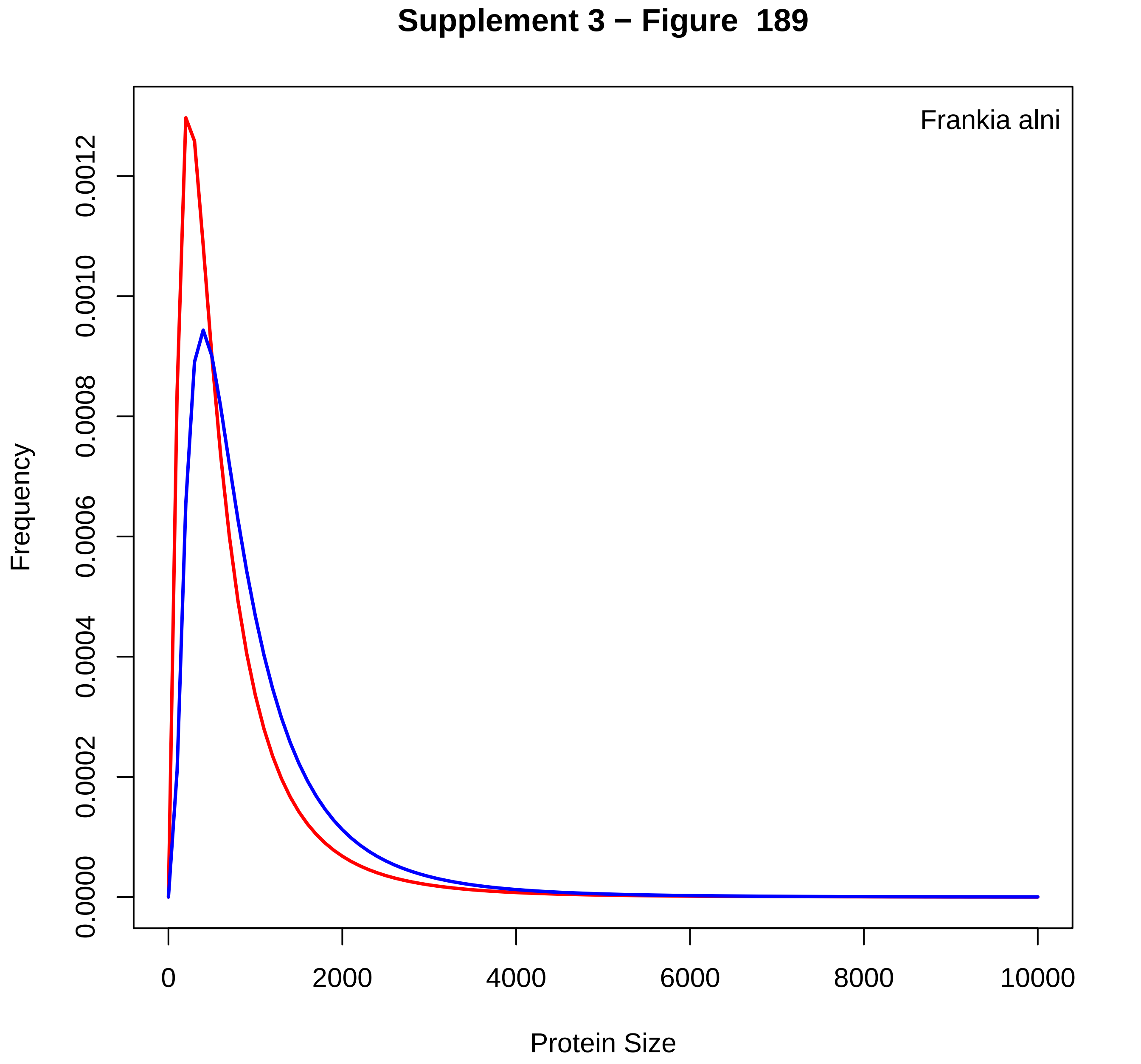

Supplement 3 − Figure 189
Frankia alni
Frequency
0.0000
0.0002
0.0004
0.0006
0.0008
0.0010
0.0012
0
2000
4000
6000
8000
10000
Protein Size

Supplement 3 − Figure 190

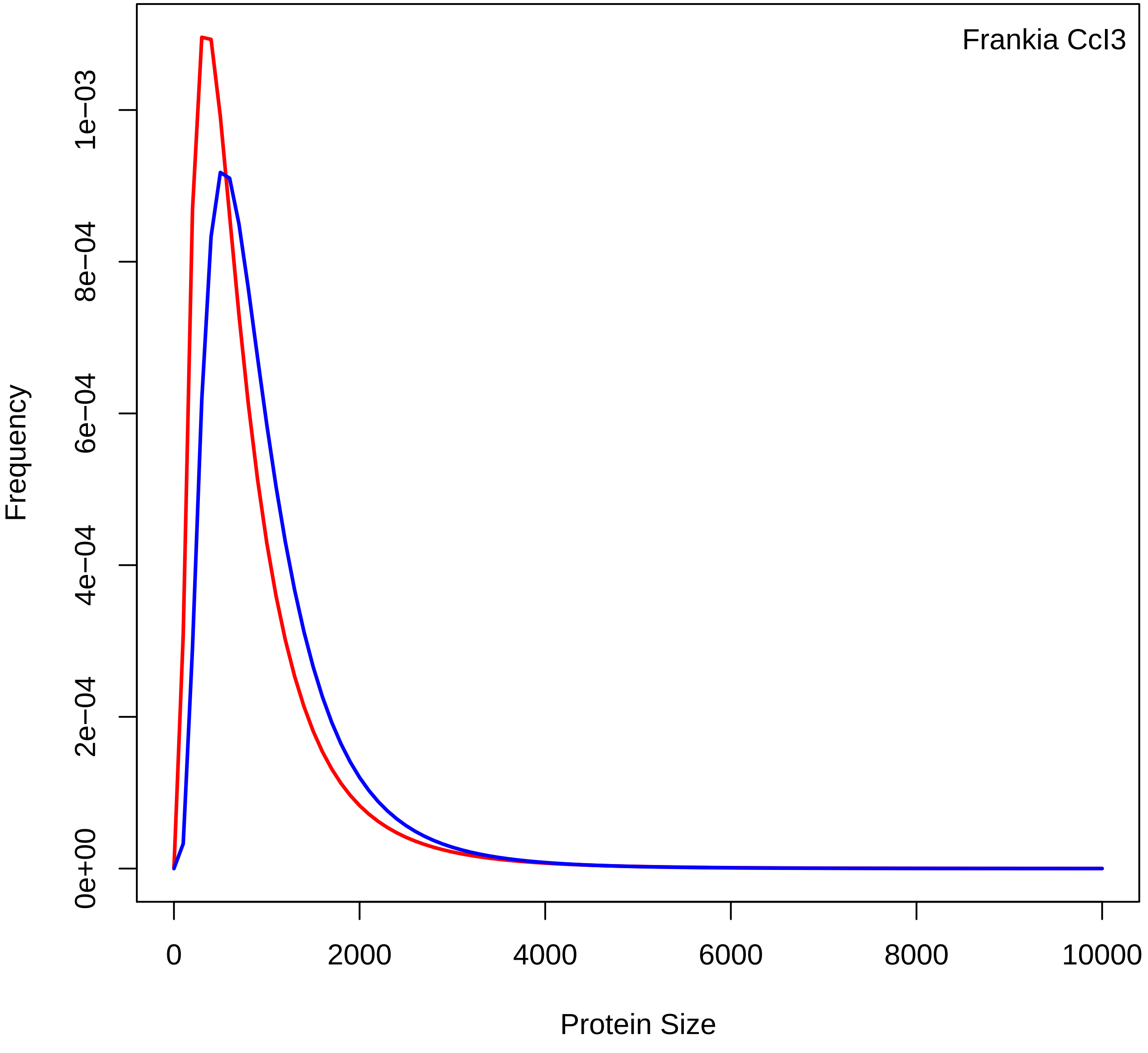

Supplement 3 − Figure 191

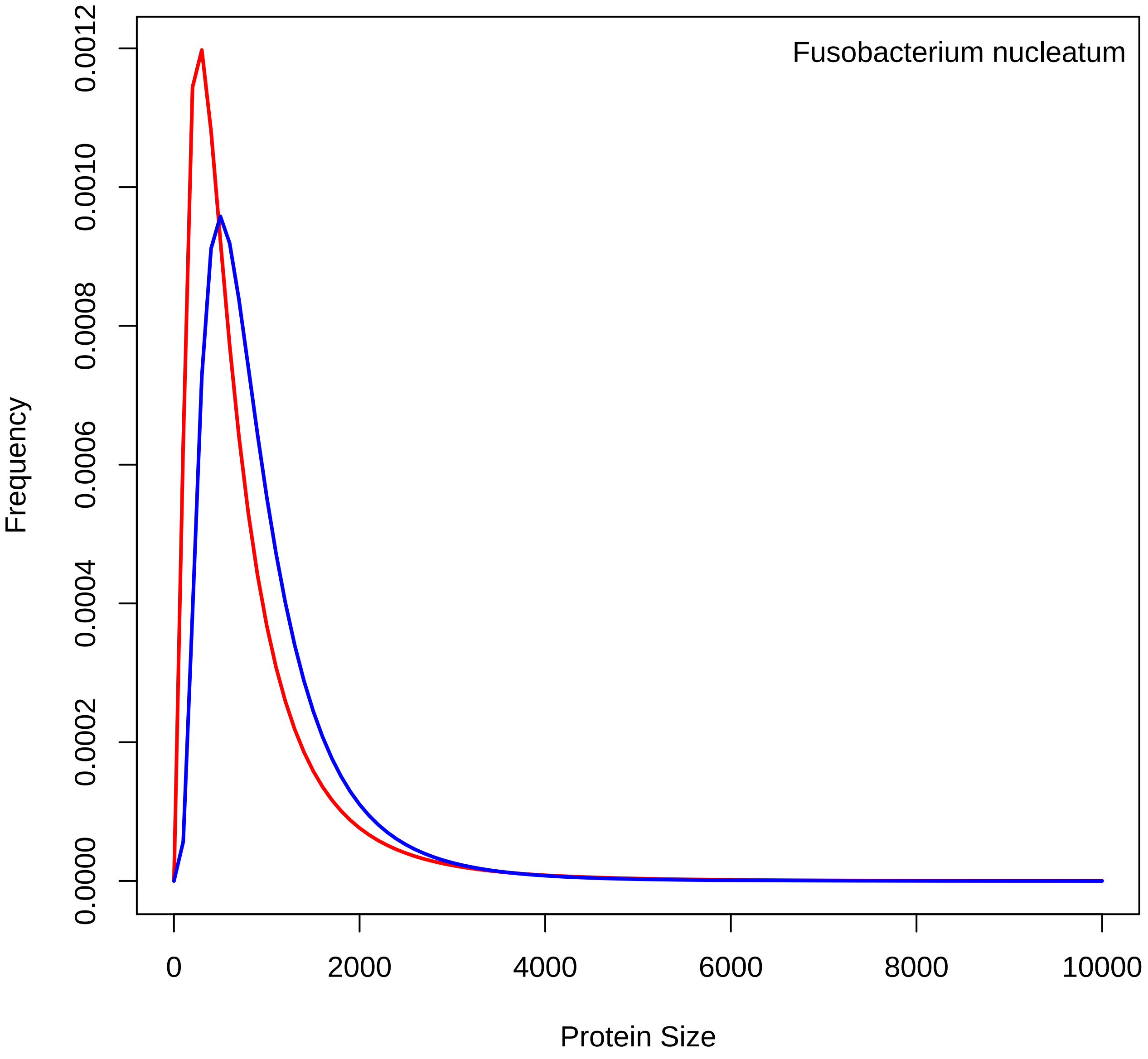

Supplement 3 − Figure 192

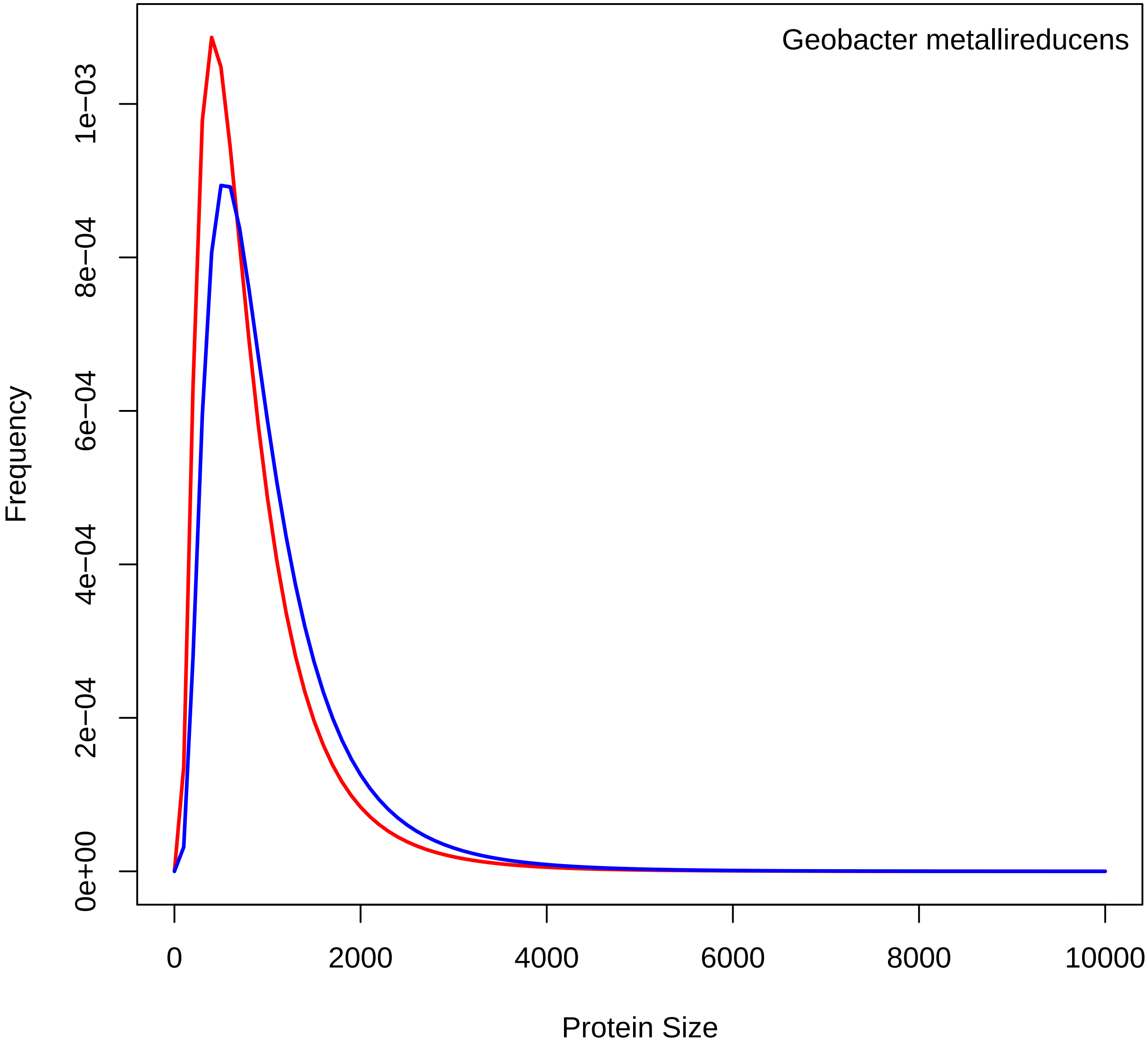

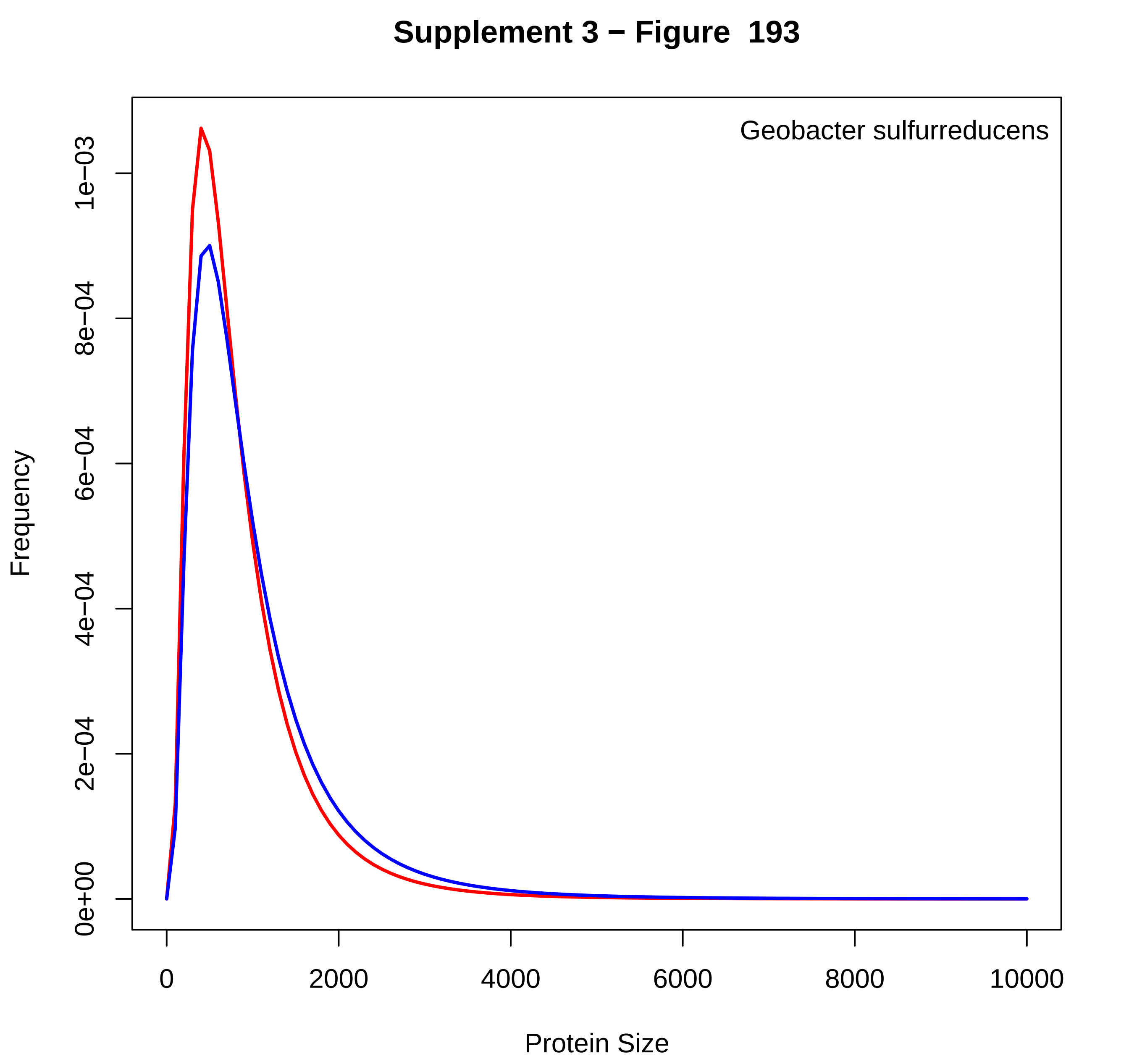
Supplement 3 − Figure 193
Geobacter sulfurreducens
Frequency
0e+00
2e−04
4e−04
6e−04
8e−04
1e−03
0
2000
4000
6000
8000
10000
Protein Size

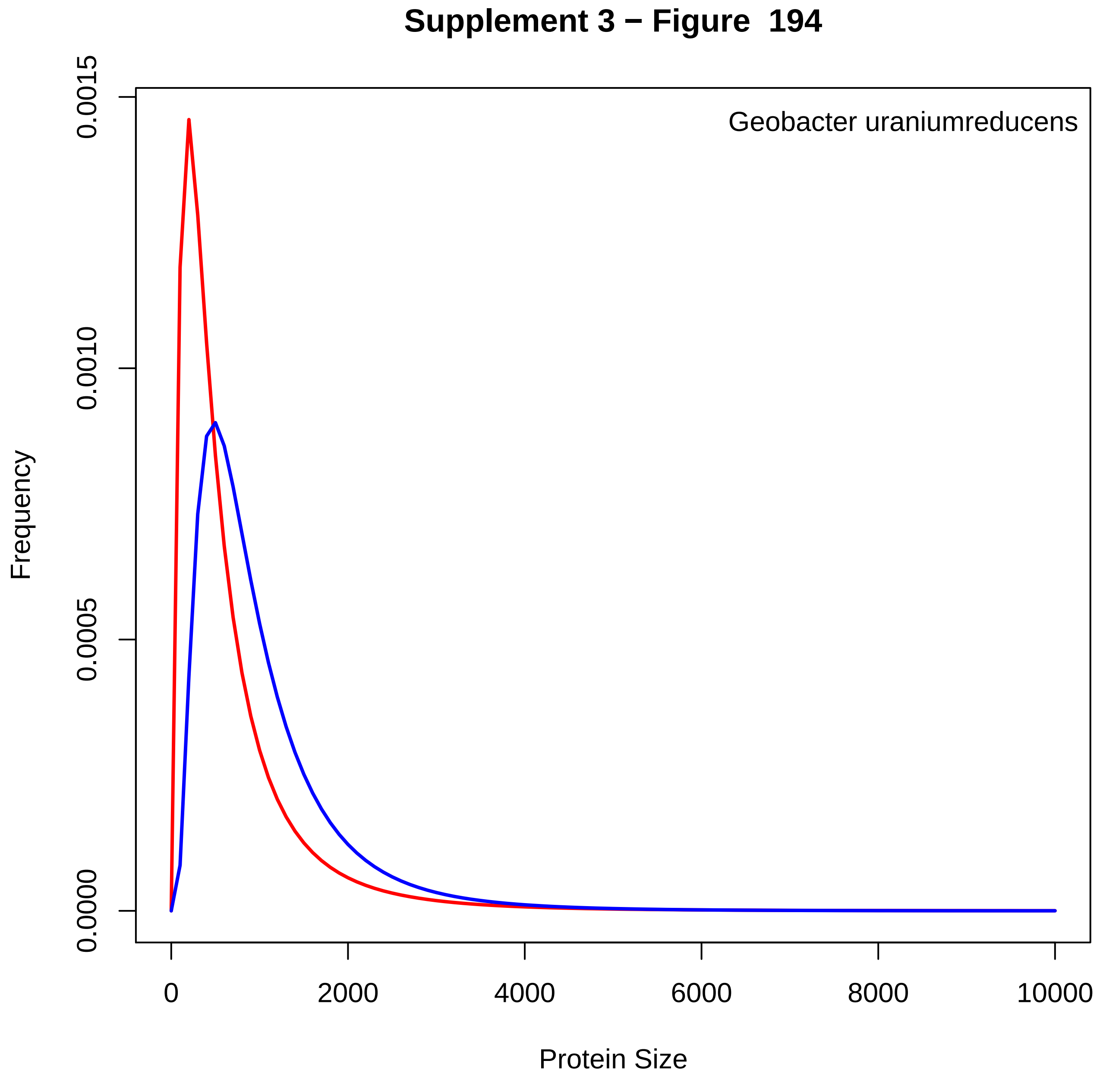
Supplement 3 − Figure 194
Geobacter uraniumreducens
Frequency
Protein Size
0.0000
0.0005
0.0010
0.0015
0
2000
4000
6000
8000
10000

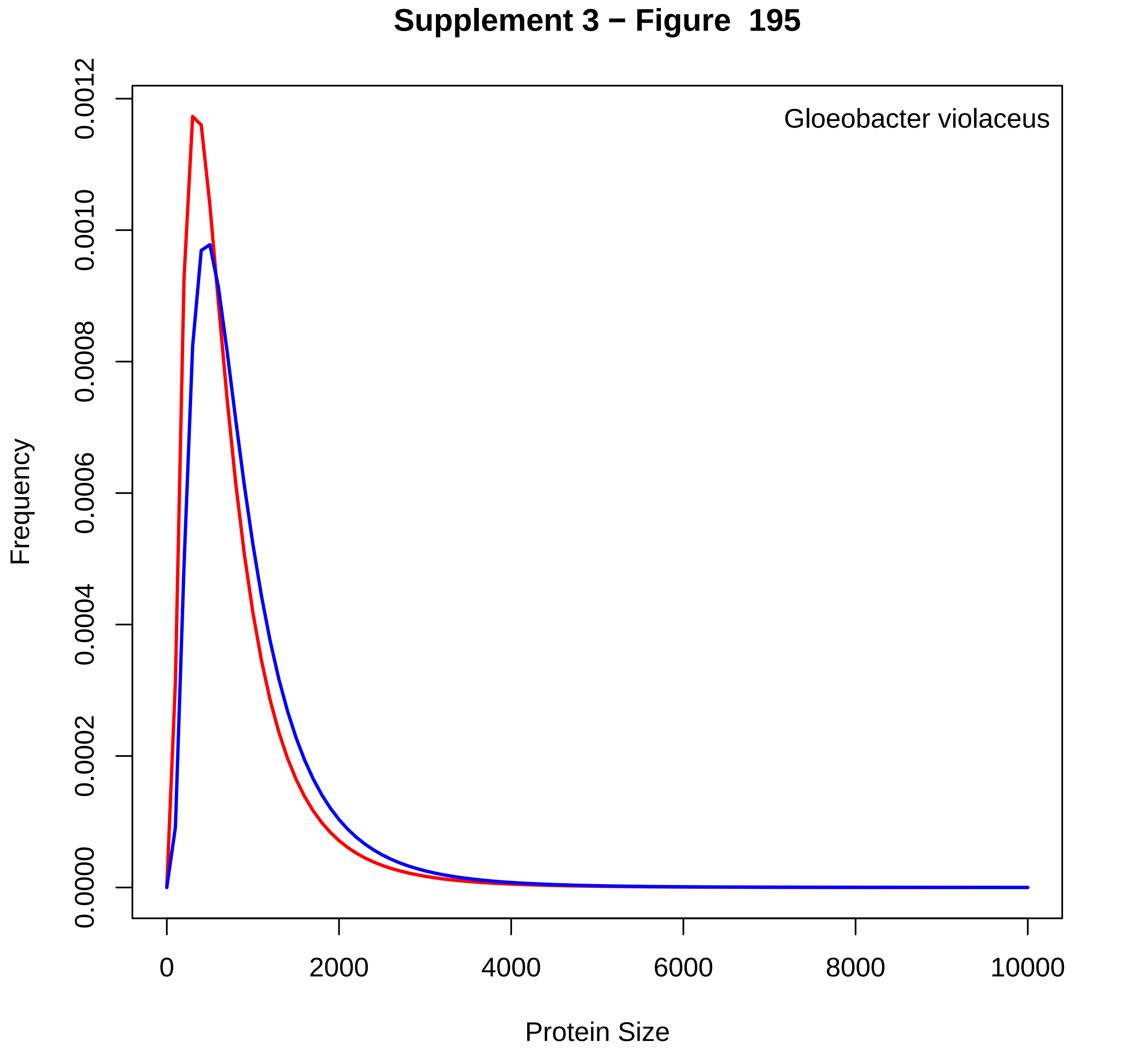

Supplement 3 − Figure 195
Gloeobacter violaceus
Frequency
0.0000
0.0002
0.0004
0.0006
0.0008
0.0010
0.0012
0
2000
4000
6000
8000
10000
Protein Size

Supplement 3 − Figure 196

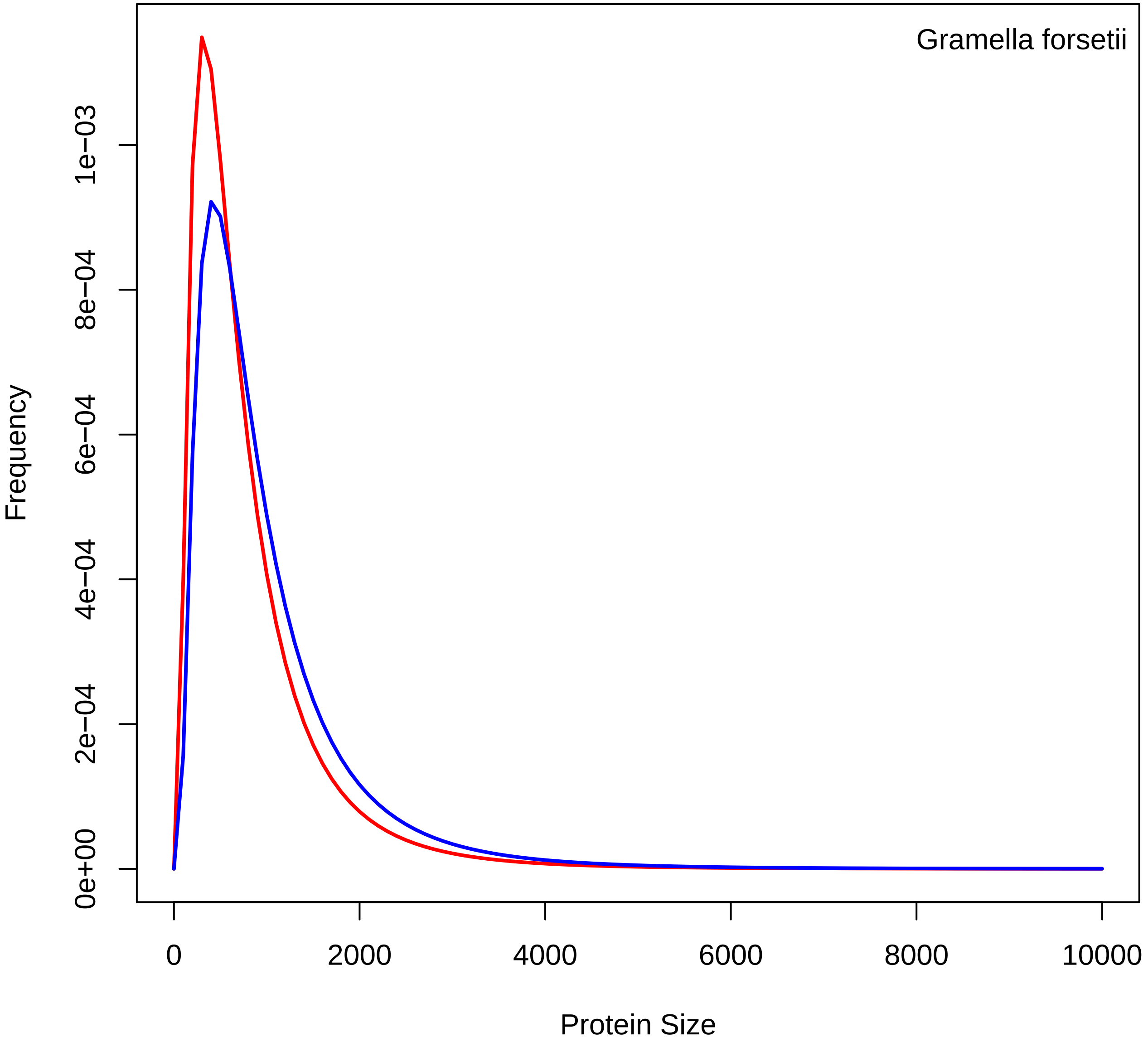

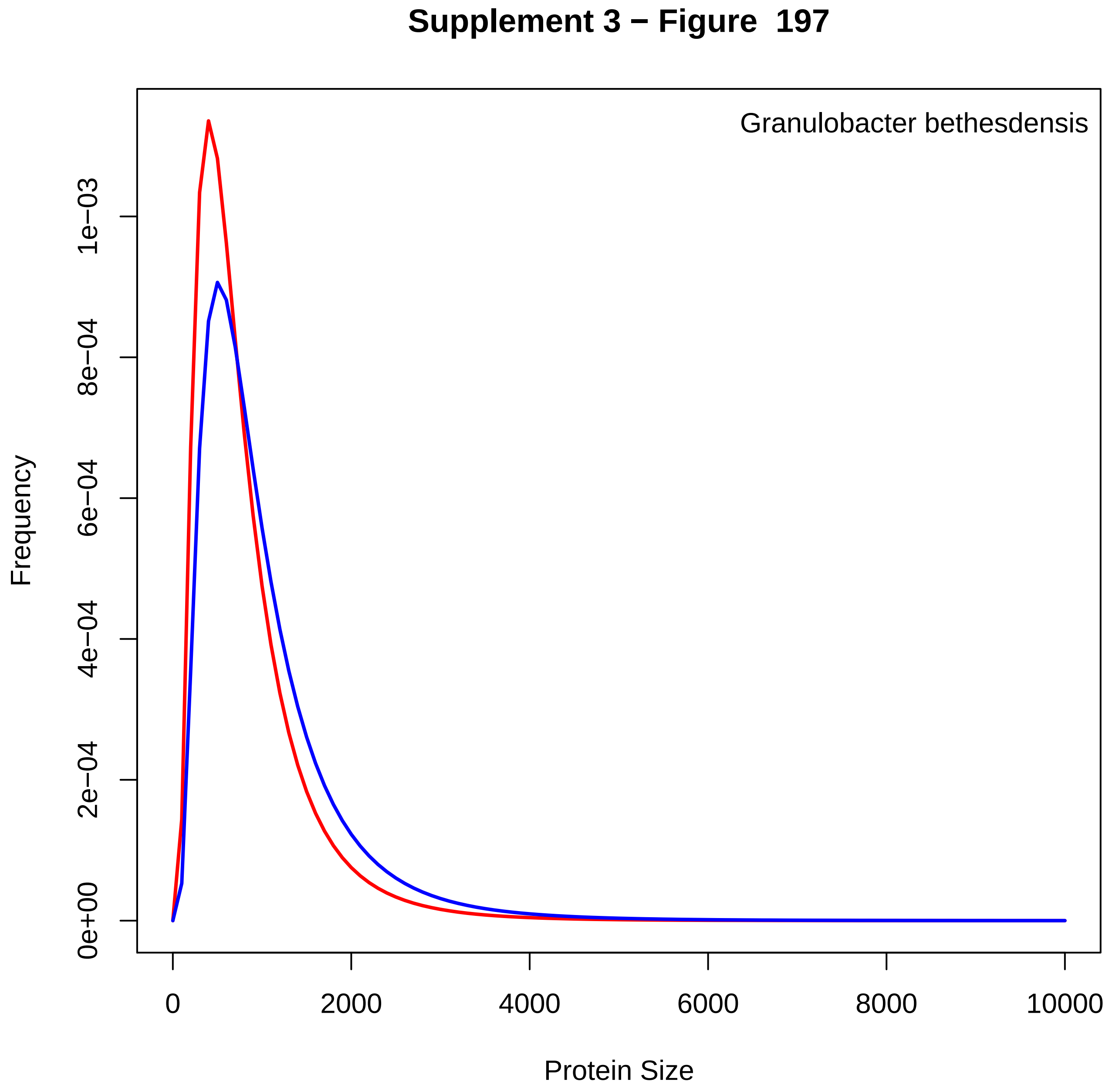

Supplement 3 − Figure 197
Granulobacter bethesdensis
Frequency
0e+00
2e−04
4e−04
6e−04
8e−04
1e−03
0
2000
4000
6000
8000
10000
Protein Size

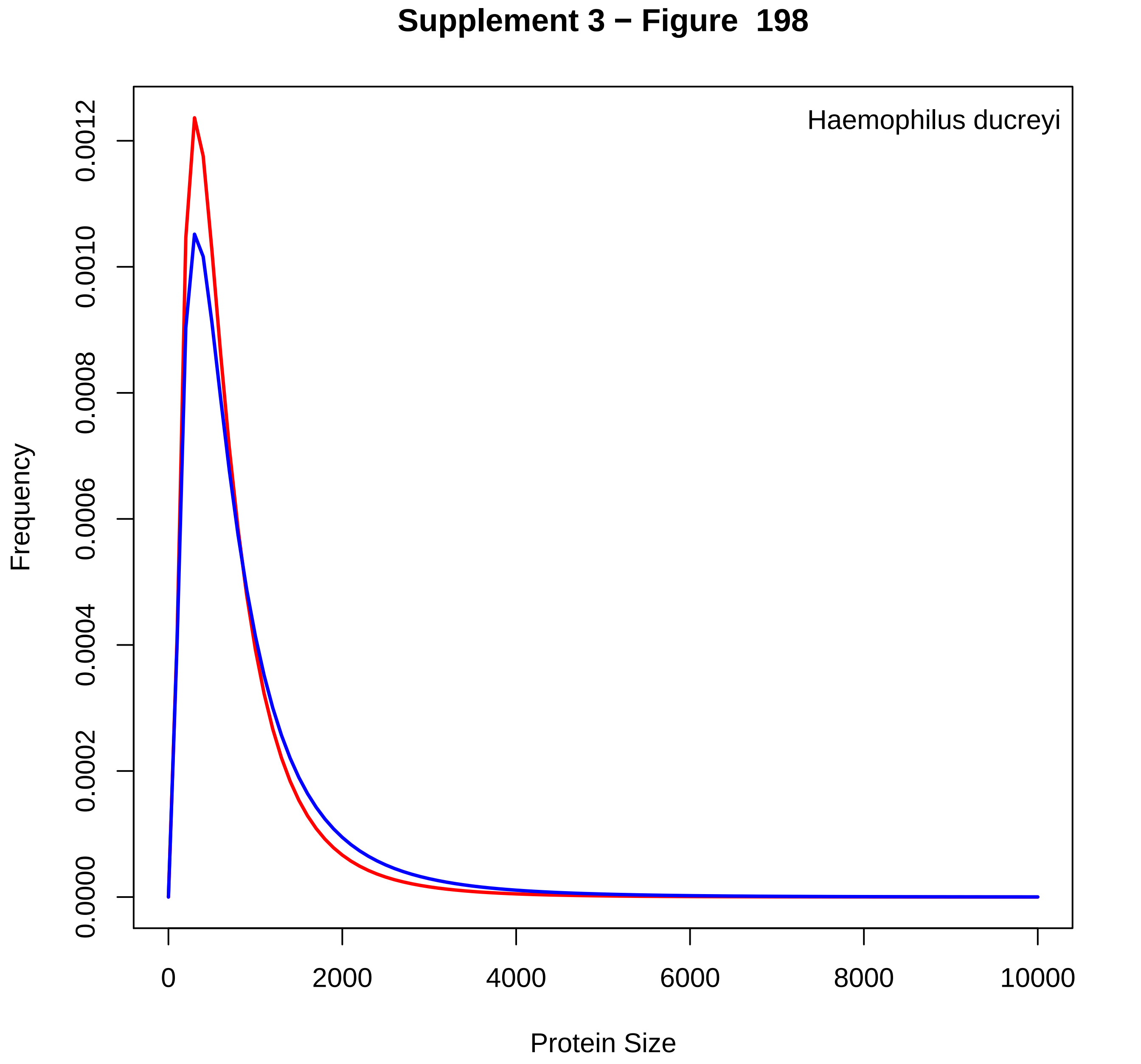

Supplement 3 − Figure 198
Haemophilus ducreyi
Frequency
0.0000
0.0002
0.0004
0.0006
0.0008
0.0010
0.0012
0
2000
4000
6000
8000
10000
Protein Size

Supplement 3 − Figure 199

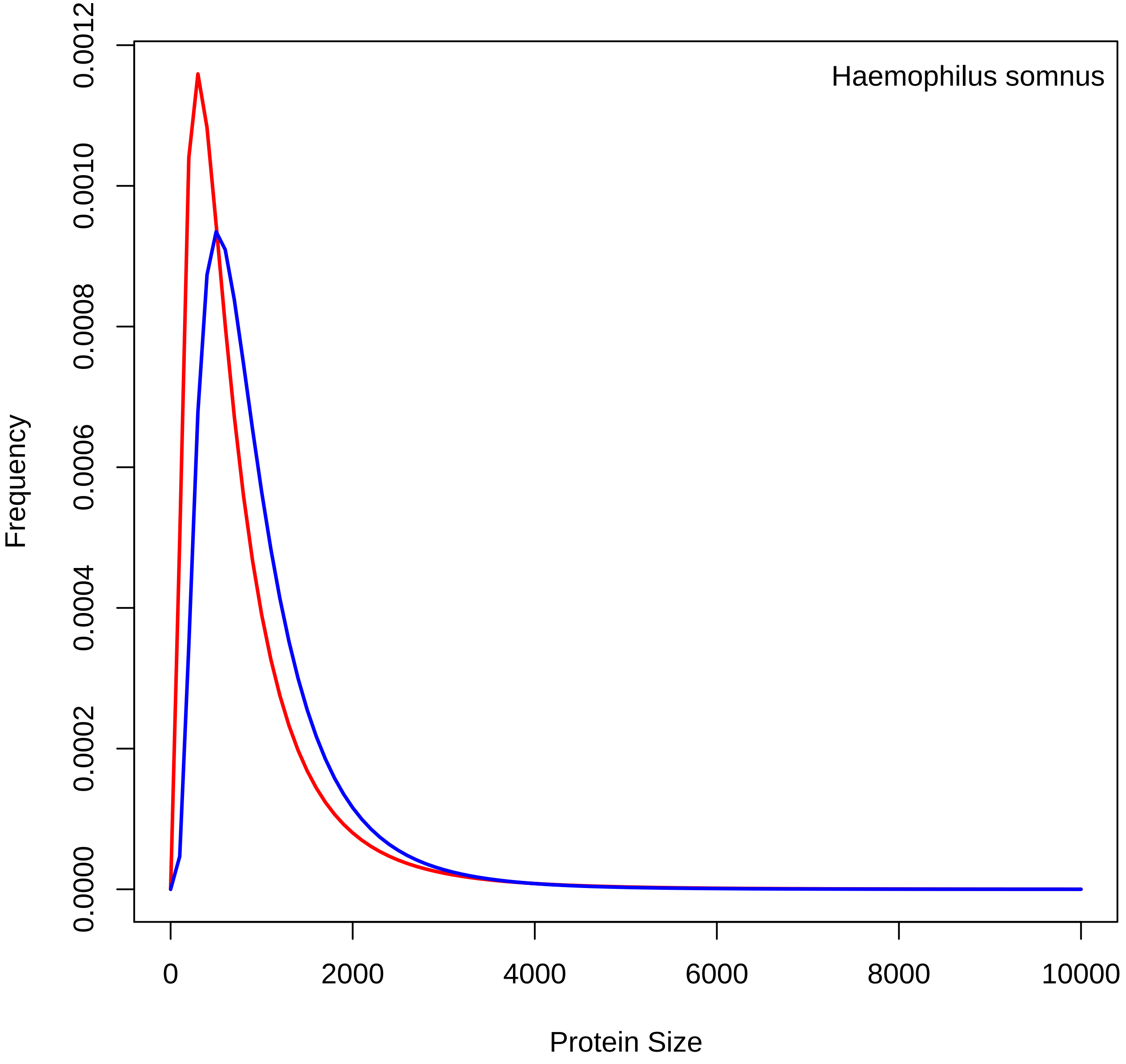

Supplement 3 − Figure 200

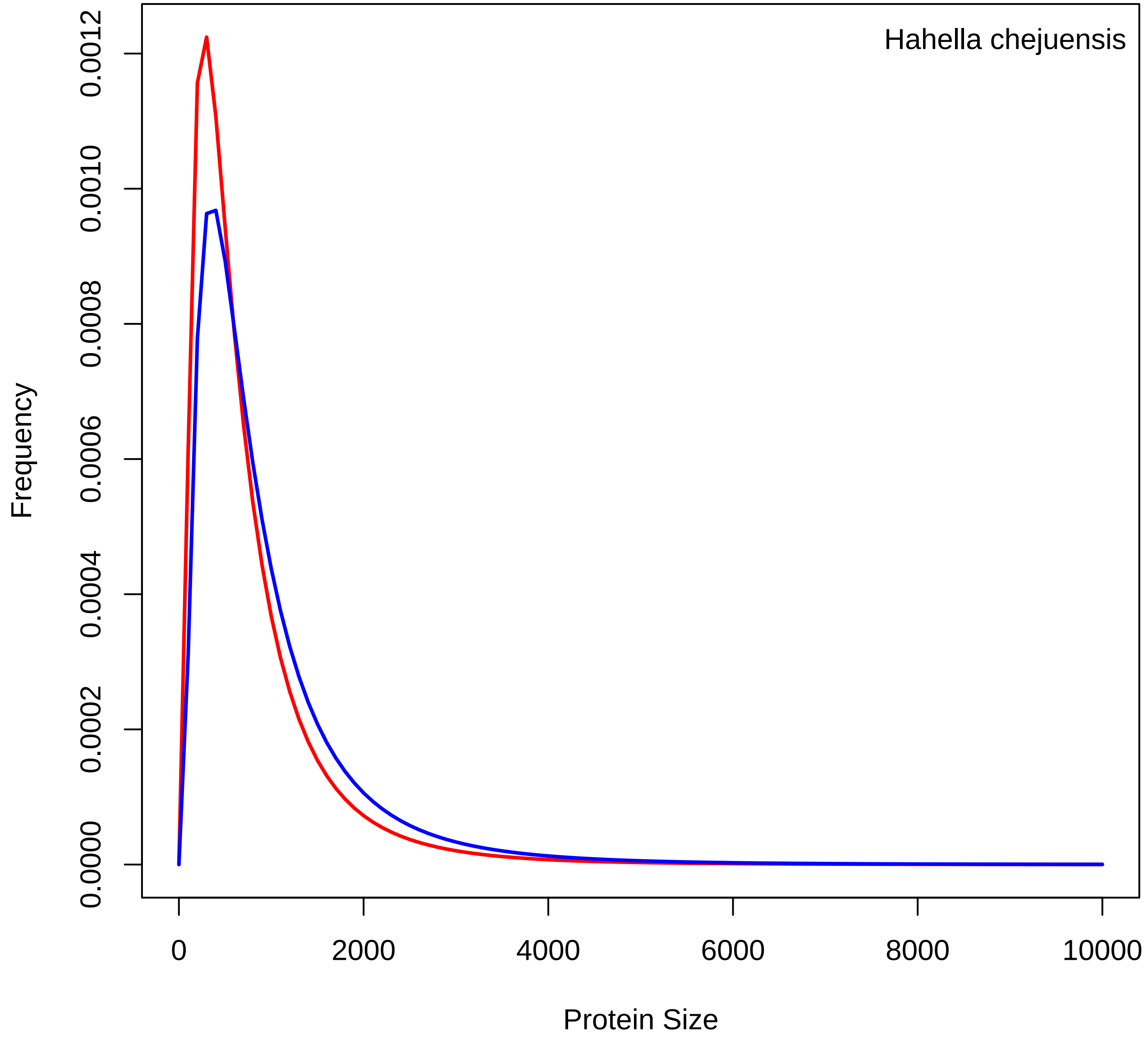

Supplement 3 − Figure 201

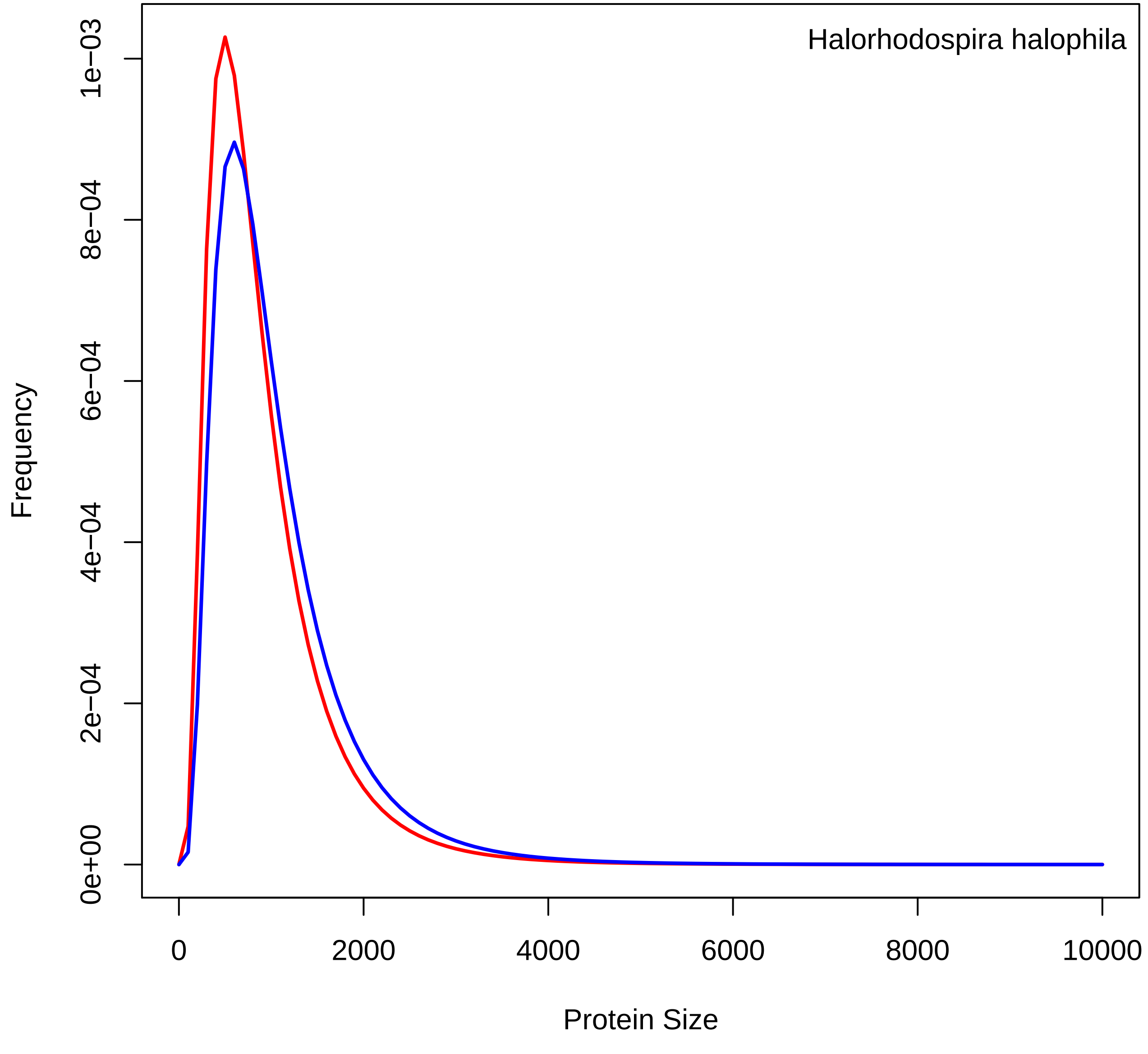

Supplement 3 − Figure 202

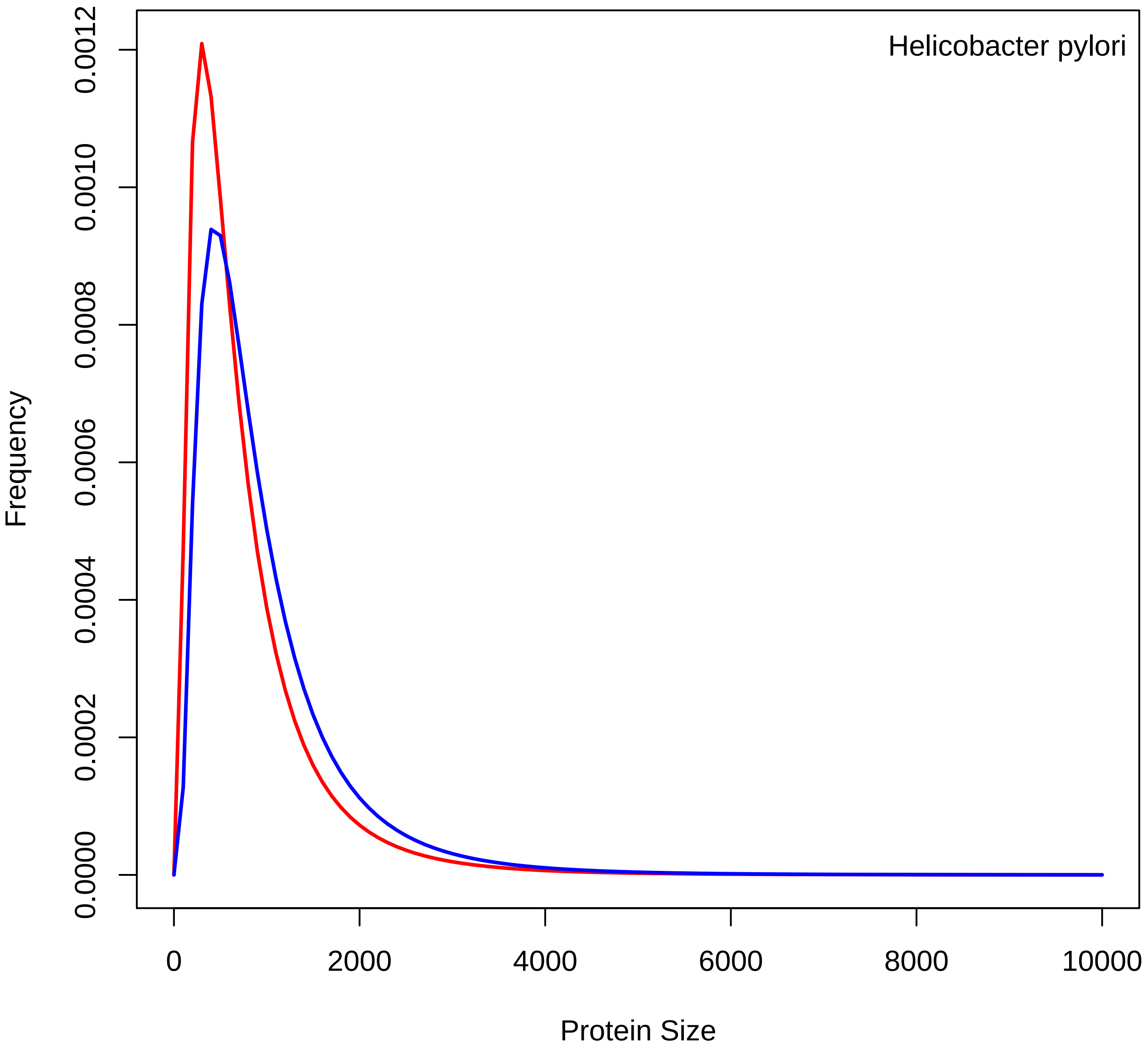

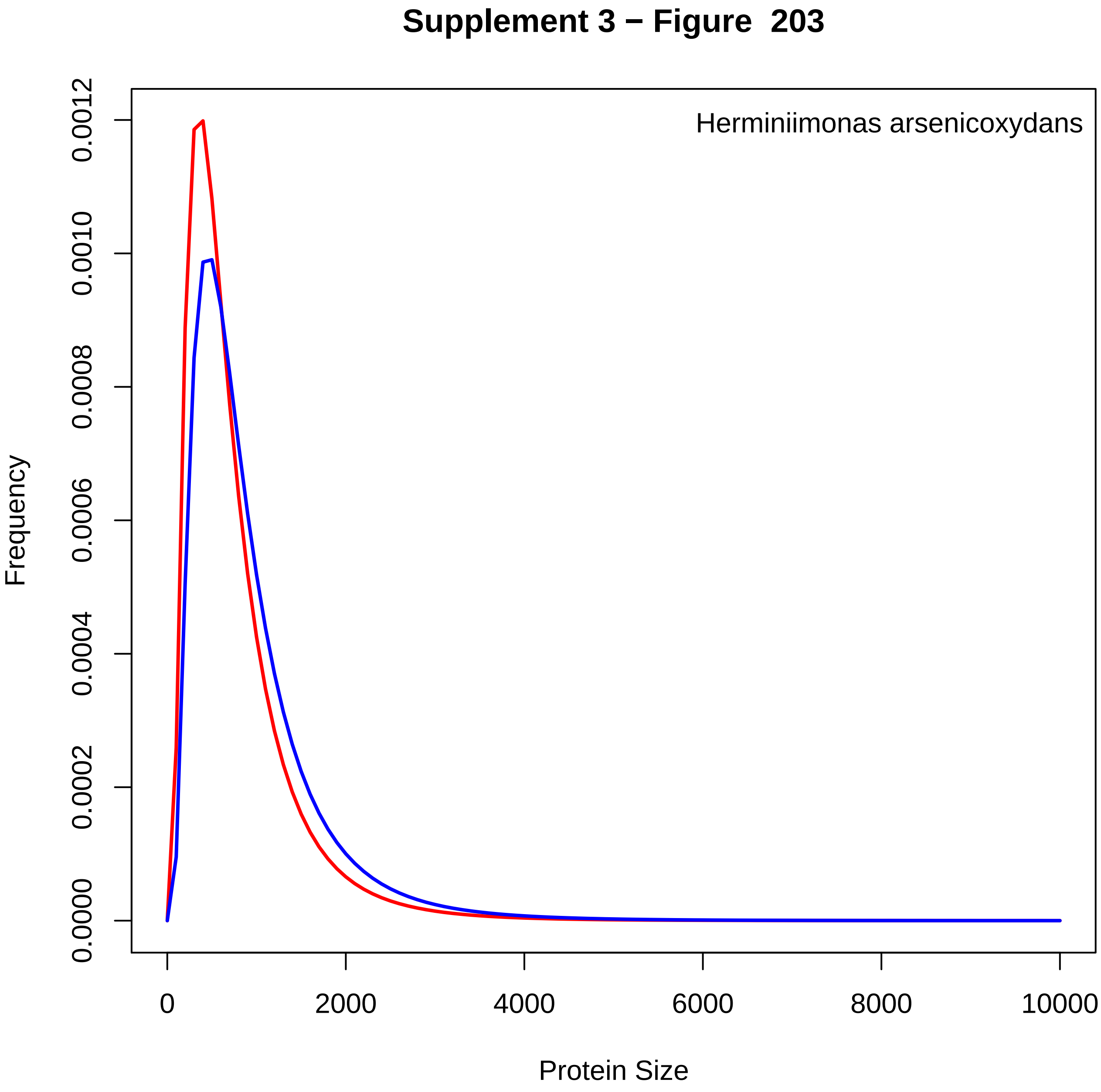

Supplement 3 − Figure 203
Herminiimonas arsenicoxydans
Frequency
0.0000
0.0002
0.0004
0.0006
0.0008
0.0010
0.0012
0
2000
4000
6000
8000
10000
Protein Size

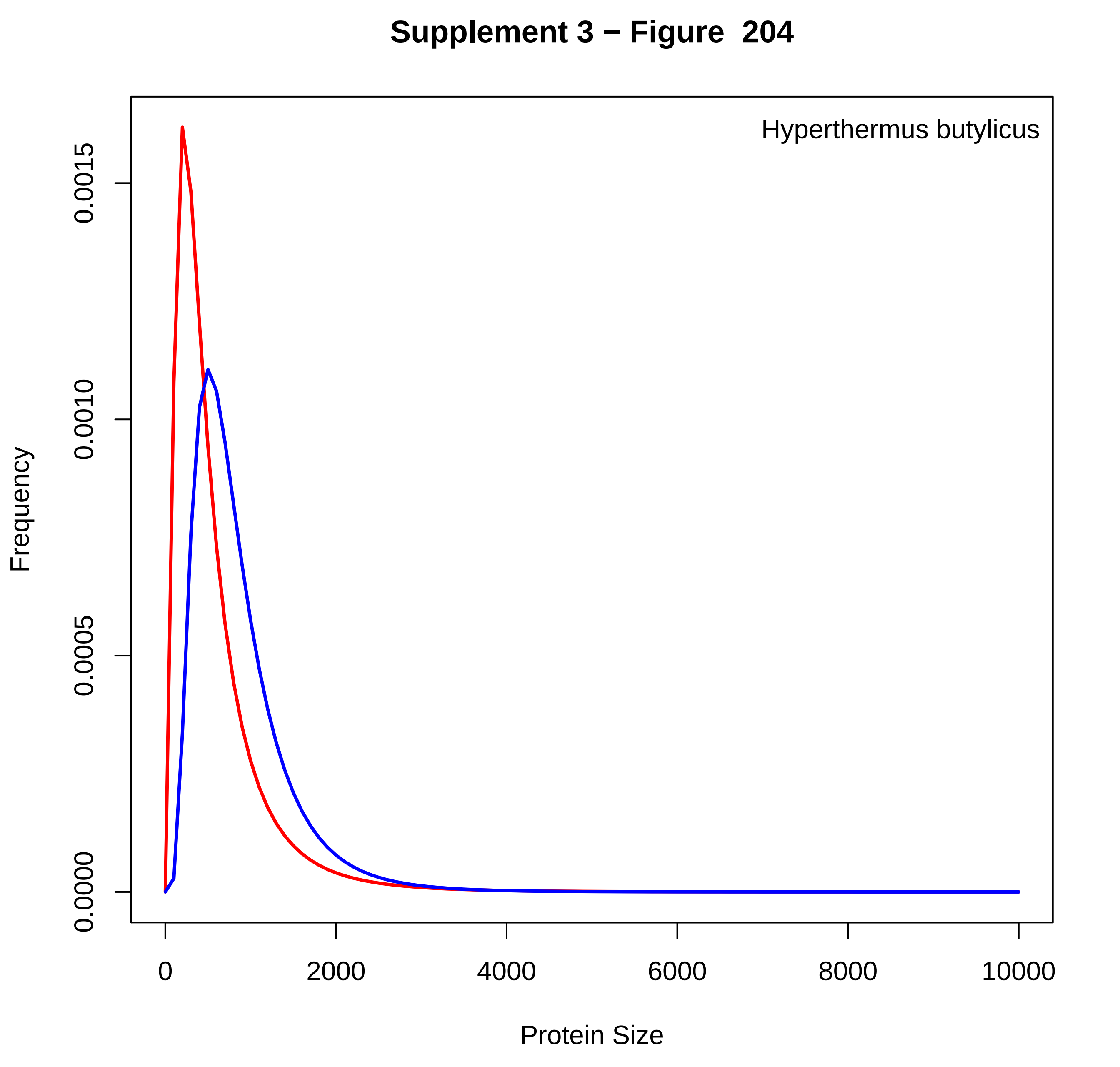
Supplement 3 − Figure 204
Hyperthermus butylicus
Frequency
0.0000
0.0005
0.0010
0.0015
0
2000
4000
6000
8000
10000
Protein Size

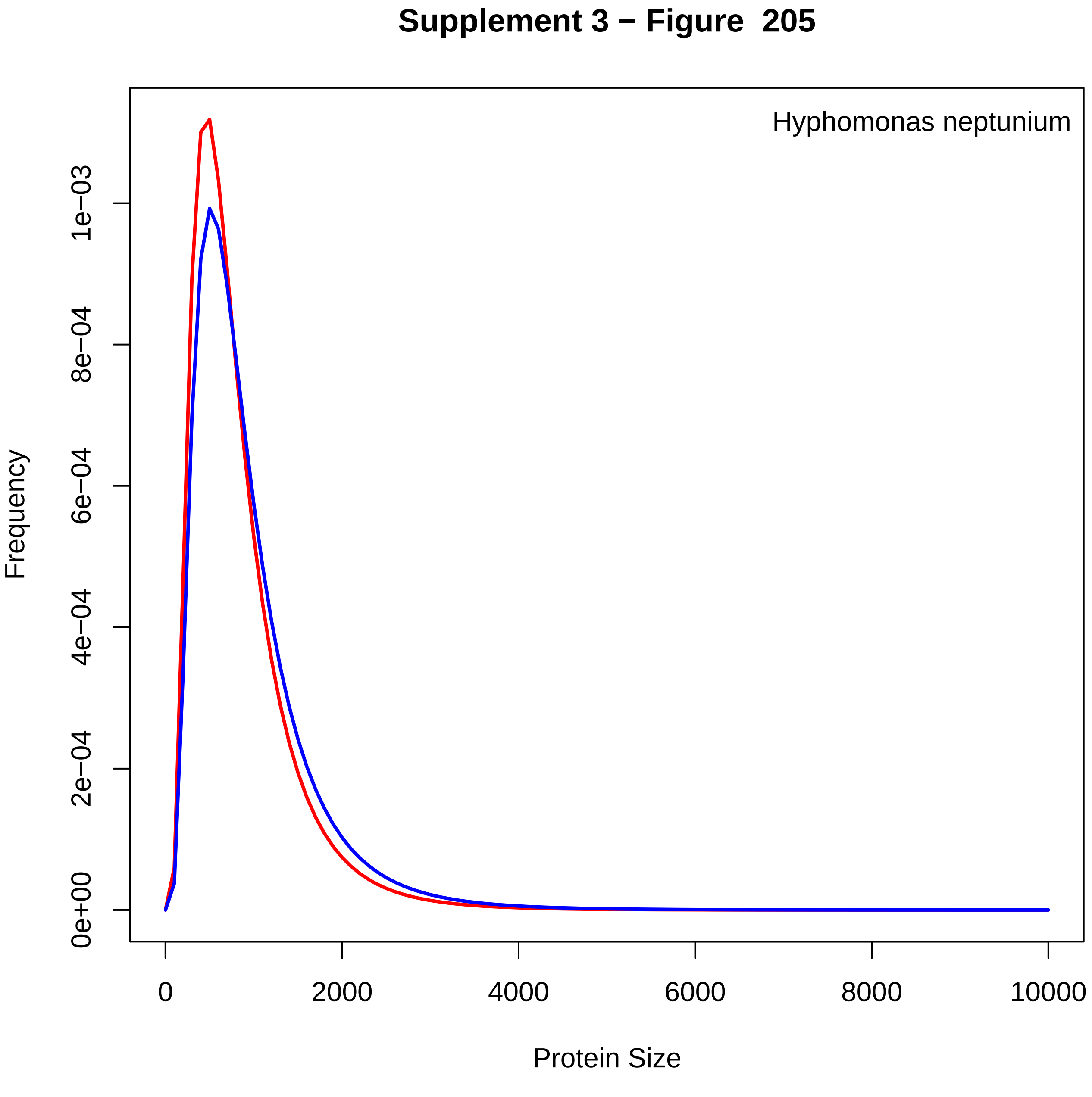
Supplement 3 − Figure 205
Hyphomonas neptunium
Frequency
0e+00
2e−04
4e−04
6e−04
8e−04
1e−03
0
2000
4000
6000
8000
10000
Protein Size

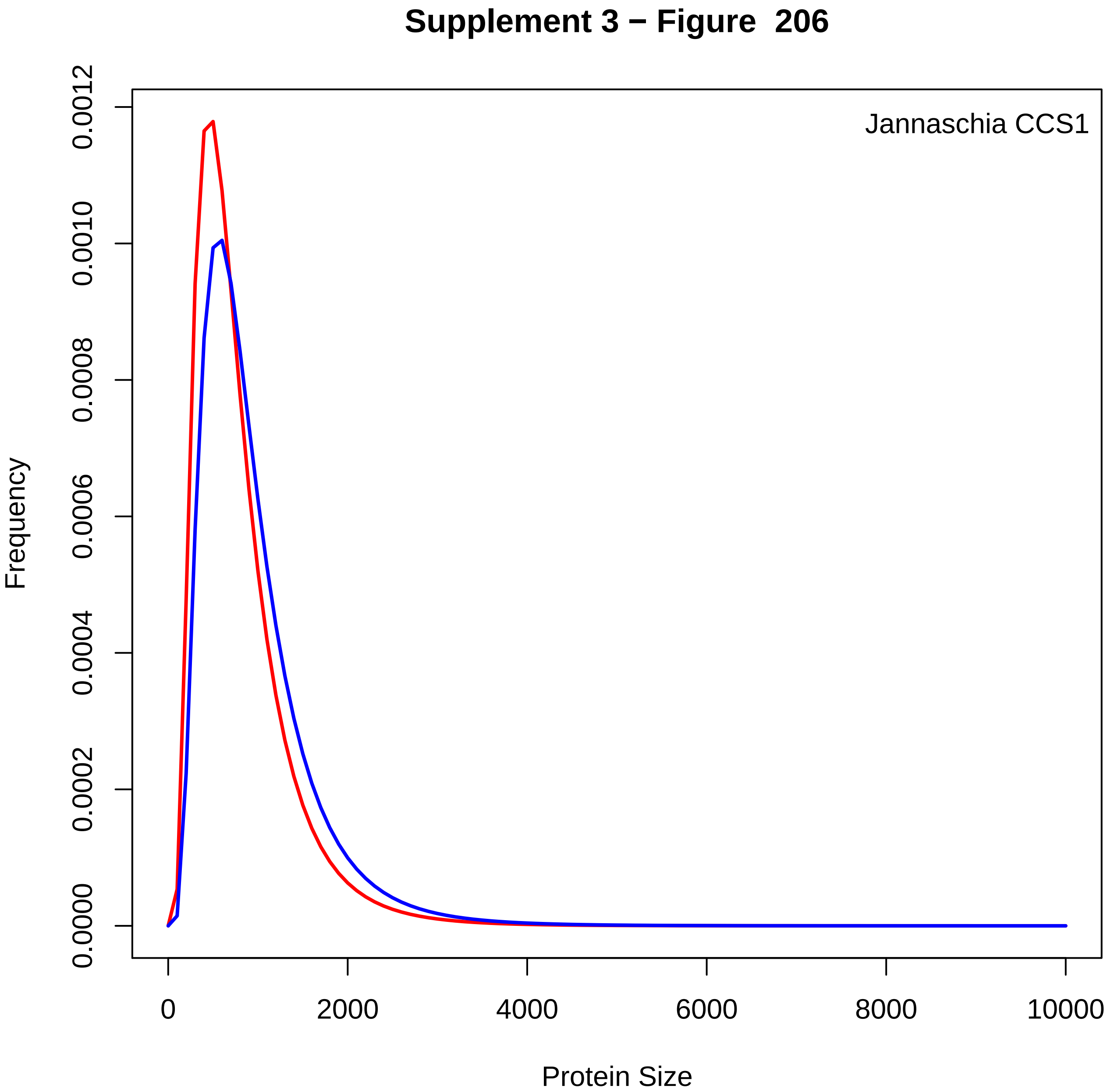
Supplement 3 − Figure 206
Jannaschia CCS1
Frequency
0.0000
0.0002
0.0004
0.0006
0.0008
0.0010
0.0012
0
2000
4000
6000
8000
10000
Protein Size

Supplement 3 − Figure 207

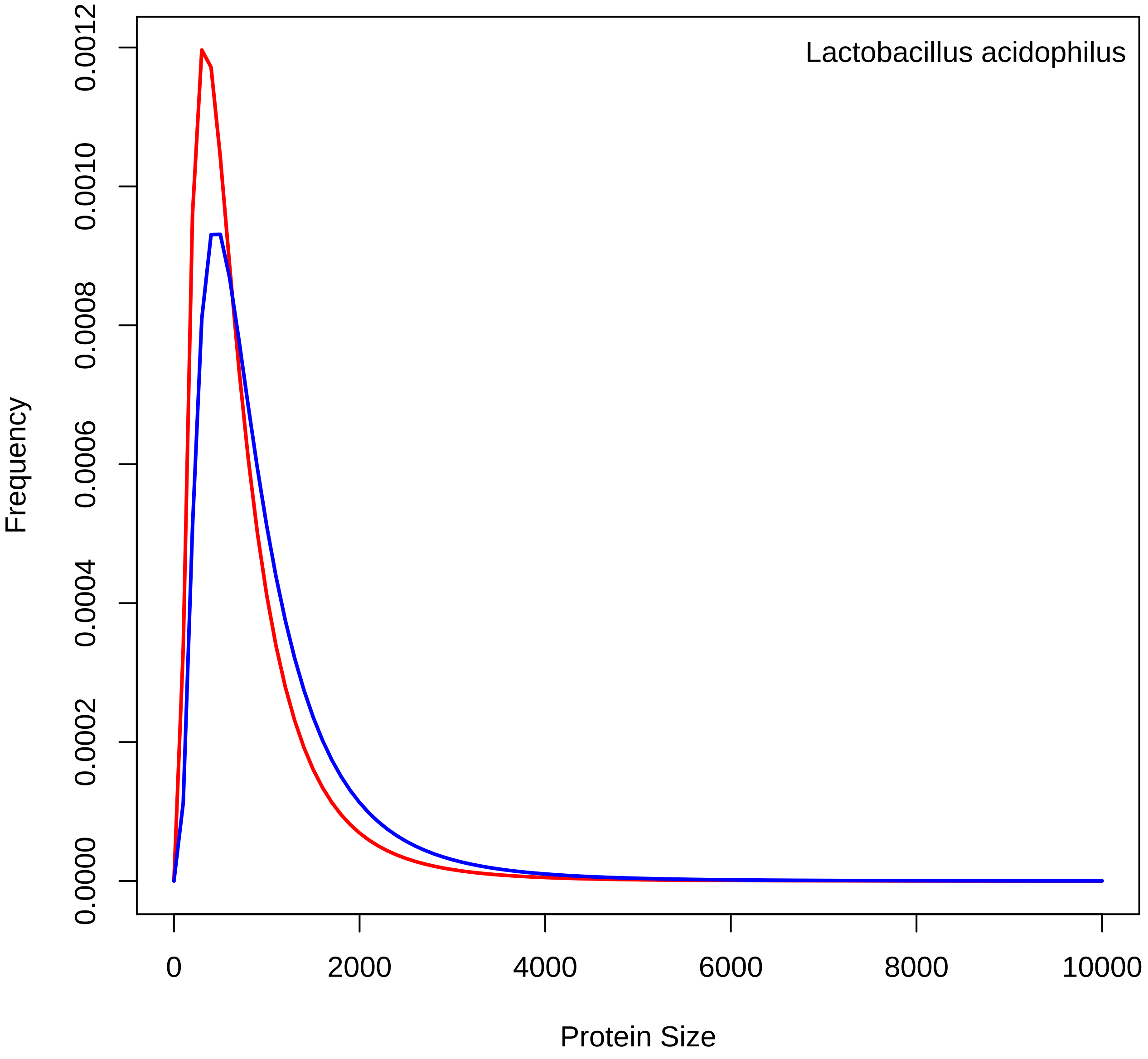

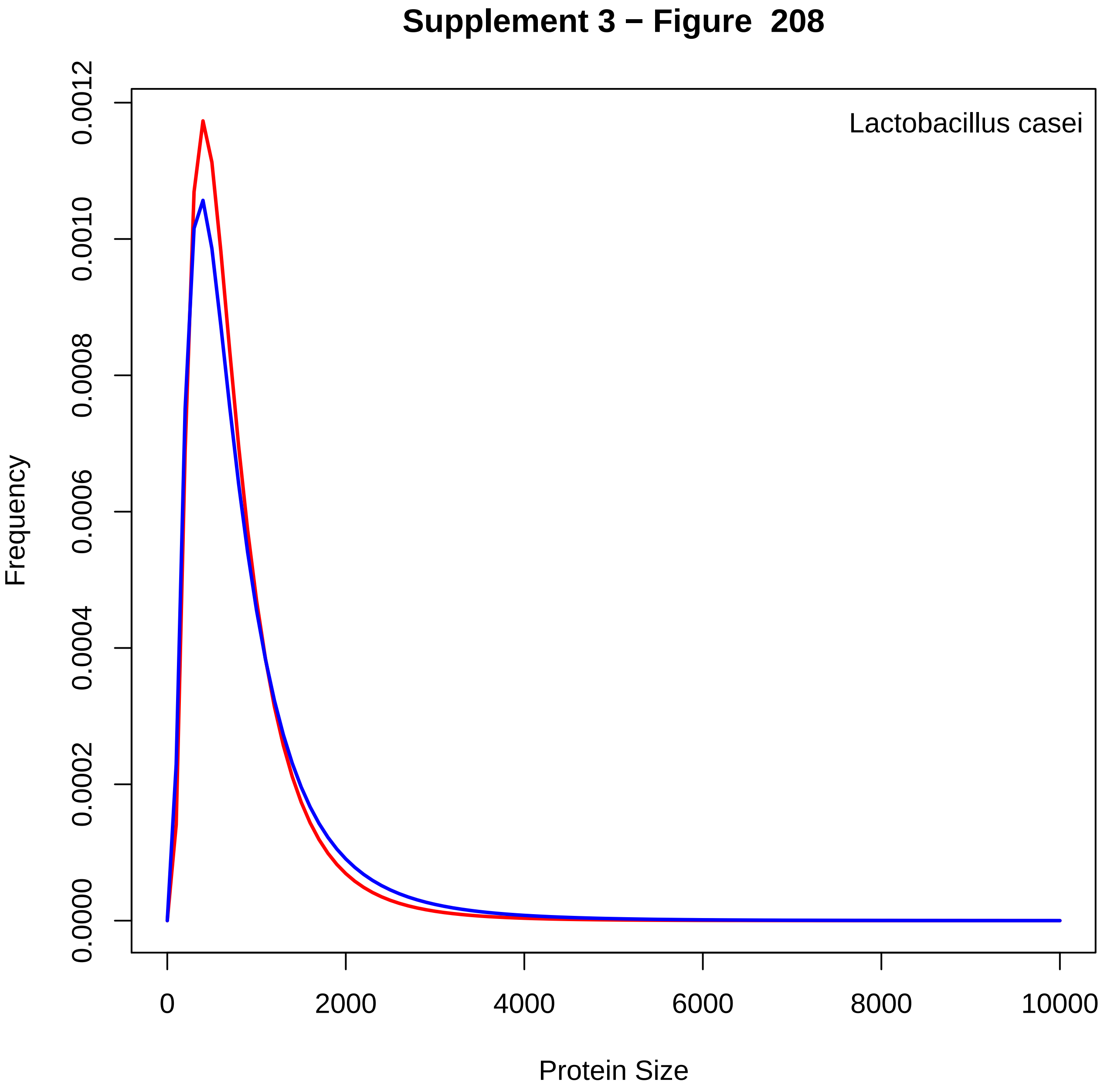
Supplement 3 − Figure 208
Lactobacillus casei
Frequency
0.0000
0.0002
0.0004
0.0006
0.0008
0.0010
0.0012
0
2000
4000
6000
8000
10000
Protein Size

Supplement 3 − Figure 209

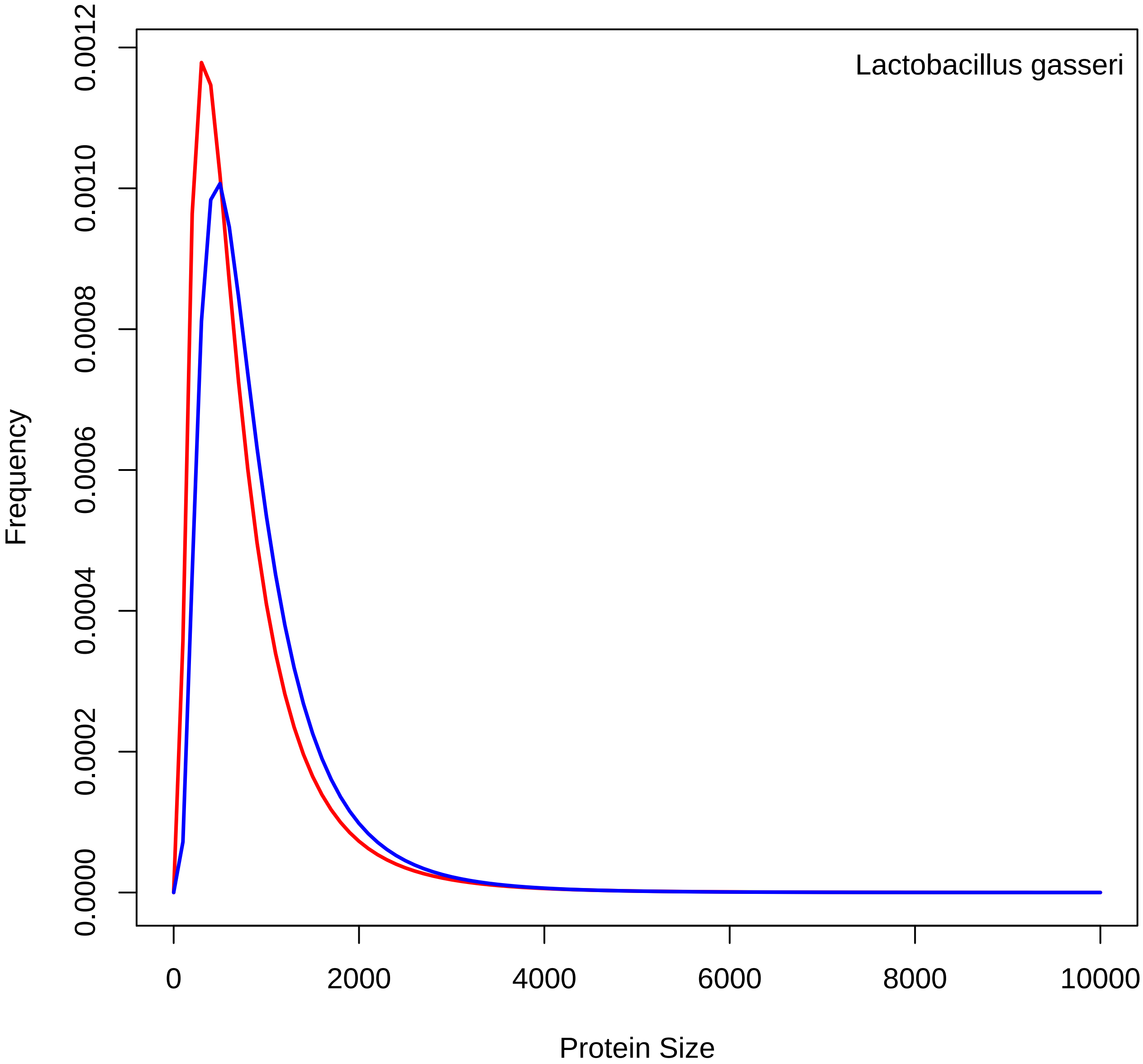

Supplement 3 − Figure 210

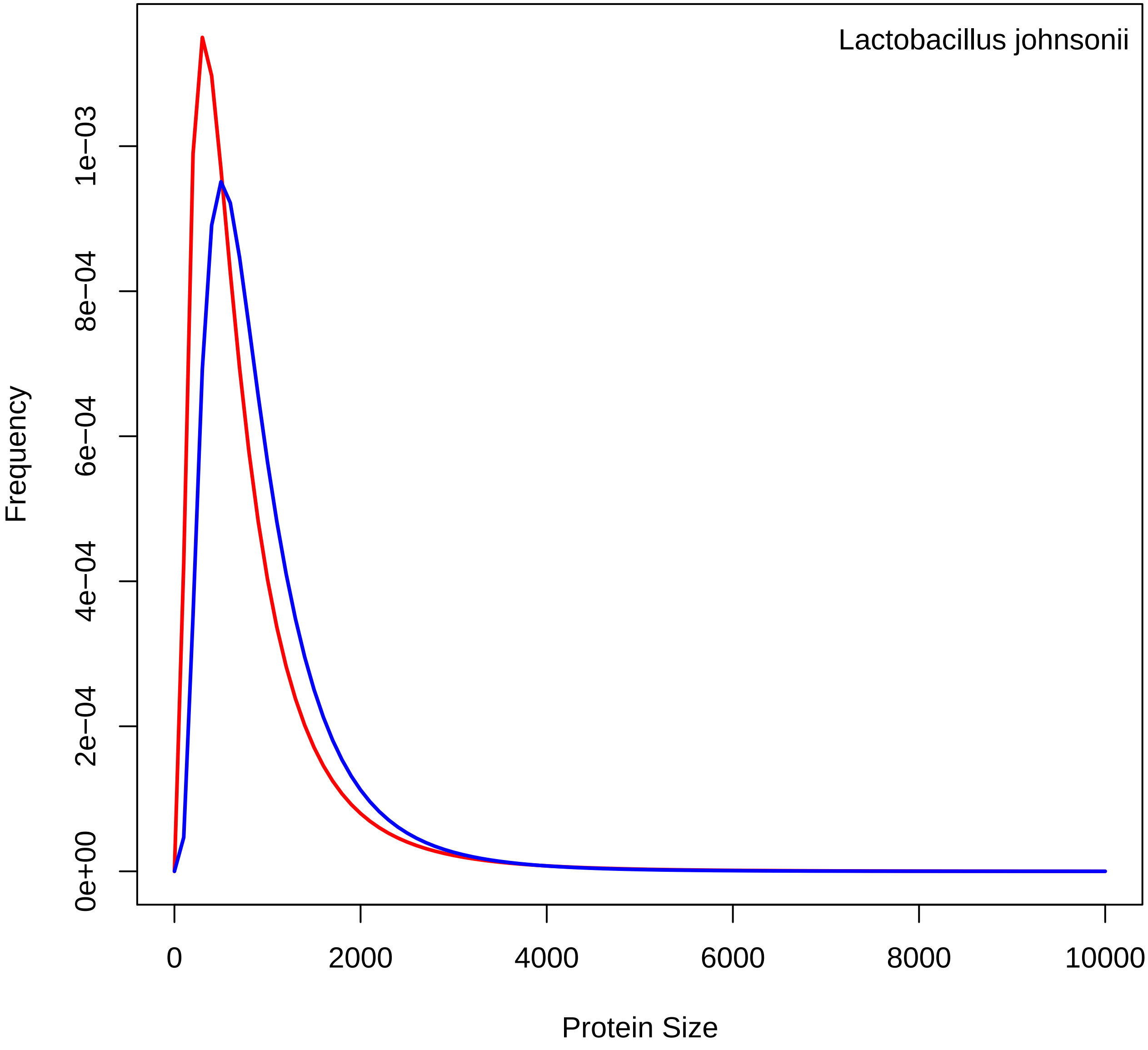

Supplement 3 − Figure 211

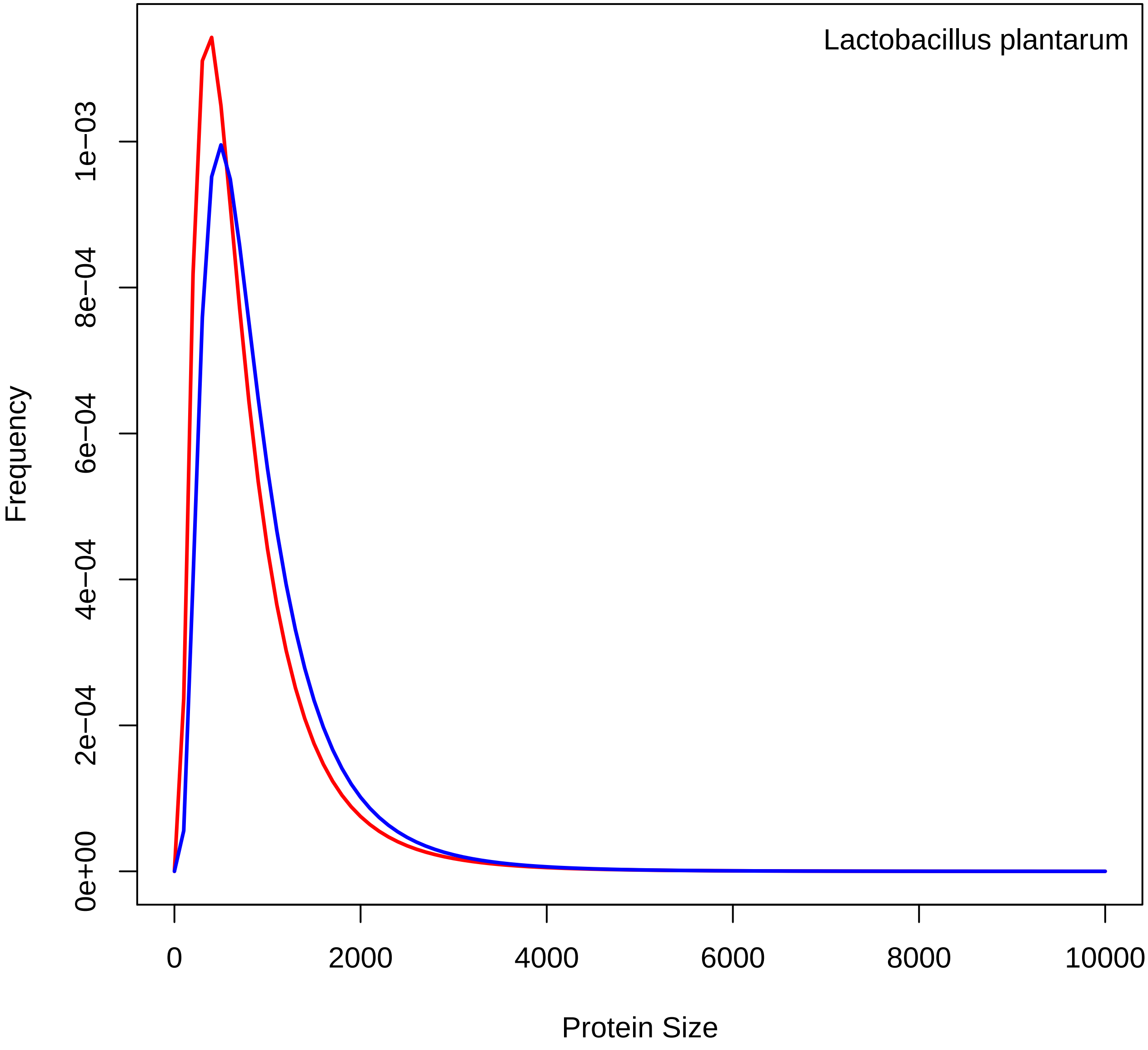

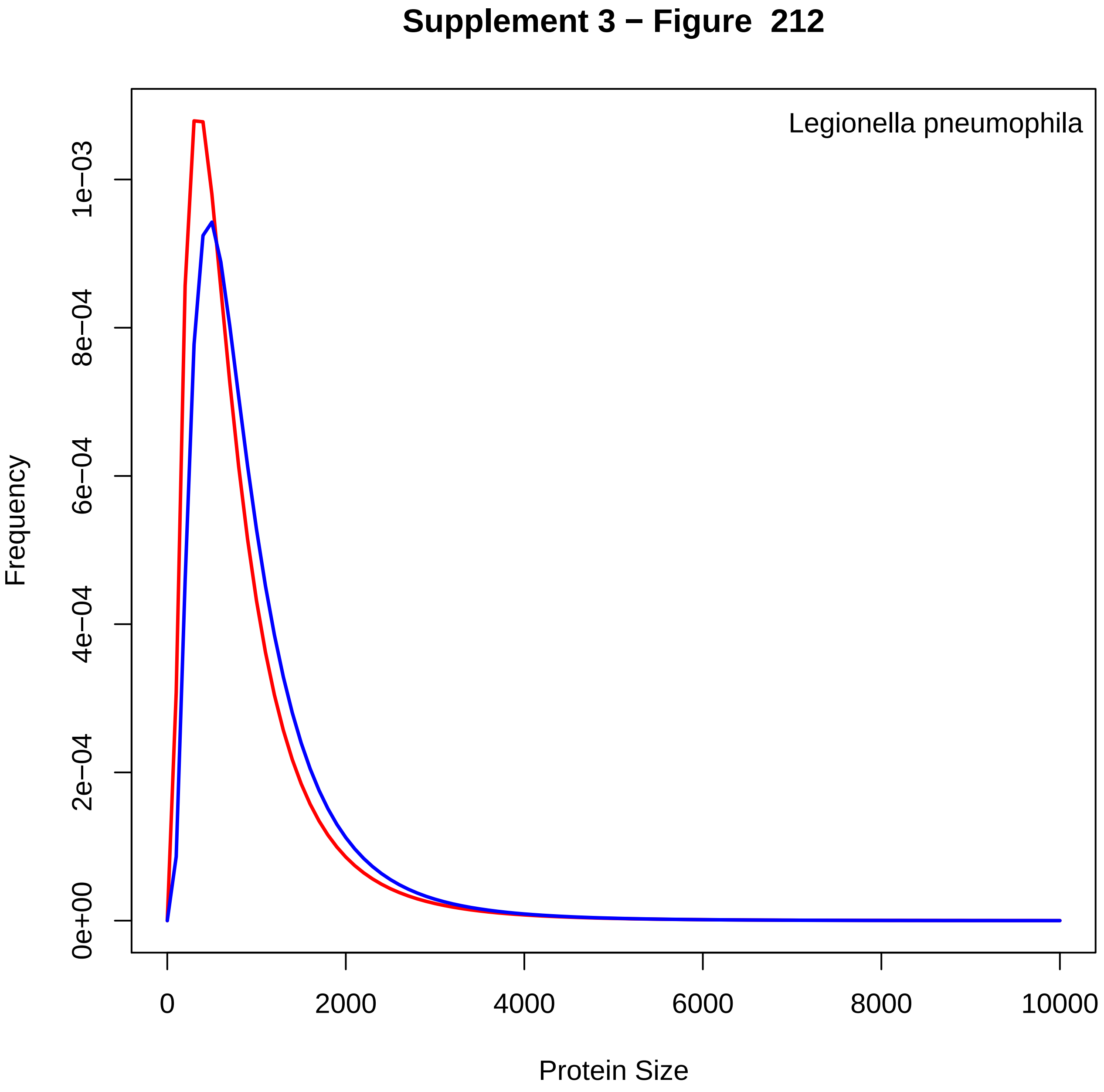
Supplement 3 − Figure 212
Legionella pneumophila
Frequency
0e+00
2e−04
4e−04
6e−04
8e−04
1e−03
0
2000
4000
6000
8000
10000
Protein Size

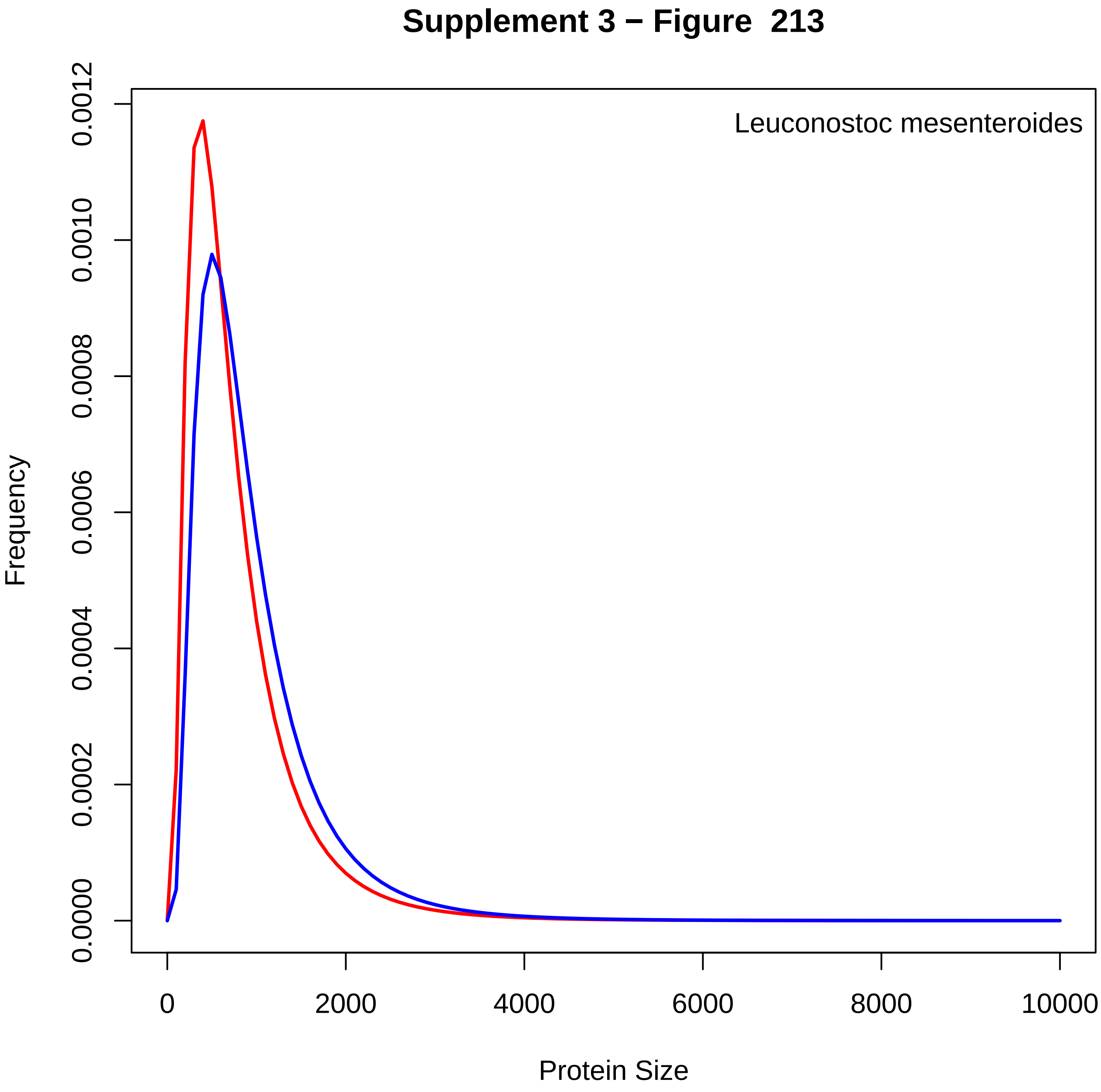

Supplement 3 − Figure 213
Leuconostoc mesenteroides
Frequency
0.0000
0.0002
0.0004
0.0006
0.0008
0.0010
0.0012
0
2000
4000
6000
8000
10000
Protein Size

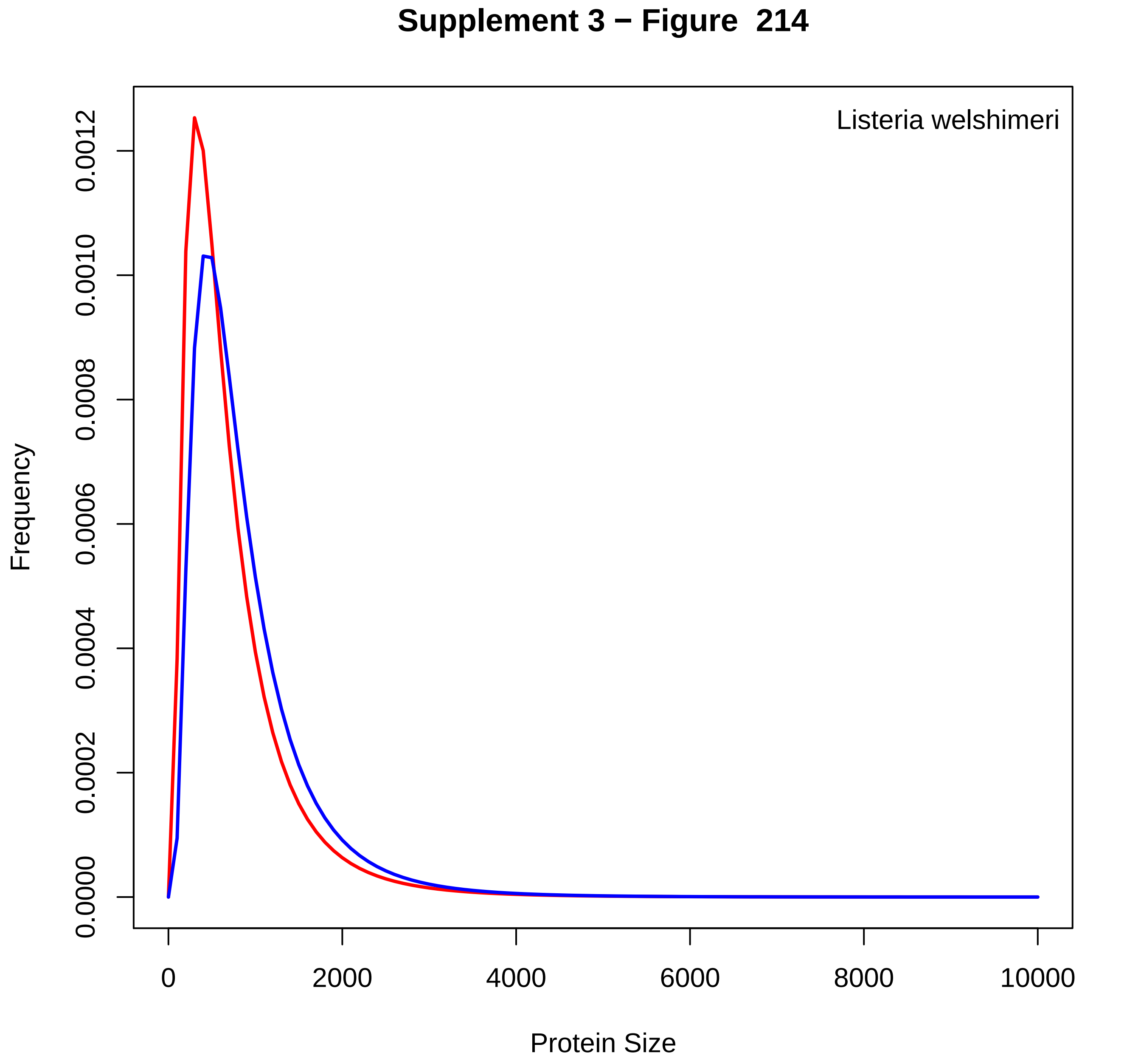

Supplement 3 − Figure 214
Listeria welshimeri
Frequency
0.0000
0.0002
0.0004
0.0006
0.0008
0.0010
0.0012
0
2000
4000
6000
8000
10000
Protein Size

Supplement 3 − Figure 215

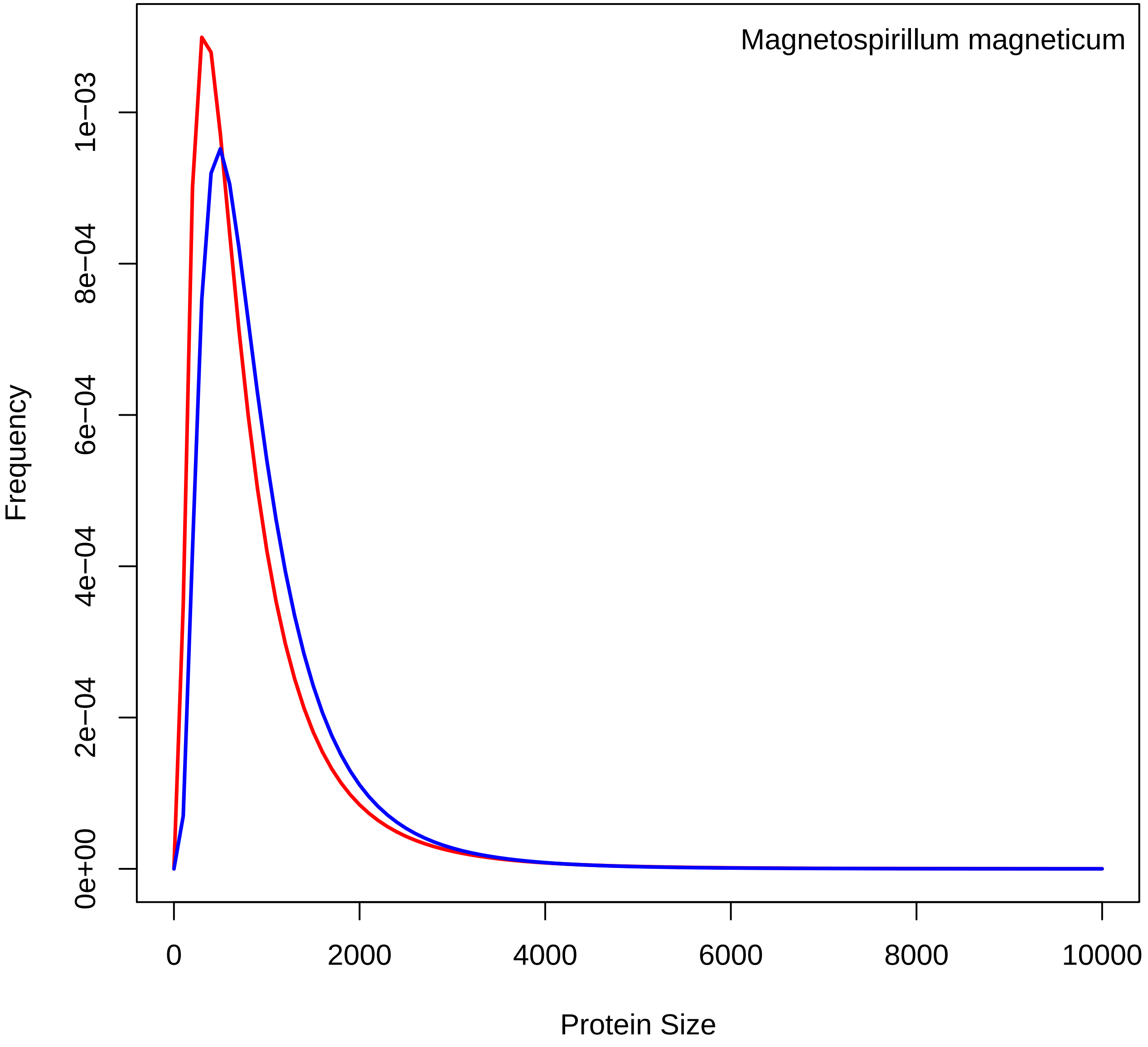

Supplement 3 − Figure 216

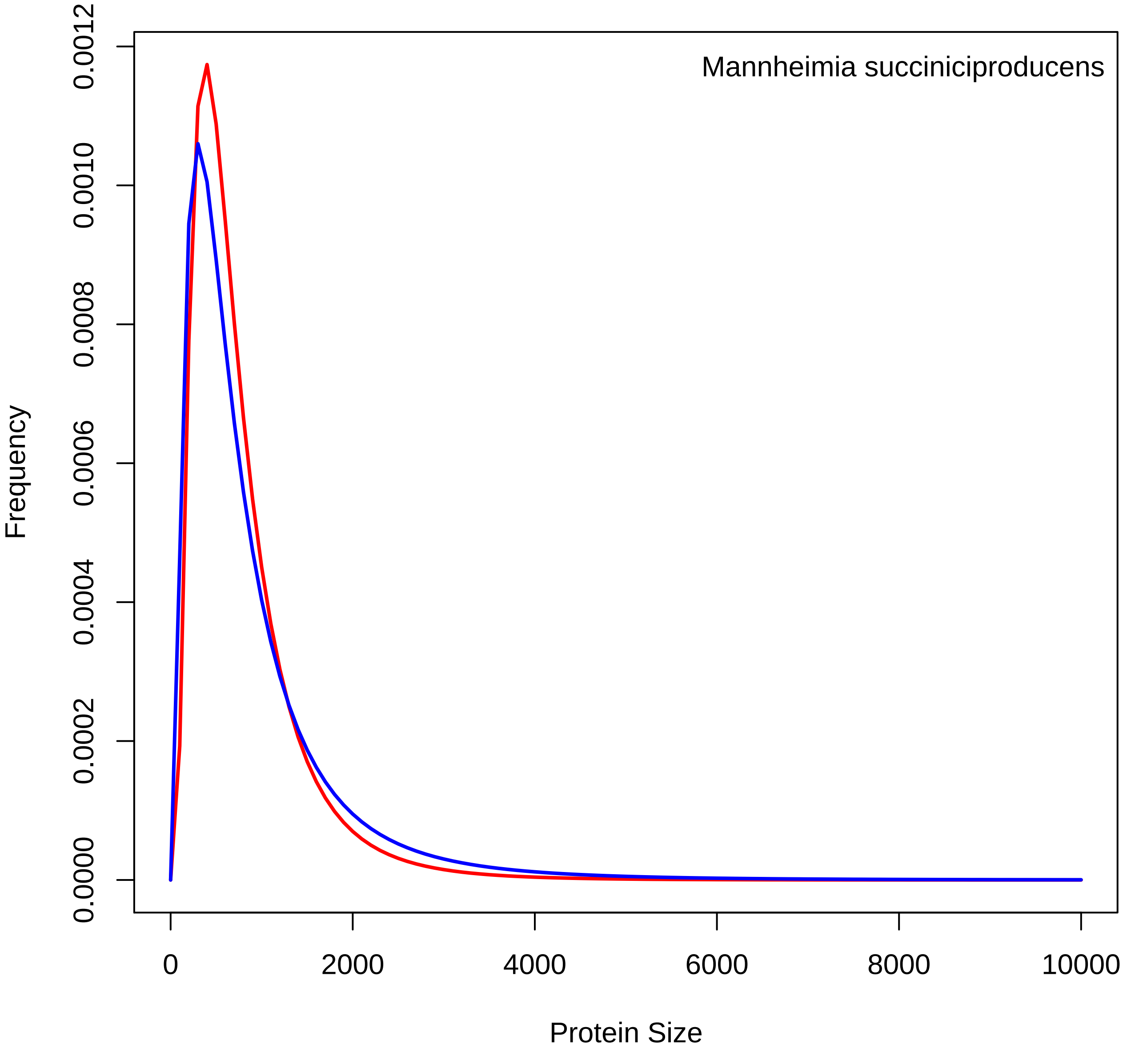

Supplement 3 − Figure 217

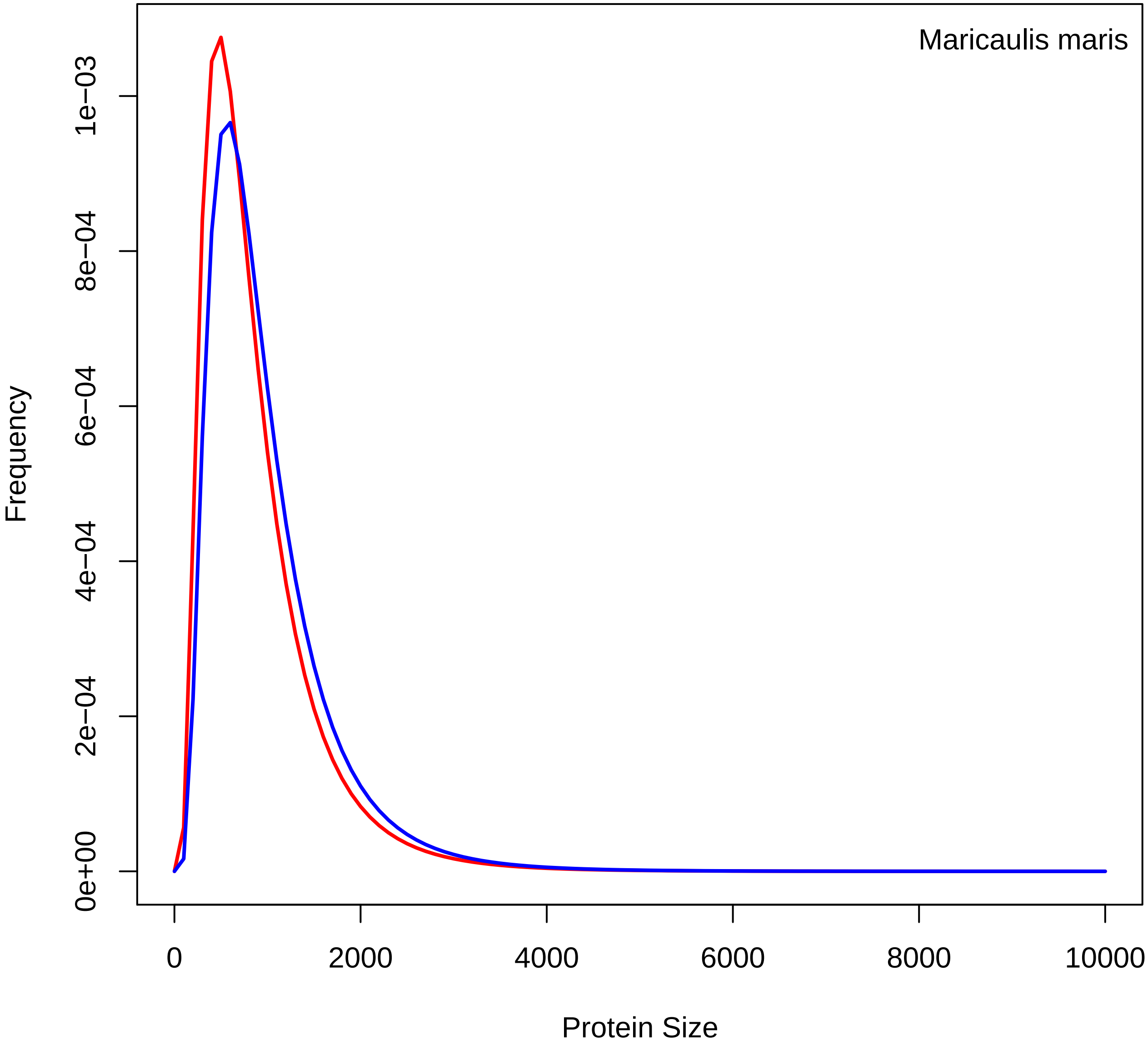

Supplement 3 − Figure 218

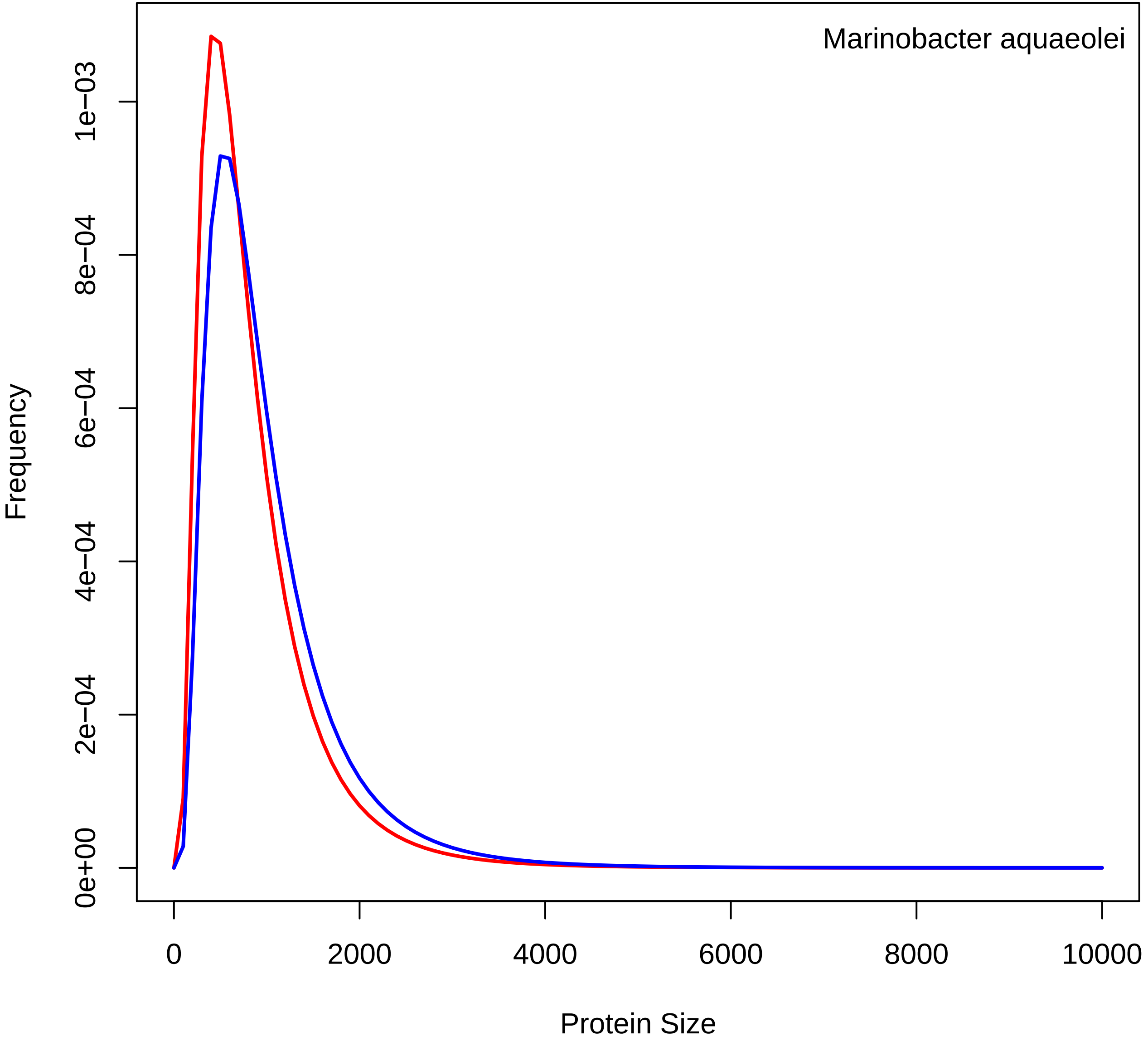

Supplement 3 − Figure 219

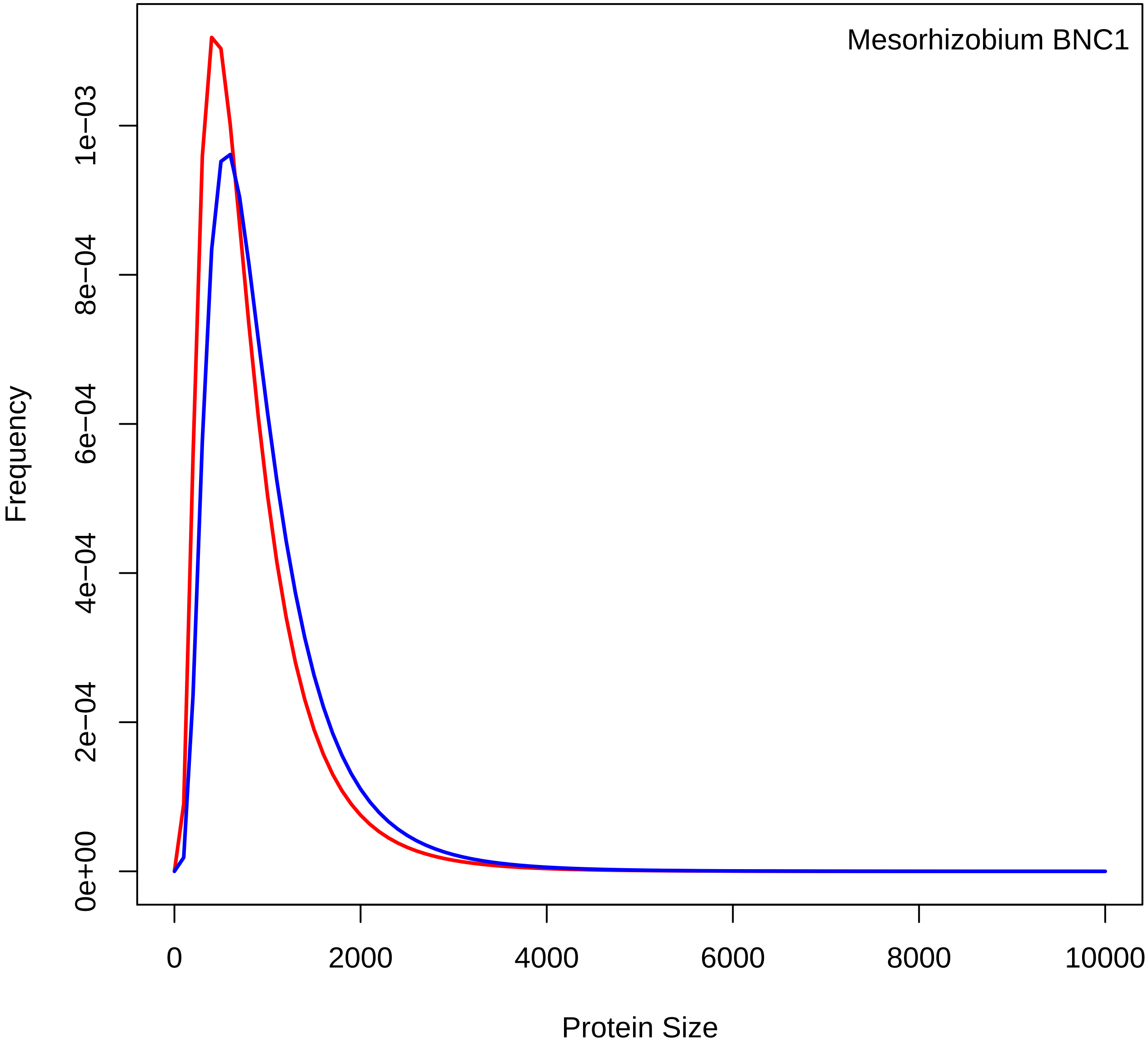

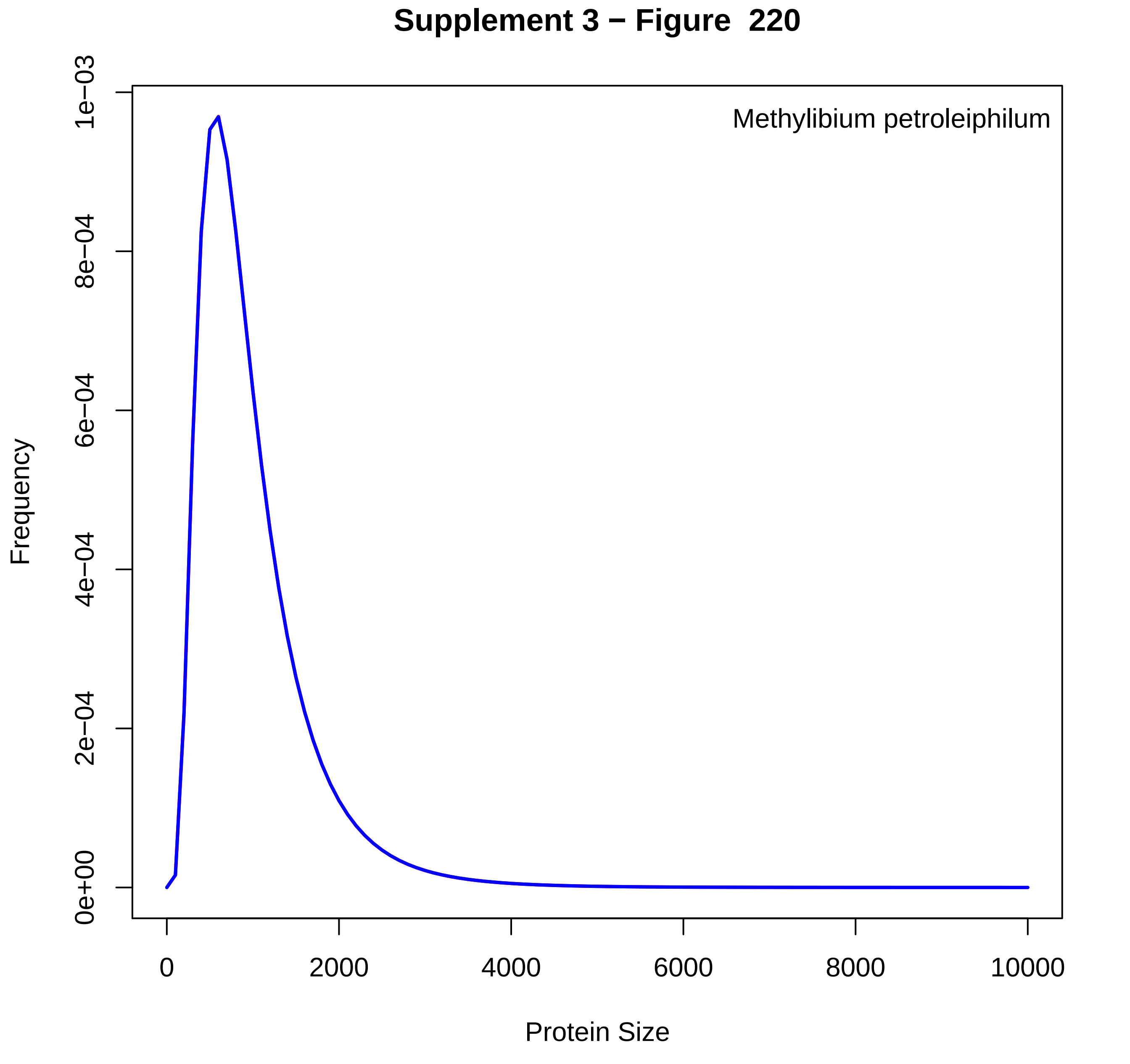
Supplement 3 − Figure 220
Methylibium petroleiphilum
Frequency
0e+00
2e−04
4e−04
6e−04
8e−04
1e−03
0
2000
4000
6000
8000
10000
Protein Size

Supplement 3 − Figure 221

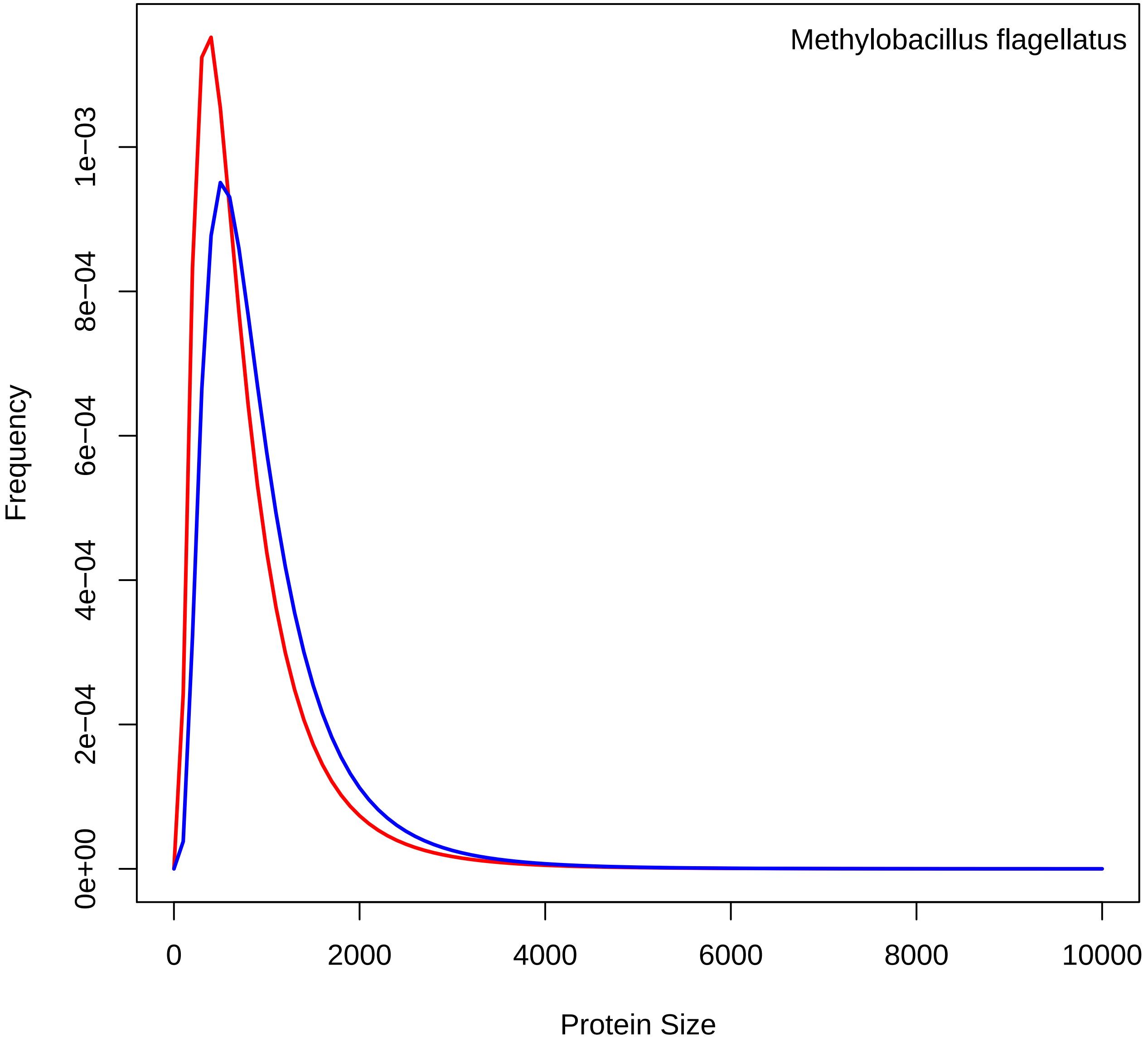

Supplement 3 − Figure 222

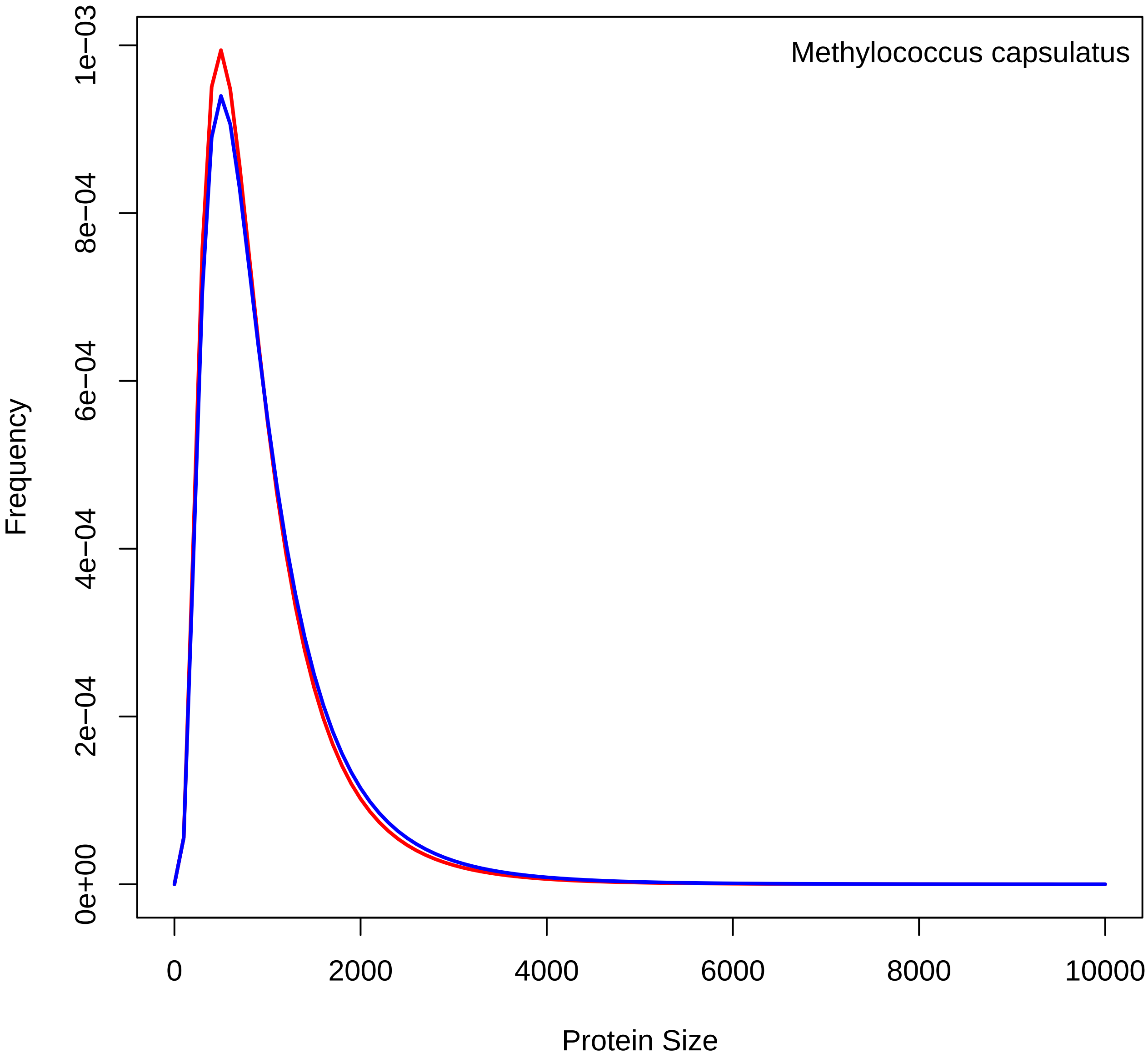

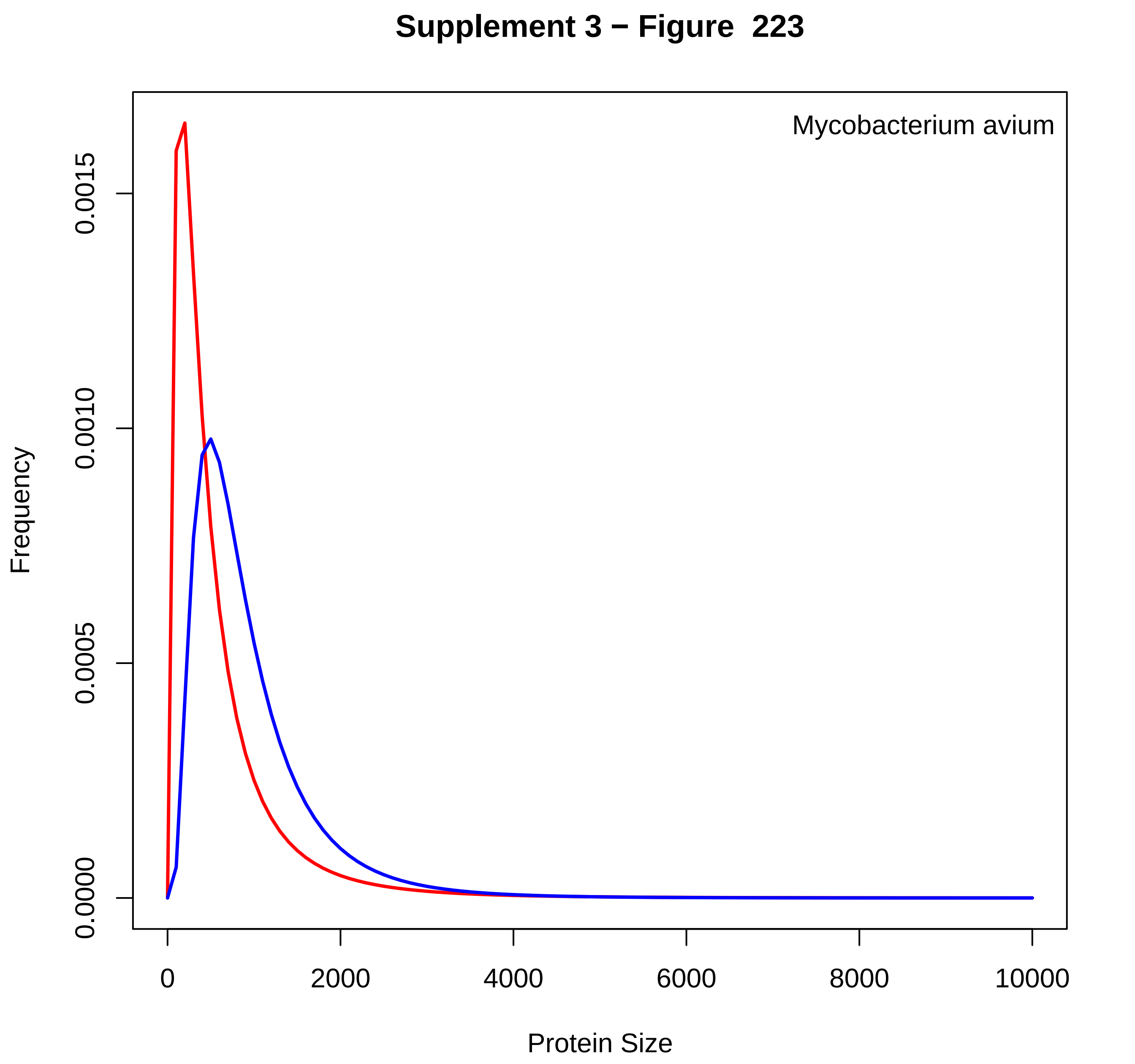
Supplement 3 − Figure 223
Mycobacterium avium
Frequency
0.0000
0.0005
0.0010
0.0015
0
2000
4000
6000
8000
10000
Protein Size

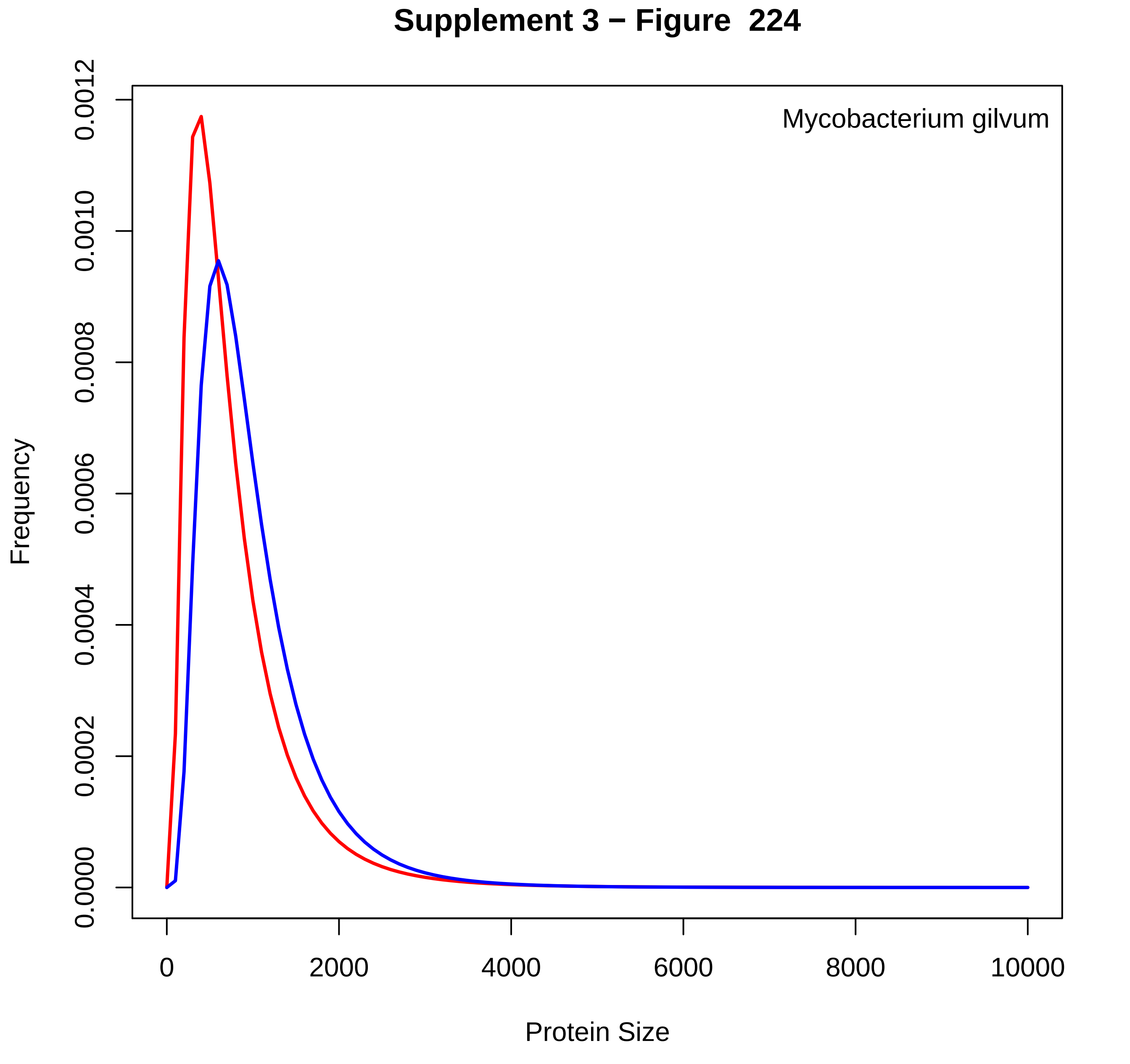

Supplement 3 − Figure 224
Mycobacterium gilvum
Frequency
0.0000
0.0002
0.0004
0.0006
0.0008
0.0010
0.0012
0
2000
4000
6000
8000
10000
Protein Size

Supplement 3 − Figure 225

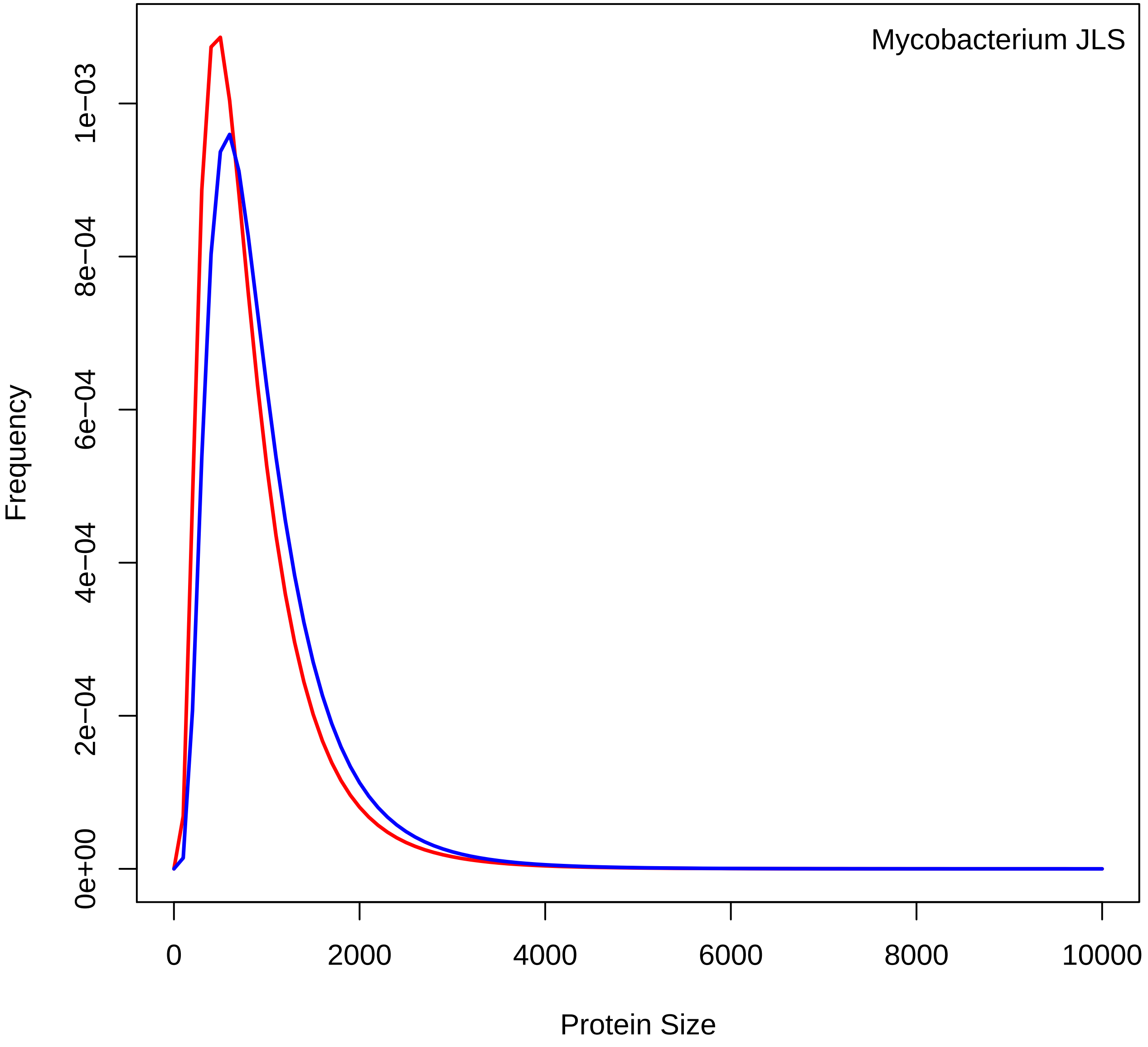

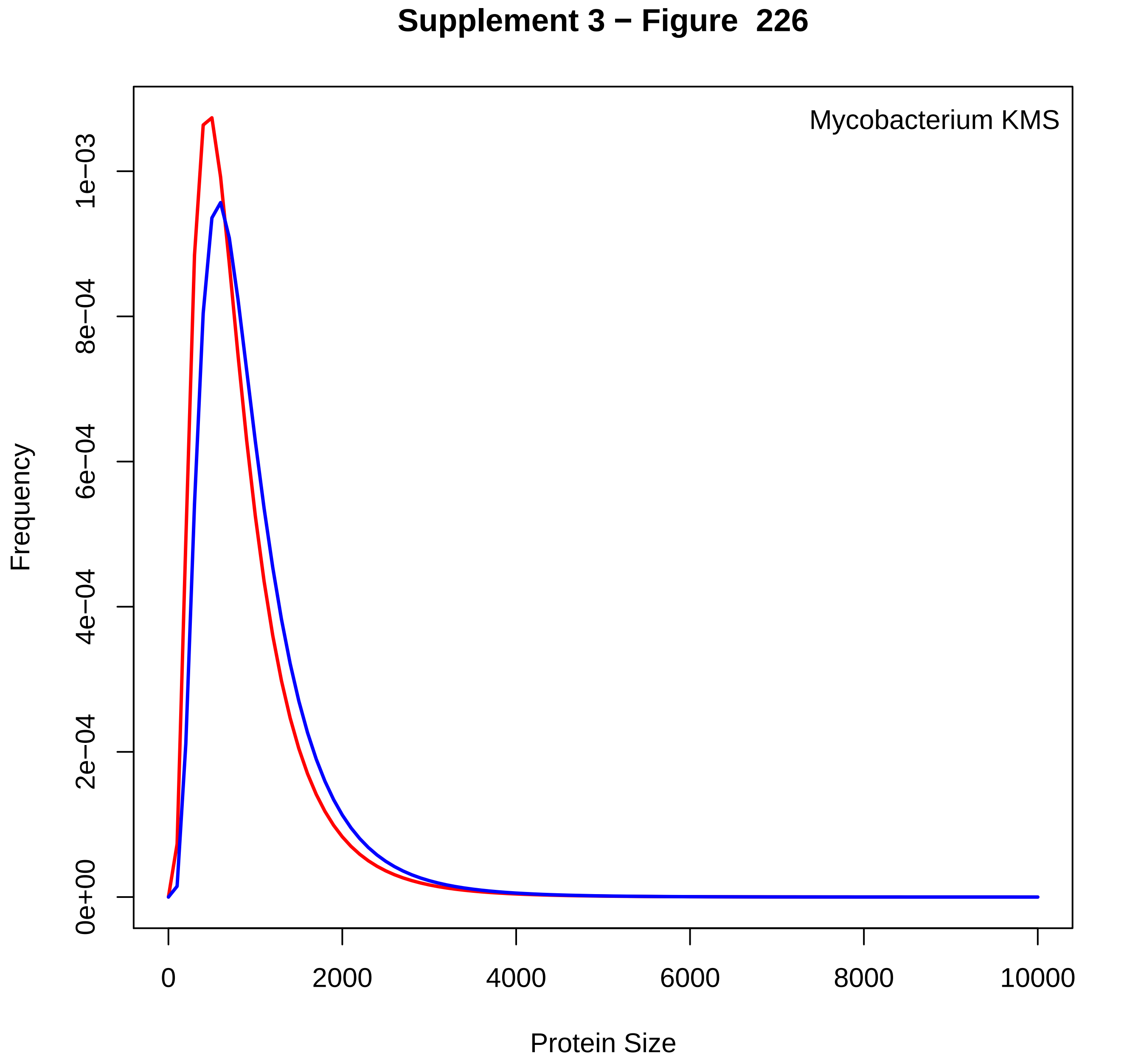

Supplement 3 − Figure 226
Mycobacterium KMS
Frequency
0e+00
2e−04
4e−04
6e−04
8e−04
1e−03
0
2000
4000
6000
8000
10000
Protein Size

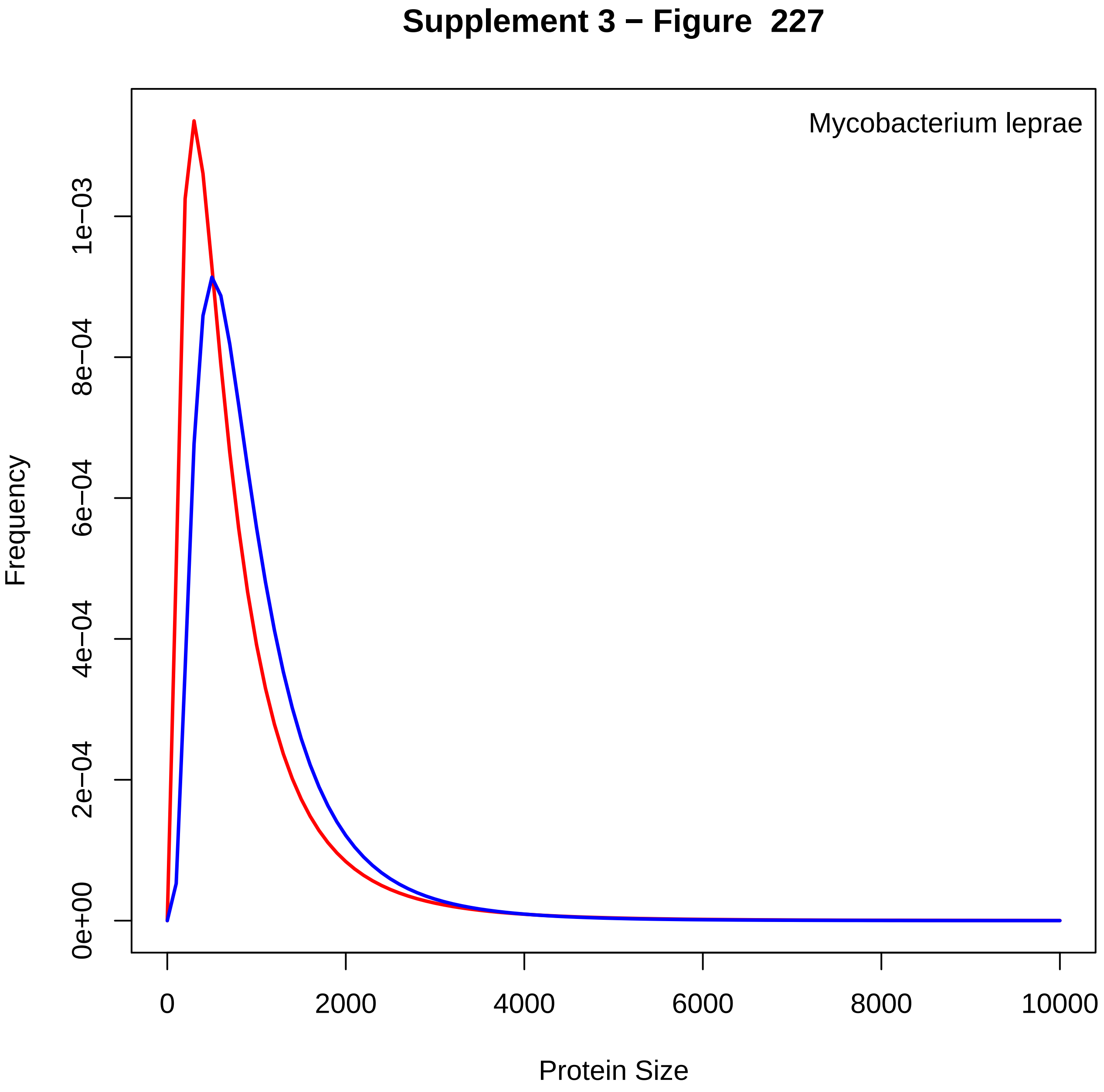
Supplement 3 − Figure 227
Mycobacterium leprae
Frequency
0e+00
2e−04
4e−04
6e−04
8e−04
1e−03
0
2000
4000
6000
8000
10000
Protein Size

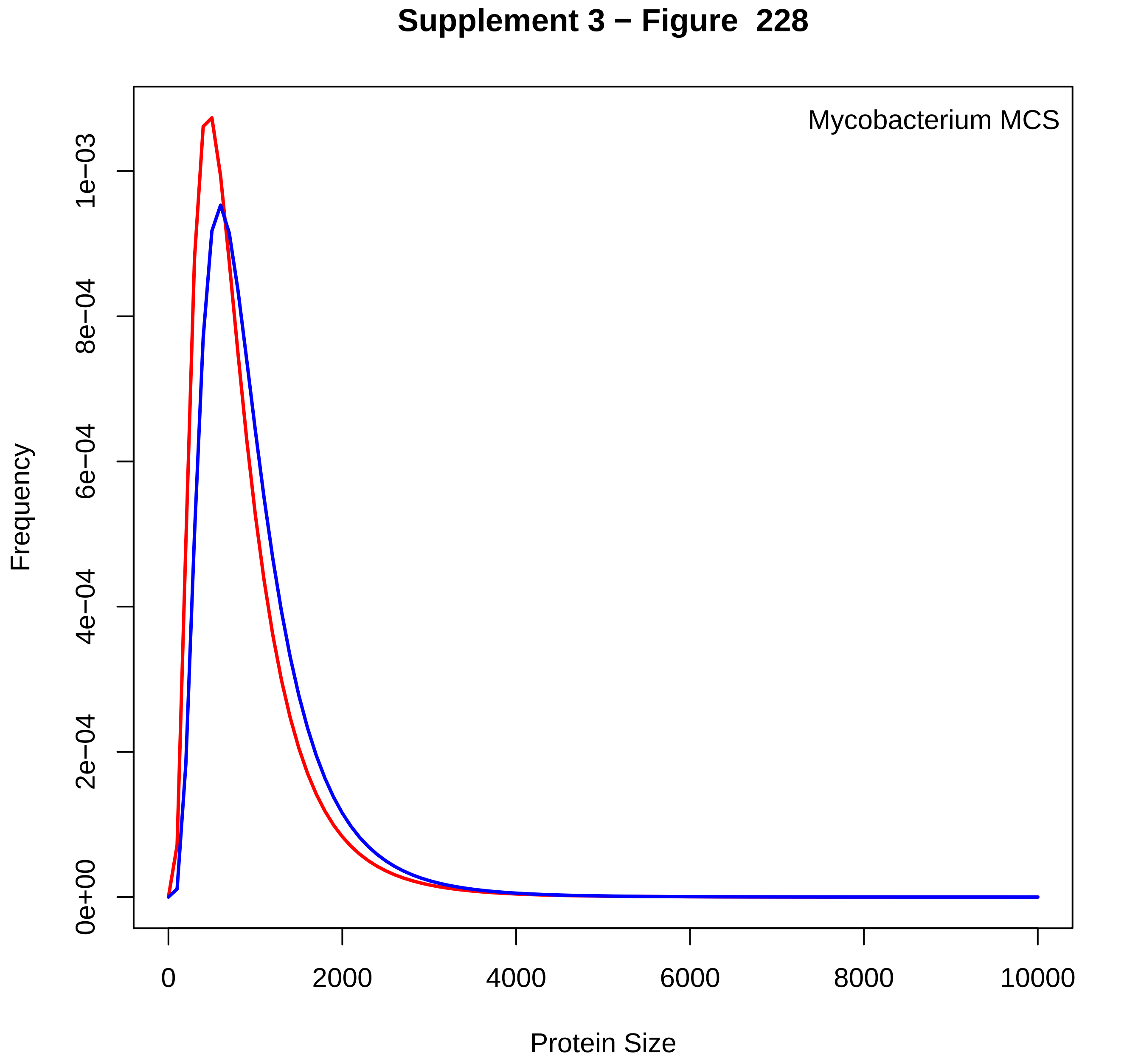
Supplement 3 − Figure 228
Mycobacterium MCS
Frequency
0e+00
2e−04
4e−04
6e−04
8e−04
1e−03
0
2000
4000
6000
8000
10000
Protein Size

Supplement 3 − Figure 229

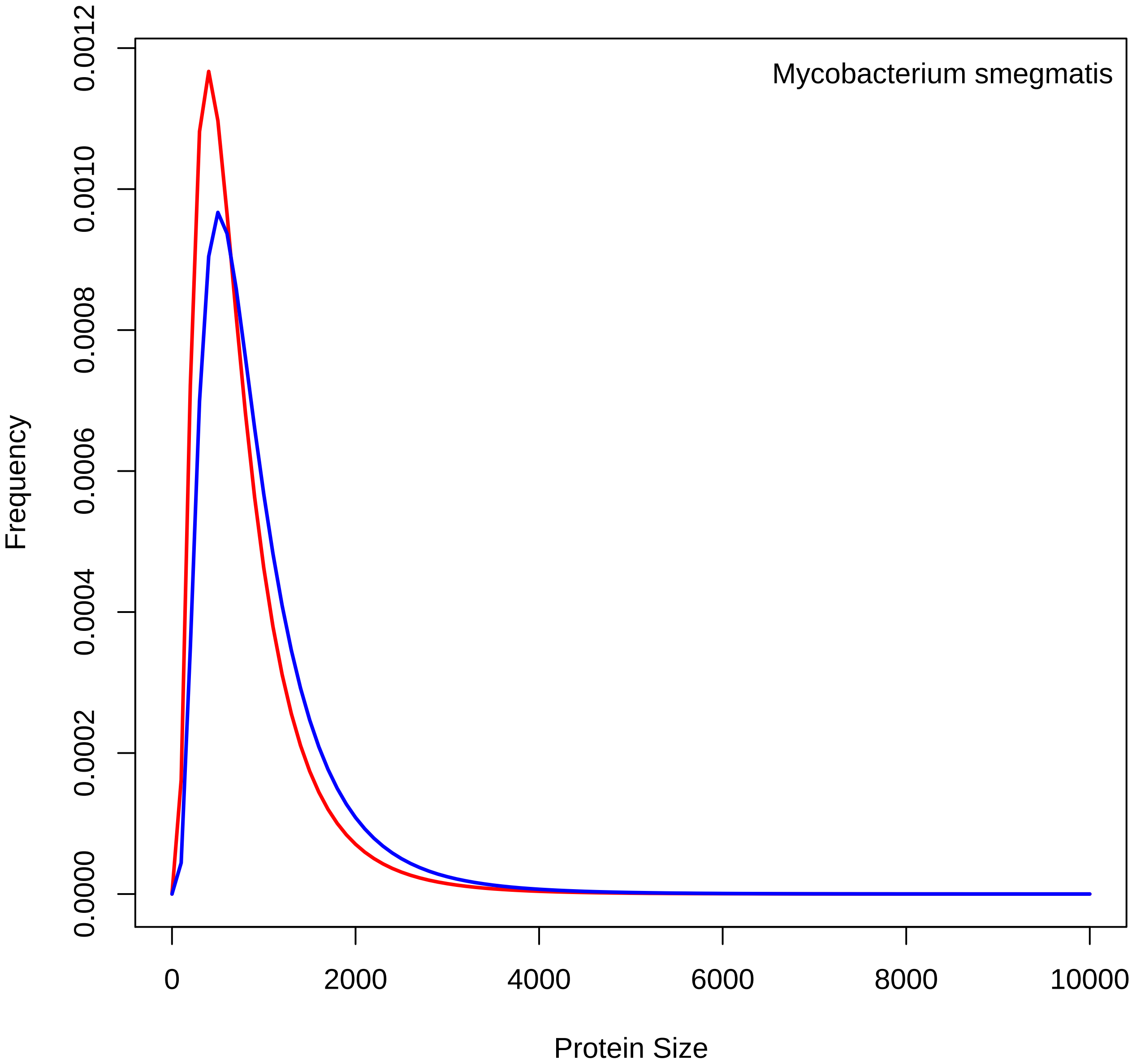

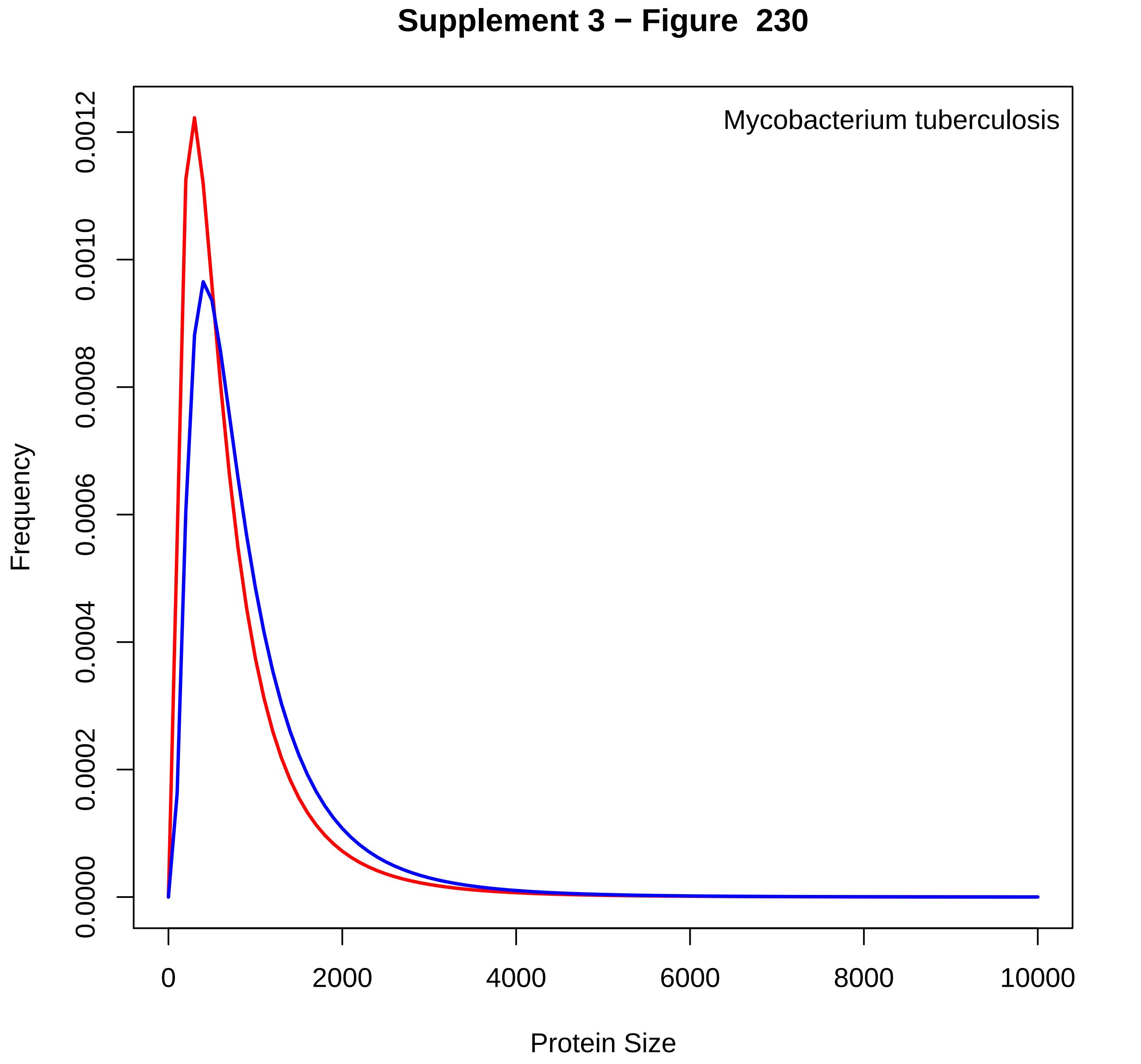
Supplement 3 − Figure 230
Mycobacterium tuberculosis
Frequency
0.0000
0.0002
0.0004
0.0006
0.0008
0.0010
0.0012
0
2000
4000
6000
8000
10000
Protein Size

Supplement 3 − Figure 231

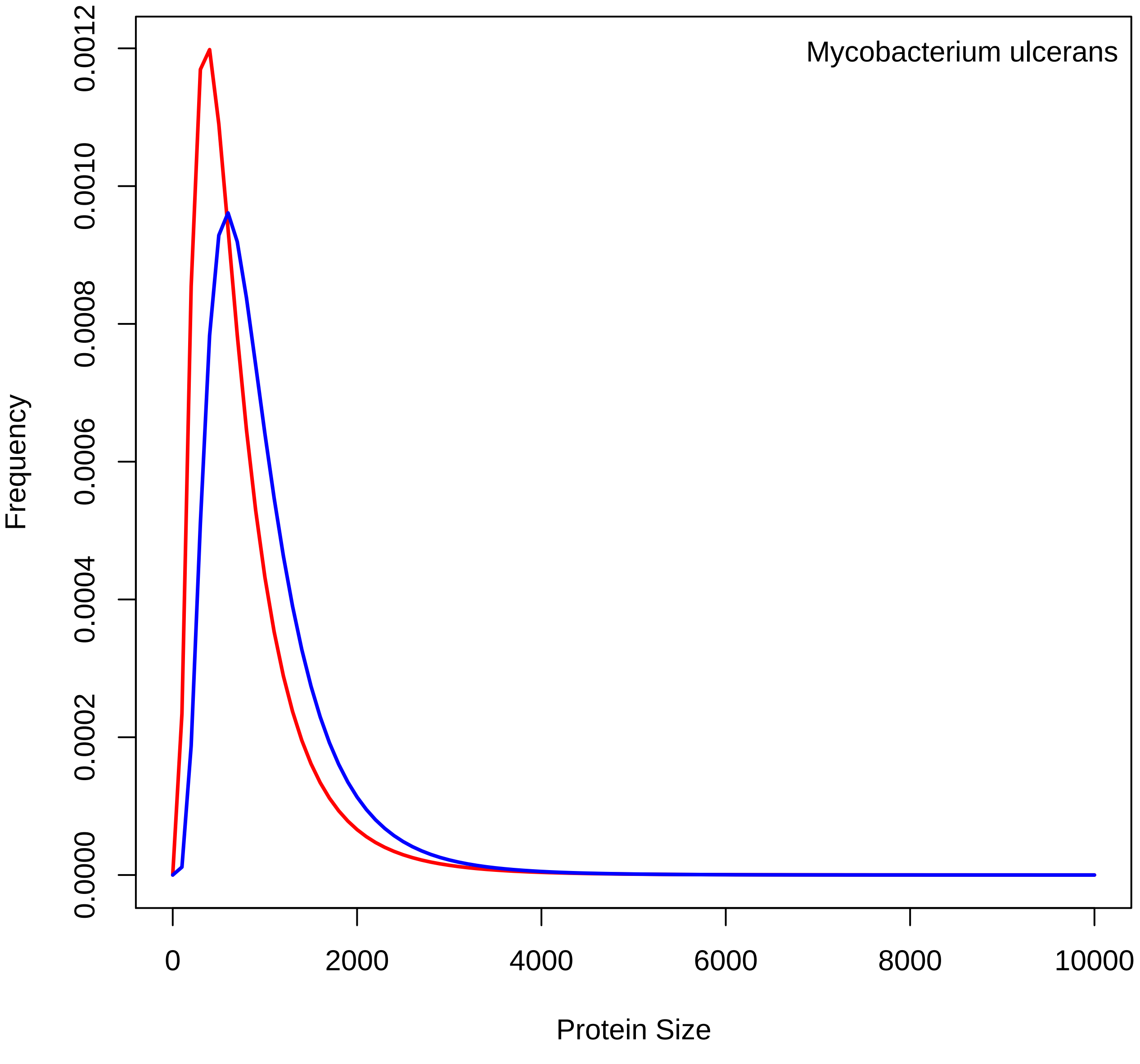

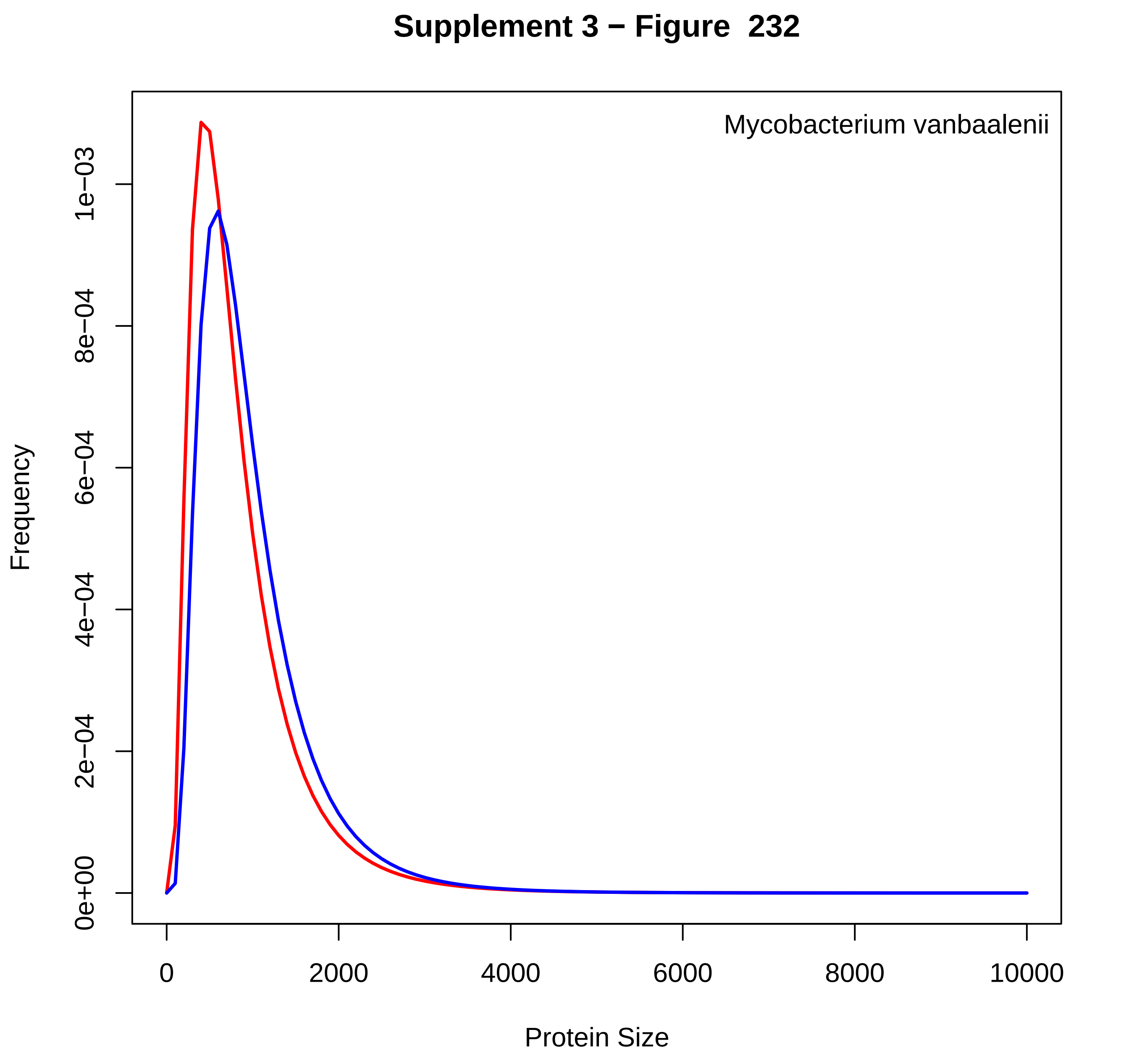

Supplement 3 − Figure 232
Mycobacterium vanbaalenii
Frequency
0e+00
2e−04
4e−04
6e−04
8e−04
1e−03
0
2000
4000
6000
8000
10000
Protein Size

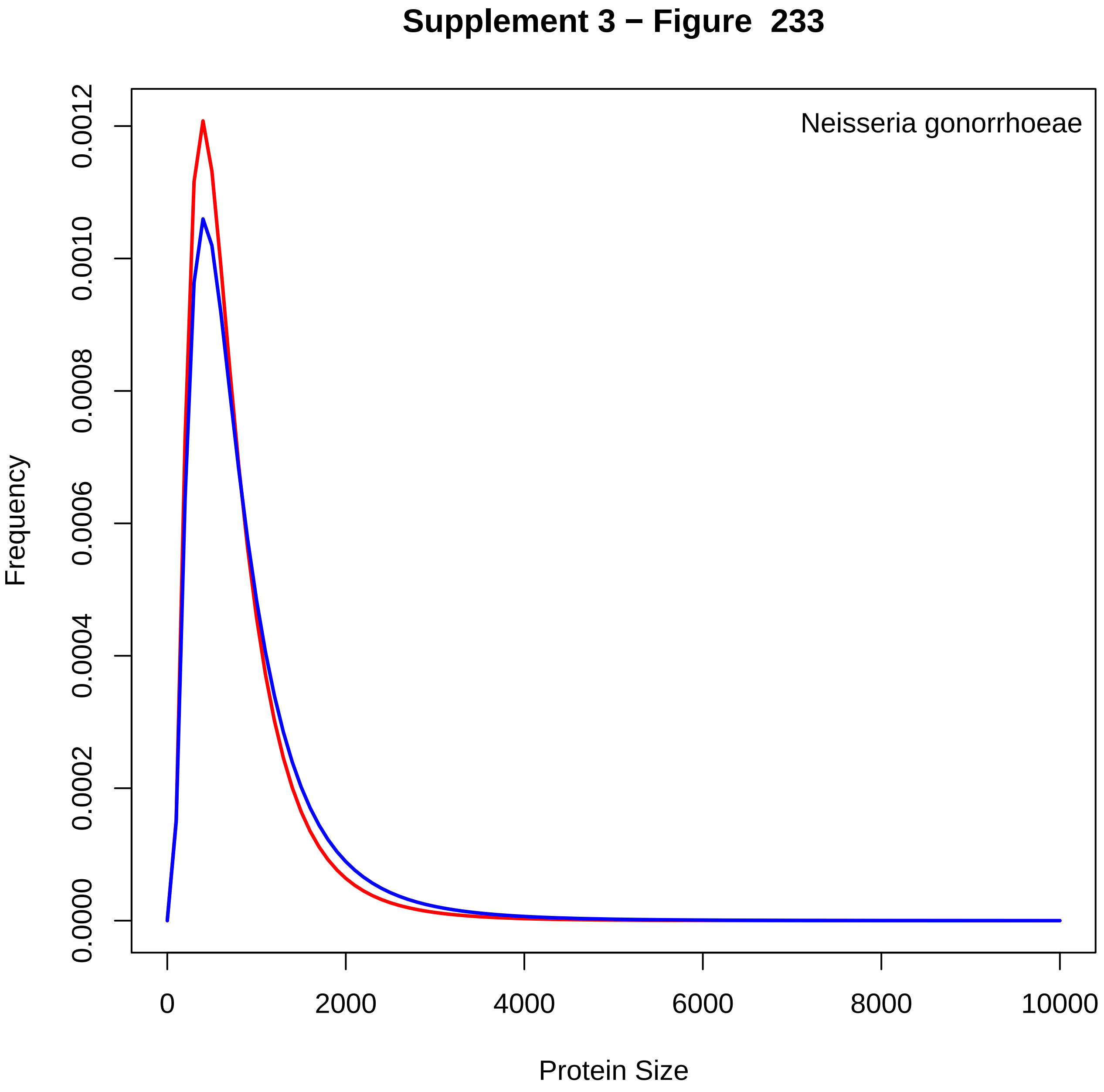
Supplement 3 − Figure 233
Neisseria gonorrhoeae
Frequency
0.0000
0.0002
0.0004
0.0006
0.0008
0.0010
0.0012
0
2000
4000
6000
8000
10000
Protein Size

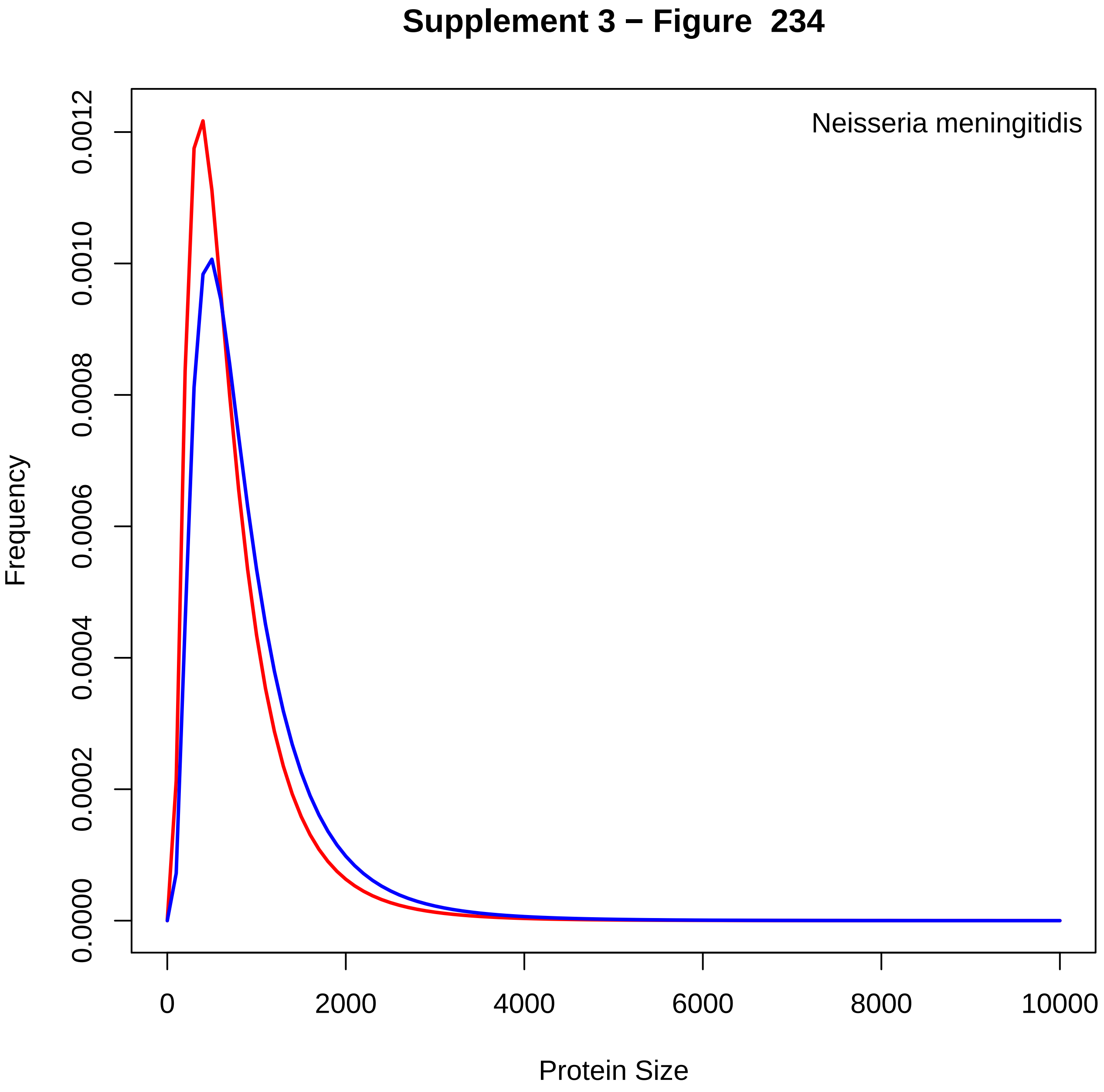

Supplement 3 − Figure 234
Neisseria meningitidis
Frequency
0.0000
0.0002
0.0004
0.0006
0.0008
0.0010
0.0012
0
2000
4000
6000
8000
10000
Protein Size

Supplement 3 − Figure 235

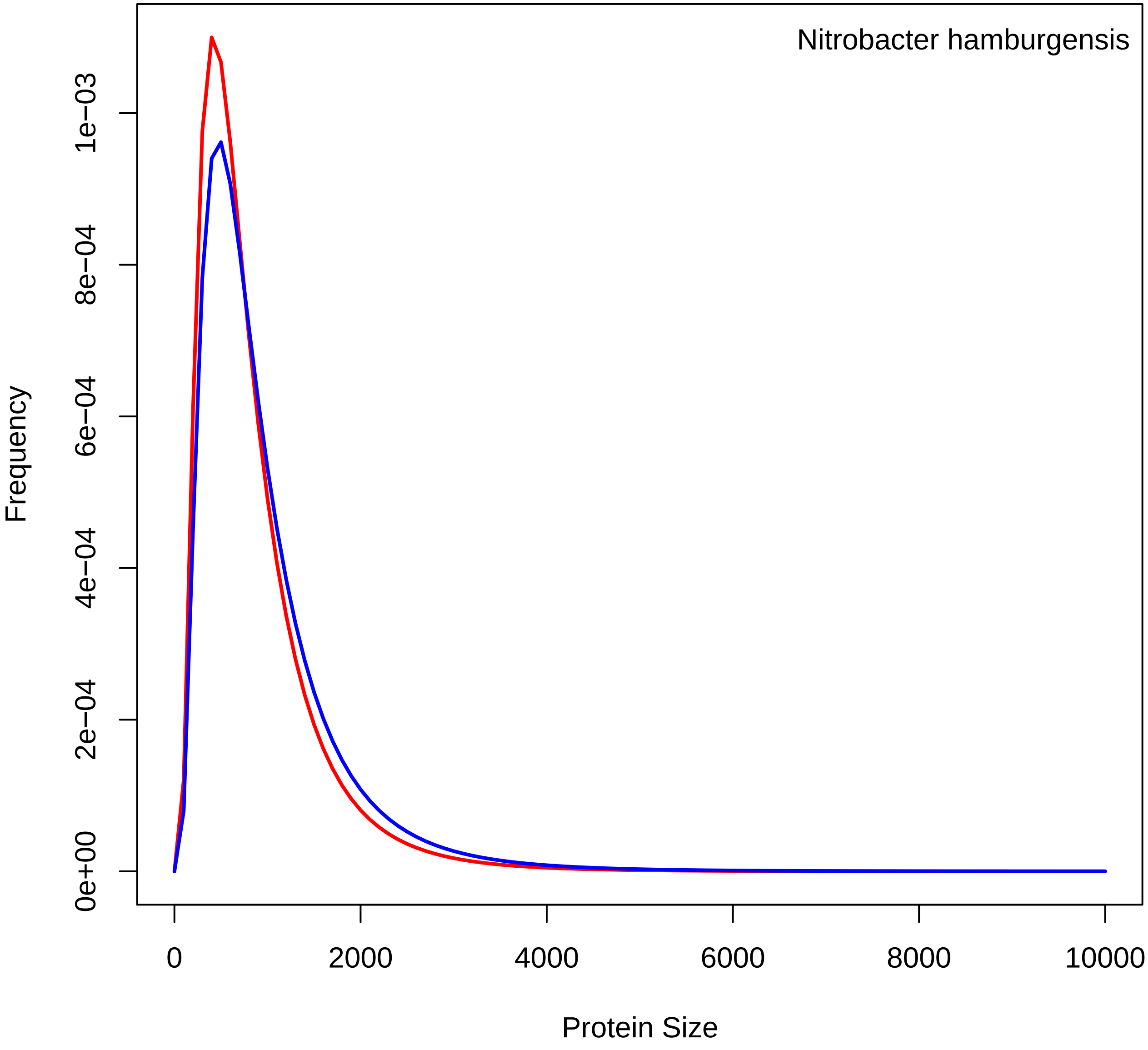

Supplement 3 − Figure 236

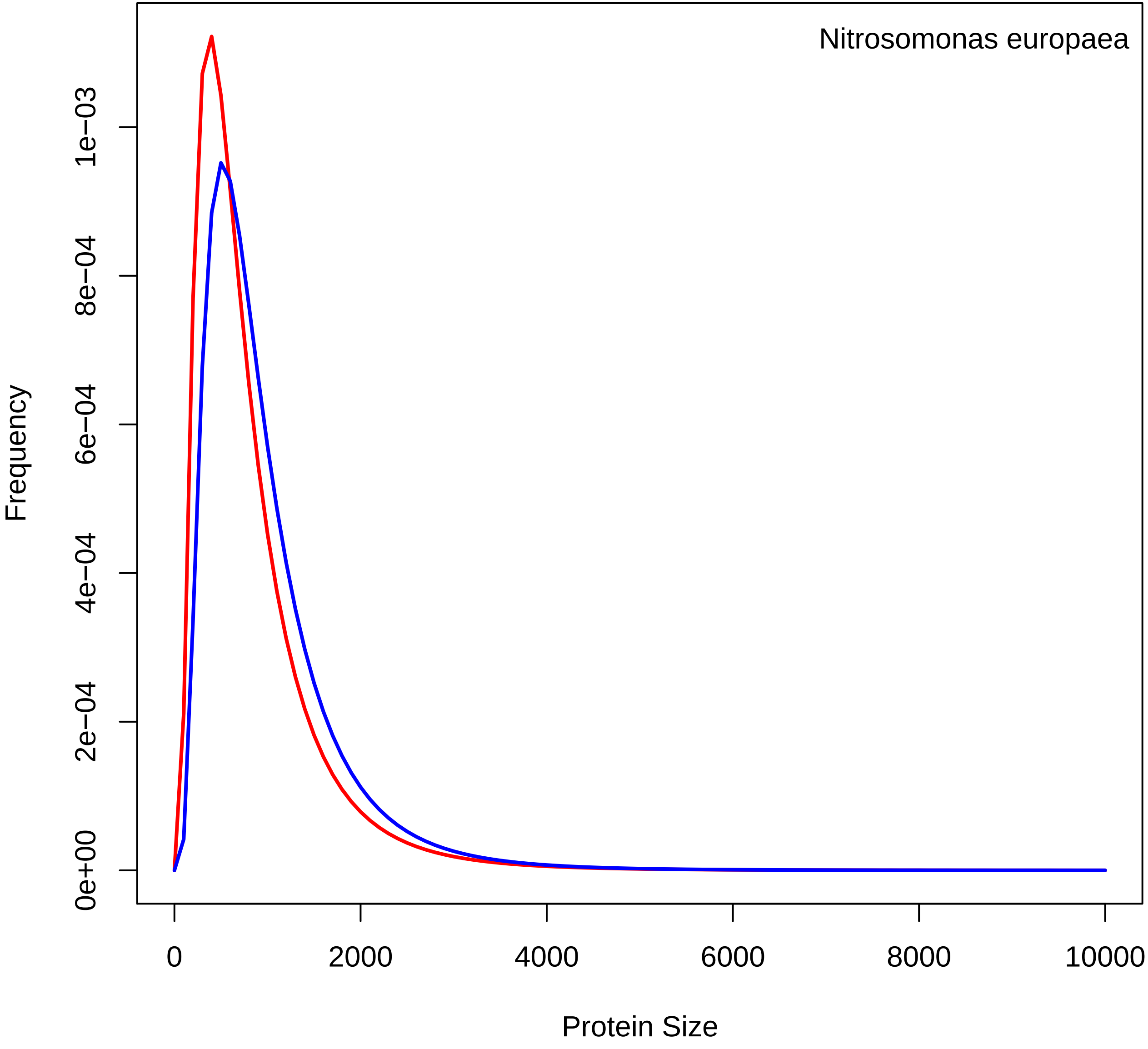

Supplement 3 − Figure 237

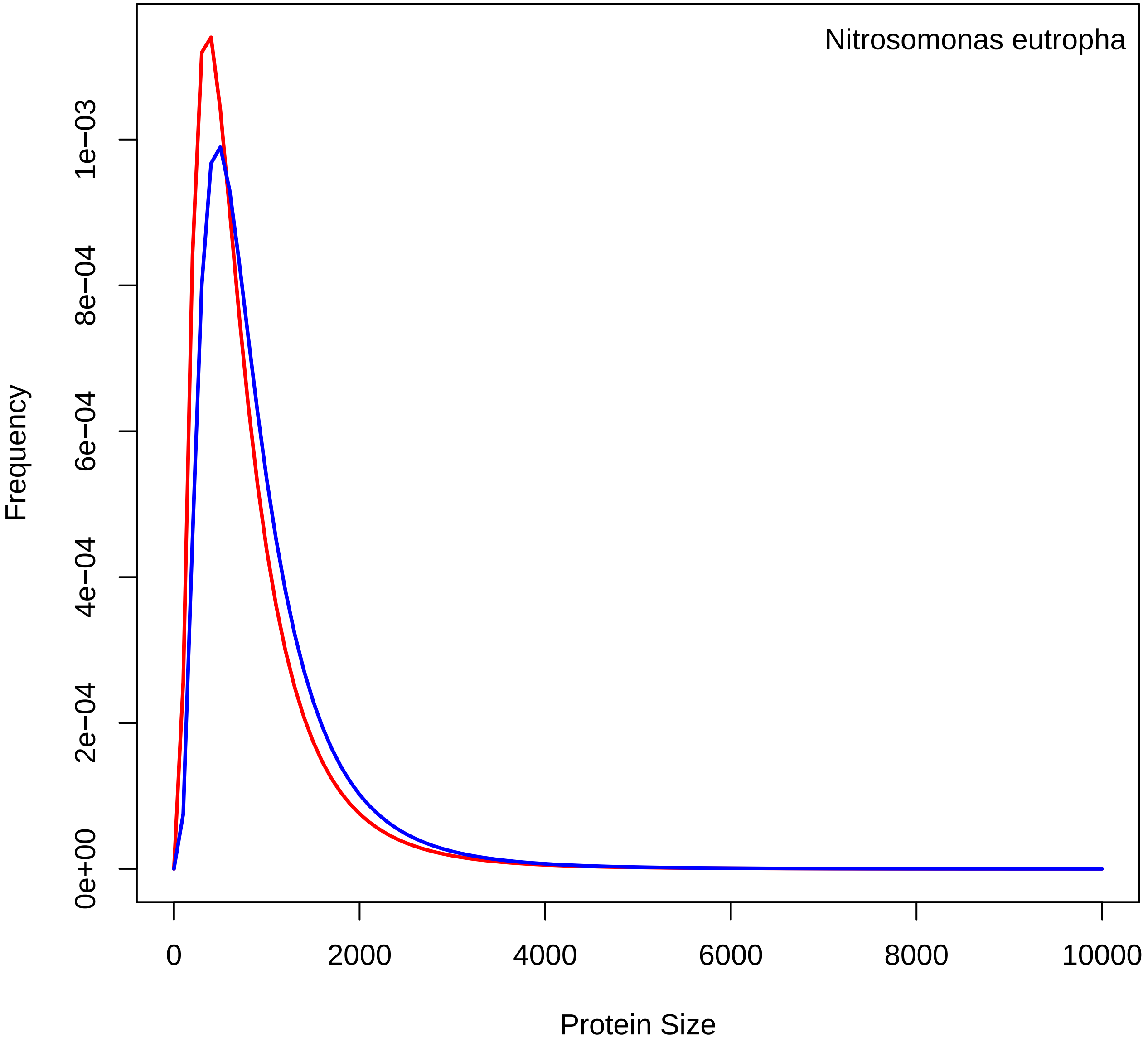

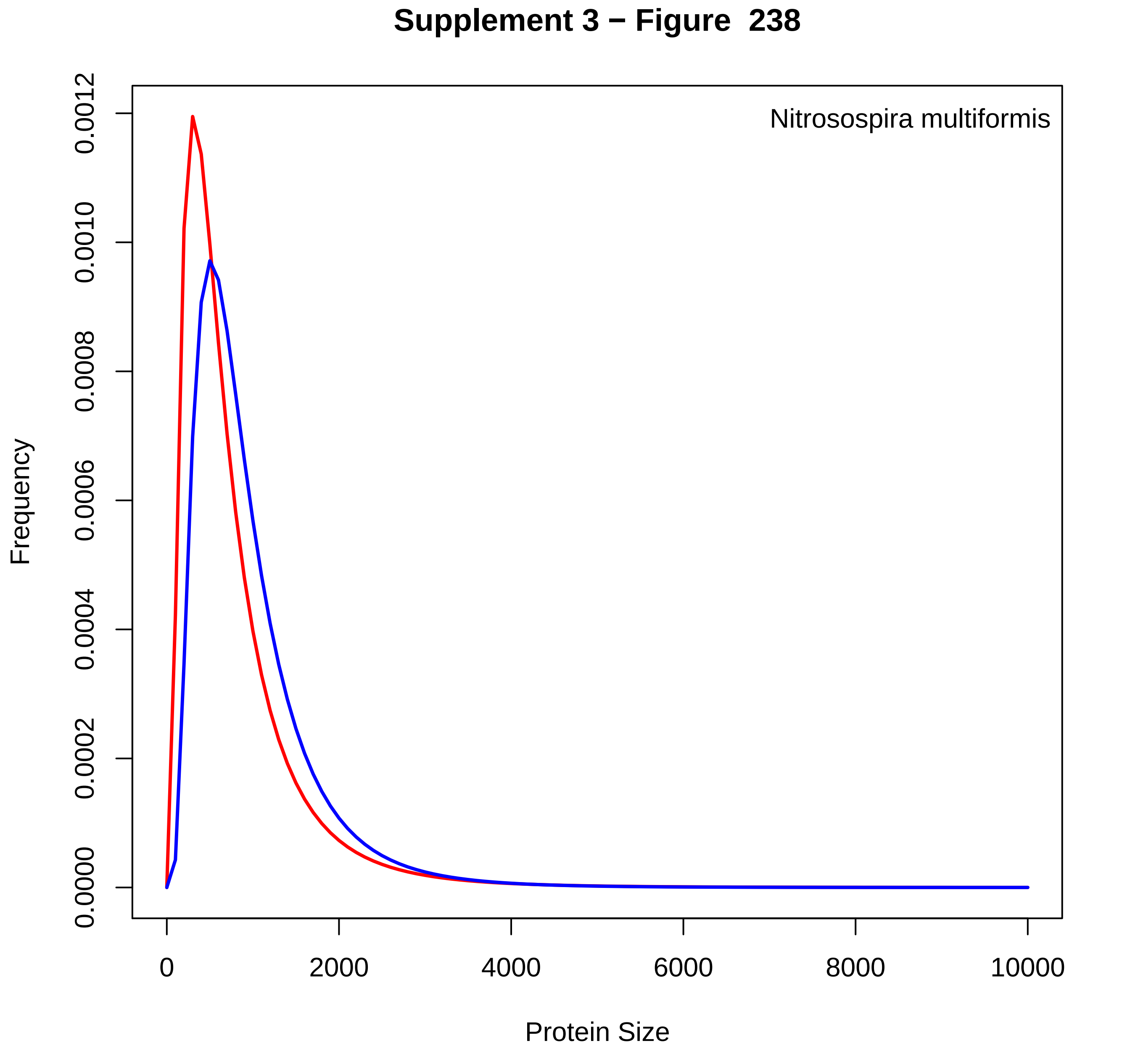
Supplement 3 − Figure 238
Nitrosospira multiformis
Frequency
0.0000
0.0002
0.0004
0.0006
0.0008
0.0010
0.0012
0
2000
4000
6000
8000
10000
Protein Size

Supplement 3 − Figure 239

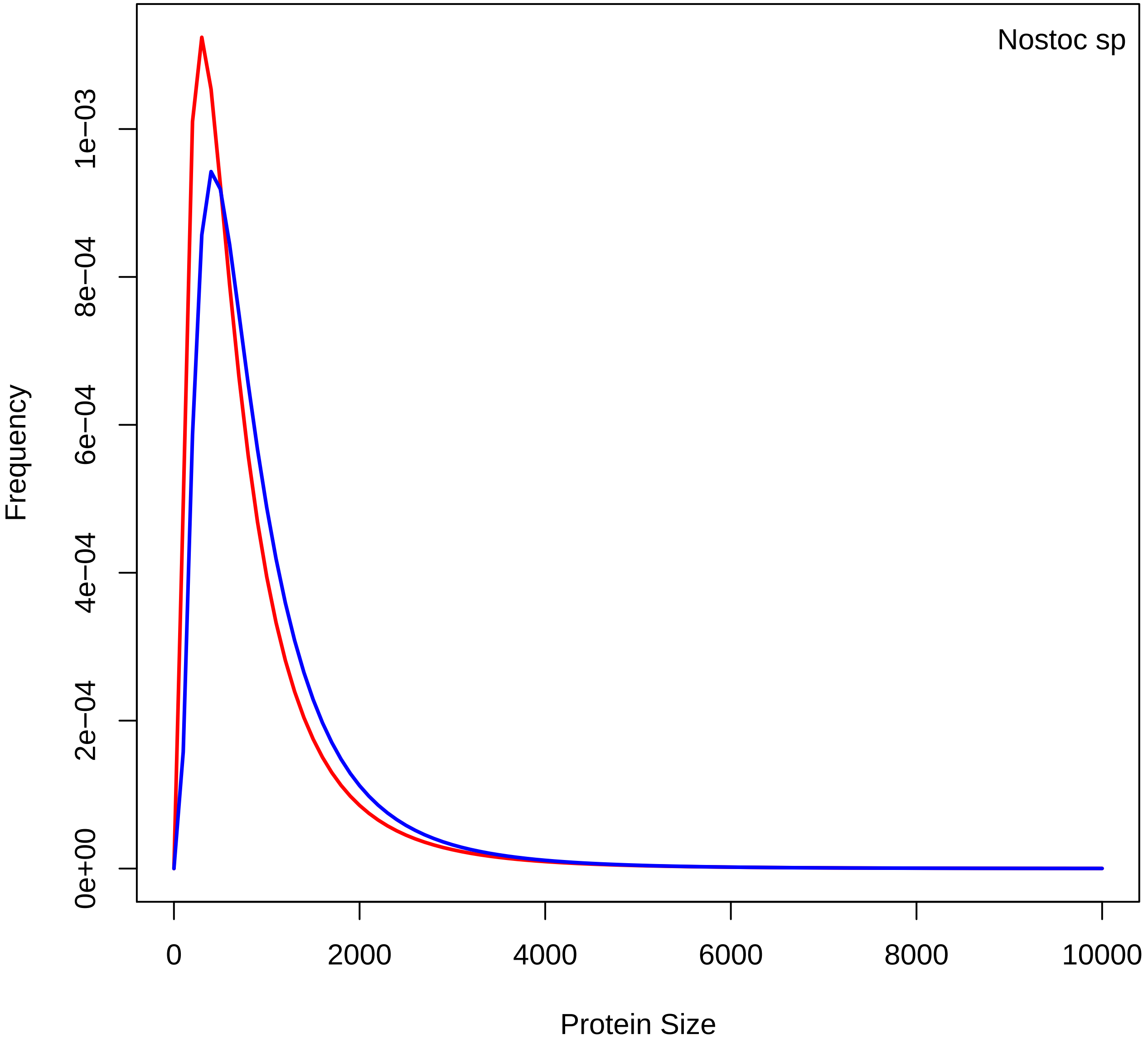

Supplement 3 − Figure 240

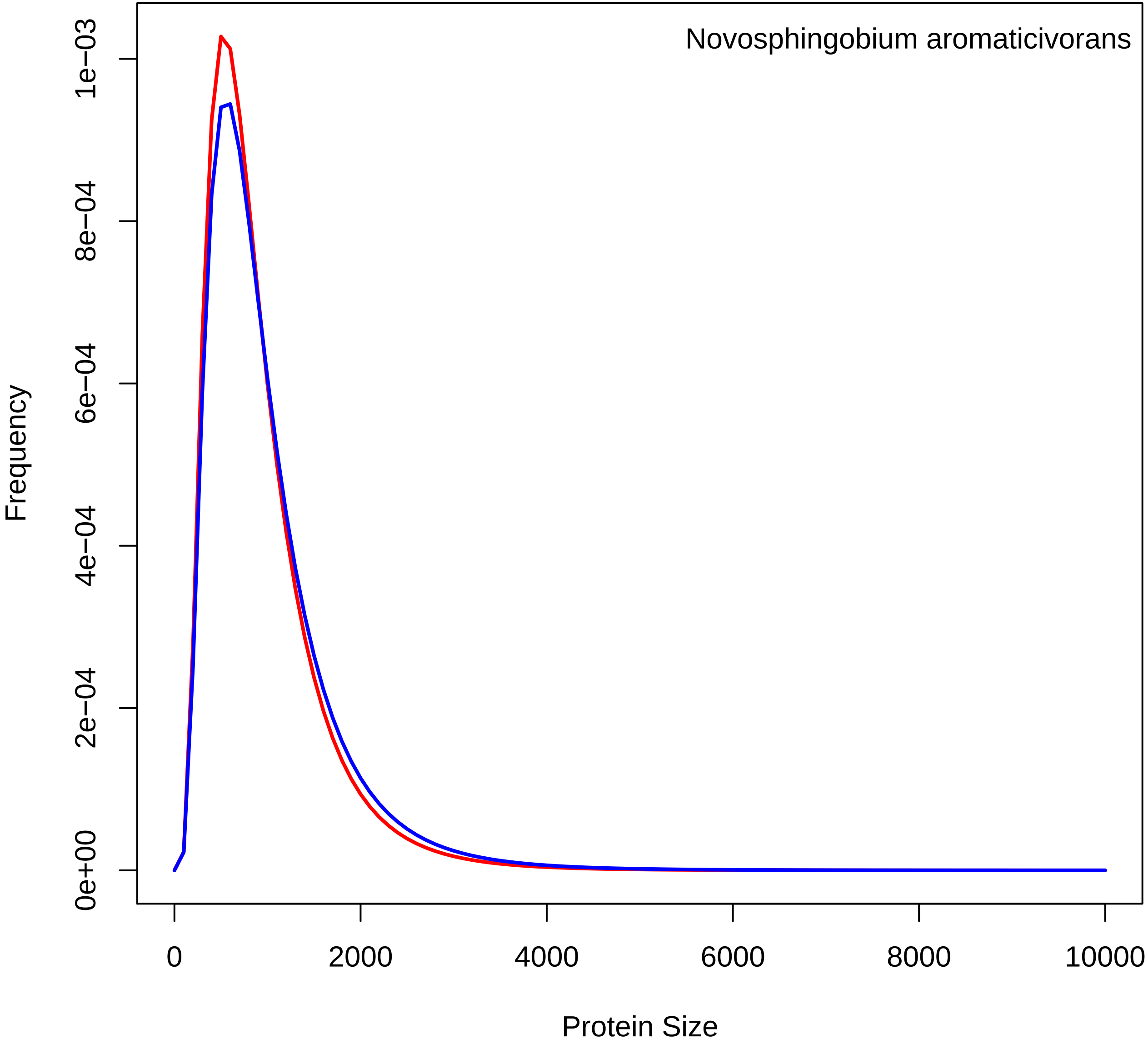

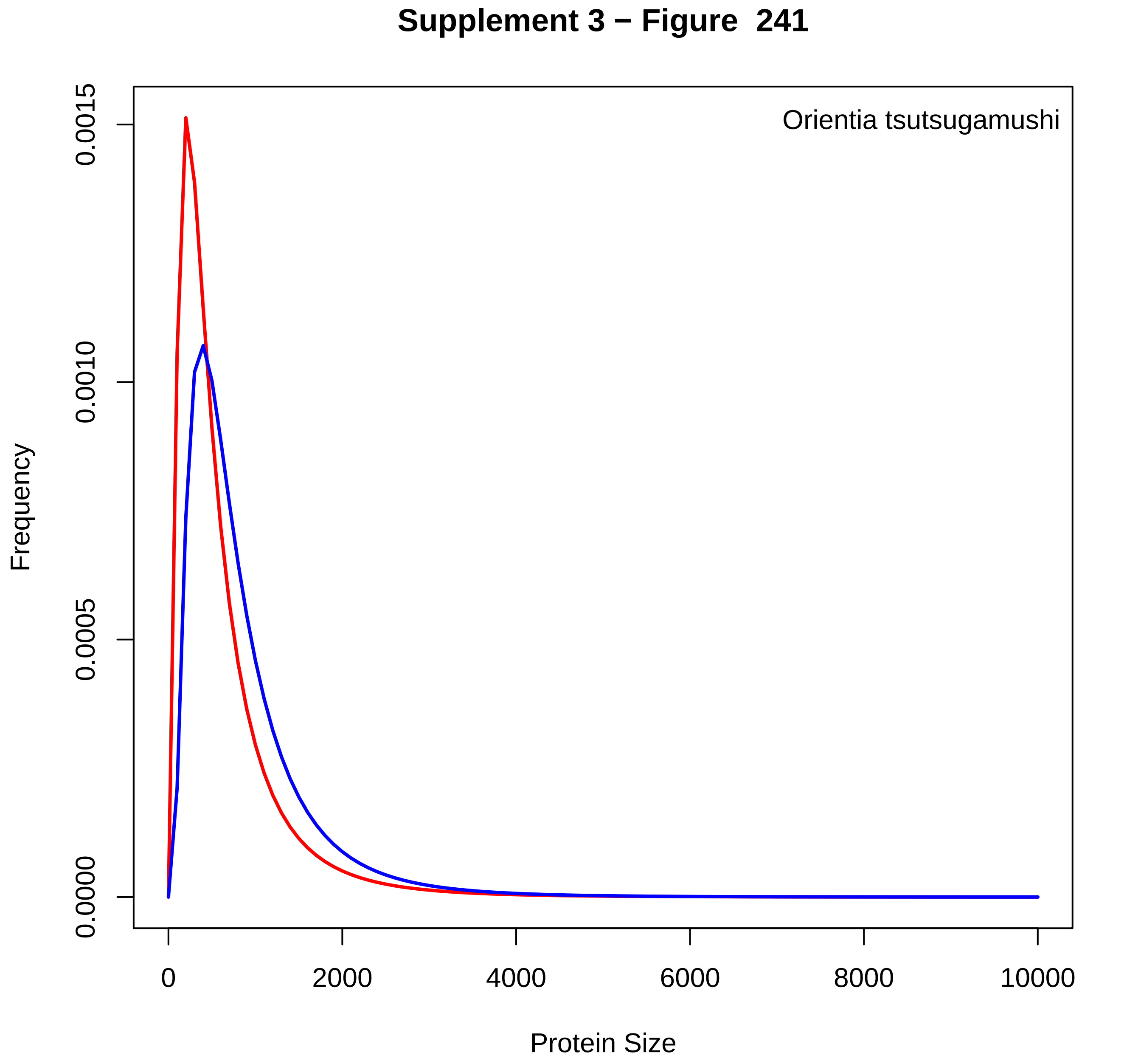
Supplement 3 − Figure 241
Orientia tsutsugamushi
Frequency
0.0000
0.0005
0.0010
0.0015
0
2000
4000
6000
8000
10000
Protein Size

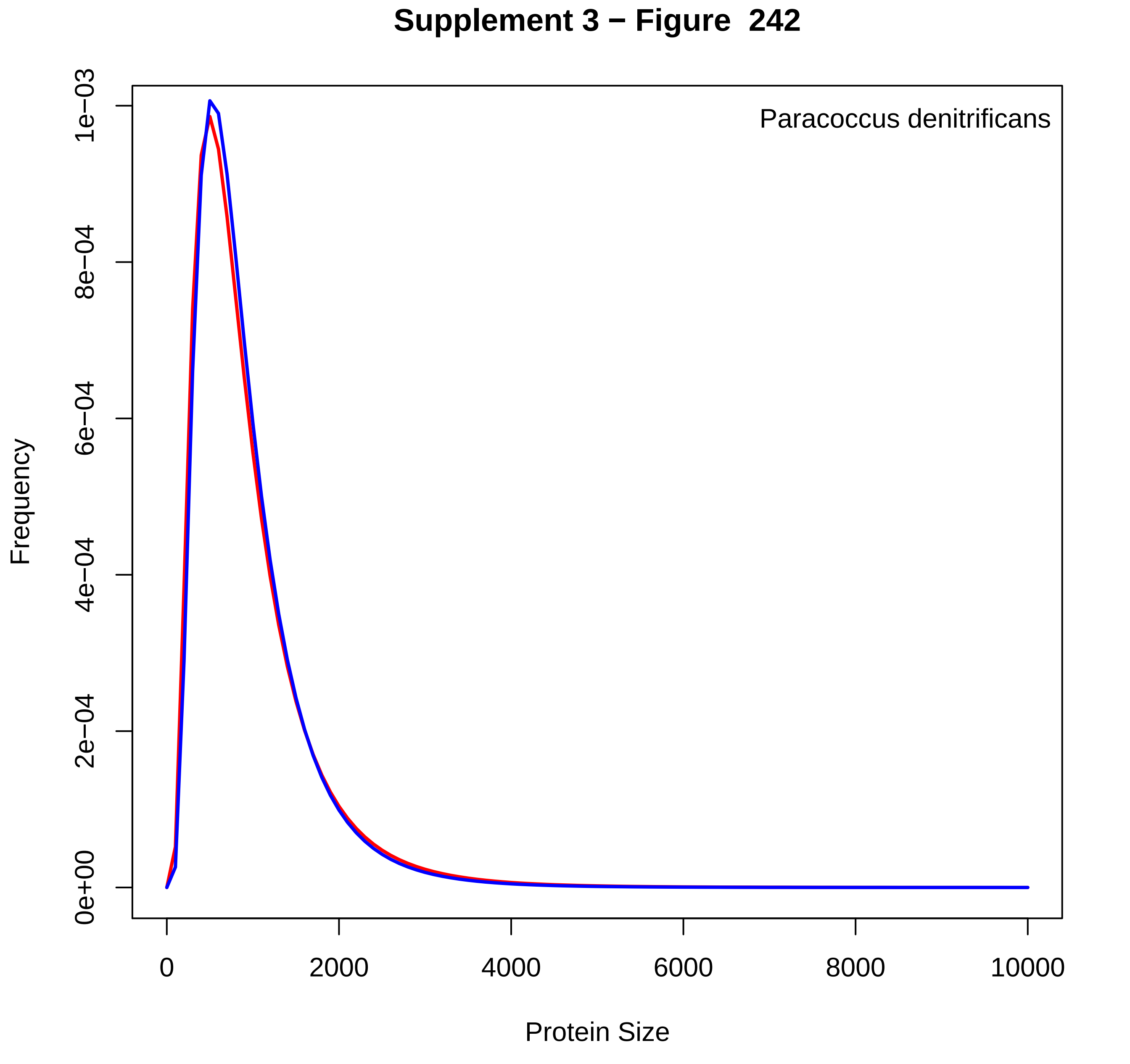
Supplement 3 − Figure 242
Paracoccus denitrificans
Frequency
0e+00
2e−04
4e−04
6e−04
8e−04
1e−03
0
2000
4000
6000
8000
10000
Protein Size

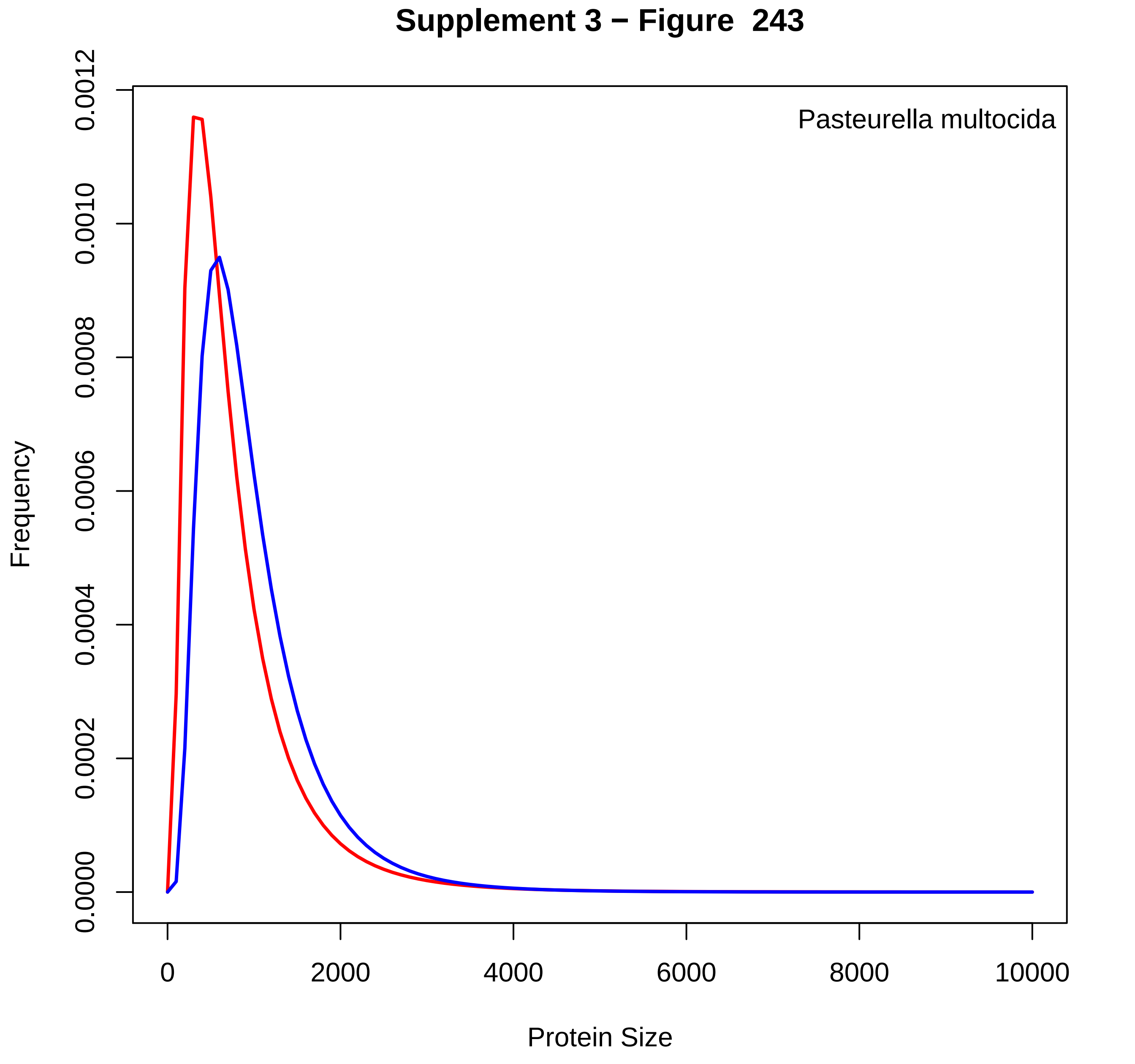
Supplement 3 − Figure 243
Pasteurella multocida
Frequency
Protein Size
0.0000
0.0002
0.0004
0.0006
0.0008
0.0010
0.0012
0
2000
4000
6000
8000
10000

Supplement 3 − Figure 244

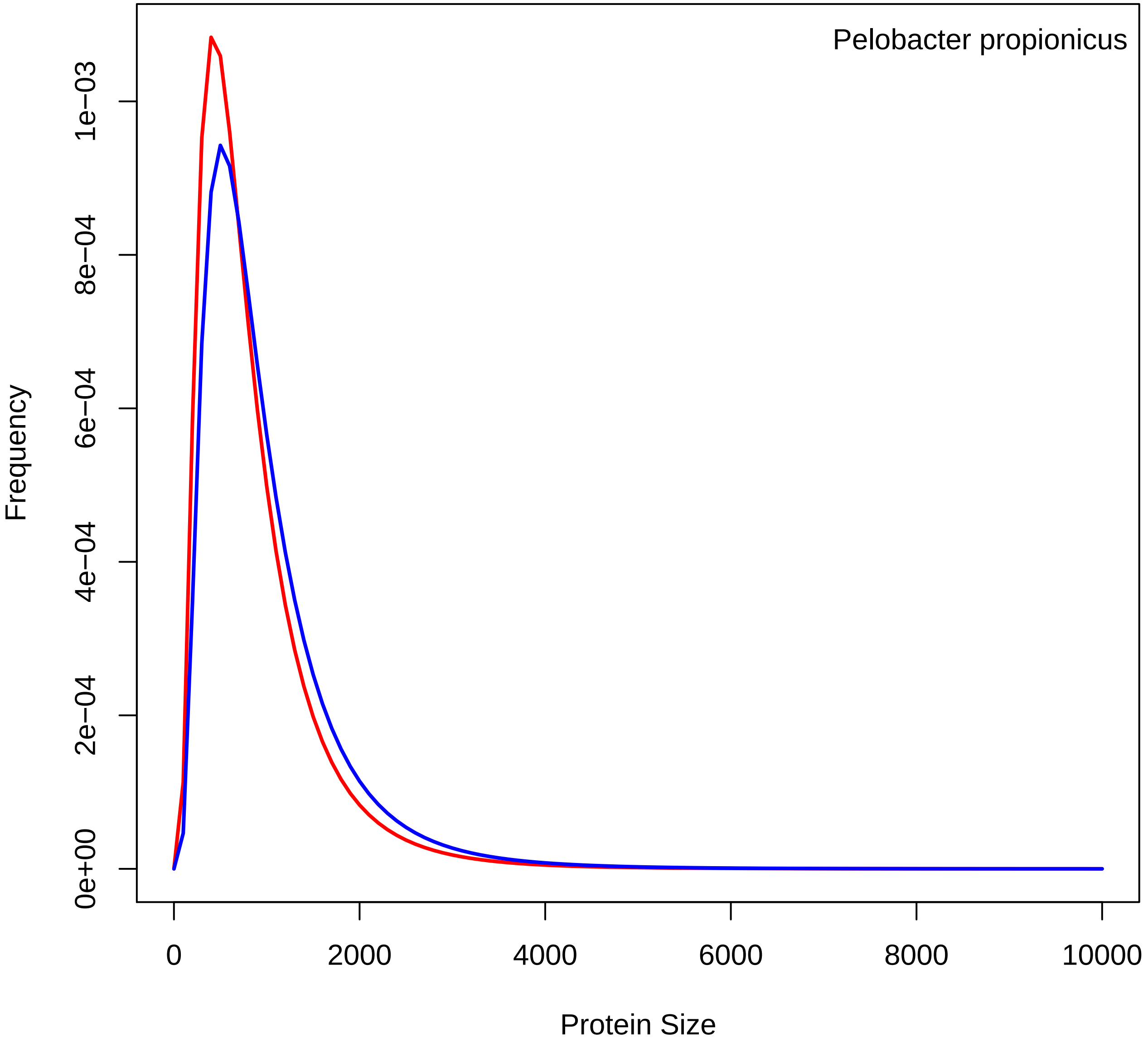

Supplement 3 − Figure 245

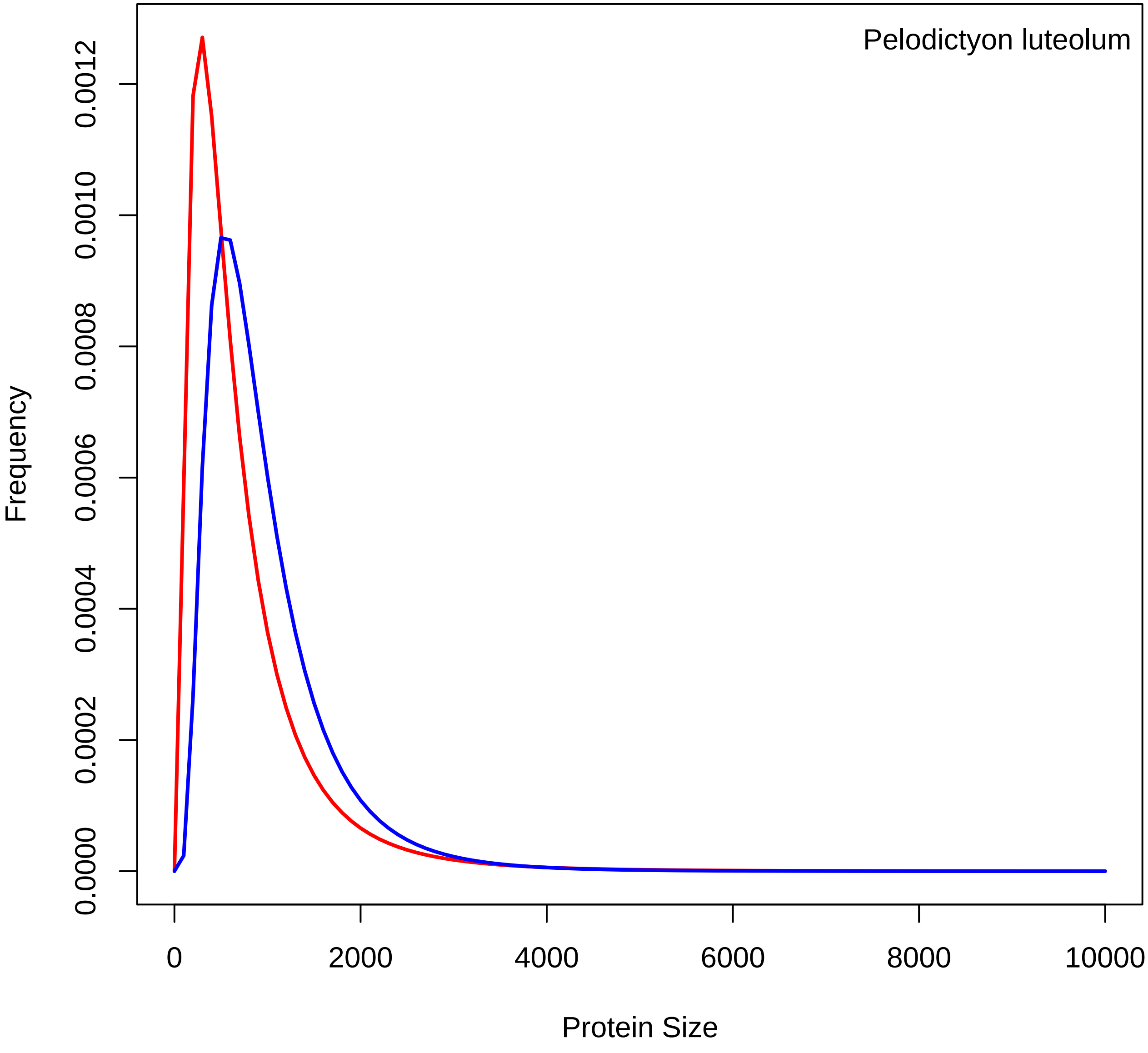

Supplement 3 − Figure 246

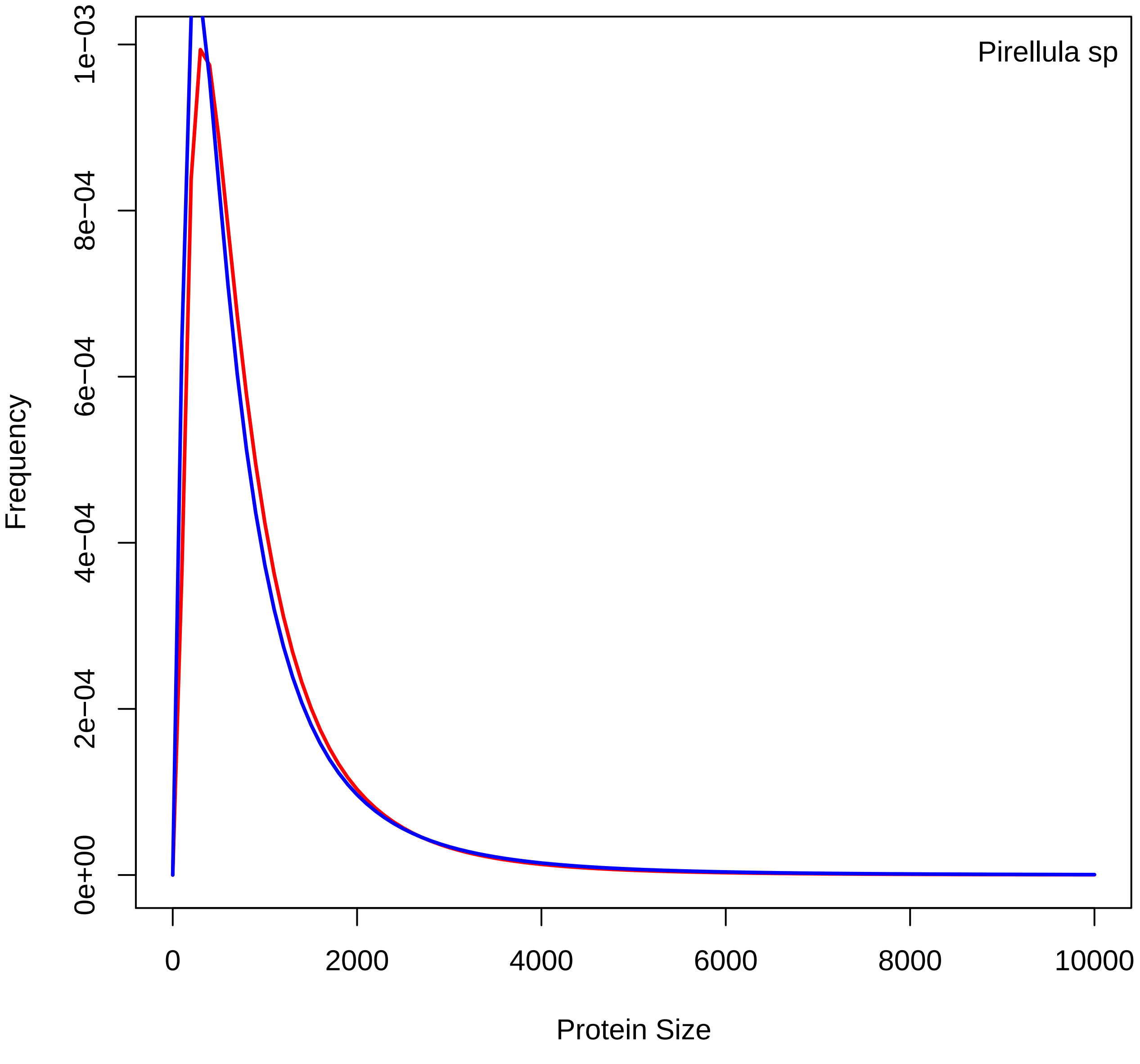

Supplement 3 − Figure 247

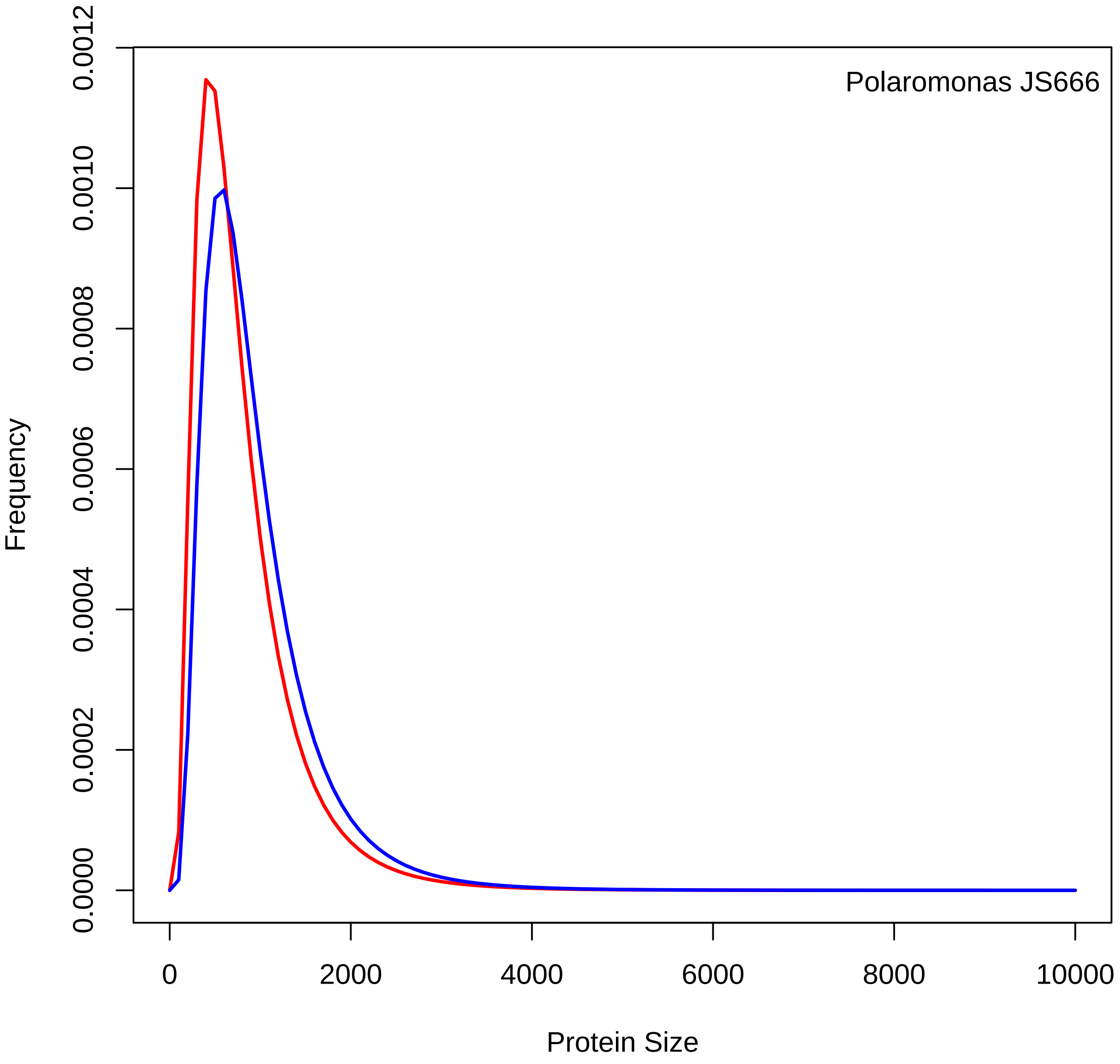

Supplement 3 − Figure 248

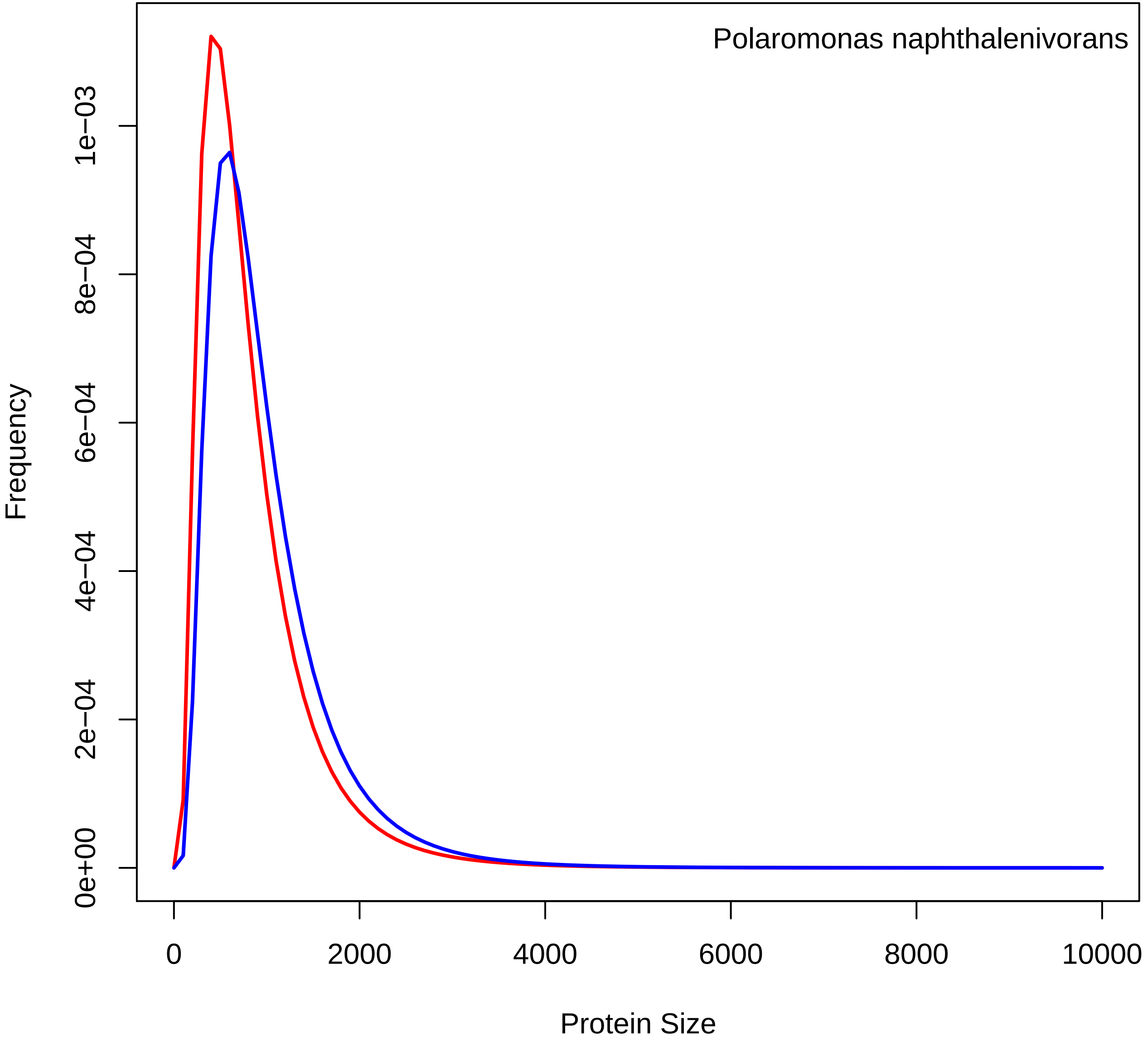

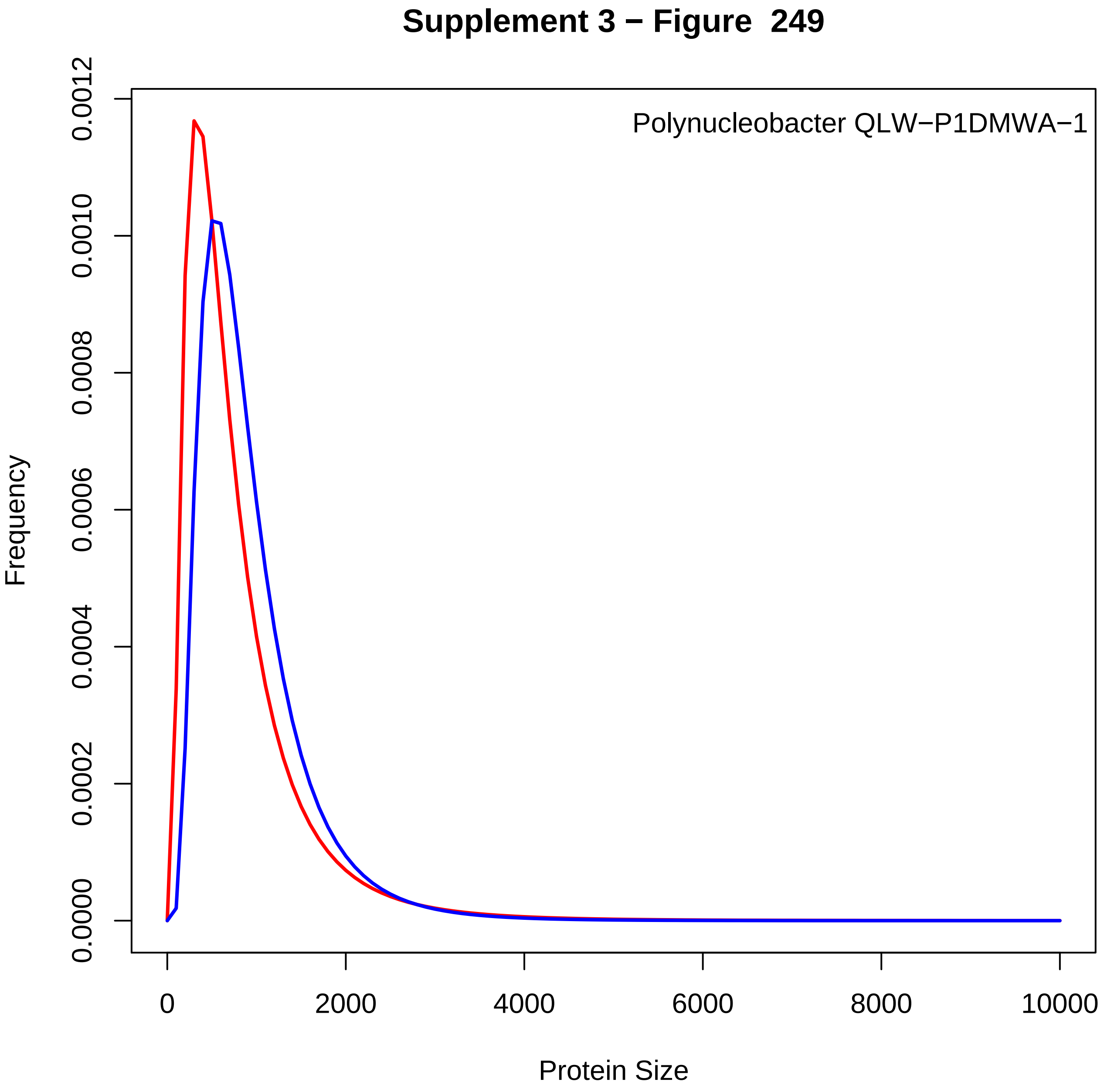
Supplement 3 − Figure 249
Polynucleobacter QLW−P1DMWA−1
Frequency
0.0000
0.0002
0.0004
0.0006
0.0008
0.0010
0.0012
0
2000
4000
6000
8000
10000
Protein Size

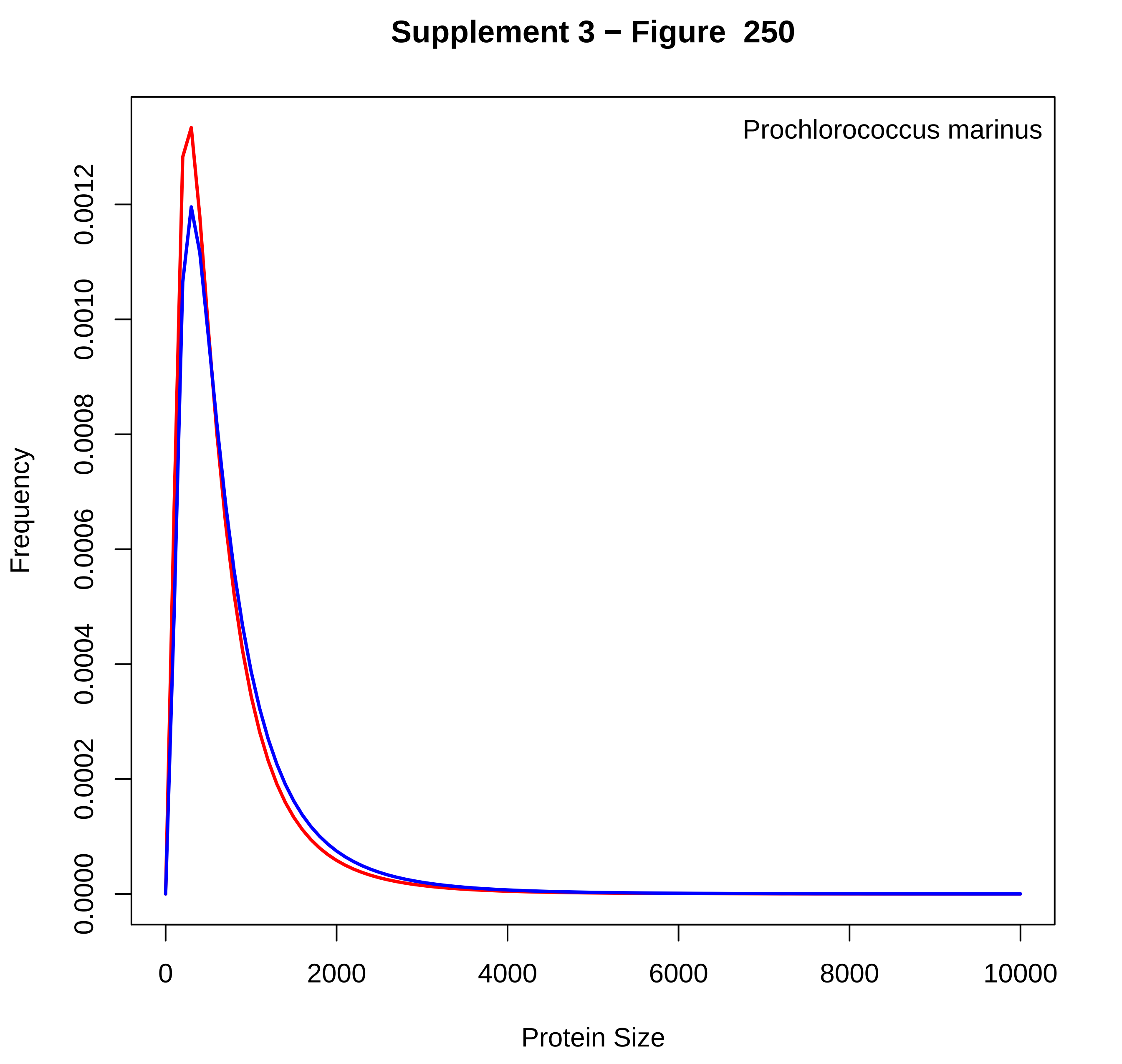
Supplement 3 − Figure 250
Prochlorococcus marinus
Frequency
0.0000
0.0002
0.0004
0.0006
0.0008
0.0010
0.0012
0
2000
4000
6000
8000
10000
Protein Size

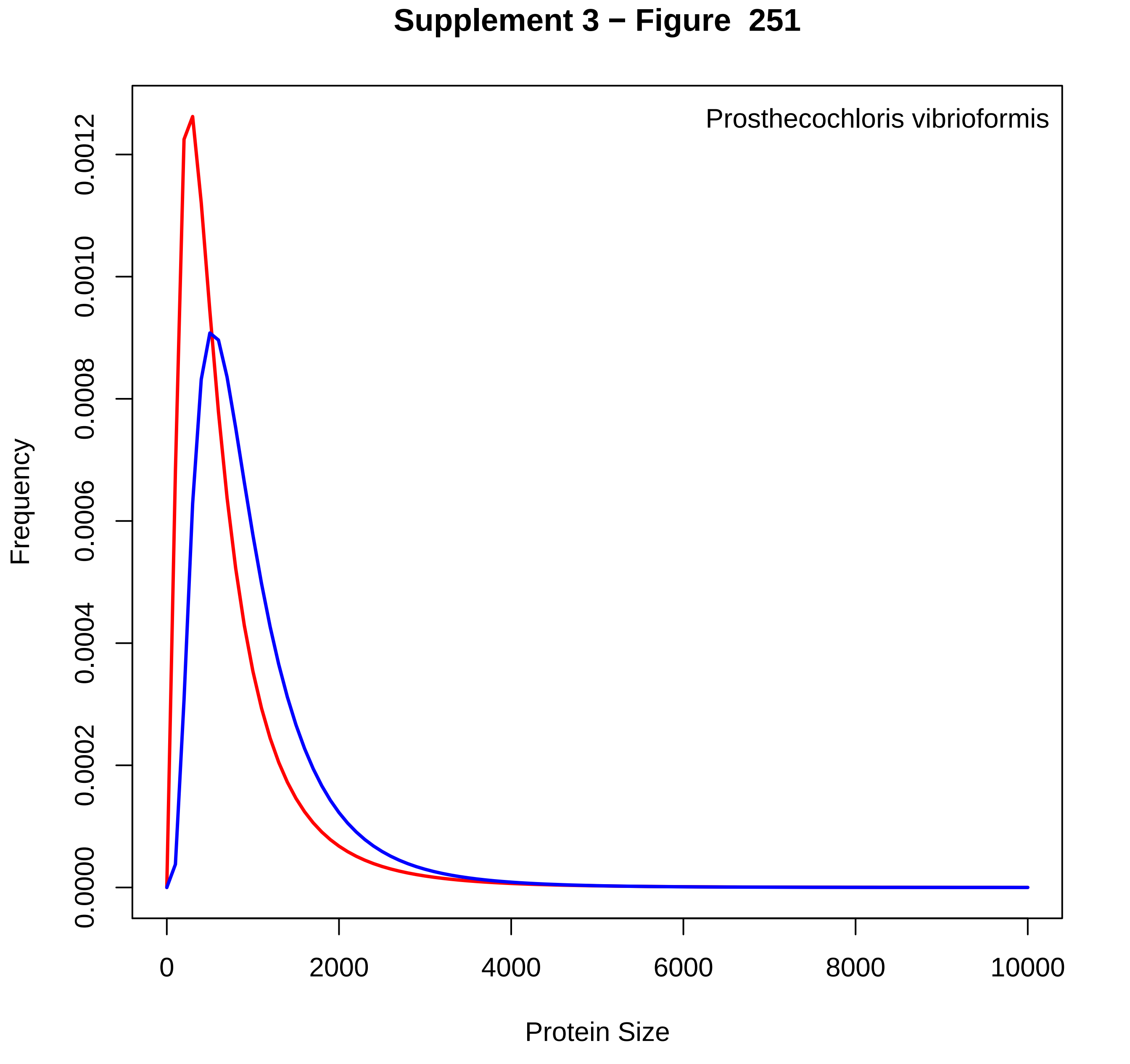
Supplement 3 − Figure 251
Prosthecochloris vibrioformis
Frequency
0.0000
0.0002
0.0004
0.0006
0.0008
0.0010
0.0012
0
2000
4000
6000
8000
10000
Protein Size

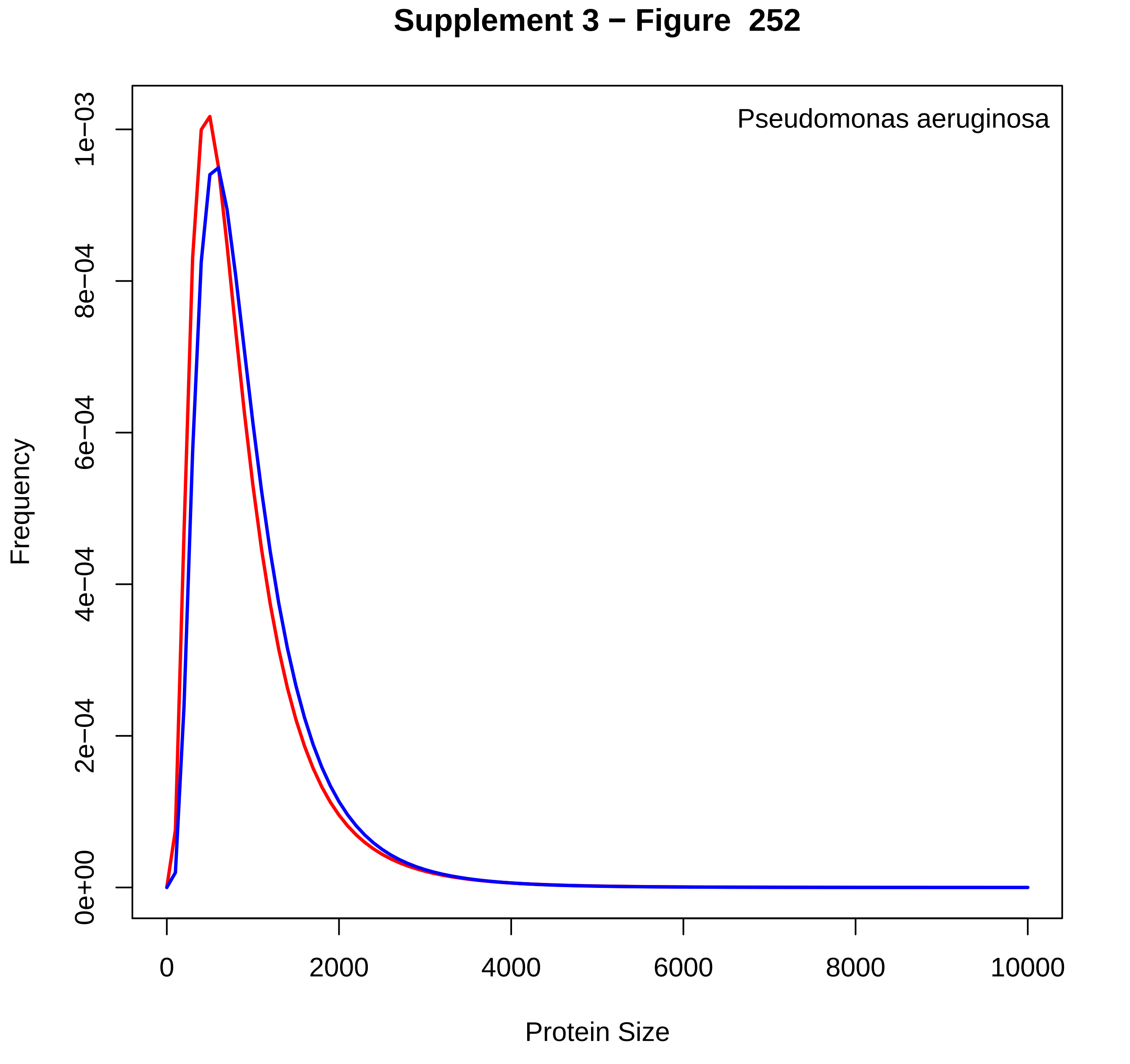
Supplement 3 − Figure 252
Pseudomonas aeruginosa
Frequency
0e+00
2e−04
4e−04
6e−04
8e−04
1e−03
0
2000
4000
6000
8000
10000
Protein Size

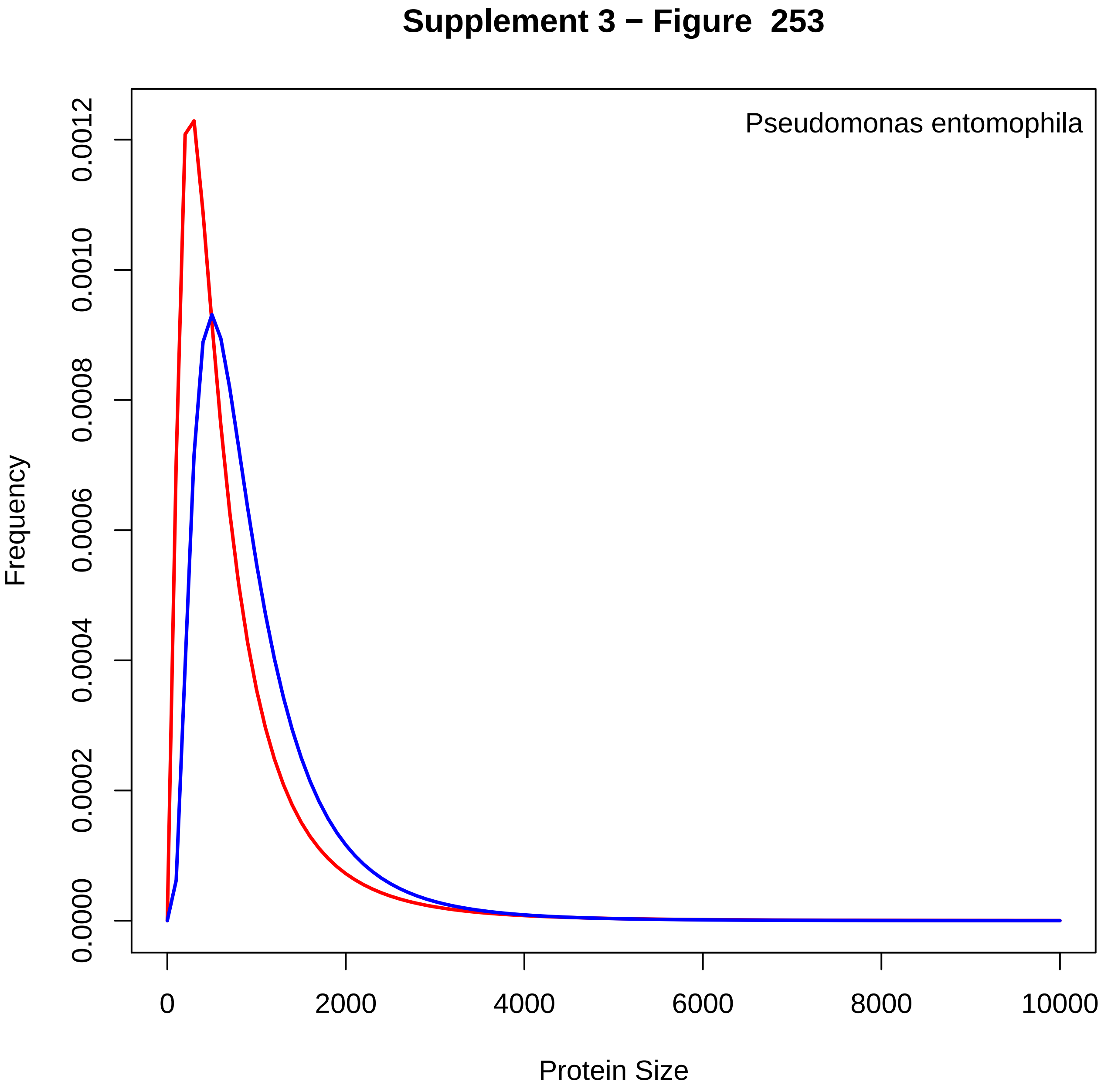
Supplement 3 − Figure 253
Pseudomonas entomophila
Frequency
0.0000
0.0002
0.0004
0.0006
0.0008
0.0010
0.0012
0
2000
4000
6000
8000
10000
Protein Size

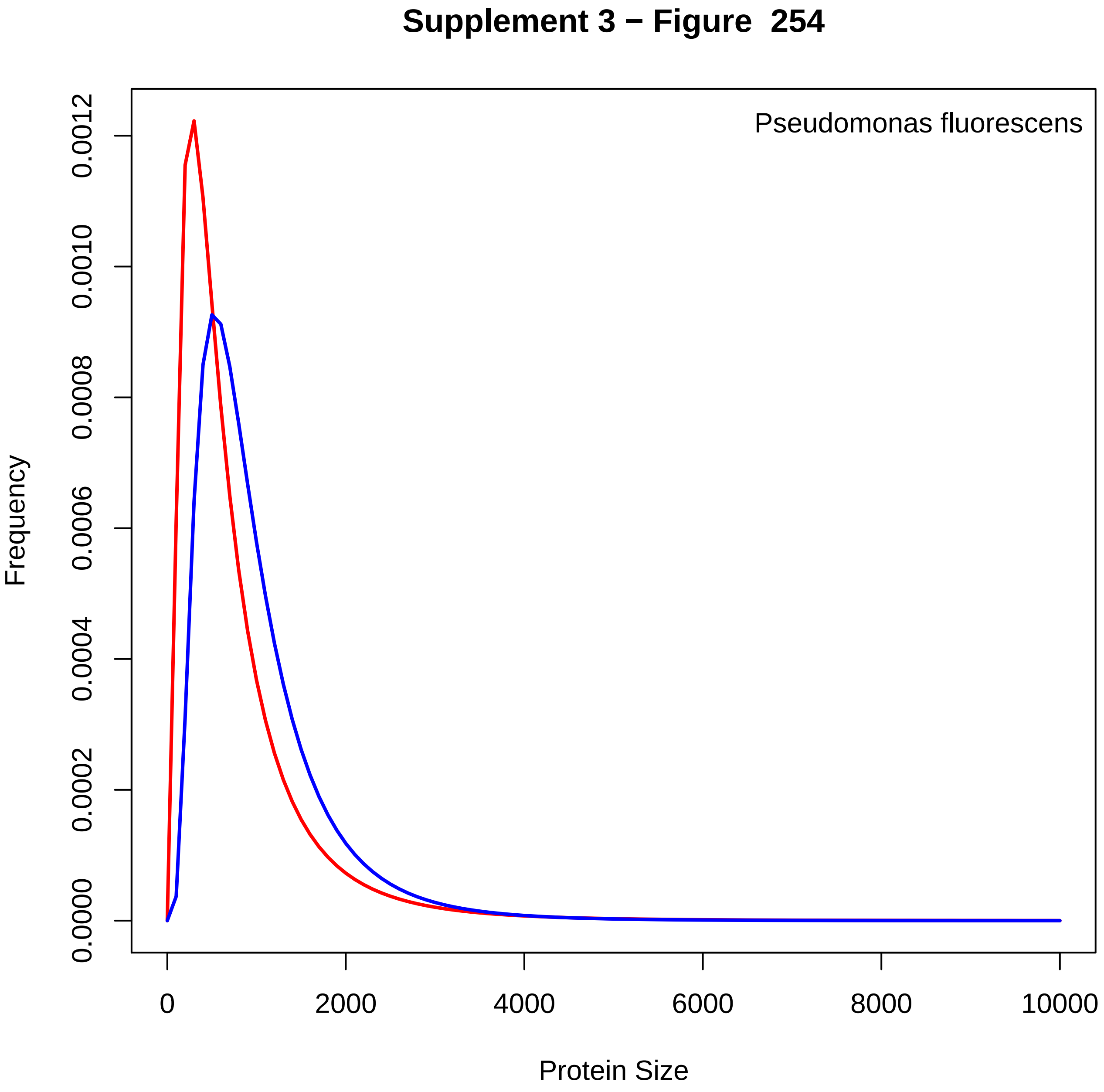
Supplement 3 − Figure 254
Pseudomonas fluorescens
Frequency
0.0000
0.0002
0.0004
0.0006
0.0008
0.0010
0.0012
0
2000
4000
6000
8000
10000
Protein Size

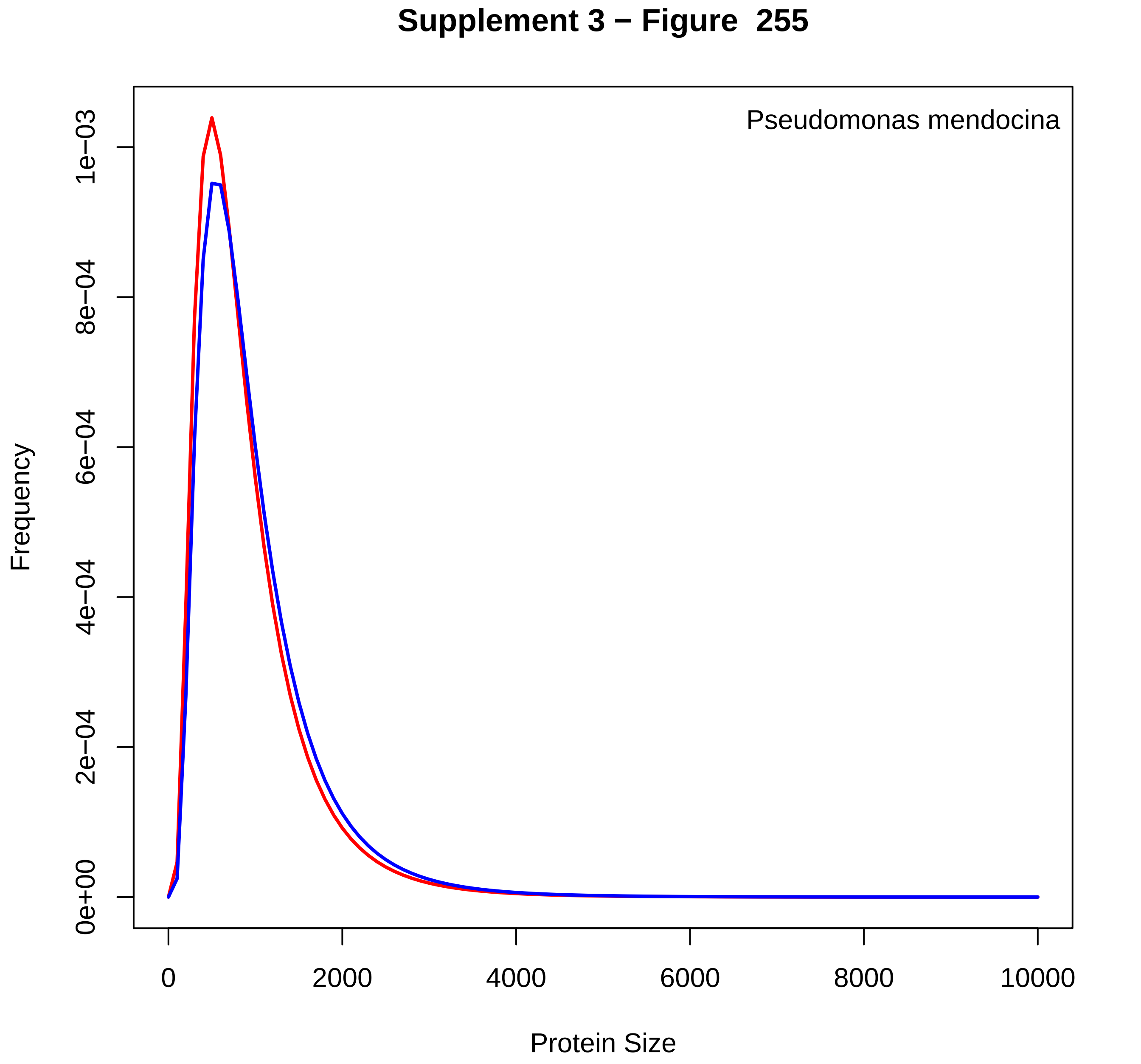
Supplement 3 − Figure 255
Pseudomonas mendocina
Frequency
Protein Size
0e+00
2e−04
4e−04
6e−04
8e−04
1e−03
0
2000
4000
6000
8000
10000

Supplement 3 − Figure 256

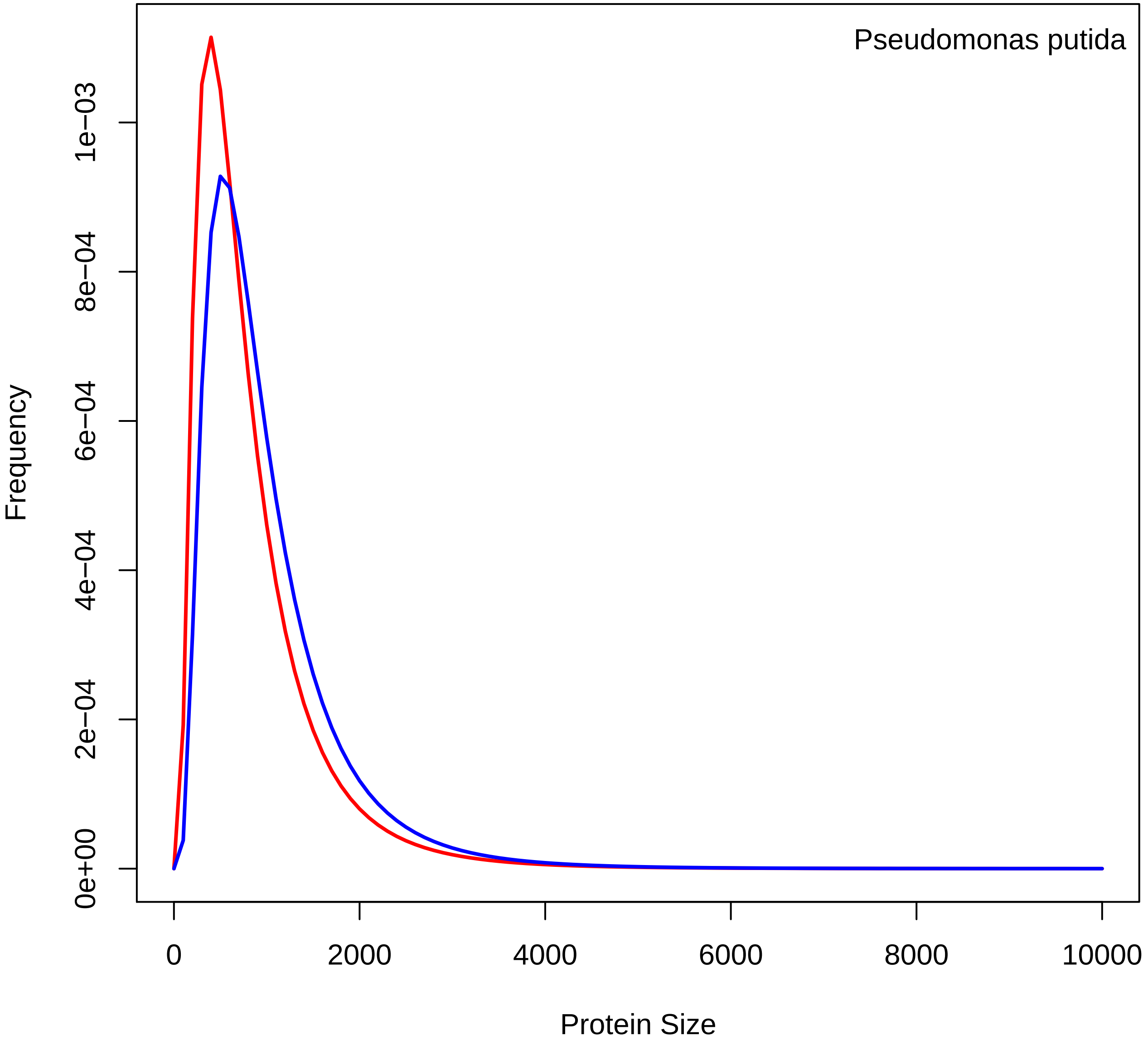

Supplement 3 − Figure 257

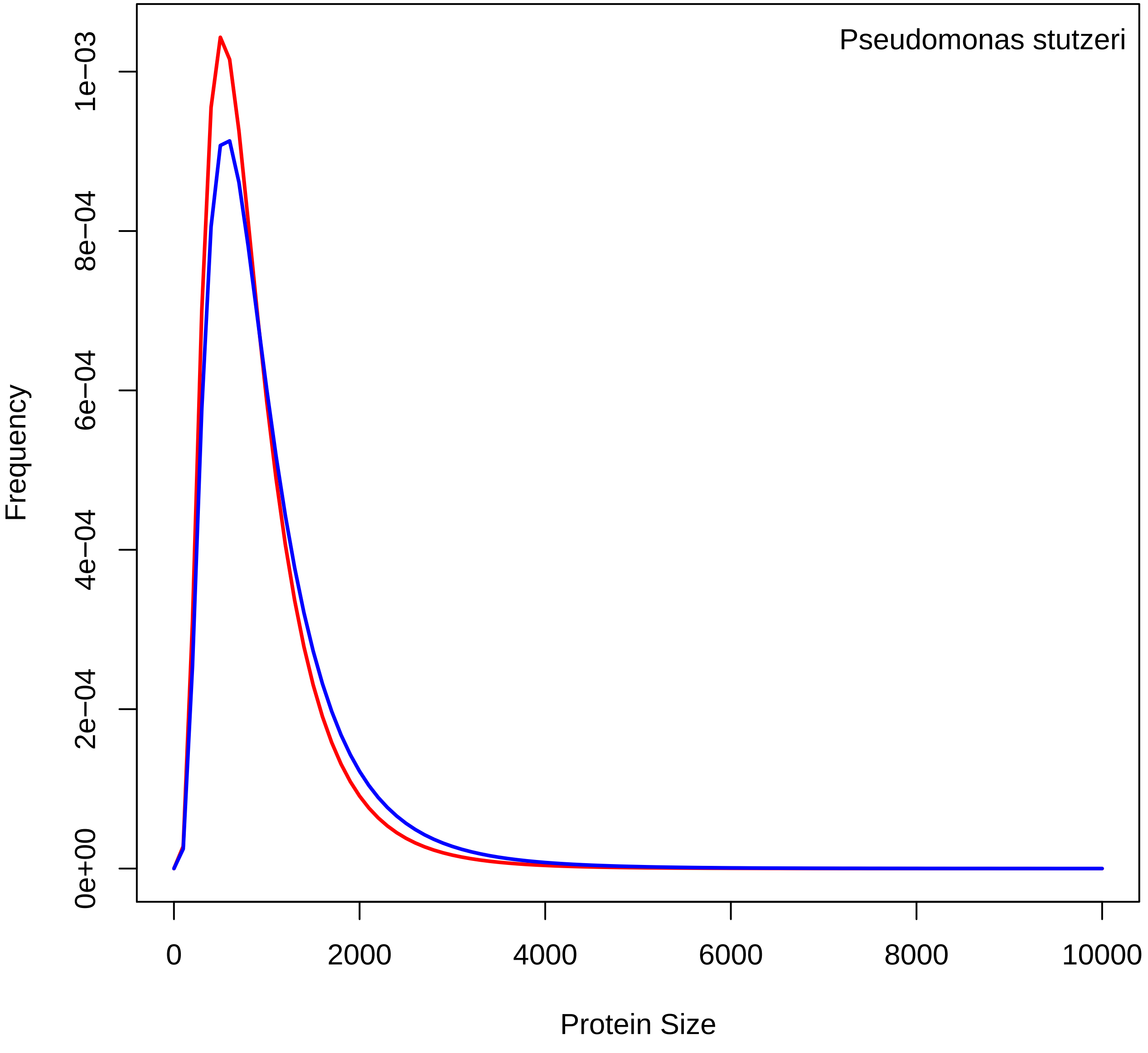

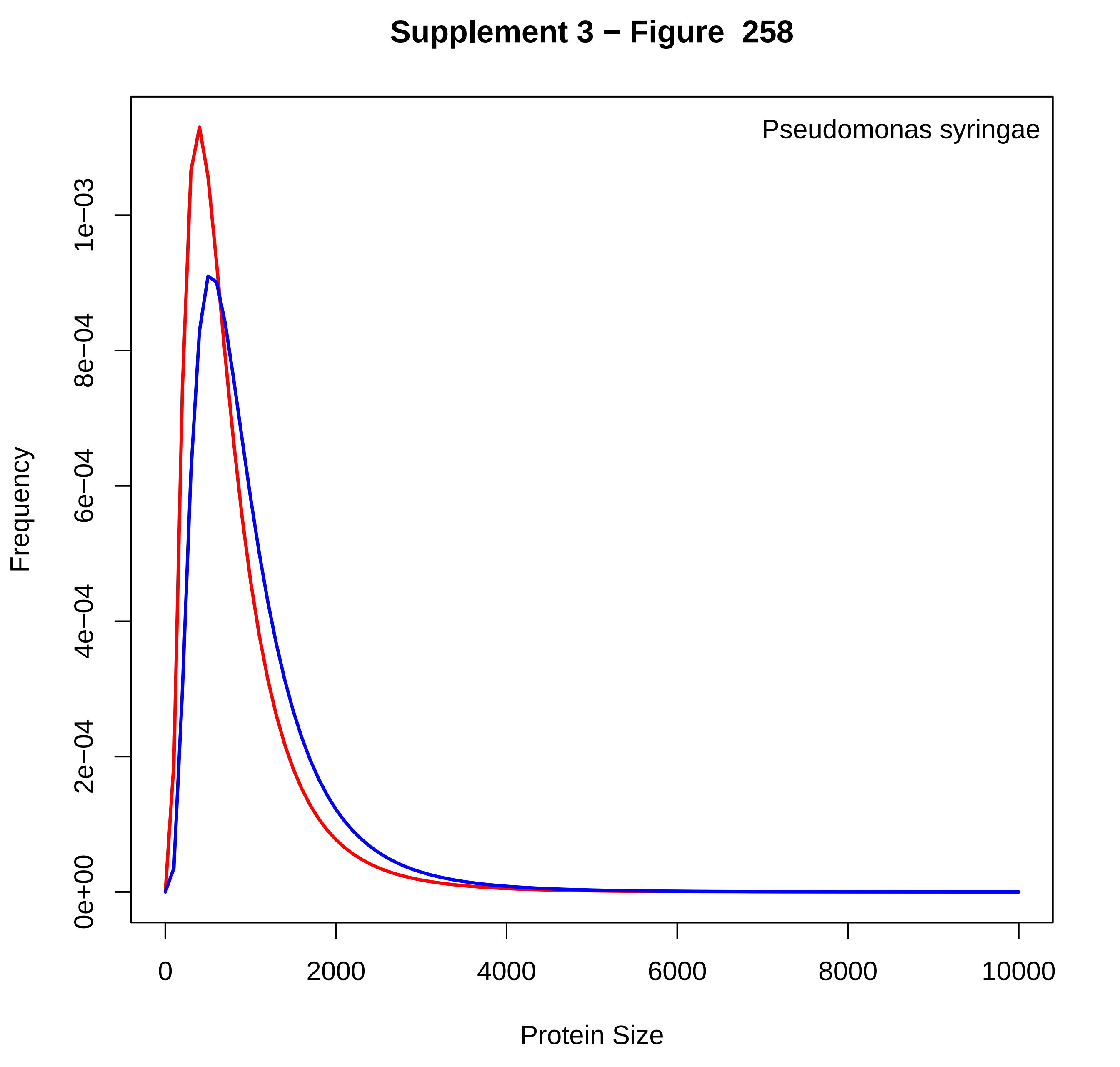
Supplement 3 − Figure 258
Pseudomonas syringae
Frequency
0e+00
2e−04
4e−04
6e−04
8e−04
1e−03
0
2000
4000
6000
8000
10000
Protein Size

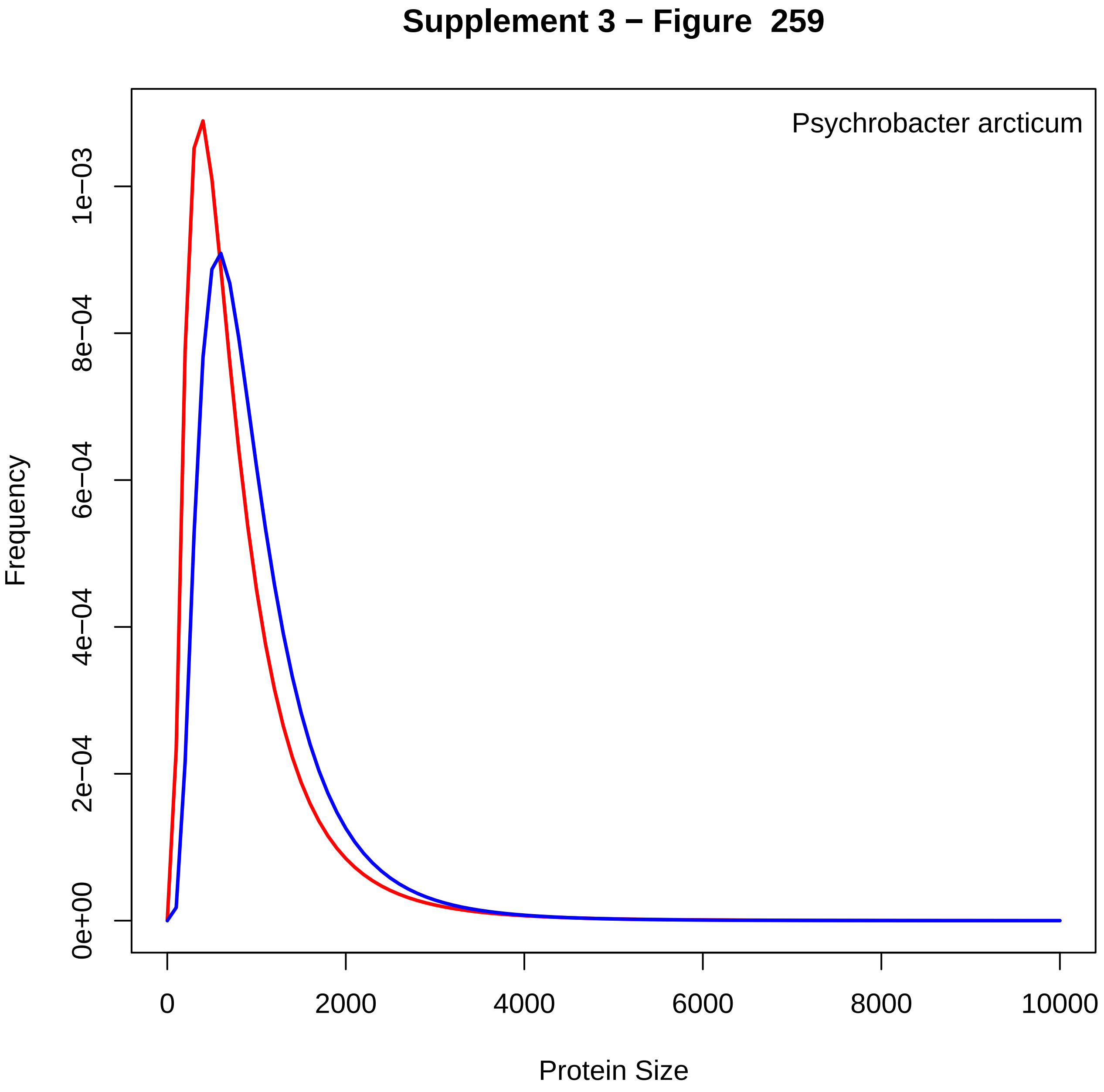

Supplement 3 − Figure 259
Psychrobacter arcticum
Frequency
0e+00
2e−04
4e−04
6e−04
8e−04
1e−03
0
2000
4000
6000
8000
10000
Protein Size

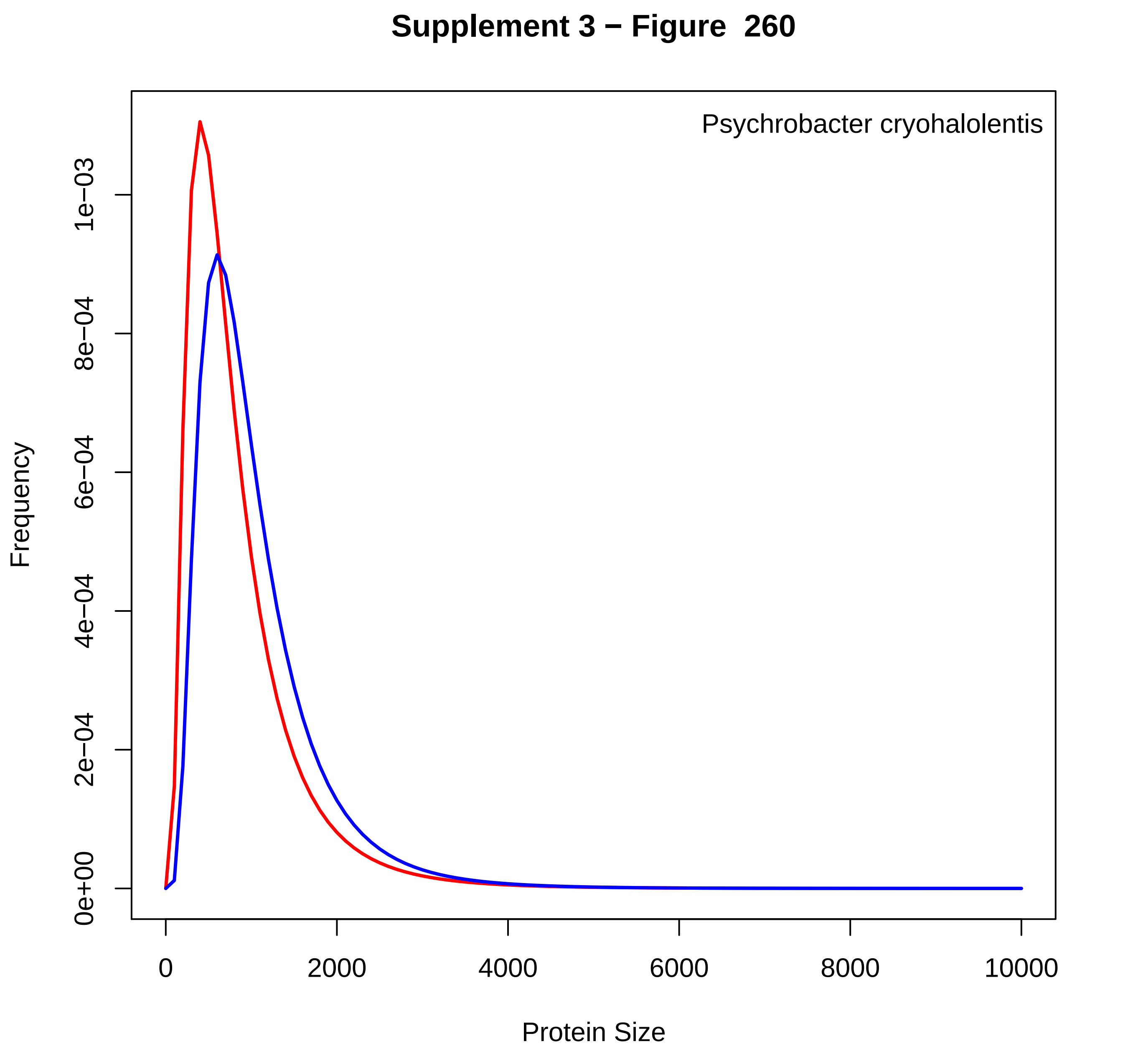
Supplement 3 − Figure 260
Psychrobacter cryohalolentis
Frequency
0e+00
2e−04
4e−04
6e−04
8e−04
1e−03
0
2000
4000
6000
8000
10000
Protein Size

Supplement 3 − Figure 261

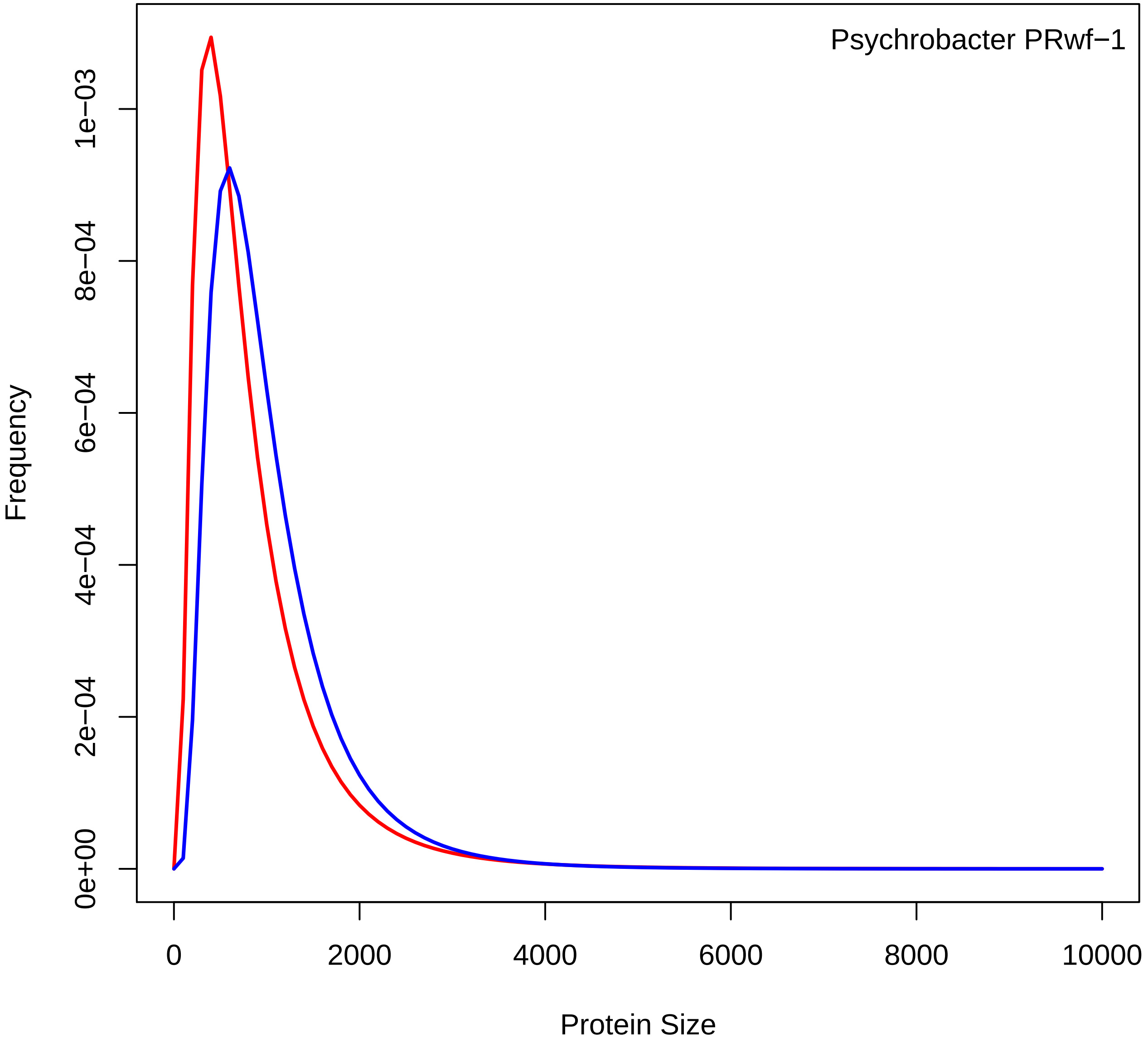

Supplement 3 − Figure 262

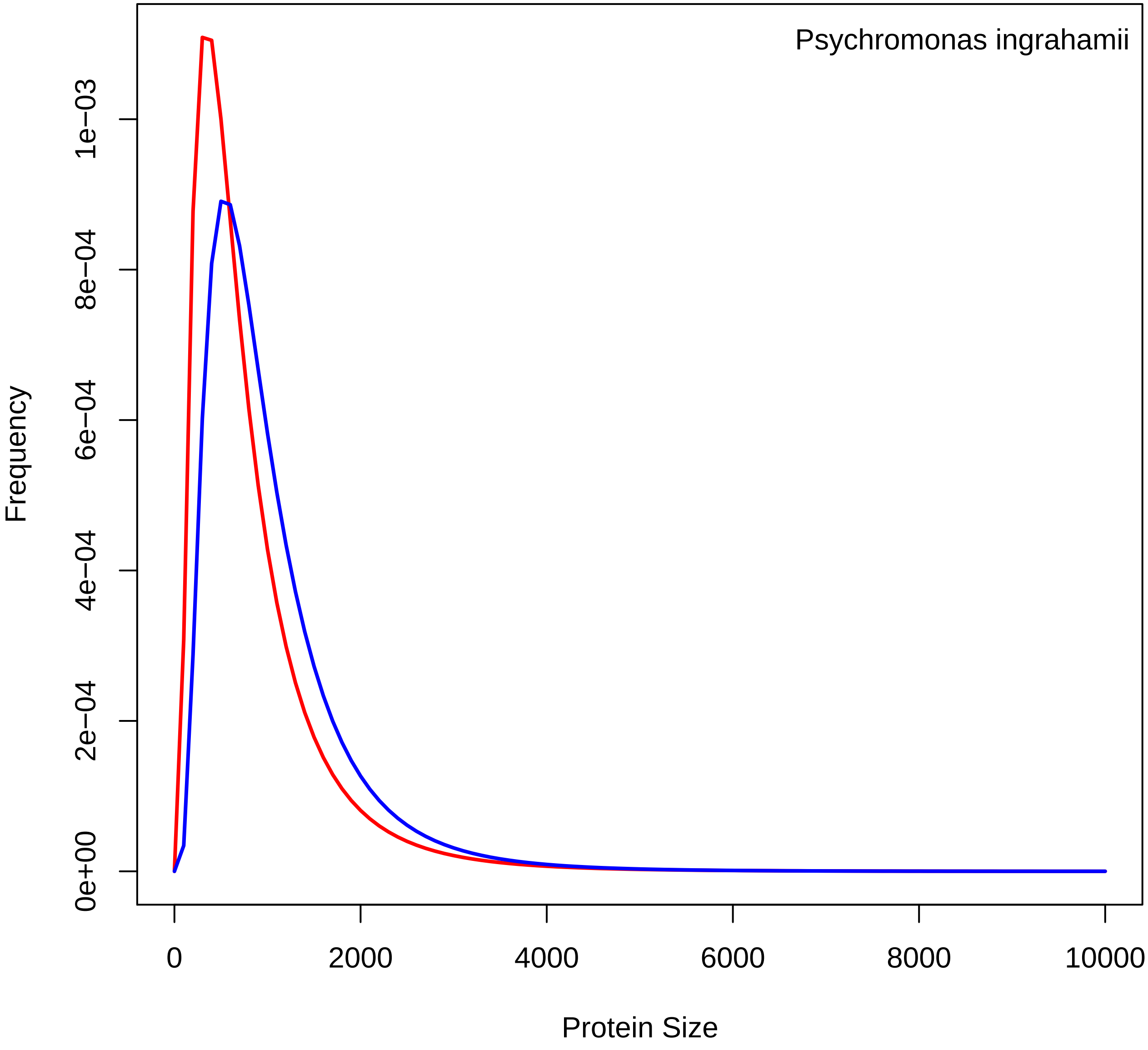

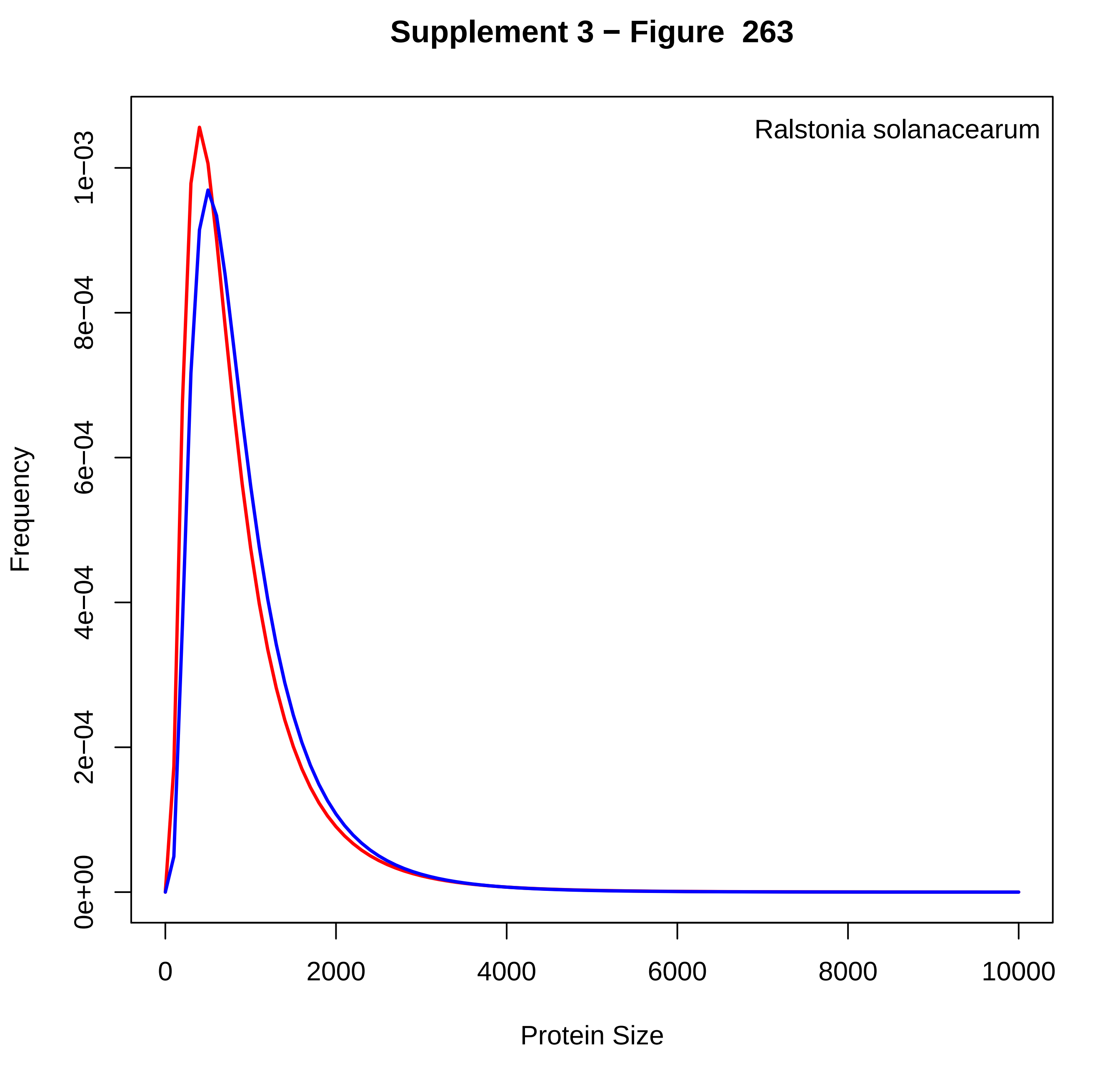

Supplement 3 − Figure 263
Ralstonia solanacearum
Frequency
0e+00
2e−04
4e−04
6e−04
8e−04
1e−03
0
2000
4000
6000
8000
10000
Protein Size

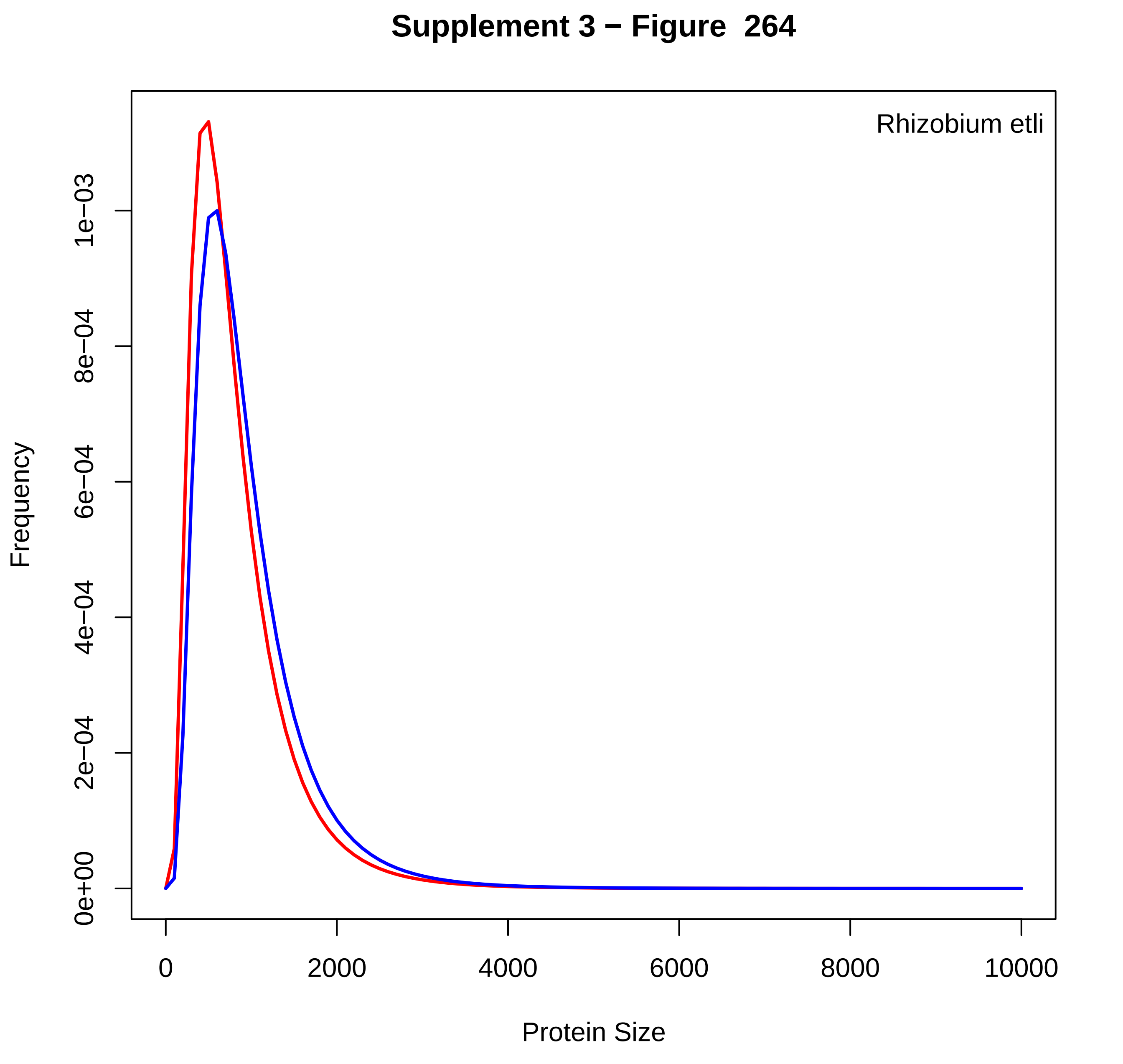
Supplement 3 − Figure 264
Rhizobium etli
Frequency
0e+00
2e−04
4e−04
6e−04
8e−04
1e−03
0
2000
4000
6000
8000
10000
Protein Size

Supplement 3 − Figure 265

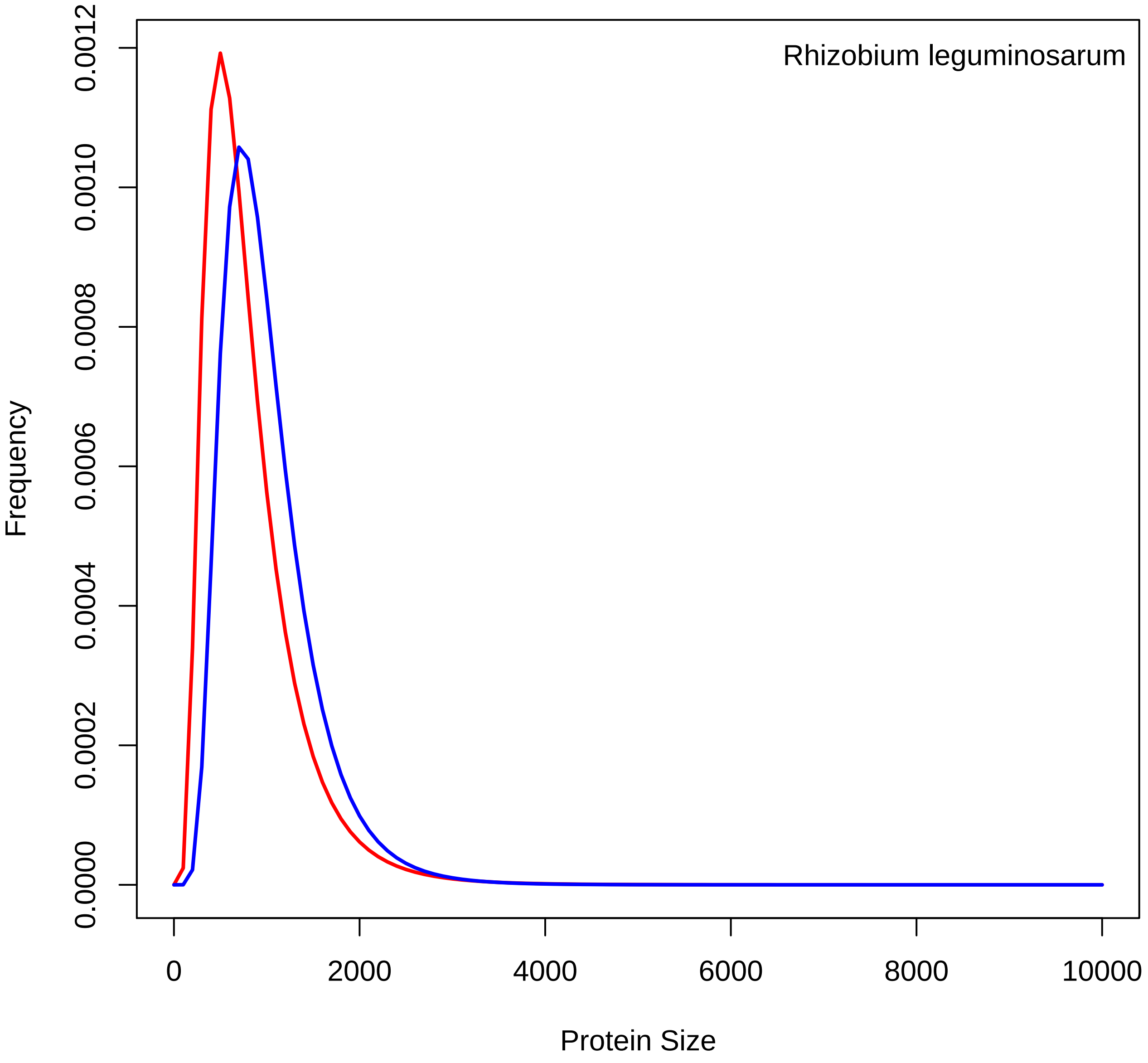

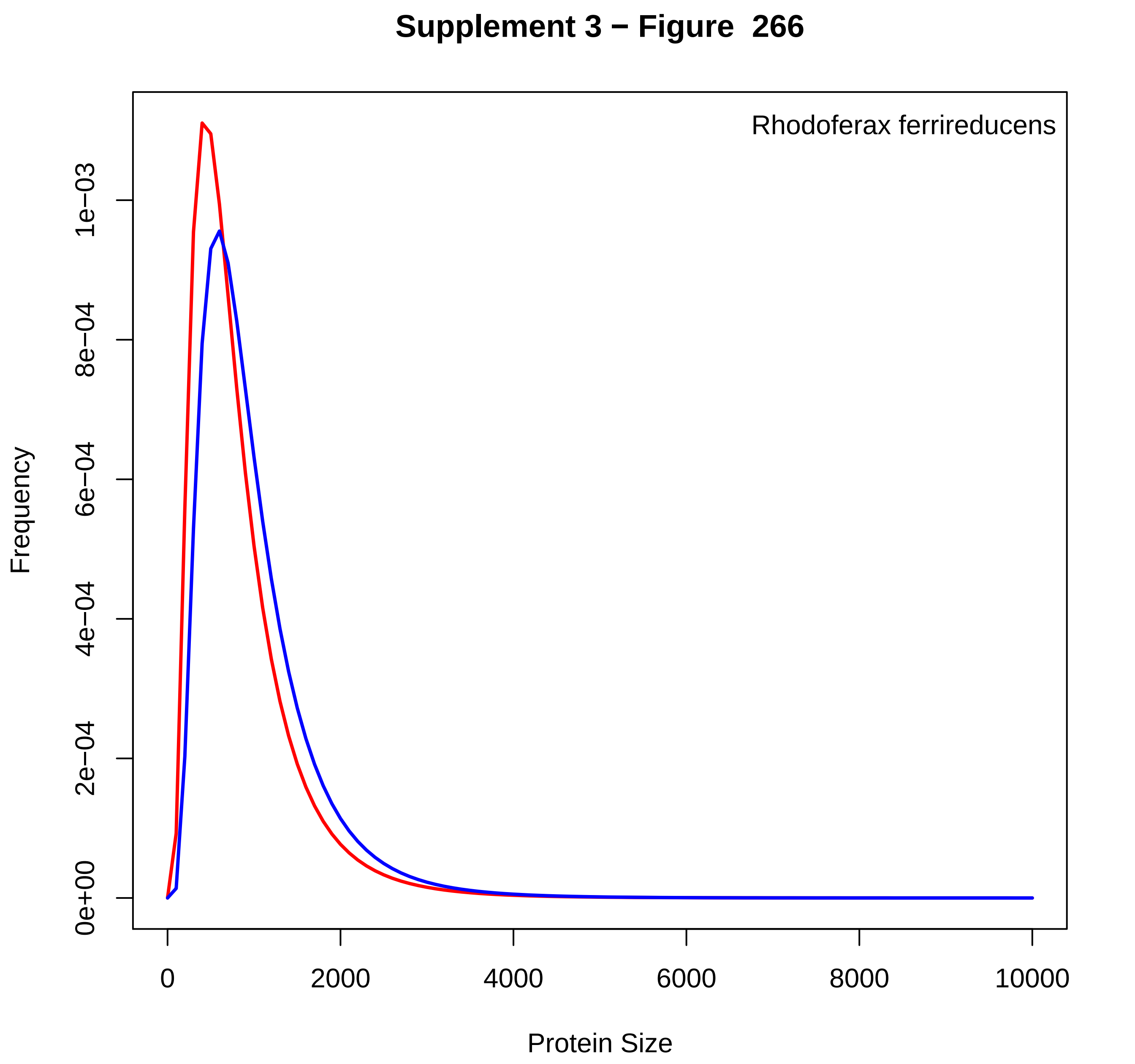
Supplement 3 − Figure 266
Rhodoferax ferrireducens
Frequency
0e+00
2e−04
4e−04
6e−04
8e−04
1e−03
0
2000
4000
6000
8000
10000
Protein Size

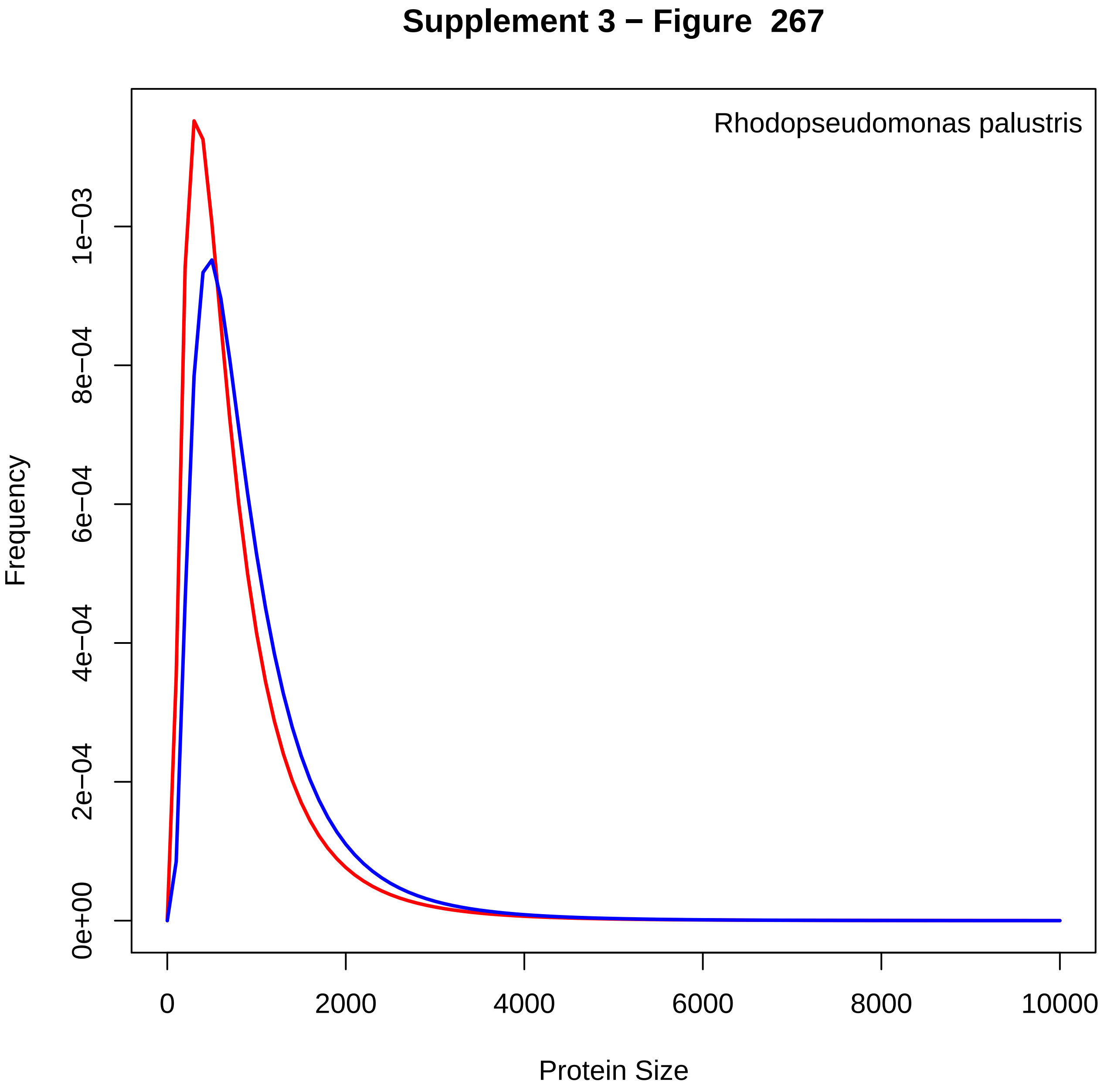
Supplement 3 − Figure 267
Rhodopseudomonas palustris
Frequency
0e+00
2e−04
4e−04
6e−04
8e−04
1e−03
0
2000
4000
6000
8000
10000
Protein Size

Supplement 3 − Figure 268

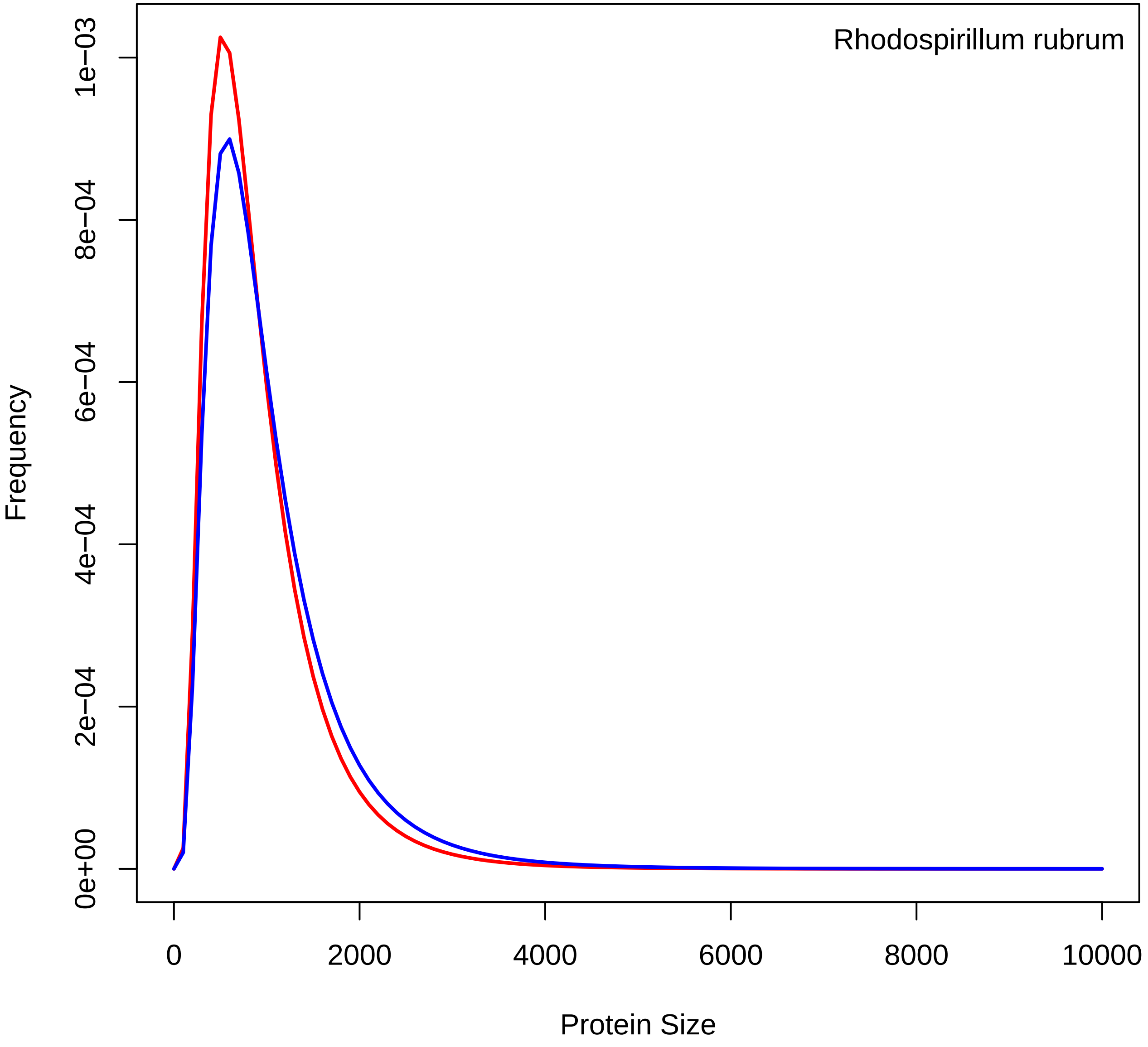

Supplement 3 − Figure 269

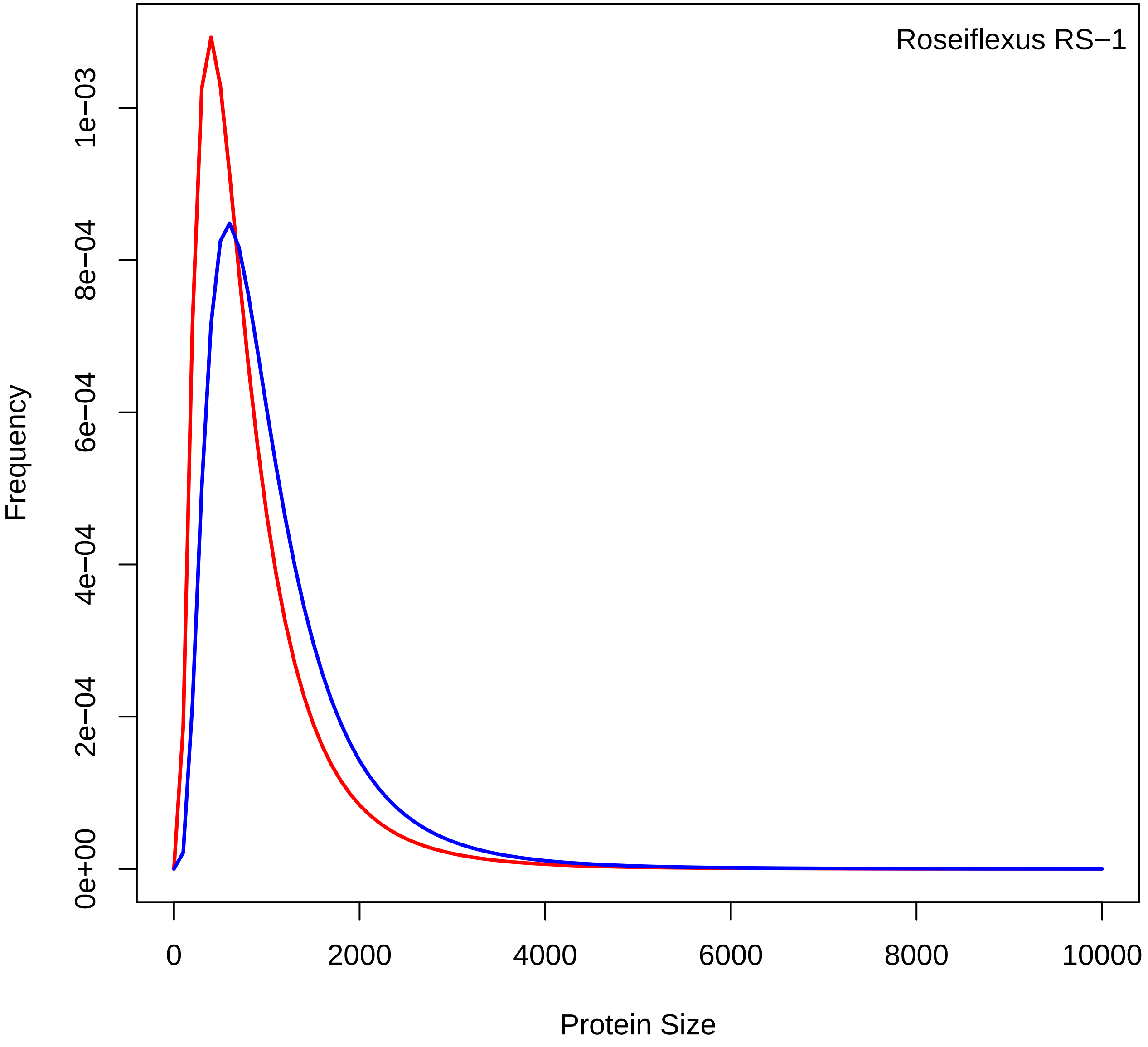

Supplement 3 − Figure 270

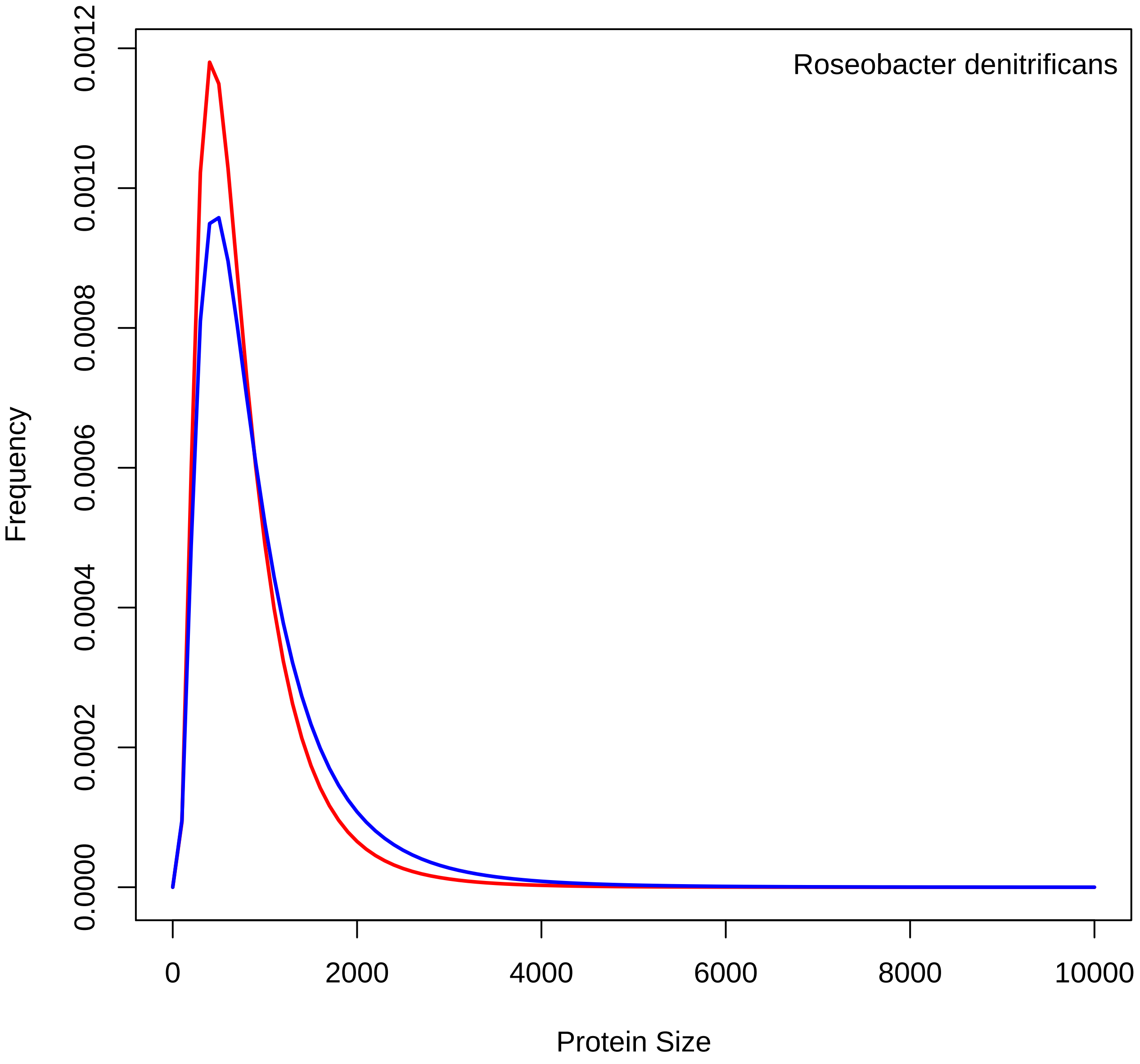

Supplement 3 − Figure 271

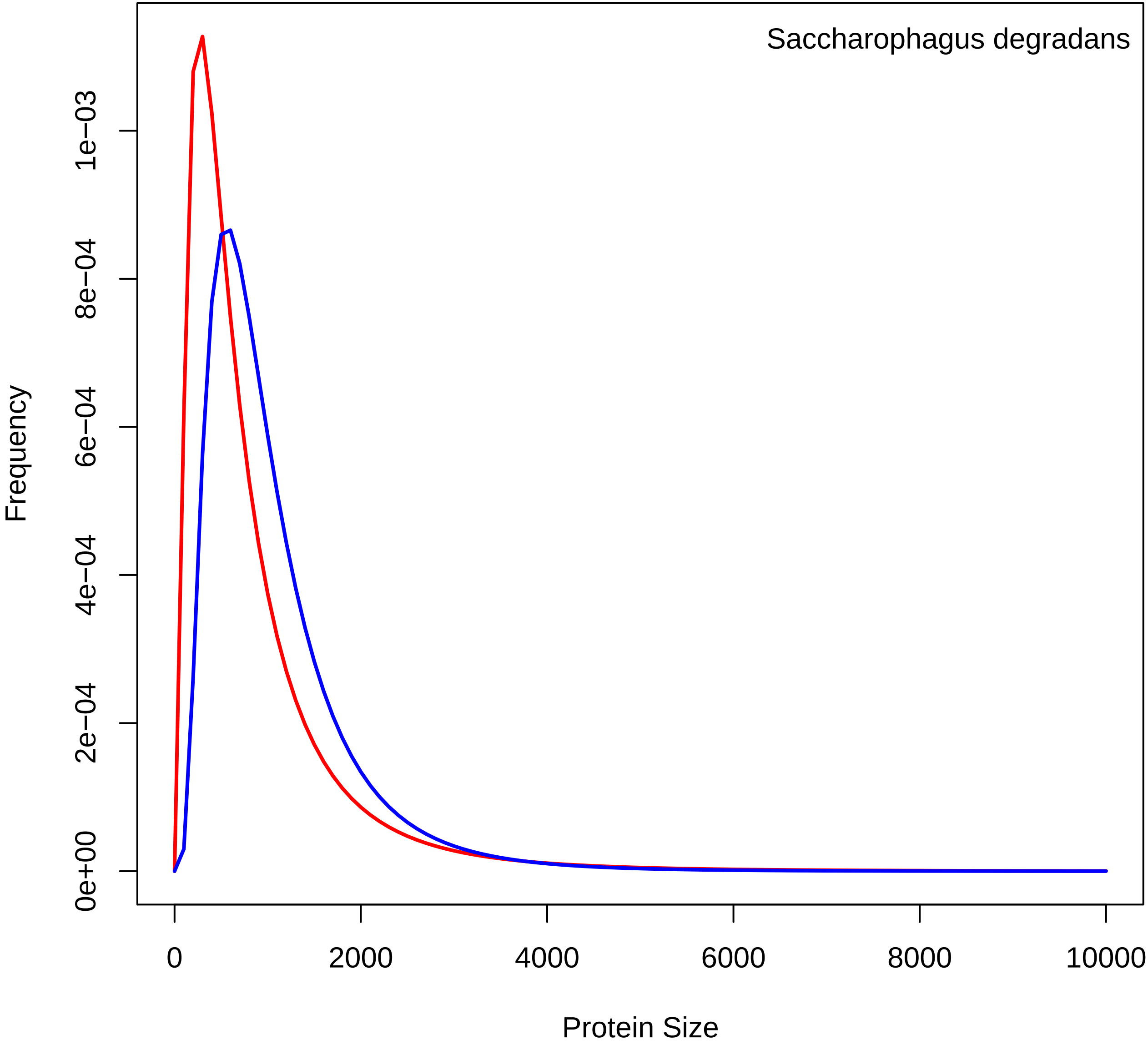

Supplement 3 − Figure 272

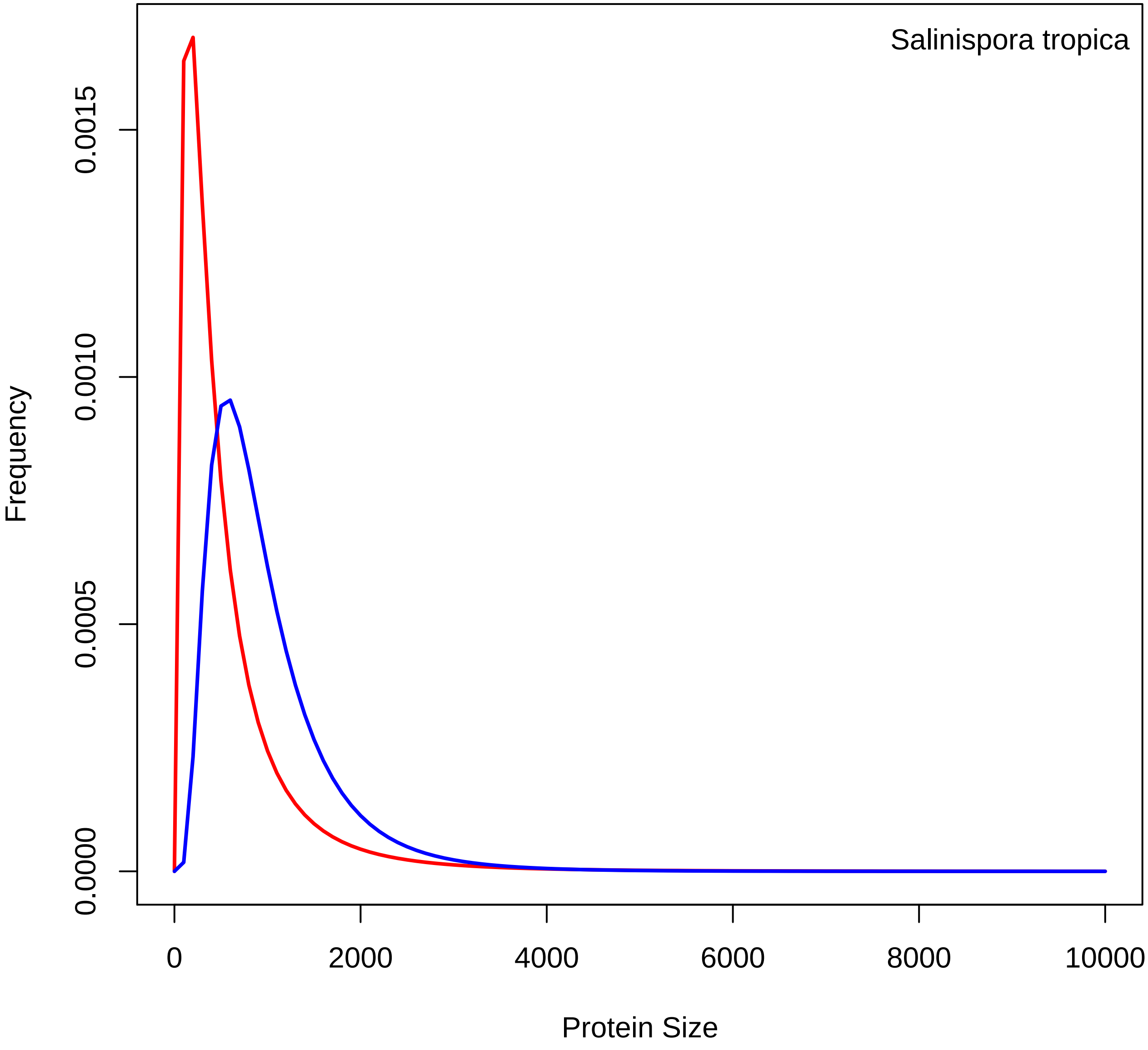

Supplement 3 − Figure 273

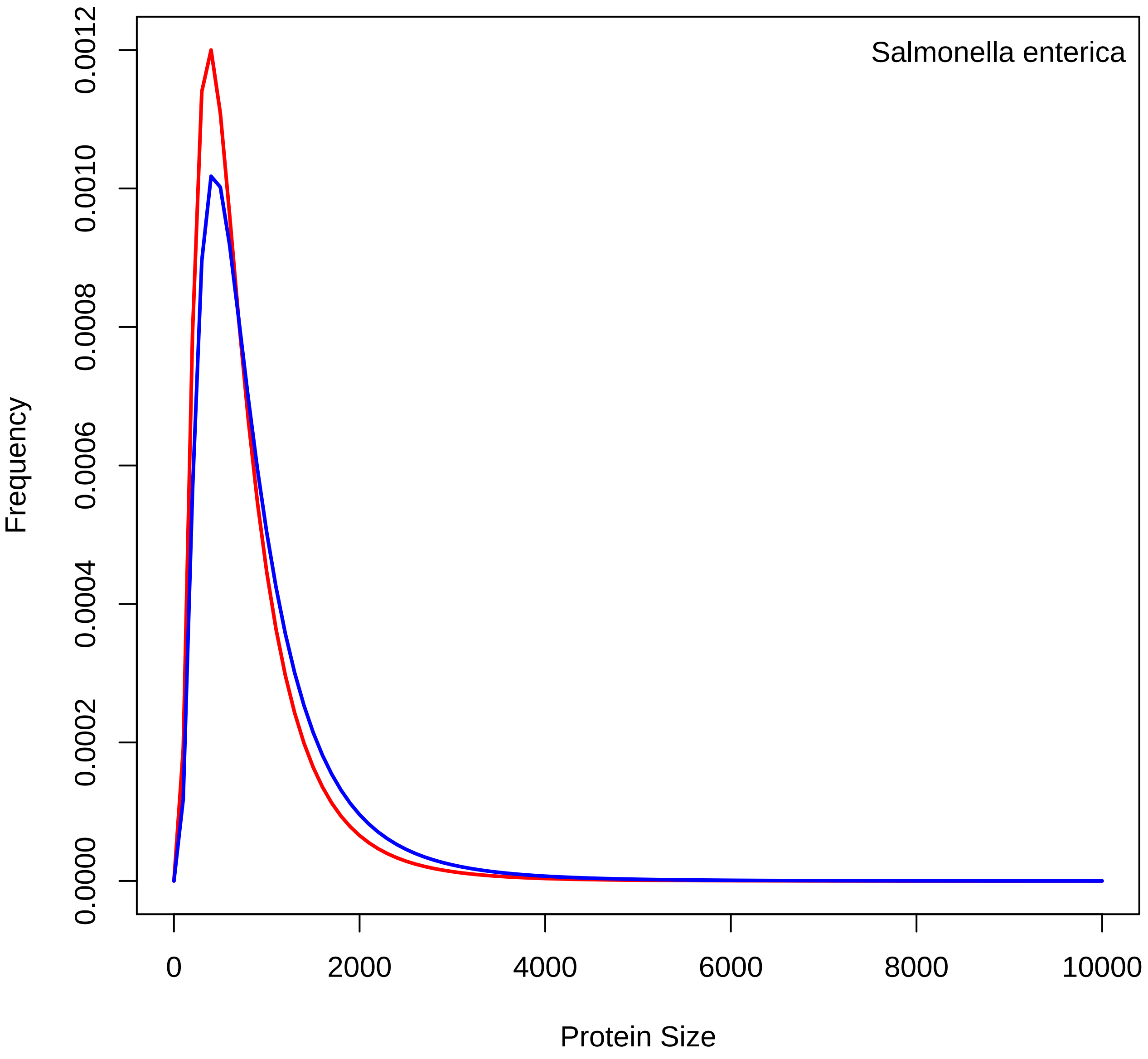

Supplement 3 − Figure 274

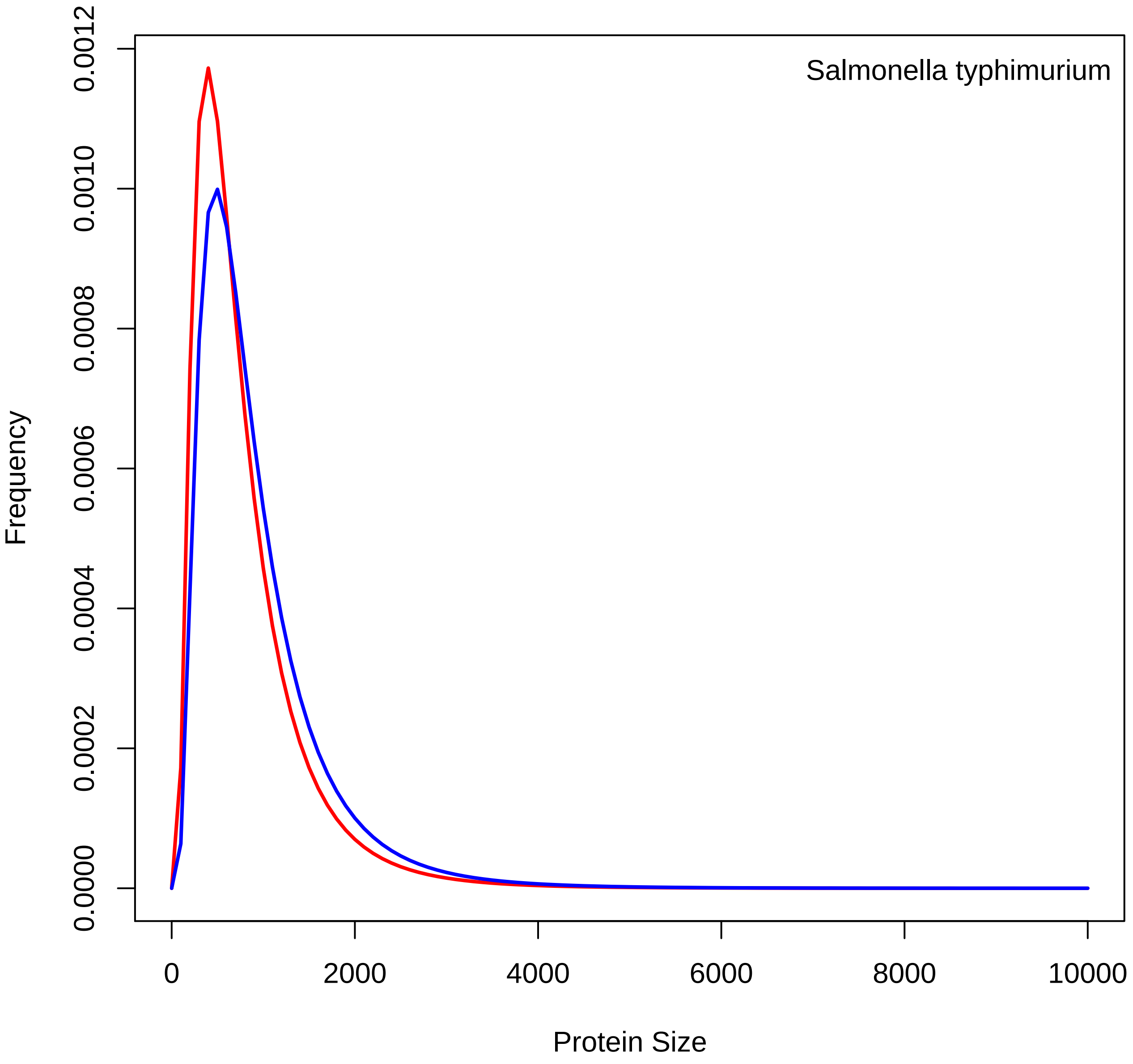

Supplement 3 − Figure 275

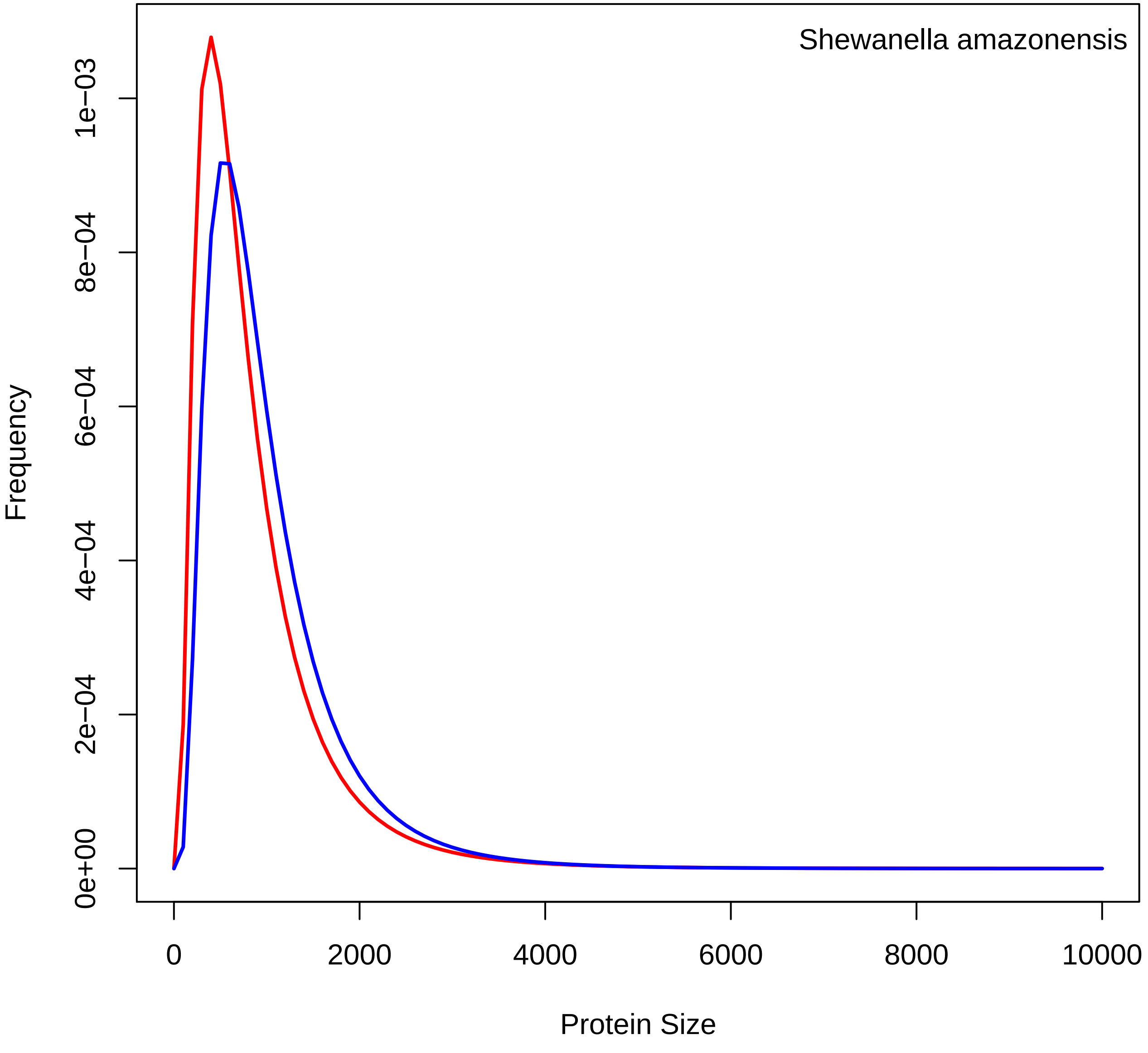

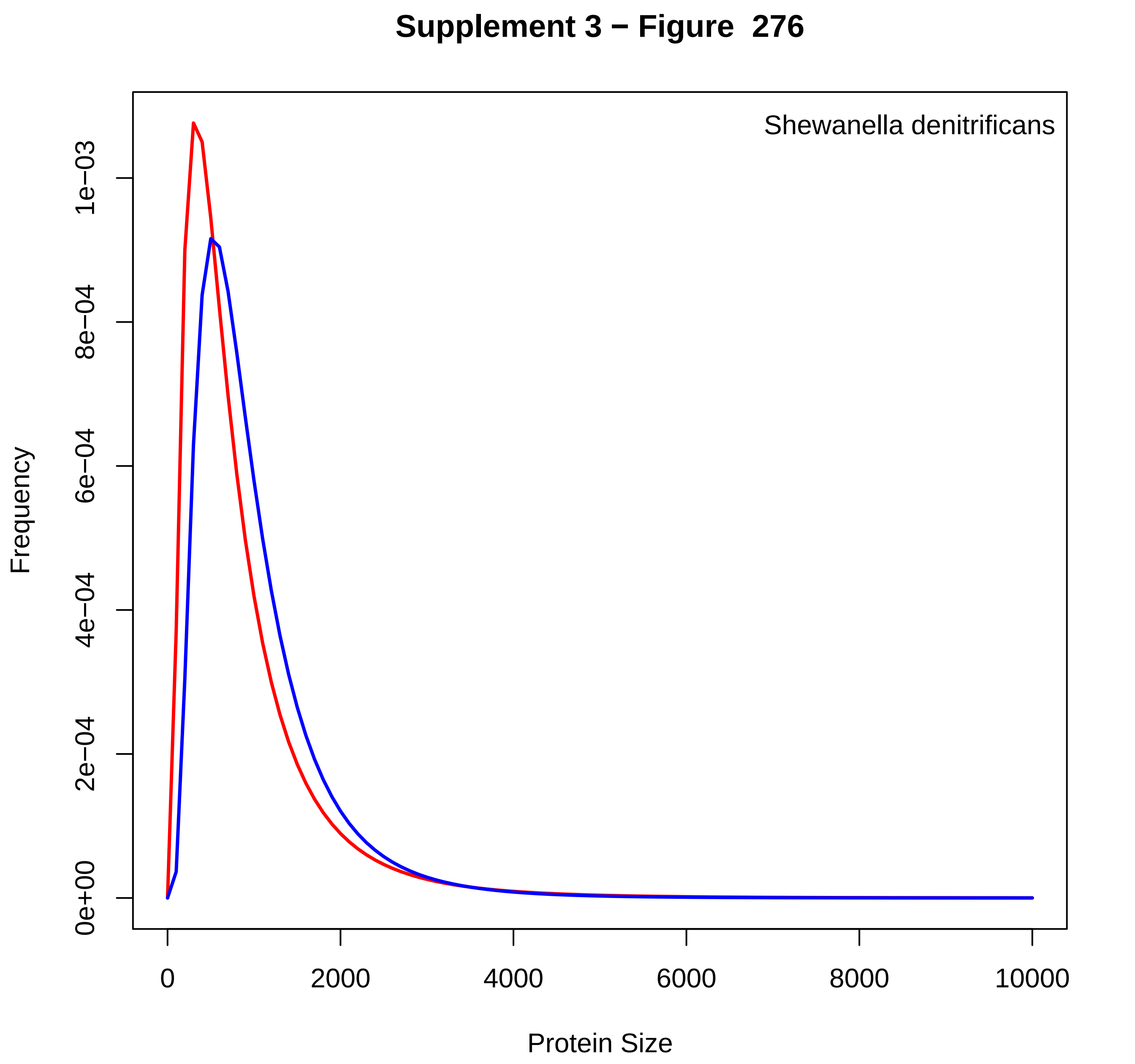

Supplement 3 − Figure 276
Shewanella denitrificans
Frequency
0e+00
2e−04
4e−04
6e−04
8e−04
1e−03
0
2000
4000
6000
8000
10000
Protein Size

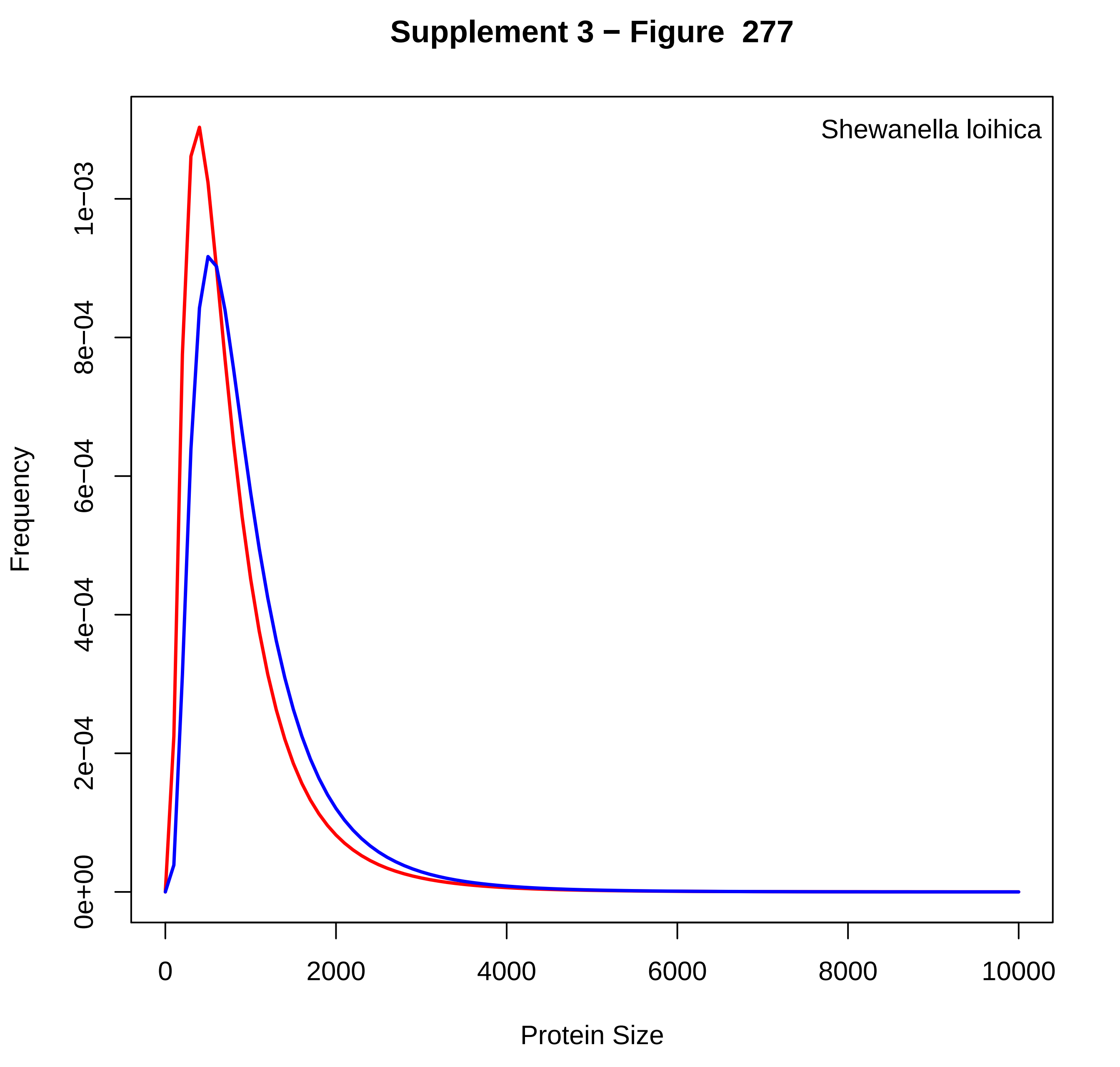

Supplement 3 − Figure 277
Shewanella loihica
Frequency
0e+00
2e−04
4e−04
6e−04
8e−04
1e−03
0
2000
4000
6000
8000
10000
Protein Size

Supplement 3 − Figure 278

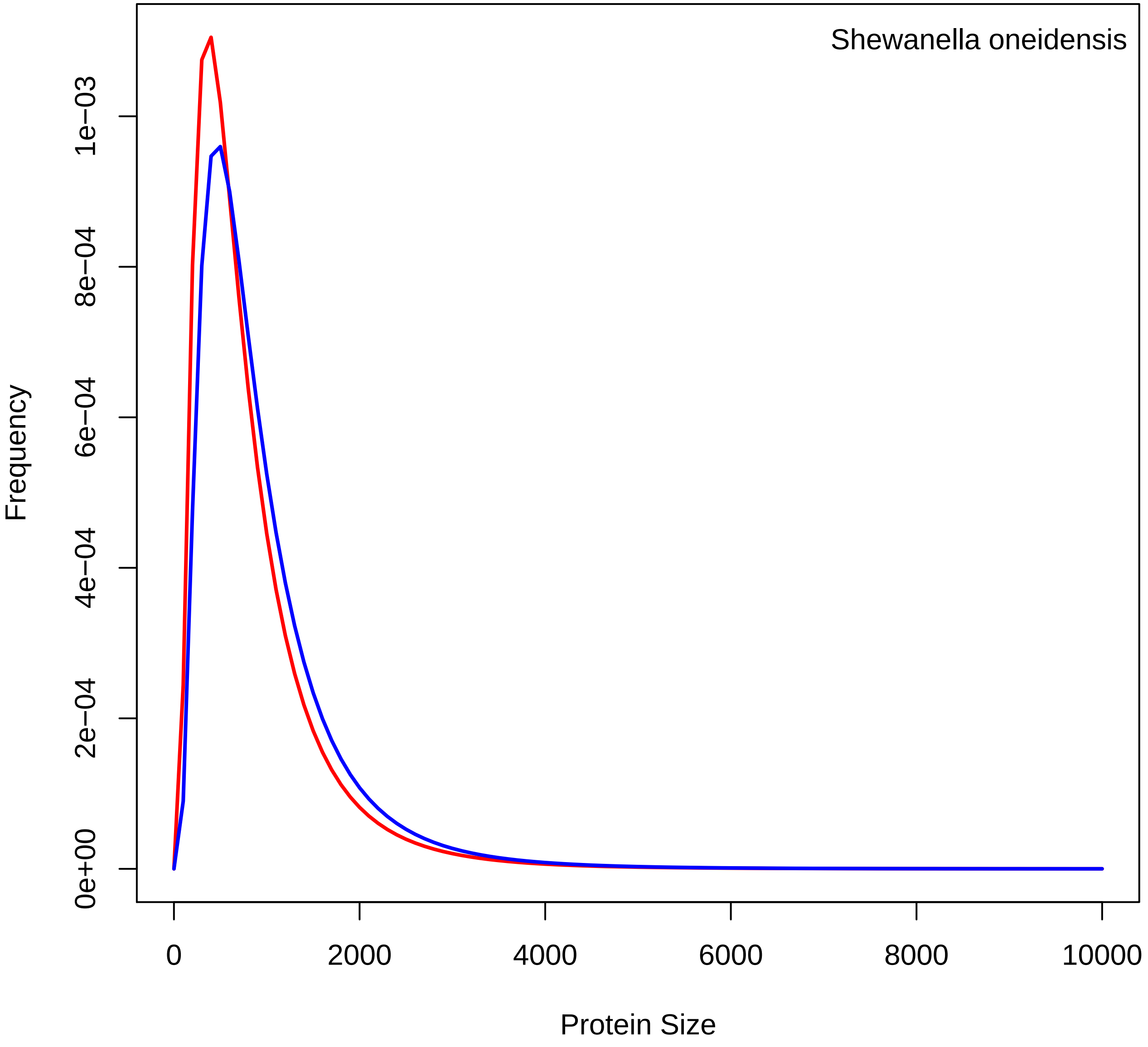

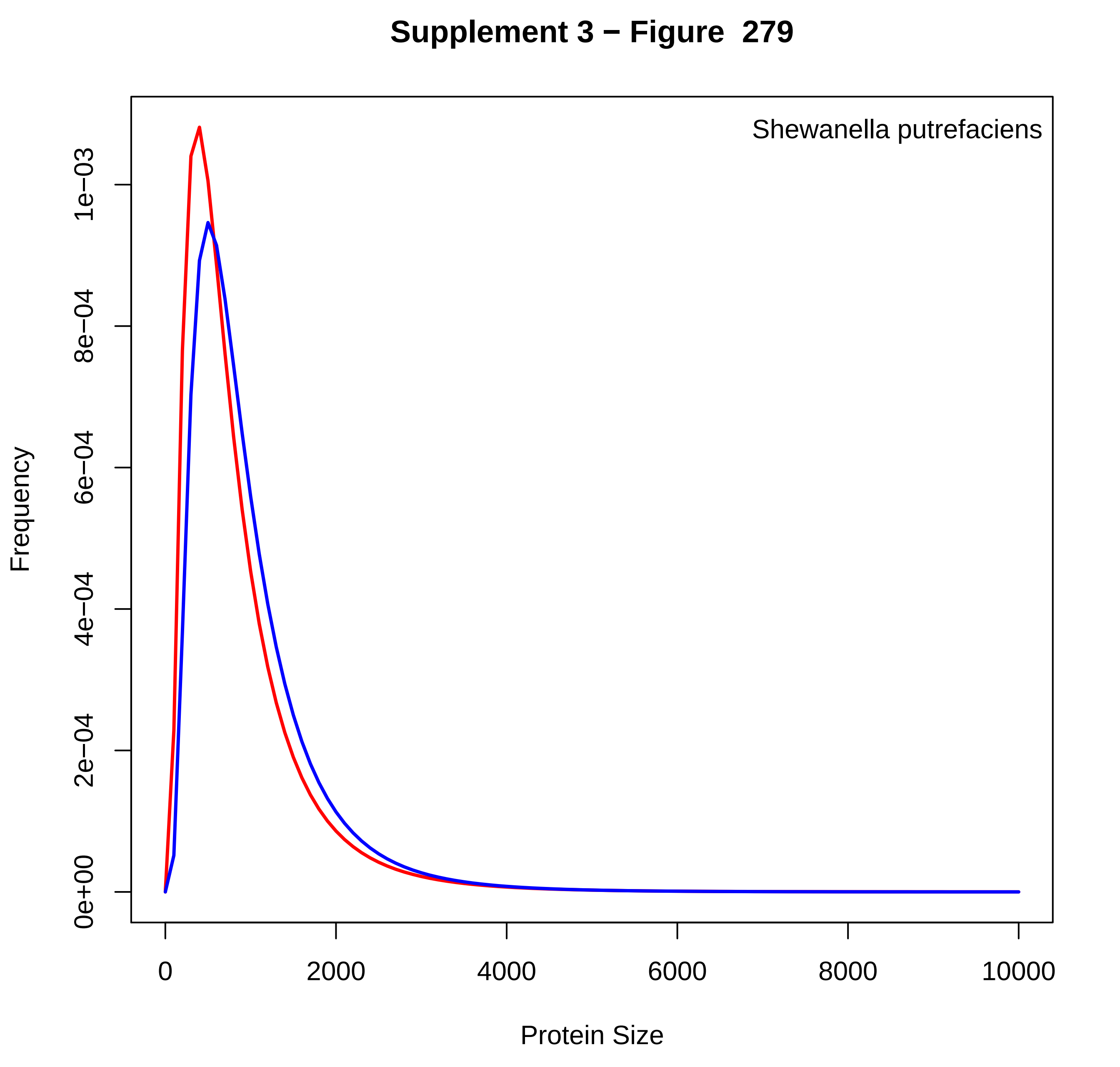

Supplement 3 − Figure 279
Shewanella putrefaciens
Frequency
0e+00
2e−04
4e−04
6e−04
8e−04
1e−03
0
2000
4000
6000
8000
10000
Protein Size

Supplement 3 − Figure 280

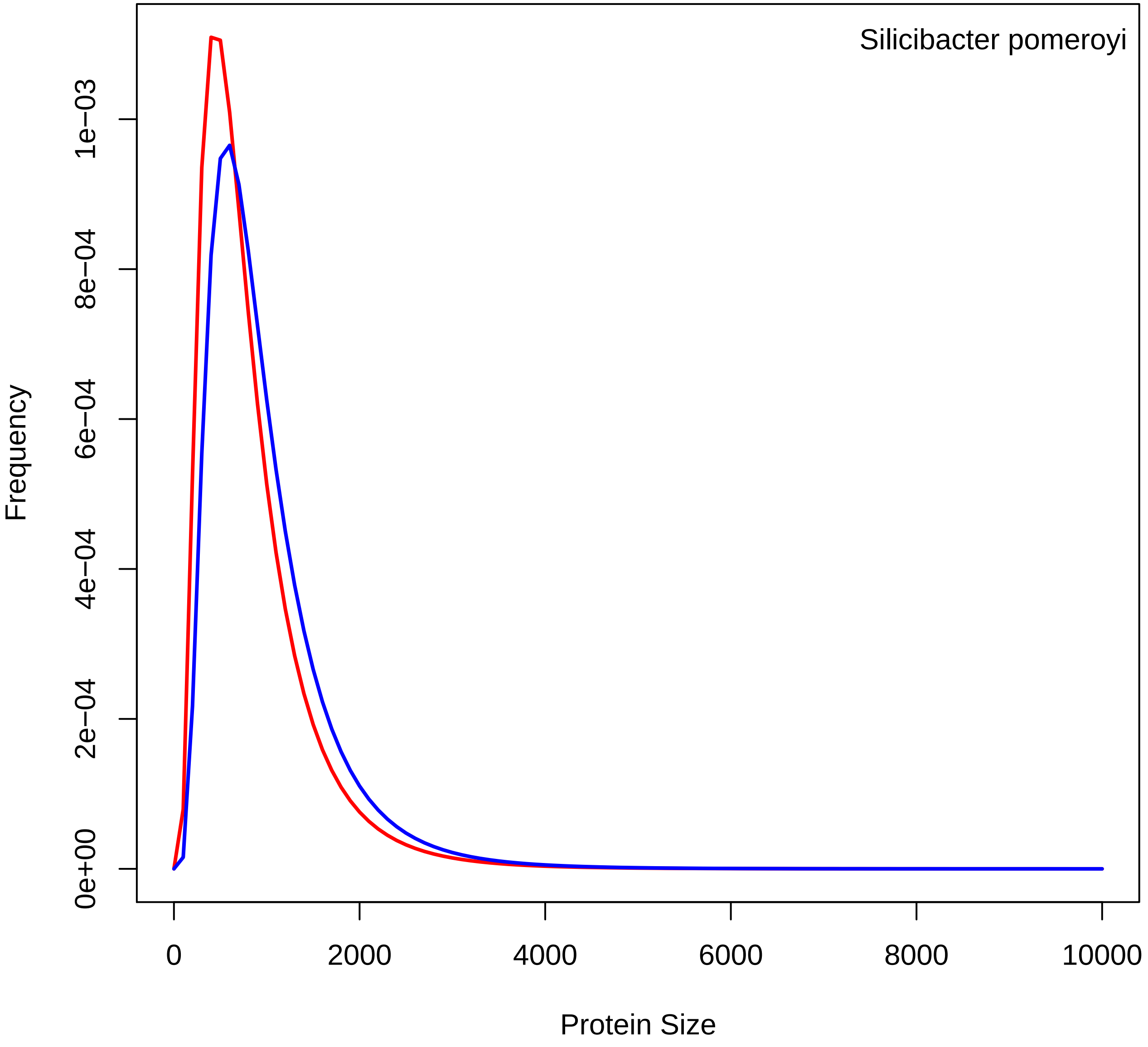

Supplement 3 − Figure 281

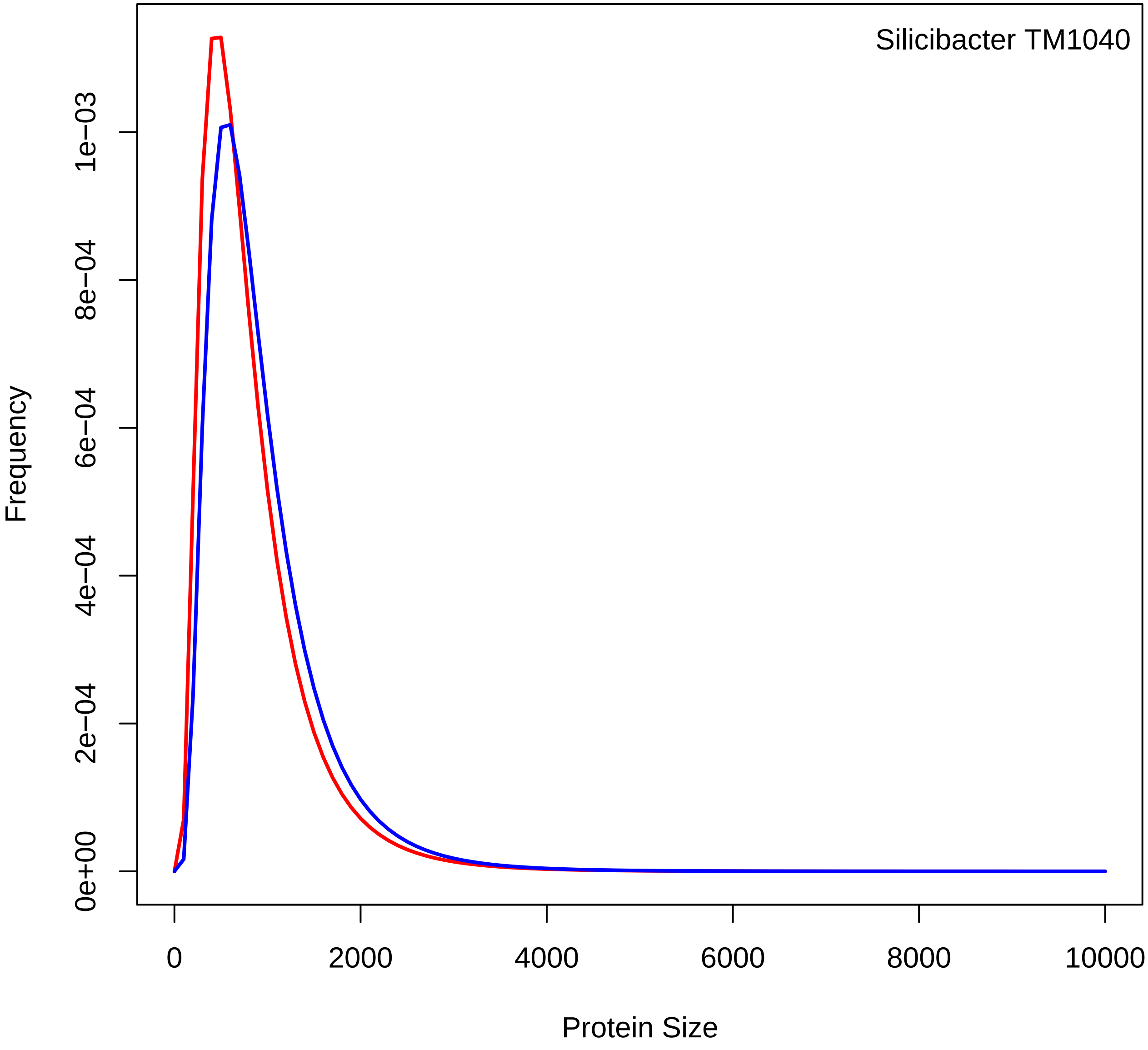

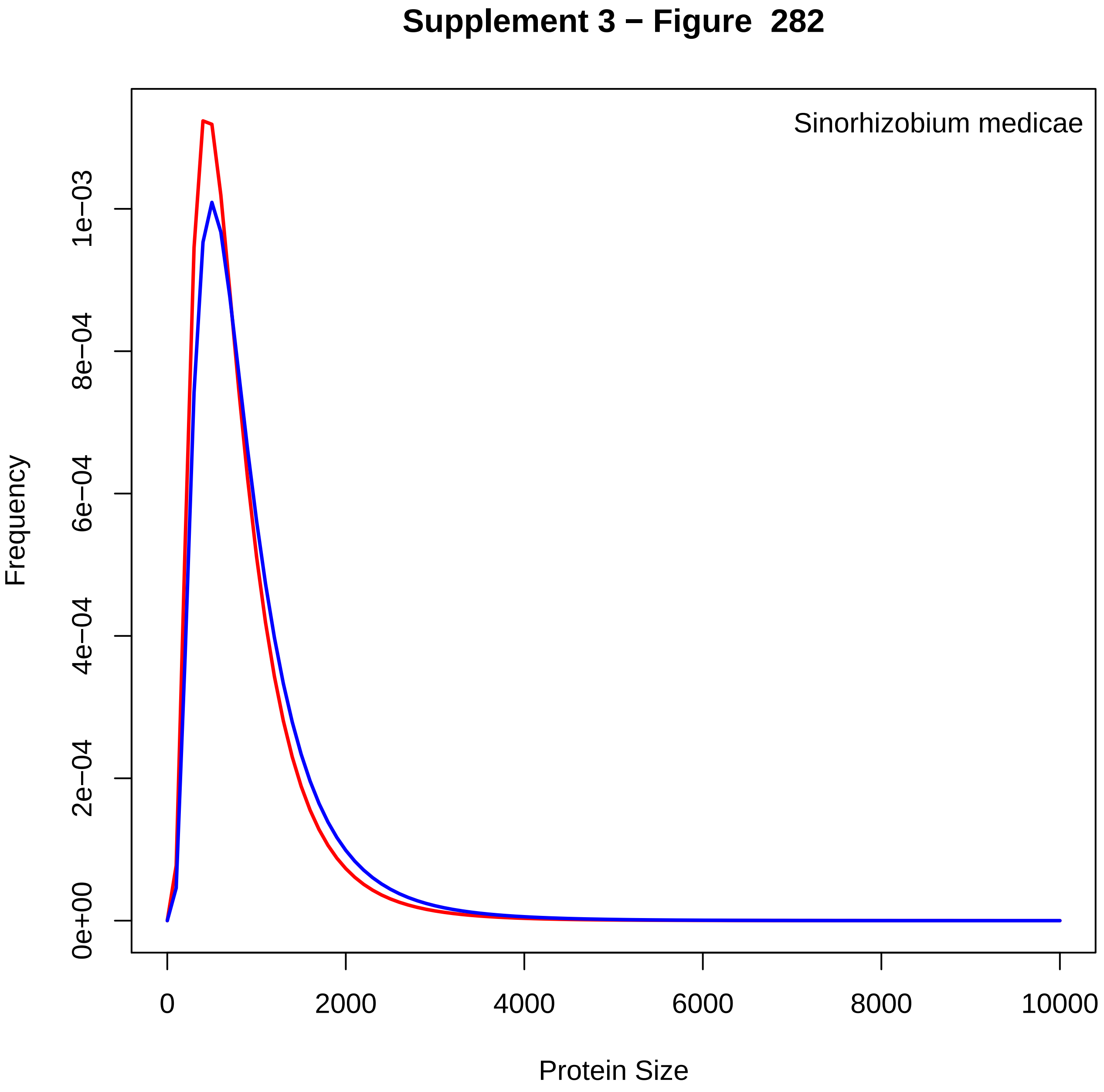
Supplement 3 − Figure 282
Sinorhizobium medicae
Frequency
0e+00
2e−04
4e−04
6e−04
8e−04
1e−03
0
2000
4000
6000
8000
10000
Protein Size

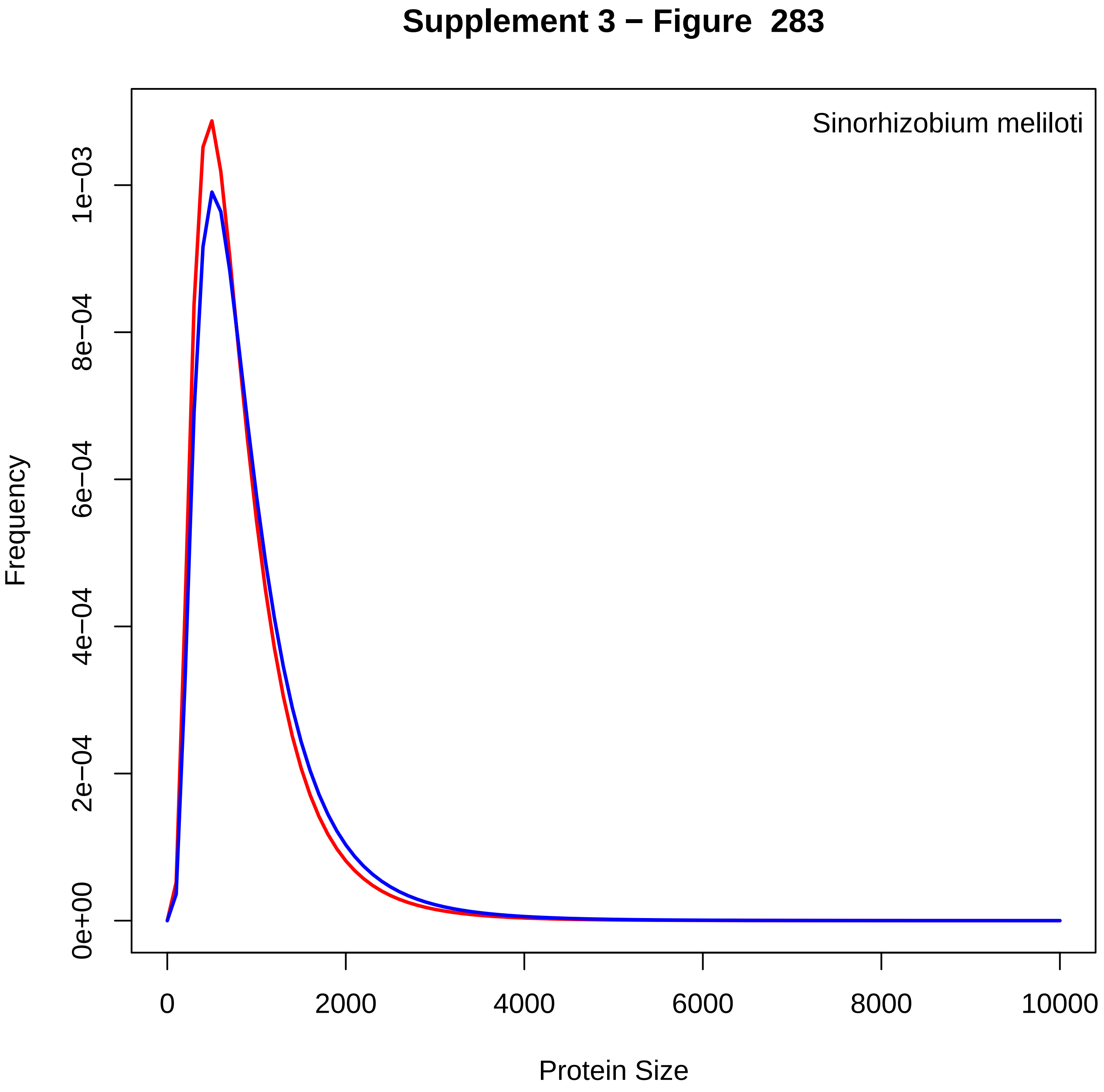

Supplement 3 − Figure 283
Sinorhizobium meliloti
Frequency
0e+00
2e−04
4e−04
6e−04
8e−04
1e−03
0
2000
4000
6000
8000
10000
Protein Size

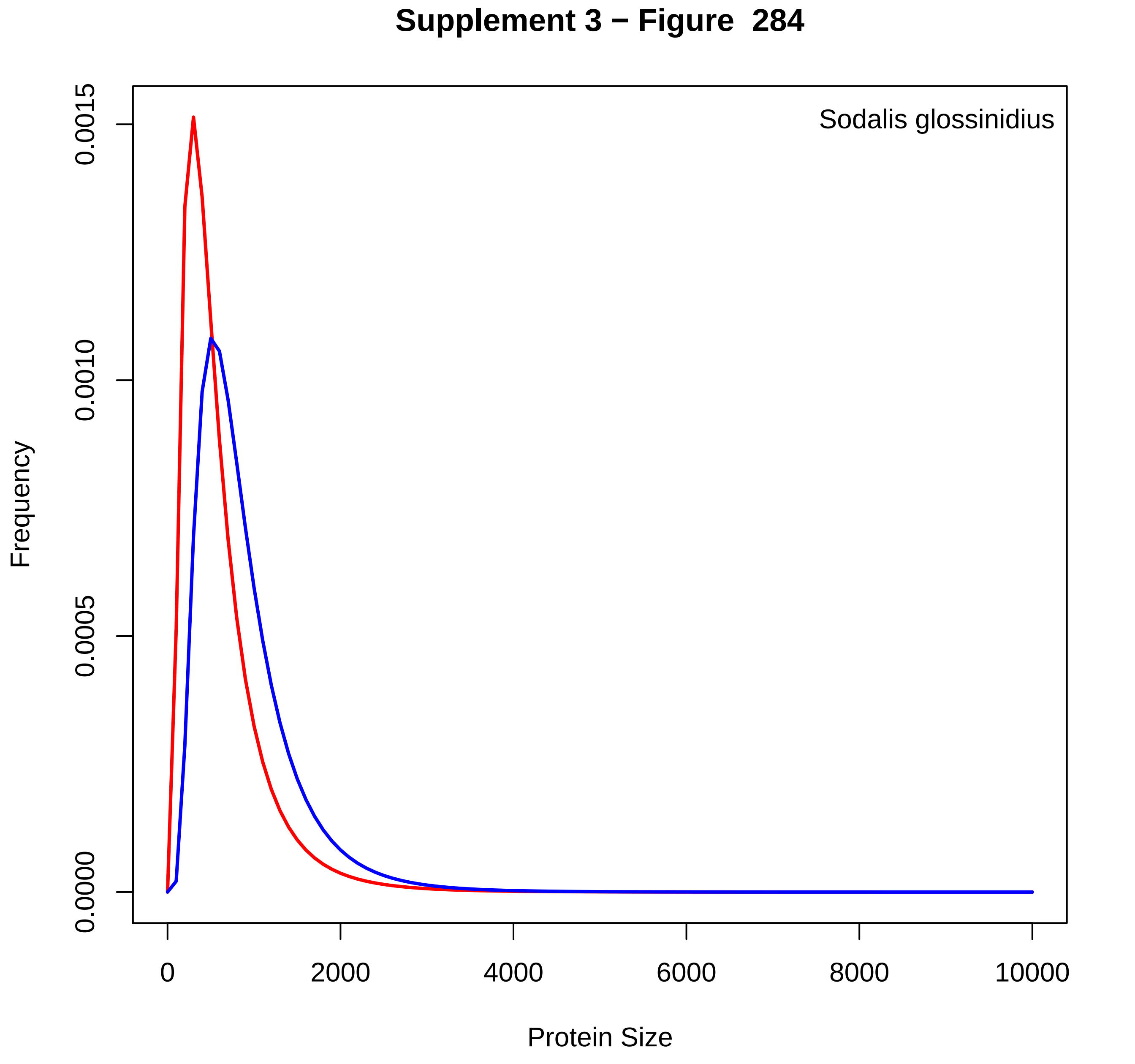

Supplement 3 − Figure 284
Sodalis glossinidius
Frequency
0.0000
0.0005
0.0010
0.0015
0
2000
4000
6000
8000
10000
Protein Size

Supplement 3 − Figure 285

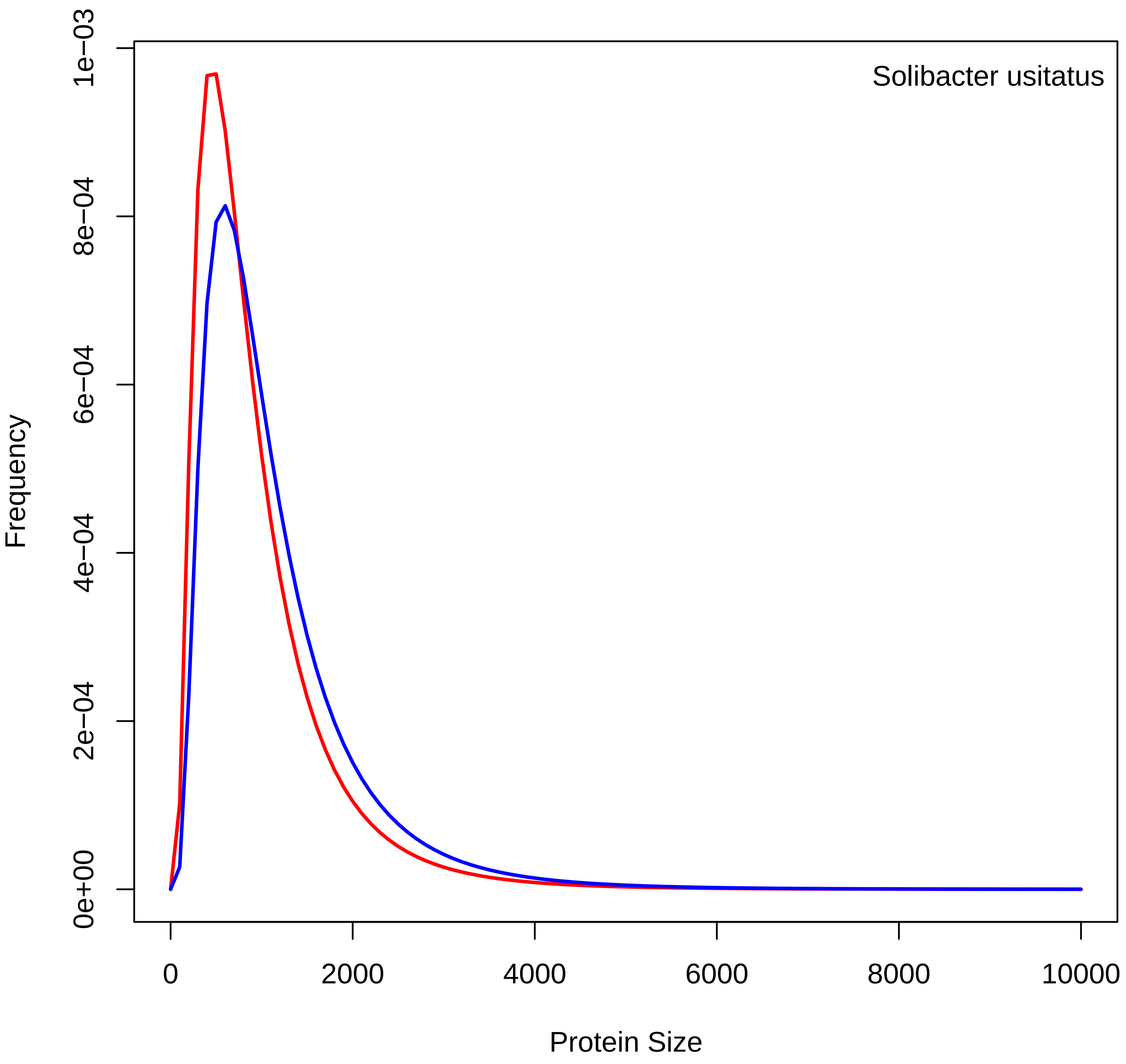

Supplement 3 − Figure 286

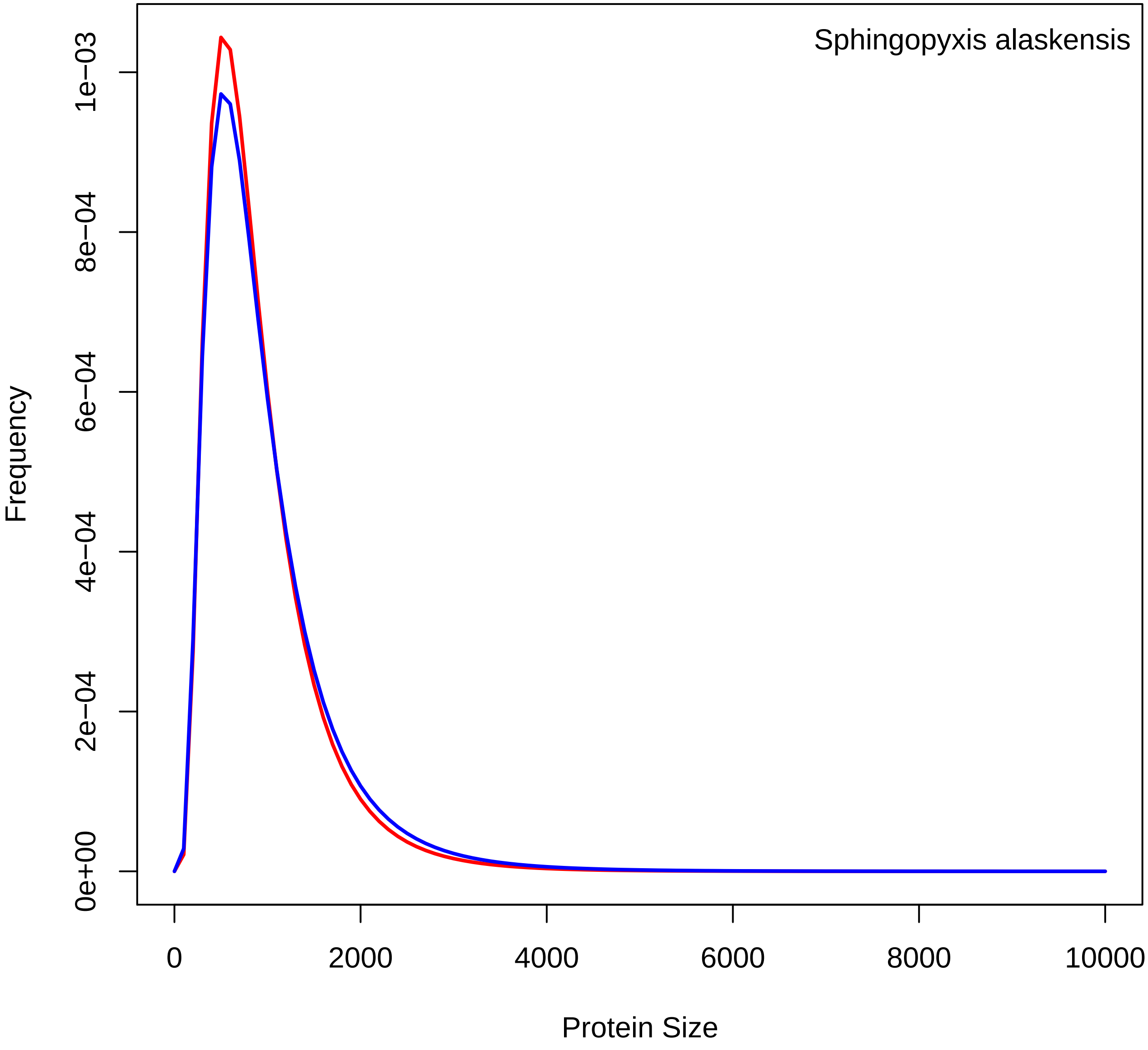

Supplement 3 − Figure 287

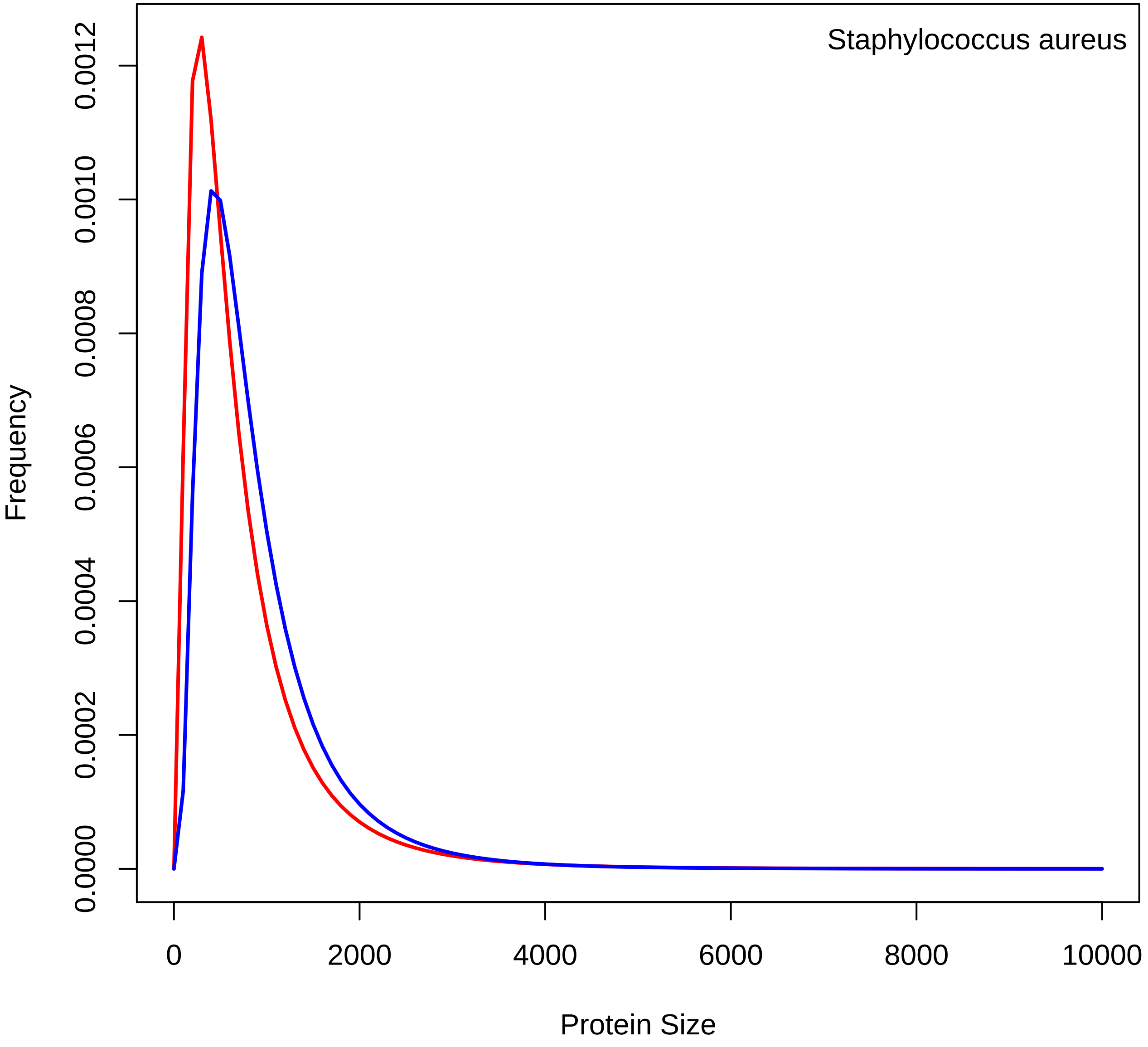

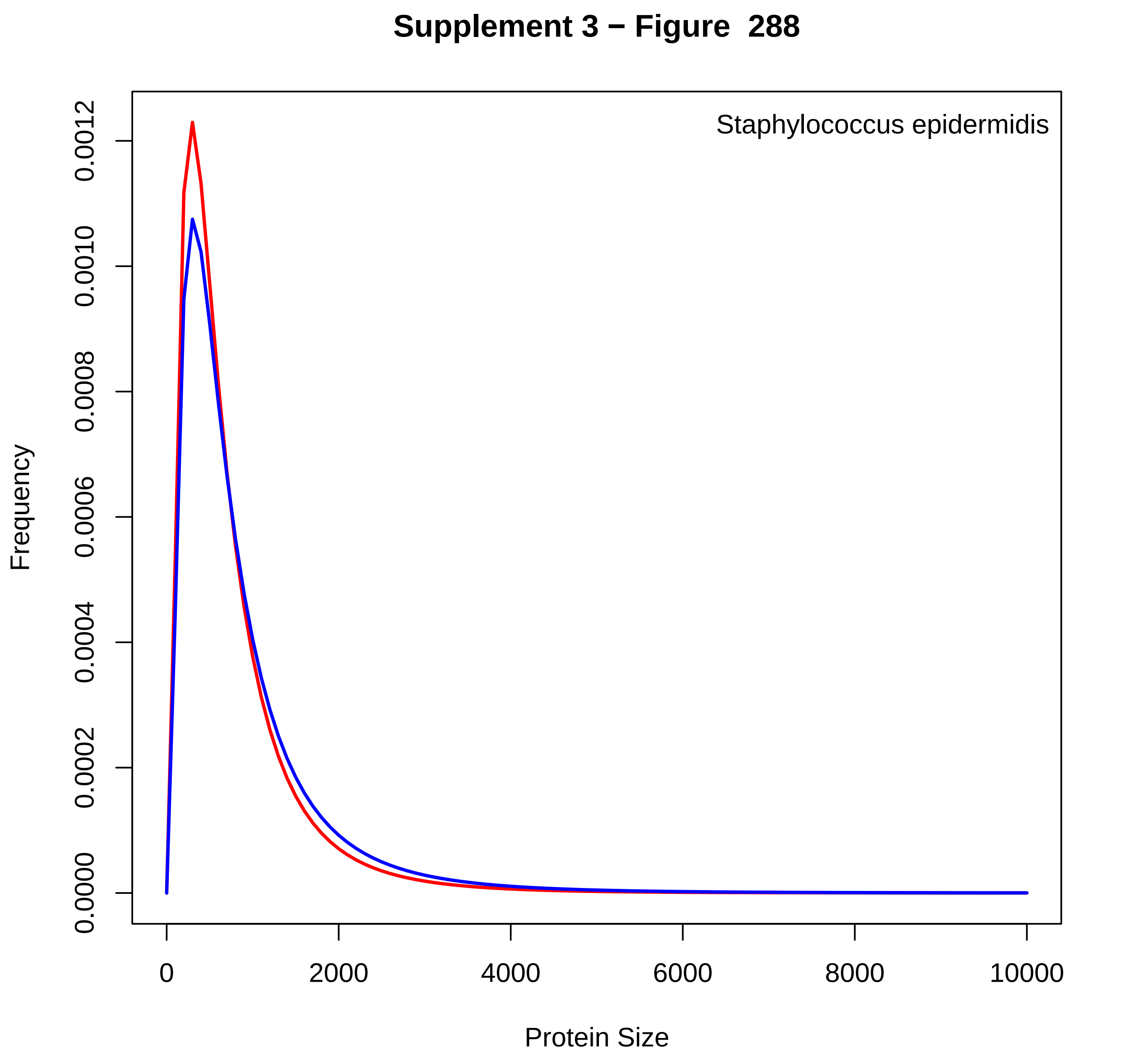
Supplement 3 − Figure 288
Staphylococcus epidermidis
Frequency
0.0000
0.0002
0.0004
0.0006
0.0008
0.0010
0.0012
0
2000
4000
6000
8000
10000
Protein Size

Supplement 3 − Figure 289

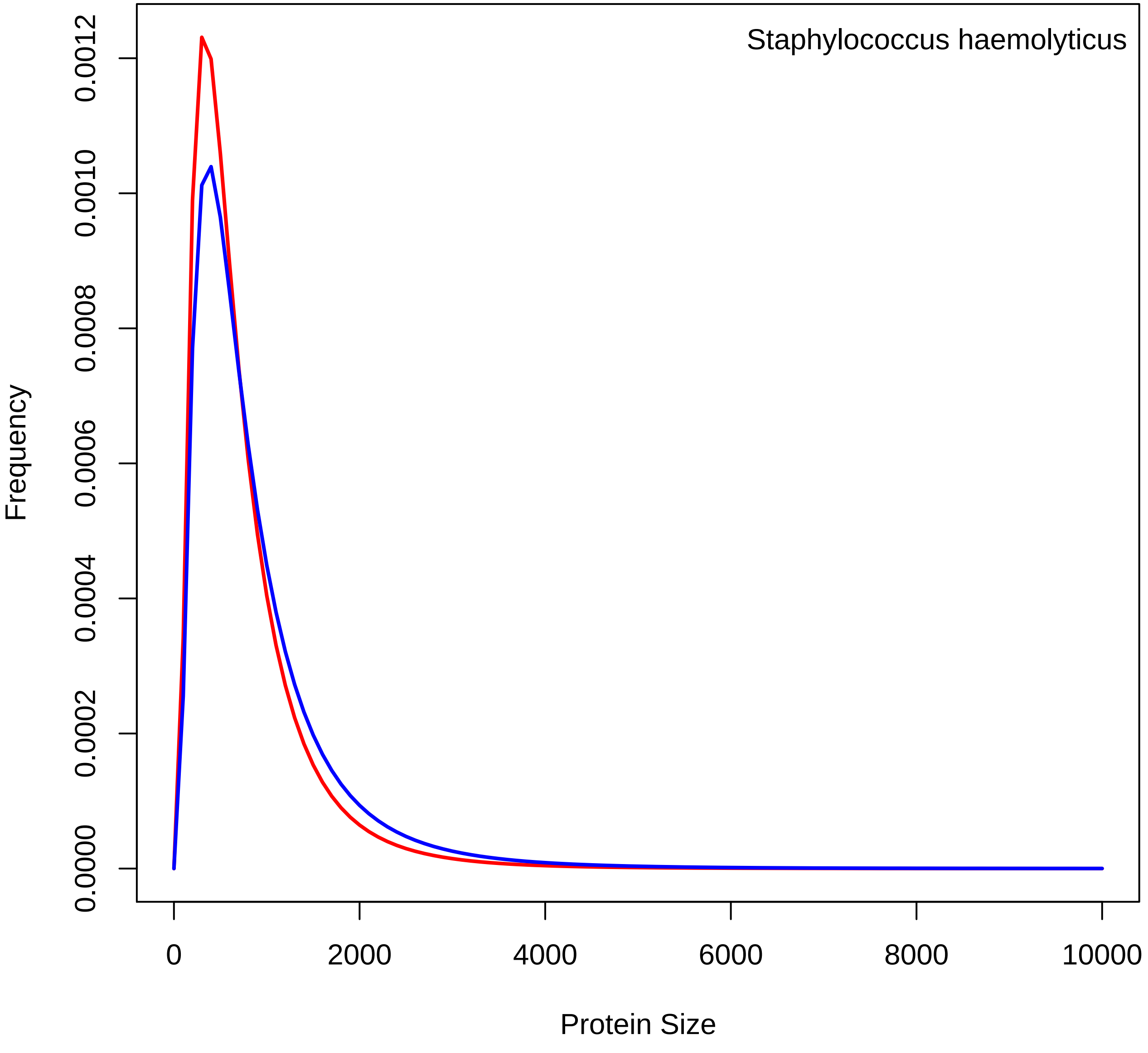

Supplement 3 − Figure 290

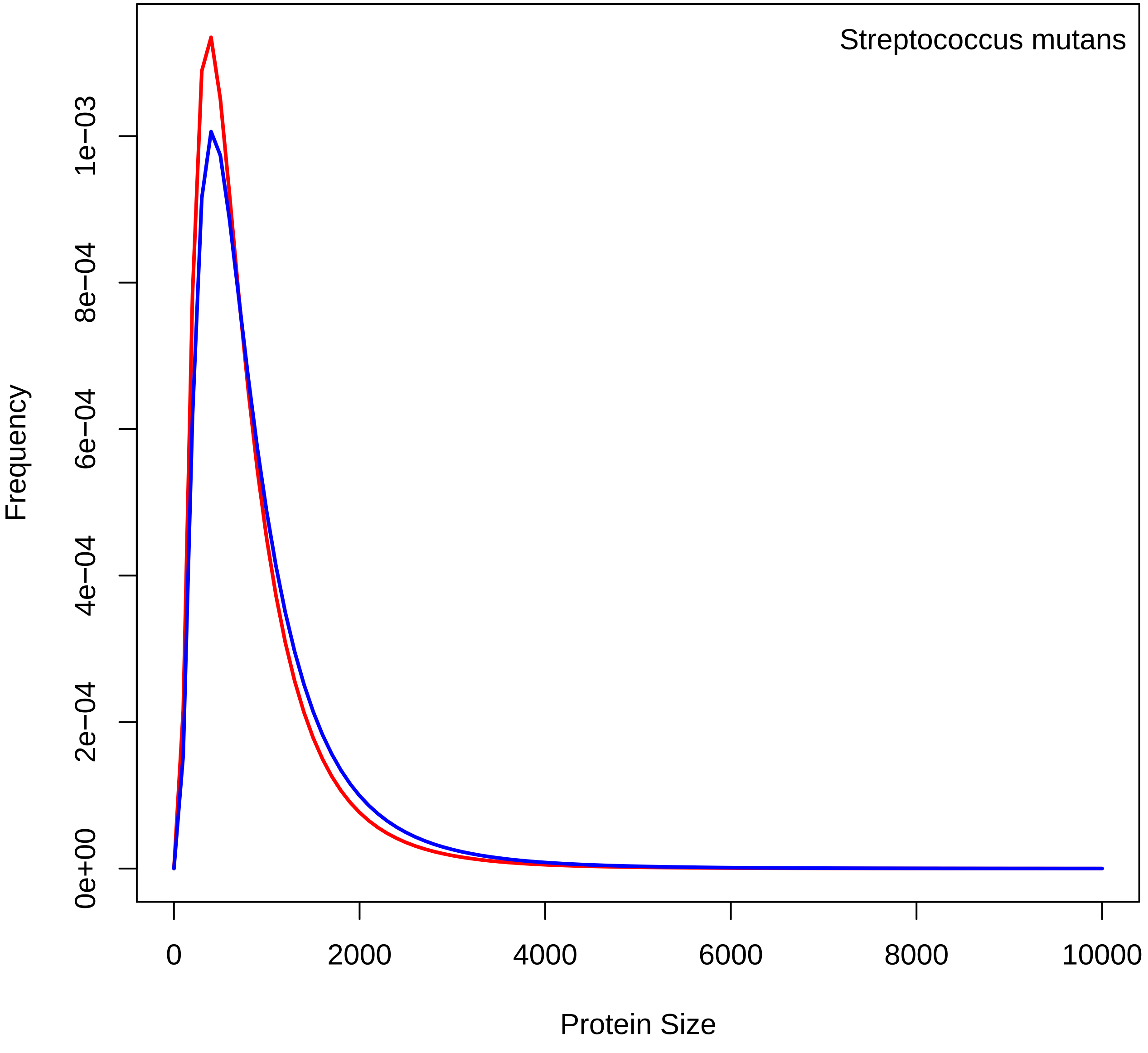

Supplement 3 − Figure 291

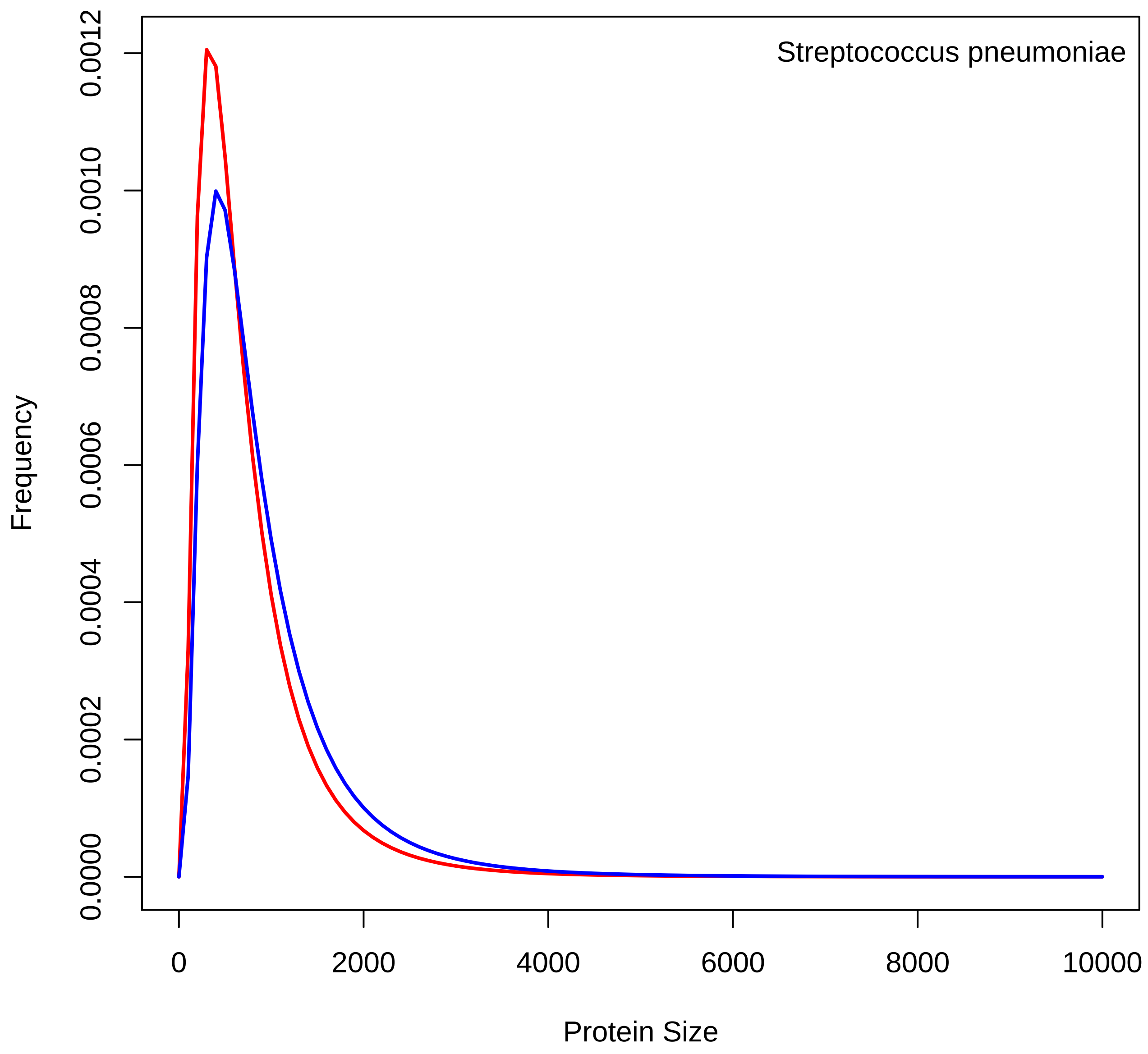

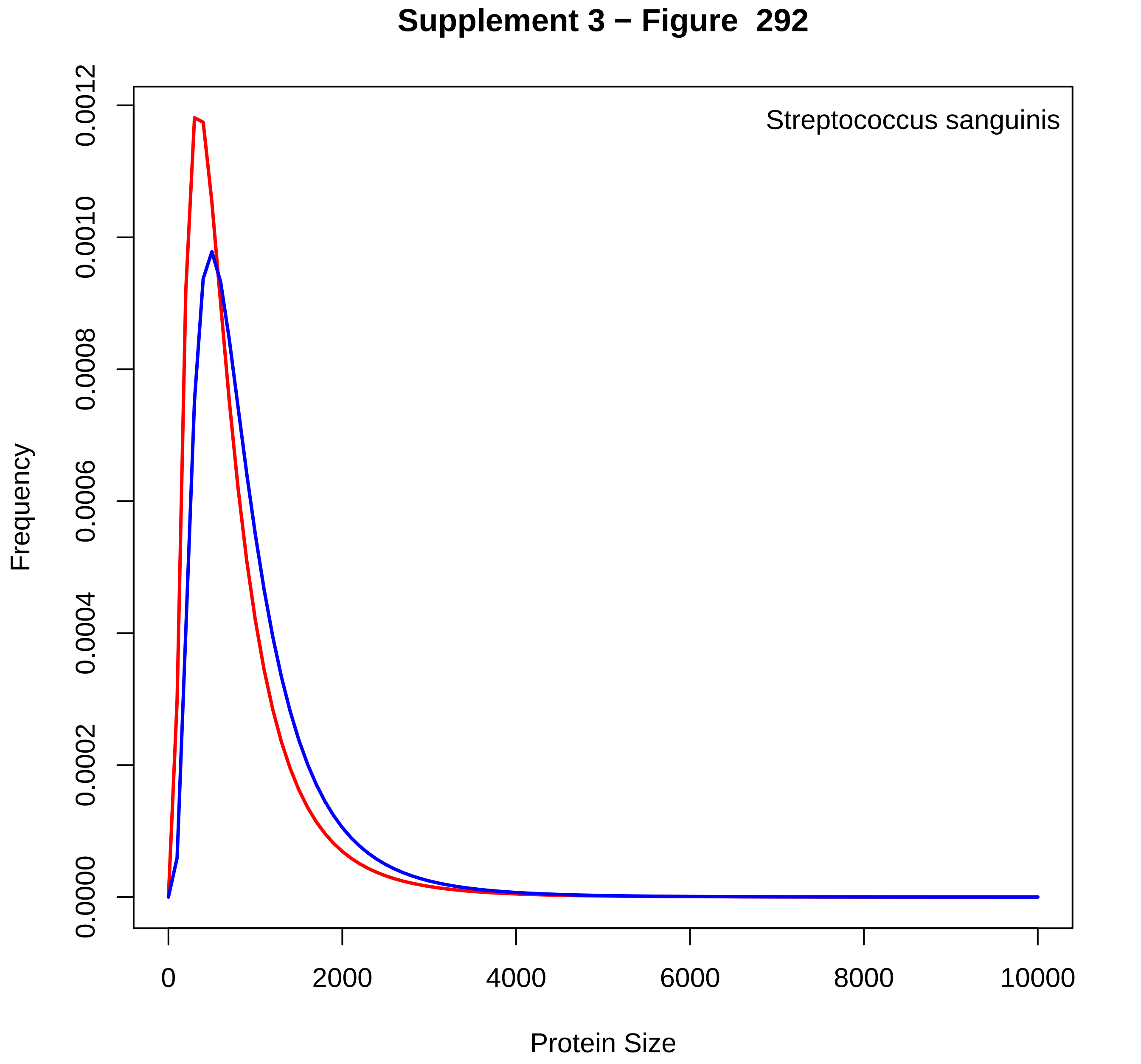
Supplement 3 − Figure 292
Streptococcus sanguinis
Frequency
0.0000
0.0002
0.0004
0.0006
0.0008
0.0010
0.0012
0
2000
4000
6000
8000
10000
Protein Size

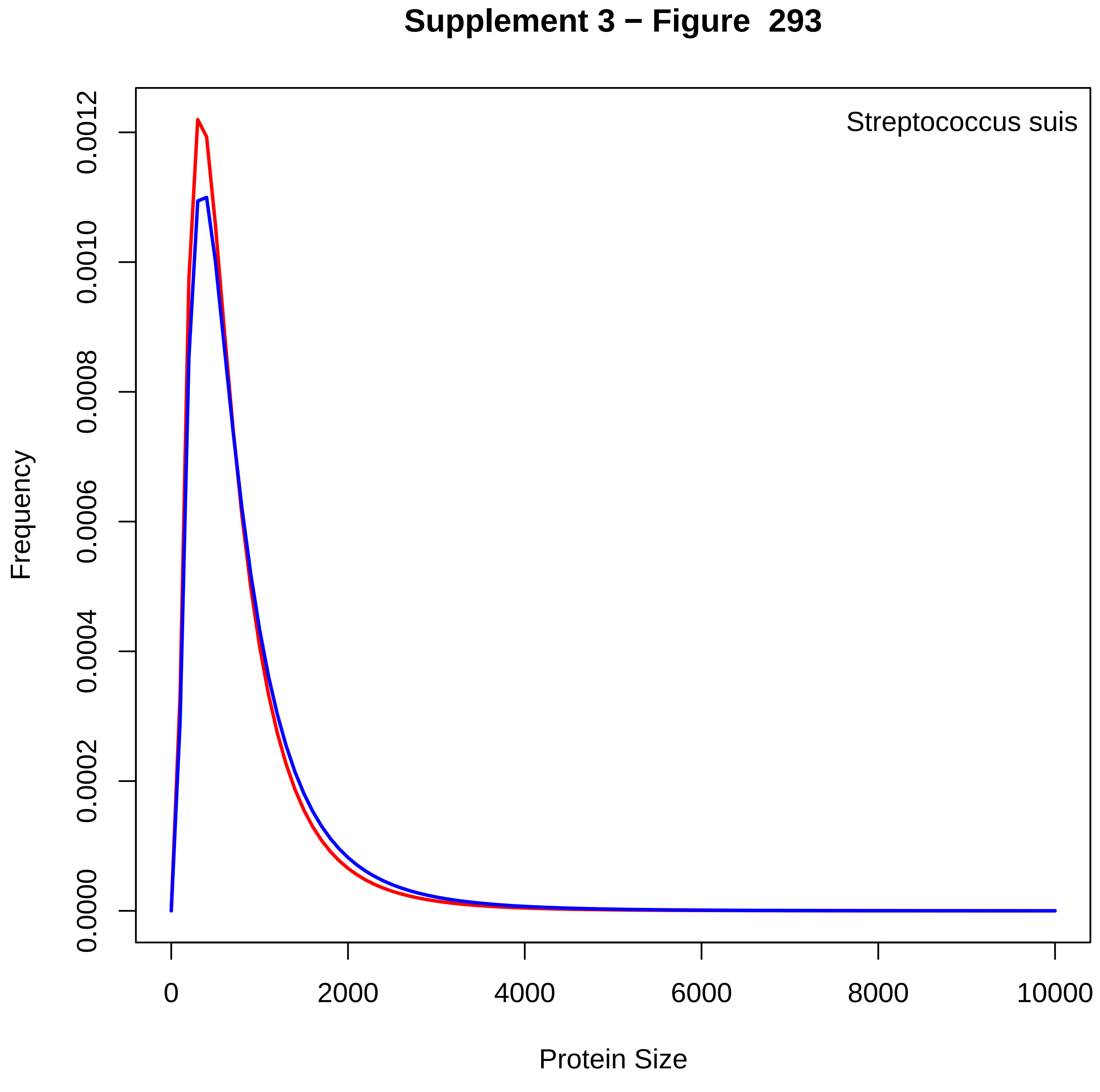
Supplement 3 − Figure 293
Streptococcus suis
Frequency
0.0000
0.0002
0.0004
0.0006
0.0008
0.0010
0.0012
0
2000
4000
6000
8000
10000
Protein Size

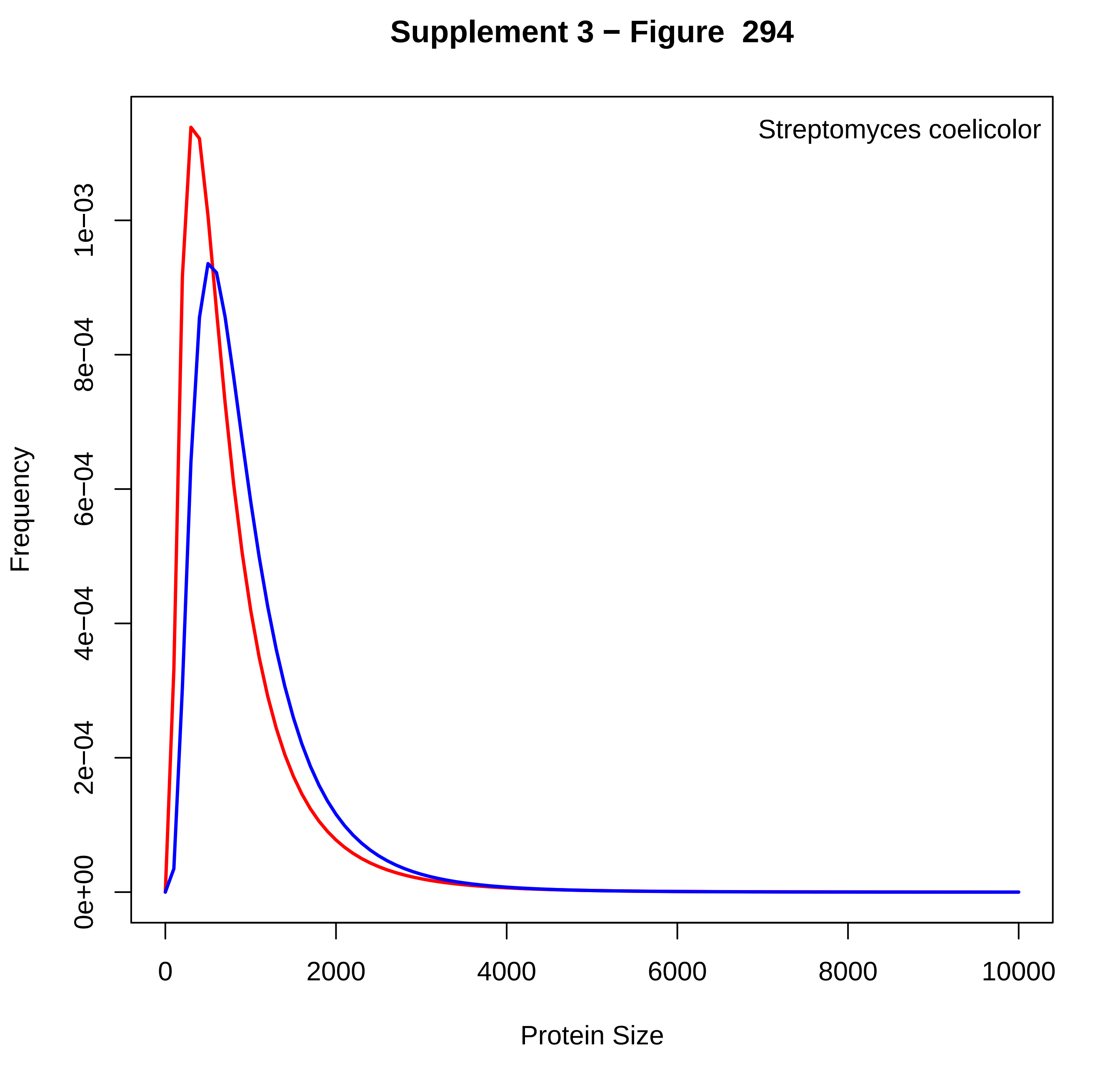
Supplement 3 − Figure 294
Streptomyces coelicolor
Frequency
0e+00
2e−04
4e−04
6e−04
8e−04
1e−03
0
2000
4000
6000
8000
10000
Protein Size

Supplement 3 − Figure 295

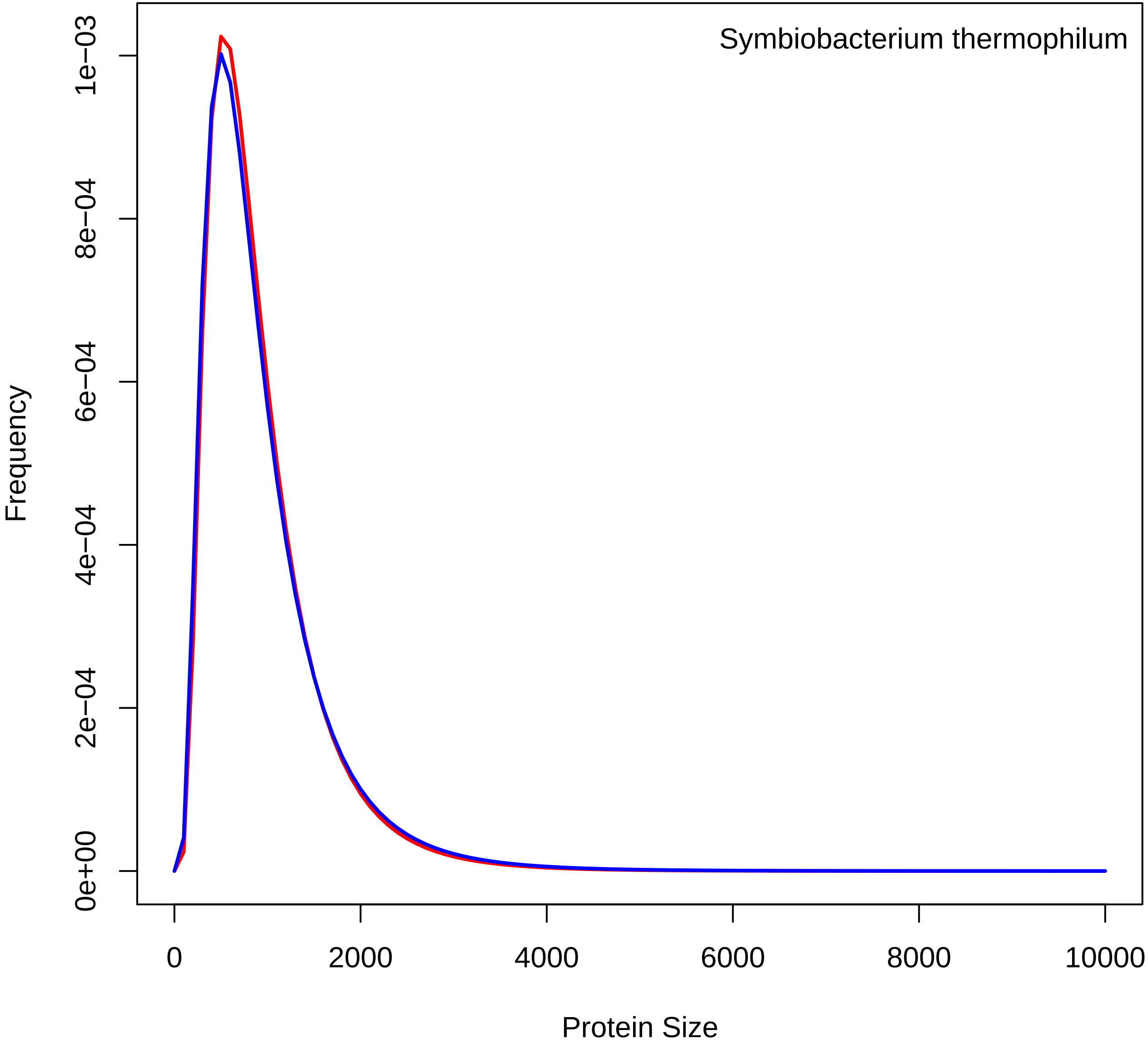

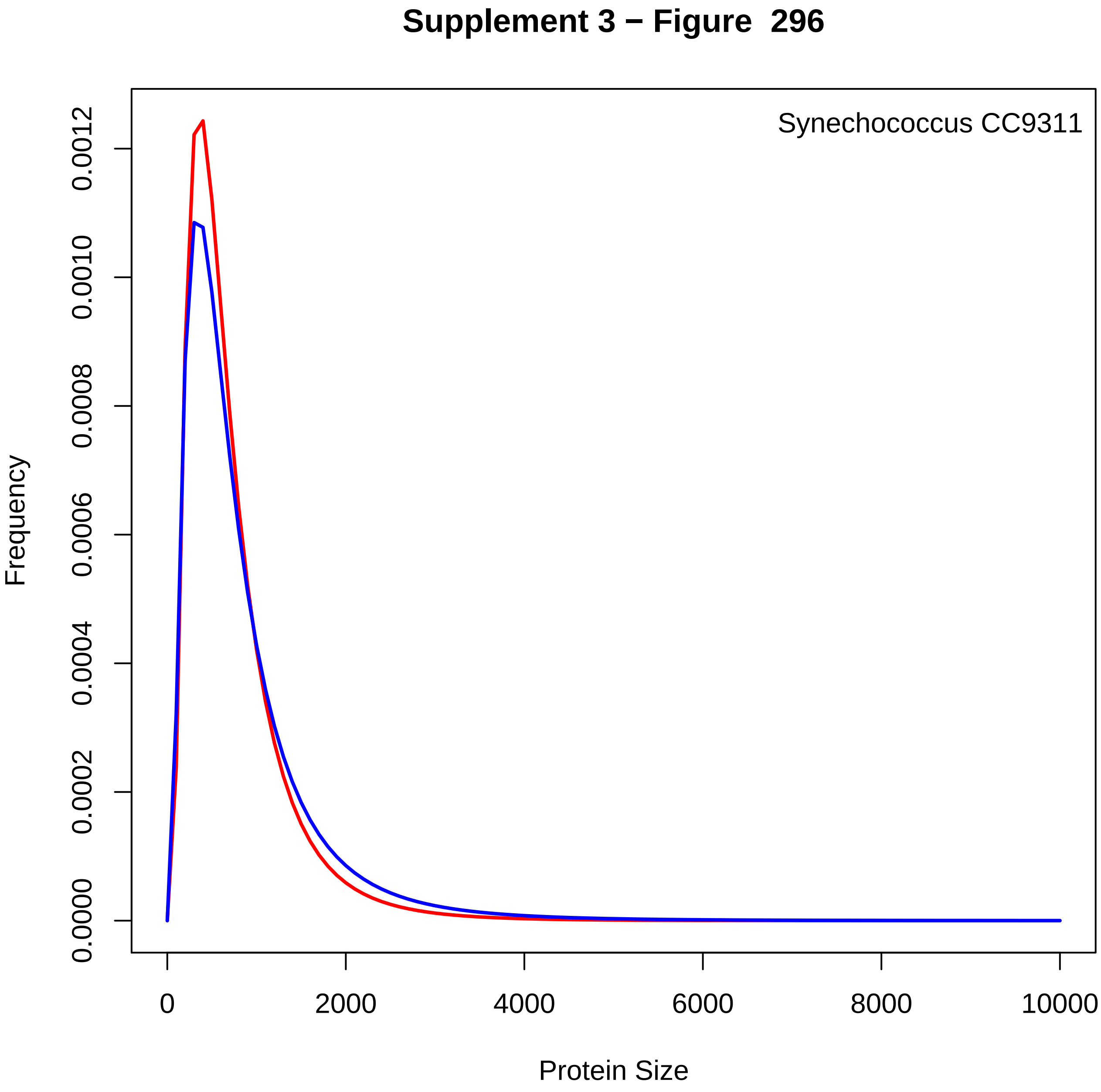
Supplement 3 − Figure 296
Synechococcus CC9311
Frequency
0.0000
0.0002
0.0004
0.0006
0.0008
0.0010
0.0012
0
2000
4000
6000
8000
10000
Protein Size

Supplement 3 − Figure 297

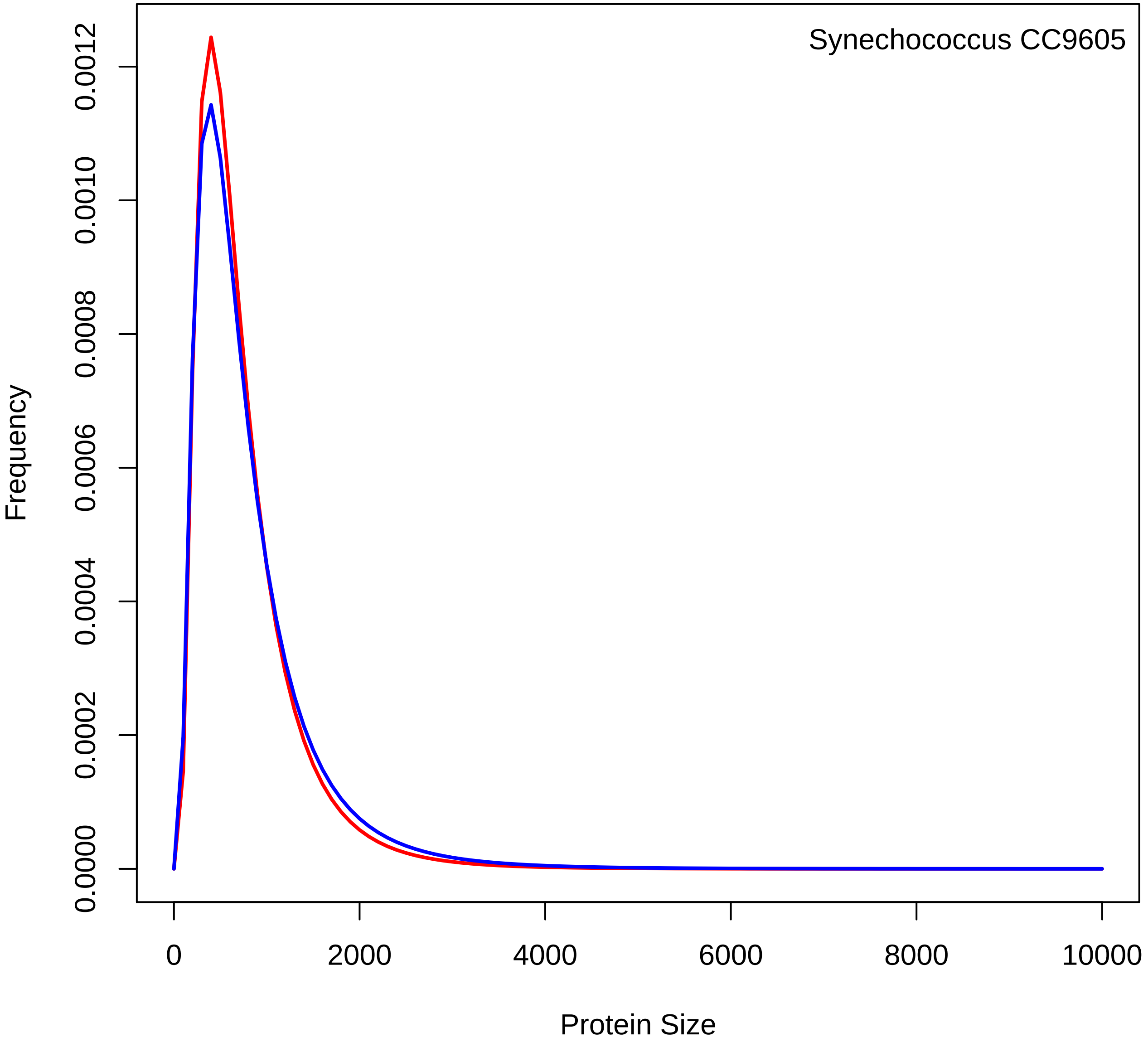

Supplement 3 − Figure 298

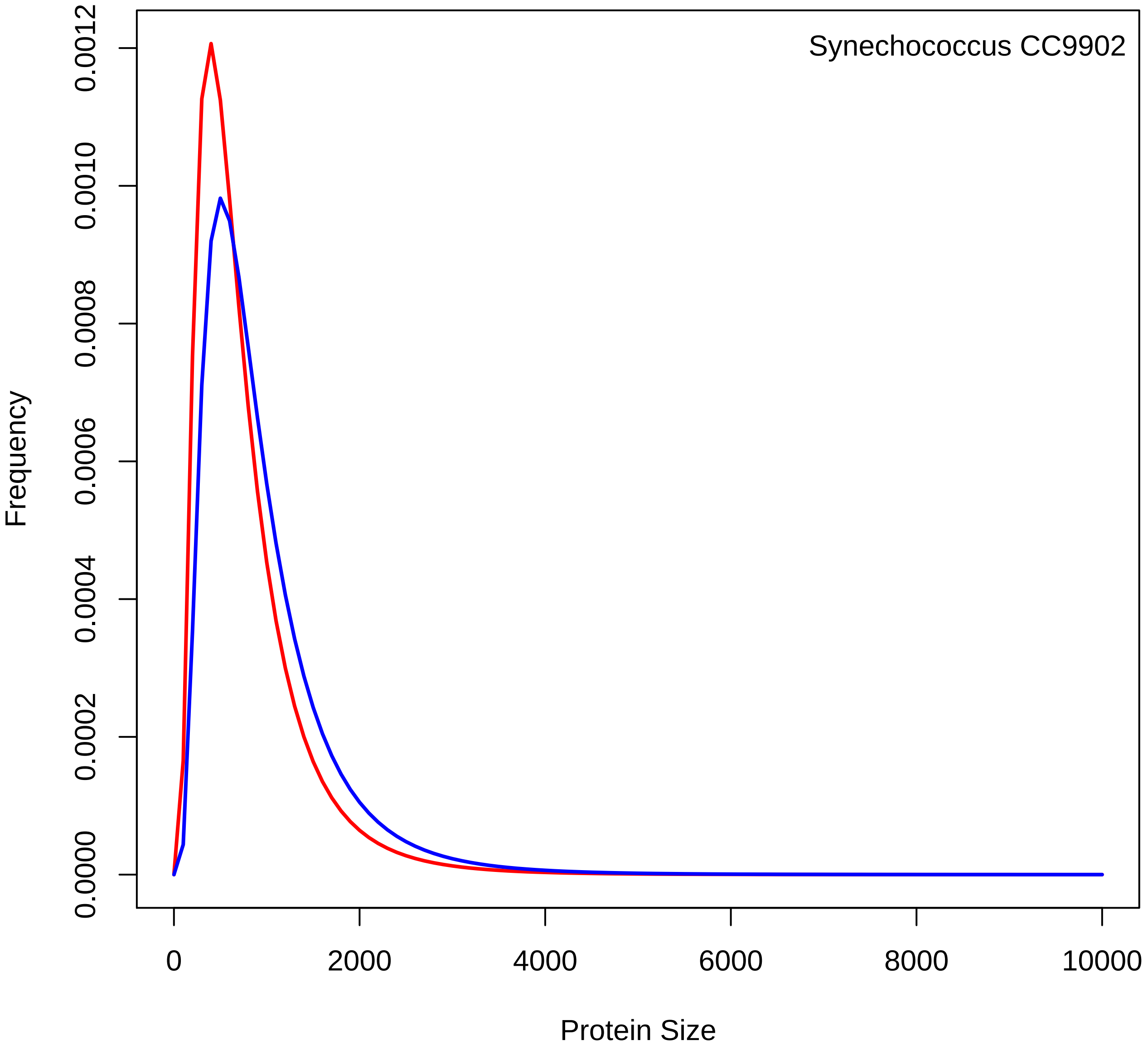

Supplement 3 − Figure 299

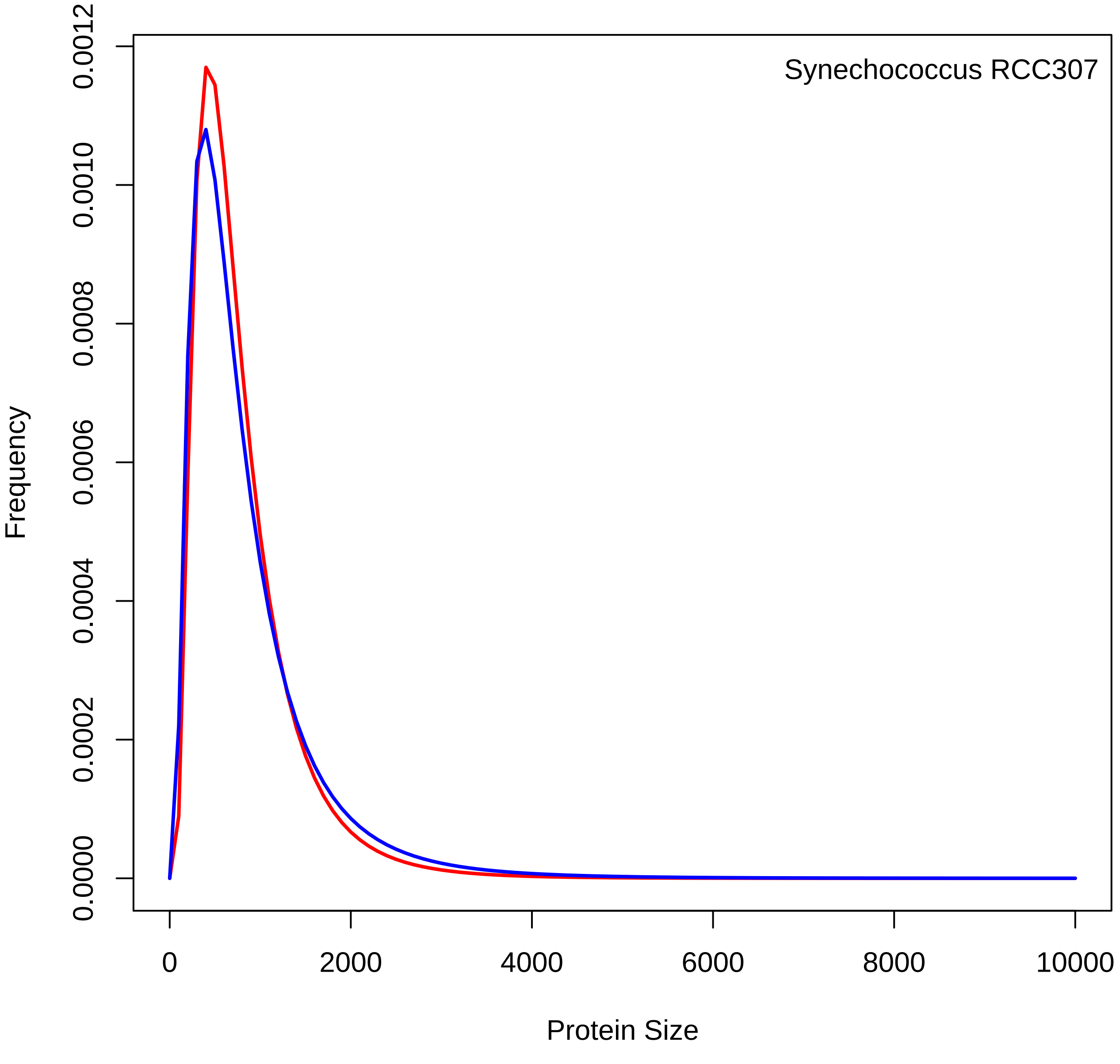

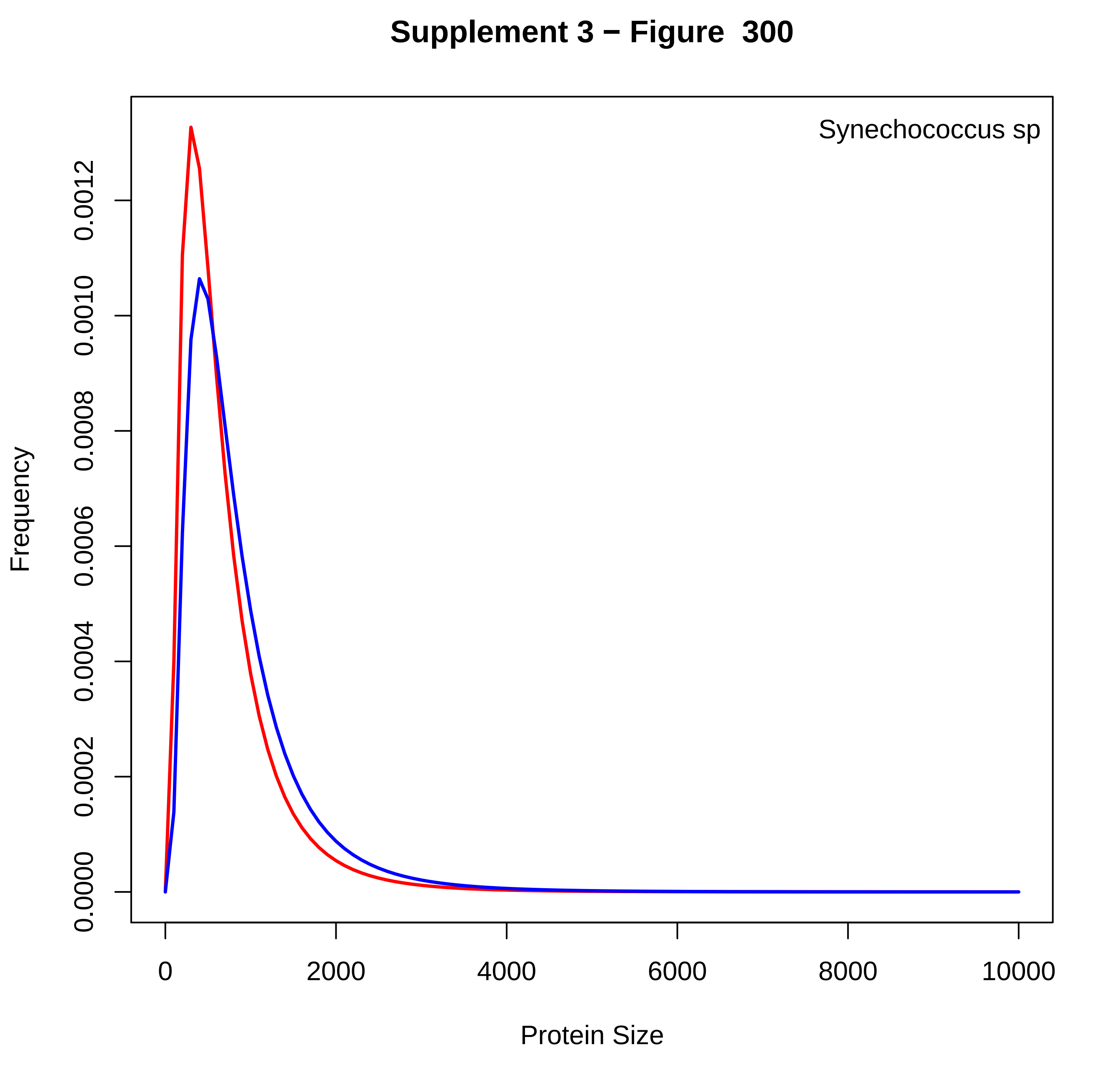
Supplement 3 − Figure 300
Synechococcus sp
Frequency
0.0000
0.0002
0.0004
0.0006
0.0008
0.0010
0.0012
0
2000
4000
6000
8000
10000
Protein Size

Supplement 3 − Figure 301

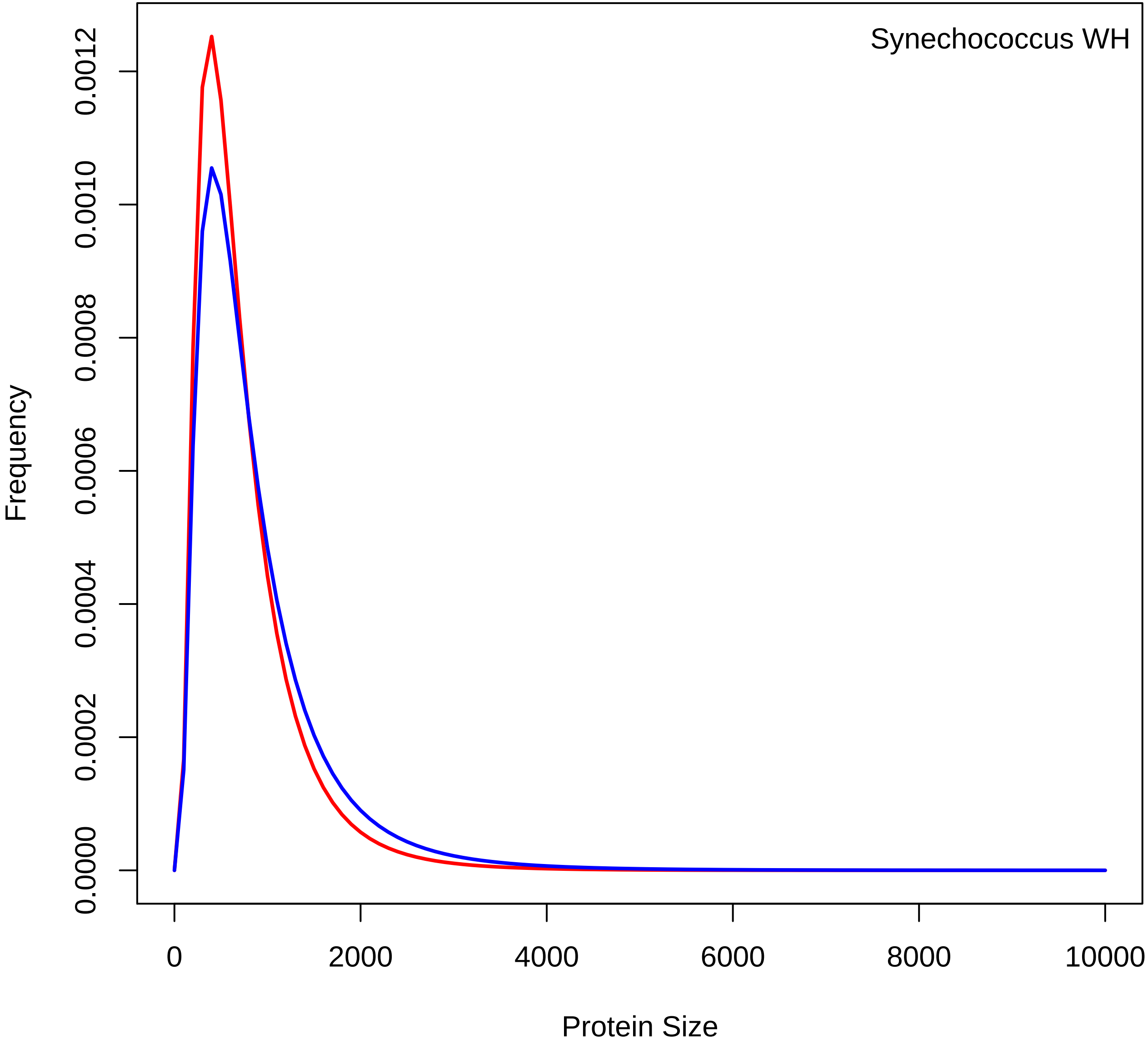

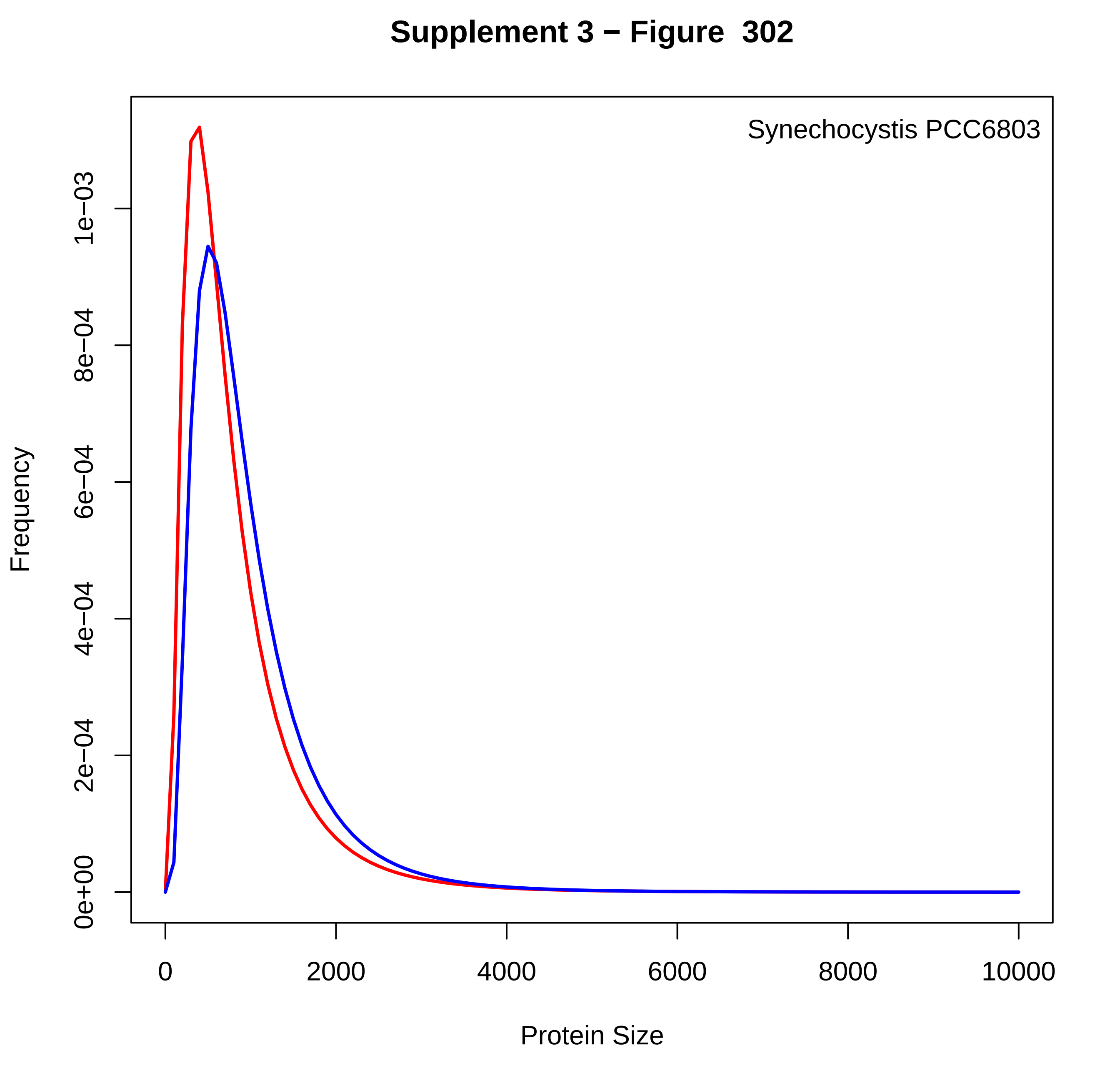
Supplement 3 − Figure 302
Synechocystis PCC6803
Frequency
0e+00
2e−04
4e−04
6e−04
8e−04
1e−03
0
2000
4000
6000
8000
10000
Protein Size

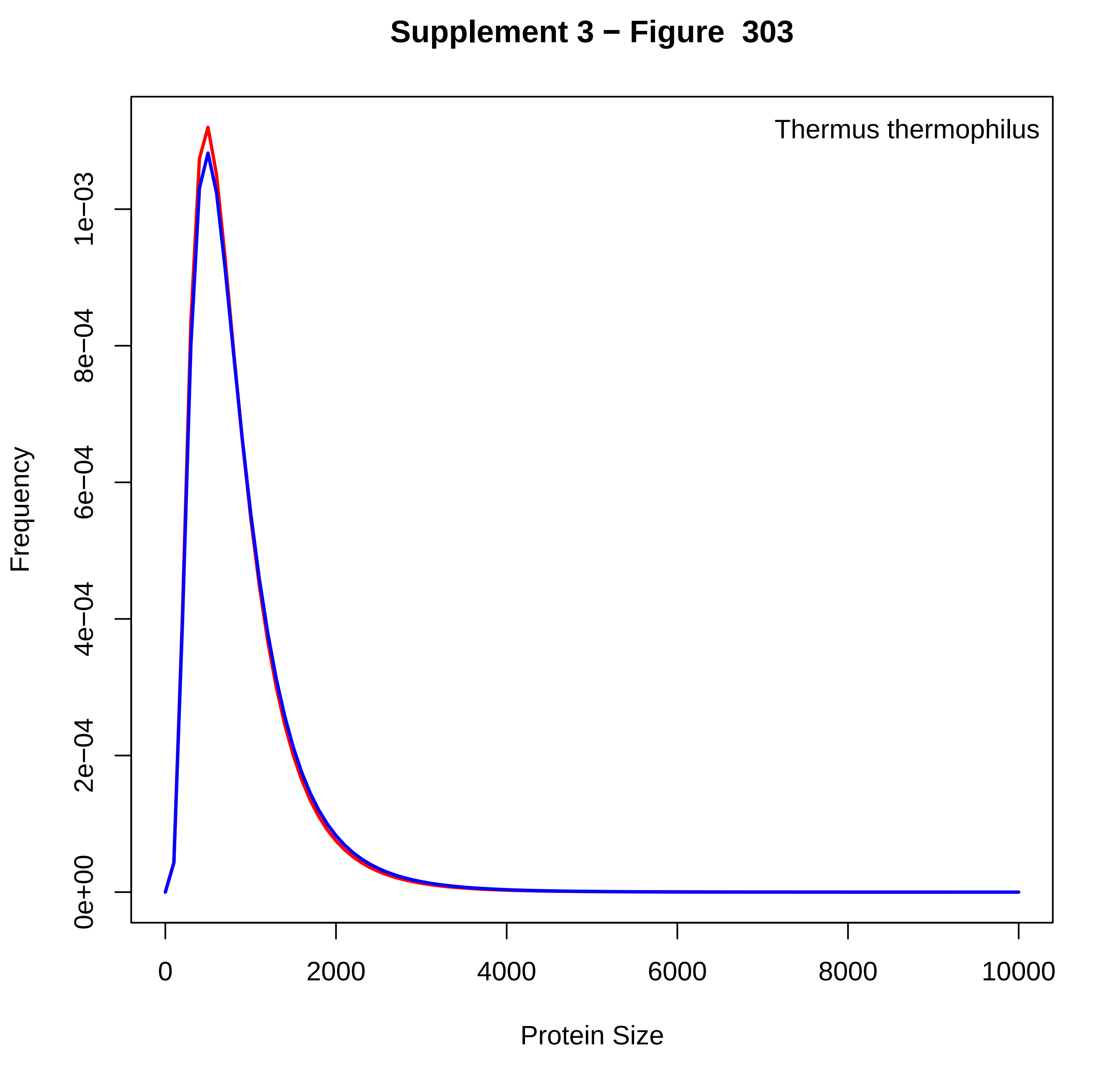
Supplement 3 − Figure 303
Thermus thermophilus
Frequency
0e+00
2e−04
4e−04
6e−04
8e−04
1e−03
0
2000
4000
6000
8000
10000
Protein Size

Supplement 3 − Figure 304

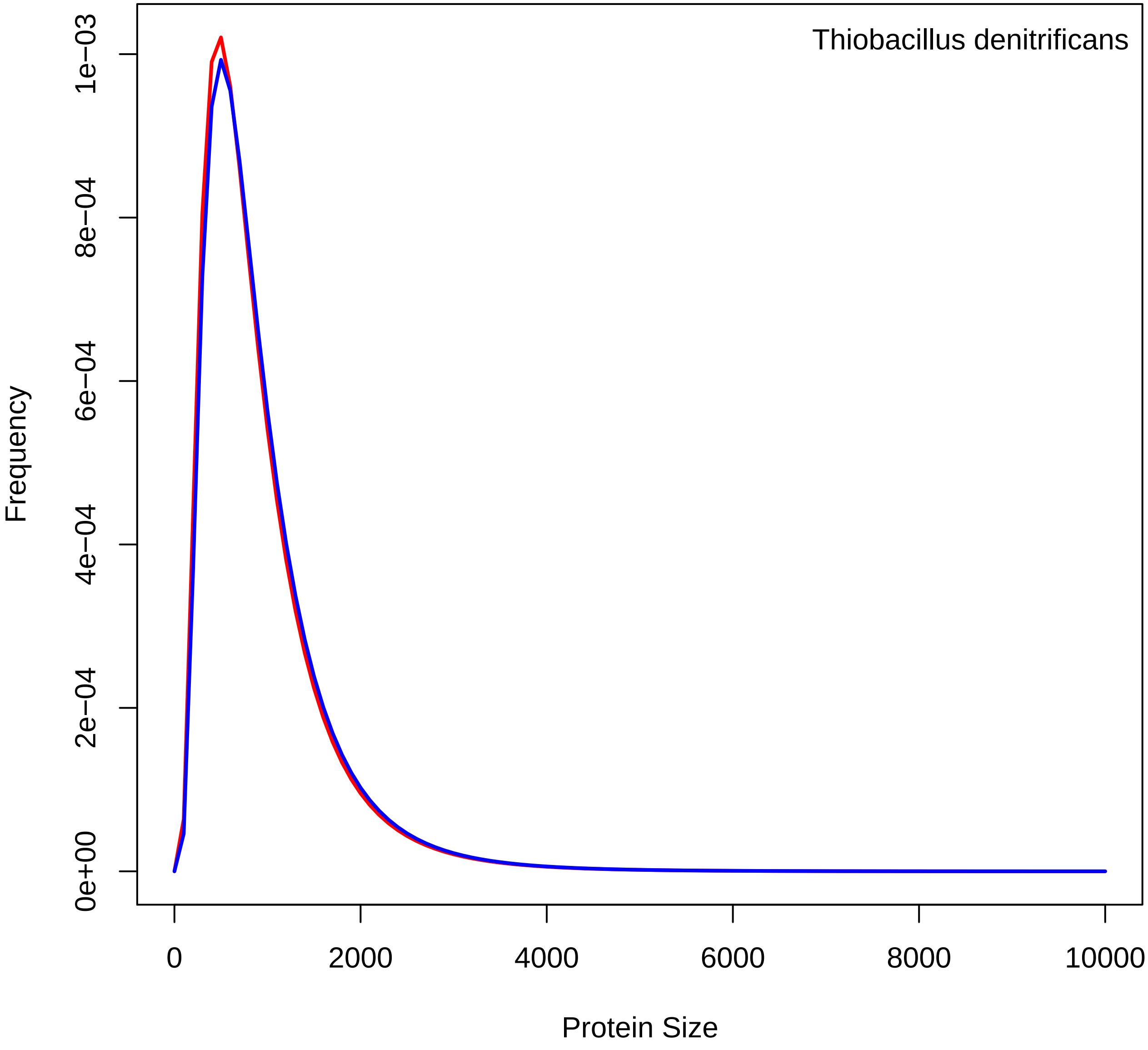

Supplement 3 − Figure 305

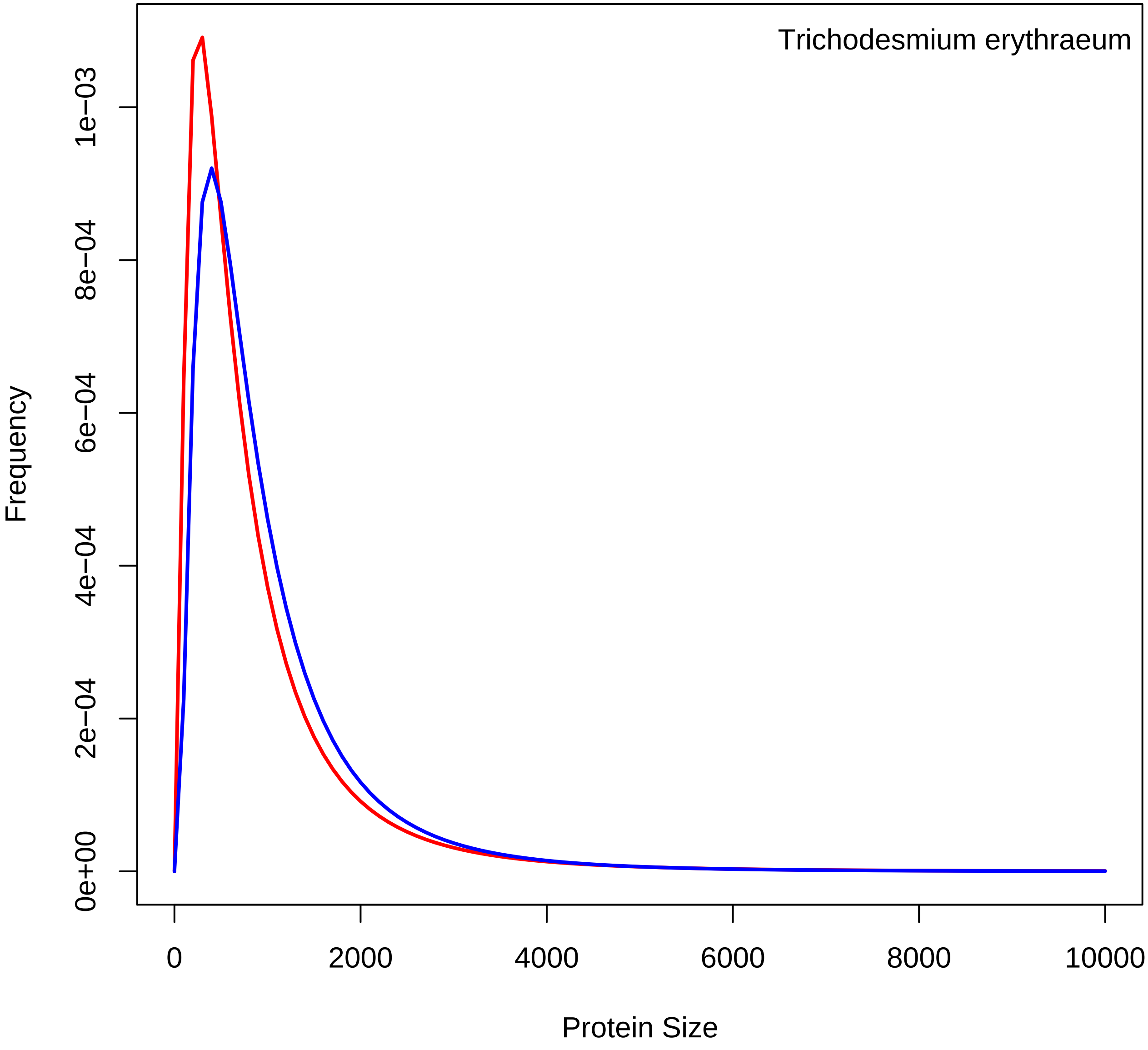

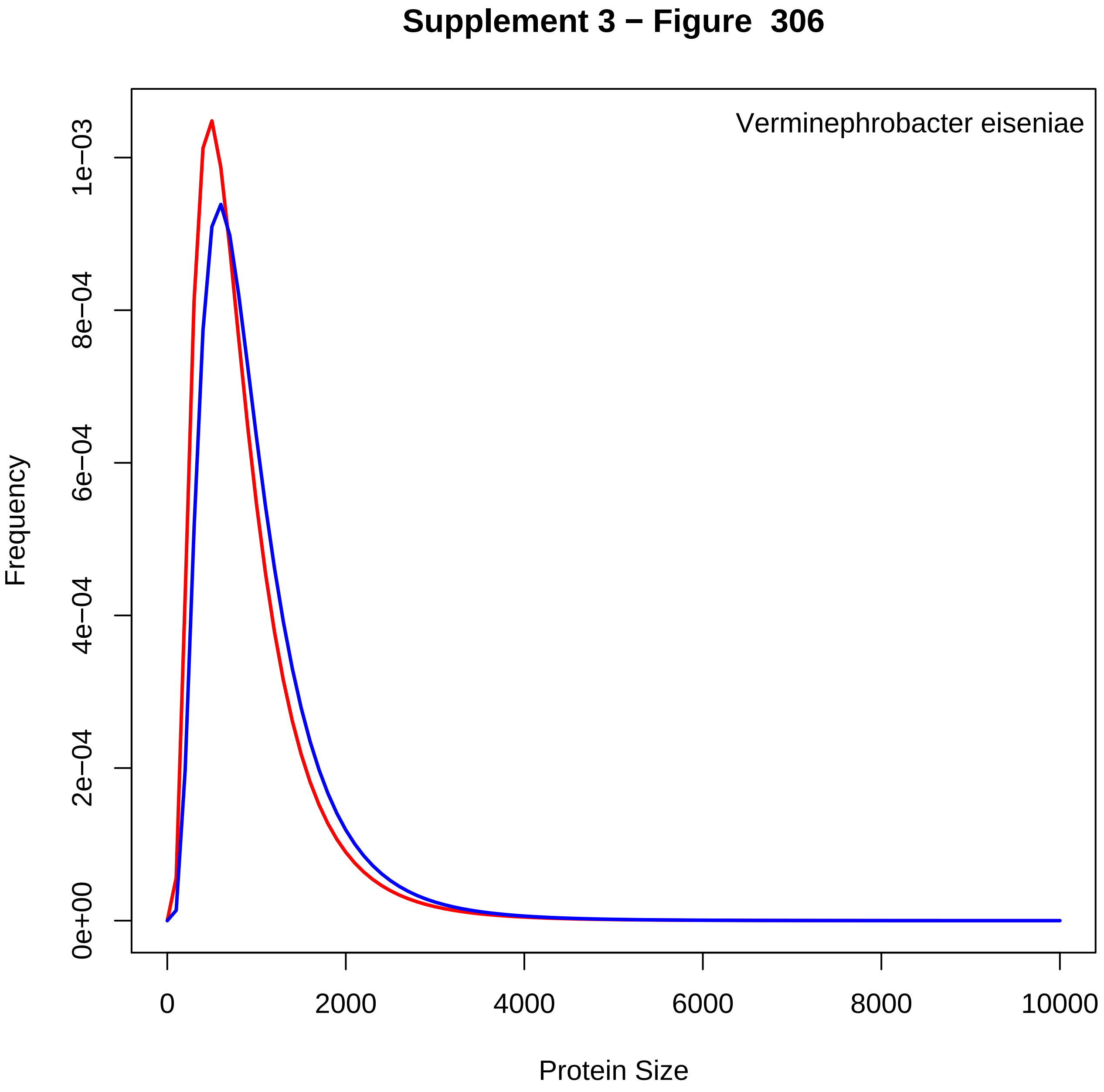

Supplement 3 − Figure 306
Verminephrobacter eiseniae
Frequency
0e+00
2e−04
4e−04
6e−04
8e−04
1e−03
0
2000
4000
6000
8000
10000
Protein Size

Supplement 3 − Figure 307

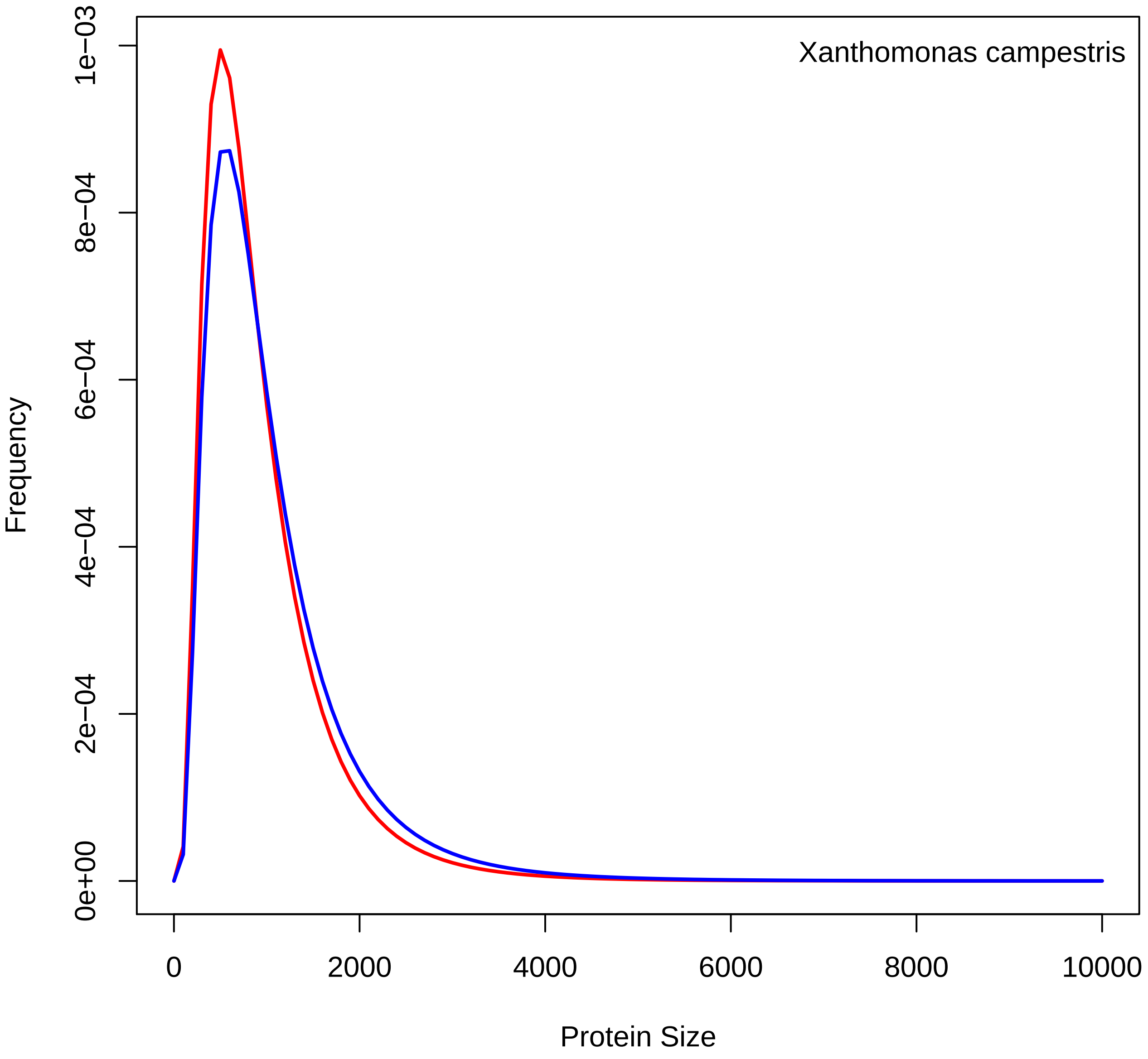

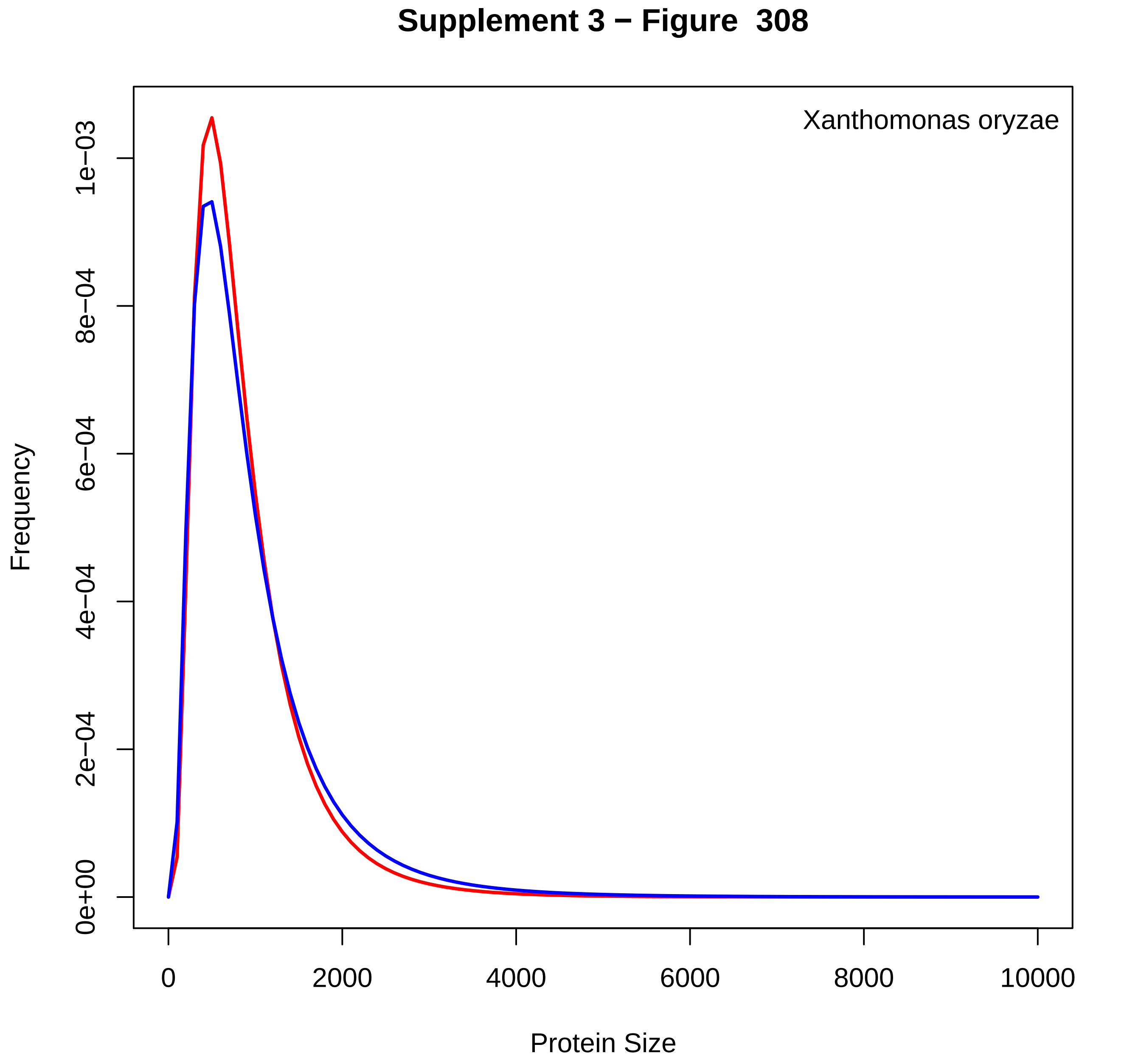
Supplement 3 − Figure 308
Xanthomonas oryzae
Frequency
0e+00
2e−04
4e−04
6e−04
8e−04
1e−03
0
2000
4000
6000
8000
10000
Protein Size

Supplement 3 − Figure 309

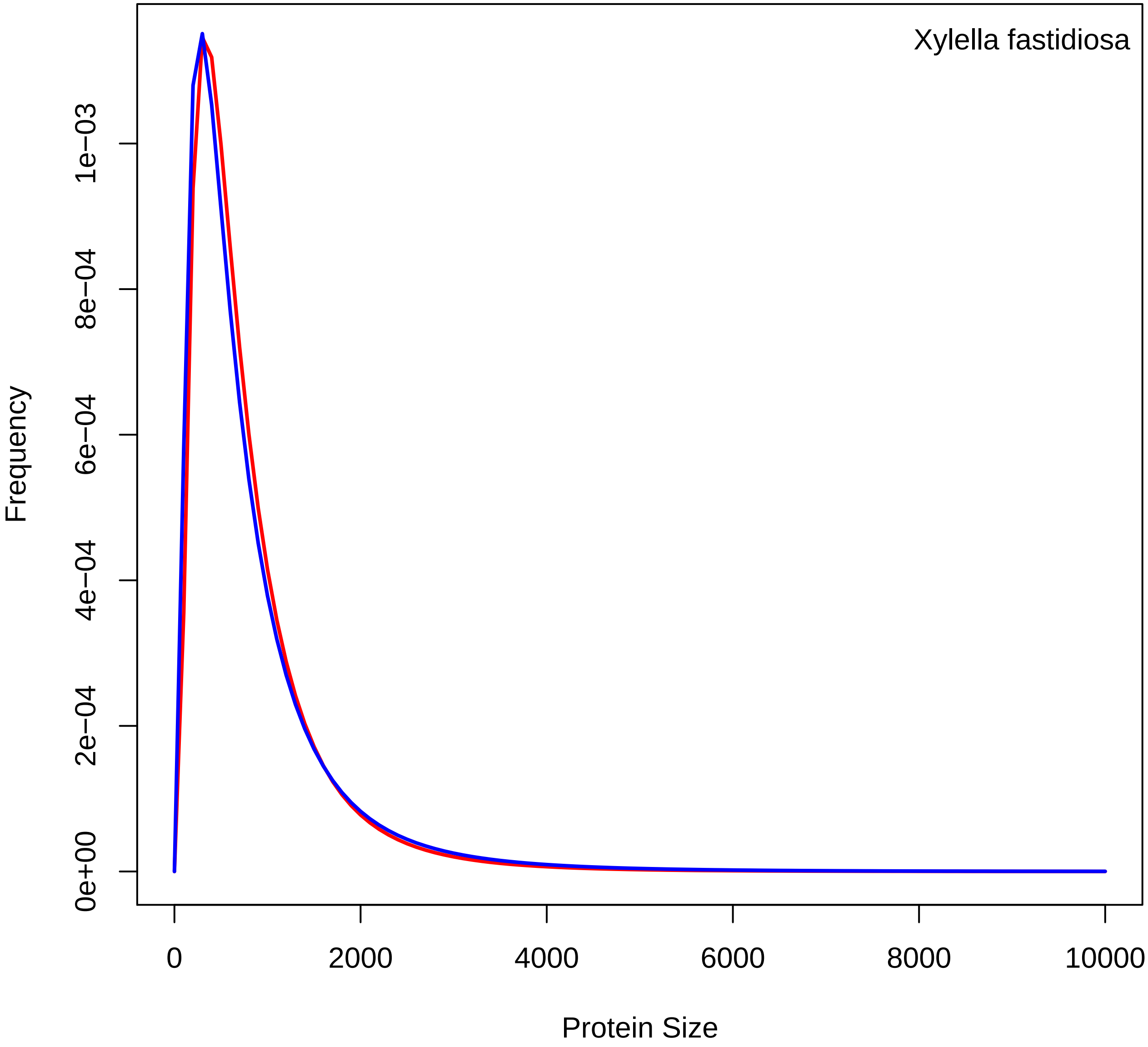

Supplement 3 − Figure 310

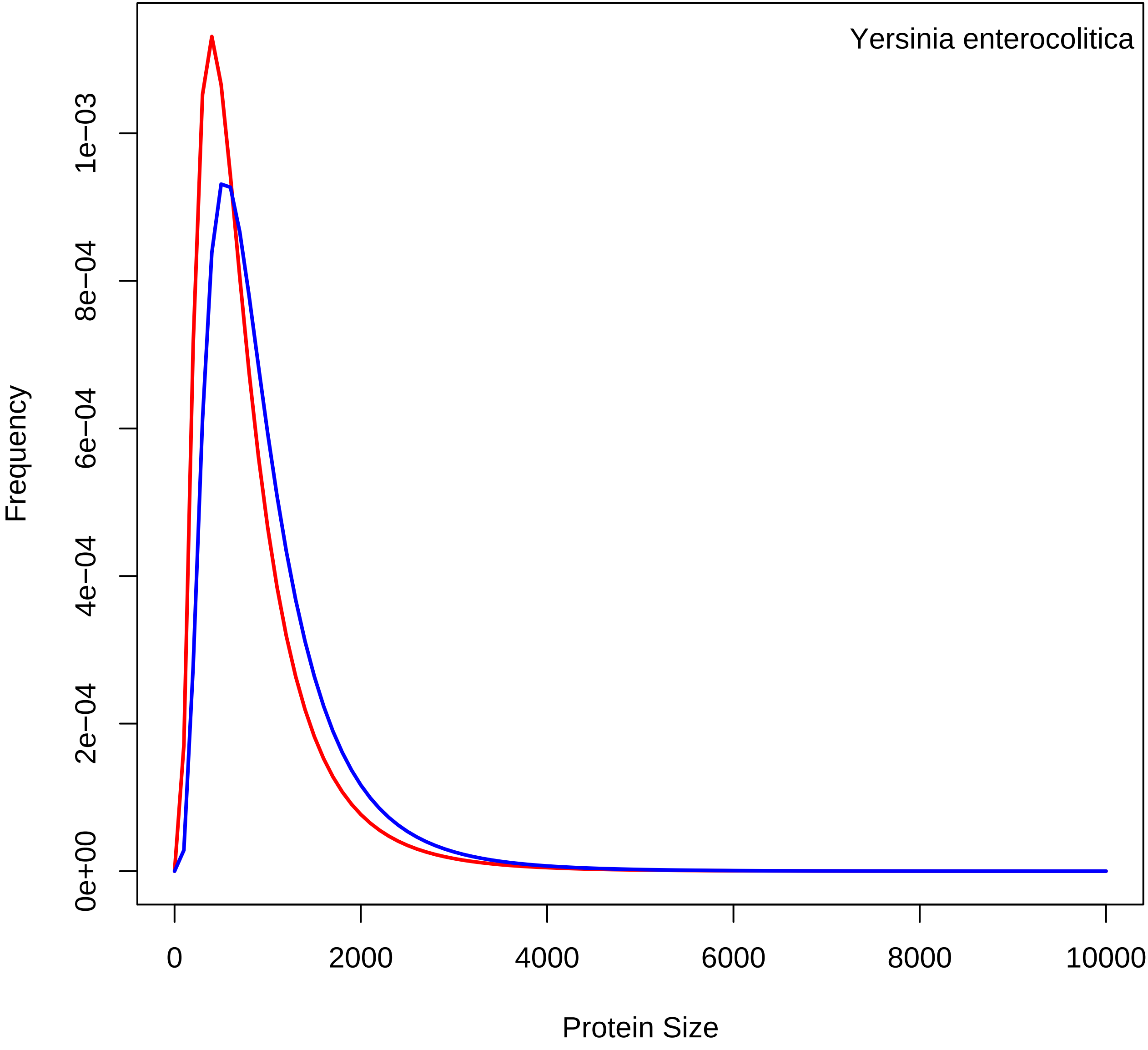

Supplement 3 − Figure 311

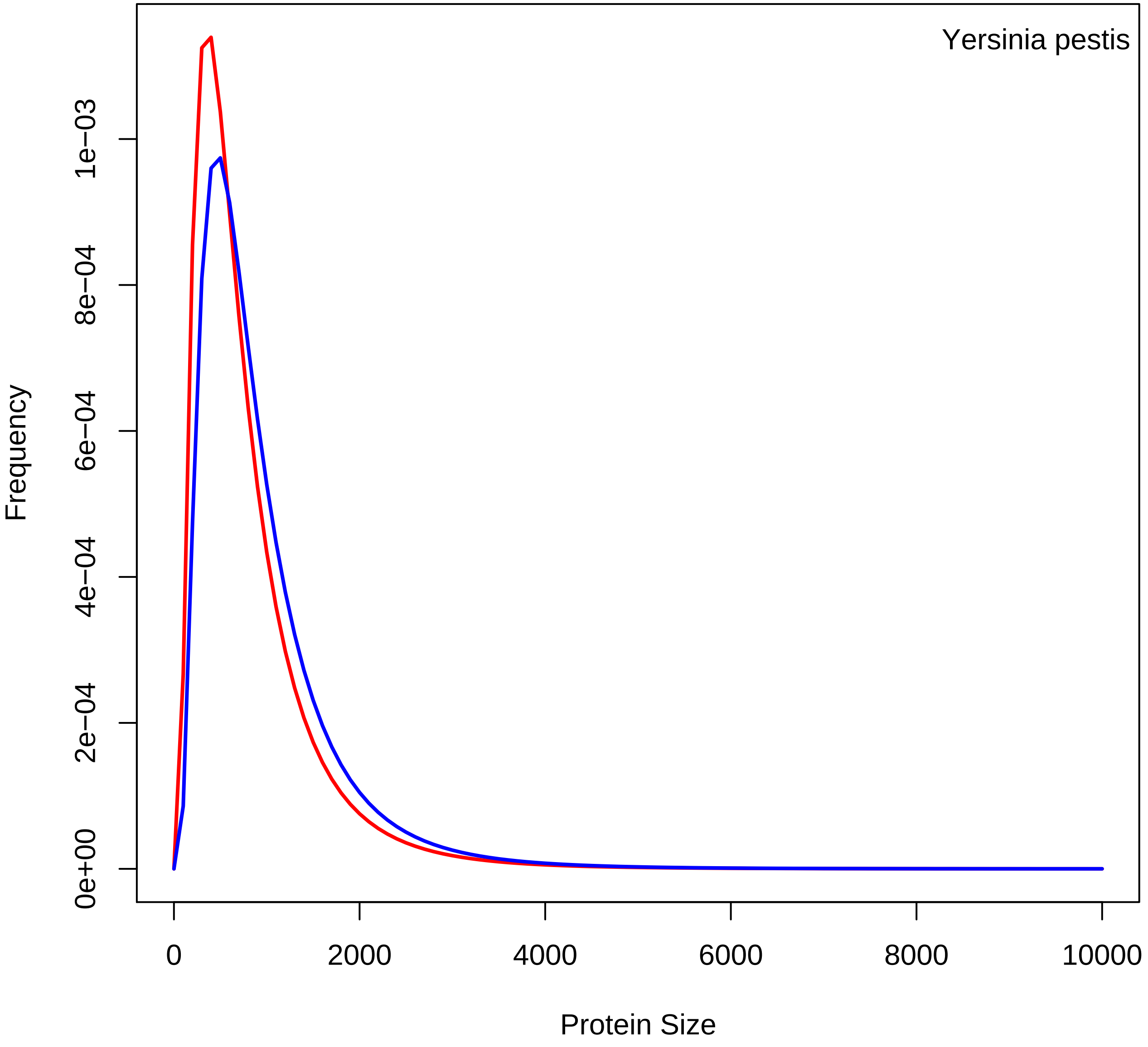

Supplement 3 − Figure 312

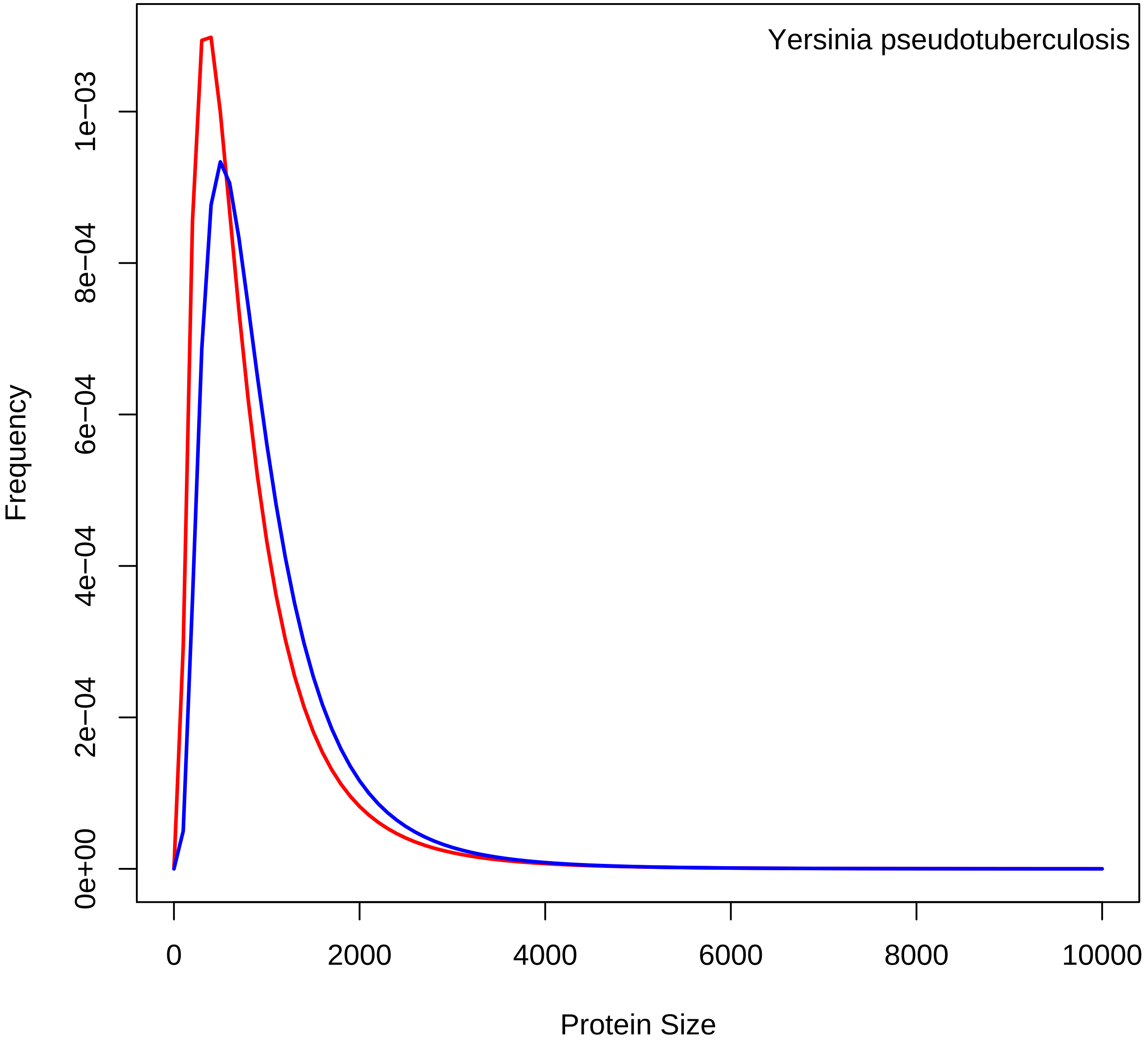

Supplement: Figure S2 — Relationships and illustrations of the shapes of the size distributions of ORFS and annotated proteins using log normal models. This supplement includes figures and analyses that depict the statistical relationships between the parameters of the exponential-log normal model and annotated proteins presented in Supplement 1. This supplement also includes figures that illustrate the shapes of each of these distributions. (0.74 MB PDF) [file pone.0006456.s003.pdf]
