# Supplementary material for: The Random Nature of Genome Architecture: Predicting Open Reading Frame Distributions
Source: PLoS One. 2009 Jul 30;4(7):e6456. doi: 10.1371/journal.pone.0006456 (PMC2714469; doi:10.1371/journal.pone.0006456)
Supplement: Figure S3 — Relationships and illustrations of the shapes of the size distributions of ORFS and annotated proteins using gamma distributions. This supplement includes figures and analyses that depict the statistical relationships between the parameters of the exponential-gamma model and annotated proteins presented in Supplement 1. This supplement also includes figures that illustrate the shapes of each of these distributions. (0.58 MB PDF) [file pone.0006456.s004.pdf]

Supplement 4

Significant relationships and illustrations of the shapes of the size distributions of ORFS described by the non-random distribution of the exponential-gamma mixture model and annotated proteins. All parameter values are listed in Supplement 1. Within this supplement, figure one depicts the statistical relationships between the parameters estimated with the exponential-gamma model and parameters estimated from annotated proteins (also see figure 4 in main document), and figures two through 312 illustrate the shapes of each of these distributions (arranged alphabetically by species to correspond with Supplement 1). In these figures red lines represent the shapes of the distributions estimated from the mixture model and blue lines show the shape of the distribution estimated from annotated proteins. It is important to note from these figures that the shapes of the size distributions of non-random ORFs deviate from the annotated proteins such that the number of small non-random ORFs is consistently greater than the number of small annotated proteins. Consequently, the scale parameters of the gamma distributions estimated from the mixture models are smaller and the peaks of the distributions shifted to the left of those for annotated proteins. Moreover, the magnitude of the shift is greater in multicellular eukaryotes than prokaryotes. Nevertheless, the scale and shape parameters estimated from the mixture model fits are significantly correlated with the parameter estimates from fits to annotated proteins (S4 – Fig. 1).

Supplement 4 – Figure 1

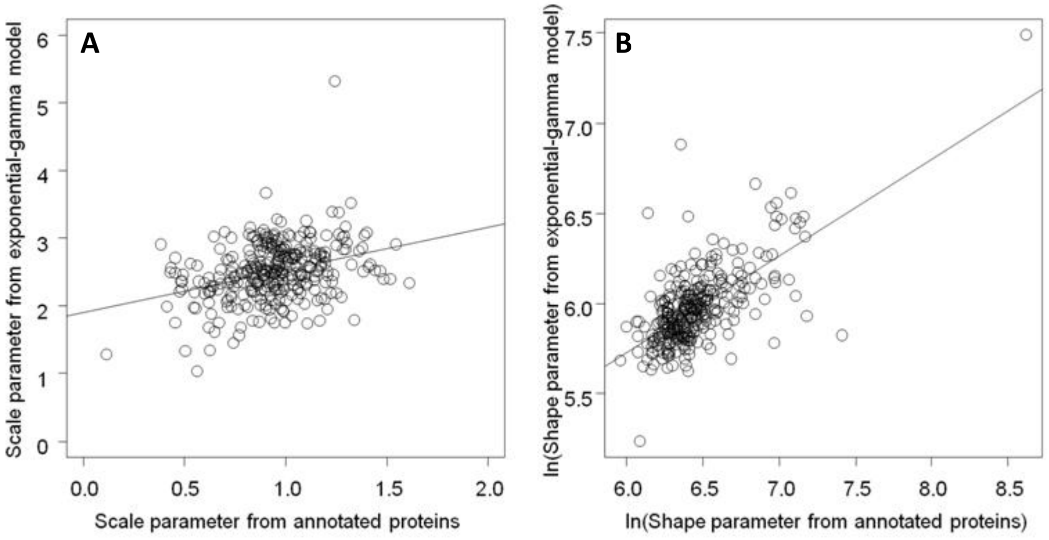

S4 - Figure 1. Plots of parameter estimates determined from the exponential-gamma mixture model fit to the ORF size distributions against parameter estimates determined from gamma model fits to annotated proteins. Panel **A** illustrates the significant relationship between the scale parameter estimates ($F_{1,309}$ = 38.53, $p < 0.0001$). Panel **B** illustrates the significant relationship between the shape parameters estimates ($F_{1,309}$ = 236.55, $p < 0.0001$).

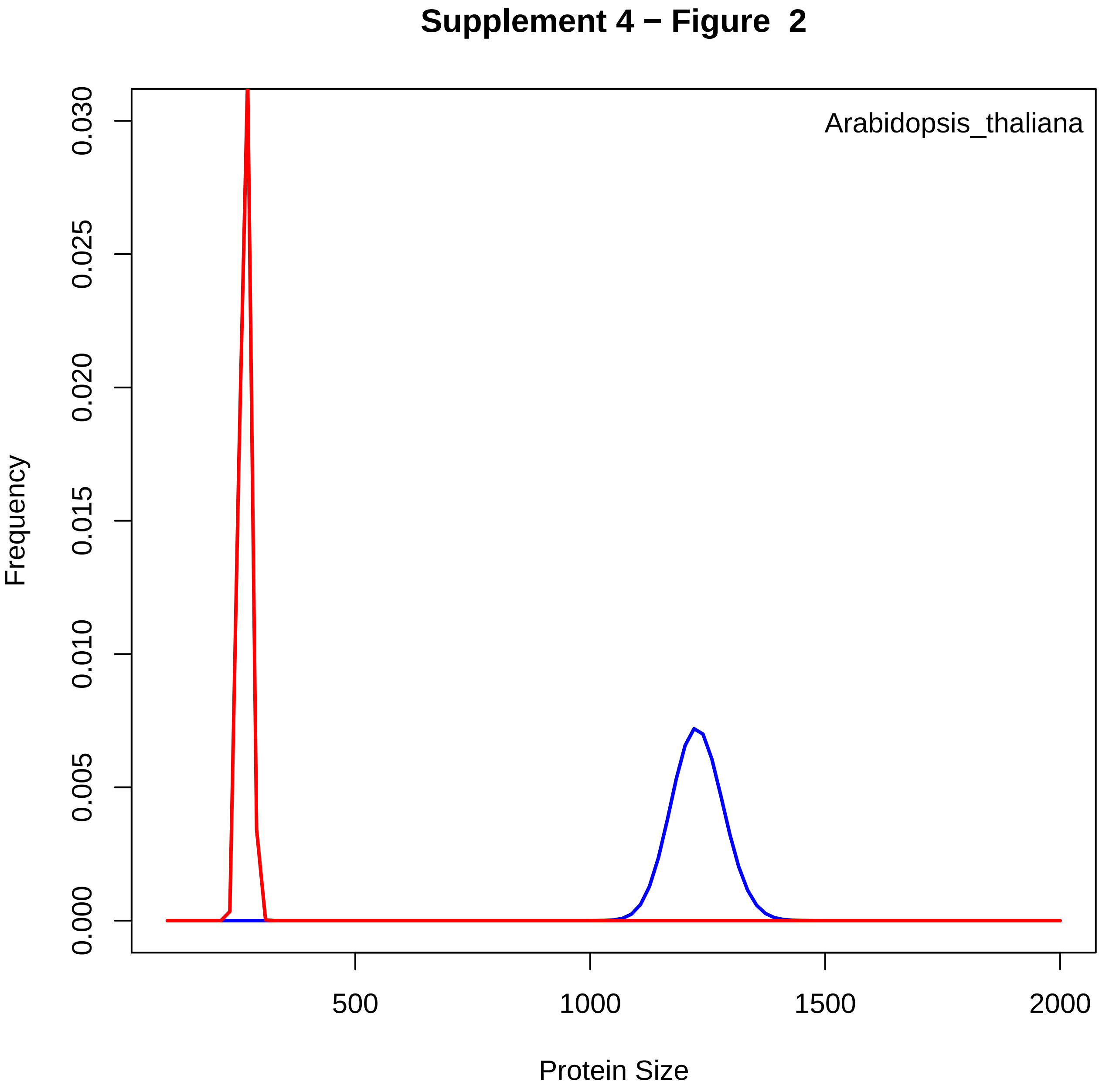
Supplement 4 − Figure 2
Arabidopsis_thaliana
Frequency
0.000
0.005
0.010
0.015
0.020
0.025
0.030
500
1000
1500
2000
Protein Size

Supplement 4 − Figure 3

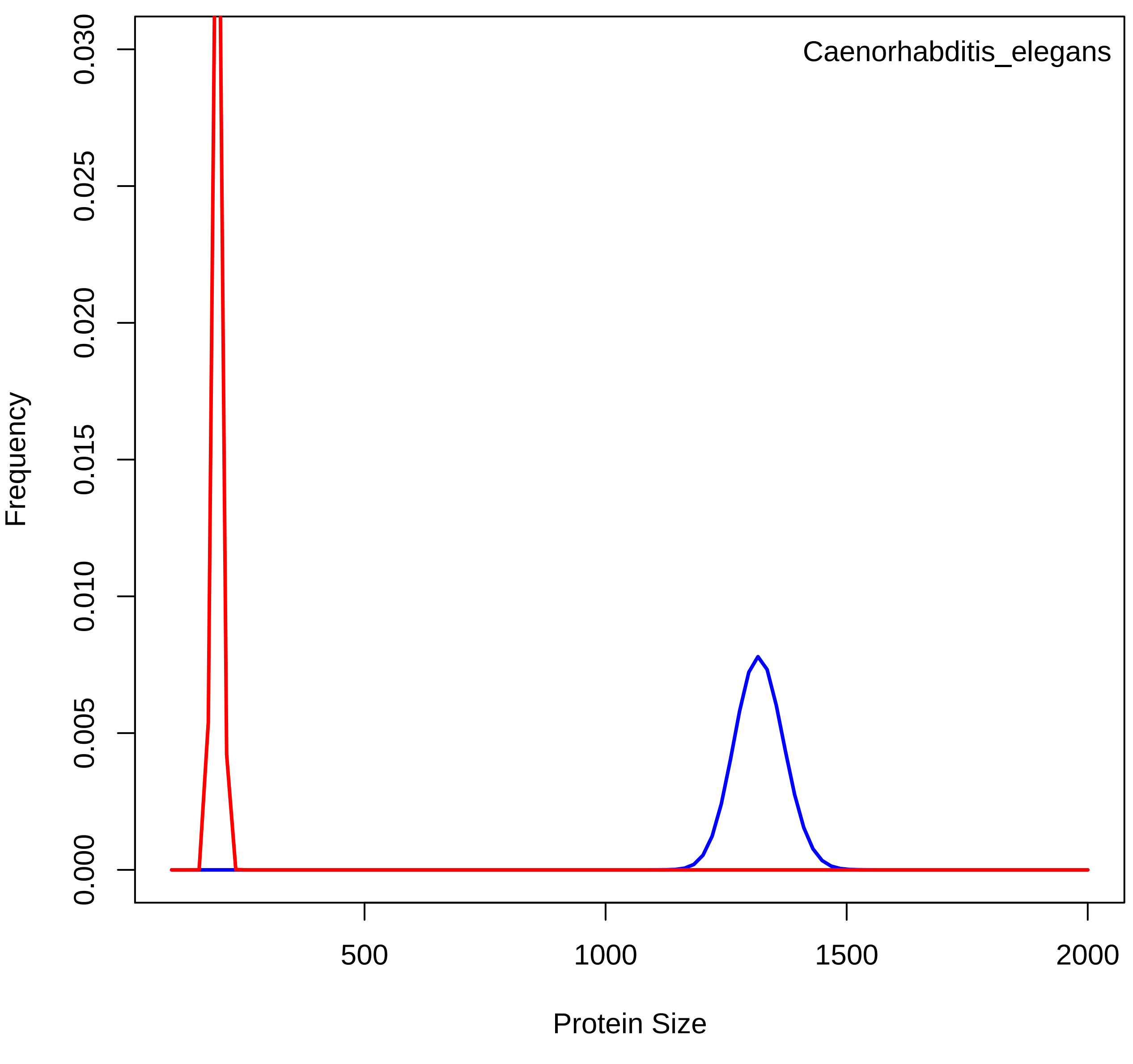

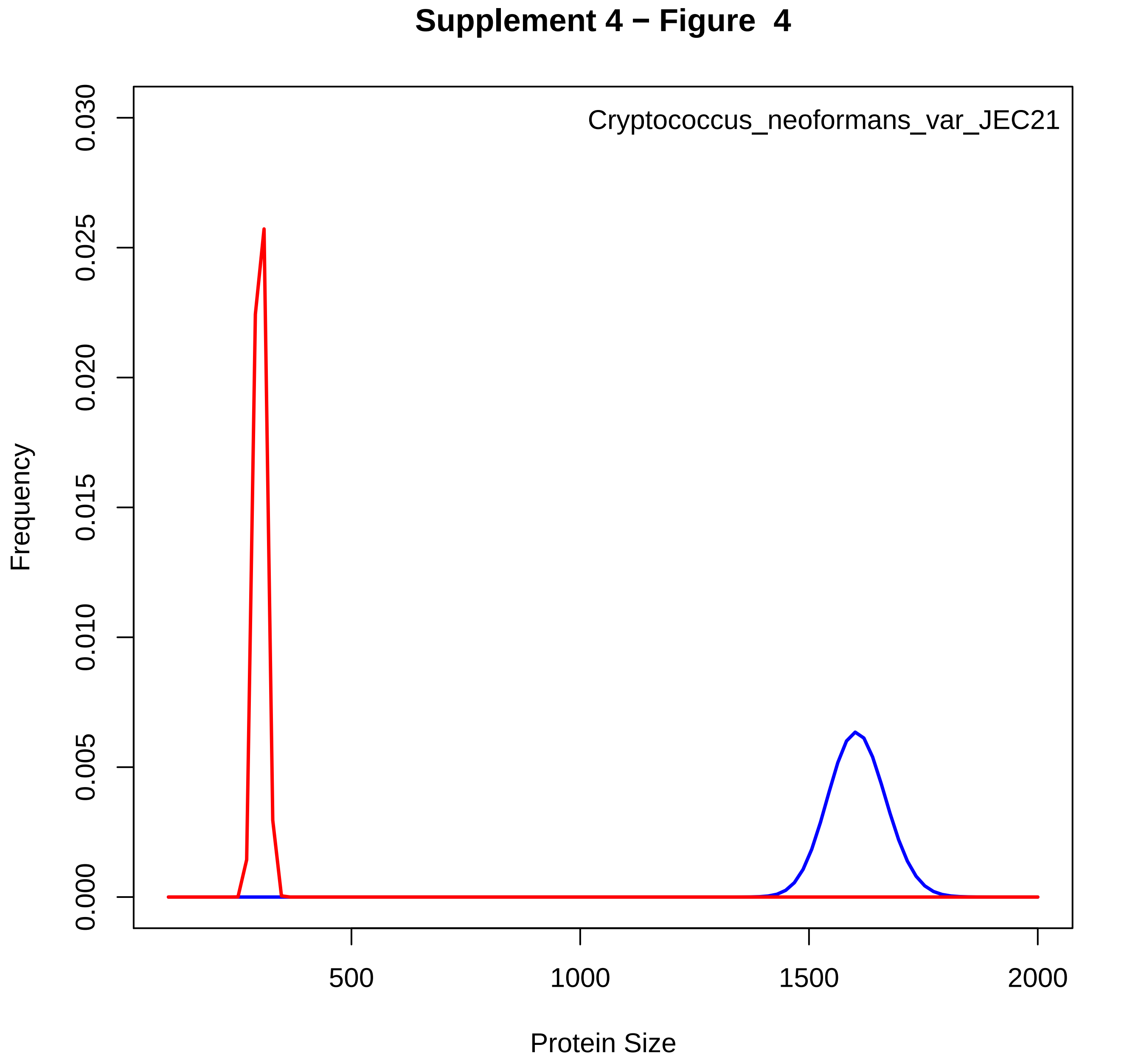
Supplement 4 − Figure 4
Cryptococcus_neoformans_var_JEC21
Frequency
0.000
0.005
0.010
0.015
0.020
0.025
0.030
500
1000
1500
2000
Protein Size

Supplement 4 − Figure 5

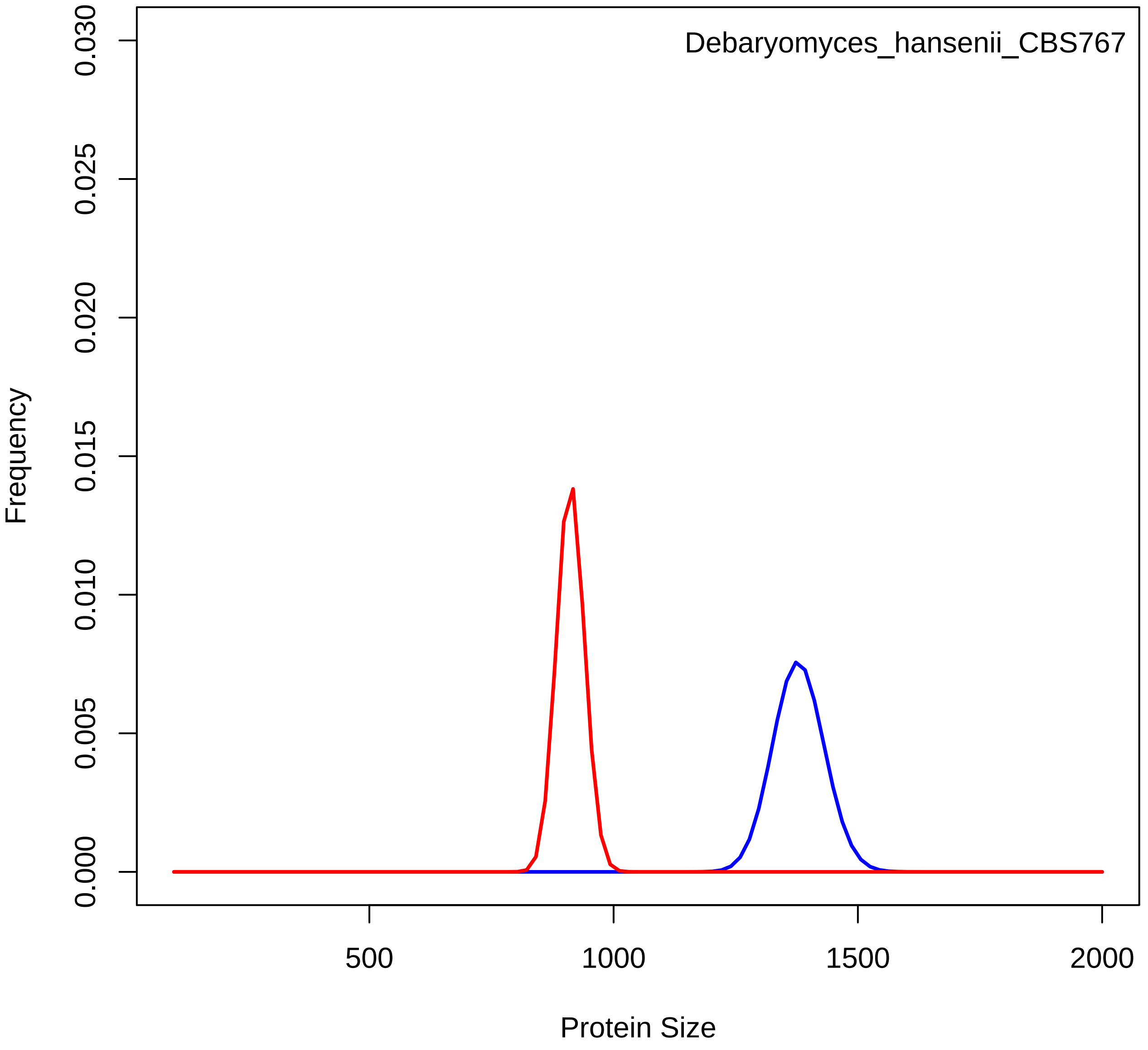

Supplement 4 − Figure 6

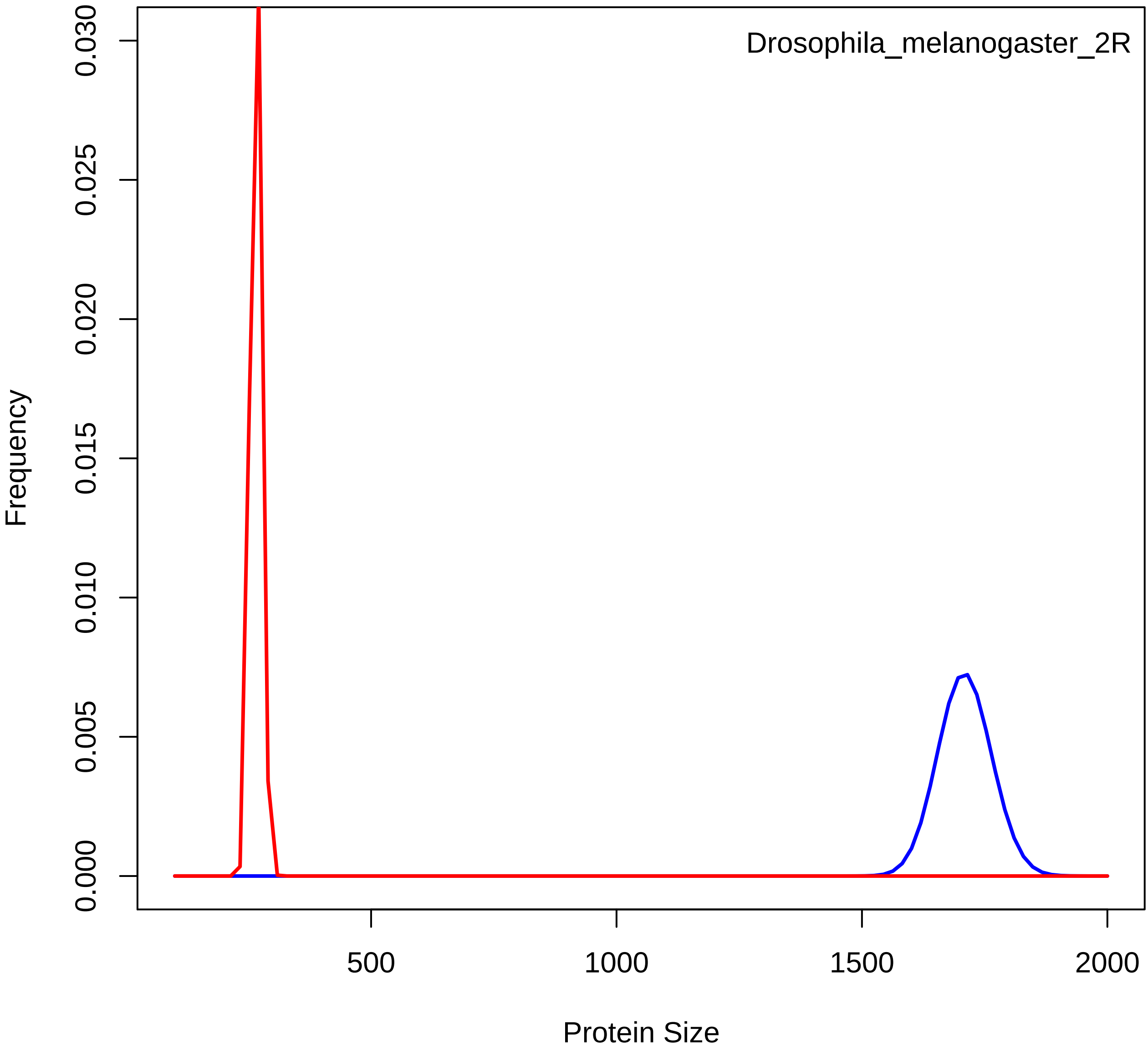

Supplement 4 − Figure 7

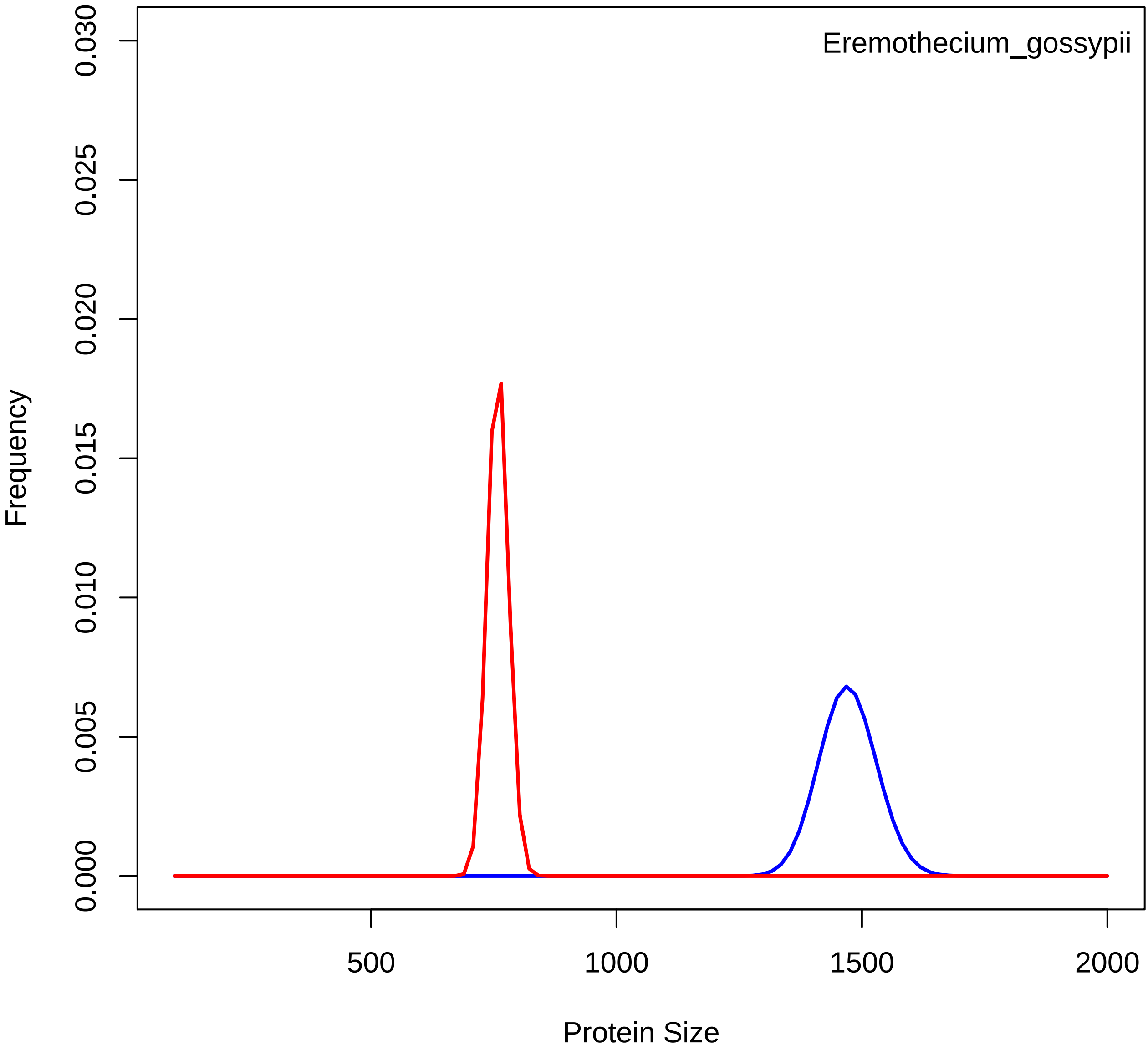

Supplement 4 − Figure 8

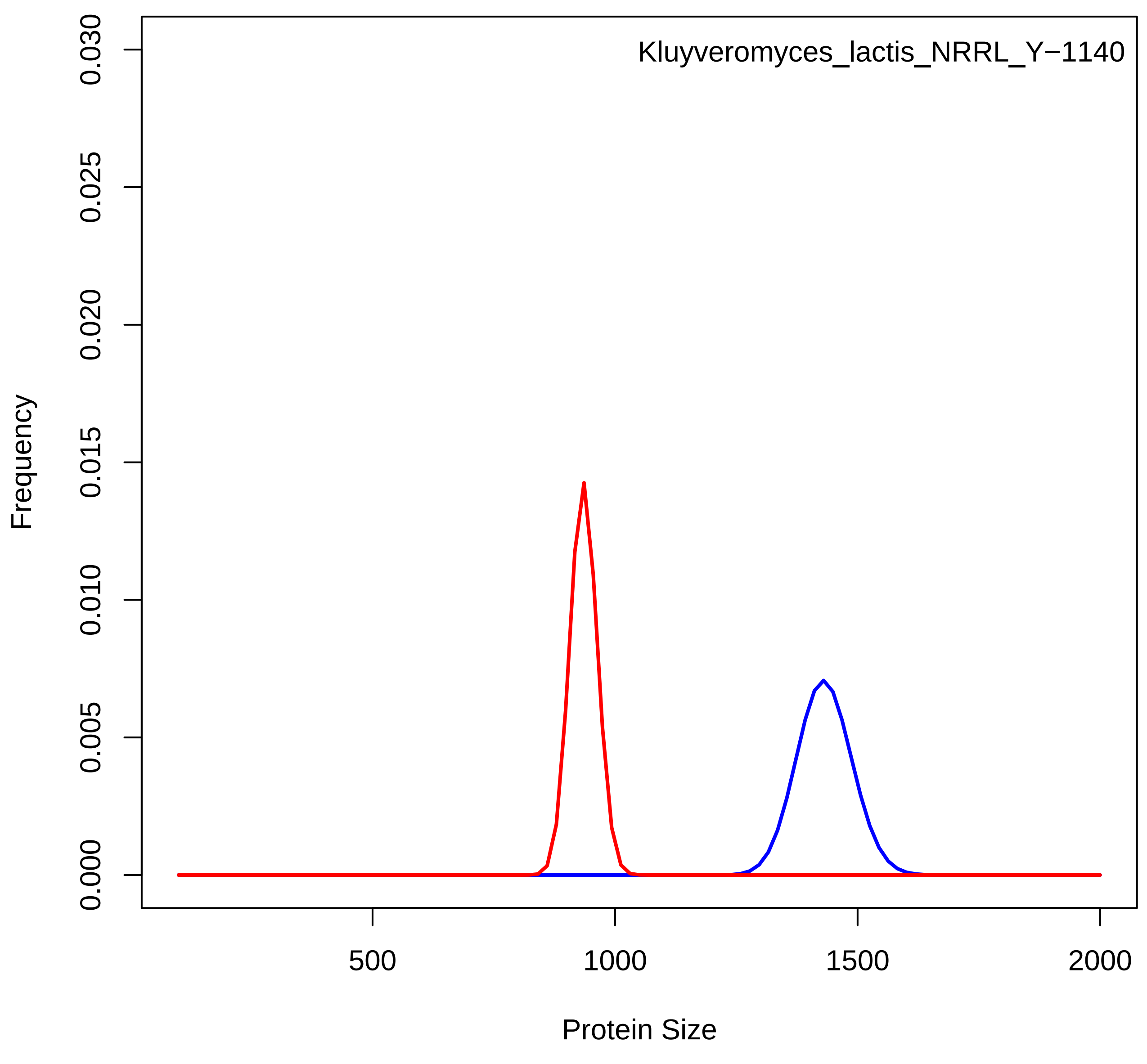

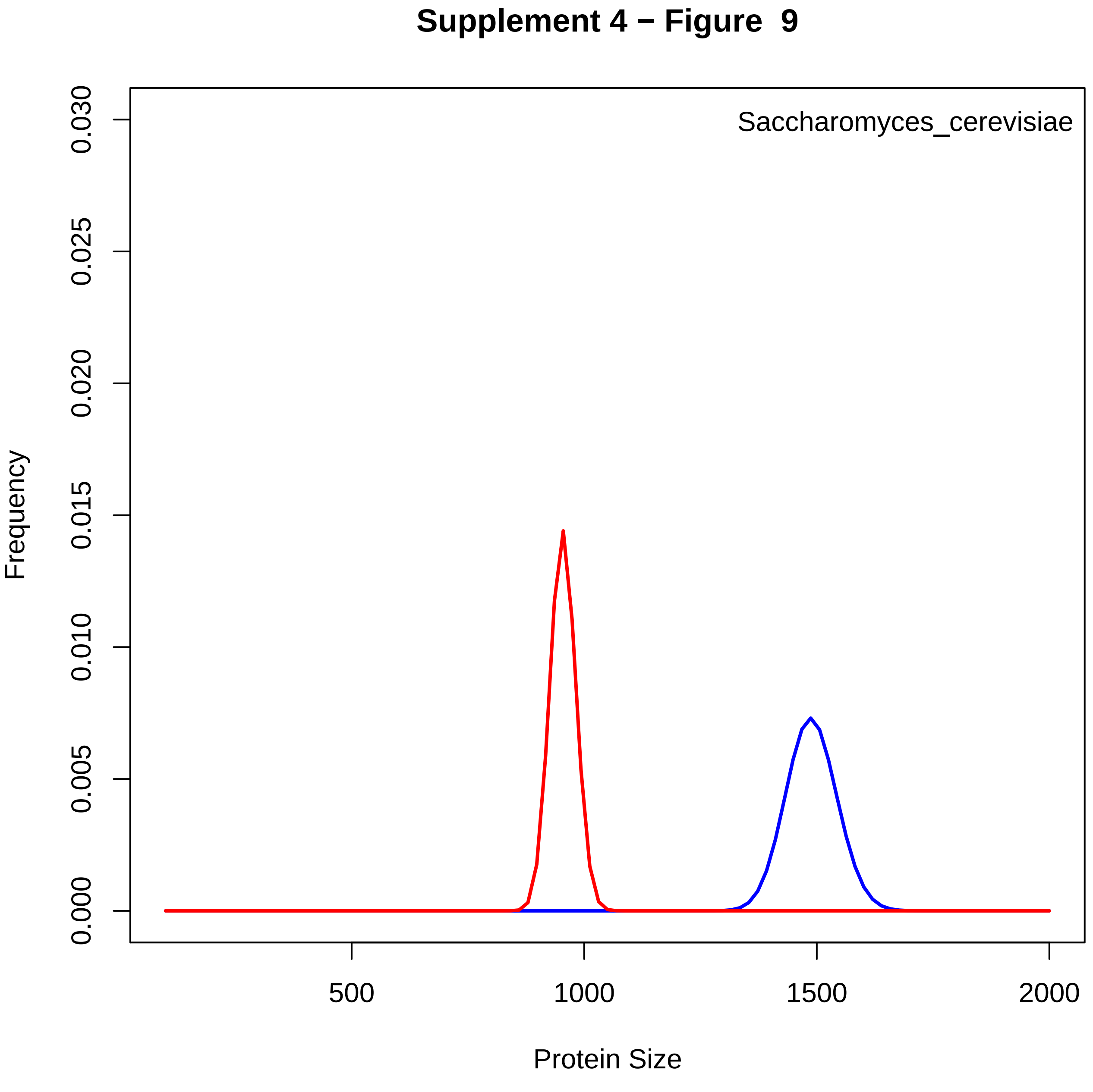
Supplement 4 − Figure 9
Saccharomyces_cerevisiae
Frequency
0.000
0.005
0.010
0.015
0.020
0.025
0.030
500
1000
1500
2000
Protein Size

Supplement 4 − Figure 10

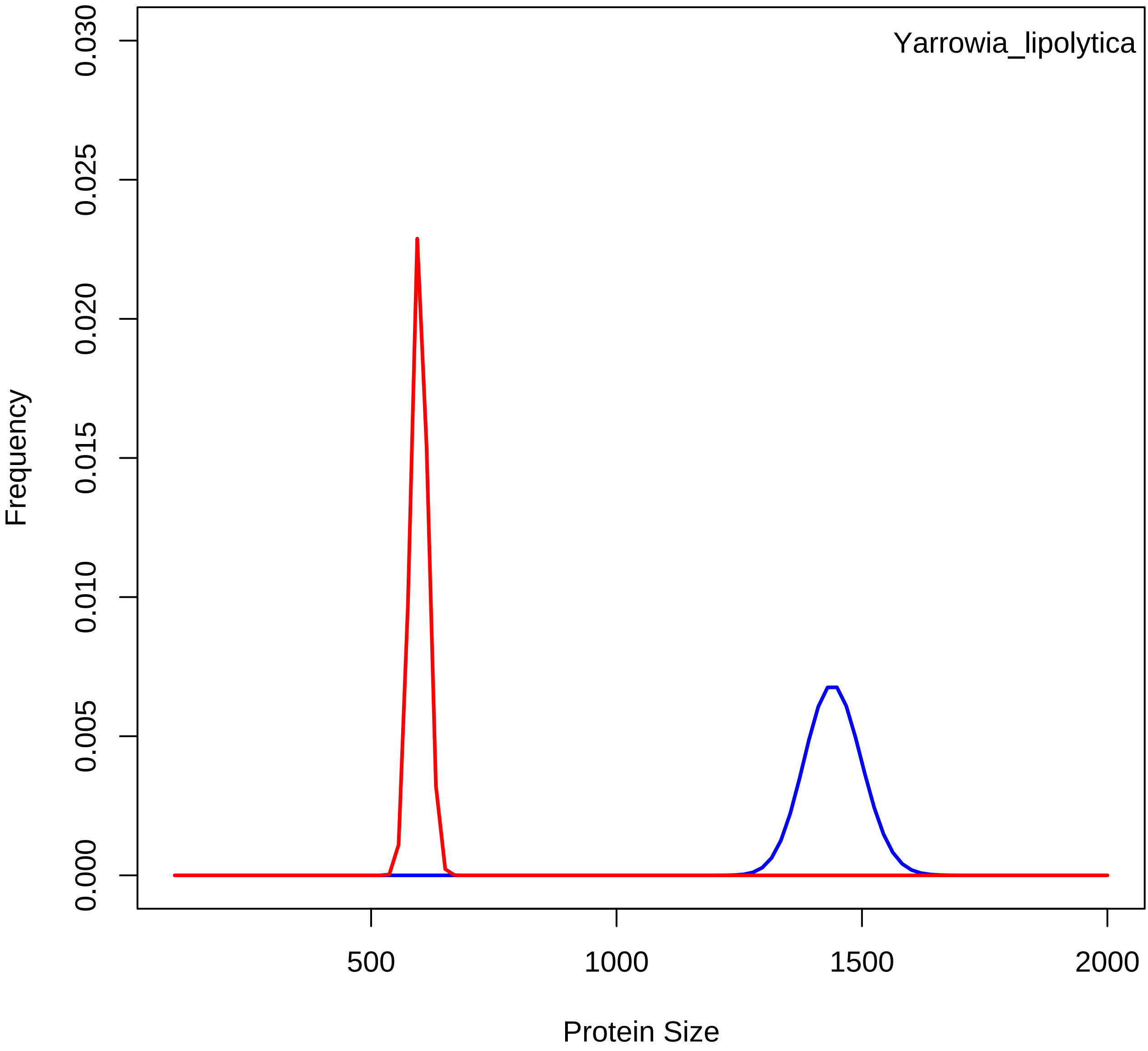

Supplement 4 − Figure 11

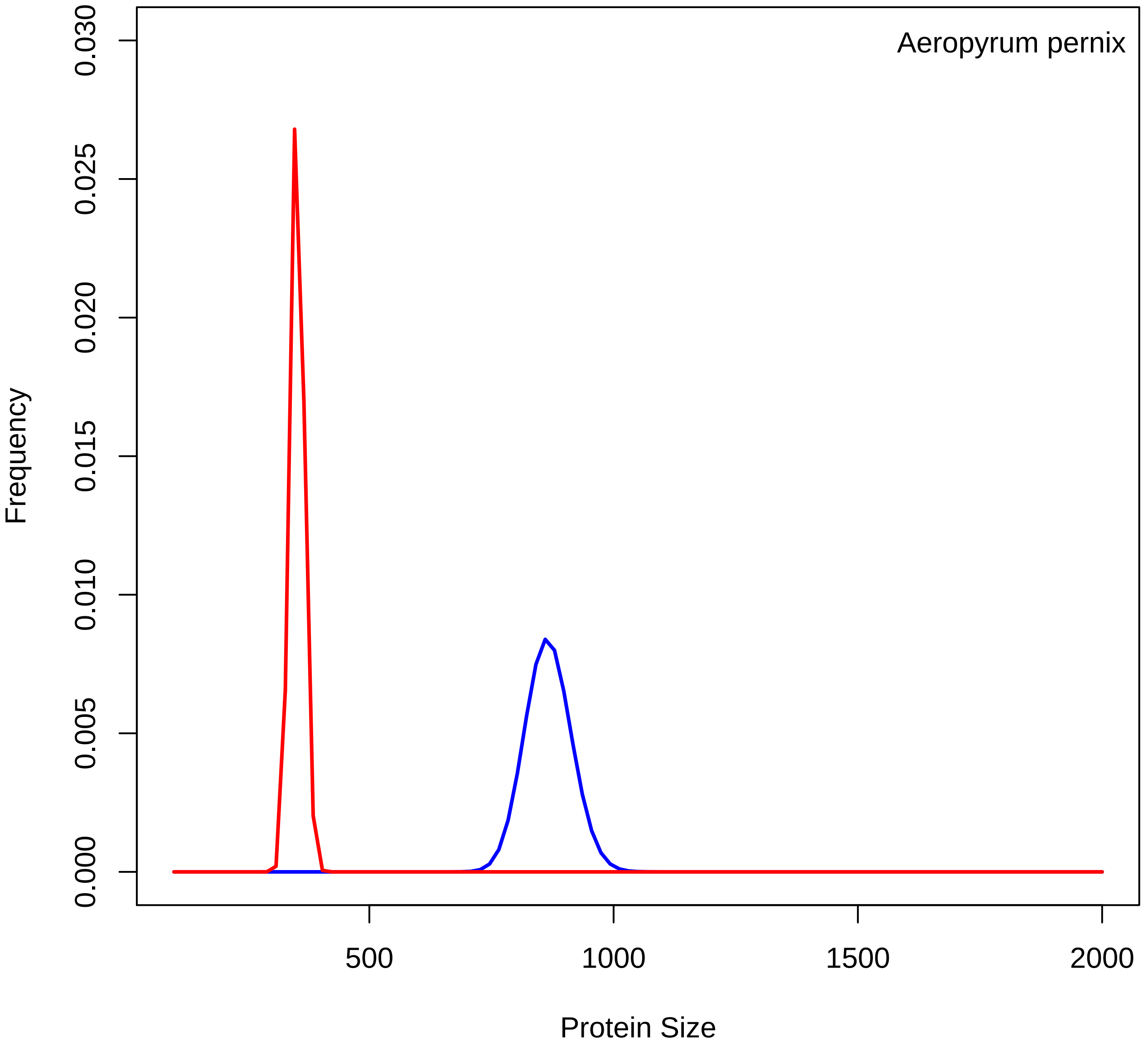

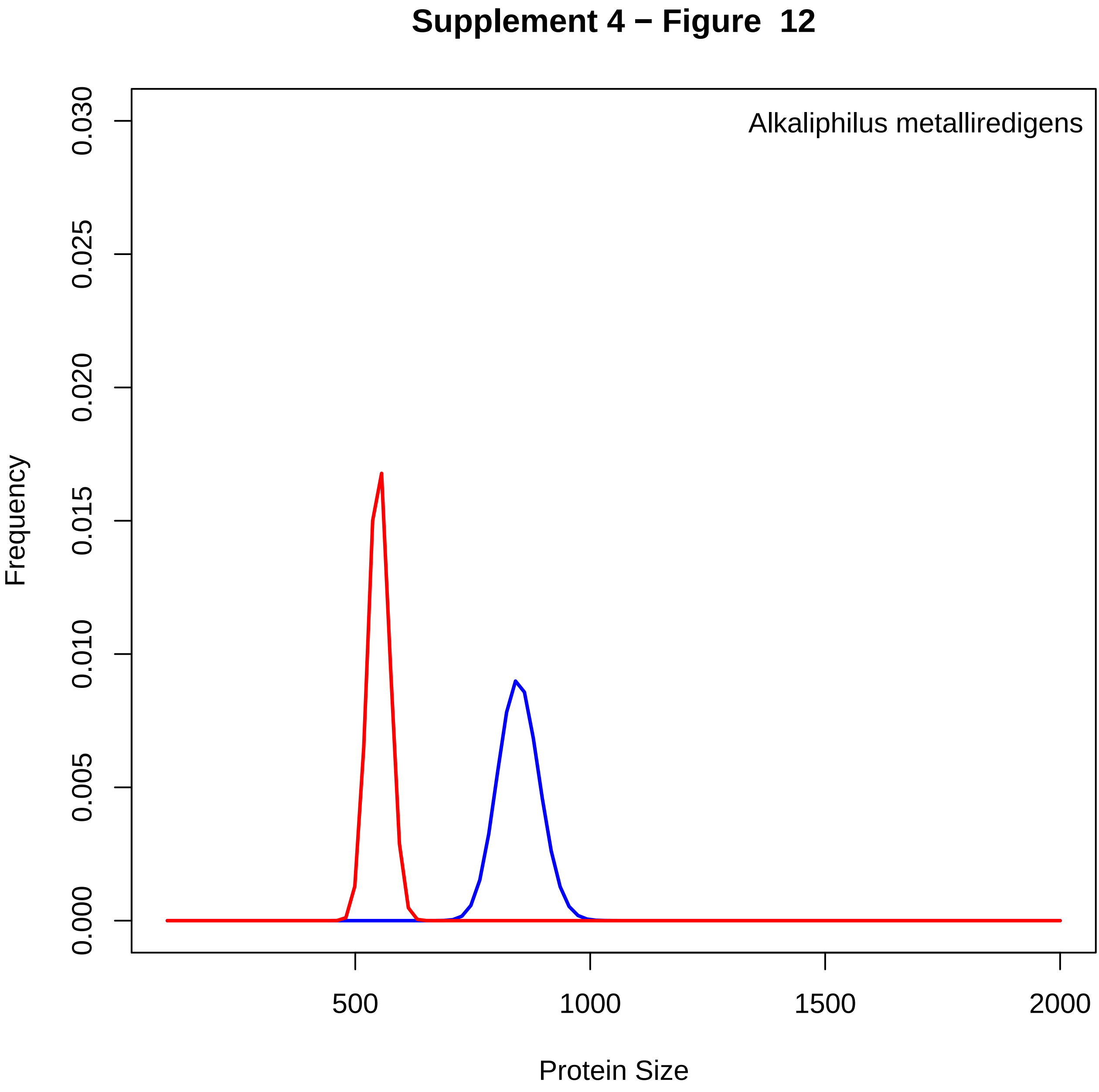

Supplement 4 − Figure 12
Alkaliphilus metalliredigens
Frequency
0.000
0.005
0.010
0.015
0.020
0.025
0.030
500
1000
1500
2000
Protein Size

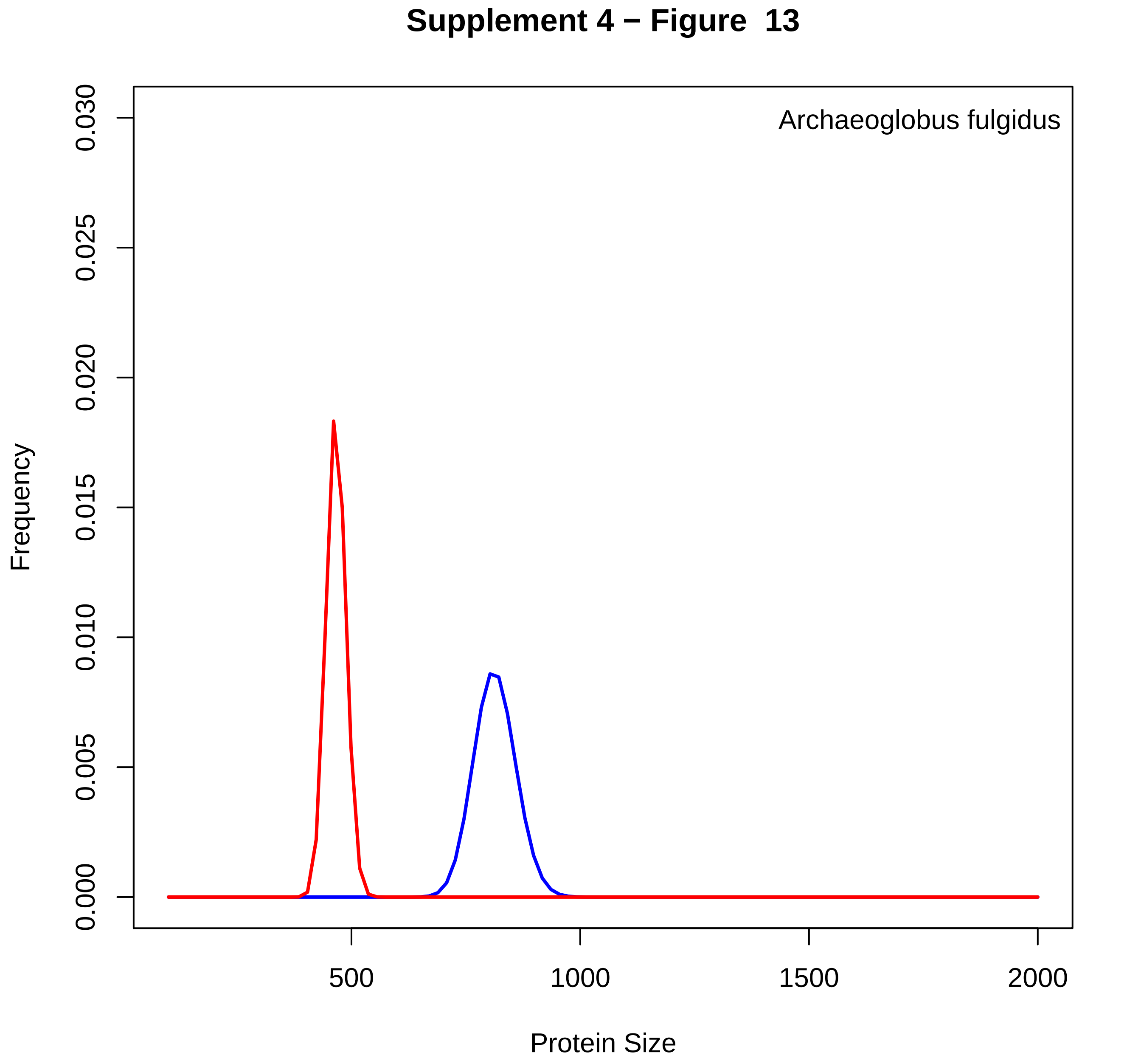
Supplement 4 − Figure 13
Archaeoglobus fulgidus
Frequency
0.000
0.005
0.010
0.015
0.020
0.025
0.030
500
1000
1500
2000
Protein Size

Supplement 4 − Figure 14

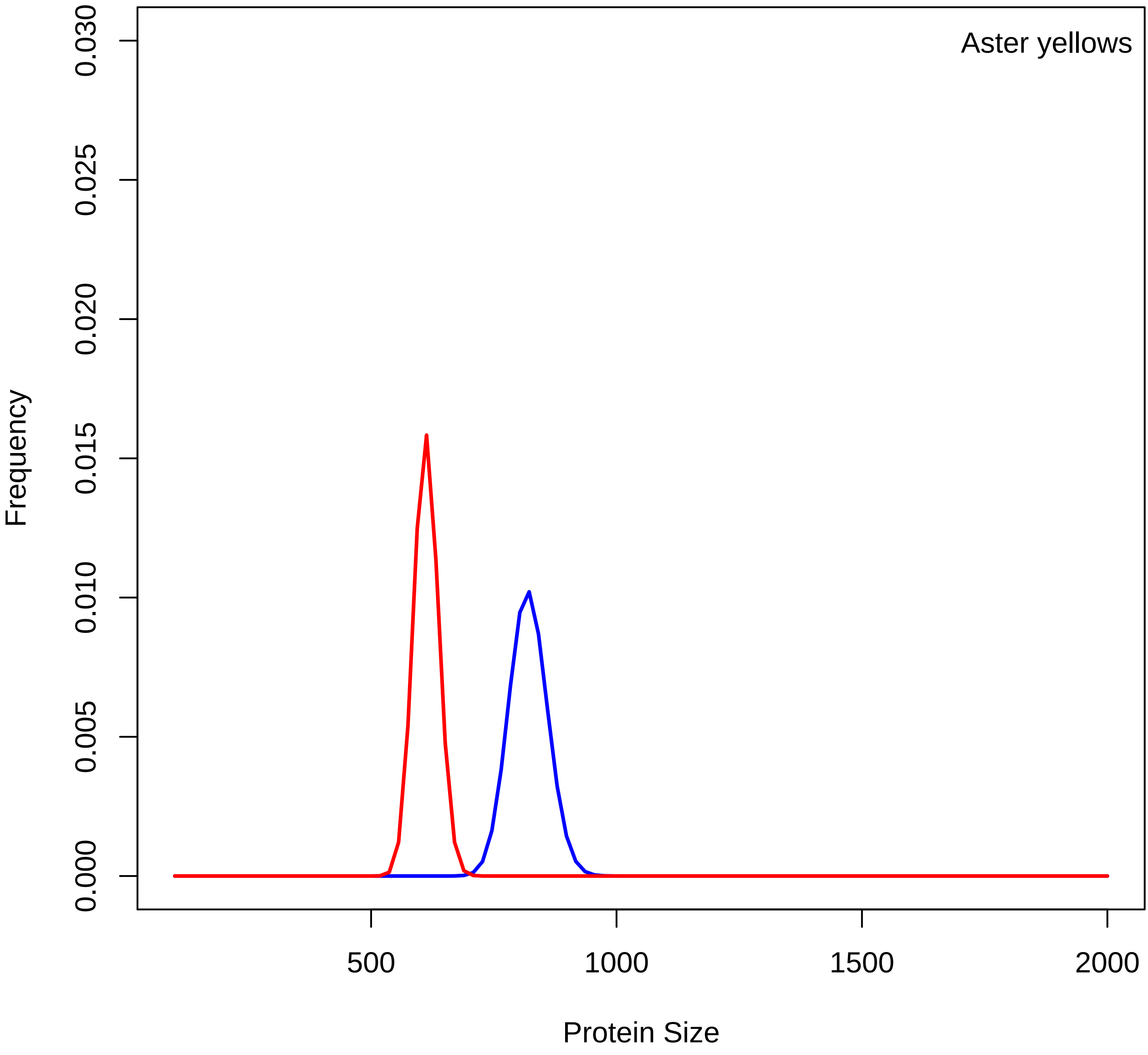

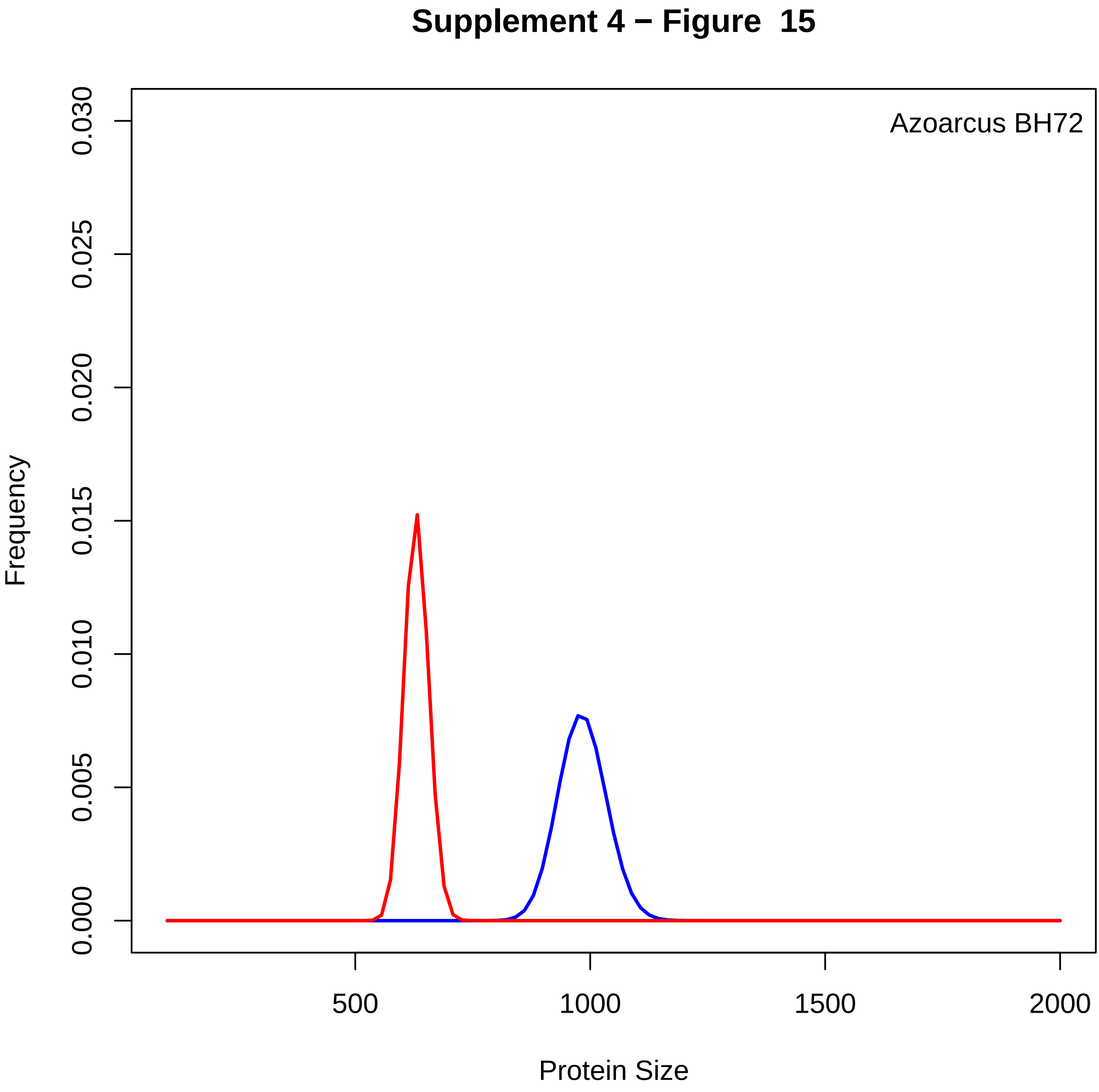
Supplement 4 − Figure 15
Azoarcus BH72
Frequency
0.000
0.005
0.010
0.015
0.020
0.025
0.030
500
1000
1500
2000
Protein Size

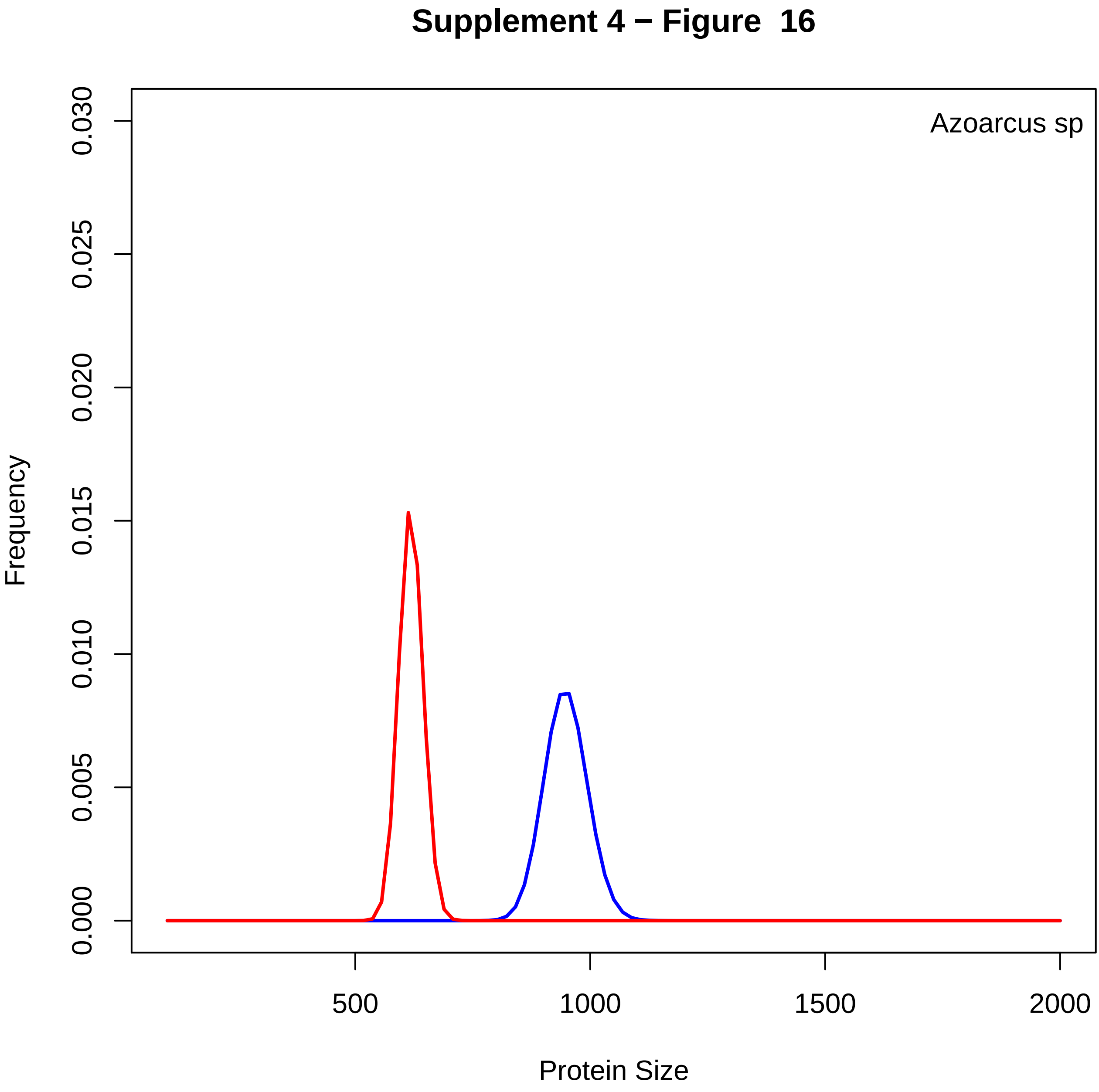
Supplement 4 − Figure 16
Azoarcus sp
Frequency
0.000
0.005
0.010
0.015
0.020
0.025
0.030
500
1000
1500
2000
Protein Size

Supplement 4 − Figure 17

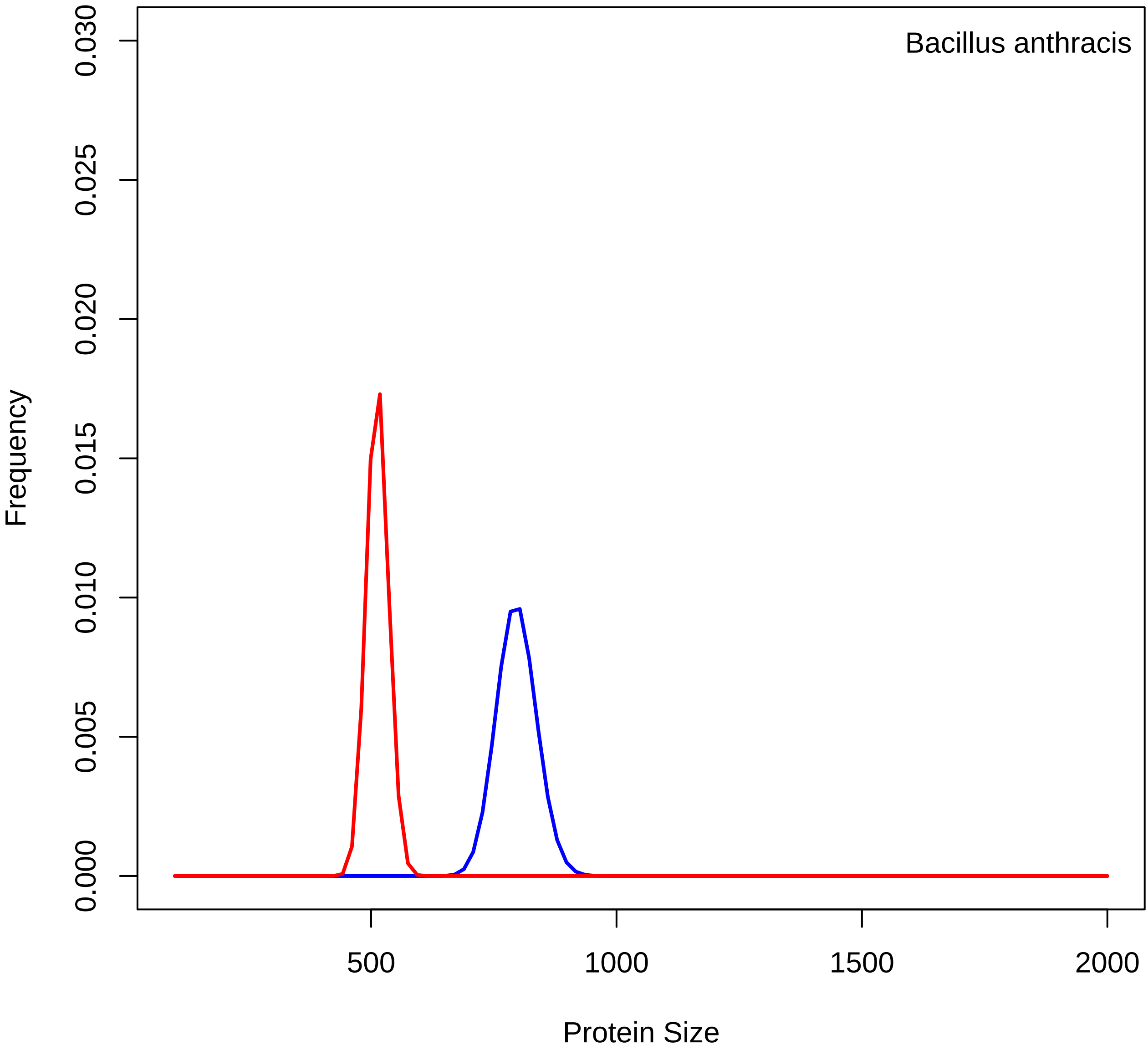

Supplement 4 − Figure 18

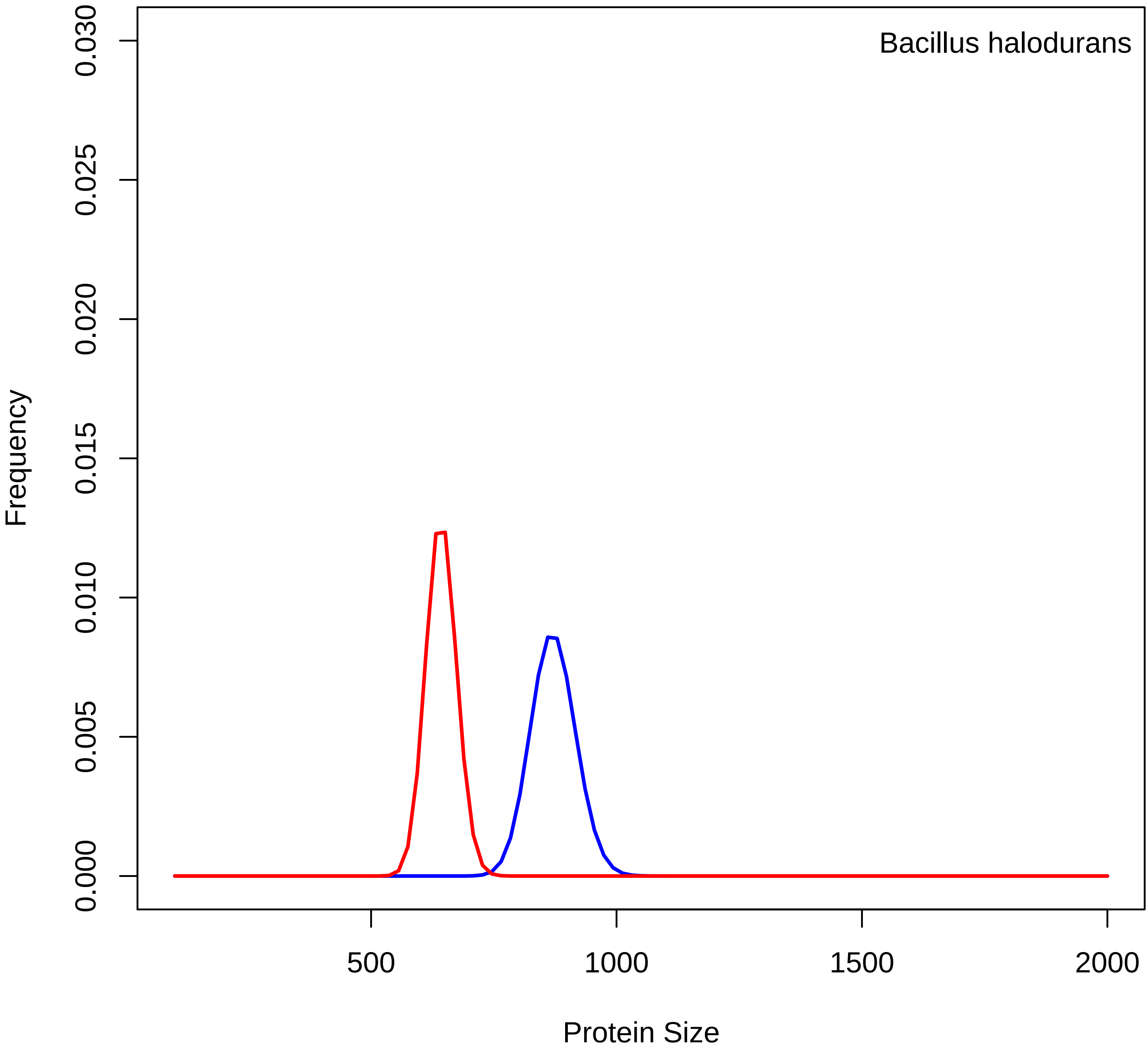

Supplement 4 − Figure 19

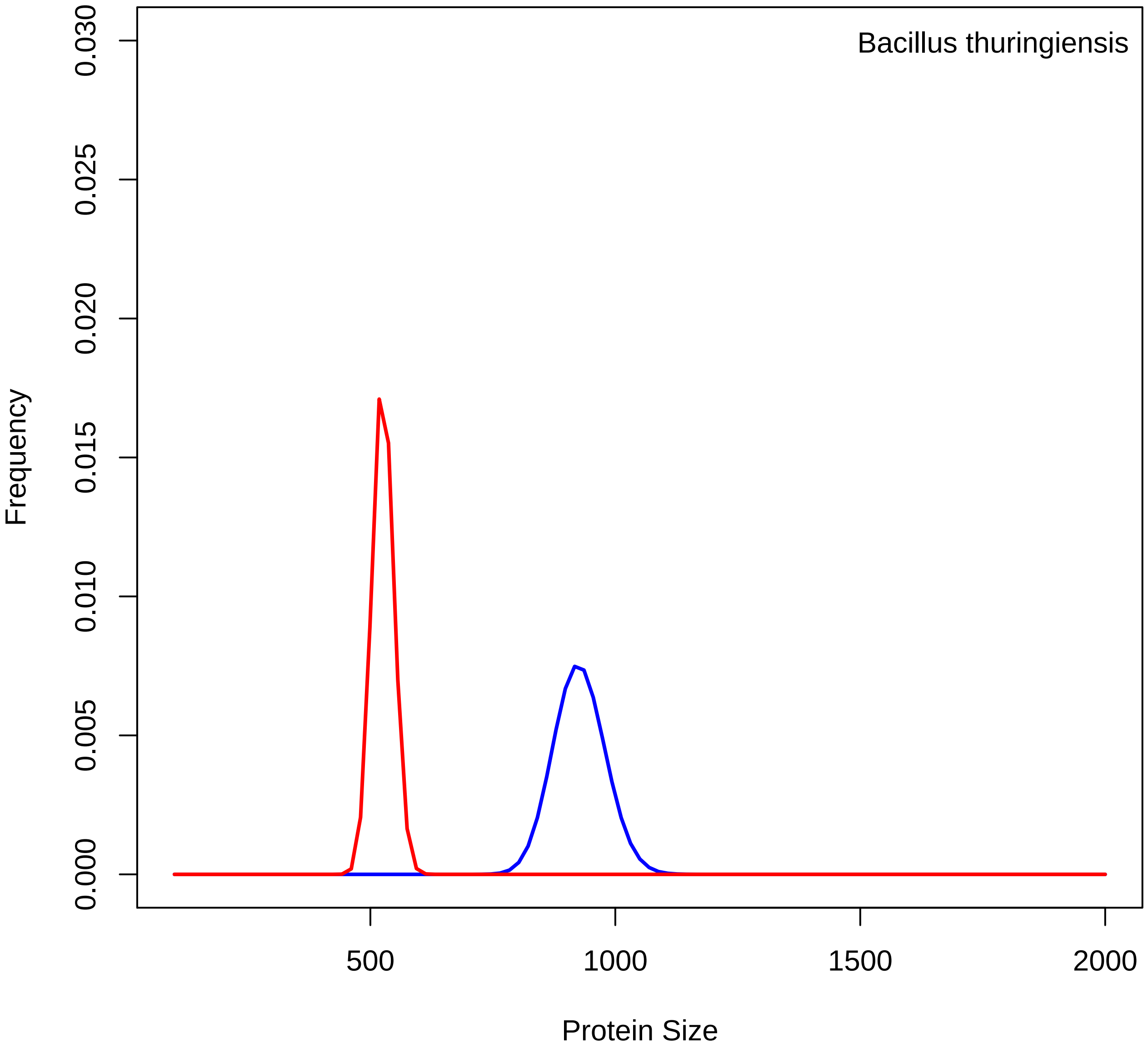

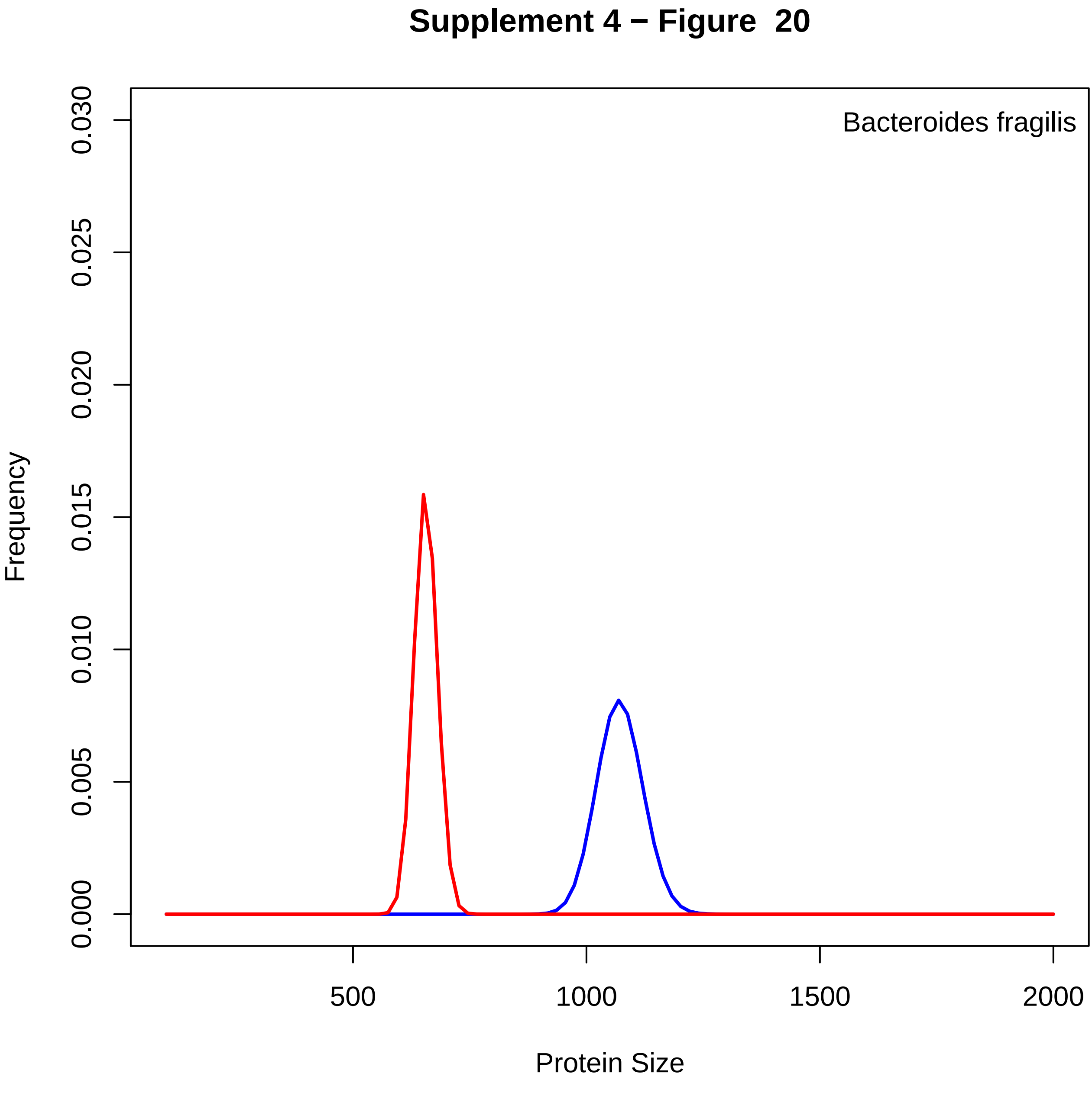
Supplement 4 − Figure 20
Bacteroides fragilis
Frequency
Protein Size
0.000
0.005
0.010
0.015
0.020
0.025
0.030
500
1000
1500
2000

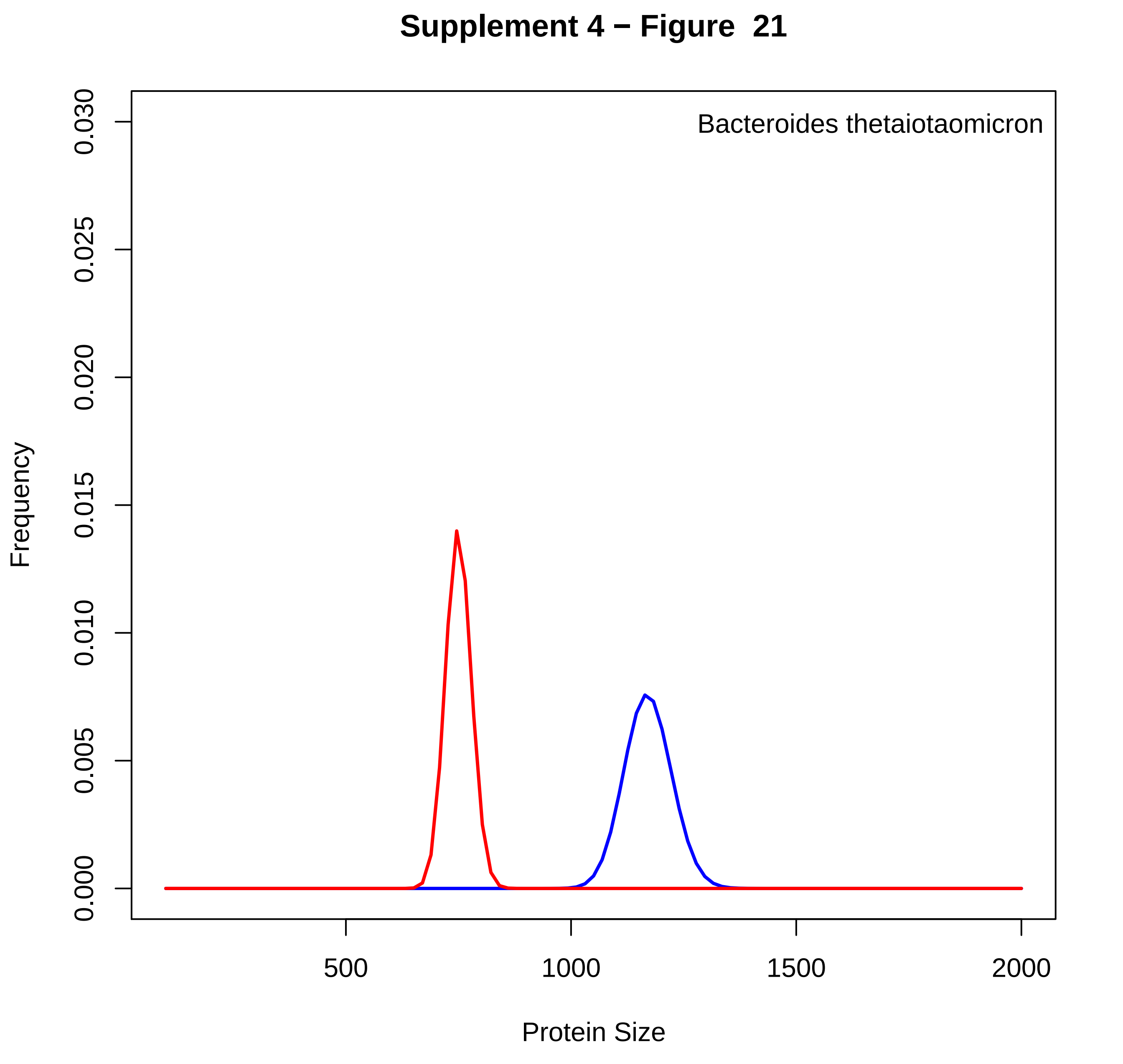
Supplement 4 − Figure 21
Bacteroides thetaiotaomicron
Frequency
0.000
0.005
0.010
0.015
0.020
0.025
0.030
500
1000
1500
2000
Protein Size

Supplement 4 − Figure 22

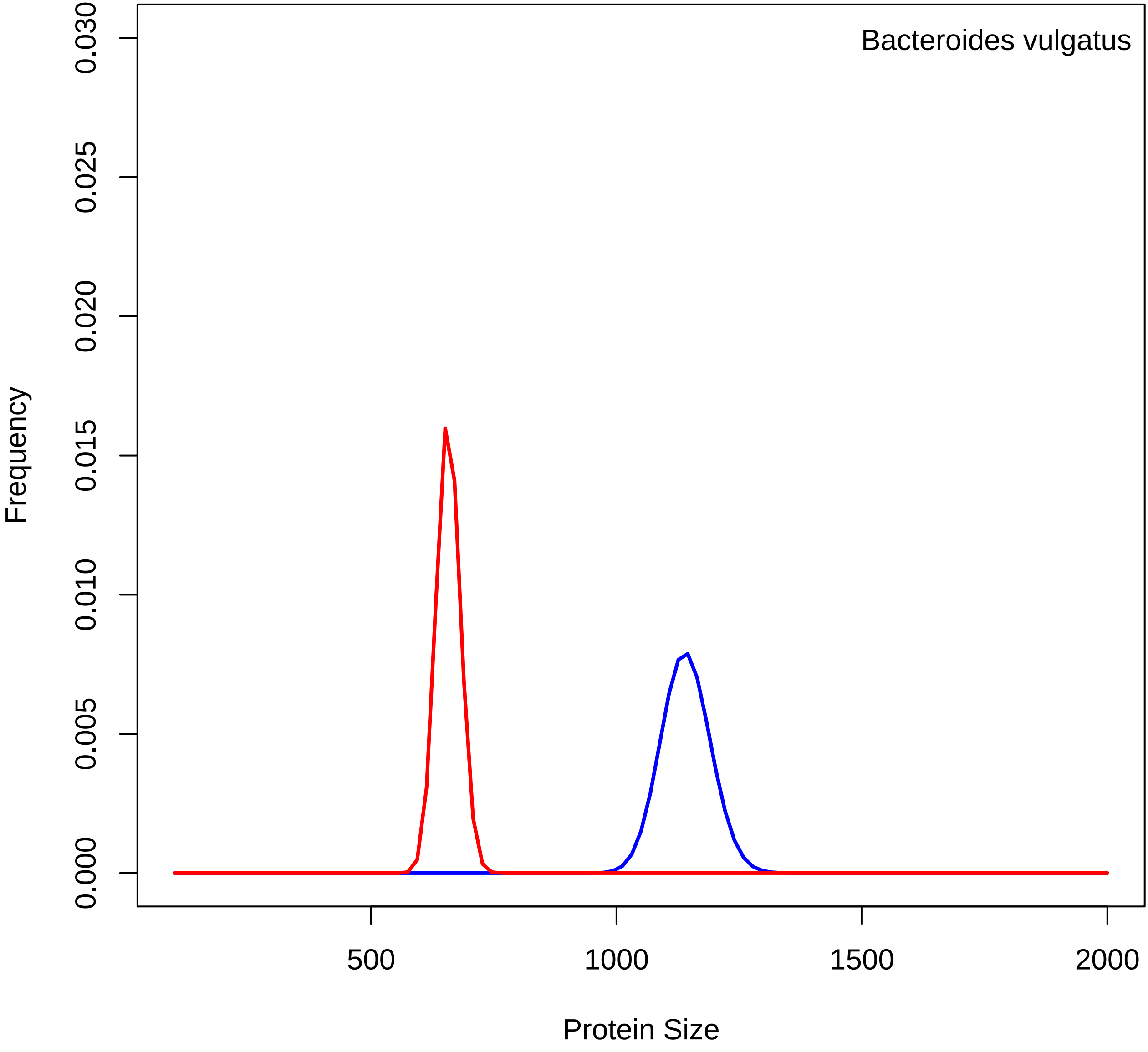

Supplement 4 − Figure 23

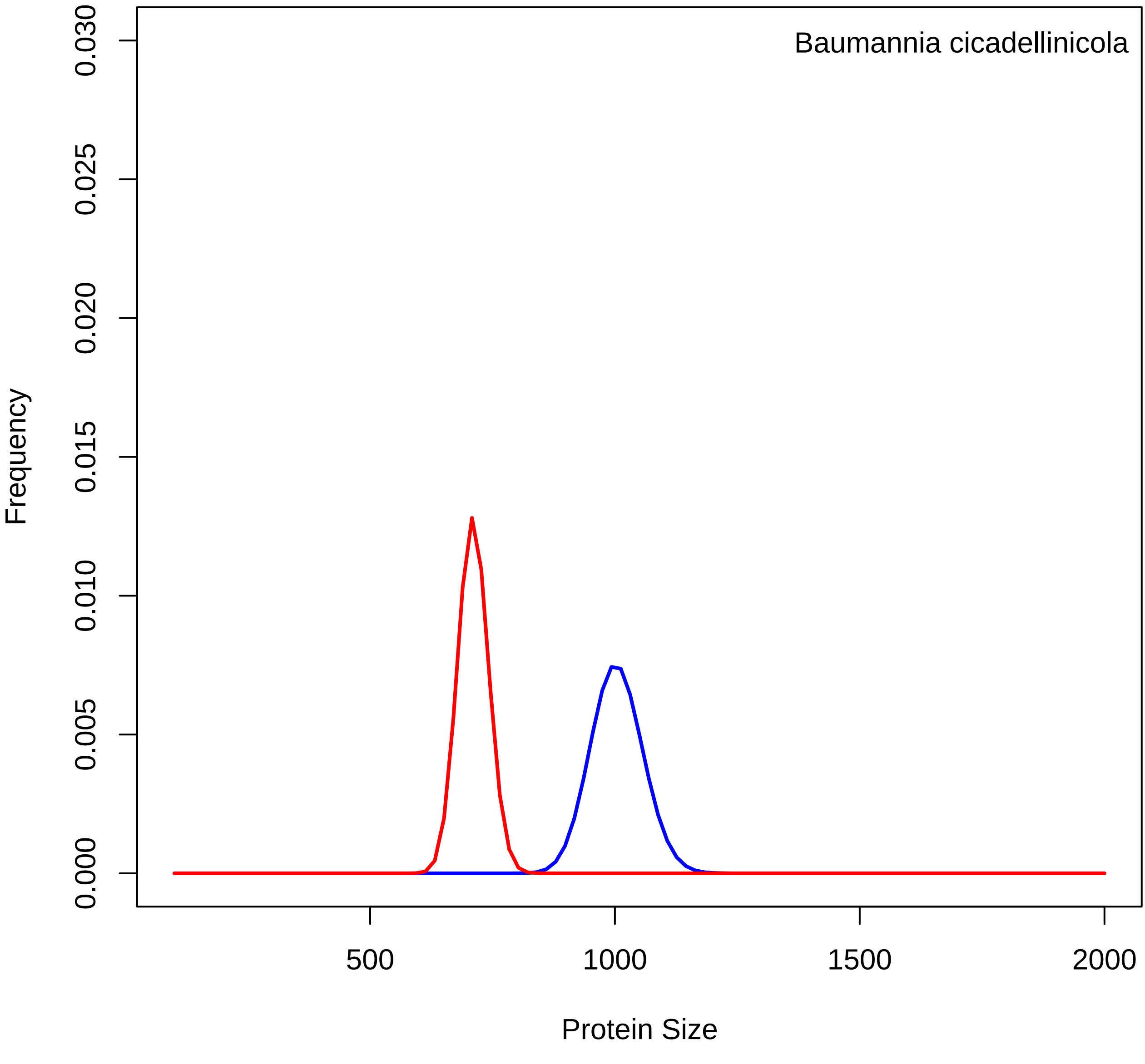

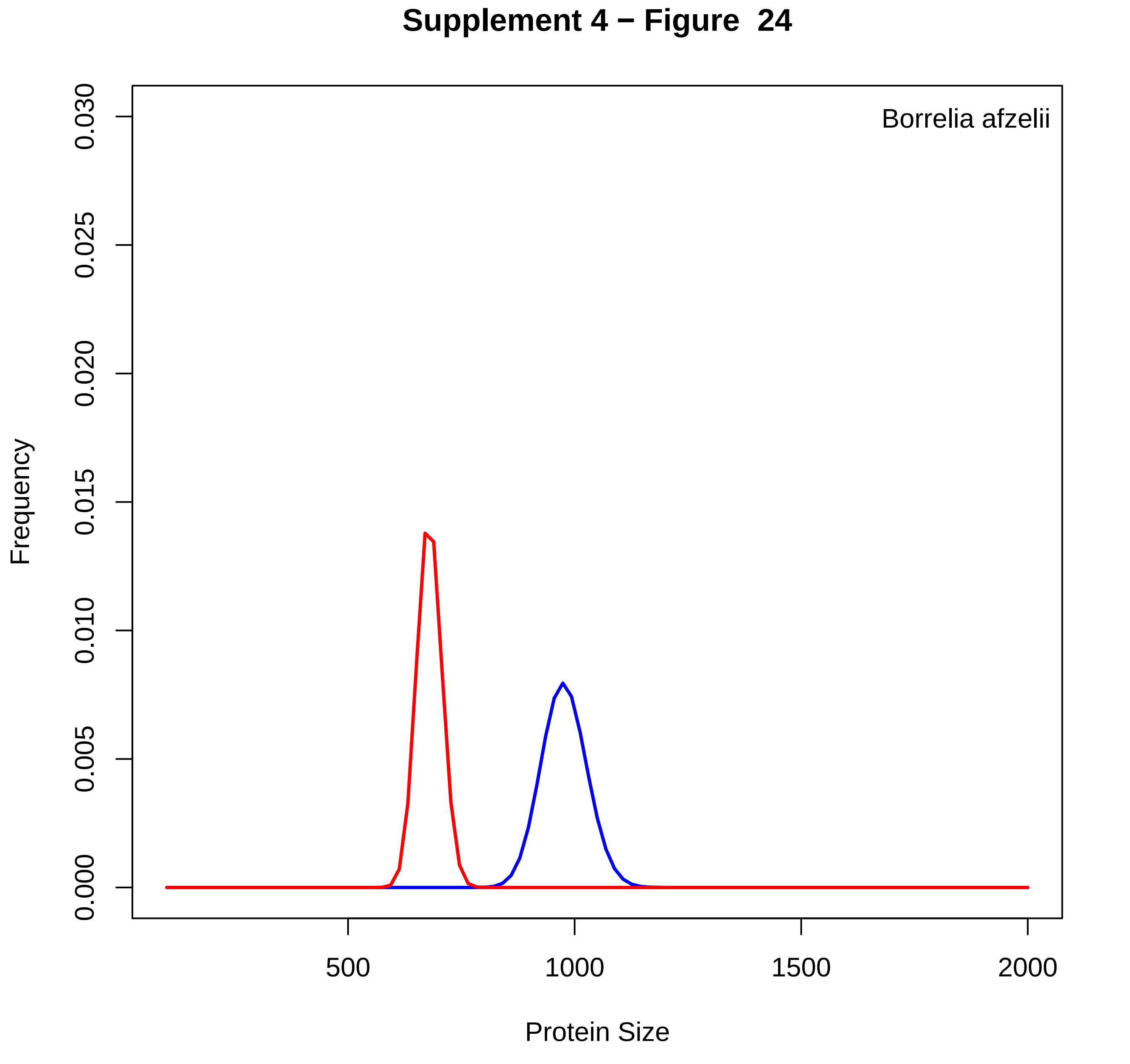
Supplement 4 − Figure 24
Borrelia afzelii
Frequency
0.000
0.005
0.010
0.015
0.020
0.025
0.030
500
1000
1500
2000
Protein Size

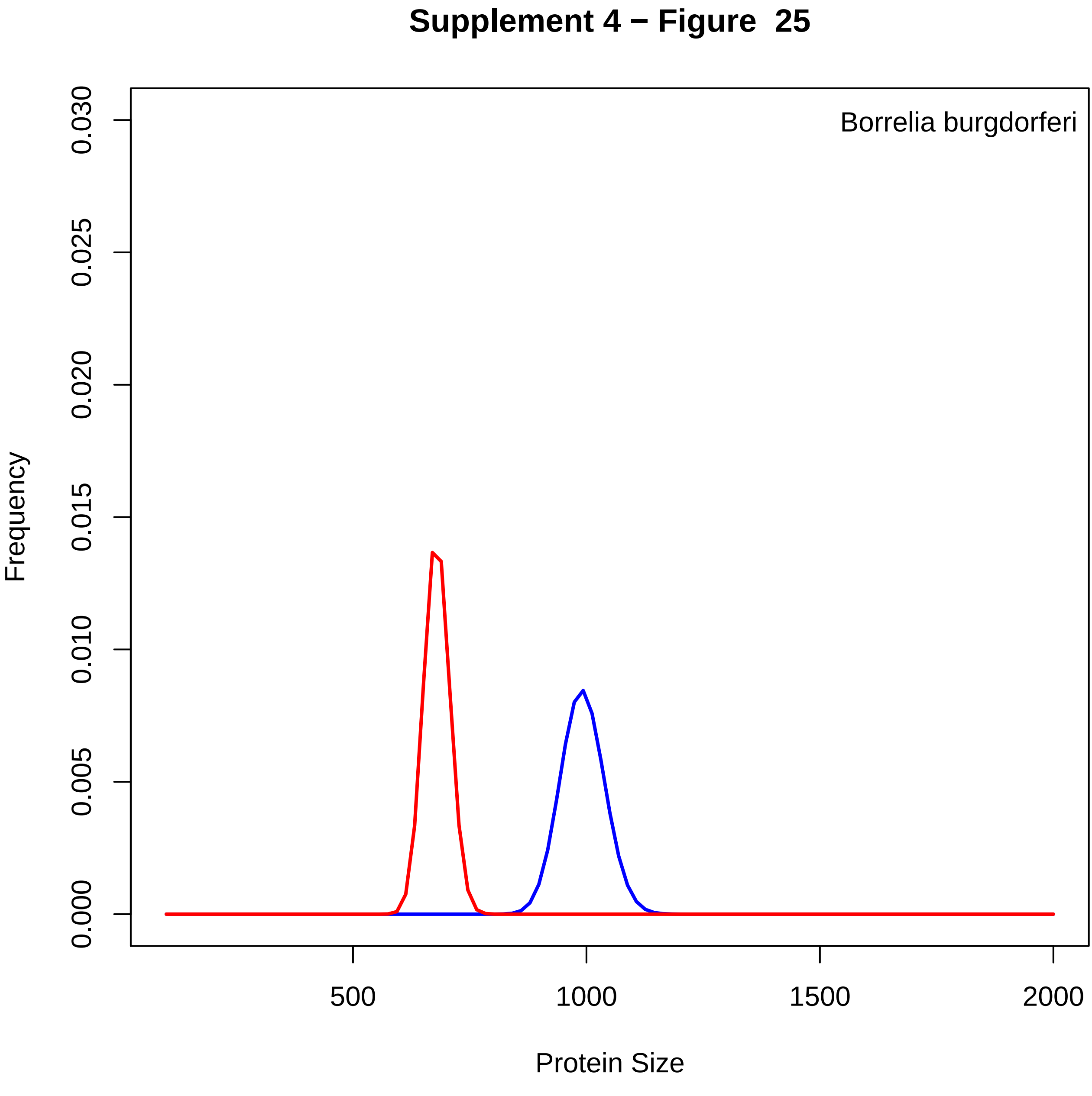
Supplement 4 − Figure 25
Borrelia burgdorferi
Frequency
0.000
0.005
0.010
0.015
0.020
0.025
0.030
500
1000
1500
2000
Protein Size

Supplement 4 − Figure 26

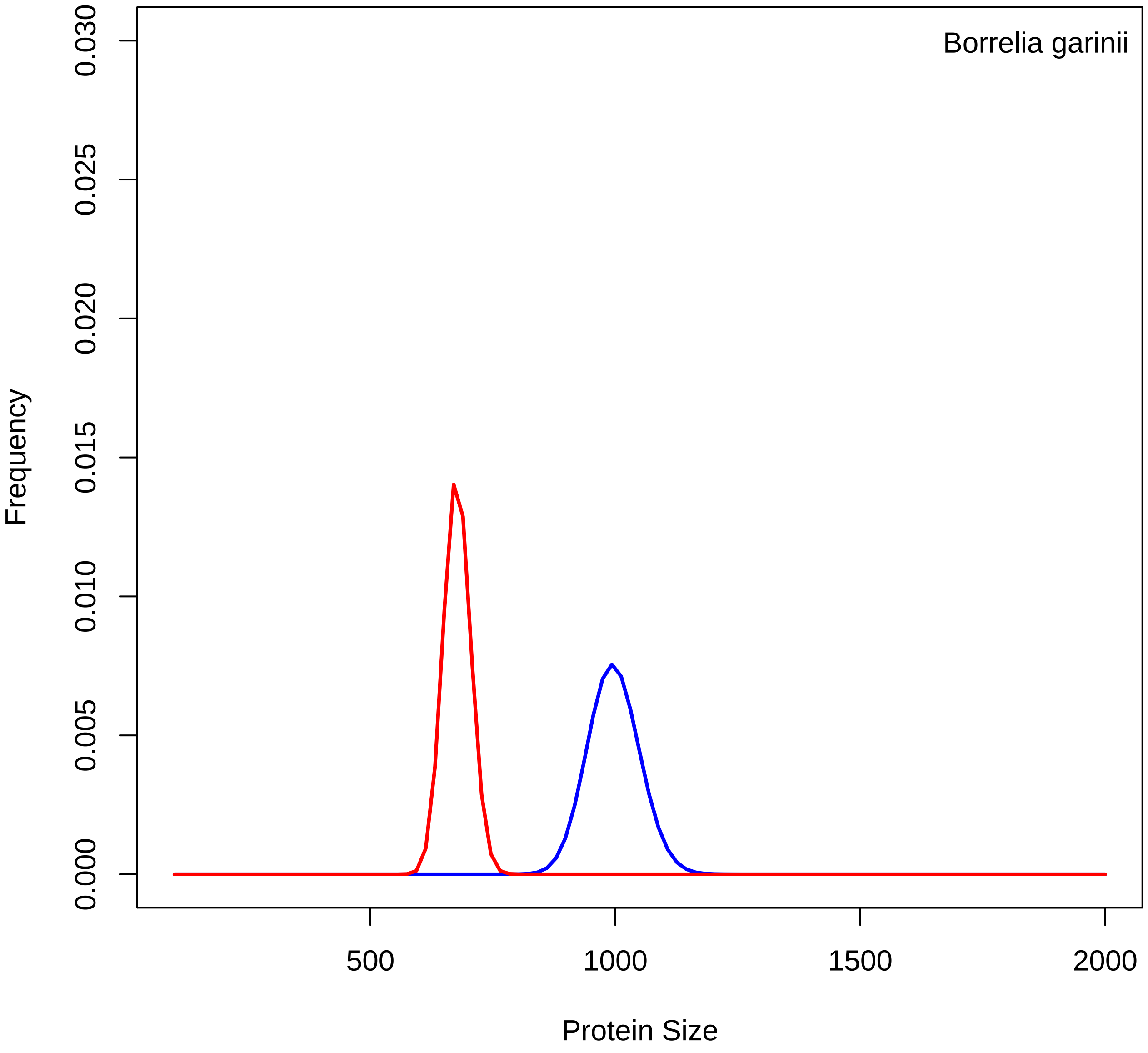

Supplement 4 − Figure 27

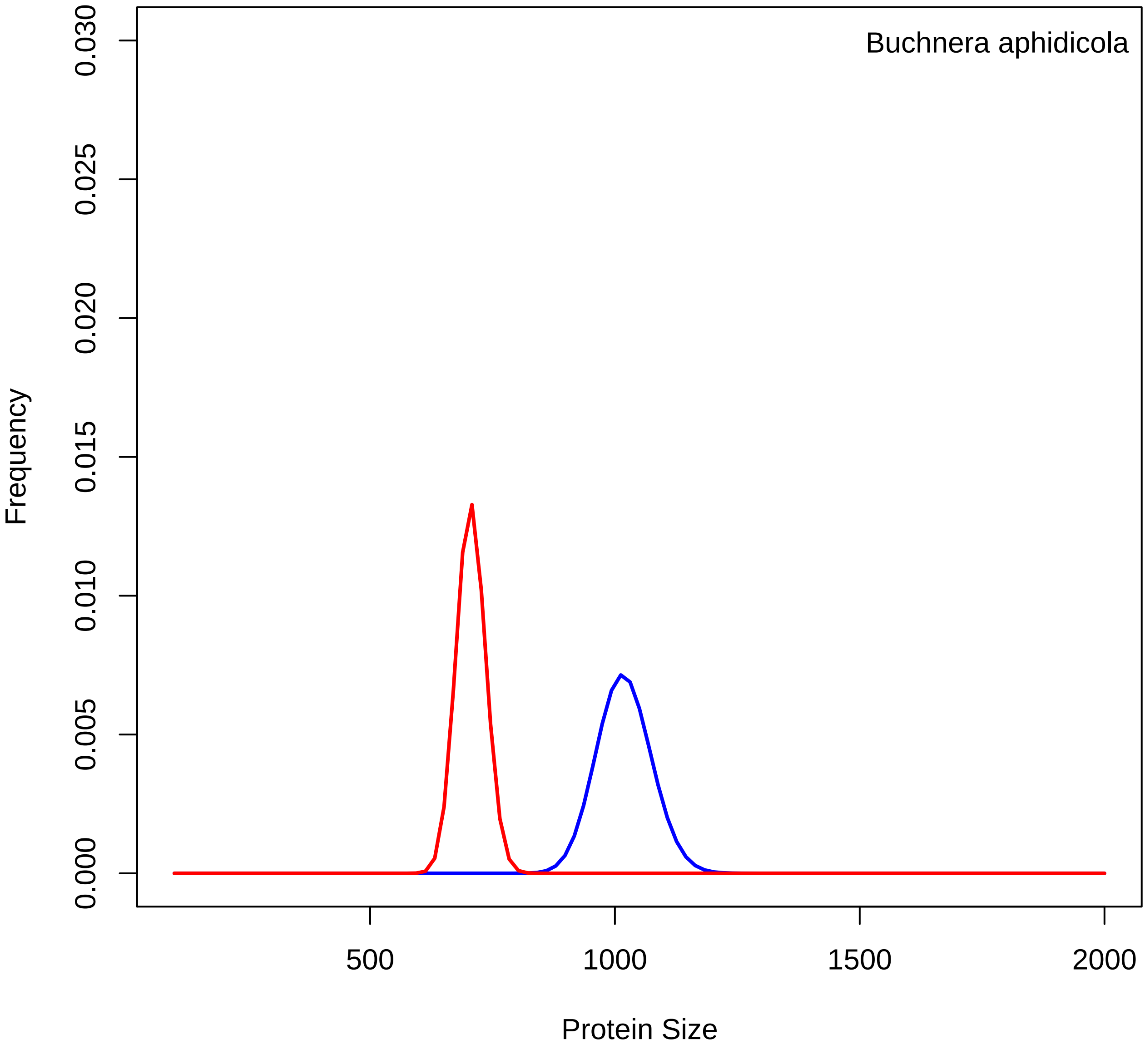

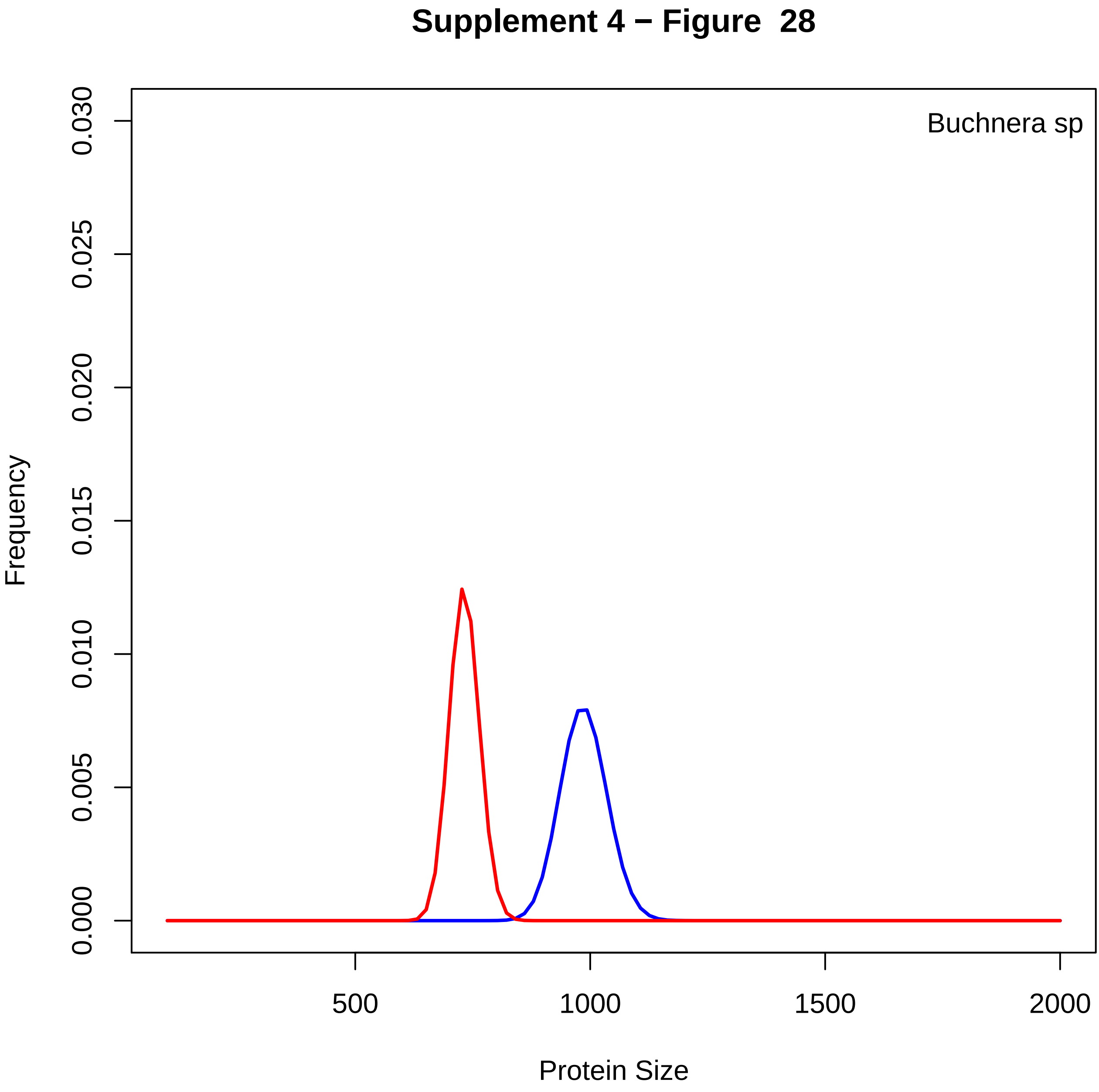

Supplement 4 − Figure 28
Buchnera sp
Frequency
0.000
0.005
0.010
0.015
0.020
0.025
0.030
500
1000
1500
2000
Protein Size

Supplement 4 − Figure 29

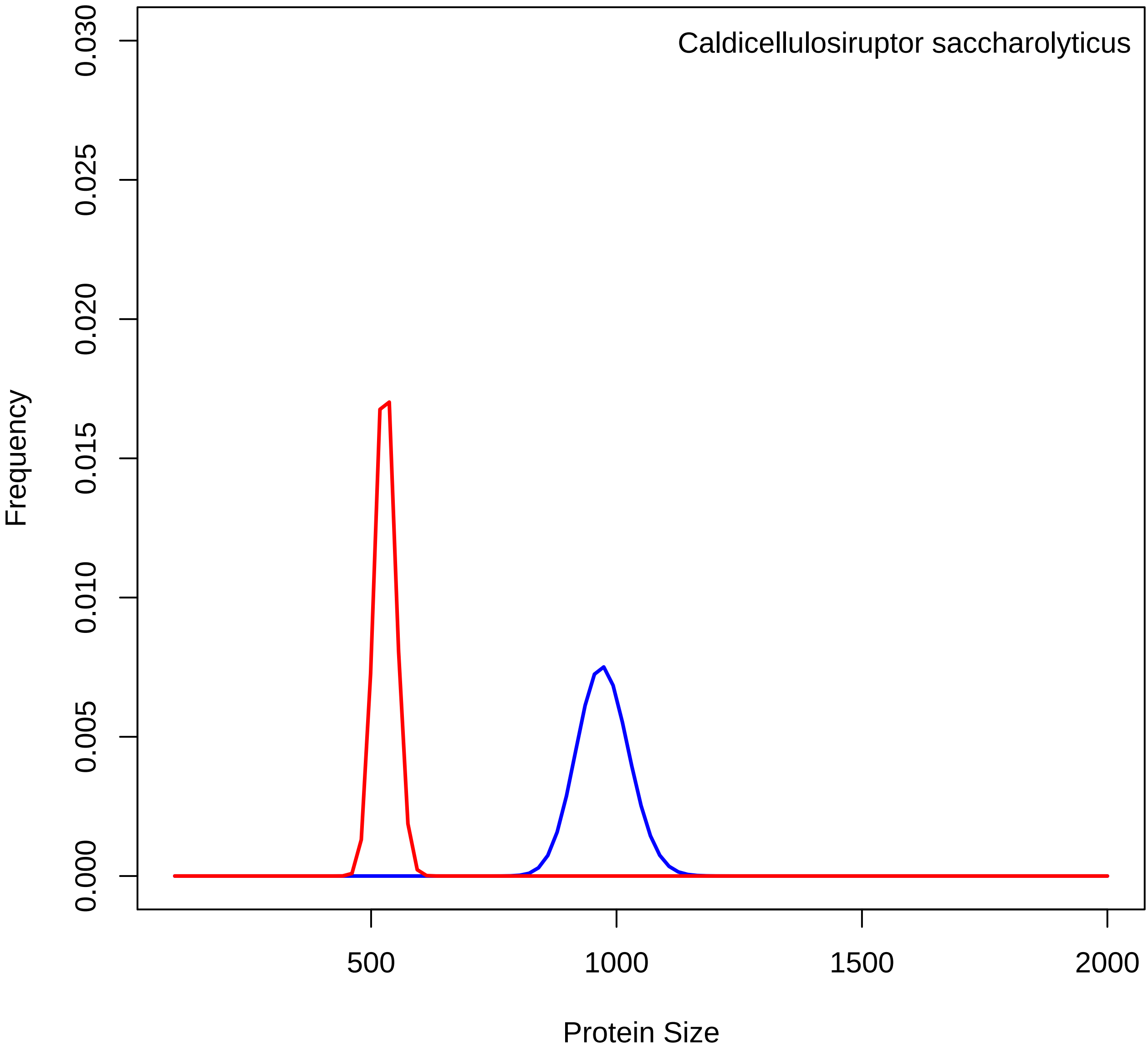

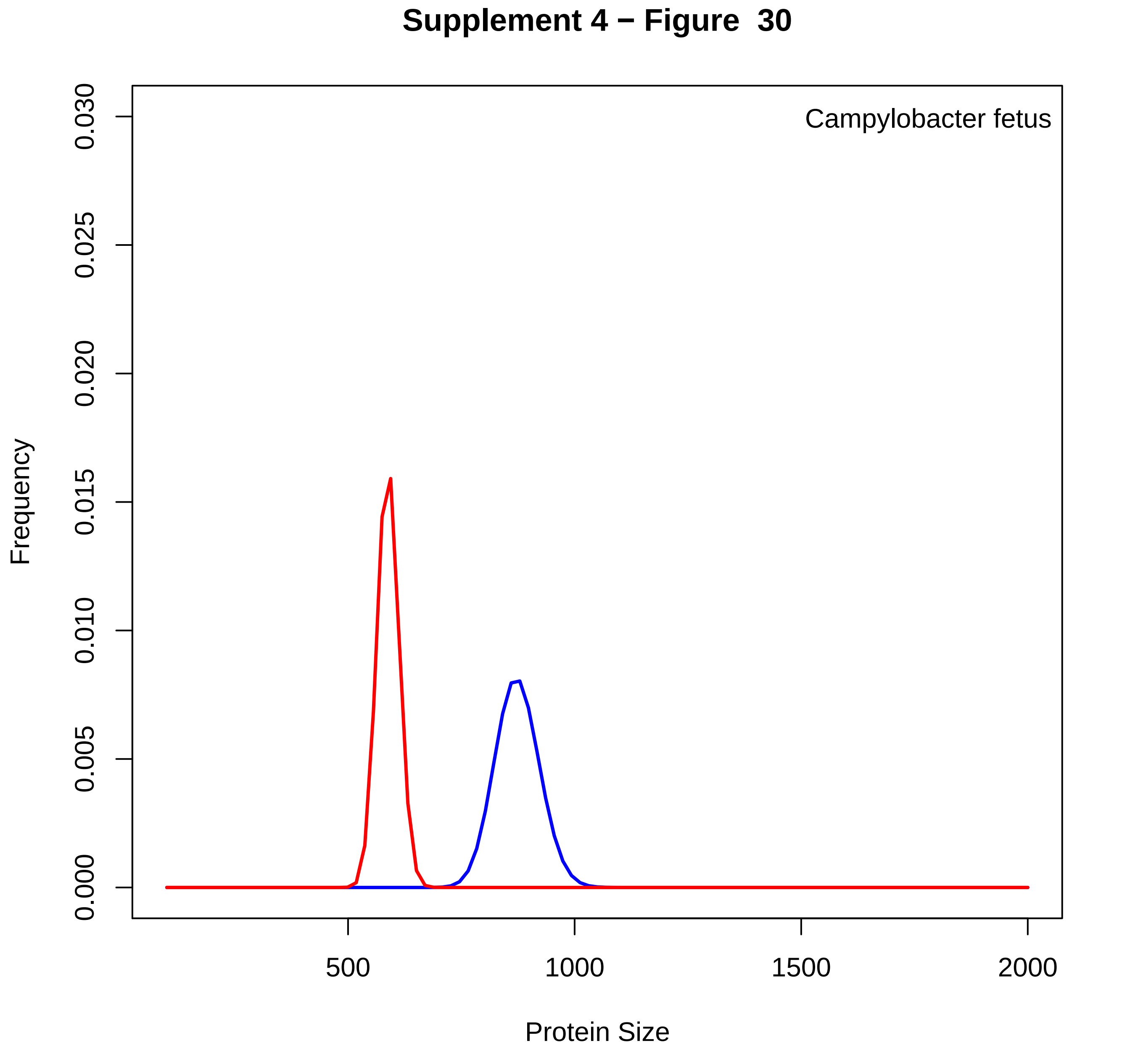
Supplement 4 − Figure 30
Campylobacter fetus
Frequency
0.000
0.005
0.010
0.015
0.020
0.025
0.030
500
1000
1500
2000
Protein Size

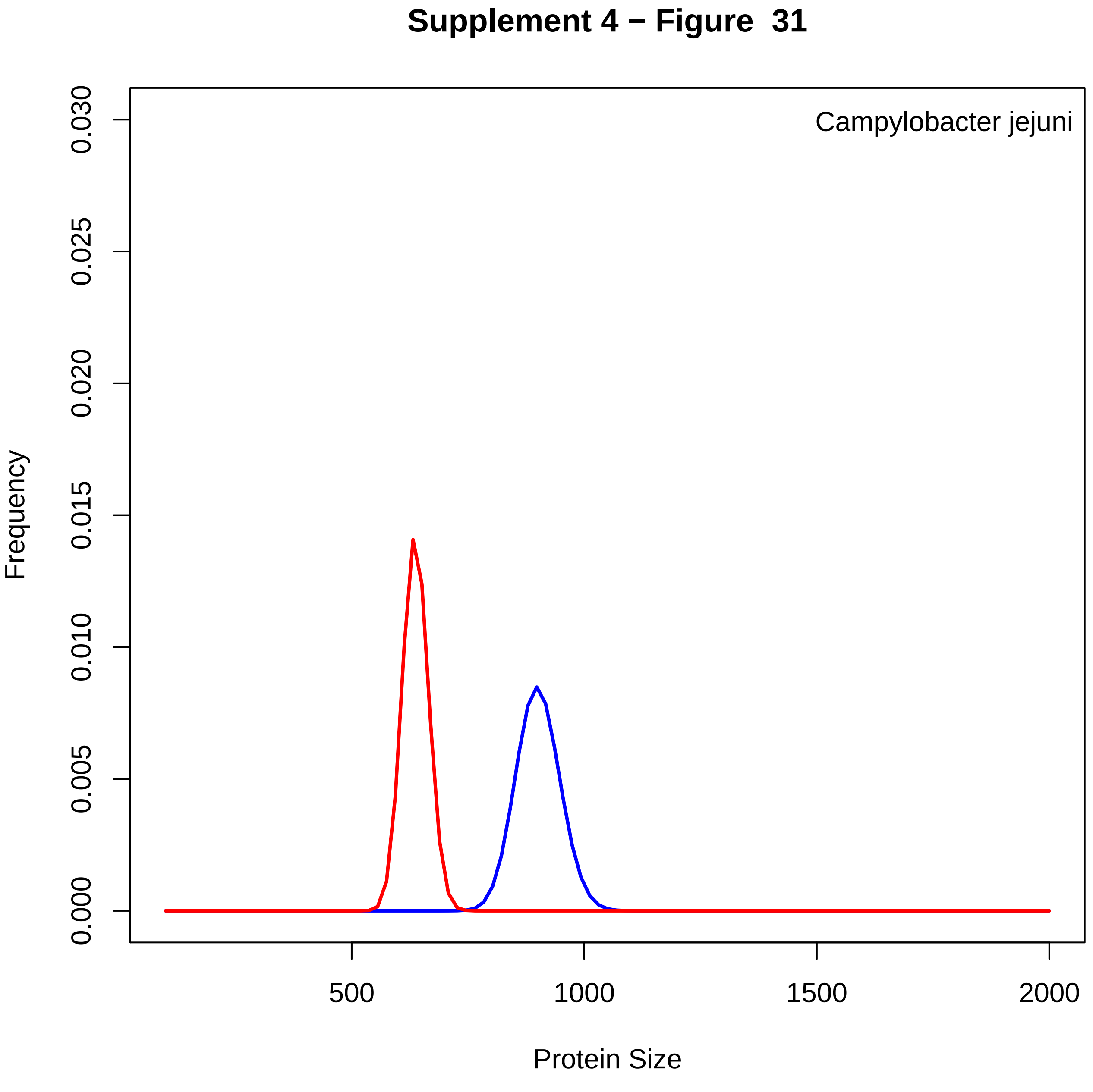
Supplement 4 − Figure 31
Campylobacter jejuni
Frequency
0.000
0.005
0.010
0.015
0.020
0.025
0.030
500
1000
1500
2000
Protein Size

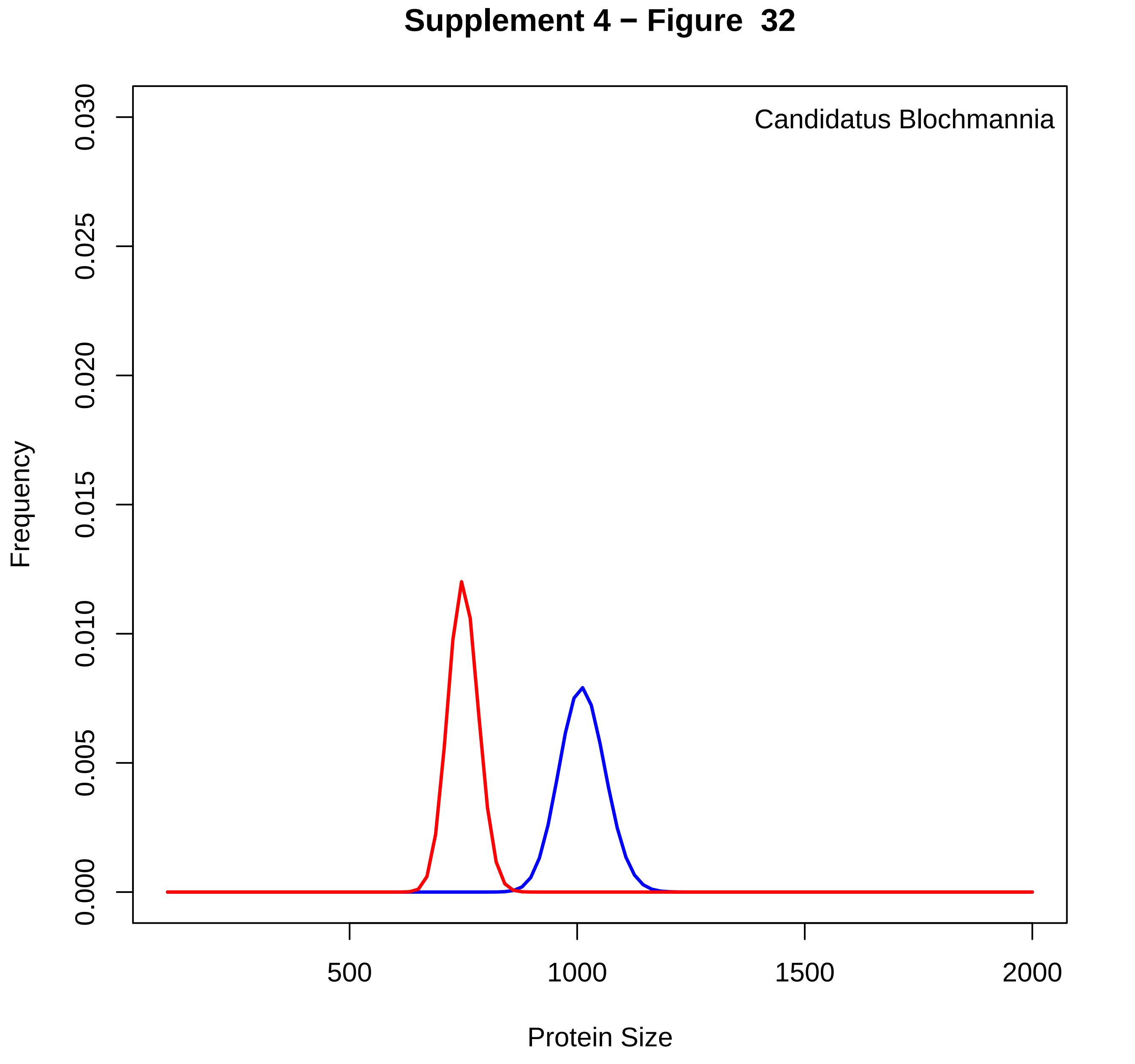

Supplement 4 − Figure 32
Candidatus Blochmannia
Frequency
0.000
0.005
0.010
0.015
0.020
0.025
0.030
500
1000
1500
2000
Protein Size

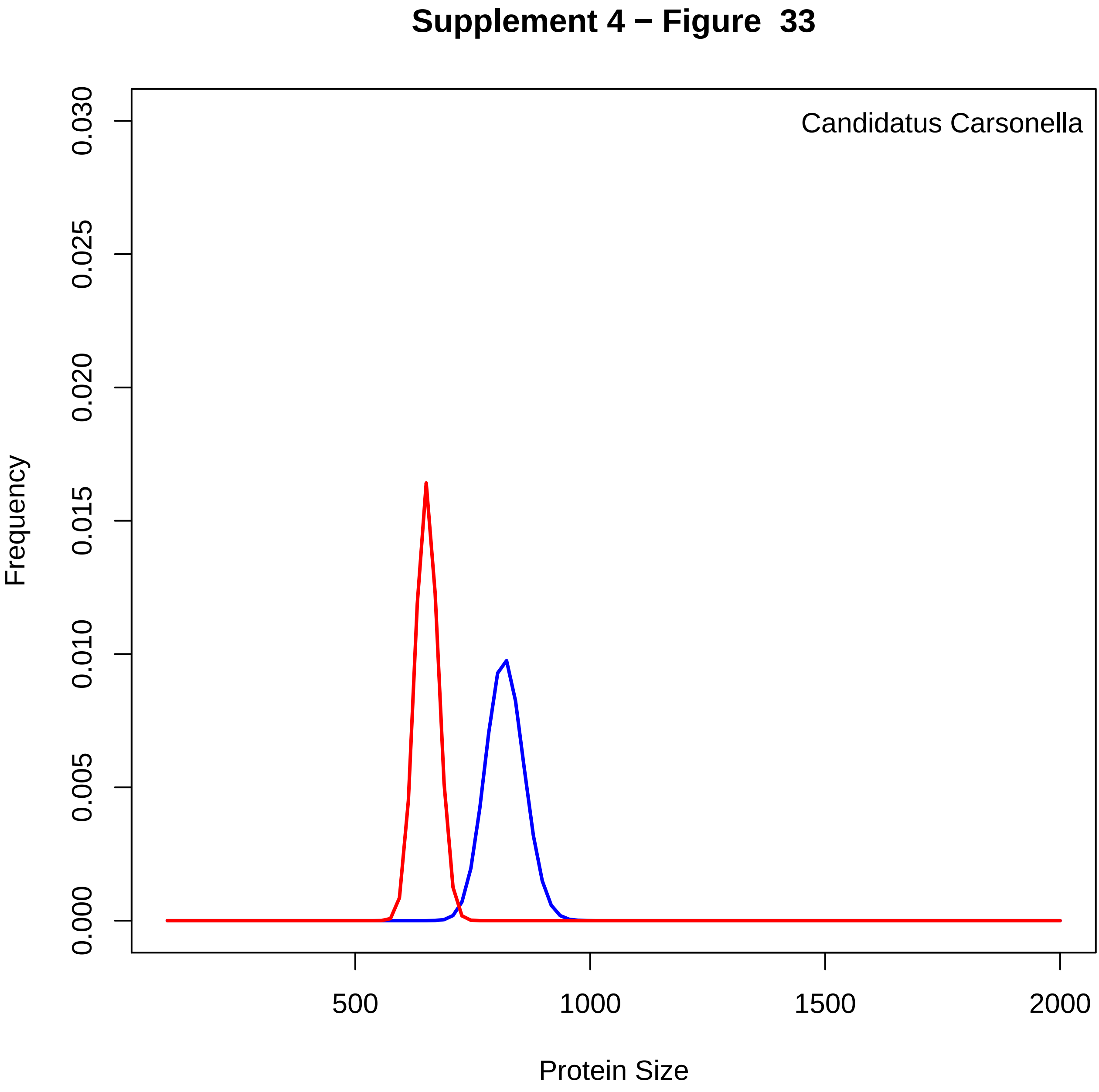
Supplement 4 − Figure 33
Candidatus Carsonella
Frequency
0.000
0.005
0.010
0.015
0.020
0.025
0.030
500
1000
1500
2000
Protein Size

Supplement 4 − Figure 34

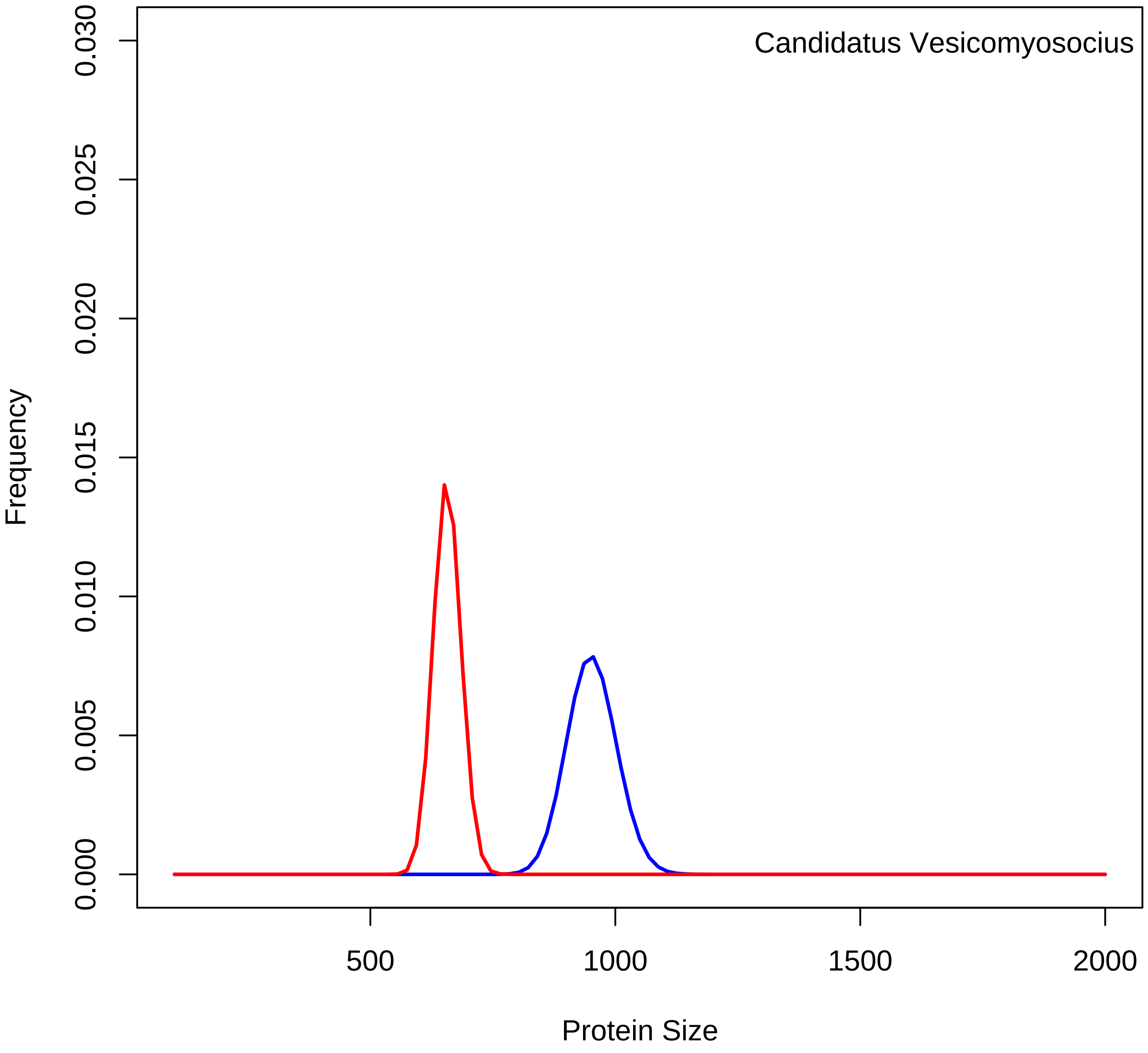

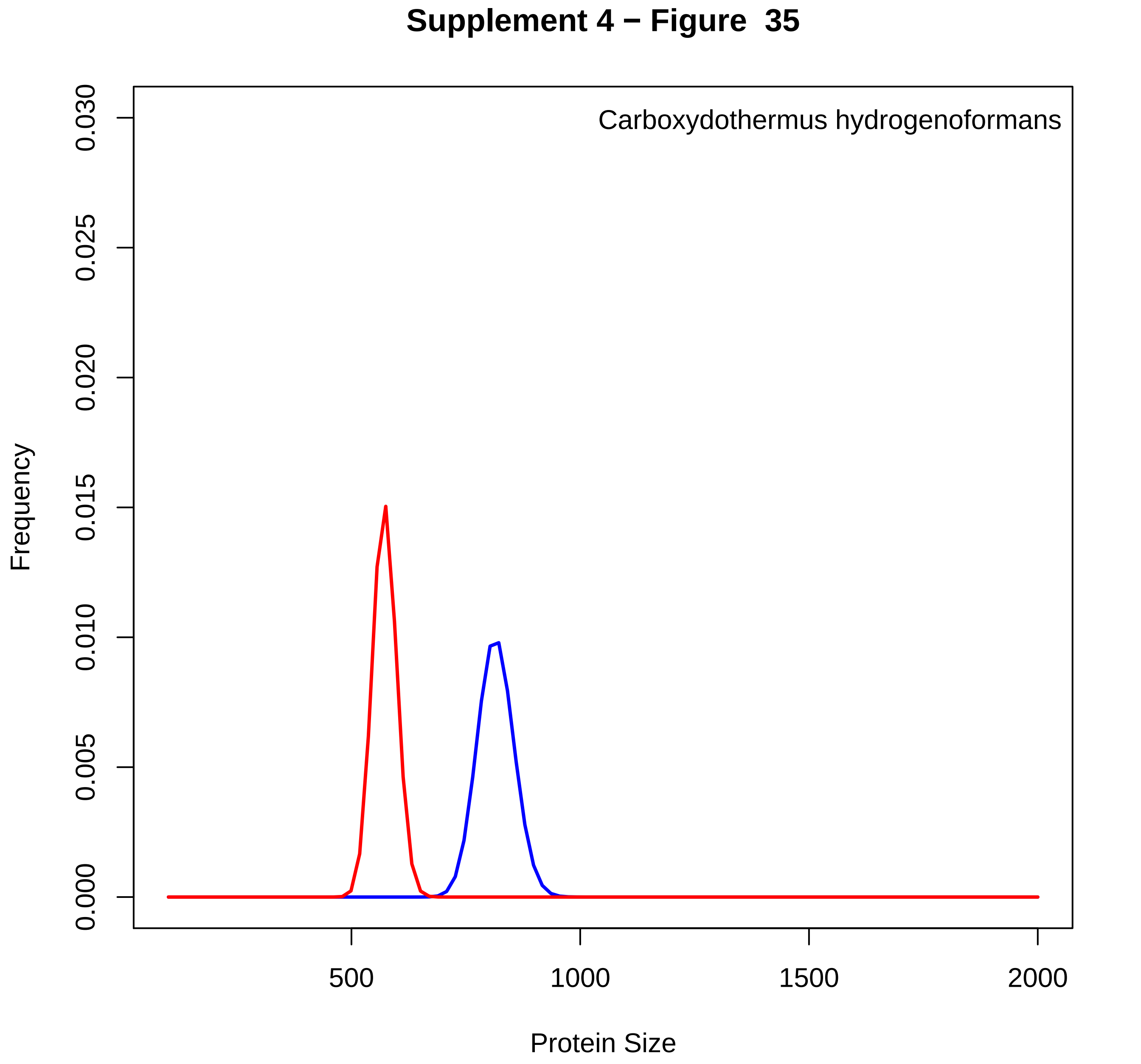
Supplement 4 − Figure 35
Carboxydothermus hydrogenoformans
Frequency
0.000
0.005
0.010
0.015
0.020
0.025
0.030
500
1000
1500
2000
Protein Size

Supplement 4 − Figure 36

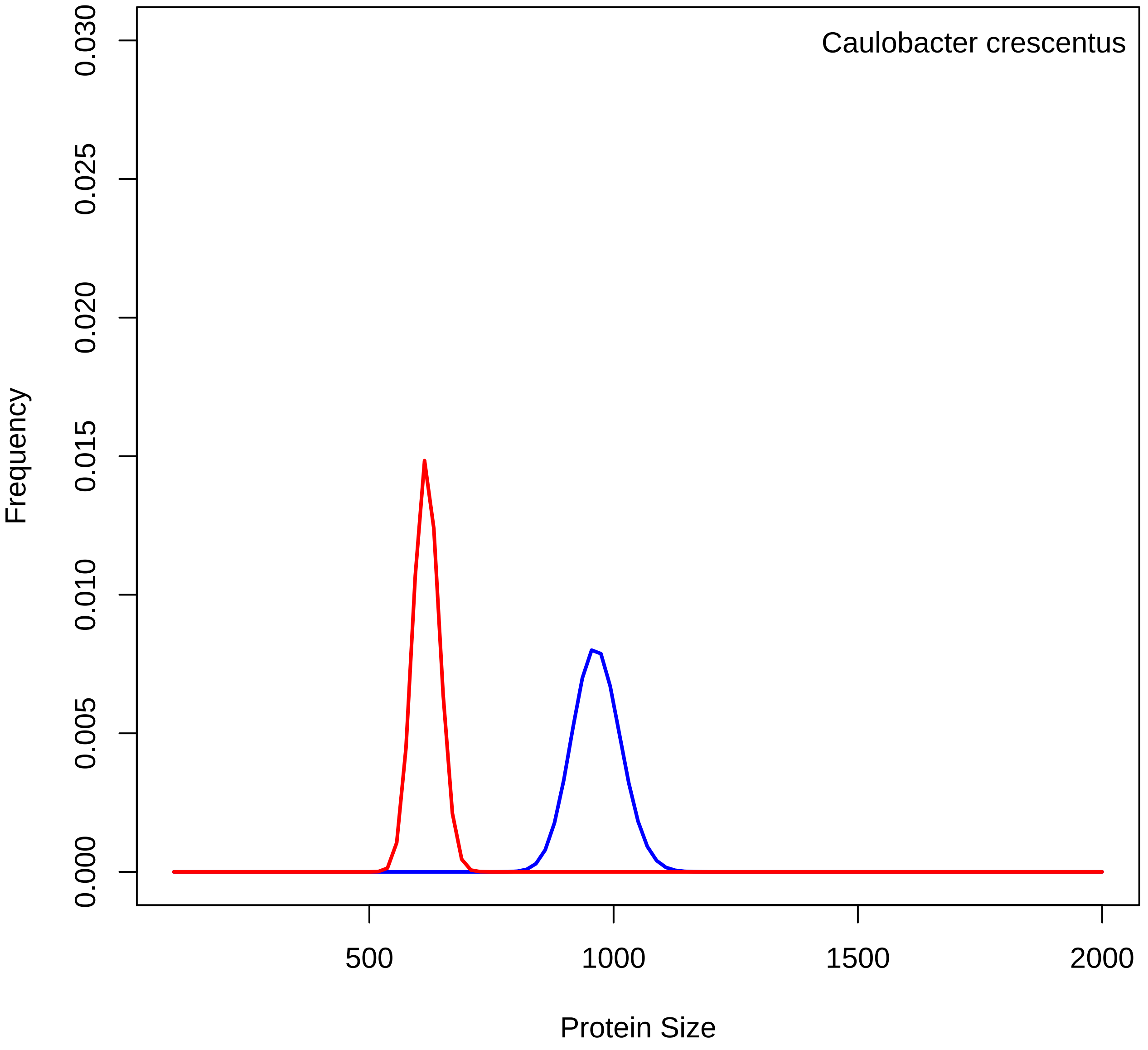

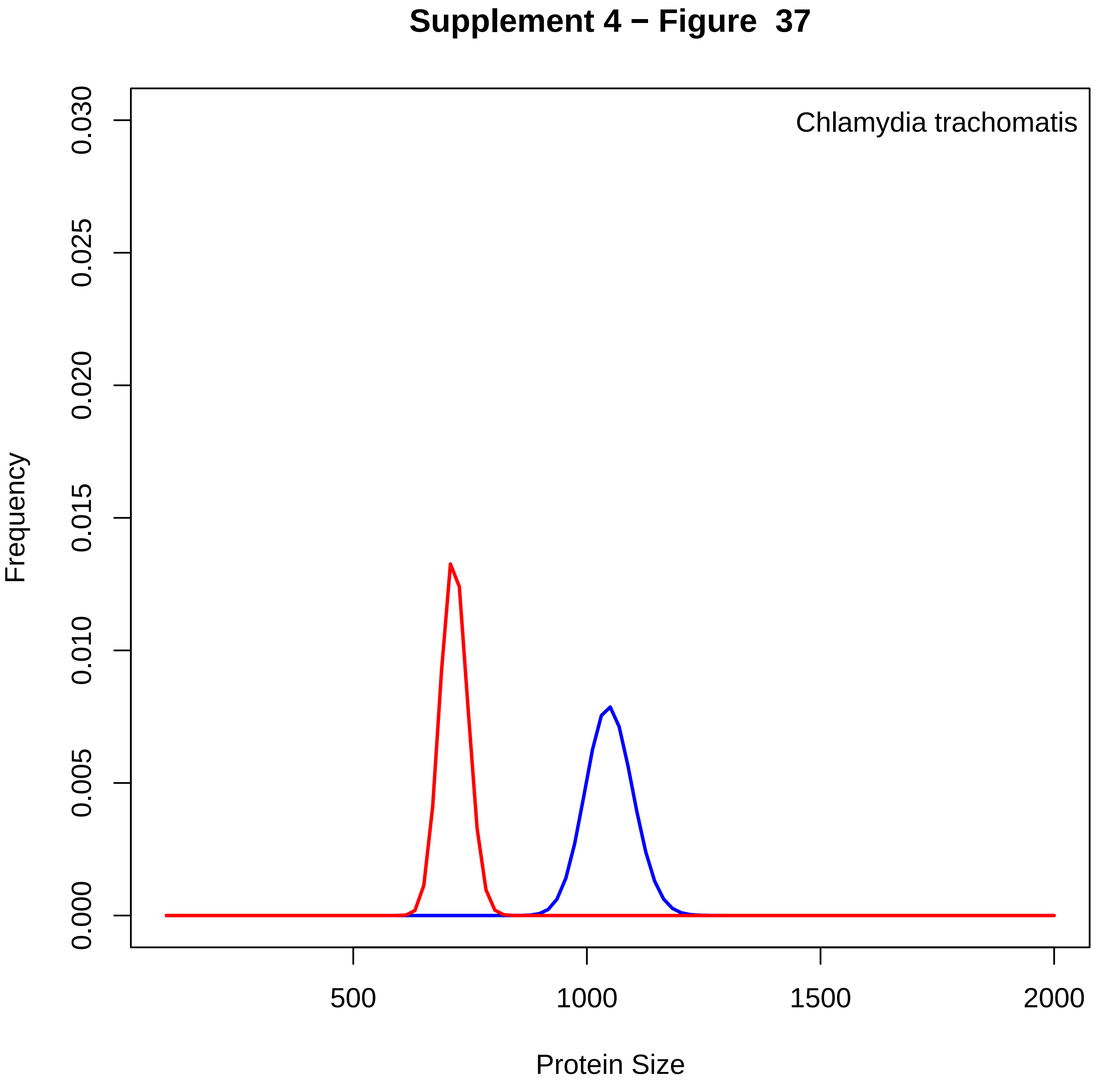

Supplement 4 − Figure 37
Chlamydia trachomatis
Frequency
0.000
0.005
0.010
0.015
0.020
0.025
0.030
500
1000
1500
2000
Protein Size

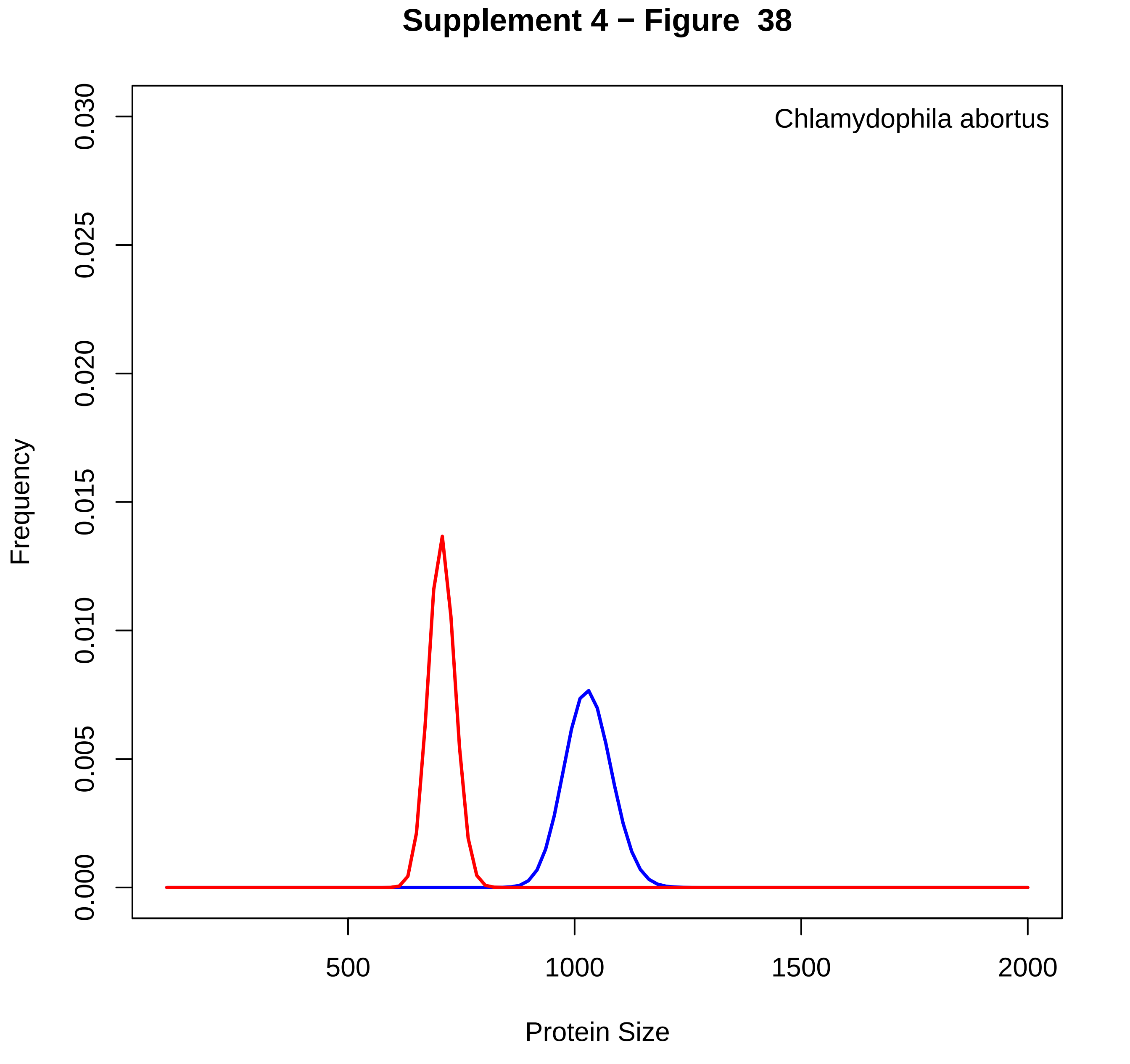
Supplement 4 − Figure 38
Chlamydophila abortus
Frequency
0.000
0.005
0.010
0.015
0.020
0.025
0.030
500
1000
1500
2000
Protein Size

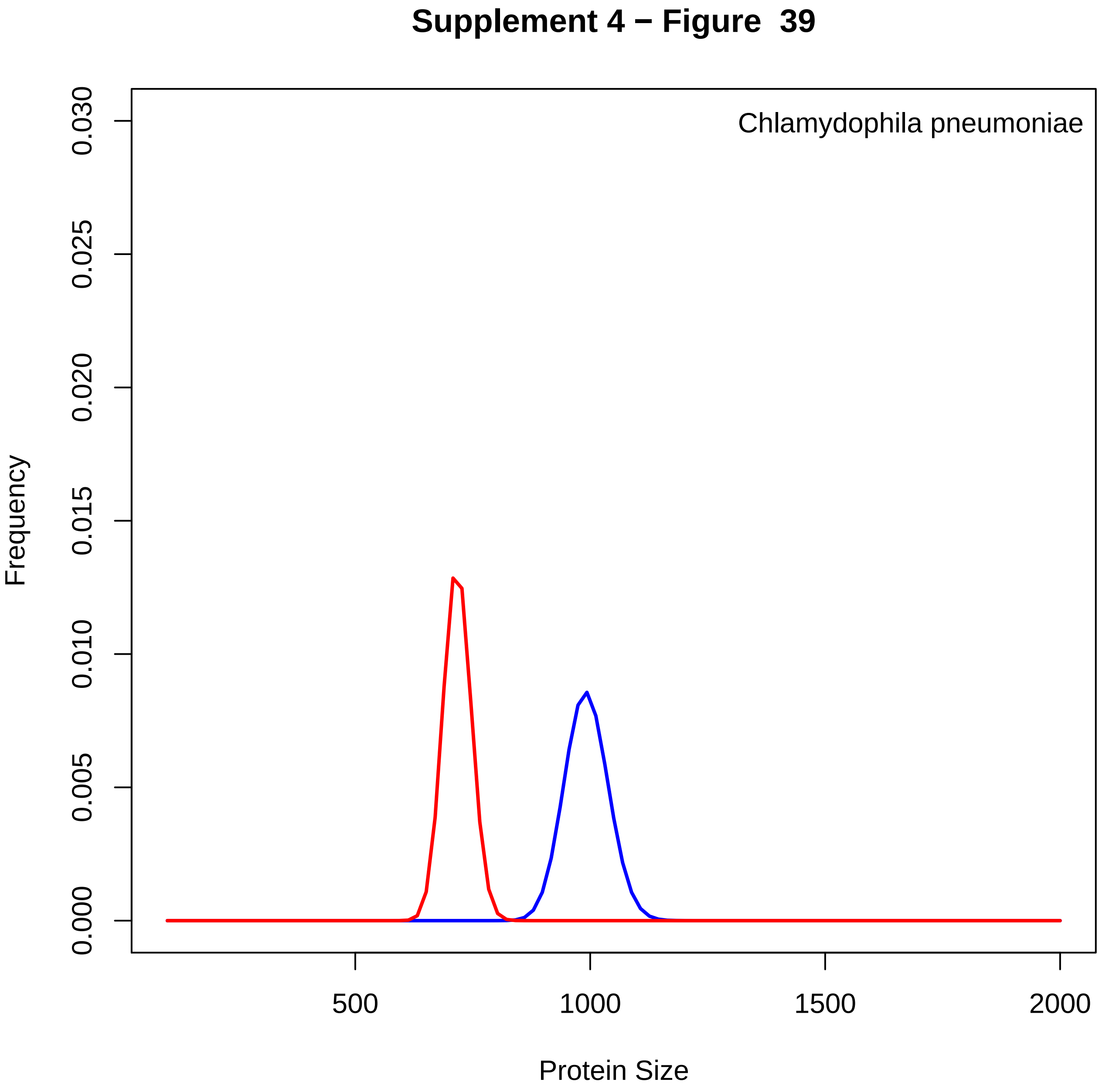
Supplement 4 − Figure 39
Chlamydophila pneumoniae
Frequency
0.000
0.005
0.010
0.015
0.020
0.025
0.030
500
1000
1500
2000
Protein Size

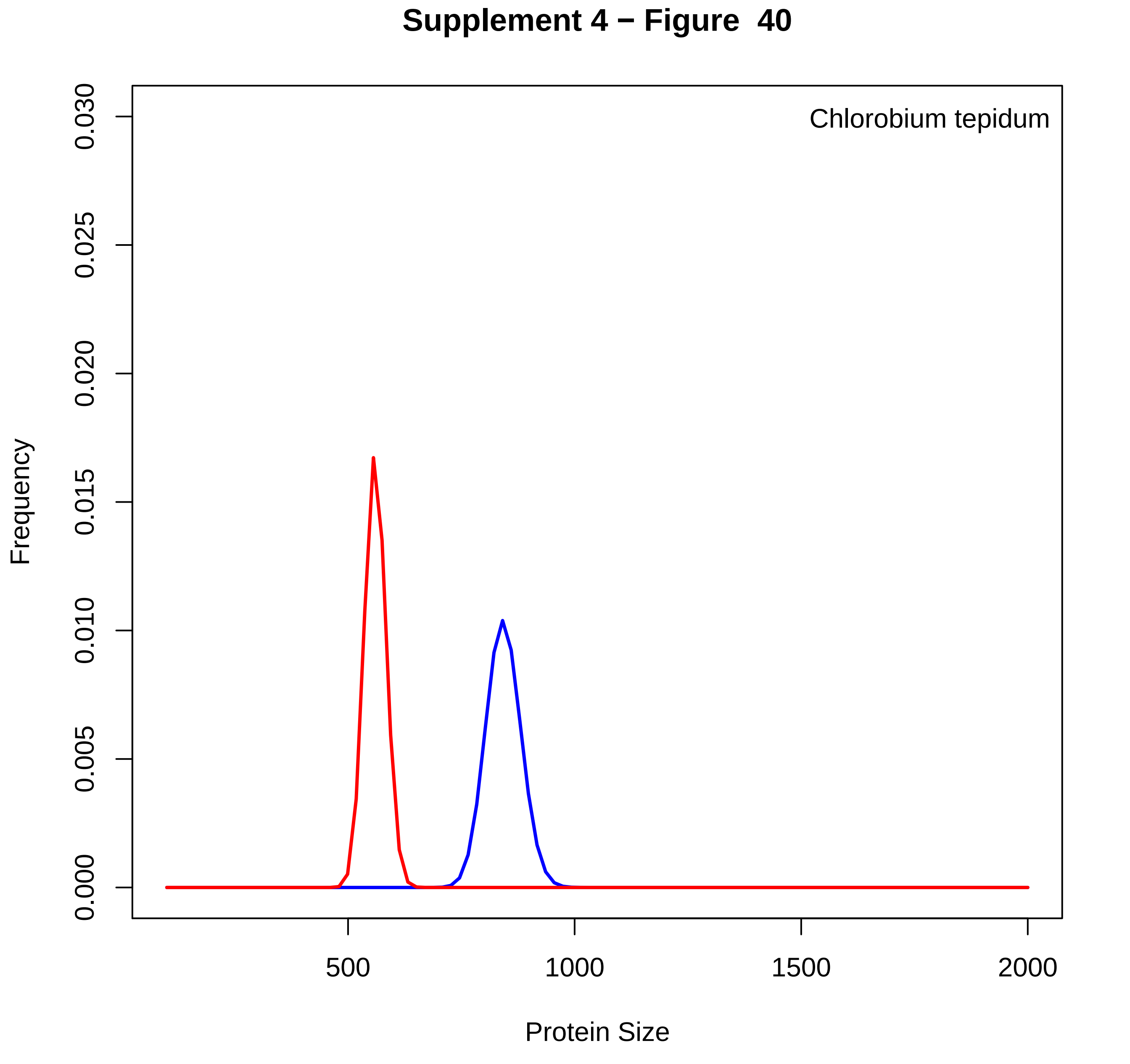
Supplement 4 − Figure 40
Chlorobium tepidum
Frequency
0.000
0.005
0.010
0.015
0.020
0.025
0.030
500
1000
1500
2000
Protein Size

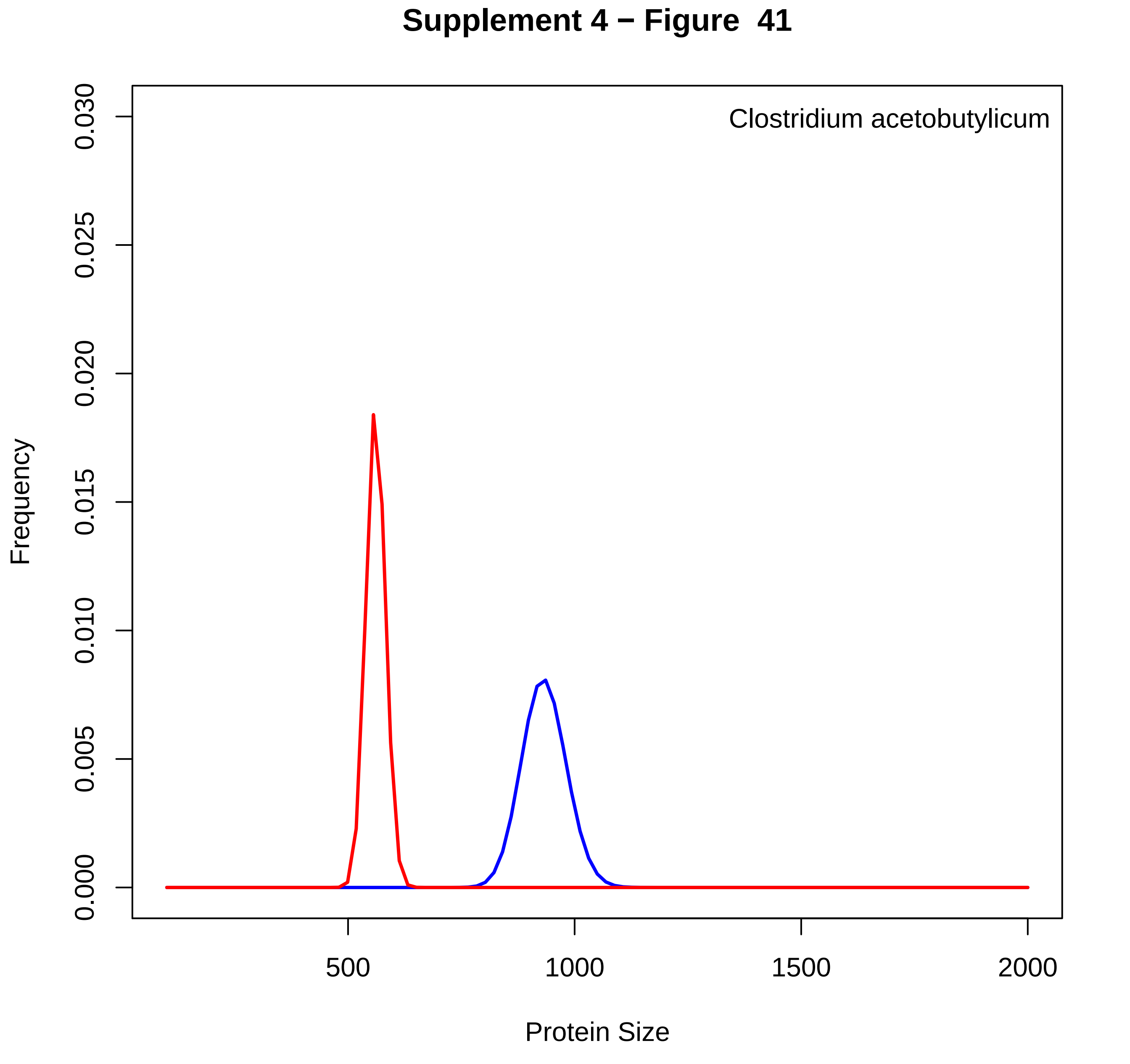
Supplement 4 − Figure 41
Clostridium acetobutylicum
Frequency
0.000
0.005
0.010
0.015
0.020
0.025
0.030
500
1000
1500
2000
Protein Size

Supplement 4 − Figure 42

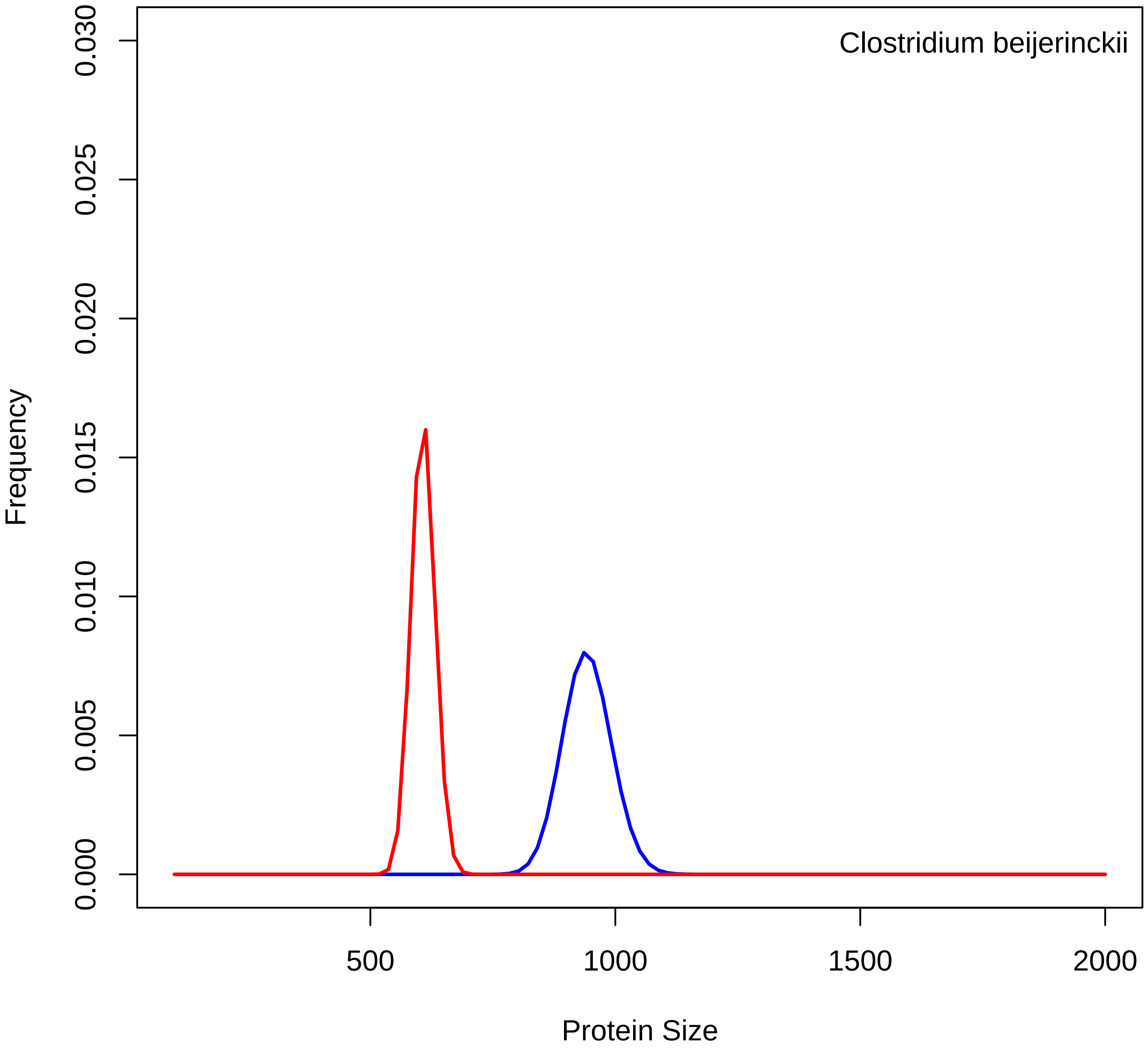

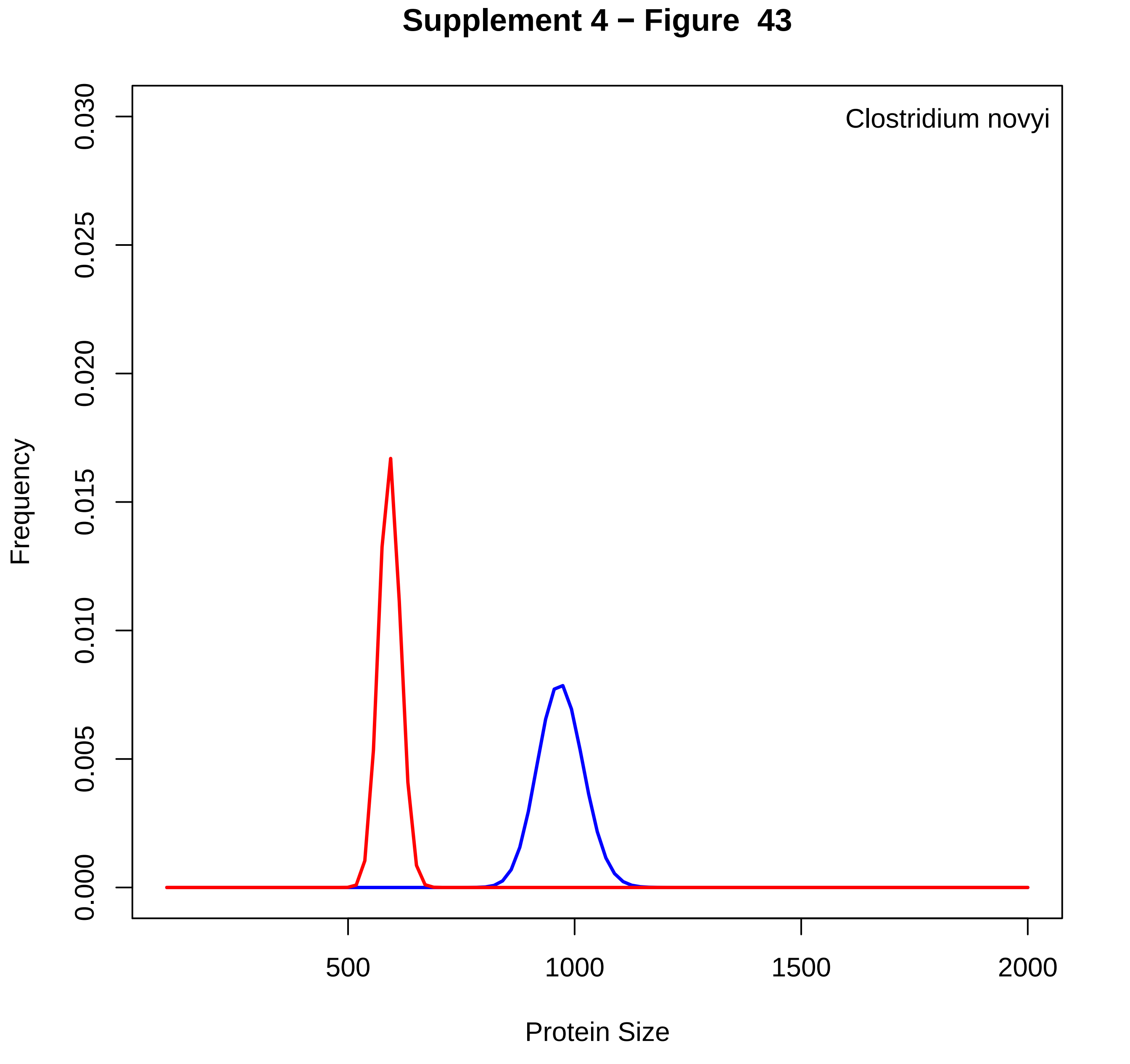
Supplement 4 − Figure 43
Clostridium novyi
Frequency
0.000
0.005
0.010
0.015
0.020
0.025
0.030
Protein Size
500
1000
1500
2000

Supplement 4 − Figure 44

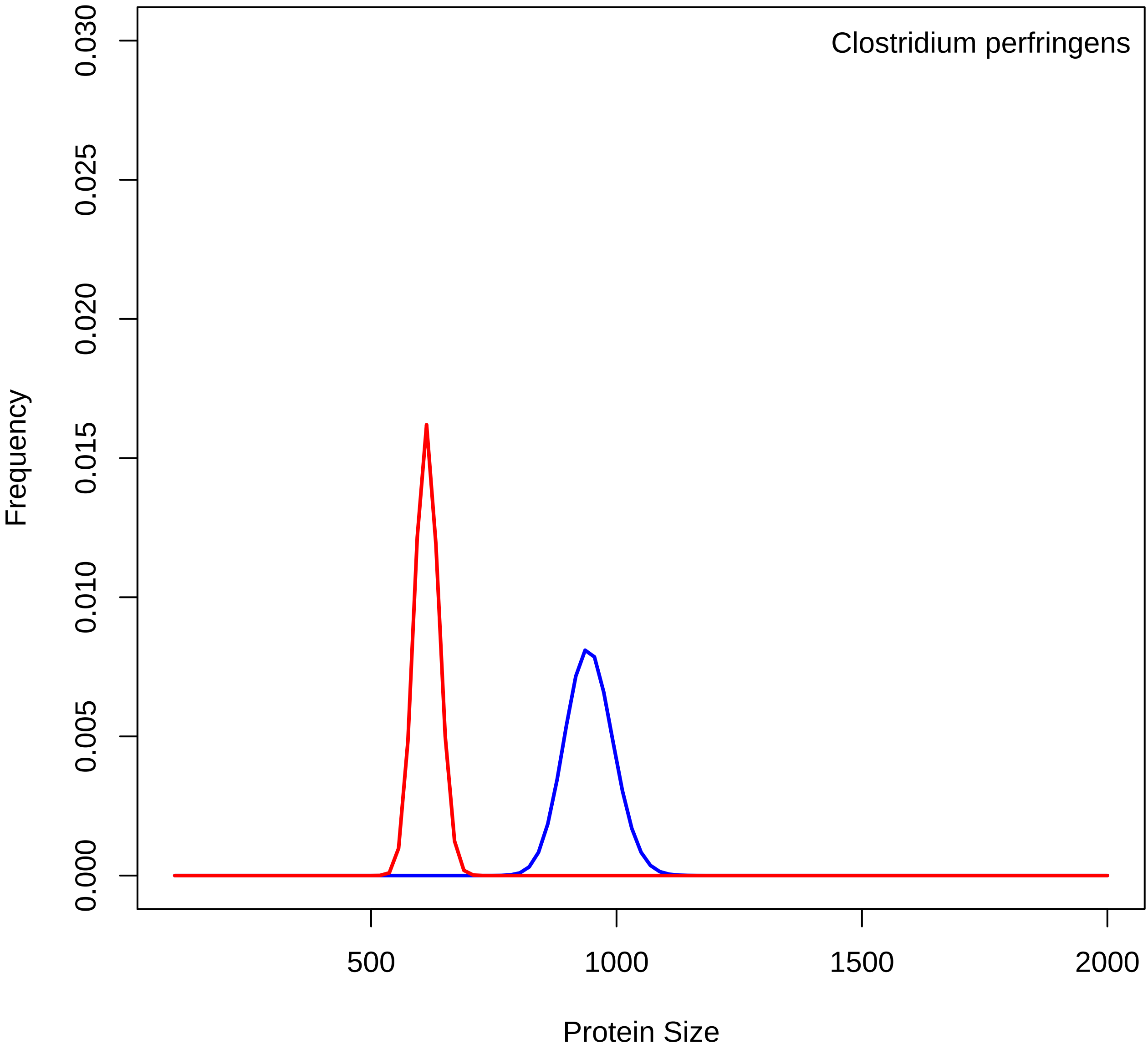

Supplement 4 − Figure 45

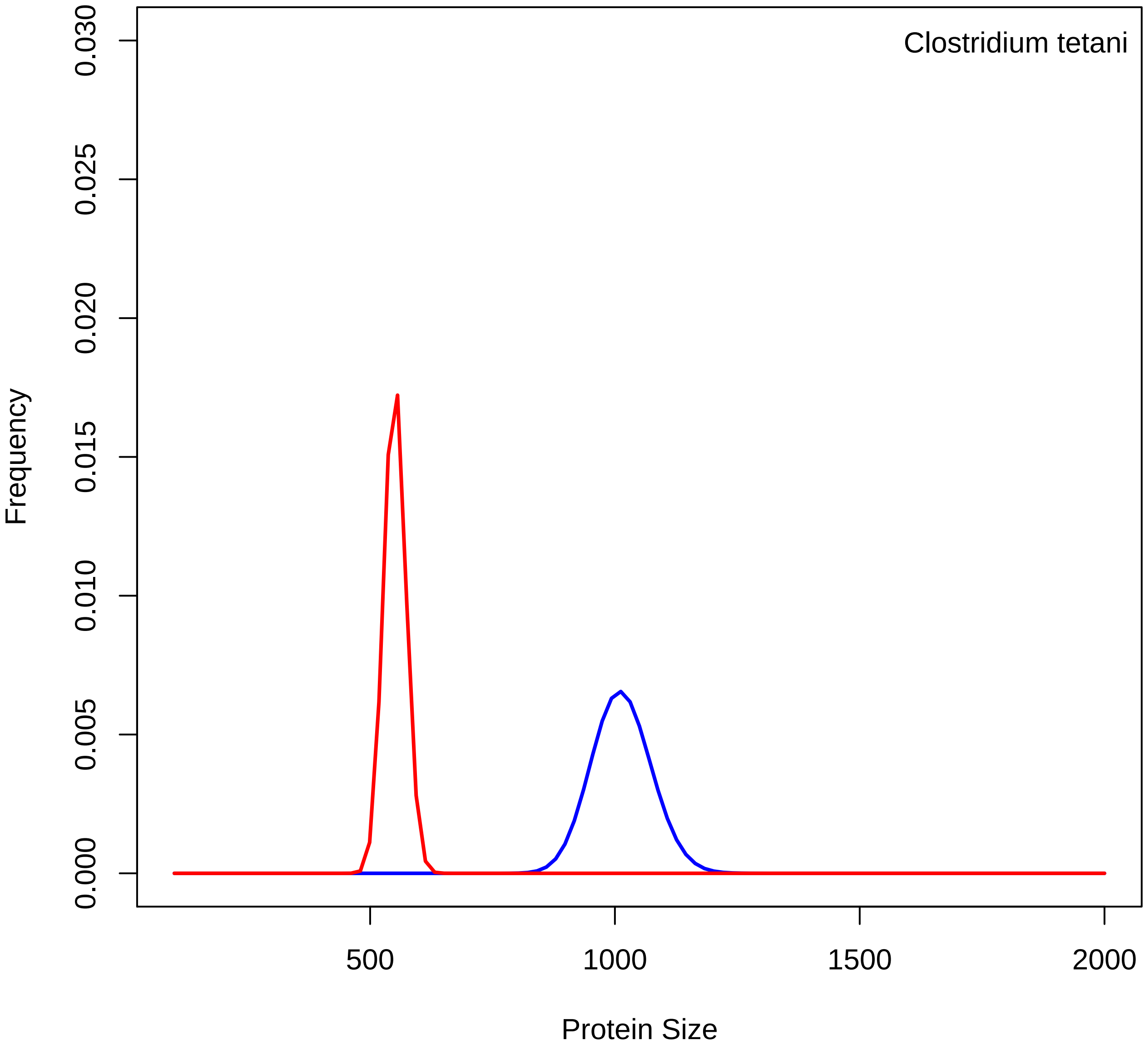

Supplement 4 − Figure 46

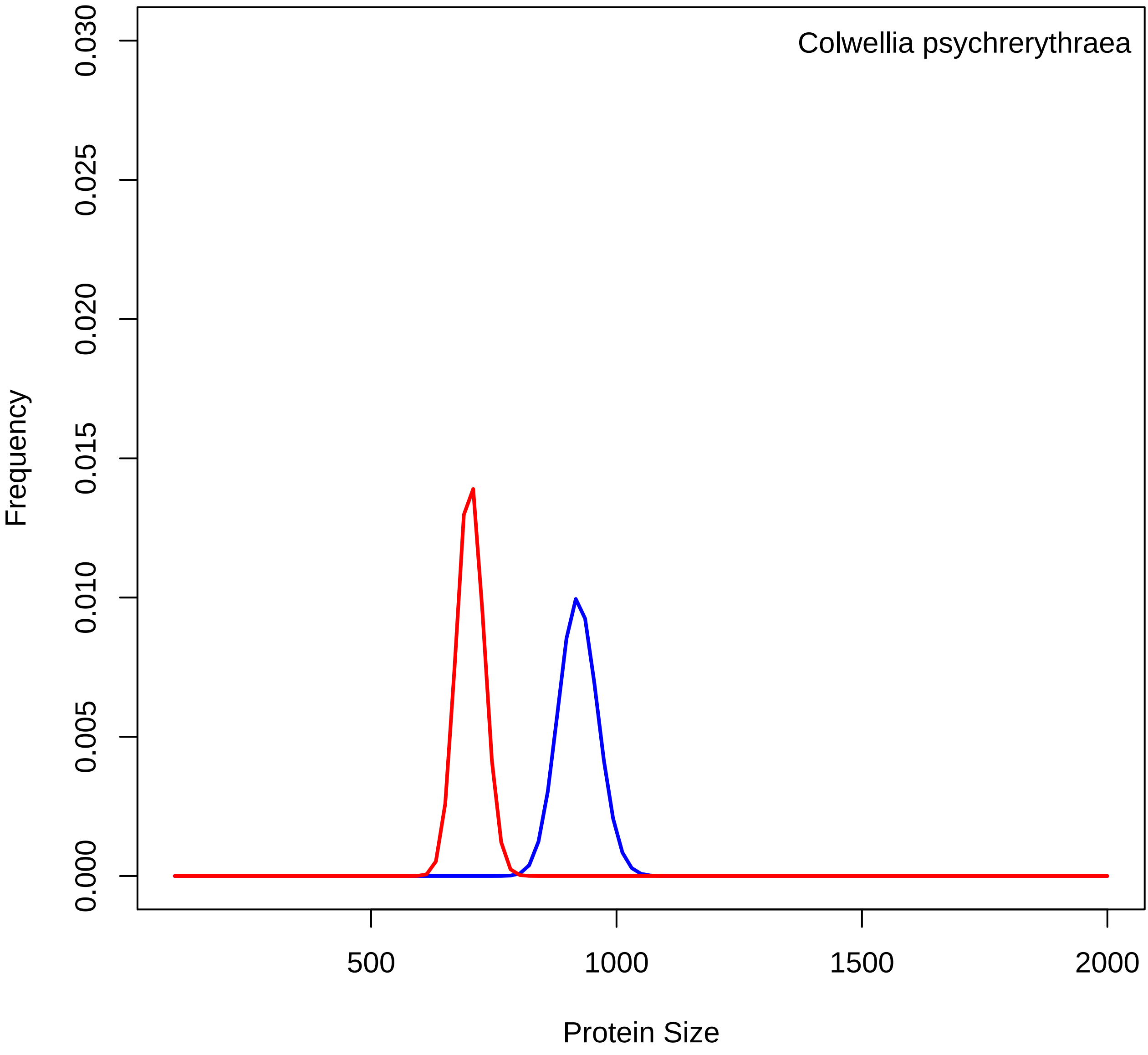

Supplement 4 − Figure 47

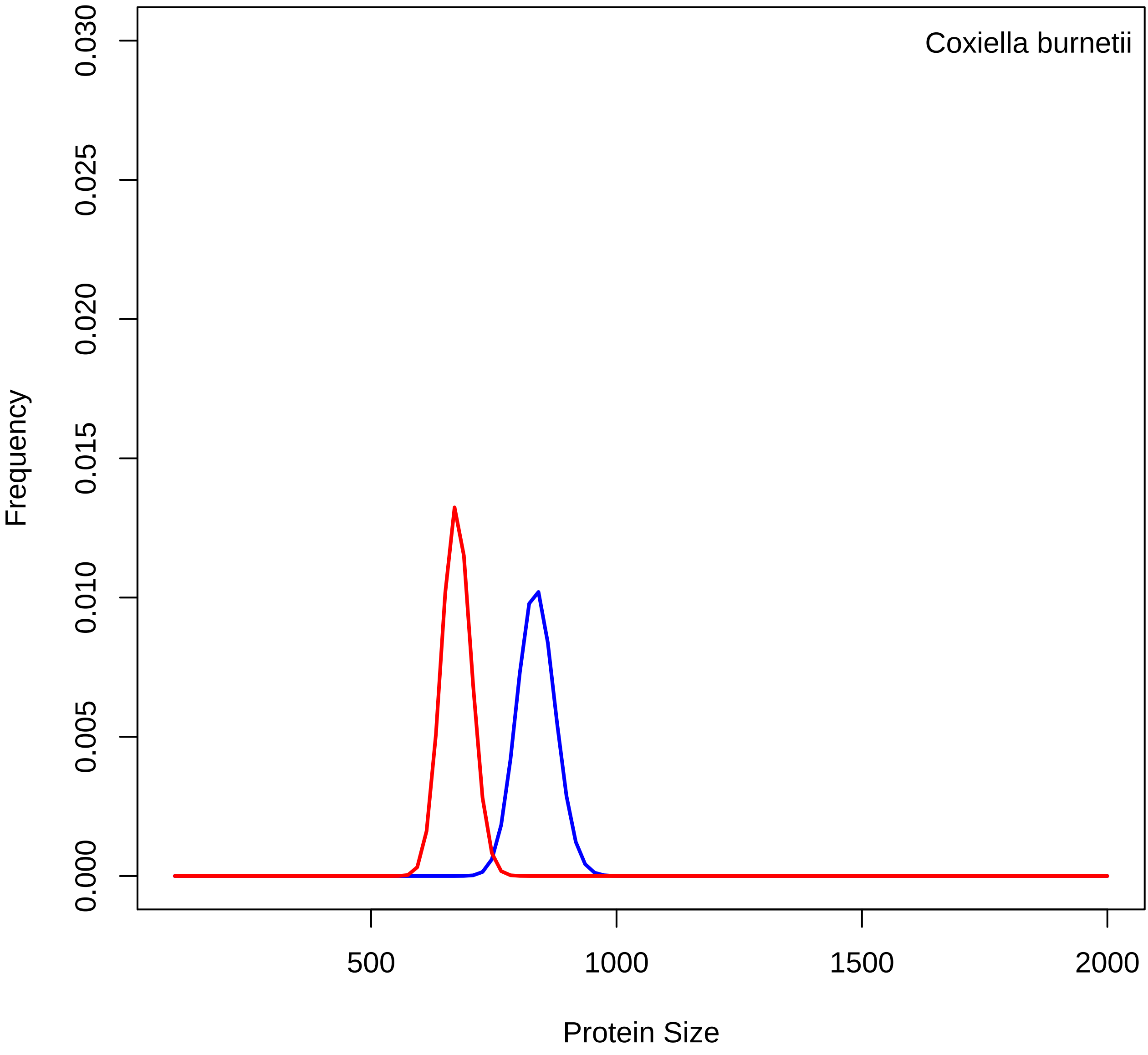

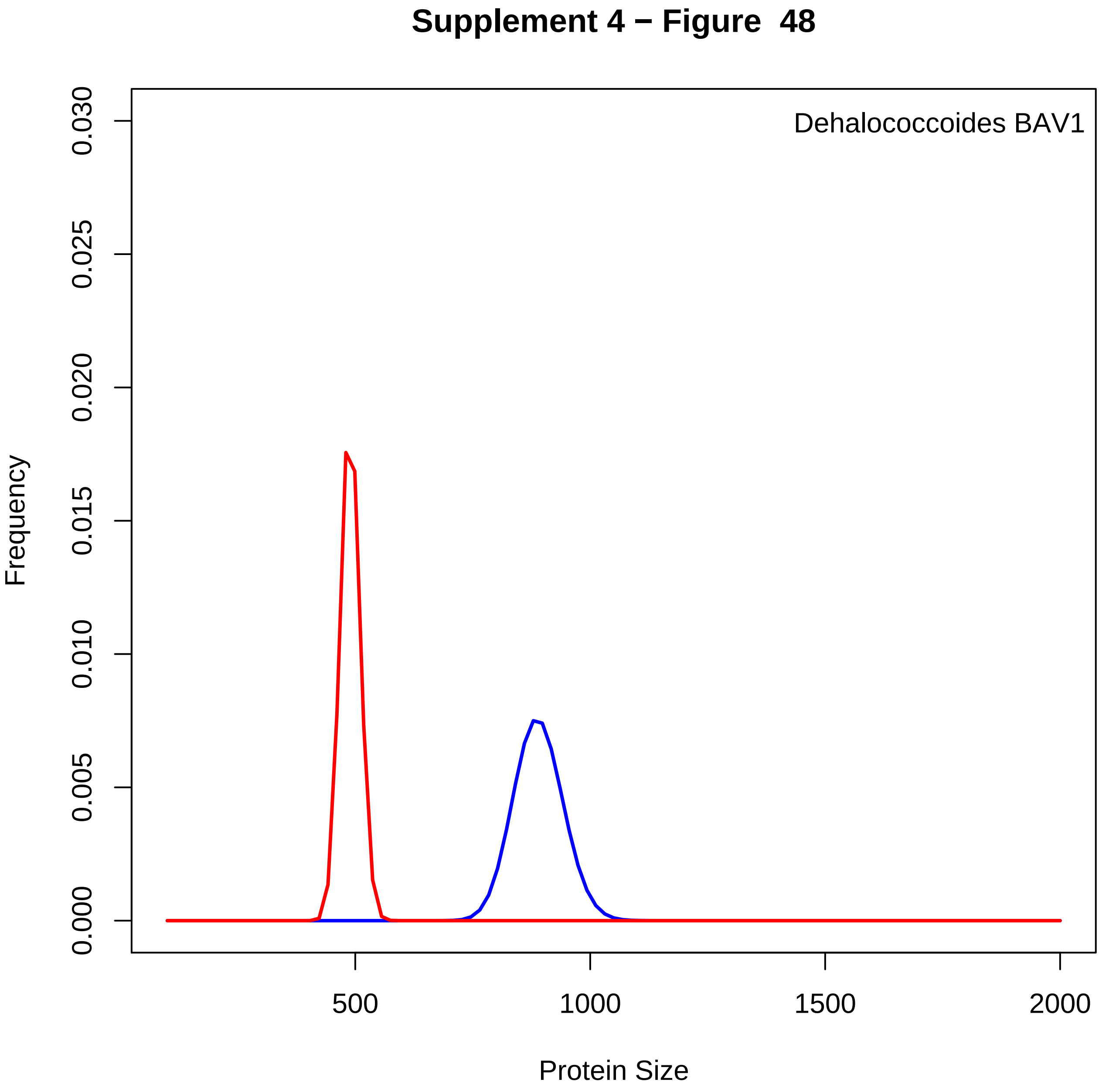
Supplement 4 − Figure 48
Dehalococcoides BAV1
Frequency
0.000
0.005
0.010
0.015
0.020
0.025
0.030
500
1000
1500
2000
Protein Size

Supplement 4 − Figure 49

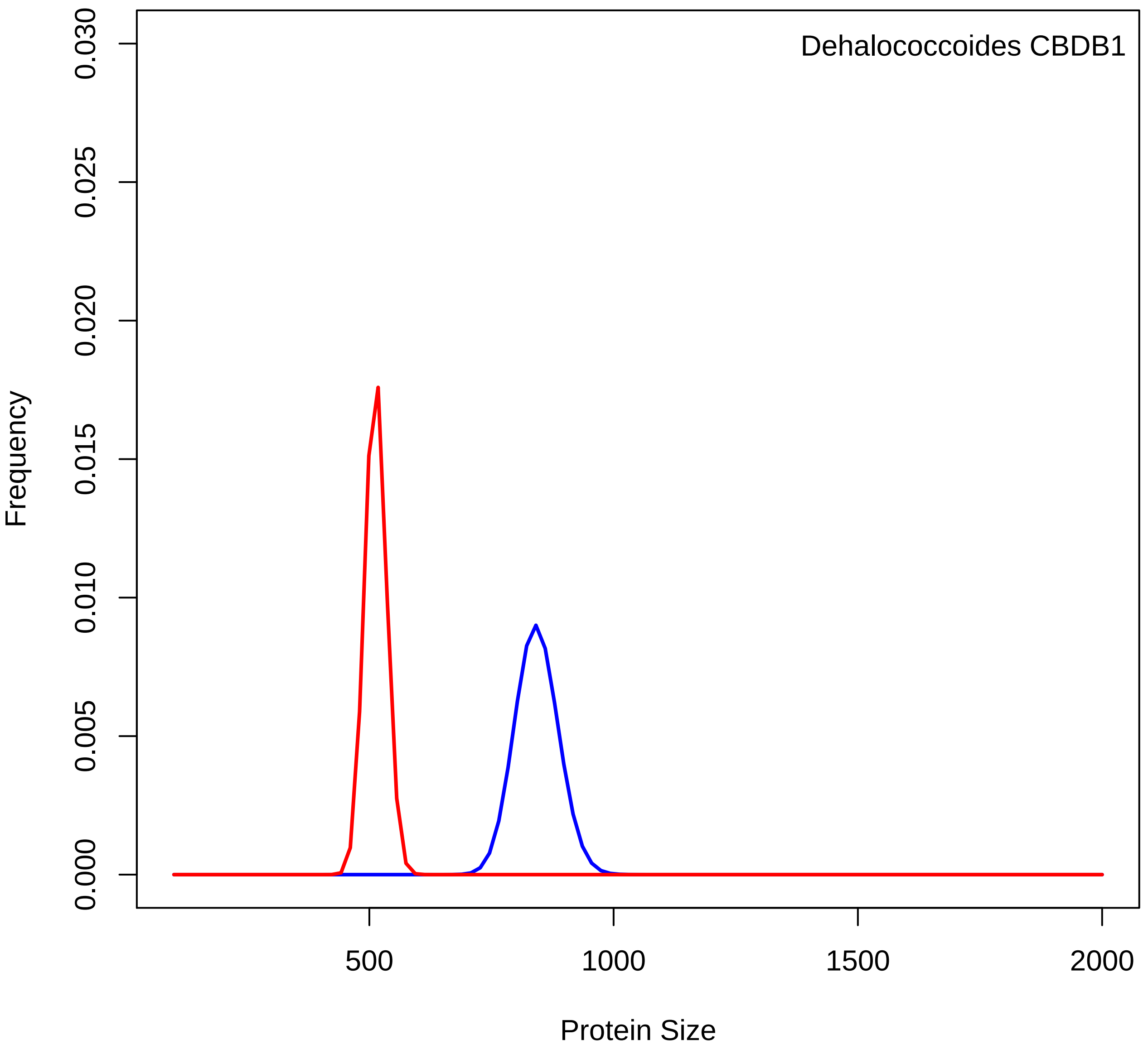

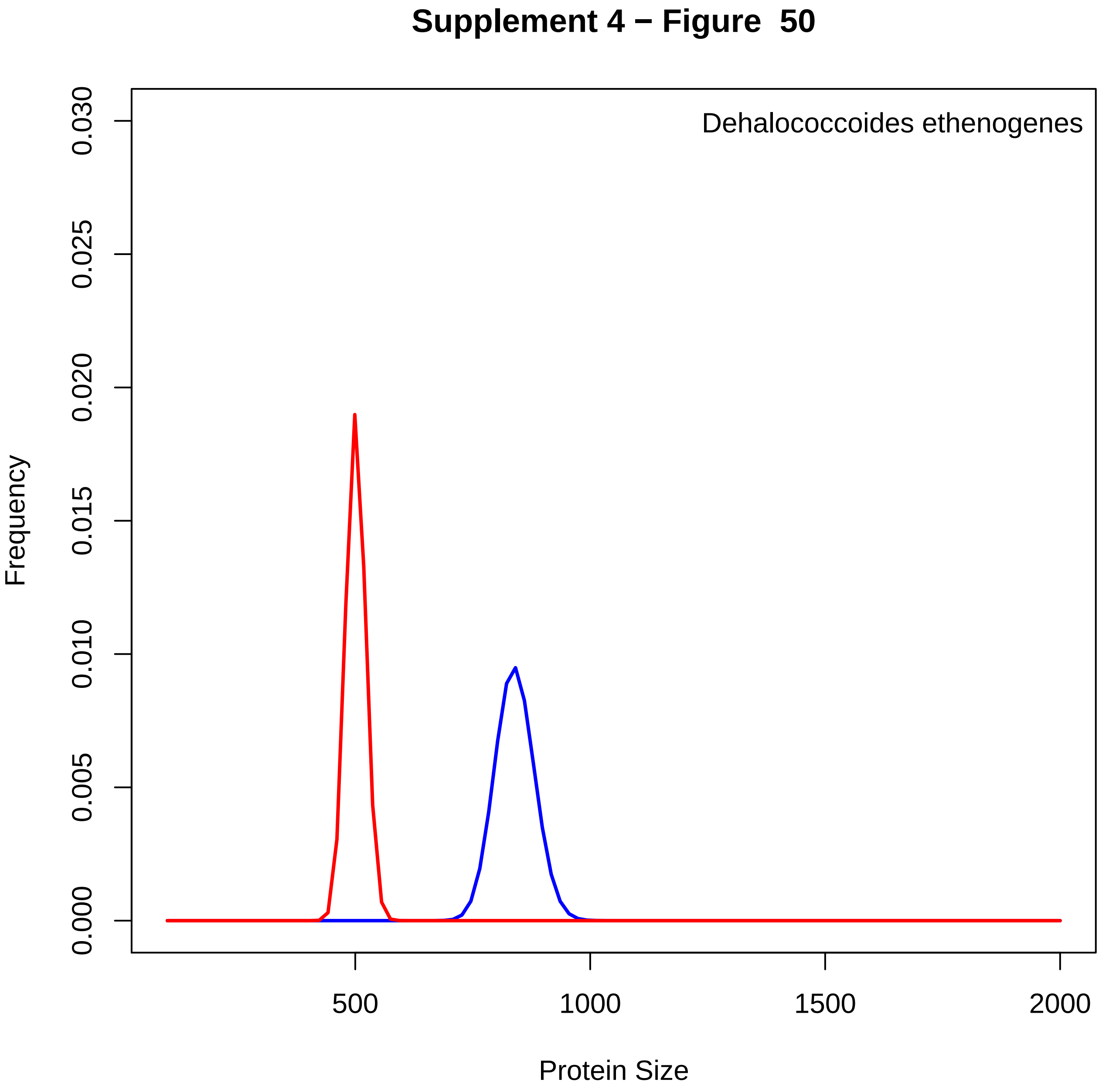
Supplement 4 − Figure 50
Dehalococcoides ethenogenes
Frequency
0.000
0.005
0.010
0.015
0.020
0.025
0.030
500
1000
1500
2000
Protein Size

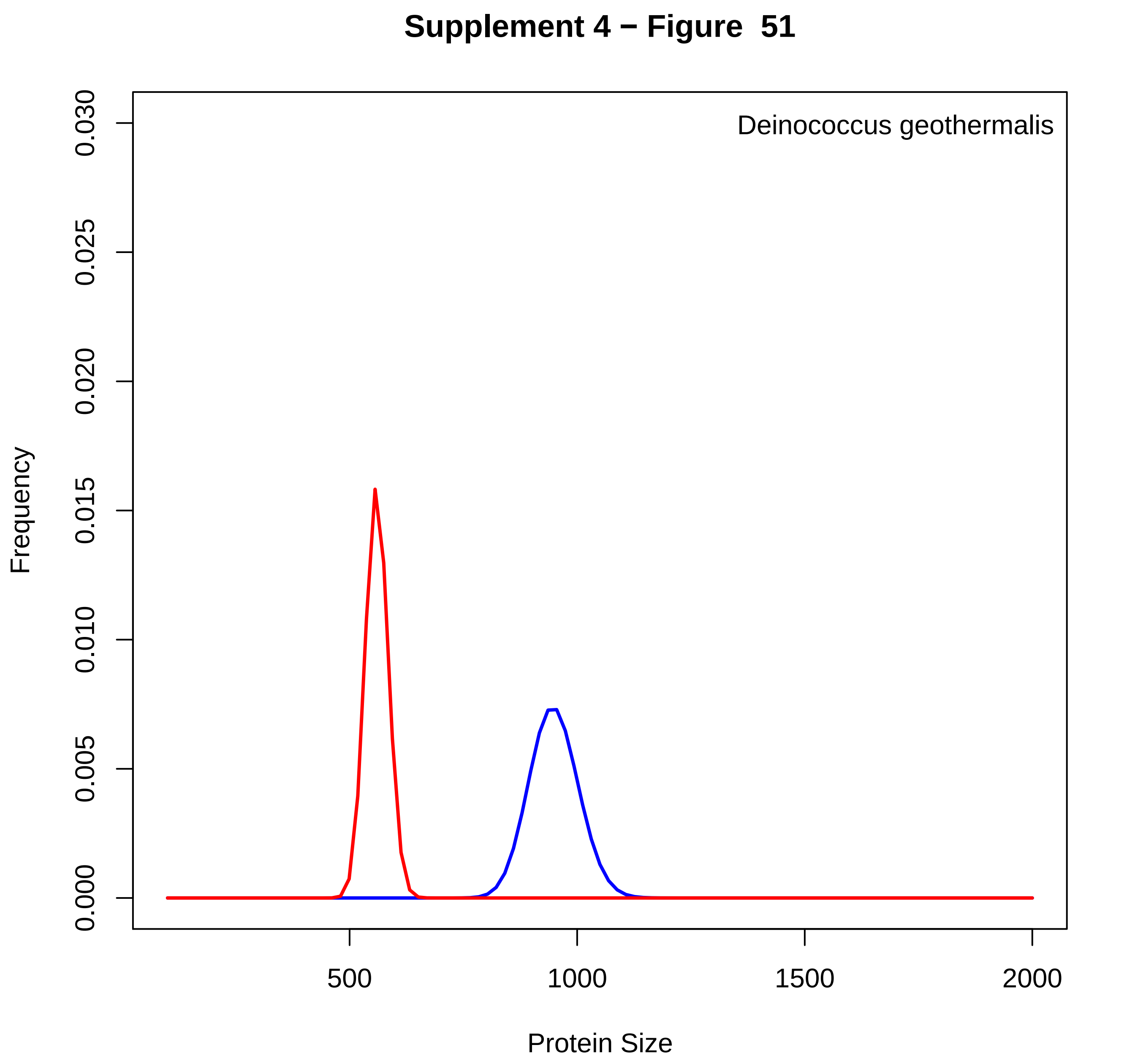
Supplement 4 − Figure 51
Deinococcus geothermalis
Frequency
0.000
0.005
0.010
0.015
0.020
0.025
0.030
500
1000
1500
2000
Protein Size

Supplement 4 − Figure 52

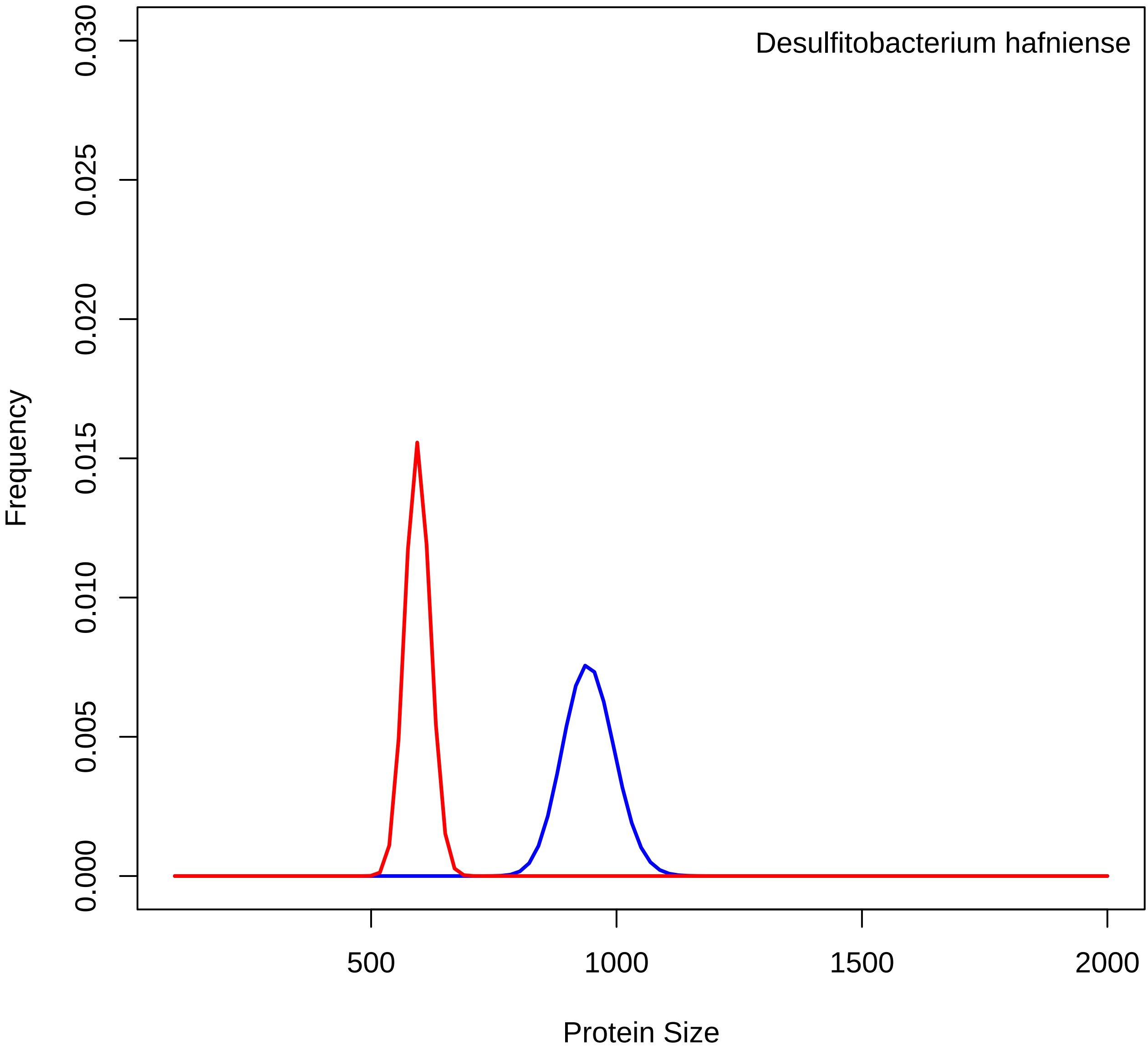

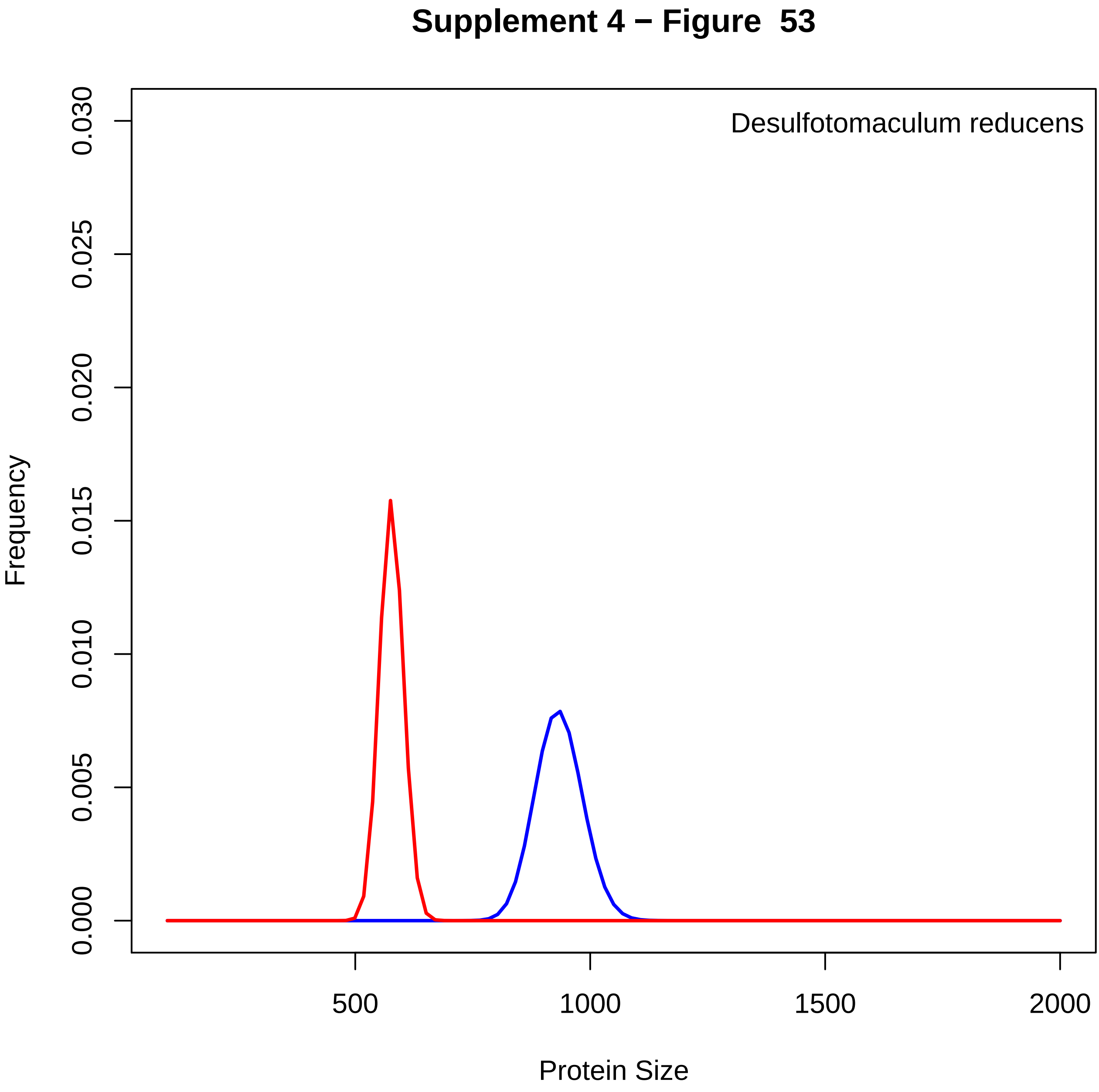
Supplement 4 − Figure 53
Desulfotomaculum reducens
Frequency
0.000
0.005
0.010
0.015
0.020
0.025
0.030
500
1000
1500
2000
Protein Size

Supplement 4 − Figure 54

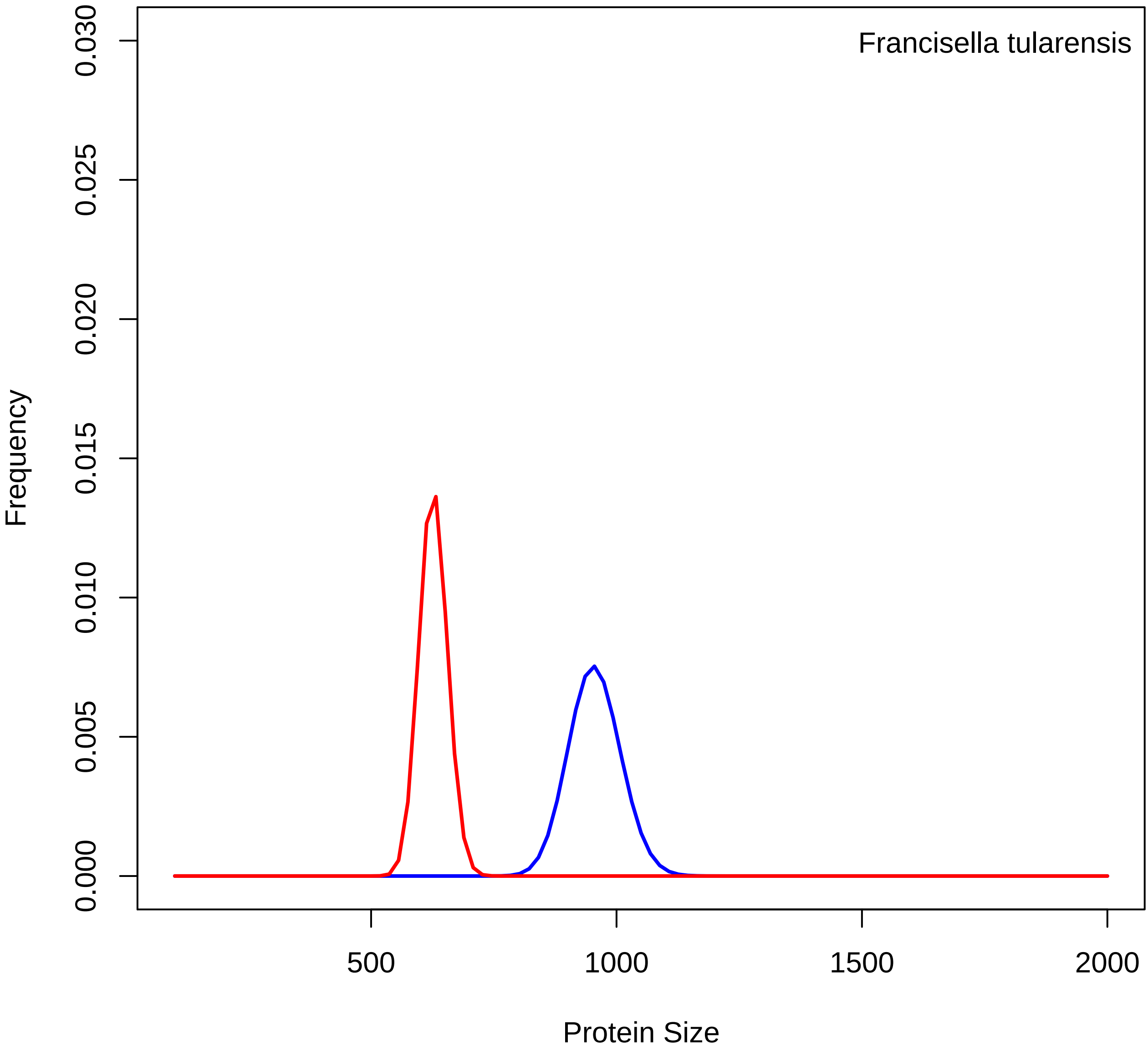

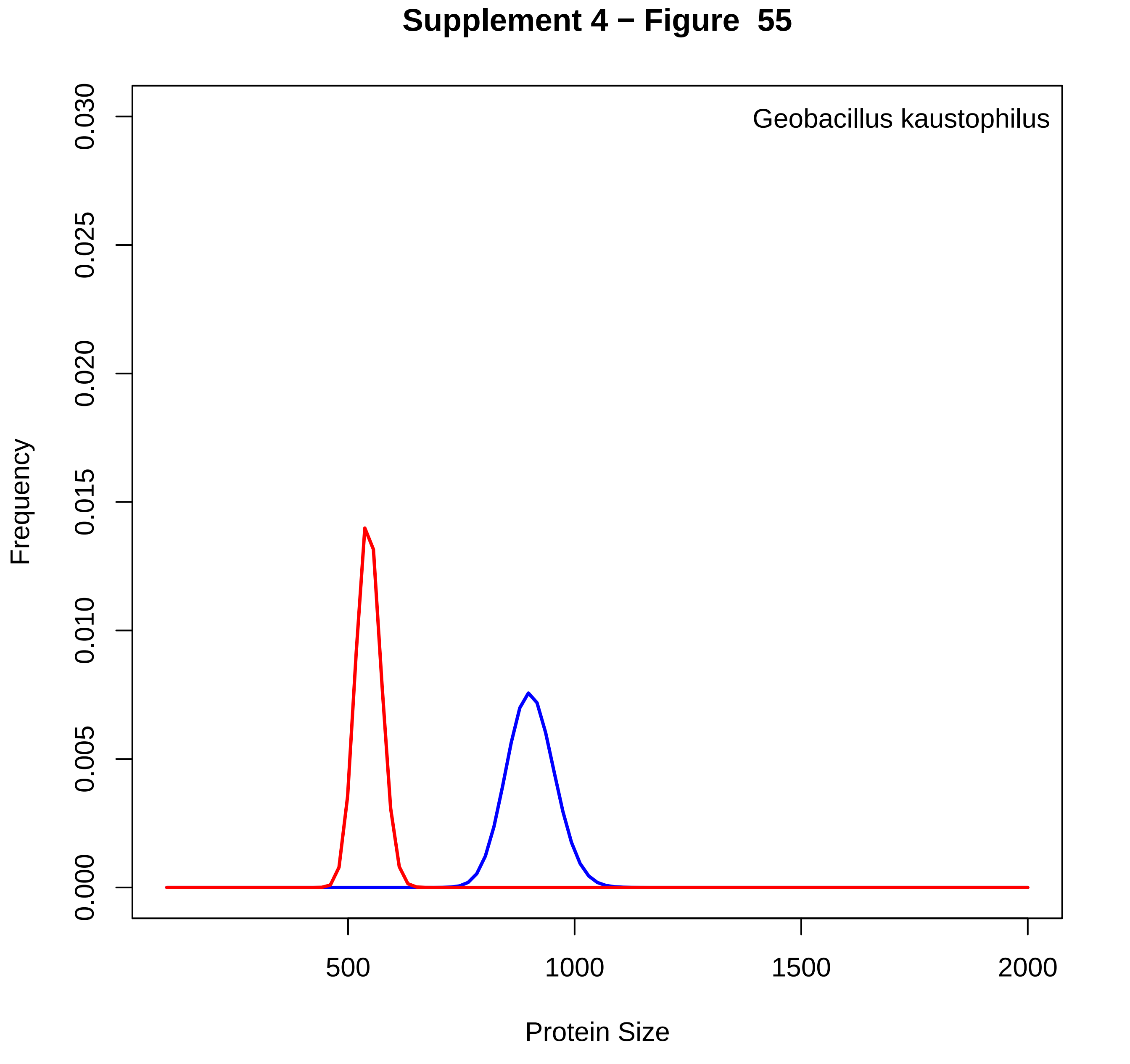

Supplement 4 − Figure 55
Geobacillus kaustophilus
Frequency
0.000
0.005
0.010
0.015
0.020
0.025
0.030
500
1000
1500
2000
Protein Size

Supplement 4 − Figure 56

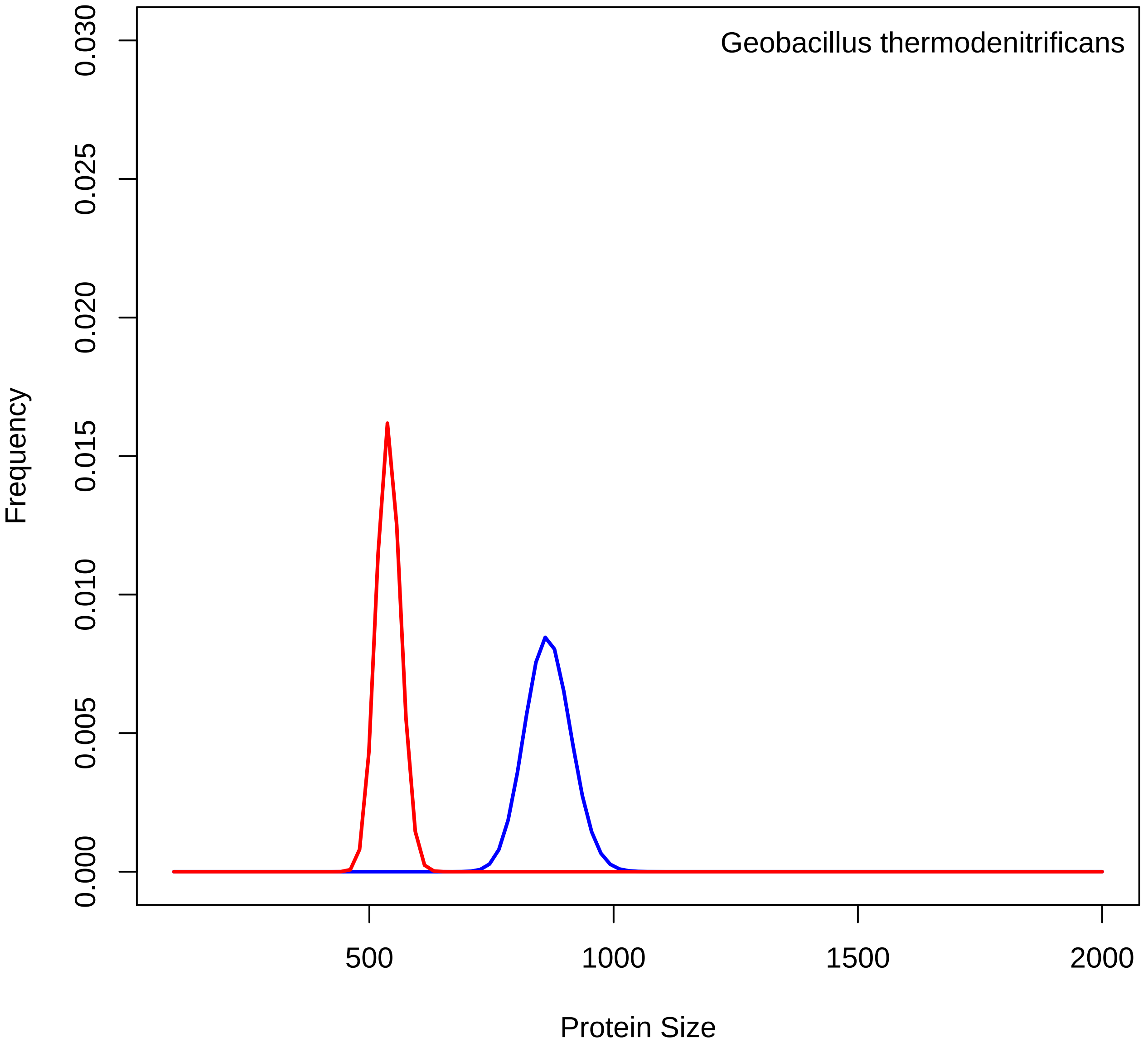

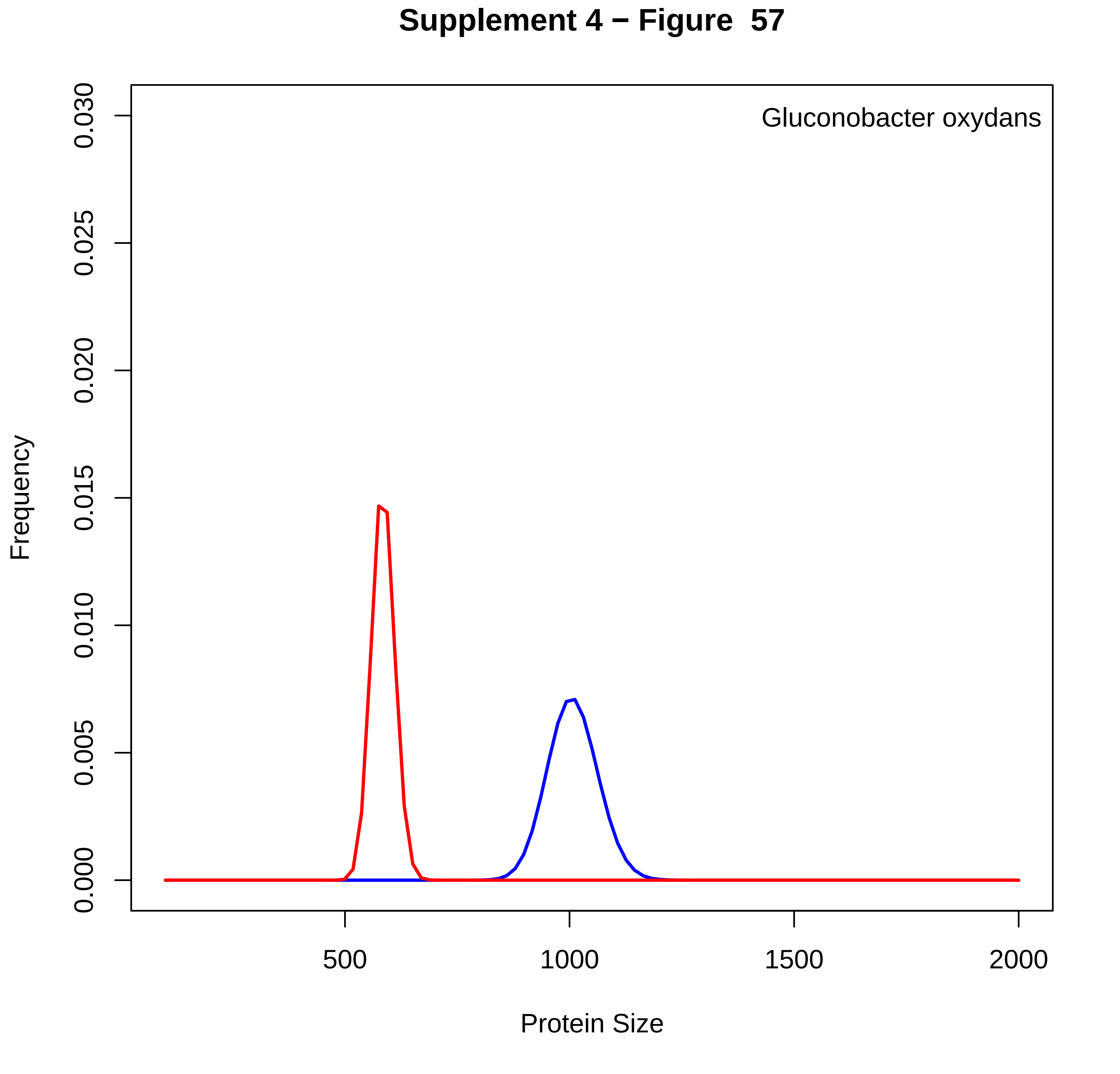

Supplement 4 − Figure 57
Gluconobacter oxydans
Frequency
0.000
0.005
0.010
0.015
0.020
0.025
0.030
500
1000
1500
2000
Protein Size

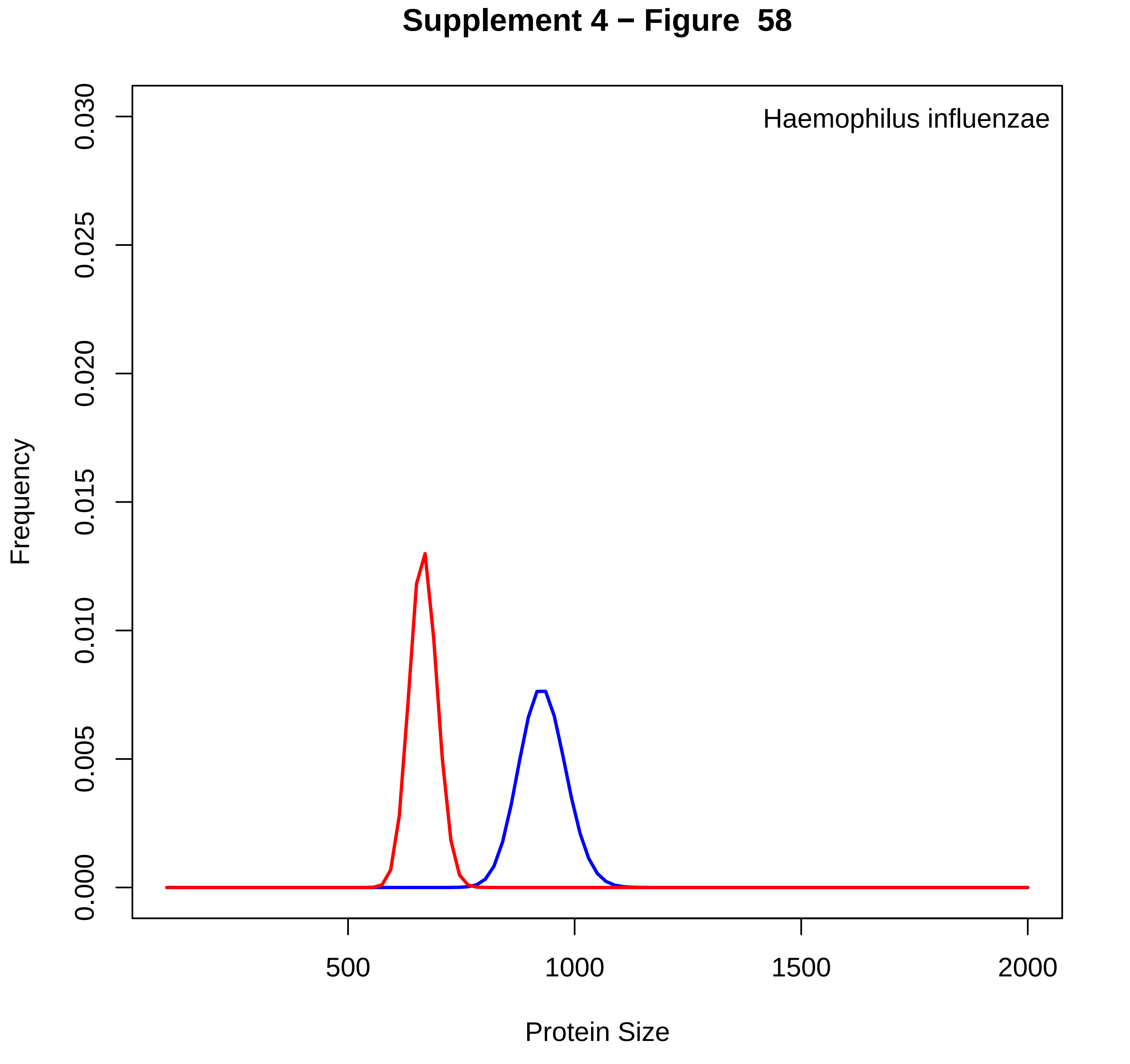

Supplement 4 − Figure 58
Haemophilus influenzae
Frequency
0.000
0.005
0.010
0.015
0.020
0.025
0.030
500
1000
1500
2000
Protein Size

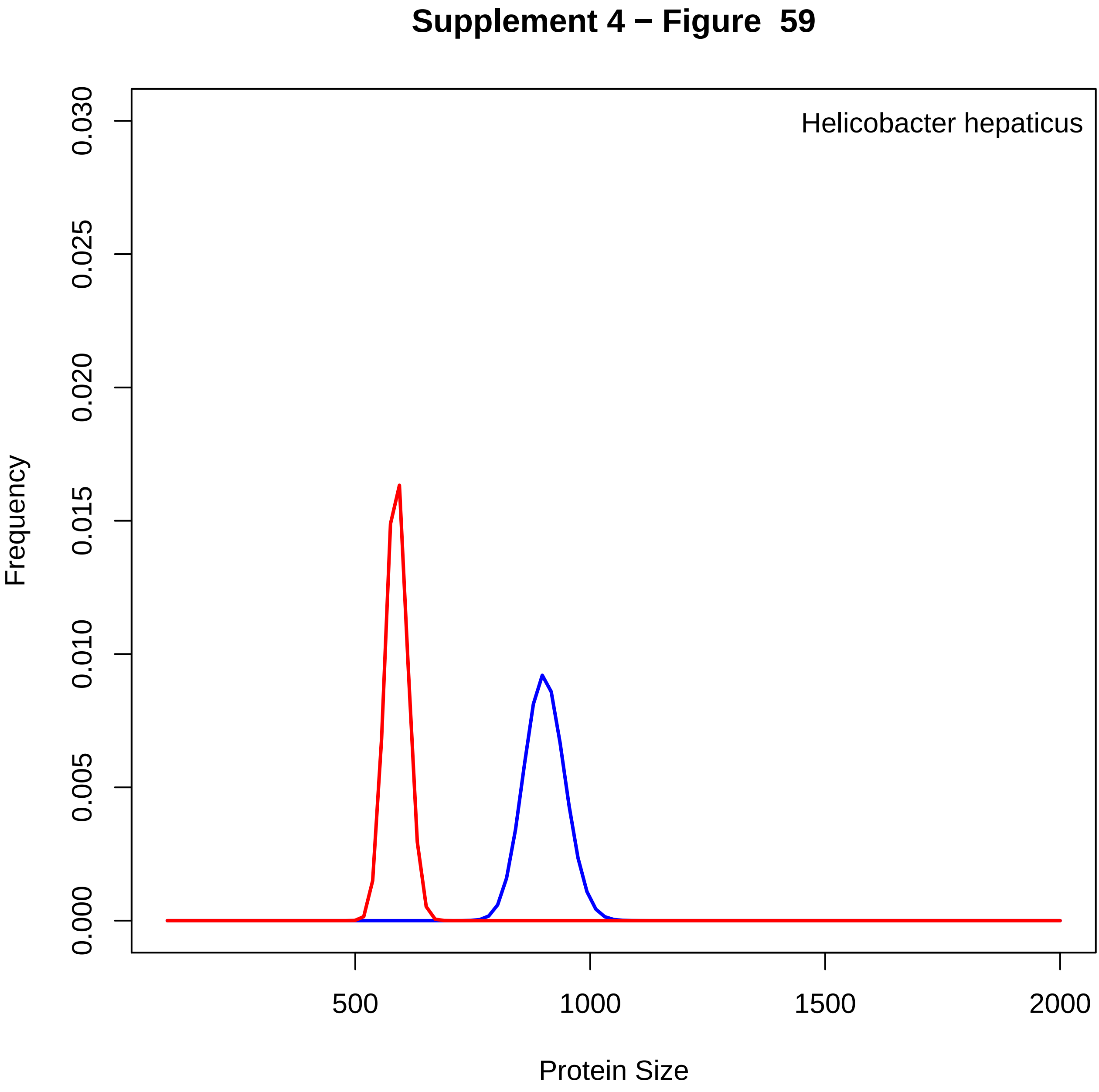
Supplement 4 − Figure 59
Helicobacter hepaticus
Frequency
0.000
0.005
0.010
0.015
0.020
0.025
0.030
500
1000
1500
2000
Protein Size

Supplement 4 − Figure 60

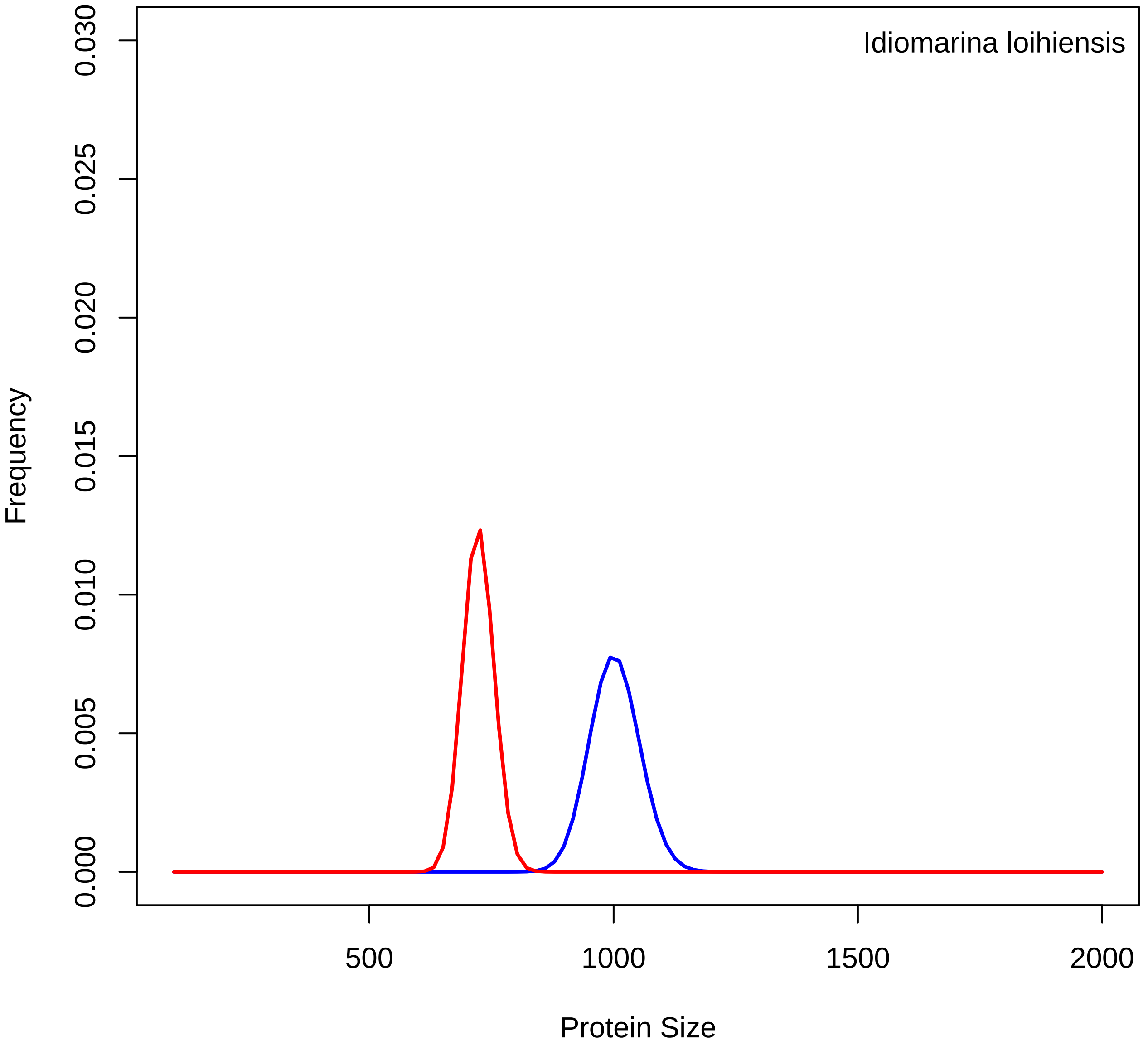

Supplement 4 − Figure 61

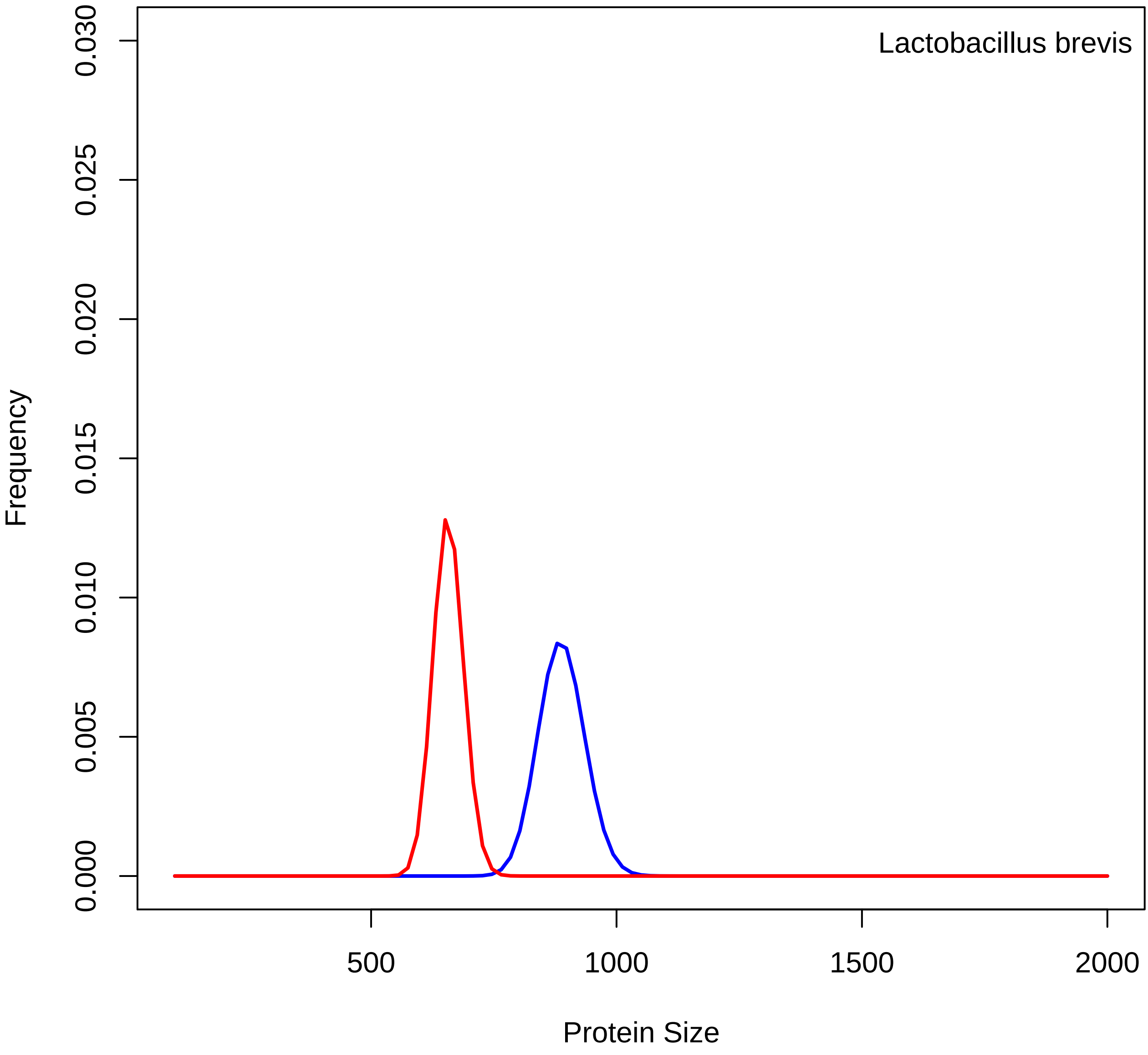

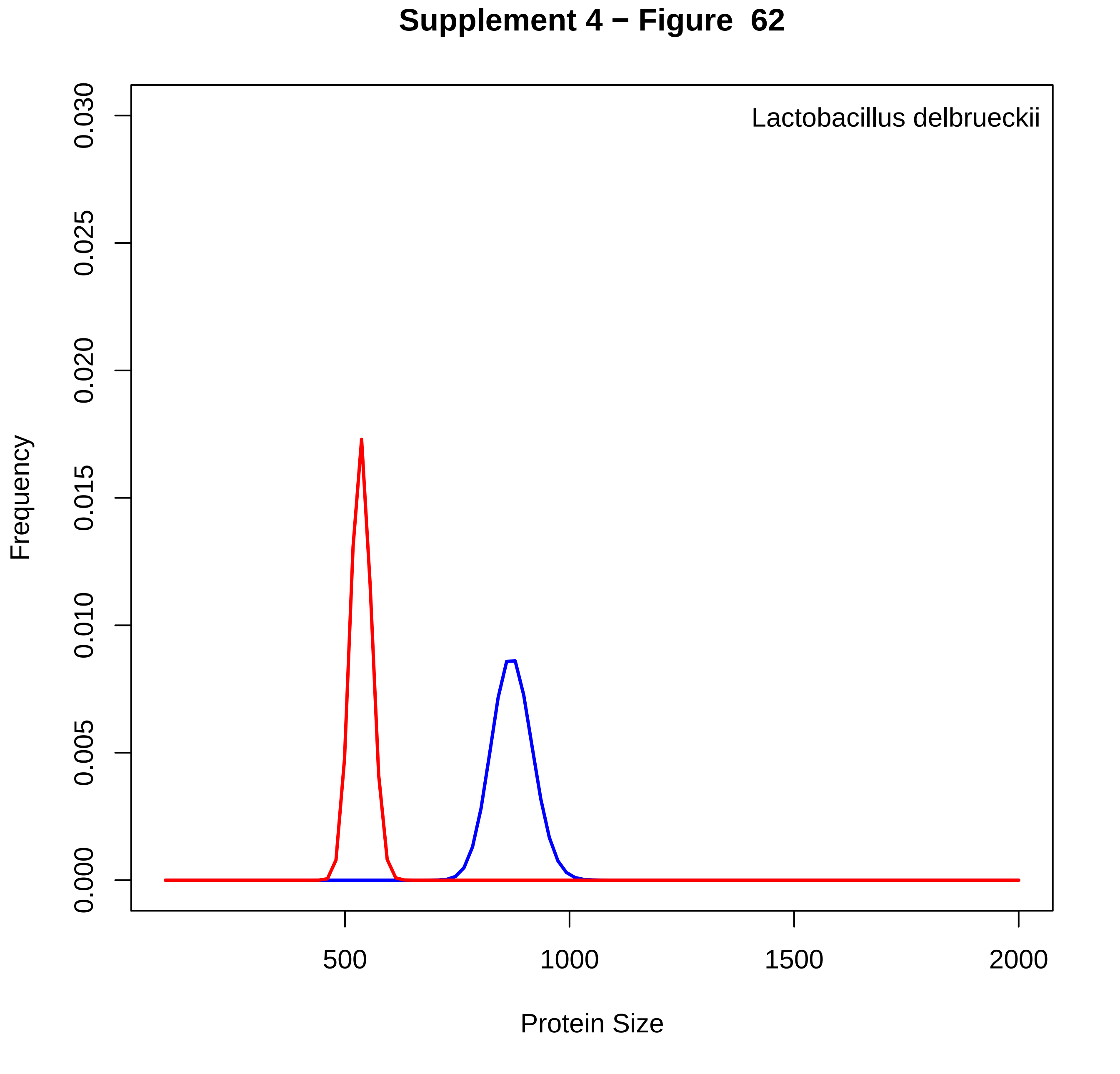

Supplement 4 − Figure 62
Lactobacillus delbrueckii
Frequency
0.000
0.005
0.010
0.015
0.020
0.025
0.030
500
1000
1500
2000
Protein Size

Supplement 4 − Figure 63

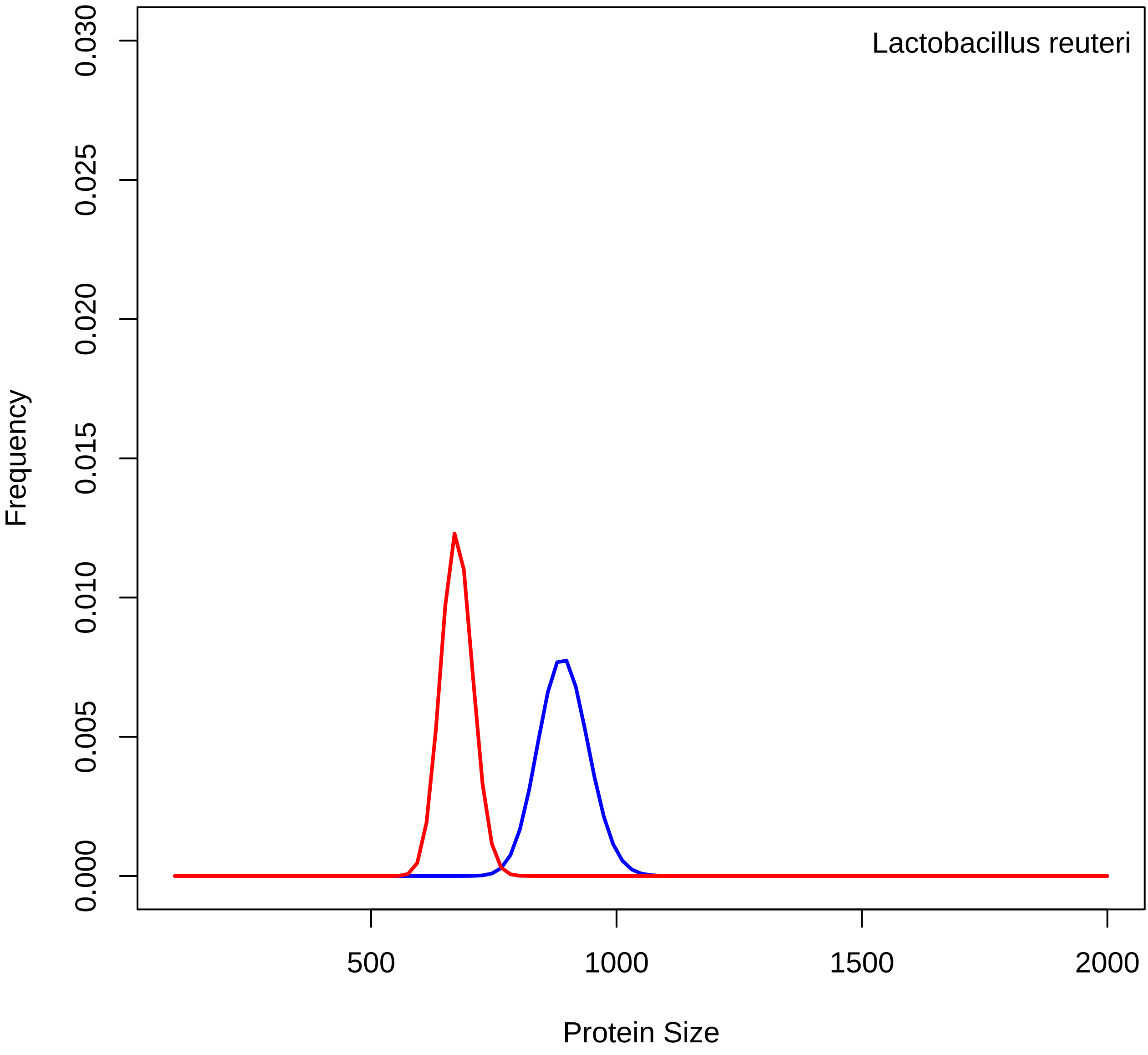

Supplement 4 − Figure 64

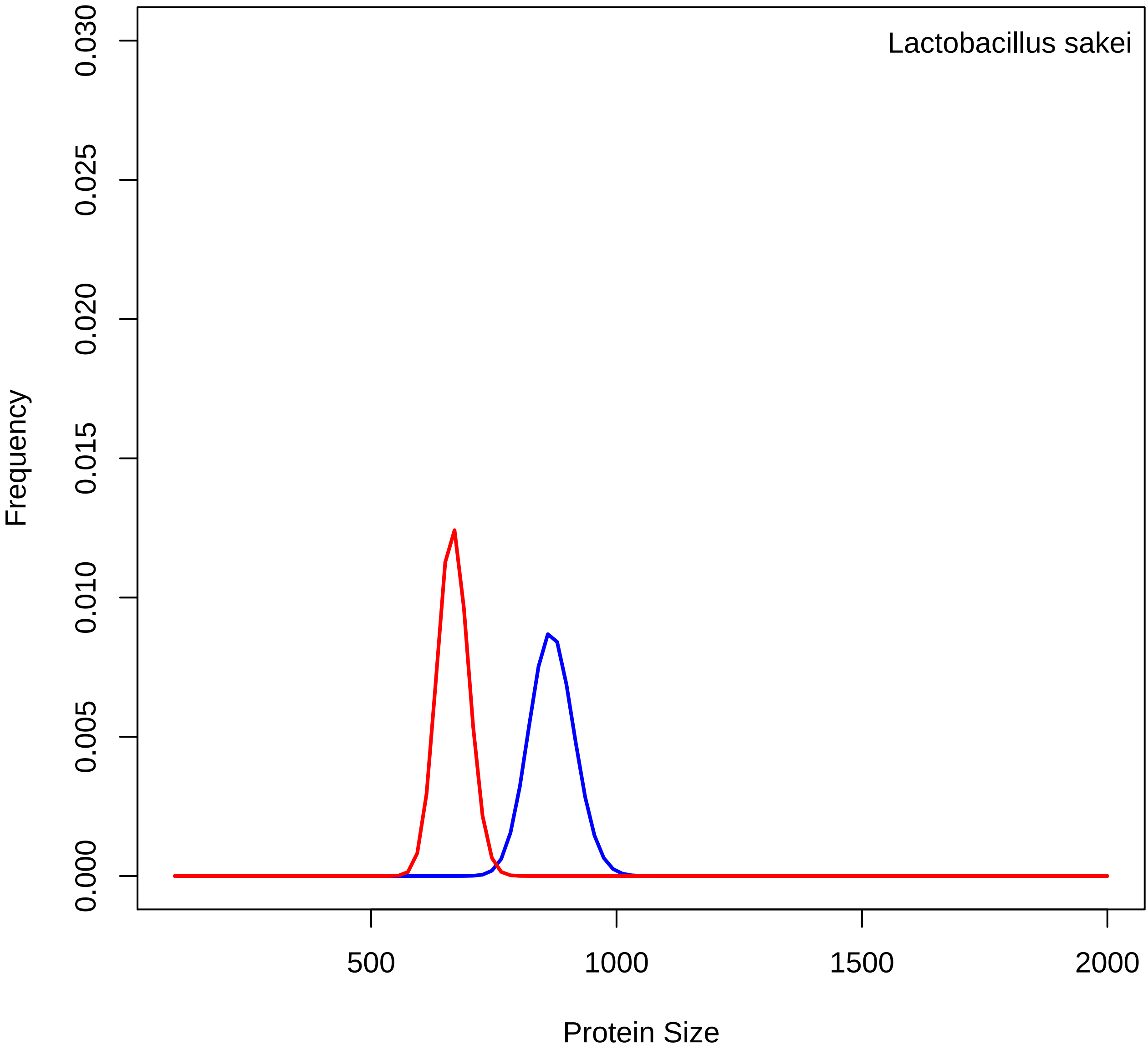

Supplement 4 − Figure 65

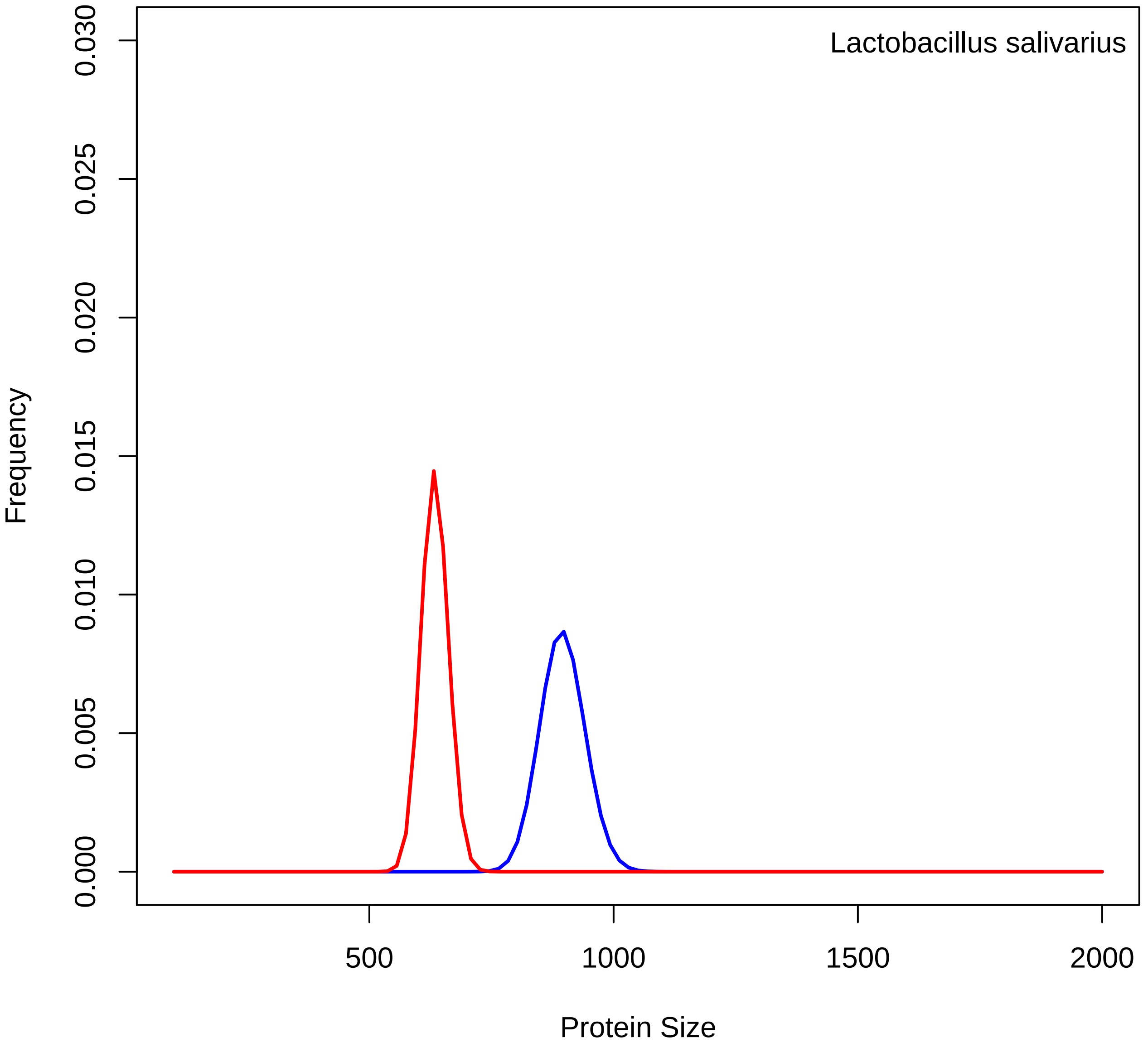

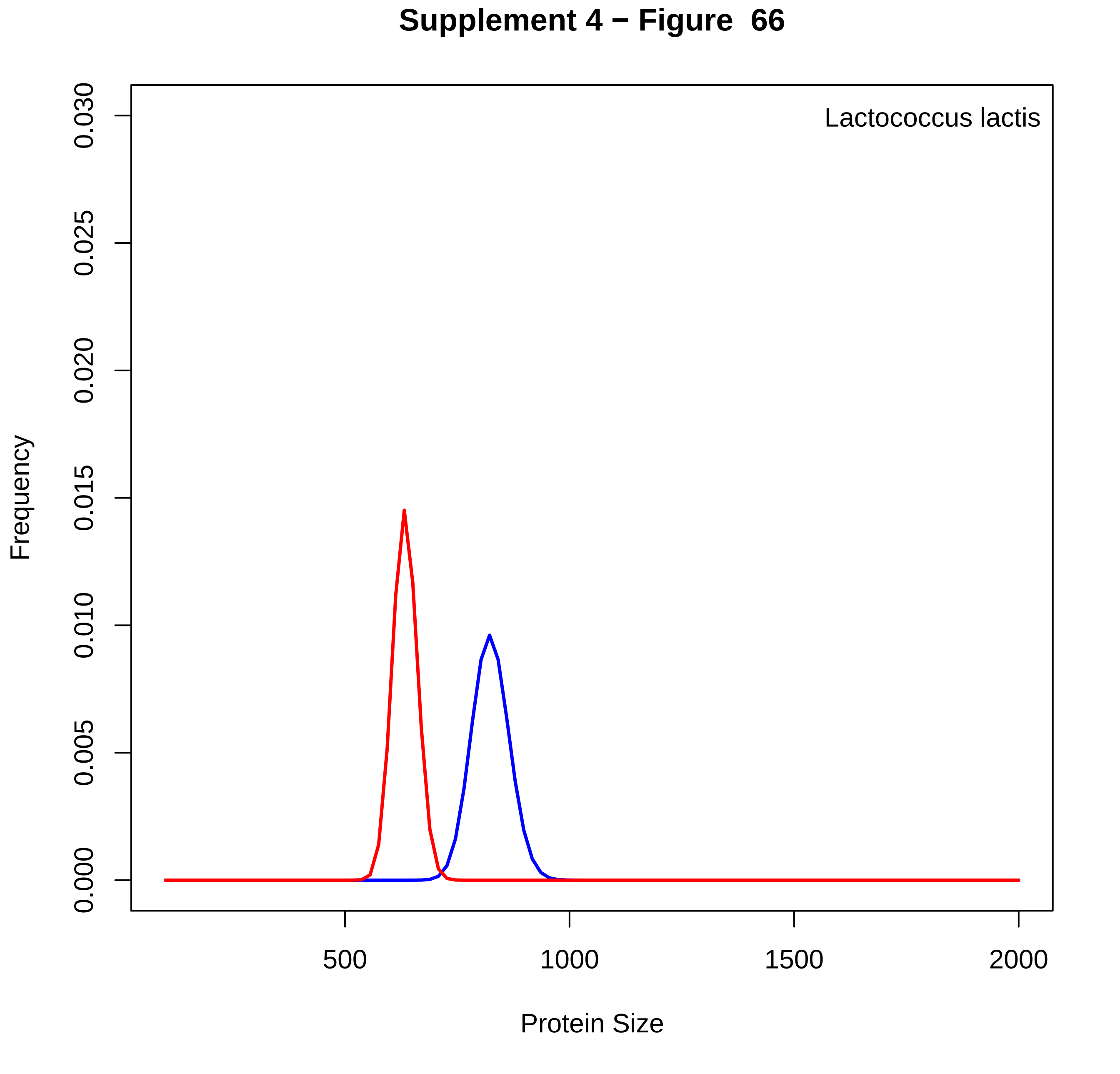
Supplement 4 − Figure 66
Lactococcus lactis
Frequency
0.000
0.005
0.010
0.015
0.020
0.025
0.030
500
1000
1500
2000
Protein Size

Supplement 4 − Figure 67

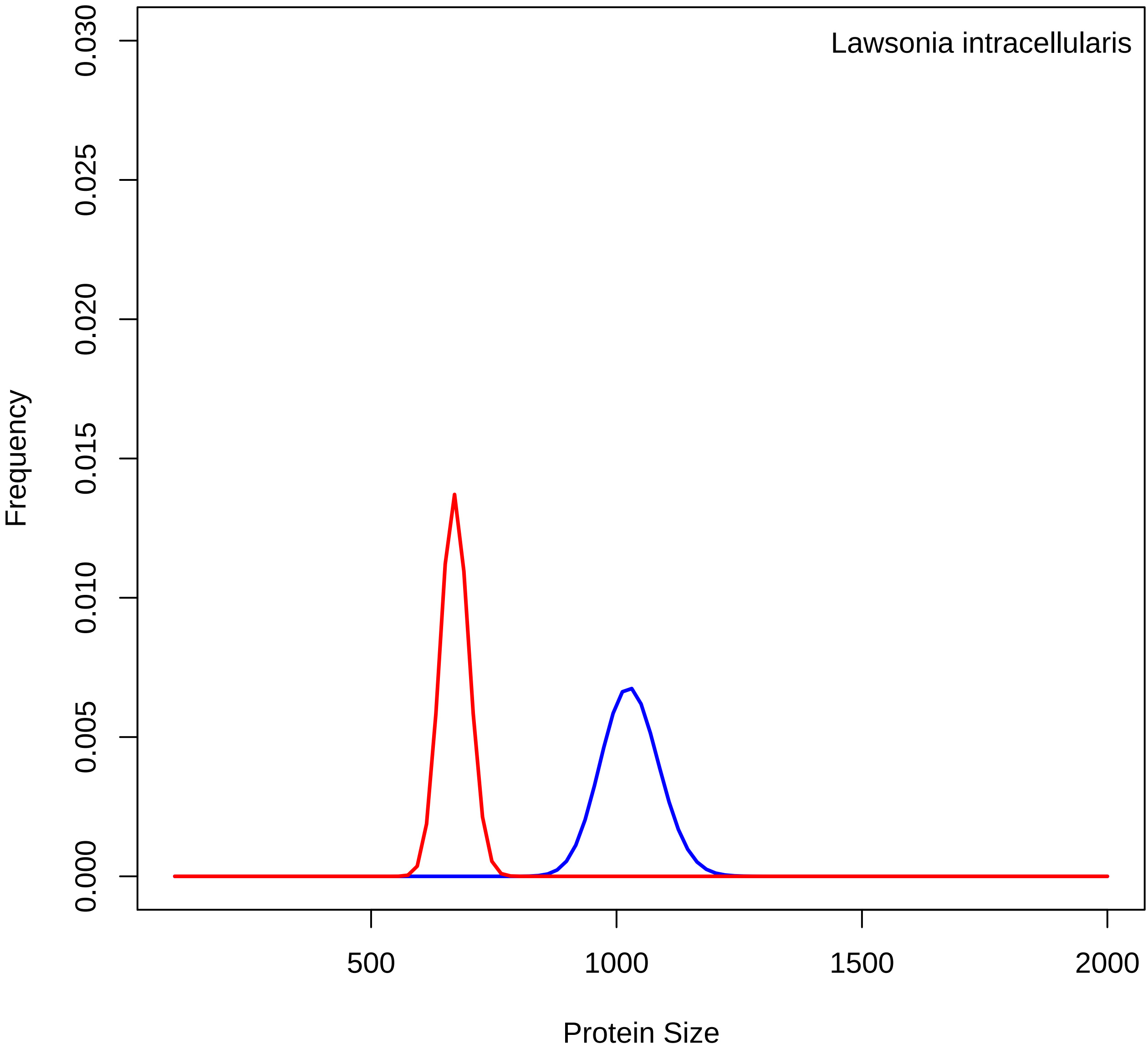

Supplement 4 − Figure 68

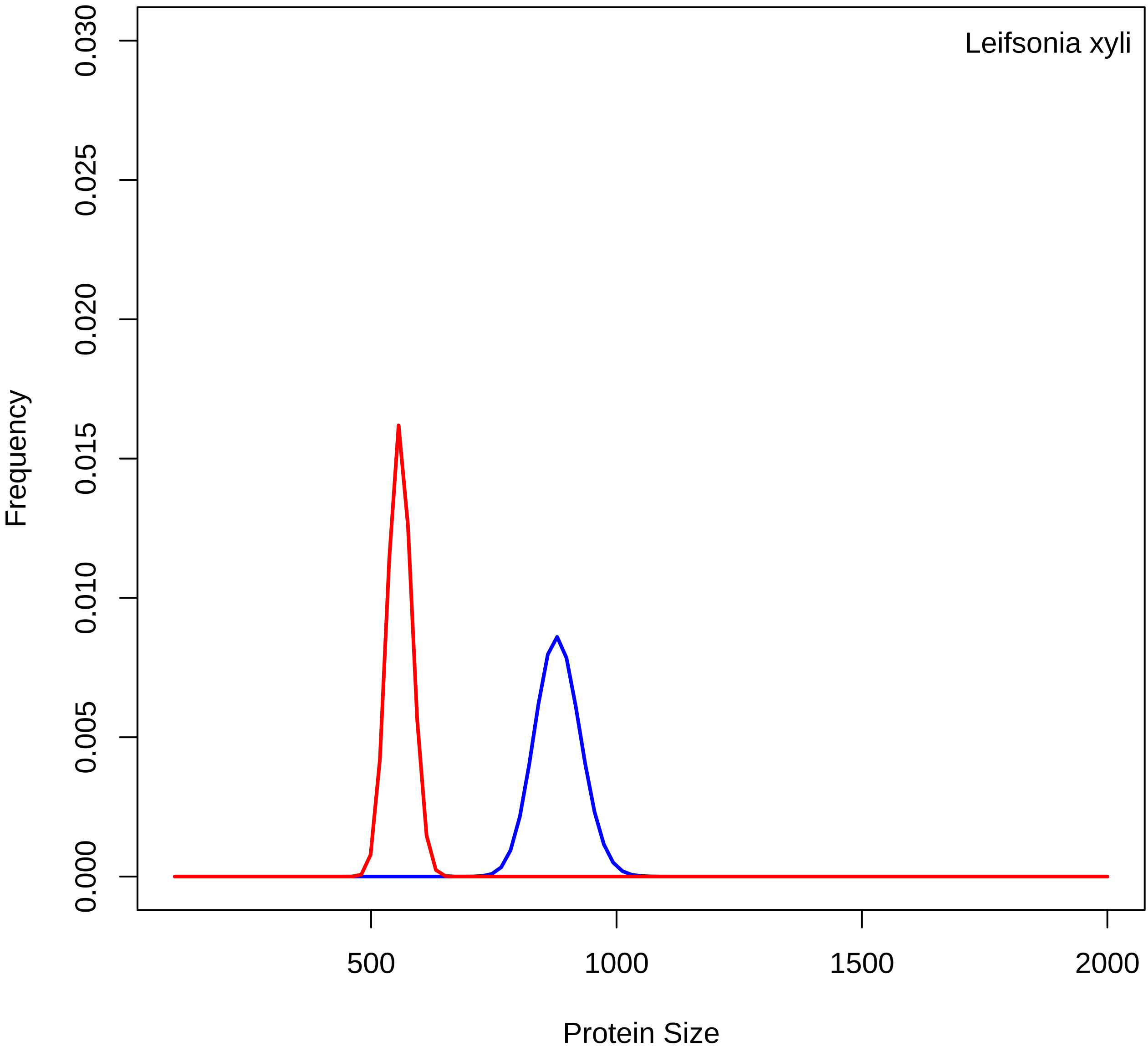

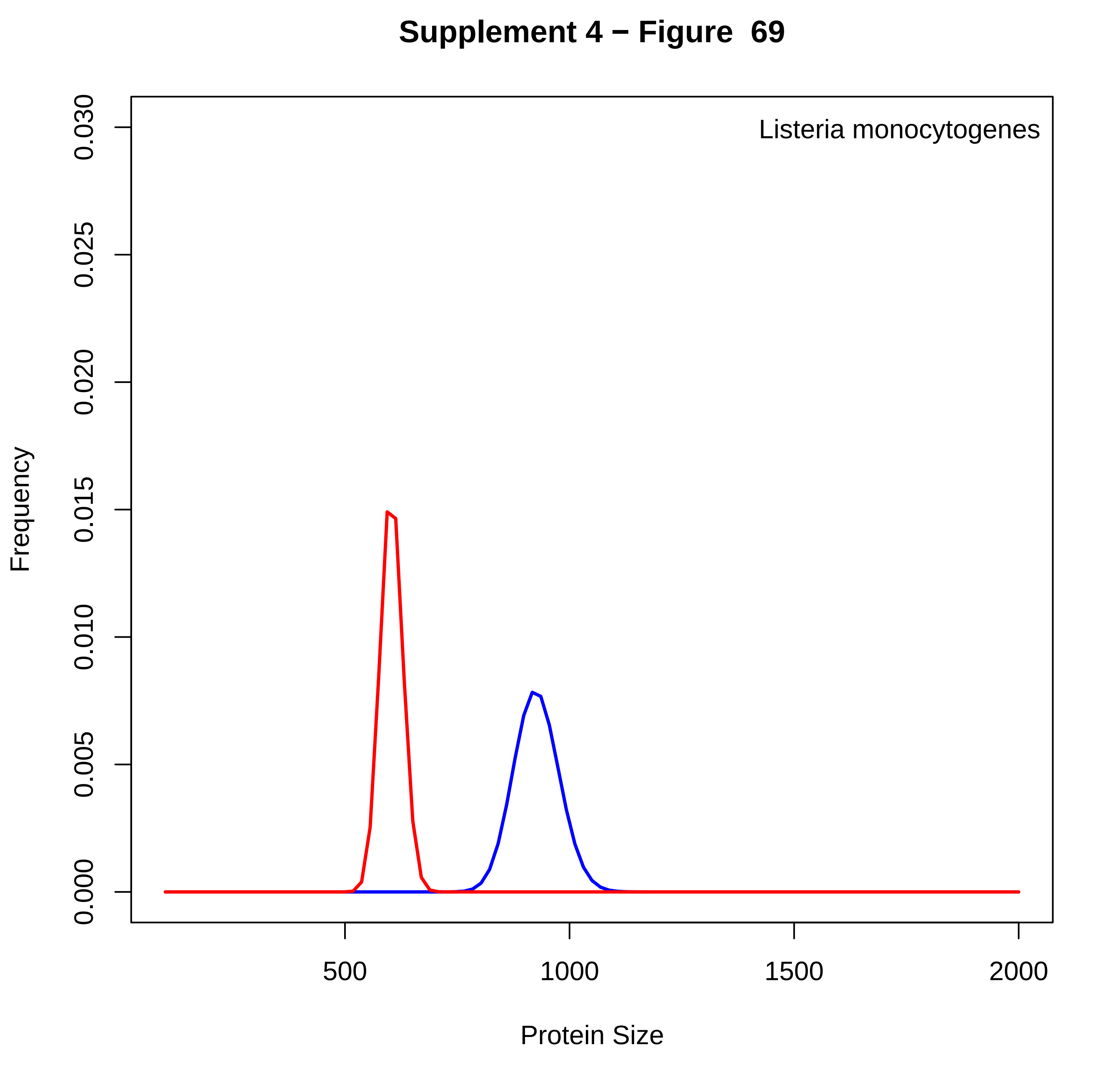

Supplement 4 − Figure 69
Listeria monocytogenes
Frequency
0.000
0.005
0.010
0.015
0.020
0.025
0.030
500
1000
1500
2000
Protein Size

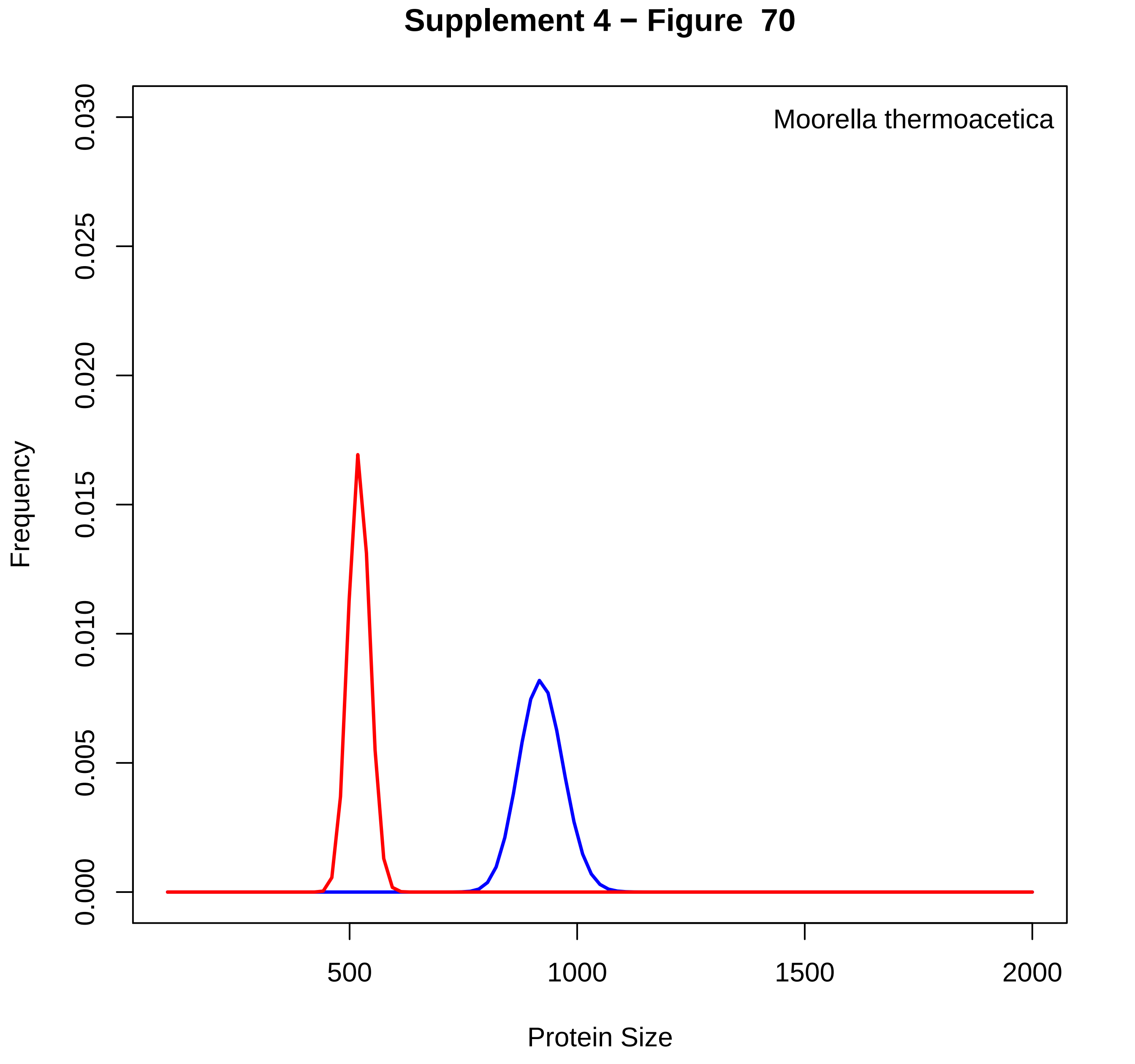
Supplement 4 − Figure 70
Moorella thermoacetica
Frequency
0.000
0.005
0.010
0.015
0.020
0.025
0.030
500
1000
1500
2000
Protein Size

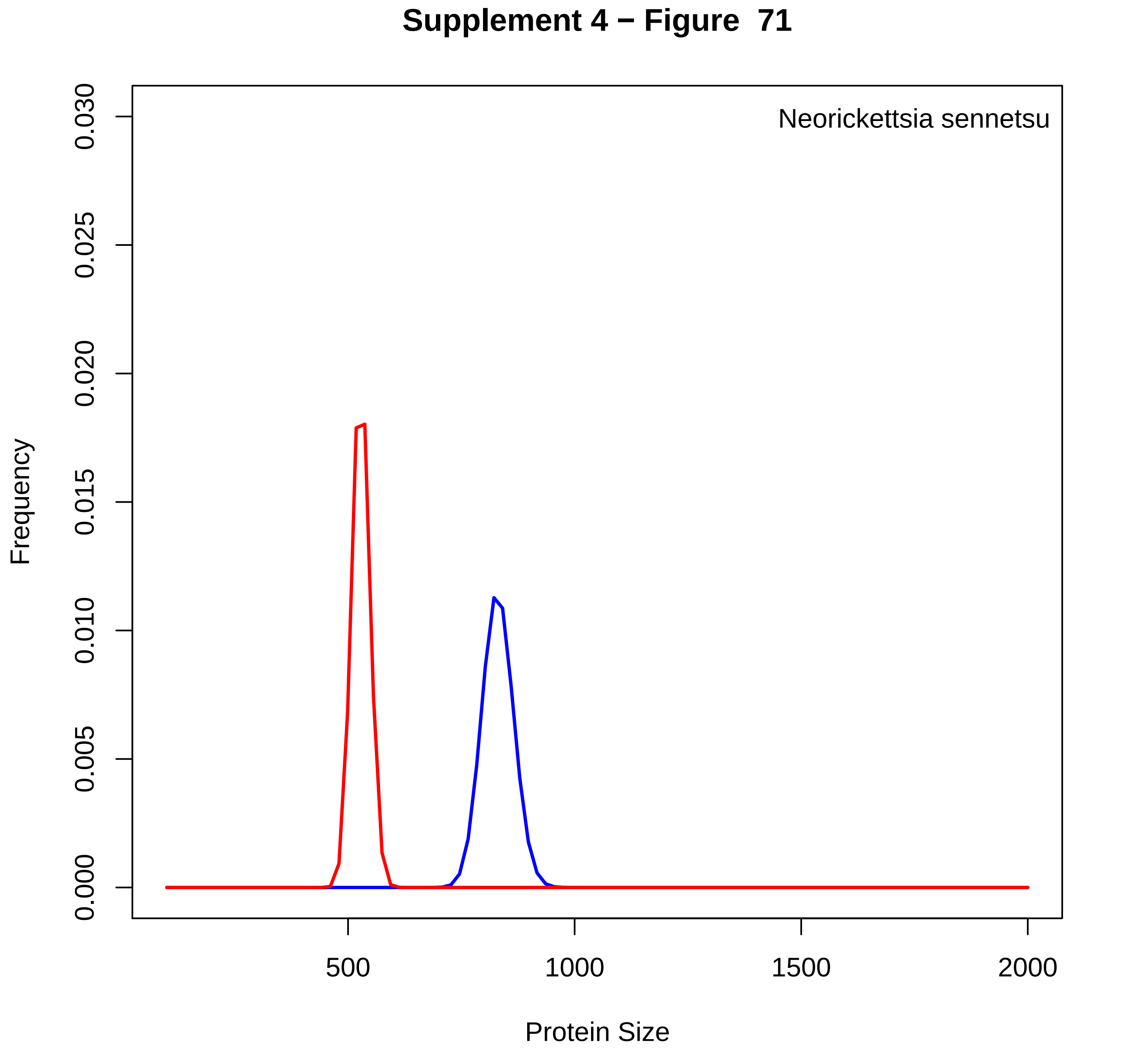
Supplement 4 − Figure 71
Neorickettsia sennetsu
Frequency
0.000
0.005
0.010
0.015
0.020
0.025
0.030
500
1000
1500
2000
Protein Size

Supplement 4 − Figure 72

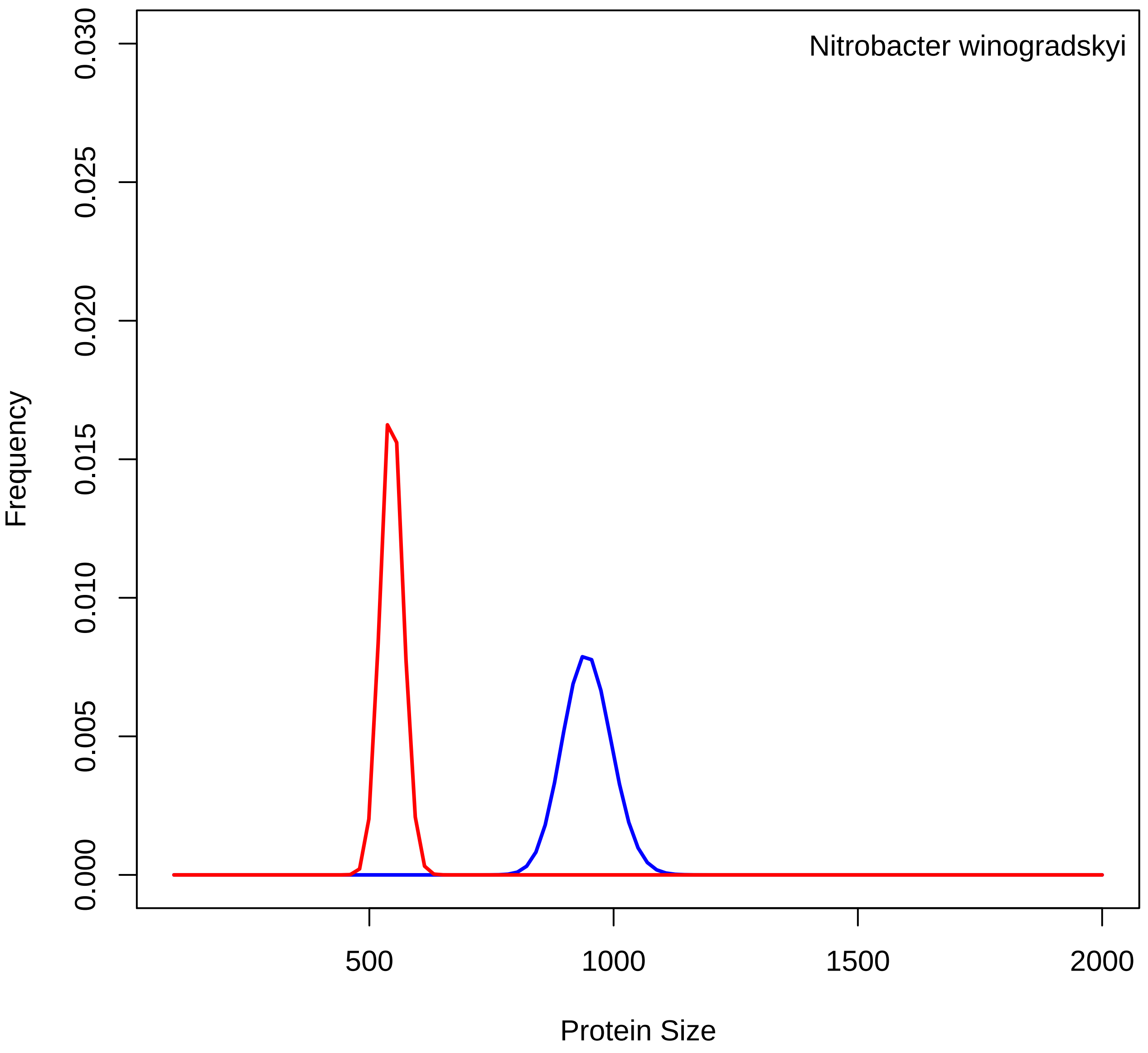

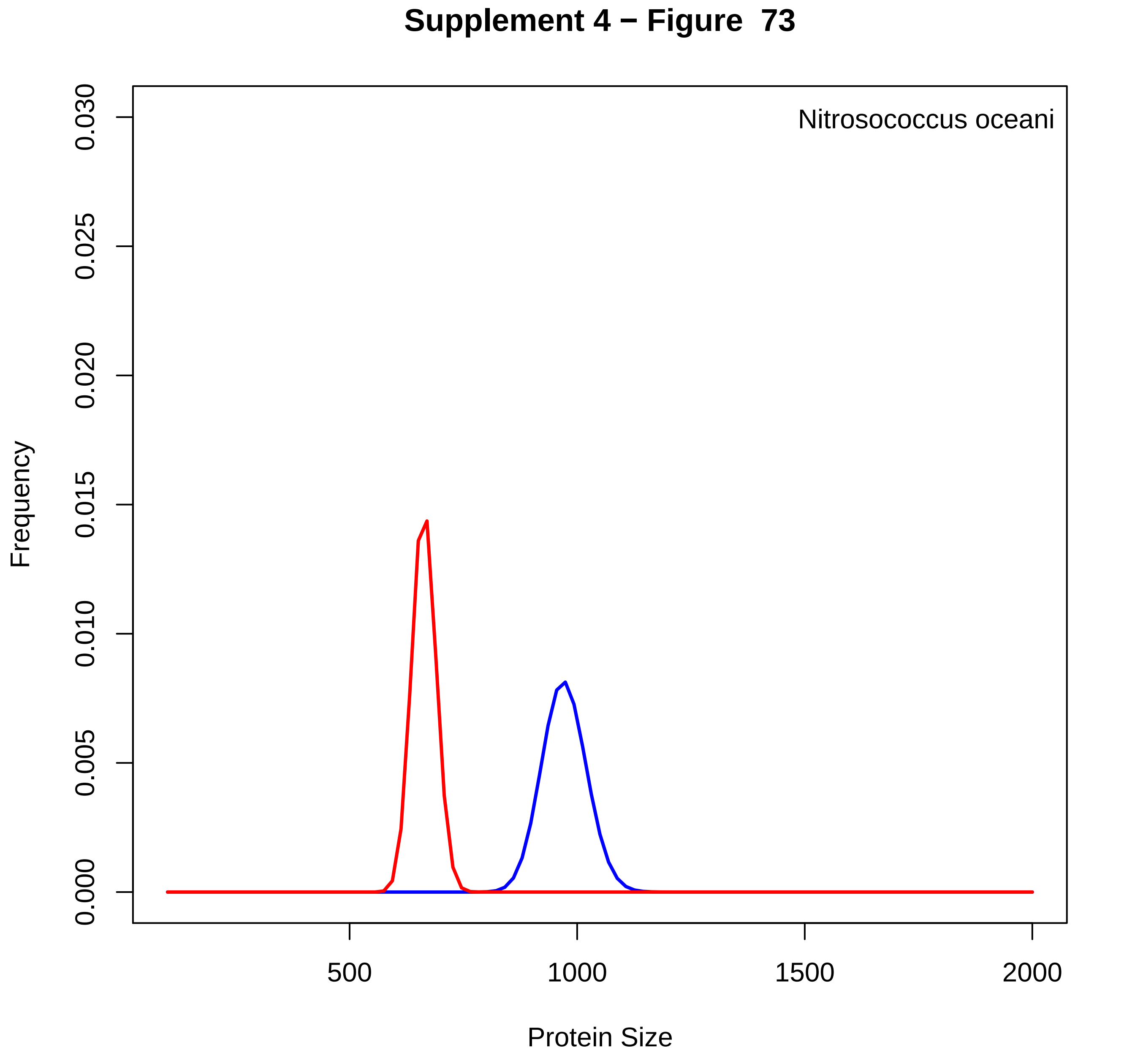
Supplement 4 − Figure 73
Nitrosococcus oceani
Frequency
Protein Size
0.000
0.005
0.010
0.015
0.020
0.025
0.030
500
1000
1500
2000

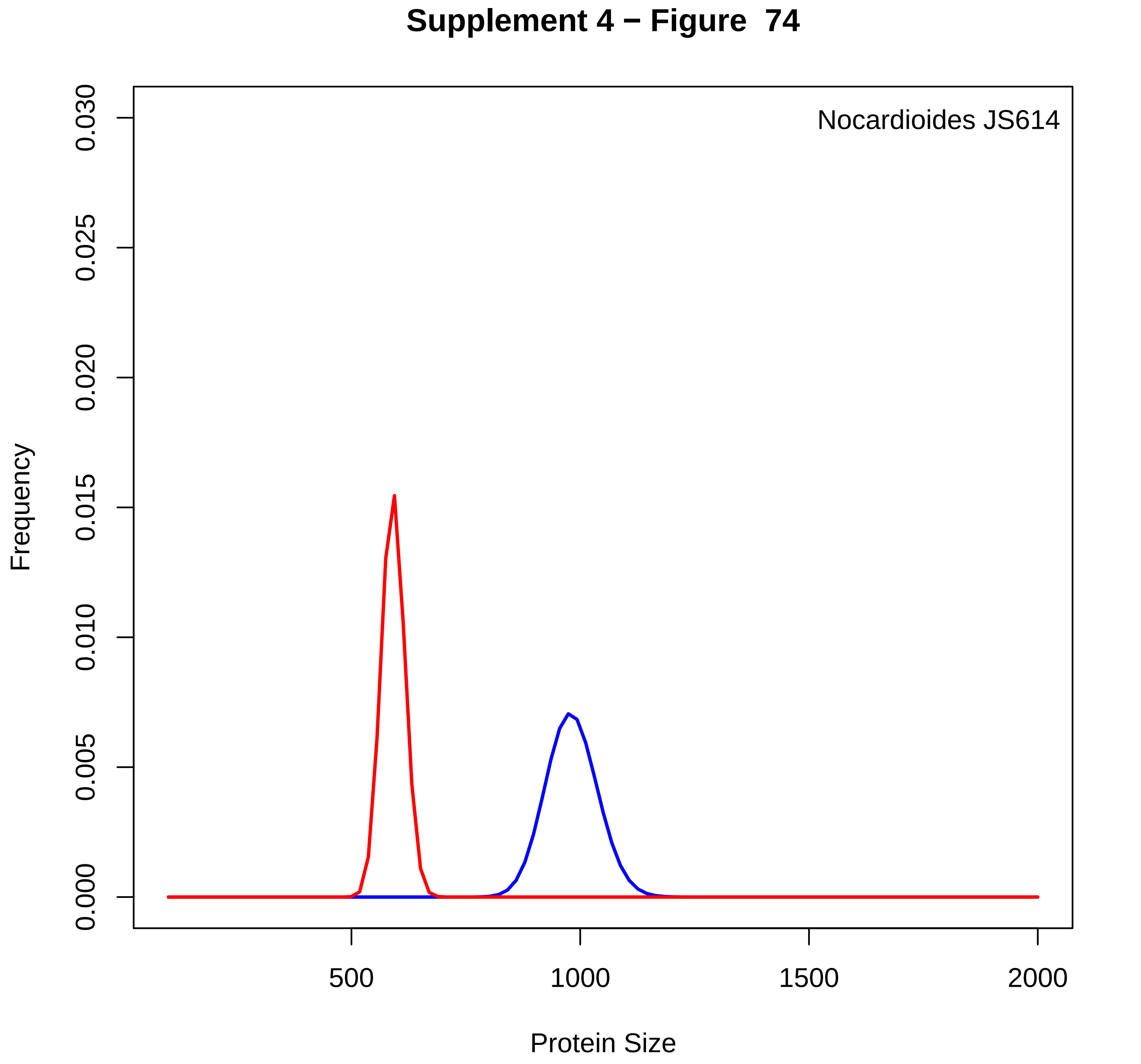

Supplement 4 − Figure 74
Nocardioides JS614
Frequency
0.000
0.005
0.010
0.015
0.020
0.025
0.030
500
1000
1500
2000
Protein Size

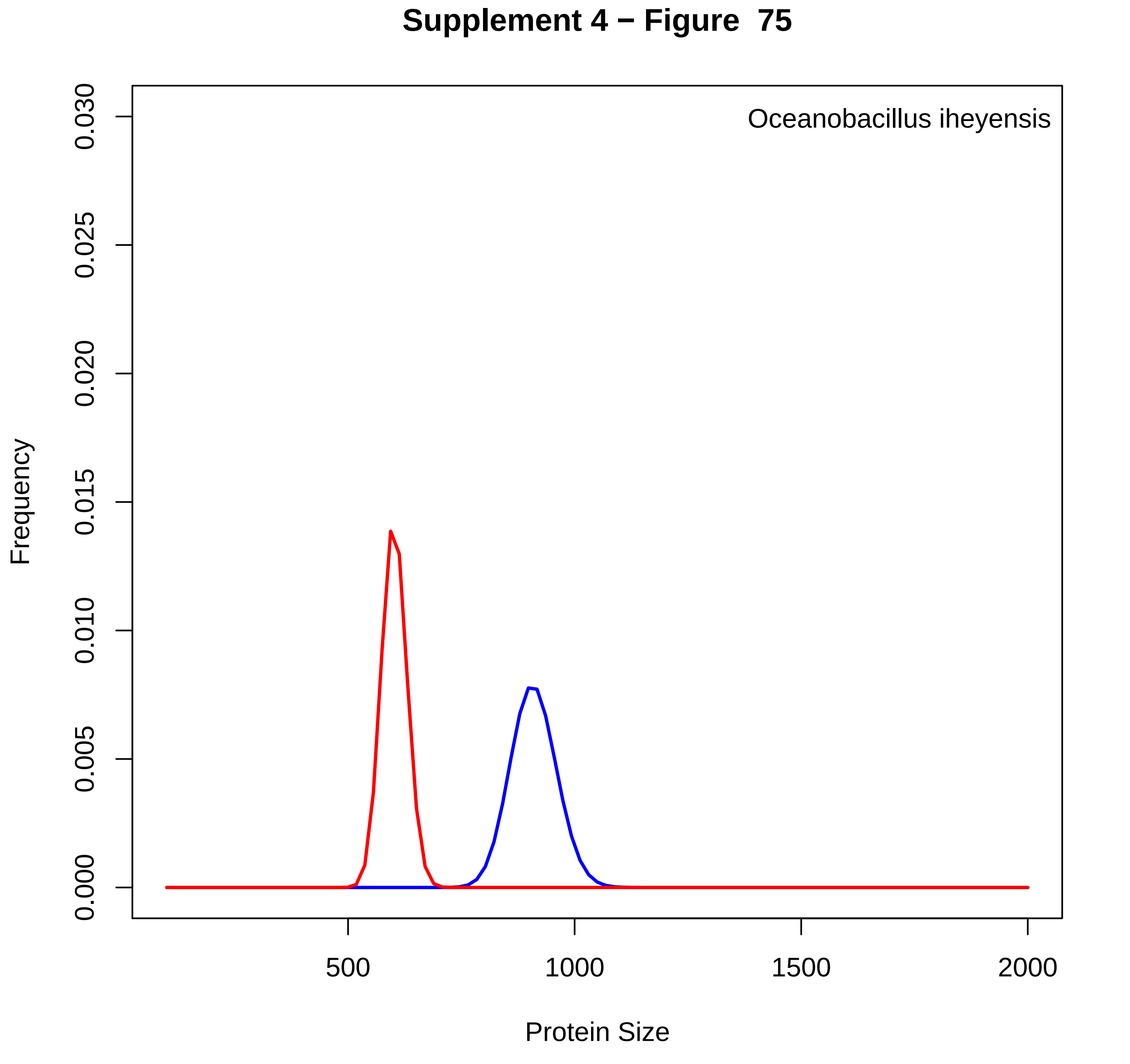
Supplement 4 − Figure 75
Oceanobacillus iheyensis
Frequency
0.000
0.005
0.010
0.015
0.020
0.025
0.030
500
1000
1500
2000
Protein Size

Supplement 4 − Figure 76

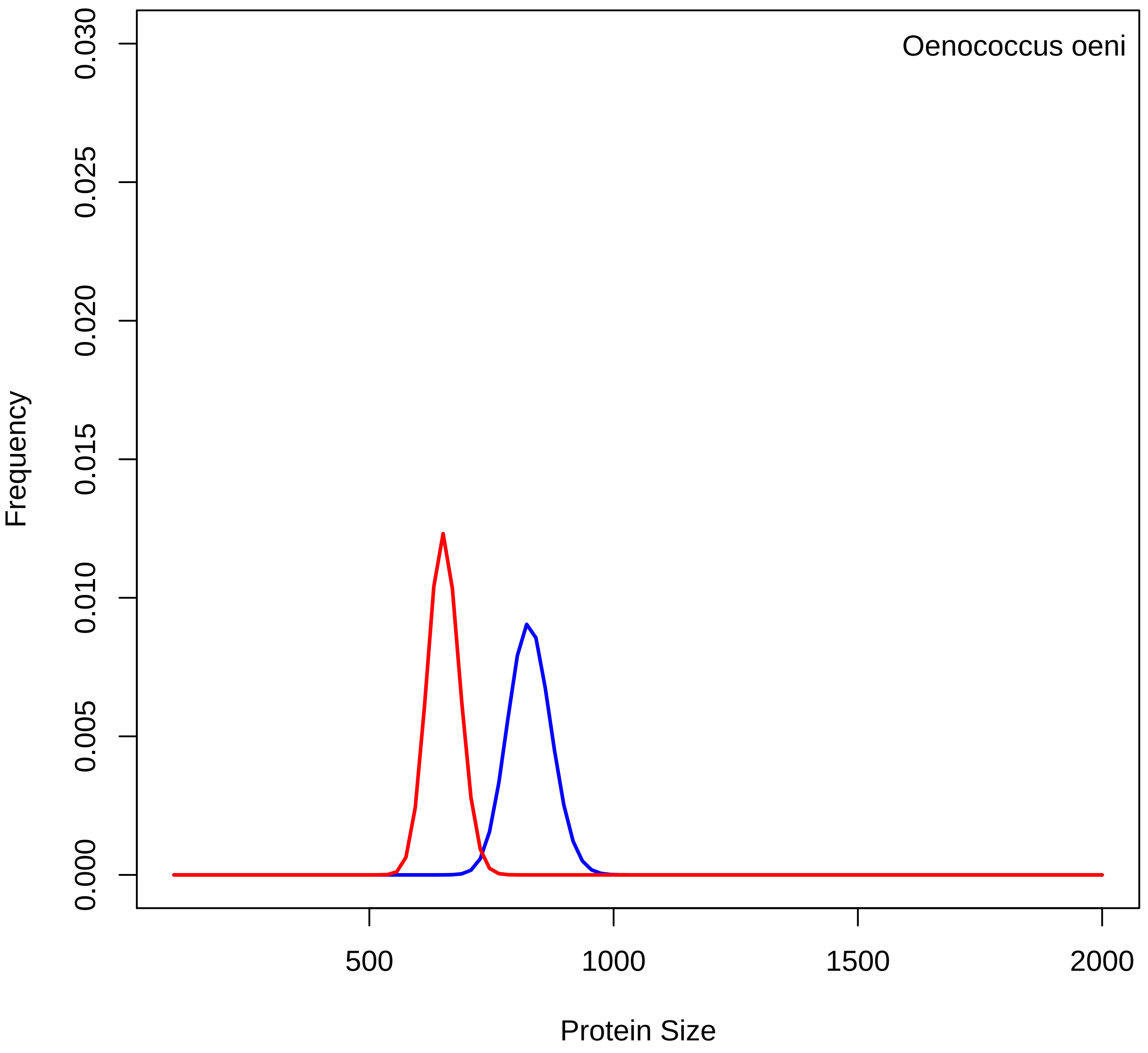

Supplement 4 − Figure 77

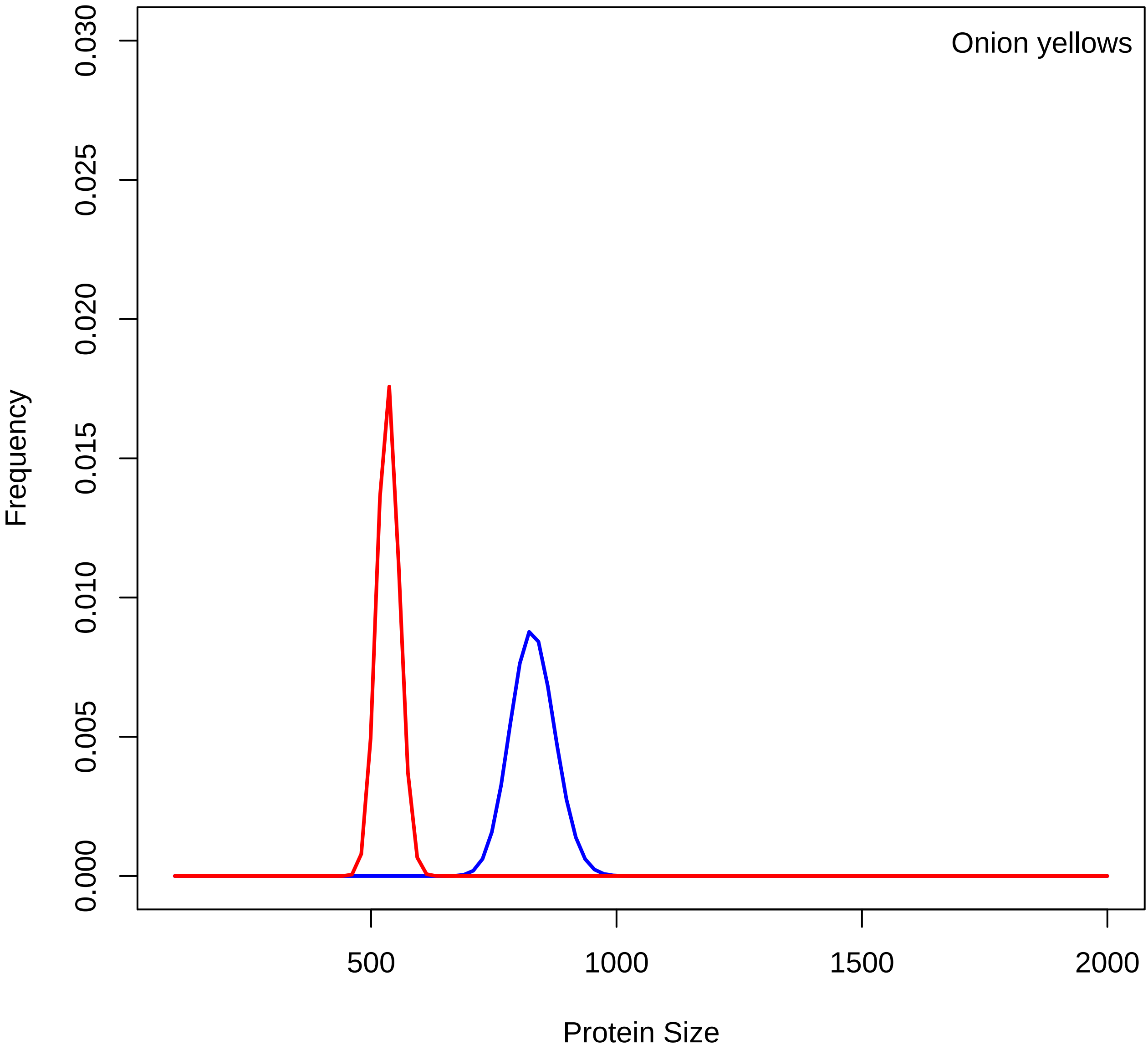

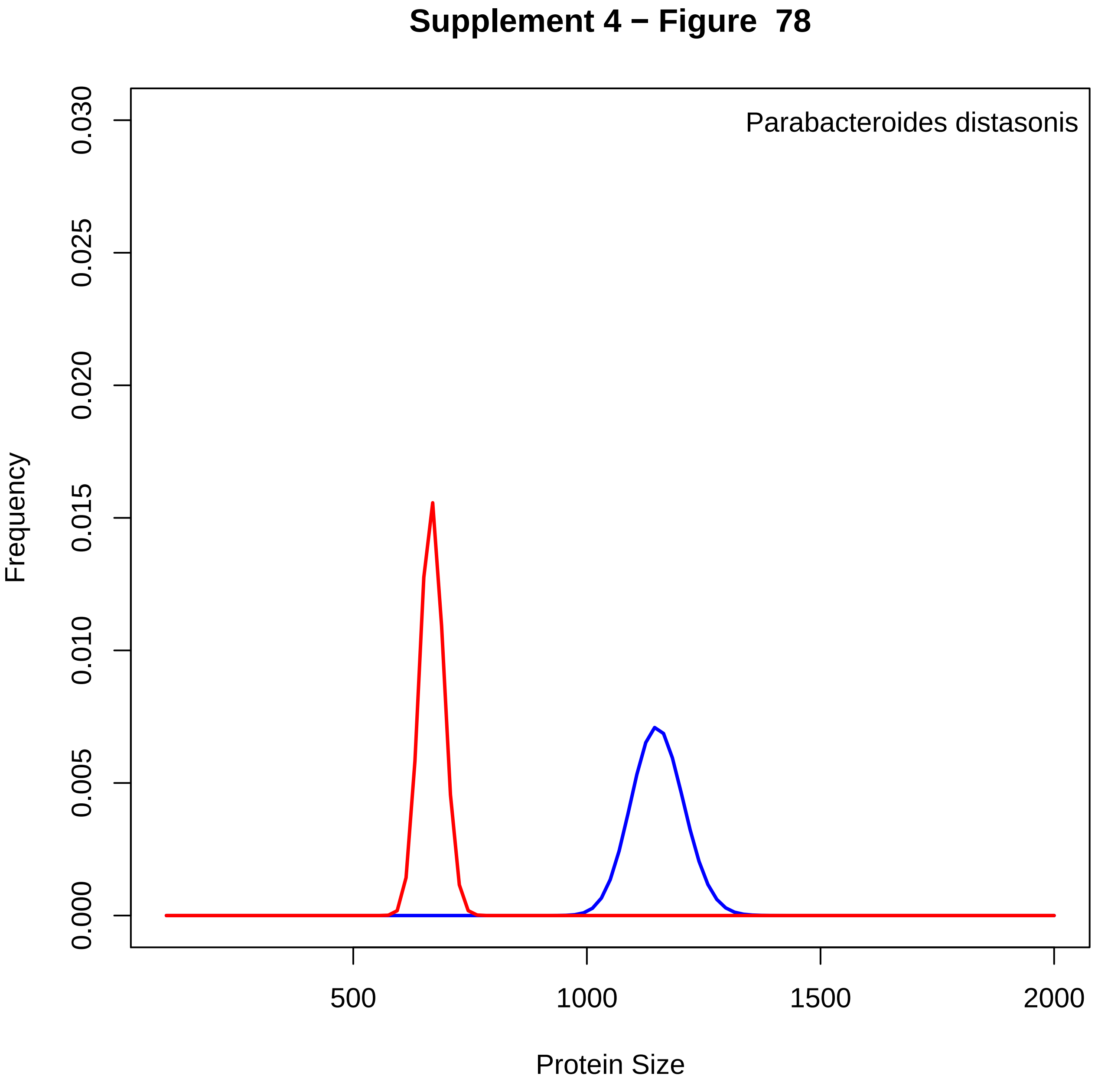
Supplement 4 − Figure 78
Parabacteroides distasonis
Frequency
0.000
0.005
0.010
0.015
0.020
0.025
0.030
500
1000
1500
2000
Protein Size

Supplement 4 − Figure 79

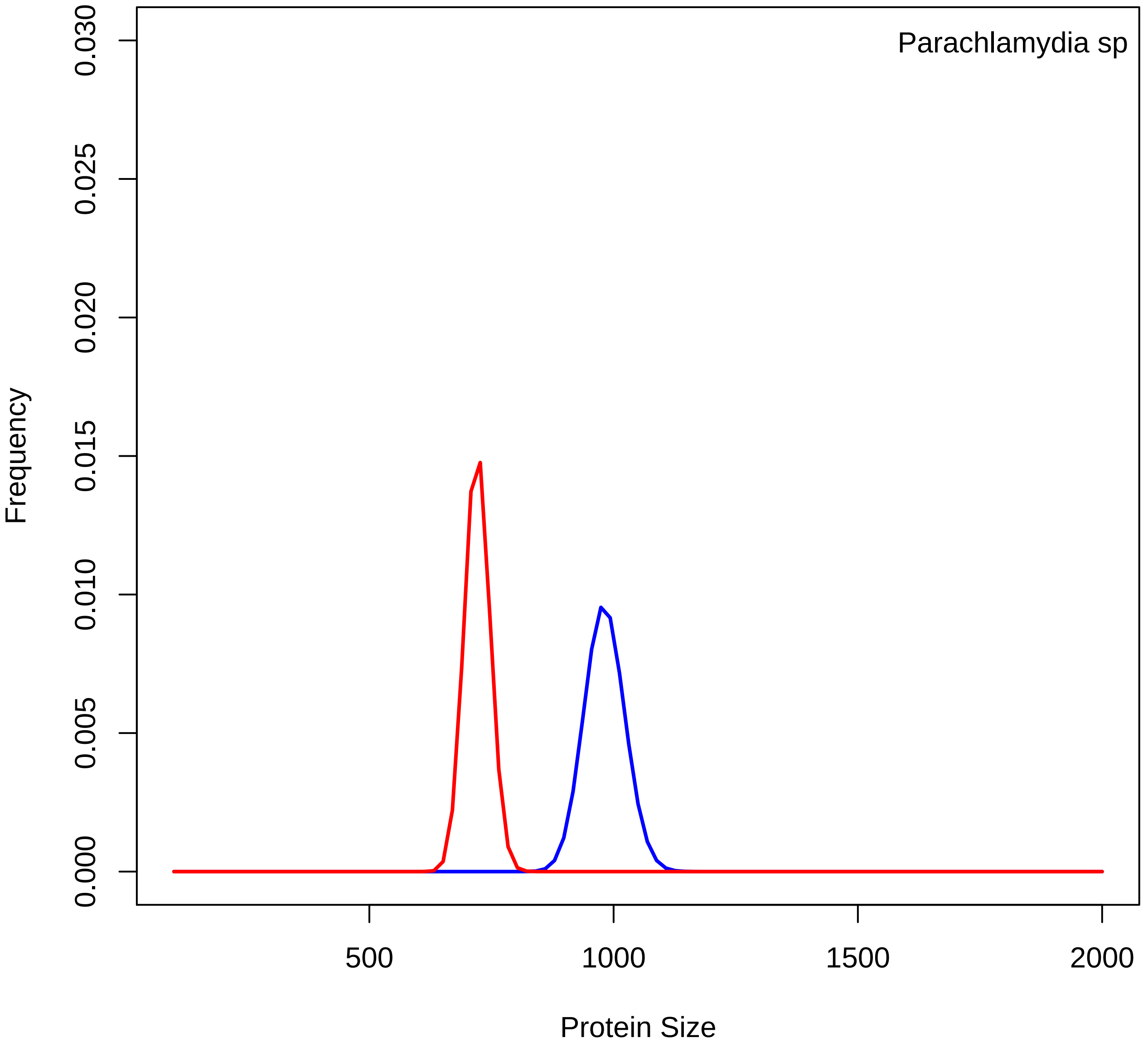

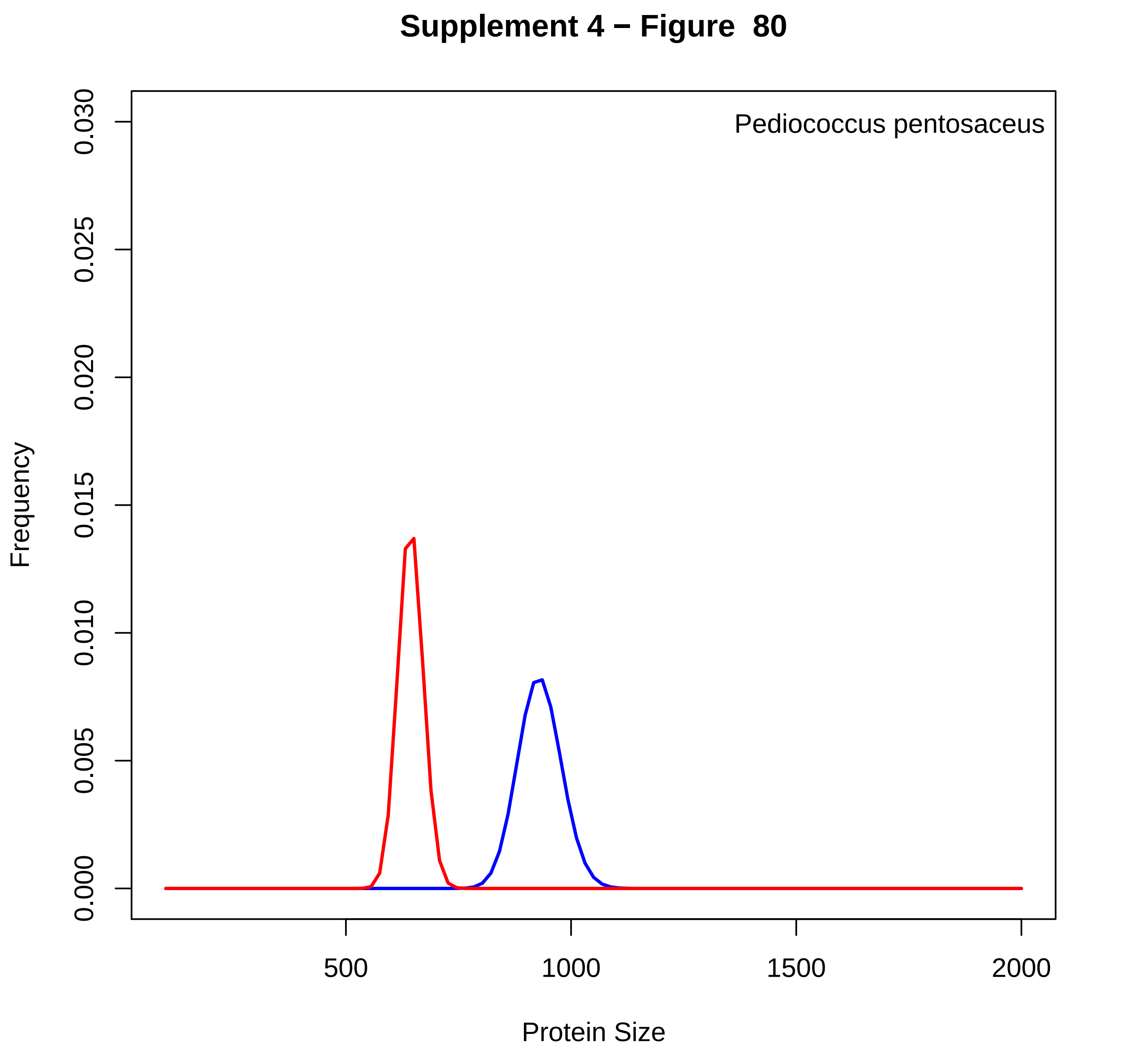
Supplement 4 − Figure 80
Pediococcus pentosaceus
Frequency
0.000
0.005
0.010
0.015
0.020
0.025
0.030
500
1000
1500
2000
Protein Size

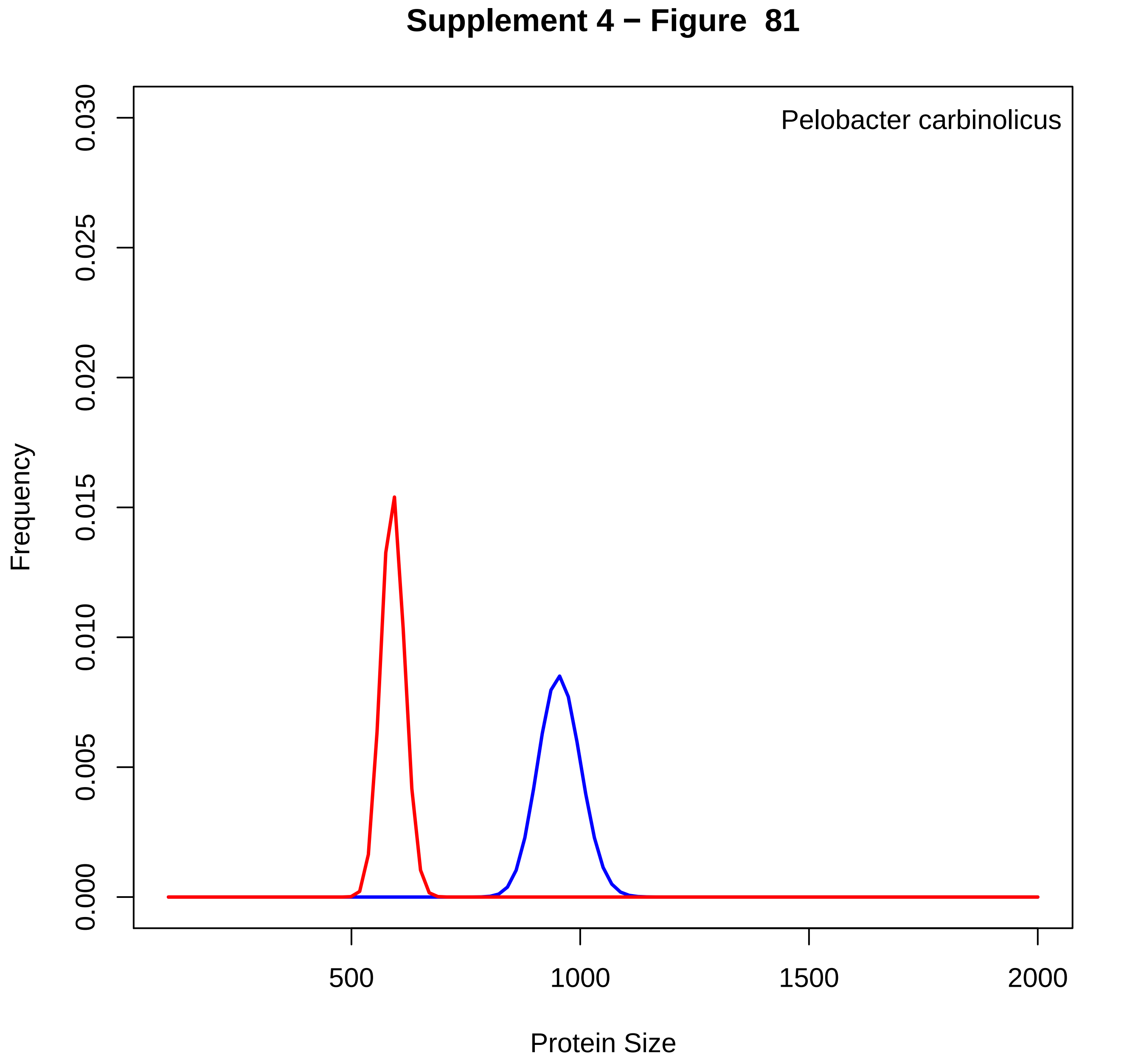
Supplement 4 − Figure 81
Pelobacter carbinolicus
Frequency
0.000
0.005
0.010
0.015
0.020
0.025
0.030
500
1000
1500
2000
Protein Size

Supplement 4 − Figure 82

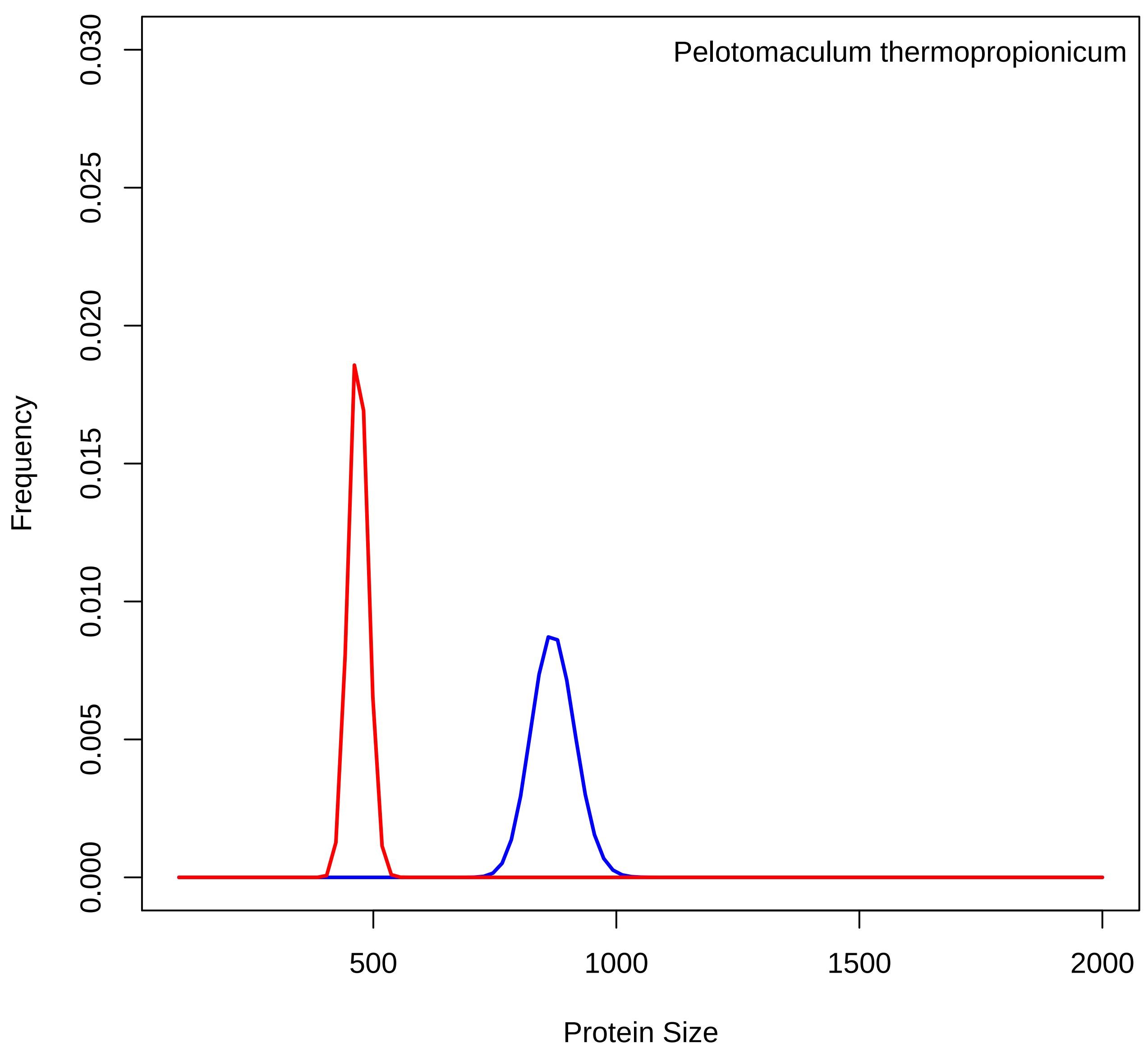

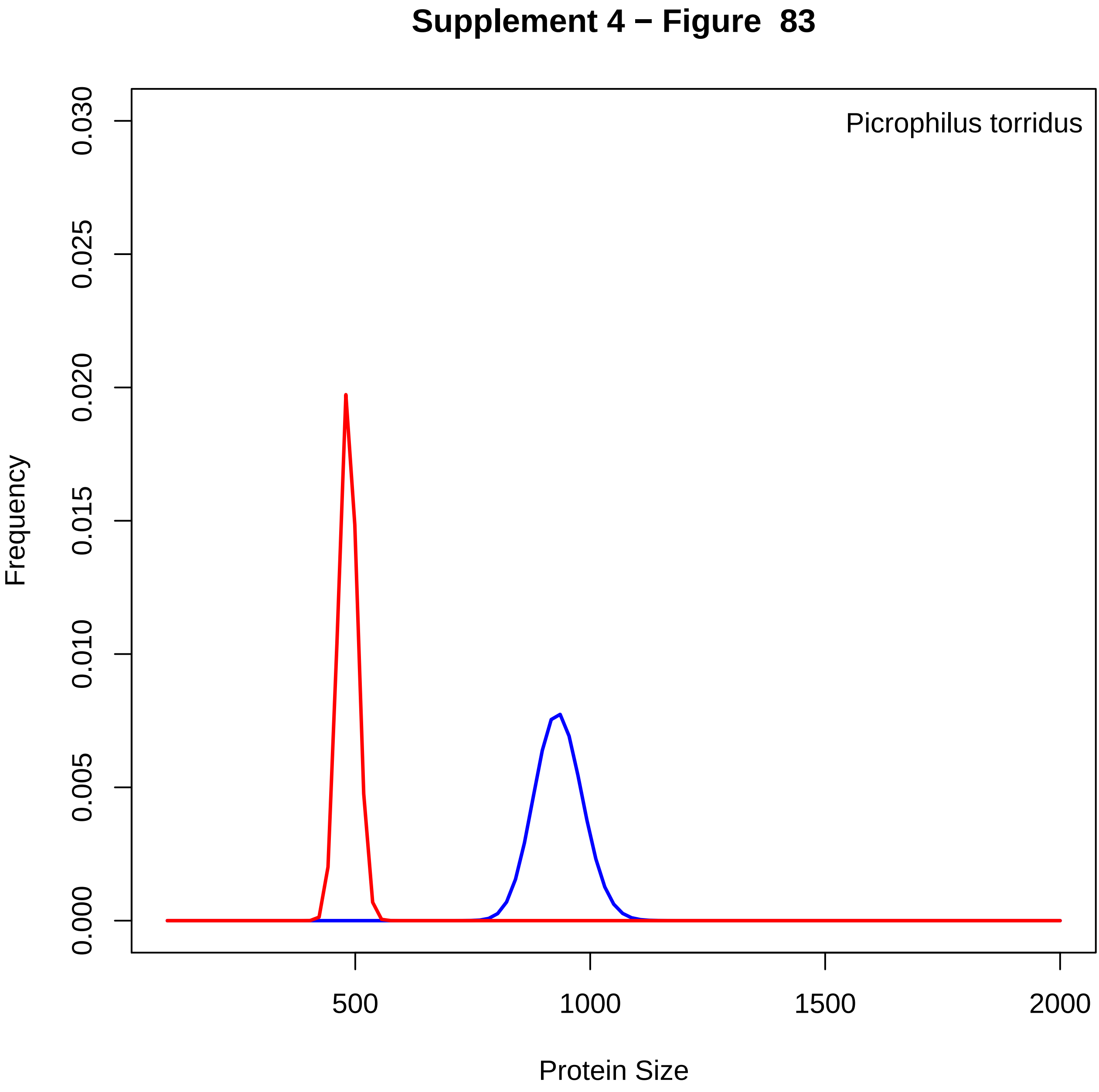

Supplement 4 − Figure 83
Picrophilus torridus
Frequency
0.000
0.005
0.010
0.015
0.020
0.025
0.030
500
1000
1500
2000
Protein Size

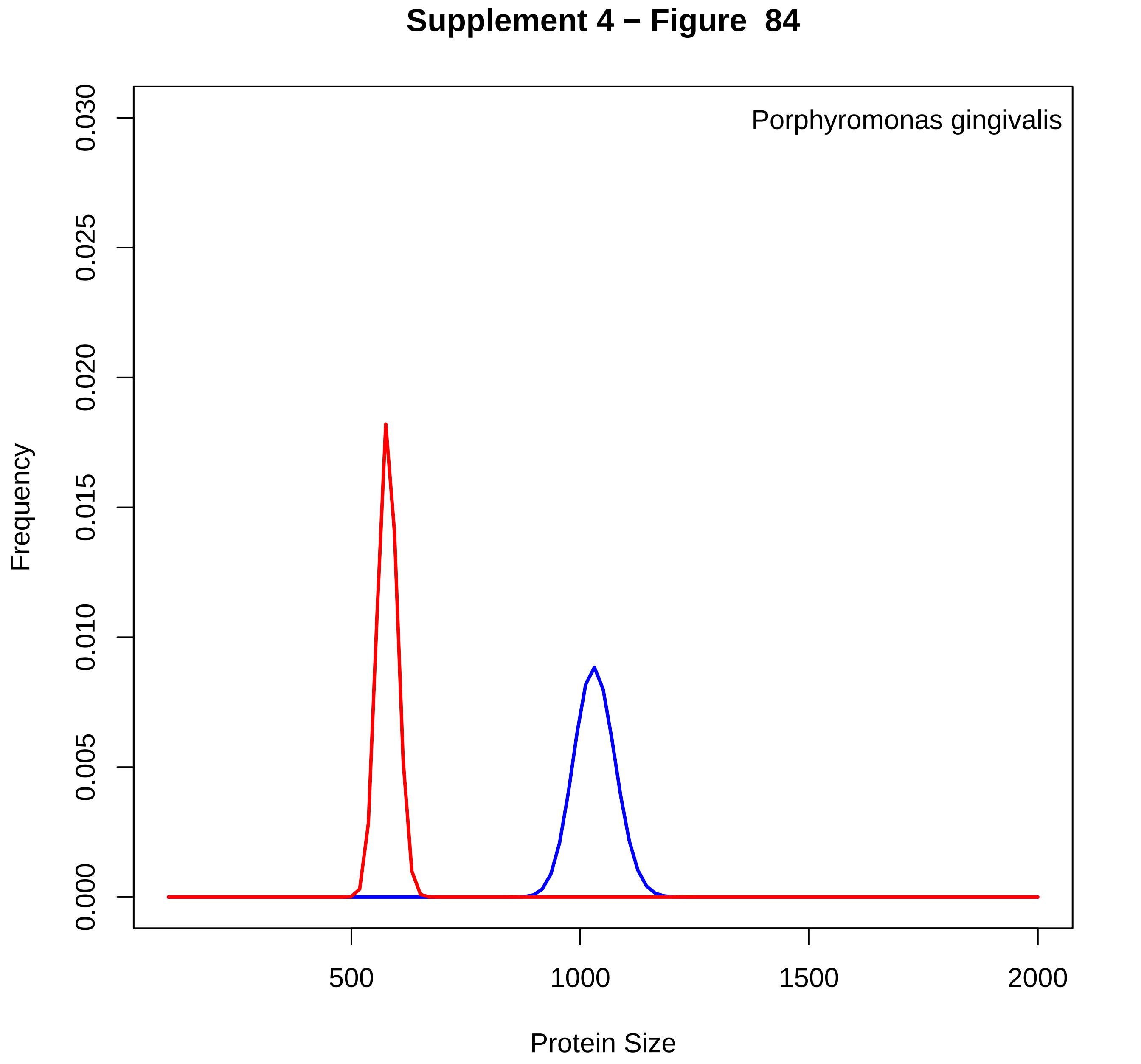
Supplement 4 − Figure 84
Porphyromonas gingivalis
Frequency
0.000
0.005
0.010
0.015
0.020
0.025
0.030
500
1000
1500
2000
Protein Size

Supplement 4 − Figure 85

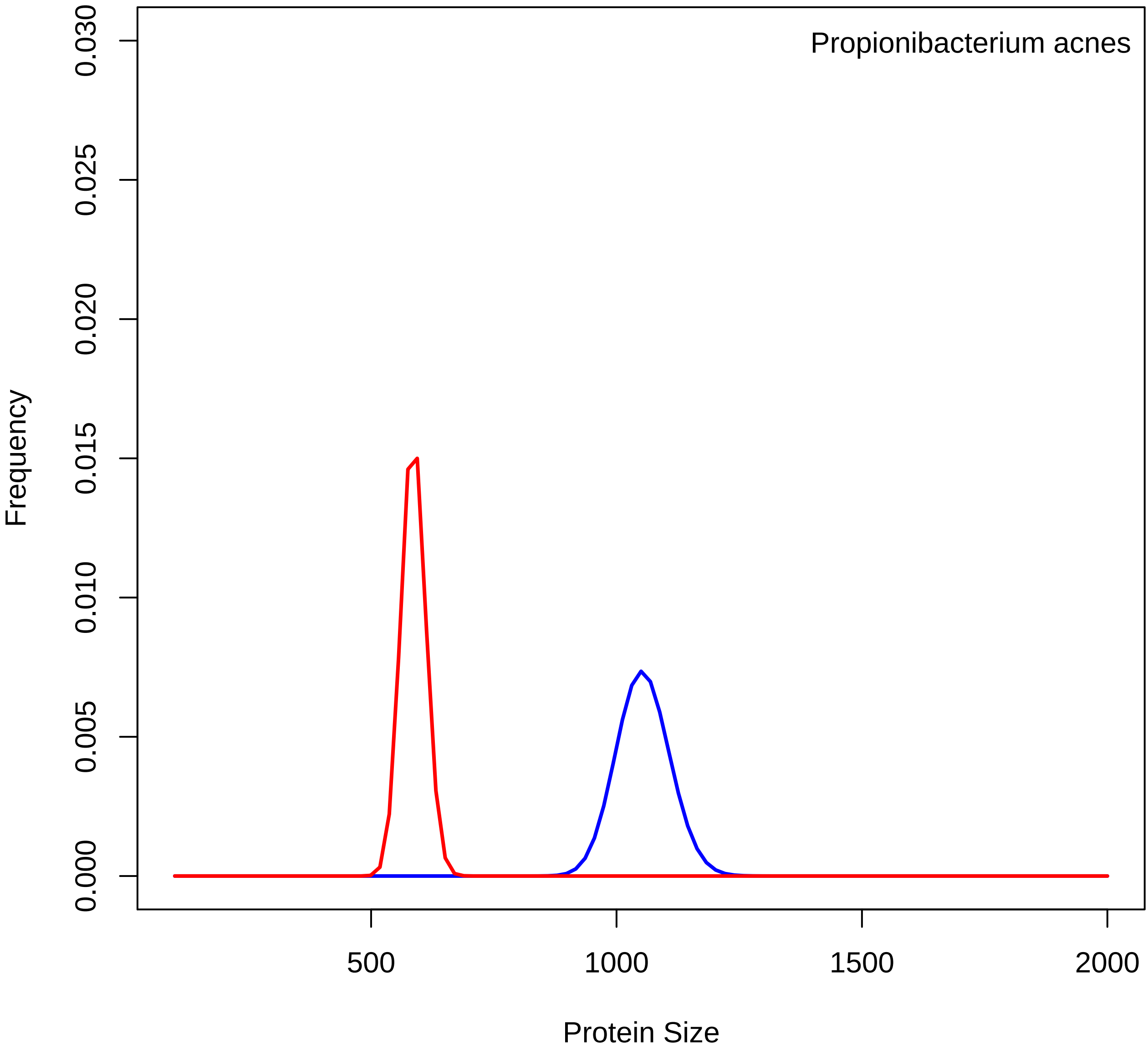

Supplement 4 − Figure 86

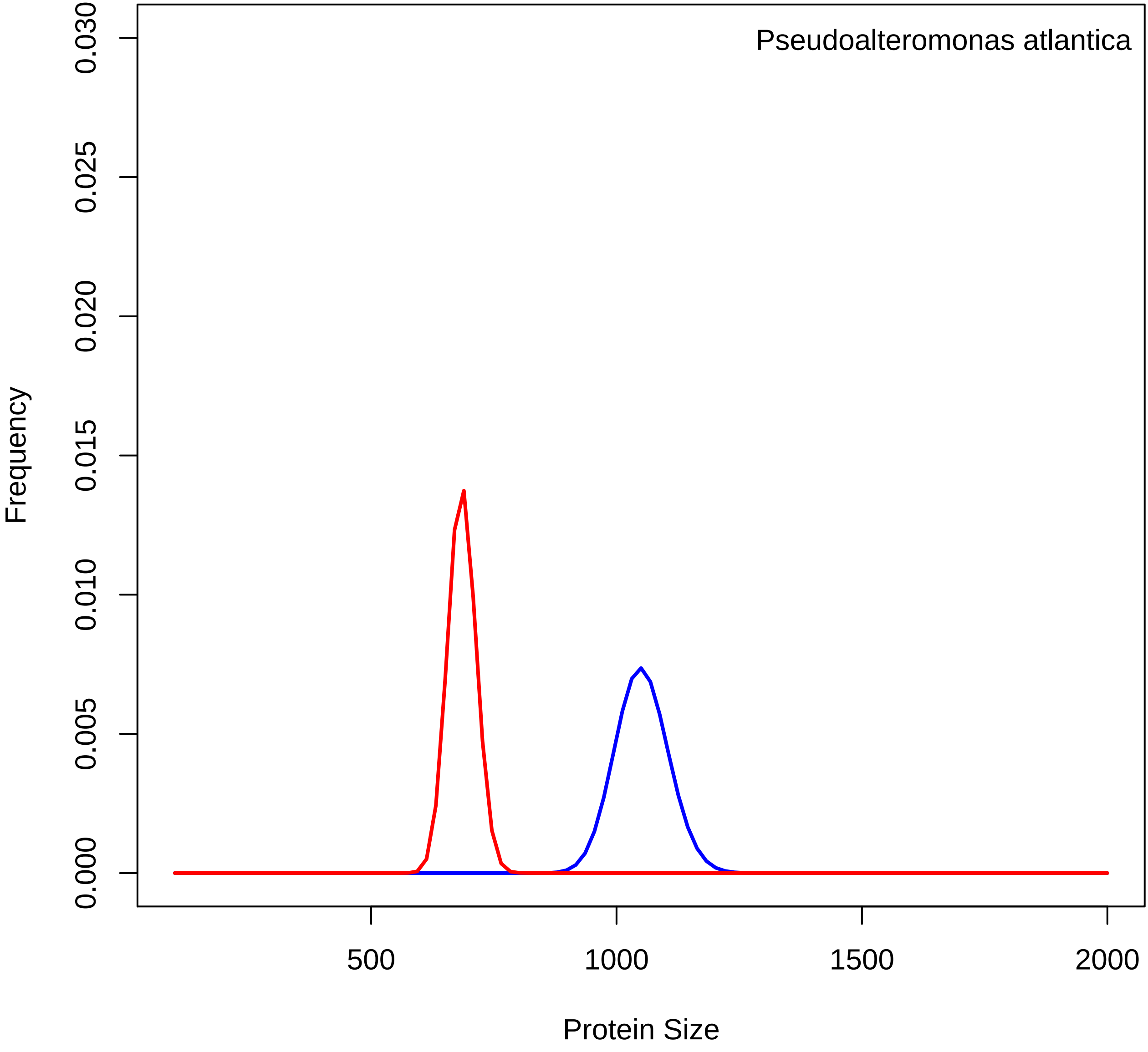

Supplement 4 − Figure 87

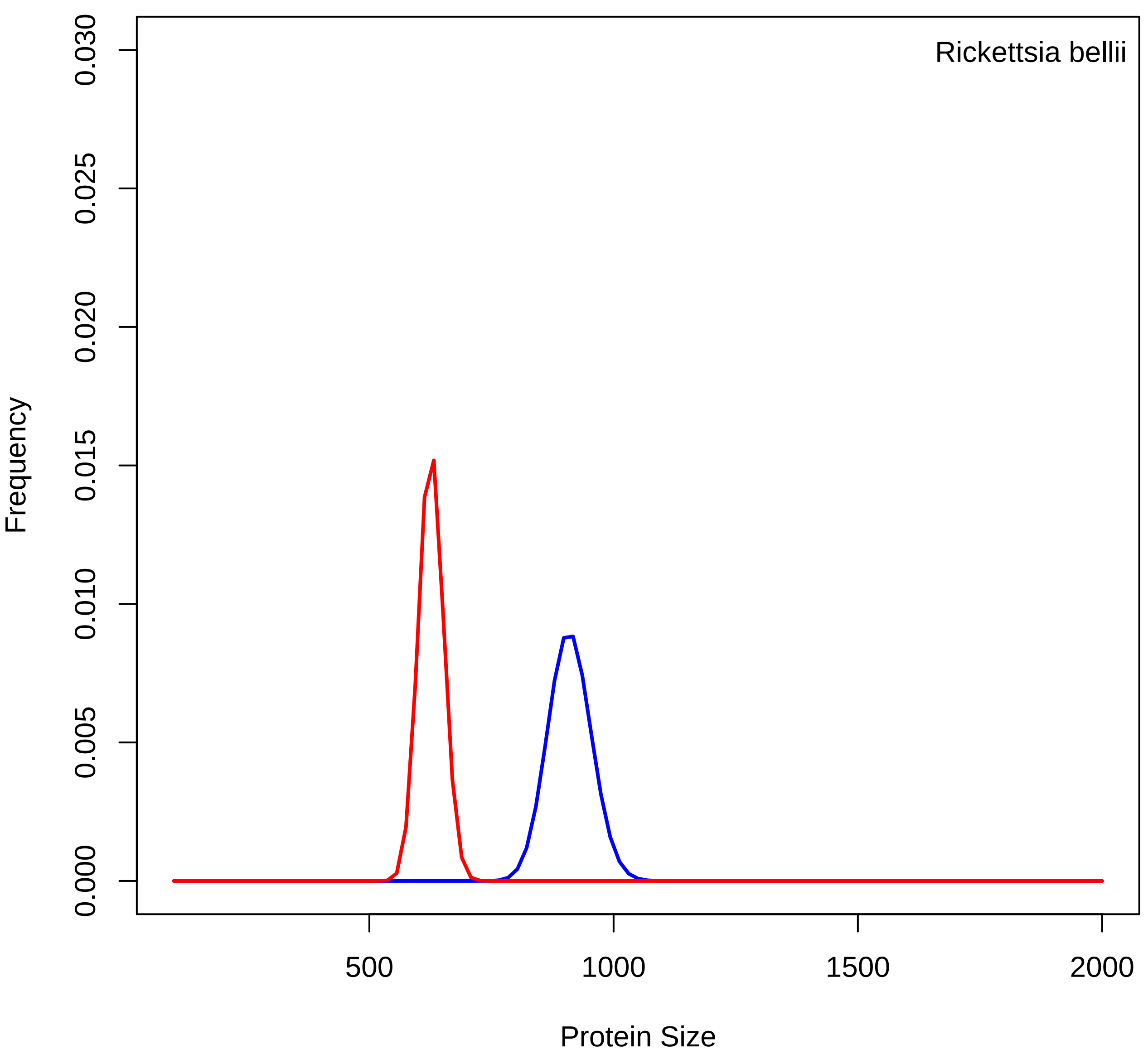

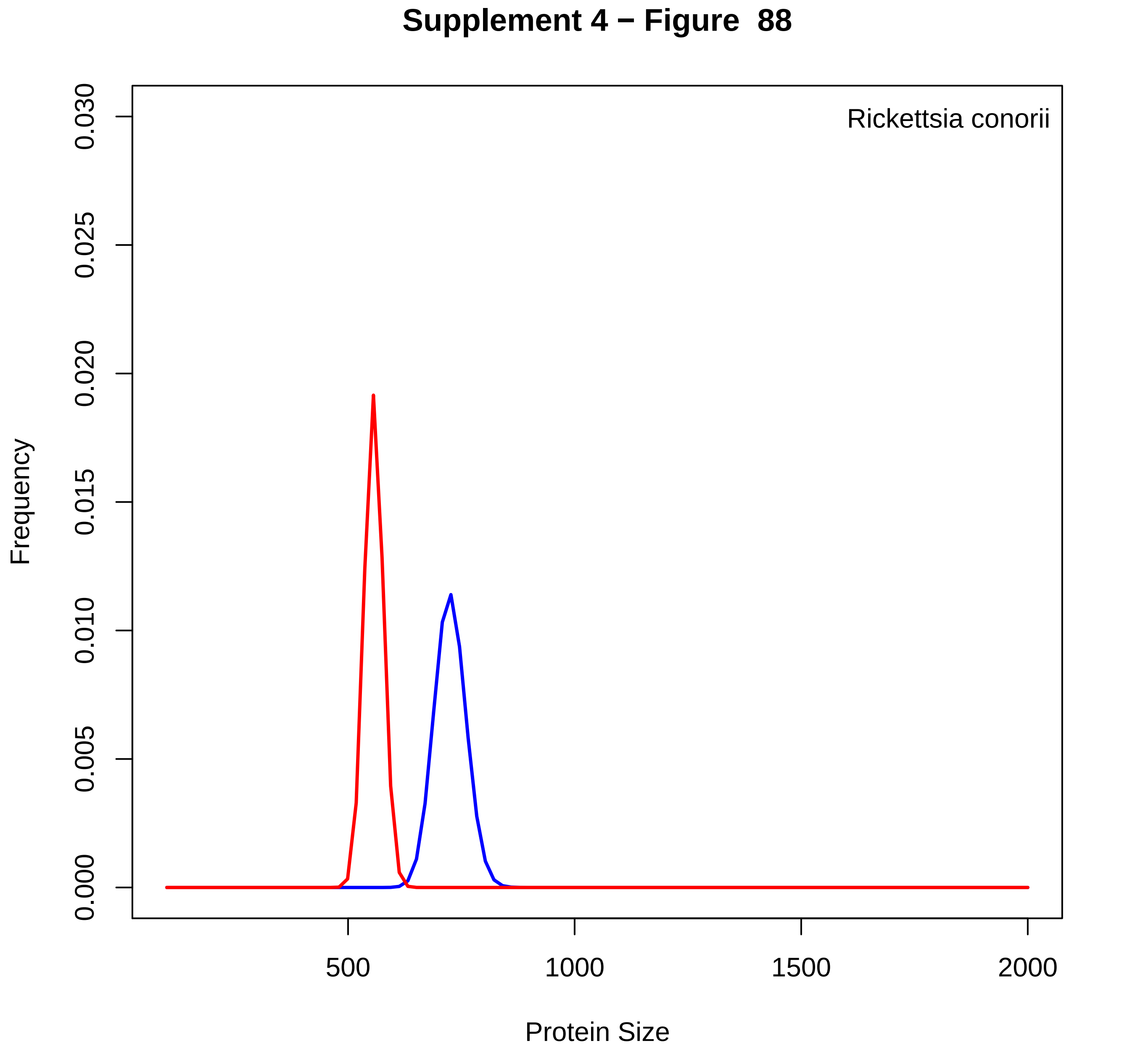
Supplement 4 − Figure 88
Rickettsia conorii
Frequency
0.000
0.005
0.010
0.015
0.020
0.025
0.030
500
1000
1500
2000
Protein Size

Supplement 4 − Figure 89

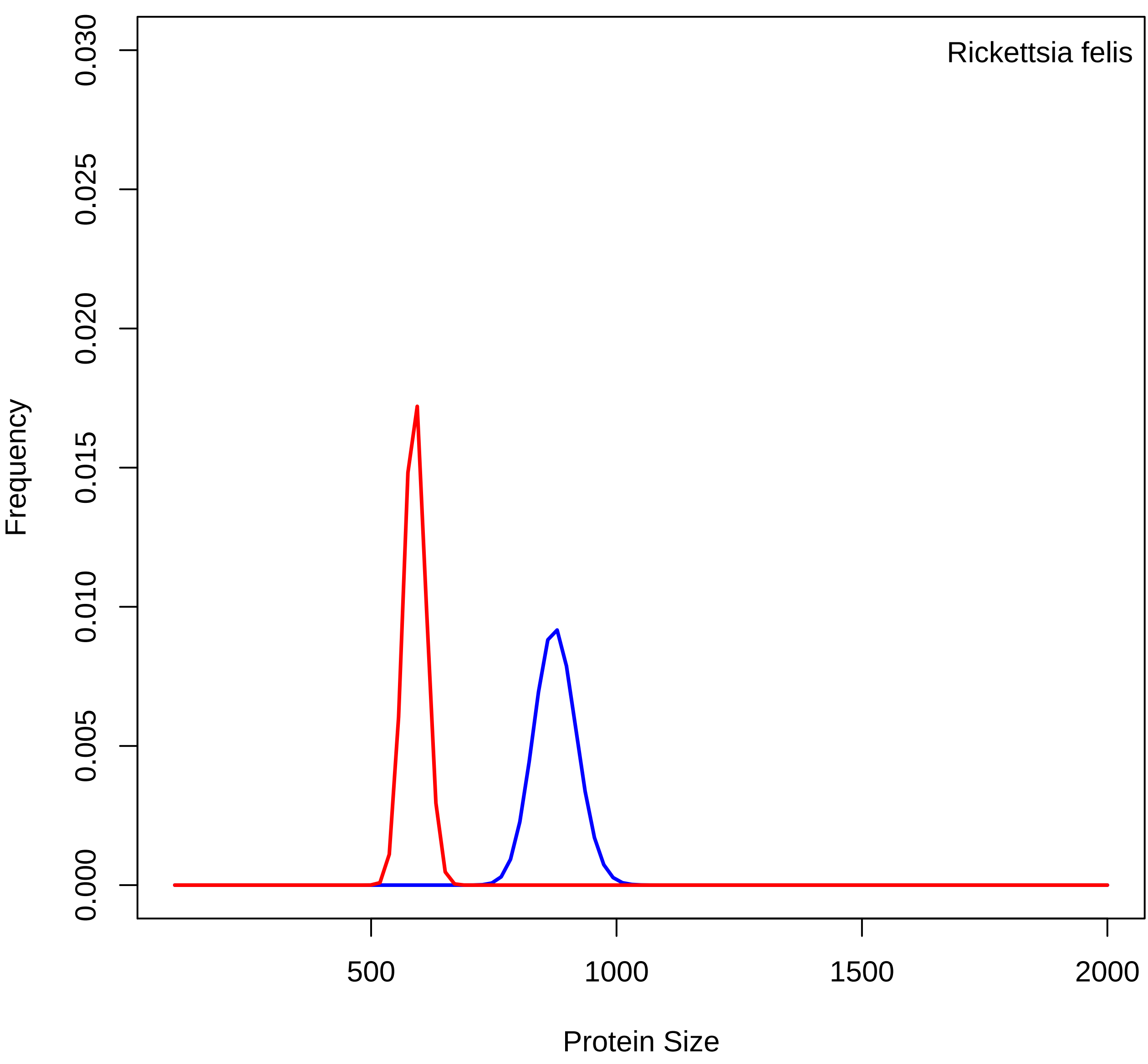

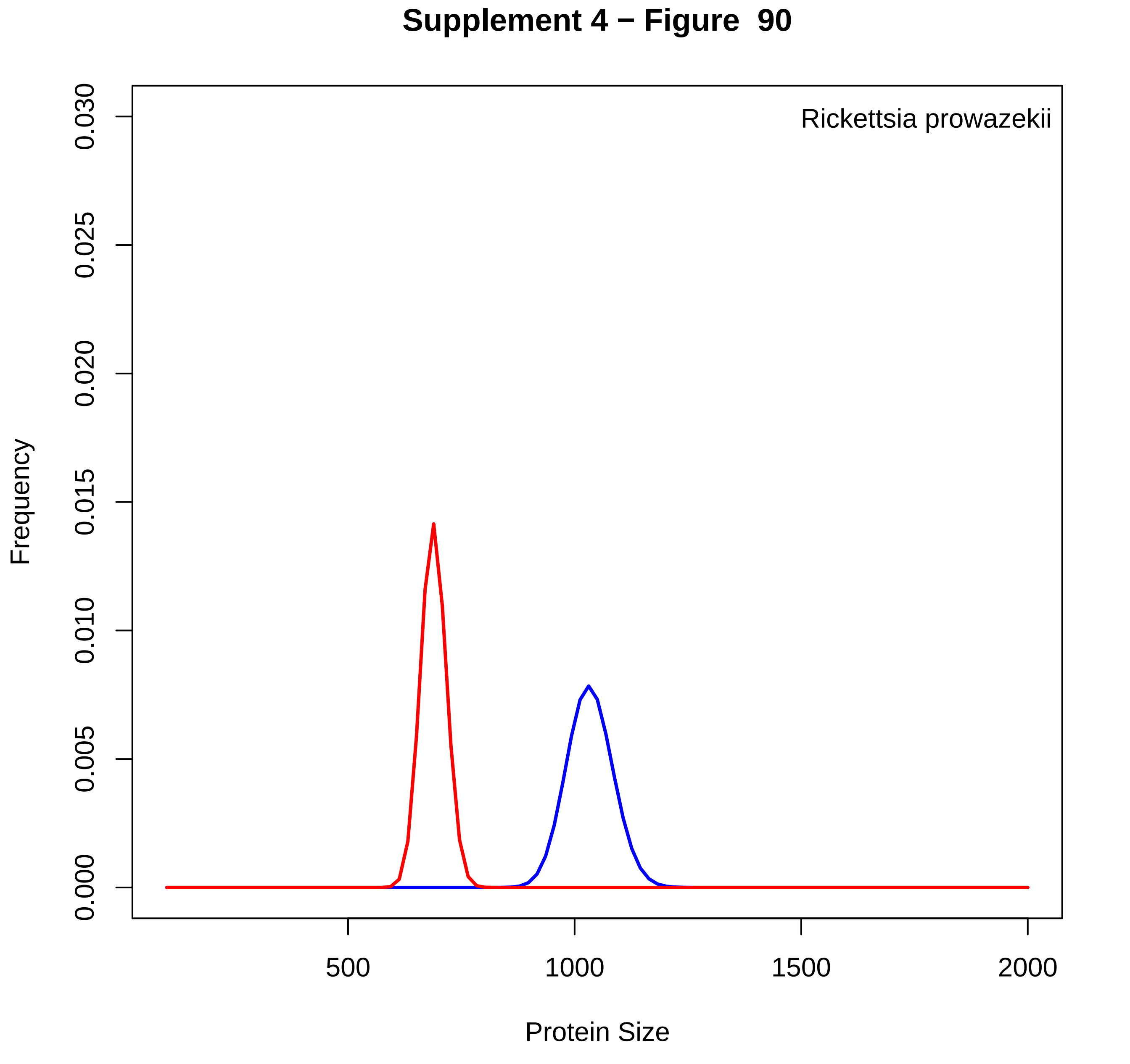
Supplement 4 − Figure 90
Rickettsia prowazekii
Frequency
0.000
0.005
0.010
0.015
0.020
0.025
0.030
500
1000
1500
2000
Protein Size

Supplement 4 − Figure 91

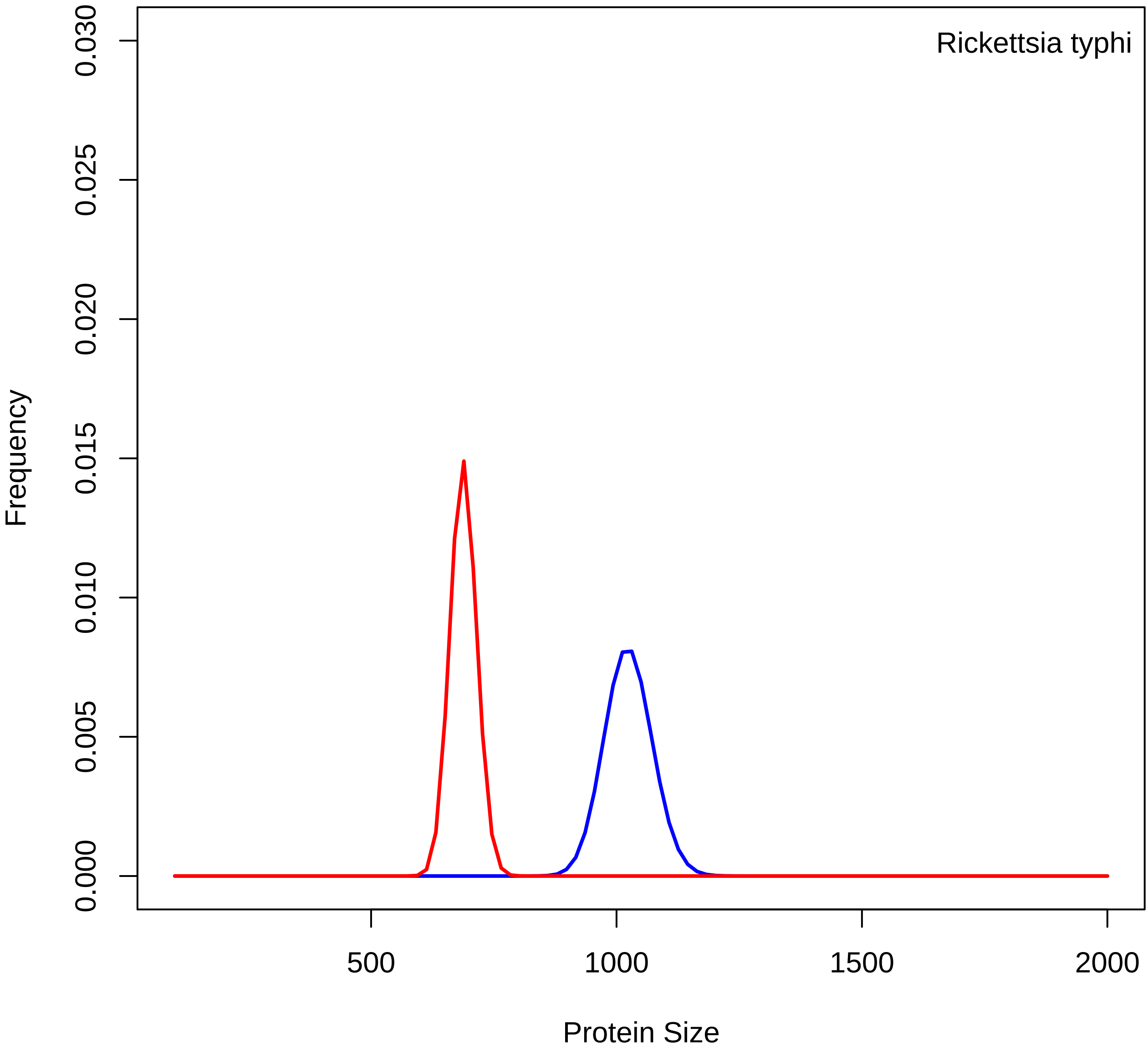

Supplement 4 − Figure 92

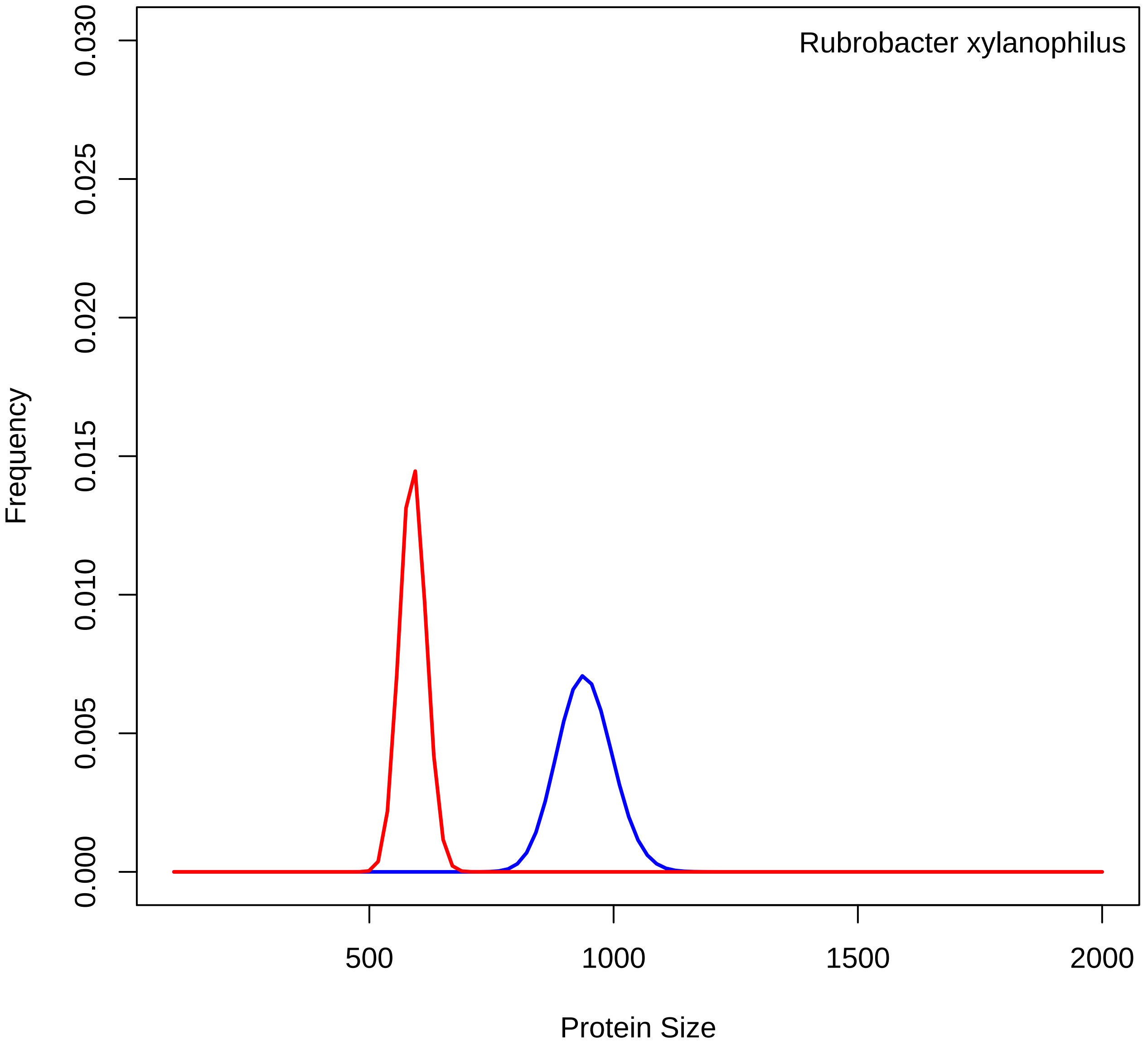

Supplement 4 − Figure 93

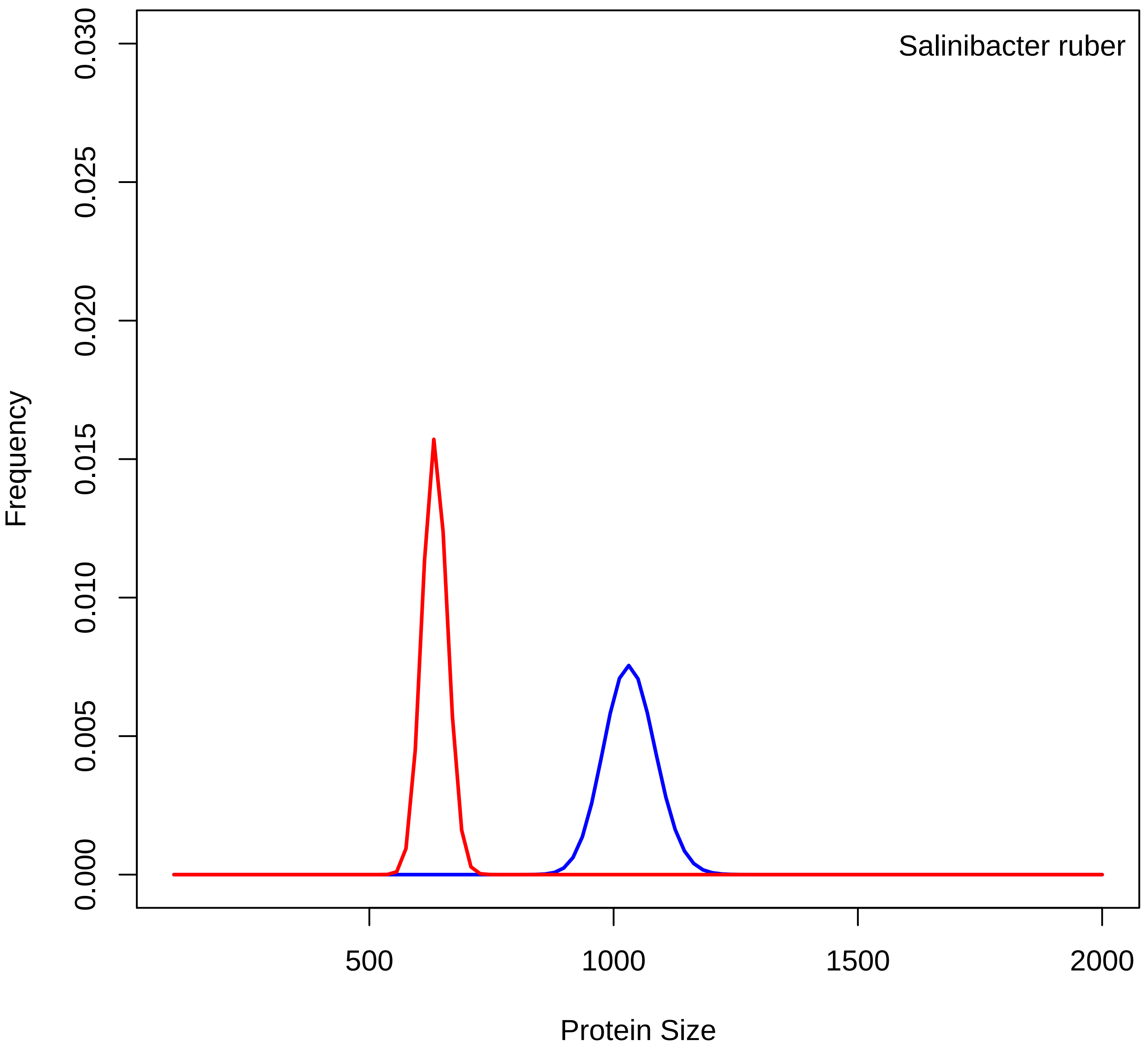

Supplement 4 − Figure 94

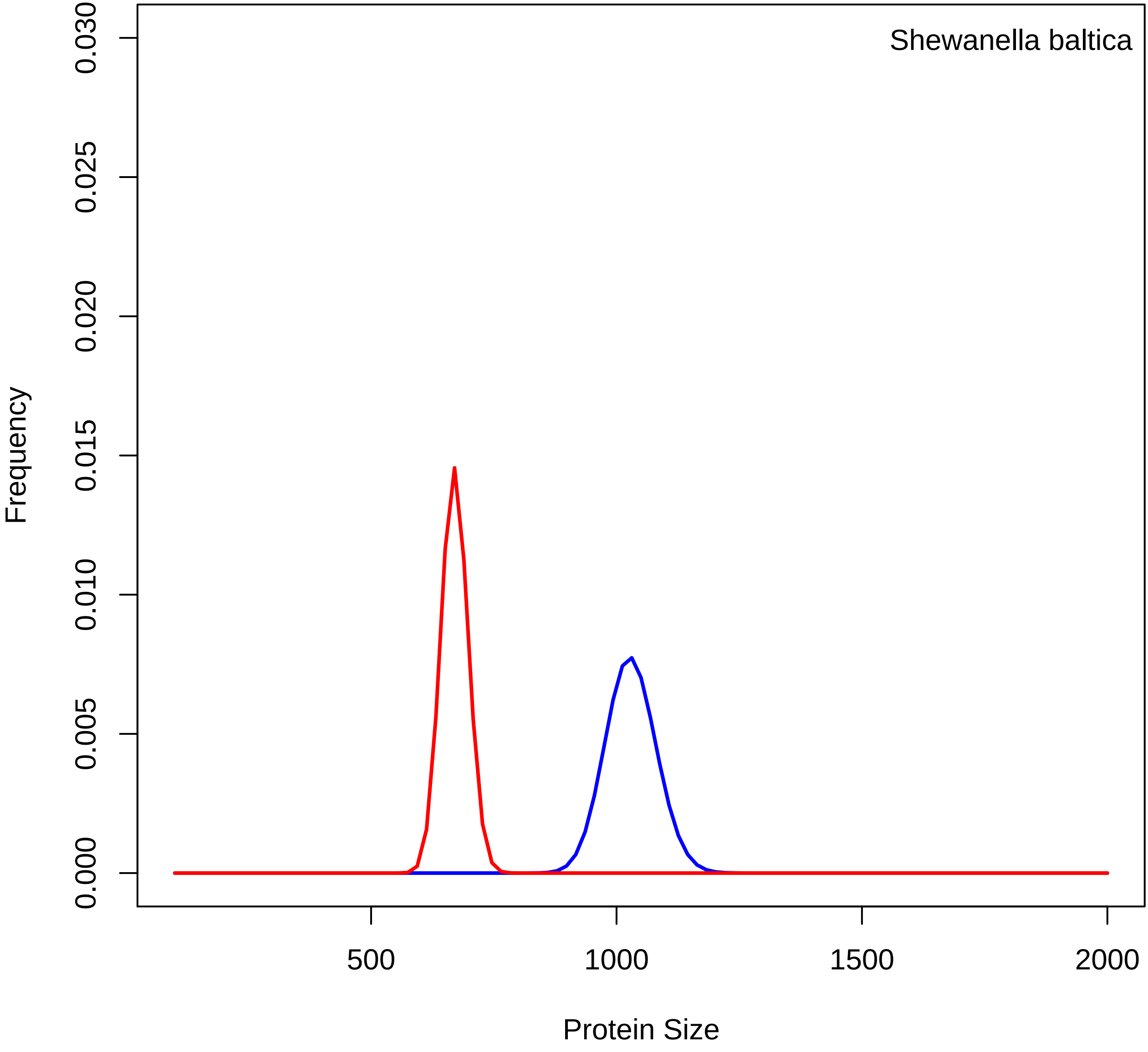

Supplement 4 − Figure 95

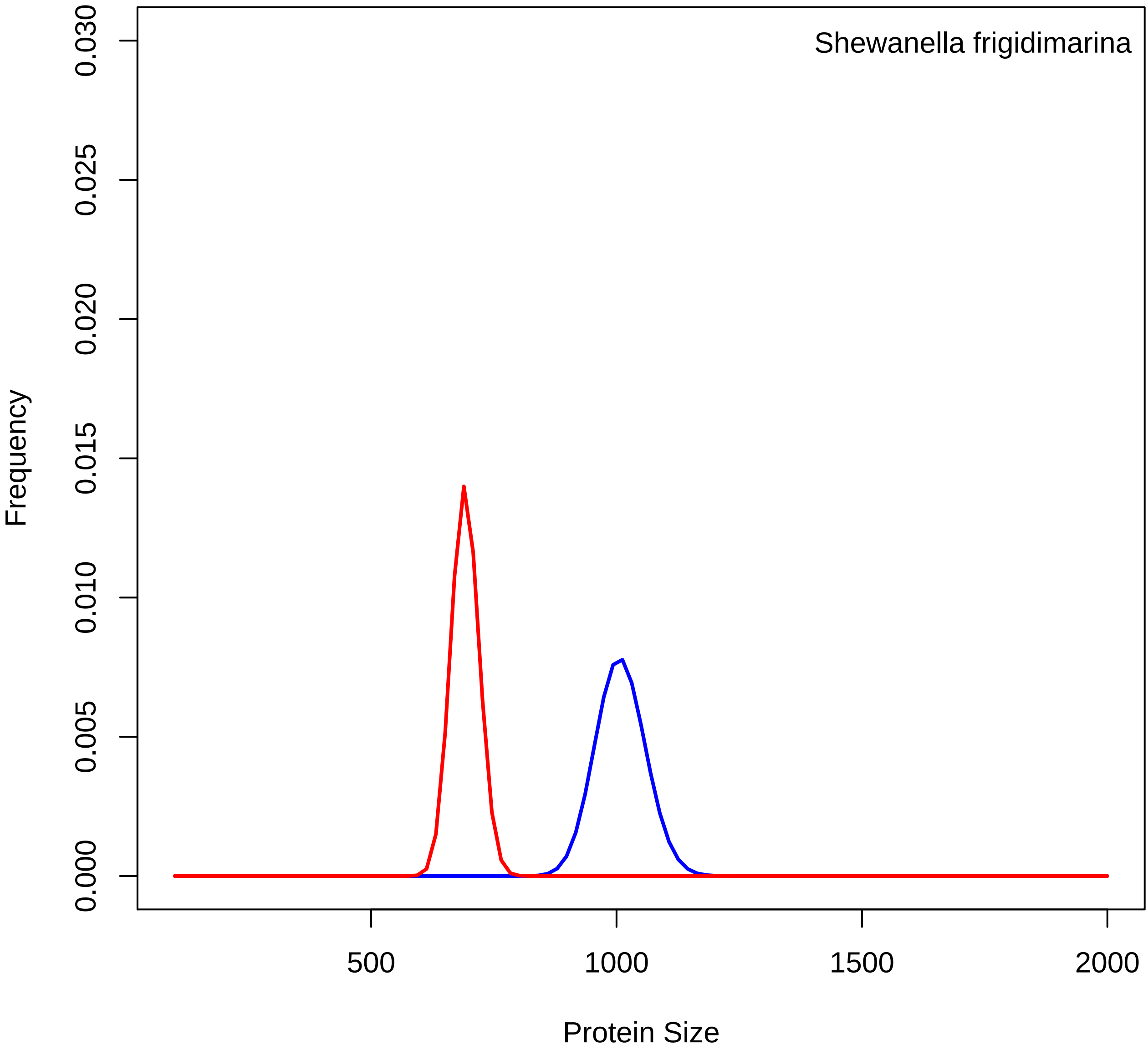

Supplement 4 − Figure 96

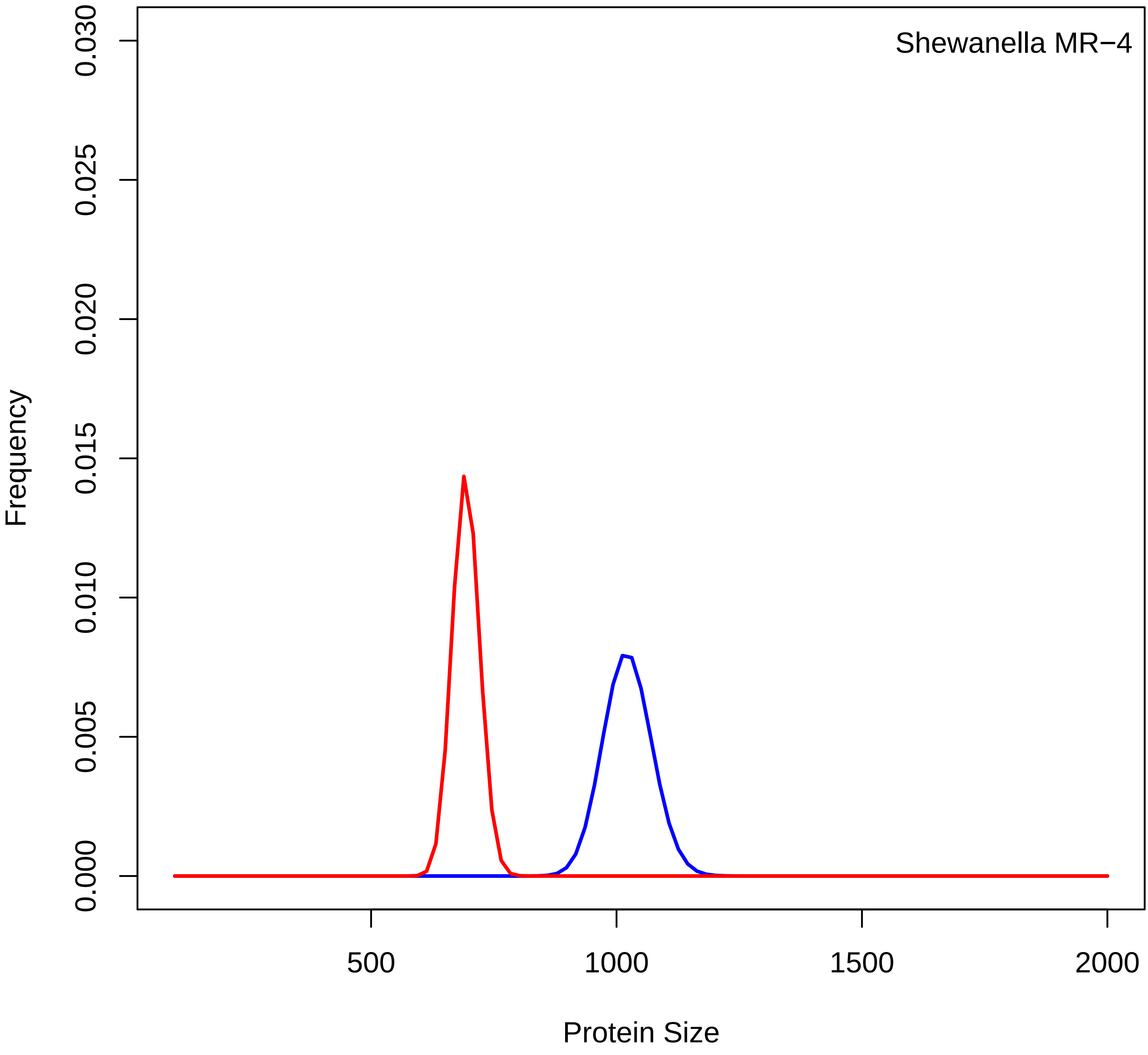

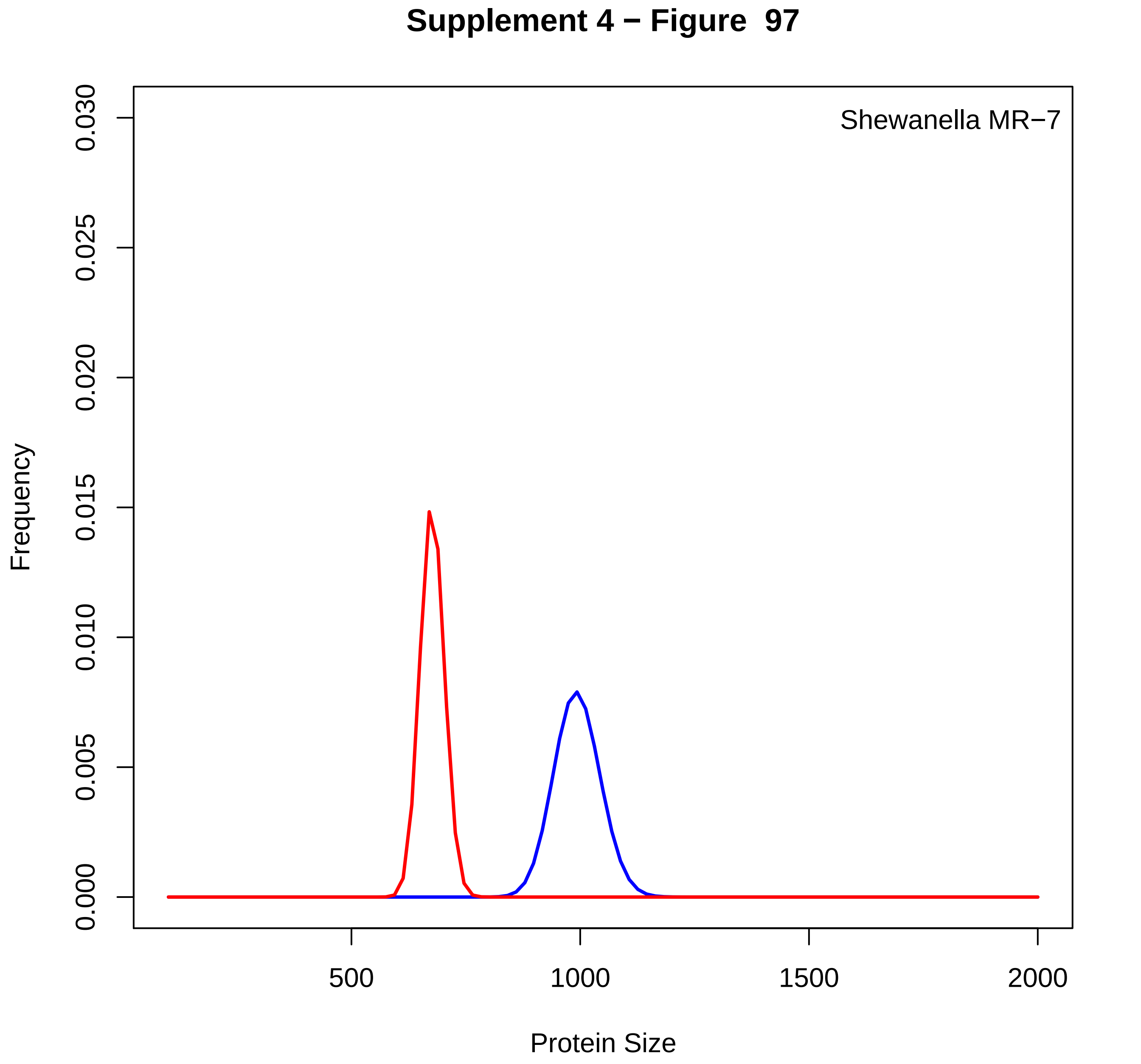
Supplement 4 − Figure 97
Shewanella MR−7
Frequency
0.000
0.005
0.010
0.015
0.020
0.025
0.030
500
1000
1500
2000
Protein Size

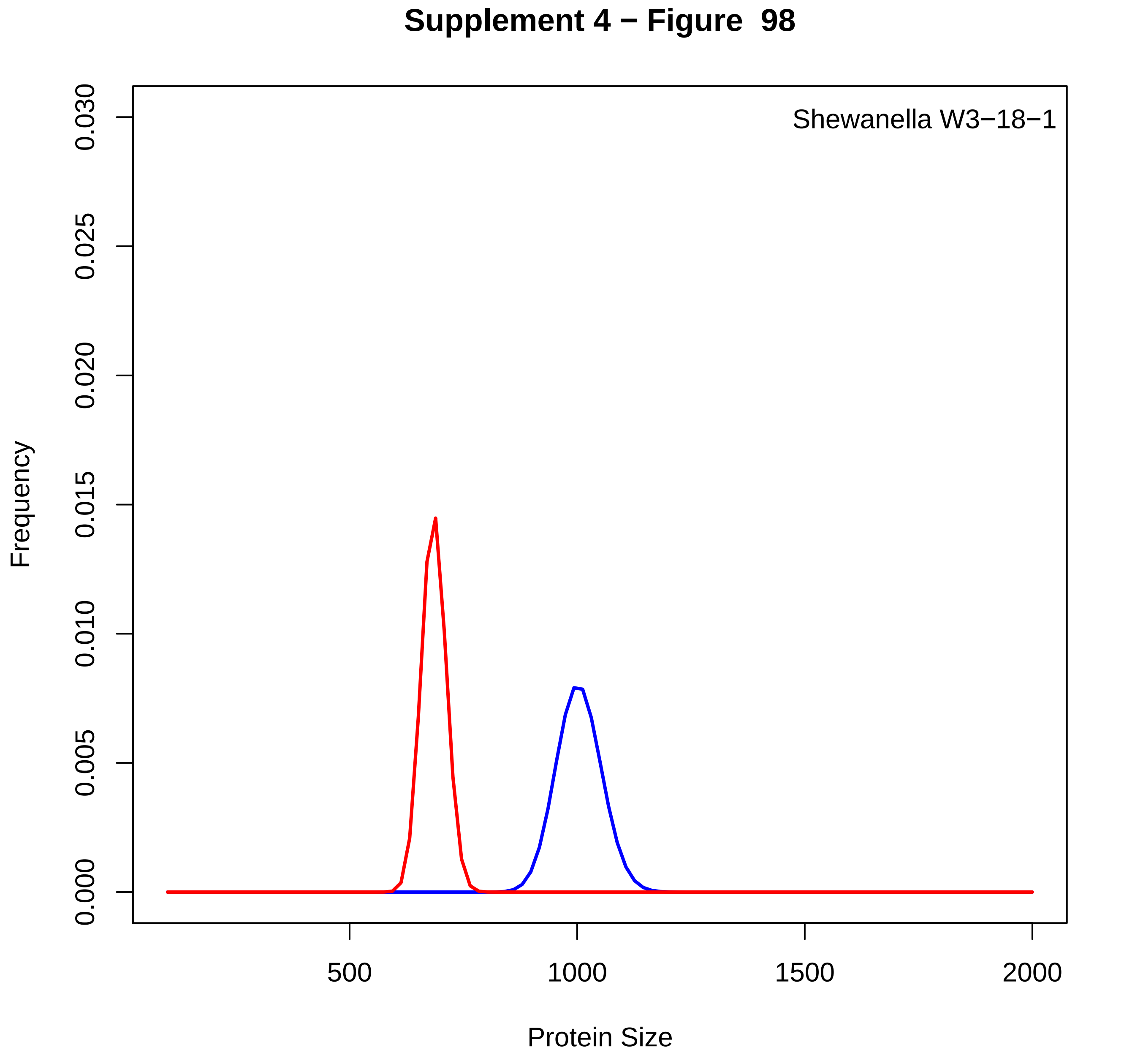
Supplement 4 − Figure 98
Shewanella W3−18−1
Frequency
0.000
0.005
0.010
0.015
0.020
0.025
0.030
500
1000
1500
2000
Protein Size

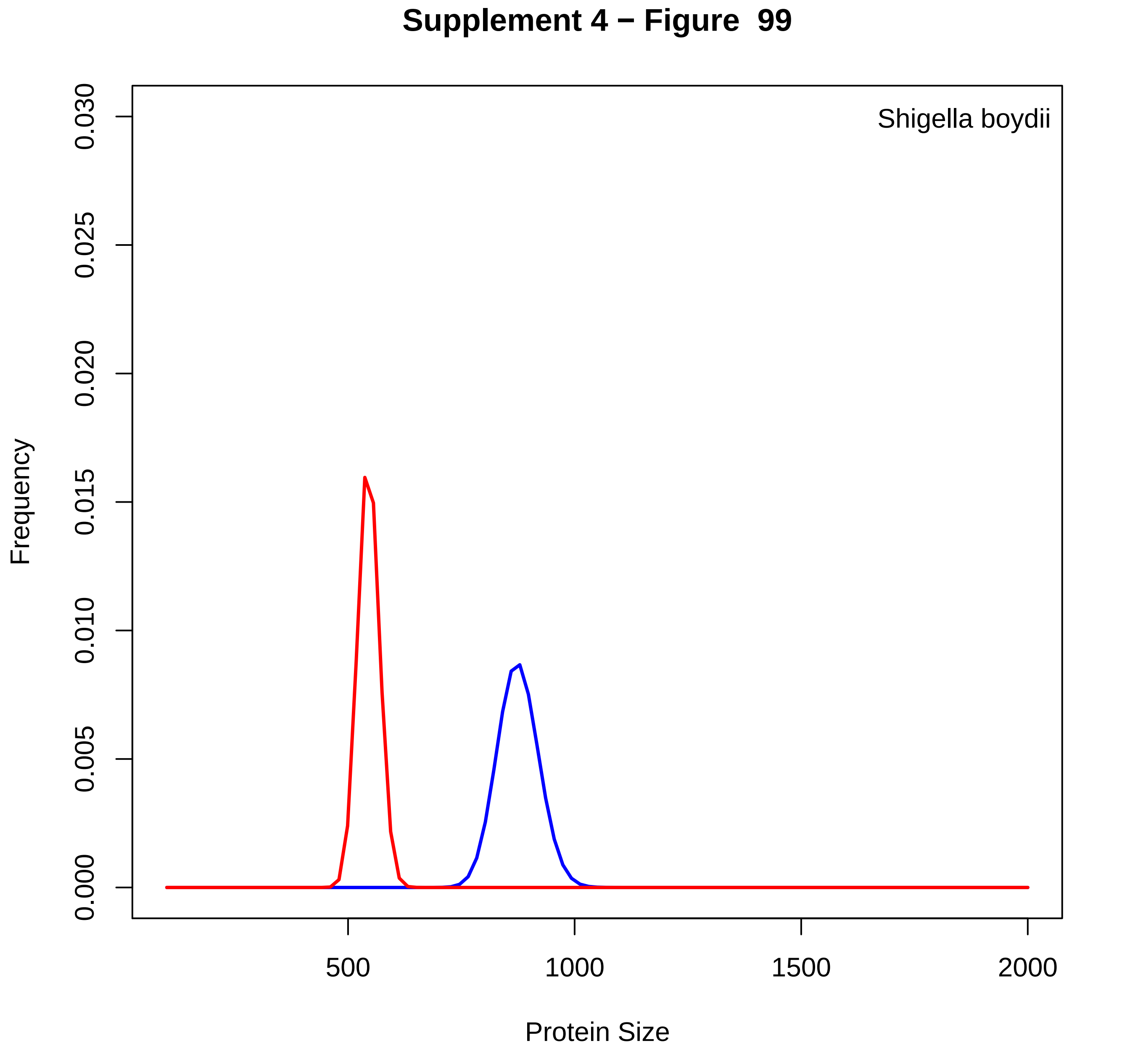
Supplement 4 − Figure 99
Shigella boydii
Frequency
0.000
0.005
0.010
0.015
0.020
0.025
0.030
500
1000
1500
2000
Protein Size

Supplement 4 − Figure 100

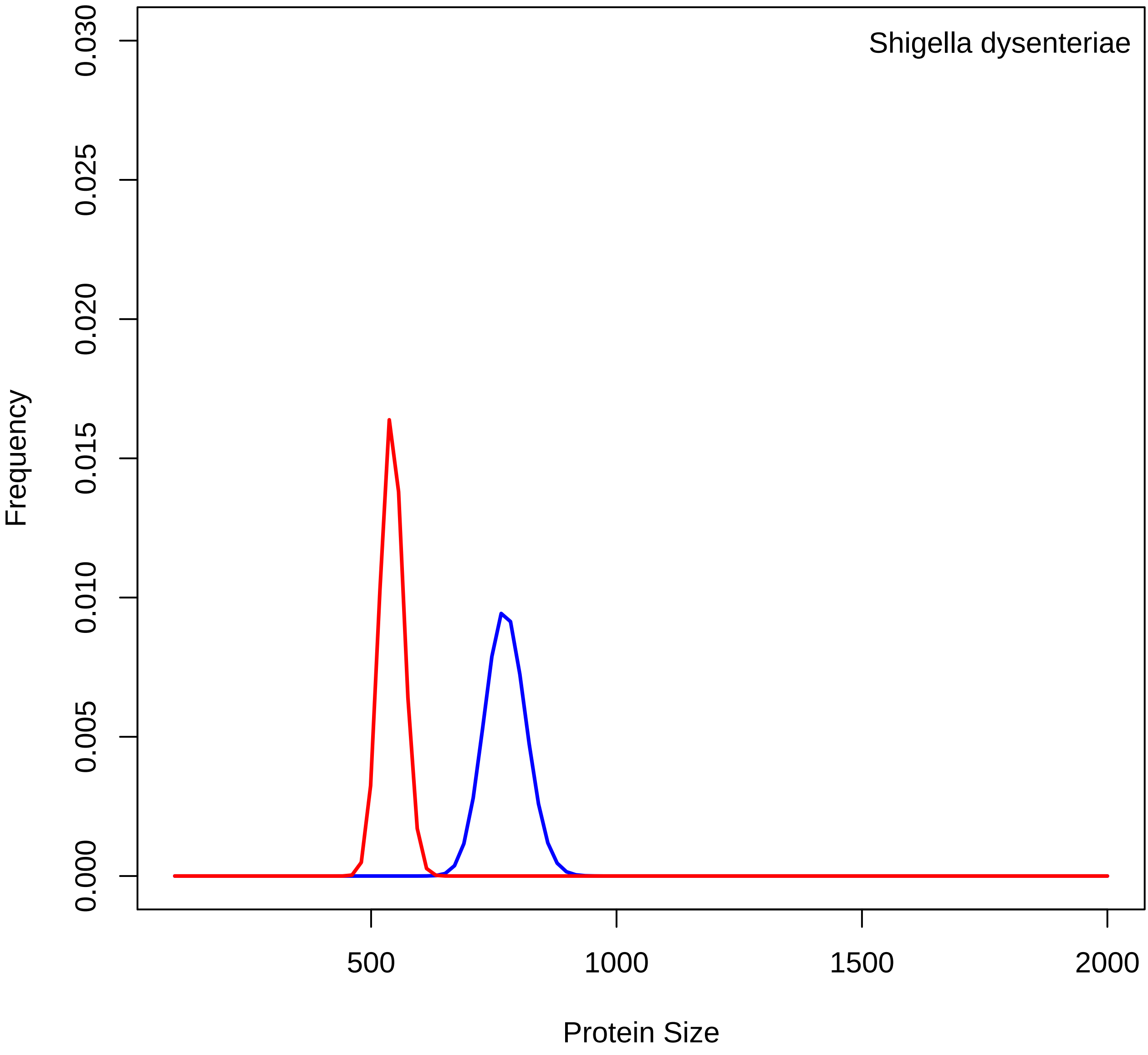

Supplement 4 − Figure 101

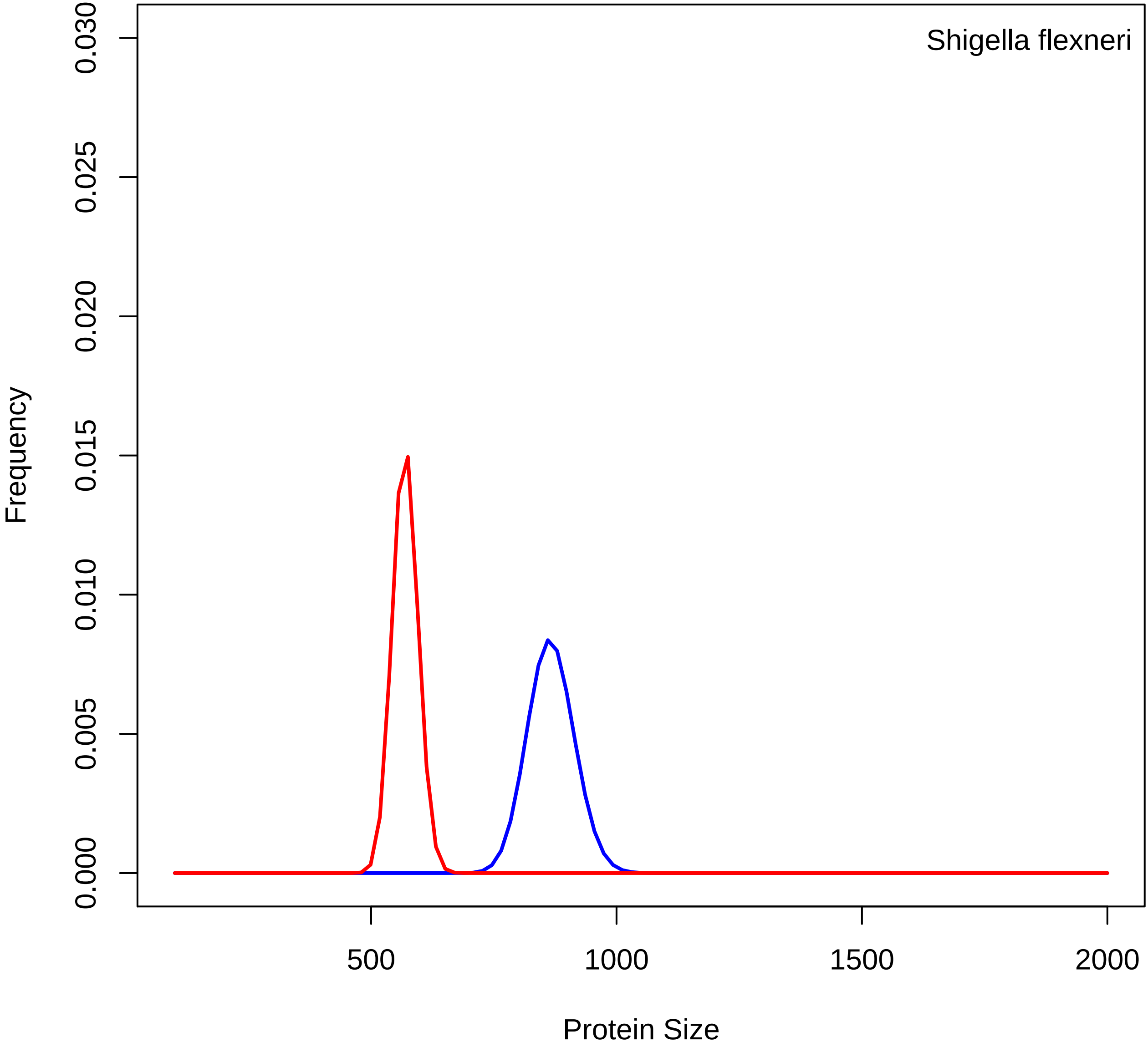

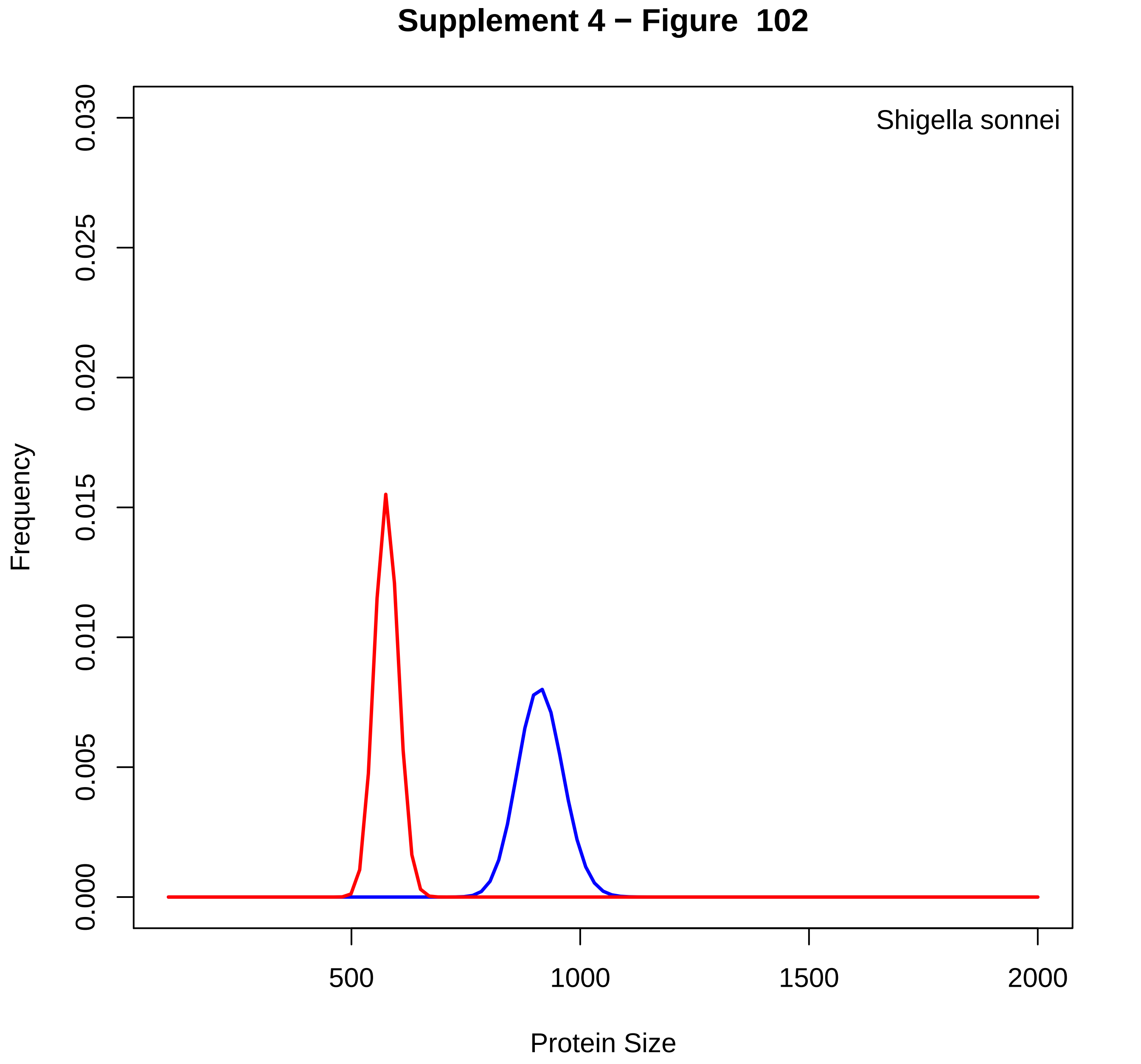
Supplement 4 − Figure 102
Shigella sonnei
Frequency
0.000
0.005
0.010
0.015
0.020
0.025
0.030
500
1000
1500
2000
Protein Size

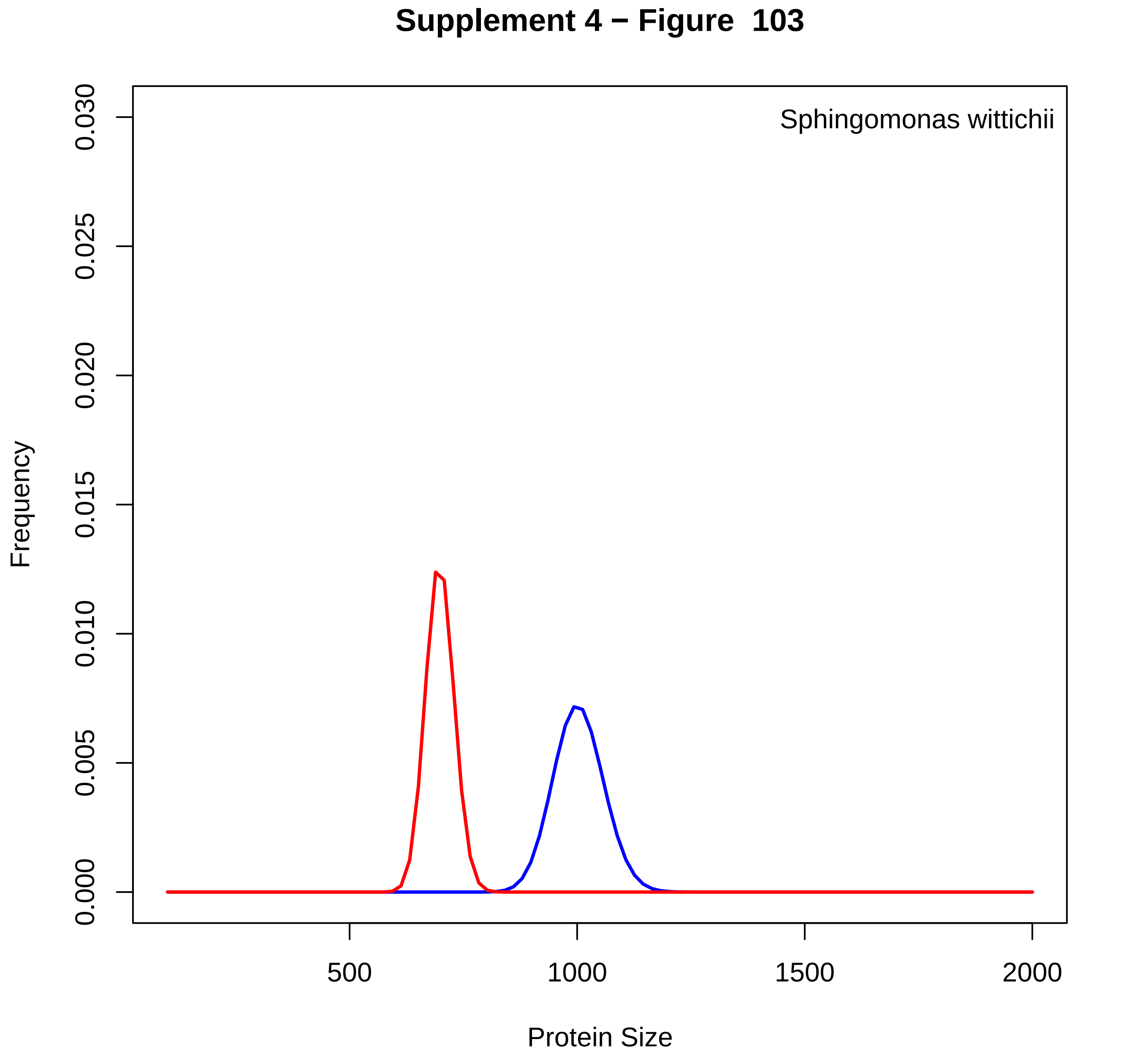
Supplement 4 − Figure 103
Sphingomonas wittichii
Frequency
0.000
0.005
0.010
0.015
0.020
0.025
0.030
500
1000
1500
2000
Protein Size

Supplement 4 − Figure 104

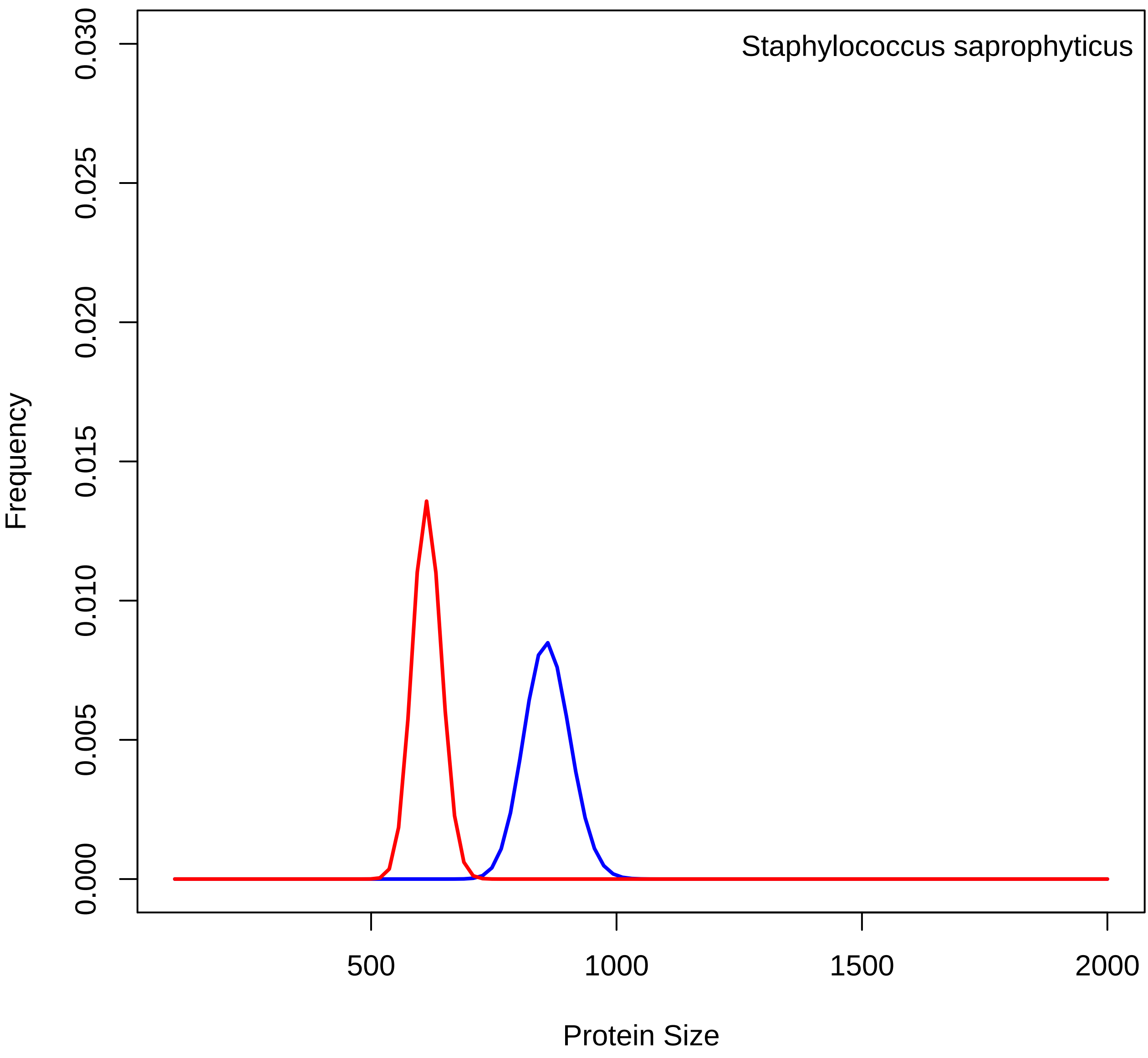

Supplement 4 − Figure 105

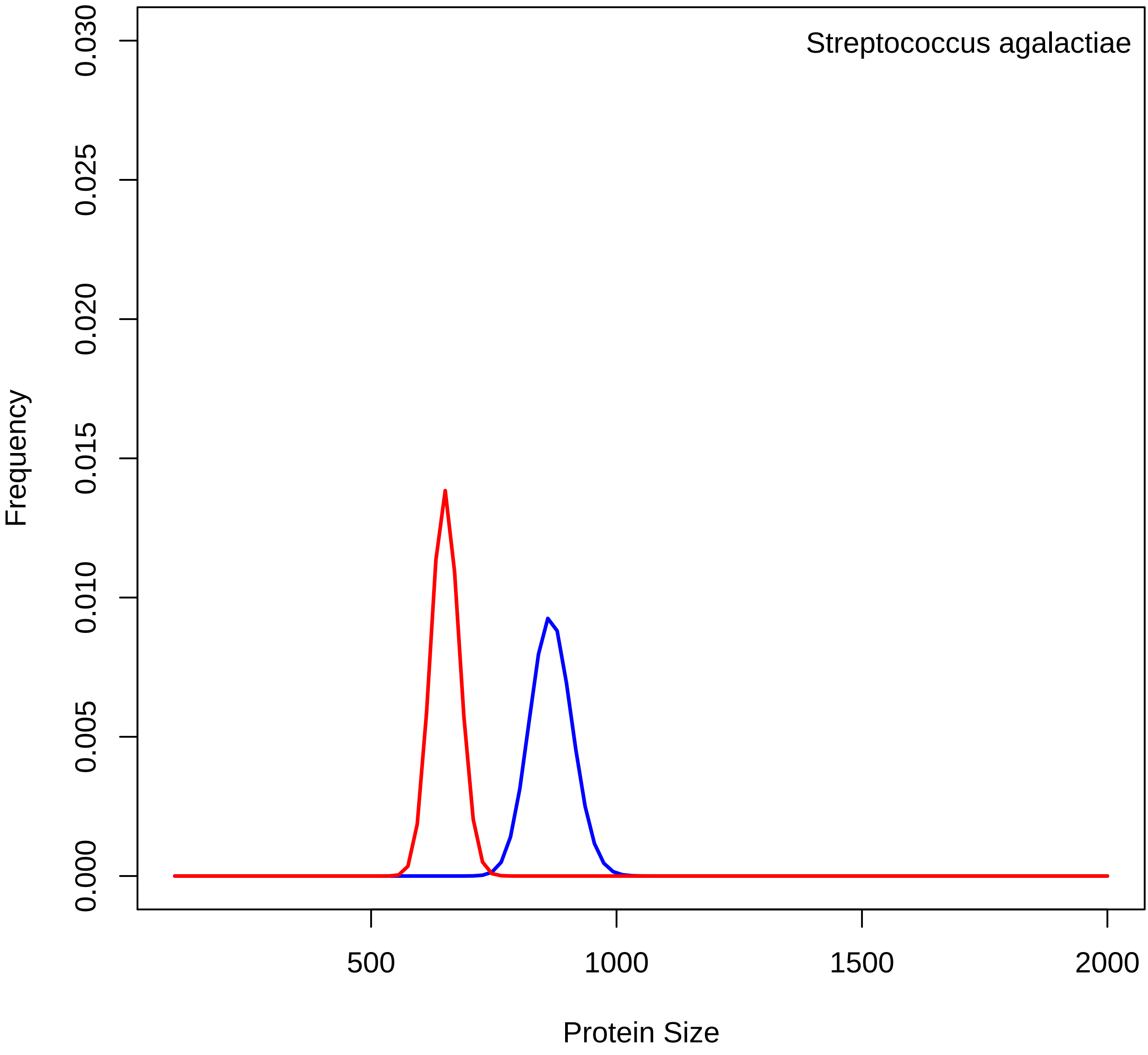

Supplement 4 − Figure 106

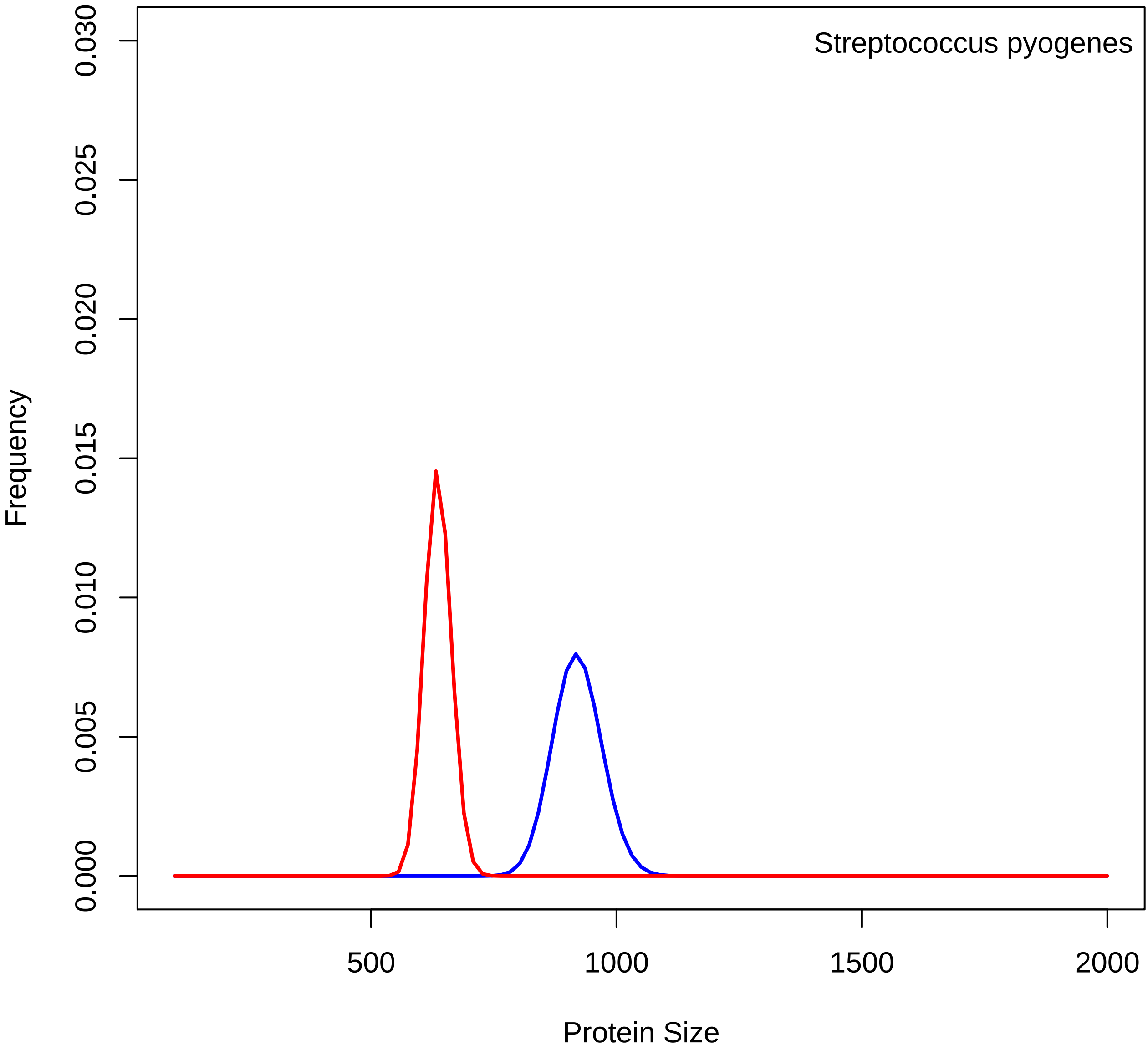

Supplement 4 − Figure 107

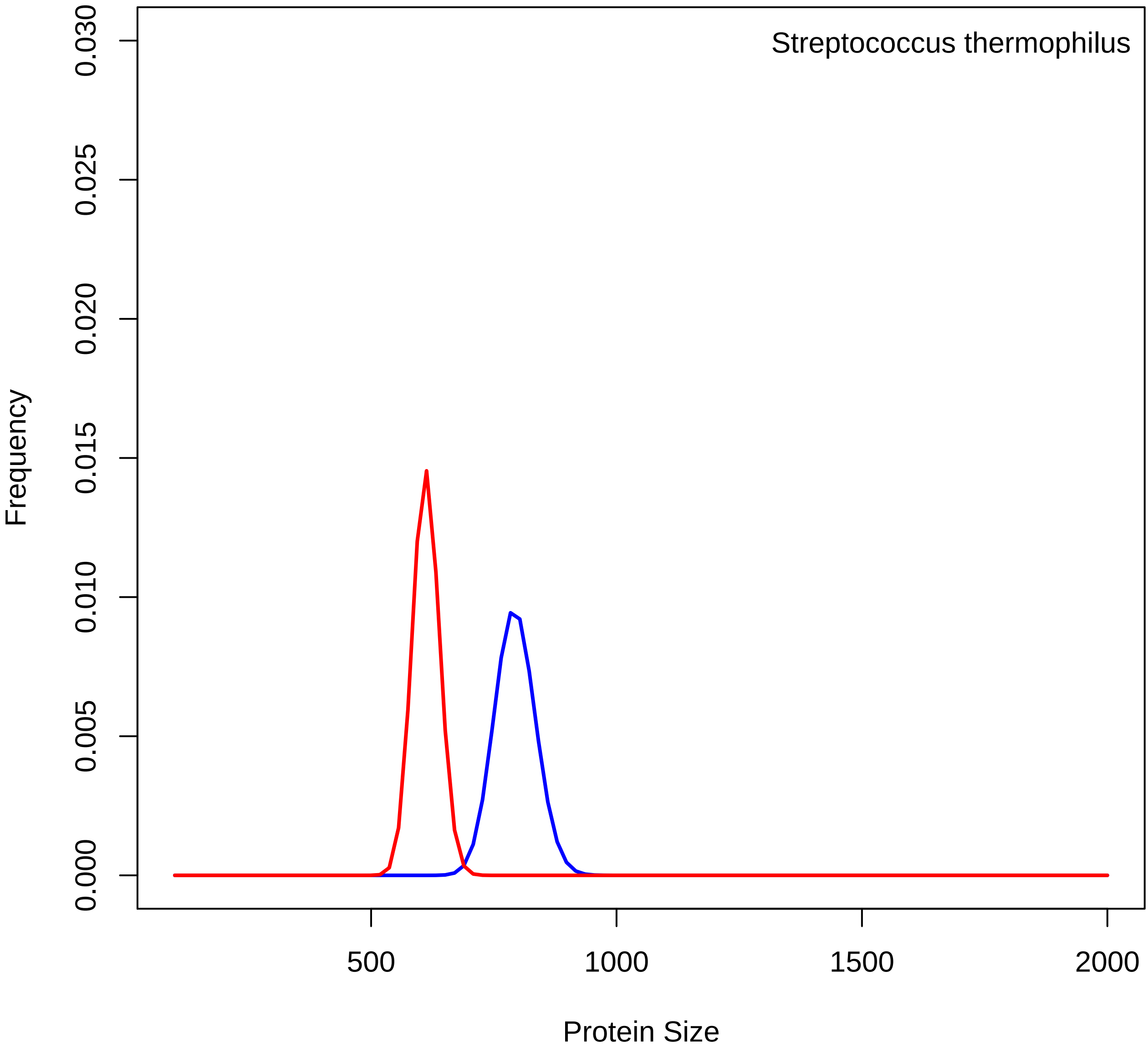

Supplement 4 − Figure 108

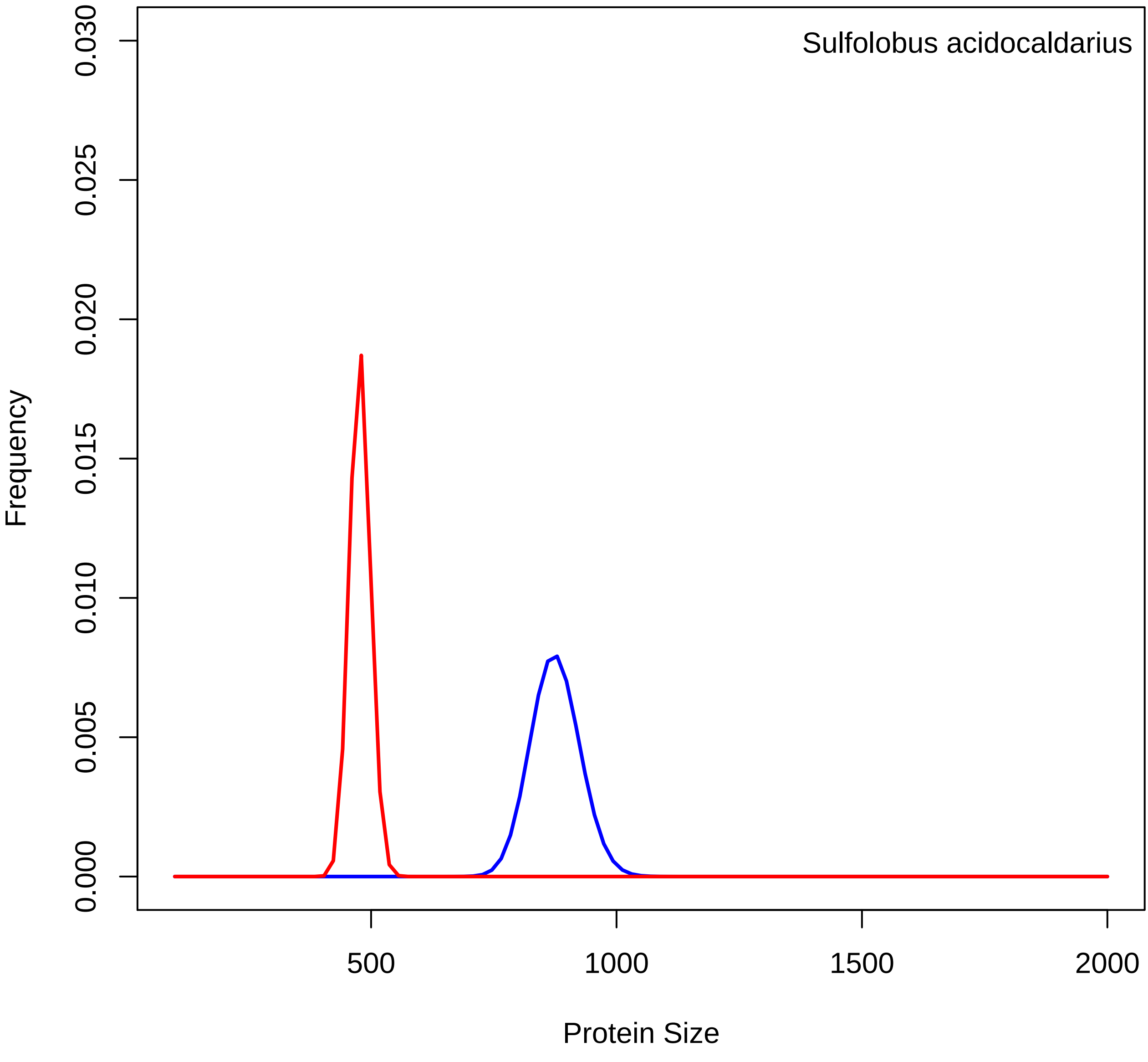

Supplement 4 − Figure 109

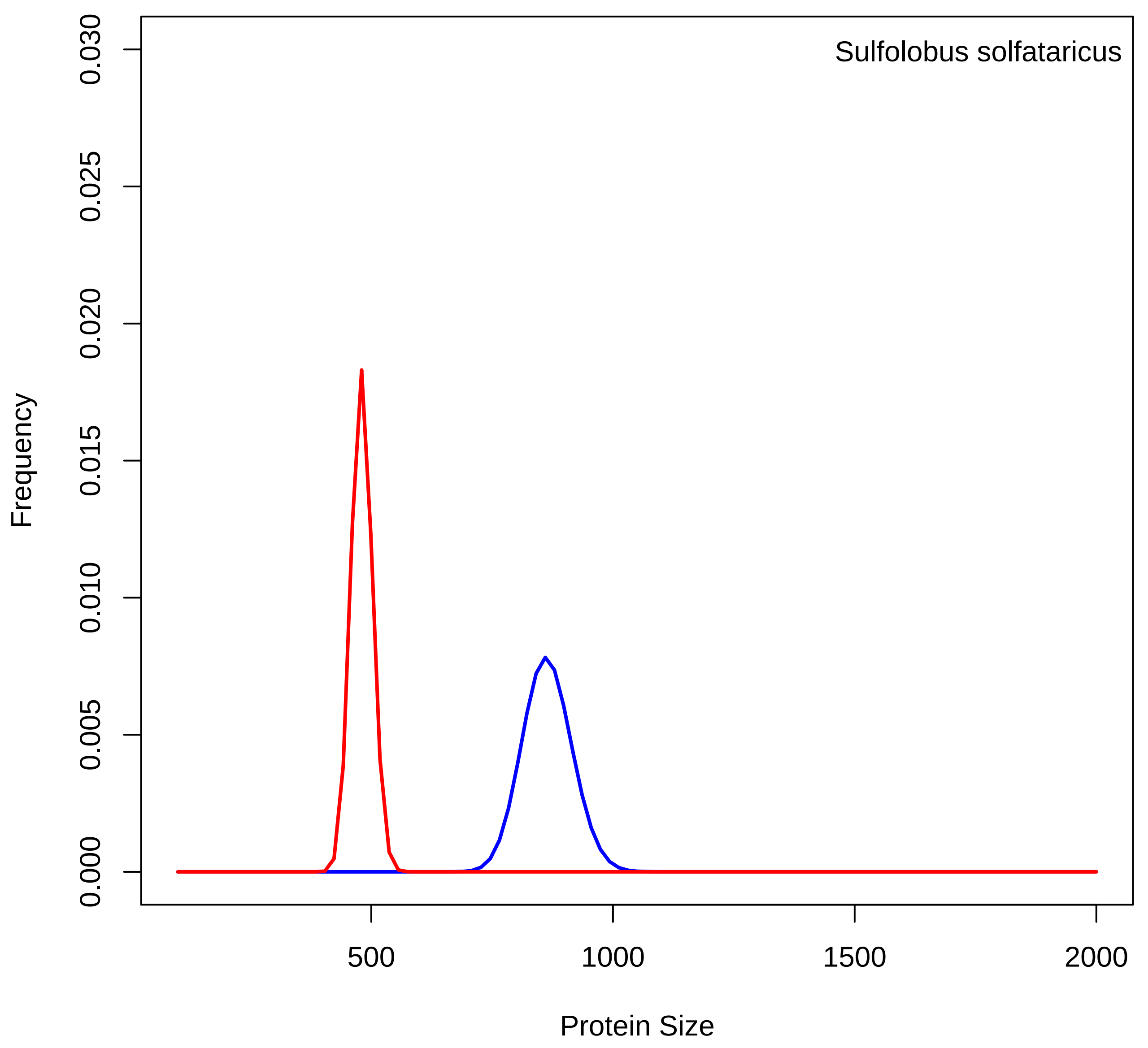

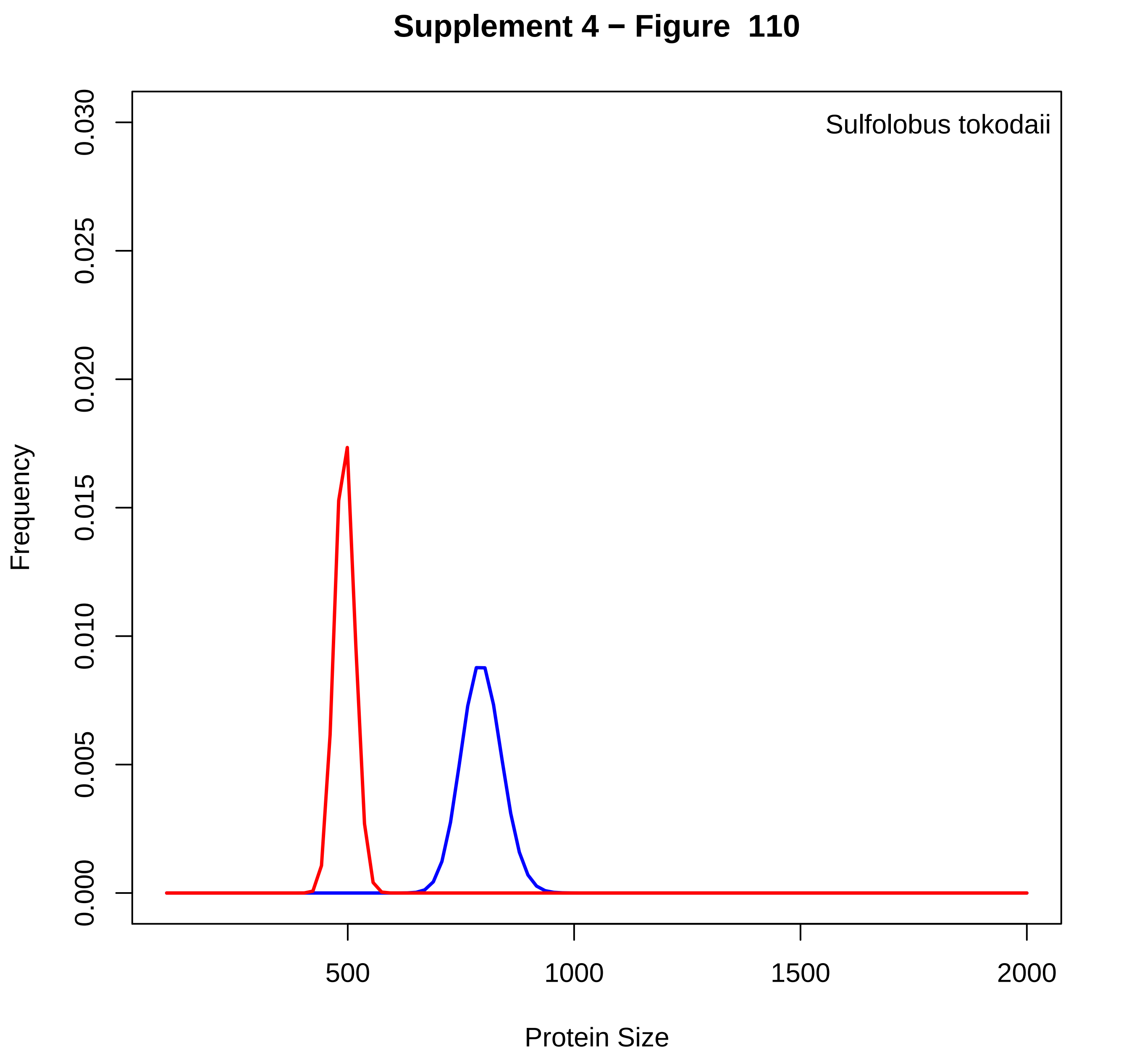
Supplement 4 − Figure 110
Sulfolobus tokodaii
Frequency
0.000
0.005
0.010
0.015
0.020
0.025
0.030
500
1000
1500
2000
Protein Size

Supplement 4 − Figure 111

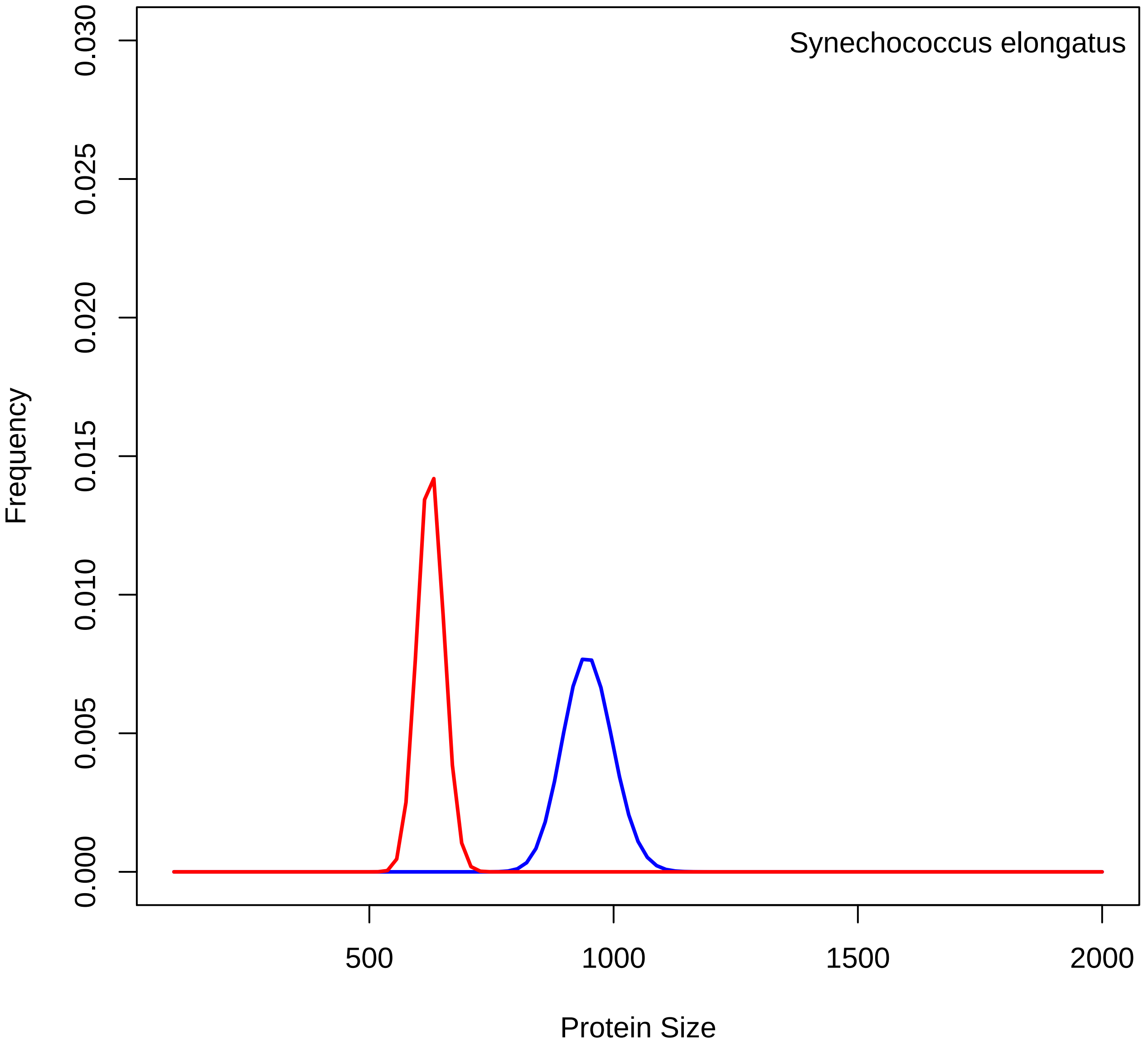

Supplement 4 − Figure 112

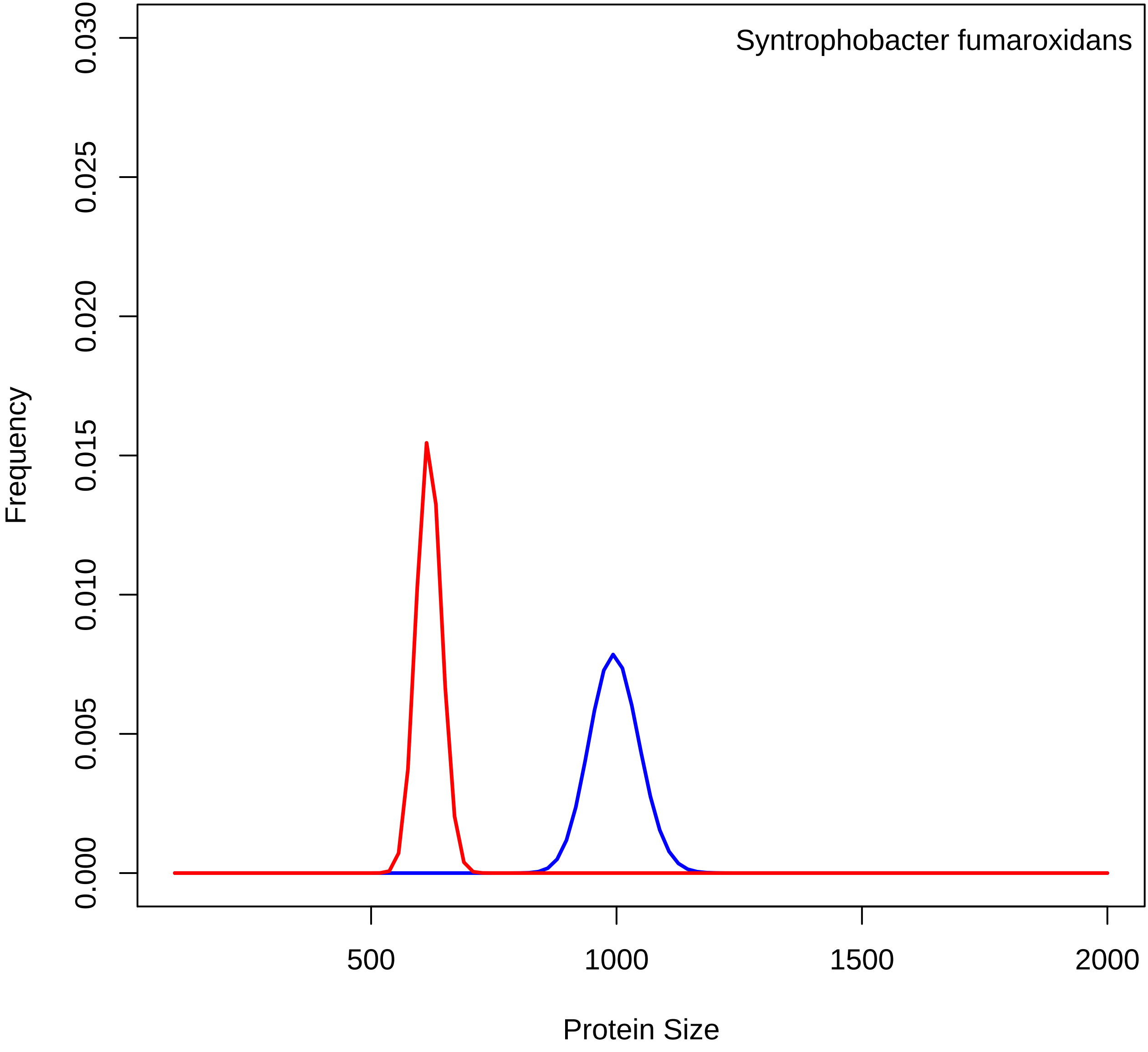

Supplement 4 − Figure 113

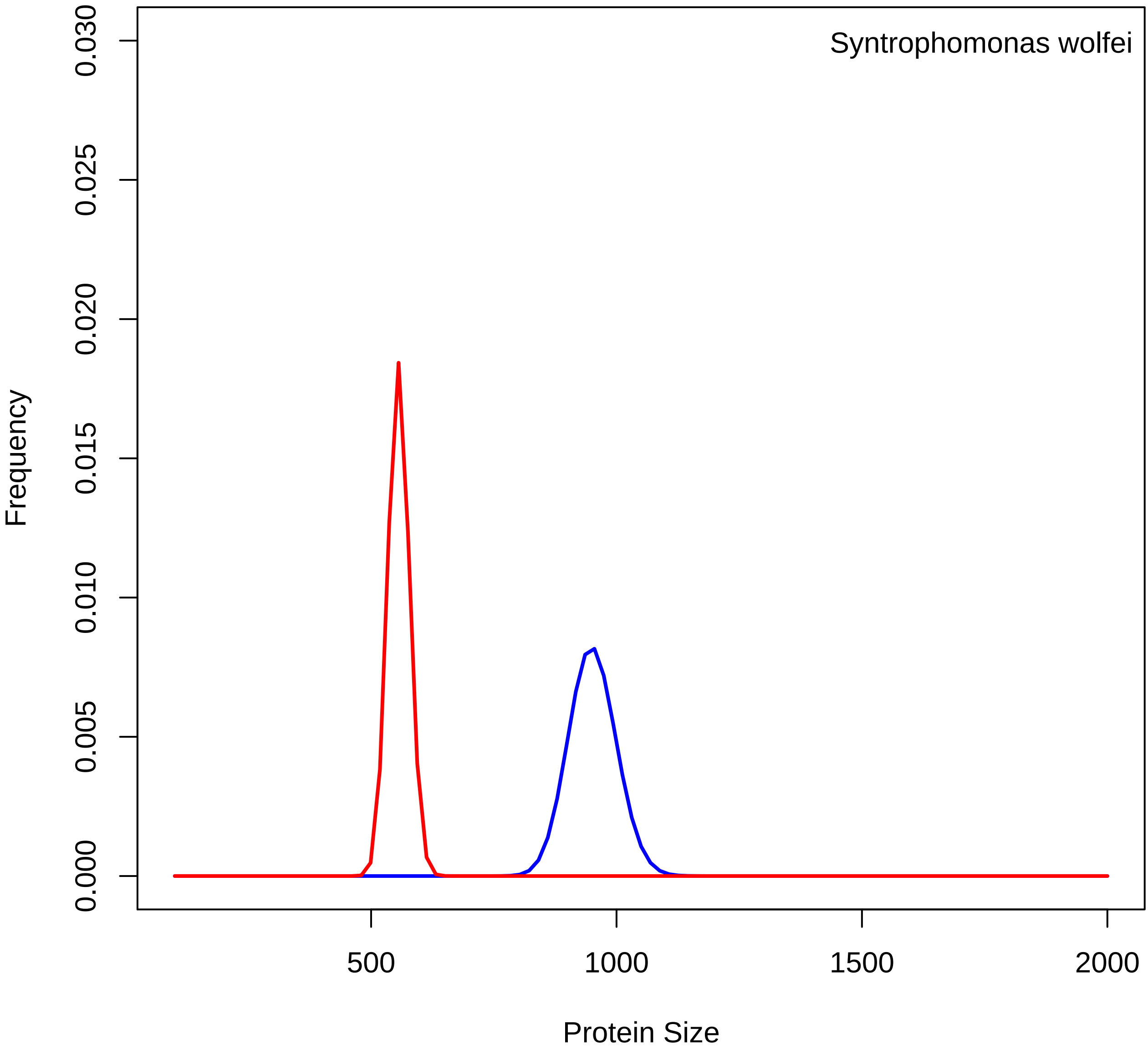

Supplement 4 − Figure 114

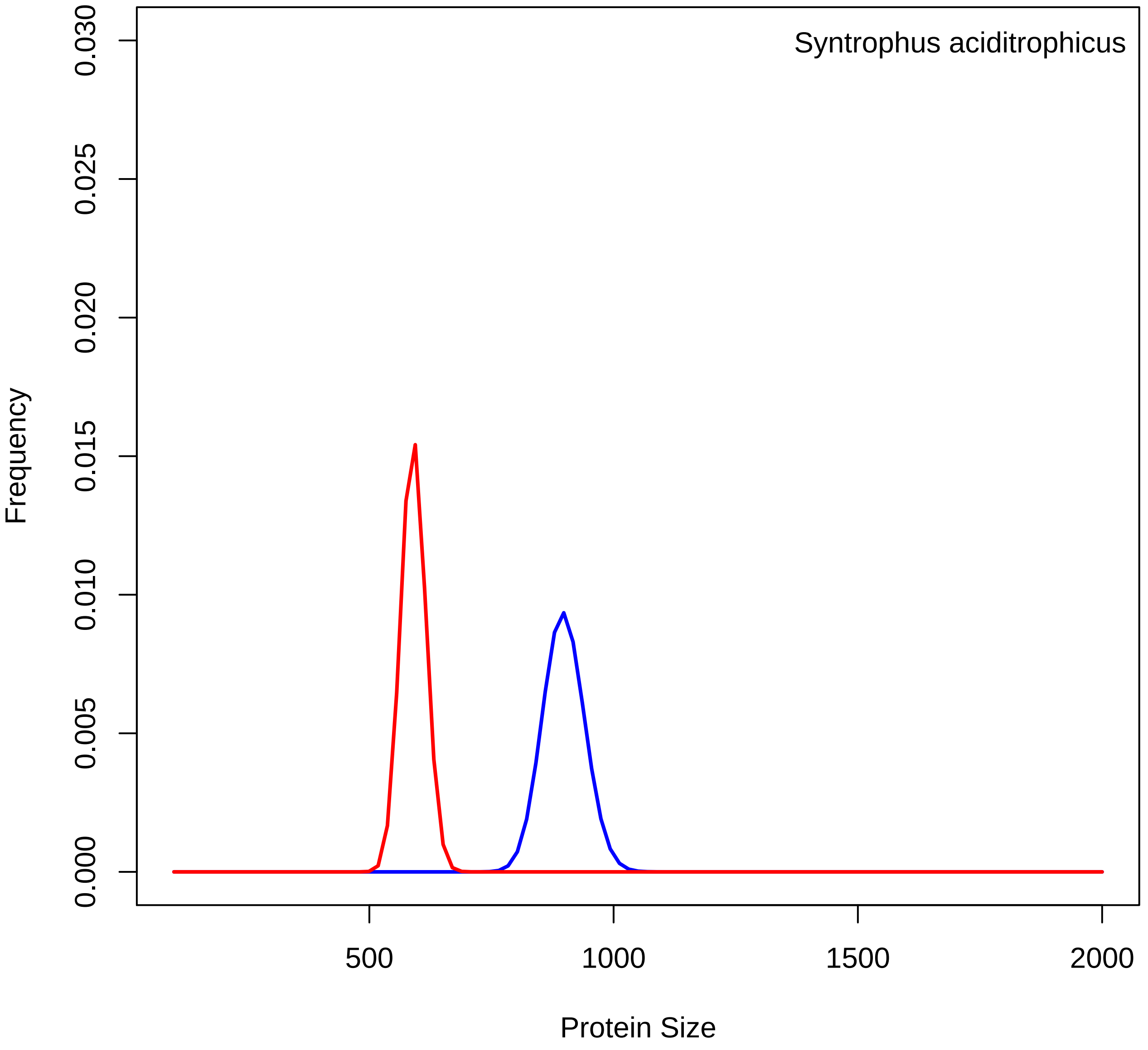

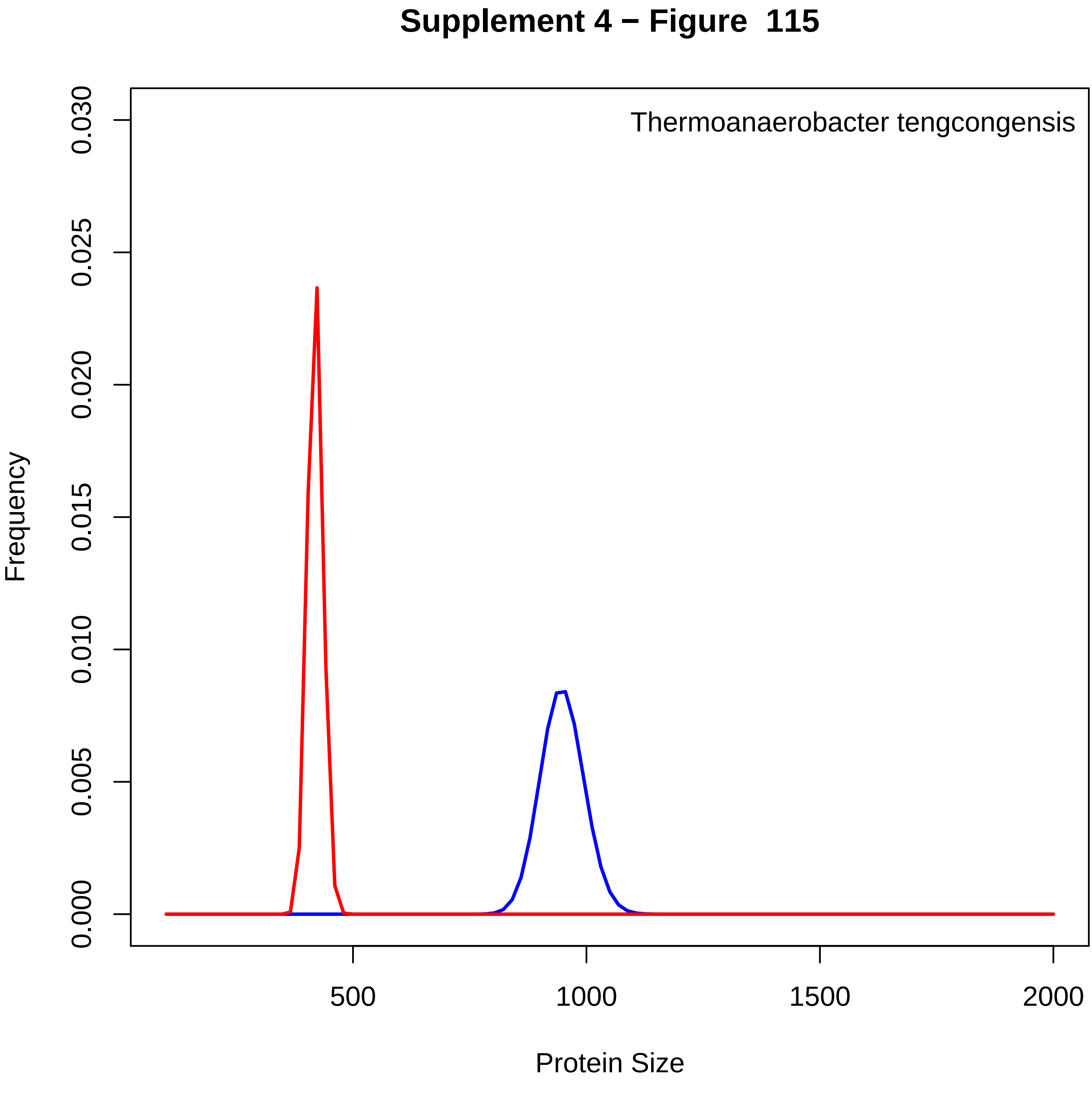

Supplement 4 − Figure 115
Thermoanaerobacter tengcongensis
Frequency
0.000
0.005
0.010
0.015
0.020
0.025
0.030
500
1000
1500
2000
Protein Size

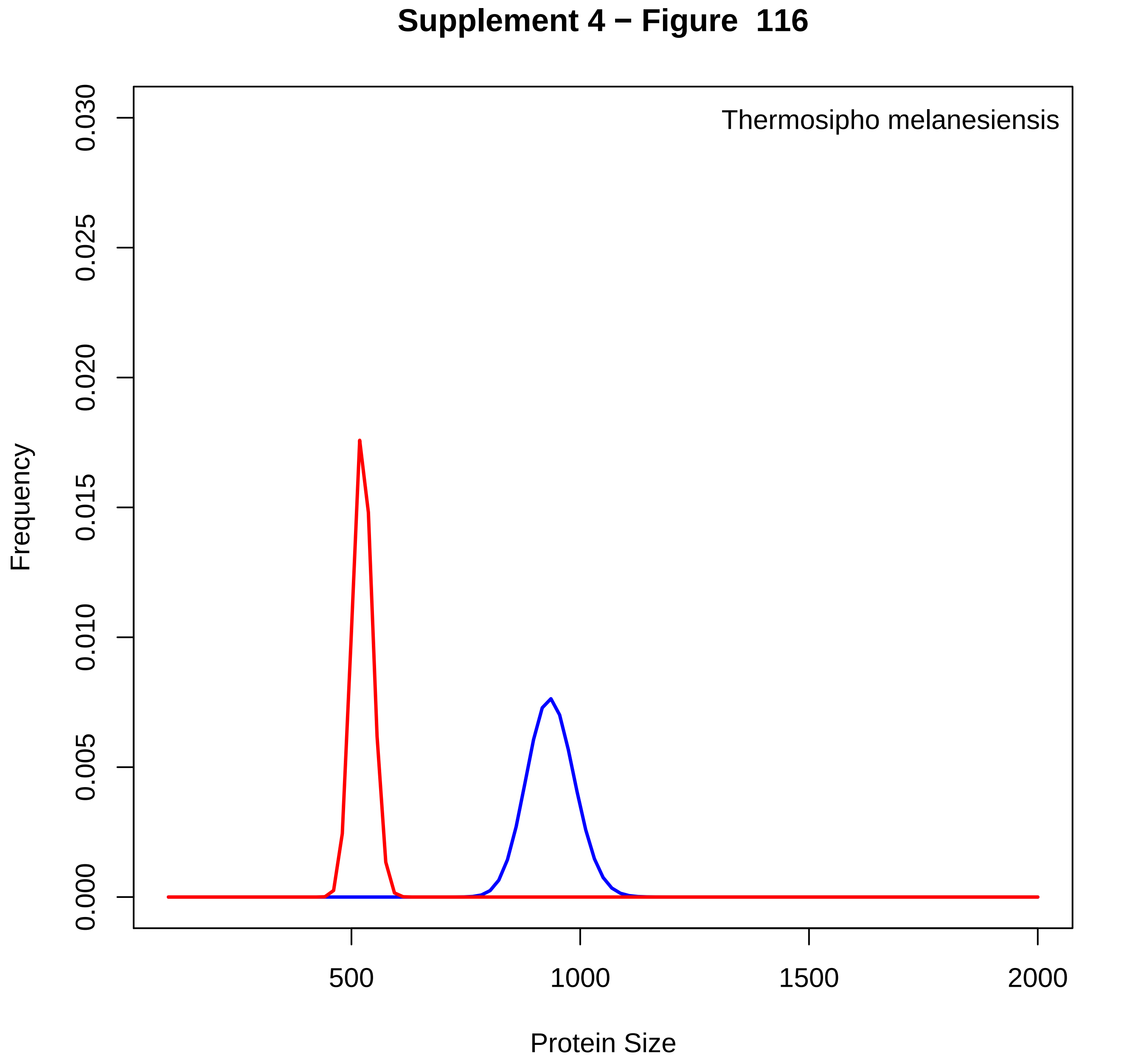
Supplement 4 − Figure 116
Thermosipho melanesiensis
Frequency
0.000
0.005
0.010
0.015
0.020
0.025
0.030
500
1000
1500
2000
Protein Size

Supplement 4 − Figure 117

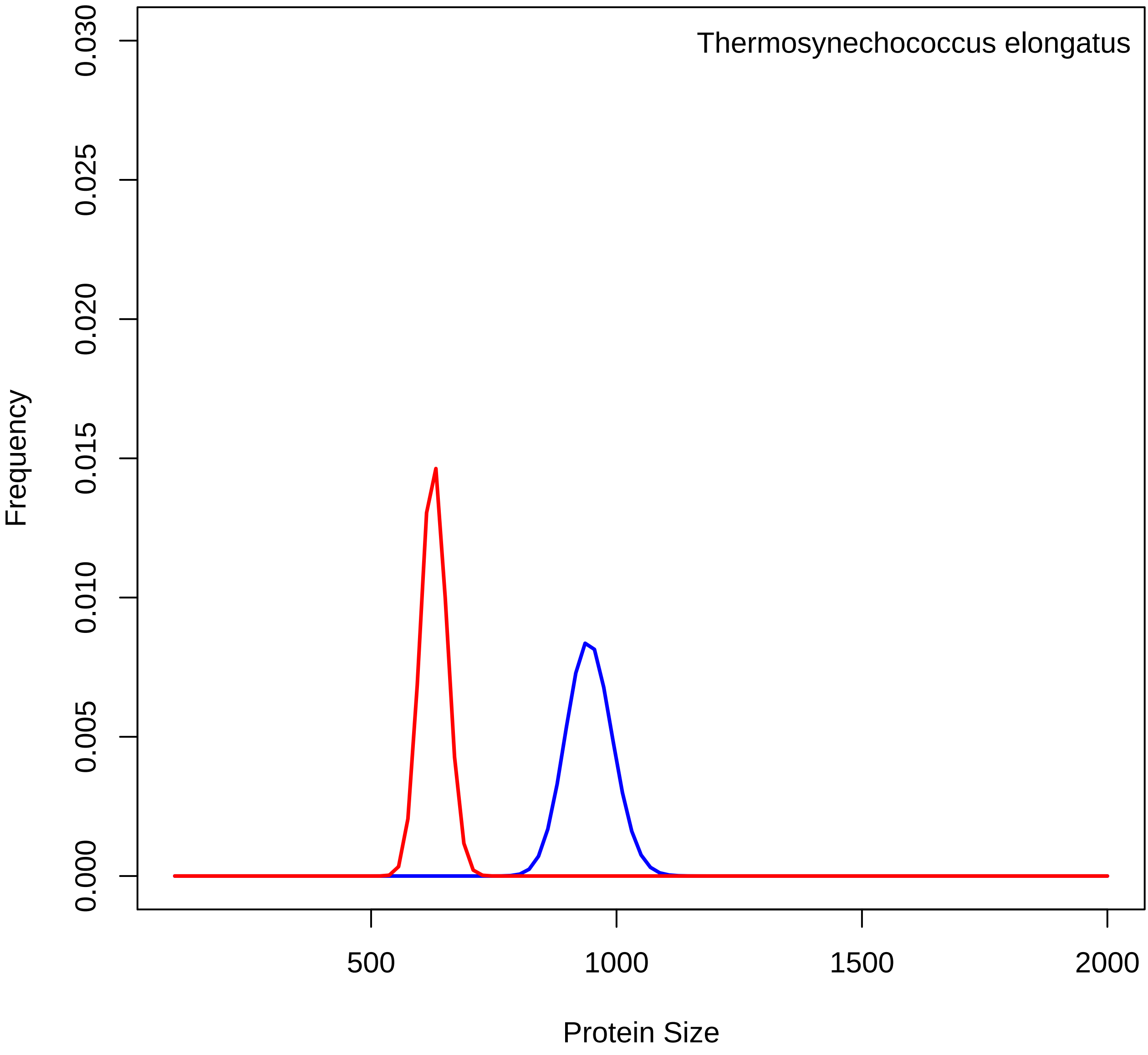

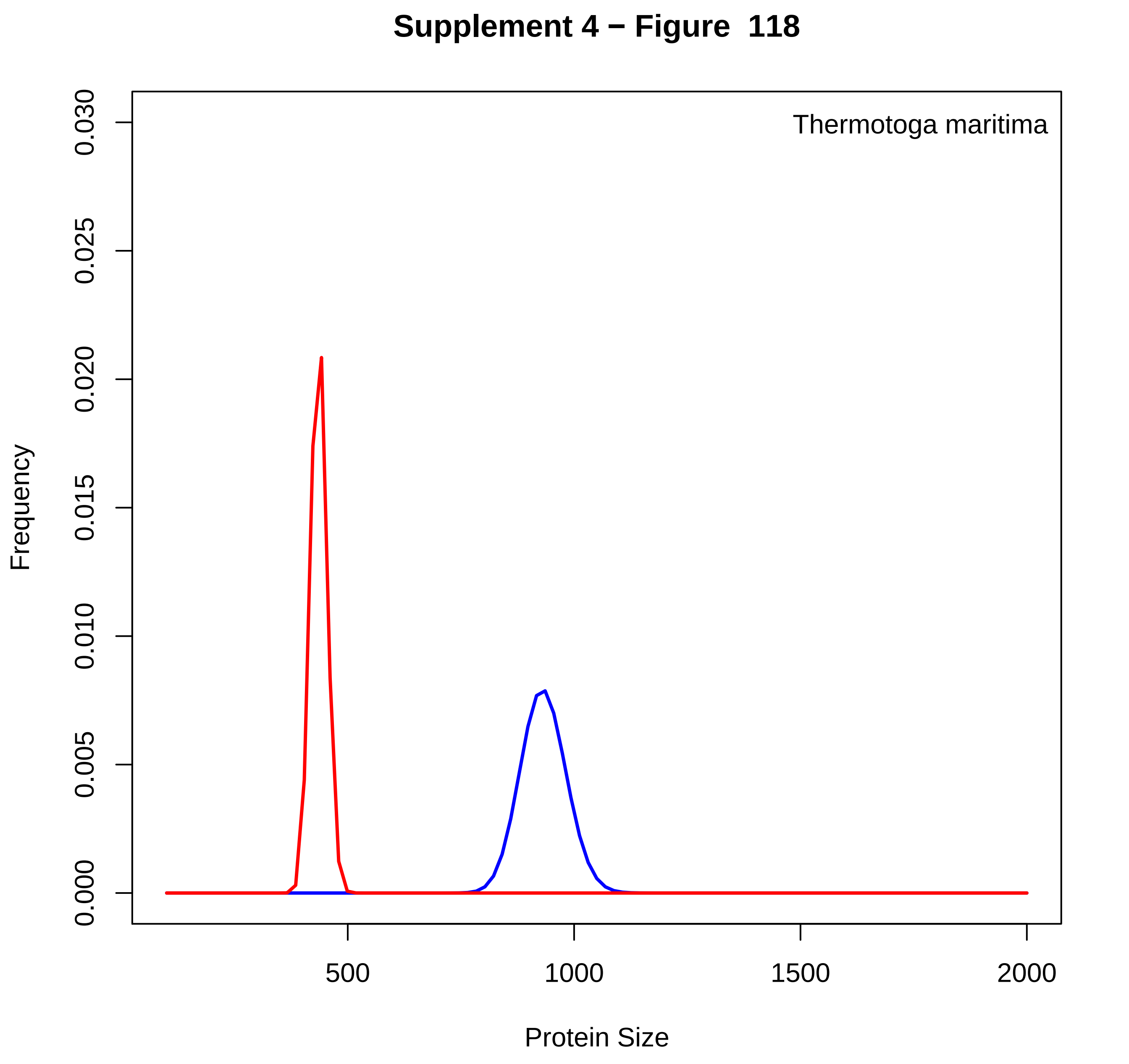

Supplement 4 − Figure 118
Thermotoga maritima
Frequency
0.000
0.005
0.010
0.015
0.020
0.025
0.030
500
1000
1500
2000
Protein Size

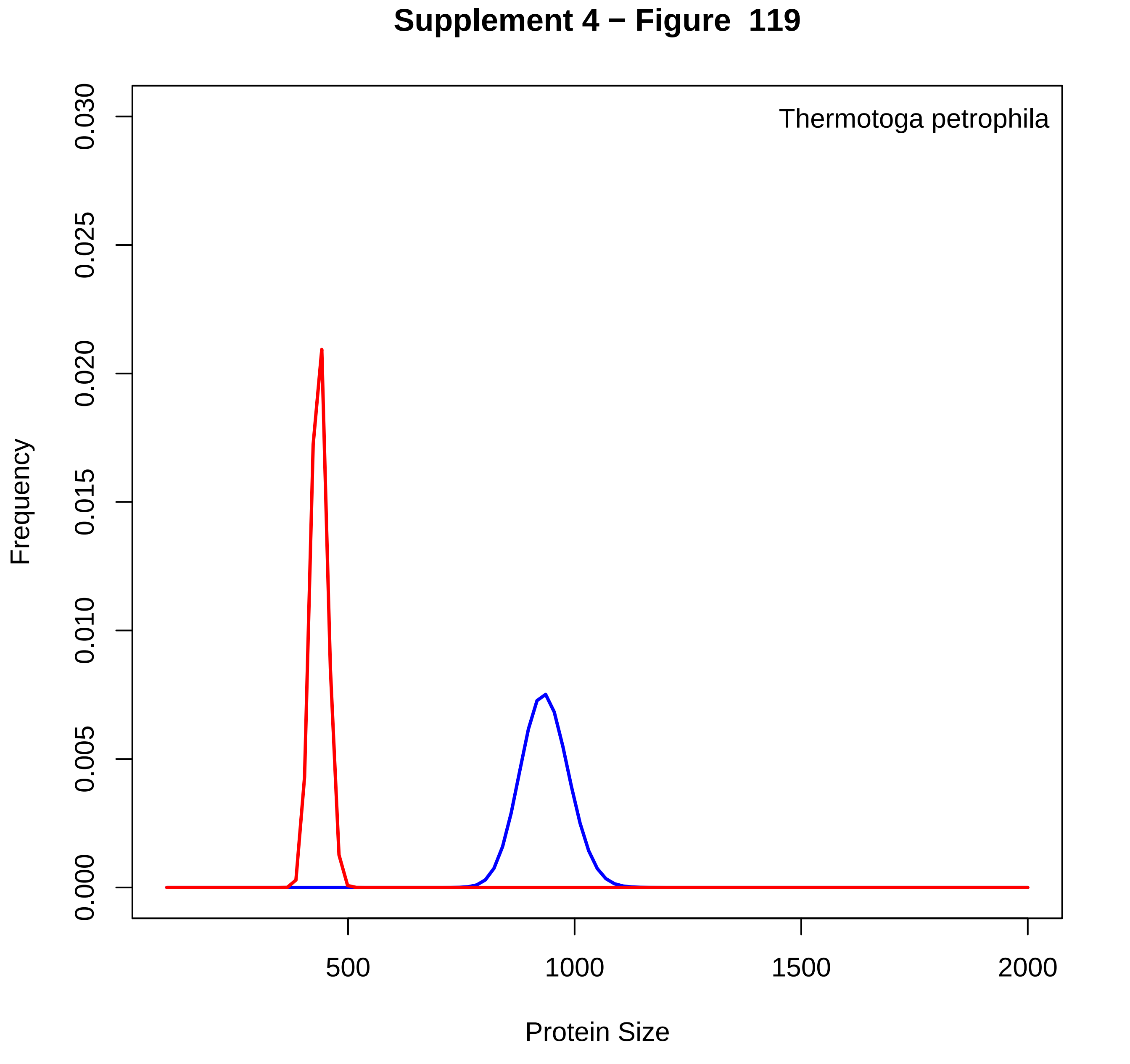
Supplement 4 − Figure 119
Thermotoga petrophila
Frequency
0.000
0.005
0.010
0.015
0.020
0.025
0.030
500
1000
1500
2000
Protein Size

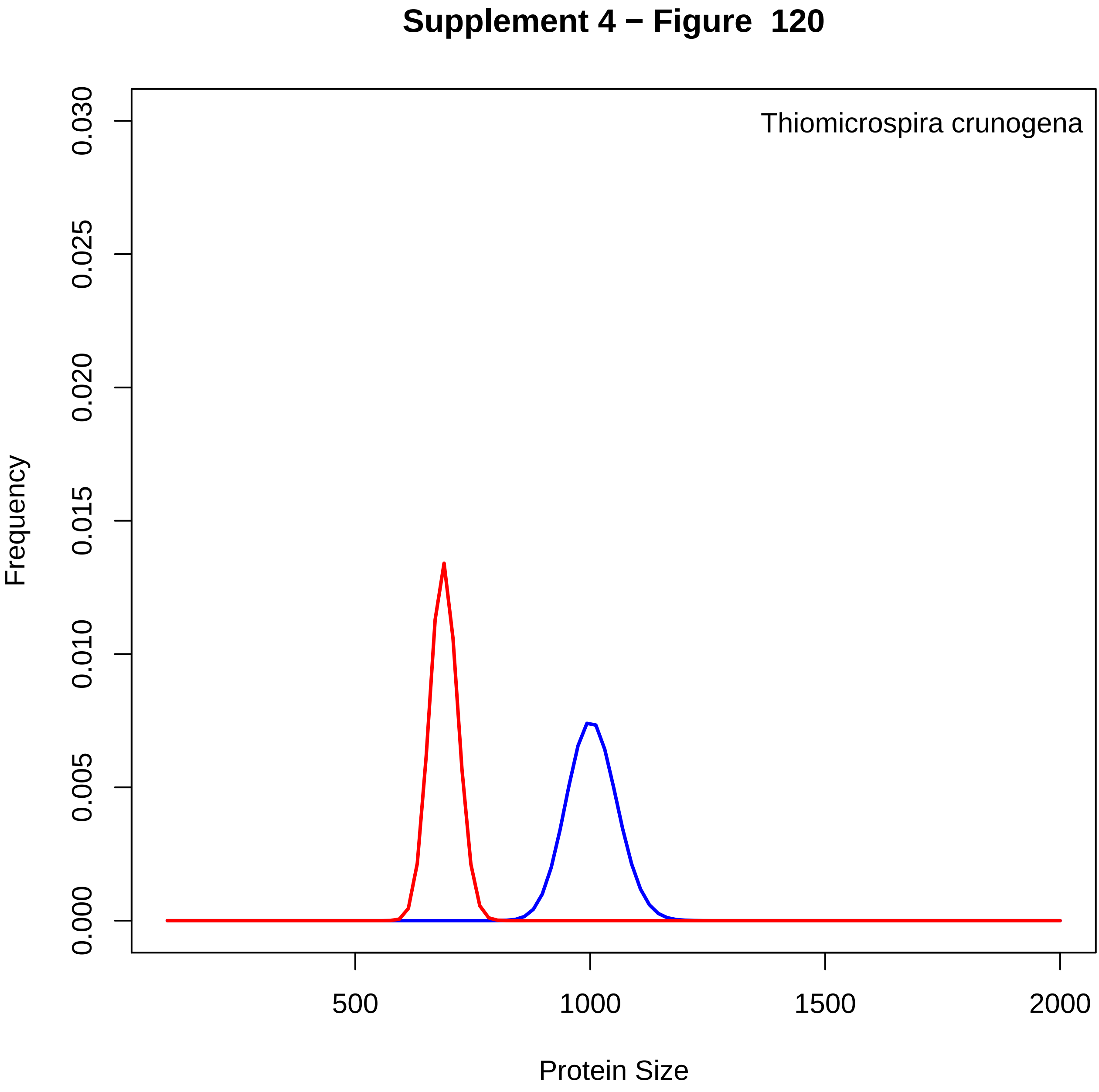

Supplement 4 − Figure 120
Thiomicrospira crunogena
Frequency
0.000
0.005
0.010
0.015
0.020
0.025
0.030
500
1000
1500
2000
Protein Size

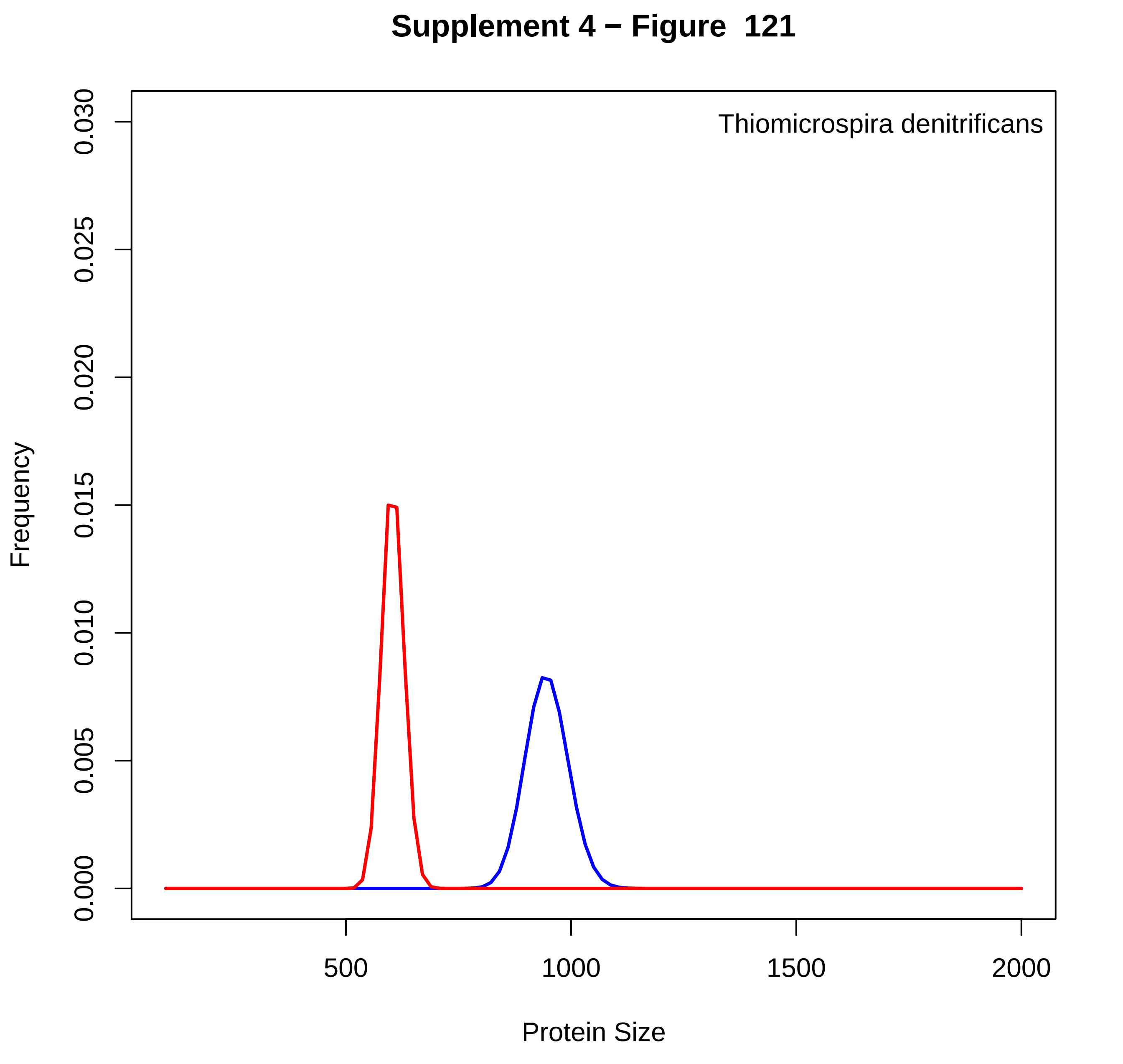

Supplement 4 − Figure 121
Thiomicrospira denitrificans
Frequency
0.000
0.005
0.010
0.015
0.020
0.025
0.030
500
1000
1500
2000
Protein Size

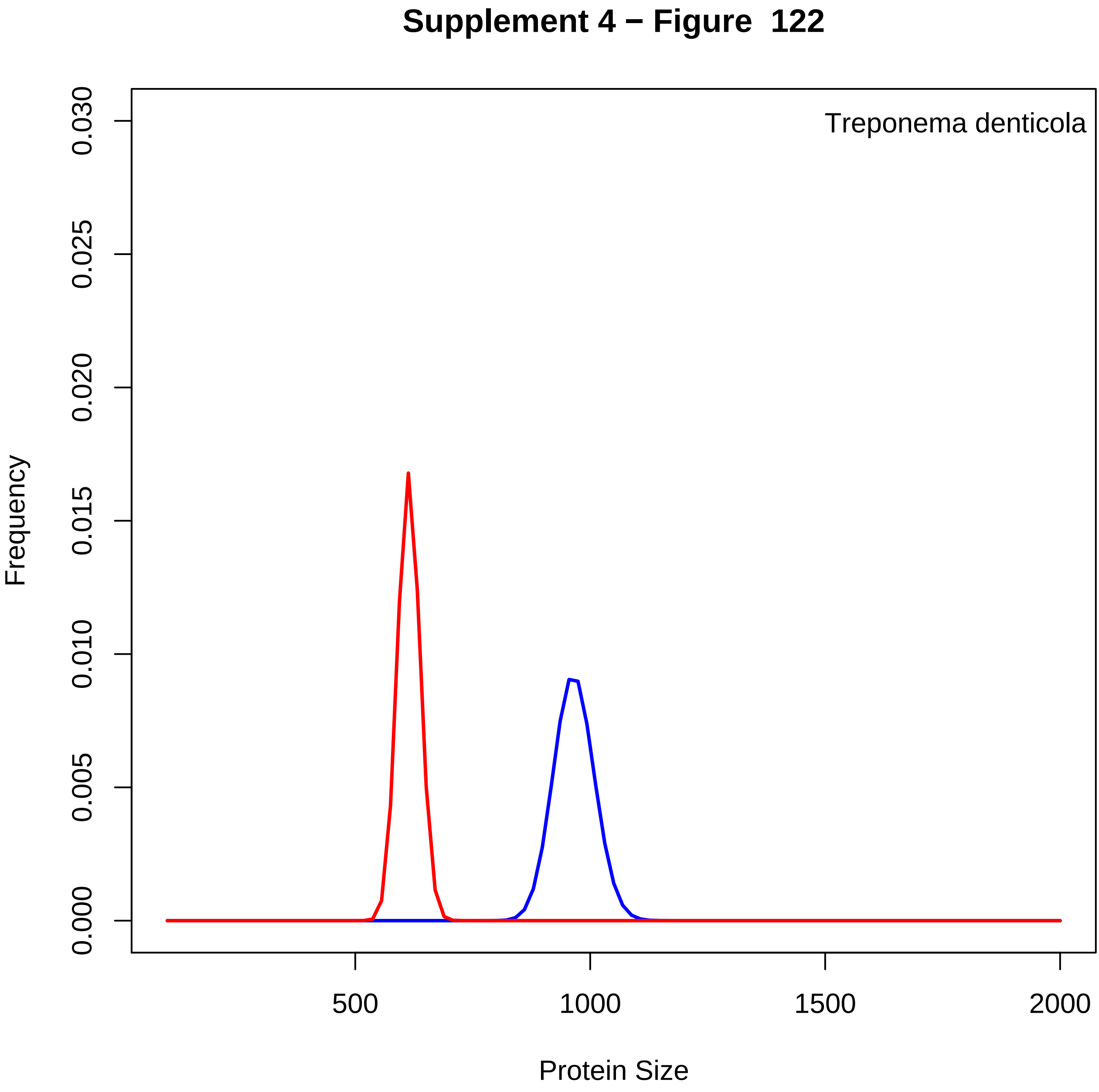

Supplement 4 − Figure 122
Treponema denticola
Frequency
0.000
0.005
0.010
0.015
0.020
0.025
0.030
500
1000
1500
2000
Protein Size

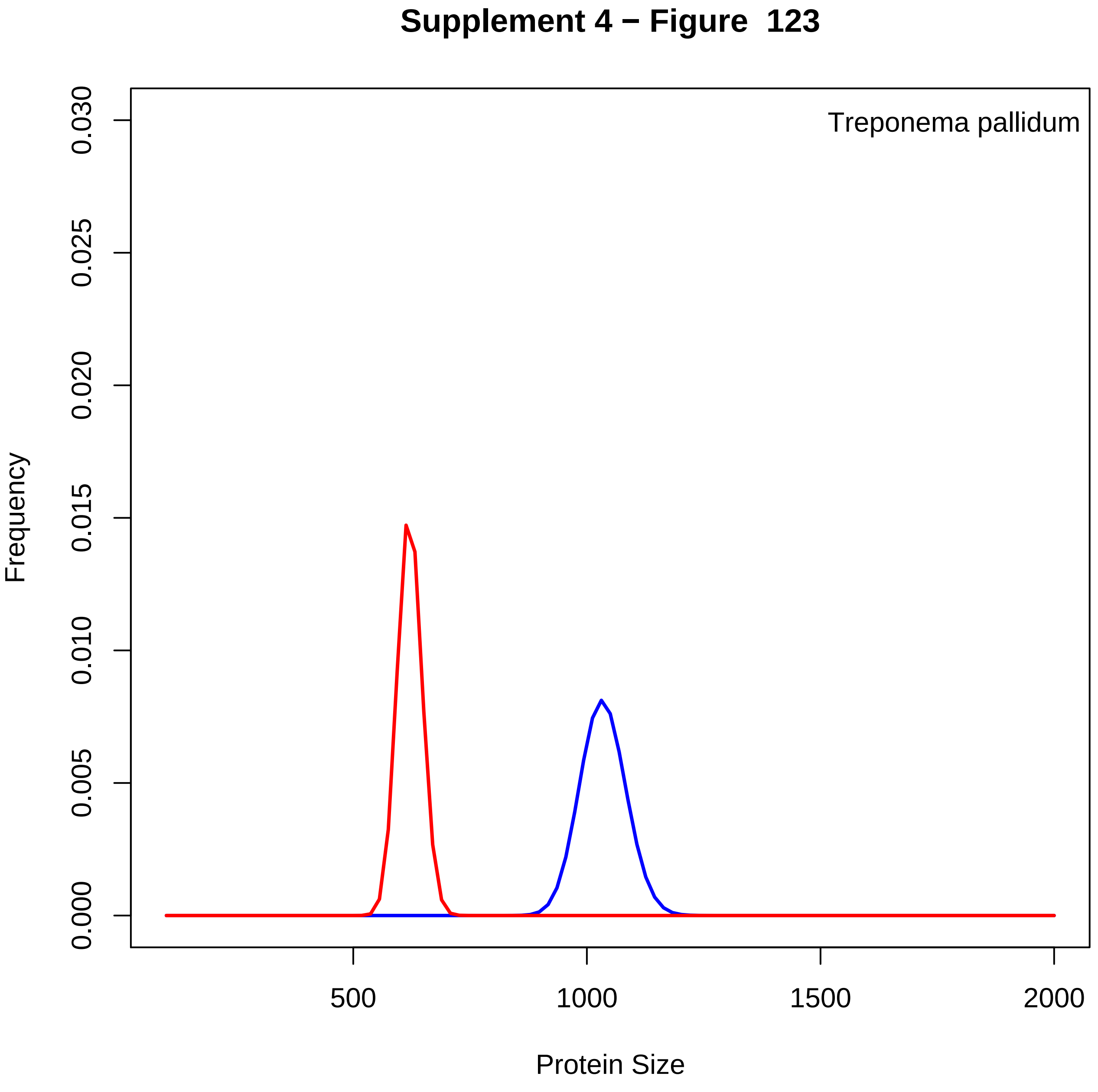
Supplement 4 − Figure 123
Treponema pallidum
Frequency
0.000
0.005
0.010
0.015
0.020
0.025
0.030
500
1000
1500
2000
Protein Size

Supplement 4 − Figure 124

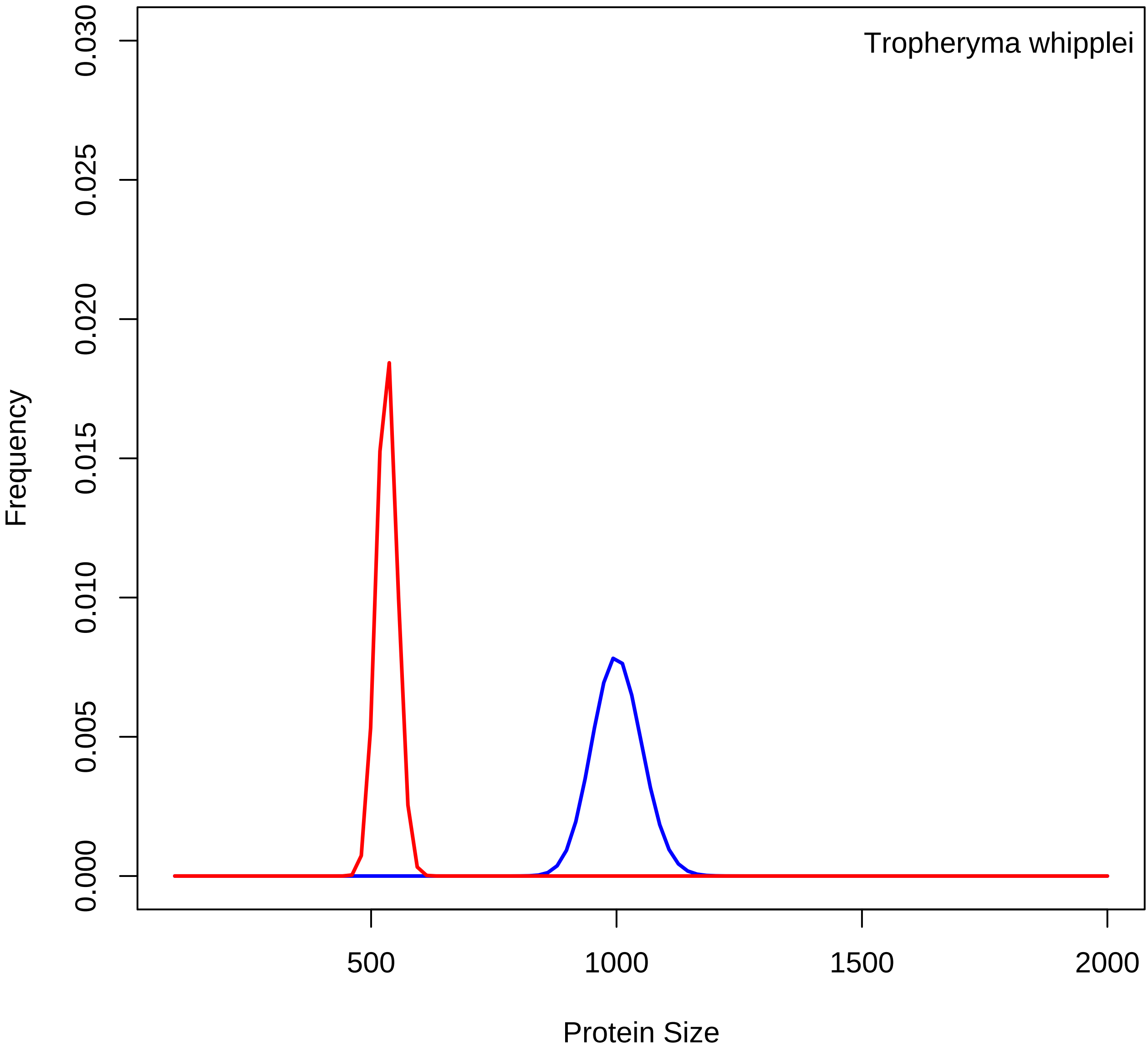

Supplement 4 − Figure 125

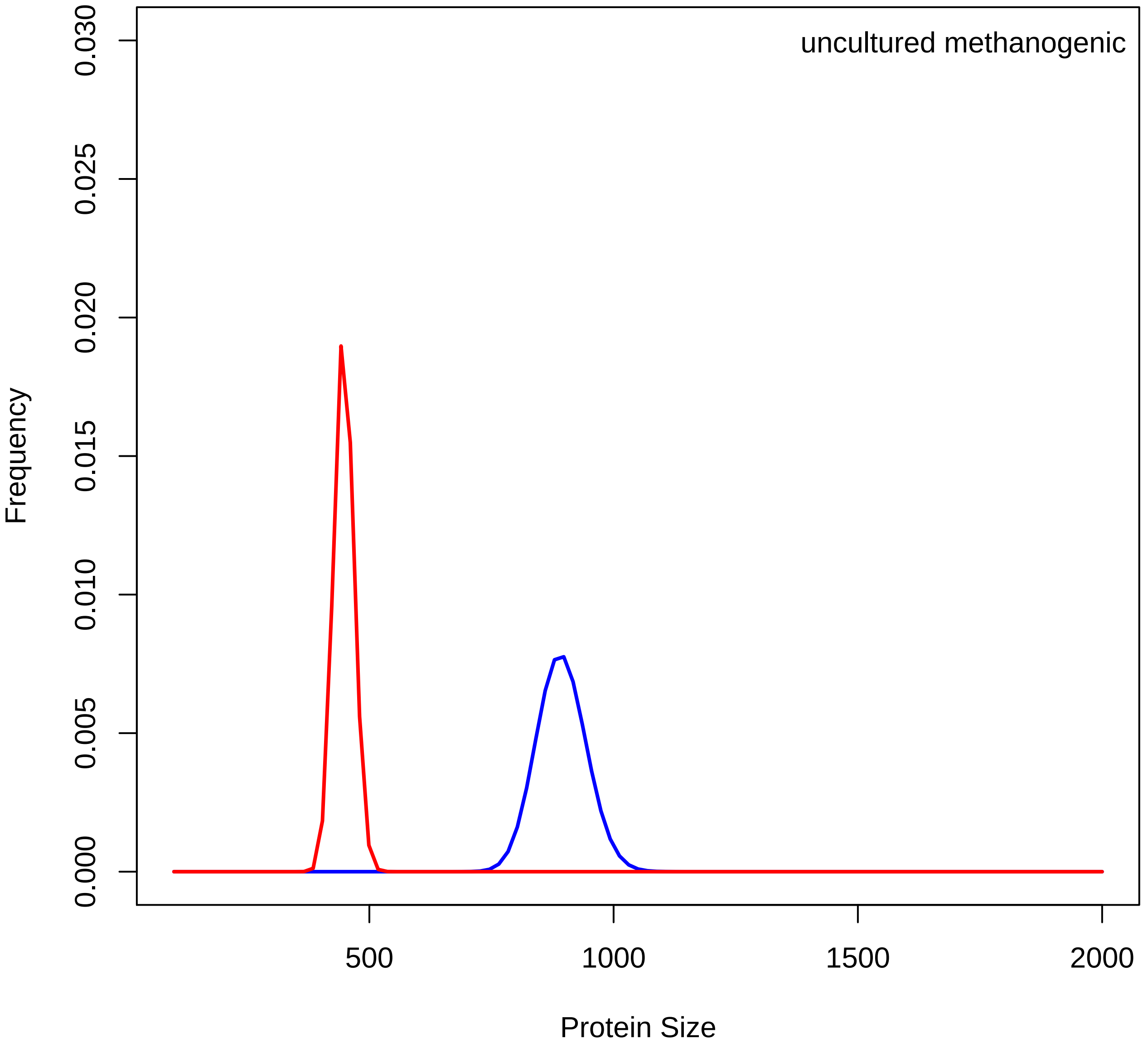

Supplement 4 − Figure 126

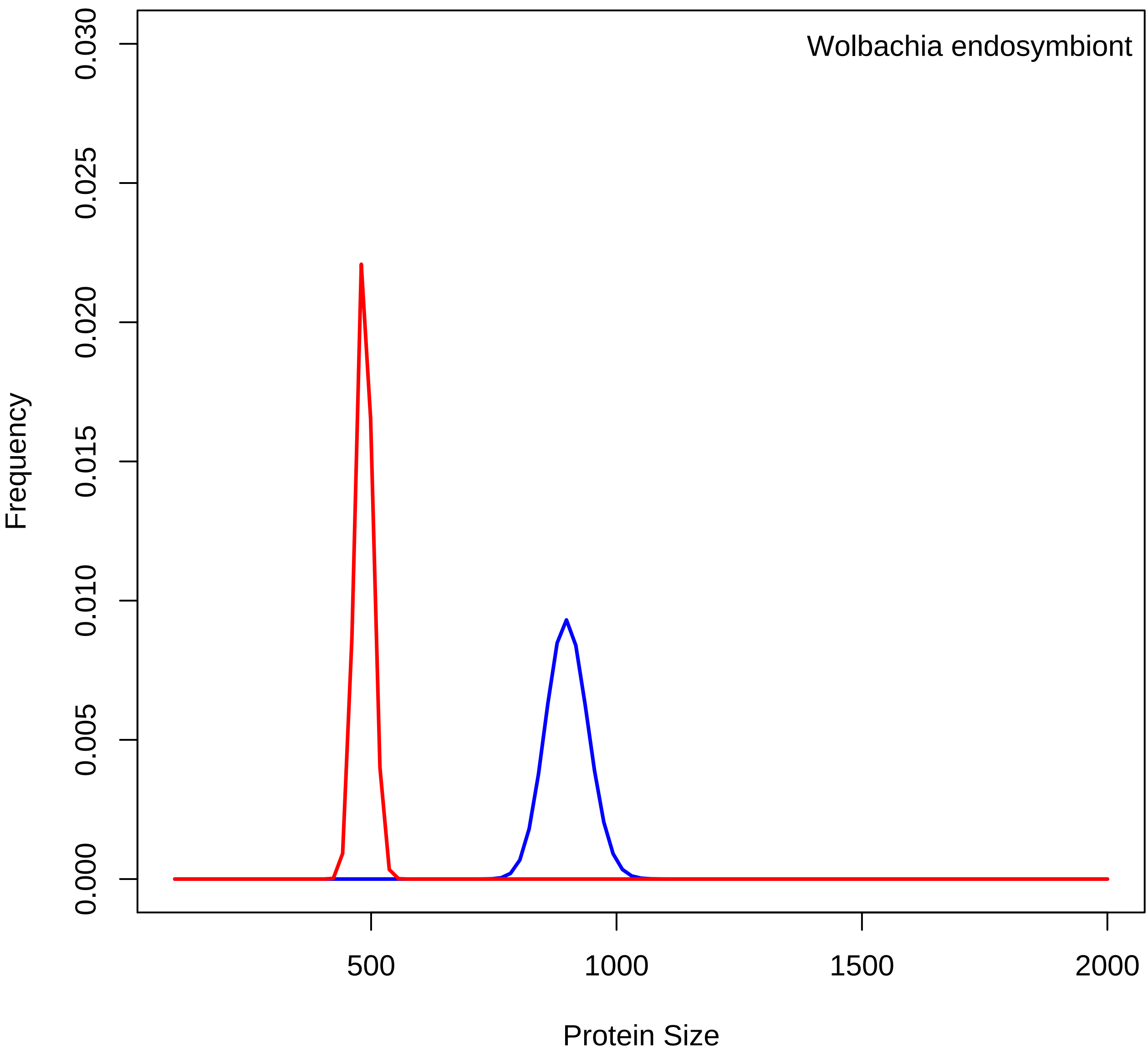

Supplement 4 − Figure 127

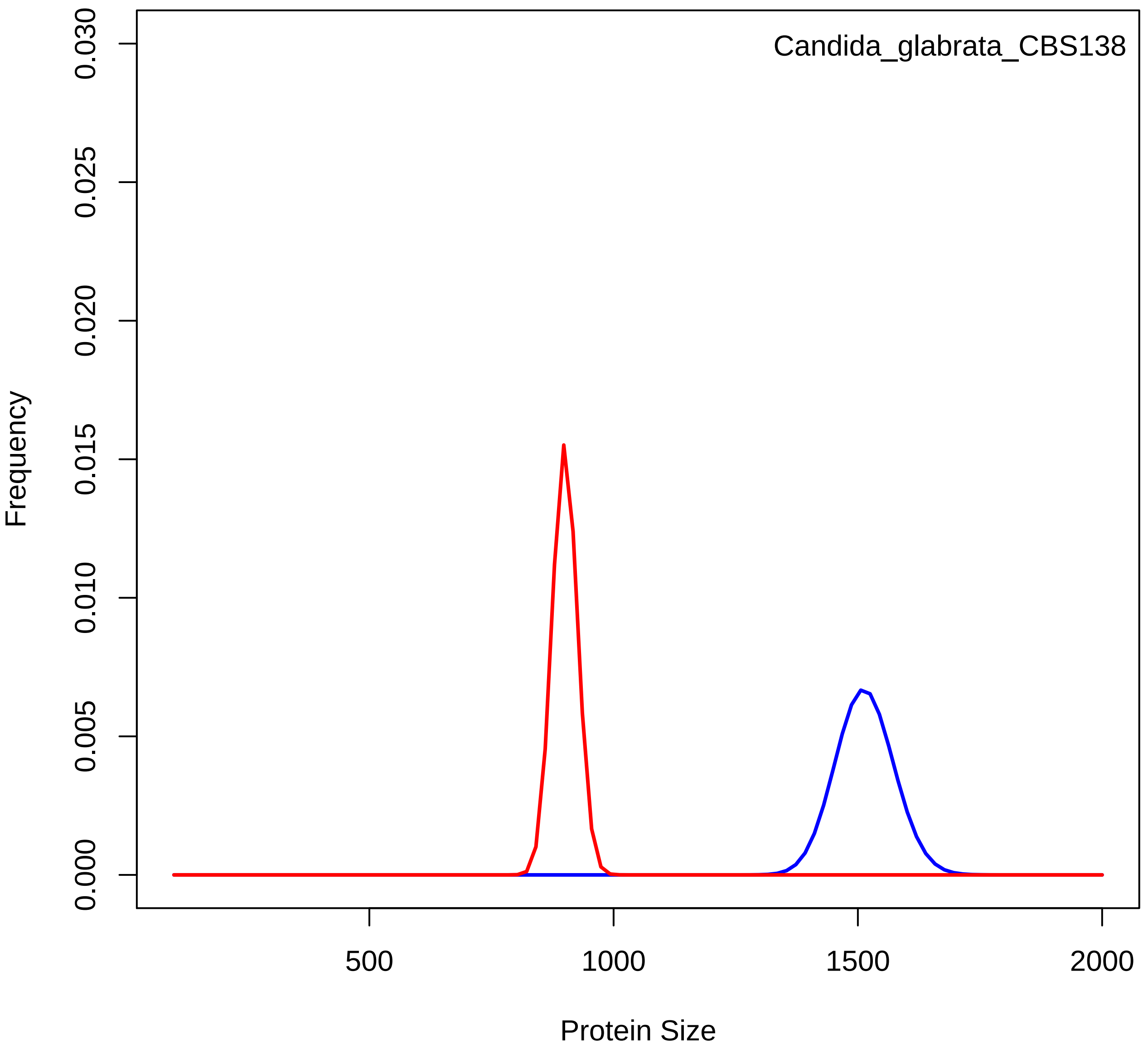

Supplement 4 − Figure 128

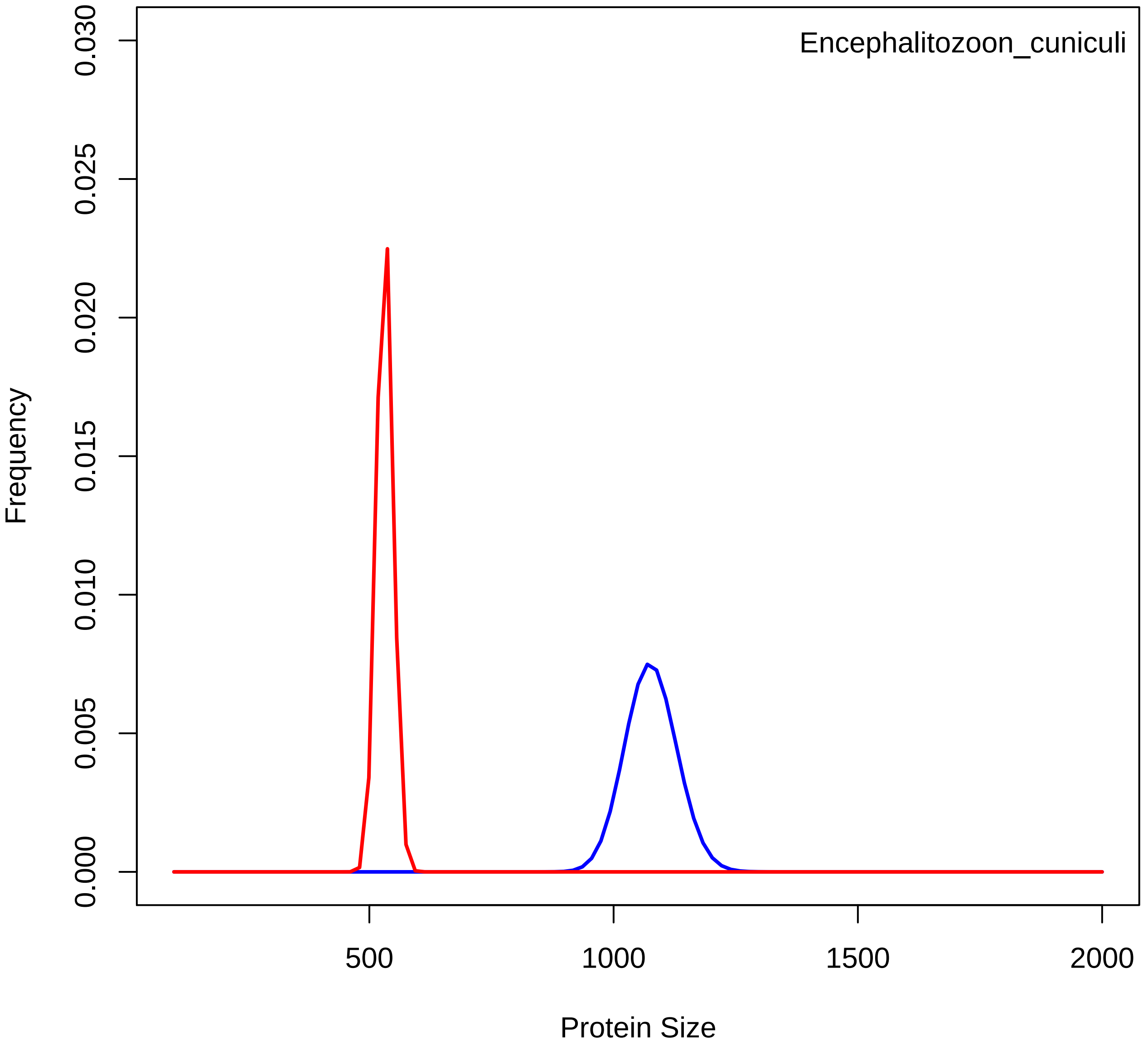

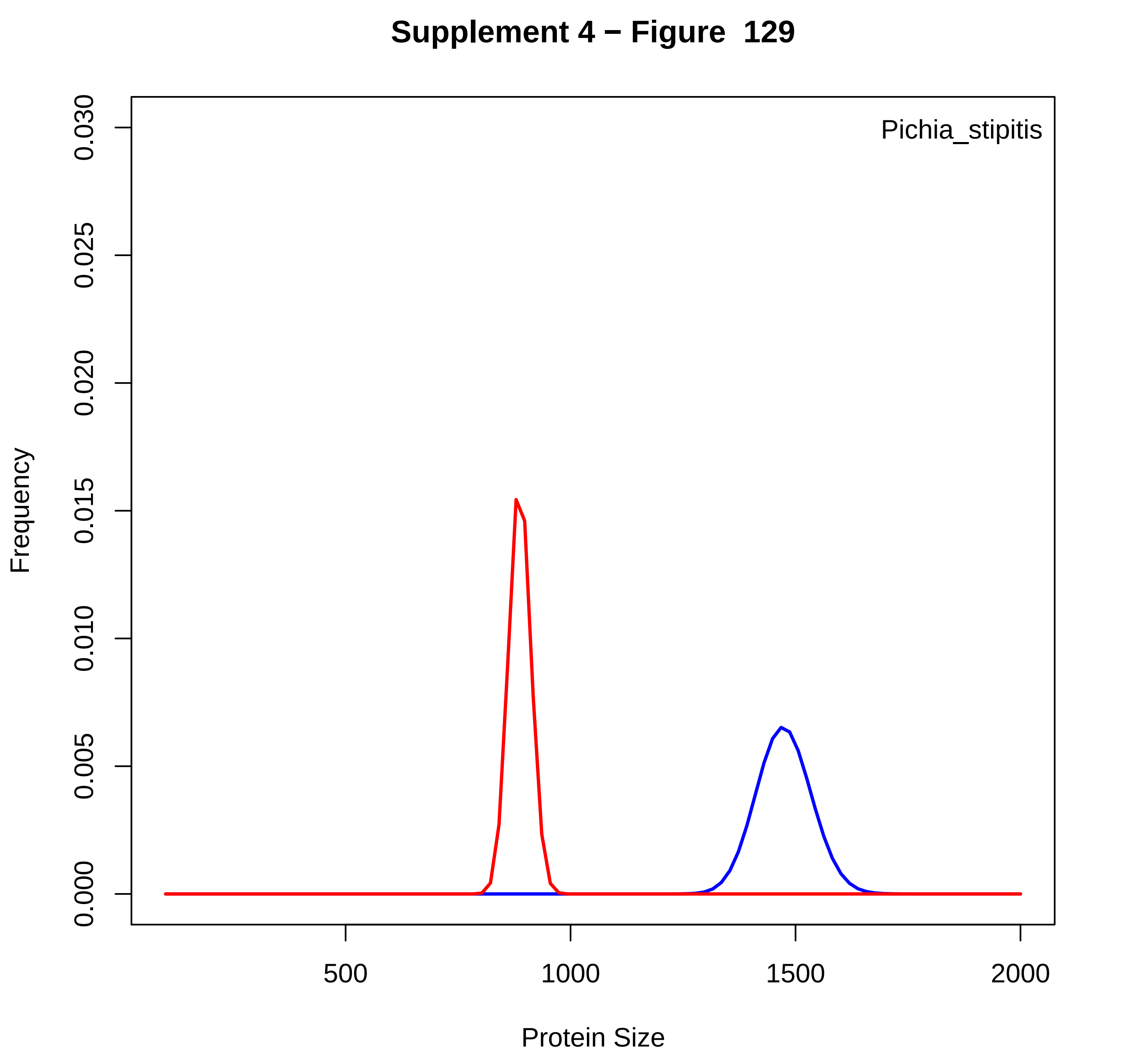

Supplement 4 − Figure 129
Pichia_stipitis
Frequency
0.000
0.005
0.010
0.015
0.020
0.025
0.030
500
1000
1500
2000
Protein Size

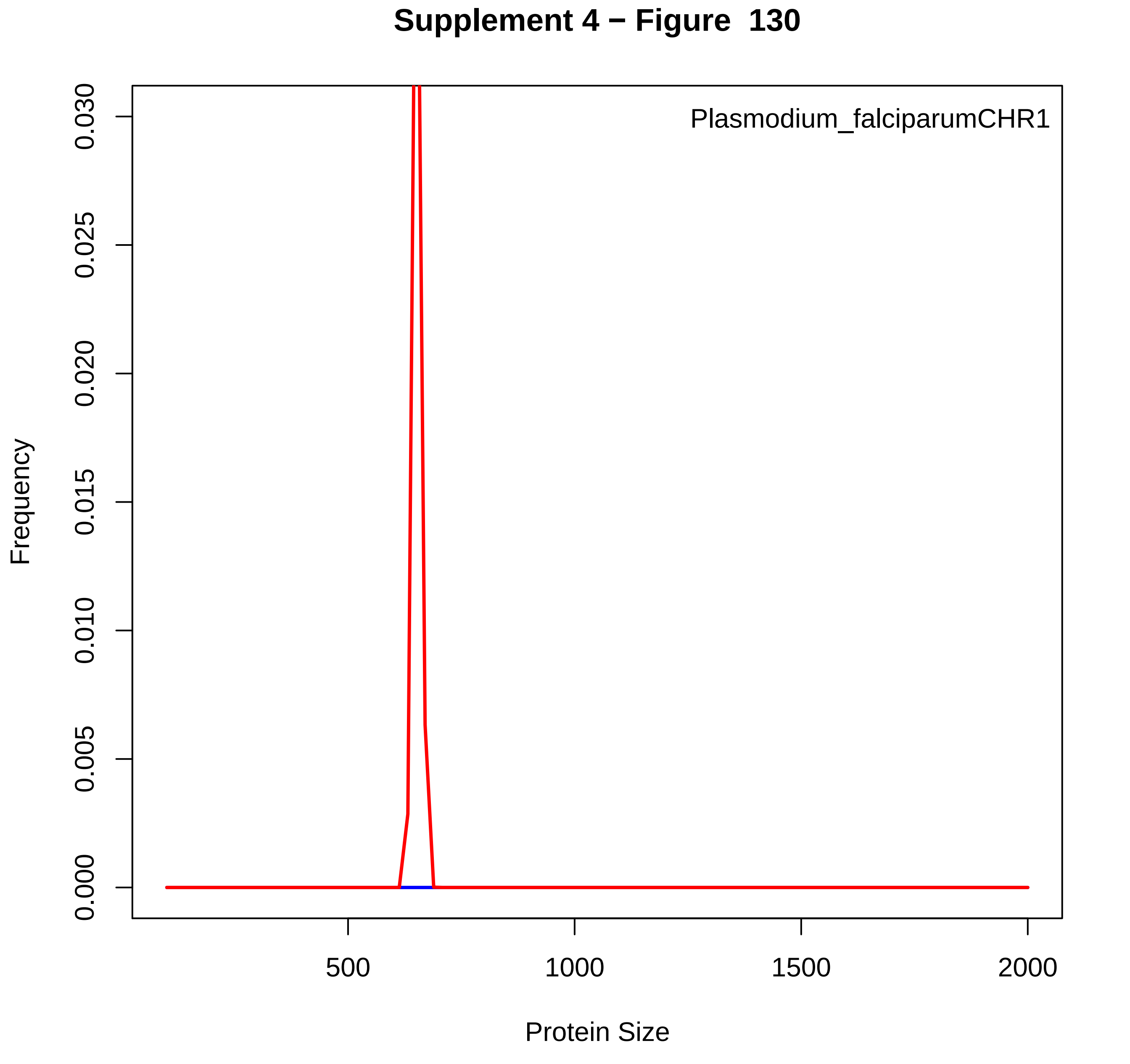

Supplement 4 − Figure 130
Plasmodium_falciparumCHR1
Frequency
0.000
0.005
0.010
0.015
0.020
0.025
0.030
Protein Size
500
1000
1500
2000

Supplement 4 − Figure 131

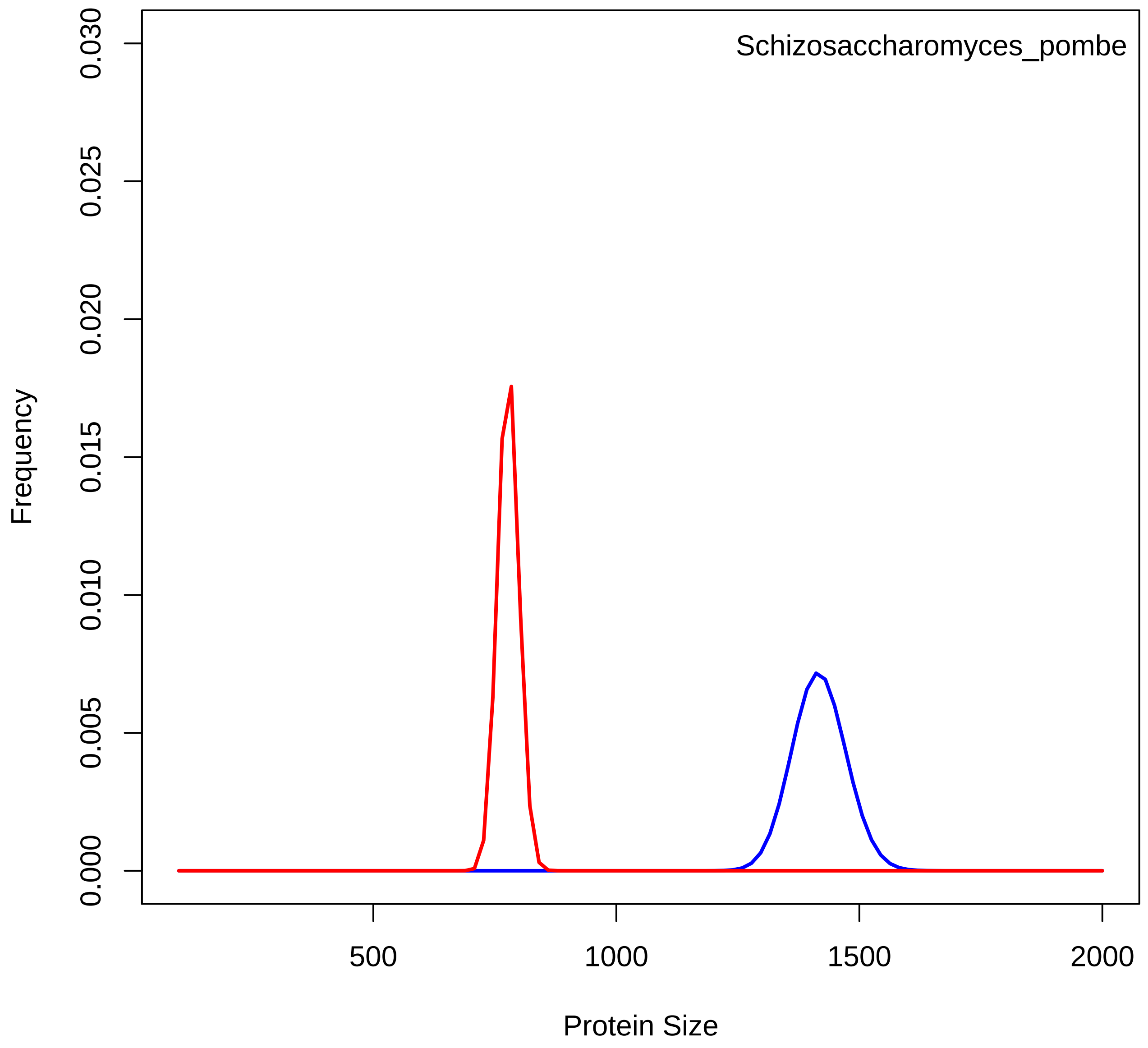

Supplement 4 − Figure 132

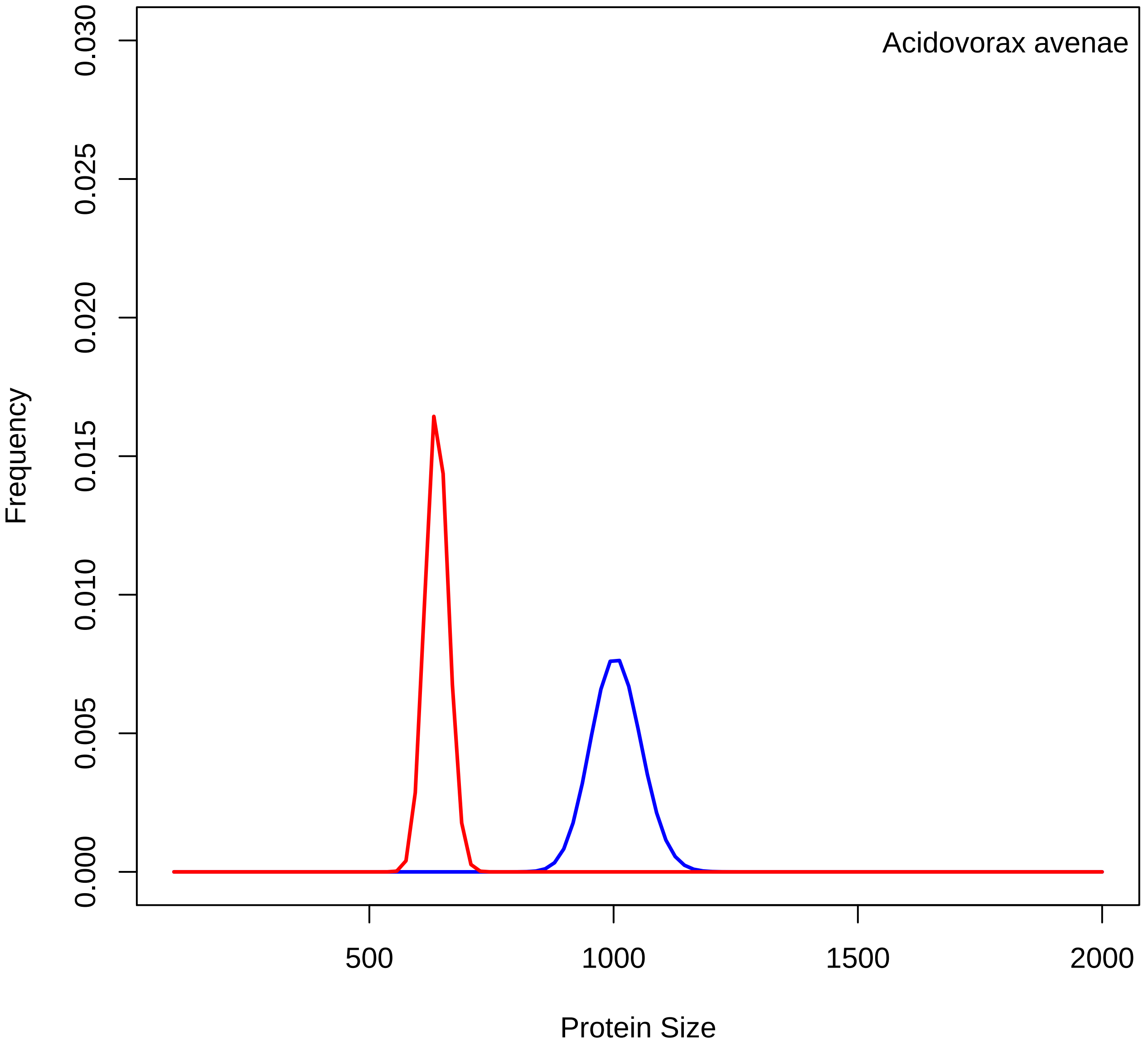

Supplement 4 − Figure 133

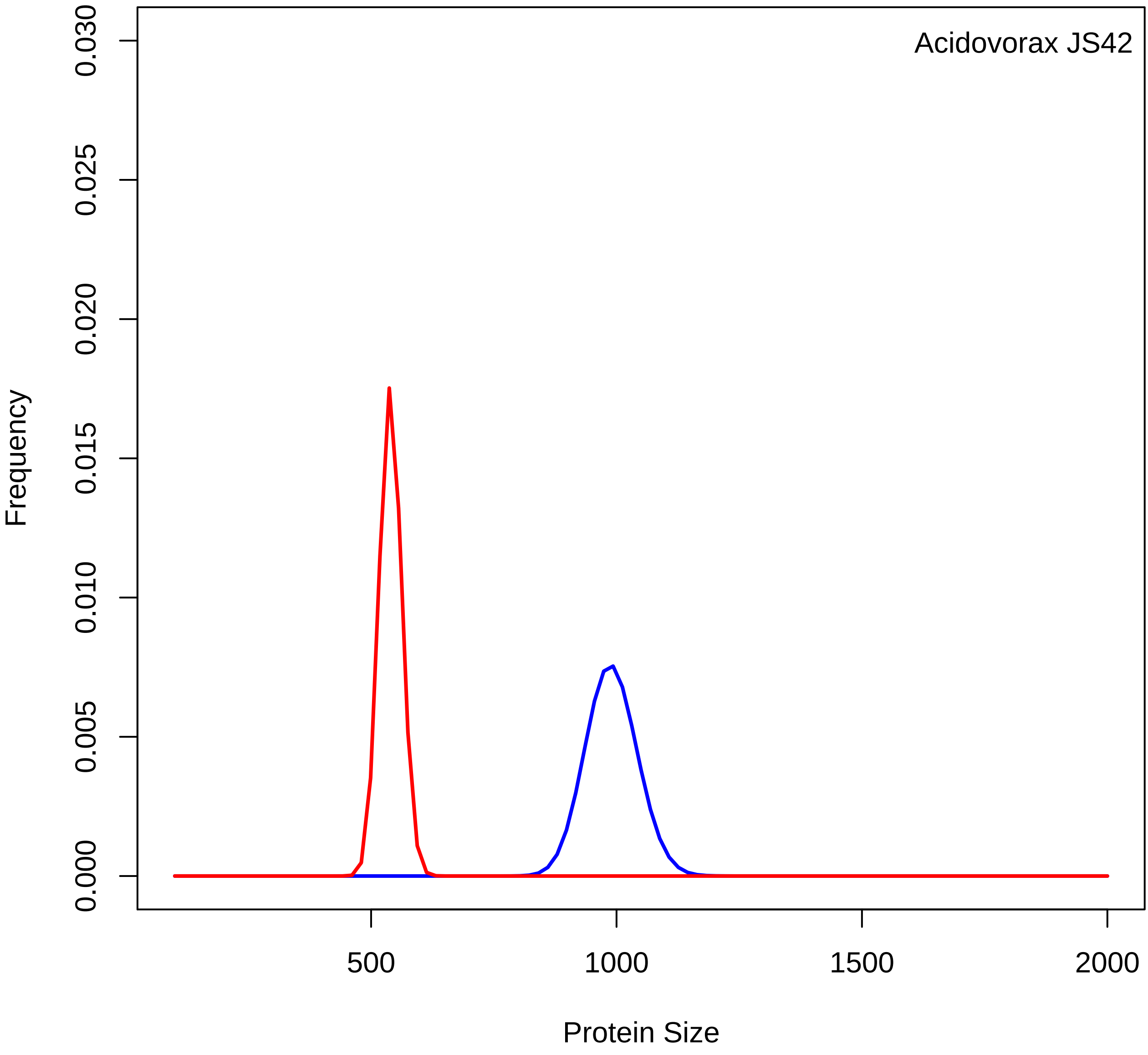

Supplement 4 − Figure 134

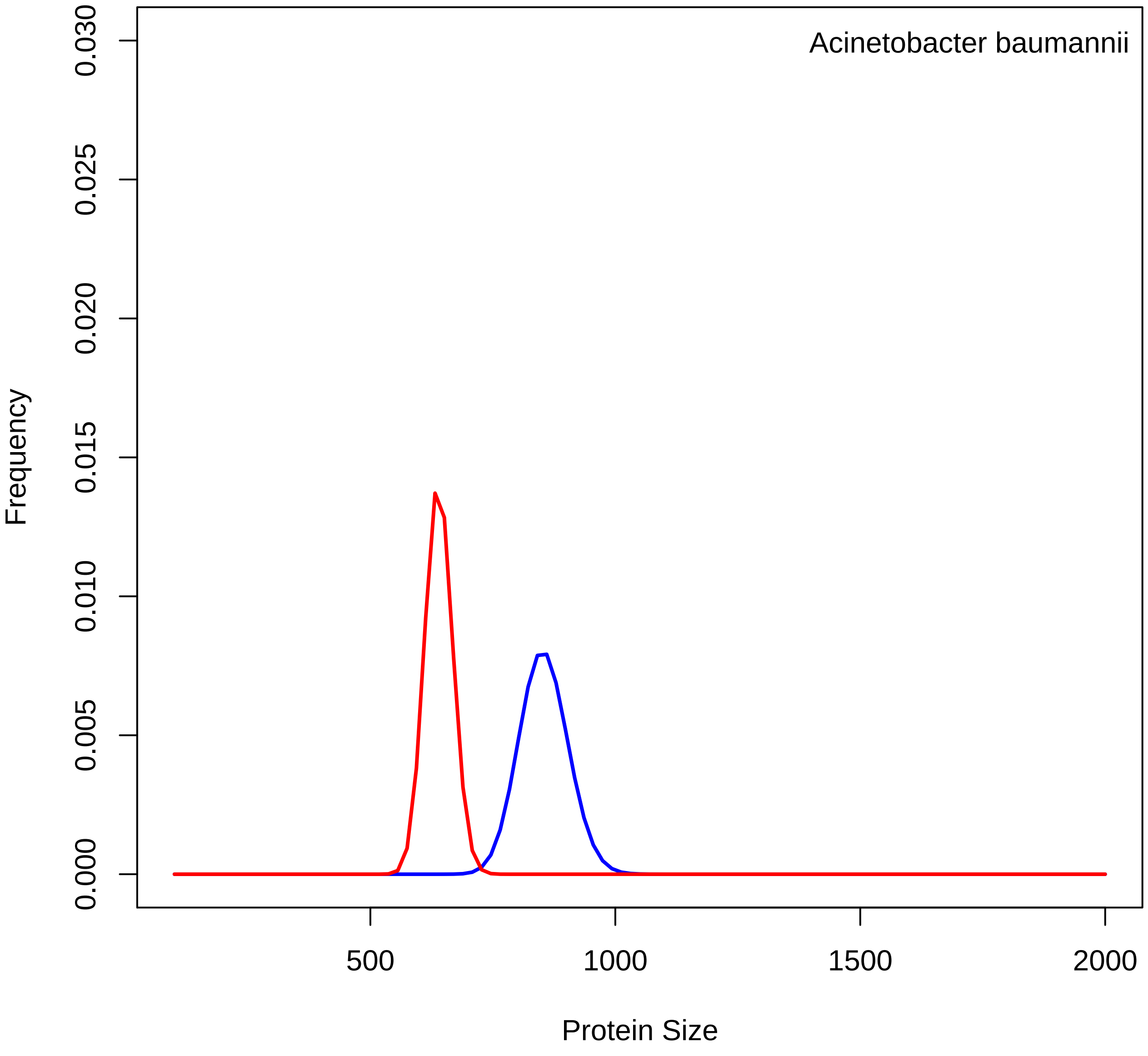

Supplement 4 − Figure 135

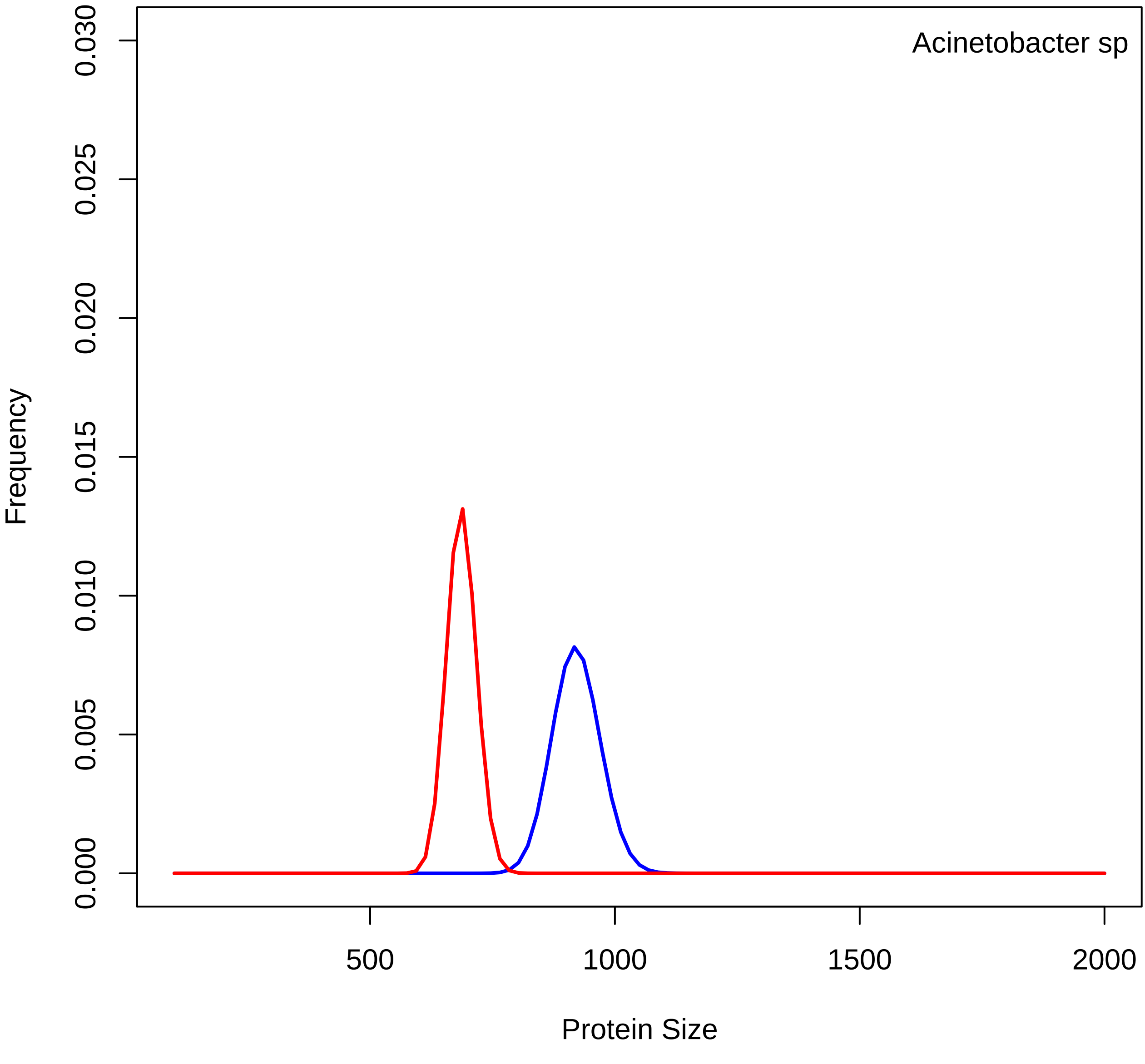

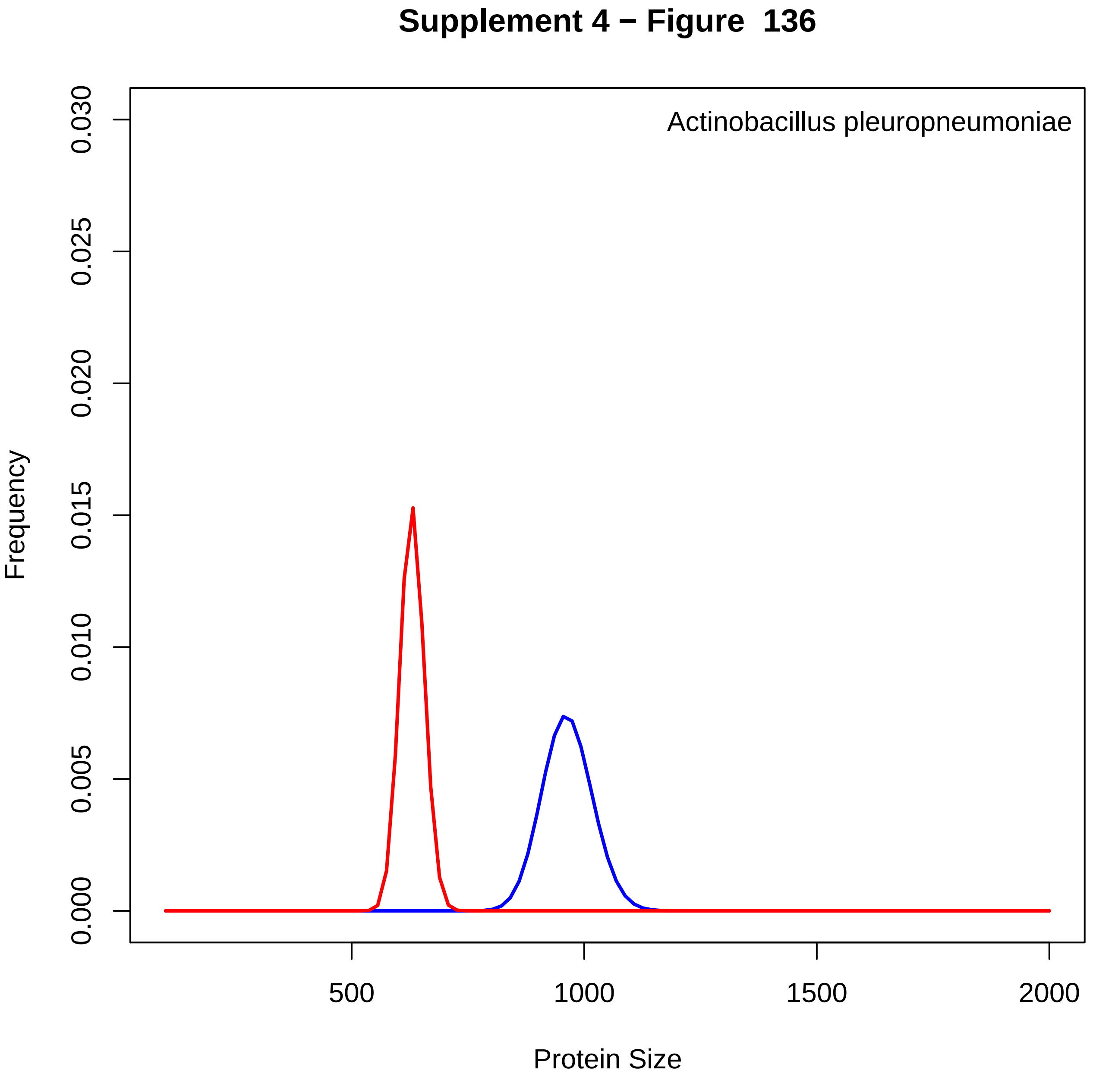
Supplement 4 − Figure 136
Actinobacillus pleuropneumoniae
Frequency
0.000
0.005
0.010
0.015
0.020
0.025
0.030
500
1000
1500
2000
Protein Size

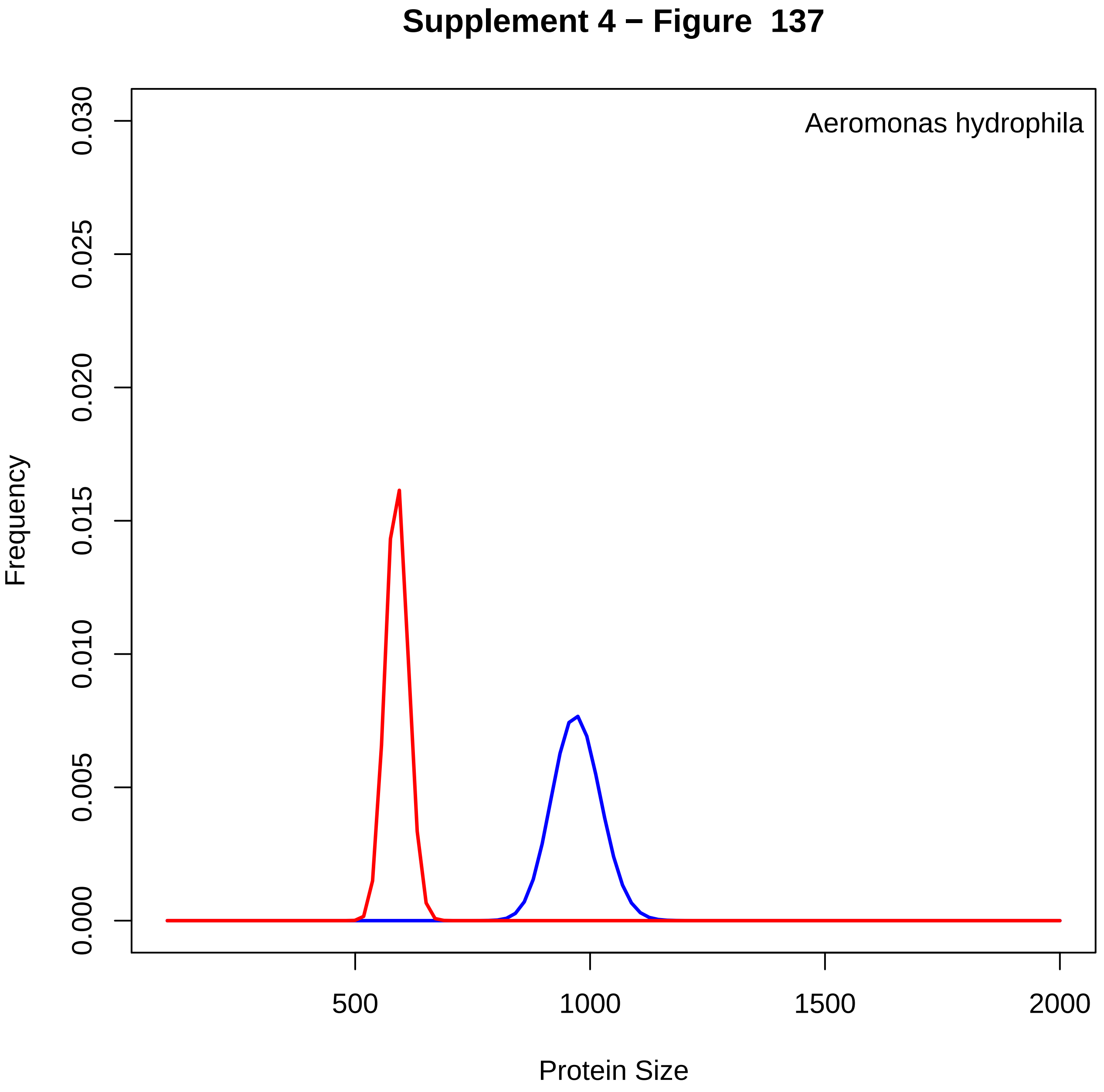
Supplement 4 − Figure 137
Aeromonas hydrophila
Frequency
0.000
0.005
0.010
0.015
0.020
0.025
0.030
500
1000
1500
2000
Protein Size

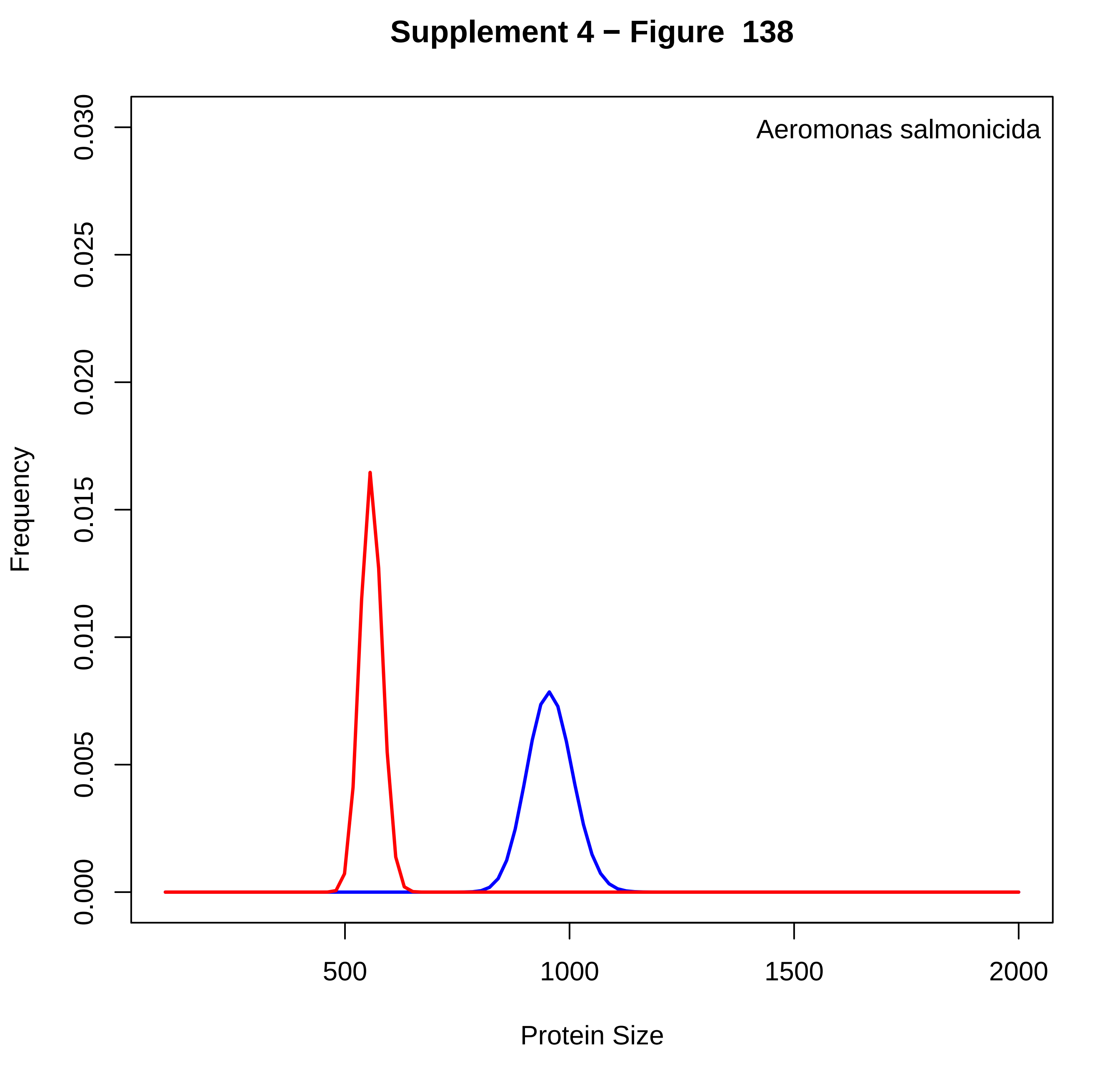
Supplement 4 − Figure 138
Aeromonas salmonicida
Frequency
0.000
0.005
0.010
0.015
0.020
0.025
0.030
500
1000
1500
2000
Protein Size

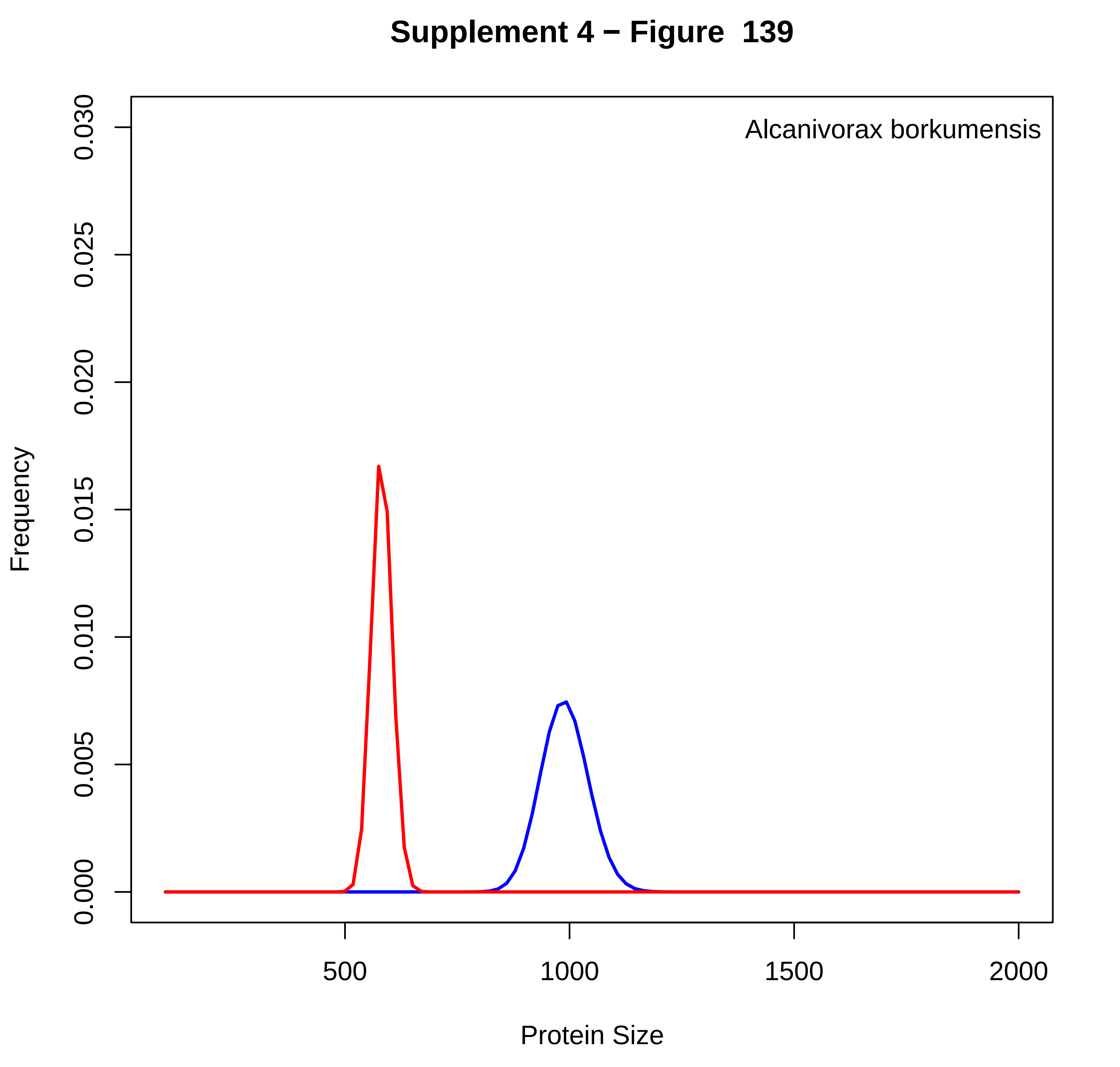

Supplement 4 − Figure 139
Alcanivorax borkumensis
Frequency
0.000
0.005
0.010
0.015
0.020
0.025
0.030
500
1000
1500
2000
Protein Size

Supplement 4 − Figure 140

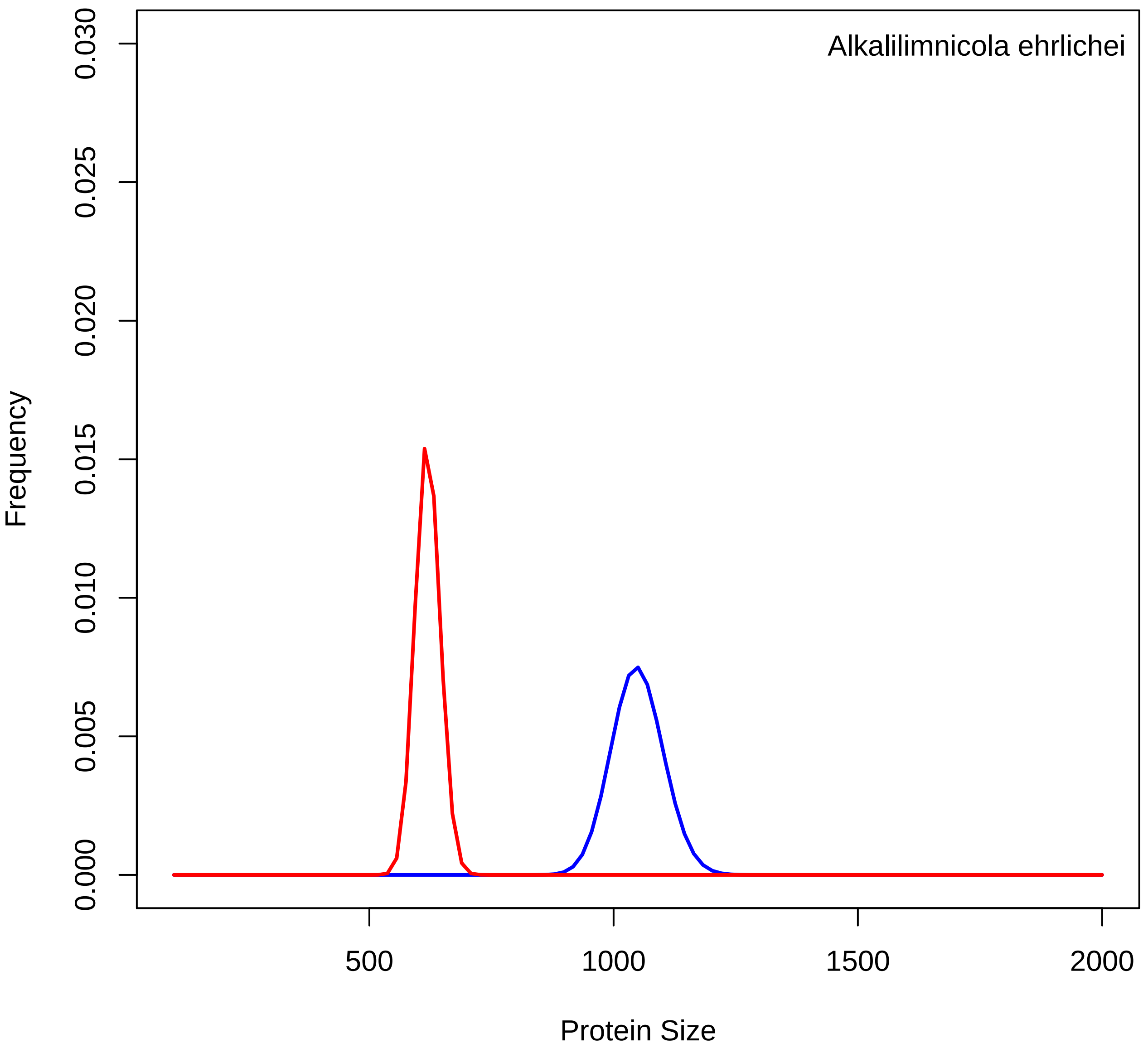

Supplement 4 − Figure 141

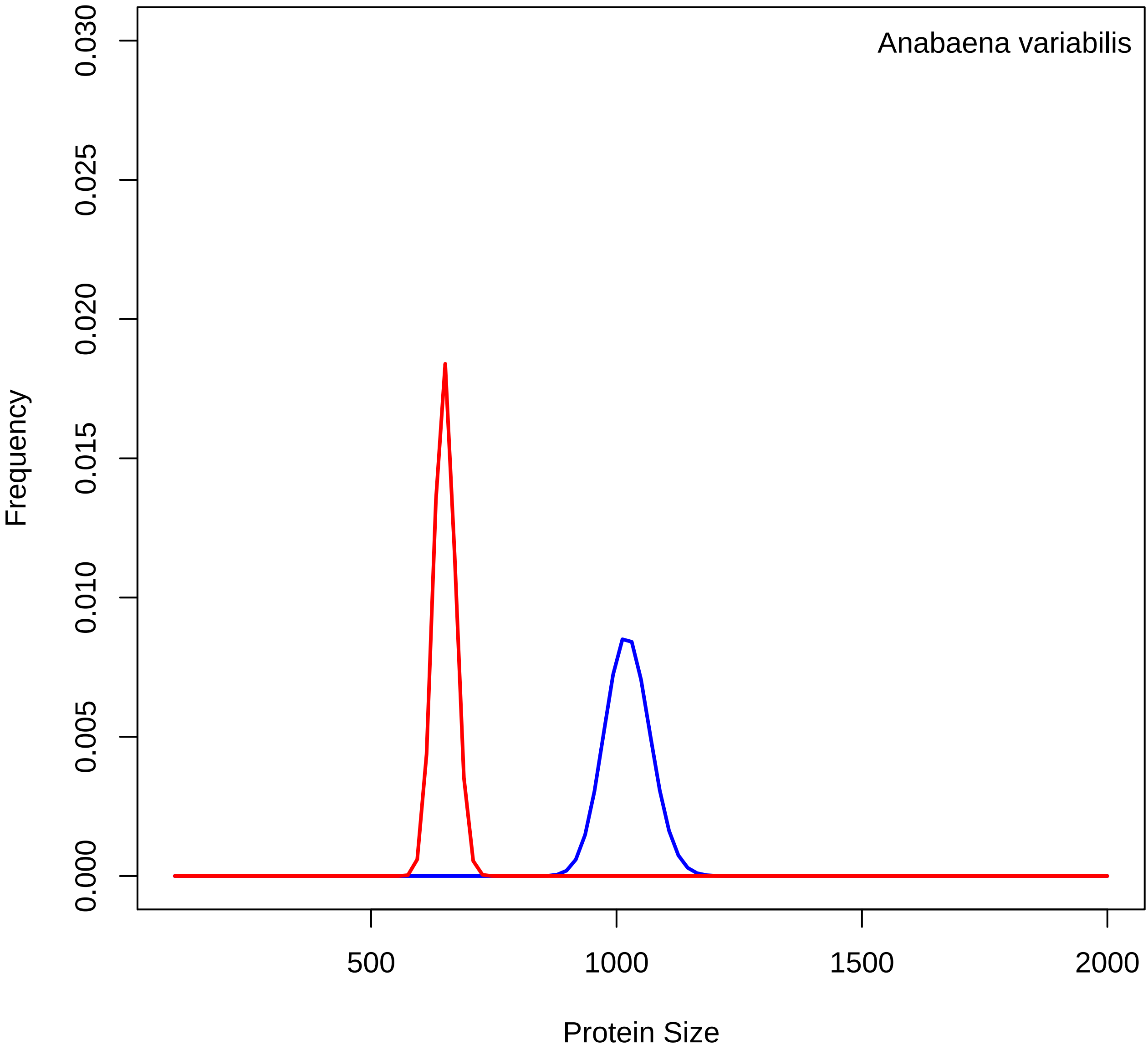

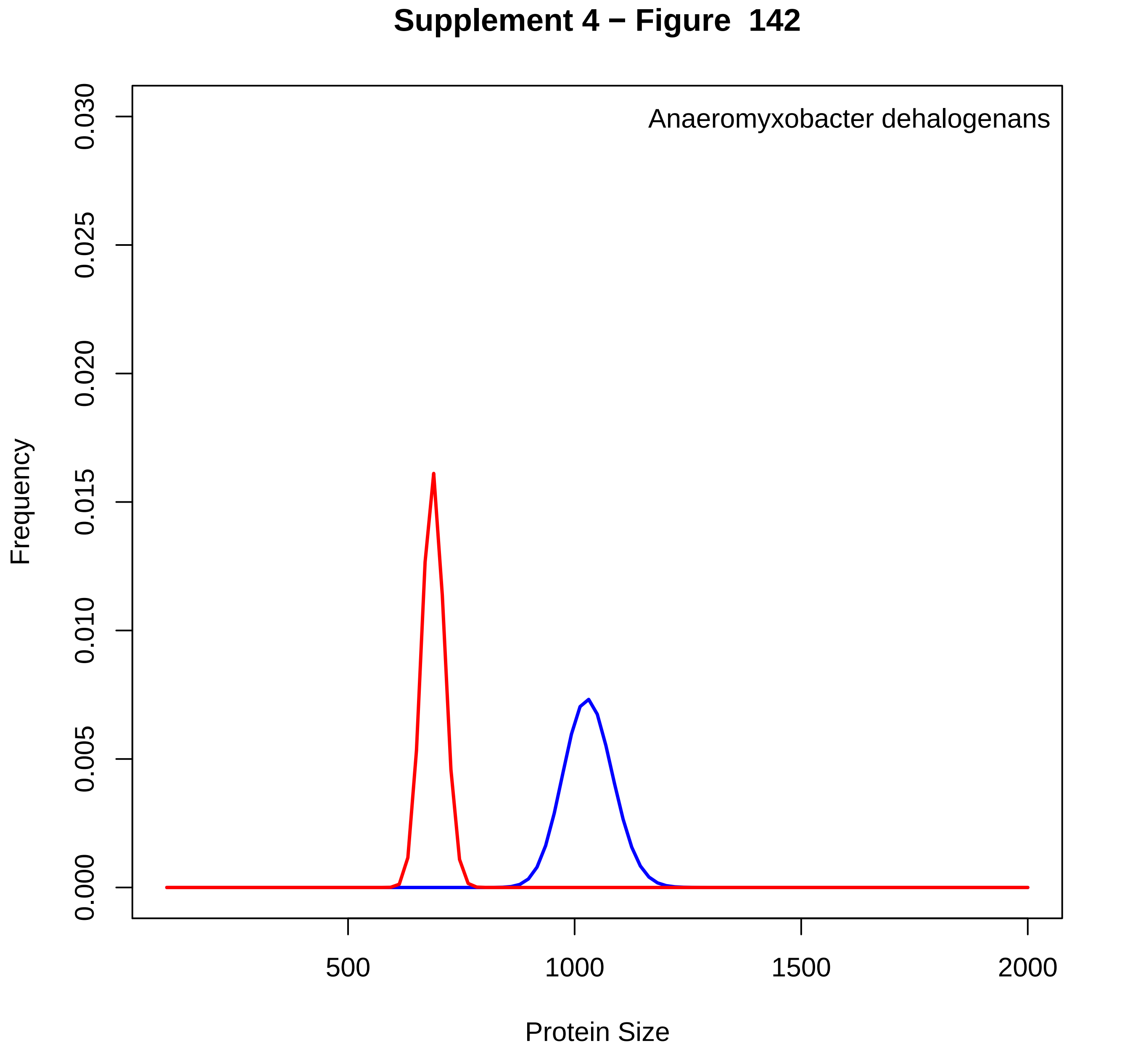

Supplement 4 − Figure 142
Anaeromyxobacter dehalogenans
Frequency
0.000
0.005
0.010
0.015
0.020
0.025
0.030
500
1000
1500
2000
Protein Size

Supplement 4 − Figure 143

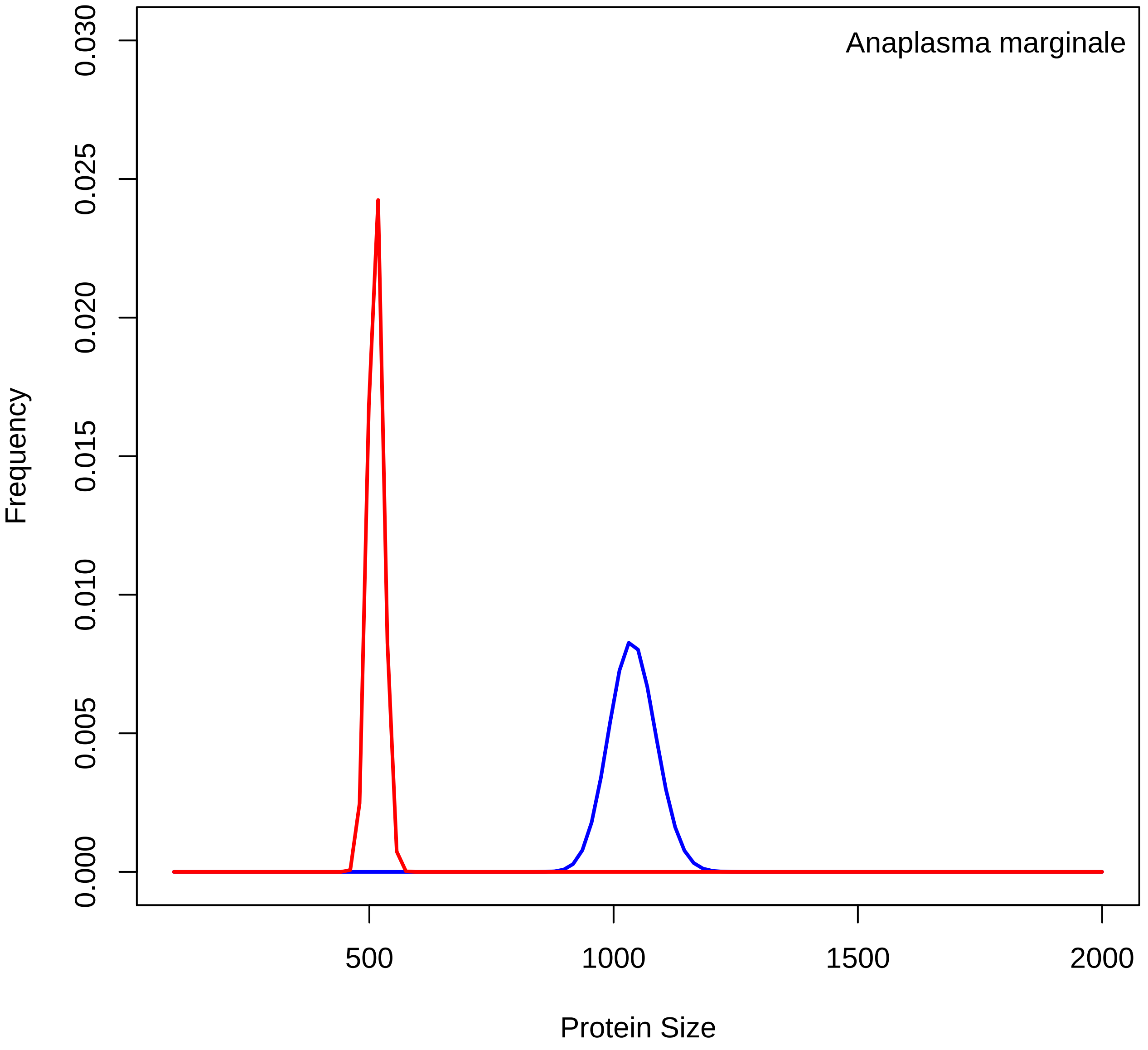

Supplement 4 − Figure 144

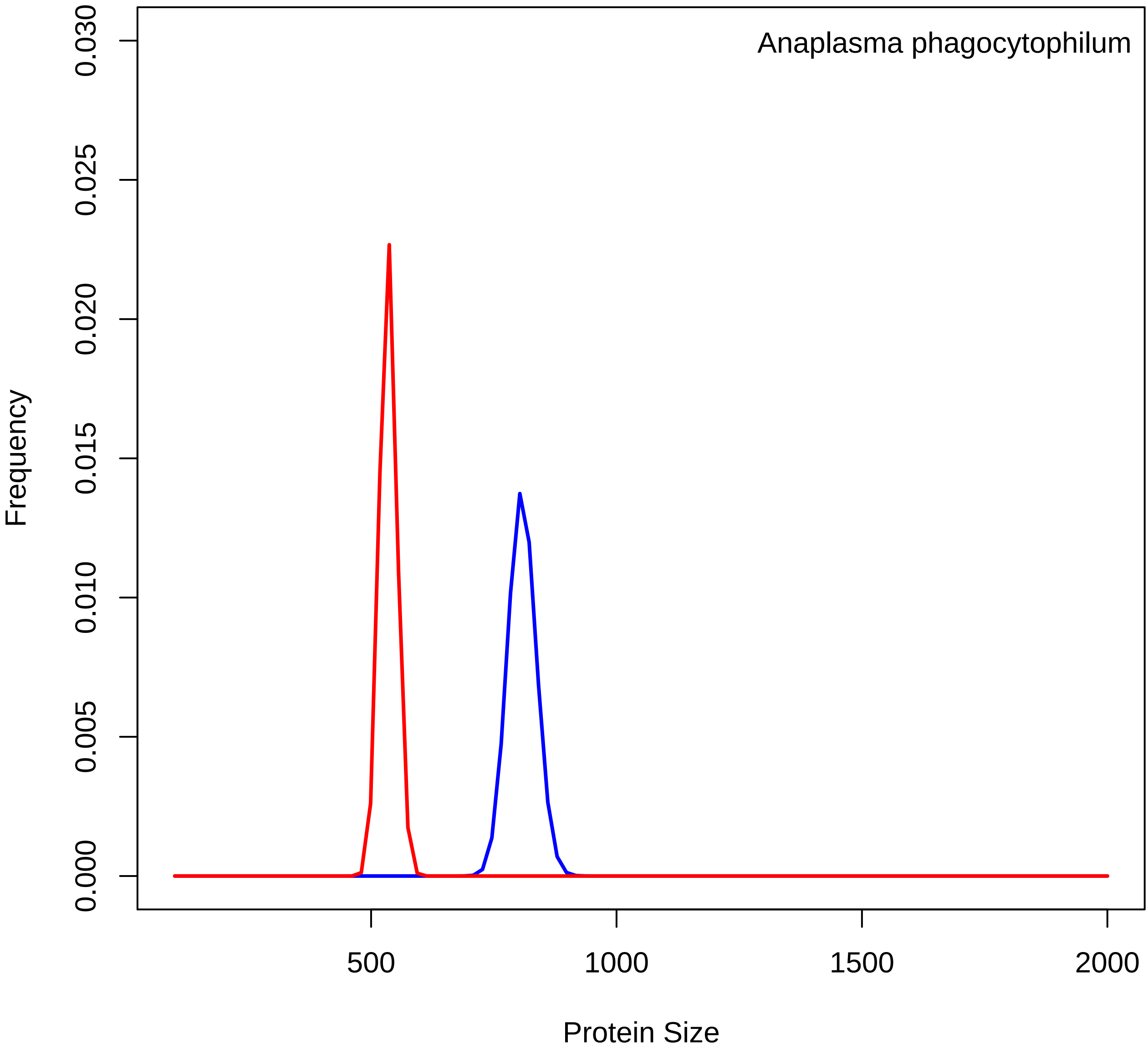

Supplement 4 − Figure 145

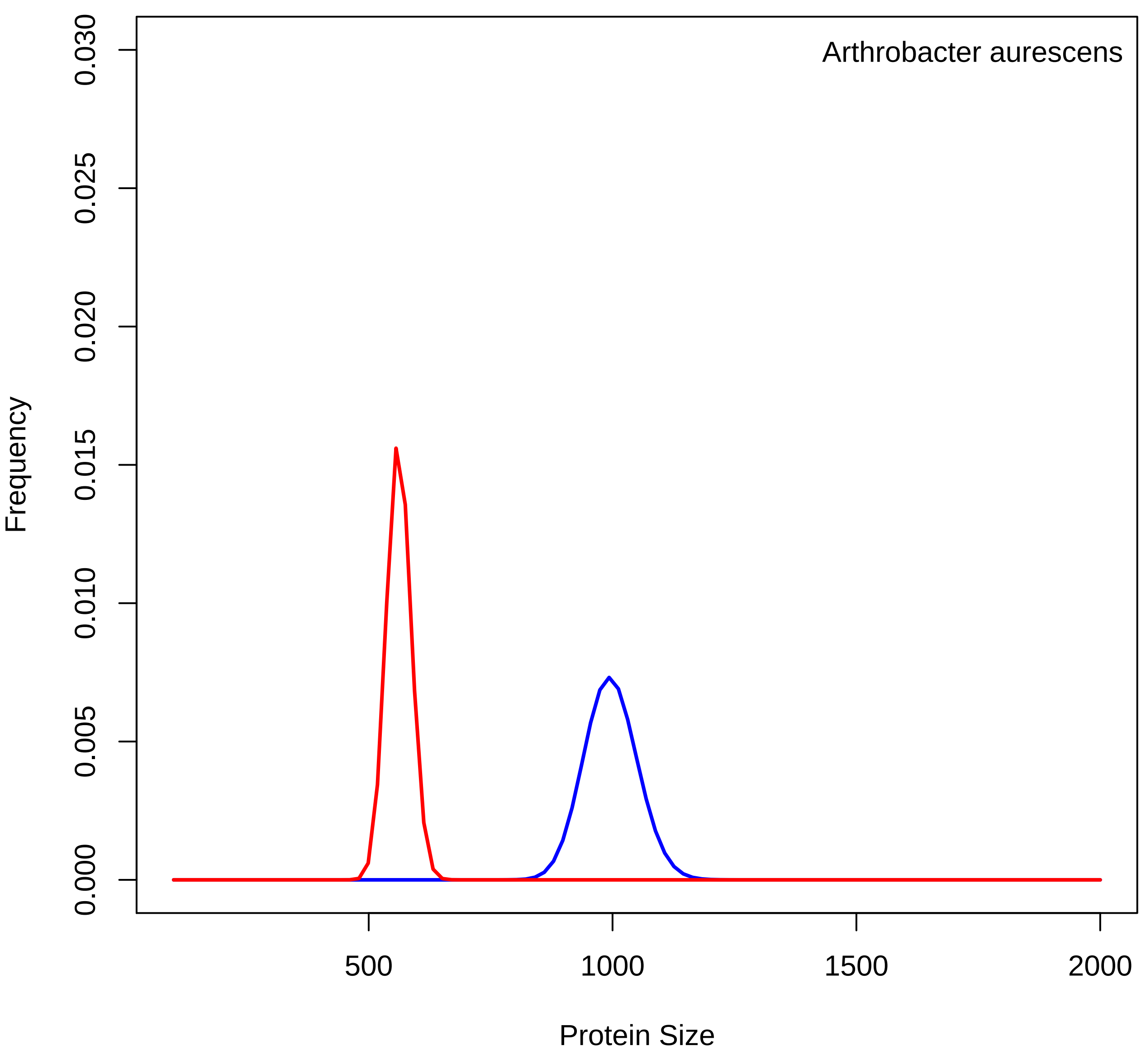

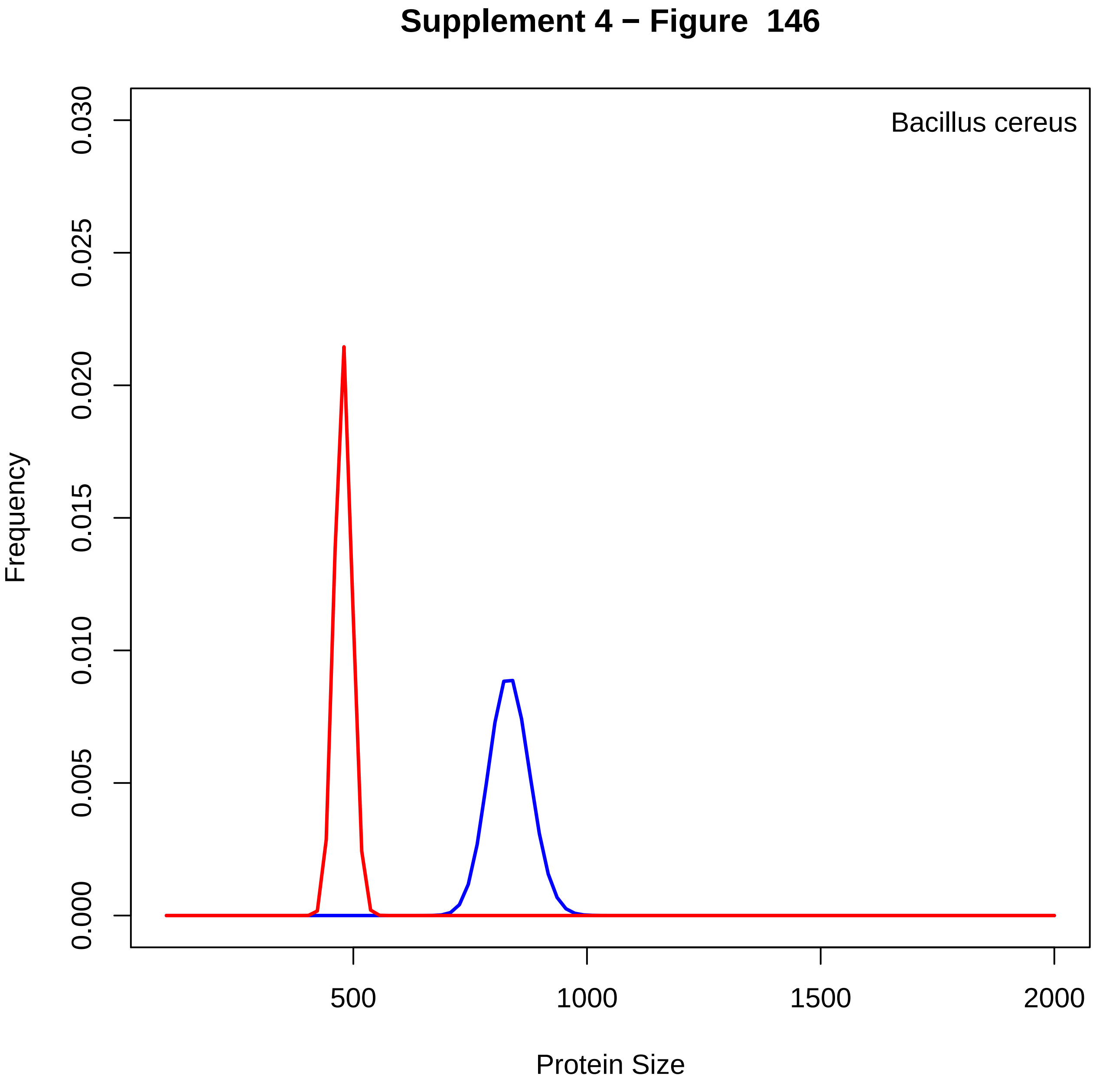
Supplement 4 − Figure 146
Bacillus cereus
Frequency
0.000
0.005
0.010
0.015
0.020
0.025
0.030
Protein Size
500
1000
1500
2000

Supplement 4 − Figure 147

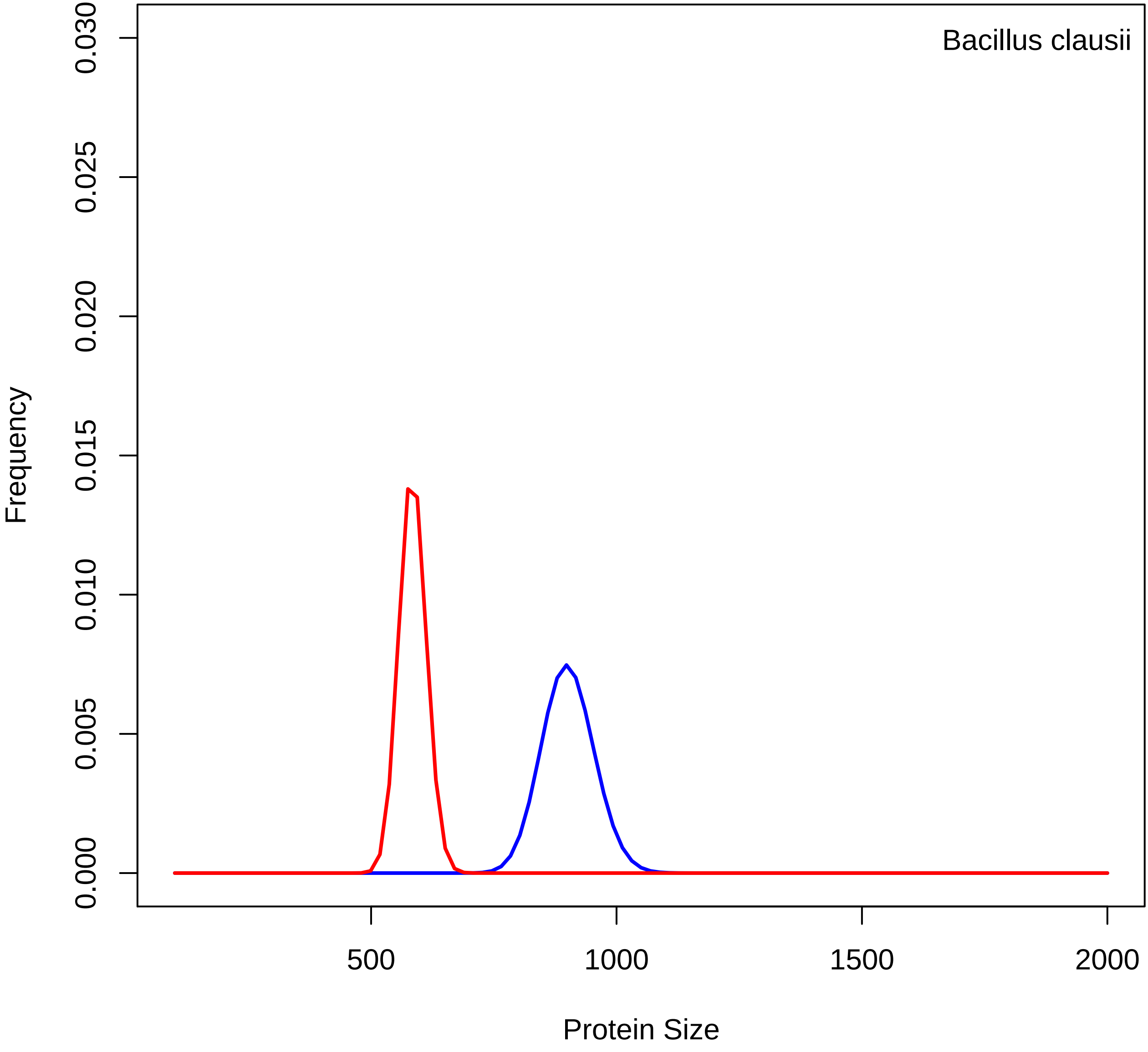

Supplement 4 − Figure 148

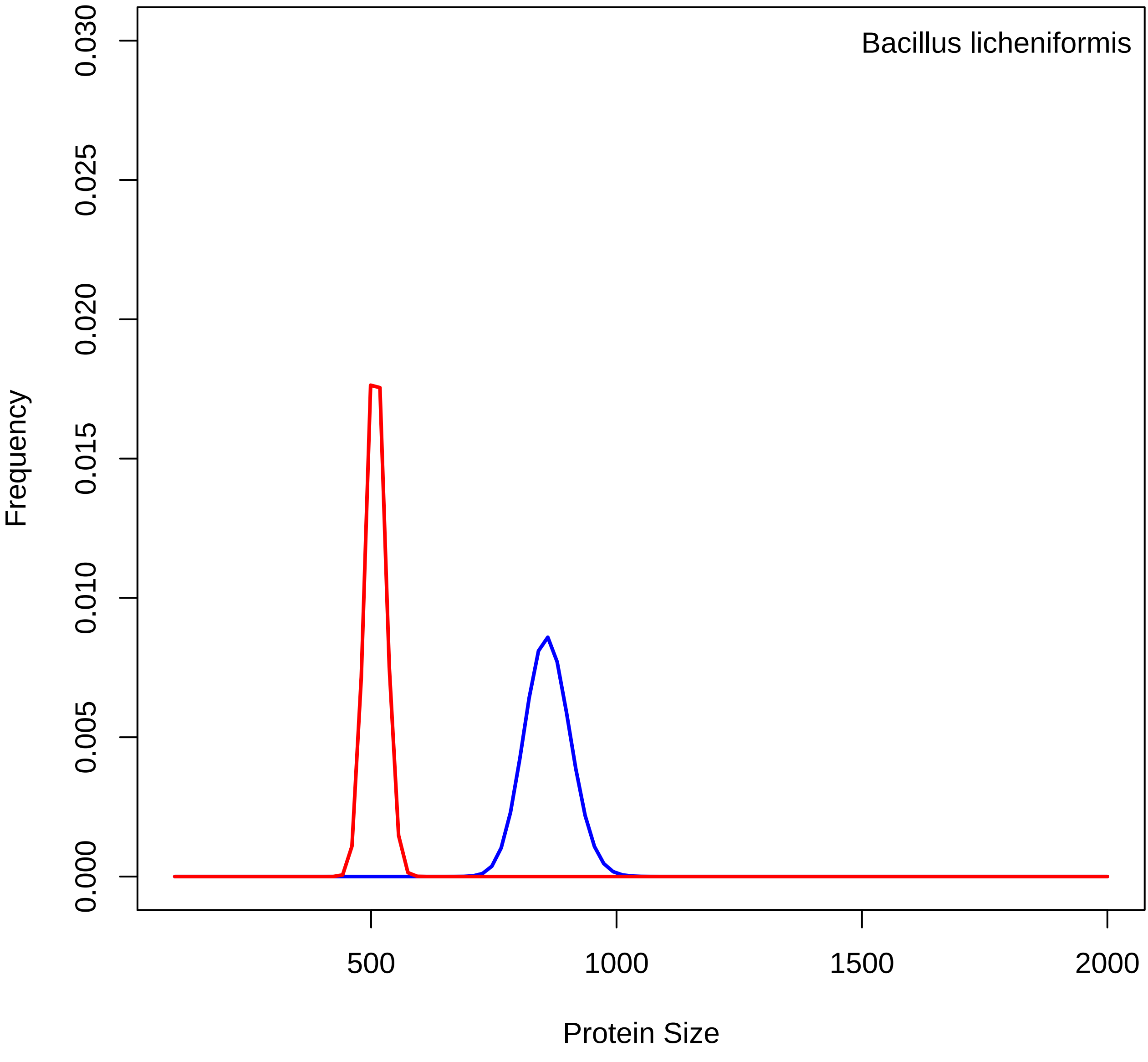

Supplement 4 − Figure 149

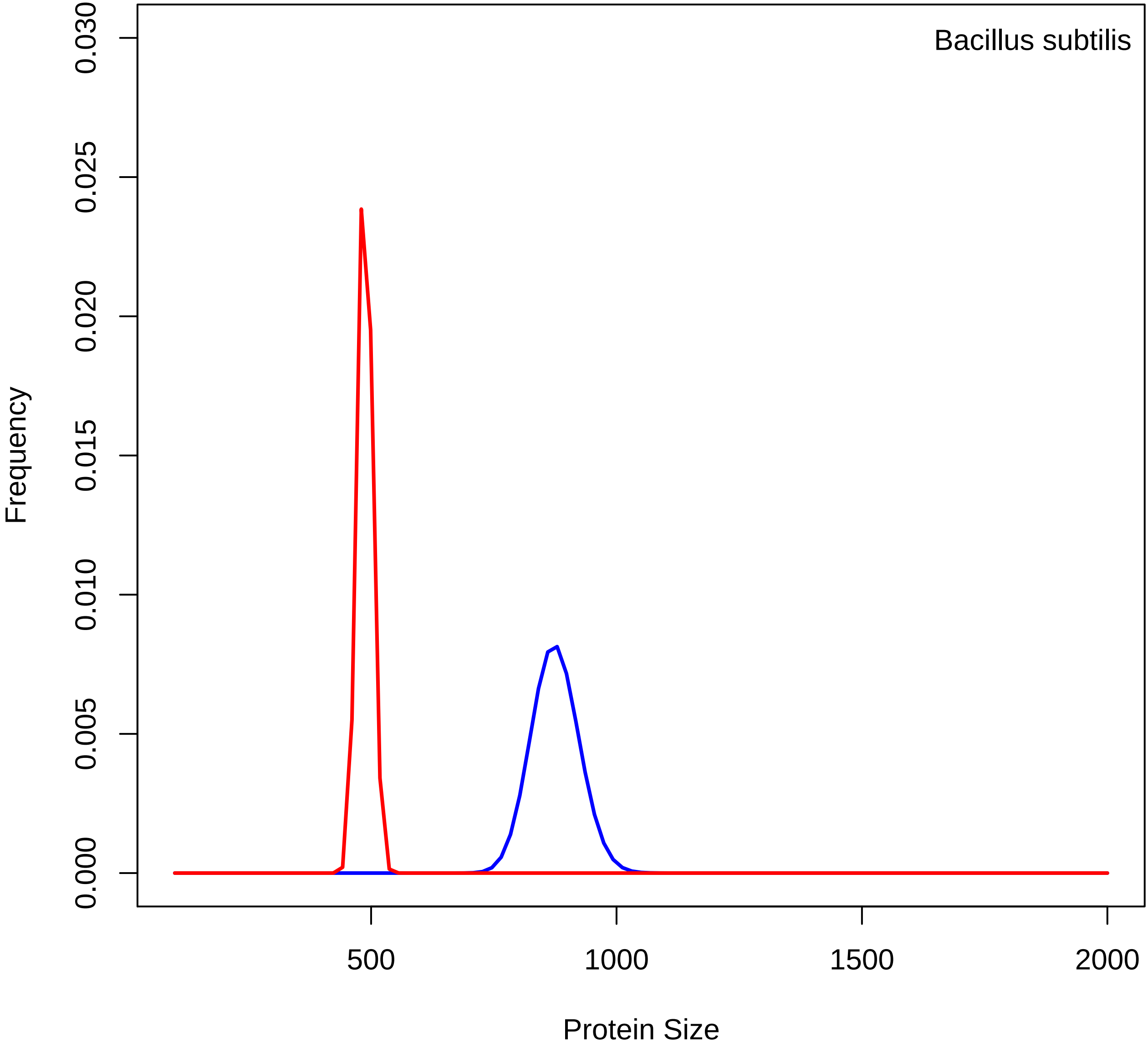

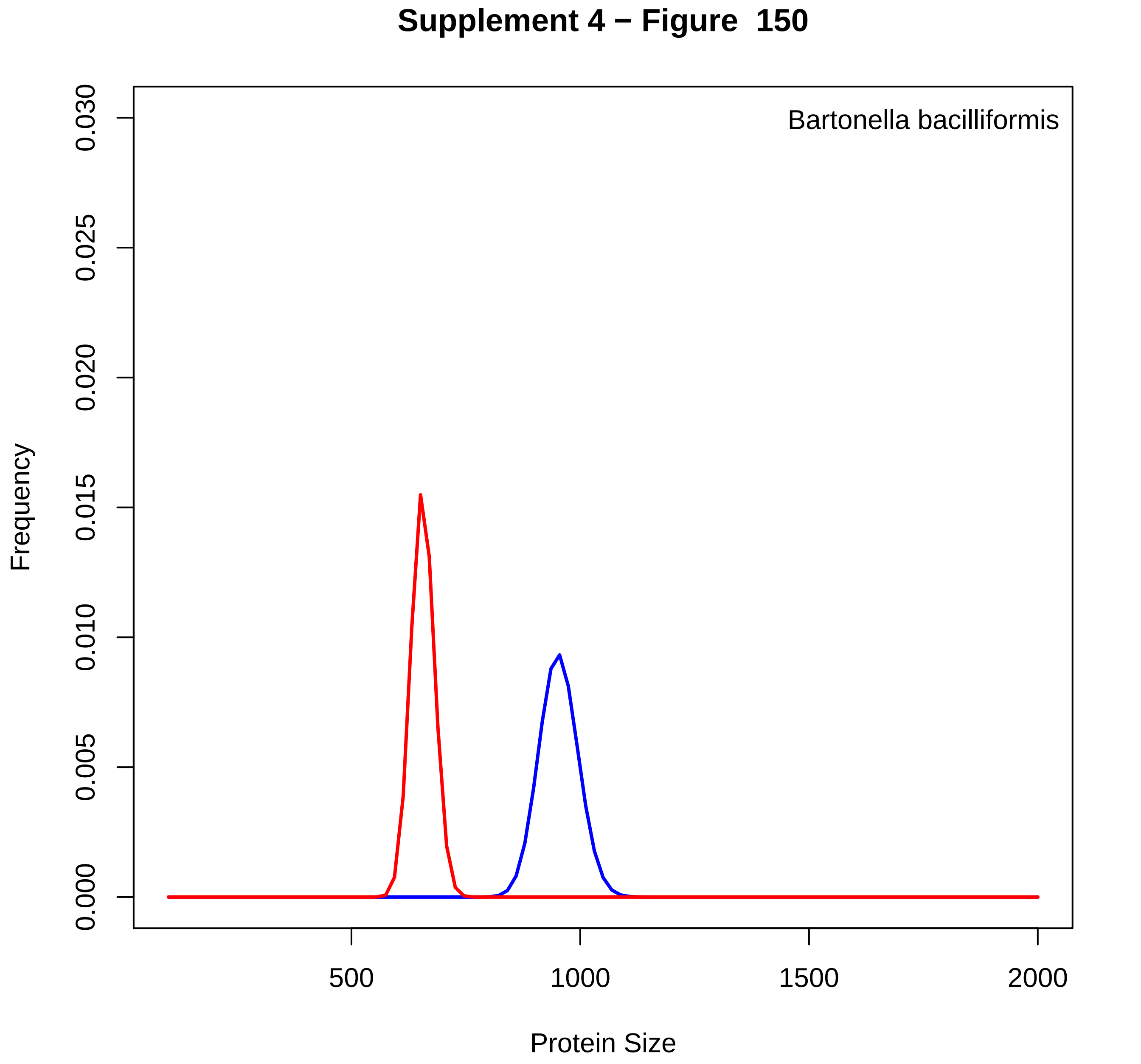
Supplement 4 − Figure 150
Bartonella bacilliformis
Frequency
0.000
0.005
0.010
0.015
0.020
0.025
0.030
500
1000
1500
2000
Protein Size

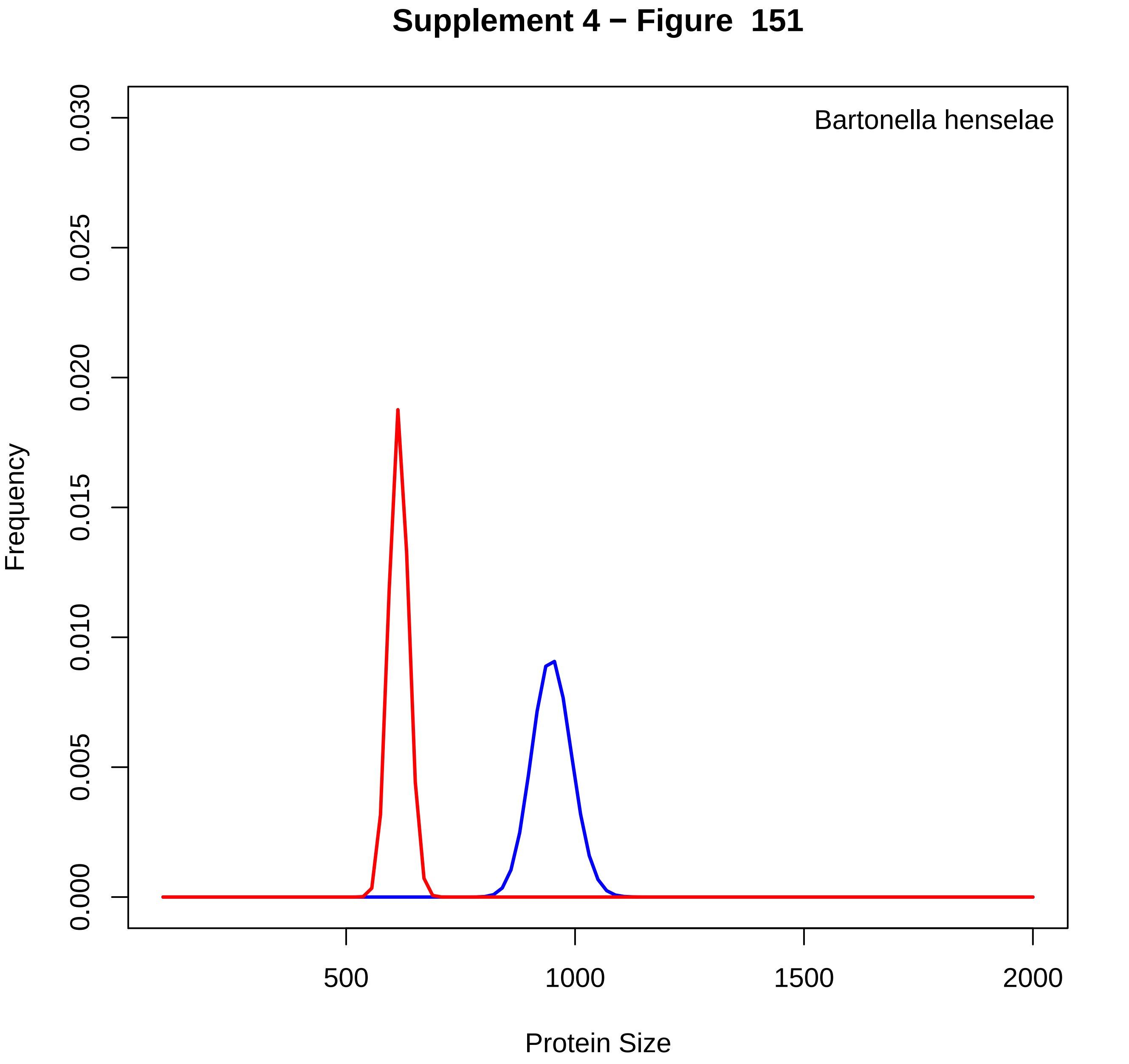
Supplement 4 − Figure 151
Bartonella henselae
Frequency
Protein Size
0.000
0.005
0.010
0.015
0.020
0.025
0.030
500
1000
1500
2000

Supplement 4 − Figure 152

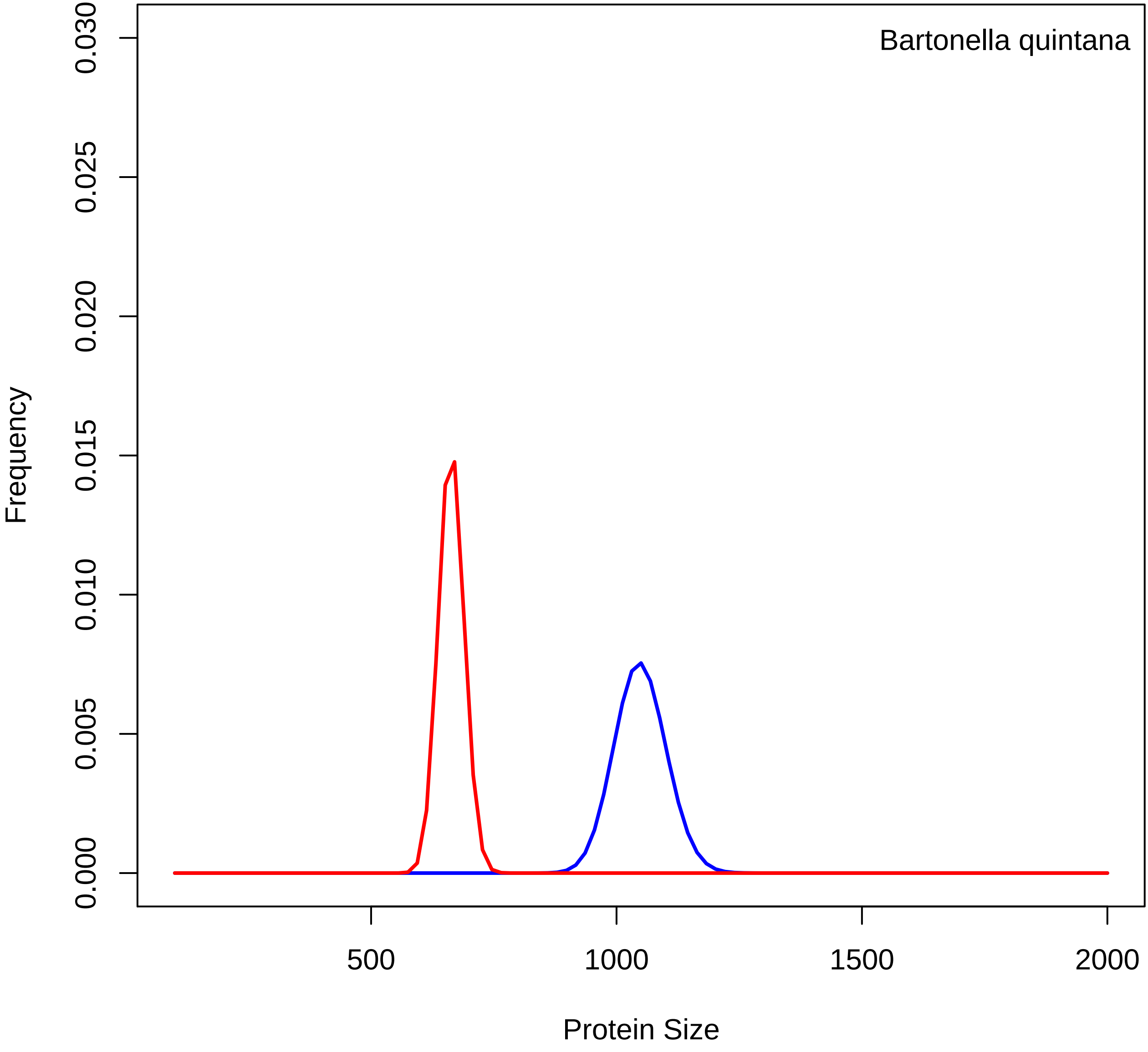

Supplement 4 − Figure 153

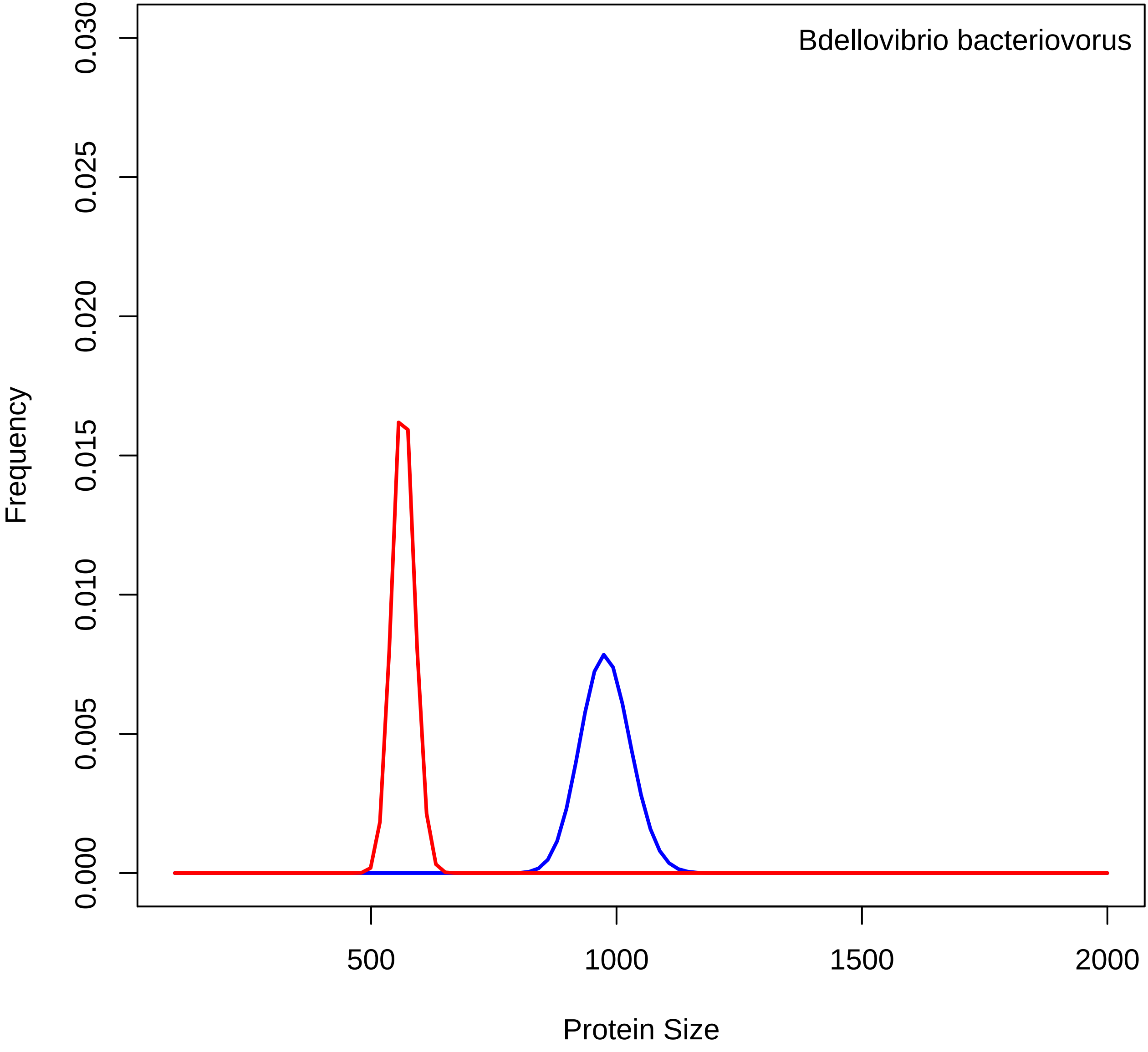

Supplement 4 − Figure 154

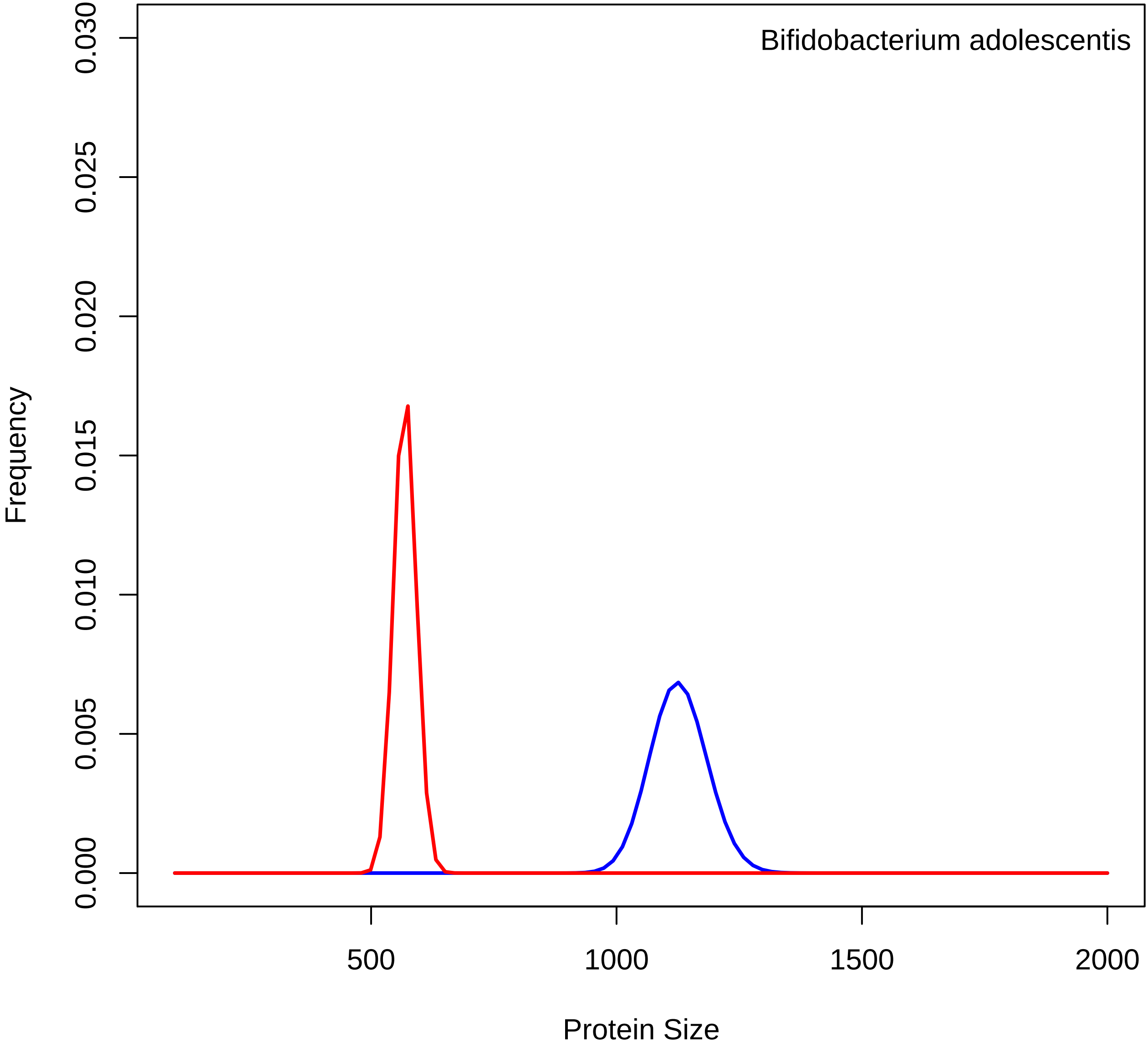

Supplement 4 − Figure 155

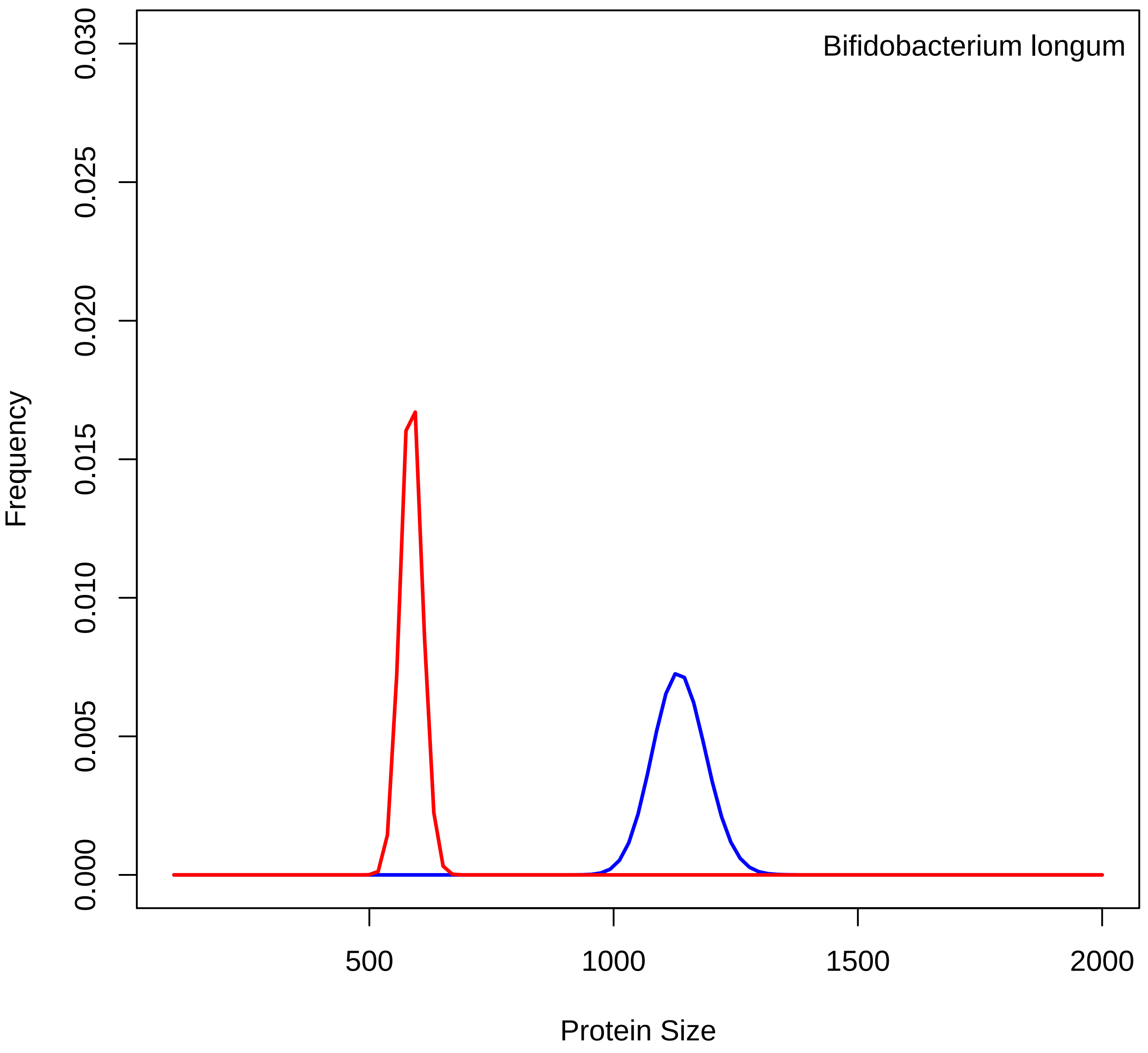

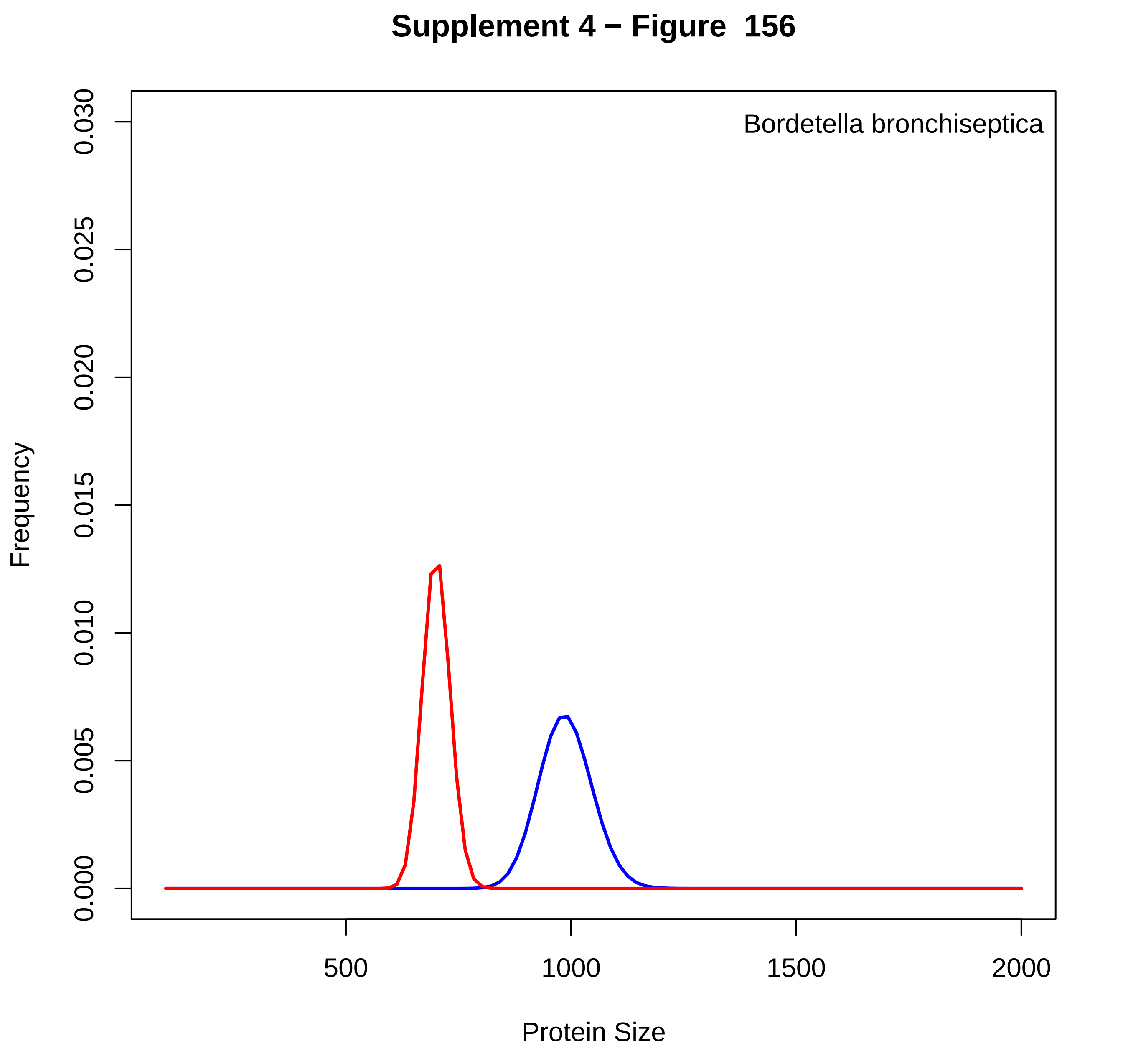
Supplement 4 − Figure 156
Bordetella bronchiseptica
Frequency
0.000
0.005
0.010
0.015
0.020
0.025
0.030
500
1000
1500
2000
Protein Size

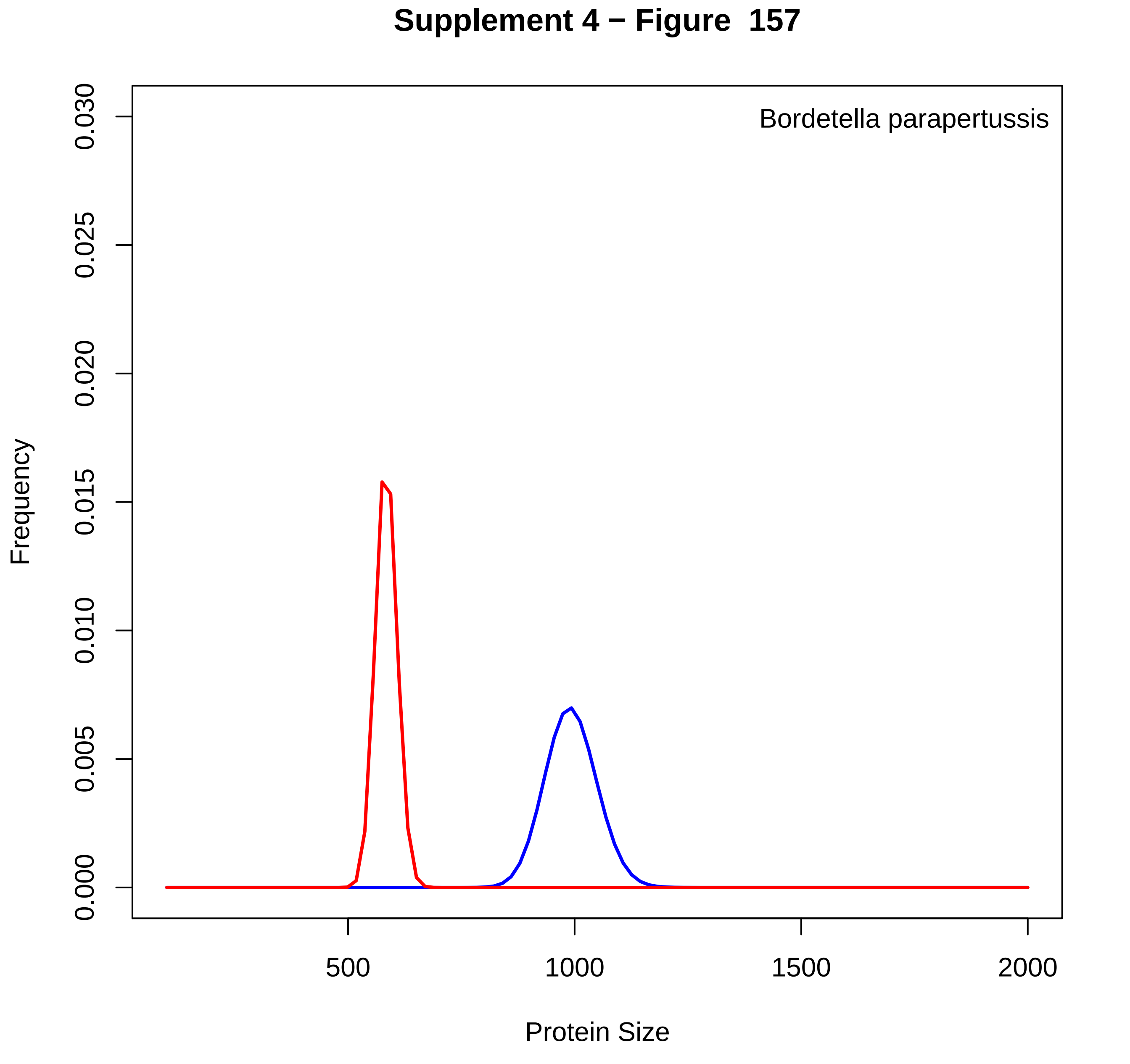

Supplement 4 − Figure 157
Bordetella parapertussis
Frequency
0.000
0.005
0.010
0.015
0.020
0.025
0.030
500
1000
1500
2000
Protein Size

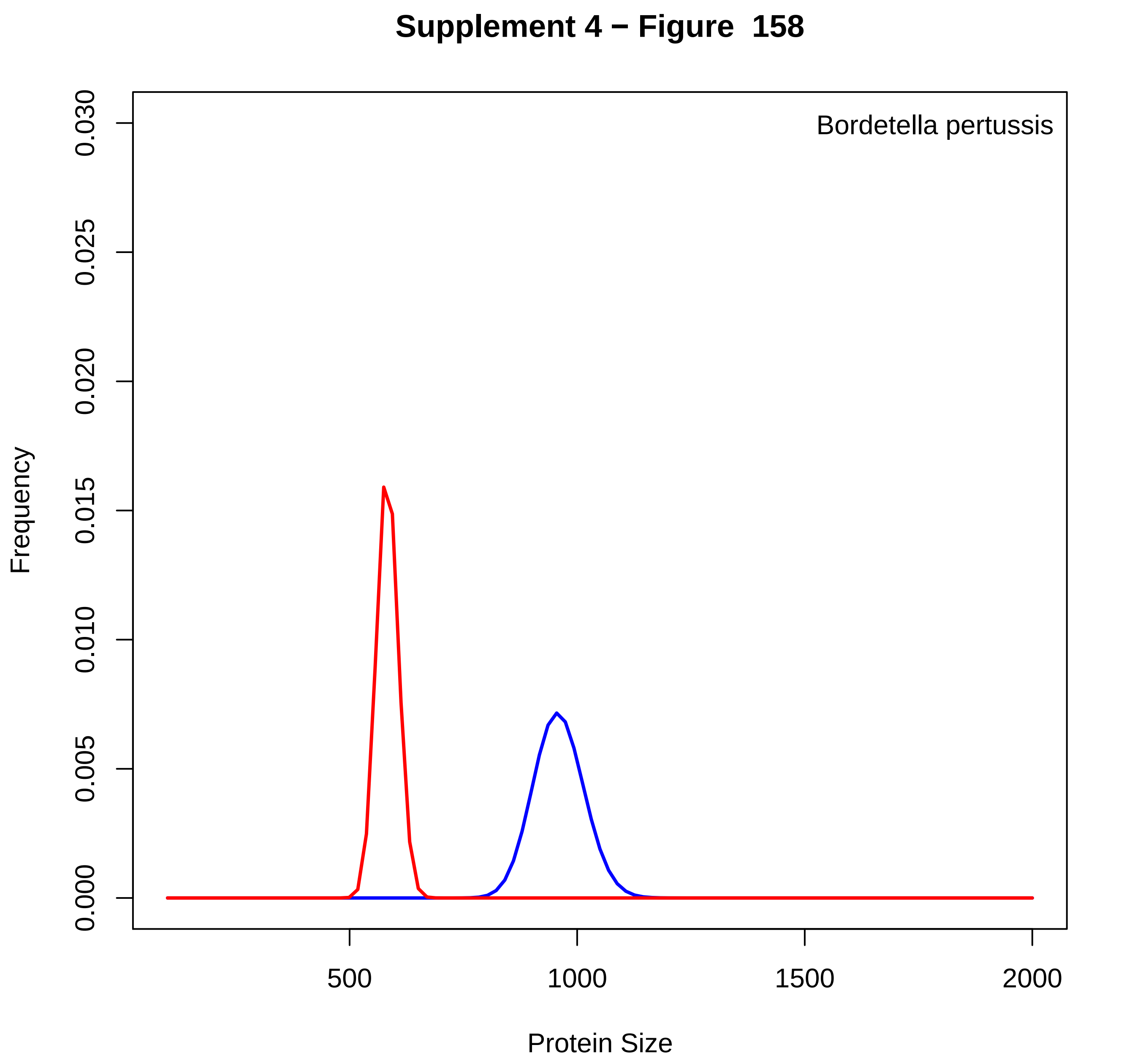
Supplement 4 − Figure 158
Bordetella pertussis
Frequency
0.000
0.005
0.010
0.015
0.020
0.025
0.030
Protein Size
500
1000
1500
2000

Supplement 4 − Figure 159

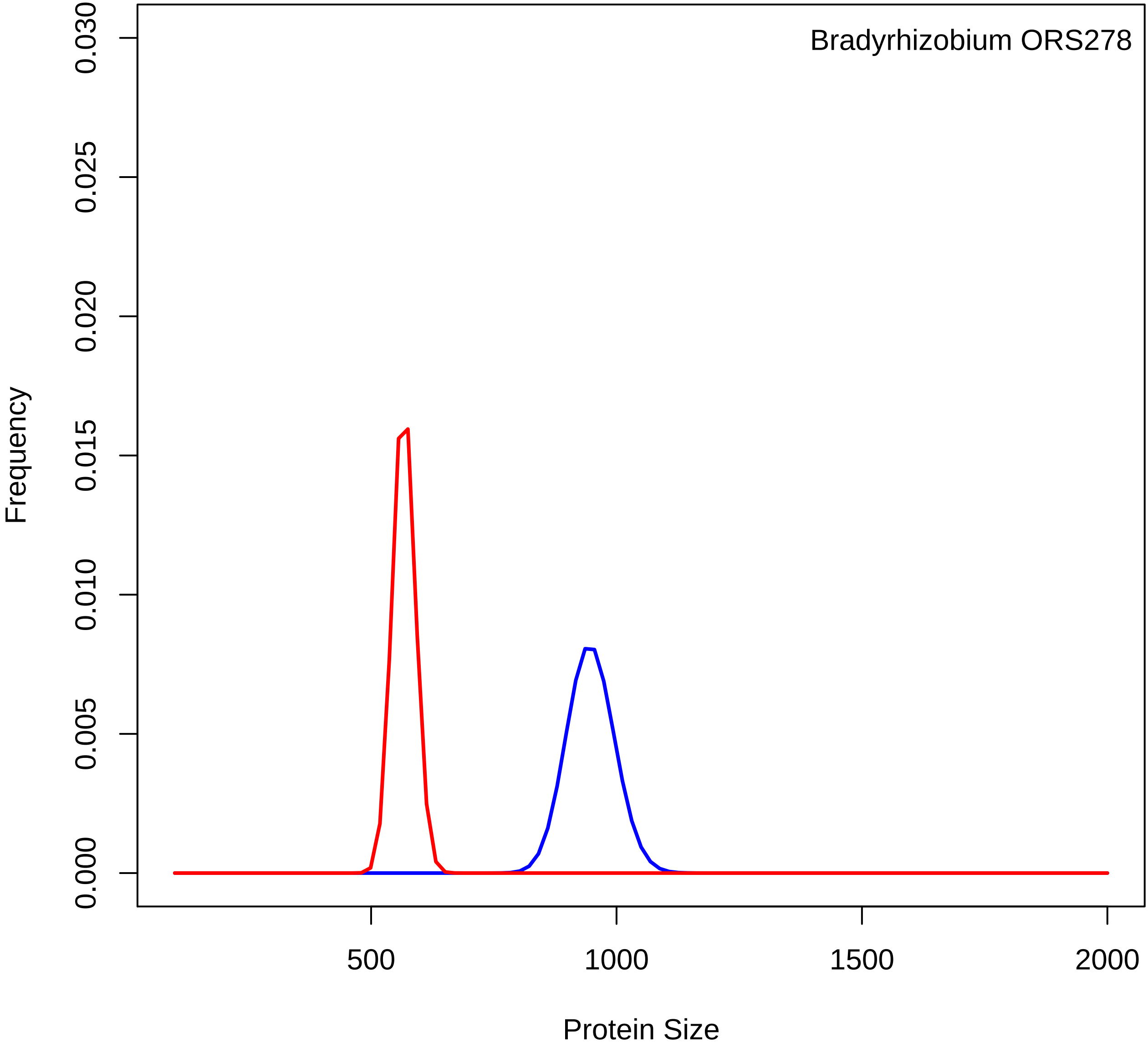

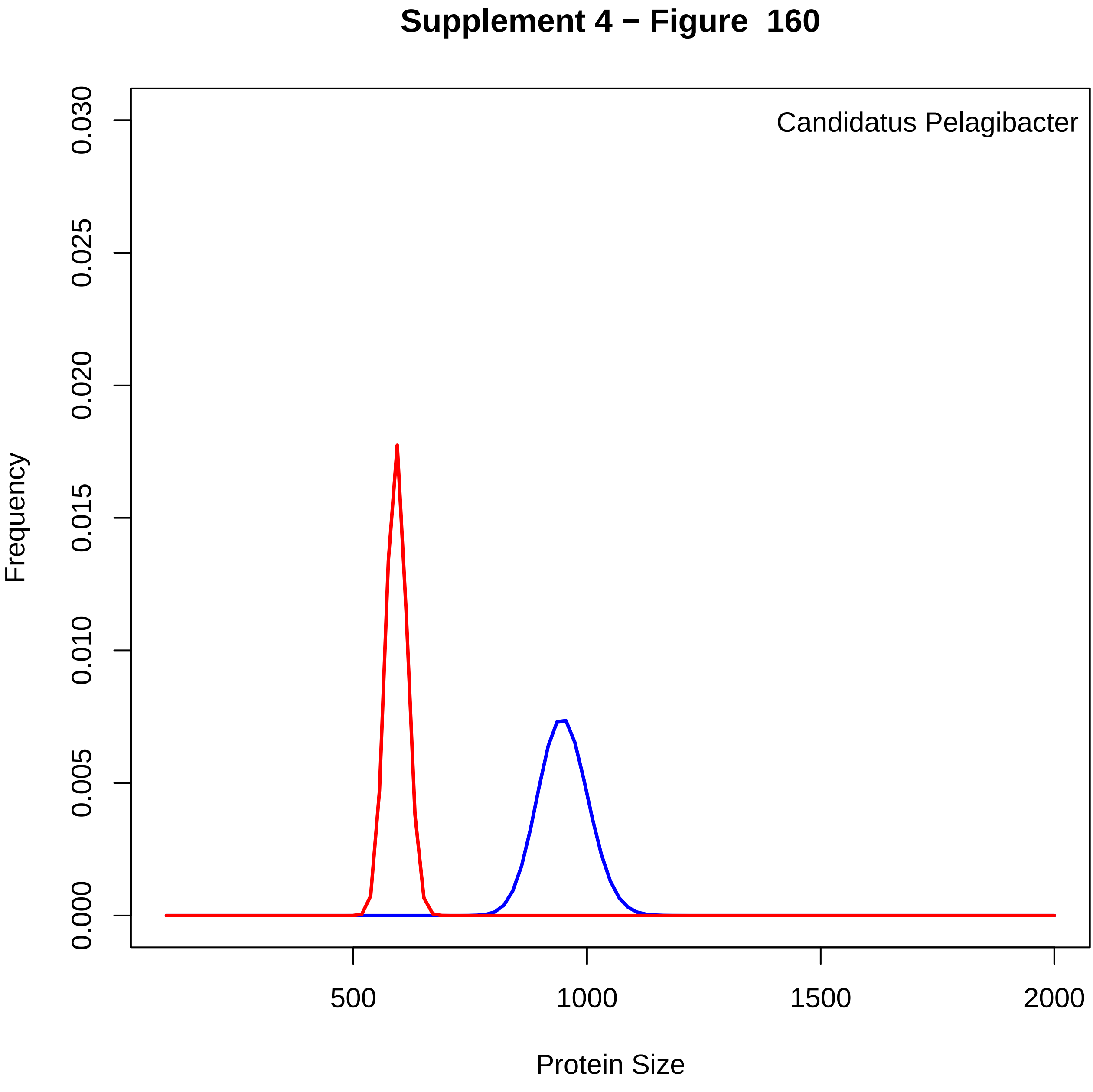
Supplement 4 − Figure 160
Candidatus Pelagibacter
Frequency
0.000
0.005
0.010
0.015
0.020
0.025
0.030
500
1000
1500
2000
Protein Size

Supplement 4 − Figure 161

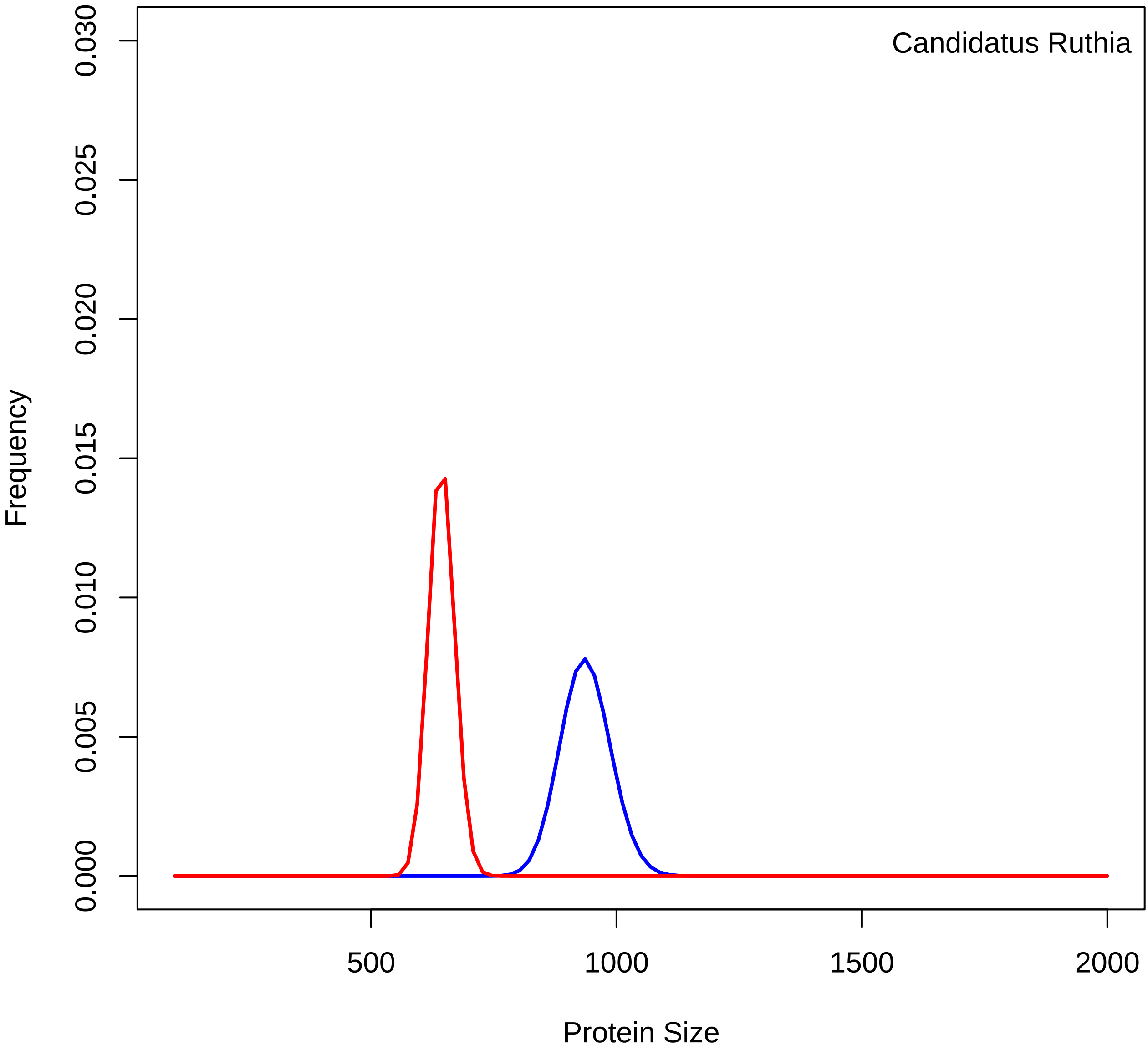

Supplement 4 − Figure 162

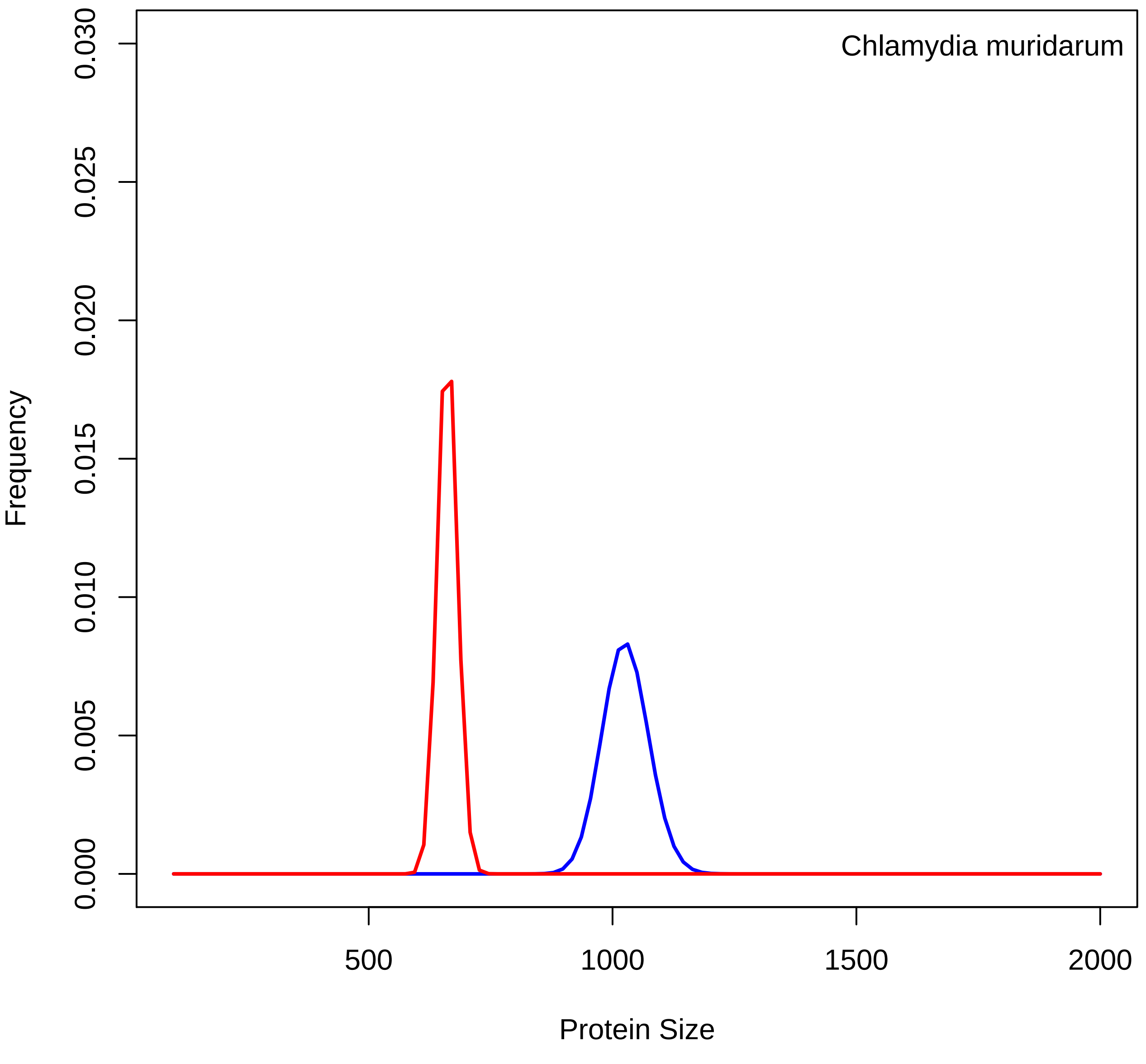

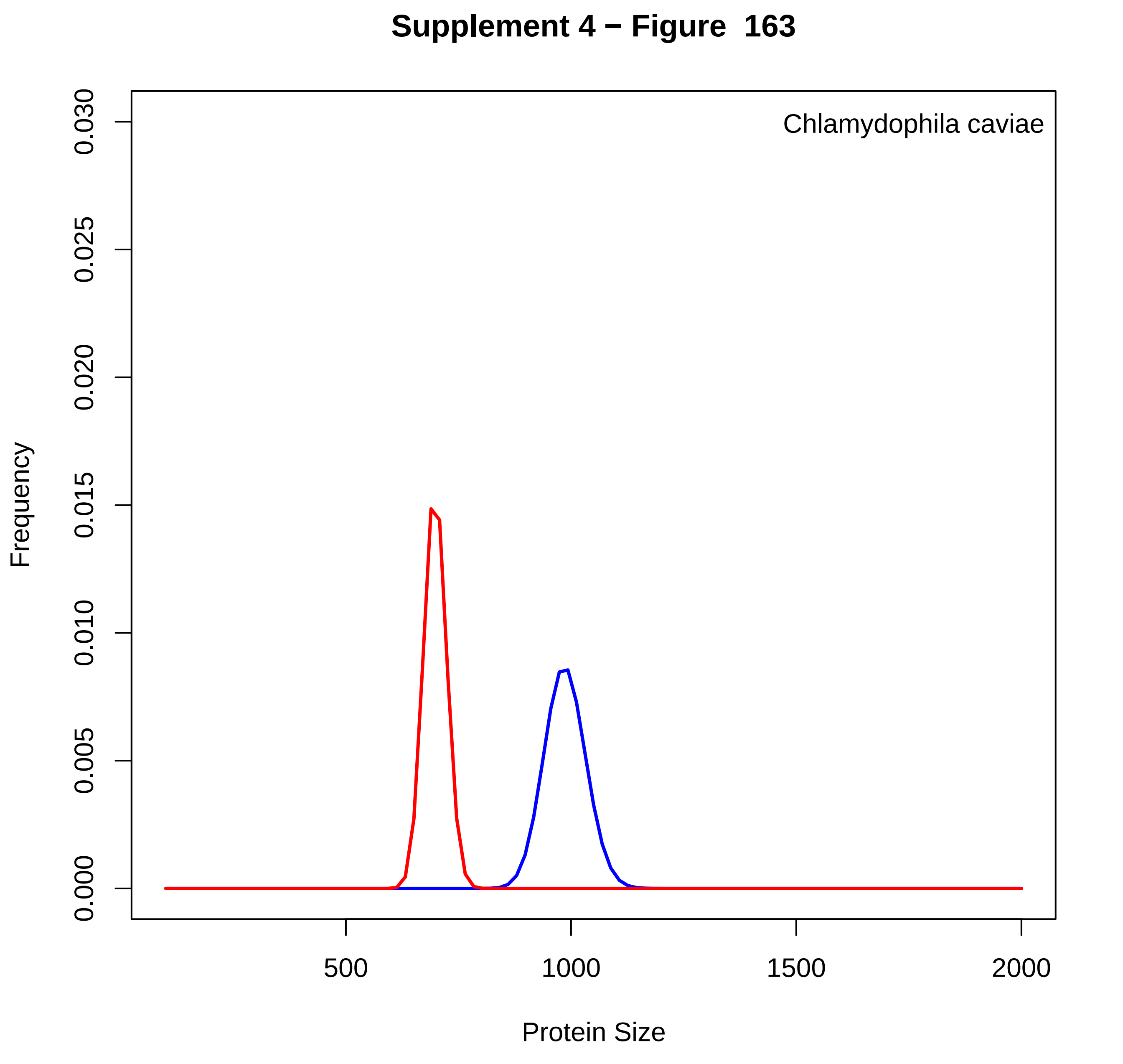
Supplement 4 − Figure 163
Chlamydophila caviae
Frequency
0.000
0.005
0.010
0.015
0.020
0.025
0.030
500
1000
1500
2000
Protein Size

Supplement 4 − Figure 164

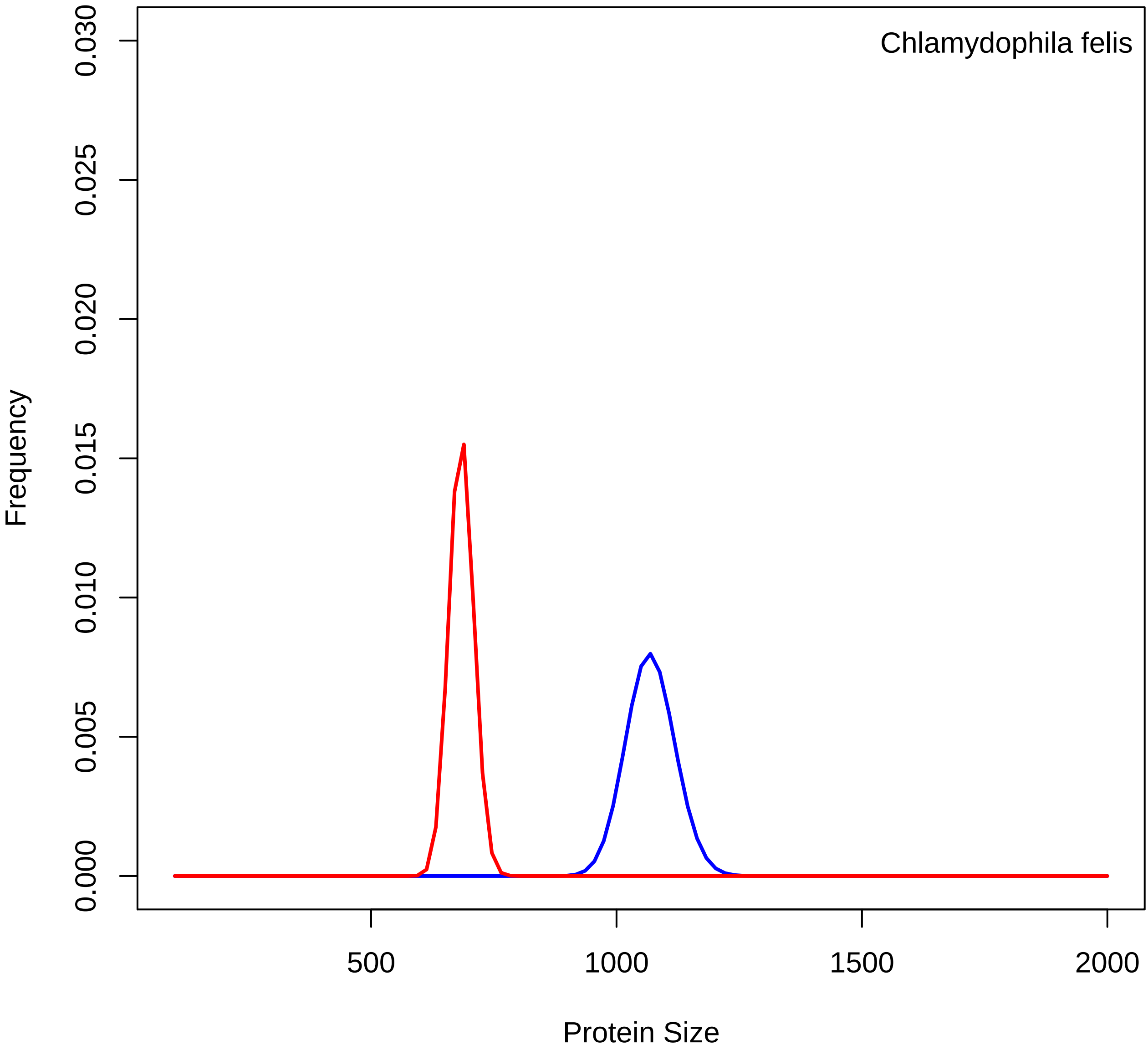

Supplement 4 − Figure 165

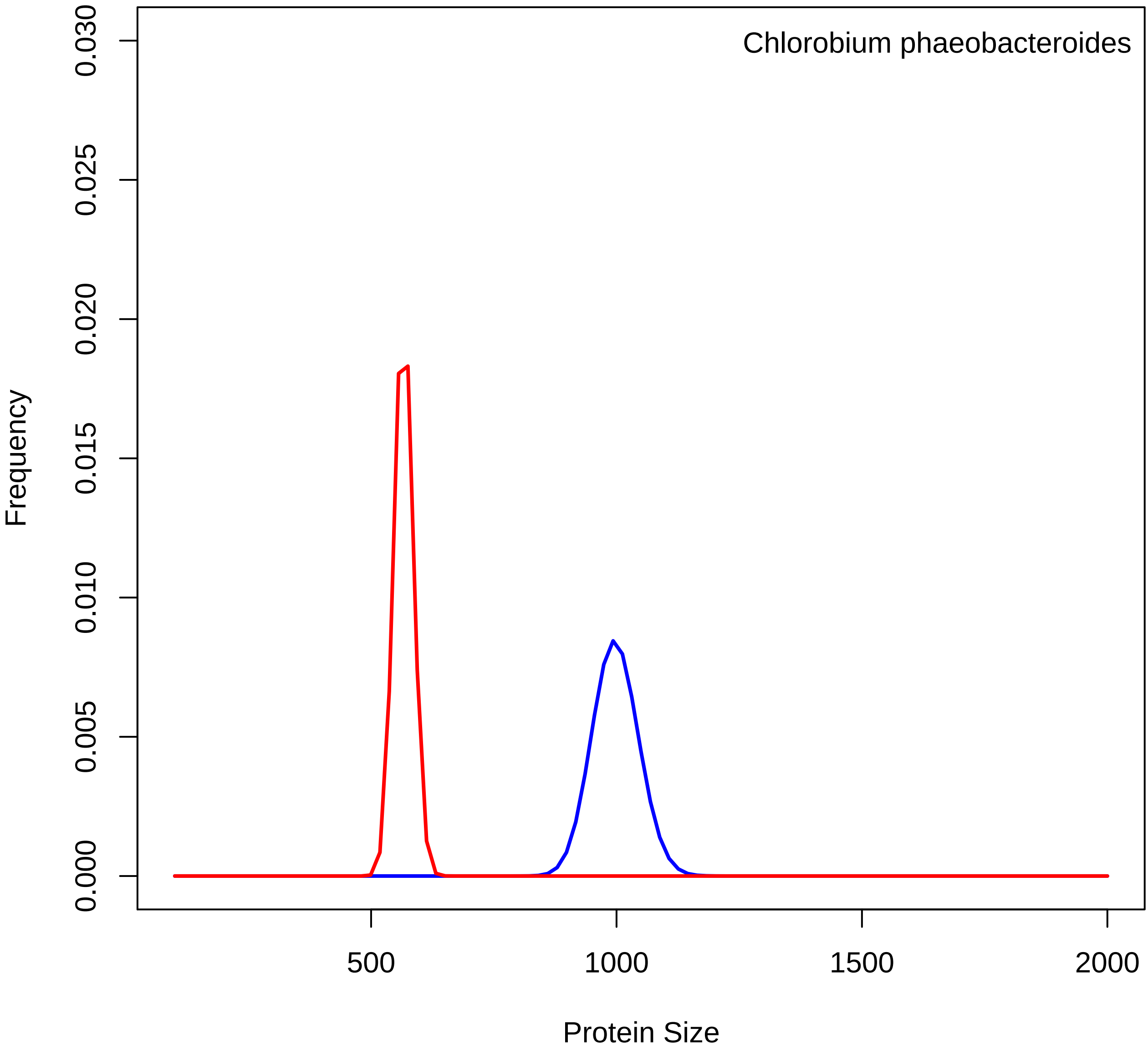

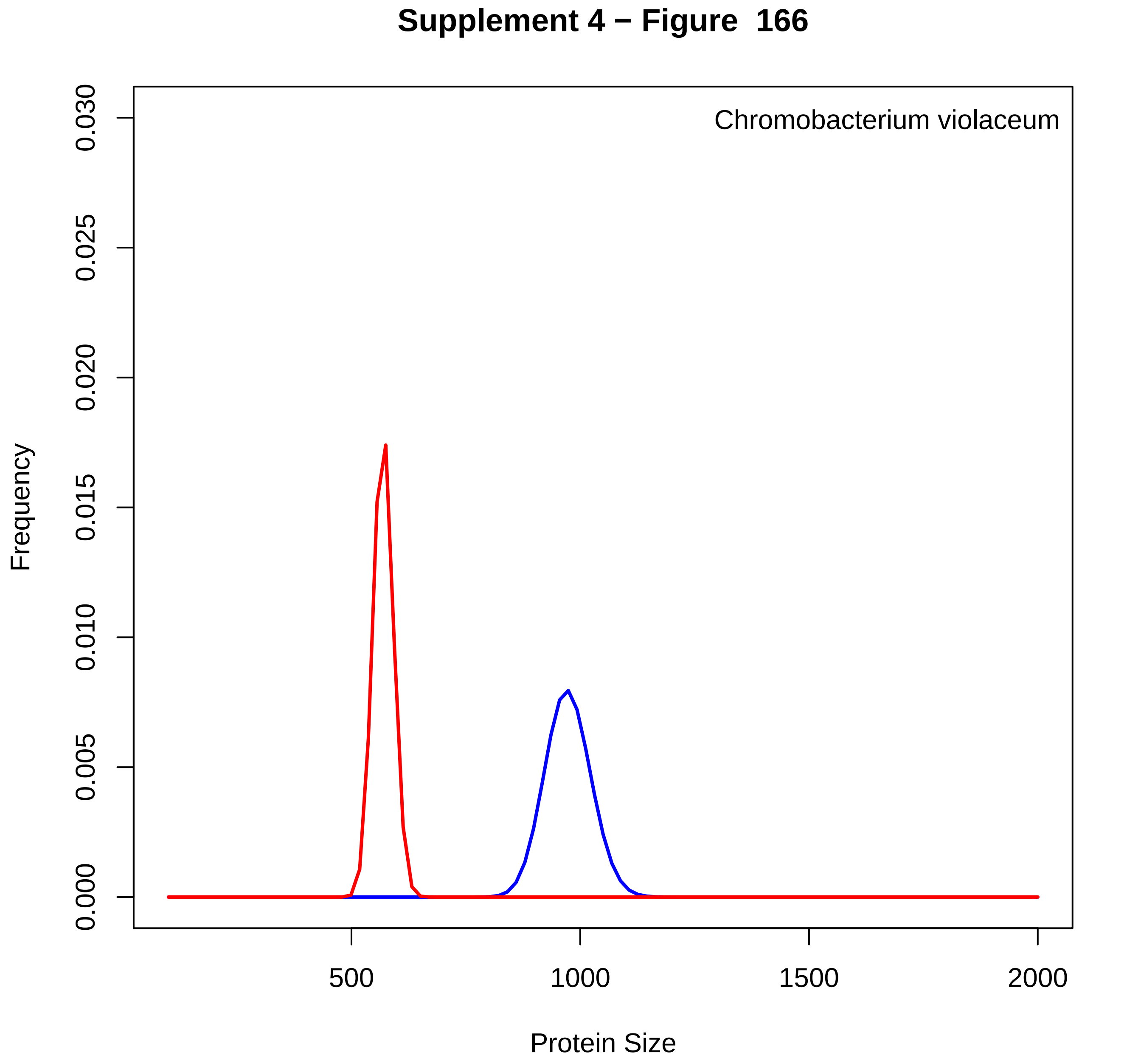
Supplement 4 − Figure 166
Chromobacterium violaceum
Frequency
0.000
0.005
0.010
0.015
0.020
0.025
0.030
500
1000
1500
2000
Protein Size

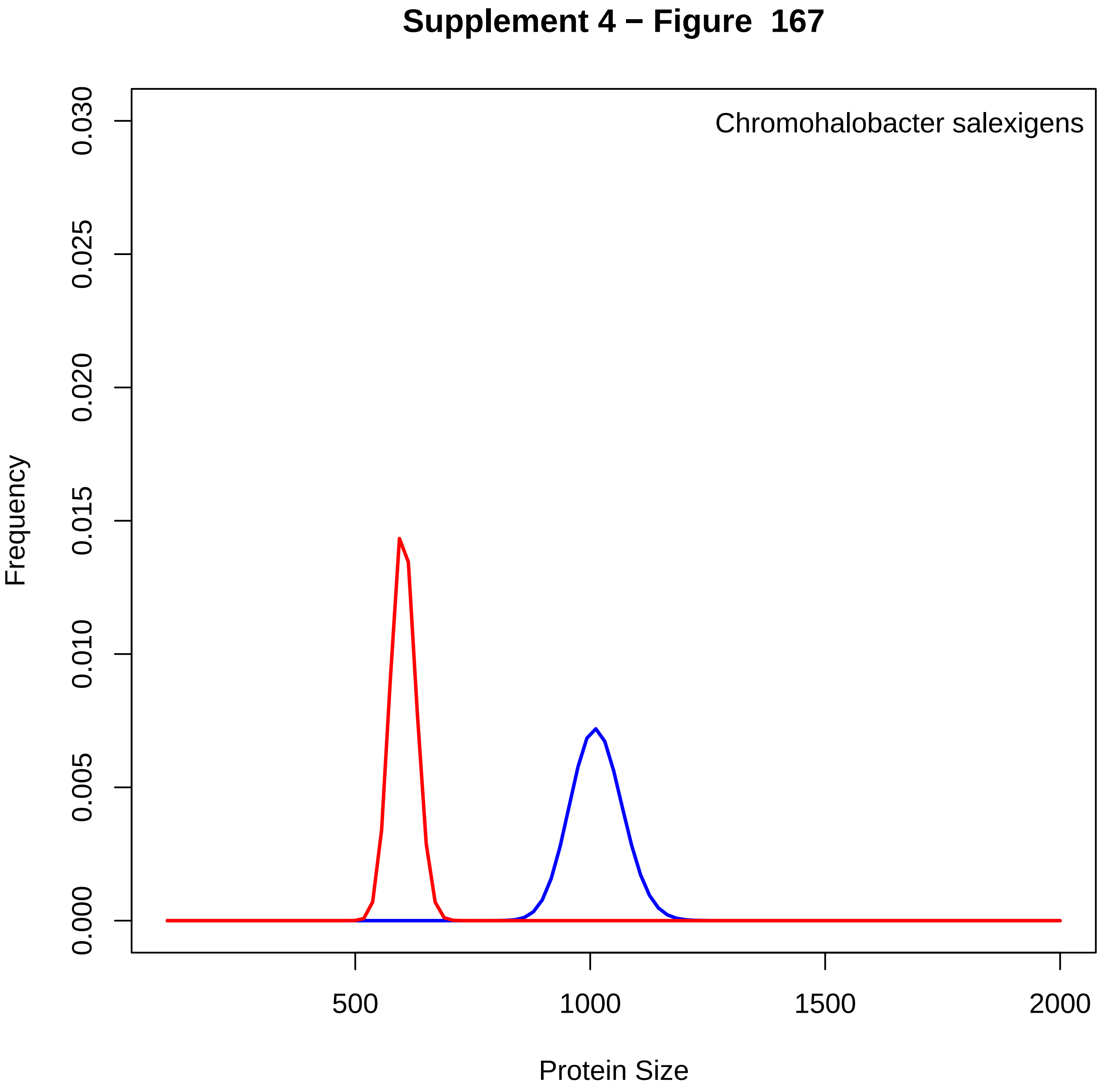
Supplement 4 − Figure 167
Chromohalobacter salexigens
Frequency
0.000
0.005
0.010
0.015
0.020
0.025
0.030
500
1000
1500
2000
Protein Size

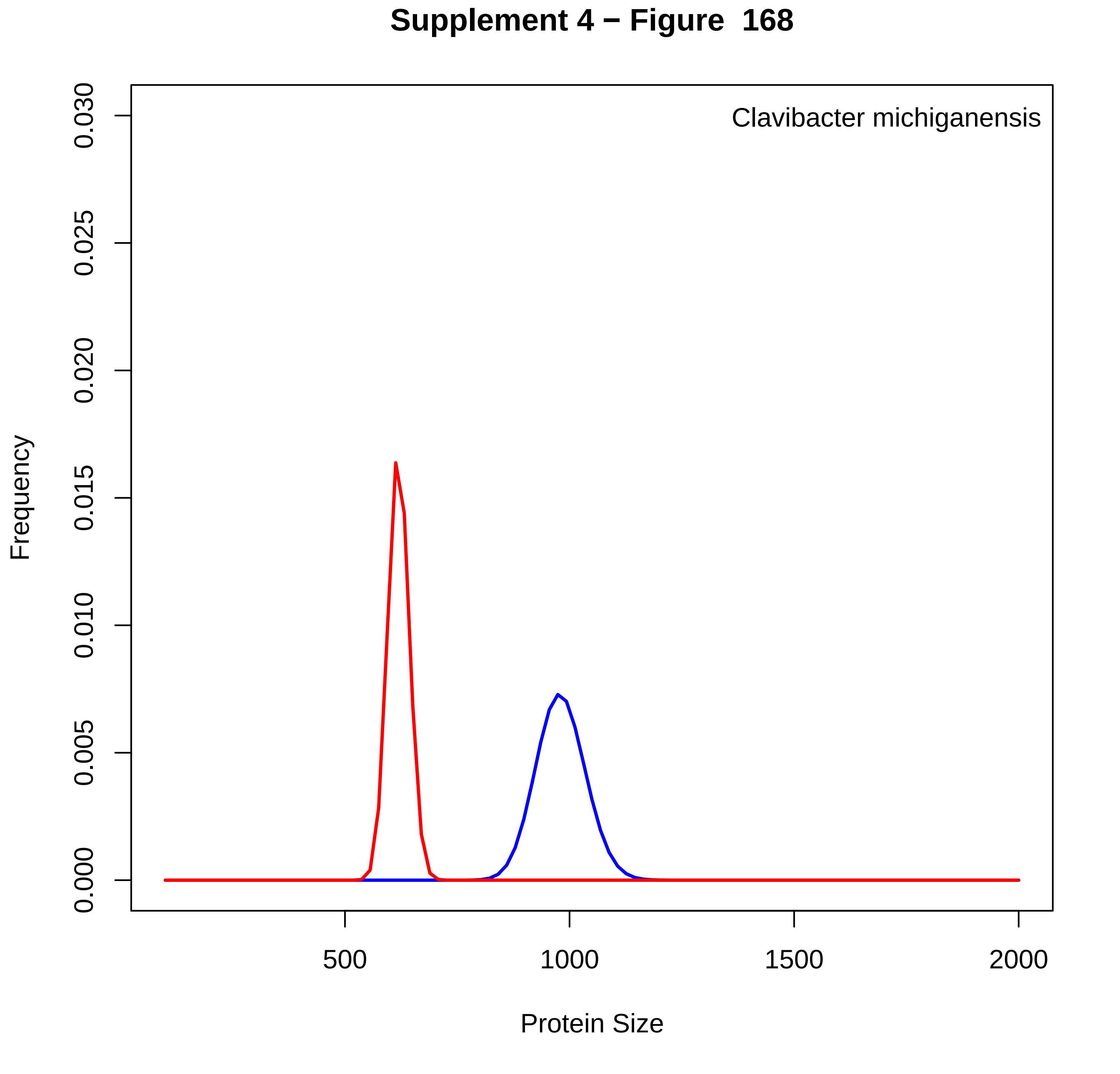

Supplement 4 − Figure 168
Clavibacter michiganensis
Frequency
0.000
0.005
0.010
0.015
0.020
0.025
0.030
500
1000
1500
2000
Protein Size

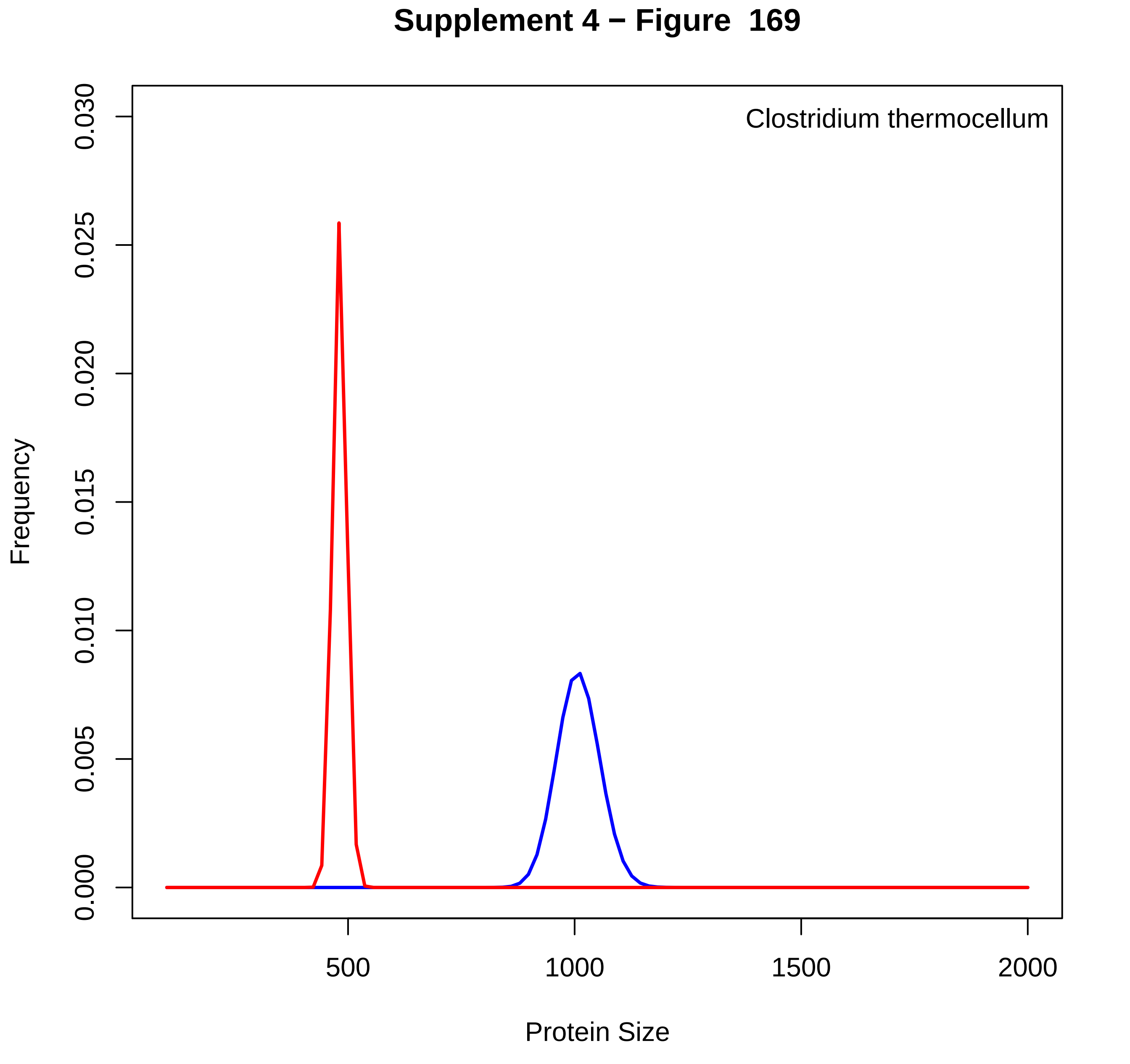

Supplement 4 − Figure 169
Clostridium thermocellum
Frequency
0.000
0.005
0.010
0.015
0.020
0.025
0.030
500
1000
1500
2000
Protein Size

Supplement 4 − Figure 170

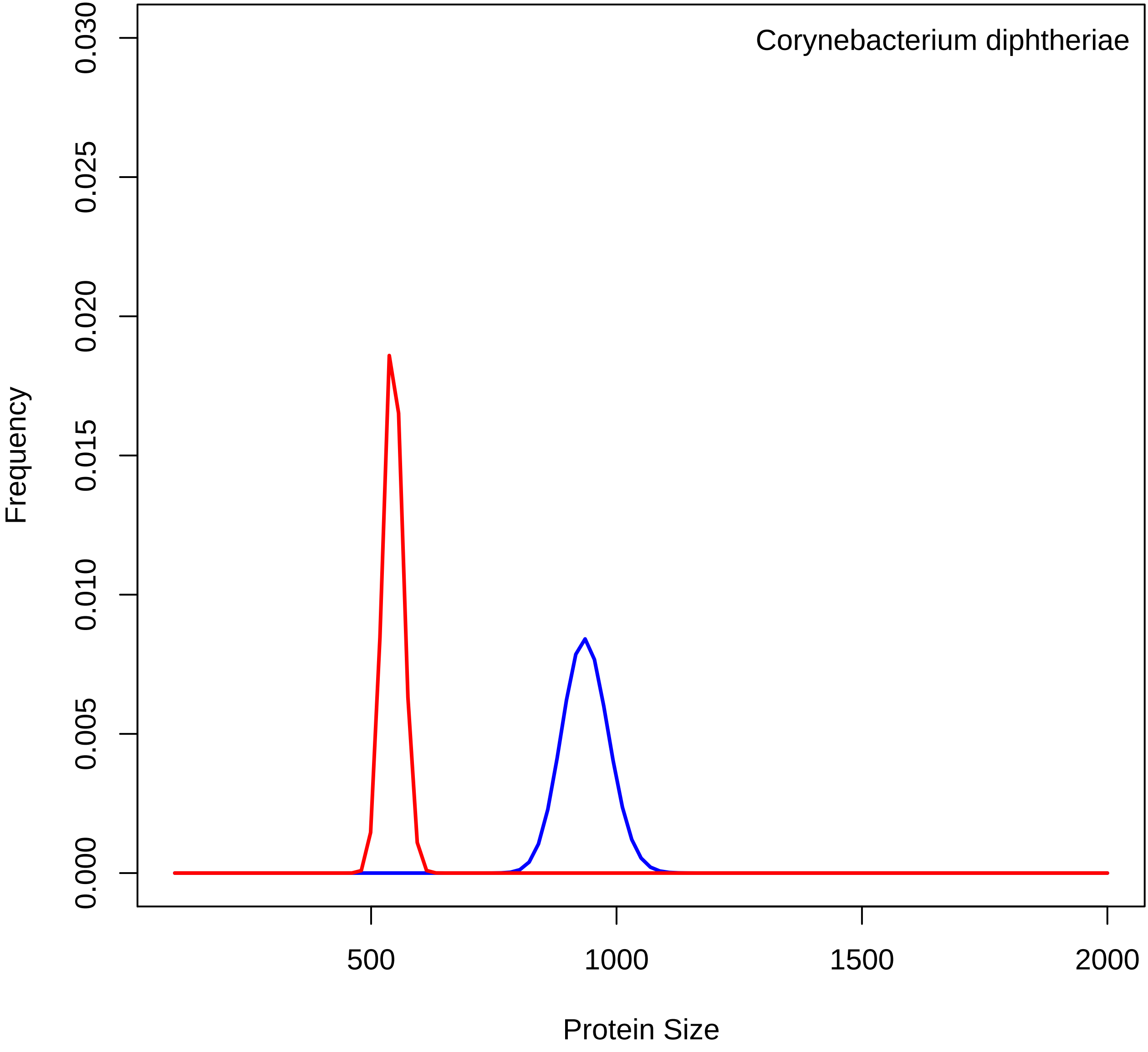

Supplement 4 − Figure 171

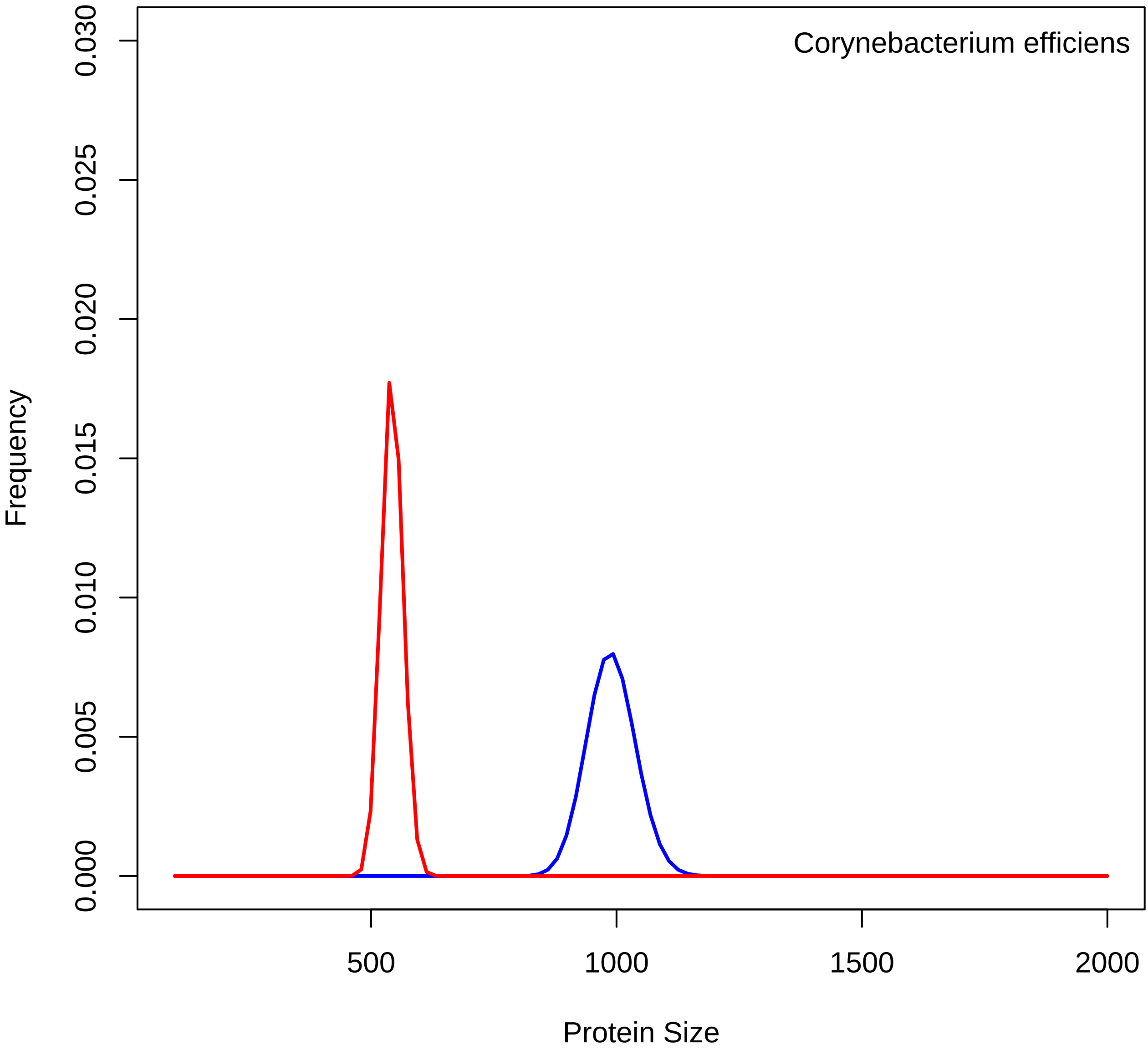

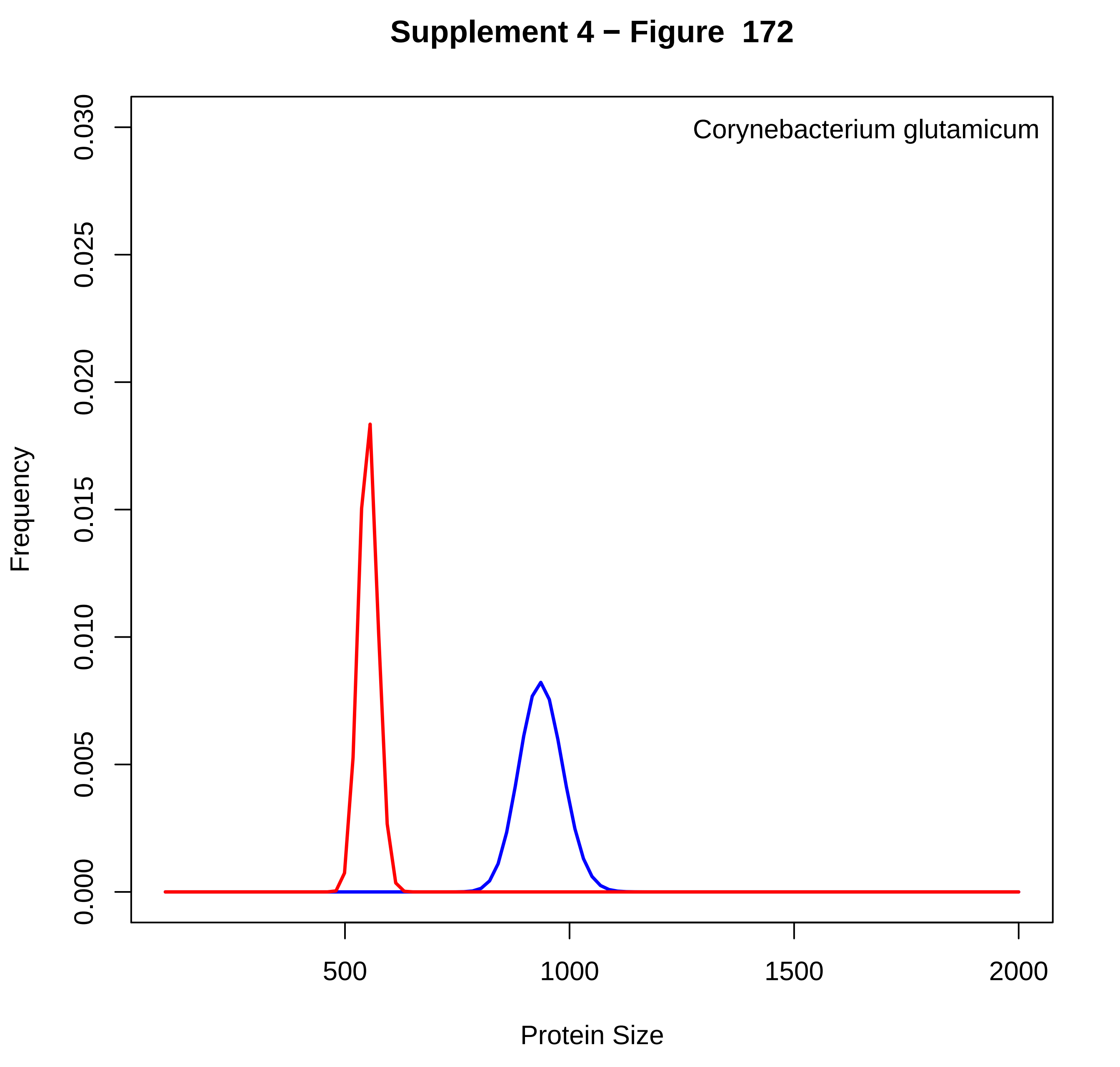

Supplement 4 − Figure 172
Corynebacterium glutamicum
Frequency
0.000
0.005
0.010
0.015
0.020
0.025
0.030
500
1000
1500
2000
Protein Size

Supplement 4 − Figure 173

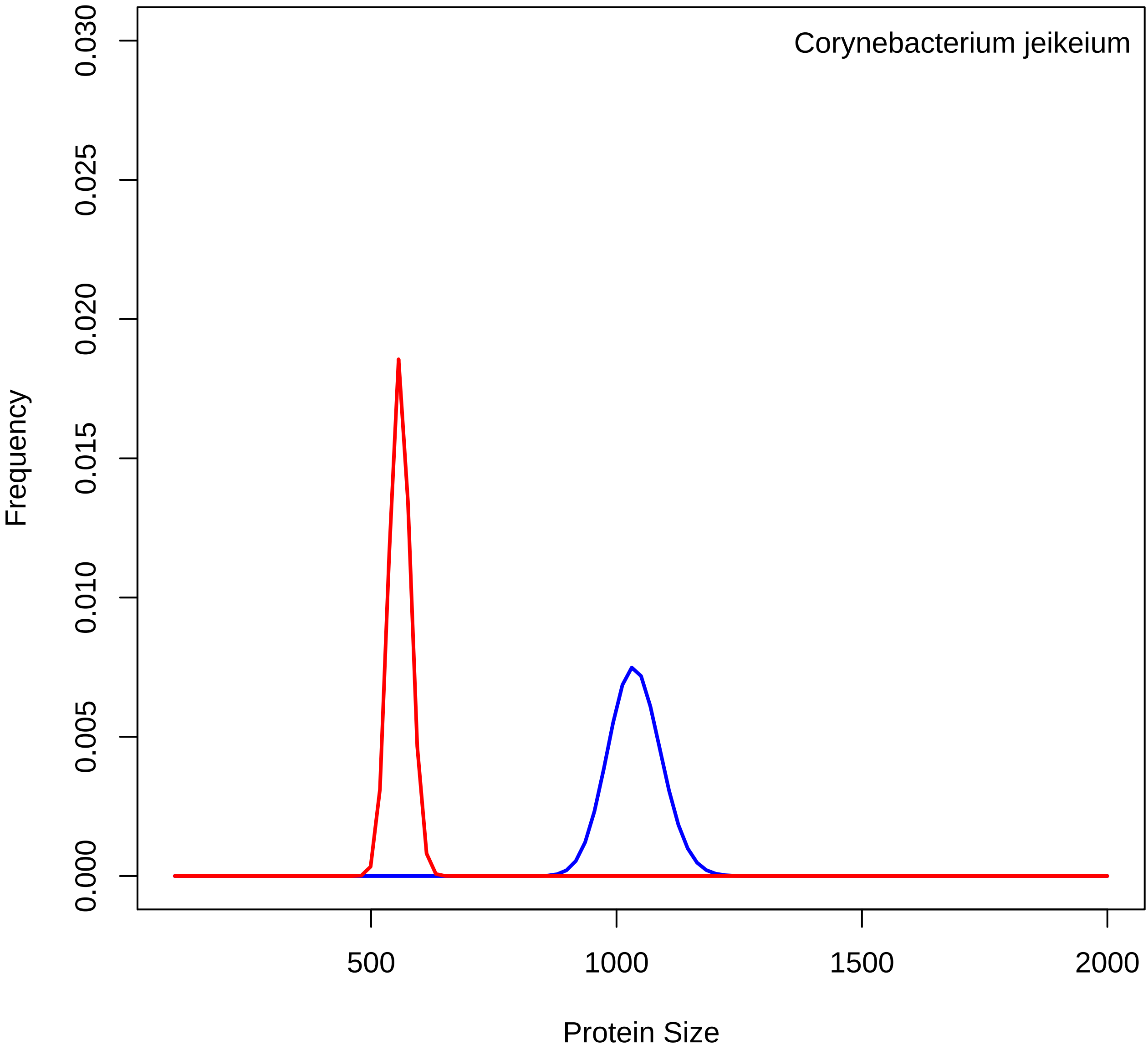

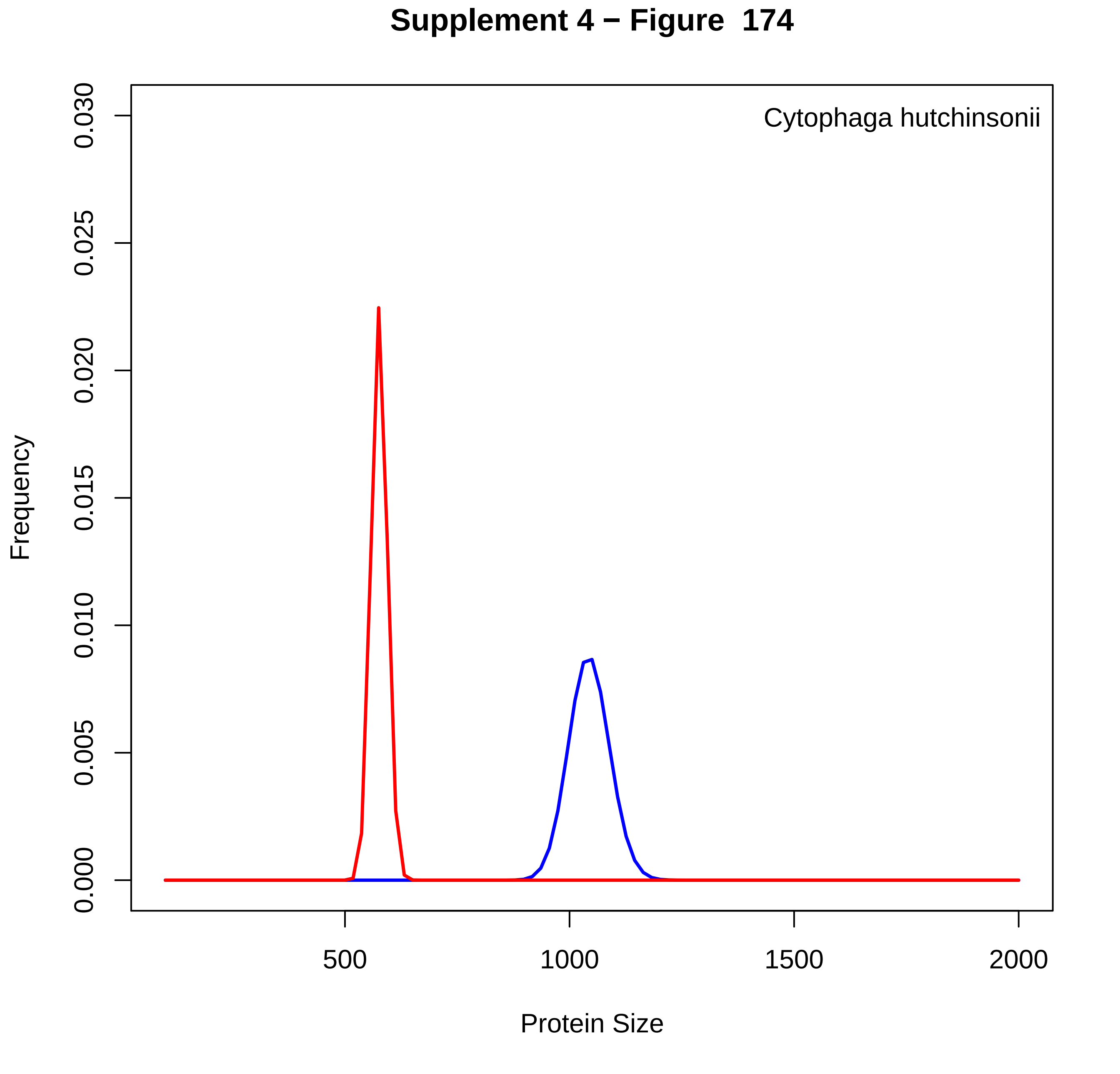
Supplement 4 − Figure 174
Cytophaga hutchinsonii
Frequency
0.000
0.005
0.010
0.015
0.020
0.025
0.030
500
1000
1500
2000
Protein Size

Supplement 4 − Figure 175

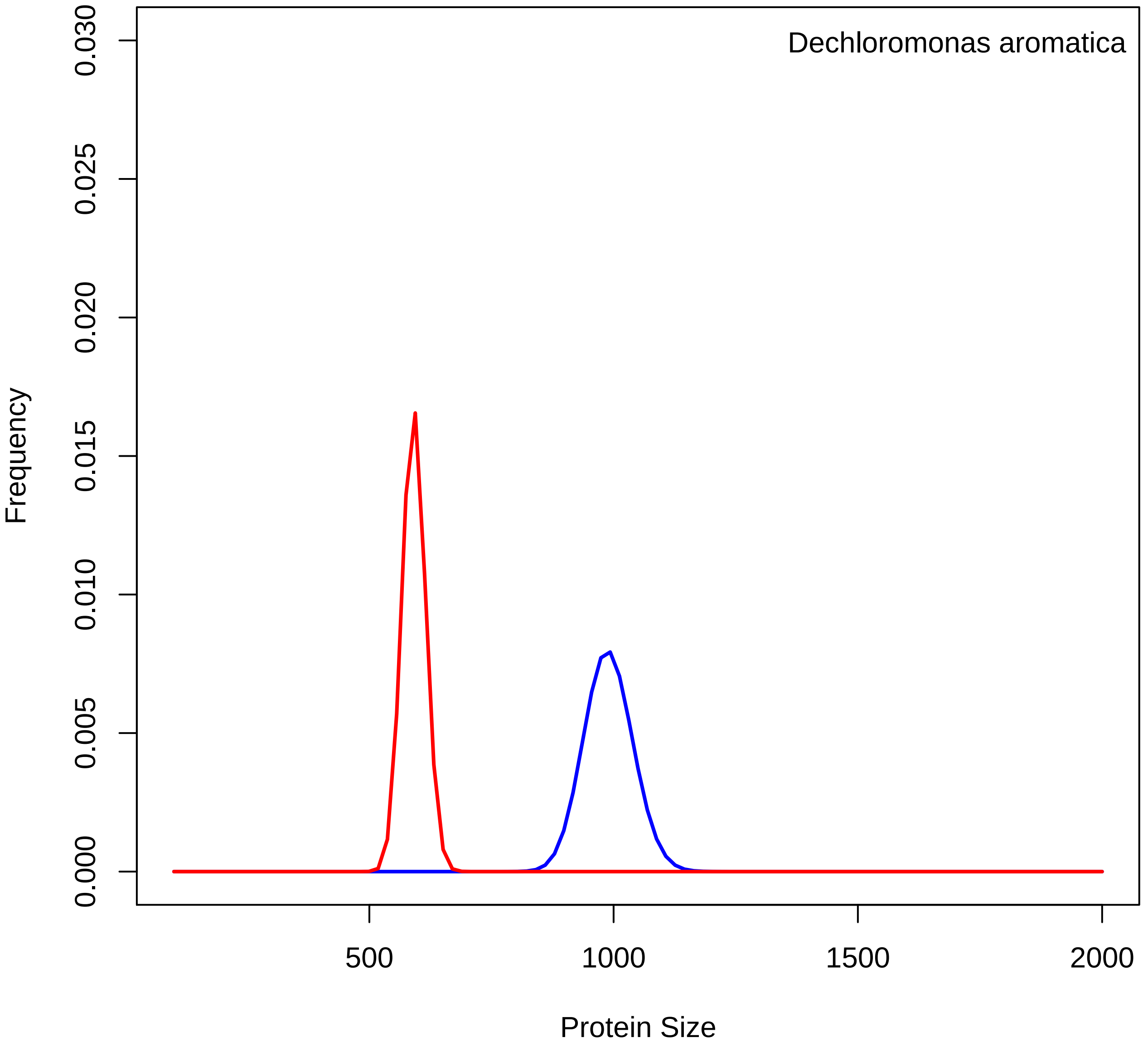

Supplement 4 − Figure 176

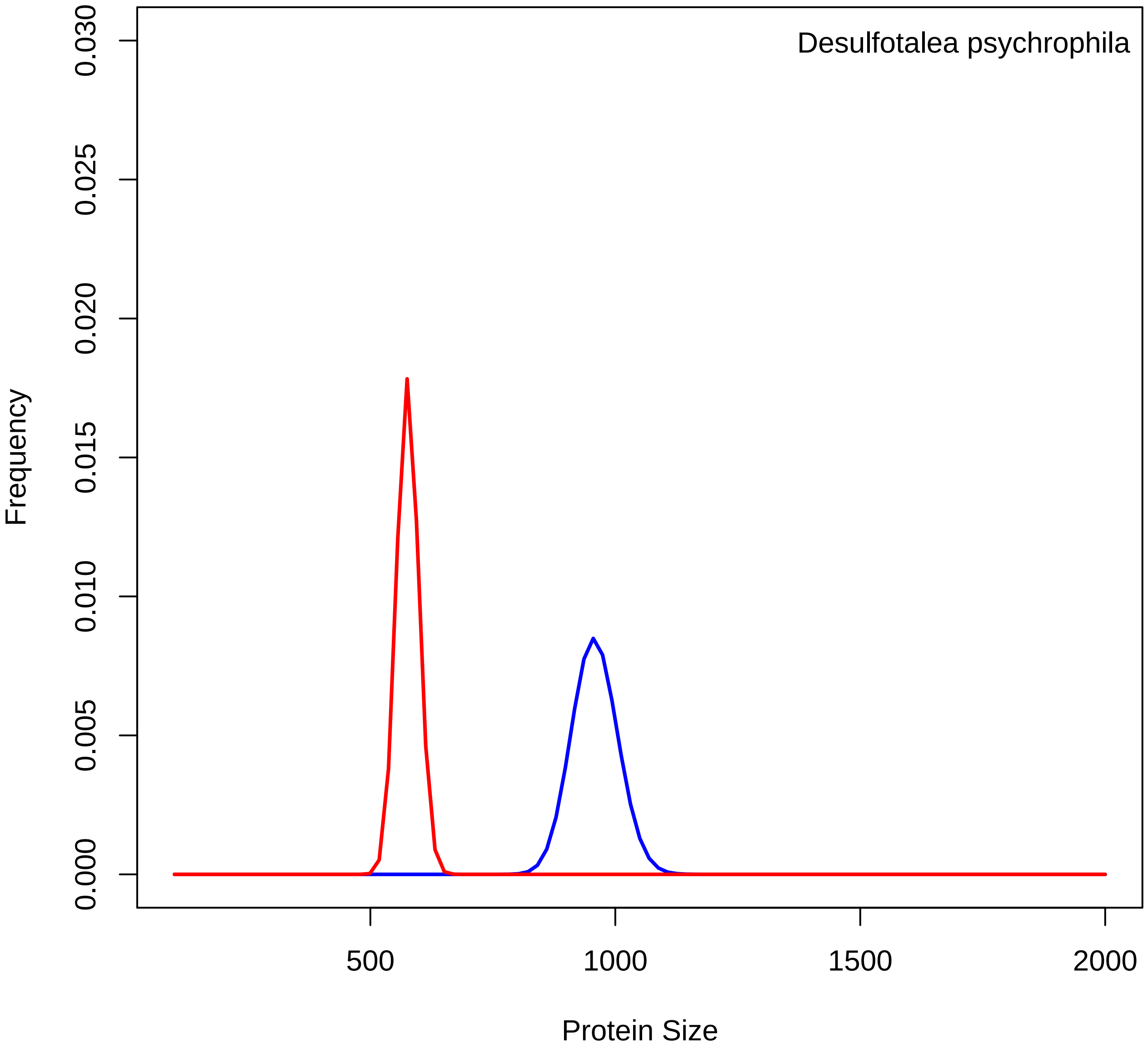

Supplement 4 − Figure 177

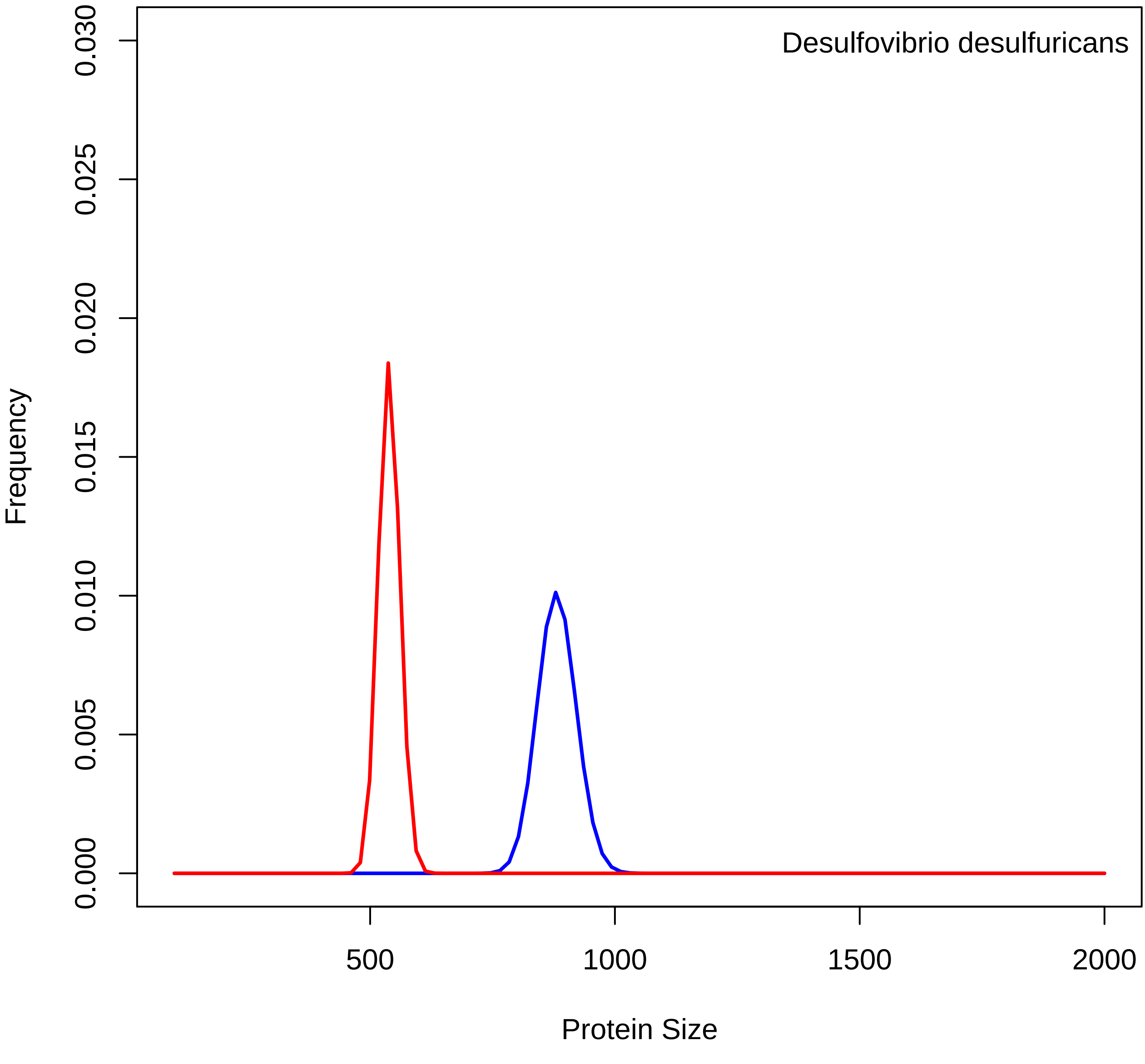

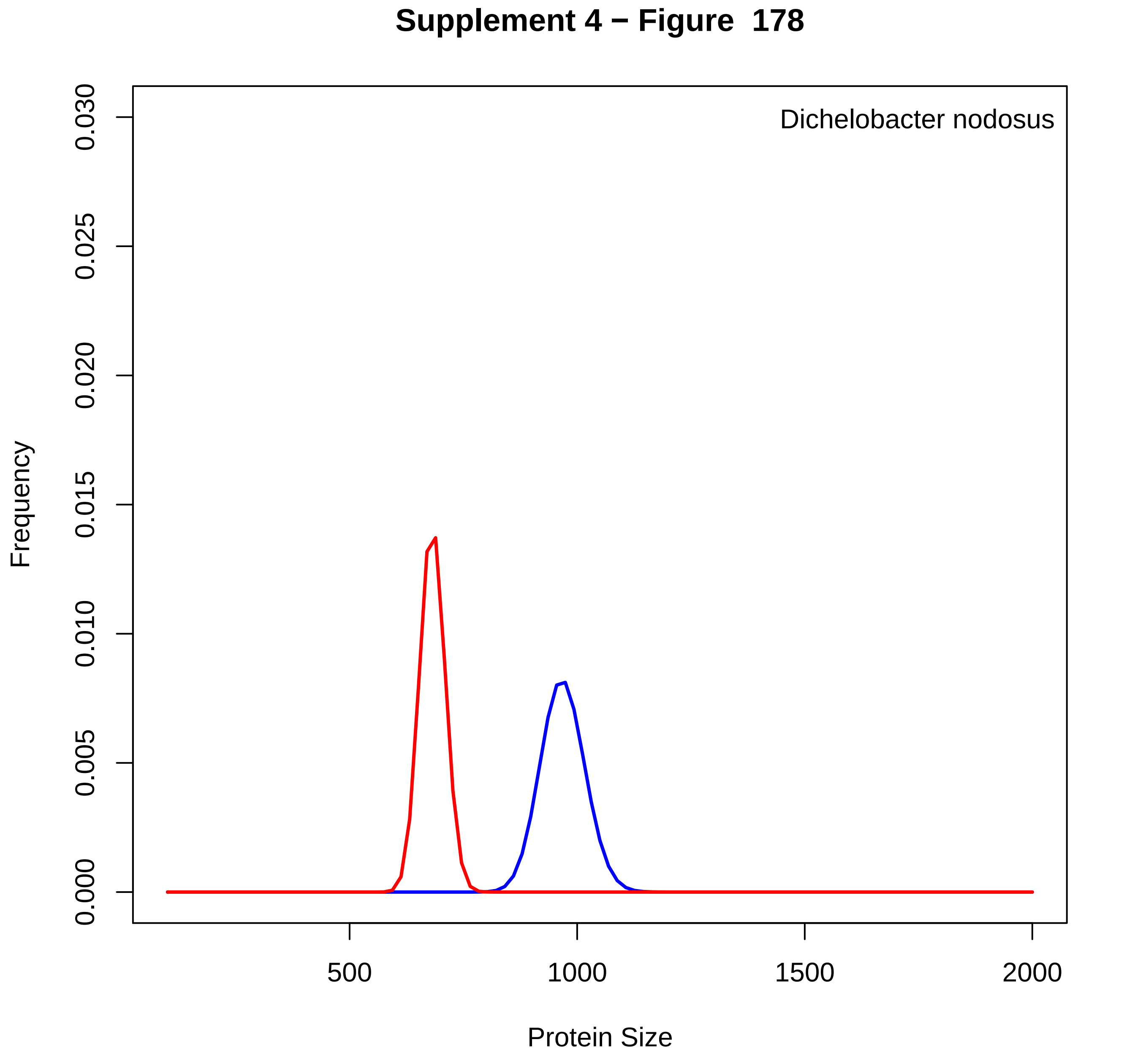
Supplement 4 − Figure 178
Dichelobacter nodosus
Frequency
0.000
0.005
0.010
0.015
0.020
0.025
0.030
500
1000
1500
2000
Protein Size

Supplement 4 − Figure 179

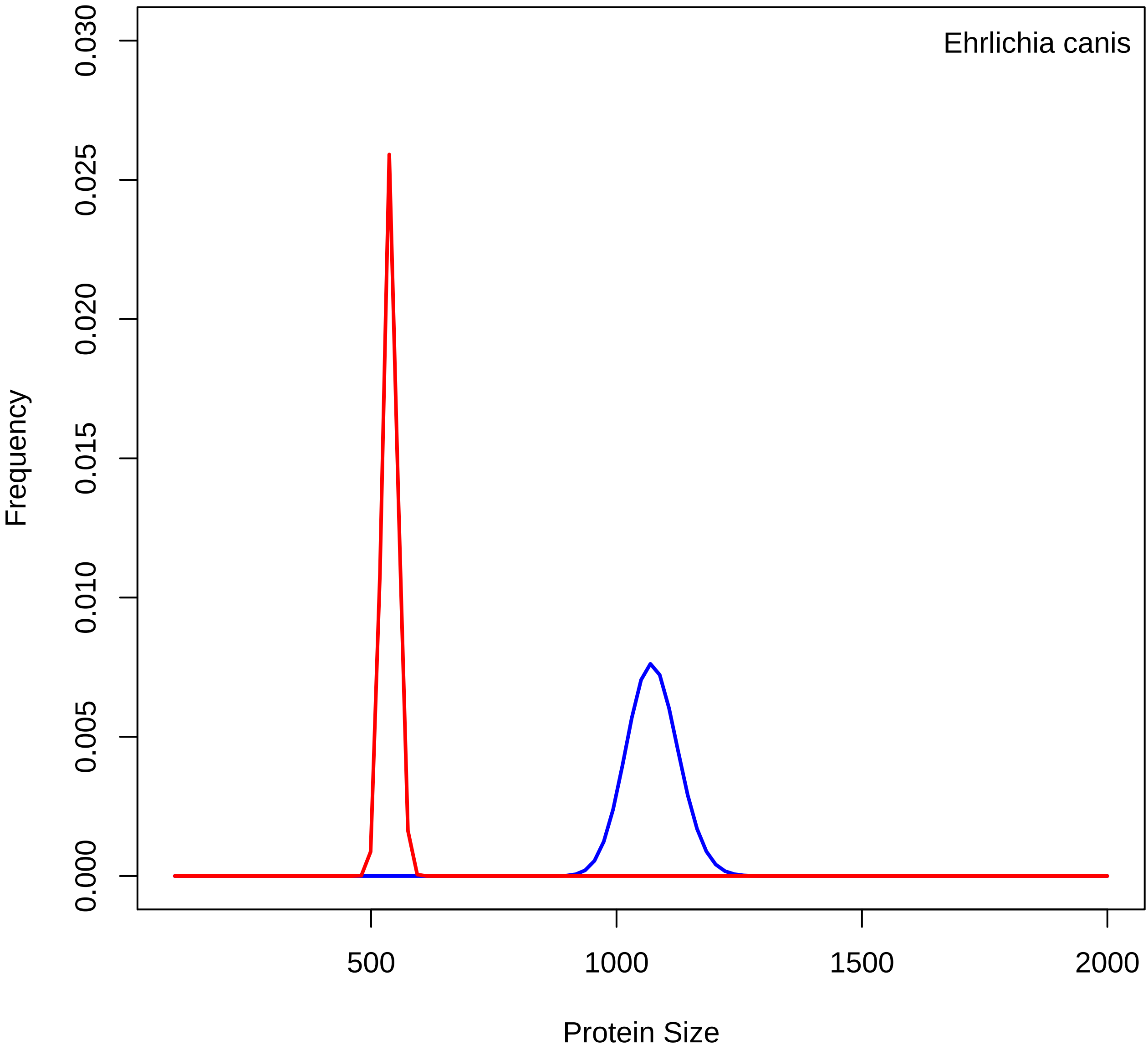

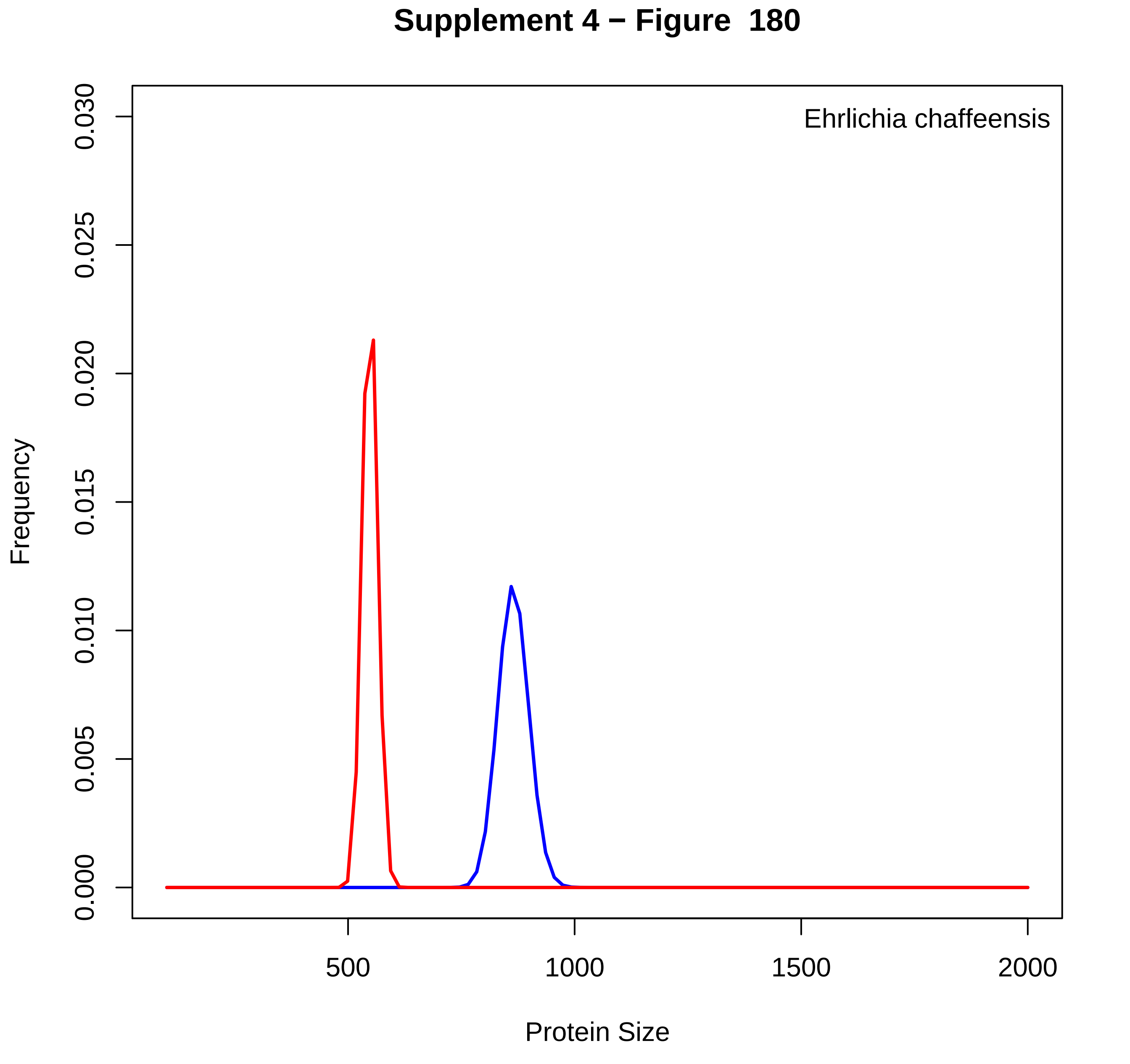
Supplement 4 − Figure 180
Ehrlichia chaffeensis
Frequency
0.000
0.005
0.010
0.015
0.020
0.025
0.030
500
1000
1500
2000
Protein Size

Supplement 4 − Figure 181

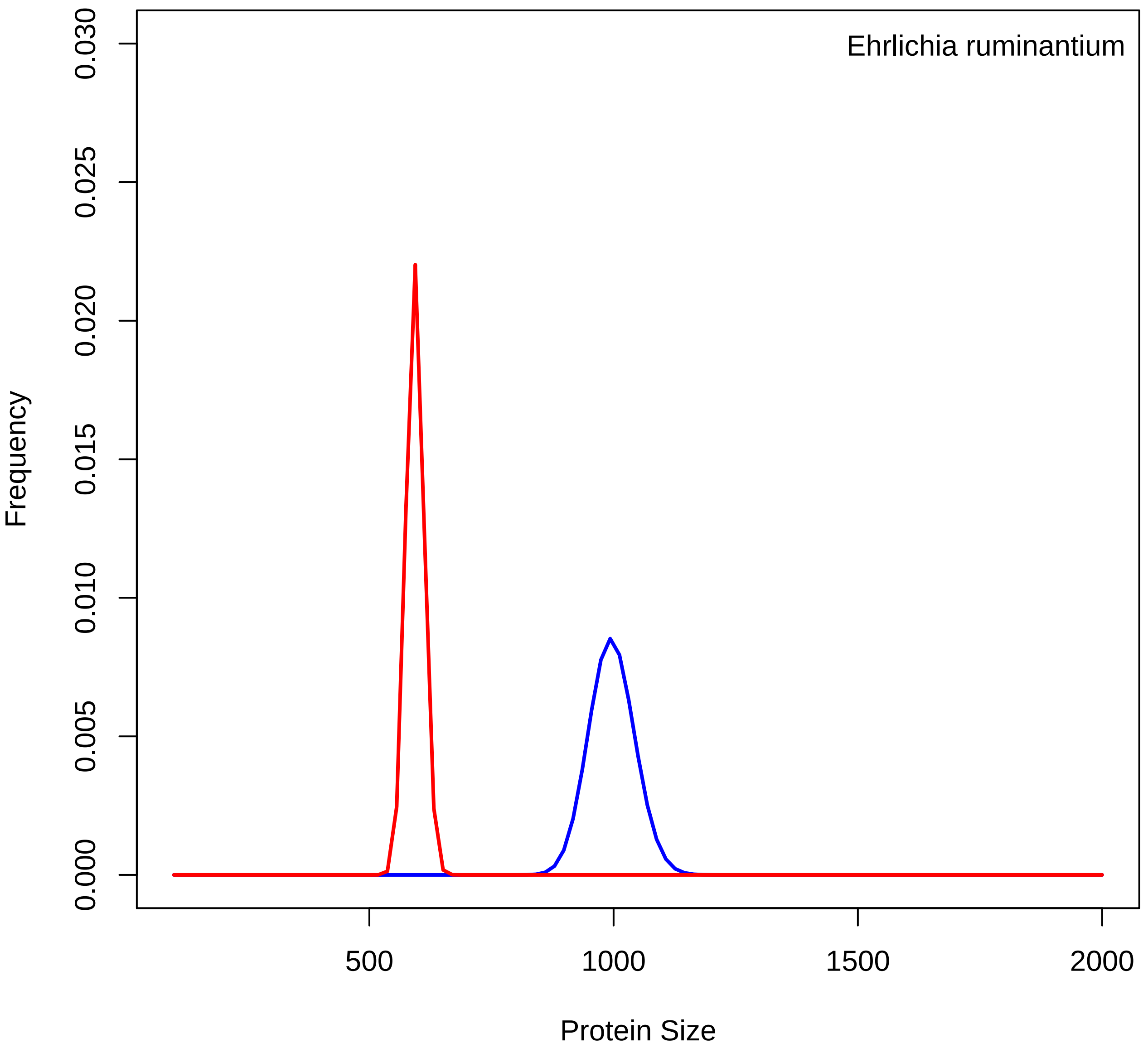

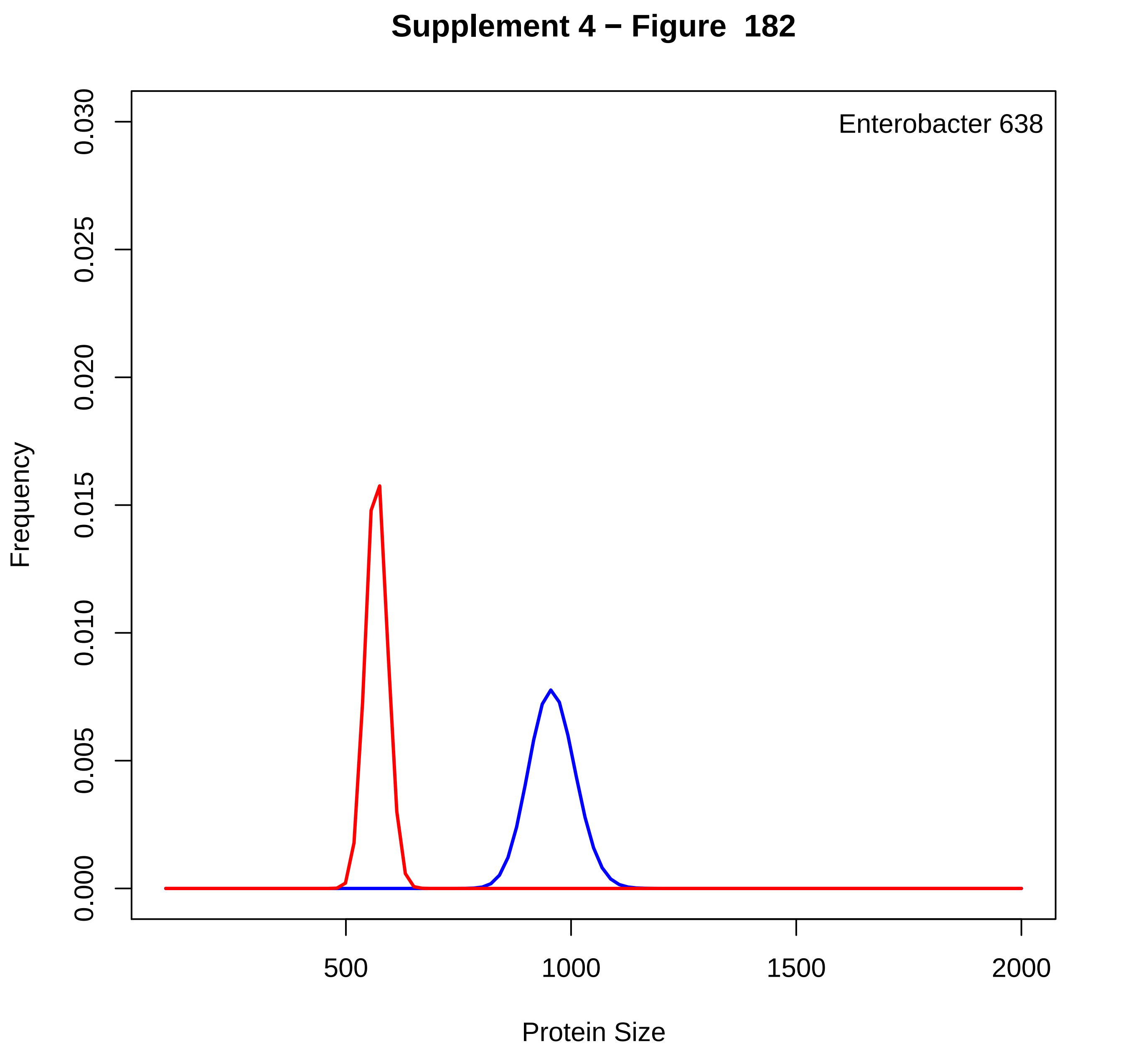
Supplement 4 − Figure 182
Enterobacter 638
Frequency
0.000
0.005
0.010
0.015
0.020
0.025
0.030
500
1000
1500
2000
Protein Size

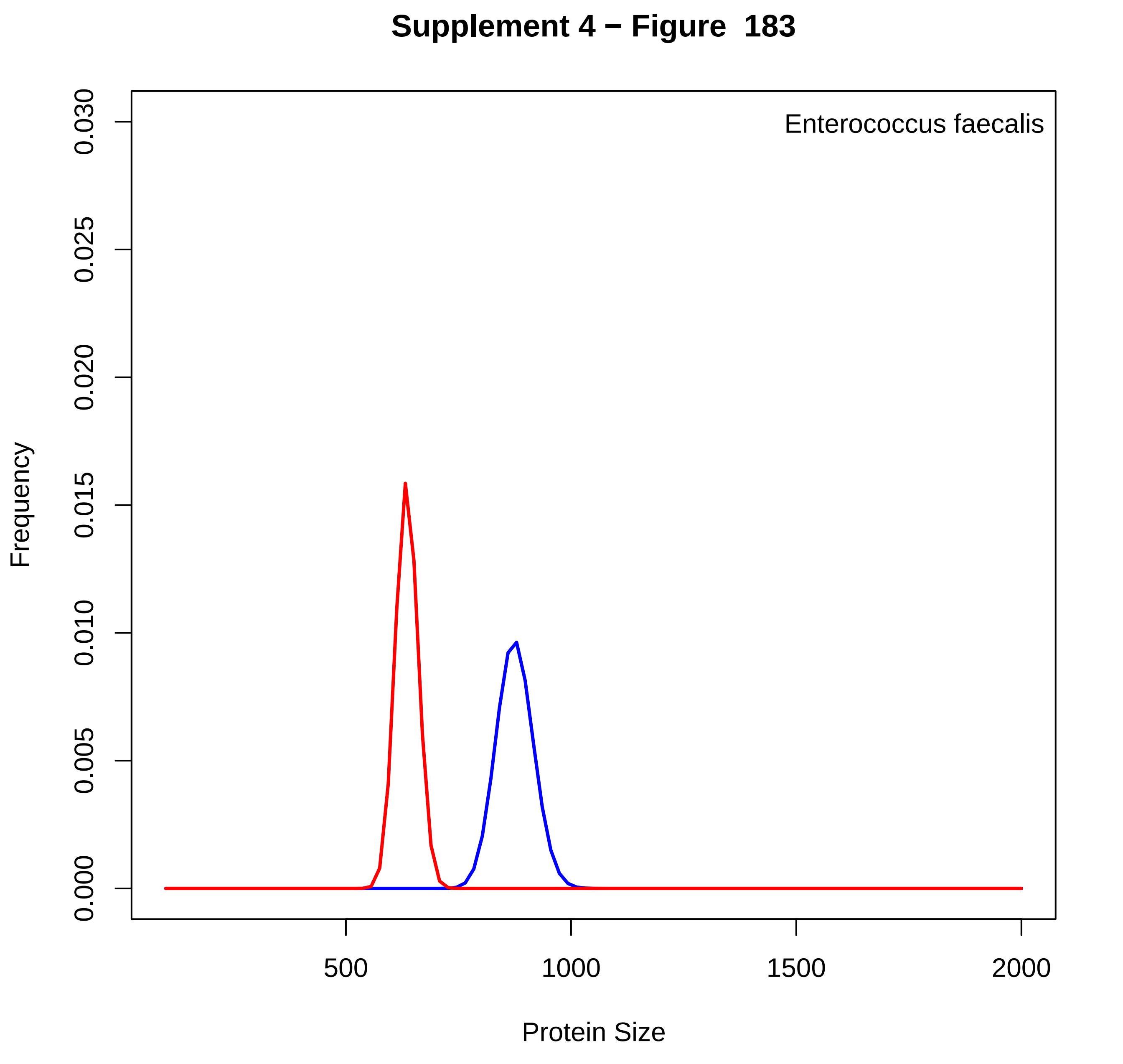

Supplement 4 − Figure 183
Enterococcus faecalis
Frequency
0.000
0.005
0.010
0.015
0.020
0.025
0.030
500
1000
1500
2000
Protein Size

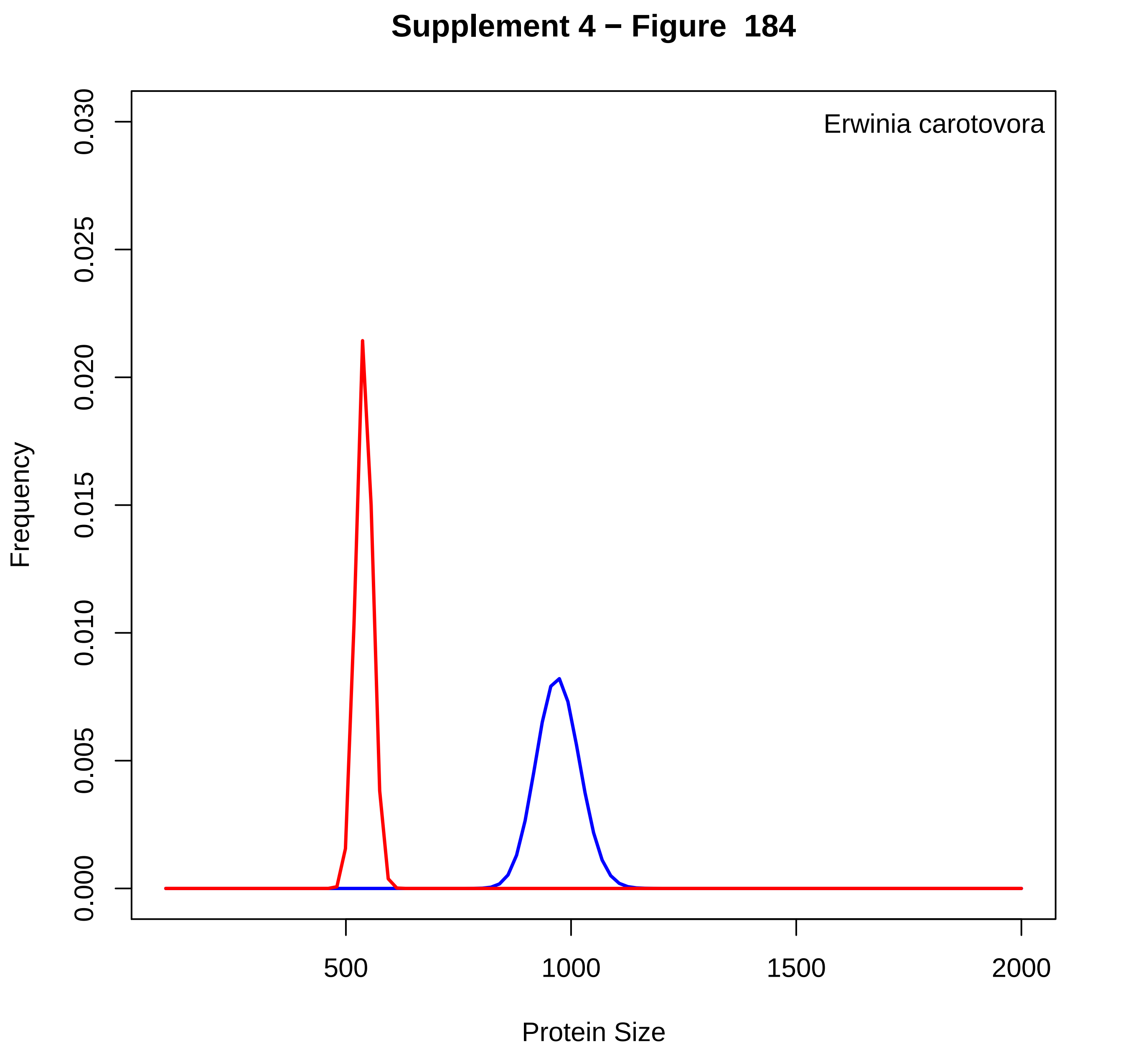
Supplement 4 − Figure 184
Erwinia carotovora
Frequency
0.000
0.005
0.010
0.015
0.020
0.025
0.030
500
1000
1500
2000
Protein Size

Supplement 4 − Figure 185

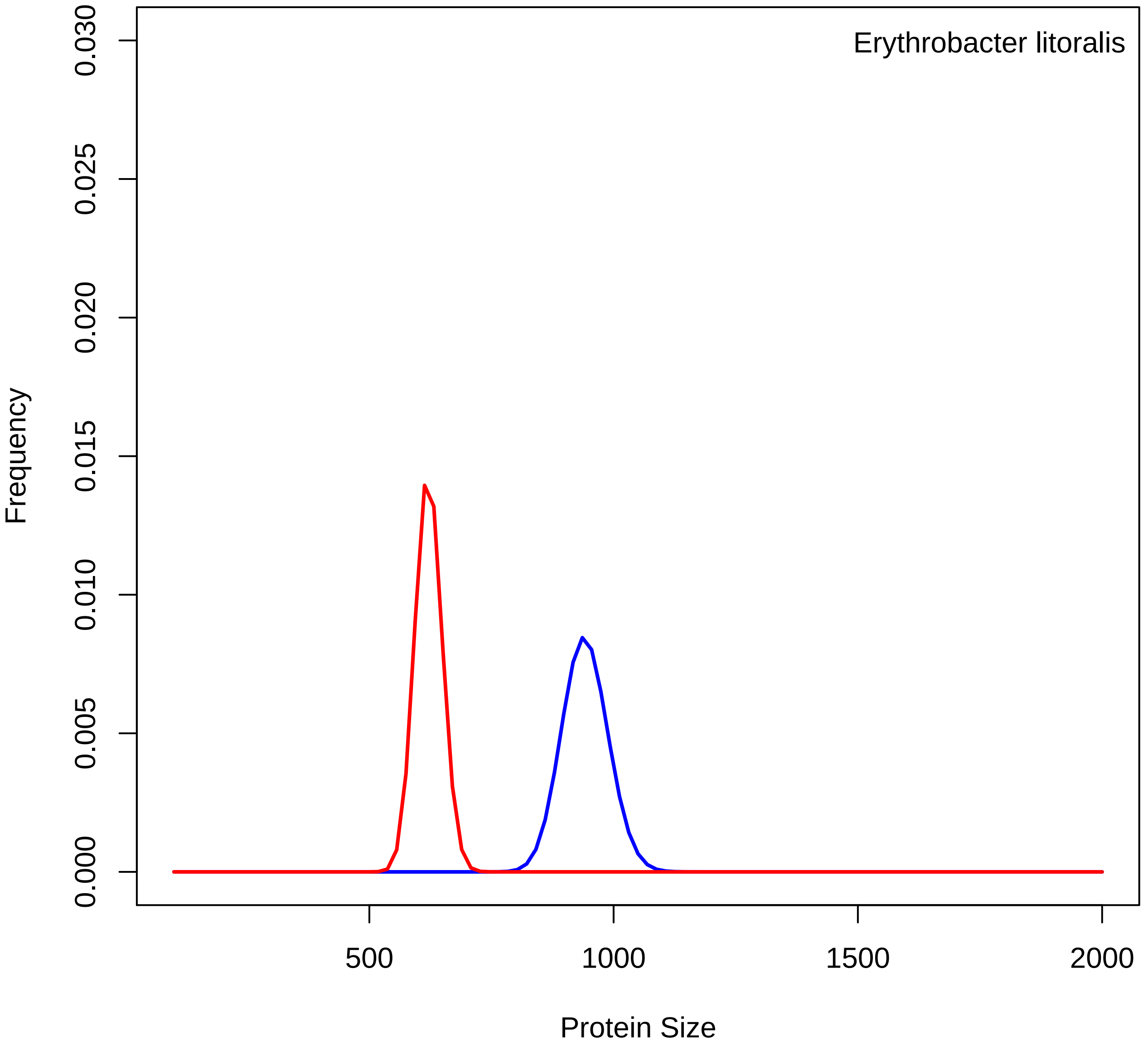

Supplement 4 − Figure 186

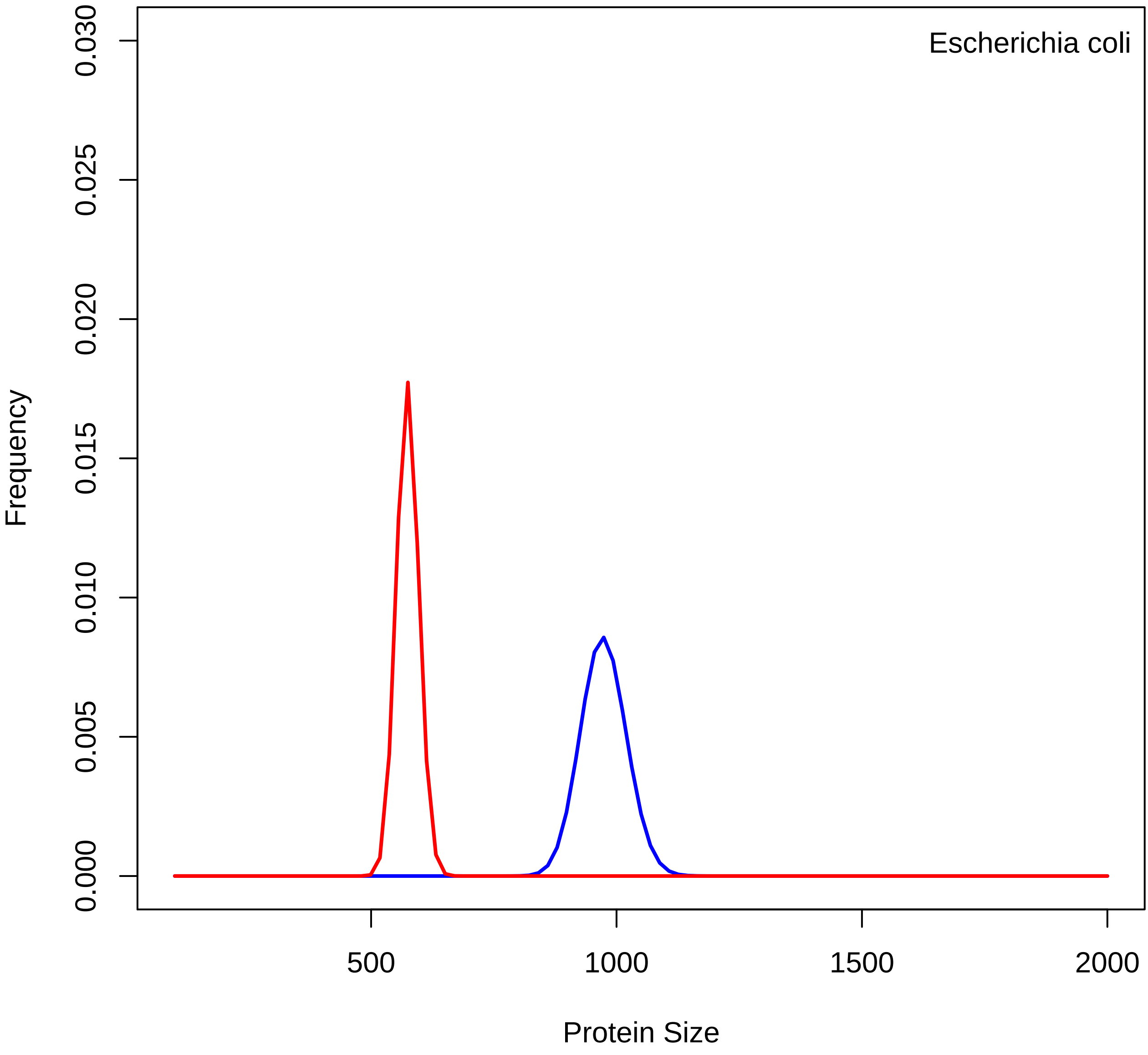

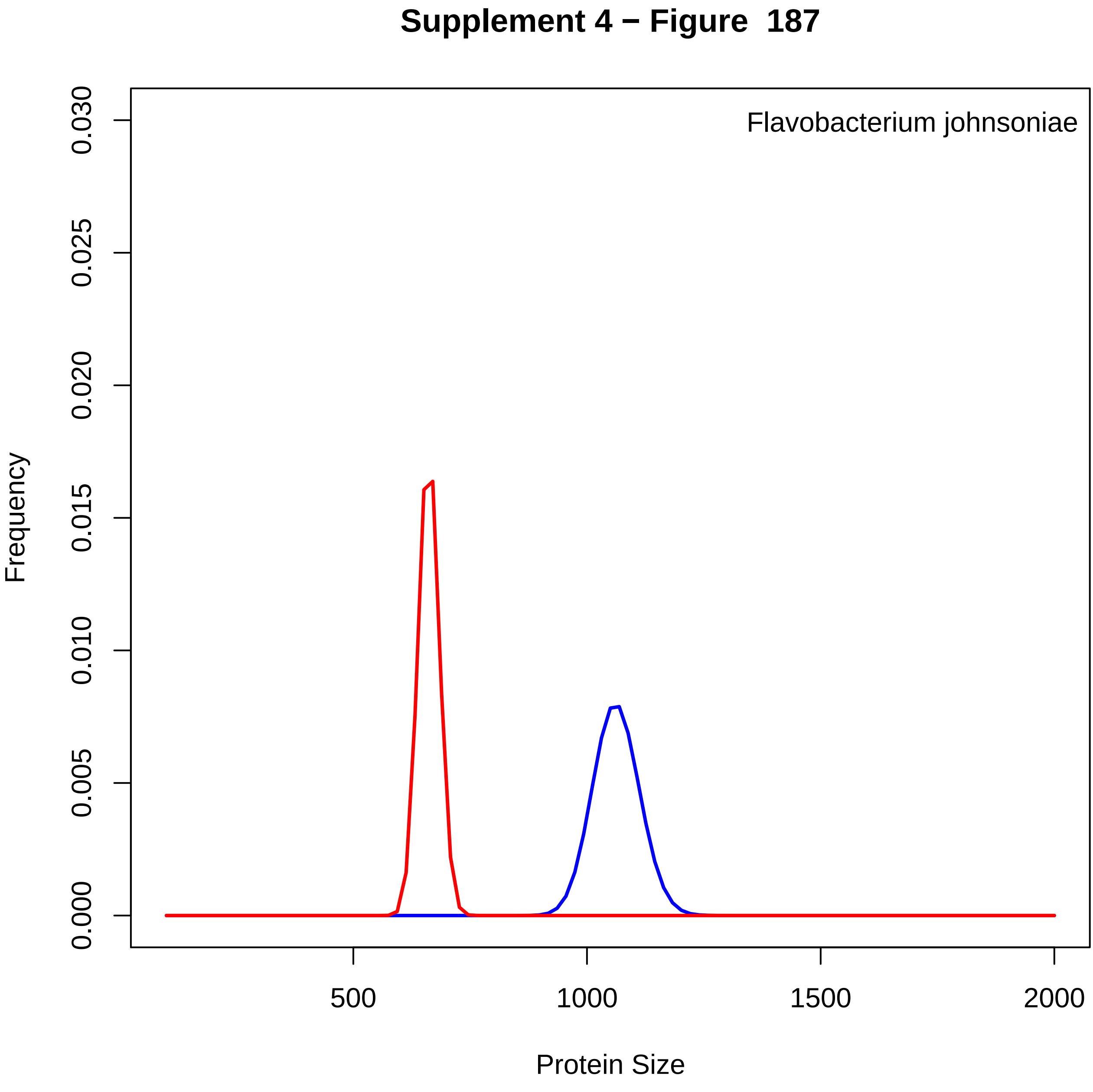
Supplement 4 − Figure 187
Flavobacterium johnsoniae
Frequency
0.000
0.005
0.010
0.015
0.020
0.025
0.030
500
1000
1500
2000
Protein Size

Supplement 4 − Figure 188

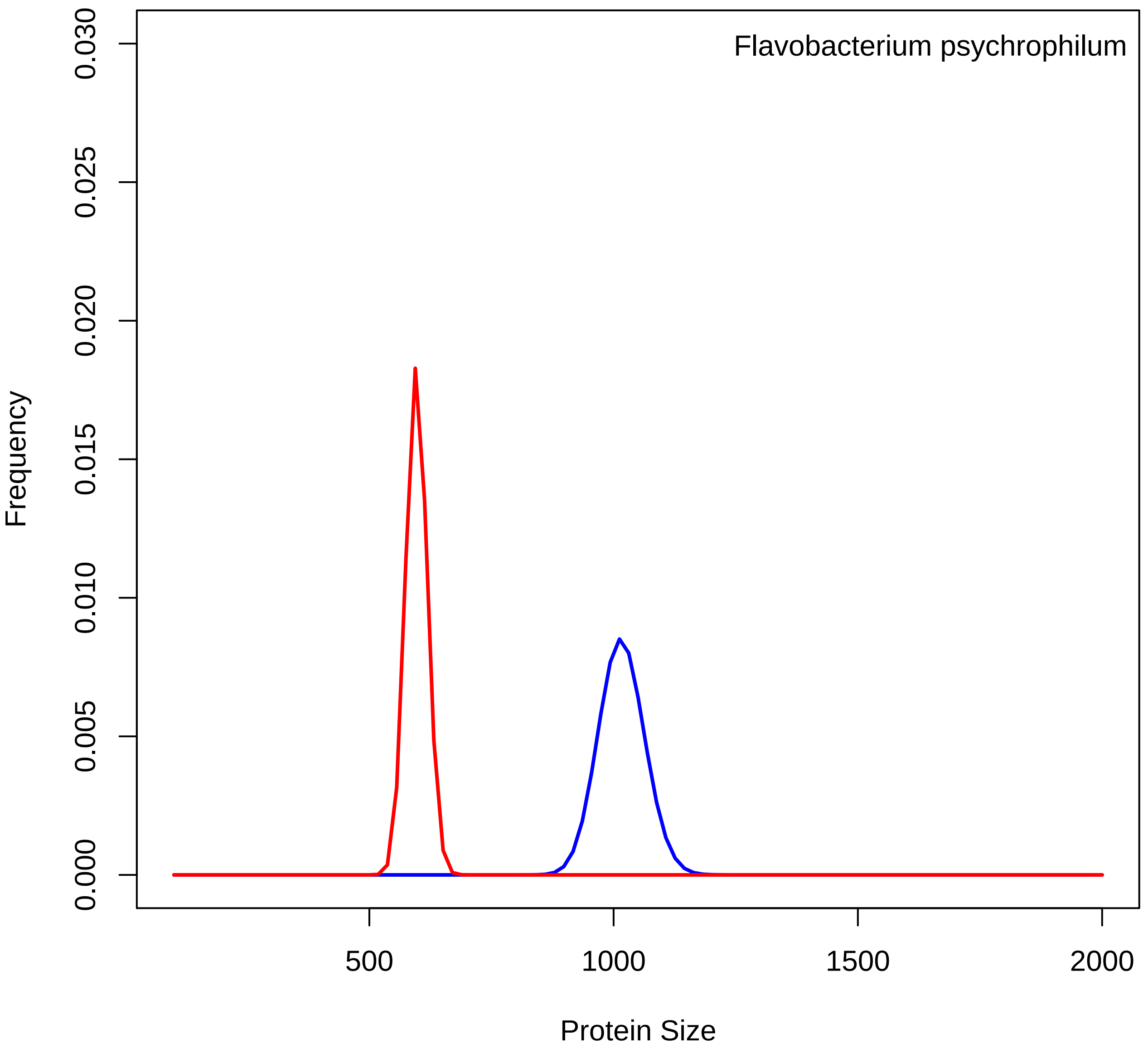

Supplement 4 − Figure 189

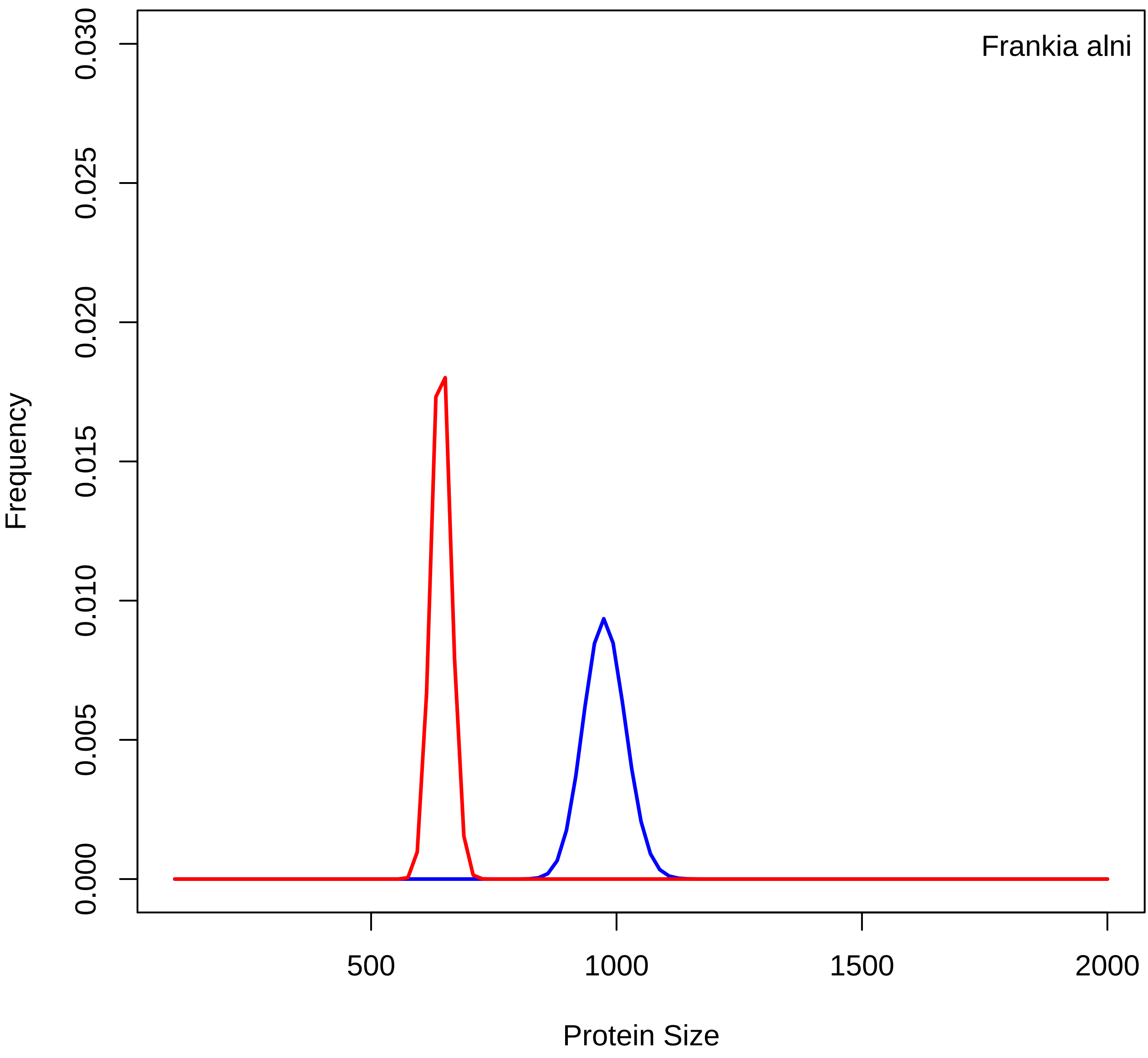

Supplement 4 − Figure 190

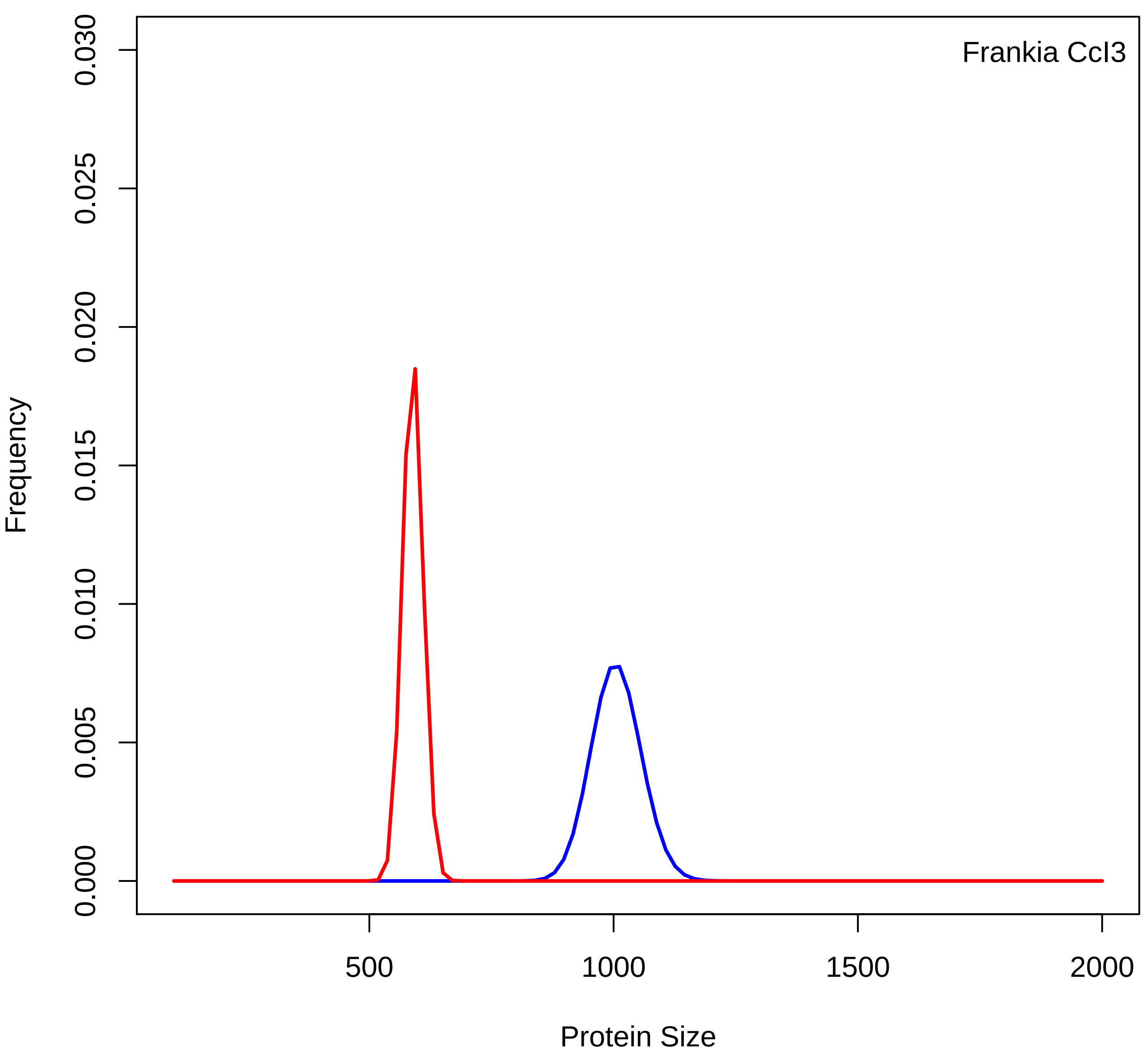

Supplement 4 − Figure 191

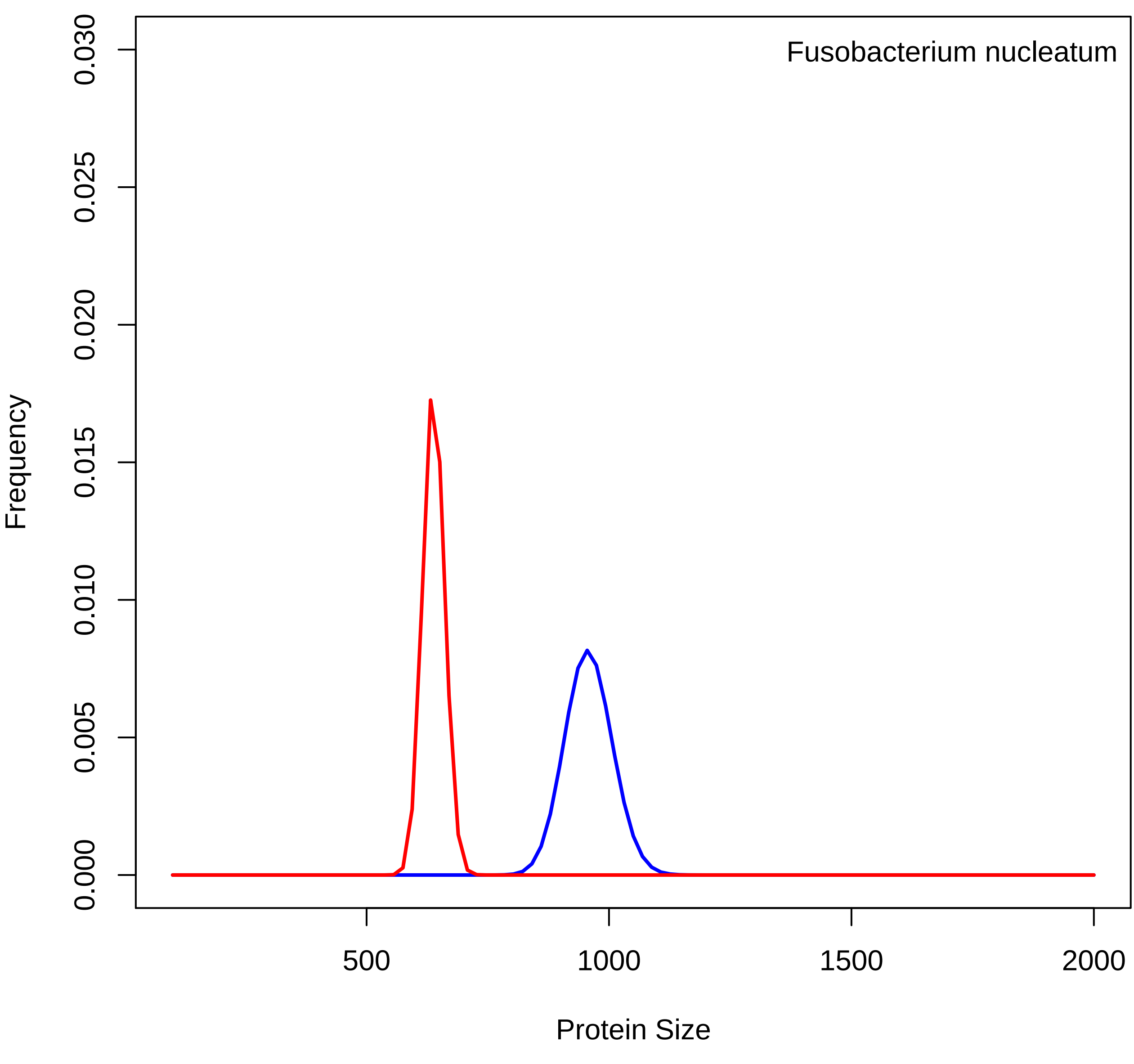

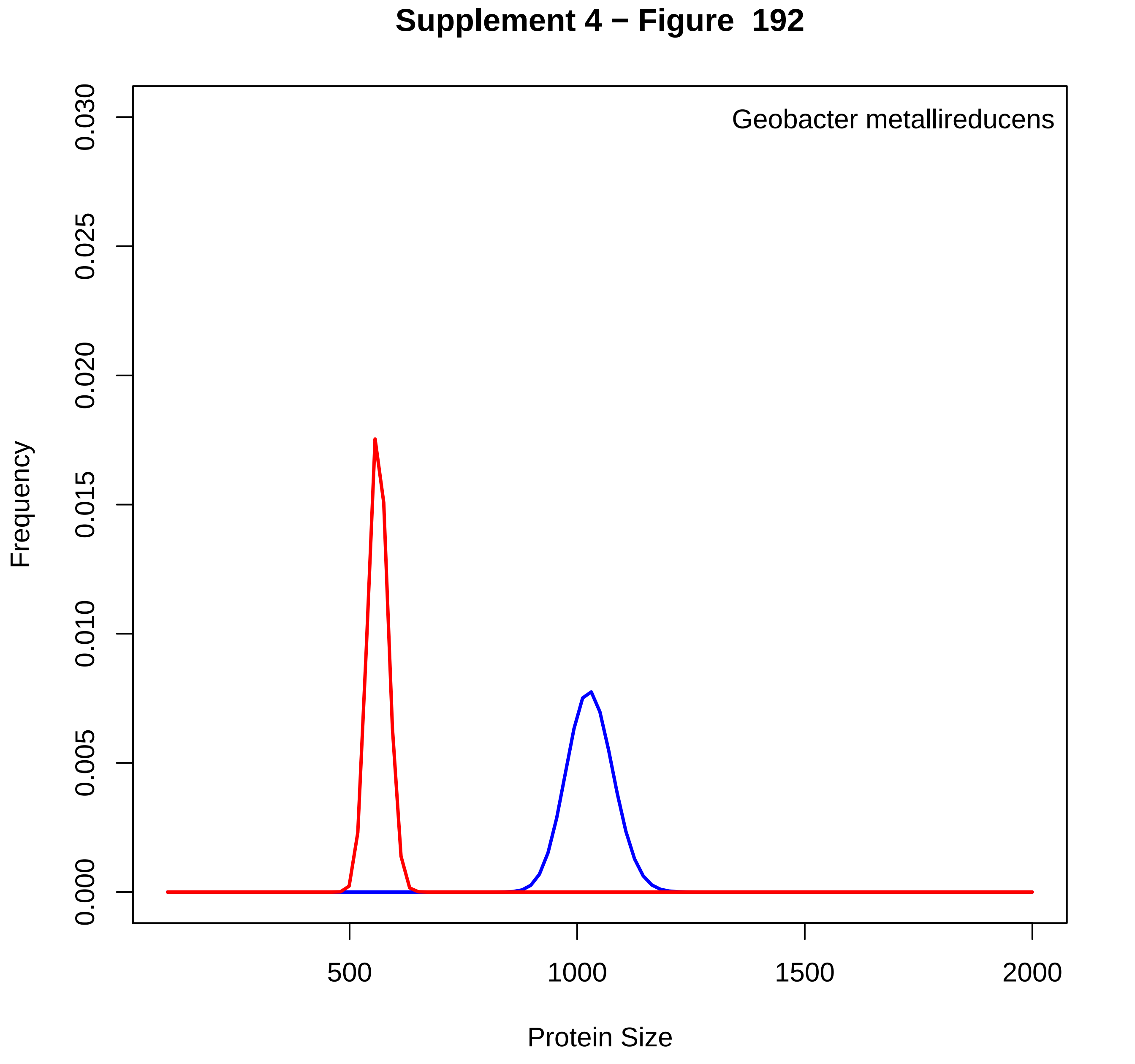

Supplement 4 − Figure 192
Geobacter metallireducens
Frequency
0.000
0.005
0.010
0.015
0.020
0.025
0.030
500
1000
1500
2000
Protein Size

Supplement 4 − Figure 193

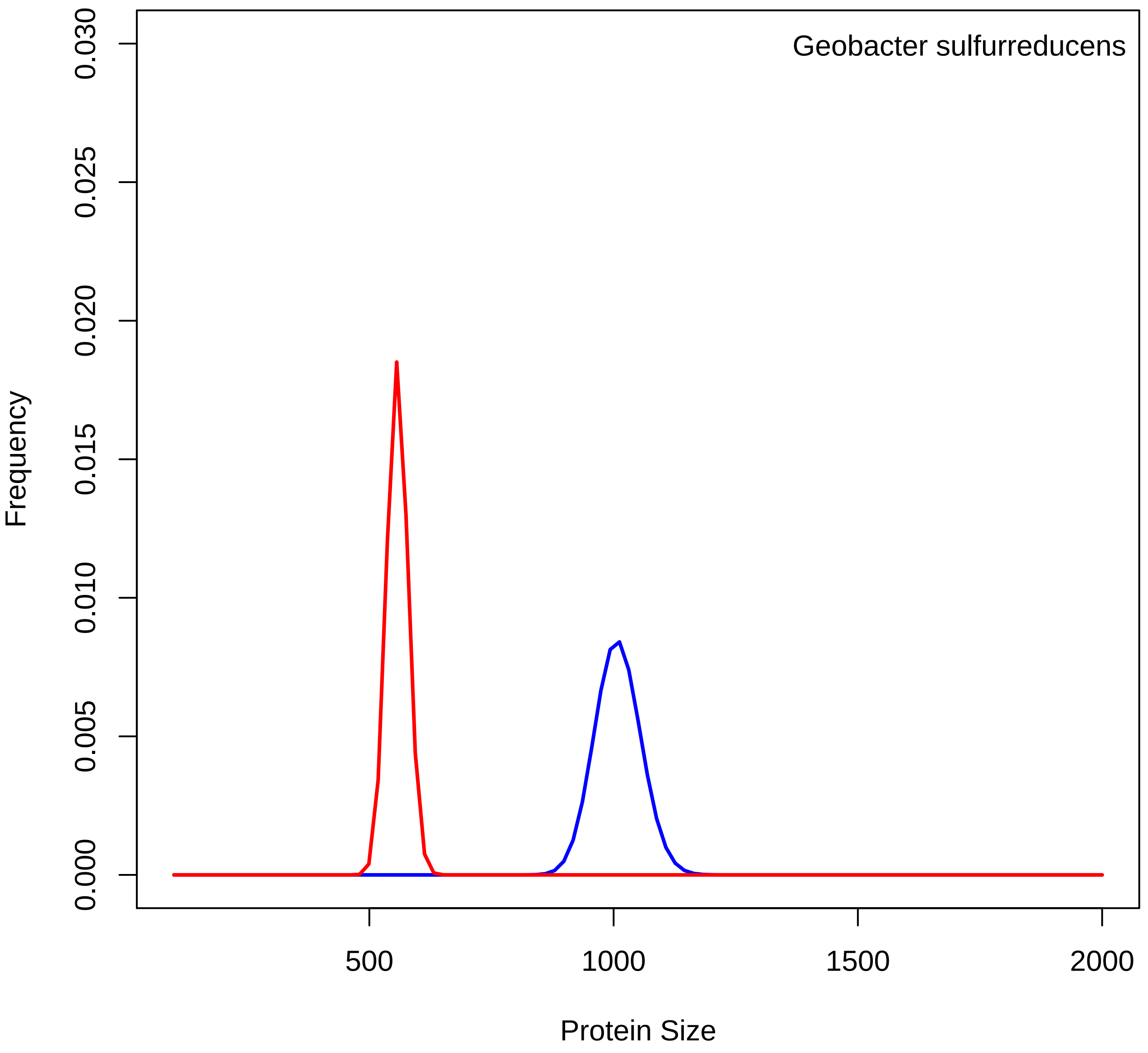

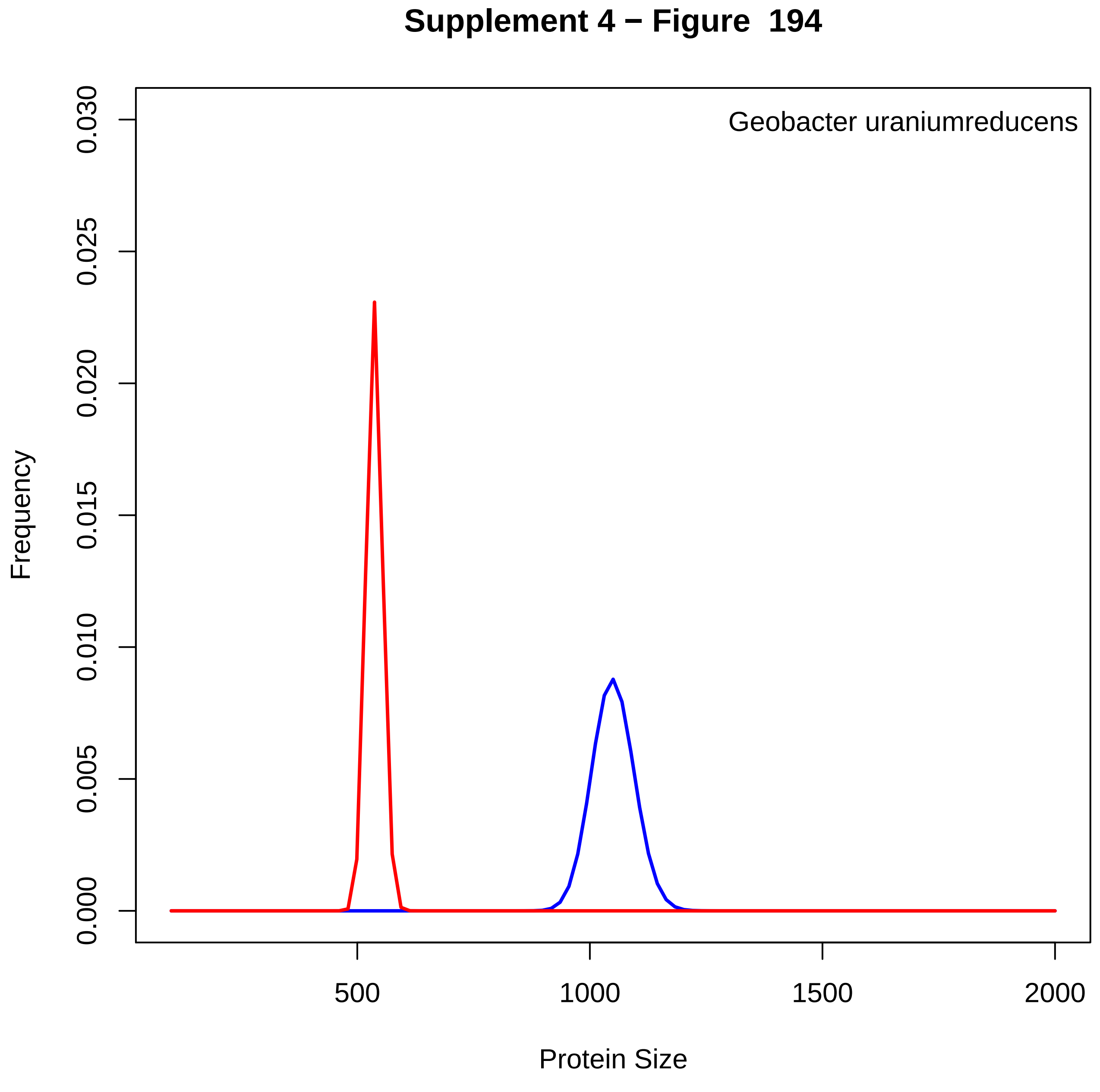
Supplement 4 − Figure 194
Geobacter uraniumreducens
Frequency
0.000
0.005
0.010
0.015
0.020
0.025
0.030
500
1000
1500
2000
Protein Size

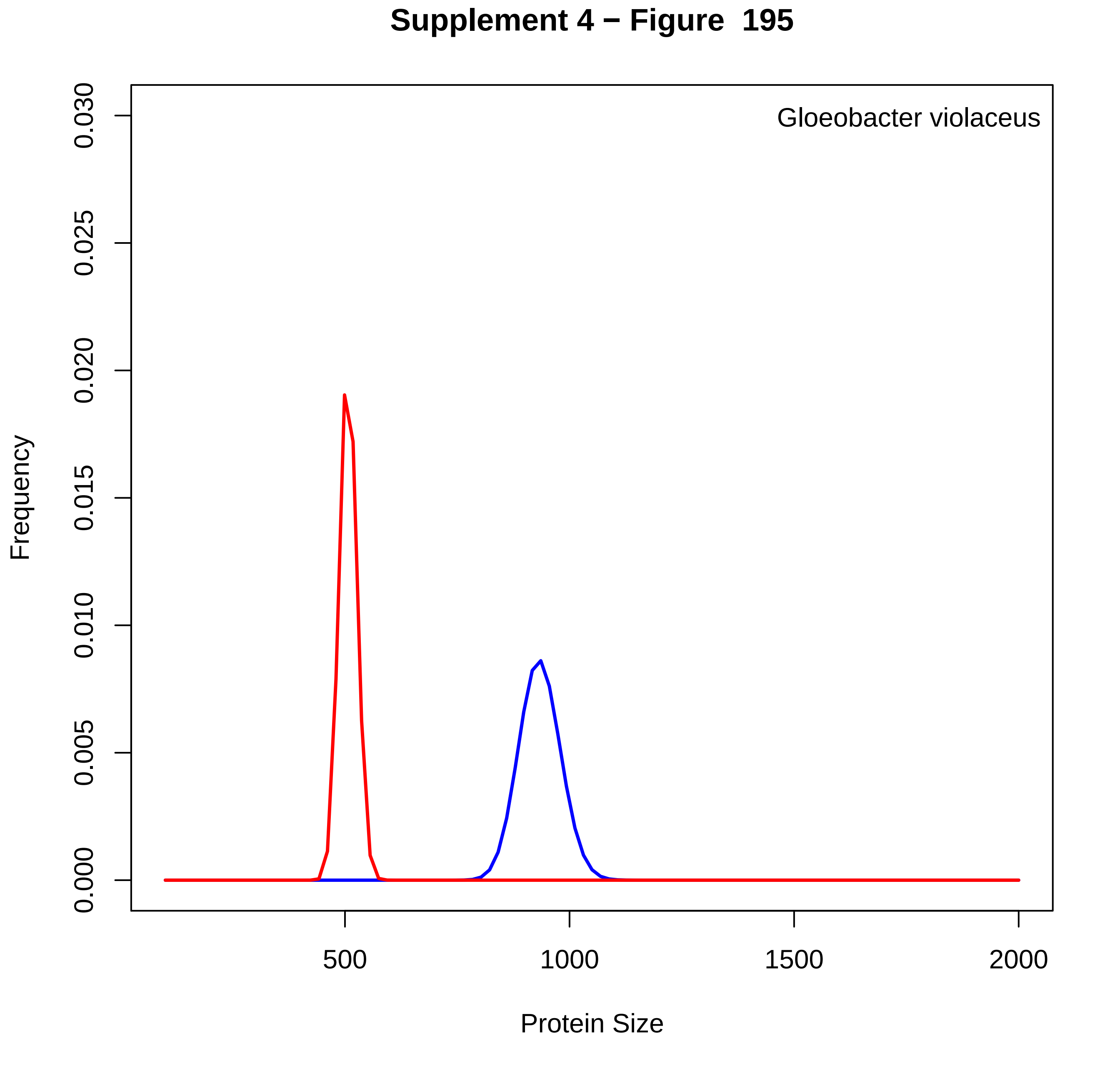

Supplement 4 − Figure 195
Gloeobacter violaceus
Frequency
0.000
0.005
0.010
0.015
0.020
0.025
0.030
500
1000
1500
2000
Protein Size

Supplement 4 − Figure 196

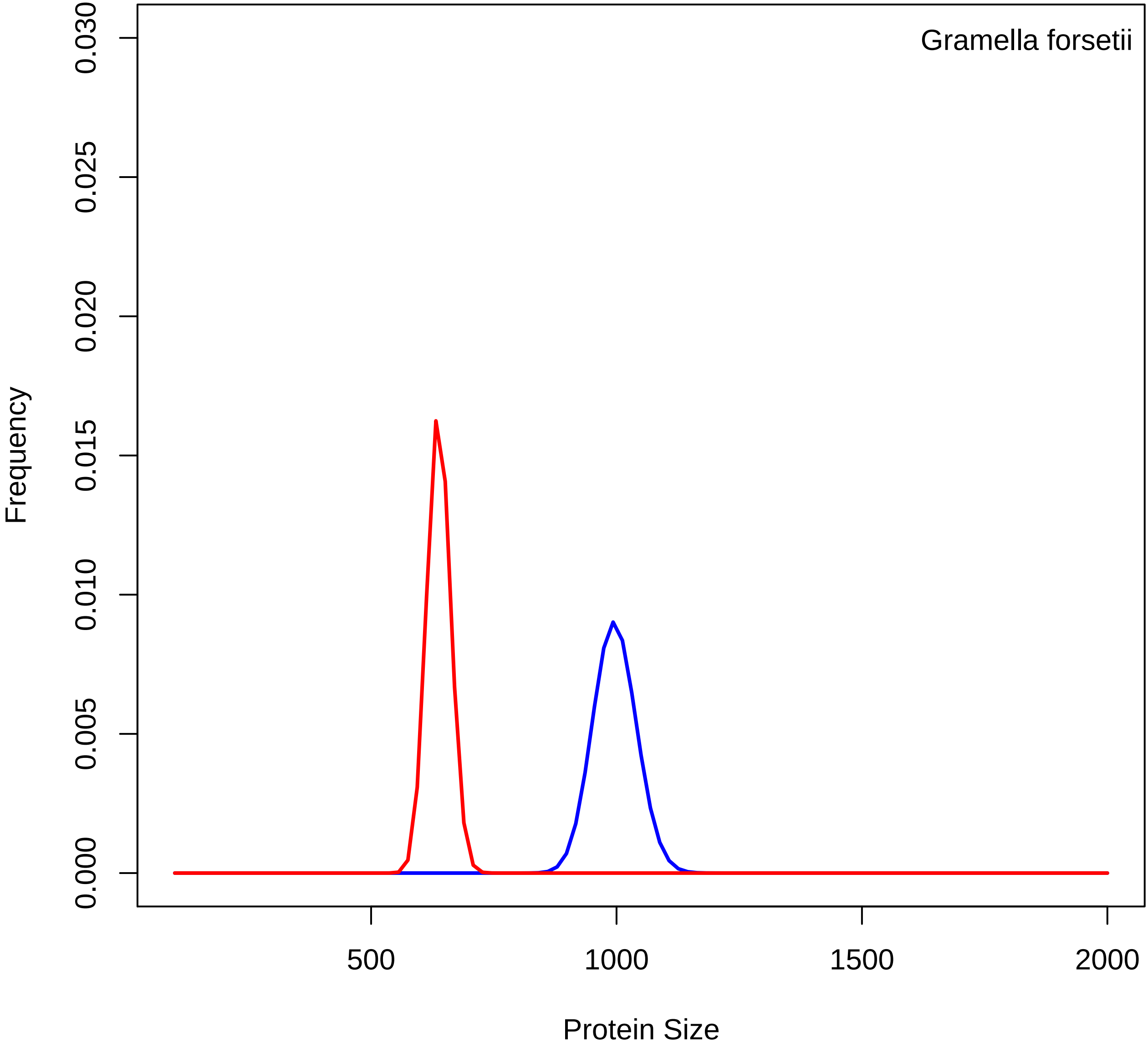

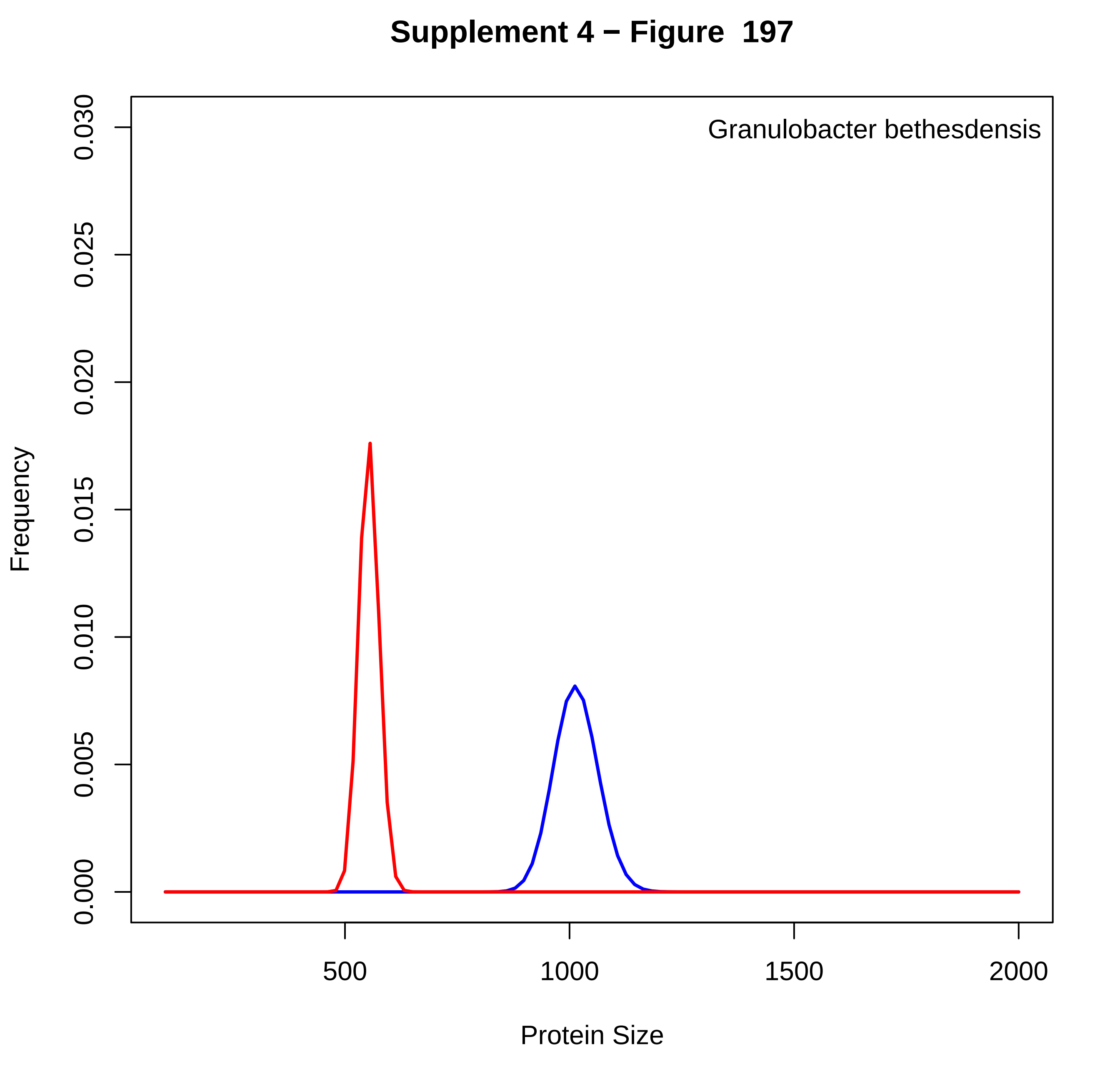
Supplement 4 − Figure 197
Granulobacter bethesdensis
Frequency
0.000
0.005
0.010
0.015
0.020
0.025
0.030
500
1000
1500
2000
Protein Size

Supplement 4 − Figure 198

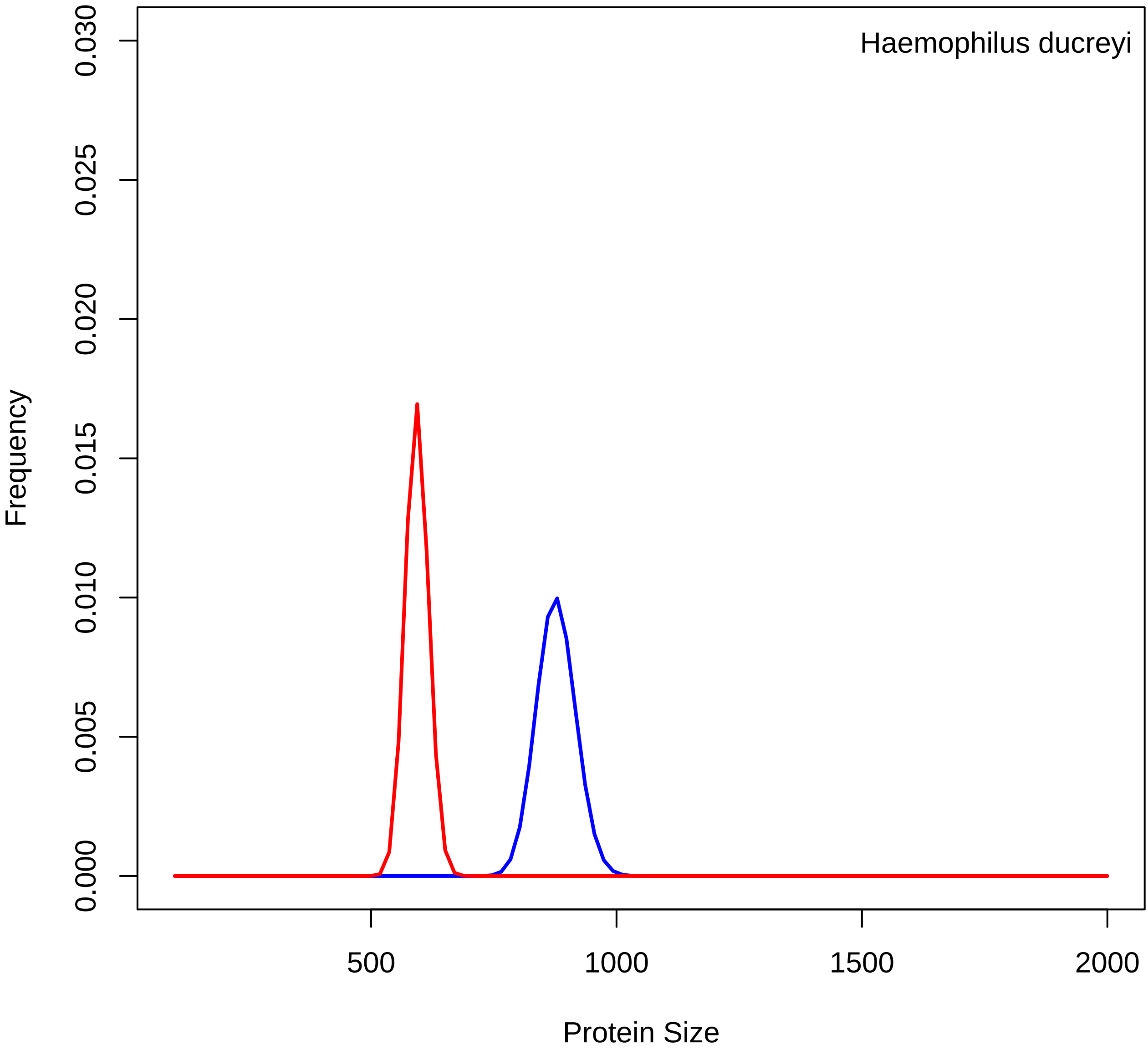

Supplement 4 − Figure 199

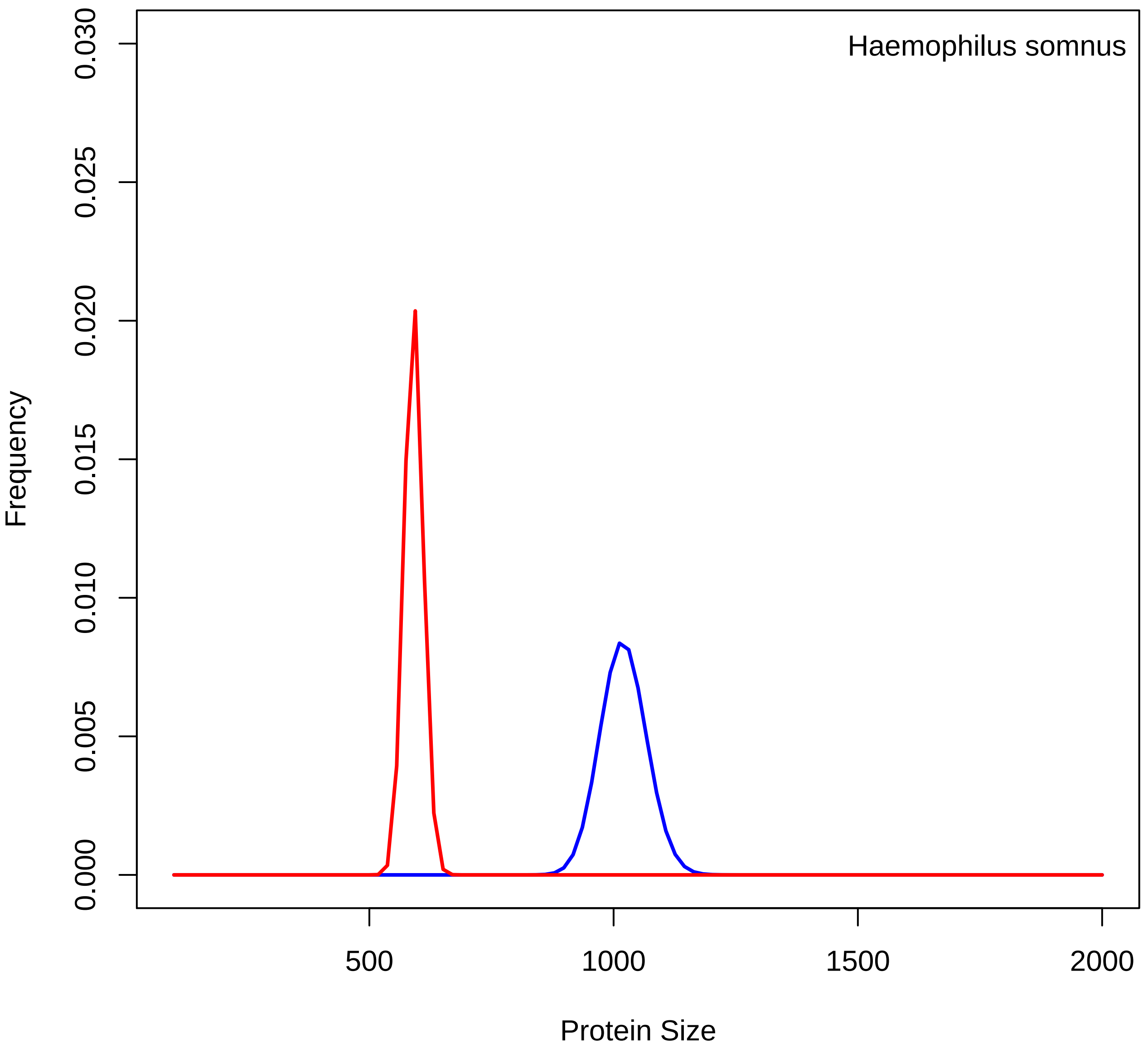

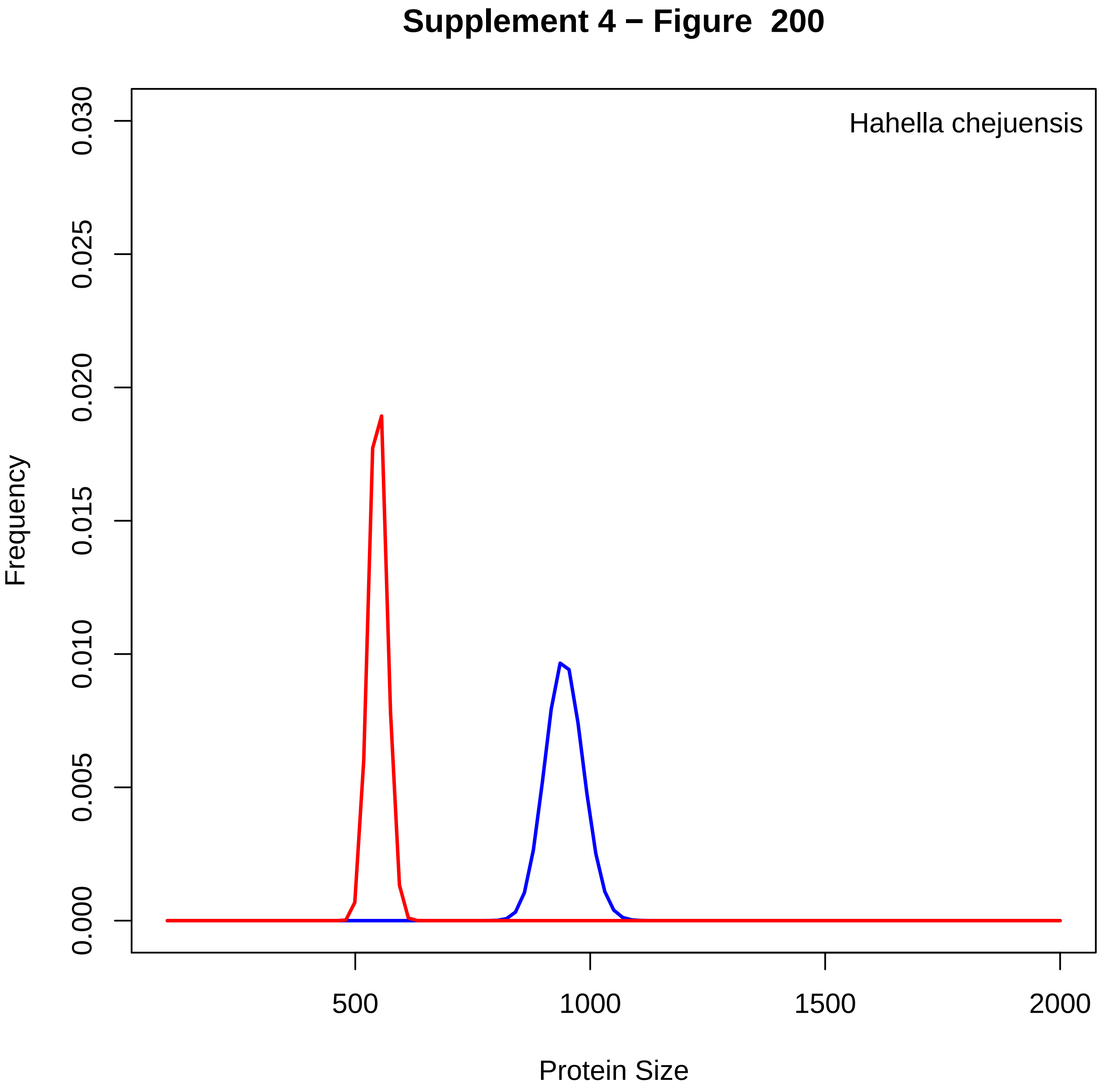

Supplement 4 − Figure 200
Hahella chejuensis
Frequency
0.000
0.005
0.010
0.015
0.020
0.025
0.030
500
1000
1500
2000
Protein Size

Supplement 4 − Figure 201

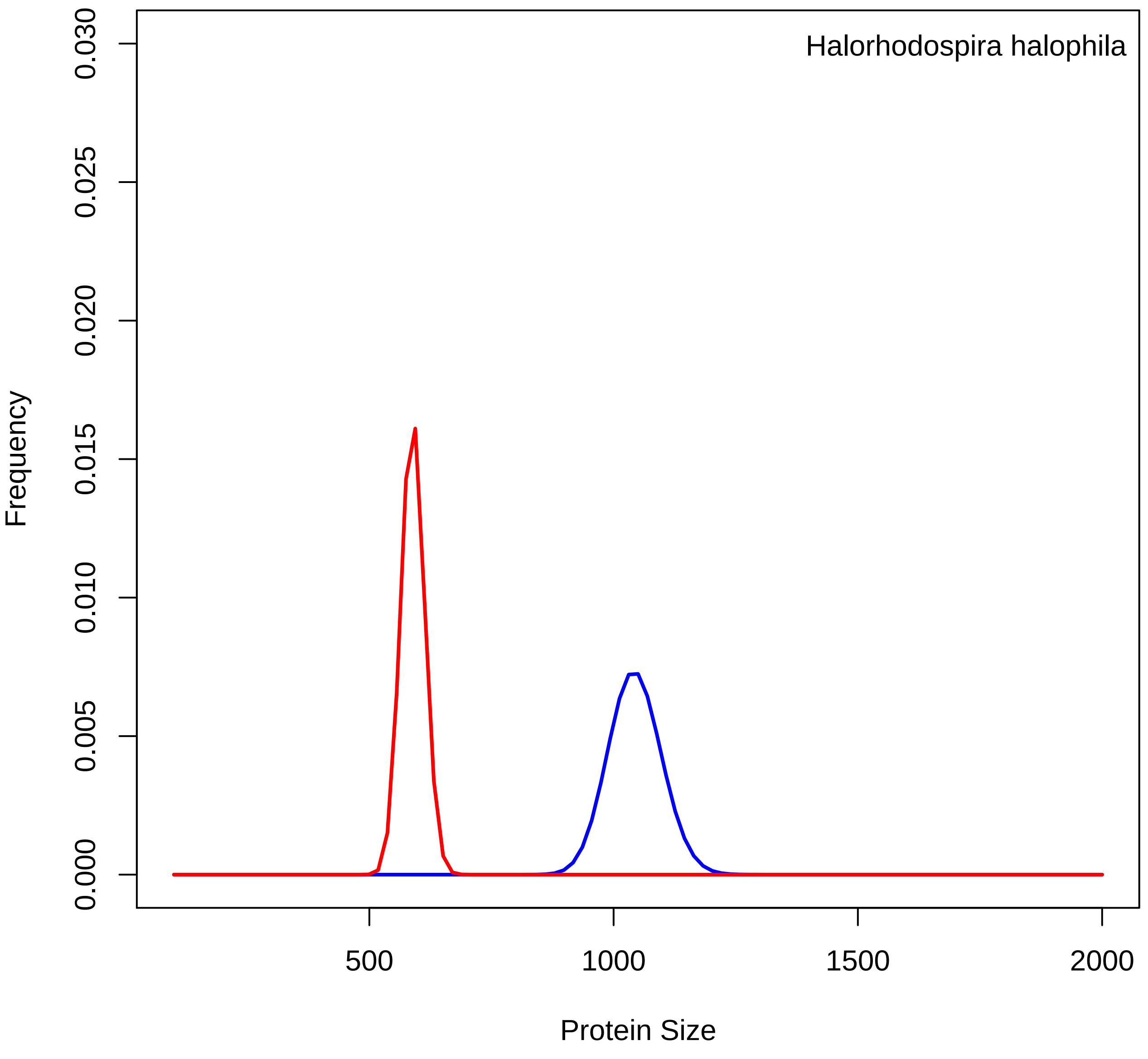

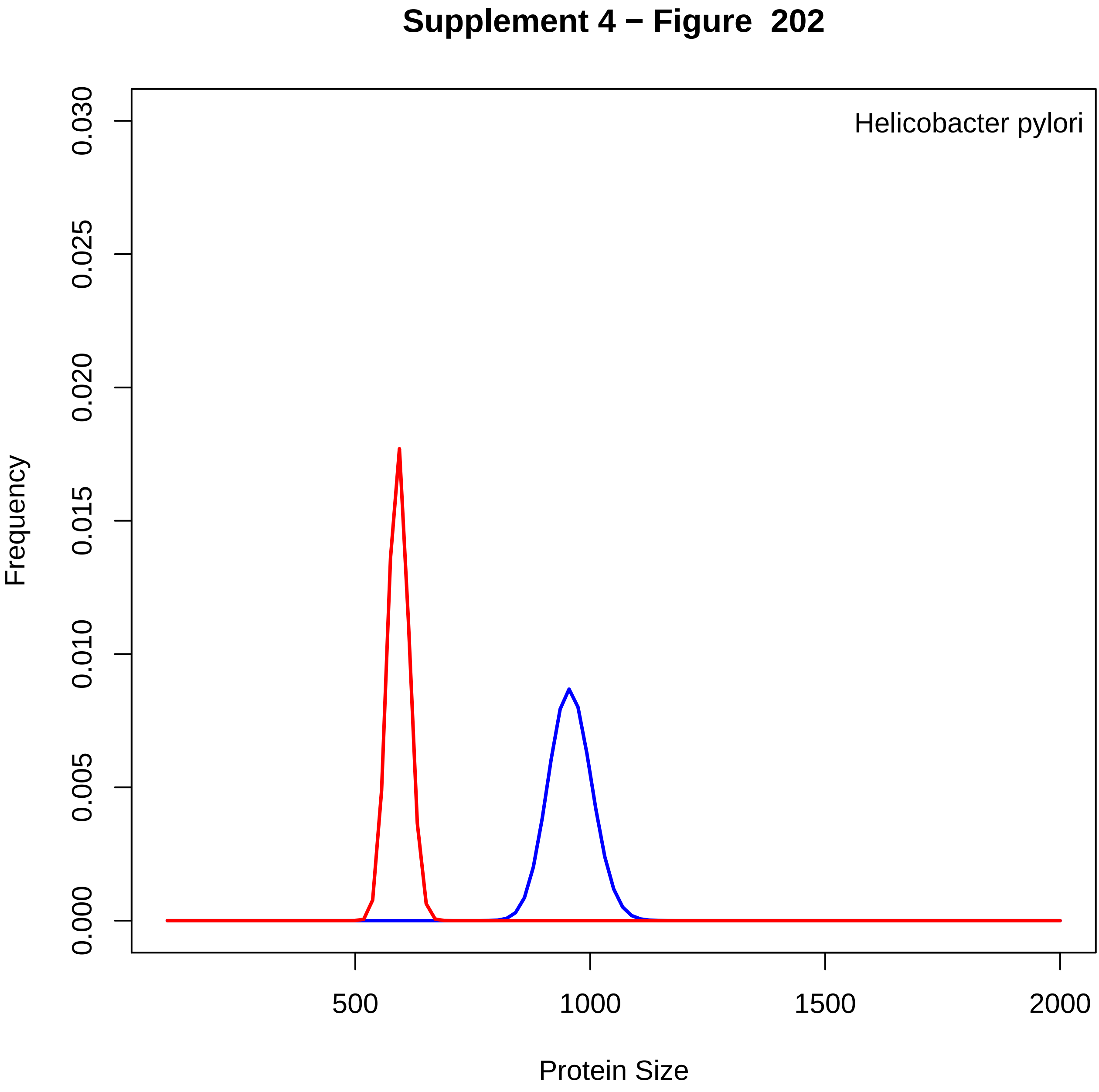
Supplement 4 − Figure 202
Helicobacter pylori
Frequency
0.000
0.005
0.010
0.015
0.020
0.025
0.030
500
1000
1500
2000
Protein Size

Supplement 4 − Figure 203

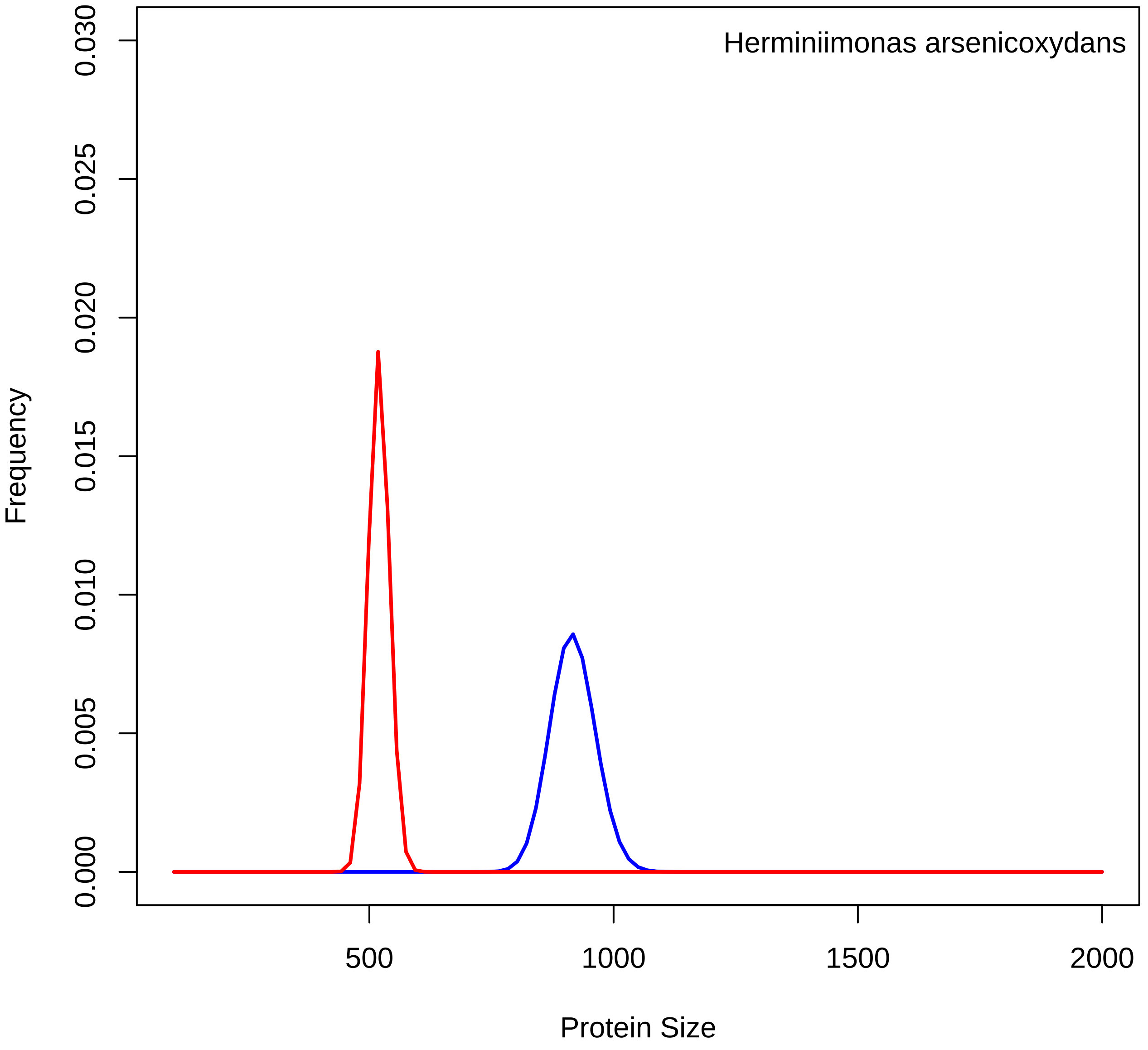

Supplement 4 − Figure 204

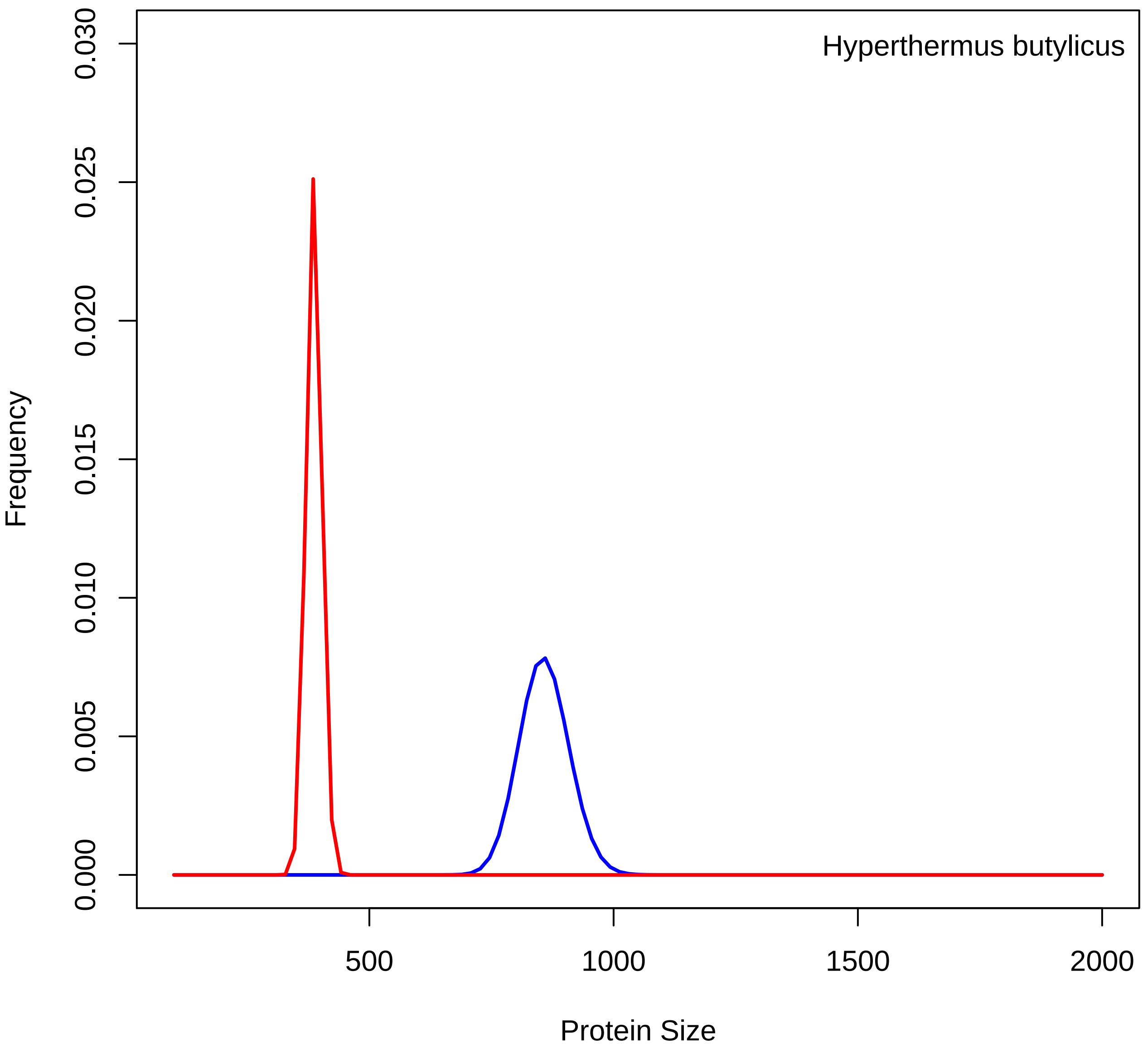

Supplement 4 − Figure 205

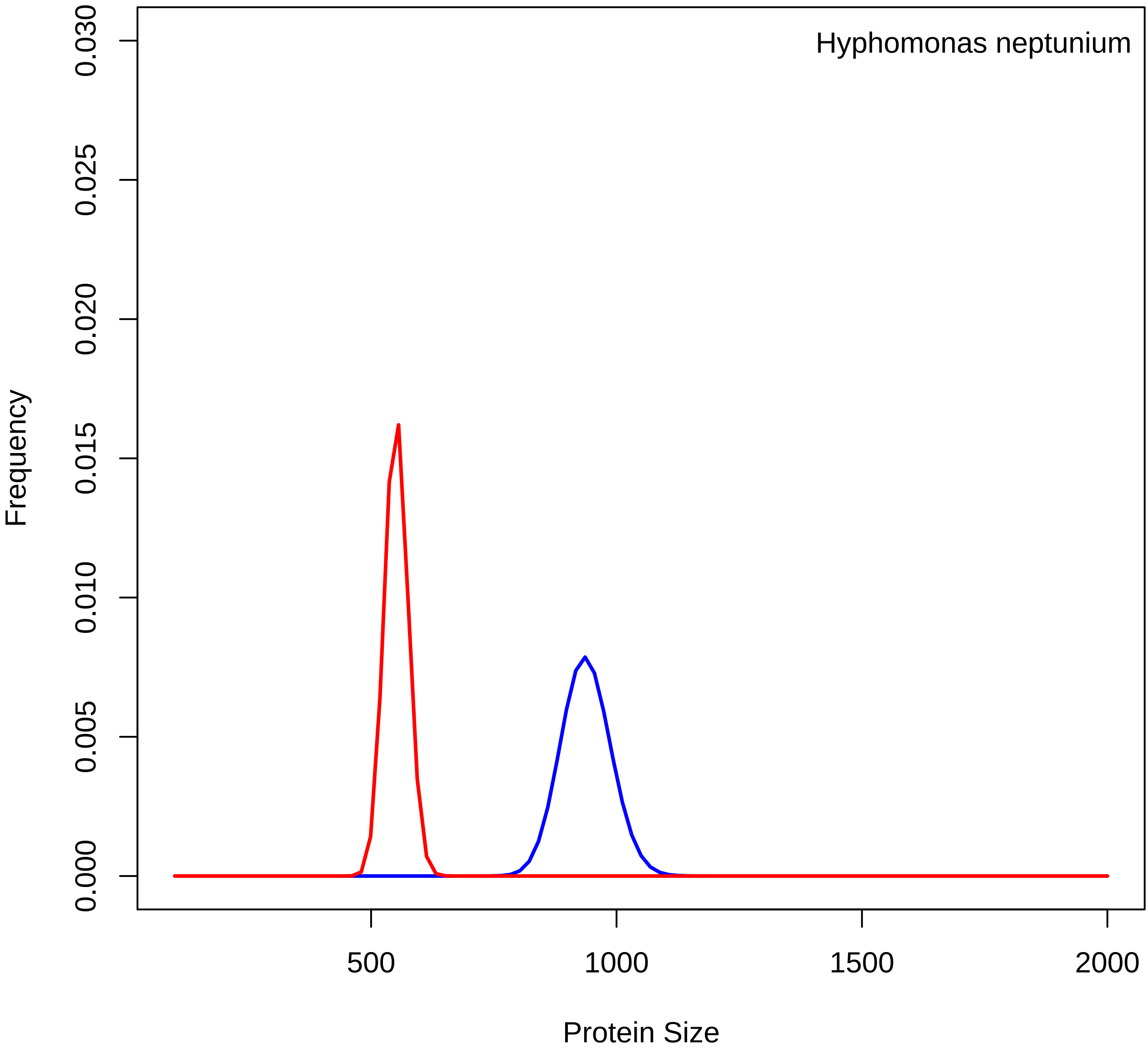

Supplement 4 − Figure 206

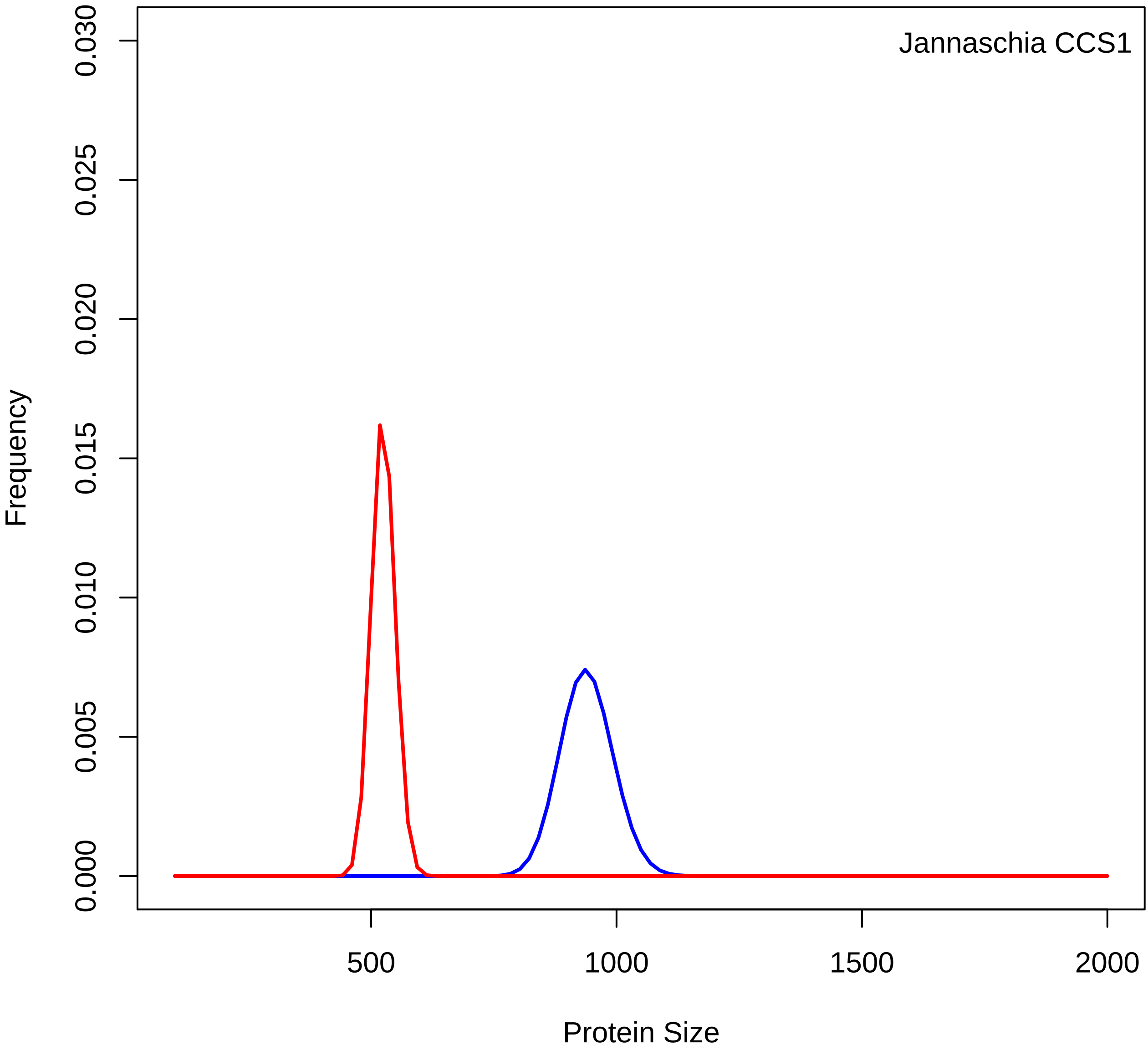

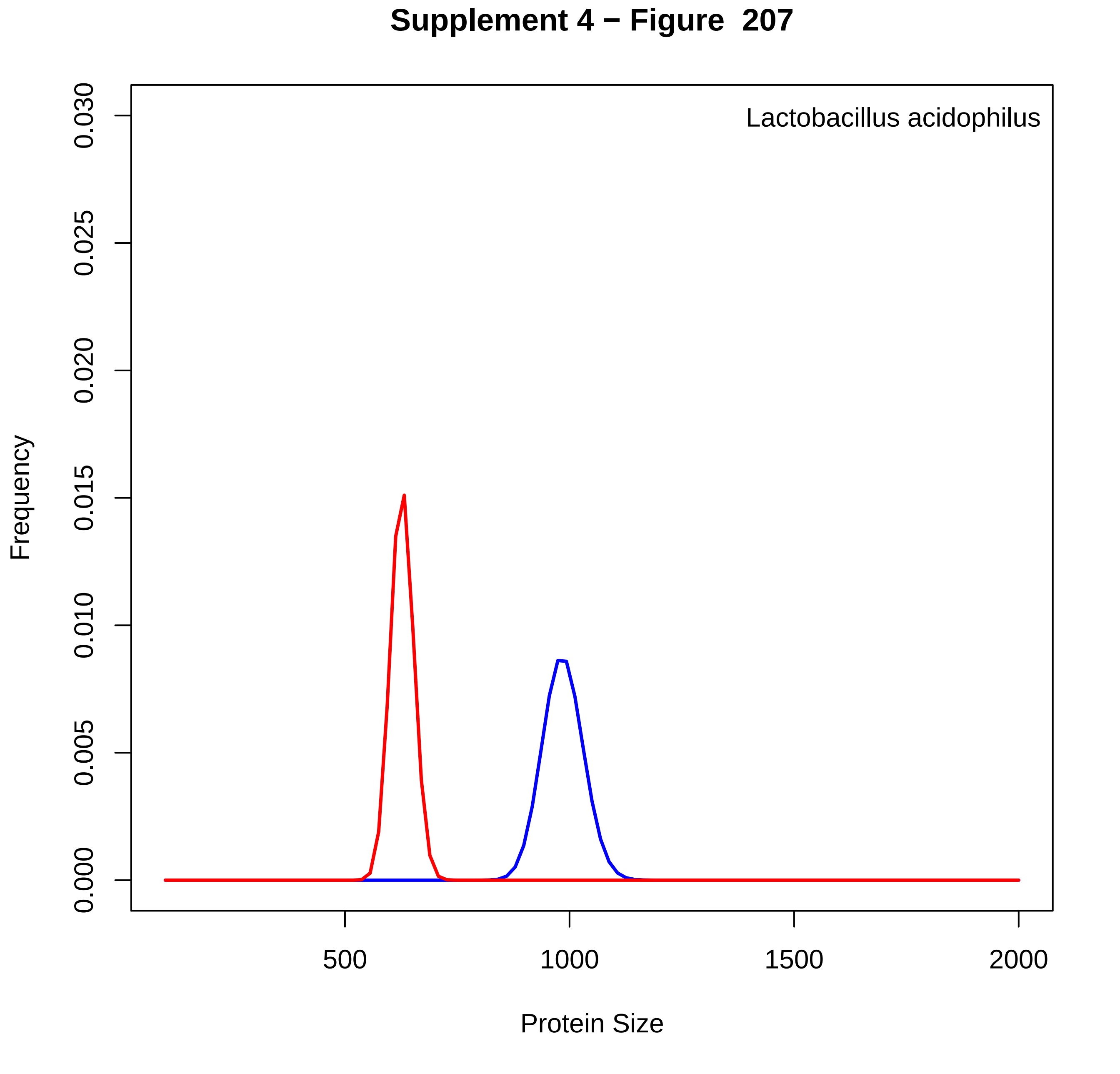

Supplement 4 − Figure 207
Lactobacillus acidophilus
Frequency
0.000
0.005
0.010
0.015
0.020
0.025
0.030
500
1000
1500
2000
Protein Size

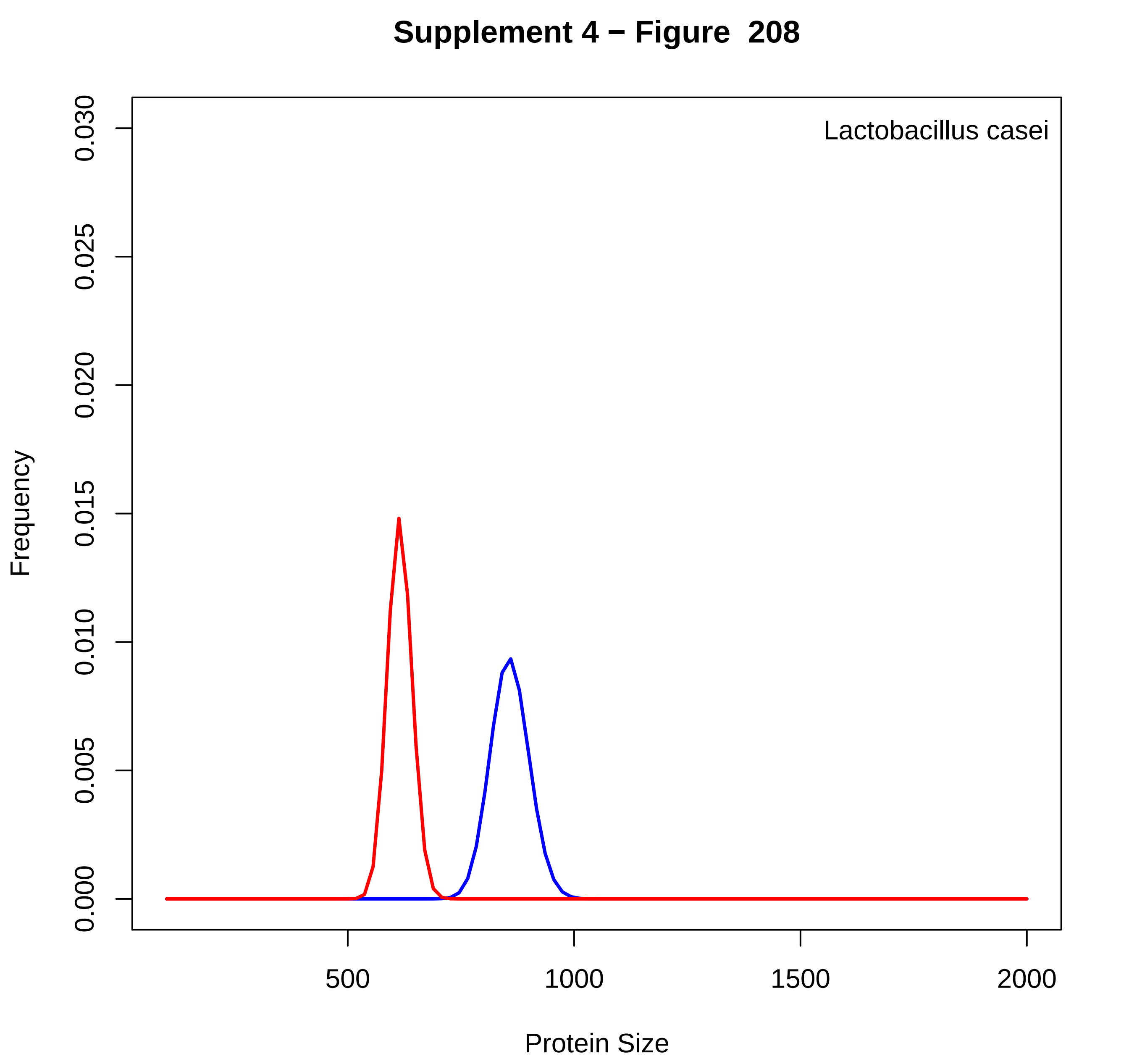
Supplement 4 − Figure 208
Lactobacillus casei
Frequency
0.000
0.005
0.010
0.015
0.020
0.025
0.030
500
1000
1500
2000
Protein Size

Supplement 4 − Figure 209

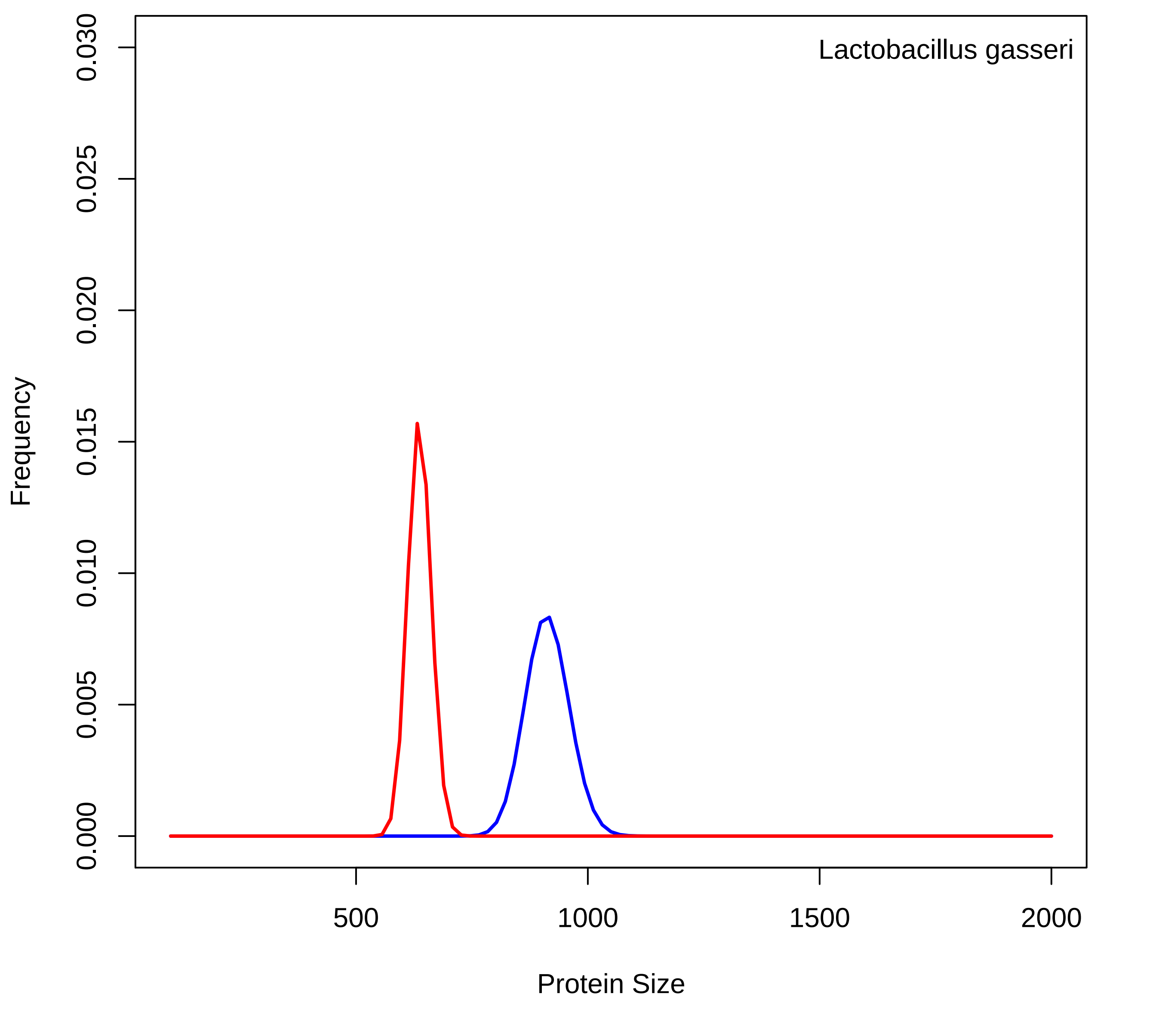

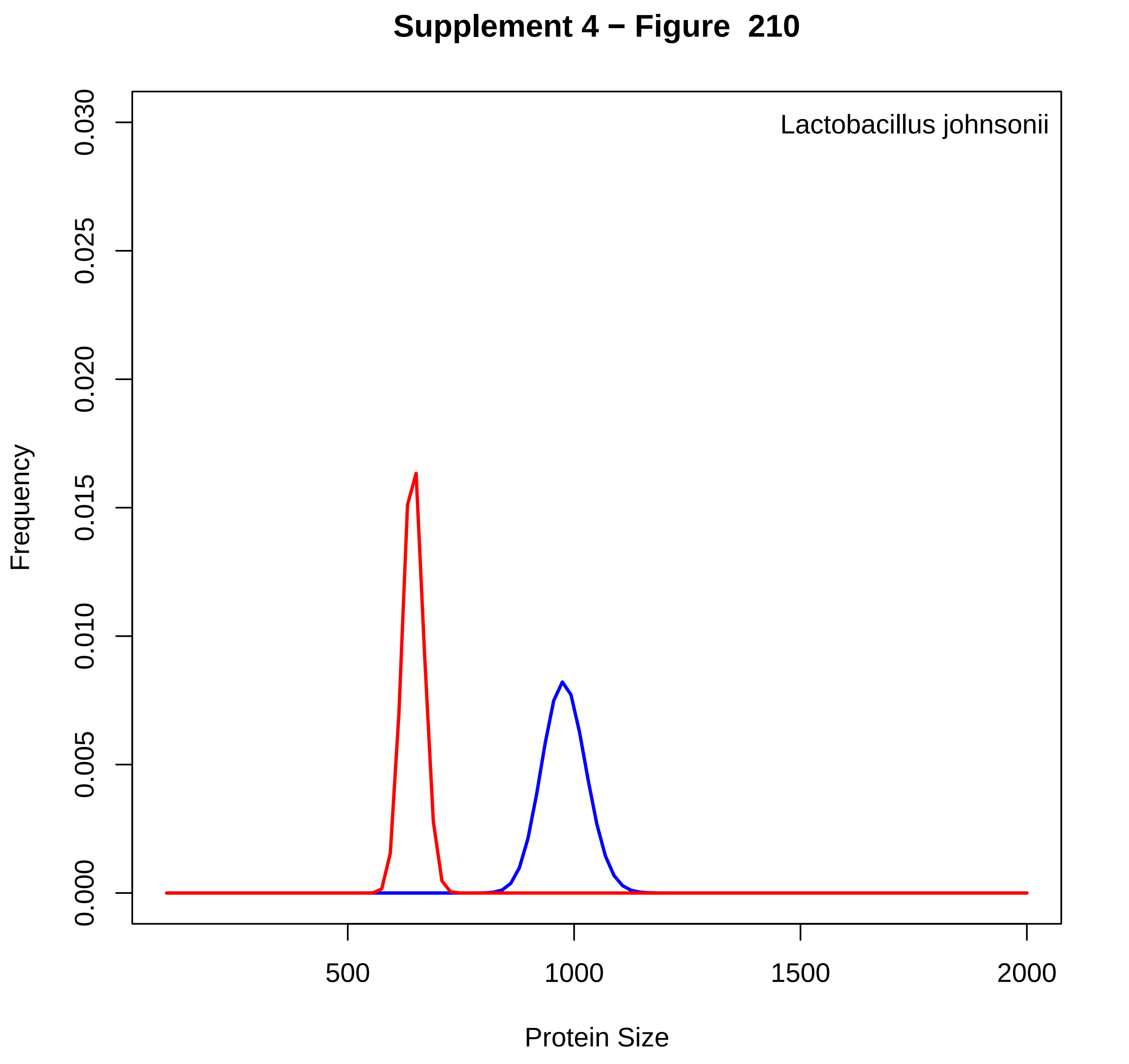

Supplement 4 − Figure 210
Lactobacillus johnsonii
Frequency
Protein Size
0.000
0.005
0.010
0.015
0.020
0.025
0.030
500
1000
1500
2000

Supplement 4 − Figure 211

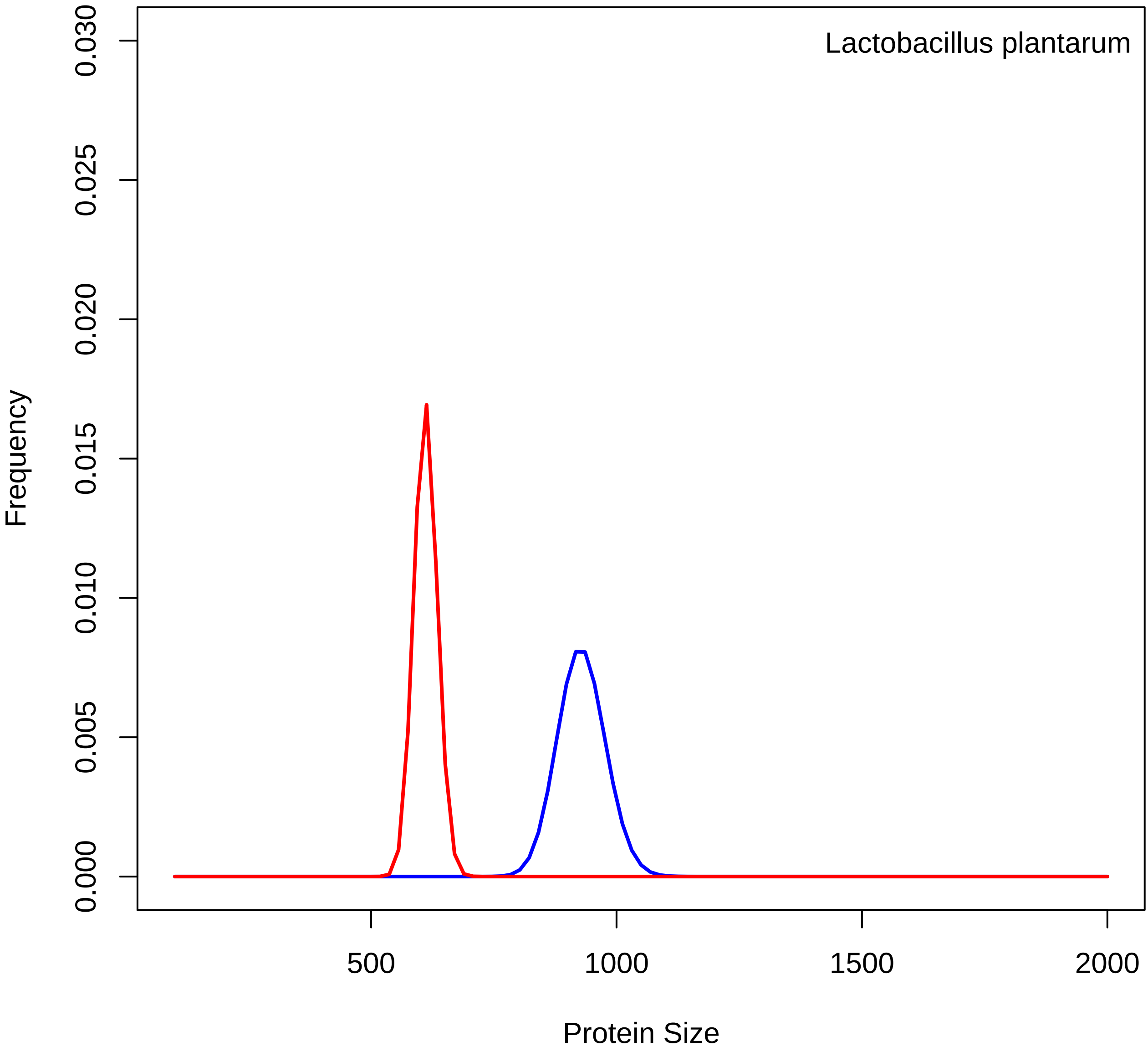

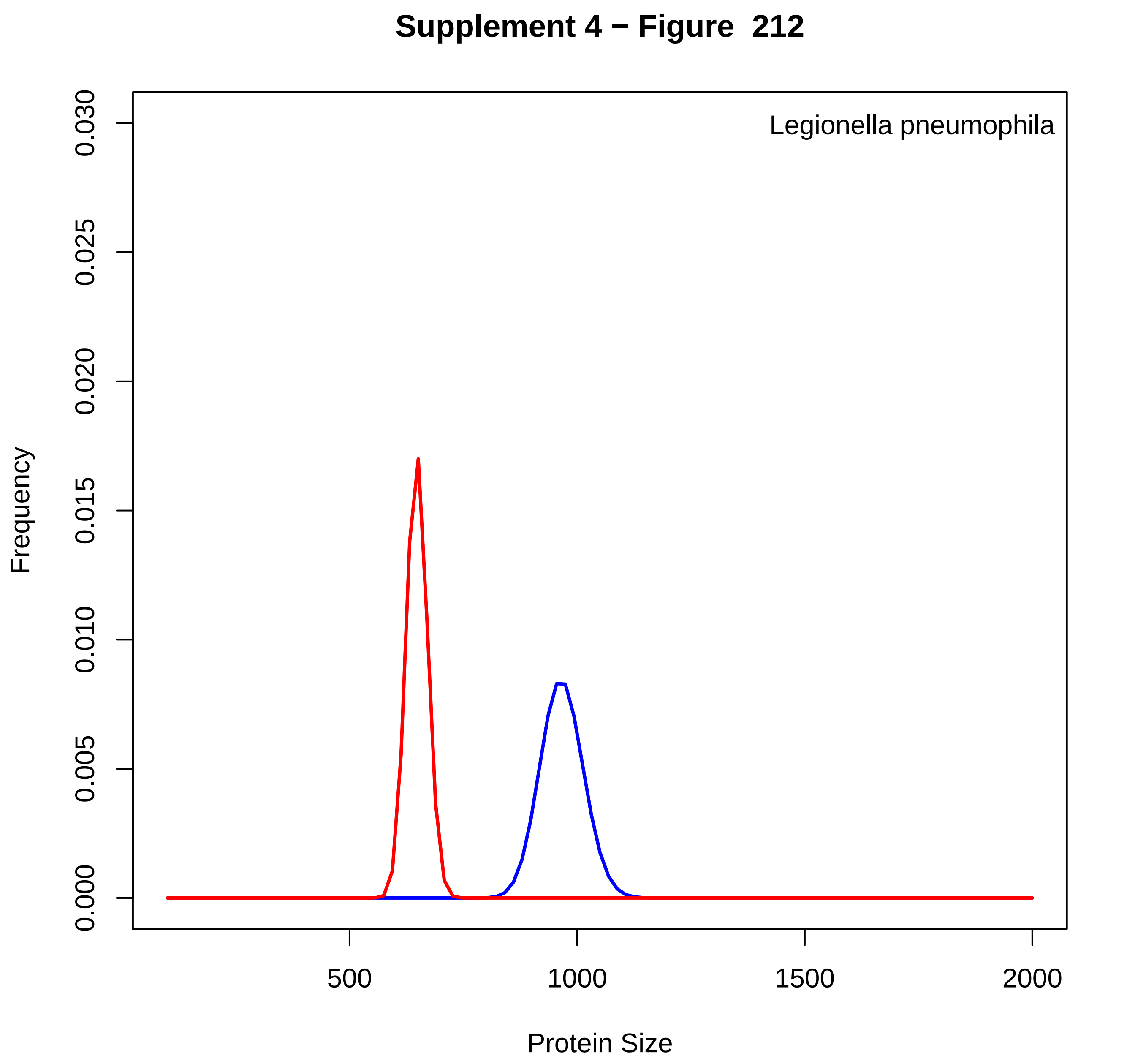
Supplement 4 − Figure 212
Legionella pneumophila
Frequency
0.000
0.005
0.010
0.015
0.020
0.025
0.030
500
1000
1500
2000
Protein Size

Supplement 4 − Figure 213

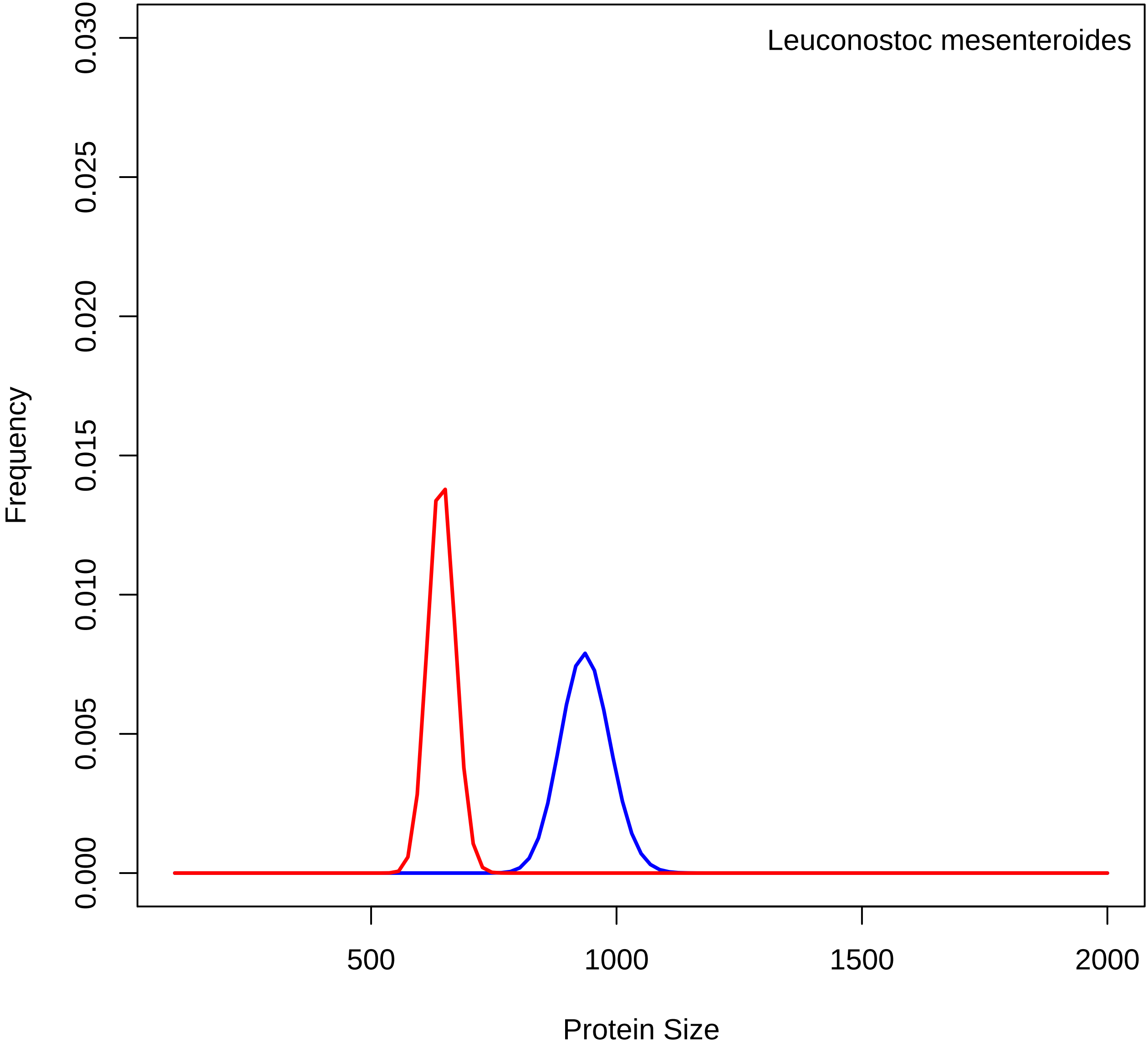

Supplement 4 − Figure 214

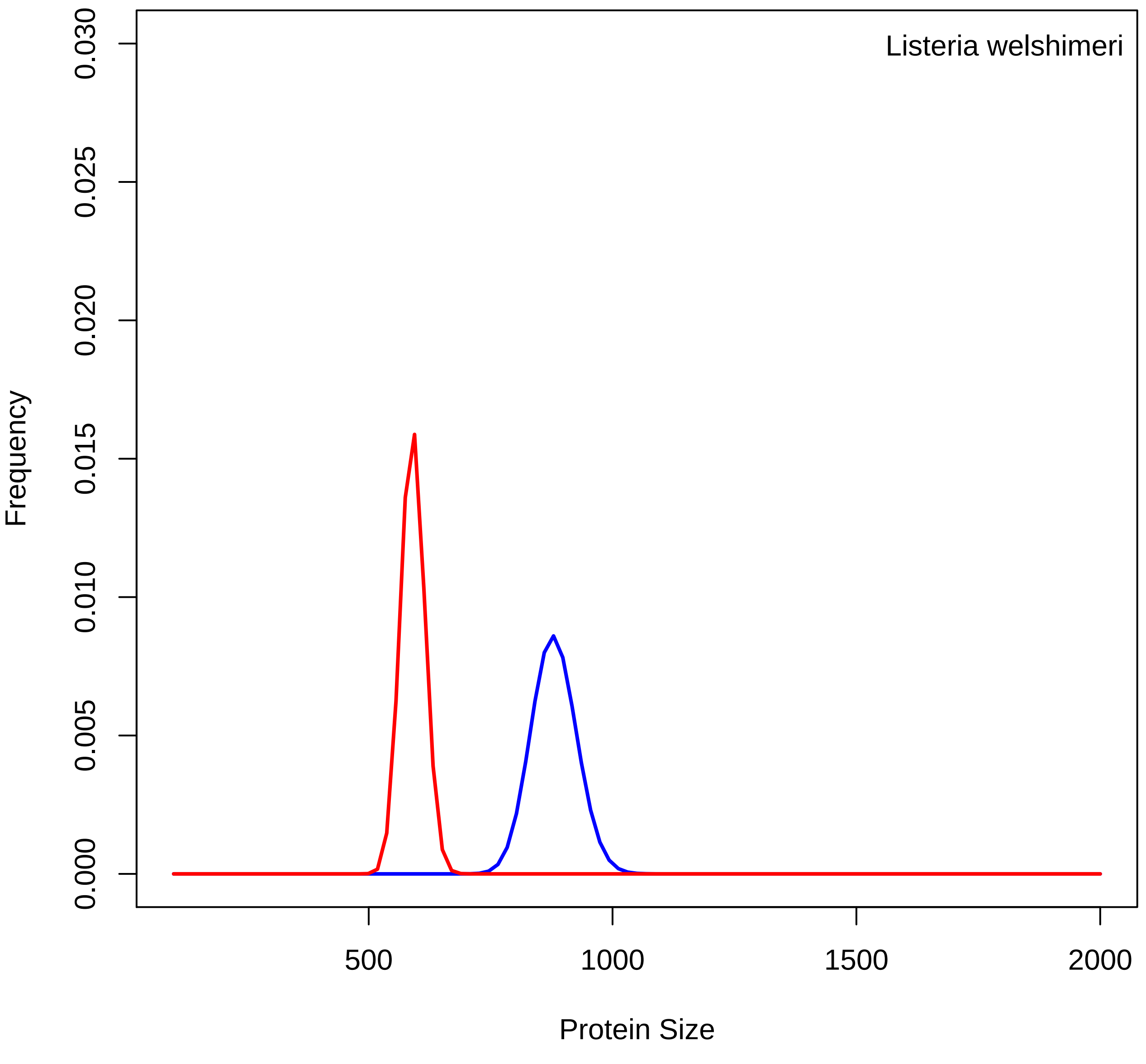

Supplement 4 − Figure 215

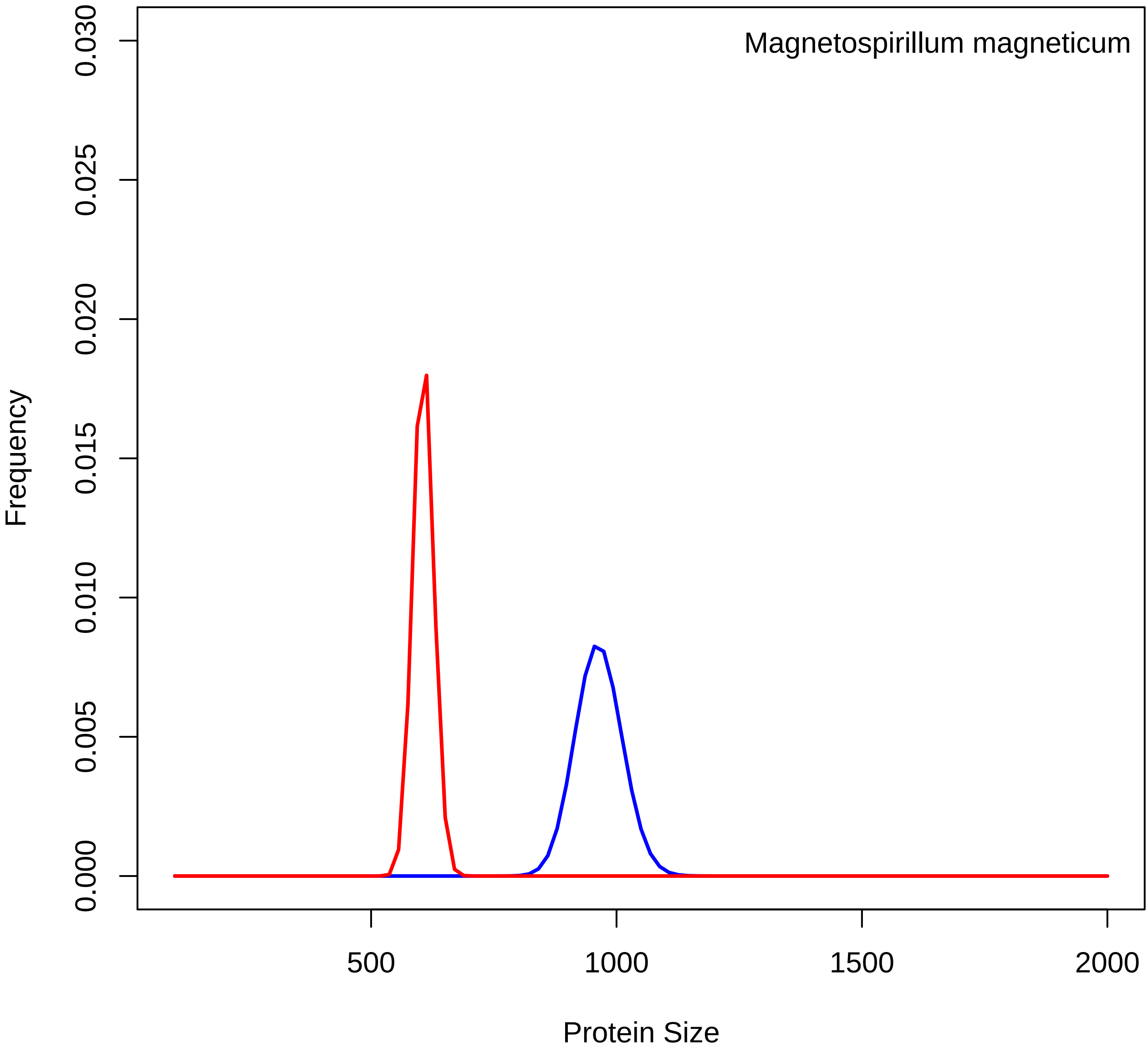

Supplement 4 − Figure 216

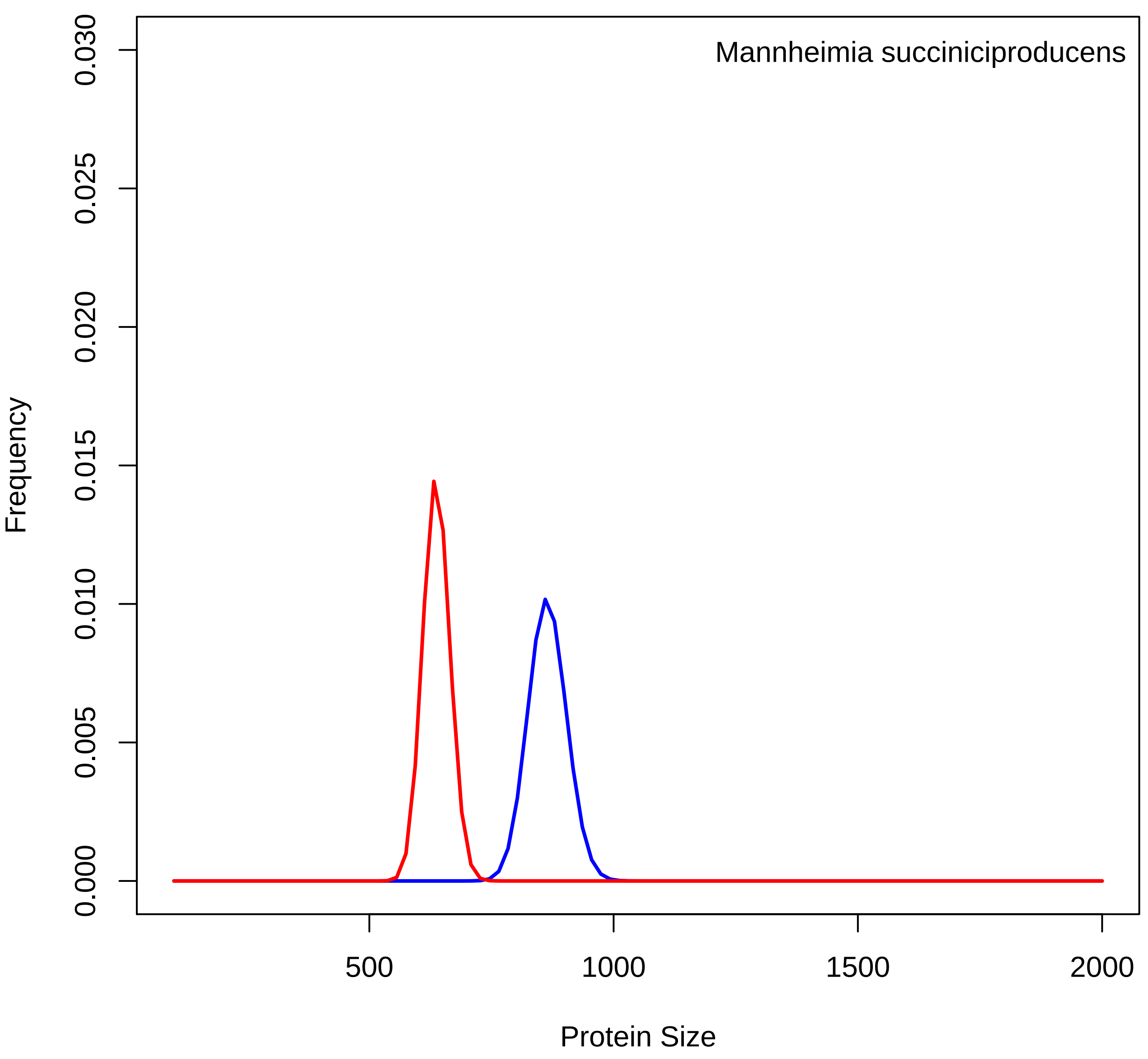

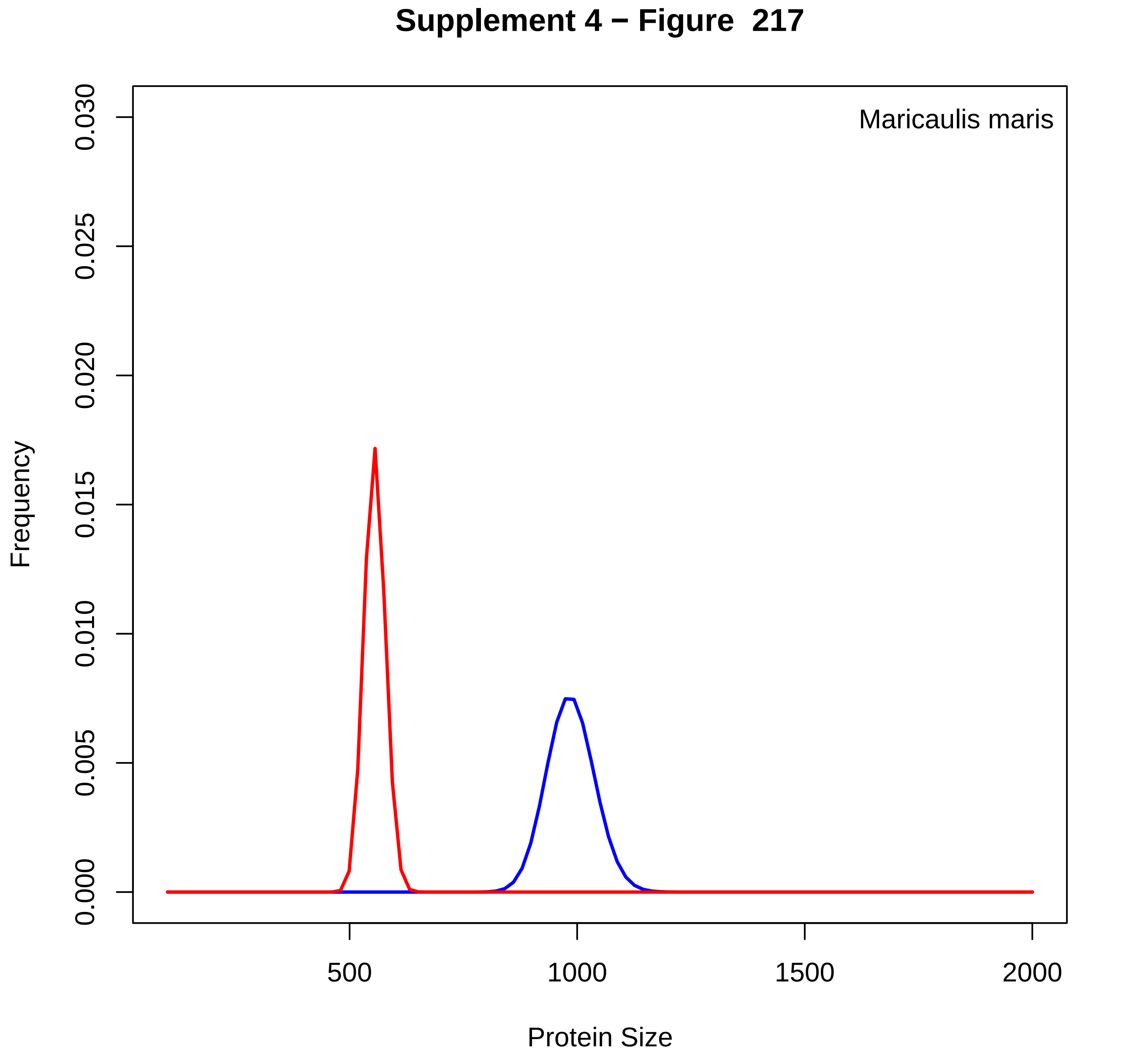

Supplement 4 − Figure 217
Maricaulis maris
Frequency
0.000
0.005
0.010
0.015
0.020
0.025
0.030
500
1000
1500
2000
Protein Size

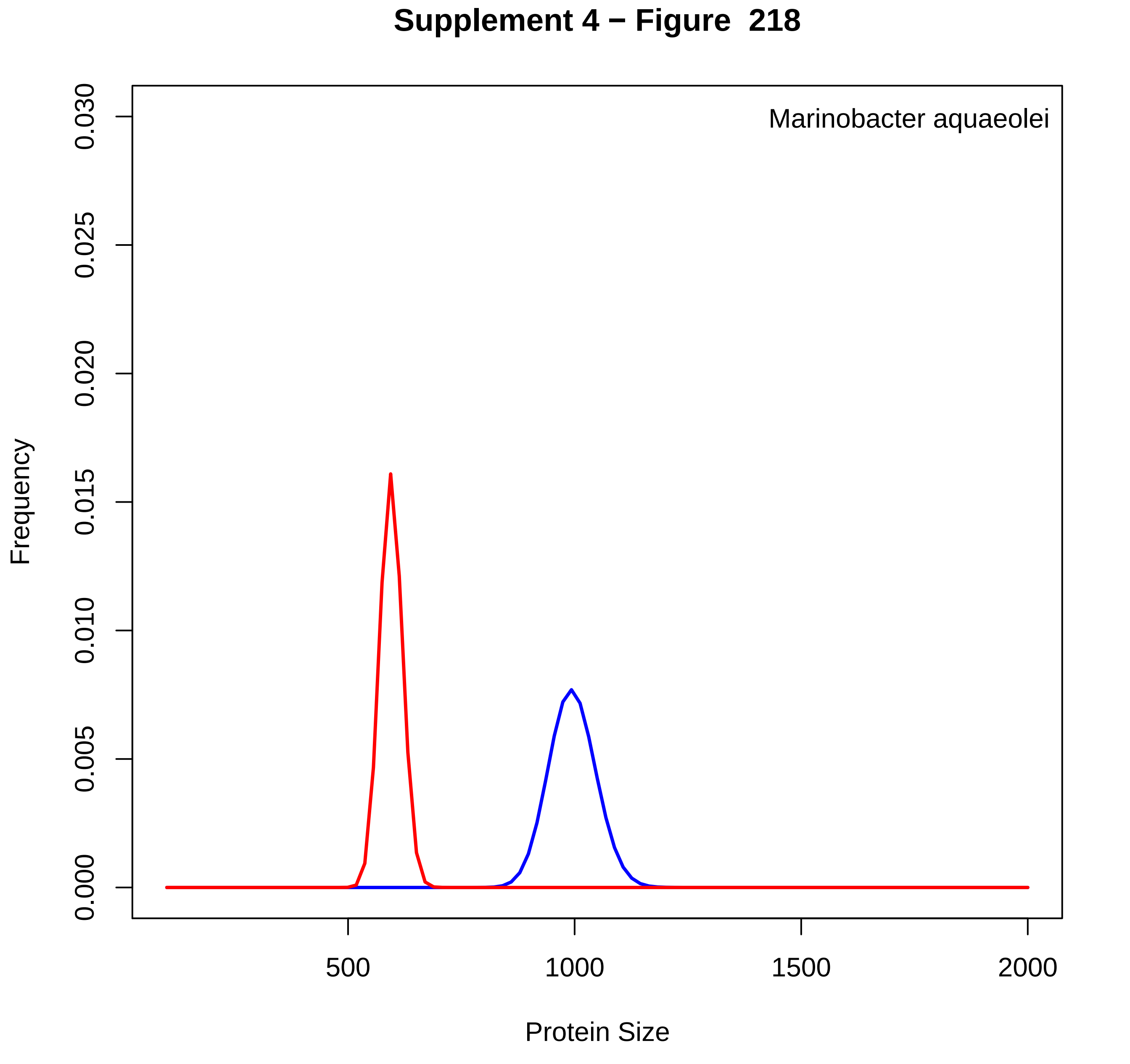
Supplement 4 − Figure 218
Marinobacter aquaeolei
Frequency
0.000
0.005
0.010
0.015
0.020
0.025
0.030
500
1000
1500
2000
Protein Size

Supplement 4 − Figure 219

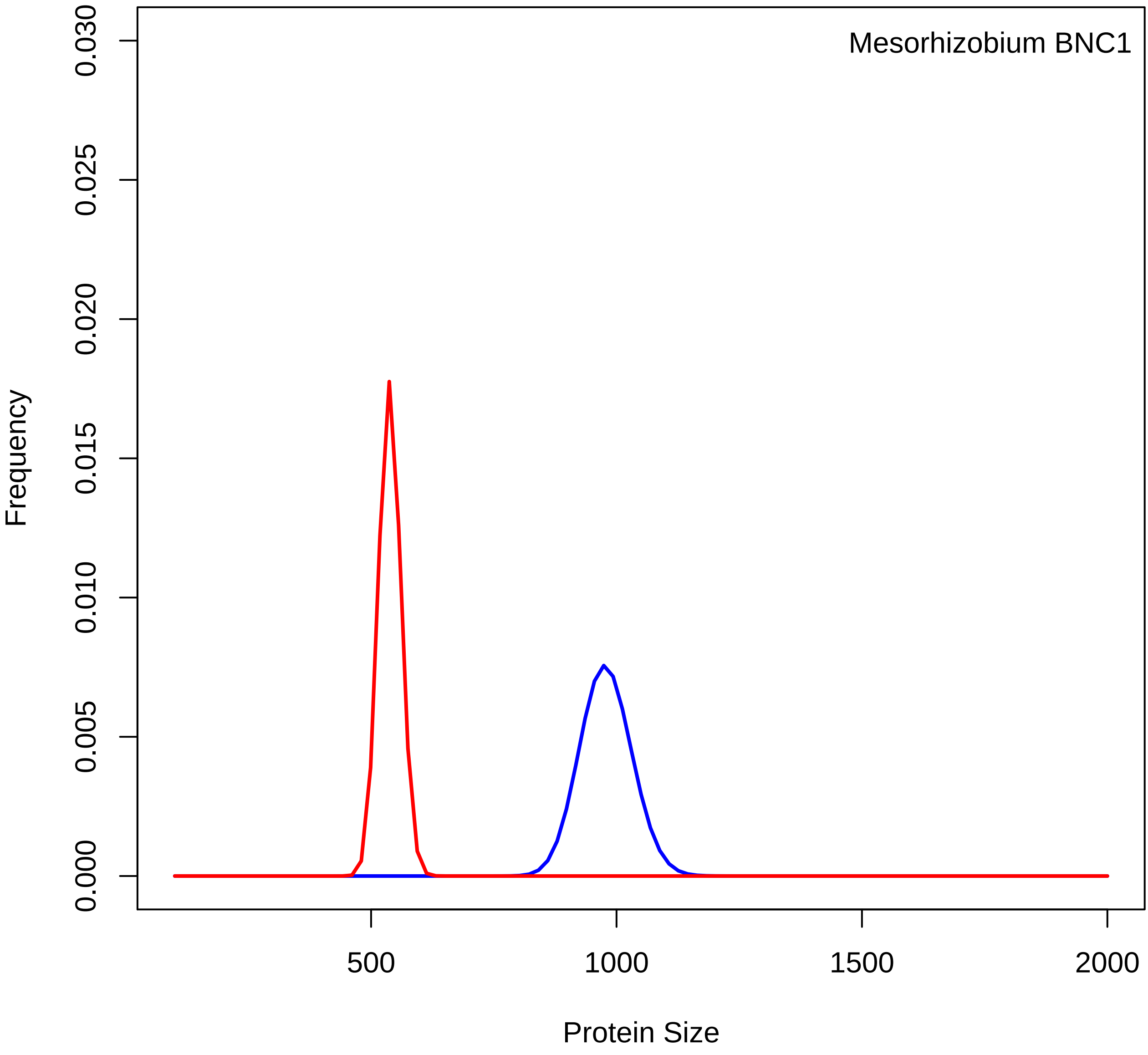

Supplement 4 − Figure 220

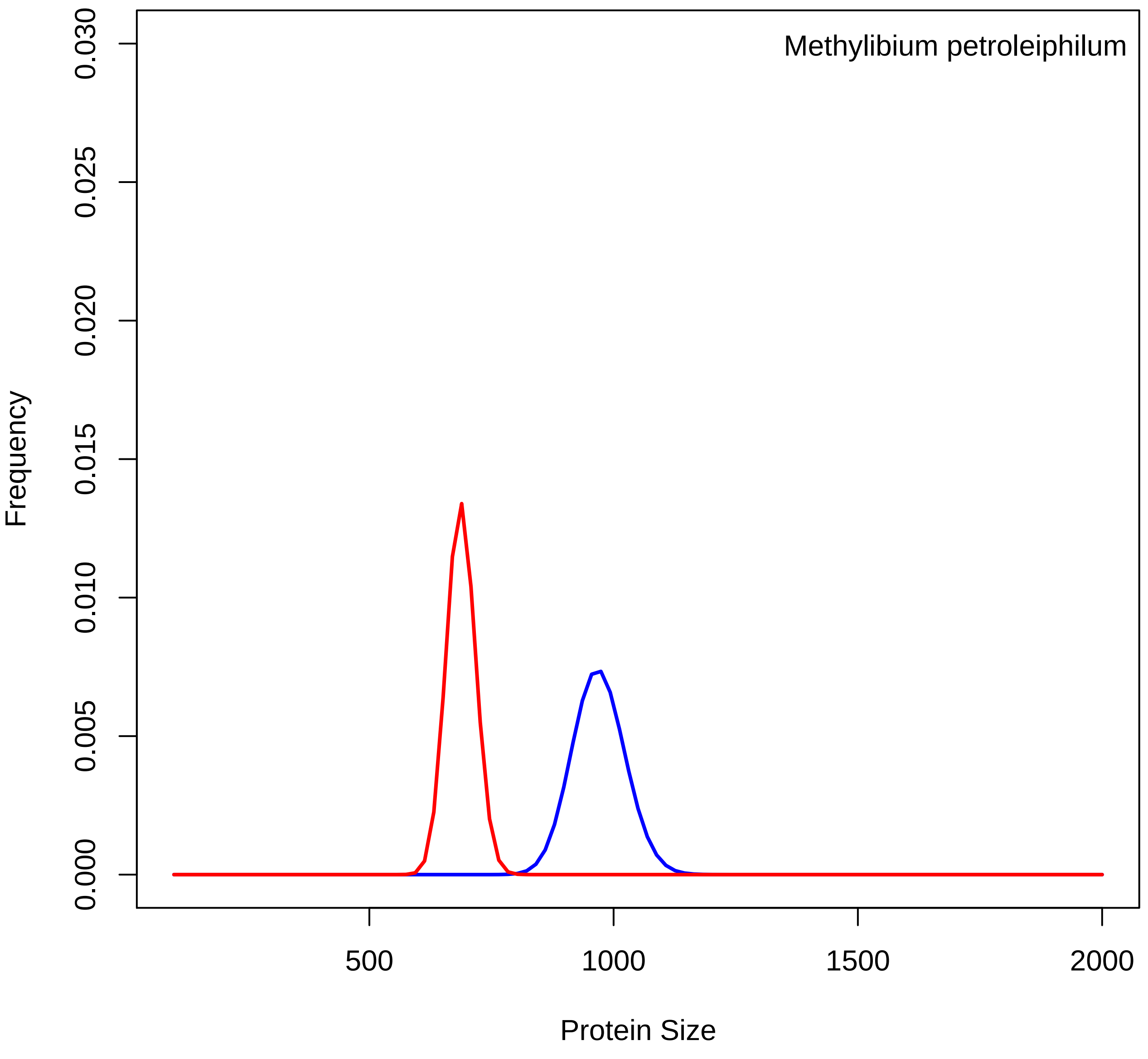

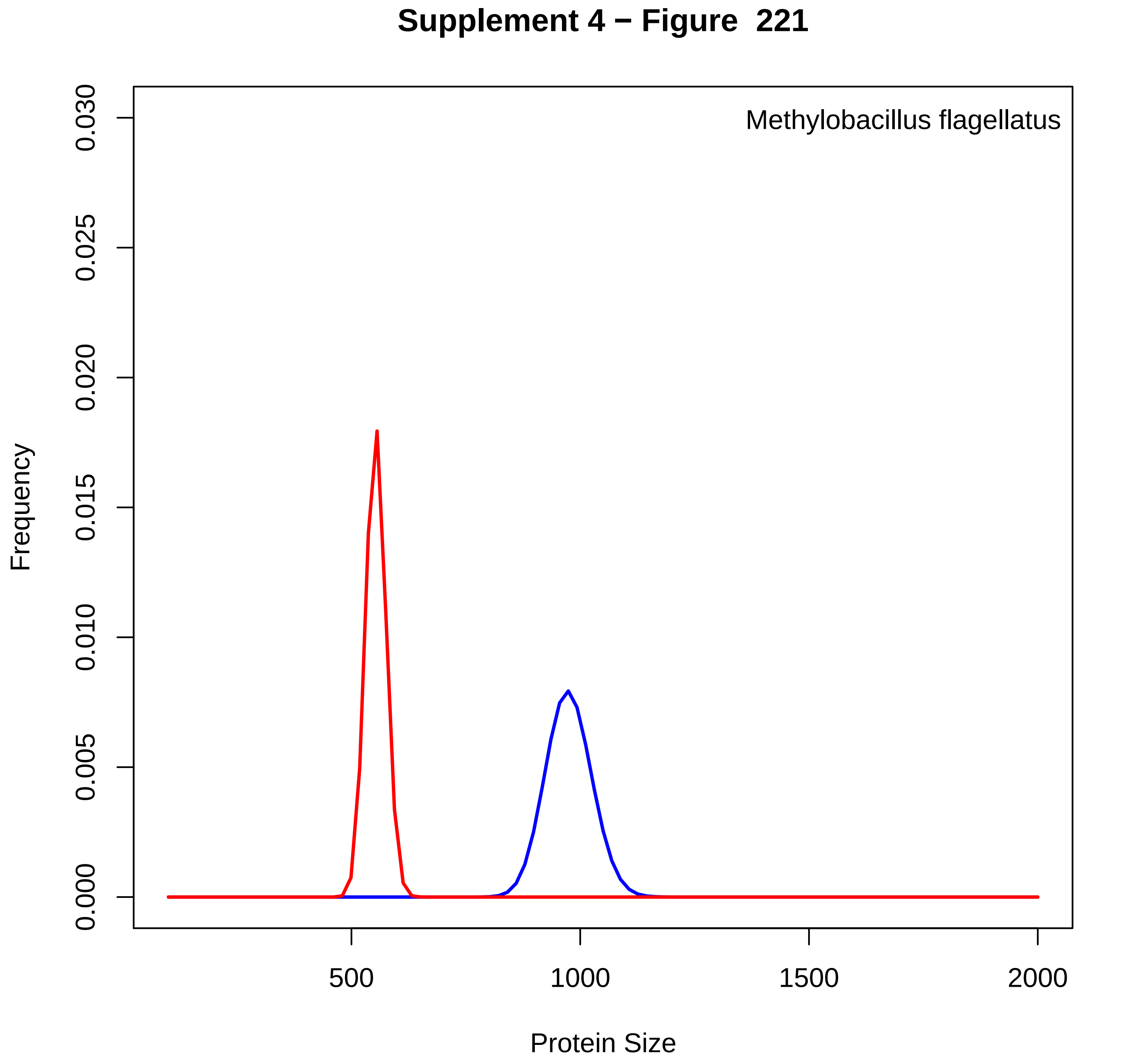

Supplement 4 − Figure 221
Methylobacillus flagellatus
Frequency
0.000
0.005
0.010
0.015
0.020
0.025
0.030
500
1000
1500
2000
Protein Size

Supplement 4 − Figure 222

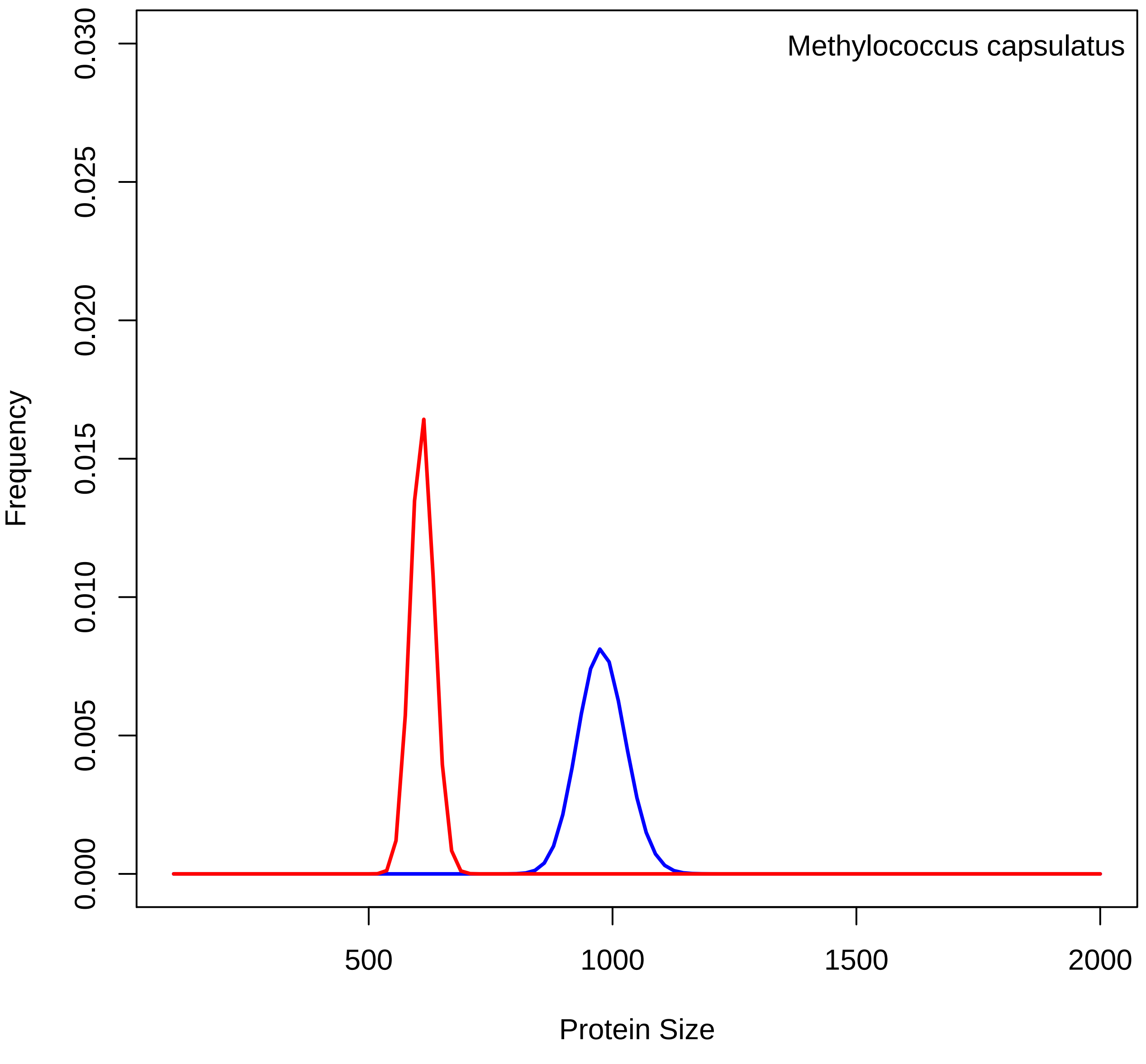

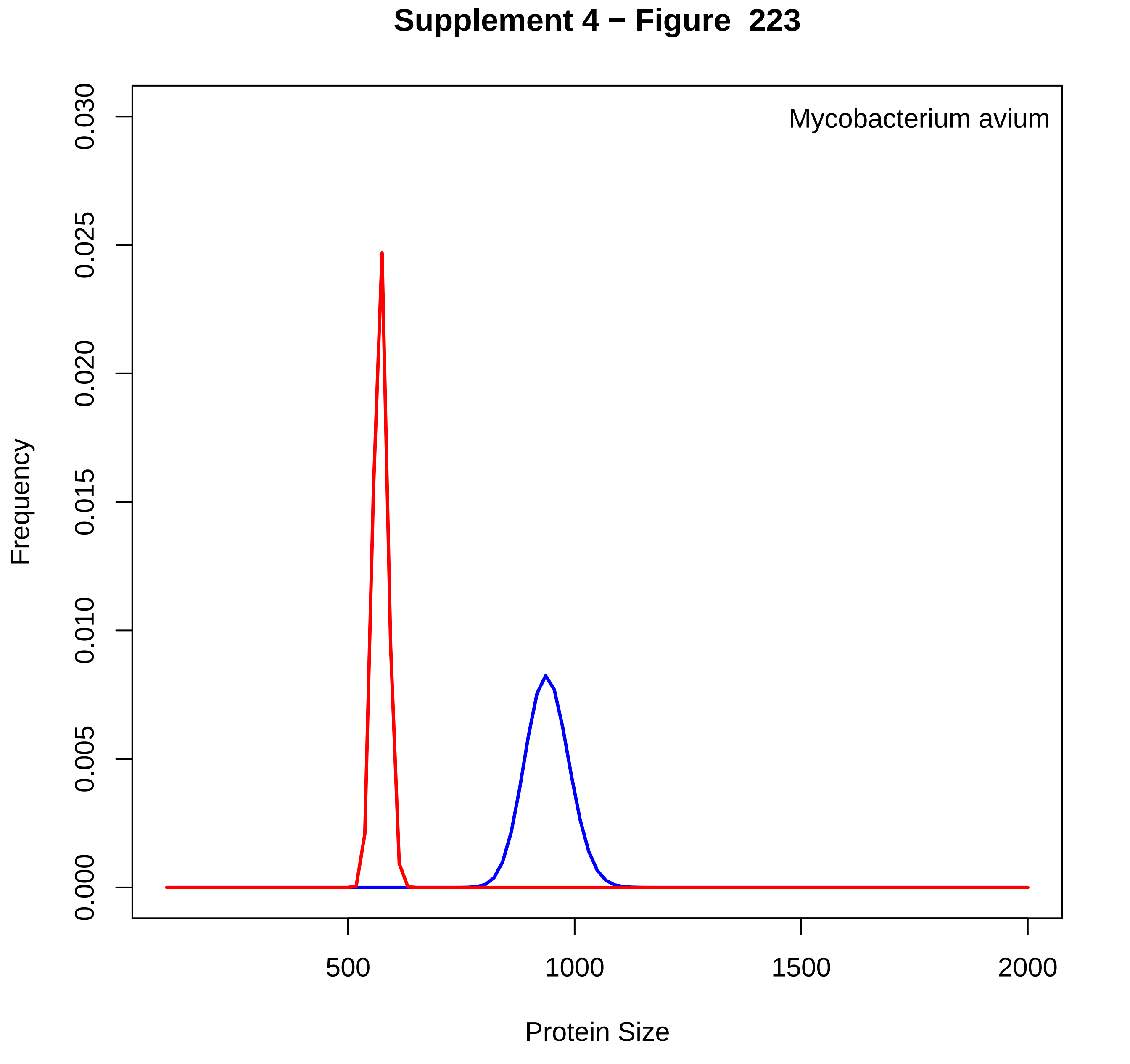
Supplement 4 − Figure 223
Mycobacterium avium
Frequency
Protein Size
0.000
0.005
0.010
0.015
0.020
0.025
0.030
500
1000
1500
2000

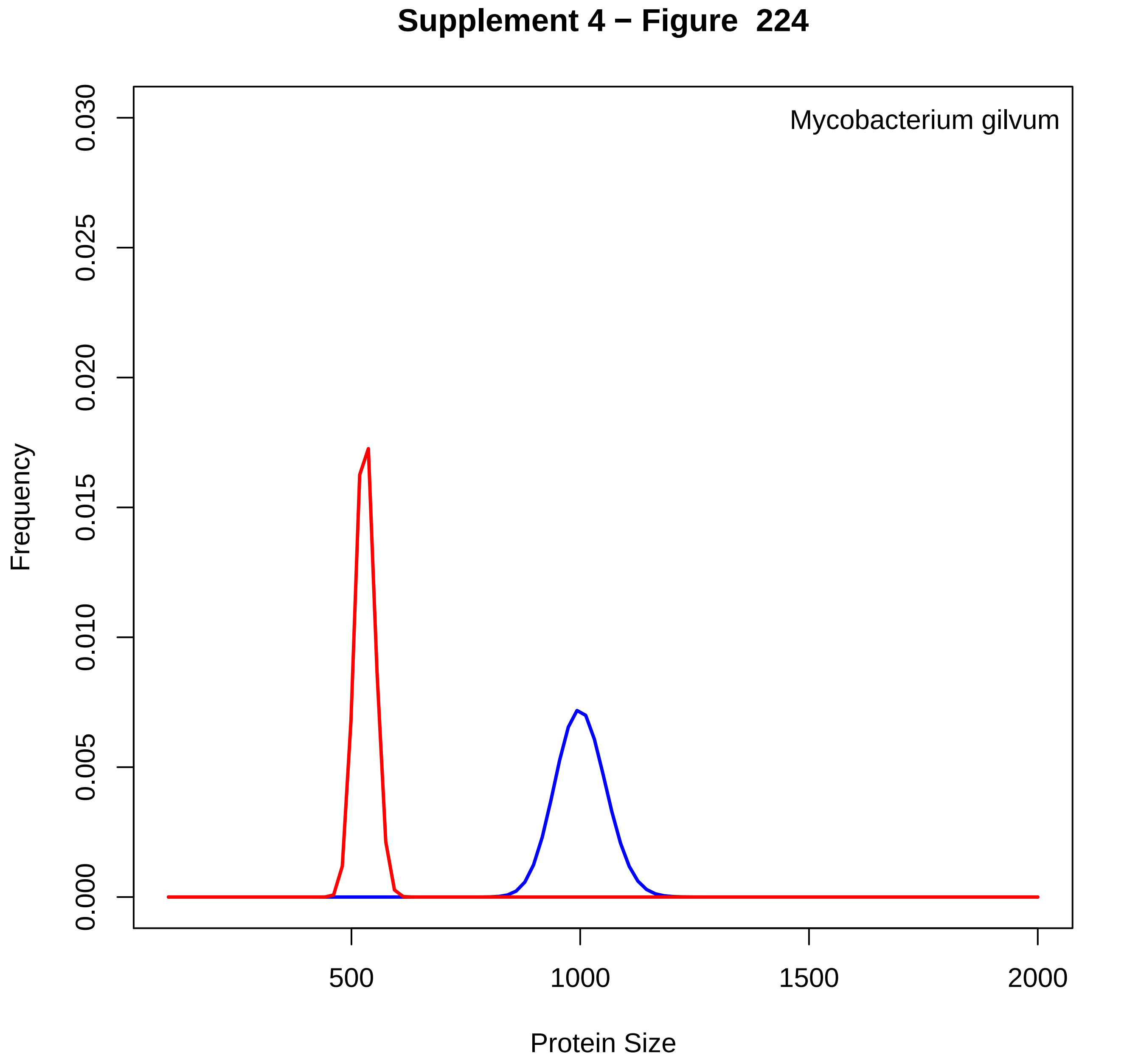
Supplement 4 − Figure 224
Mycobacterium gilvum
Frequency
0.000
0.005
0.010
0.015
0.020
0.025
0.030
500
1000
1500
2000
Protein Size

Supplement 4 − Figure 225

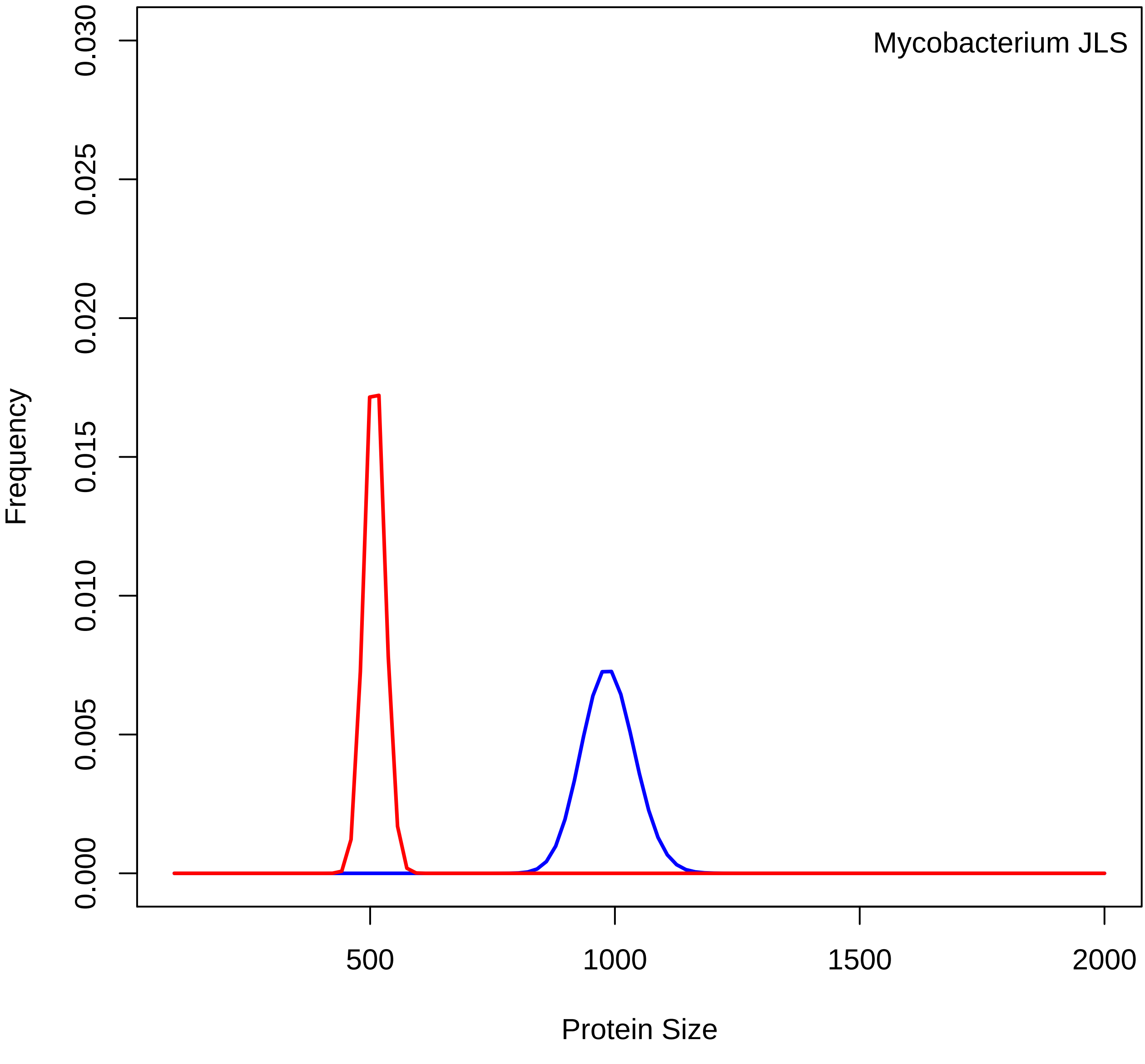

Supplement 4 − Figure 226

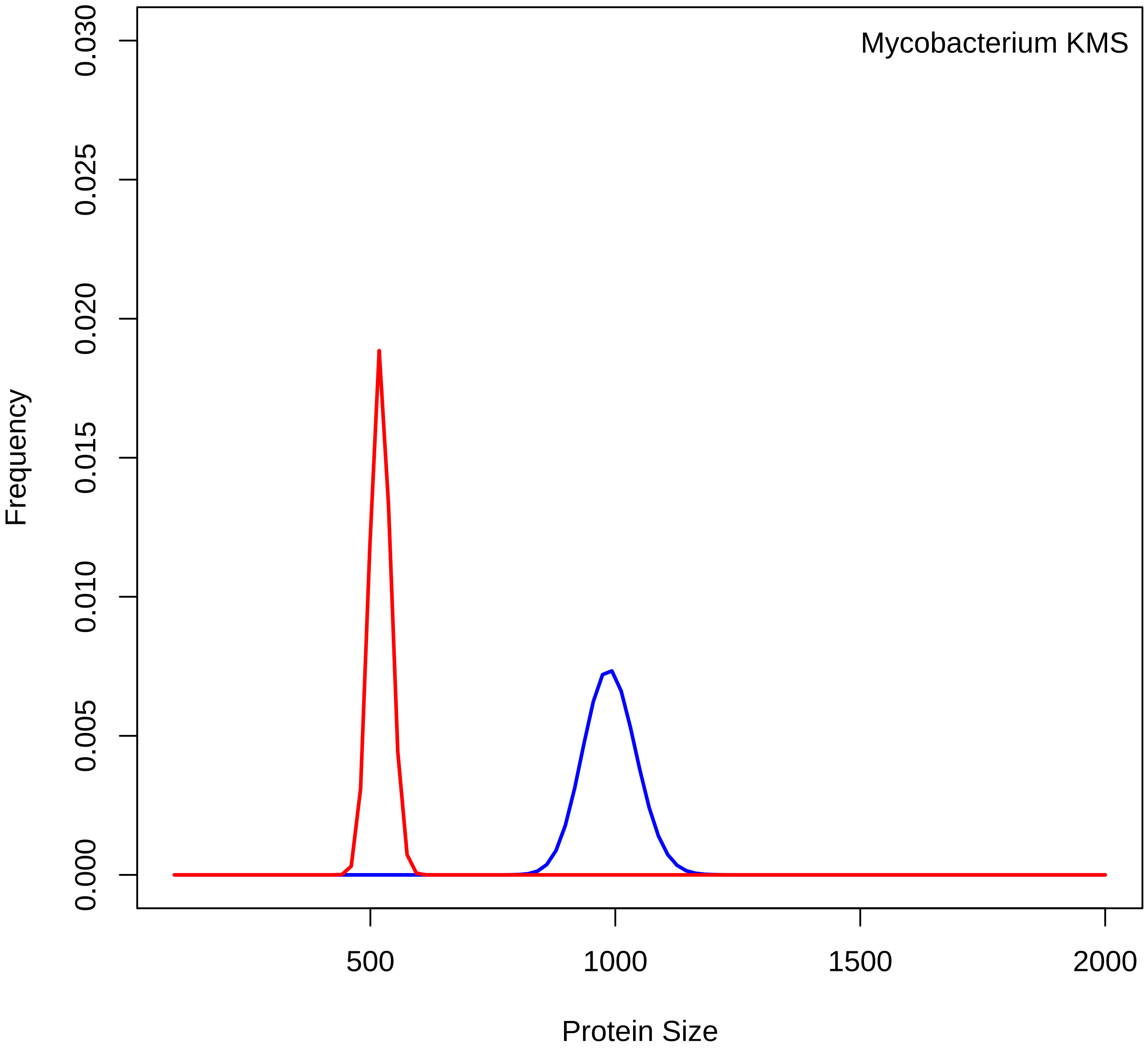

Supplement 4 − Figure 227

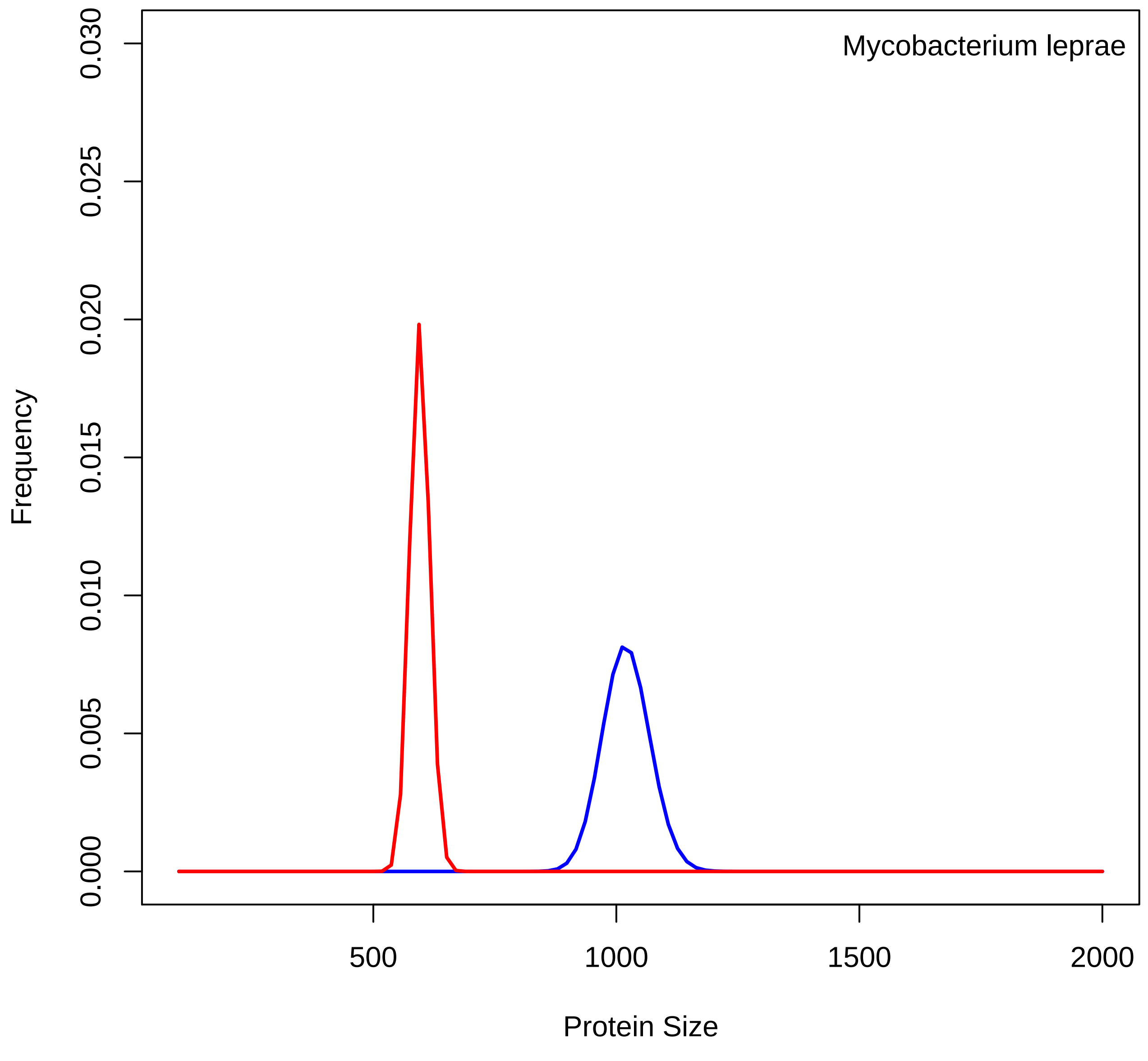

Supplement 4 − Figure 228

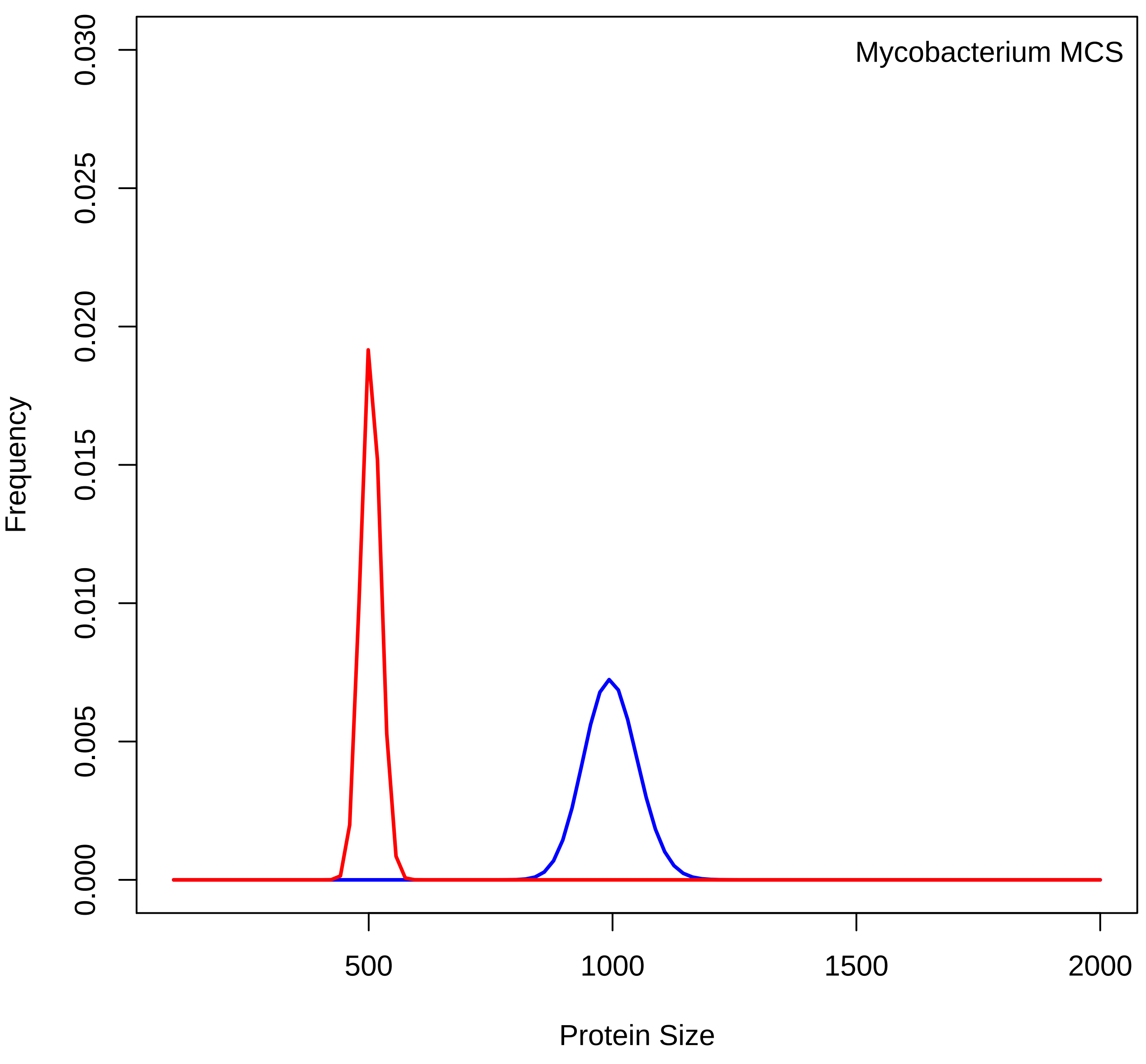

Supplement 4 − Figure 229

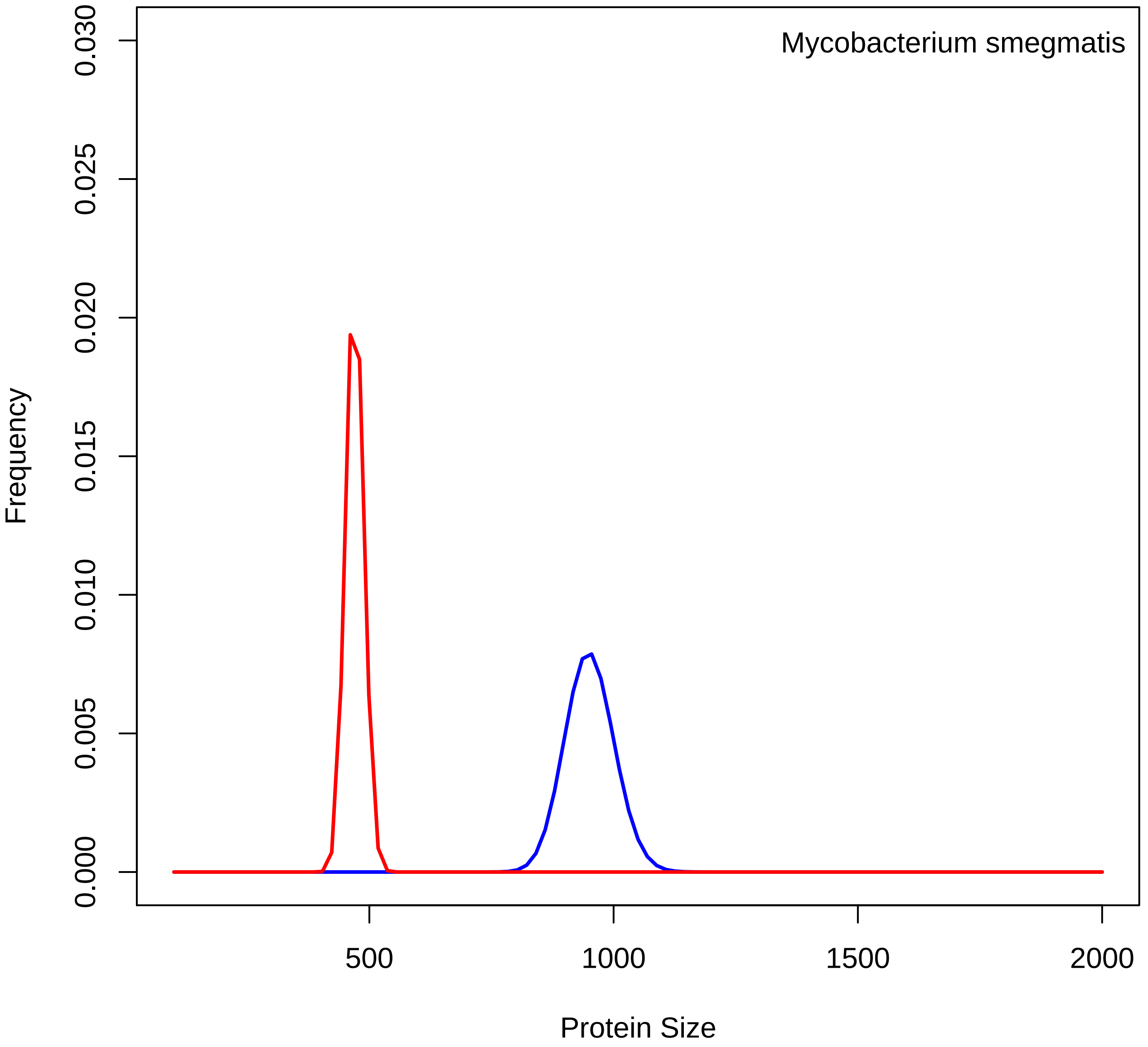

Supplement 4 − Figure 230

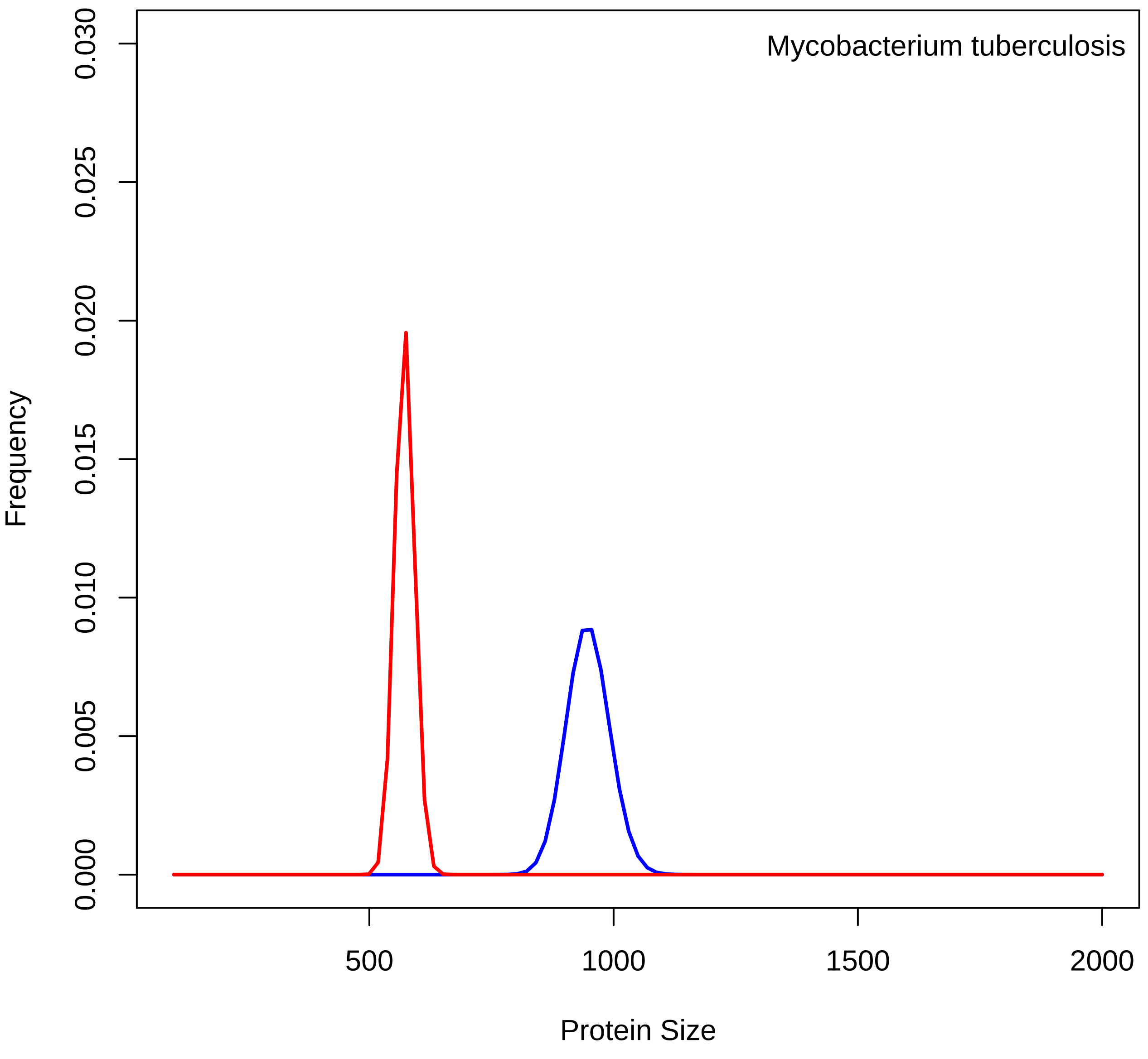

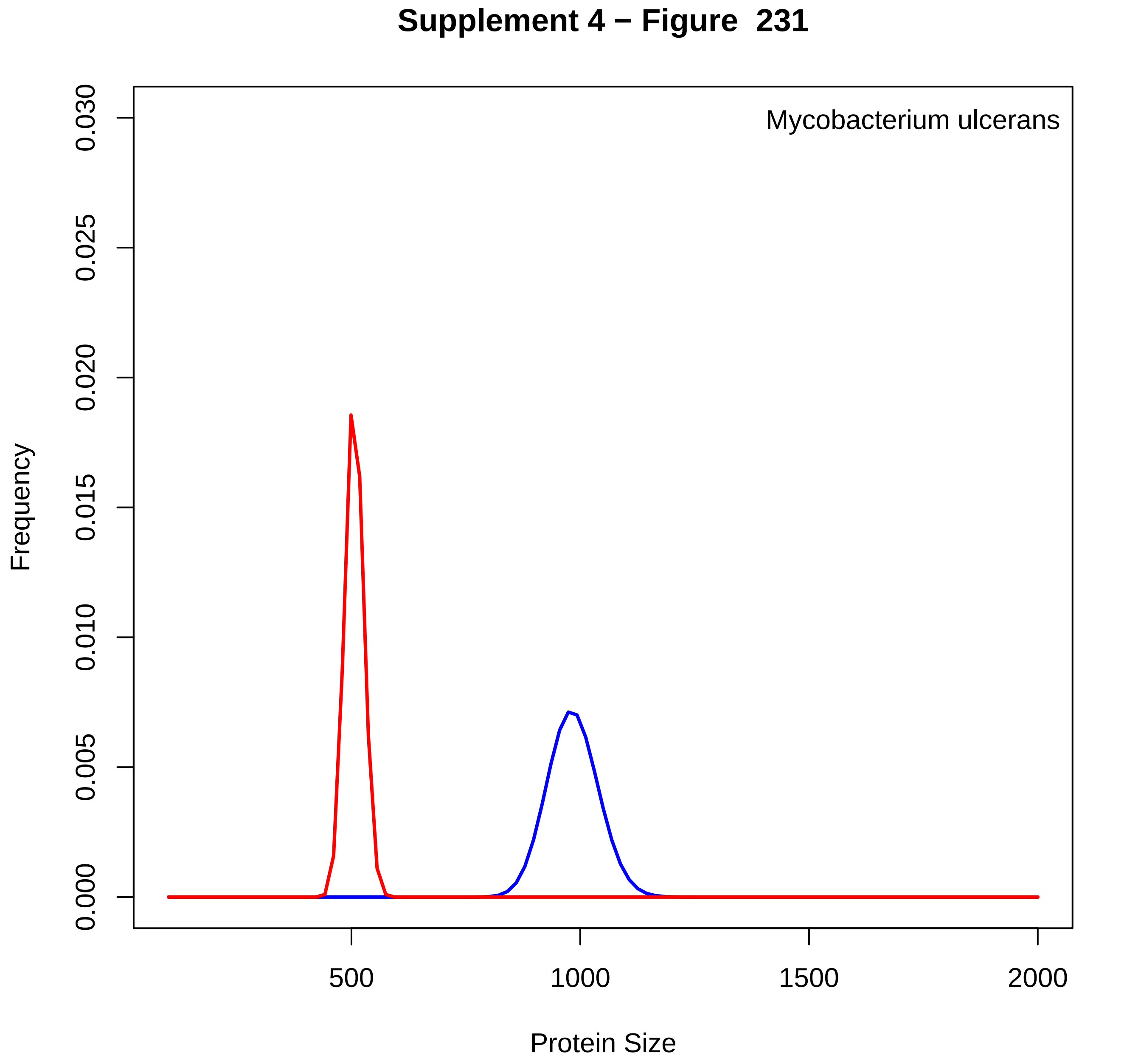

Supplement 4 − Figure 231
Mycobacterium ulcerans
Frequency
0.000
0.005
0.010
0.015
0.020
0.025
0.030
500
1000
1500
2000
Protein Size

Supplement 4 − Figure 232

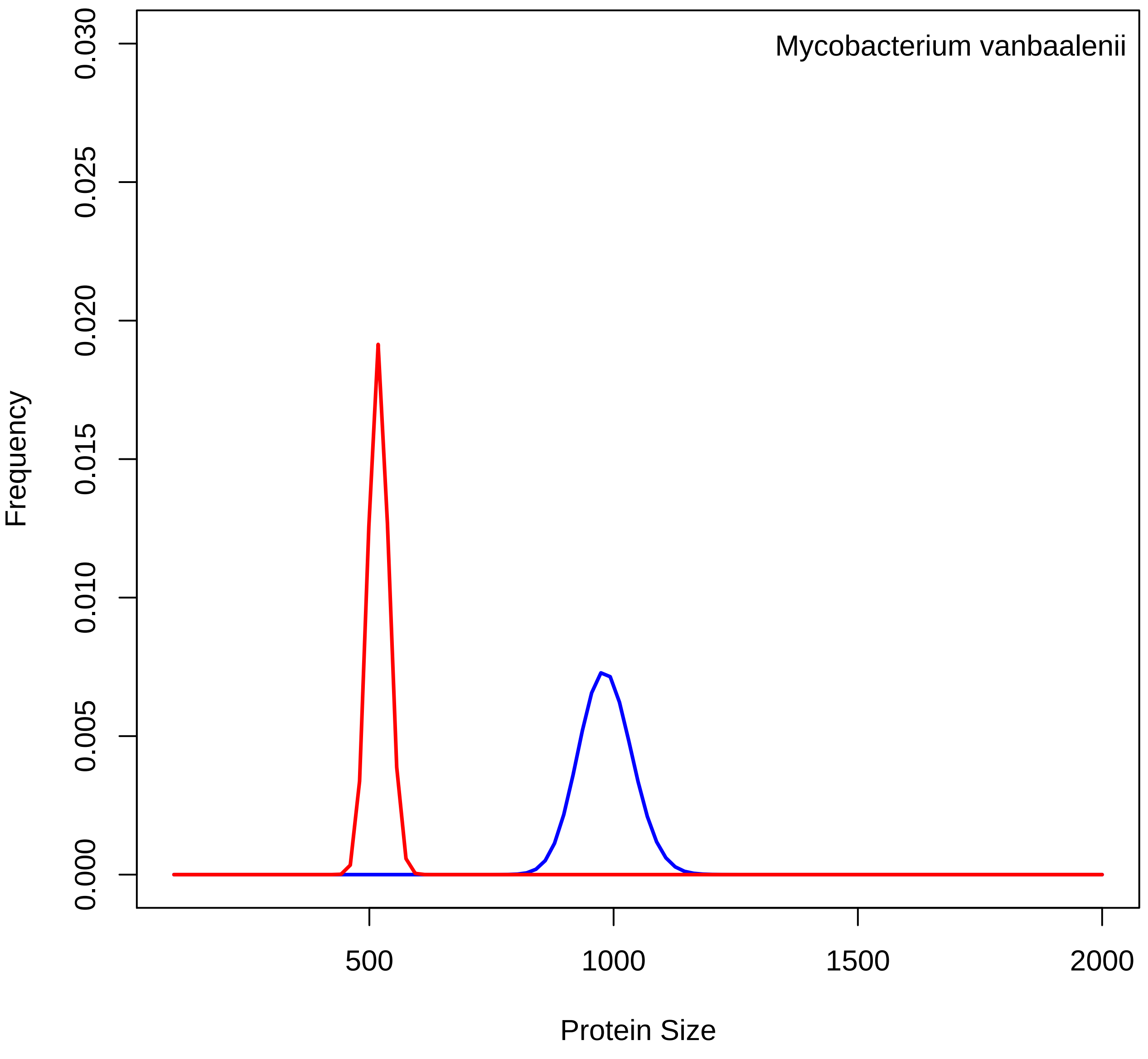

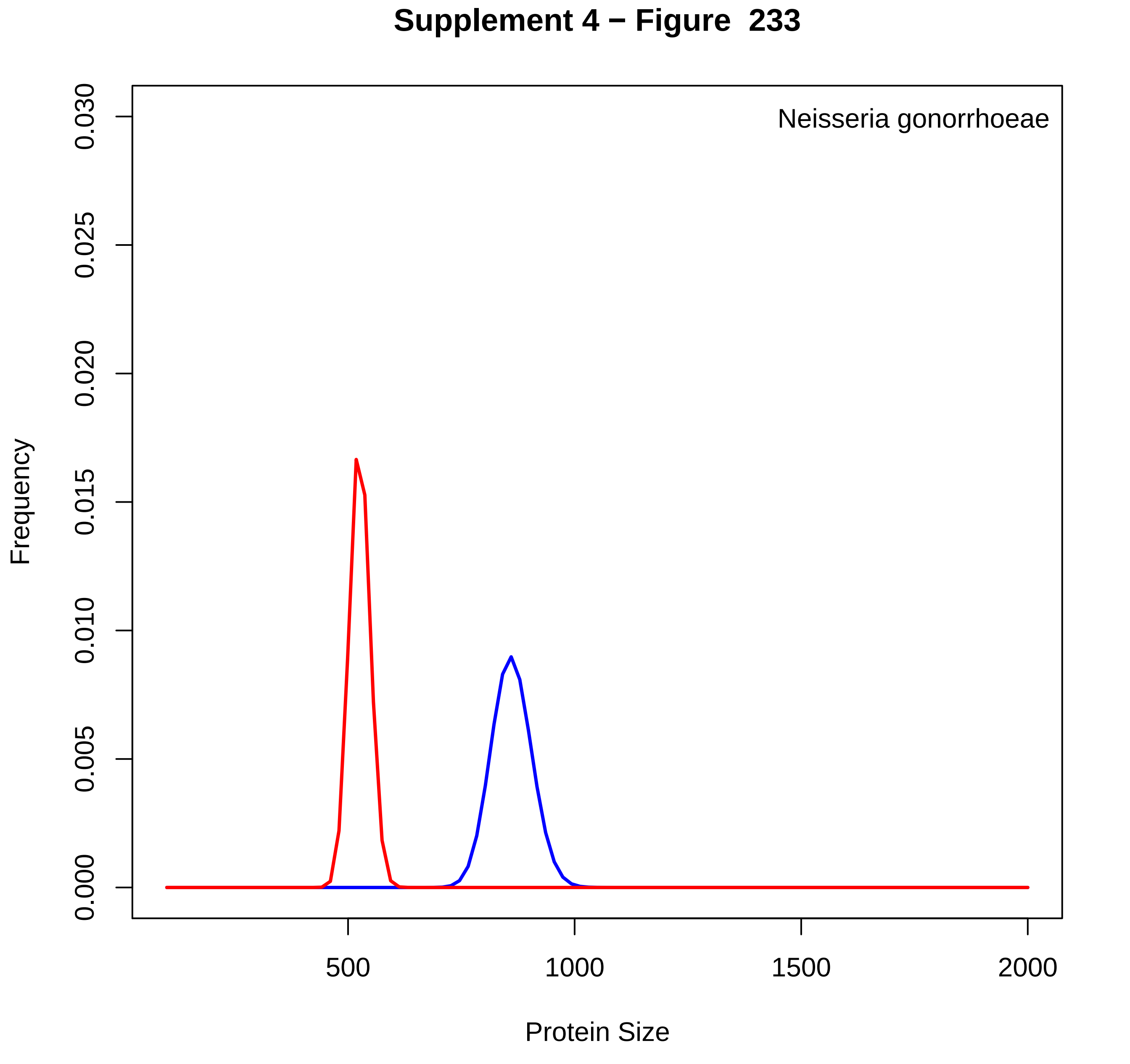
Supplement 4 − Figure 233
Neisseria gonorrhoeae
Frequency
0.000
0.005
0.010
0.015
0.020
0.025
0.030
500
1000
1500
2000
Protein Size

Supplement 4 − Figure 234

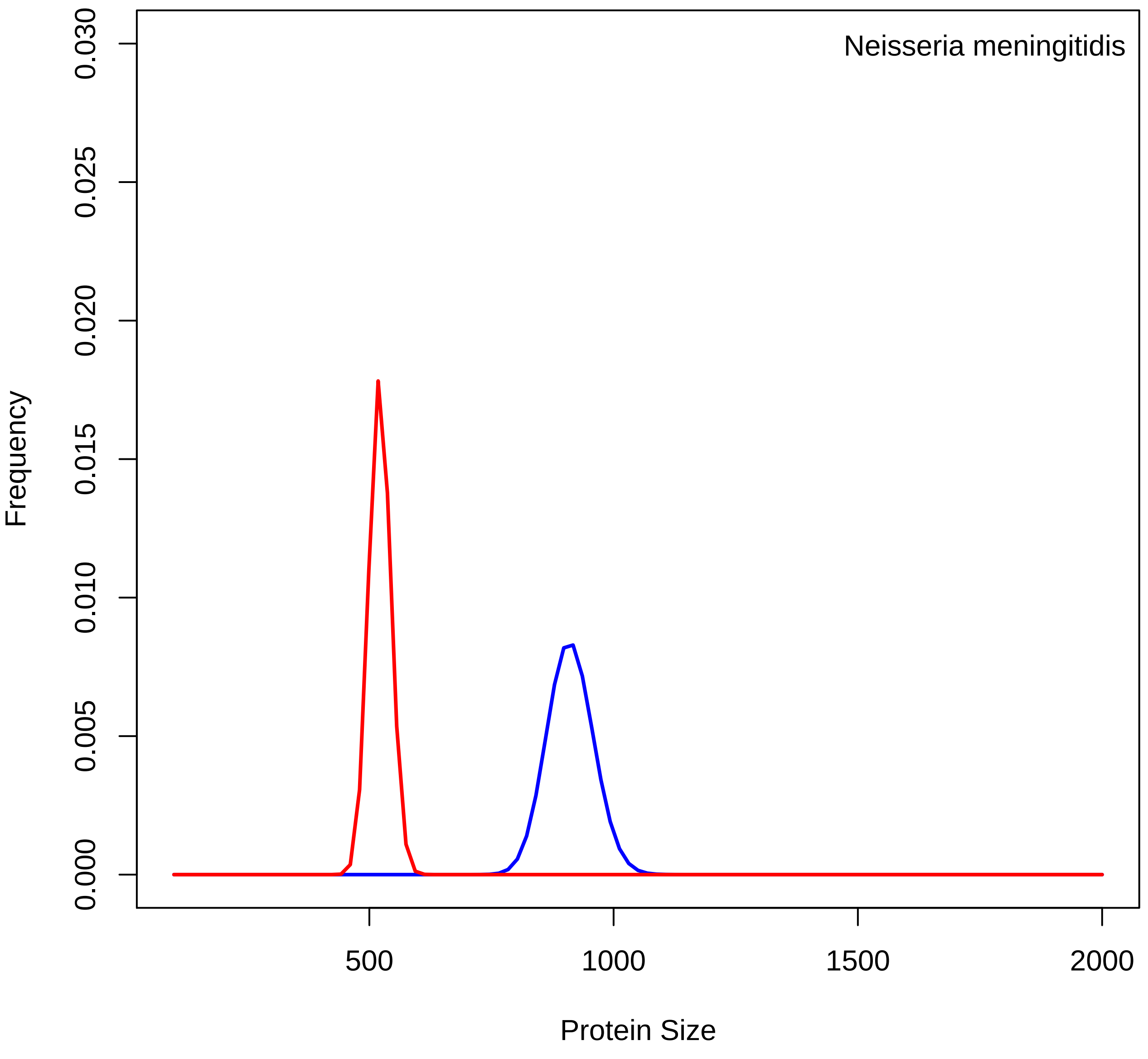

Supplement 4 − Figure 235

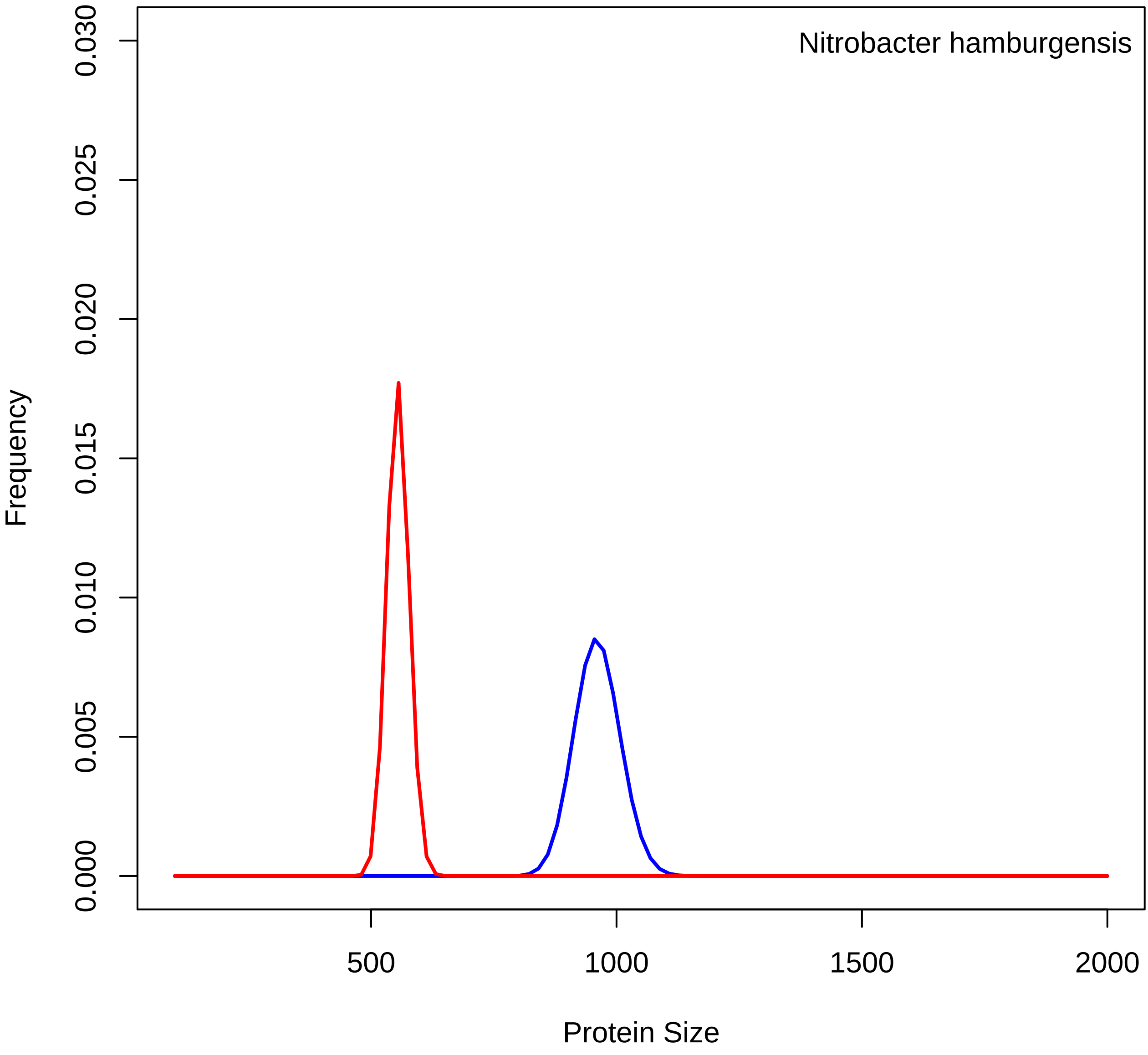

Supplement 4 − Figure 236

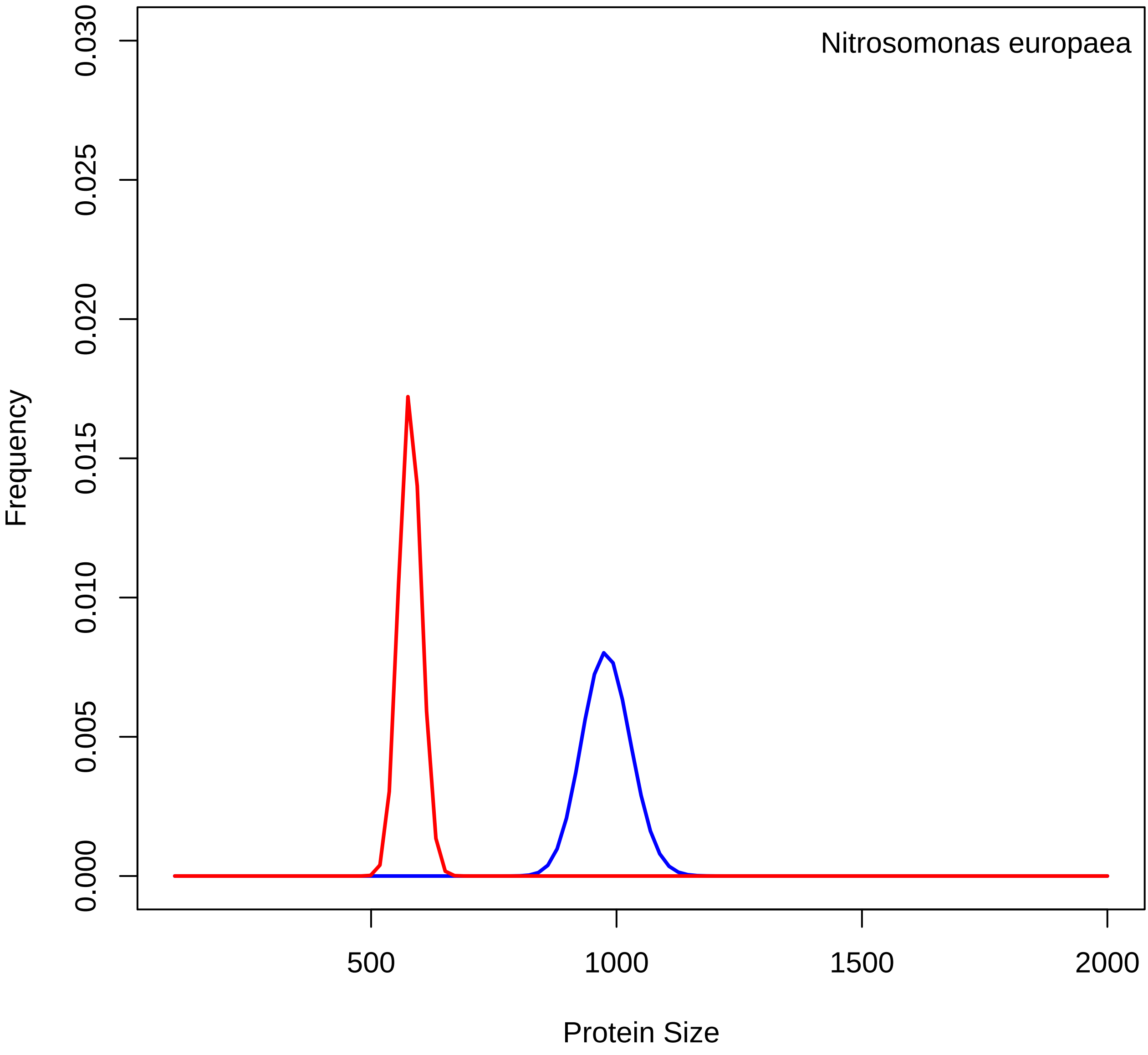

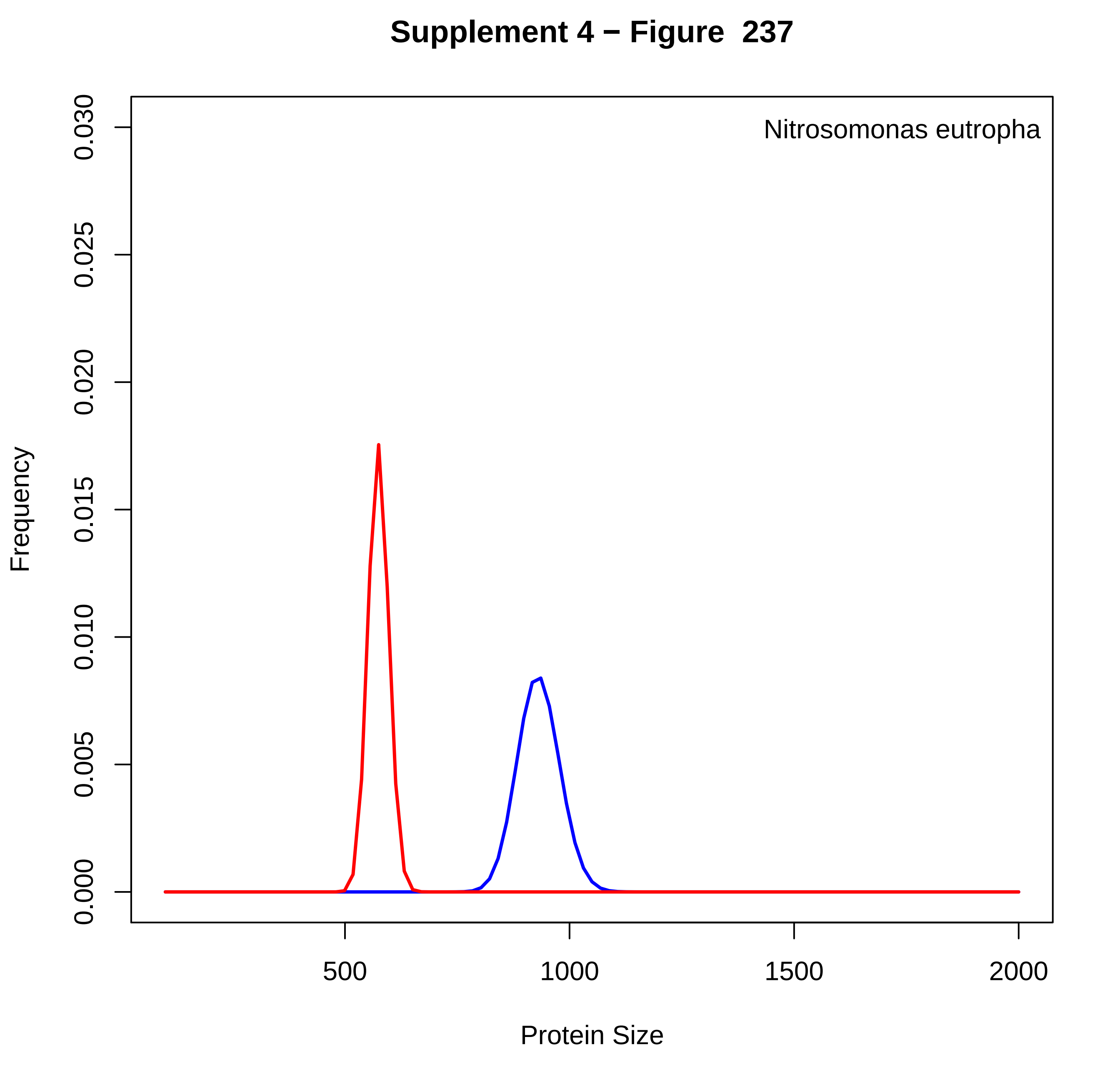
Supplement 4 − Figure 237
Nitrosomonas eutropha
Frequency
0.000
0.005
0.010
0.015
0.020
0.025
0.030
500
1000
1500
2000
Protein Size

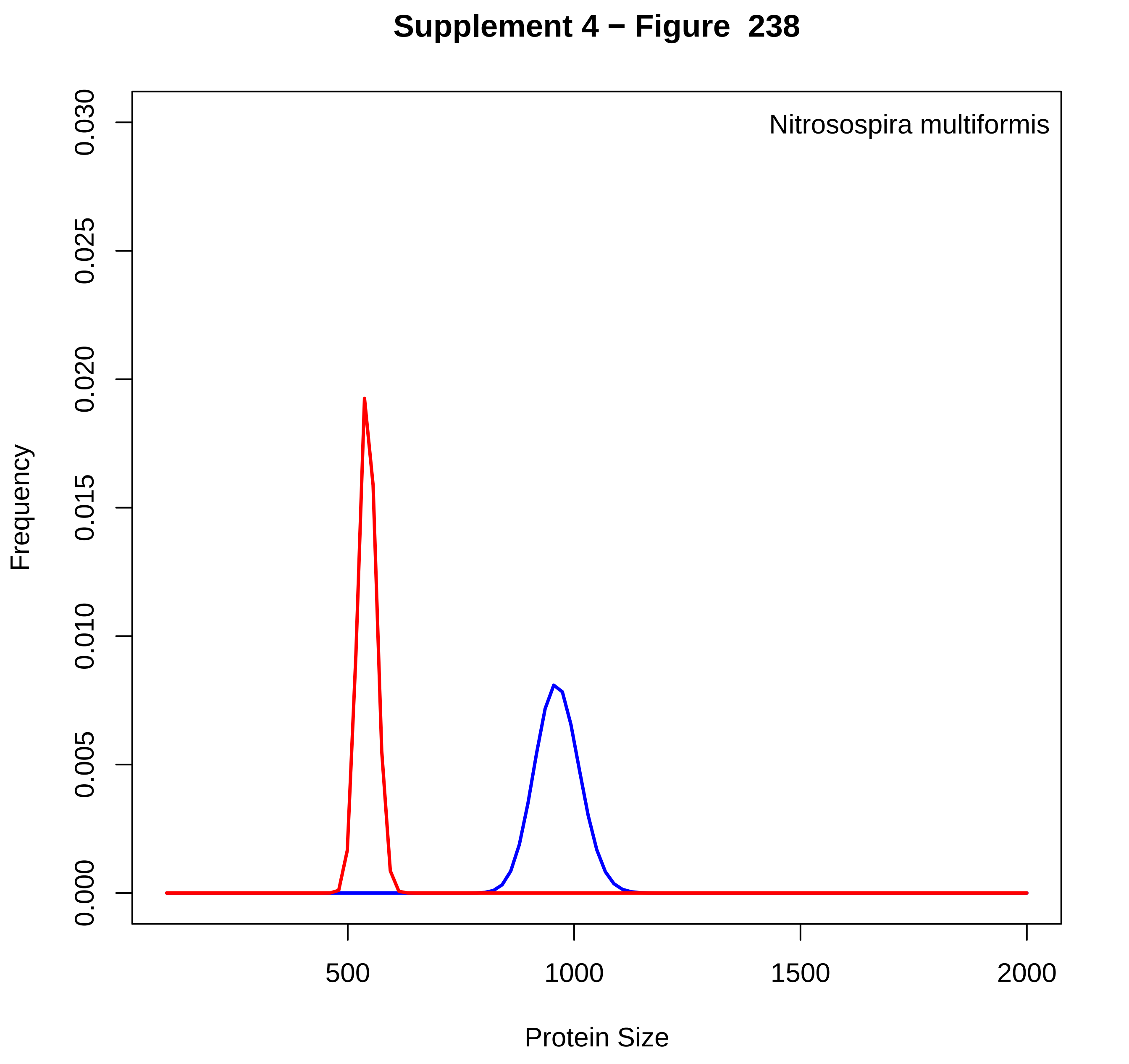
Supplement 4 − Figure 238
Nitrosospira multiformis
Frequency
0.000
0.005
0.010
0.015
0.020
0.025
0.030
500
1000
1500
2000
Protein Size

Supplement 4 − Figure 239

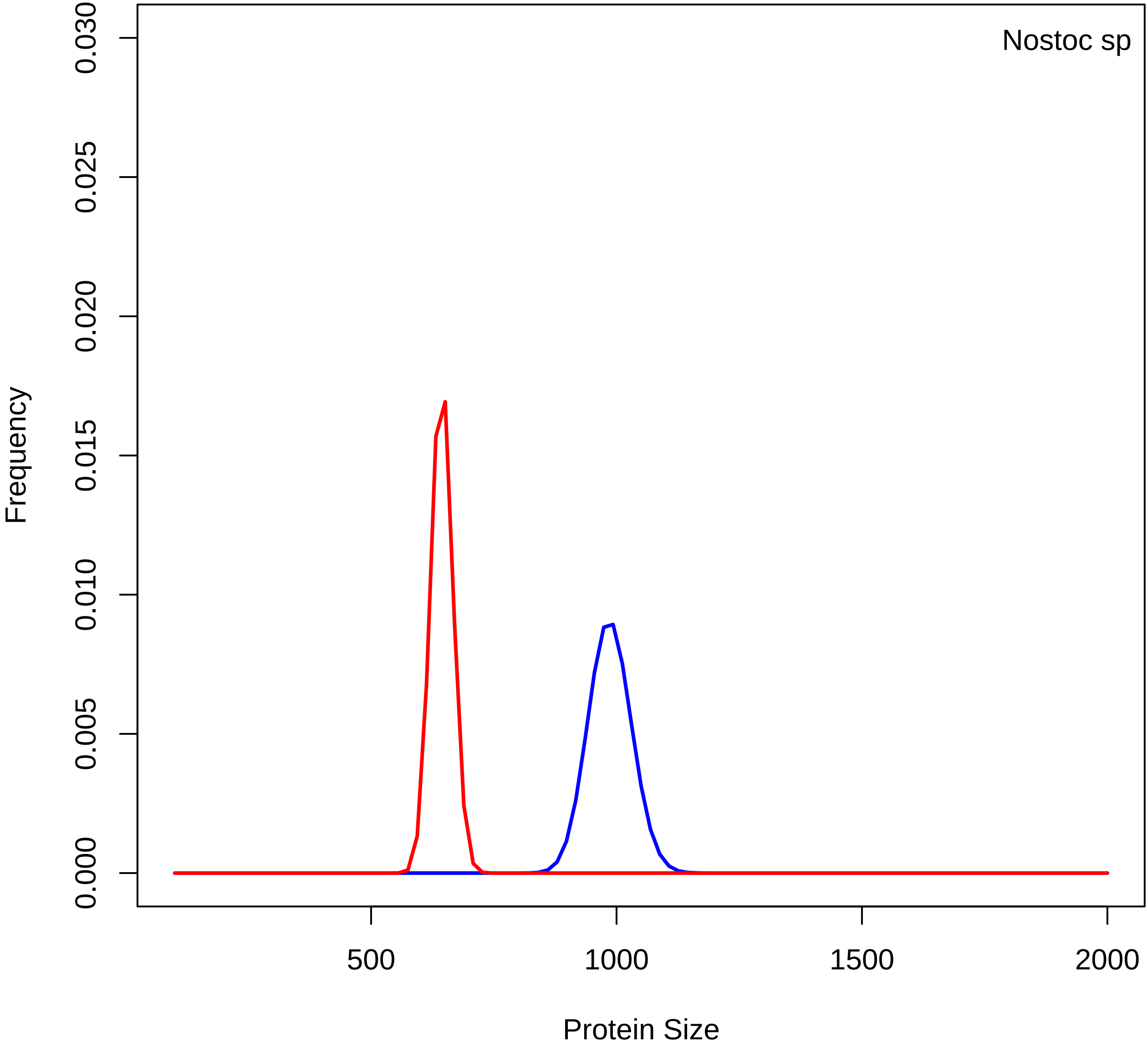

Supplement 4 − Figure 240

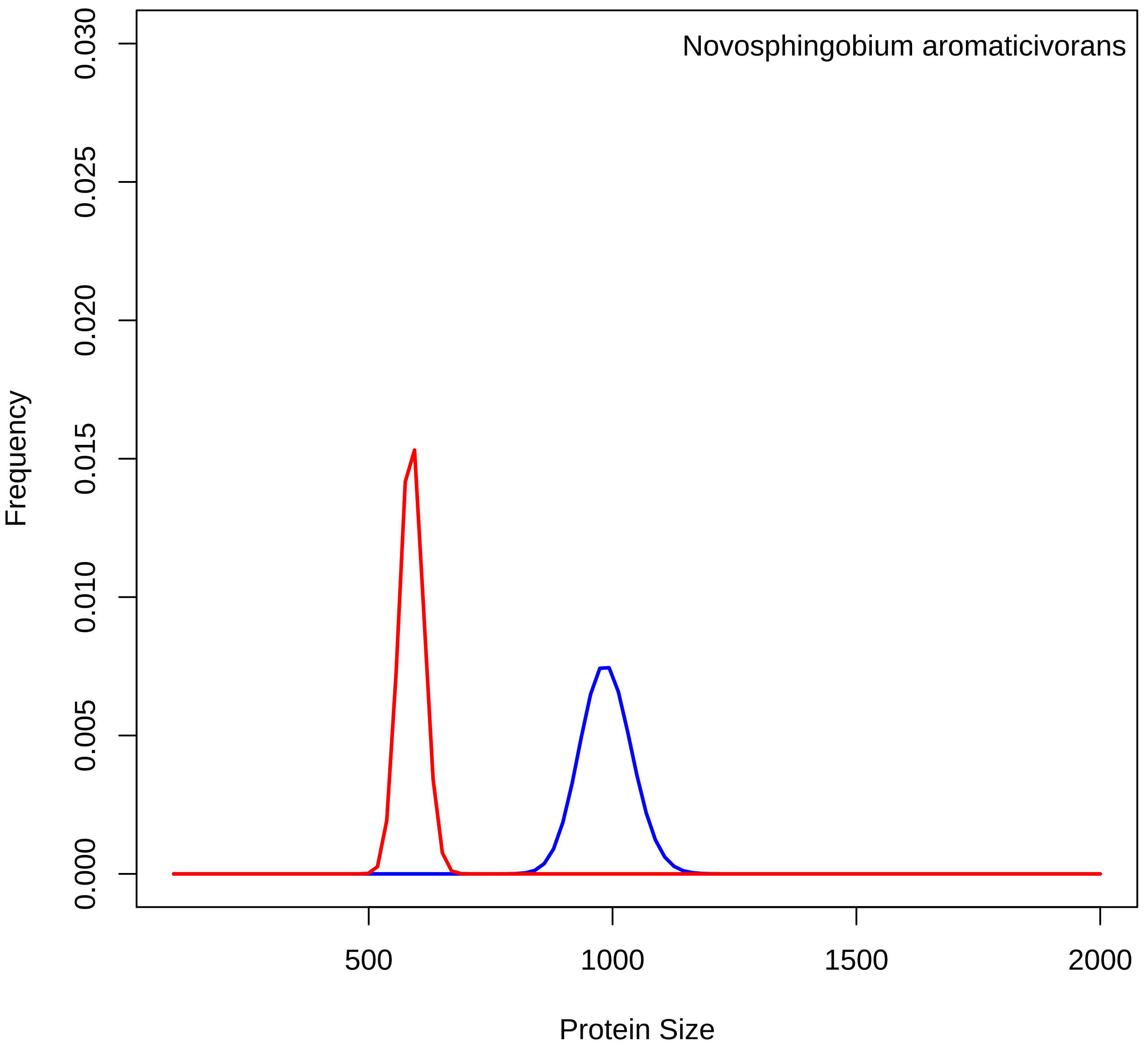

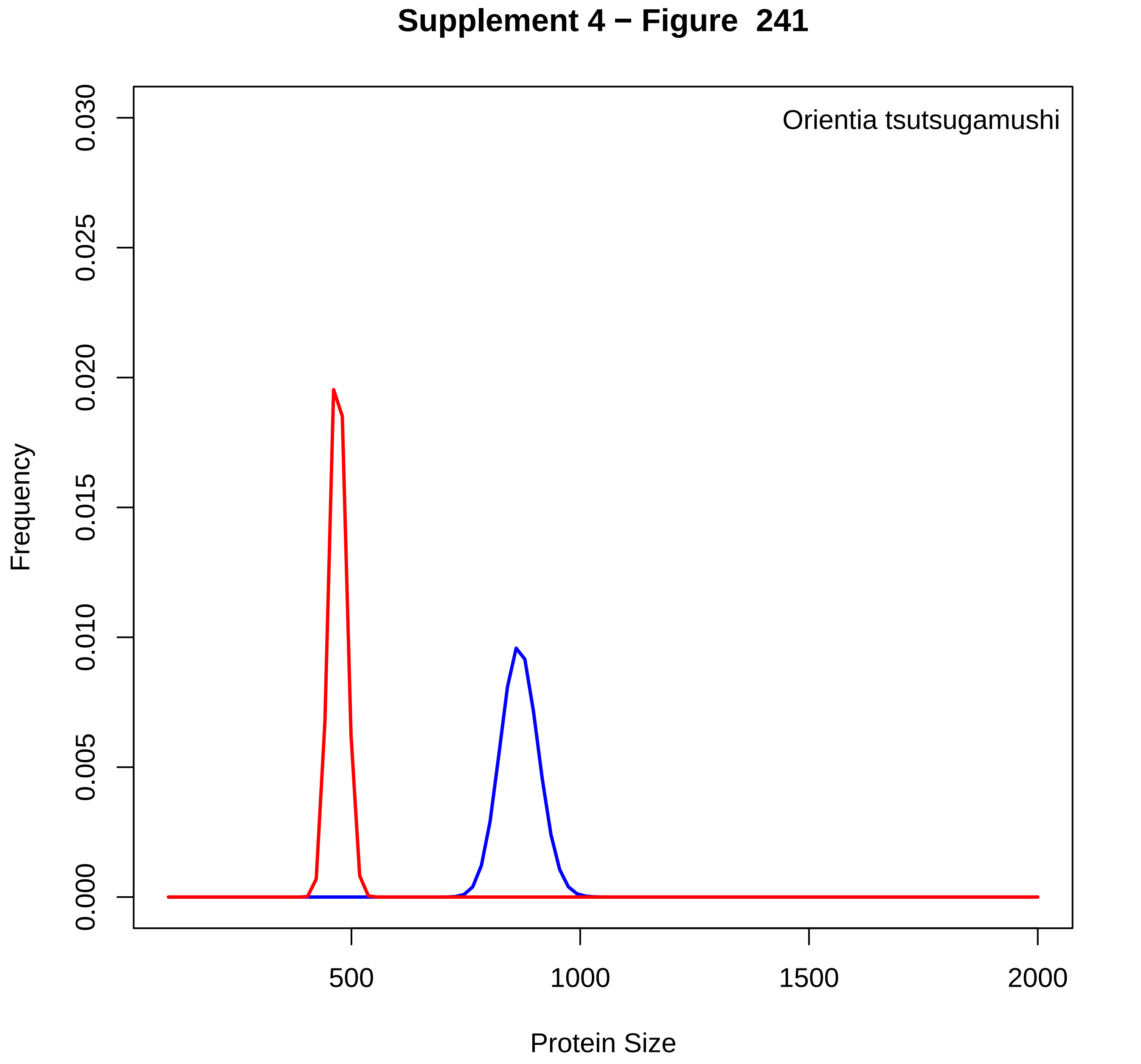
Supplement 4 − Figure 241
Orientia tsutsugamushi
Frequency
0.000
0.005
0.010
0.015
0.020
0.025
0.030
500
1000
1500
2000
Protein Size

Supplement 4 − Figure 242

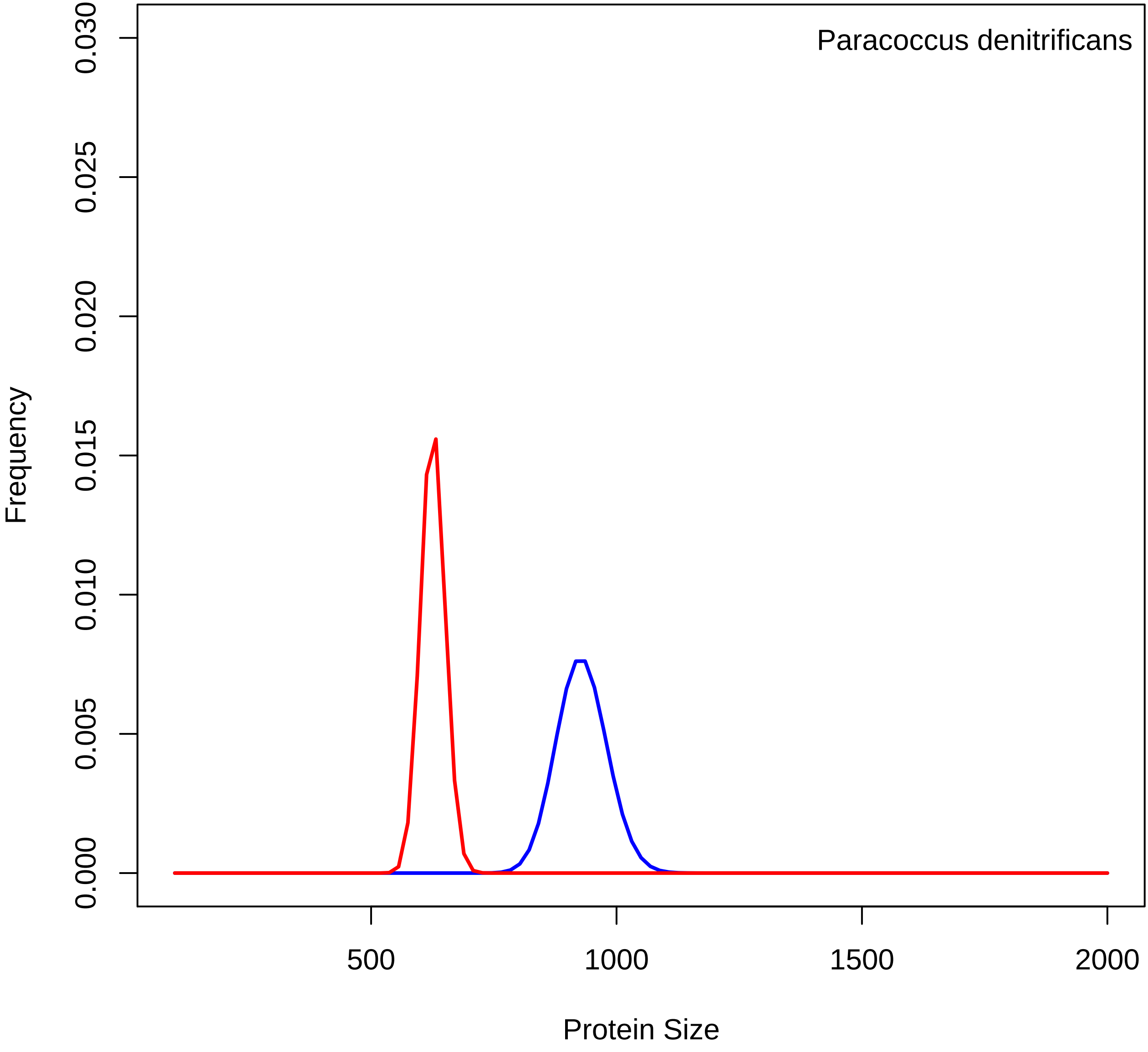

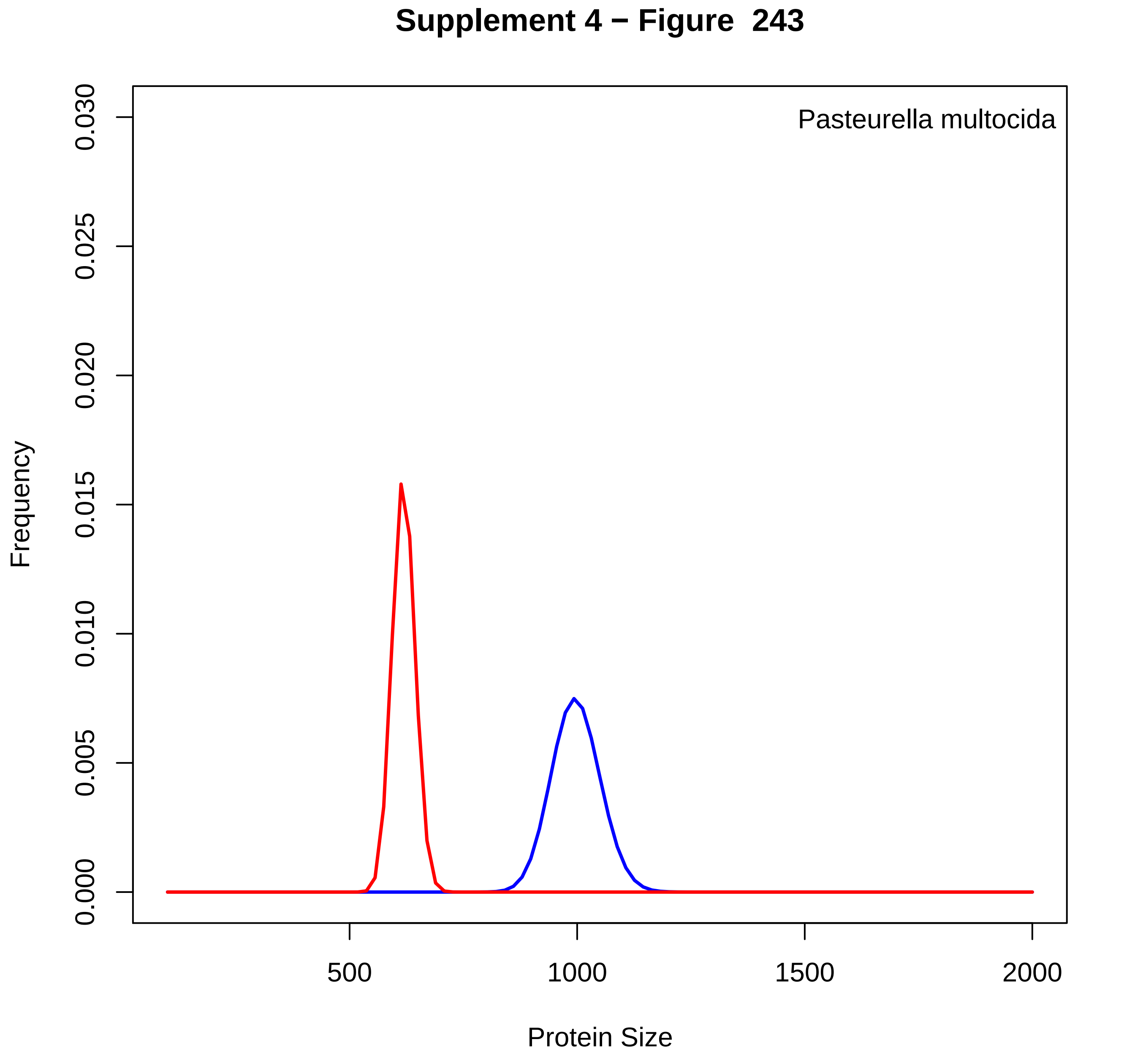
Supplement 4 − Figure 243
Pasteurella multocida
Frequency
0.000
0.005
0.010
0.015
0.020
0.025
0.030
500
1000
1500
2000
Protein Size

Supplement 4 − Figure 244

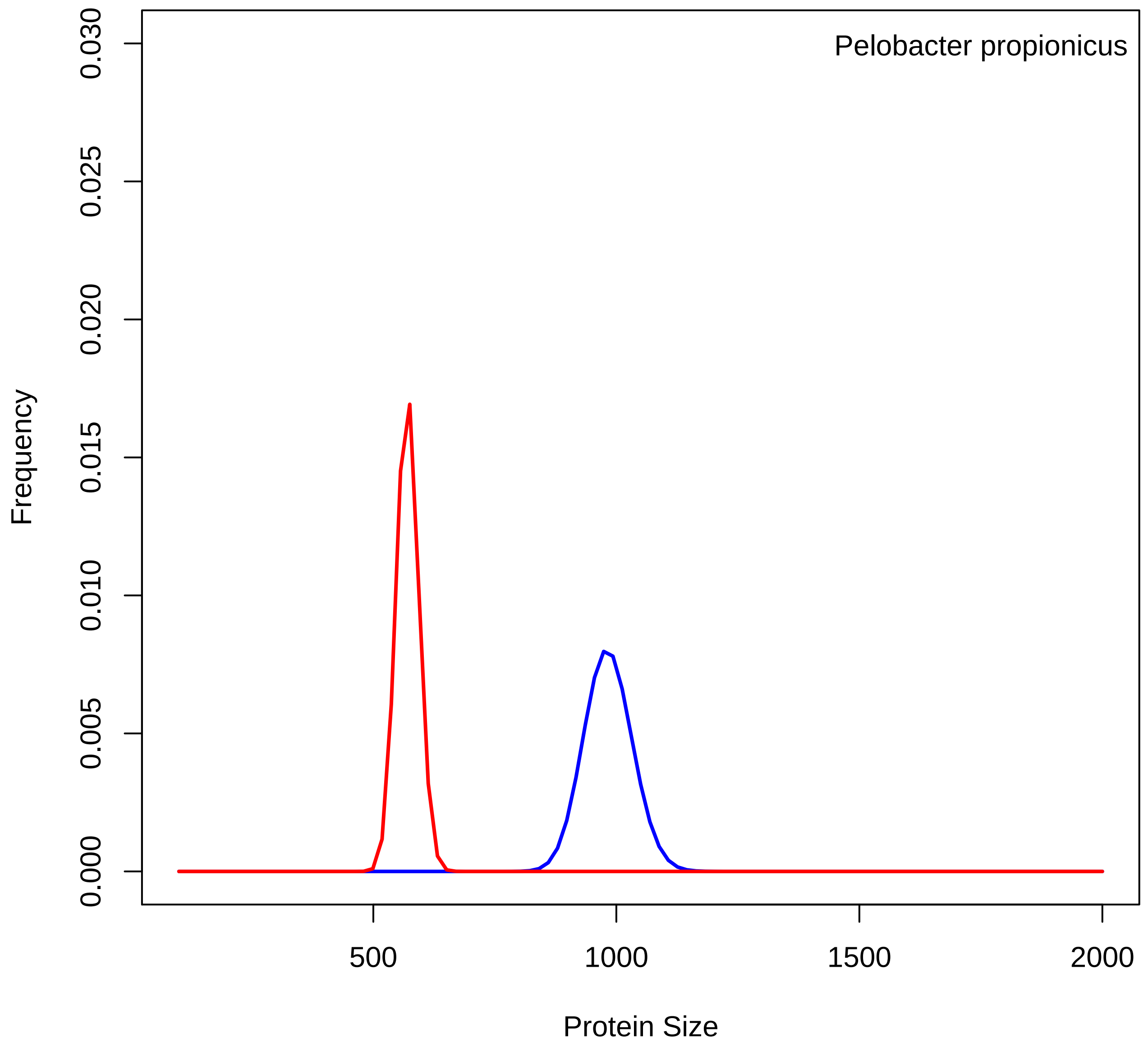

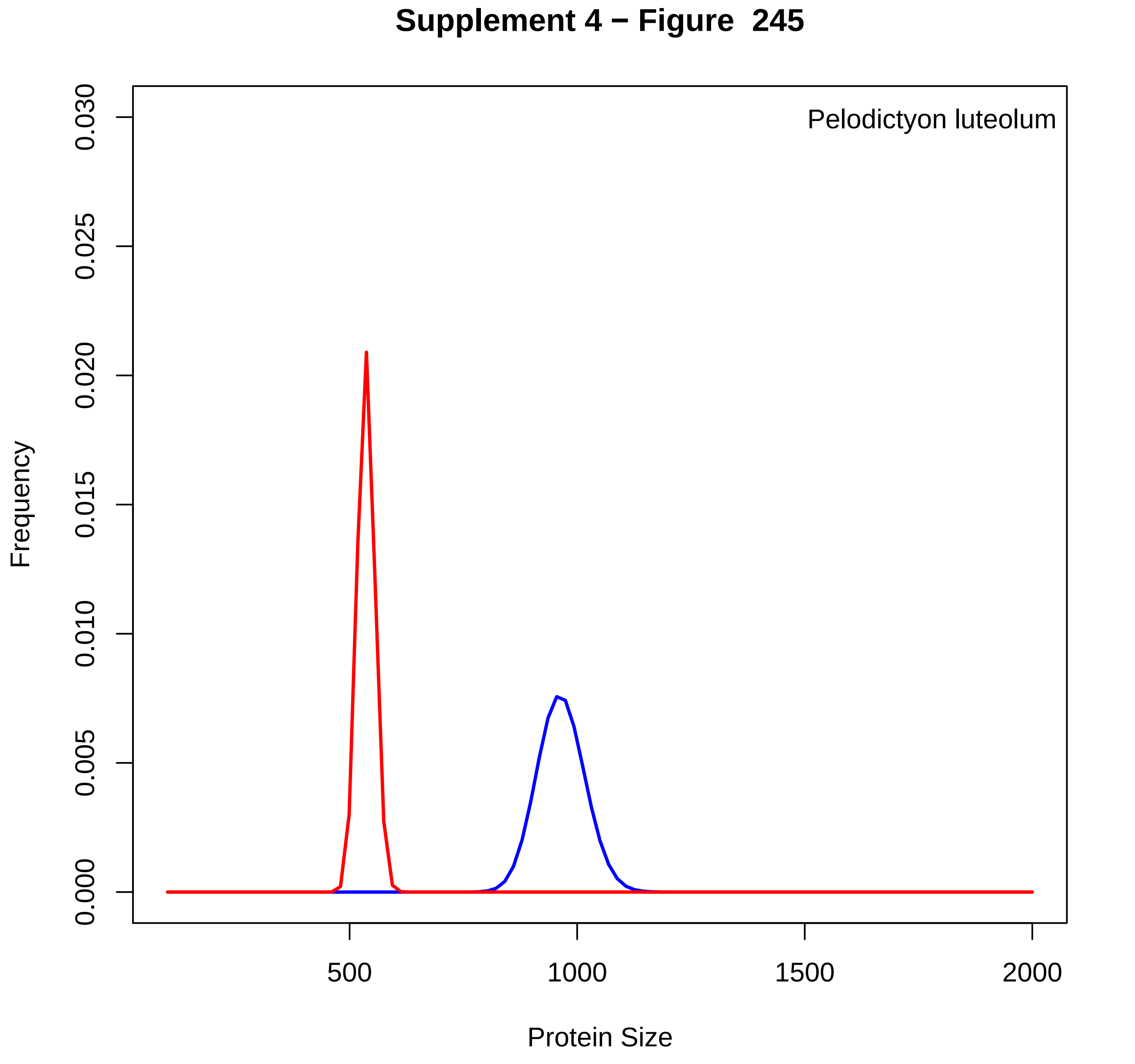
Supplement 4 − Figure 245
Pelodictyon luteolum
Frequency
0.000
0.005
0.010
0.015
0.020
0.025
0.030
500
1000
1500
2000
Protein Size

Supplement 4 − Figure 246

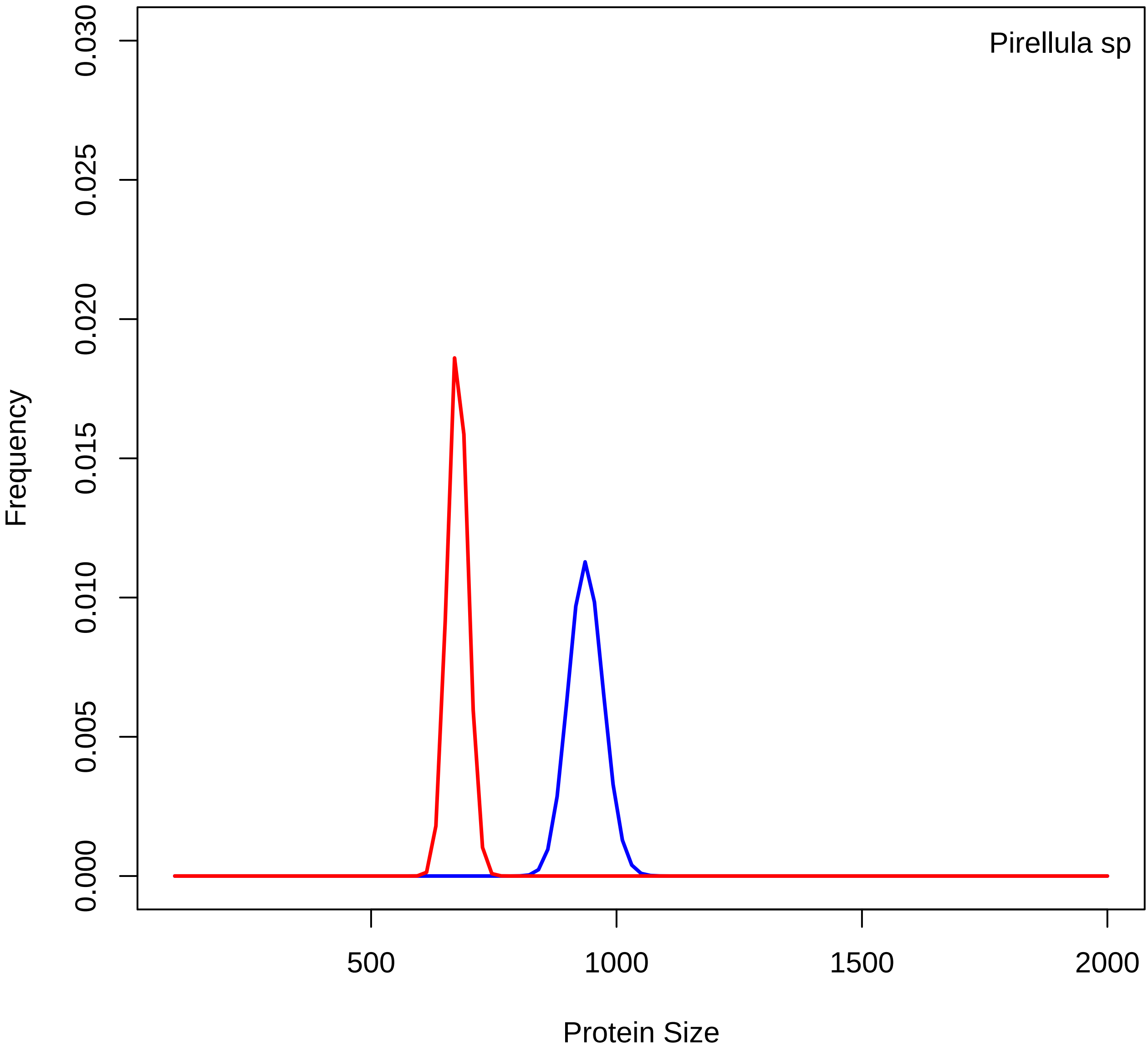

Supplement 4 − Figure 247

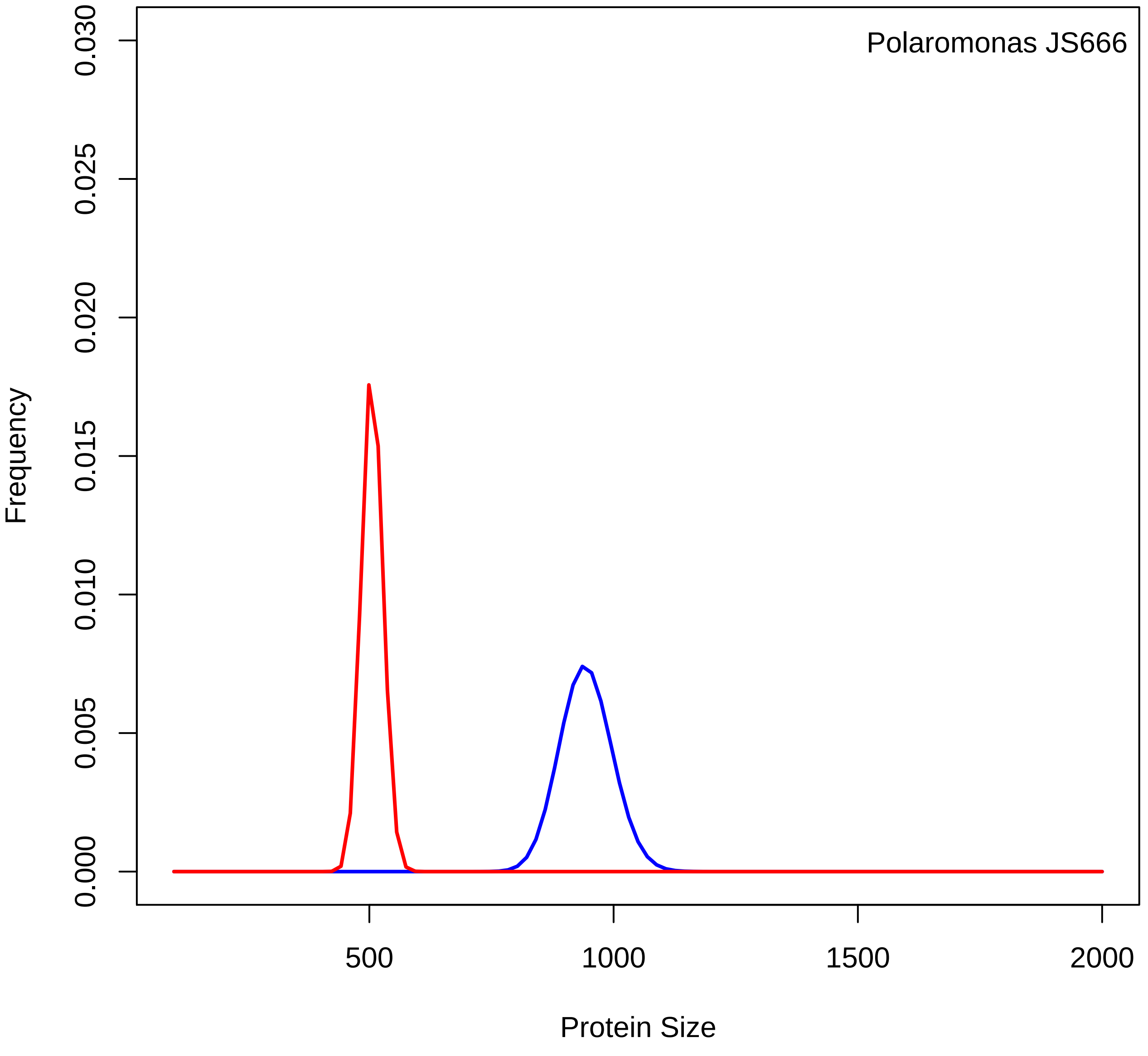

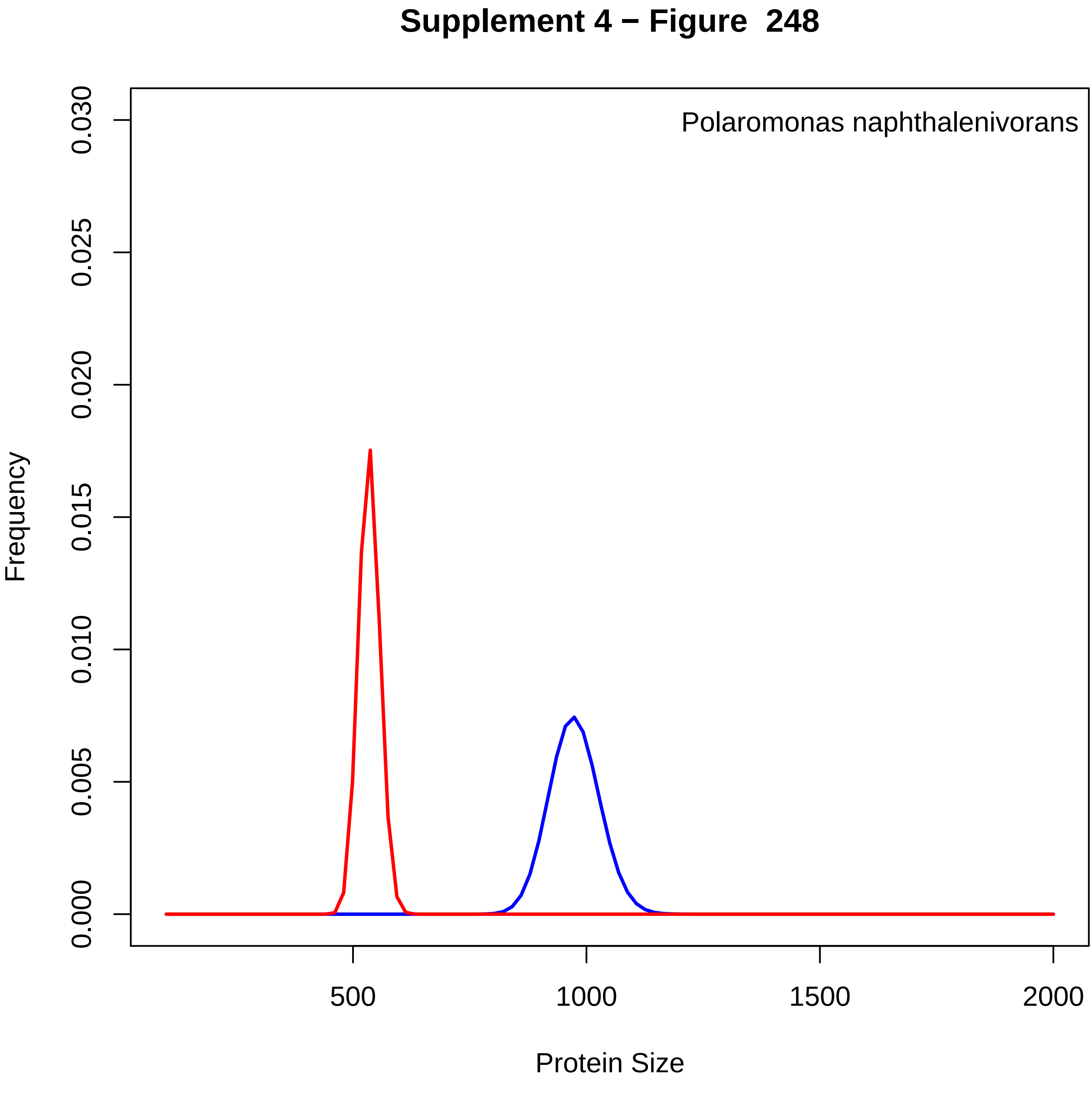
Supplement 4 − Figure 248
Polaromonas naphthalenivorans
Frequency
0.000
0.005
0.010
0.015
0.020
0.025
0.030
500
1000
1500
2000
Protein Size

Supplement 4 − Figure 249

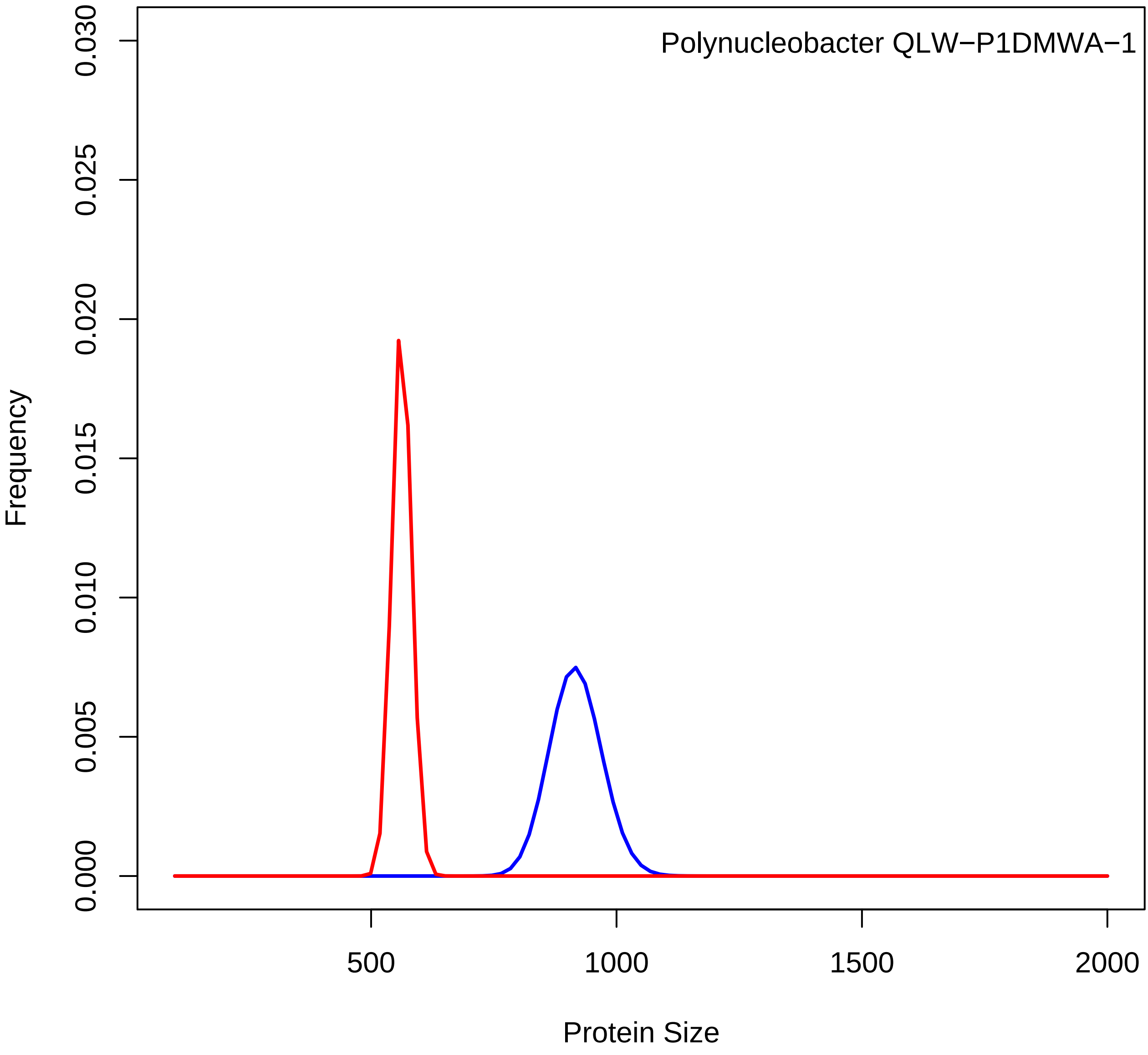

Supplement 4 − Figure 250

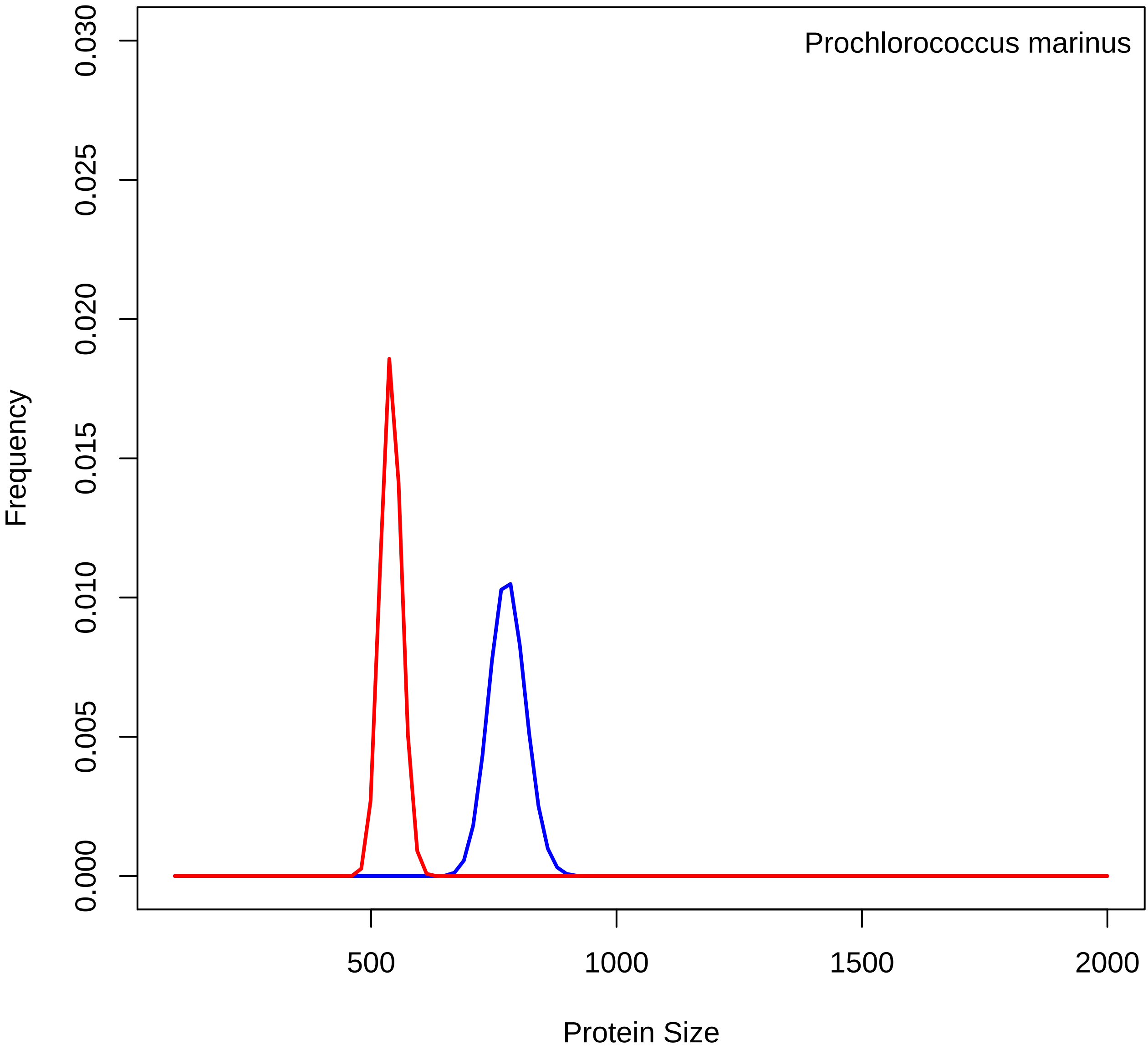

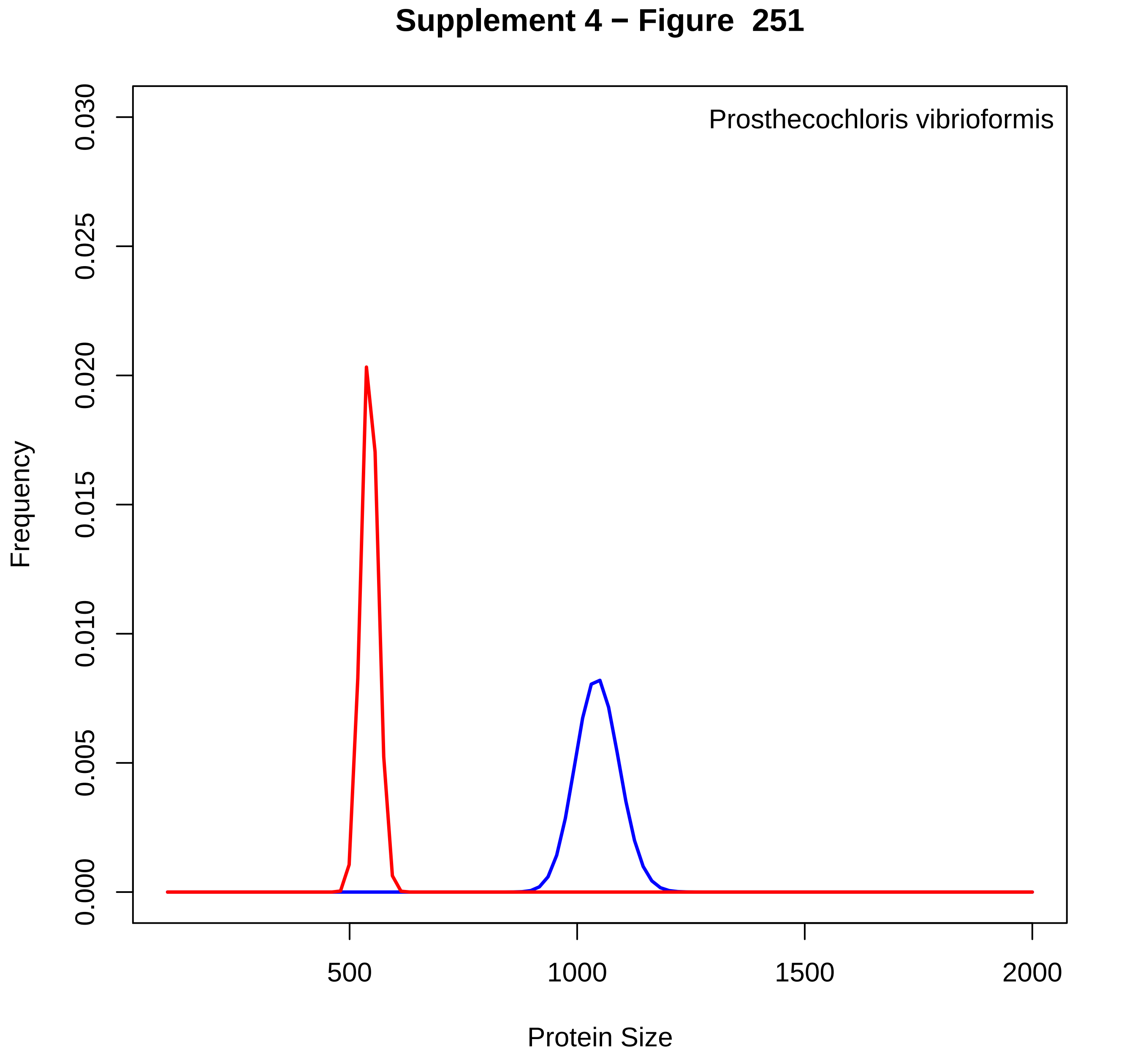
Supplement 4 − Figure 251
Prosthecochloris vibrioformis
Frequency
0.000
0.005
0.010
0.015
0.020
0.025
0.030
500
1000
1500
2000
Protein Size

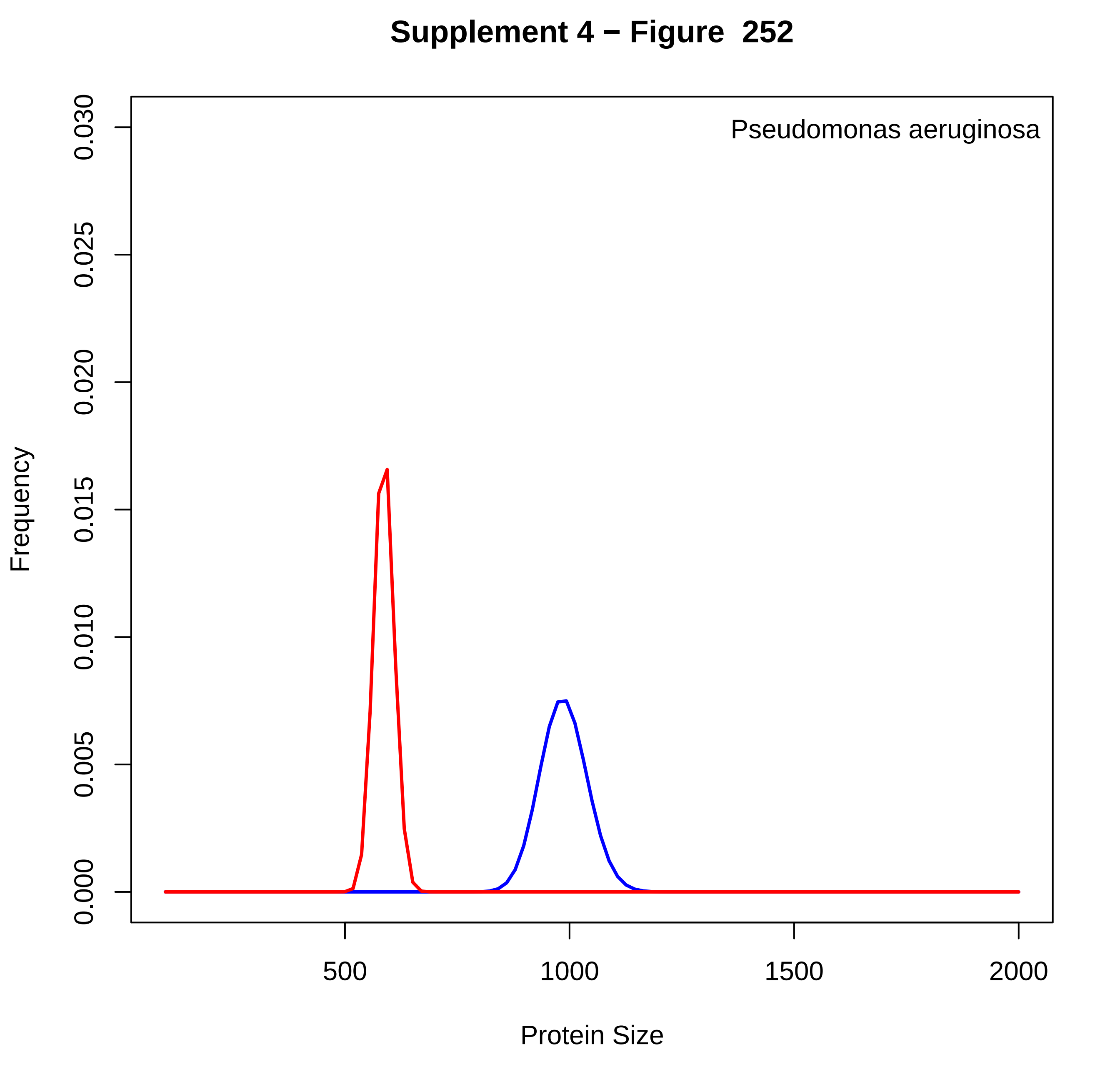
Supplement 4 − Figure 252
Pseudomonas aeruginosa
Frequency
0.000
0.005
0.010
0.015
0.020
0.025
0.030
500
1000
1500
2000
Protein Size

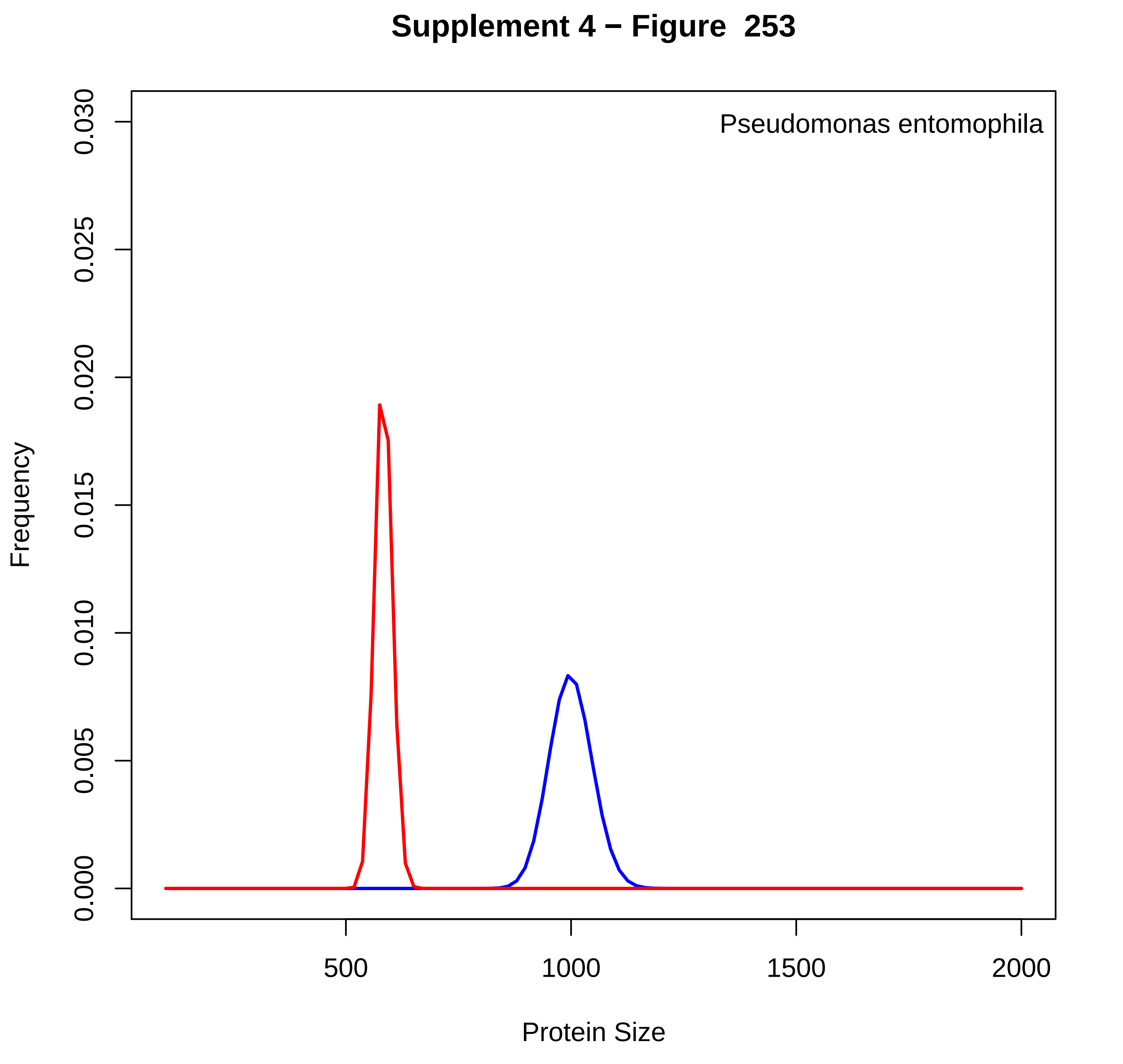
Supplement 4 − Figure 253
Pseudomonas entomophila
Frequency
0.000
0.005
0.010
0.015
0.020
0.025
0.030
500
1000
1500
2000
Protein Size

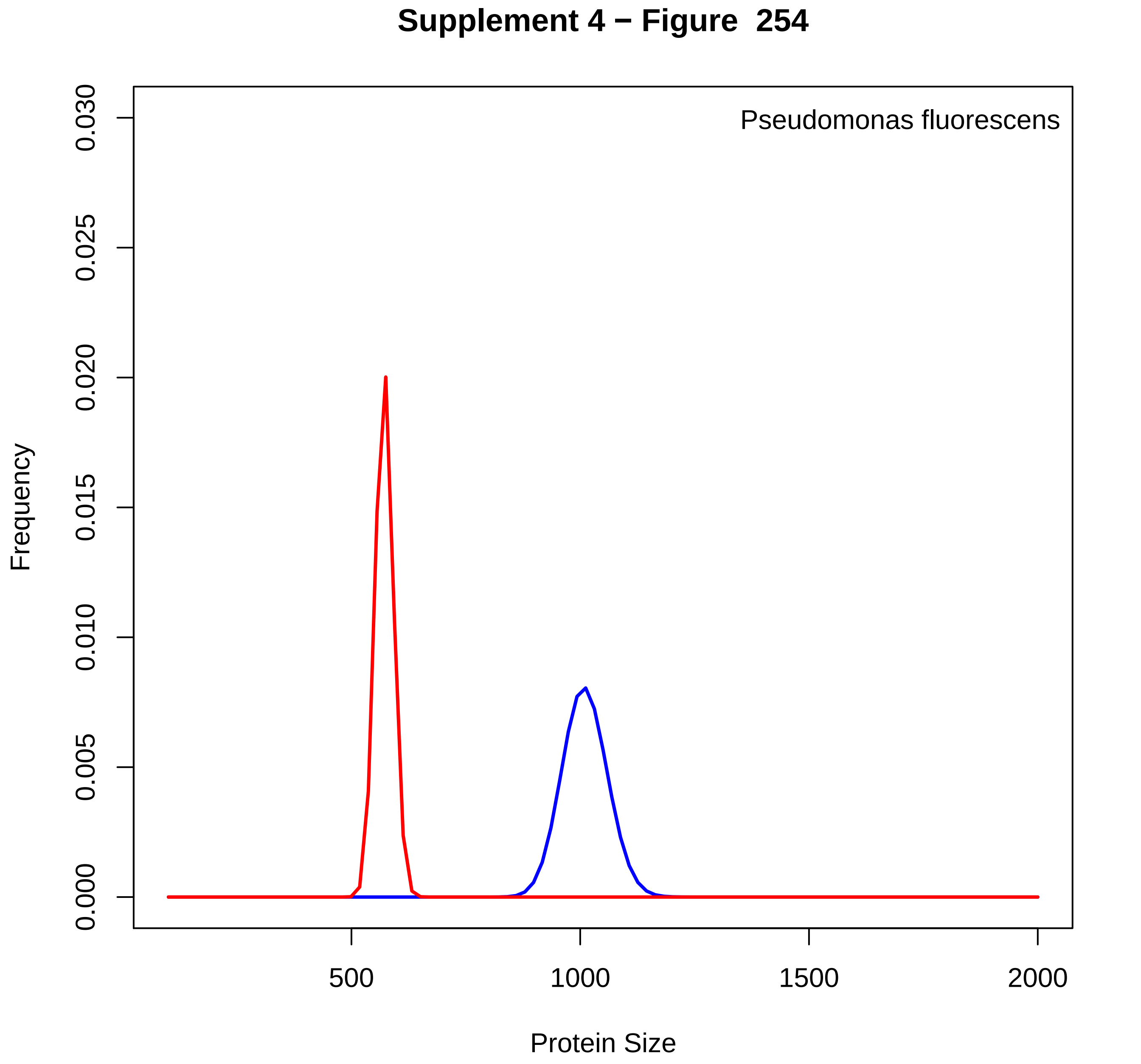
Supplement 4 − Figure 254
Pseudomonas fluorescens
Frequency
0.000
0.005
0.010
0.015
0.020
0.025
0.030
500
1000
1500
2000
Protein Size

Supplement 4 − Figure 255

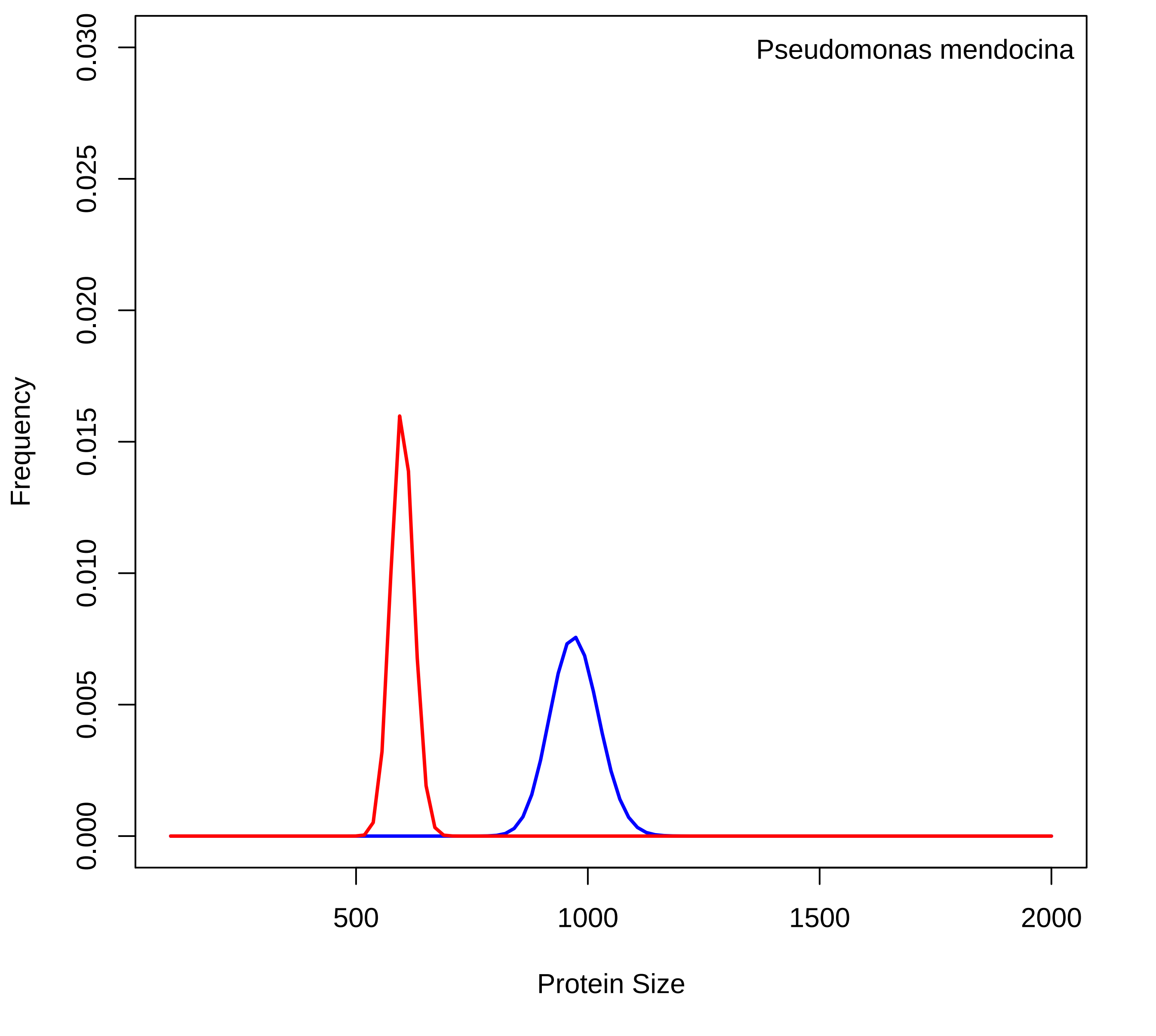

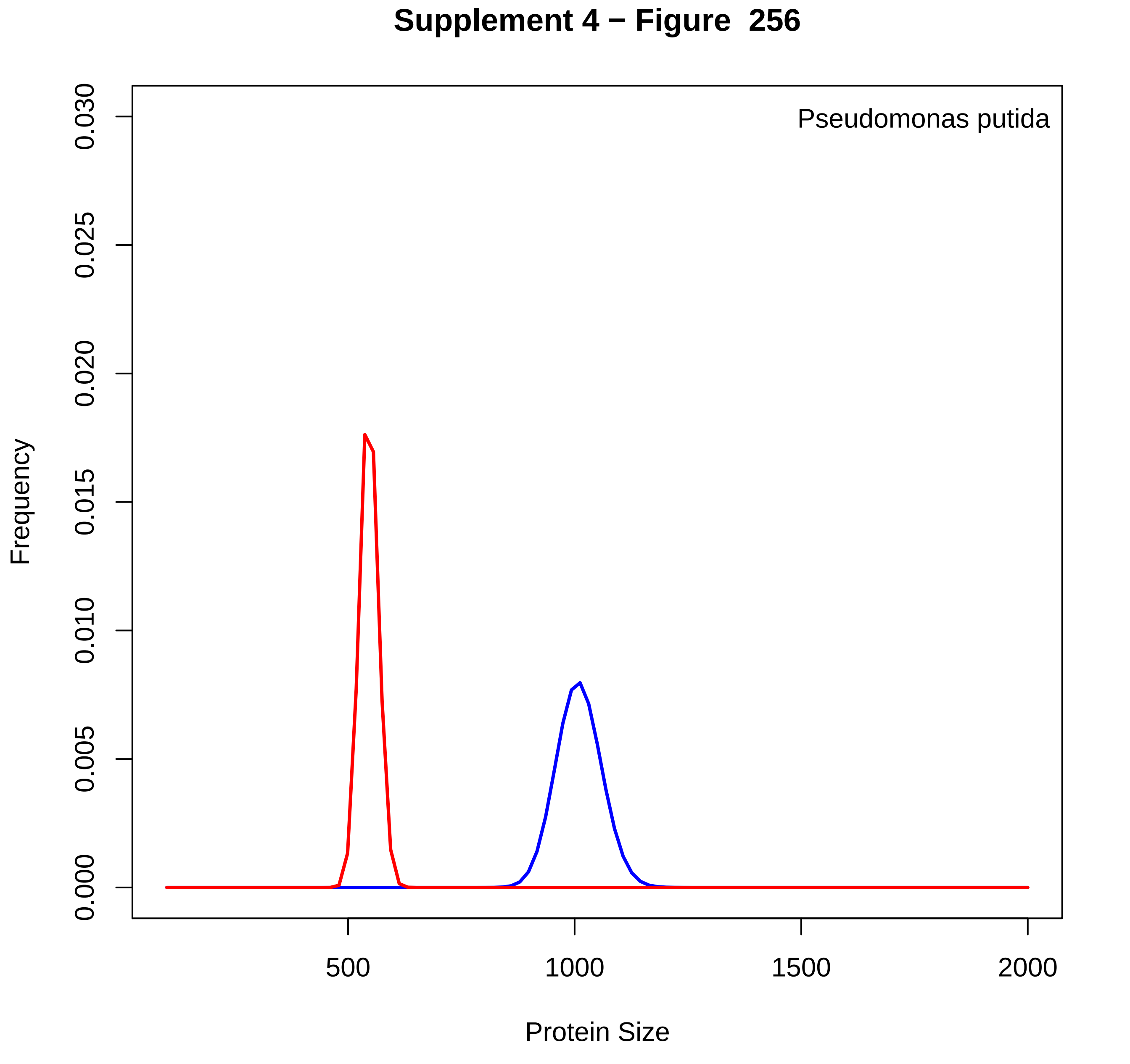
Supplement 4 − Figure 256
Pseudomonas putida
Frequency
0.000
0.005
0.010
0.015
0.020
0.025
0.030
500
1000
1500
2000
Protein Size

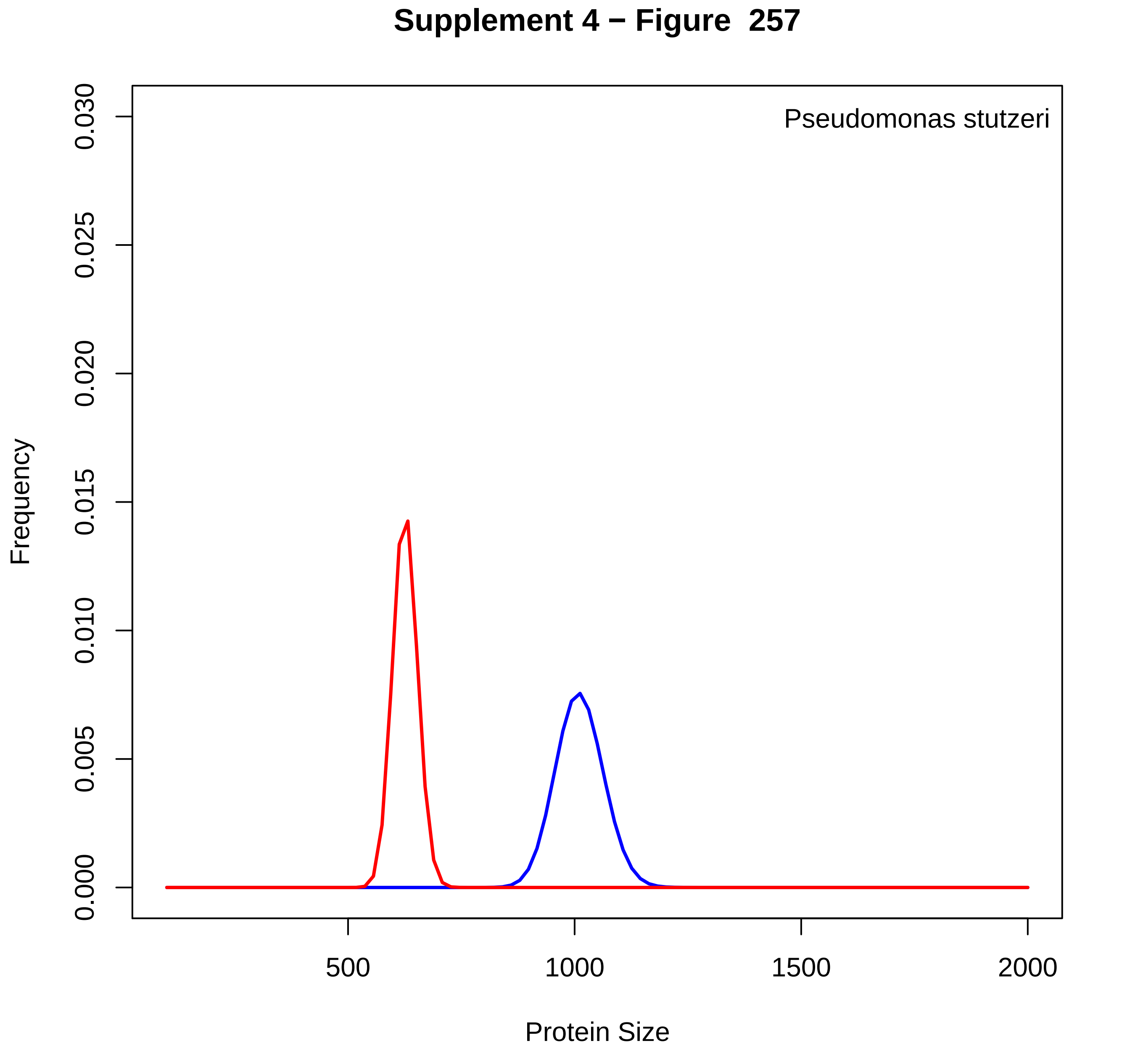
Supplement 4 − Figure 257
Pseudomonas stutzeri
Frequency
0.000
0.005
0.010
0.015
0.020
0.025
0.030
500
1000
1500
2000
Protein Size

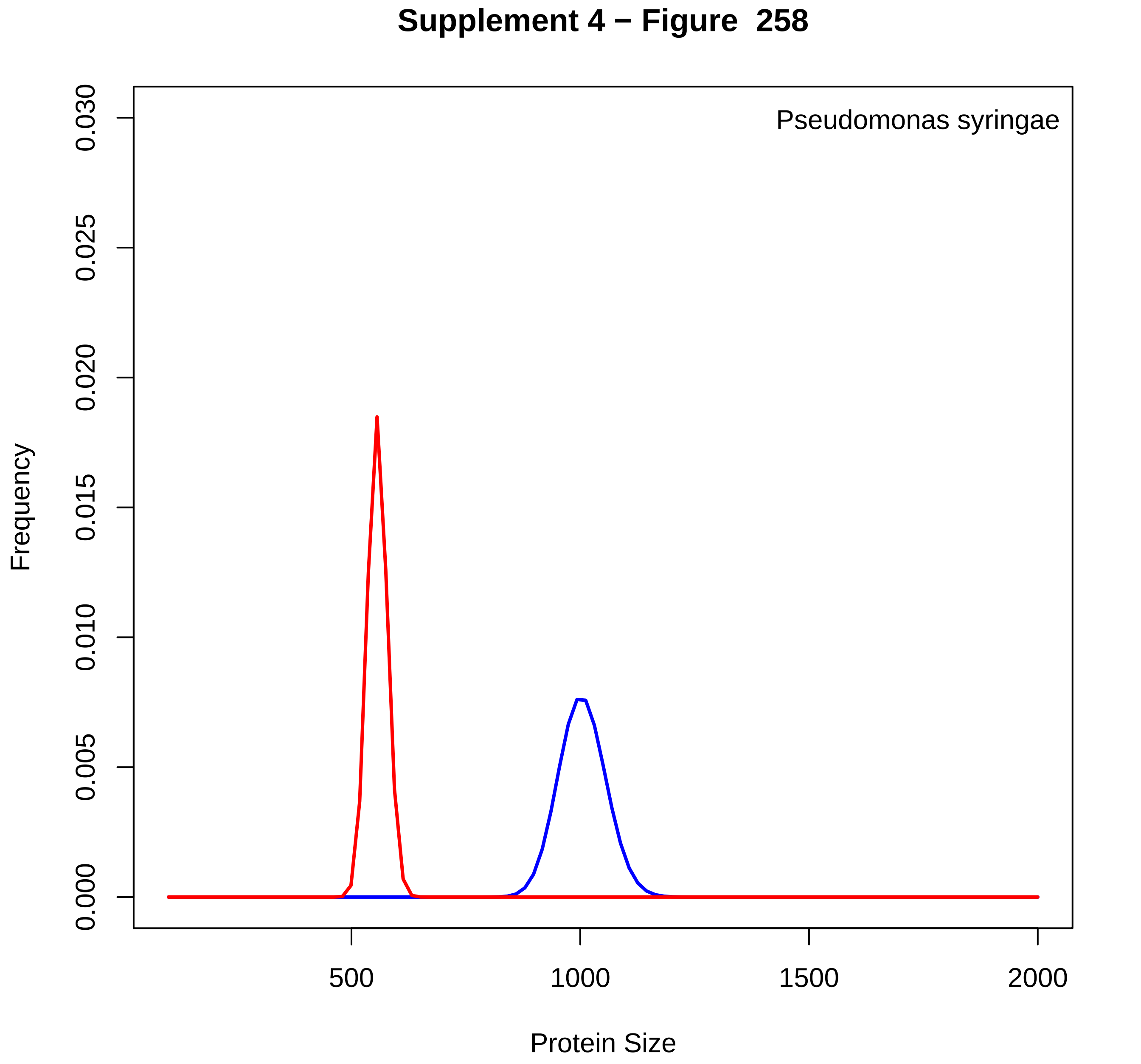
Supplement 4 − Figure 258
Pseudomonas syringae
Frequency
0.000
0.005
0.010
0.015
0.020
0.025
0.030
500
1000
1500
2000
Protein Size

Supplement 4 − Figure 259

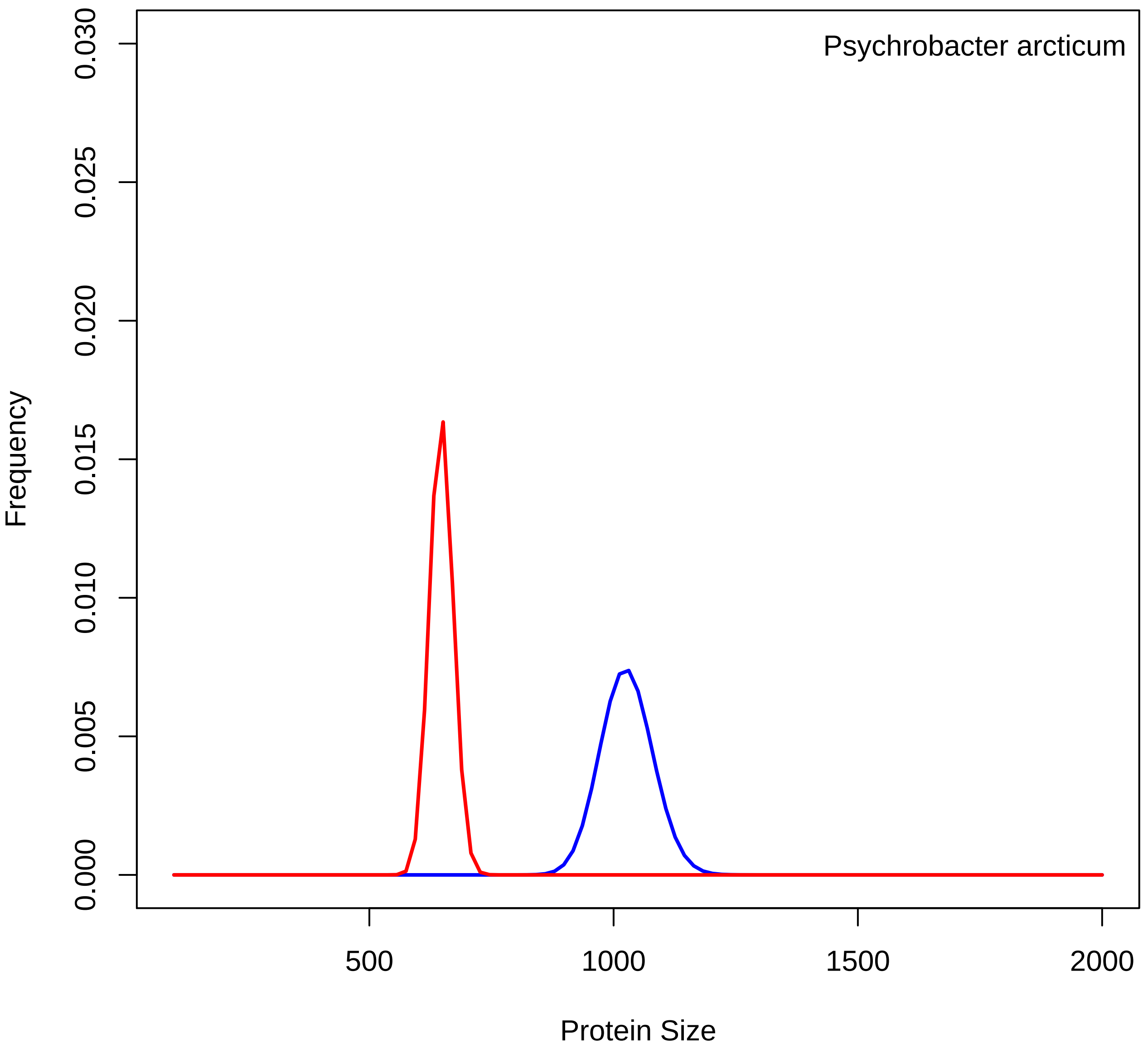

Supplement 4 − Figure 260

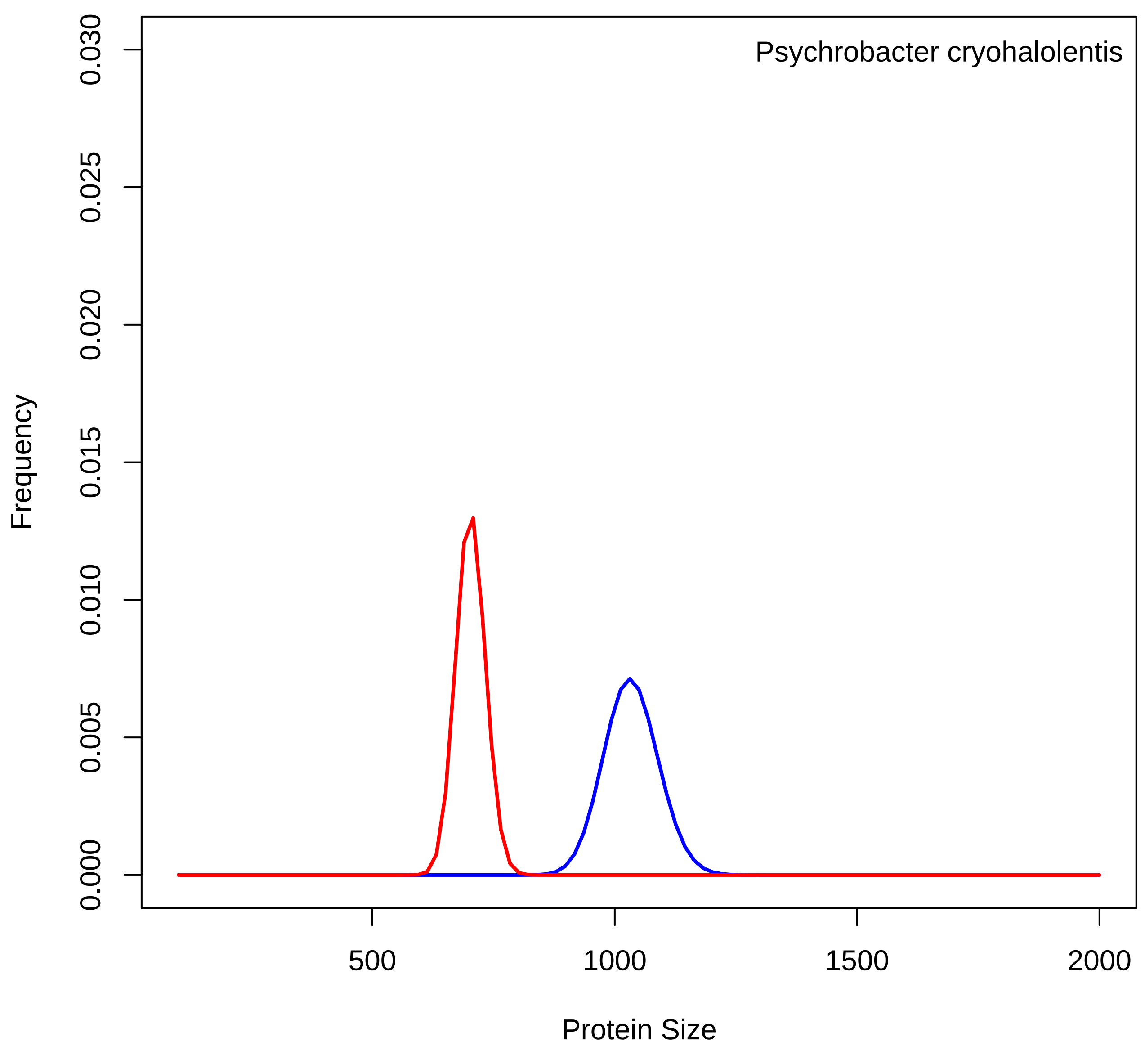

Supplement 4 − Figure 261

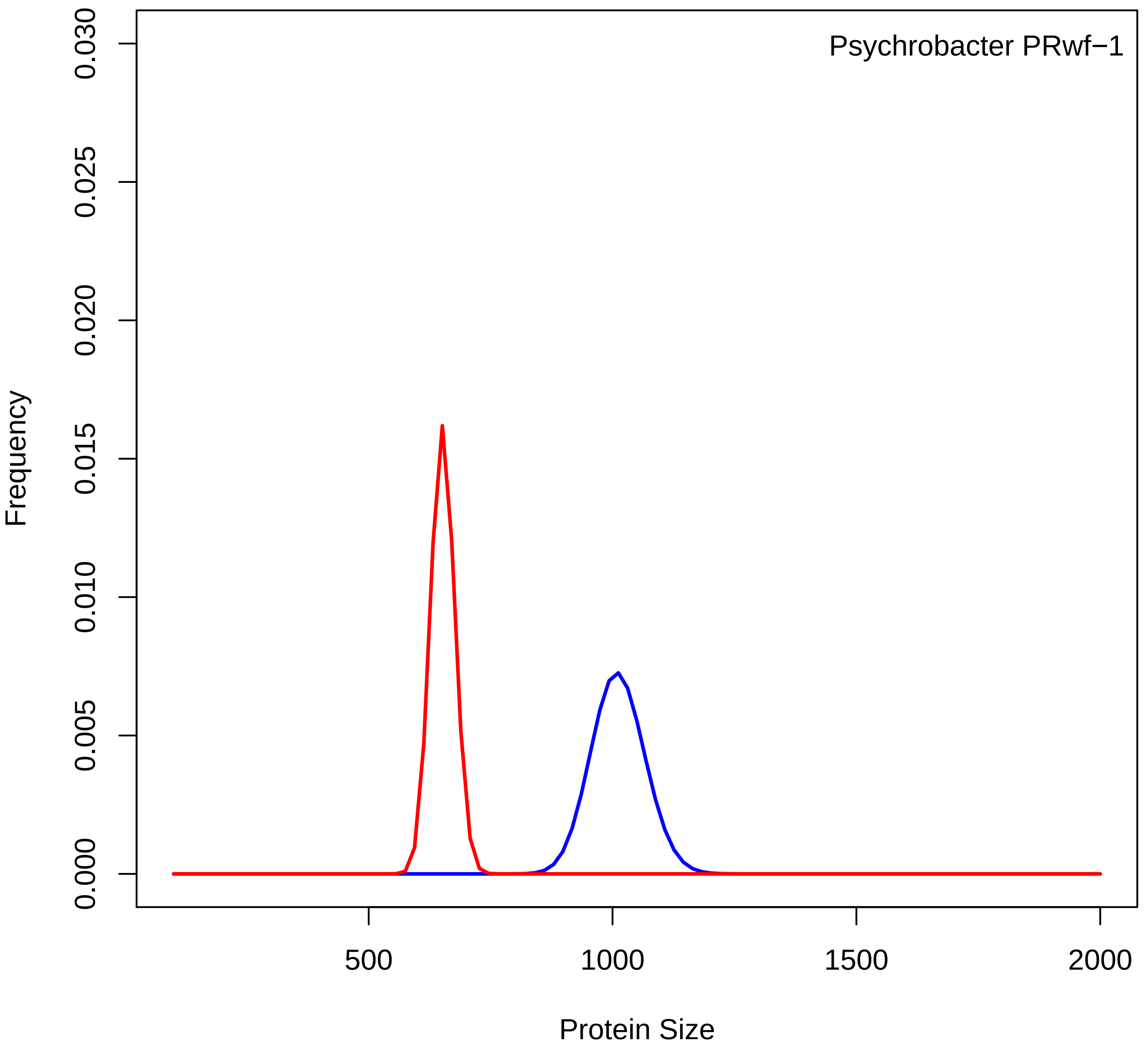

Supplement 4 − Figure 262

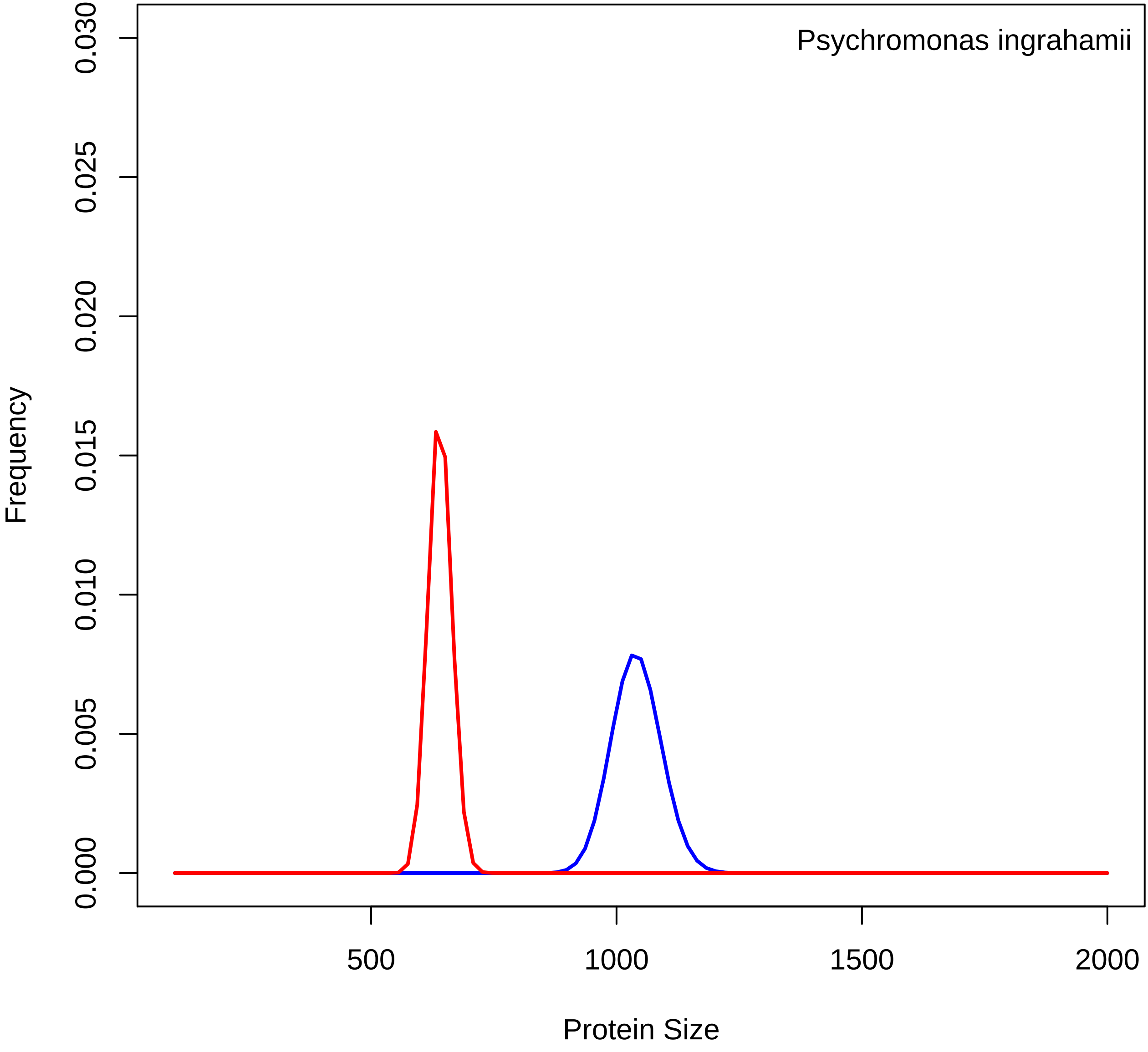

Supplement 4 − Figure 263

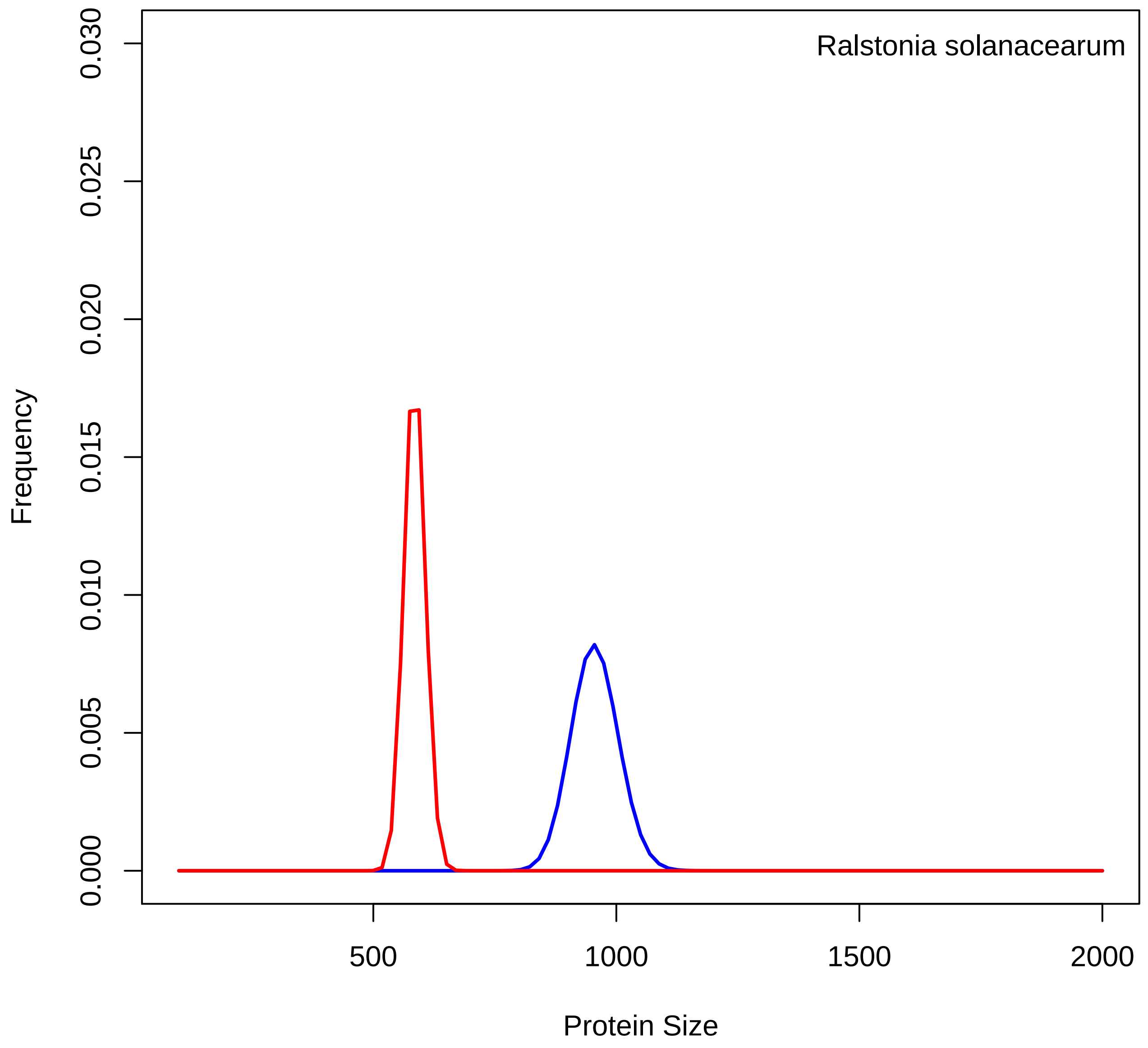

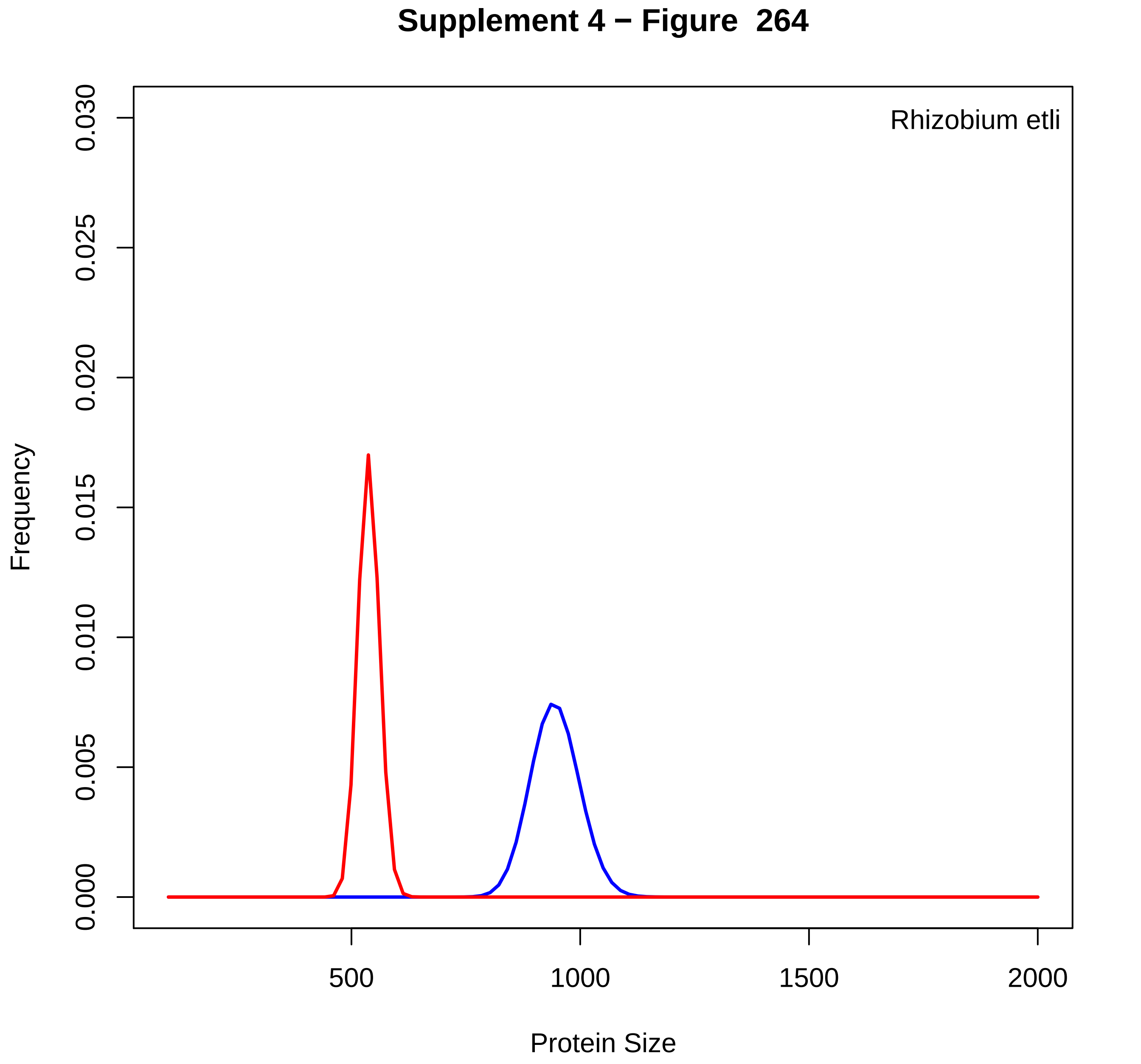
Supplement 4 − Figure 264
Rhizobium etli
Frequency
0.000
0.005
0.010
0.015
0.020
0.025
0.030
500
1000
1500
2000
Protein Size

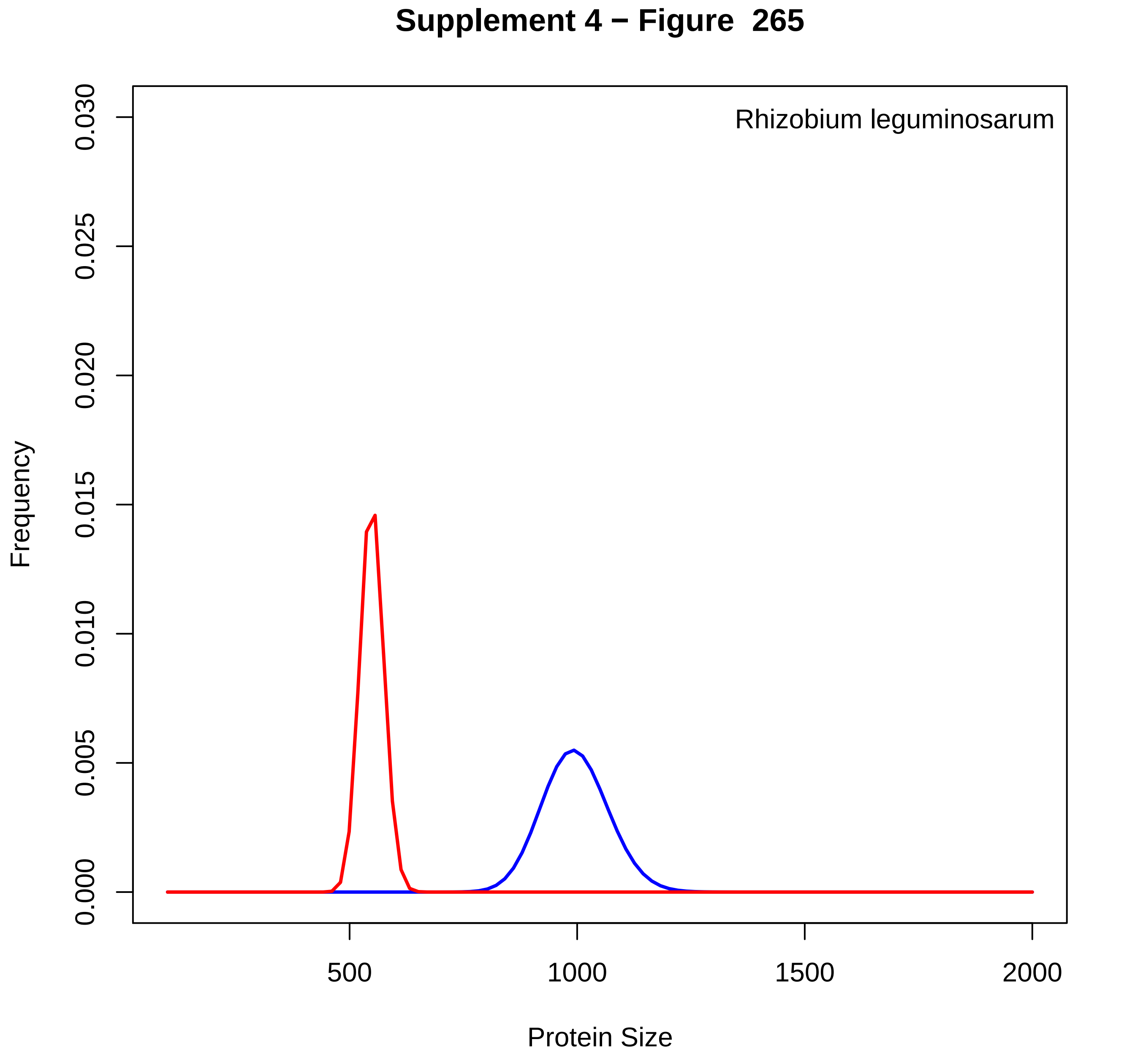

Supplement 4 − Figure 265
Rhizobium leguminosarum
Frequency
0.000
0.005
0.010
0.015
0.020
0.025
0.030
500
1000
1500
2000
Protein Size

Supplement 4 − Figure 266

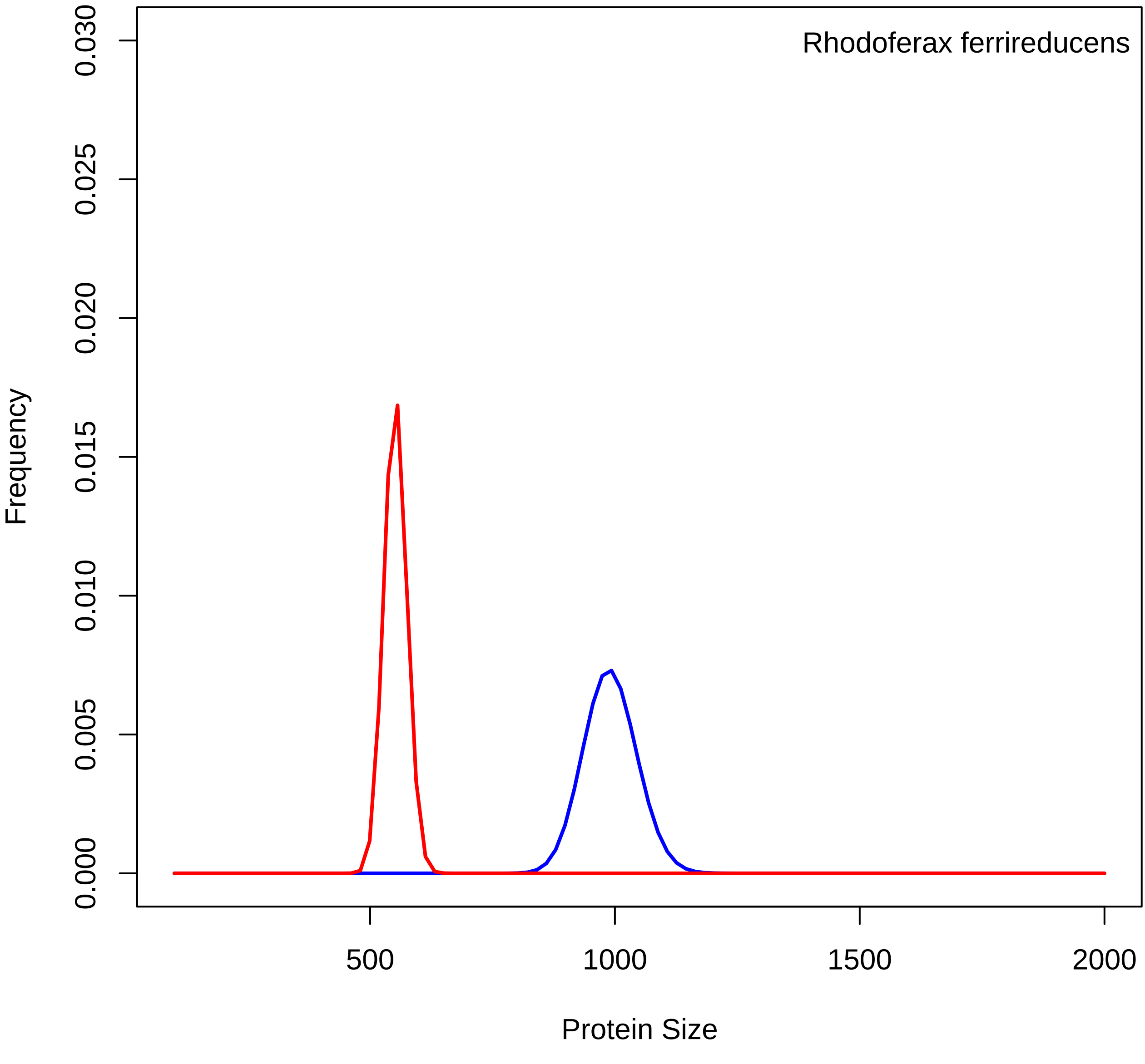

Supplement 4 − Figure 267

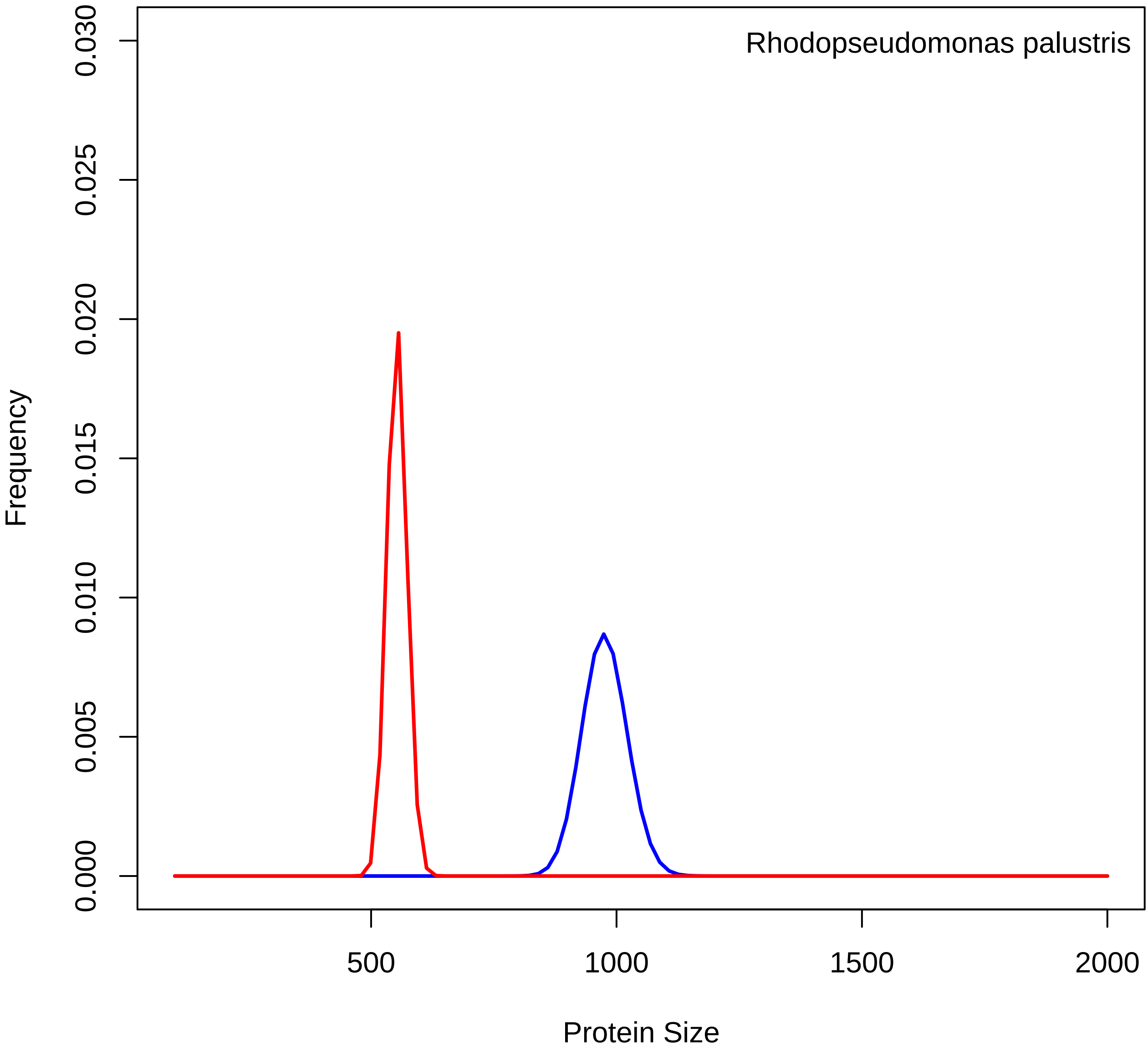

Supplement 4 − Figure 268

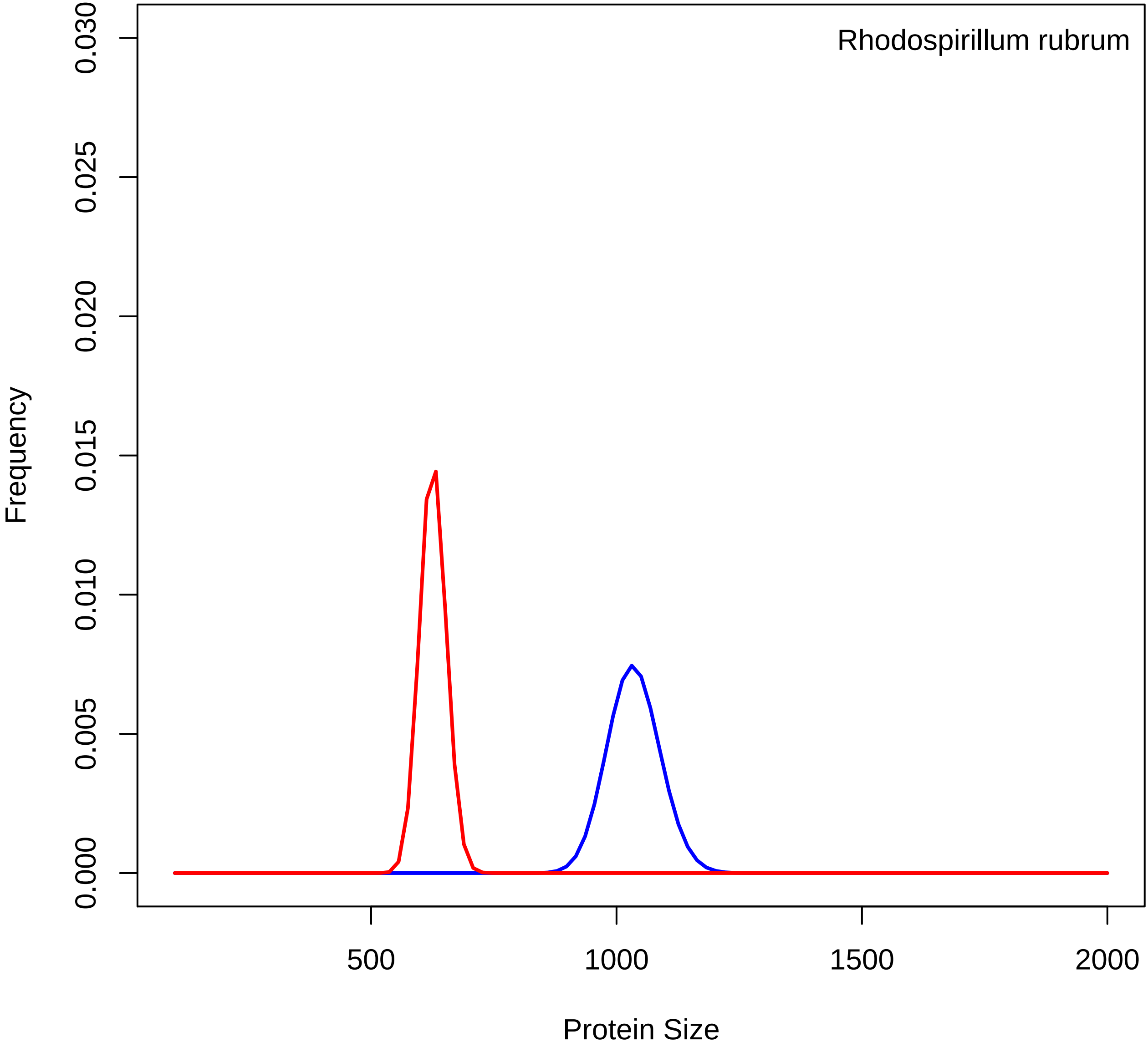

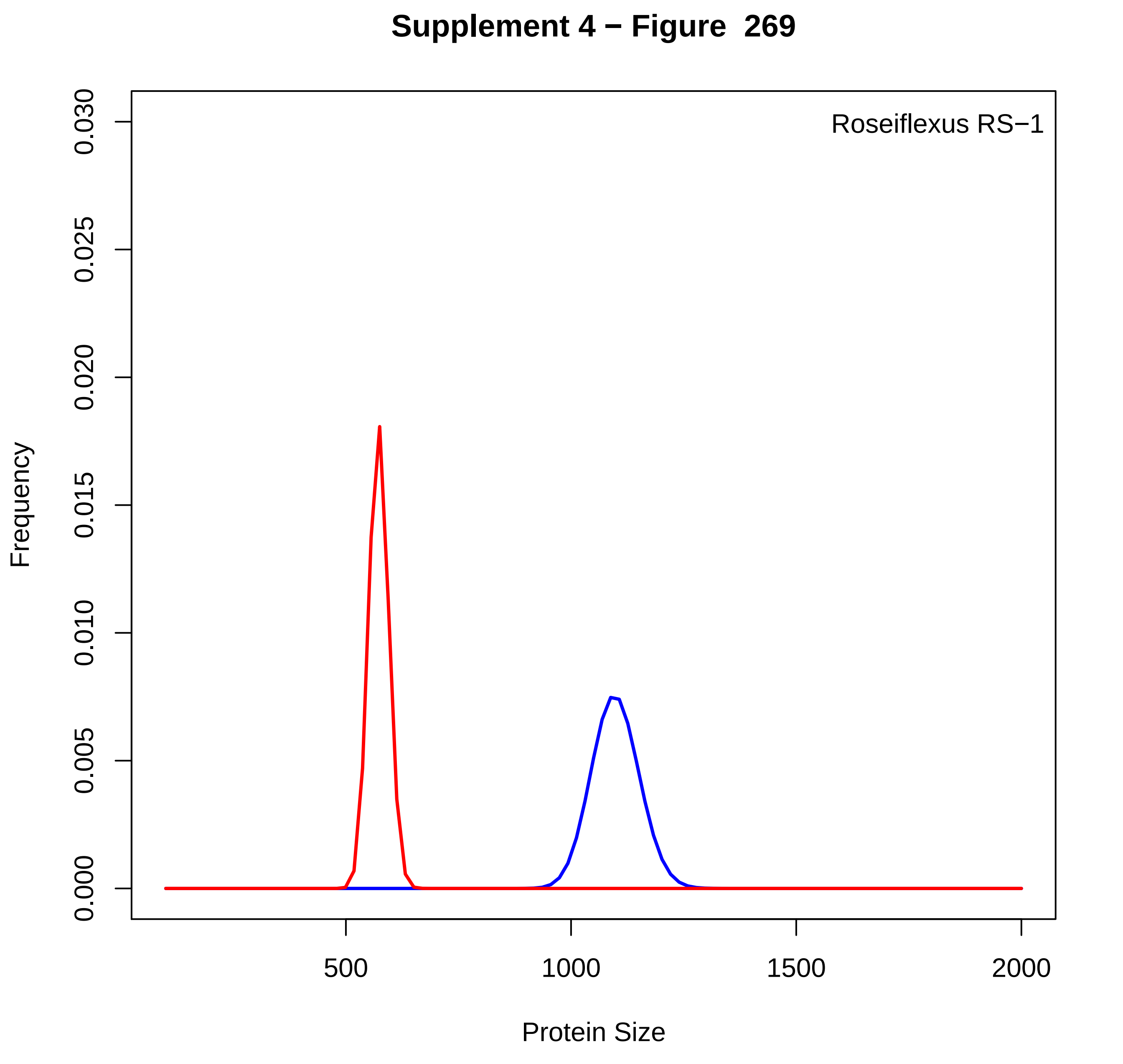
Supplement 4 − Figure 269
Roseiflexus RS−1
Frequency
Protein Size
0.000
0.005
0.010
0.015
0.020
0.025
0.030
500
1000
1500
2000

Supplement 4 − Figure 270

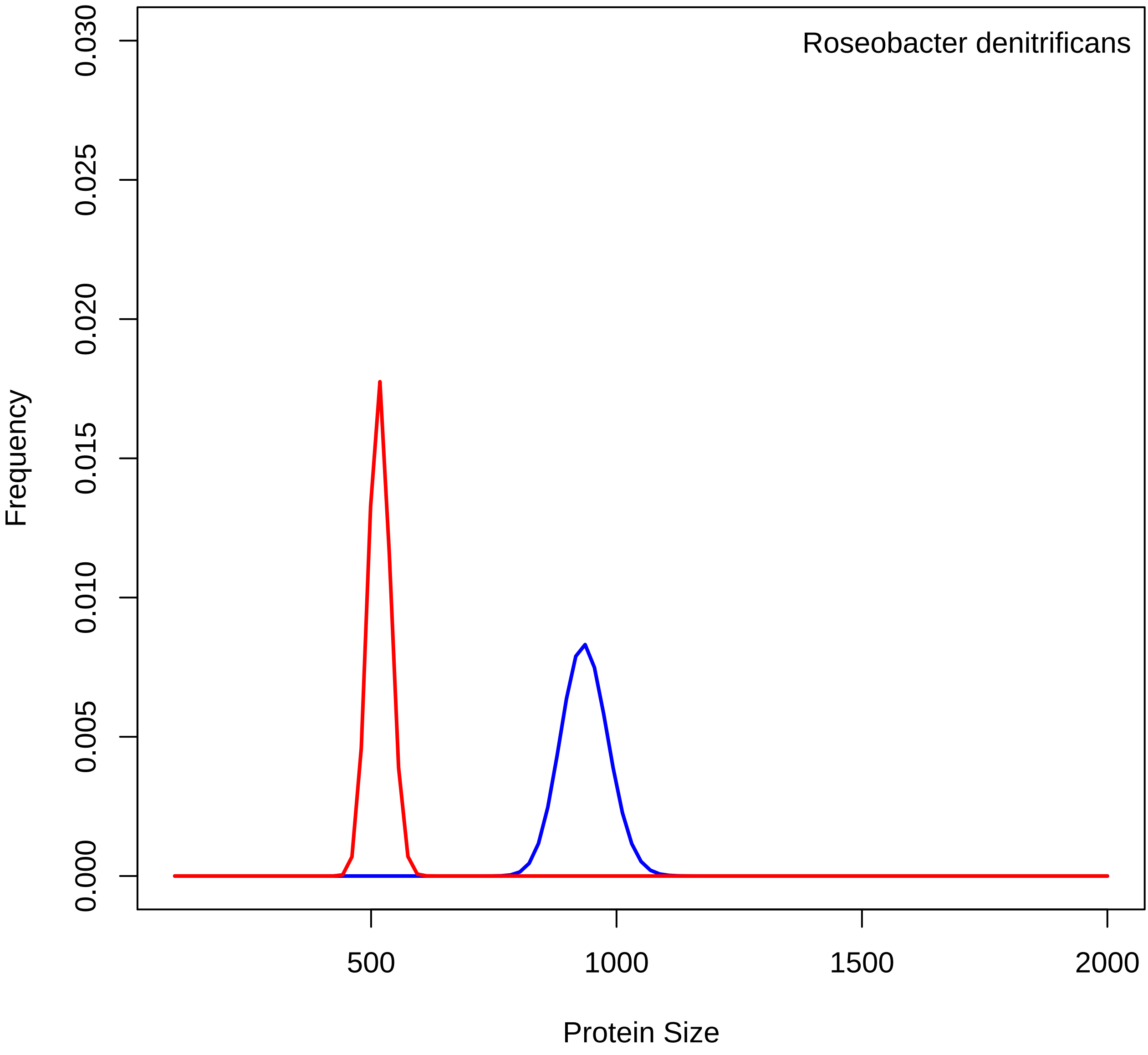

Supplement 4 − Figure 271

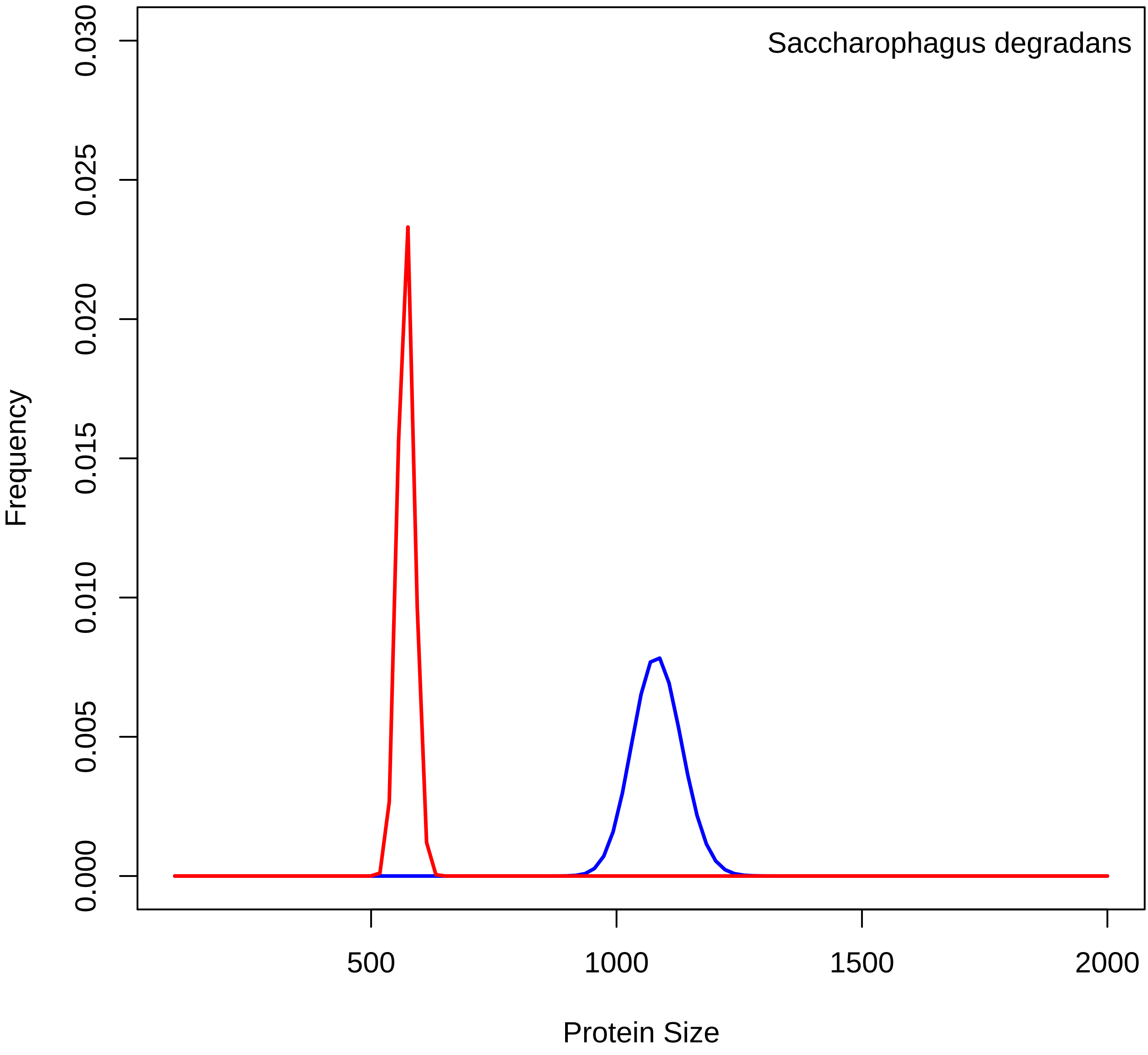

Supplement 4 − Figure 272

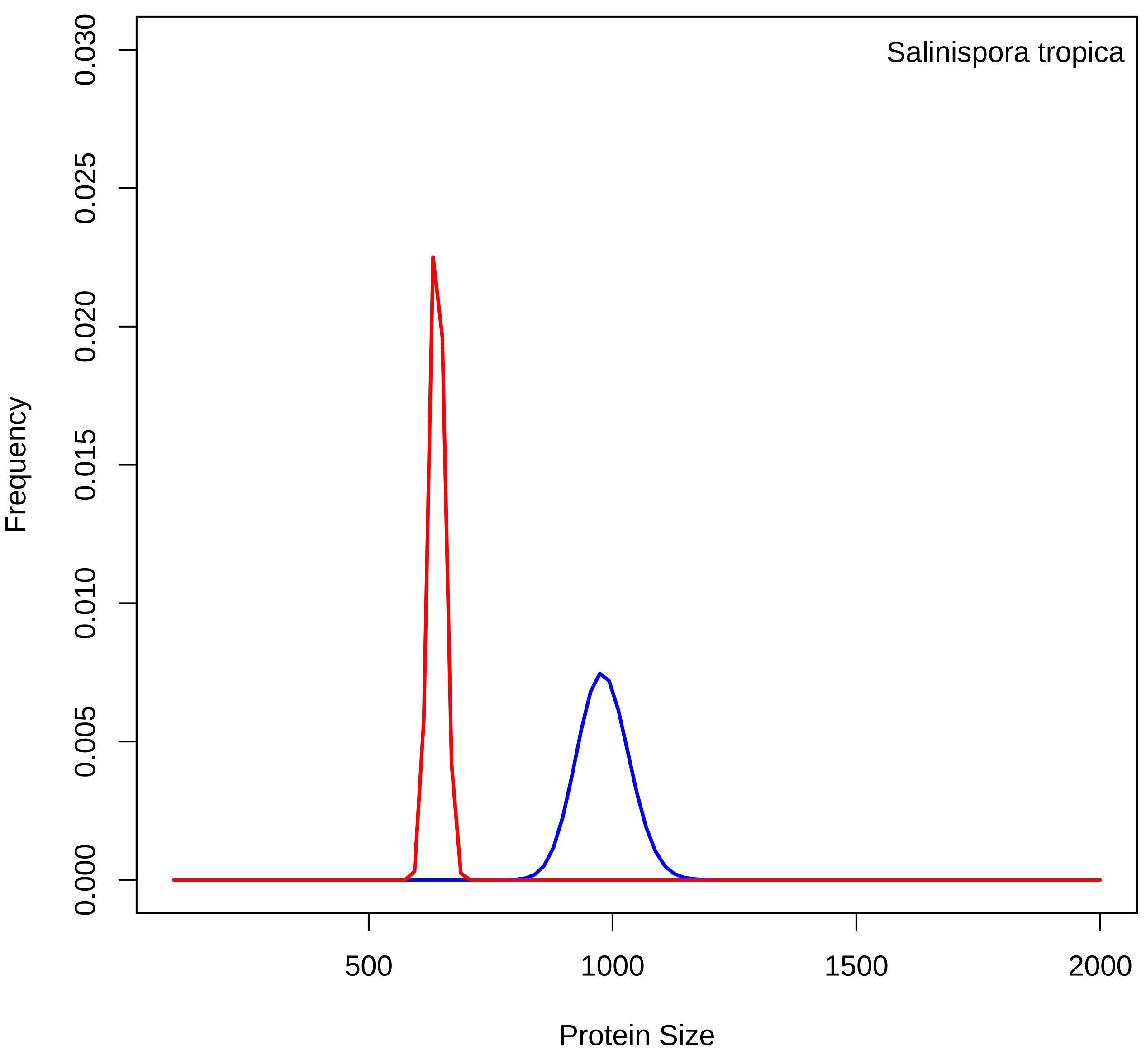

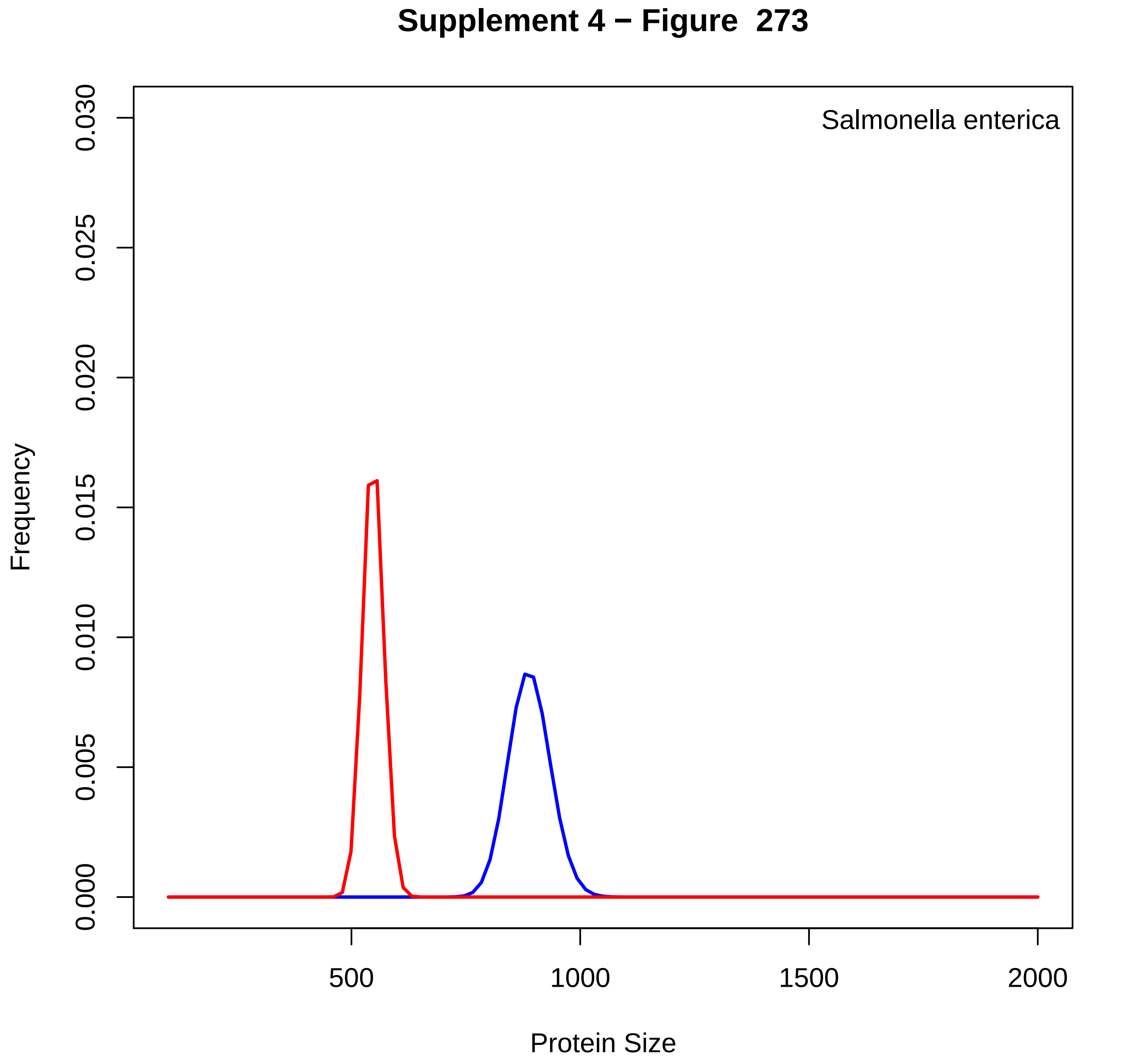
Supplement 4 − Figure 273
Salmonella enterica
Frequency
0.000
0.005
0.010
0.015
0.020
0.025
0.030
500
1000
1500
2000
Protein Size

Supplement 4 − Figure 274

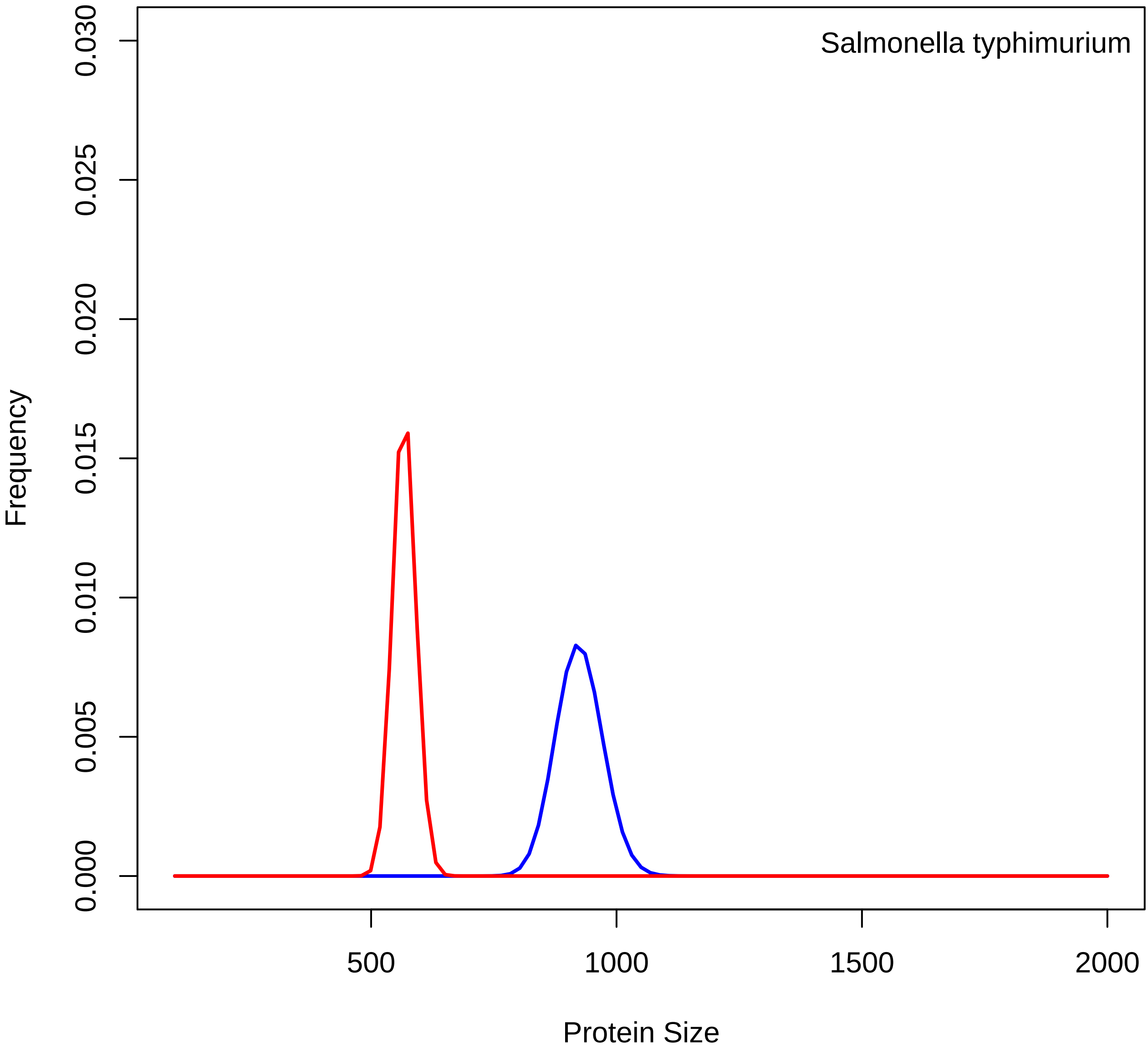

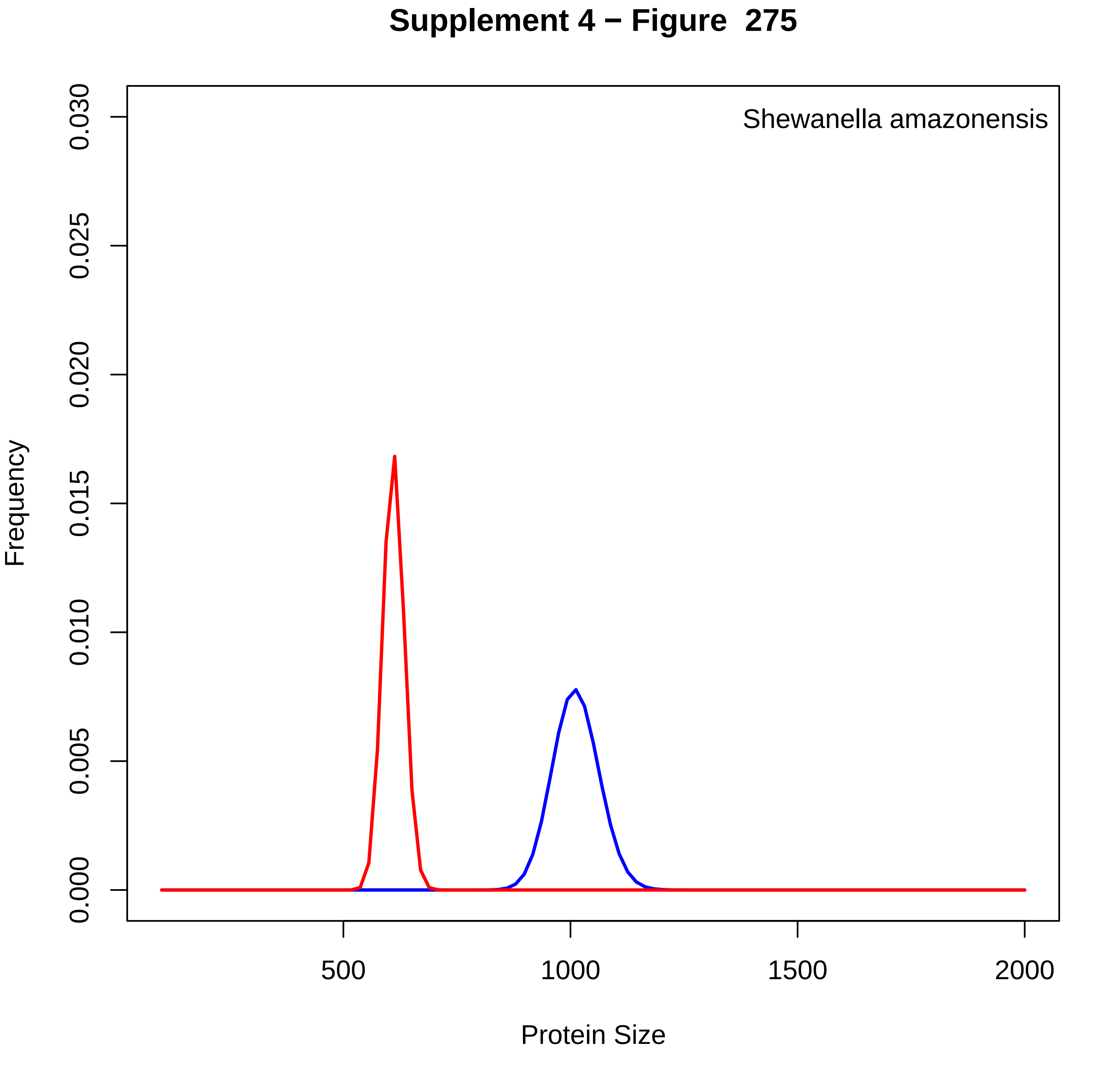
Supplement 4 − Figure 275
Shewanella amazonensis
Frequency
0.000
0.005
0.010
0.015
0.020
0.025
0.030
500
1000
1500
2000
Protein Size

Supplement 4 − Figure 276

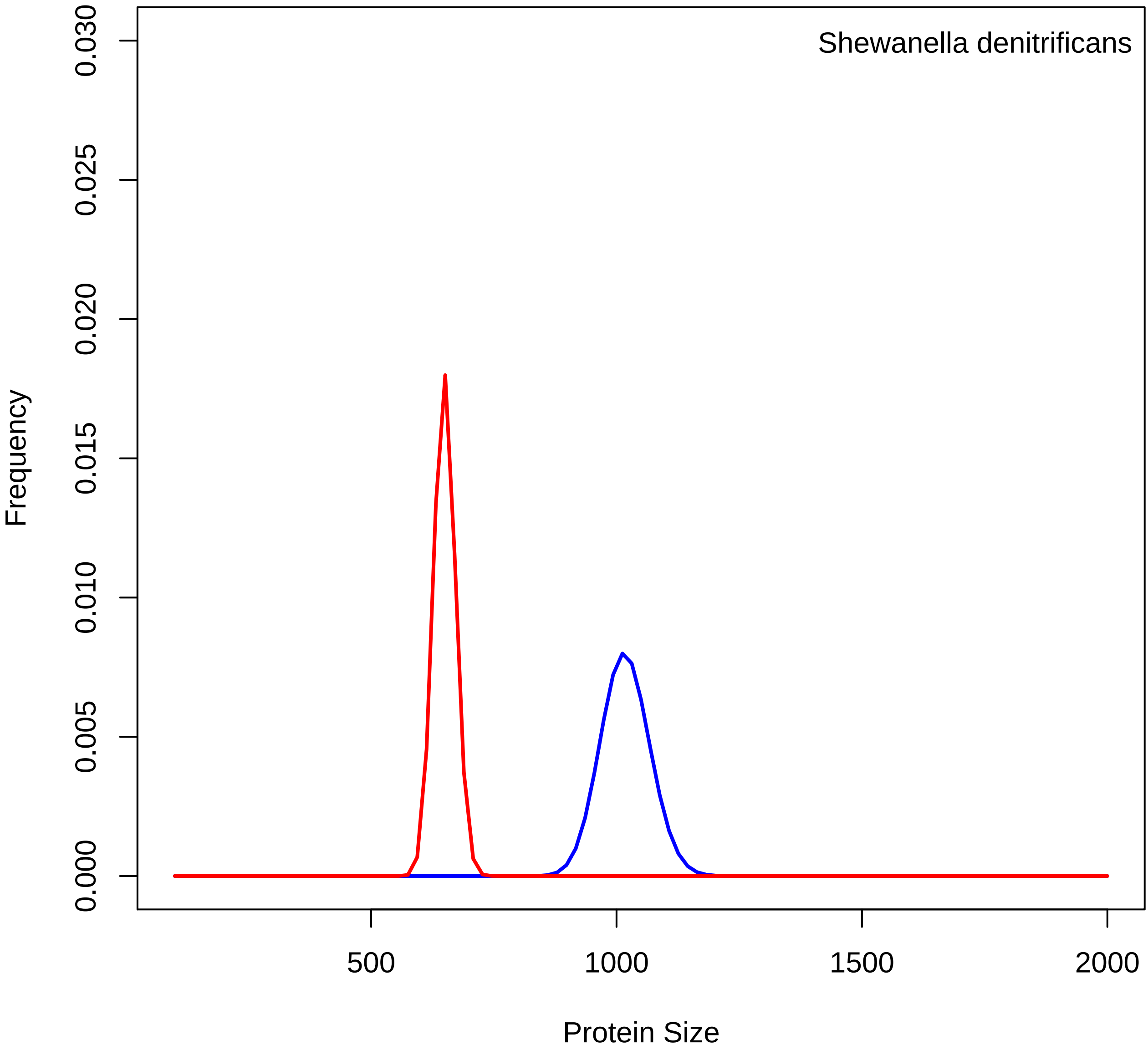

Supplement 4 − Figure 277

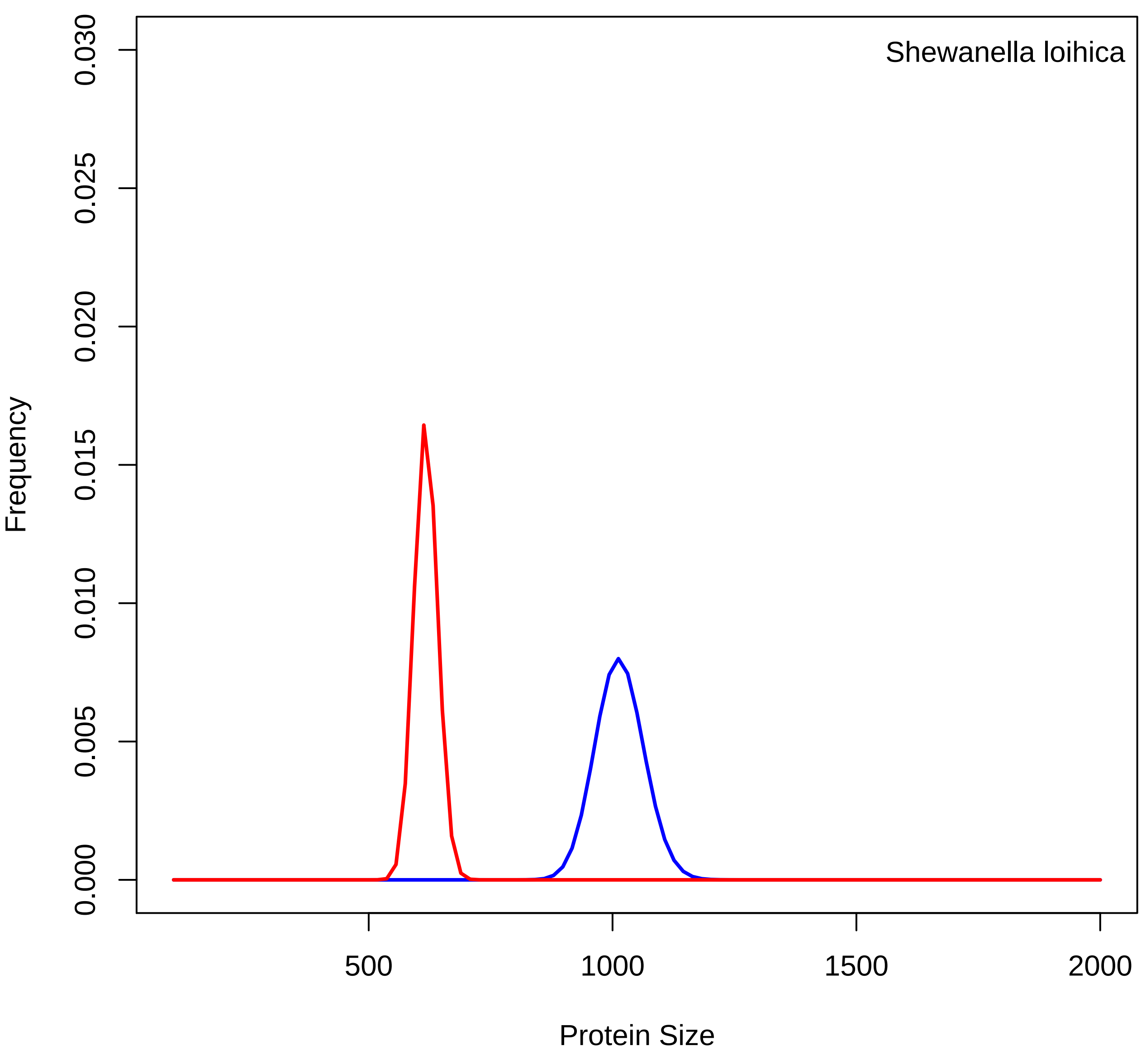

Supplement 4 − Figure 278

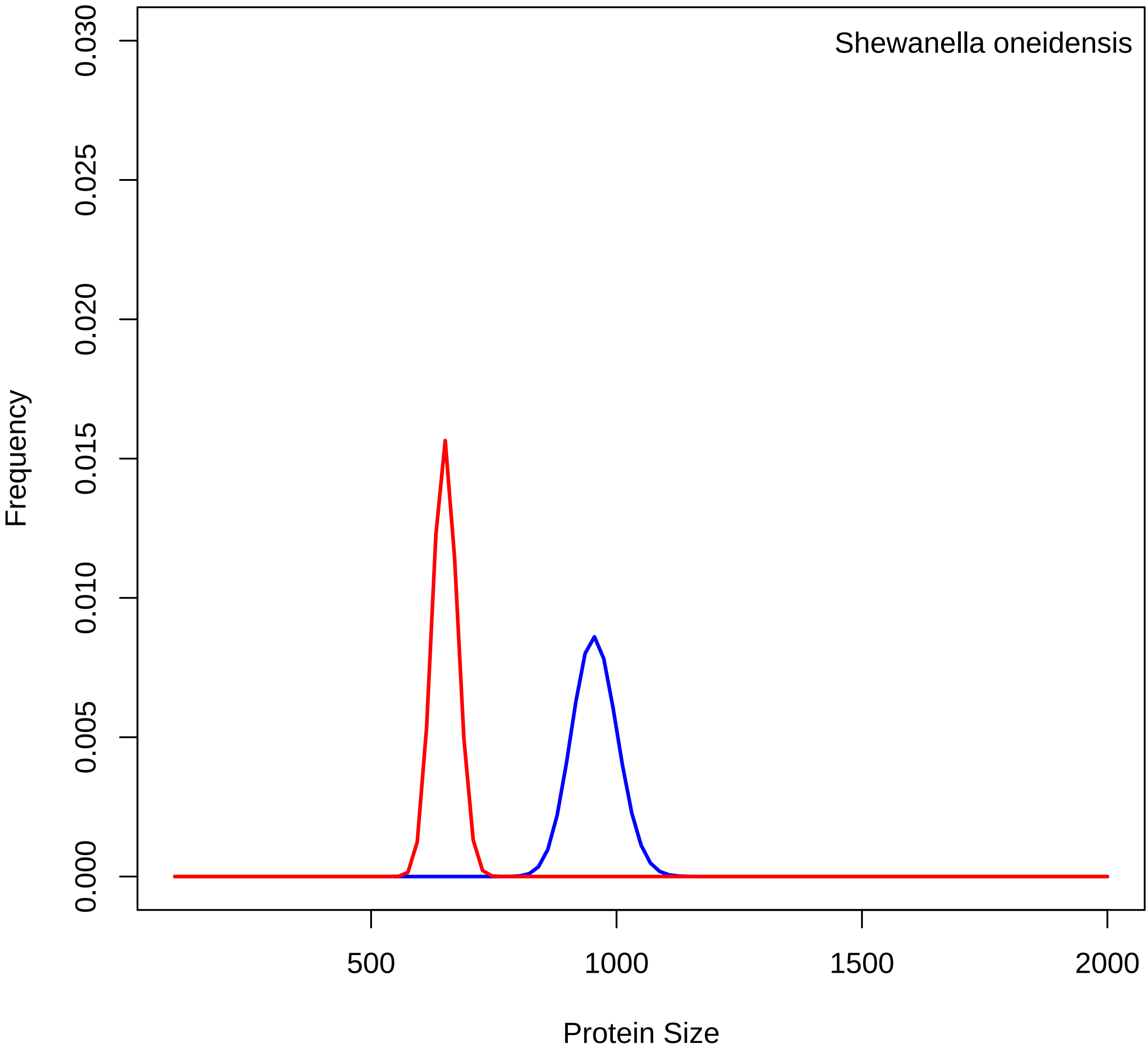

Supplement 4 − Figure 279

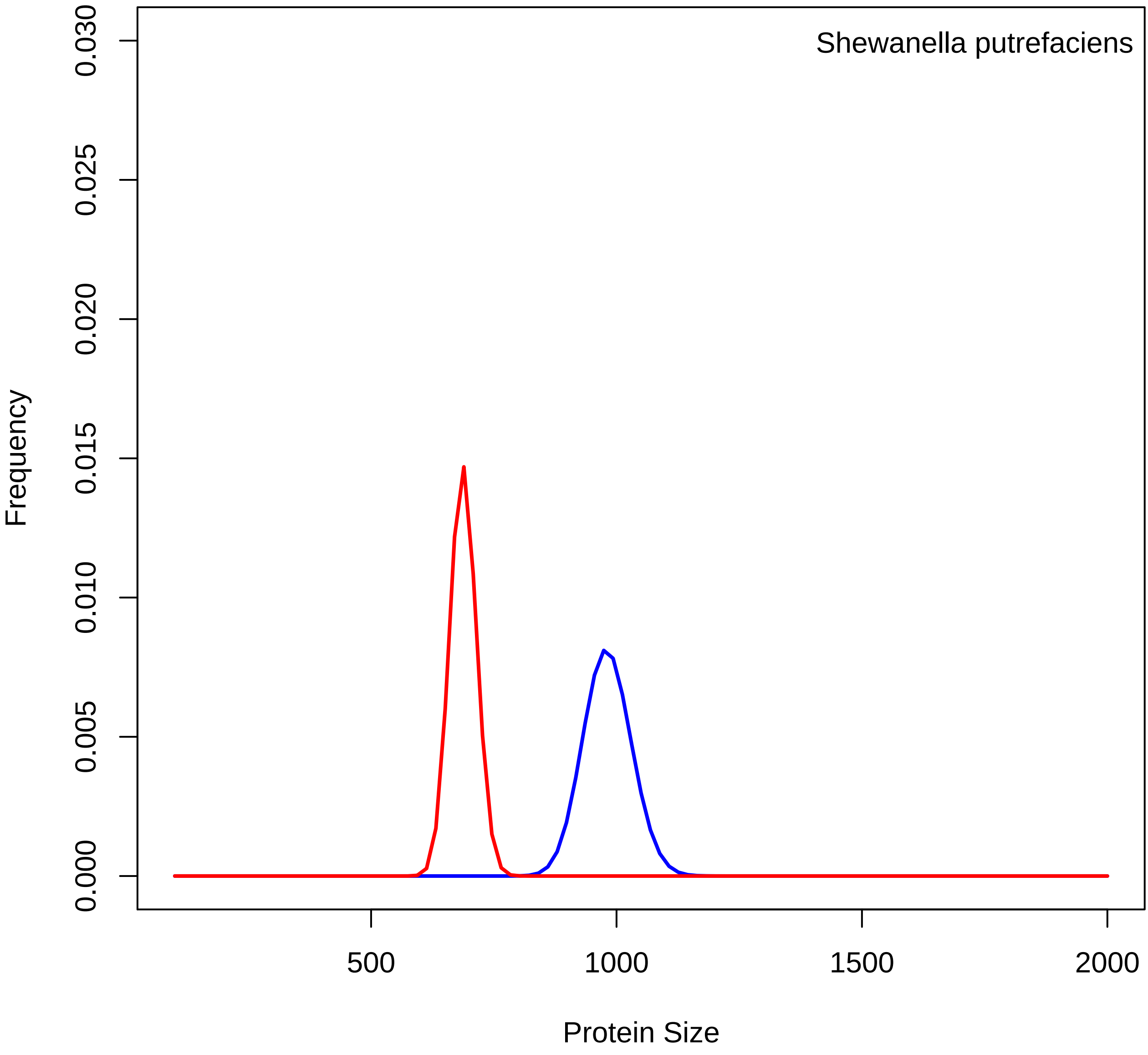

Supplement 4 − Figure 280

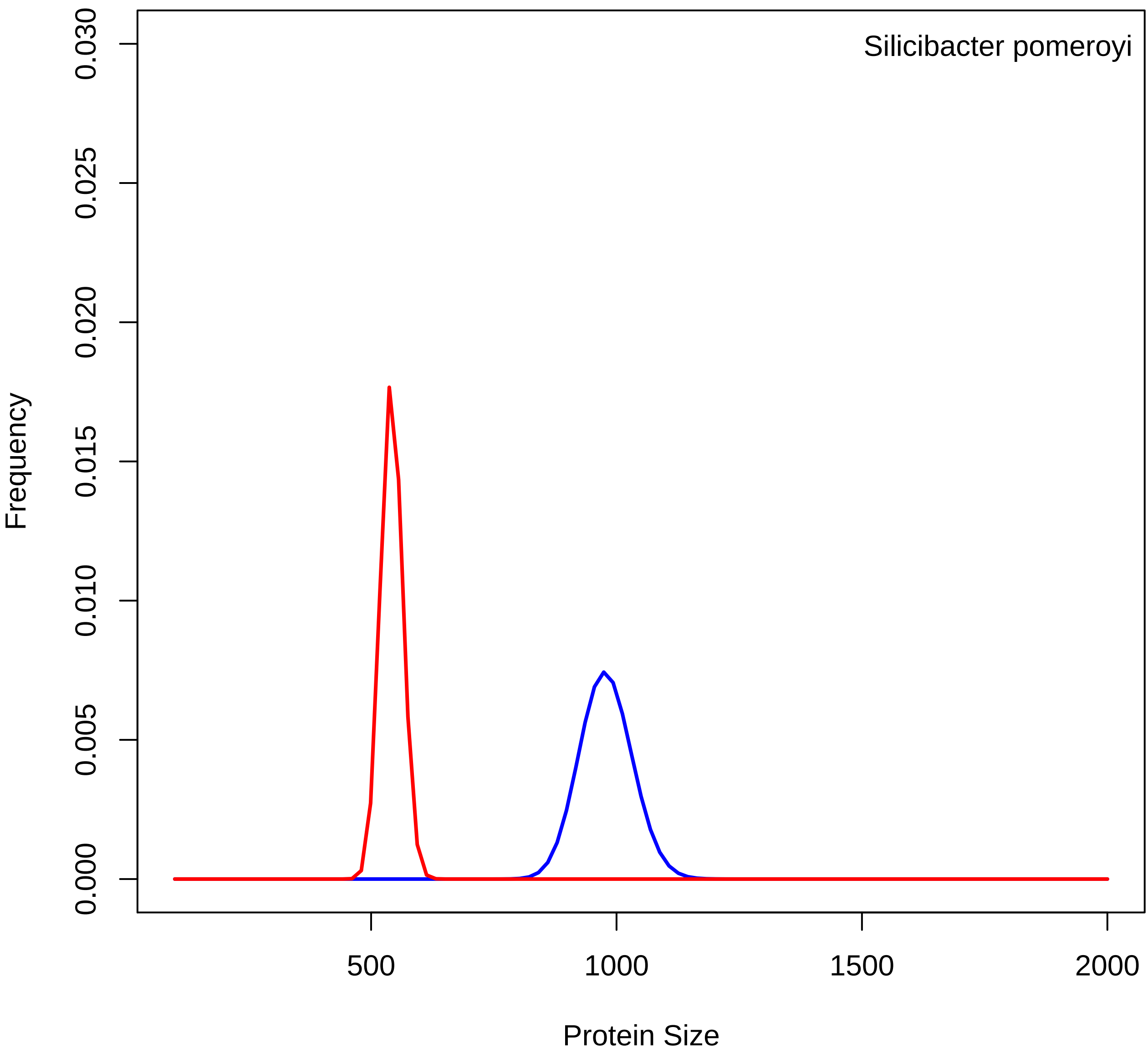

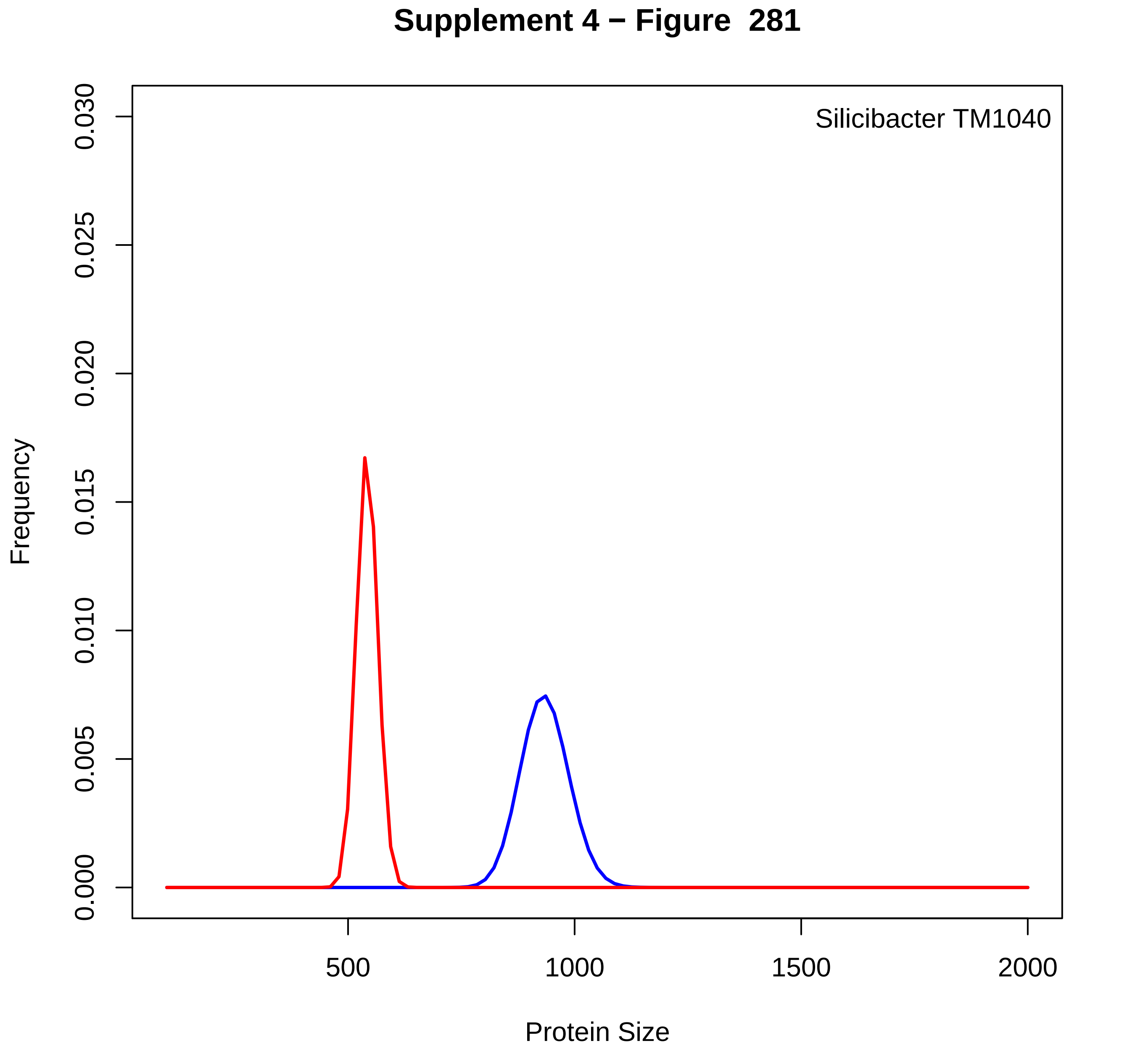
Supplement 4 − Figure 281
Silicibacter TM1040
Frequency
0.000
0.005
0.010
0.015
0.020
0.025
0.030
500
1000
1500
2000
Protein Size

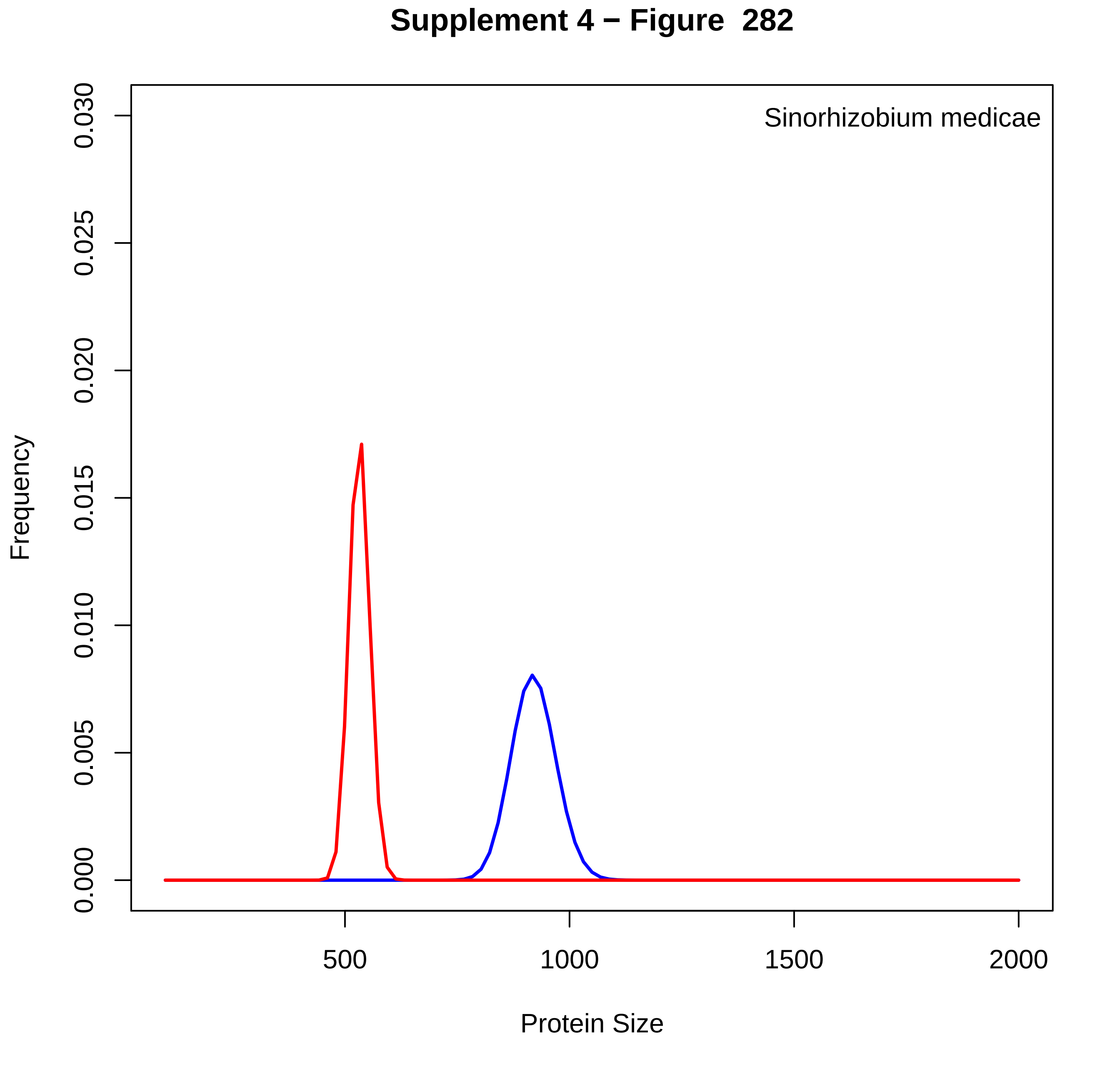
Supplement 4 − Figure 282
Sinorhizobium medicae
Frequency
0.000
0.005
0.010
0.015
0.020
0.025
0.030
500
1000
1500
2000
Protein Size

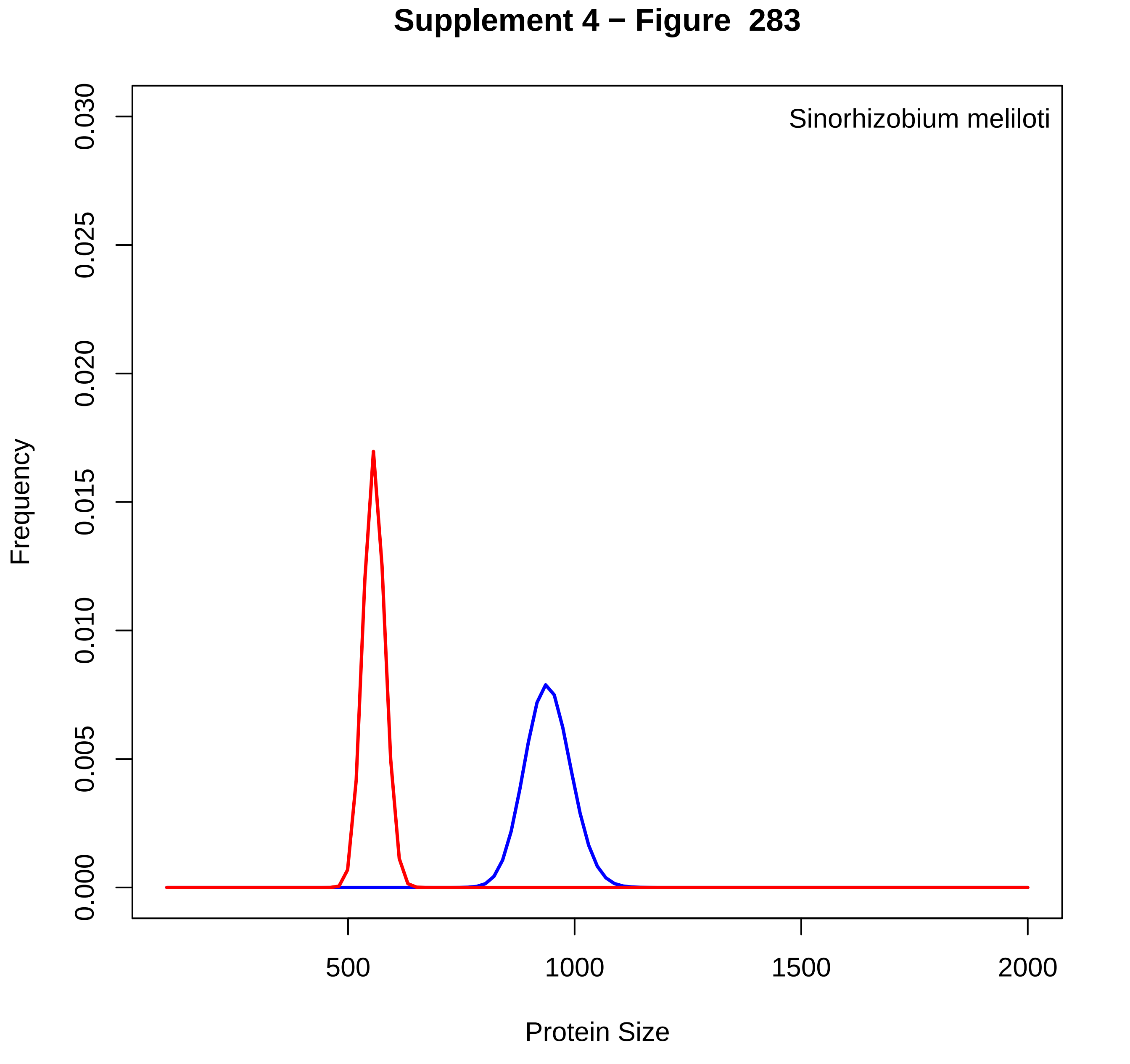

Supplement 4 − Figure 283
Sinorhizobium meliloti
Frequency
0.000
0.005
0.010
0.015
0.020
0.025
0.030
500
1000
1500
2000
Protein Size

Supplement 4 – Figure 284

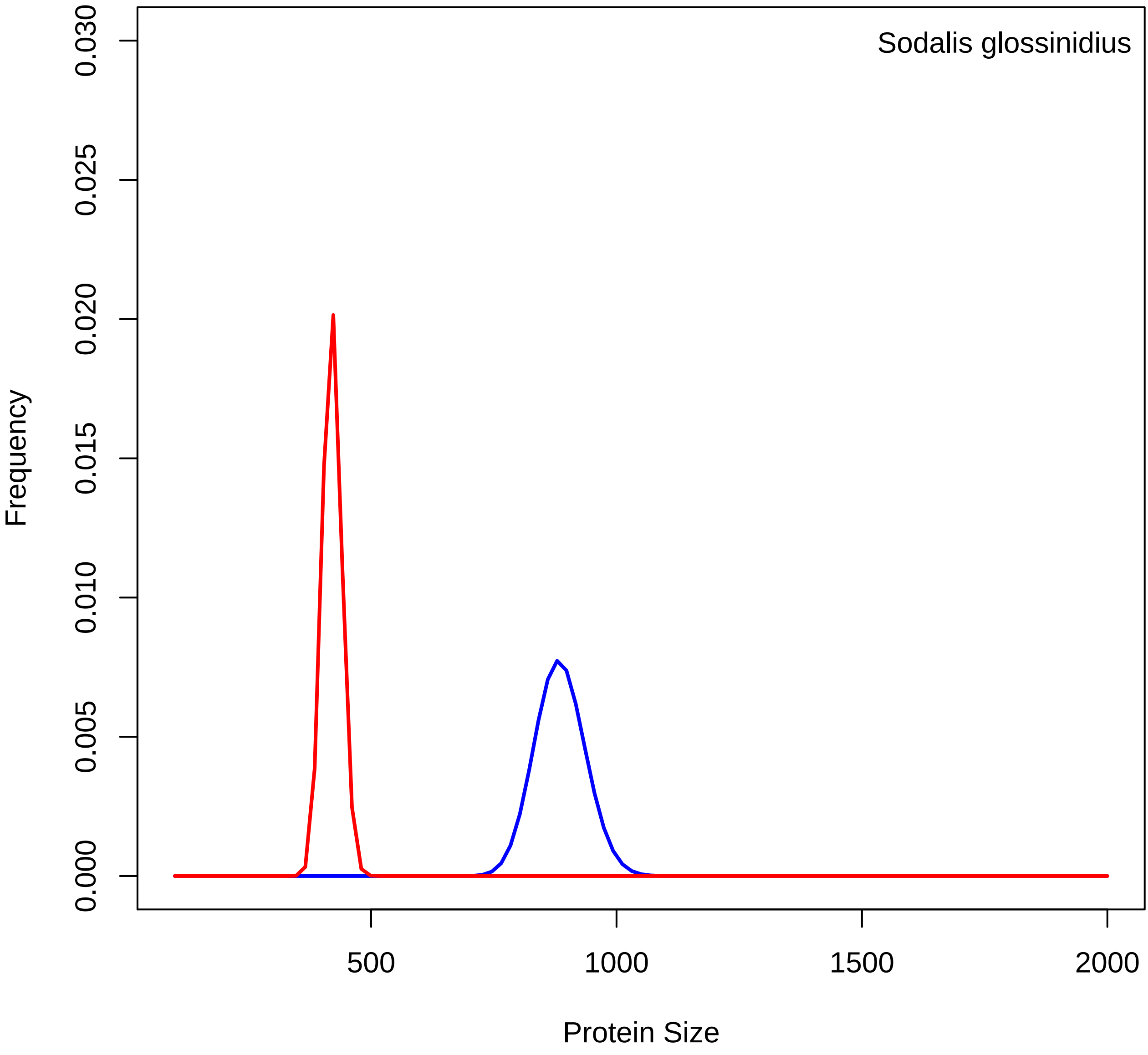

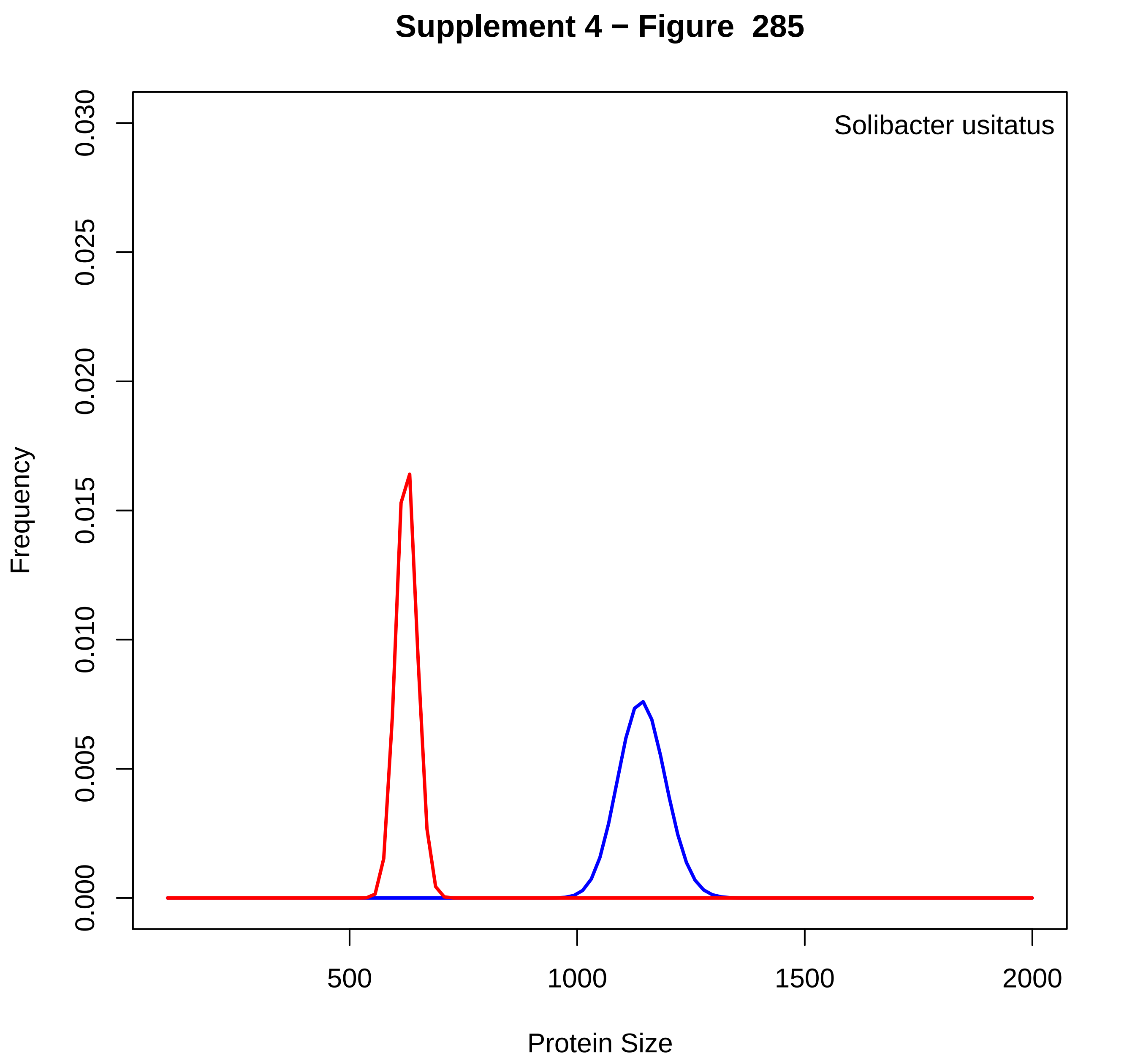
Supplement 4 − Figure 285
Solibacter usitatus
Frequency
0.000
0.005
0.010
0.015
0.020
0.025
0.030
500
1000
1500
2000
Protein Size

Supplement 4 − Figure 286

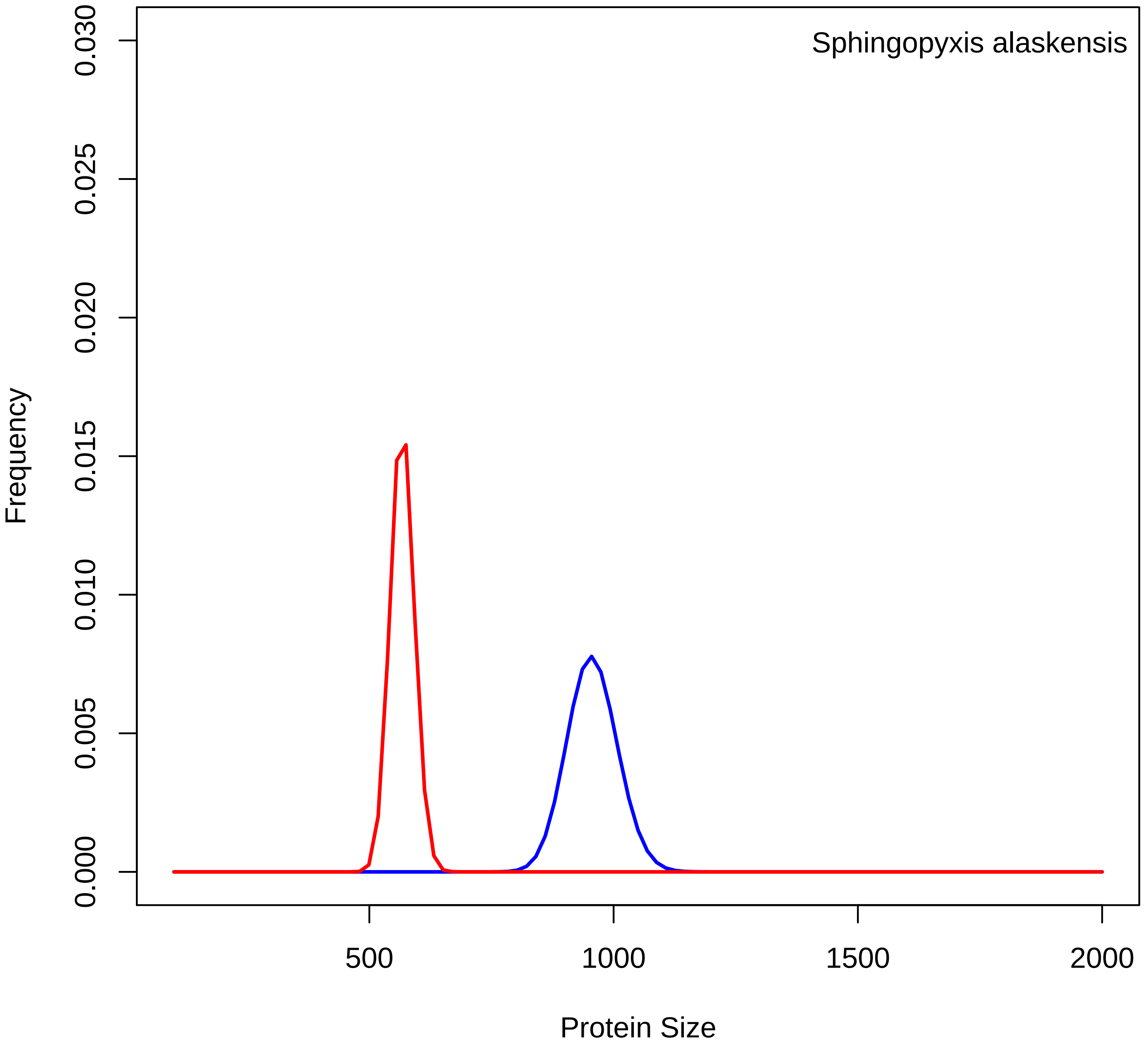

Supplement 4 − Figure 287

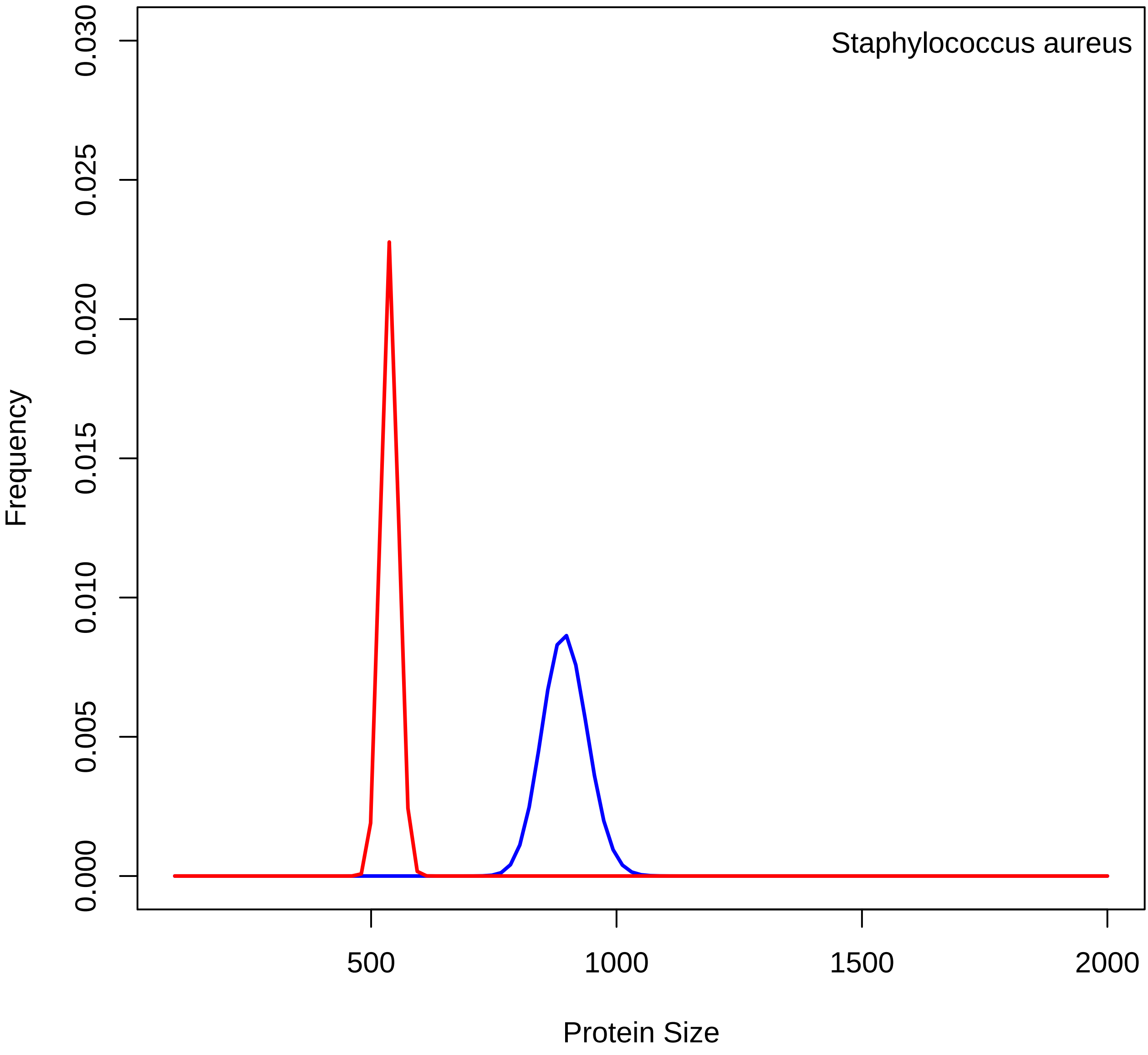

Supplement 4 − Figure 288

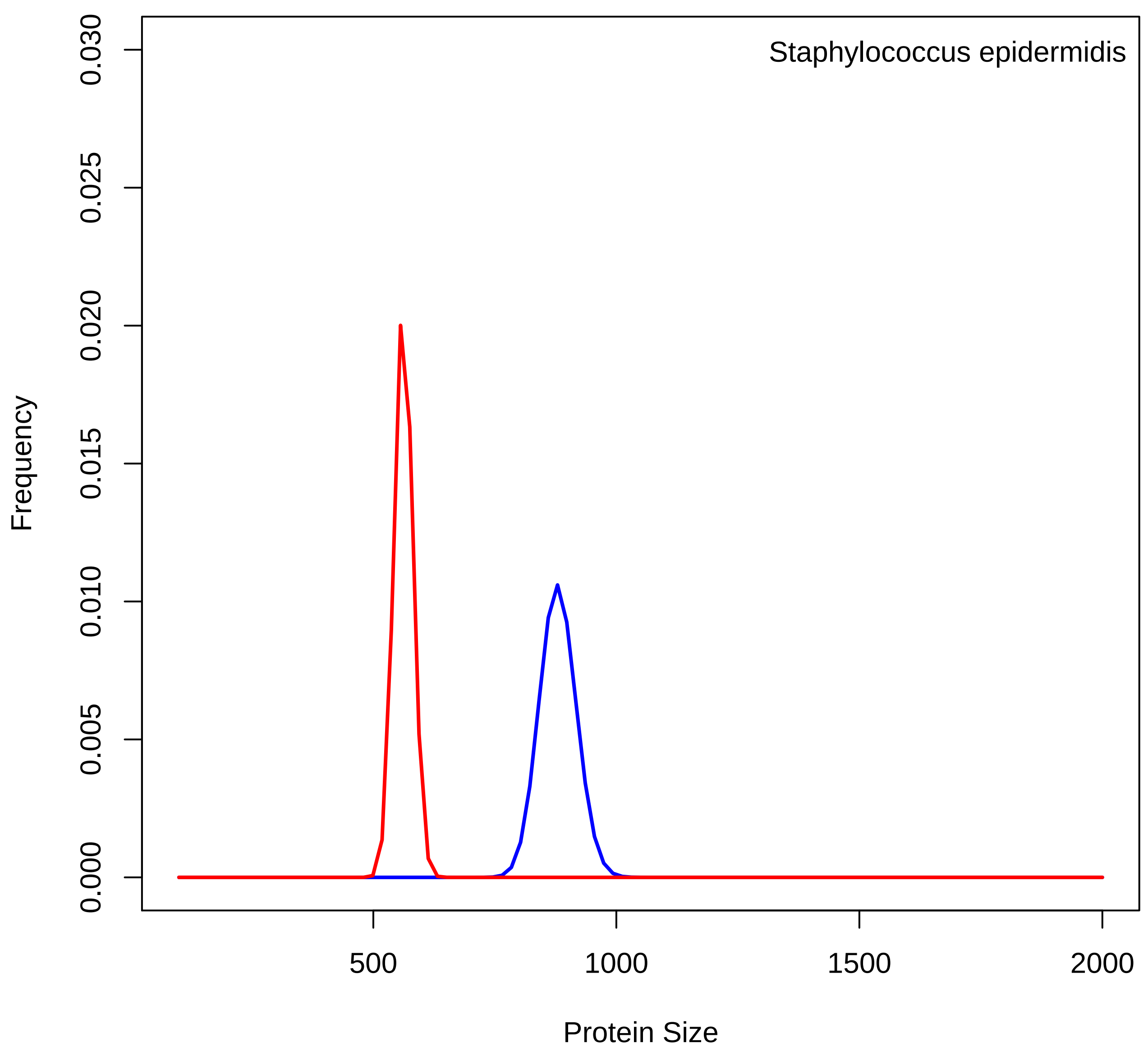

Supplement 4 − Figure 289

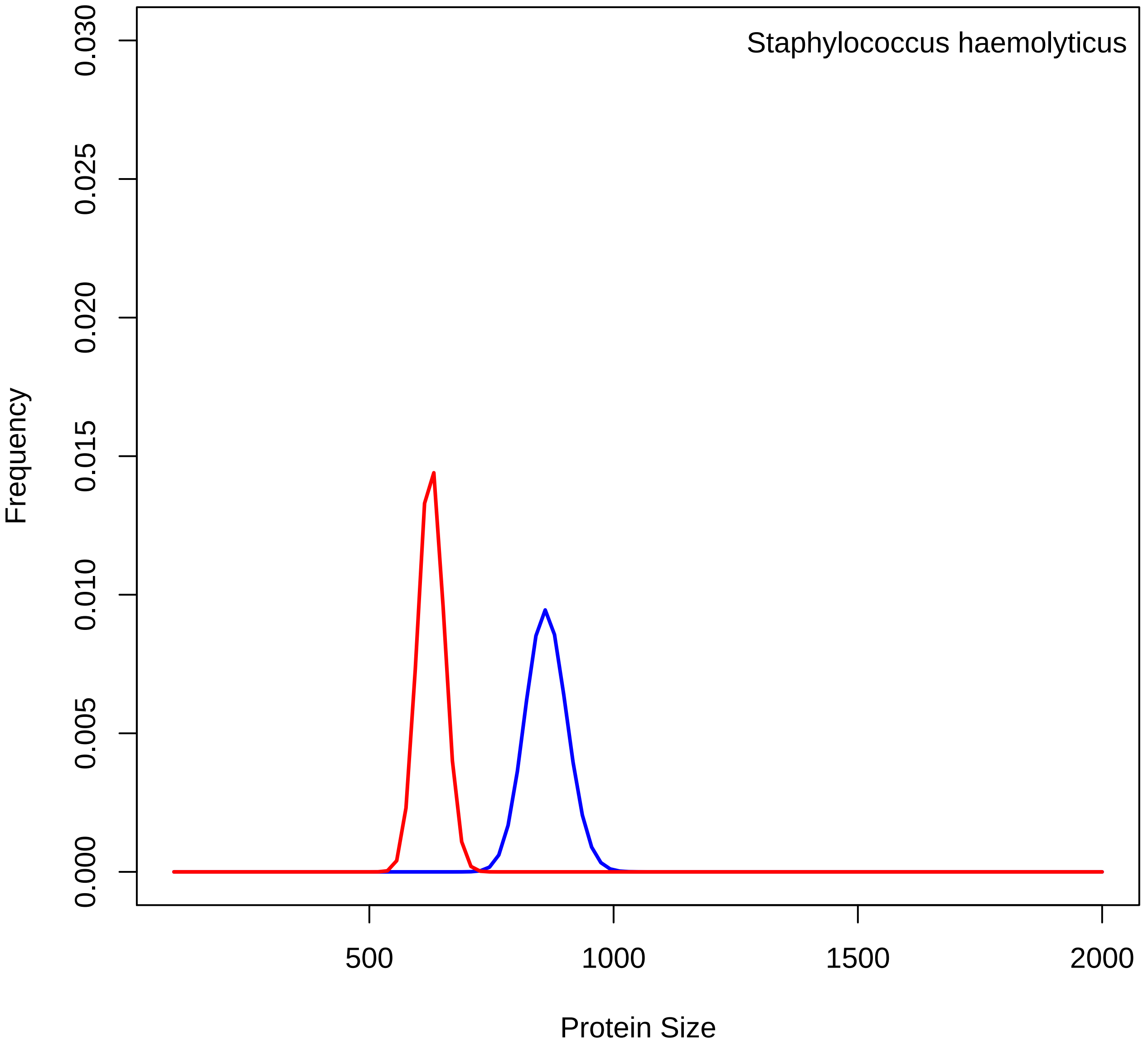

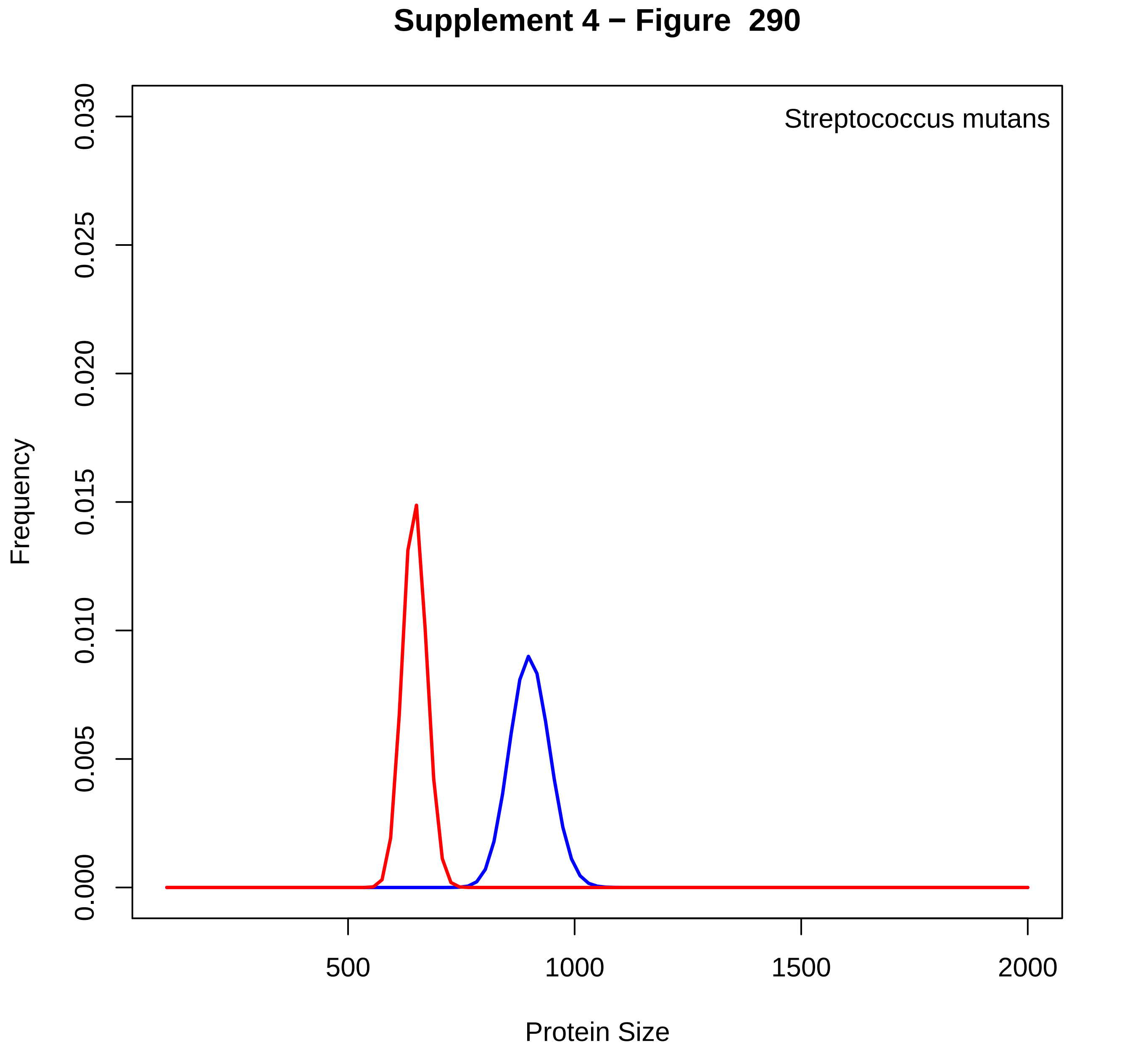

Supplement 4 − Figure 290
Streptococcus mutans
Frequency
0.000
0.005
0.010
0.015
0.020
0.025
0.030
500
1000
1500
2000
Protein Size

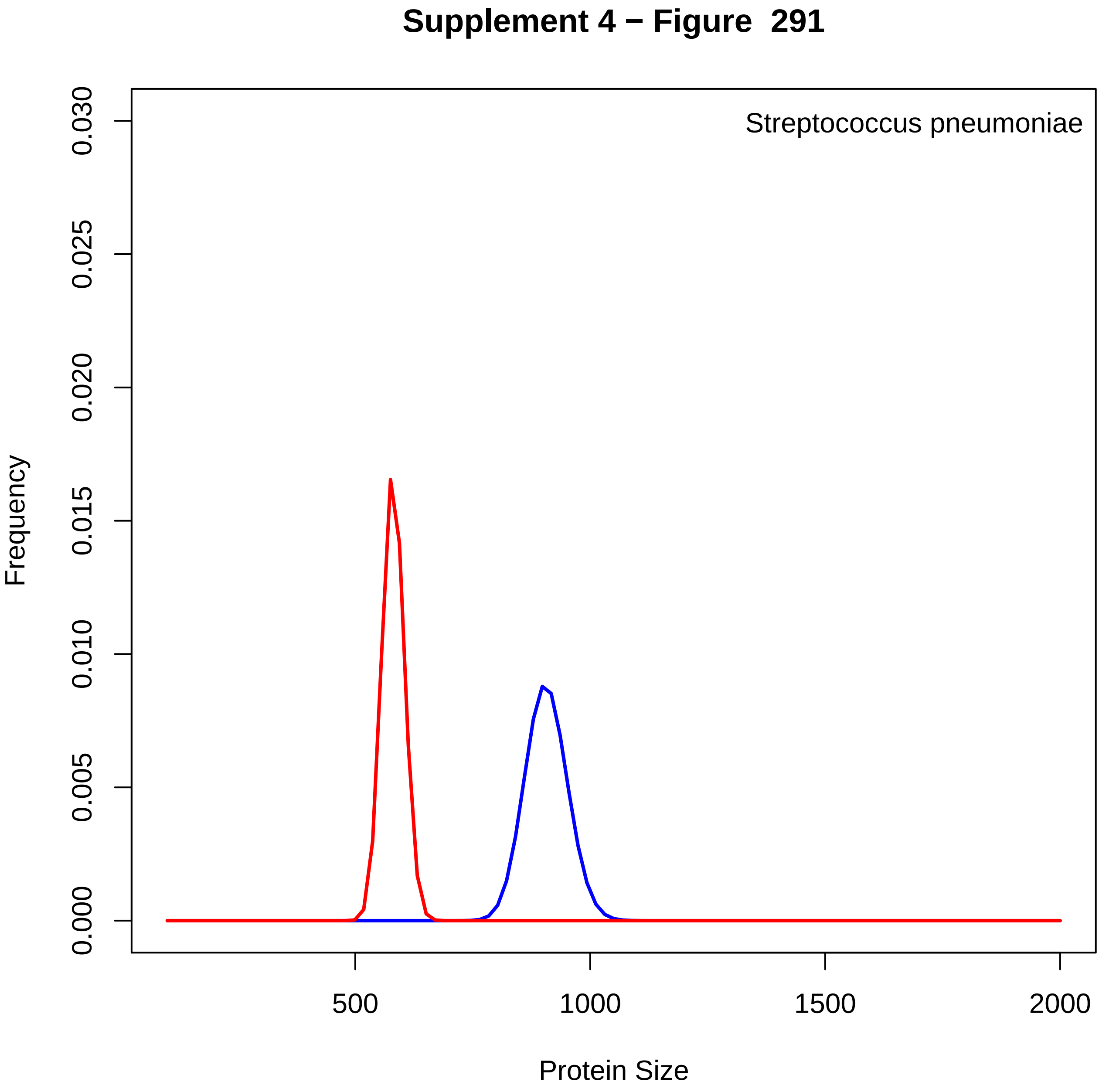
Supplement 4 − Figure 291
Streptococcus pneumoniae
Frequency
0.000
0.005
0.010
0.015
0.020
0.025
0.030
500
1000
1500
2000
Protein Size

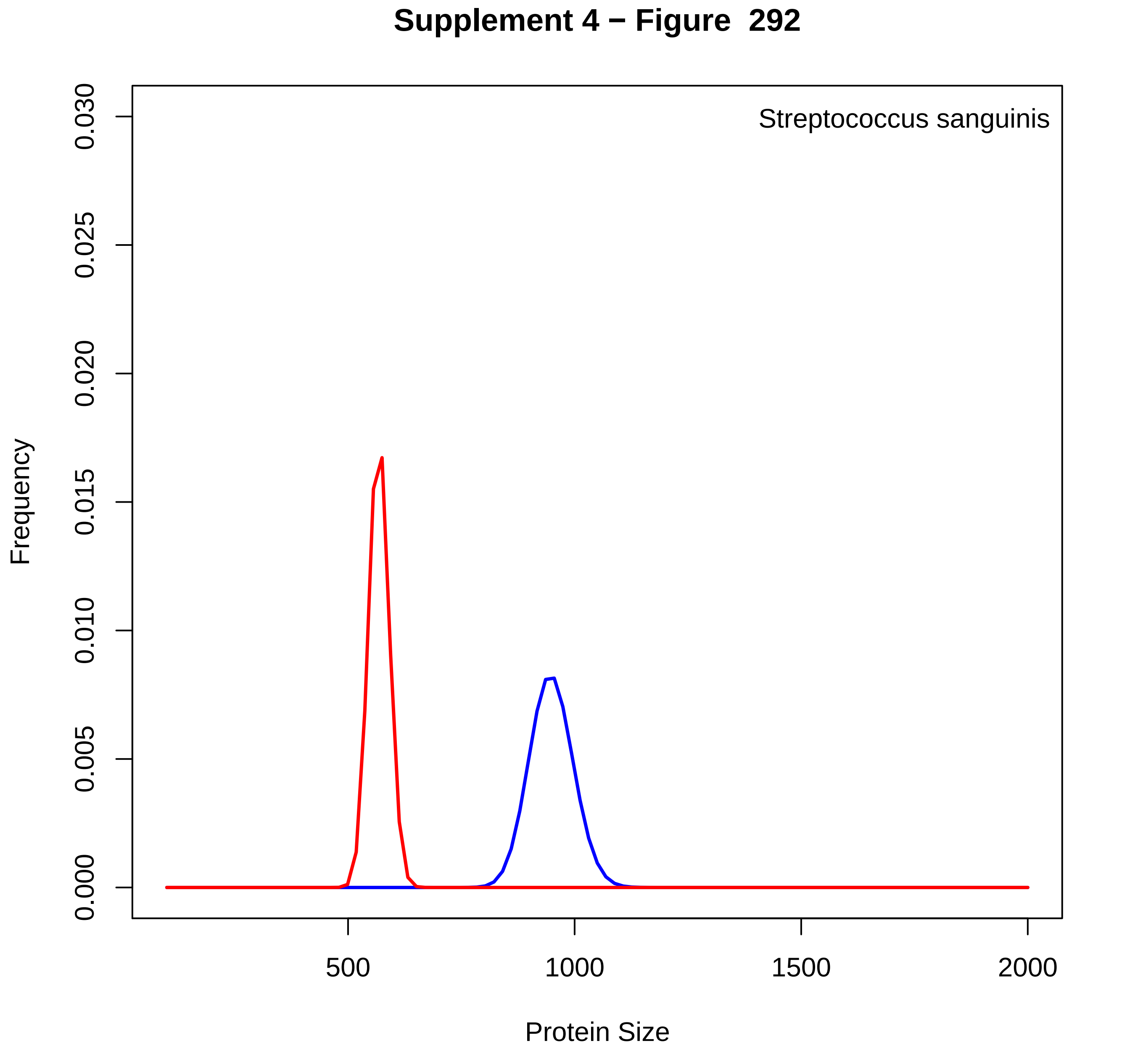
Supplement 4 − Figure 292
Streptococcus sanguinis
Frequency
0.000
0.005
0.010
0.015
0.020
0.025
0.030
500
1000
1500
2000
Protein Size

Supplement 4 − Figure 293

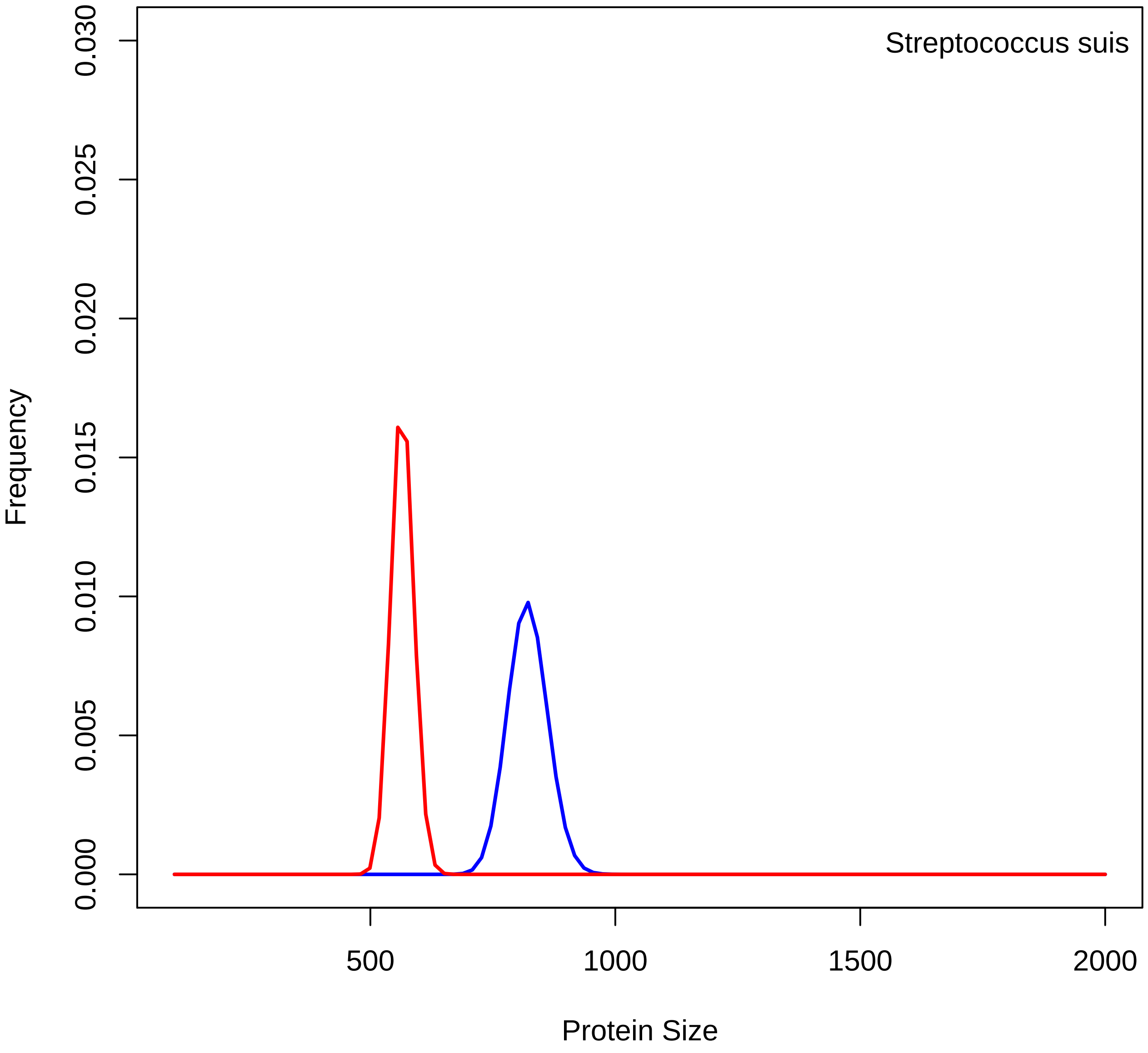

Supplement 4 − Figure 294

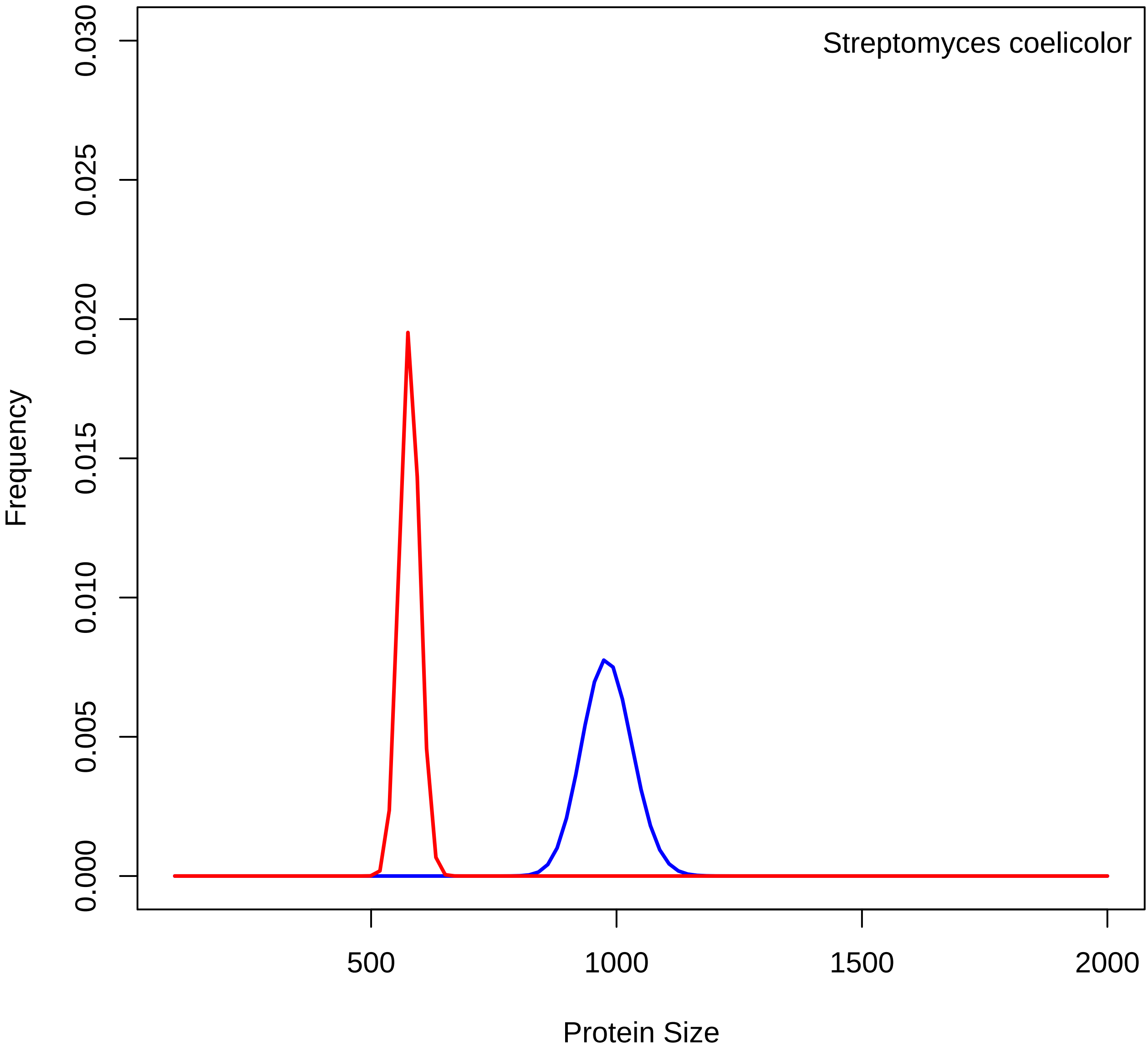

Supplement 4 − Figure 295

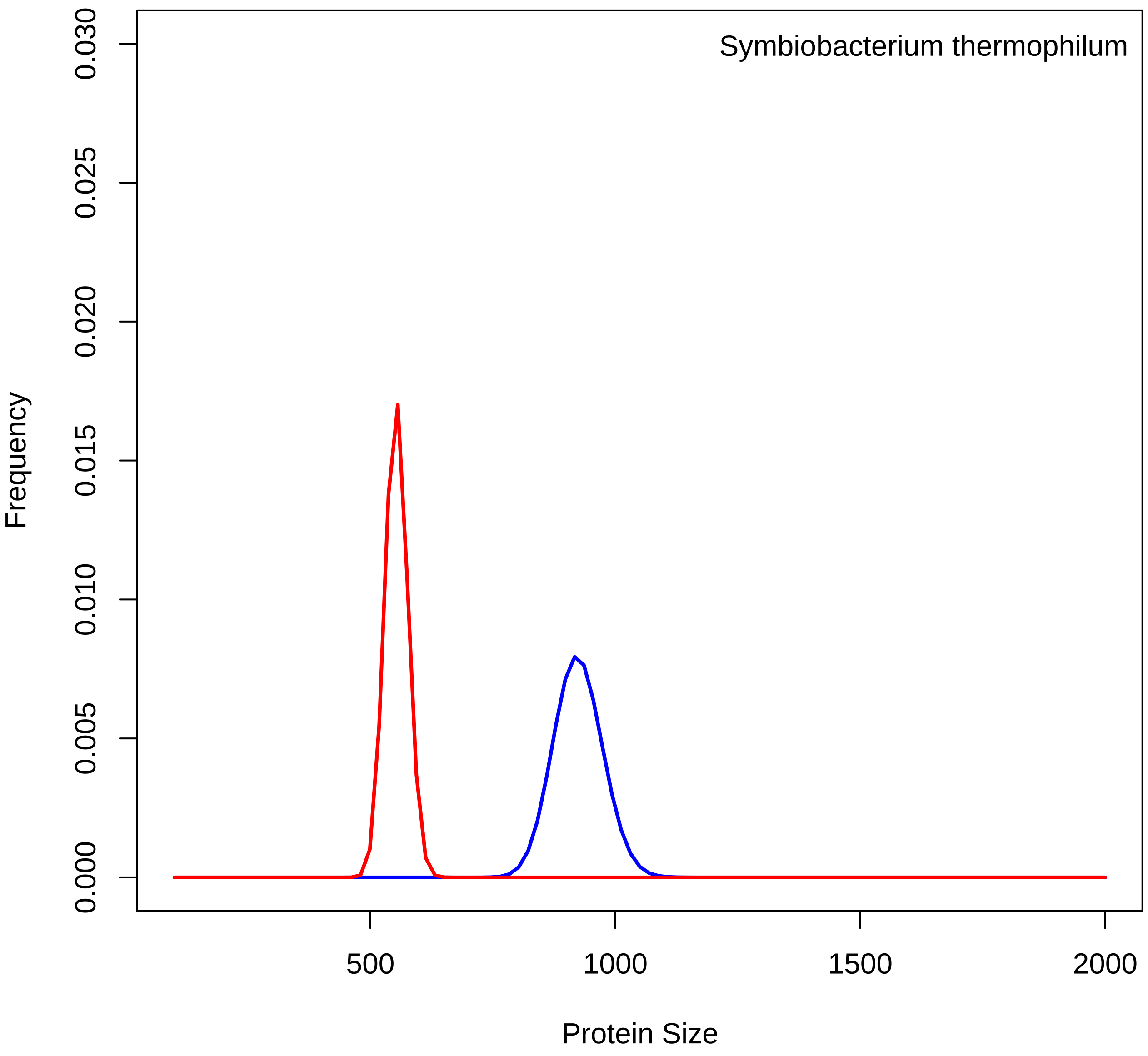

Supplement 4 − Figure 296

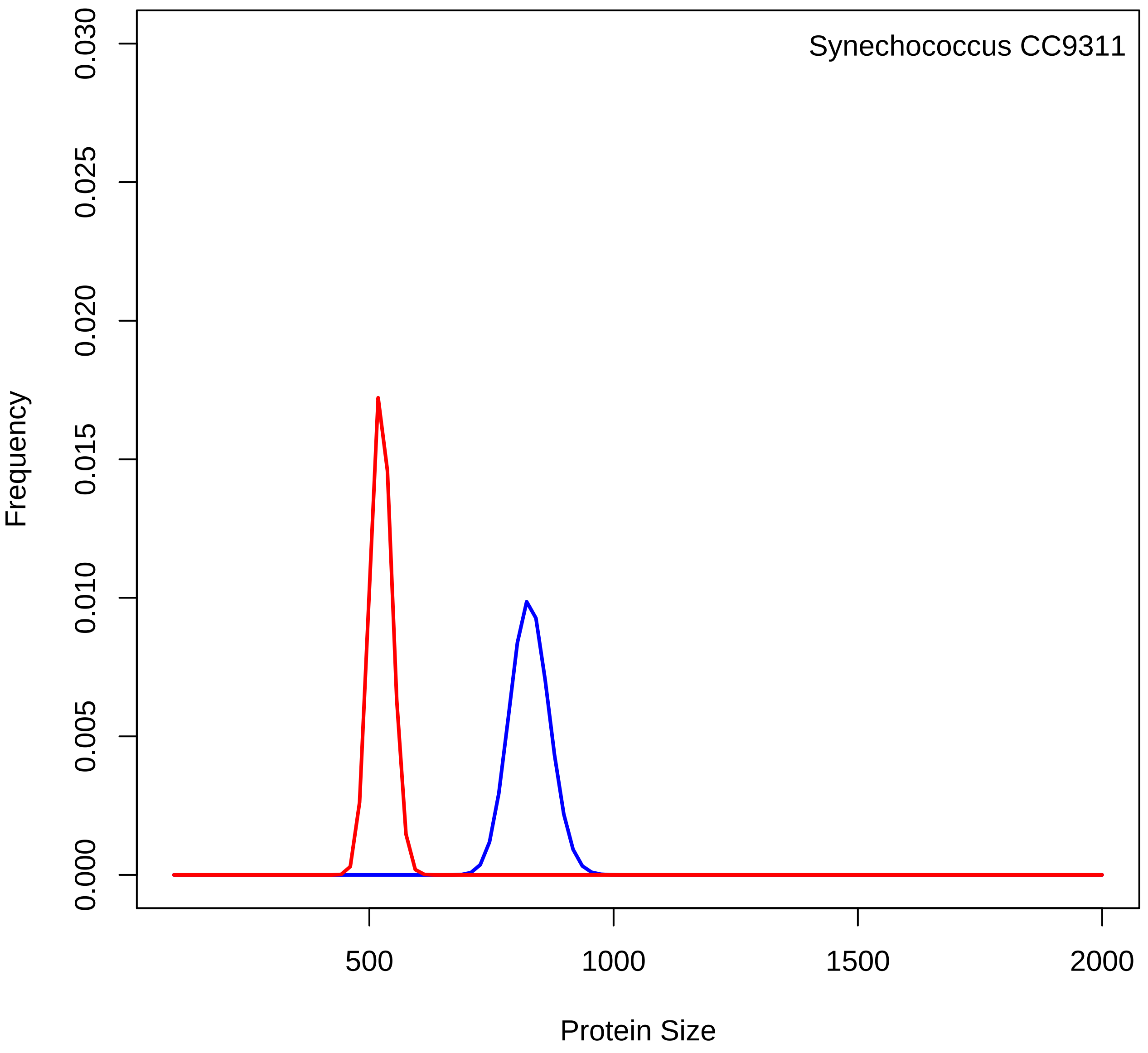

Supplement 4 − Figure 297

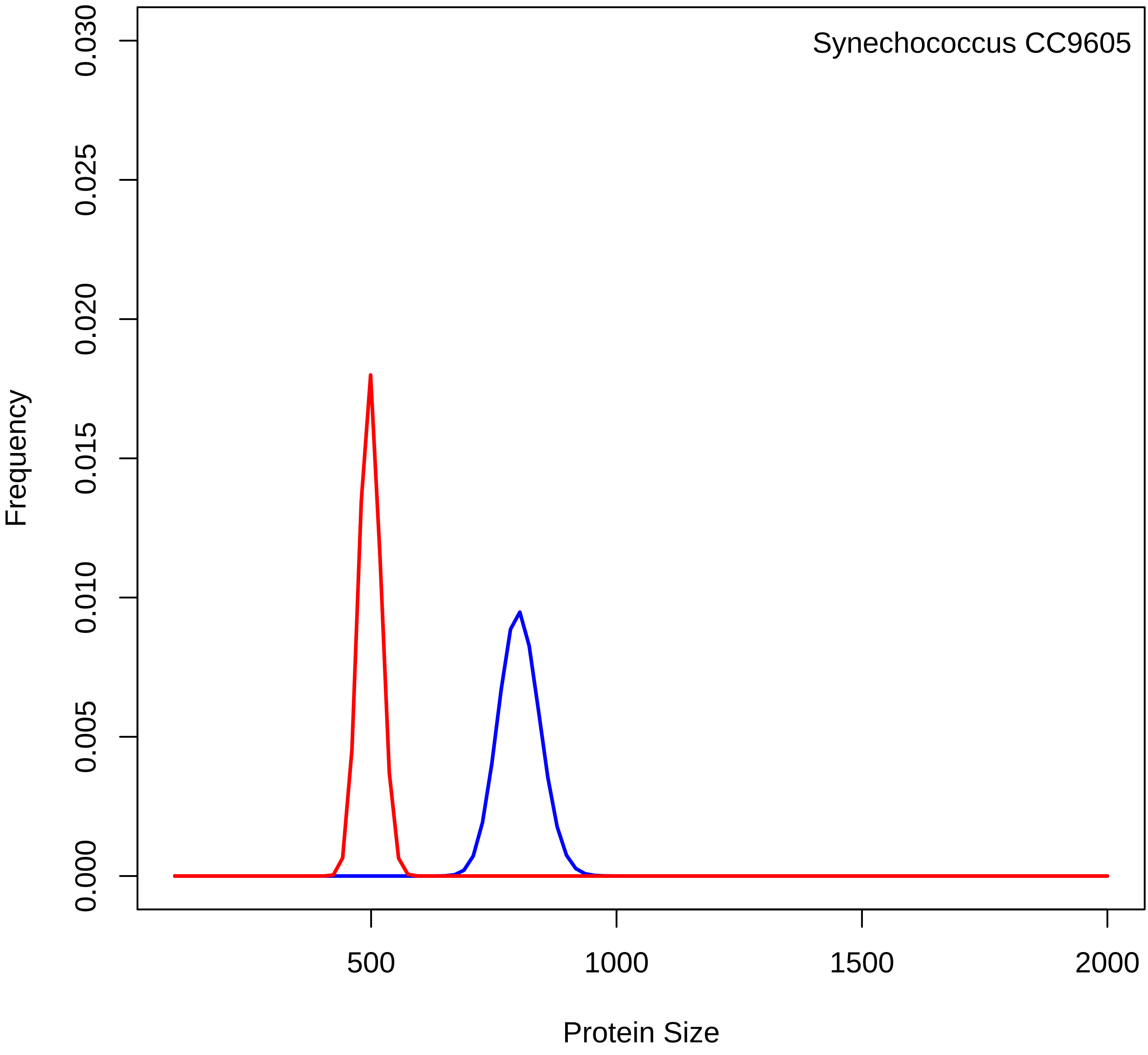

Supplement 4 − Figure 298

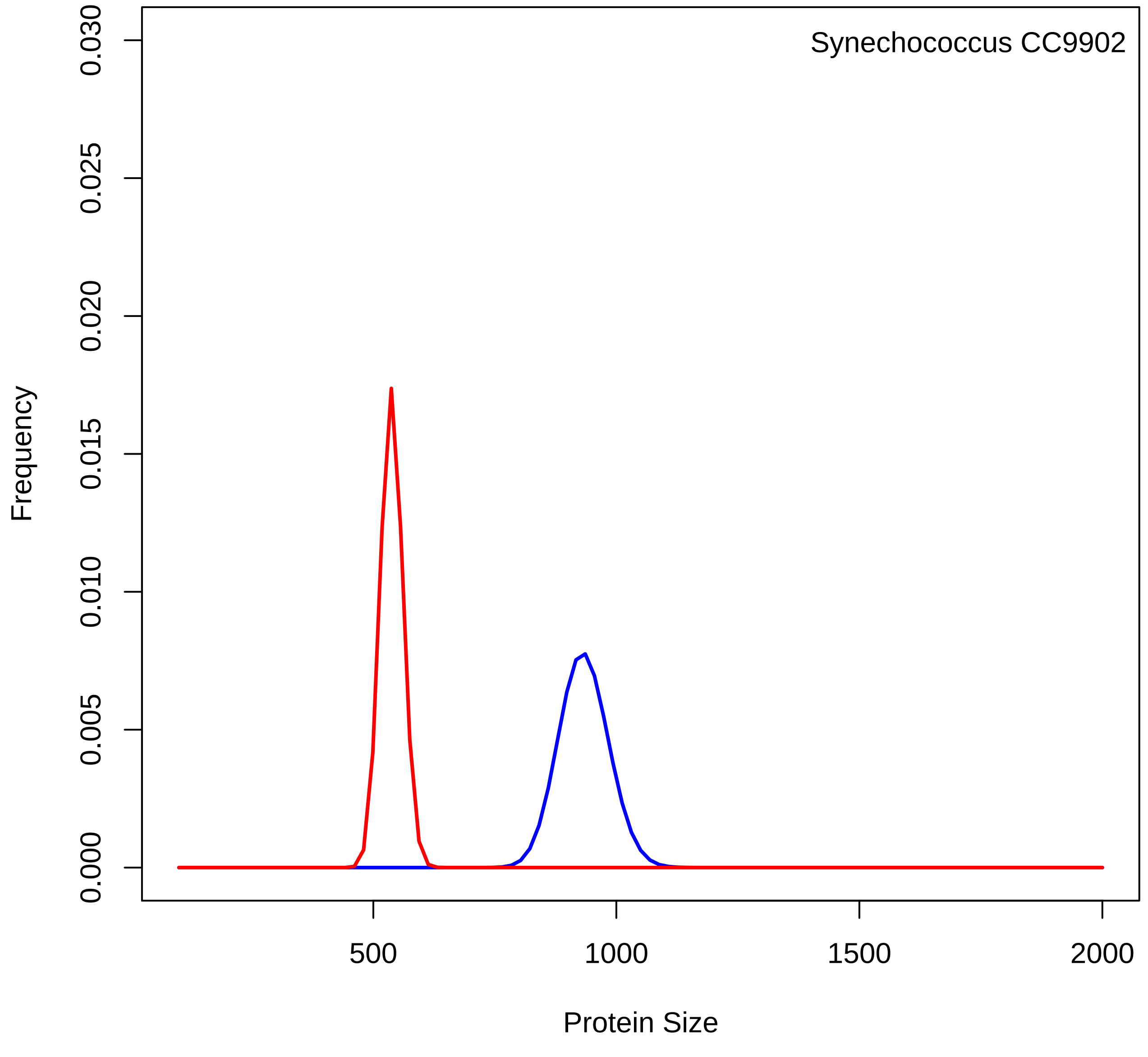

Supplement 4 − Figure 299

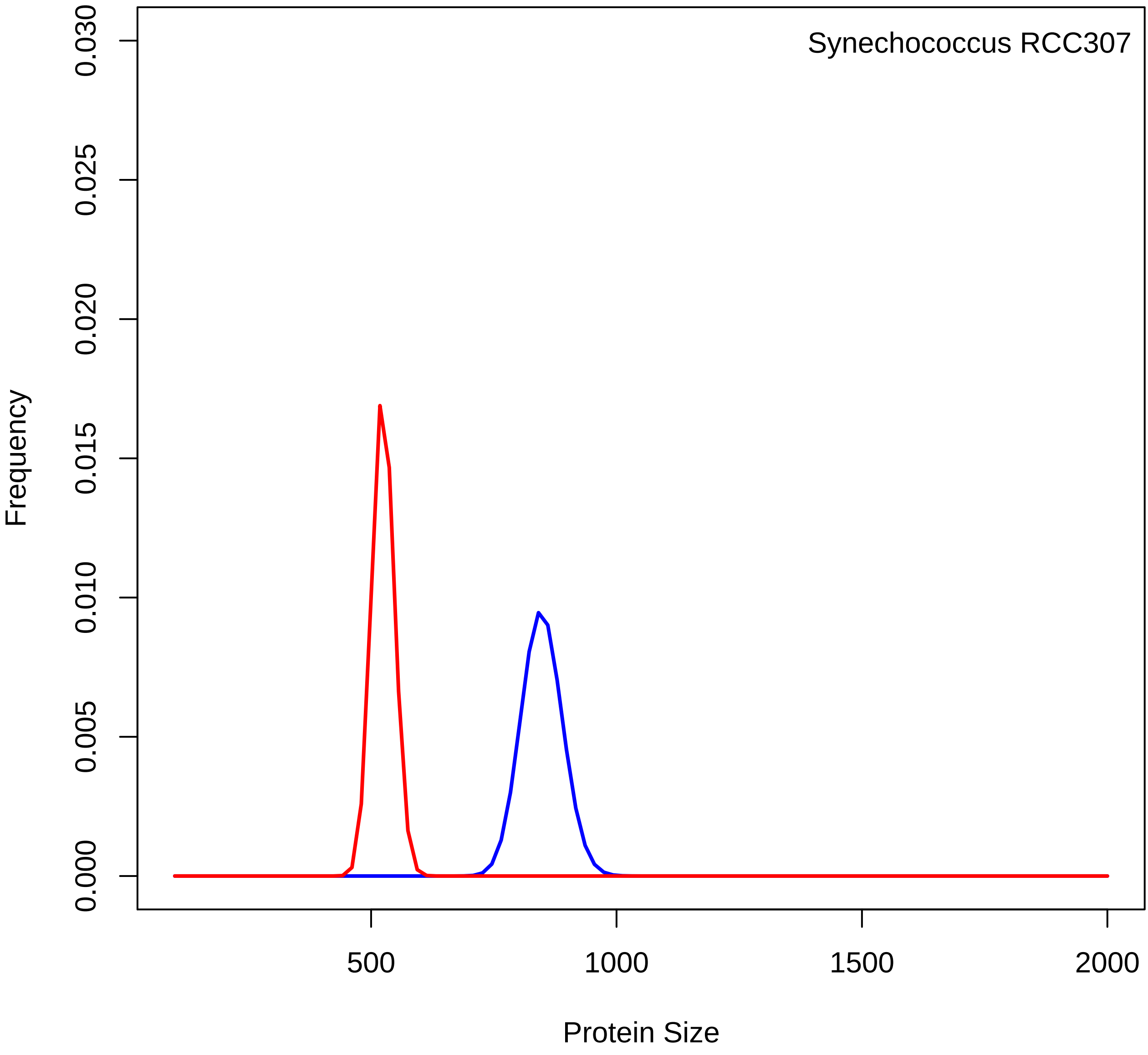

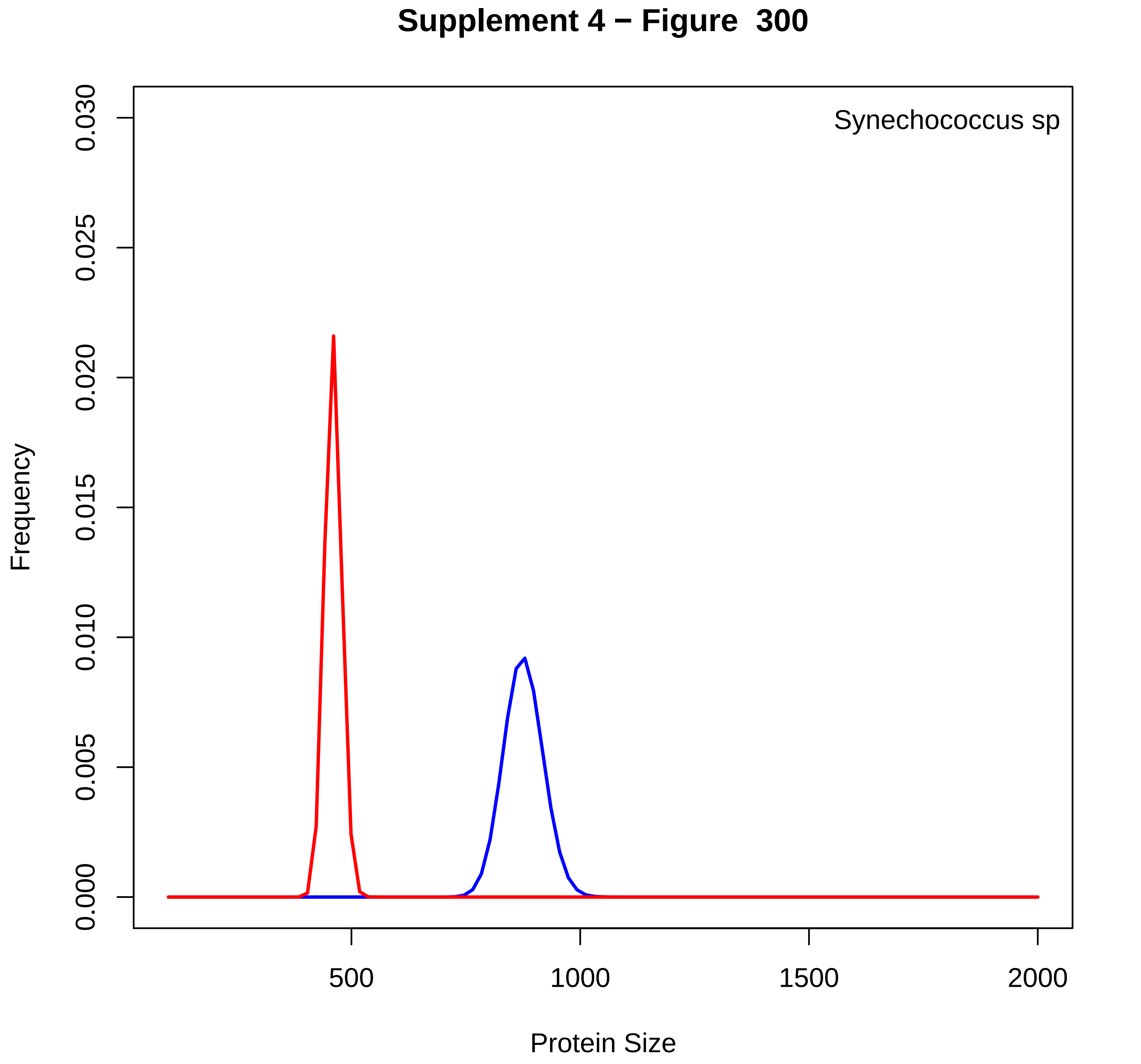
Supplement 4 − Figure 300
Synechococcus sp
Frequency
0.000
0.005
0.010
0.015
0.020
0.025
0.030
500
1000
1500
2000
Protein Size

Supplement 4 − Figure 301

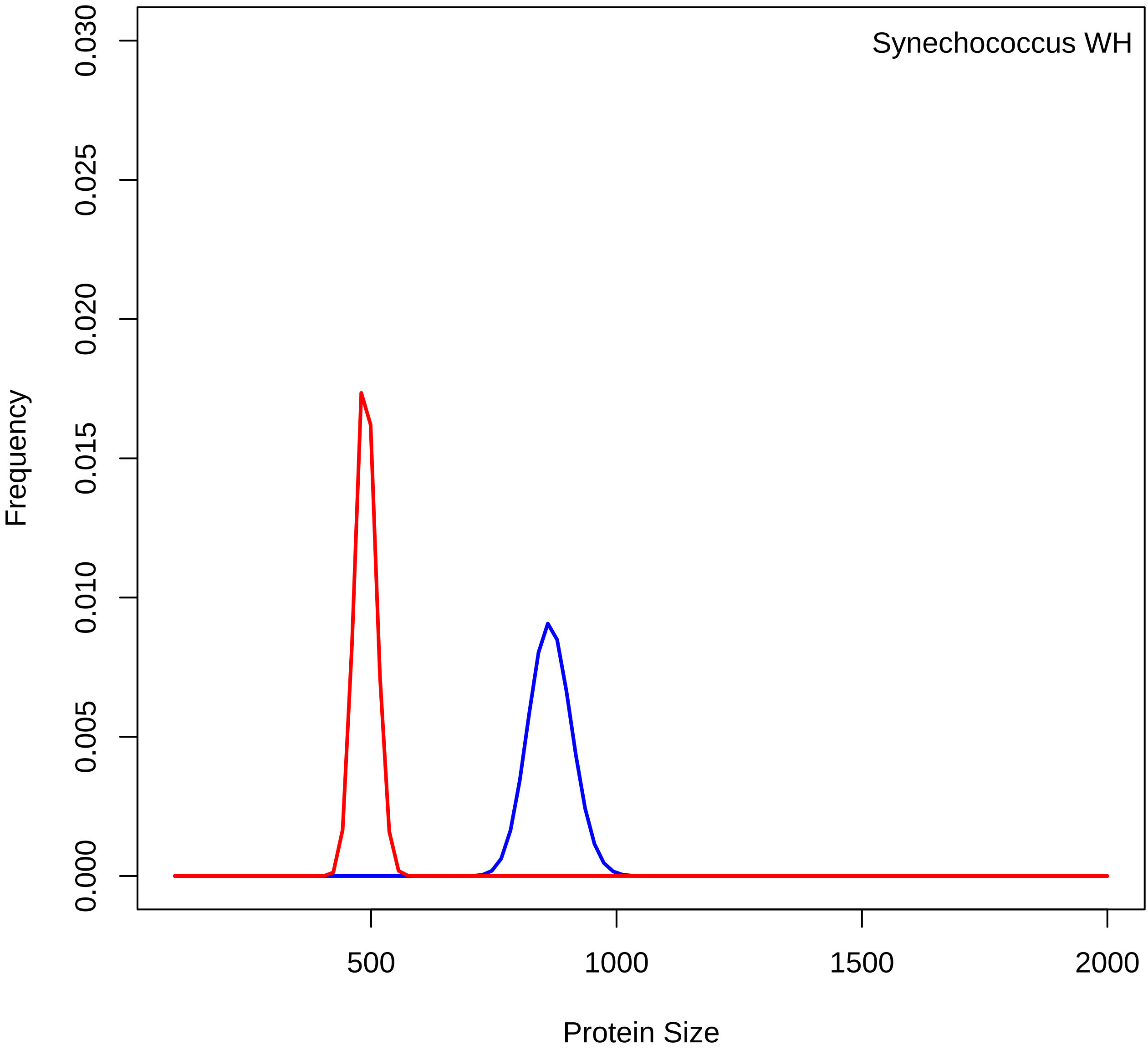

Supplement 4 − Figure 302

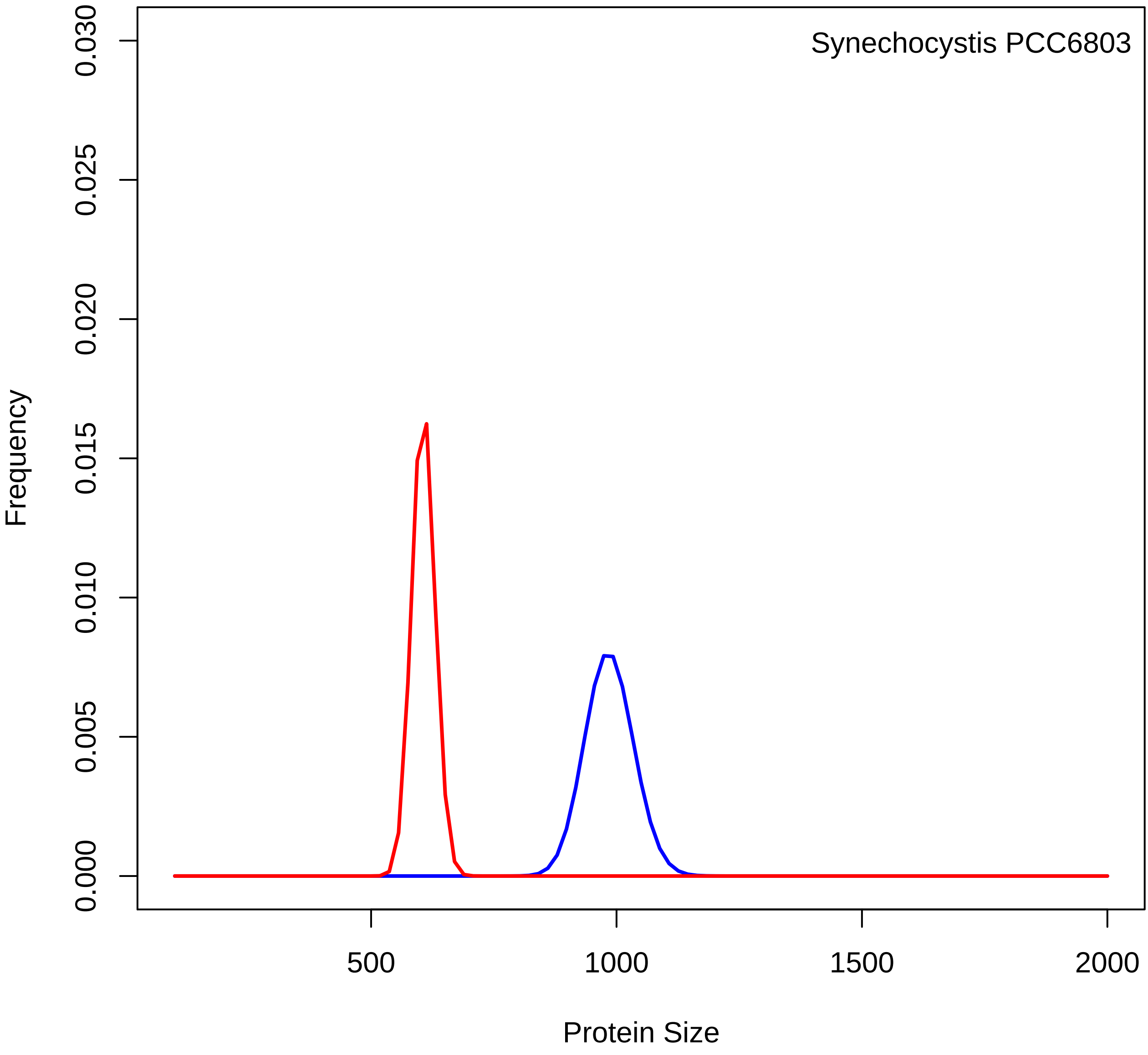

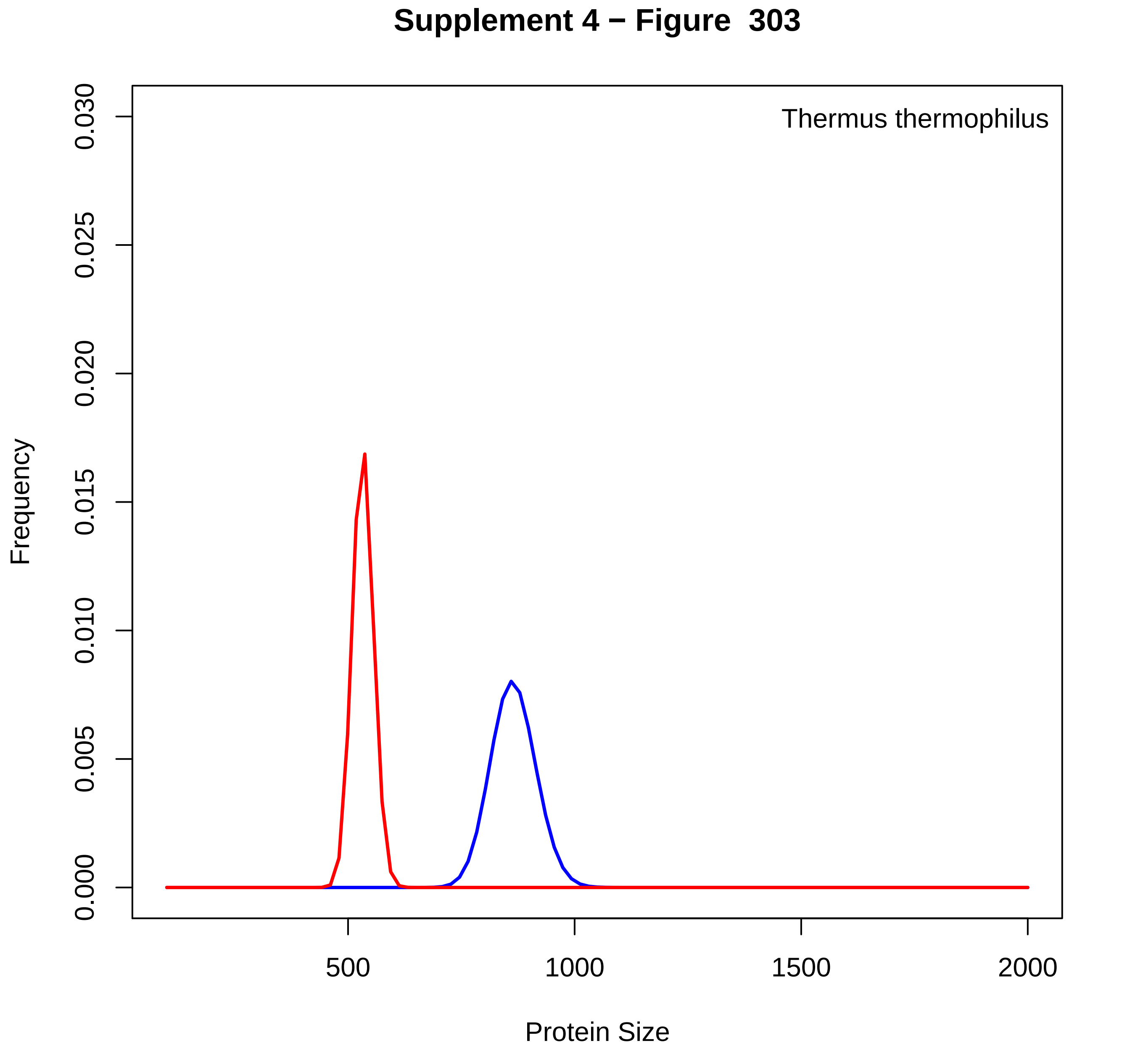

Supplement 4 − Figure 303
Thermus thermophilus
Frequency
0.000
0.005
0.010
0.015
0.020
0.025
0.030
500
1000
1500
2000
Protein Size

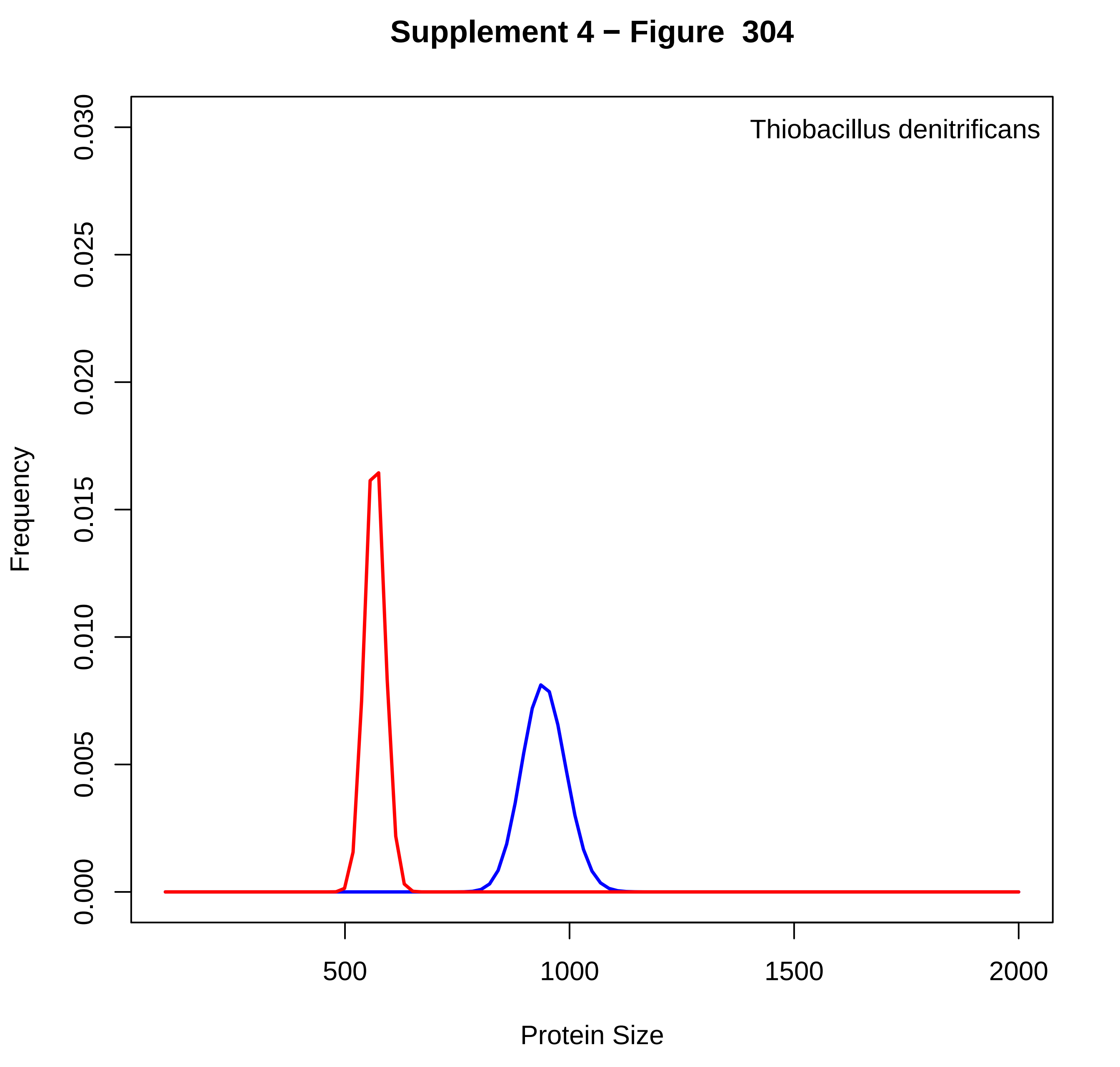
Supplement 4 − Figure 304
Thiobacillus denitrificans
Frequency
0.000
0.005
0.010
0.015
0.020
0.025
0.030
500
1000
1500
2000
Protein Size

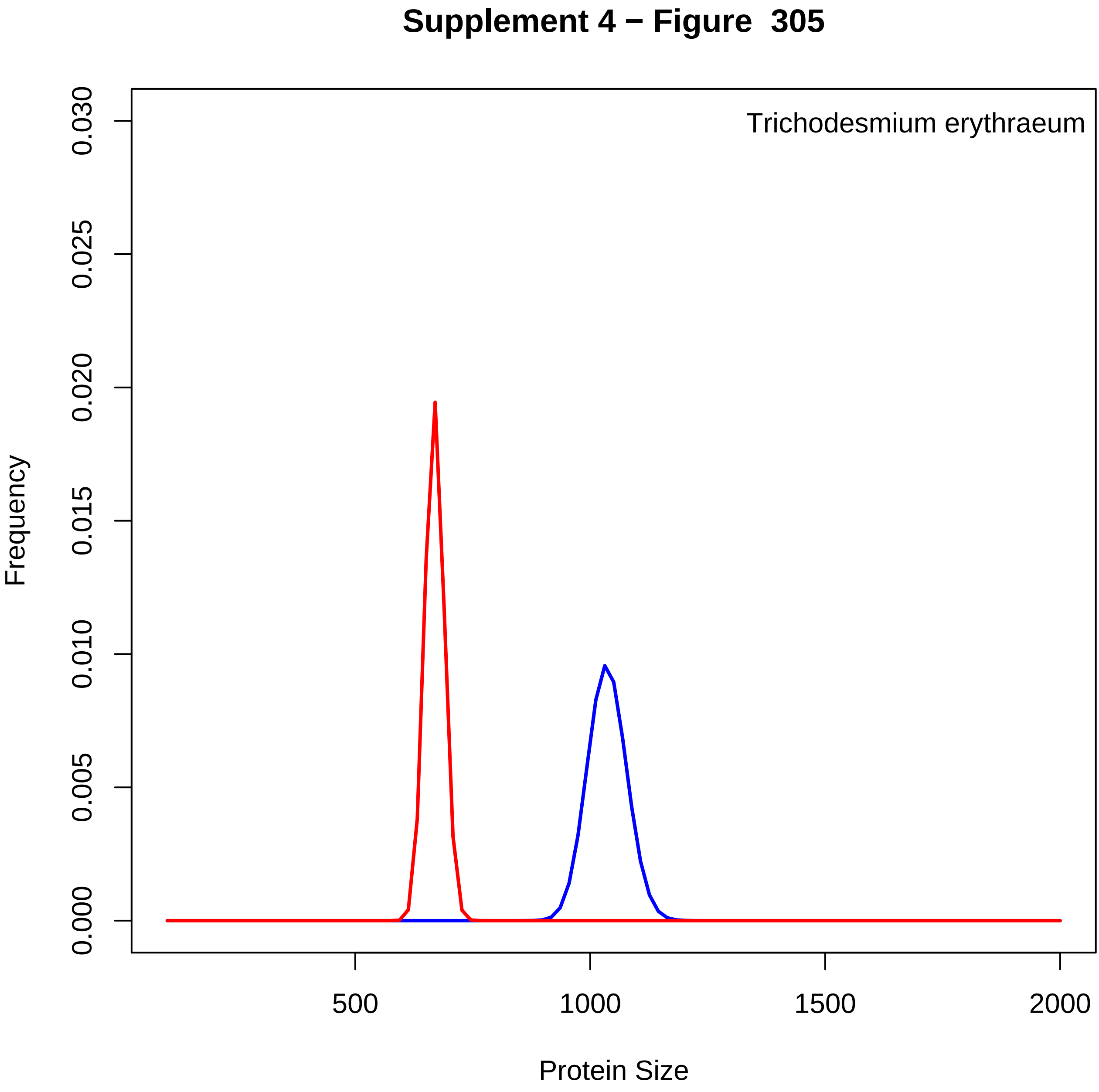
Supplement 4 − Figure 305
Trichodesmium erythraeum
Frequency
0.000
0.005
0.010
0.015
0.020
0.025
0.030
500
1000
1500
2000
Protein Size

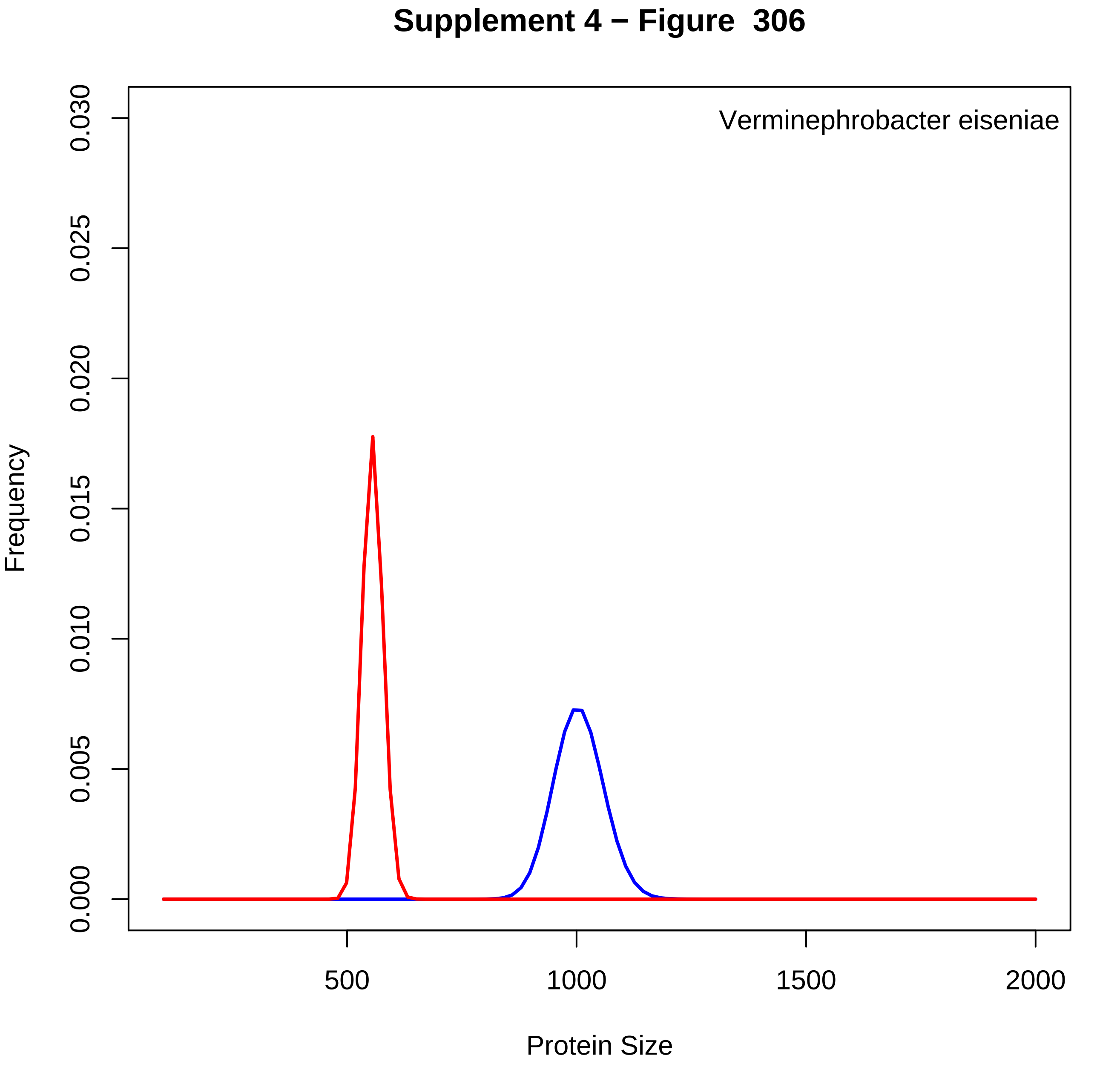

Supplement 4 − Figure 306
Verminephrobacter eiseniae
Frequency
0.000
0.005
0.010
0.015
0.020
0.025
0.030
500
1000
1500
2000
Protein Size

Supplement 4 − Figure 307

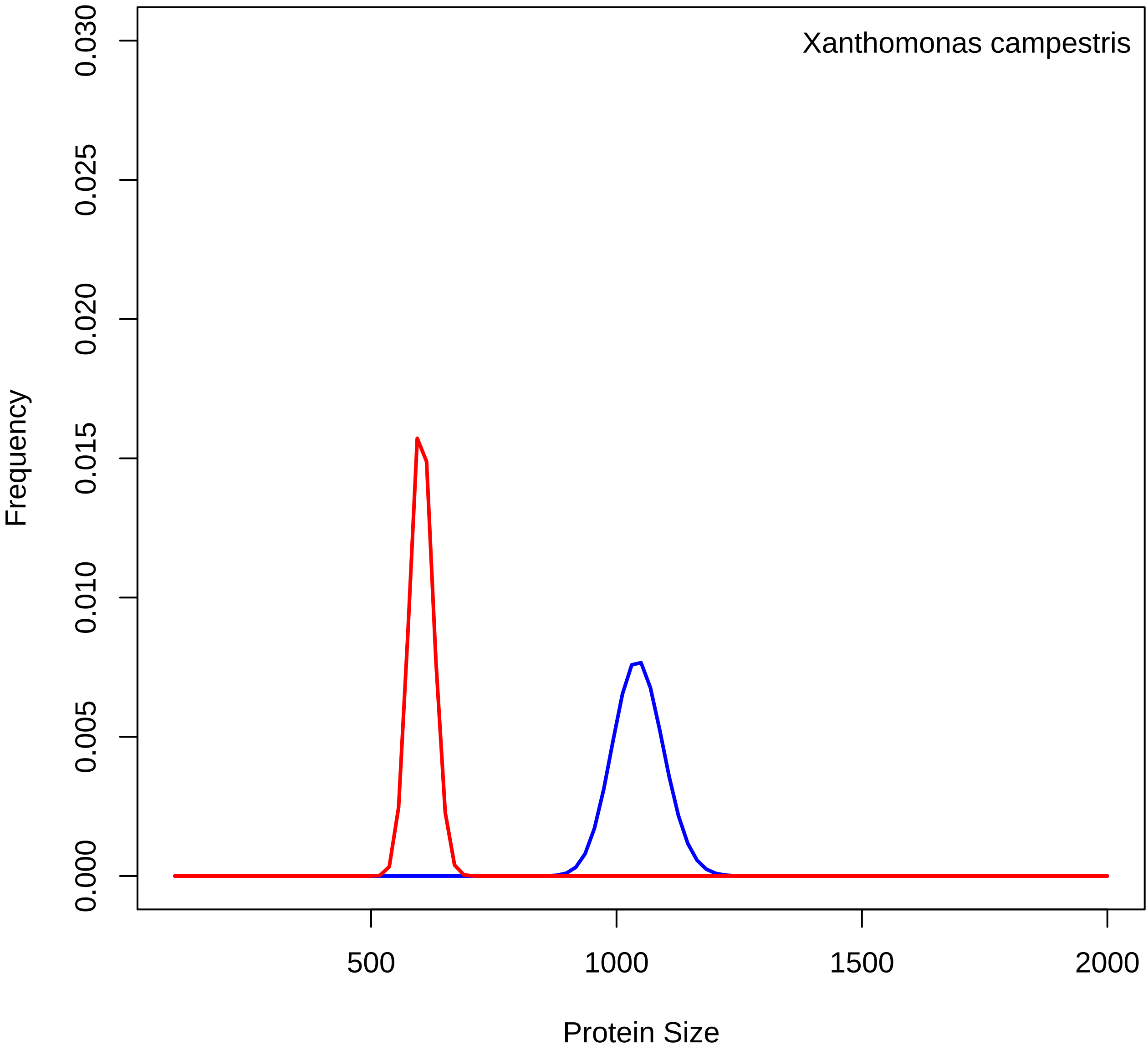

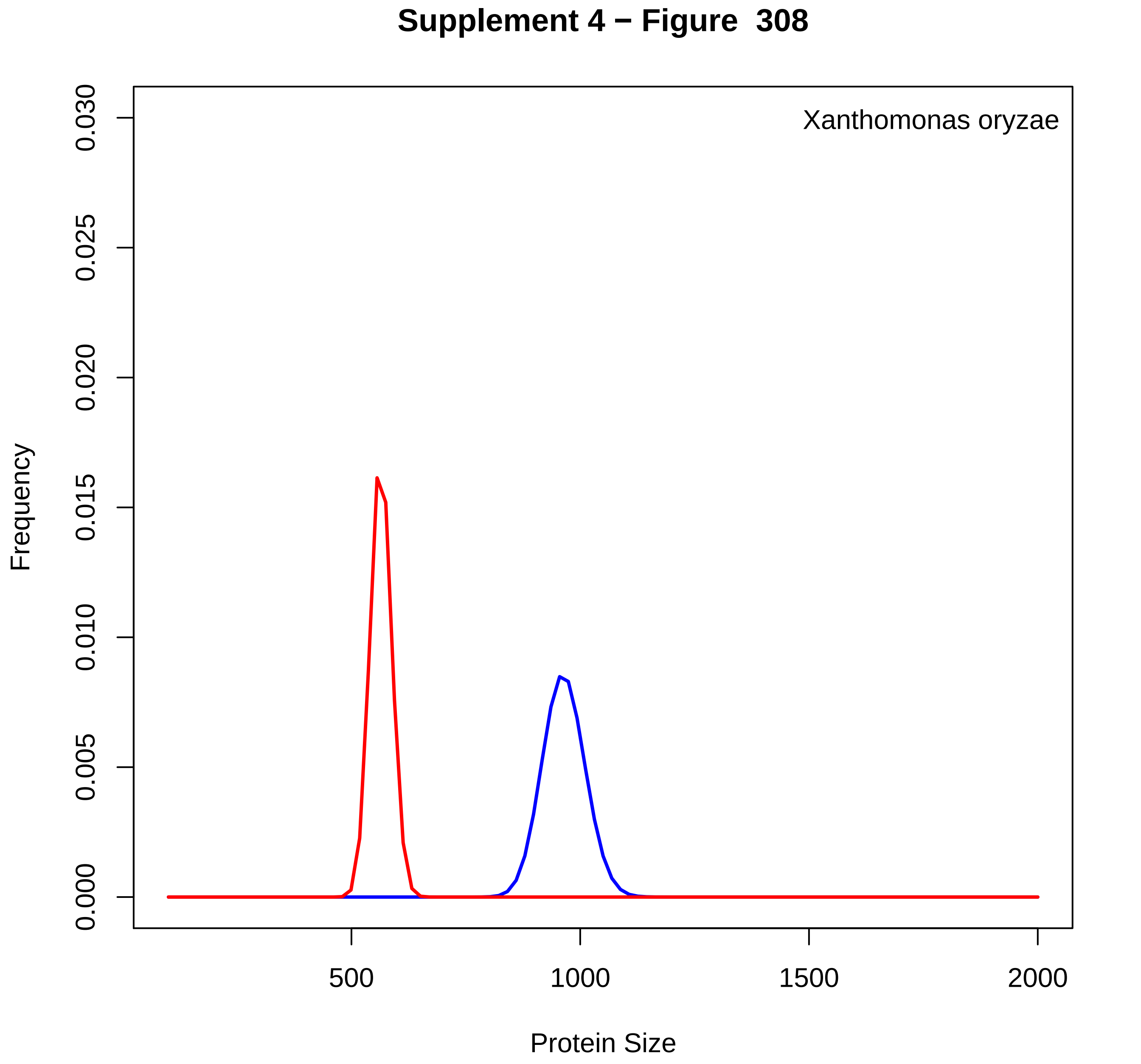

Supplement 4 − Figure 308
Xanthomonas oryzae
Frequency
0.000
0.005
0.010
0.015
0.020
0.025
0.030
500
1000
1500
2000
Protein Size

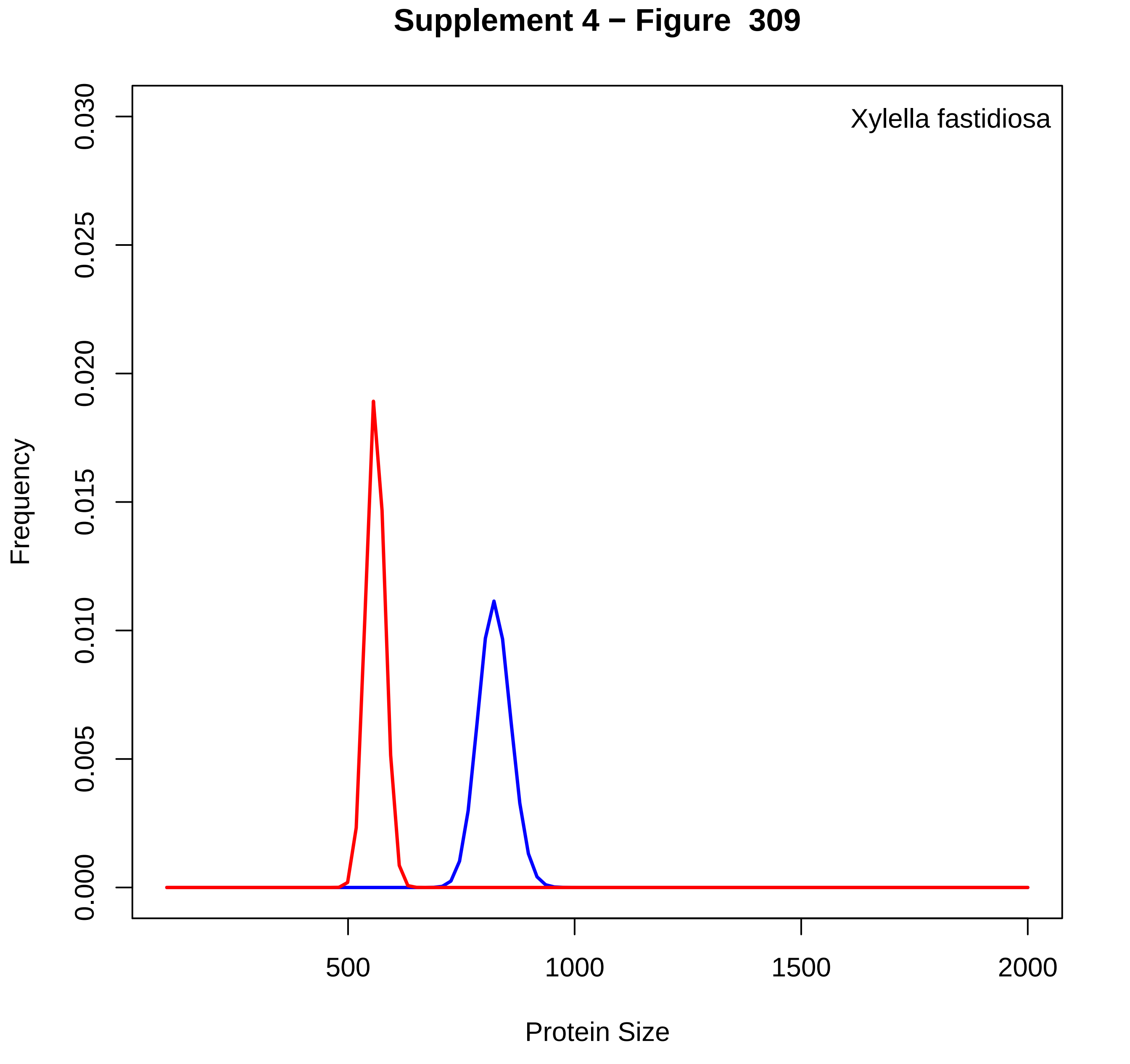
Supplement 4 − Figure 309
Xylella fastidiosa
Frequency
0.000
0.005
0.010
0.015
0.020
0.025
0.030
500
1000
1500
2000
Protein Size

Supplement 4 − Figure 310

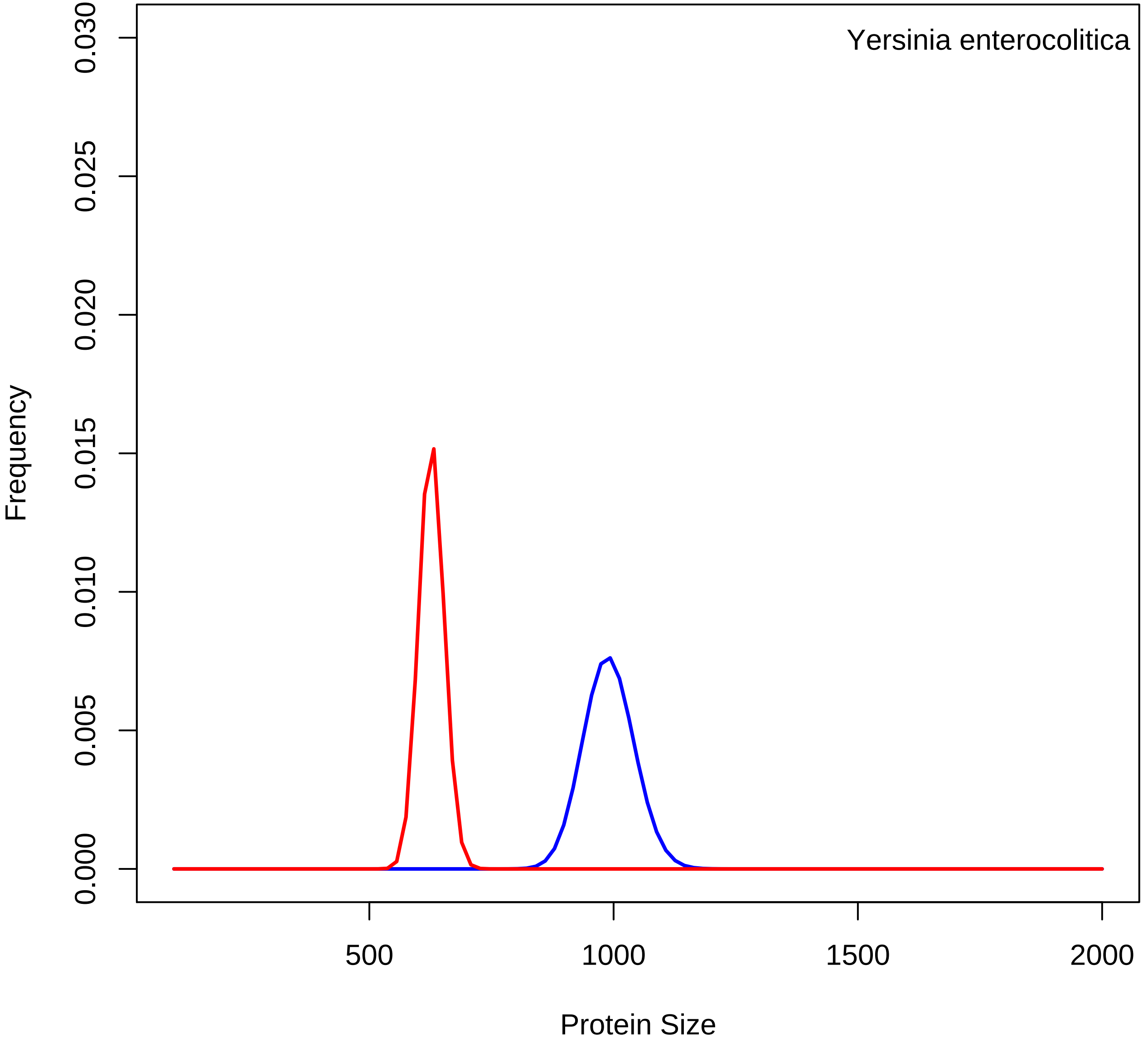

Supplement 4 − Figure 311

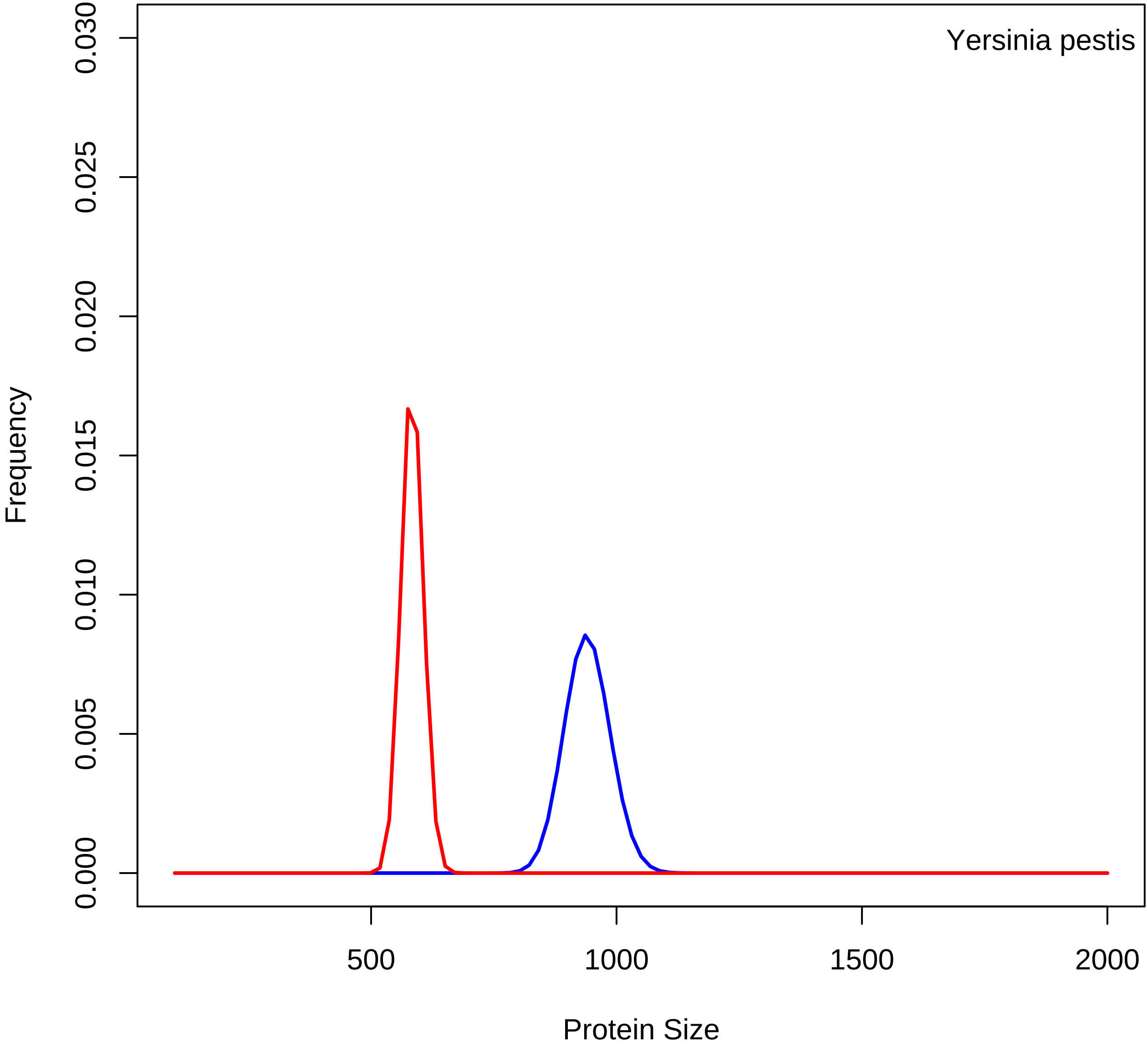

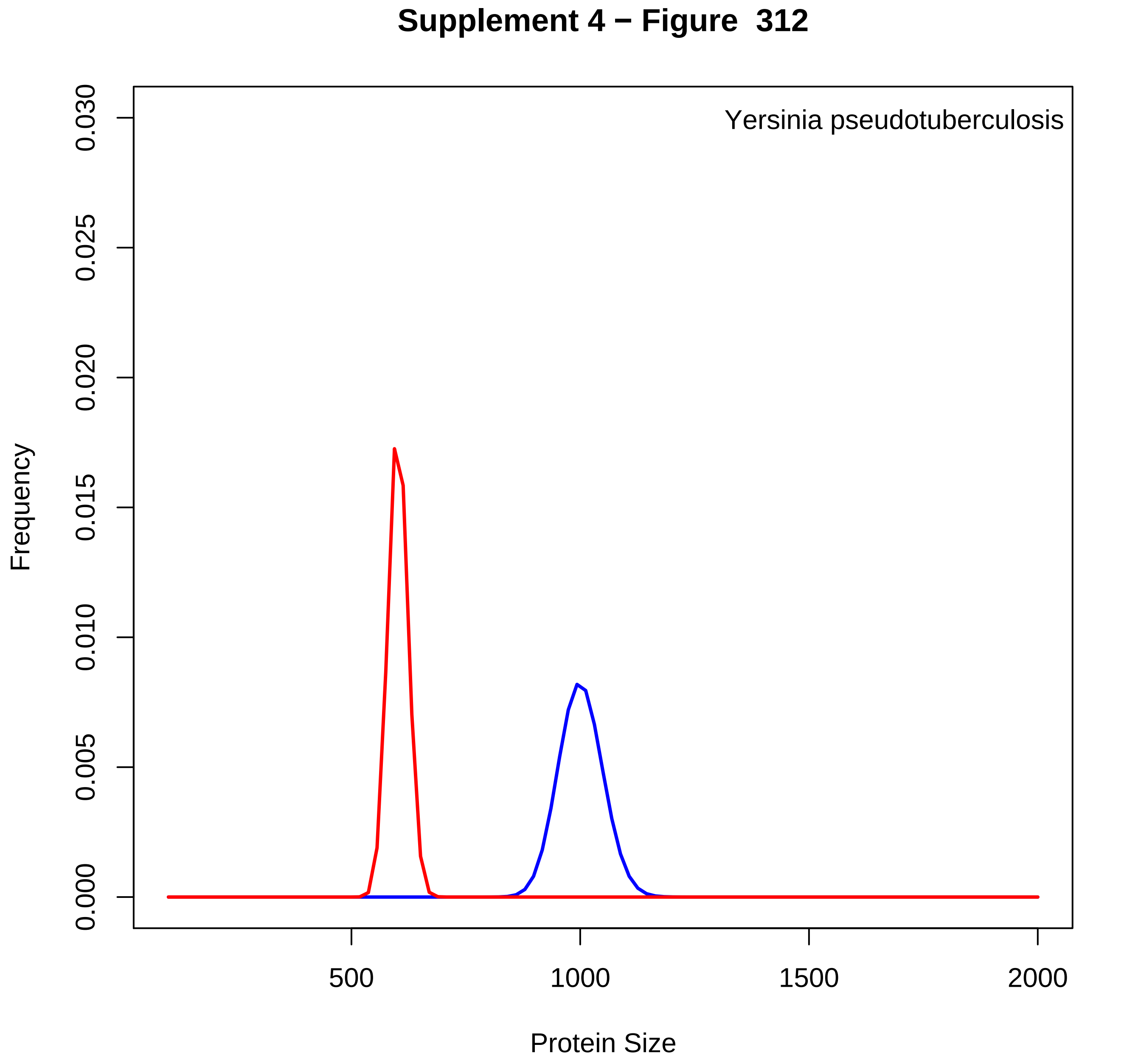
Supplement 4 − Figure 312
Yersinia pseudotuberculosis
Frequency
0.000
0.005
0.010
0.015
0.020
0.025
0.030
Protein Size
500
1000
1500
2000
